# Prediction of NHC-catalyzed chemoselective functionalizations of carbonyl compounds: a general mechanistic map†

**DOI:** 10.1039/d0sc01793k

**Published:** 2020-06-22

**Authors:** Xue Li, Jun Xu, Shi-Jun Li, Ling-Bo Qu, Zhongjun Li, Yonggui Robin Chi, Donghui Wei, Yu Lan

**Affiliations:** College of Chemistry, Institute of Green Catalysis, Zhengzhou University 100 Science Avenue Zhengzhou Henan 450001 China donghuiwei@zzu.edu.cn; College of Pharmacy, Guizhou University of Traditional Chinese Medicine Guiyang China; Division of Chemistry & Biological Chemistry, School of Physical & Mathematical Sciences, Nanyang Technological University Singapore 637371 Singapore; College of Chemistry and Chemical Engineering, Chongqing University Chongqing 400044 China lanyu@cqu.edu.cn

## Abstract

Generally, N-heterocyclic carbene (NHC) complexed with carbonyl compounds would transform into several important active intermediates, *i.e.*, enolates, Breslow intermediates, or acylazolium intermediates, which act as either a nucleophile (Nu) or an electrophile (E) to react with the other E/Nu partner. Hence, the key to predicting the origin of chemoselectivity is to compute the activity (*i.e.*, electrophilic index *ω* for E and nucleophilic index *N* for Nu) and stability of the intermediates and products, which are suggested in a general mechanistic map of these reactions. To support this point, we selected and studied different cases of the NHC-catalyzed reactions of carbonyl compounds in the presence of a base and/or an oxidant, in which multiple possible pathways involving acylazolium, enolate, Breslow, and α,β-unsaturated acylazolium intermediates were proposed and a novel index *ω* + *N* of the E and Nu partners was employed to exactly predict the energy barrier of the chemoselective step in theory. This work provides a guide for determining the general principle behind organocatalytic reactions with various chemoselectivities, and suggests a general application of the reaction index in predicting the chemoselectivity of the nucleophilic and electrophilic reactions.

## Abstract 2

A novel index *ω* + *N* can be used to predict the chemoselectivity according to the general NHC-catalyzed reaction mechanism.

## Graphical abstract

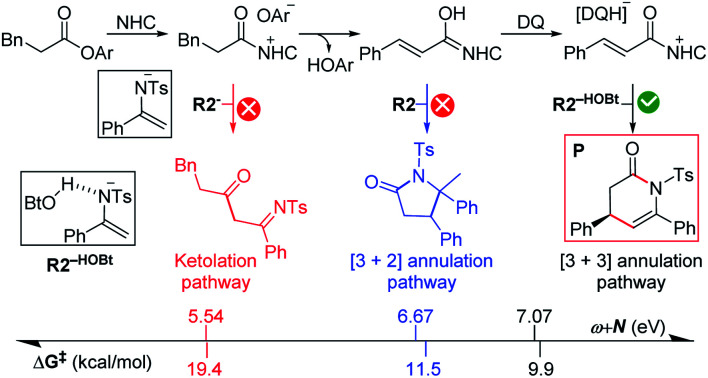

## Introduction

**Scheme 1:**
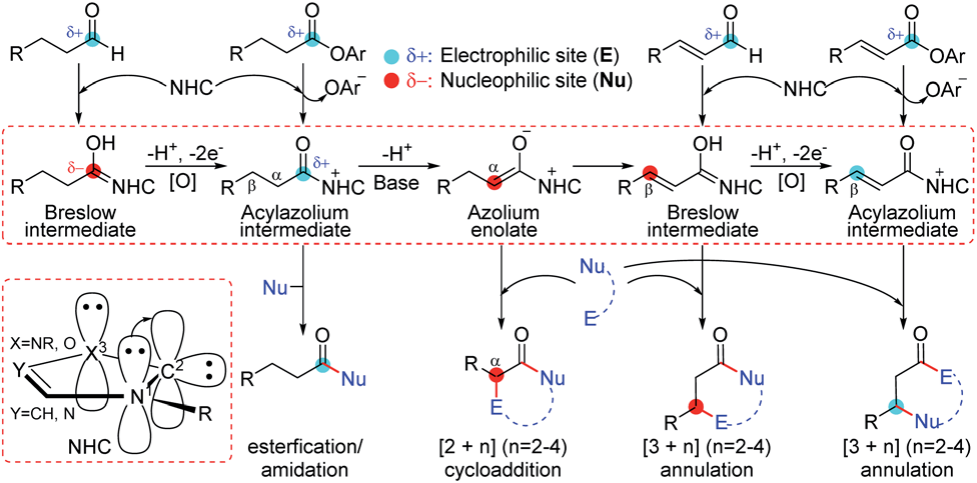
NHC-catalyzed transformation modes of carbonyl compounds.

The combination of a nitrogen atom with a lone pair of electrons and a 6e carbon atom in N-heterocyclic carbenes (NHCs) provides a conjugation between the two atoms, which can significantly stabilize the unoccupied orbital of the carbon atom to achieve a typical singlet carbene.^1^ In this kind of chemistry, NHCs usually act as nucleophiles by using the occupied sp^2^ orbital of the carbon atom to activate polarized unsaturated substrates, which can reversibly form a covalent bond with active molecules.^2^ Further transformations of the forming intermediates can reverse the electrophilic substrates to nucleophiles by the electron-donation of the conjugative nitrogen atoms. Therefore, NHCs can be used as powerful catalysts in organocatalysis due to the special characteristics of nucleophilicity,^3^ good leaving group ability,^4^ and tunable electronic^5^ and steric properties.^6^ As an example (Scheme 1), the reaction of NHCs with aldehydes can generate a formal enol, namely Breslow intermediates, which can be used as a nucleophile to react with electrophilic partners for the synthesis of a variety of functional molecules including biologically active natural products,^7^ organic materials,^8^ pharmaceuticals,^9^ agrochemicals, *etc.*^10^ Indeed, the other electrophiles, such as ketenes, enals, olefins, or imines, can also be activated by NHCs according to their nucleophilicity, which significantly increases the complexity of carbene chemistry.^11^

In NHC-mediated transformations, the introduction of an external base or oxidant increases the complication of the reaction mechanism because the additives can probably reverse the electronic properties of NHC-involved active intermediates. As a contrast, the mechanism for the NHC-catalyzed cycloaddition of ketenes only involves three steps of adsorption, cycloaddition, and dissociation in the absence of external additives. Alternatively, when a Brønsted base participates in the NHC-catalyzed functionalization of aldehydes,^12^ enals,^13^ or esters,^14^ the transformations would involve more steps due to the extra proton transfer in those reactions. Furthermore, the situation becomes much more complex in the presence of an external oxidant,^15^ because the key step of hydrogen transfer to oxidants might take place through hydride transfer to oxygen or carbon (HTO/HTC),^16^ single electron transfer-hydrogen atom transfer (SET-HAT),^17^ or even HAT-SET^18^ processes. However, the lack of a universal principle to disclose the mechanism of the NHC-catalyzed carbonyl compound transformations restricts the rational design of both NHC-catalysts and reaction types. Hence, predicting the chemoselectivity and designing a map for the general mechanisms of the NHC-catalyzed transformations of carbonyl compounds in the presence of an external base and/or oxidant are still challenging and highly desirable in this field.

In NHC-catalysis, carbonyl compounds are one of the most commonly used electrophiles that can be activated by the adsorption of NHCs. When external additives are added, further deprotonation and/or oxidation greatly increase the complexity of the reaction mechanism. For instance, an aldehyde can be activated by the NHC catalyst to afford the Breslow intermediate, which can be oxidized to the acylazolium intermediate by an oxidant (Scheme 1). The deprotonation of the acylazolium assisted by a base can generate azolium enolate, which can isomerize to an α,β-unsaturated Breslow intermediate through proton transfer. Further oxidation can provide the α,β-unsaturated acylazolium species. Previous experiments have demonstrated that all the above mentioned intermediates can react with specific nucleophiles or electrophiles to achieve the functionalization of carbonyl compounds.^13*a*–*i*,14,15^ However, the origin of chemoselectivity for the competitive transformation of corresponding intermediates still remains a challenge for synthetic chemists and could be solved by the exploration of reaction mechanisms. In this work, a series of NHC-catalyzed carbonyl compound transformation reactions will be considered theoretically, and we will try to predict the origin of chemoselectivity from a general perspective. We hope that the understanding of the reaction mechanism and origin of chemoselectivity would be helpful for the design of new transformations in NHC catalysis.

## Computational details

The Gaussian09 program^19^ and M06-2X (ref. 20) functional were employed to perform all the calculations, which have been widely used to study the mechanisms and selectivities^21^ of various catalytic reactions.^22^ All the geometries were optimized at the 6-31G(d,p) basis set in THF and other solvents such as toluene, in which the solvent effect was simulated by the appropriate integral equation formalism polarizable continuum model (IEF-PCM).^23^ The harmonic vibrational frequency calculations were performed at the same computational level as that of the optimization, which ensures no imaginary frequency in the intermediate and only one imaginary frequency in the transition state. The discussed energies of all the optimized geometries were gained by adding the single-point energies and the Gibbs free energy corrections at the M06-2X-D3/6-31++G(2df,2pd)/IEF-PCM_THF_//M06-2X/6-31G(d,p)/IEF-PCM_THF_ computational level (L1).

In addition, the global reactivity index (GRI) analysis was performed on the active intermediates to evaluate their nucleophilic (nucleophilic index *N*)^24^ or electrophilic (electrophilic index *ω*)^25^ reactivities on the basis of the equations: *N* = *E*_H(R)_ − *E*_H(TCNE)_ (*E*_H(TCNE)_ = −0.38586 a.u.), *ω* = *μ*^2^/2*η*, *μ* = (*E*_H_ + *E*_L_)/2, and *η* = (*E*_L_ − *E*_H_),^26^ while the local reactivity index (electrophilic (*P*^+^_k_) and nucleophilic (*P*^−^_k_) Parr functions)^27^ analysis was also performed to uncover the nucleophilic or electrophilic sites. To confirm the reliability of the computational level (L1), other density functional theory (DFT) methods (*i.e.*, B3LYP-D3,^28^ CAM-B3LYP-D3,^29^ ωB97X-D,^30^ and MP2) at different levels (L2–6) were carried out on the crucial stereoselective step. Furthermore, other configurations of the stereoselective transition states have been additionally considered, to confirm that all the discussed geometries had the lowest energy. More computational results and details can be found in the ESI.†

## Results and discussion

### General mechanistic map for NHC-catalyzed chemoselective functionalizations of esters

**Scheme 2:**
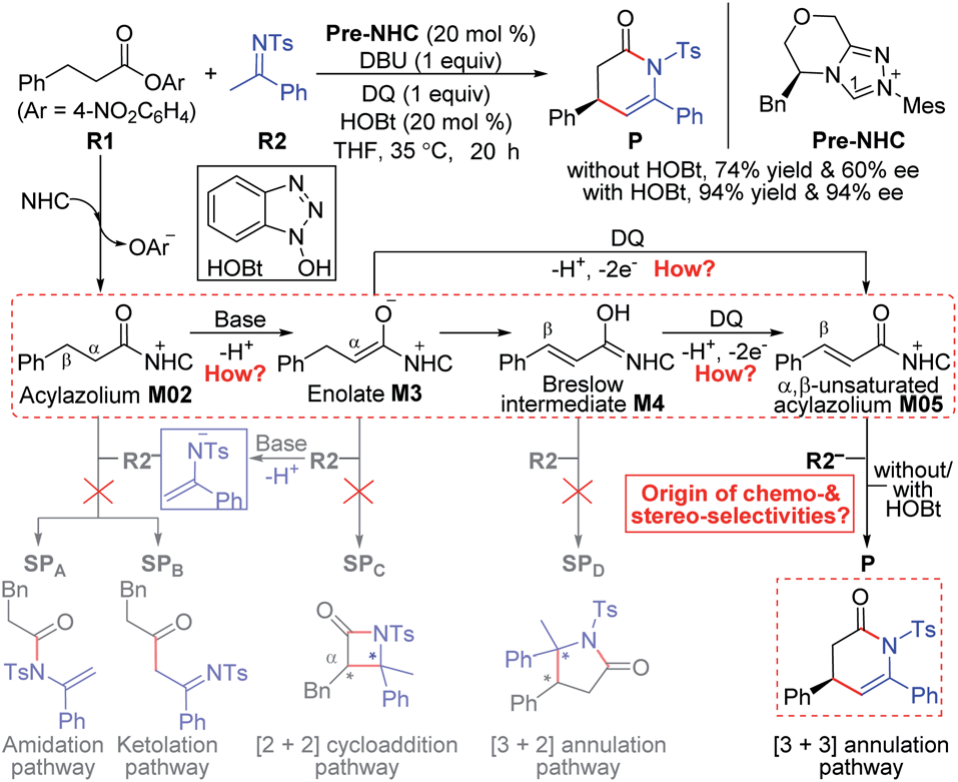
Possible reaction pathways of NHC-catalyzed reactions between saturated carboxylic esters and imines.

As a selected model reaction (Scheme 2), when the NHC precursor **Pre-NHC** was used as a catalyst, ester **R1** can react with imine **R2** to afford the [3 + 3] oxidative annulation product **P** with up to 76% yield and a 60% enantiomeric excess (ee) value in the presence of the base DBU and oxidant 3,3′,5,5′-tetra-*tert*-butyl diphenoquinone (DQ).^31^ The Chi group also found that the additive HOBt can significantly improve the yield (from 76% to 94%) and enantioselectivity (from 60% to 94% ee).^31^ According to our proposed transformation model in Scheme 1, when acylazolium intermediate **M02** is formed, it can be deprotonated to form an enolate intermediate, **M3**. Moreover, its Breslow type isomer **M4** can be oxidized to an α,β-unsaturated acylazolium species, **M05**. As shown in Scheme 2, all these intermediates can react with nucleophile **R2−** or electrophile **R2** to afford the corresponding amidation/ketolation, [2 + 2] cycloaddition, [3 + 2] annulation, and [3 + 3] annulation products. However, only the [3 + 3] oxidative annulation product **P** was observed experimentally, while the origin of chemoselectivity for this transformation still remains unclear. Moreover, the enantioselectivity for [3 + 3] oxidative annulation with additive effects should also be studied. Therefore, this transformation was selected in our theoretical calculation as a model reaction to reveal the common model for the NHC-catalyzed carbonyl compound transformations.

**Fig. 1:**
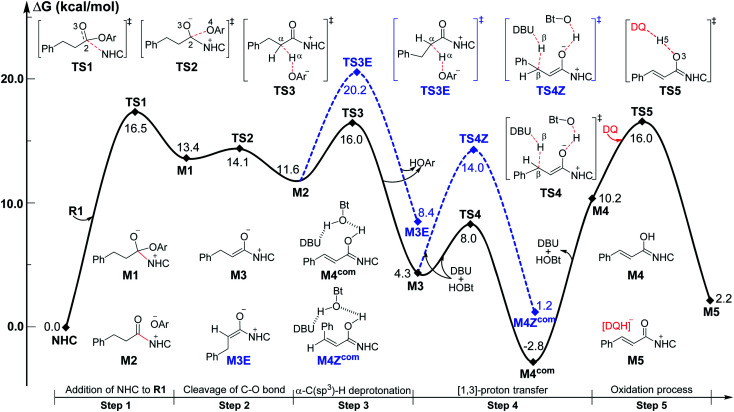
Gibbs free energy profiles of the NHC-catalyzed oxidative α,β-C(sp^3^)–H functionalization of the ester.

**Fig. 2:**
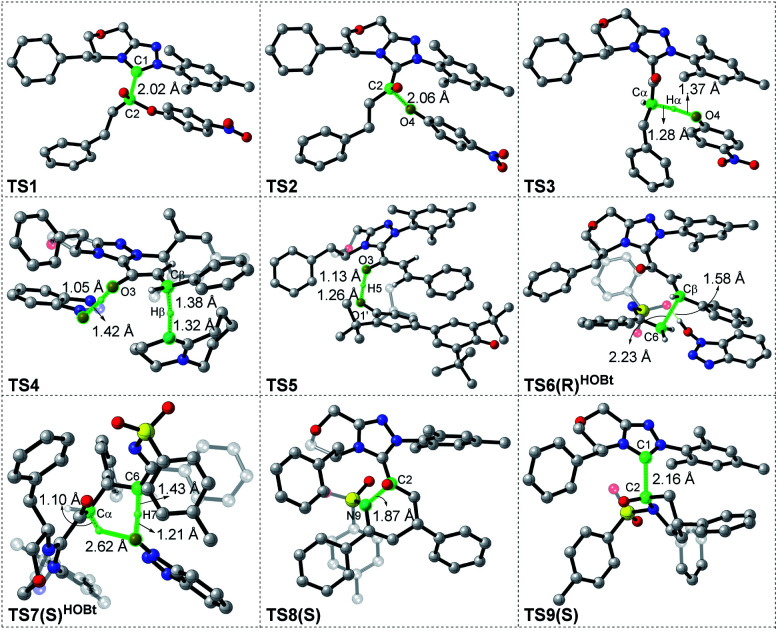
Optimized geometries of transition states in the [3 + 3] annulation pathway with HOBt; the hydrogen atoms that are not involved in the reaction have been omitted (distances: Å).

**Fig. 3:**
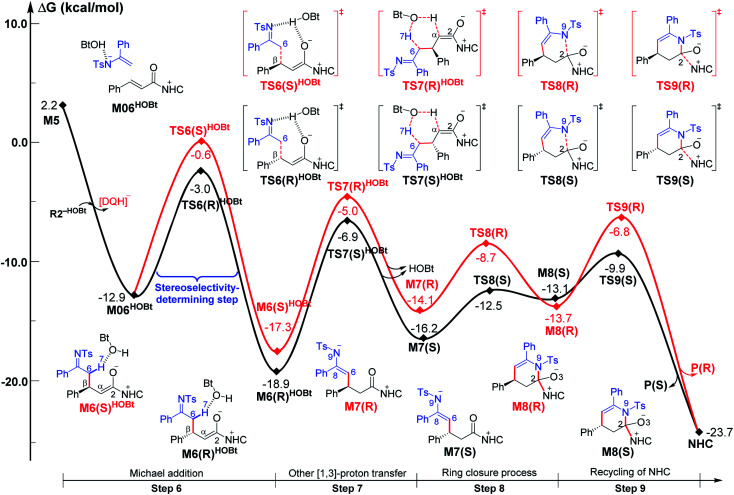
Gibbs free energy profiles for NHC-catalyzed oxidative [3 + 3] annulation of the ester with imine in the presence of HOBt.

Among the proposed pathways, the [3 + 3] annulation pathway was mainly discussed in detail for the NHC-catalyzed reactions, while the other four possible pathways were divorced from the key active intermediates. Both the non-free-carbene pathway and carbene generation pathway were considered, and the computed results indicated that the DBU-assisted deprotonation of azolium catalyst **Pre-NHC** to afford free NHC can be easily happen (Fig. S1 of the ESI†).^32^ The [3 + 3] annulation pathway contains nine steps, as shown in Fig. 1–3.

As shown in Fig. 1, the active site C1 of the NHC nucleophilically attacks the carbonyl carbon C2 of ester **R1** initially through transition state **TS1**, forming zwitterionic intermediate **M1** with an energy barrier of 16.5 kcal mol^−1^. The structural transformation from NHC and aryl ester to the zwitterionic (tetrahedral) intermediate needs to overcome an energy barrier of 16.5 kcal mol^−1^, which is close to that reported in ref. 33. Subsequently, the dissociation of OAr^−^ takes place *via* a C2–O4 bond cleavage transition state **TS2** with an energy barrier of only 0.7 kcal mol^−1^, affording intermediate **M2** (the complexation of the acylazolium intermediate **M02** and OAr^−^). Then, an α-C(sp^3^)–H deprotonation of **M2** is assisted by anionic OAr^−^ through transition states **TS3**/**TS3E** to generate the corresponding enolate intermediates **M3**/**M3E**. The relative free energy of transition state **TS3** is 4.2 kcal mol^−1^ lower than that of **TS3E**; hence, the pathway associated with **TS3**/**M3** is preferred. In addition, the base DBU-assisted α-C(sp^3^)–H deprotonation pathway (Fig. S2 and S3 of the ESI†) was also considered theoretically; however, much higher activation barriers were observed *via* transition states **TS3DBU**/**TS3EDBU**.

After the α-C(sp^3^)–H deprotonation of **M2**, intermediate **M3** undergoes a DBU and HOBt cooperatively assisted [1,3]-proton transfer process *via* transition states **TS4**/**TS4Z** (Δ*G*^‡^ = 3.7/9.7 kcal mol^−1^, Fig. 1), affording the related complexes **M4com**/**M4Zcom**. These complexes subsequently dissociate into Breslow intermediates **M4**/**M4Z** and DBU + HOBt. The energies of **TS4**/**M4com** are 6.0/4.0 kcal mol^−1^ lower than those of **TS4Z**/**M4Zcom**, implying that the pathway associated with **TS4** is energetically preferred. Then, intermediate **M4** is oxidized into a complex **M5**, which consists of the unsaturated acylazolium intermediate **M05** and anion [DQH]^−^. The oxidation occurs *via* transition state **TS5** (Δ*G*^‡^ = 5.8 kcal mol^−1^, Fig. 1) through the most favorable HTO pathway, which is determined after computing and comparing the four proposed oxidative pathways, namely, HTO, HTC, HAT-SET, and SET-HAT.^34^ More information on the natural population analysis (NPA) charge and other possible oxidative pathways can be found in the ESI.†

**Scheme 3:**
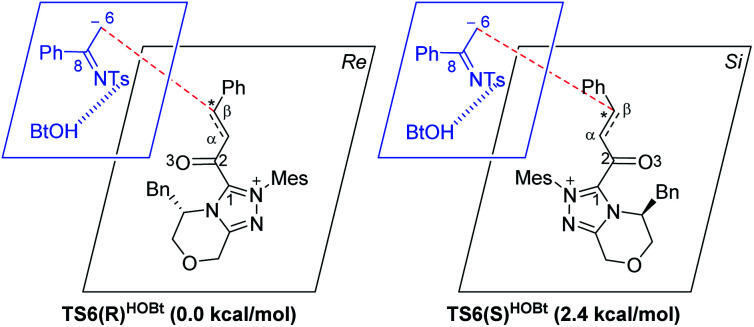
Stereochemical possibilities for the catalytic Michael addition process (unit in kcal mol^−1^).

For the Michael addition process, the two reacting parts are generally nucleophilic (Nu) and electrophilic (E). Obviously, intermediates **M5** or **M05** are the electrophilic parts as their electrophilic indexes (*ω*) equal to 3.093 and 2.979 eV, respectively, so the other reacting part related to **R2** should be nucleophilic. Thus, imine **R2** must be first deprotonated by a base (*i.e.*, OAr^−^, DBU, or [DQH]^−^) to afford the nucleophilic **R2−** anion. As revealed by the computed results in Fig. S9 of the ESI,† the deprotonation of **R2** by base [DQH]^−^ through transition state **TS0DQH−** (Δ*G*^‡^ = 12.5 kcal mol^−1^, Fig. S9 and S10 of the ESI†) is the most favorable pathway among the three possible deprotonation pathways, affording the stable nucleophilic intermediate **R2−HOBt** by interacting with HOBt and dissociating DQH_2_. As depicted in Scheme 3, the nucleophilic site C6 (with *P*^−^_k_(C6) = 0.61) of anionic **R2−HOBt** can attack the *Re*-/*Si*-faces of the electrophilic site Cβ (with *P*^+^_k_(Cβ) = 0.24) of **M5** through transition state **TS6(R/S)HOBt** (Δ*G*^‡^ = 9.9/12.3 kcal mol^−1^, Fig. 3) in the process of forming intermediate **M6(R/S)HOBt** and releasing anion [DQH]^−^, in which a Cβ–C6 bond is formed. The energy difference of 2.4 kcal mol^−1^ between 
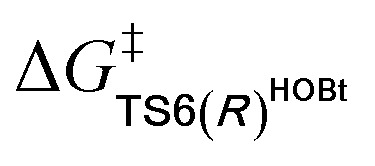
 and 
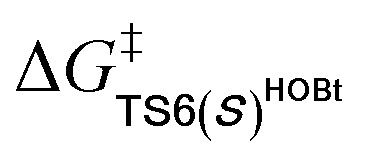
 corresponds to the calculated 96.6% ee value, which is close to the 94% ee value in the presence of HOBt observed in the experiment.^31^ The letters “*R*/*S*” in **M6(R/S)HOBt** represent the chirality of the Cβ center. In addition, since the energies of **TS6(R)HOBt** and **M6(R)HOBt** locate below those of **TS6(S)HOBt** and **M6(S)HOBt**, the processes that follow **M6(S)HOBt** are unnecessary to discuss in detail.

Next, **M6(R)HOBt** conducts the other [1,3]-proton transfer process with the help of the protic media HOBt through transition state **TS7(S)HOBt** (Δ*G*^‡^ = 12.0 kcal mol^−1^), generating intermediate **M7(S)** and dissociating HOBt. It should be noted that the *R*-chirality of the Cβ atom in **TS6**/**M6** is apparently converted to the *S*-chirality in **TS7**/**M7**, since the unsaturated bond is changed from the C2=Cα bond in **TS6**/**M6** to the C6=C8 bond in **TS7**/**M7**. Hereafter, **M7(S)** undergoes a ring closure process *via* a six-membered ring transition state **TS8(S)** (Δ*G*^‡^ = 3.7 kcal mol^−1^) to produce **M8(S)**. Eventually, the catalyst NHC is recycled and a six-membered ring main product of lactam **P(S)** is produced through transition state **TS9(S)** (Δ*G*^‡^ = 3.2 kcal mol^−1^). The energy of **P(S)** is found to be 23.7 kcal mol^−1^ below the energy of the reactant, suggesting that the entire reaction is exothermic. In addition, the difference between the [3 + 3] annulation pathways with or without HOBt is only reflected in steps 6 and 7. Fig. S11 of the ESI† shows that the energy barriers of steps 6 and 7 *via* transition states **TS6(R/S)** and **TS7(S/R)HOAr** are 13.6/14.4 and 13.2/10.4 kcal mol^−1^ in the [3 + 3] annulation pathway without HOBt, respectively. Apparently, the energy barrier difference between Δ*G***TS6(R)**^‡^ and Δ*G***TS6(S)**^‡^ is only 0.8 kcal mol^−1^ and can be theoretically converted to a 59% ee value, which is very close to the observed 60% ee value in the experiment,^31^ indicating that HOBt is indeed able to advance the stereoselectivity.

To ensure the enantioselective transition states associated with the lowest energy configurations, we have performed a conformational study *via* constructing different configurations of the **TS6(R/S)** and **TS6(R/S)HOBt** by rotating 90° per time of the dihedral *Φ*1(N–C1–C2–Cα) (see Schemes 3 and S5 of the ESI†). Therefore, eight (2 × 4) possible conformations were constructed and computed for either **TS6(R/S)** or **TS6(R/S)HOBt**, which can be named **TS6(R/S)**, **TS6(R/S)′**, **TS6(R/S)′′**, **TS6(R/S)′′′**, and **TS6(R/S)HOBt**, **TS6(R/S)HOBt′**, **TS6(R/S)HOBt′′**, **TS6(R/S)HOBt′′′**, respectively. The computed results in Table S3 of the ESI† indicate that the energies of the **TS6(R/S)** and **TS6(R/S)HOBt** are the lowest among the distinctive conformers.

According to the recent work reported by Singleton and Plata, the calculated proton transfer barrier might be unreliable and has a large error in the alcohol-mediated Morita–Baylis–Hillman (MBH) reactions using the most popular DFT methods, *i.e.*, B3LYP and M06-2X,^35^ which would be due to the absence of explicit solvents in the models.^36^ Therefore, to test whether the same problem exists in the NHC-catalyzed reactions, we have additionally constructed the models with 100 explicit THF solvents, and computed the energy barriers of the proton transfer processes (steps 4 and 5) at the ONIOM(B3LYP/6-31G(d,p):UFF) and ONIOM(M06-2X/6-31G(d,p):UFF) levels.

As summarized in Table 1, the calculated results indicate that the free energy barriers ΔΔ*G*_tot_ (with the normal Gibbs free energy correction), ΔΔ*G*_50%_ (with 50% of the Gibbs free energy correction), and ΔΔ*G*_explicit_ (calculated in the explicit solvents without the implicit model) are close, and the free energy barriers obtained by the two DFT methods have tiny differences, which is remarkably different from the computed results reported in Singleton's work. As mentioned above, the computational errors in this system should not be significant and the calculated results using the M06-2X method are consistent with the experiment. More details on the intrinsic reaction coordinate (IRC) results of transition states **TS4** and **TS5**, which were calculated by the different DFT methods in both implicit and explicit models, have been provided in Fig. S18 and S19 of the ESI.†

**Table 1:** Energy barriers for the proton transfer steps 4 and 5

Method	kcal mol^−1^
M06-2X(ΔΔ*G*_tot_[**TS4**-**IM3**])^a^	13.4
M06-2X(ΔΔ*G*_50%_[**TS4**-**IM3**])	13.0
M06-2X(ΔΔ*G*[**TS4explicit**-**IM3explicit**])^b^	11.3
B3LYP(ΔΔ*G*_tot_[**TS4B3LYP**-**IM3B3LYP**])^a^	12.1
B3LYP(ΔΔ*G*_50%_[**TS4B3LYP**-**IM3B3LYP**])	13.0
B3LYP(ΔΔ*G*[**TS4explicit**-**IM3explicit**])^b^	10.9
M06-2X(ΔΔ*G*_tot_[**TS5**-**IM4**])^a^	6.8
M06-2X(ΔΔ*G*_50%_[**TS5**-**IM4**])	7.9
M06-2X(ΔΔ*G*[**TS5explicit**-**IM4explicit**])^b^	6.4
B3LYP(ΔΔ*G*_tot_[**TS5B3LYP**-**IM4B3LYP**])^a^	7.0
B3LYP(ΔΔ*G*_50%_[**TS5B3LYP**/**IM4B3LYP**])	5.6

a The transition state **TS4/5** and intermediate **IM3/4** obtained from the IRC calculations were calculated at the level of M06-2X/6-31G(d,p)/IEF-PCM_THF_ or B3LYP/6-31G(d,p)/IEF-PCM_THF_.

b The transition state **TS4/5explicit** was first located in the sphere with a radius of 15 Å of the explicit solvents at the ONIOM(M06-2X/6-31G(d,p):UFF) or ONIOM(B3LYP/6-31G(d,p):UFF) levels. Then IRC calculation was performed to locate the corresponding intermediate **IM3/4explicit**.

### Roles of HOBt and DBU

**Fig. 4:**
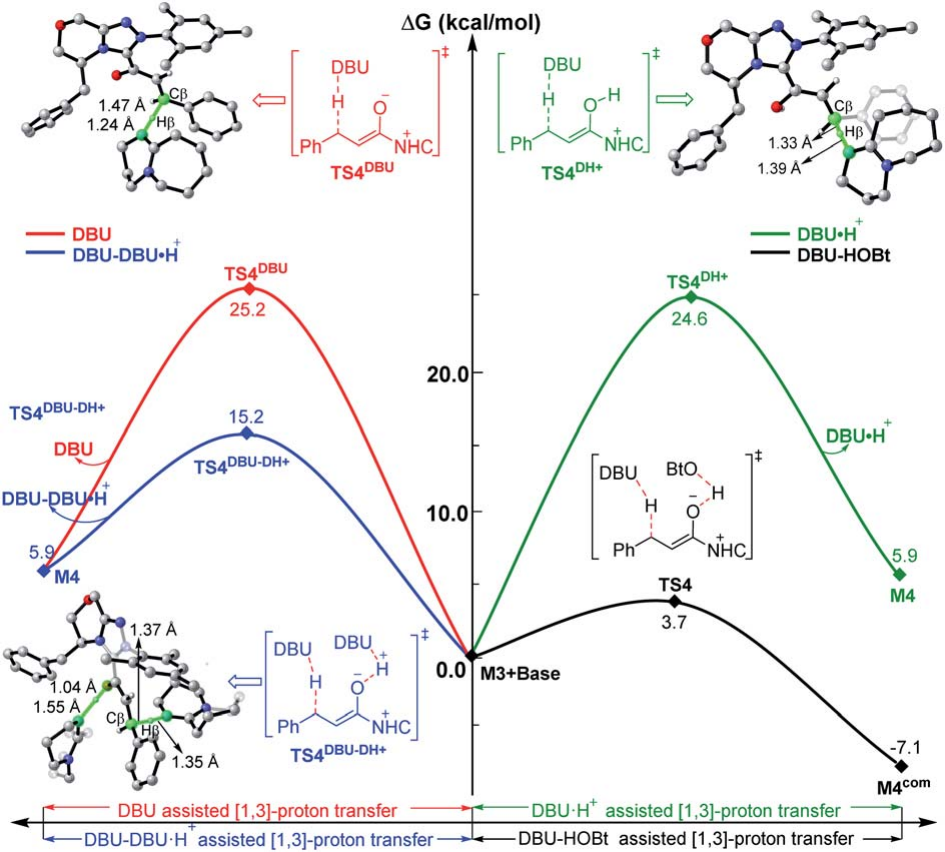
Relative Gibbs free energy profiles of different types of base-assisted [1,3]-proton transfer pathways, and the energies of the minima are relative to the energy of **M3** + base (0.0 kcal mol^−1^).

To explore the roles of the additive HOBt and base DBU, we have provided more evidence in both experiment and theory. As shown in Fig. 4, the DBU and HOBt cooperatively assisted [1,3]-proton transfer *via* transition state **TS4** (Δ*G*^‡^ = 3.7 kcal mol^−1^) is the most energetically favorable pathway among all the possible [1,3]-proton transfer pathways, including DBU and DBU·H^+^ cooperatively assisted [1,3]-proton transfer pathway *via***TS4DBU–DH+** (Δ*G*^‡^ = 15.2 kcal mol^−1^) and DBU or DBU·H^+^ separately assisted [1,3]-proton transfer pathway *via***TS4DBU** (Δ*G*^‡^ = 25.2 kcal mol^−1^)/**TS4DH+** (Δ*G*^‡^ = 24.6 kcal mol^−1^), implying that DBU serves as a base and the participation of HOBt can significantly lower the energy barrier of the key 1,3-proton transfer (*i.e.*, β-C–H deprotonation for the transformation from the Cα–Cβ single bond of saturated ester to the Cα=Cβ double bond), which thus causes the reaction to happen faster and improves the yield of the reaction. In order to prove this point, we have collaborated with Chi's group and the experimental results indicate that the yield indeed can be improved from 76% to 94% by adding the HOBt in the reaction (see Scheme 2).^31^ In contrast, when unsaturated ester is used as the reactant in the NHC-catalyzed [3 + 3] annulation for the formation of the same product,^14*a*^ the DFT calculations in Scheme S6 and Fig. S22 of the ESI† demonstrated that the addition of HOBt or other alcohols should be not necessary for improving reaction rate or yield, since the β-C–H deprotonation is not involved in the reaction, and the experimental observations also confirmed this conclusion. Hence, the participation of HOBt for the reaction is critical in promoting the reaction yield and enantioselectivity.

### Origin of chemoselectivity

For the other four proposed pathways divorced from the active intermediates **M2**, **M3**, and **M4**, the detailed discussions of their related energy profiles have been provided in the ESI† for the production of **SPA**/**SPB**, **SPC**, and **SPD**, as conjectured in Scheme 2. To pursue the origin of the chemoselectivity, we just need to compare the energy barriers involved in the several possible pathways: the possible amidation, ketolation, [2 + 2] cycloaddition, [3 + 2] annulation, and [3 + 3] annulation pathways.

**Fig. 5:**
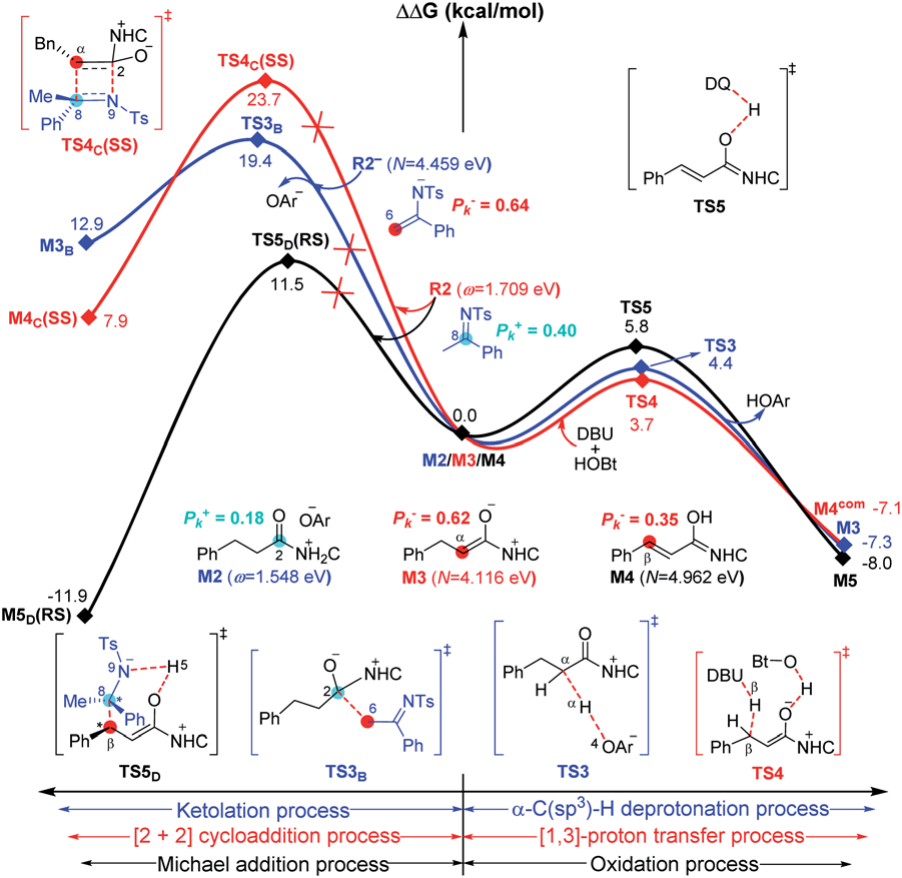
Gibbs free energy profiles for the possible processes between ketolation and α-C(sp^3^)–H deprotonation (blue line), [2 + 2] cycloaddition and [1,3]-proton transfer (red line), and Michael addition and oxidation (black line) processes involved in the four competing pathways.

Both of the amidation and ketolation pathways are divorced from **M2**, and the energy profile of the ketolation pathway lies below that of the amidation pathway, which is shown in Fig. S12 of the ESI.† Subsequently, we compared the other four possible pathways. As shown in Fig. 5, since **M2** (*ω* = 1.548 eV, *P*^+^_k_(C2) = 0.18) and **R2−** (*N* = 4.459 eV, *P*^−^_k_(C6) = 0.64) are separately electrophilic and nucleophilic, the nucleophilic addition of **R2−** onto **M2** through transition state **TS3B** is likely to happen. However, the much higher energy of **TS3B** in the ketolation pathway compared to that of **TS3** indicates that it is impossible to occur under the experimental conditions. In addition to the isomerization from enolate **M3** to Breslow intermediate **M4**, since enolate **M3** (*N* = 4.116 eV, *P*^−^_k_(Cα) = 0.62) and **R2** (*ω* = 1.709 eV, *P*^+^_k_(C8) = 0.40) are nucleophilic and electrophilic, respectively, the [2 + 2] cycloaddition between **M3** and **R2***via* transition state **TS4C(SS)** is likely to occur. However, the much higher energy of **TS4C(SS)** in the [2 + 2] cycloaddition pathway compared to that of **TS4** demonstrates the unfavorability of the corresponding pathway. In contrast to the oxidation of Breslow intermediate **M4**, since **M4** (*N* = 4.962 eV, *P*^−^_k_(Cβ) = 0.62) and **R2** (*ω* = 1.709 eV, *P*^+^_k_(C8) = 0.40) are nucleophilic and electrophilic, respectively, we have located a transition state **TS5D(RS)** for the nucleophilic addition of **M4** onto **R2** combined with a proton transfer. The energy barrier of the single-step reaction *via***TS5D(RS)** in the [3 + 2] annulation pathway is 11.5 kcal mol^−1^ and not high. However, as summarized in Table S9 of the ESI,† the energy barriers of the four possible reactions (Δ*G*^‡^_total_) *via***TS3B**, **TS4C(SS)**, **TS5D(RS)**, and **TS6(R)HOBt** are 31.0, 28.0, 24.5, and 9.9 kcal mol^−1^, hence, only the product **P** can be formed in theory, which is consistent with the experimental observations.^31^

**Fig. 6:**
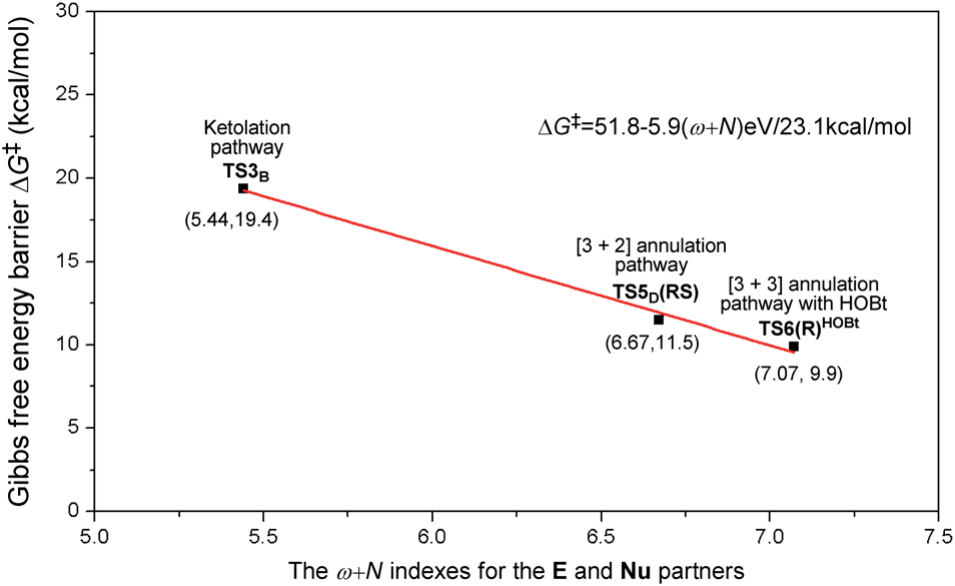
The linear relationship between the single-step energy barriers, Δ*G*^‡^, (unit: kcal mol^−1^) of chemoselective transition states and their corresponding *ω* + *N* indexes (unit: eV) of the E and Nu partners.

As is well accepted by chemists, for the nucleophilic or electrophilic addition processes, if the two reacting partners have much stronger nucleophilicities or electrophilicities, their corresponding transition states would be related to lower energy barriers. Therefore, based on the empirical point, to uncover the origin of this reaction chemoselectivity, we assumed that the larger electrophilic (*ω*) and nucleophilic (*N*) indexes *ω* + *N* of the stable nucleophile (Nu) → electrophile (E) partners (**R2−** → **M2**, **M4** → **R2**, and **R2−HOBt** → **M5**) would lead to lower energy barriers, Δ*G*^‡^, of the nucleophilic or electrophilic addition processes associated with the transition states (**TS3B**, **TS5D(RS)**, and **TS6(R)HOBt**) involved in the three pathways. As summarized in Table S9 of the ESI,† the decreasing single-step energy barriers, Δ*G*^‡^, are presented a linear relationship with the corresponding increasing *ω* + *N* indexes as shown in Fig. 6. It should be noted that the energy barrier *via* a four-membered ring transition state **TS4C(SS)** does not completely correspond to the activity of the reaction partners (**M3** → **R2**), which is due to the large ring strain of the four-membered ring involved in **TS4C(SS)**; thus we did not consider its data for the linear relationship depicted in Fig. 6.

Considering the above, we suggested a new application of the *ω* + *N* index from the reacting ability between the Nu and E to simply and quickly predict the energy barrier of the chemoselective step (Δ*G*^‡^_p_ = 51.8–5.9(*ω* + *N*) eV/23.1 kcal mol^−1^), which would be one of the key factors for exploring the origin of chemoselectivity of the possible reactions commonly involved in a large amount of NHC-catalyzed reactions of saturated or unsaturated carbonyl compounds.

### General principle for predicting the chemoselectivity of NHC-mediated reactions of carbonyl compounds

**Fig. 7:**
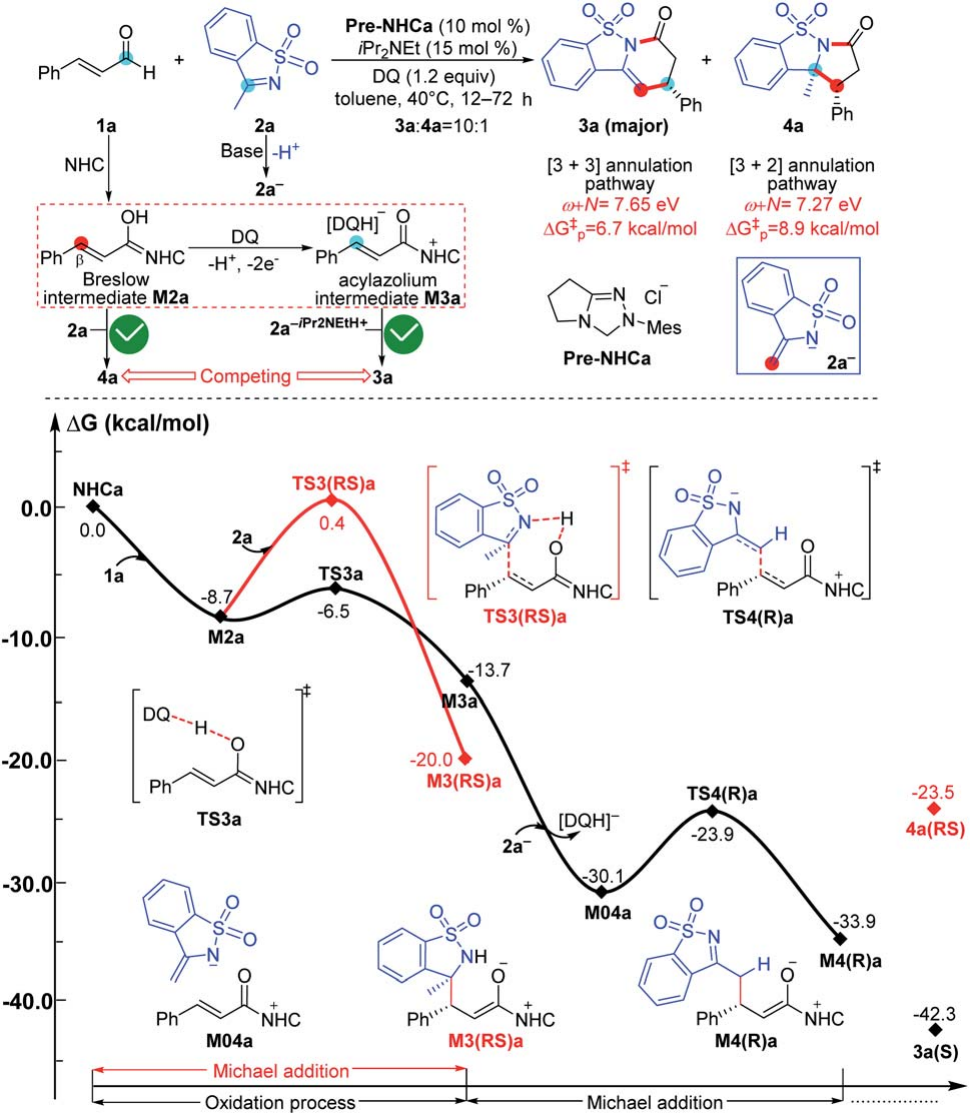
Relative Gibbs free energy profiles of NHC-catalyzed chemoselective [3 + *n*] (*n* = 2, 3) annulation of enals with imines. (“Δ*G*^‡^_p_” is calculated by the correlation Δ*G*^‡^ = 51.8–5.9(*ω* + *N*) eV/23.1 kcal mol^−1^ and represents the predicted energy barrier of nucleophilic or electrophilic addition.)

**Fig. 8:**
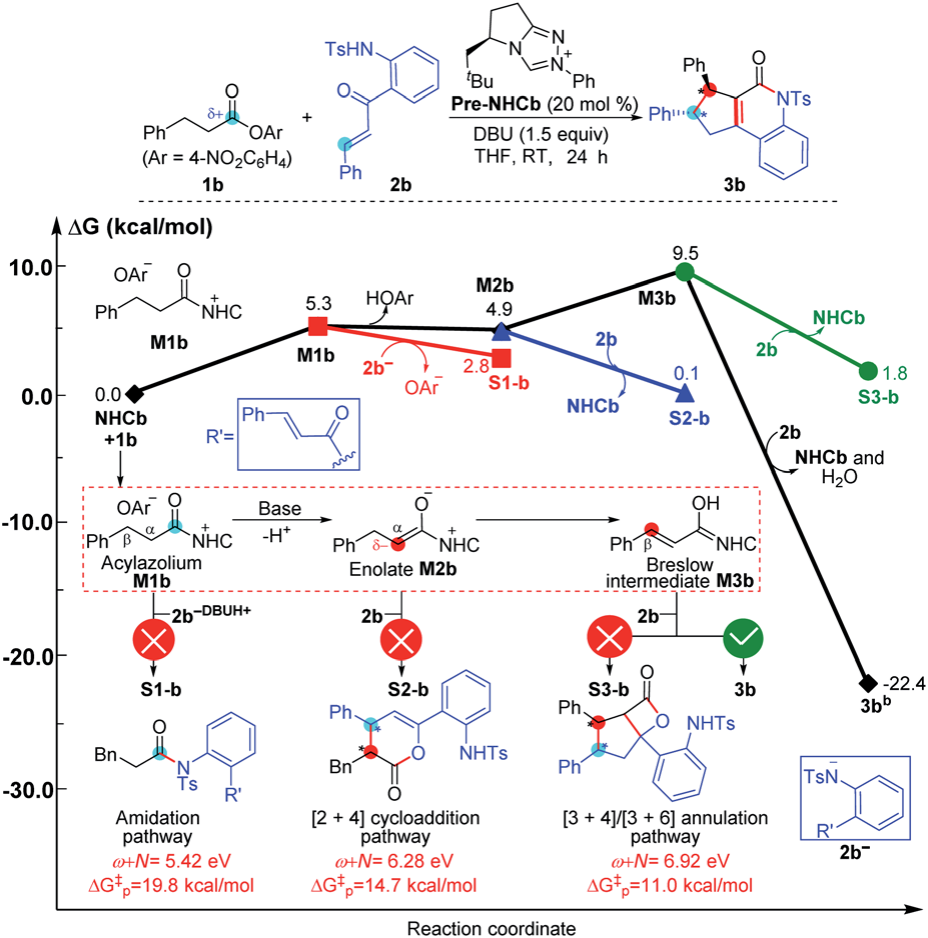
Relative Gibbs free energy profiles of NHC-catalyzed reactions of saturated carboxylic esters with *o*-tosylamino enones.

**Fig. 9:**
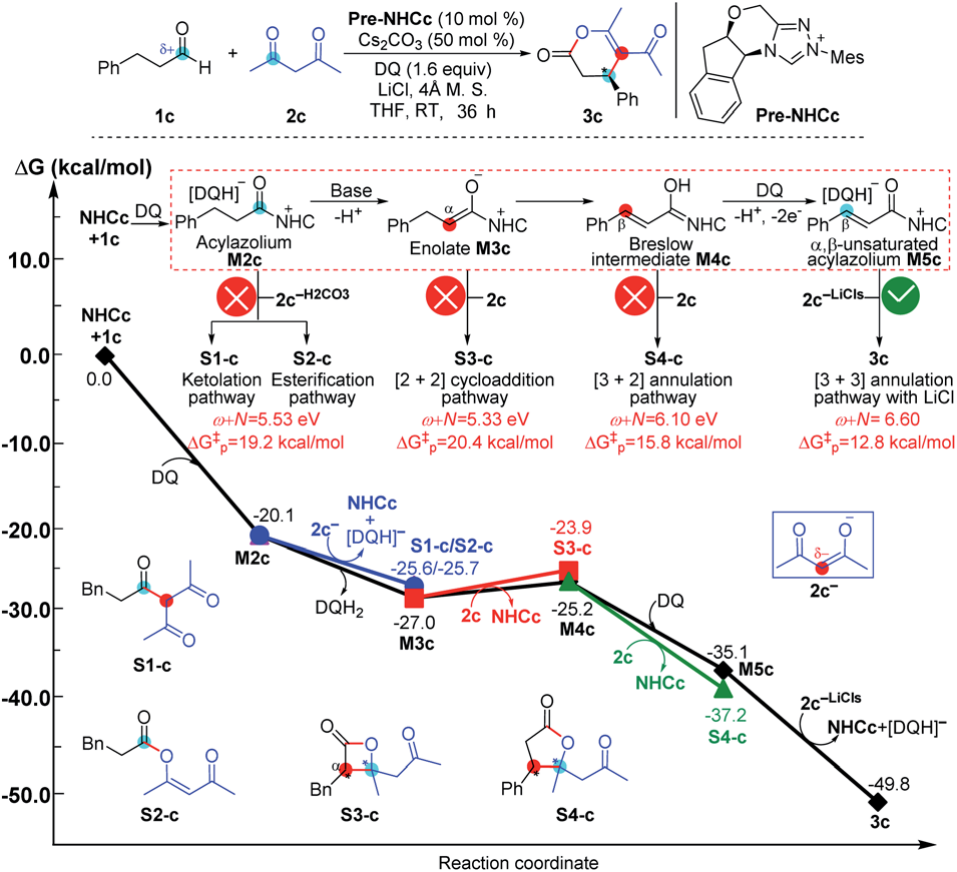
Relative Gibbs free energy profiles of NHC-catalyzed reactions of simple aldehydes with acetylacetones.

To ensure that the *ω* + *N* index of Nu and E partners can be used to generally predict the single-step energy barriers and even the chemoselectivity in these types of reactions based on our proposed general mechanistic map, we selected three additional NHC-mediated reactions of carbonyl compounds presented in Fig. 7–9 as valuable cases for testing.^13*j*,14*b*,17*a*^

As shown in Fig. 7, the NHC-catalyzed chemoselective [3 + *n*] (*n* = 2, 3) annulation of enal with imine was selected as one special case,^13*j*^ and two competing pathways including the [3 + 2] and [3 + 3] annulation pathways were considered according to the general mechanistic map suggested in Scheme 1. The relevant *ω* + *N* indexes of the Nu and E partners are 7.27 and 7.65 eV, which correspond to the predicted energy barriers of 8.9 and 6.7 kcal mol^−1^, respectively, based on the correlation Δ*G*^‡^_p_ = 51.8–5.9(*ω* + *N*) eV/23.1 kcal mol^−1^ depicted in Fig. 6. Apparently, the predicted energy barriers are close to the calculated energy barriers (9.1 and 6.2 kcal mol^−1^, Fig. 7) for the Michael addition process in producing products **3a** and **4a**. Both of the two transformations should be irreversible, so the chemoselectivity mainly be controlled by kinetics. In kinetics, the [3 + 3] annulation pathway for affording **3a** is much more energetically favorable than the [3 + 2] annulation pathway for generating **4a**, which is in agreement with the experimental results that the ratio of **3a** : **4a** is 10 : 1.^13*j*^

As depicted in Fig. 8, the NHC-catalyzed reactions of saturated carboxylic esters with *o*-tosylamino enones probably proceed through the following four possible pathways: the amidation, [2 + 4] cycloaddition, [3 + 4], and [3 + 6] annulation pathways. The corresponding *ω* + *N* indexes of the Nu and E partners in the four pathways are equal to 5.42, 6.28, 6.92, and 6.92 eV, which indicates that the corresponding Δ*G*^‡^ would be relatively lower in the [3 + 4]/[3 + 6] annulation pathway. In thermodynamics, the energy of **3b** is much lower than those of **S1-b**, **S2-b**, and **S3-b**, and only the transformation to product **3b** is irreversible at room temperature. In summary, we can predict that the formation of **3b** through the [3 + 6] annulation pathway is more favorable in both kinetics and thermodynamics. Hence, only the product **3b** can be formed in theory, which is in agreement with the experimental observations.^14*b*^

As shown in Fig. 9, the NHC-catalyzed reaction of simple aldehydes with acetylacetones was selected as another case, and five pathways, including the ketolation/esterification, [2 + 2] cycloaddition, [3 + 2] annulation, and [3 + 3] annulation pathways with LiCl, were proposed based on the general mechanistic map (Scheme 1). The relevant *ω* + *N* indexes of the Nu and E partners correspond to 5.53, 5.33, 6.10, and 6.60 eV, and the energy of main product **3c** was found to be much lower than those of **S1-c**, **S2-c**, **S3-c**, and **S4-c**. The above analyses imply that the [3 + 3] annulation pathway with LiCl for affording **3c** is more energetically favorable than the other four pathways for generating **S1-c**, **S2-c**, **S3-c**, and **S4-c** in both kinetics and thermodynamics. Hence, the main product should be **3c** in theory, which is still in agreement with the results observed in experiments.^17*a*^ All the side products were proposed according to the experimental references,^13*j*,15*a*,37^ and the justifications of the side products were provided in Schemes S7–S9 of the ESI.†

The reactivity indices could be useful for catalyst screening, but we still suggest that the searching of the transition state should be necessary to explore the origin of chemo- and stereo-selectivities. Noteworthy, we have also computed the key transition states involved in the multiple pathways for generating different products in the reaction models depicted in Fig. 8 and 9. As shown in Fig. S23 of the ESI,† the order of the calculated Gibbs free energy barrier (*i.e.*, Δ*G*^‡^(**TS4b**) = 11.2 kcal mol^−1^ < Δ*G*^‡^(**S2-TS3b**) = 18.3 kcal mol^−1^ < Δ*G*^‡^(**S1-TS2b**) = 32.6 kcal mol^−1^) is the same with that of their predicted values (*i.e.*, Δ*G*^‡^_p_ ([3 + 4]/[3 + 6] annulation pathway) = 11.0 kcal mol^−1^ < Δ*G*^‡^_p_ ([2 + 4] cycloaddition pathway) = 14.7 kcal mol^−1^ < Δ*G*^‡^_p_ (amidation pathway) = 19.8 kcal mol^−1^), indicating that the conclusion on the origin of chemoselectivity should be reliable. Meanwhile, a similar conclusion can be obtained from the calculated results in Fig. S24 of the ESI;† the computed energy barriers of the transition states can be used to explain the chemoselectivity well.

## Conclusions

In summary, the introduction of an external base or oxidant indeed increases the complication of the reaction mechanism for the four NHC-mediated reactions of carbonyl compounds in theory, as the additives can reverse the electronic properties of NHC-involved active intermediates, *i.e.*, acylazolium intermediate, enolate, Breslow intermediate, or α,β-unsaturated acylazolium intermediate.

As revealed by the DFT calculations of the NHC-mediated transformations, the nucleophilic enolate is obtained from the base-assisted deprotonation of the electrophilic acylazolium intermediate. The nucleophilic Breslow intermediate is generated from a base and protic media cooperatively assisted isomerization of a nucleophilic enolate, while the electrophilic α,β-unsaturated acylazolium intermediate is produced from the oxidation of a nucleophilic Breslow intermediate by an oxidant. These active intermediates could probably undergo multiple competing pathways, including the amidation/ketolation, [2 + *n*] (*n* = 2, 4) cycloaddition, and [3 + *n*] (*n* = 2, 4, 6) annulation pathways, when they react with other nucleophilic (Nu) or electrophilic (E) partners. This could lead to the generation of chemoselective products and greatly increases the number of theoretical calculations. Hence, we suggested an exact mechanistic map and a simple rule by merely computing the *ω* + *N* indexes of the stable E and Nu partners and relative energies of intermediates and products to predict the energy barriers *via* the chemoselective transition states, and even predict the origin of chemoselectivity to significantly reduce the number of theoretical computations.

Notably, this simple rule has been successfully used in several cases of NHC-mediated reactions of saturated/unsaturated esters or aldehydes. Finally, we hope that the obtained insights will facilitate rational design according to the prediction of organocatalytic reactions with special chemoselectivities. Therefore, this work provides a theoretical method for searching and identifying the active intermediates, possible pathways, and even main products in NHC chemistry.

## Conflicts of interest

There are no conflicts to declare.

## Supplementary Material

SC-011-D0SC01793K-s001
