# Supplementary material for: Prediction of NHC-catalyzed chemoselective functionalizations of carbonyl compounds: a general mechanistic map
Source: Chem Sci. 2020 Jun 22;11(27):7214–25. doi: 10.1039/d0sc01793k (PMC8159411; doi:10.1039/d0sc01793k)
Supplement: SC-011-D0SC01793K-s001 [file SC-011-D0SC01793K-s001.pdf]

Electronic Supplementary Material (ESI) for Chemical Science.
This journal is © The Royal Society of Chemistry 2020

Supporting Information for

Prediction of NHC-catalyzed chemoselective functionalizations of carbonyl compounds: A general mechanistic map

Xue Li,[a] Jun Xu,[b] Shi-Jun Li,[a] Ling-Bo Qu,[a] Zhongjun Li,[a] Yonggui Robin Chi,[c] Donghui Wei*[a] and Yu Lan,*[a,d]

[a]College of Chemistry, Institute of Green Catalysis, Zhengzhou University, 100 Science Avenue, Zhengzhou, Henan 450001, China
[b]College of Pharmacy, Guizhou University of Traditional Chinese Medicine, Guiyang, China
[c]Division of Chemistry & Biological Chemistry, School of Physical & Mathematical Sciences, Nanyang Technological University, Singapore 637371, Singapore
[d]College of Chemistry and Chemical Engineering, Chongqing University, Chongqing 400044, China
E-mail: donghuiwei@zzu.edu.cn (D. W.); lanyu@cqu.edu.cn (Y. L.).

Table of Contents

1. Computational details for ee value and other test S1
2. Additional computational results S1
   2.1 Base-assisted deprotonation pathways of precursor Pre-NHC S1
   2.2 Different base-assisted α-C(sp^3)−H bond deprotonation pathways S2
   2.3 Different base-assisted [1,3]-proton transfer pathways S3
   2.4 Five possible pathways for the oxidation process S5
   2.5 The possible intermolecular SET processes S9
   2.6 Different deprotonation pathways of R2 .... S9
   2.7 Other possible pathways for the formations of P/SP_A/SP_B/SP_C/SP_D S10
   2.8 A conformational search of TS6 .... S15
   2.9 Origin of stereoselectivity S16
   2.10 Test results of computational methods S17
   2.11 The possible pathways for [1,3]-proton transfer of step 7 .... S18
   2.12 Roles of NHC and DQ S20
   2.13 Analyses of other cases S22
3 Cartesian coordinates and energy values of optimized structures S27
   3.1 Optimized geometries of the stationary points S27
   3.2 Absolute SPE and GFE of the optimized structures by M06-2X S28
   3.3 Absolute SPE and GFE of the optimized structures by other DFT methods S32
   3.4 Absolute SPE and GFE of the optimized structures for other cases S33
   3.5 The Cartesian coordinates of all the stationary points S36
   3.6 The frequencies of all the transition states S836

1. Computational details for ee value and other test

In addition, the extent of enantioselectivity, in terms of enantiomeric excess (ee %), was calculated by using the Boltzmann distribution of diastereomeric transition states with the following equations:

$$\frac{[R]}{[S]}=\frac{\exp\left(-\Delta G^{\ddagger}_{[R]}/RT\right)}{\exp\left(-\Delta G^{\ddagger}_{[S]}/RT\right)}=\exp\left(\Delta\Delta G^{\ddagger}/RT\right)$$

$$ee=\frac{[R]-[S]}{[R]+[S]}\times100\%=\frac{\frac{[R]}{[S]}-1}{\frac{[R]}{[S]}+1}\times100\%$$

where $\Delta G^{\ddagger}$ is the Gibbs free energy barrier of the competing diastereomeric transition state and $\Delta\Delta G^{\ddagger}$ is the Gibbs free energy difference between the two diastereomeric transition states.

As summarized in **Table S1**, the relative energies of the generated zwitterionic species **TS1$^{\mathbf{HOBt}}$** and **M1$^{\mathbf{HOBt}}$** in the presence of HOBt are almost the same as those of **TS1** and **M1** in the absence of HOBt, so it should be not necessary to consider the role of H-donor in all the reaction steps. Noteworthy, our calculated results indicate that H-donor (*i.e.*, HOBt) can indeed significantly affect the C–H deprotonation and stereoselectivity-determining processes, in which we have already added the HOBt in the model.

Table S1 Relative Energies (kcal/mol) of **TS1** and **M1** with/without the presence of HOBt

Without HOBt		With HOBt	
	ΔG		ΔG
**NHC+R1**	0.0	**NHC+R1$^{\mathbf{HOBt}}$**	0.0
**TS1**	16.5	**TS1$^{\mathbf{HOBt}}$**	15.6
**M1**	13.4	**M1$^{\mathbf{HOBt}}$**	14.1

2. Additional computational results

2.1 Base-assisted deprotonation pathways of precursor Pre-NHC

To determine whether the free carbenes are involved in the catalytic reaction, based on the previous work (*Angew. Chem. Int. Ed.* 2017, *56,* 16395-16398), we have additionally considered and compared two different reaction pathways, *i.e.*, the non-free-carbene pathway and carbene generation pathway. In the non-free-carbene pathway, the **pre-NHC** catalyst can be deprotonated by base DBU, which is coupled with the C–C bond formation between the catalyst with substrate *via* transition state **TS0$^{\mathbf{DBU+R1}}$** (**Fig. S1**). However, we have tried but failed to locate the transition state **TS0$^{\mathbf{DBU+R1}}$**, herein we only can obtain the transition state **TS0$^{\mathbf{DBU+R1}}$** (**Fig. S1**), which

seems to be same with the carbene generation transition state with a very low free energy barrier (2.7 kcal/mol). In the model without the presence of the substrate, the carbene generation transition state **TS0$^{\mathbf{DBU}}$** (**Fig. S1**) can also be located, and has an energy barrier of 0.2 kcal/mol. Therefore, we think that the free carbene can be easily generated in this reaction system. In addition, the carbene carbon has also been confirmed as the real catalytic site in the previous reference (see *J. Mol. Catal. A Chem.* 2011, 334, 108-115).

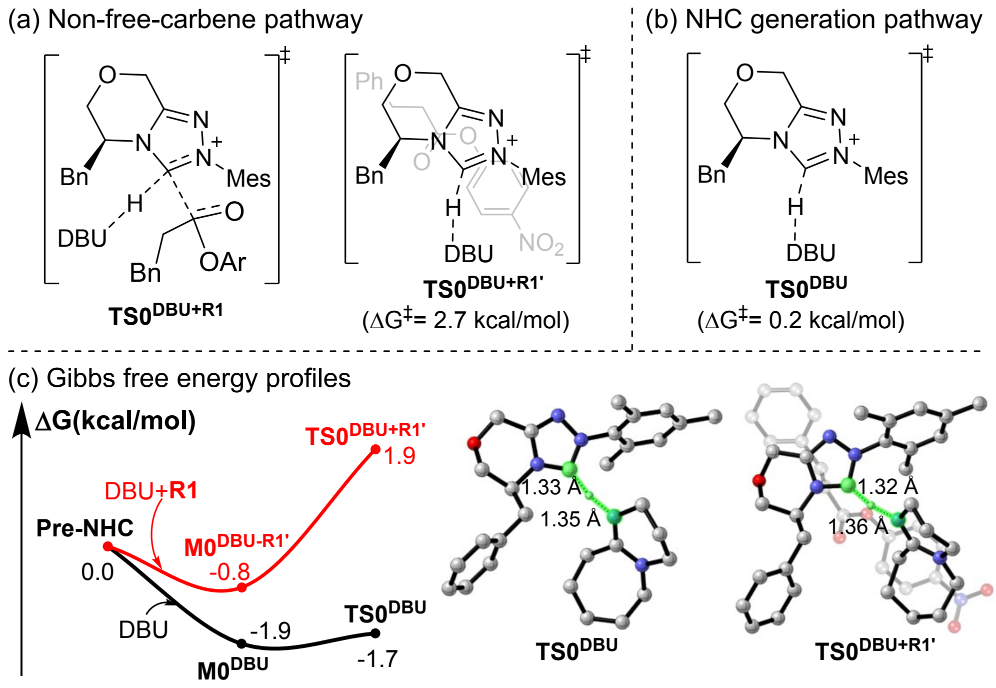

Fig. S1 Relative Gibbs free energy profiles of the non-free-carbene pathway and NHC generation pathway for the deprotonation of precursor **Pre-NHC** *via* the related transition states.

2.2 Different base-assisted α-C(sp³)−H bond deprotonation pathways

As shown in **Figs. S2-3**, the α-C(sp³)−H bond can be deprotonated by two bases, *i.e.*, DBU and OAr⁻, through the transition states **TS3$^{\mathbf{DBU}}$/TS3E$^{\mathbf{DBU}}$** and **TS3/TS3E**, respectively. Obviously, the DBU-assisted α-C(sp³)−H deprotonation pathway *via* **TS3$^{\mathbf{DBU}}$/TS3E$^{\mathbf{DBU}}$** ($\Delta G^{\ddagger}$ = 7.3/12.1 kcal/mol) is less energetically favorable than the OAr⁻-assisted α-C(sp³)−H deprotonation pathway *via* **TS3/TS3E** ($\Delta G^{\ddagger}$ = 4.4/8.6 kcal/mol), indicating that DBU is not involved in the α-C(sp³)−H deprotonation process.

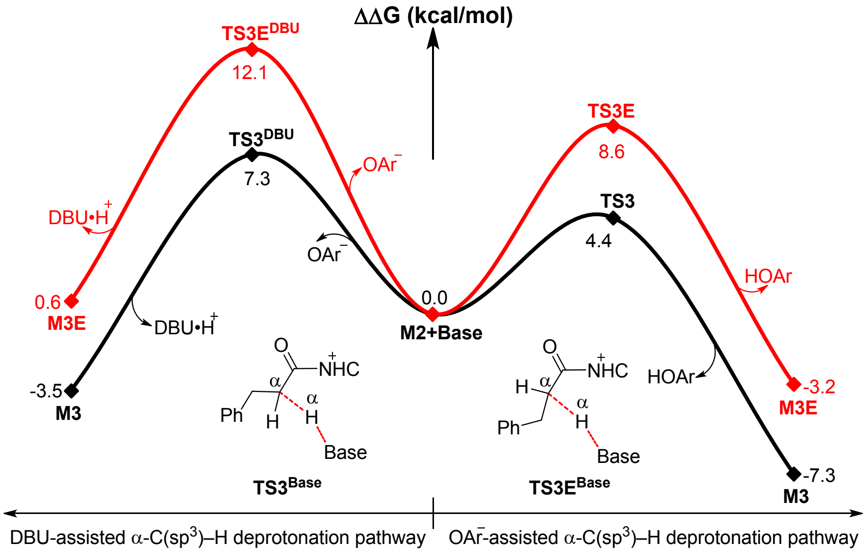

Fig. S2 Relative Gibbs free energy profiles of the DBU-assisted and OAr⁻-assisted α-C(sp^3)−H deprotonation pathways, and the energies of the minima are relative to the energy of **M2**+Base (0.0 kcal/mol).

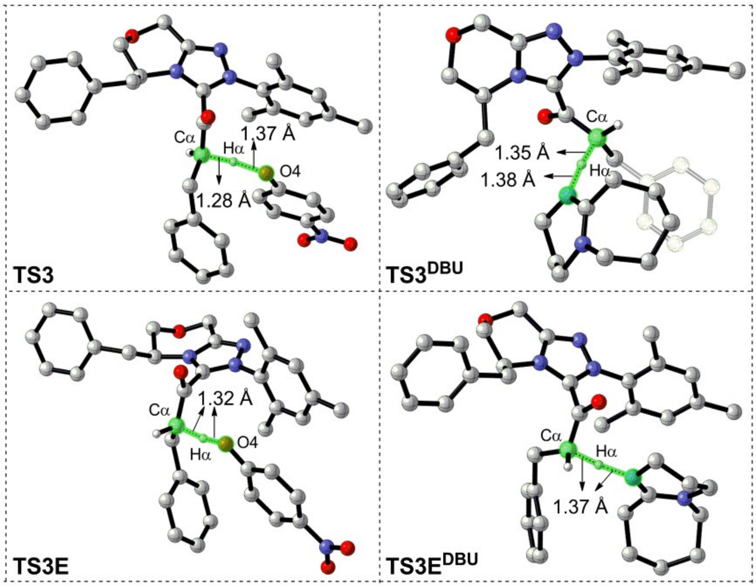

Fig. S3 Optimized geometries of all the α-C(sp^3)−H deprotonation transition states; the hydrogen atoms that are not involved in the reaction have been omitted. Distances are in angstroms.

2.3 Different base-assisted [1,3]-proton transfer pathways

As shown in **Figs. S4-5**, the [1,3]-proton transfer occurs along with the protonation of enol oxygen atom. This process can be assisted by several bases/protic media, *i.e.*, DBU, DBU·H^+, HOAr, HOBt, DBU cooperated with DBU·H^+, or DBU cooperated with HOBt, through the transition states **$TS4^{DBU}$/$TS4Z^{DBU}$**, **$TS4^{DH+}$/$TS4Z^{DH+}$**, **$TS4^{HOAr}$/$TS4Z^{HOAr}$**, **$TS4^{HOBt}$/$TS4Z^{HOBt}$**, **$TS4^{DBU\text{-}DH+}$/$TS4Z^{DBU\text{-}DH+}$**, and **TS4/TS4Z**, respectively. Obviously, the energy barriers of 3.7/9.7 kcal/mol

via **TS4/TS4Z** are lower than those of 14.8~31.1 kcal/mol *via* **TS4^{DBU}/TS4Z^{DBU}**, **TS4^{DH+}/TS4Z^{DH+}**, **TS4^{HOAr}/TS4Z^{HOAr}**, **TS4^{HOBt}/TS4Z^{HOBt}**, and **TS4$^{D\text{-}DH+}$/TS4Z$^{D\text{-}DH+}$**, respectively. Hence, the DBU and HOBt cooperatively assisted pathway is the most preferential among the six distinctive types of protic media assisted [1,3]-proton transfer pathways, and the remaining ones can be safely excluded.

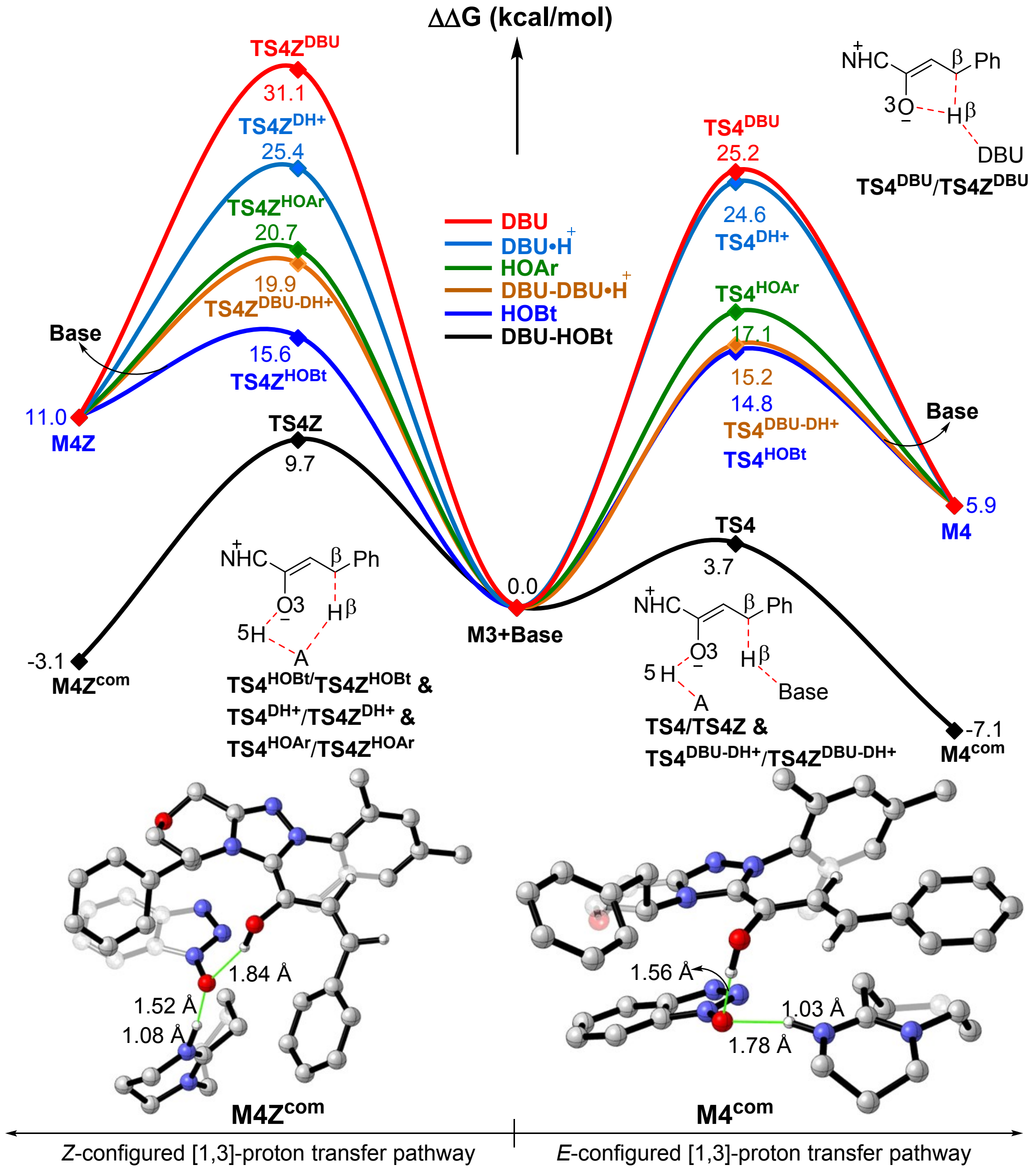

Fig. S4 Relative Gibbs free energy profiles of six different types of base-assisted [1,3]-proton transfer pathways, and the energies of the minima are relative to the energy of **M3**+Base (0.0 kcal/mol).

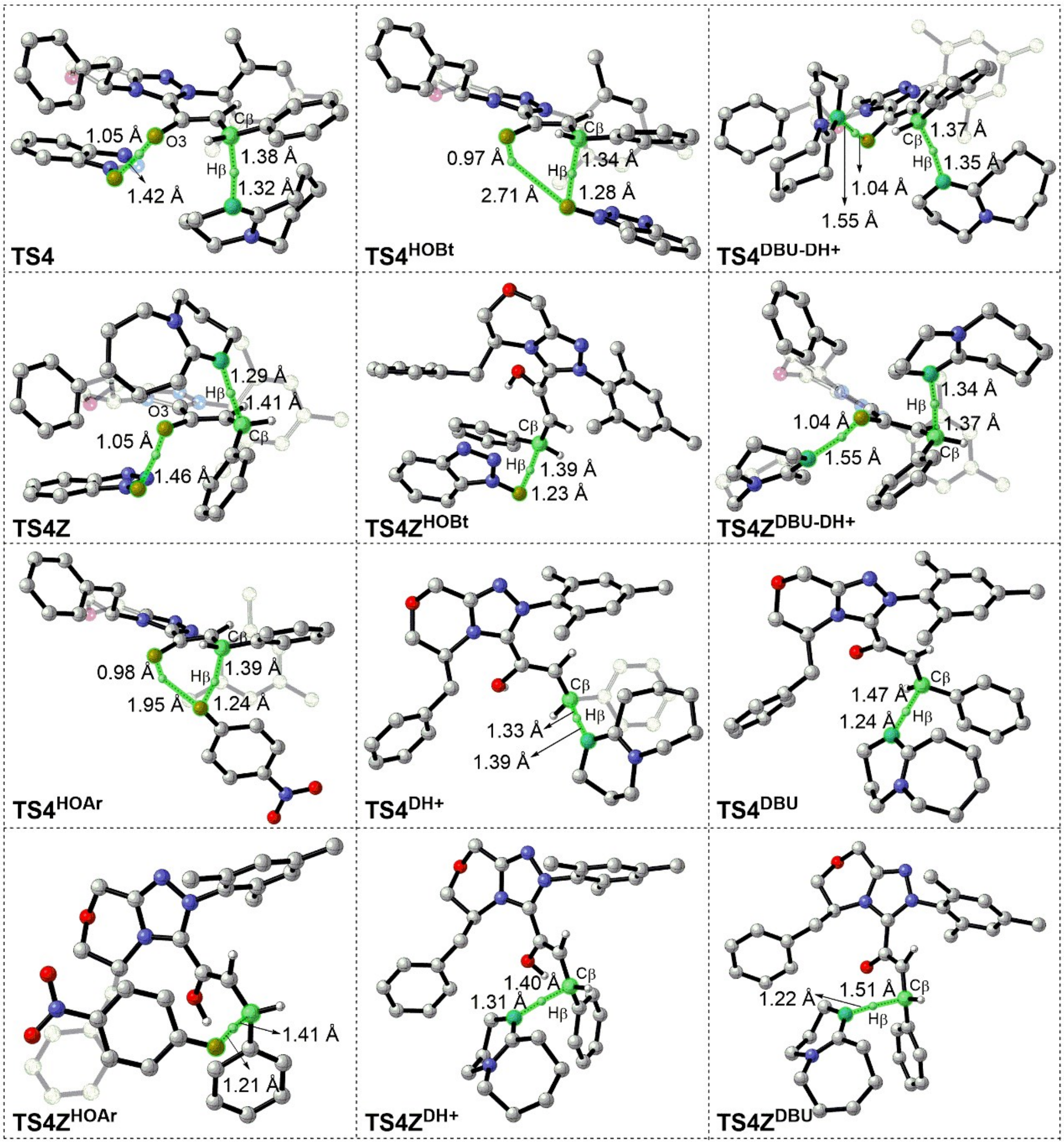

Fig. S5 Optimized geometries of all the [1,3]-proton transfer transition states; the hydrogen atoms that are not involved in the reaction have been omitted. Distances are in angstroms.

2.4 Five possible pathways for the oxidation process

As discussed in the introduction, we have considered and investigated the other three possible pathways (*i.e.*, the HTC, SET-HAT, and HAT-SET pathways). As presented in **Schemes S1-2**, in the HTC pathway, the hydride H5 can transfer from the O3 atom in **M4** to four different types of carbon (*i.e.*, C2', C3', C4', and C5') atom of DQ to give the intermediate $\mathbf{M5_{HTC}}$ *via* the corresponding transition states $\mathbf{TS5_{HTC}}^{\mathbf{C2'}}$ ($\Delta G^{\ddagger}$ = 42.6 kcal/mol, **Fig. S6**), $\mathbf{TS5_{HTC}}$ ($\Delta G^{\ddagger}$ = 22.2 kcal/mol, **Fig. S6**), $\mathbf{TS5_{HTC}}^{\mathbf{C4'}}$ ($\Delta G^{\ddagger}$ = 33.5 kcal/mol, **Fig. S6**), and $\mathbf{TS5_{HTC}}^{\mathbf{C5'}}$ ($\Delta G^{\ddagger}$ = 17.1 kcal/mol, **Fig. S6**), respectively. Therefore, all the four HTC pathways are much higher than the HTO pathway *via* **TS5** ($\Delta G^{\ddagger}$ = 5.8 kcal/mol, **Fig. 1**) and can be safely excluded.

For the SET-HAT and HAT-SET pathways, each of them contains two processes. In the

SET-HAT pathway, the first process is a single electron transfer from **M4** to DQ in forming the intermediate radical **M5**$_{\mathbf{SET}}$ and the DQ•− radical, which needs to overcome a relative high Gibbs free energy (11.8 kcal/mol) to accomplish the SET process in contrast to that of the HTO process. So it is unnecessary to consider and investigate the following HAT process, and the SET-HAT pathway can be ignored. In the HAT-SET pathway, the first process is a HAT process releasing 10.1 kcal/mol Gibbs free energy to render the radical intermediate **M5**$_{\mathbf{HAT}}$ and the [DQH]· radical, which is followed by a single electron transfer from the radical **M5**$_{\mathbf{HAT}}$ to the [DQH]· radical. The HAT process is exothermic, indicating this process is easy to happen. Whereas, the SET process needs to absorb a relative high Gibbs free energy of 8.8 kcal/mol, which is higher than the energy barrier *via* **TS5** ($\Delta G^{\ddagger}$ = 5.8 kcal/mol, **Scheme S1**). Hence, the HAT-SET pathway can be neglected.

Scheme S1 Four possible pathways for the oxidation process (energy unit in kcal/mol)

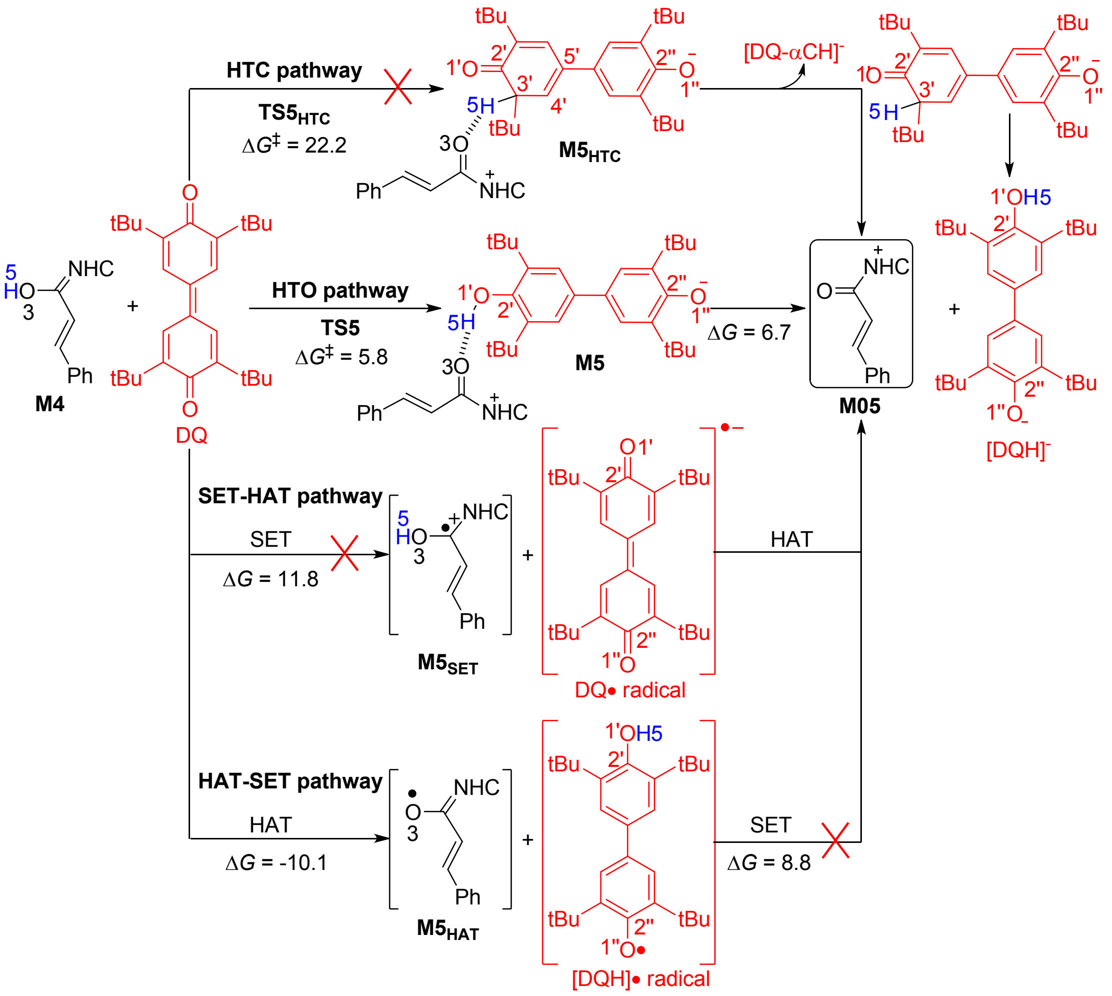

Scheme S2 The other possible HTC pathways for the oxidation process (energy unit in kcal/mol)

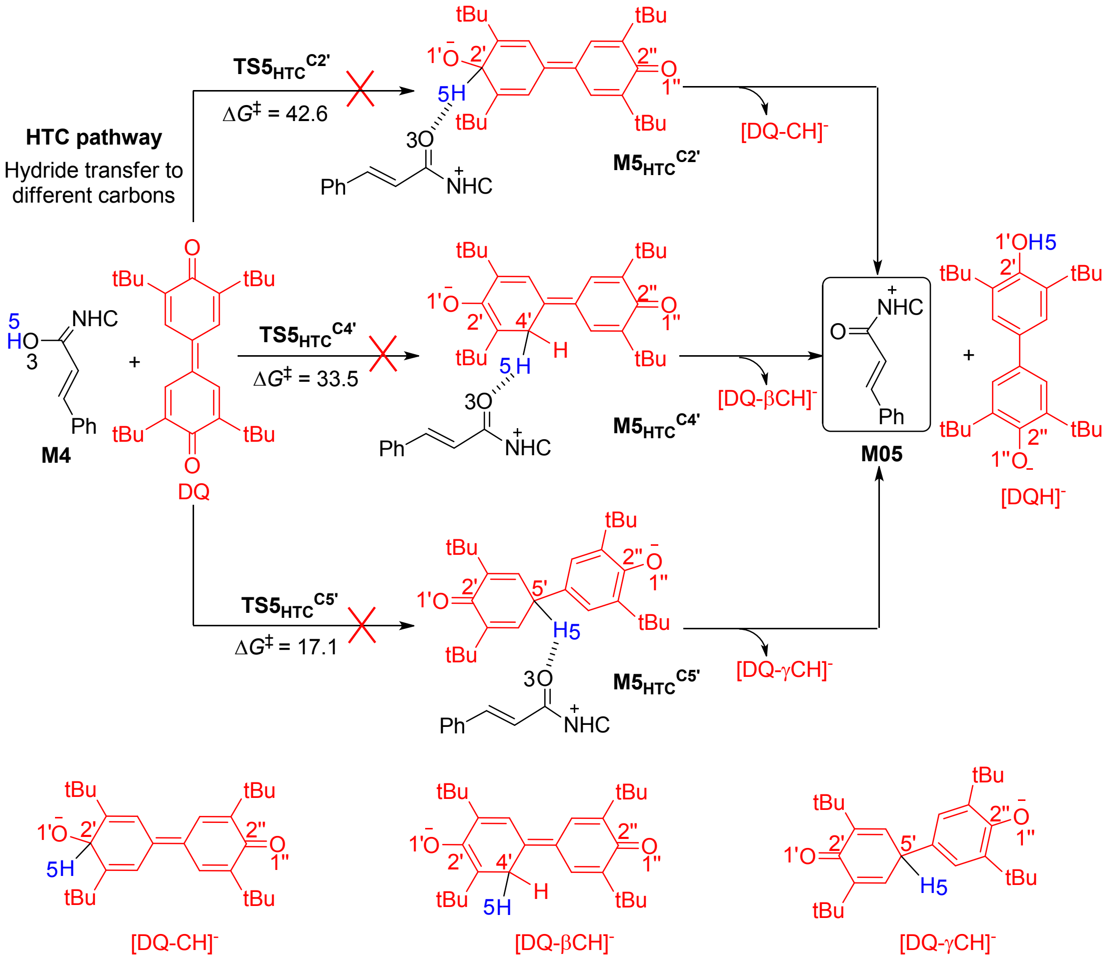

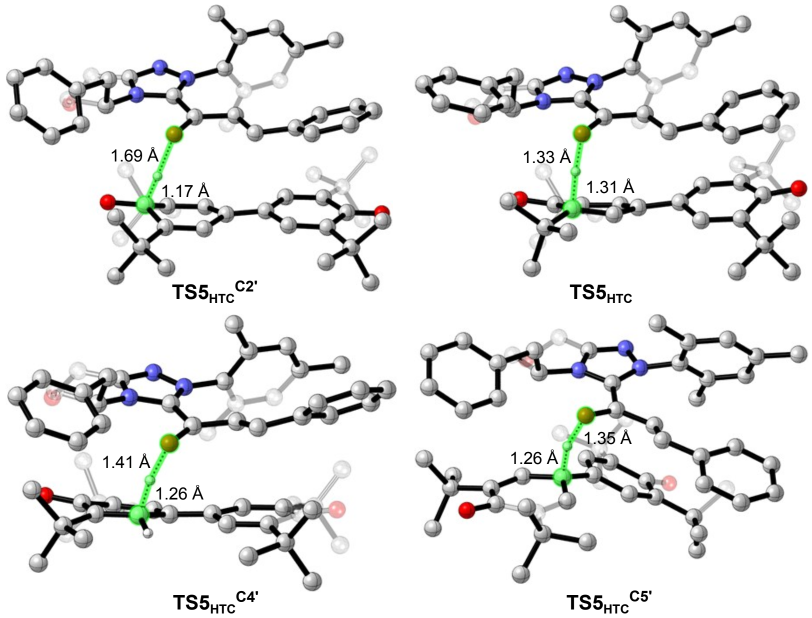

Fig. S6 Optimized geometries of the transition states for the possible HTC pathways of the oxidation process calculated at the L1 level of theory. Most of the hydrogen atoms are omitted for the sake of clarity.

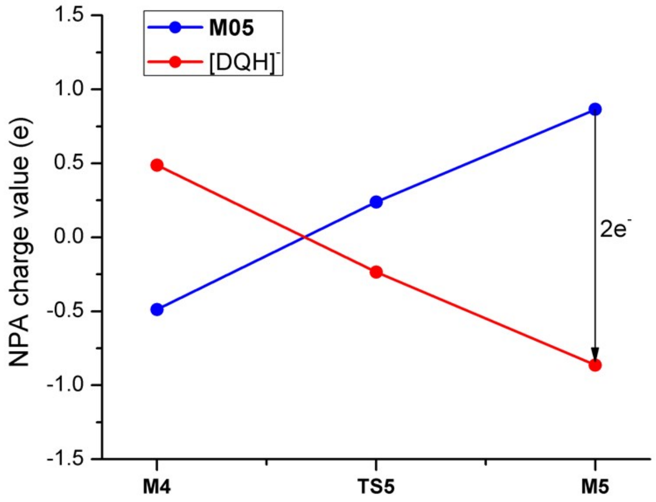

Fig. S7 The NPA charge evolution during the oxidation process by DQ (**M05** and [DQH]- refer to the fragments involved in **M4**, **TS5**, and **M5**, respectively.)

As depicted in **Scheme S3**, after the α-C(sp^3)−H deprotonation, we assumed that the enolate **M3** can be oxidized by DQ through the HTO pathways *via* transition states **TS4-5°/TS4-5Z^{O}** in affording α,β-unsaturated acylazolium intermediates **M05/M05Z**. The energy barriers of the HTO pathways *via* **TS4-5°/TS4-5Z^{O}** ($\Delta G^{\ddagger}$ = 37.1/38.6 kcal/mol, **Fig. S8**) are extremely higher than those of the DBU and HOBt cooperatively assisted [1,3]-proton transfer pathways *via* **TS4/TS4Z** ($\Delta G^{\ddagger}$ = 3.7/9.7 kcal/mol, **Fig. 1**), so the two oxidation pathways are impossible to happen under the experimental conditions and can be safely excluded.

Scheme S3 The possible oxidation pathway for the transformation from enolate to α,β-unsaturated acylazolium intermediate in a one-step (energy unit in kcal/mol)

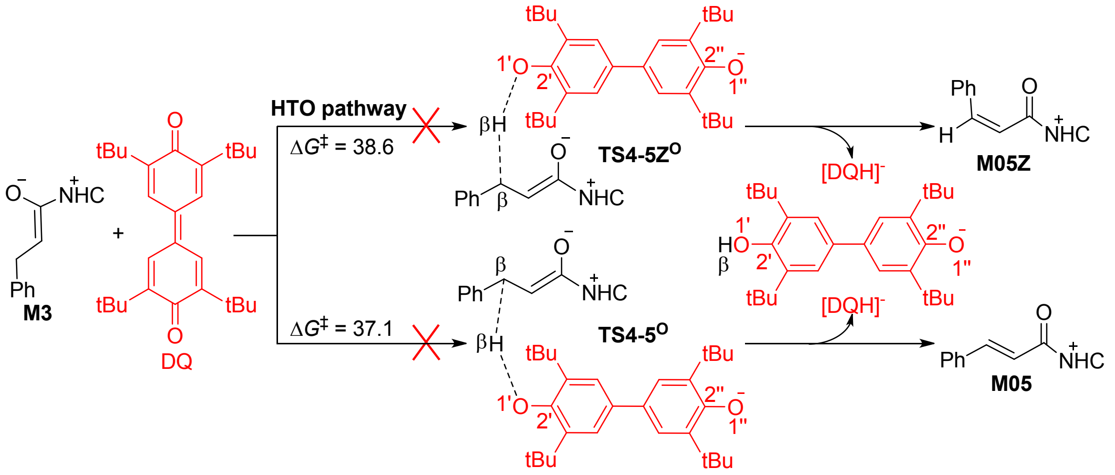

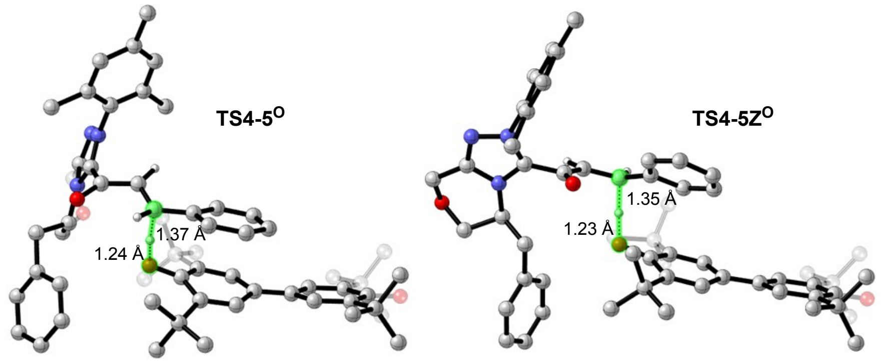

Fig. S8 Optimized geometries of the transition states **TS4-5°/TS4-5Z^{O}** for the possible HTO pathways calculated at the L1 level of theory. Most of the hydrogen atoms are omitted for the sake of clarity.

2.5 The possible intermolecular SET processes

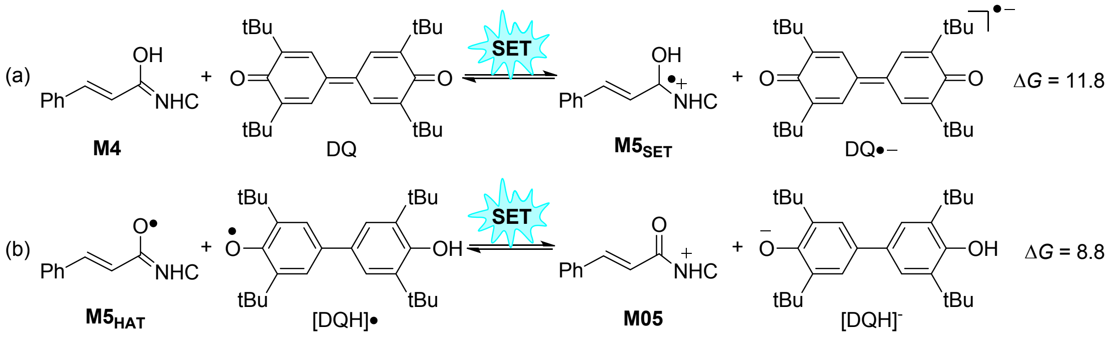

Scheme S4 The possible intermolecular single electron transfer processes for intermediates **M4** and **M5$_{HAT}$** associated with the related thermodynamic data (energy unit: kcal/mol)

2.6 Different deprotonation pathways of R2

As shown in **Figs. S9-10**, **R2** can be deprotonated by four bases, *i.e.*, OAr^{-}, DBU, and [DQH]$^{-}$, through the transition states **TS0^{Base}**. Obviously, the [DQH]$^{-}$-assisted deprotonation *via* **TS0^{DQH-}** ($\Delta G^{\ddagger}$ = 12.5 kcal/mol) is the most favorable among the three deprotonations. So, other two base-assisted deprotonations can be neglected.

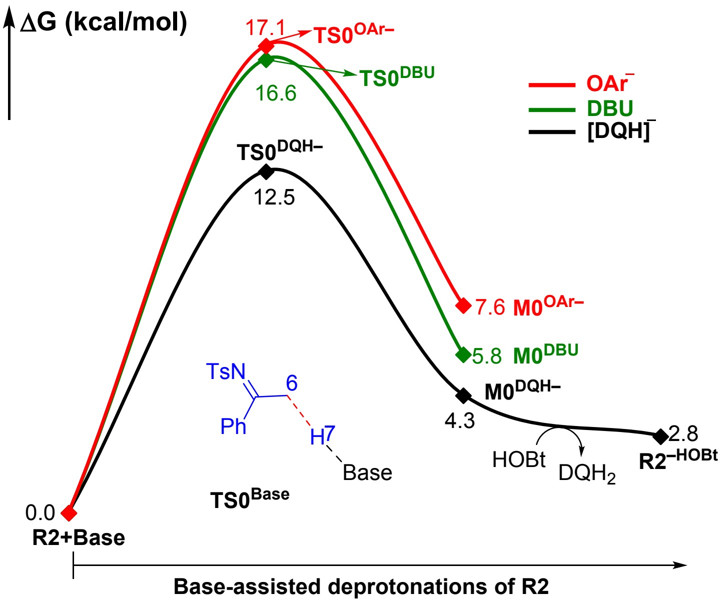

Fig. S9 Relative Gibbs free energy profiles of different kinds of base-assisted deprotonations of imine **R2**; the energies of the minima are relative to the energy of **R2**+Base (0.0 kcal/mol).

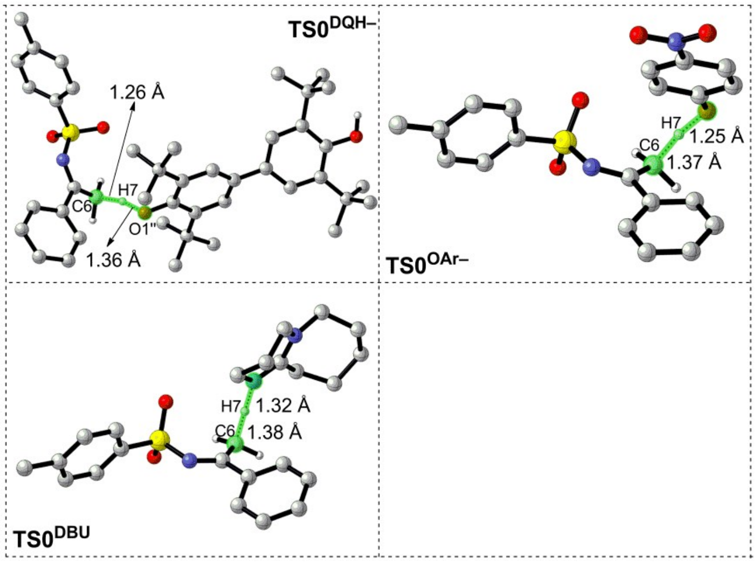

Fig. S10 Optimized geometries of all the deprotonation transition states of **R2**; the hydrogen atoms that are not involved in the reaction have been omitted. Distances are in angstroms.

2.7 Other possible pathways for the formations of P/SP$_A$/SP$_B$/SP$_C$/SP$_D$

The NHC-catalyzed [3 + 3] annulation pathway without HOBt. The difference between the [3 + 3] annulation pathways with or without HOBt is only reflected in steps 6-7. As shown in **Fig. S11**, the energy barriers of steps 6 and 7 *via* transition states **TS6(R/S)** and **TS7(S/R)^{HOAr}** are 13.6/14.4 and 13.2/10.4 kcal/mol in the [3 + 3] annulation pathway without HOBt, respectively. The energy barriers *via* **TS6(R/S)** are higher than those *via* **TS6(R/S)^{HOBt}** ($\Delta G^{\ddagger}$ = 9.9/12.3 kcal/mol), hence the [3 + 3] annulation pathway without HOBt is unnecessary to consider and

discuss.

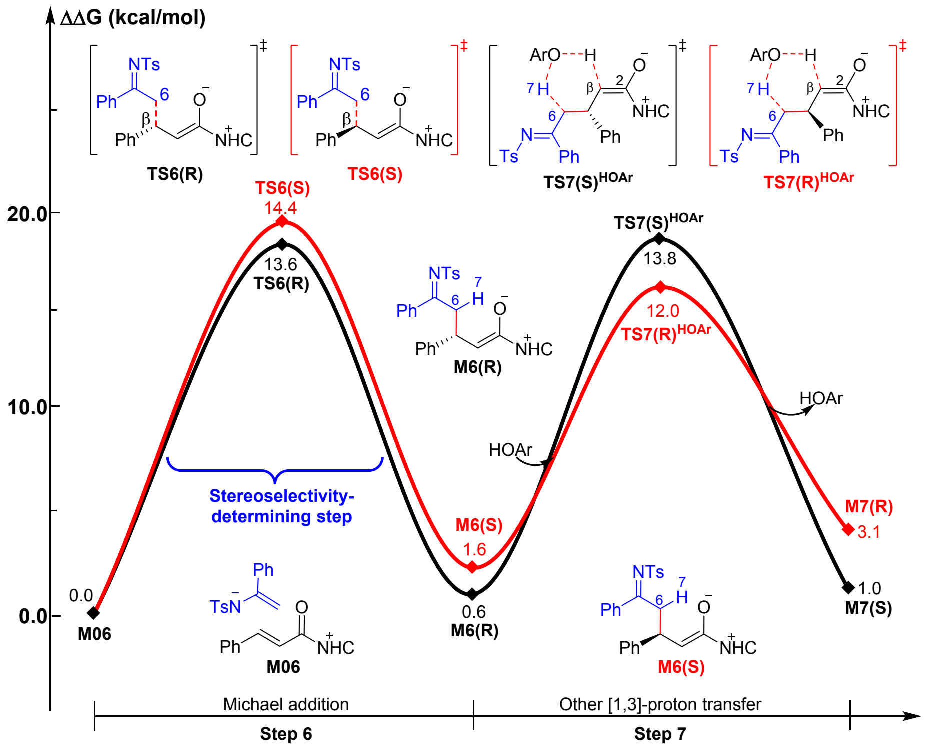

Fig. S11 Relative Gibbs free energy profiles for the divorced steps 6-7 of the [3 + 3] annulation pathway without HOBt, and the energies of all the minima are relative to the energy of **M06**.

The NHC-catalyzed ketolation and amidation pathways. Since **R2**⁻ has two nucleophilic sites (*i.e.*, C6 and N9 atoms, as P_k^-(C6) = 0.64, P_k^-(N9) = 0.43 in **Table S2**), the electrophilic site C2 atom of electrophile **M2/M02** can be attacked by not only the iminyl nitrogen N9 atom but also the olefine carbon C6 atom of nucleophile **R2**⁻, which would finally lead to the formation of products amide **SP$_A$** and ketone **SP$_B$**, separately. Thus, the pathway associated with the production of amide **SP$_A$**/ketone **SP$_B$** shares steps 1-2, and is divorced in step 3, which consists of the complexation of electrophile **M2** with nucleophile **R2**⁻ and recycling of catalyst NHC.

As shown in **Fig. S12**, the N9/C6 atom of **R2**⁻ nucleophilically attacks the C2 atom of **M2** through transition state **TS3$_A$/TS3$_B$** ($\Delta G^\ddagger$ = 19.5/19.4 kcal/mol) in generating intermediates **M3$_A$/M3$_B$** and releasing anion OAr⁻, respectively. Then, the catalyst NHC and product amide **SP$_A$**/ketone **SP$_B$** are generated separately *via* transition state **TS4$_A$/TS4$_B$** ($\Delta G^\ddagger$ = 3.7/4.5 kcal/mol). The energies of **SP$_A$/SP$_B$** are extremely higher than those of **P(RR/RS/SR/SS)** and located above than the energy of reactant, indicating that the product **SP$_A$/SP$_B$** cannot be formed in this overall endergonic reaction. Besides, all the energies of the NHC-catalyzed ketolation pathway are located above than those of the NHC-catalyzed amidation pathway, suggesting that the ketolation pathway is slighter favorable than the amidation pathway.

Table S2 Electrophilic and Nucleophilic Parr Functions (P_k^+ and P_k^-) For the Atoms (C6, C8, and N9) of Substrate **R2, R2**$^-$, **R2**$^{-\mathbf{HOBt}}$

**Parr function**	C6		C8		N9	
	P_k^+	P_k^-	P_k^+	P_k^-	P_k^+	P_k^-
**R2**	0. 01	0.01	0.40	−0.06	0.19	0.18
**R2**$^-$	0.20	0.64	0.02	−0.13	0.0	0.43
**R2**$^{-\mathbf{HOBt}}$	0.22	0.61	0.04	−0.09	0.0	0.37

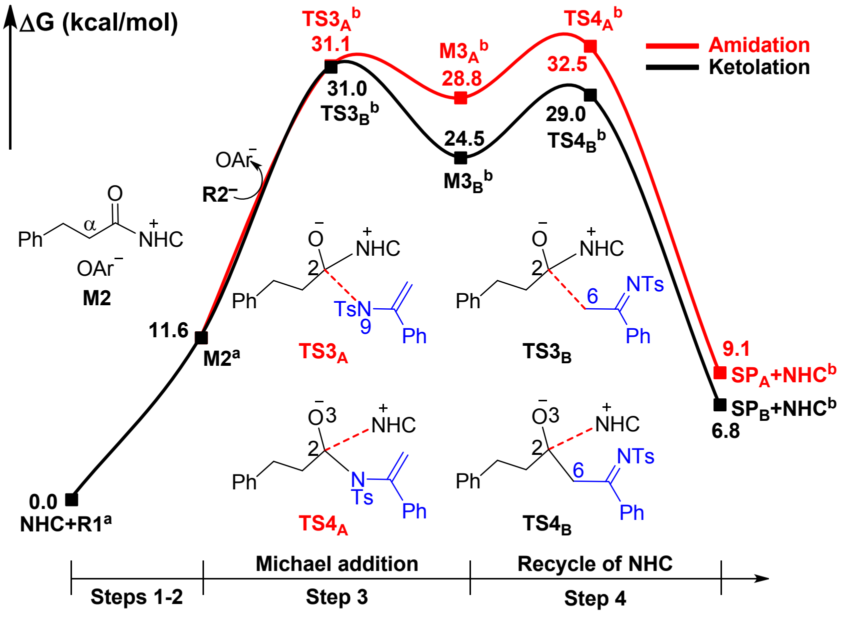

Fig. S12 Relative Gibbs free energy profiles for the NHC-catalyzed ketolation and amidation; the energies of all the minima are relative to the energy of NHC+**R1**+**R2**. Superscripts "a" and "b" represent the addition of the energy of **R2** and HOAr, respectively.

The NHC-catalyzed [2 + 2] cycloaddition pathway. As can be seen in **Table S1**, the C8 atom with P_k^+ value of 0.40 is more electrophilic than the N9 atom with P_k^+ value of 0.19 for the same reactant **R2**, so the addition of nucleophilic site Cα atom of **M3** to the electrophilic site C8 atom of **R2** can result in the formation of a four-membered lactam **SP**$_C$, whose associated pathway shares steps 1-3, is divorced in step 4, and is made up of the concerted [2 + 2] cycloaddition and recycling of catalyst NHC; while the addition of nucleophilic site Cα atom of **M3** to the electrophilic site N9 atom of **R2** can be ignored.

As shown in **Fig. S13**, the C2=Cα bond of **M3** combines with the C8=N9 bond of **R2** through four addition modes (*i.e.*, *Si-Si*, *Si-Re*, *Re-Si*, *Re-Re* modes) *via* transition states **TS4**$_C$**(RR/RS/SR/SS)** ($\Delta G^‡$ = 24.5/28.9/24.8/23.7 kcal/mol) in generating intermediates **M4**$_C$**(RR/RS/SR/SS)**, respectively. Then, the catalyst NHC and product lactam **SP**$_C$**(RR/RS/SR/SS)** are dissociated *via* transition state **TS5**$_C$**(RR/RS/SR/SS)** ($\Delta G^‡$ = 2.7/4.6/4.1/6.9 kcal/mol). The energies of **SP**$_C$**(RR/RS/SR/SS)** are extremely higher than those of

P(RR/RS/SR/SS) and located much above than the energy of reactant, indicating that the product $\mathbf{SP_C}$ cannot be formed in this reaction.

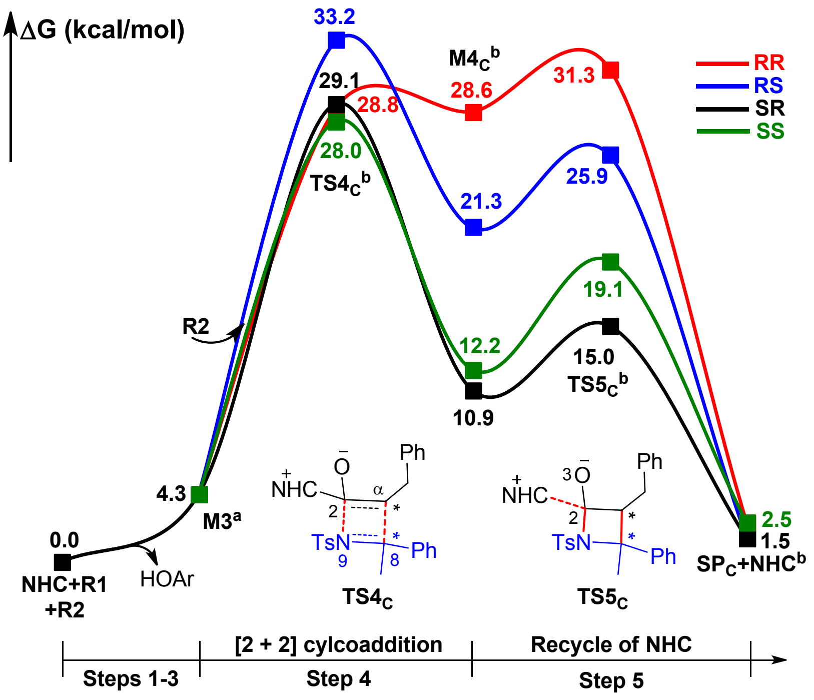

Fig. S13 Relative Gibbs free energy profiles for the NHC-catalyzed [2 + 2] cycloaddition; the energies of all the minima are relative to the energy of NHC+**R1**+**R2**. Superscripts "a" and "b" represent the addition of the energy of **R2**+HOAr and HOAr, respectively.

The NHC-catalyzed [3 + 2] annulation pathway. Similarly, the addition of nucleophilic site Cβ atom of **M4** to the electrophilic site N9 atom of **R2** can also be ignored for the [3 + 2] annulation reaction. The pathway for the production of a five-membered lactam $\mathbf{SP_D}$ shares steps 1-4, is divorced in step 5, and involves the following steps: the nucleophilic addition and coupled with proton transfer, [1,4]-proton transfer, and releasing of catalyst NHC.

As shown in **Fig. S14**, **M4** nucleophilically attacks to **R2** through four addition modes (*i.e.*, *Si-Si*, *Si-Re*, *Re-Si*, *Re-Re* modes) *via* the corresponding transition states $\mathbf{TS5_D}$**(RR/RS/SR/SS)** ($\Delta G^{\ddagger}$ = 11.8/11.5/20.0/14.6 kcal/mol) affording the related intermediates $\mathbf{M5_D}$**(RR/RS/SR/SS)**, in which two bonds of Cβ−C8 and N9−H5 are formed and a O3−H5 bond is breaking. Subsequently, the H5 atom in $\mathbf{M5_D}$**(RR/RS/SR/SS)** transfers from the N9 atom to Cα atom *via* the corresponding transition states $\mathbf{TS6_D}$**(RR/RS/SR/SS)** ($\Delta G^{\ddagger}$ = 23.1/25.4/21.3/22.4 kcal/mol) affording the related intermediates $\mathbf{M6_D}$**(RR/RS/SR/SS)**. Next, $\mathbf{M6_C}$**(RR/RS/SR/SS)** goes through a ring closure process through the relevant transition states $\mathbf{TS7_D}$**(RR/RS/SR/SS)** ($\Delta G^{\ddagger}$ = 2.8/4.2/5.8/4.4 kcal/mol) forming the corresponding intermediates $\mathbf{M7_D}$**(RR/RS/SR/SS).** Finally, the catalyst NHC and product lactam $\mathbf{SP_D}$**(RR/RS/SR/SS)** are dissociated *via* transition state $\mathbf{TS8_D}$**(RR/RS/SR/SS)** ($\Delta G^{\ddagger}$ = 5.6/7.0/8.6/12.6 kcal/mol). The energies of $\mathbf{SP_C}$**(RR/RS/SR/SS)** are

much higher than those of **P(RR/RS/SR/SS)**, indicating that the product **SP**$_C$ cannot be formed in this reaction.

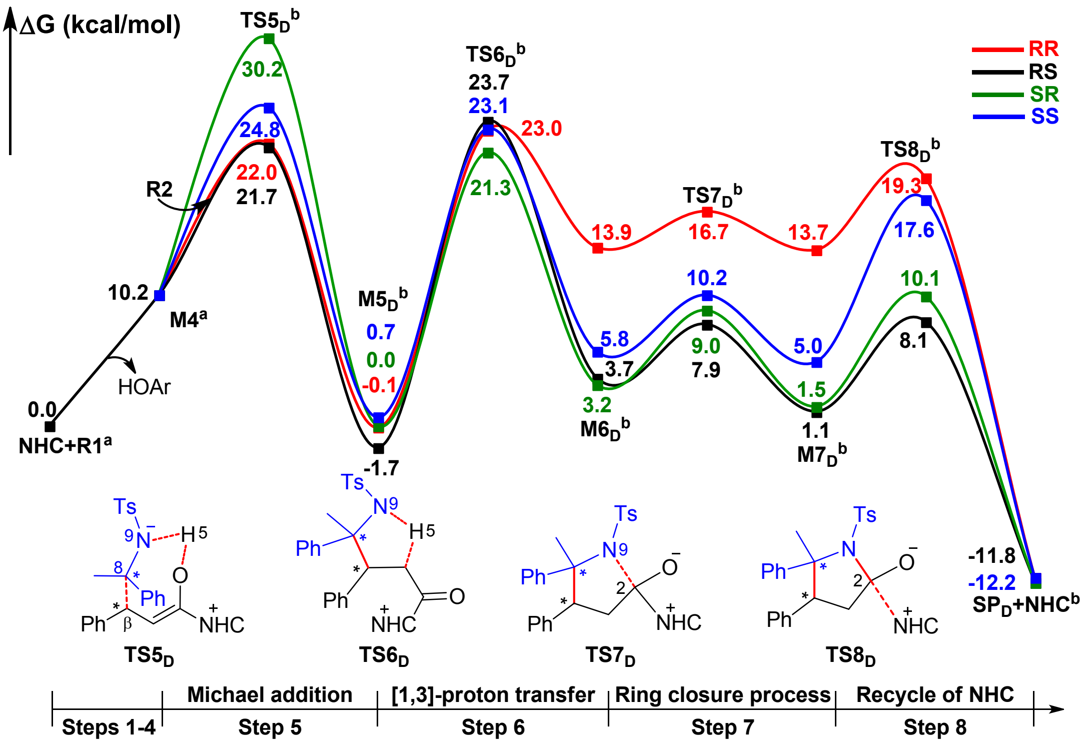

Fig. S14 Relative Gibbs free energy profiles for the NHC-catalyzed [3 + 2] annulation; the energies of all the minima are relative to the energy of NHC+**R1**+**R2**. Superscripts "a" and "b" represent the addition of the energy of HOAr+**R2** and HOAr, respectively.

Considering the flexibility coordination of HOBt, we have additionally accounted for other coordination of HOBt for the energetically favorable transition state **TS6(R)**$^{\mathbf{HOBt}}$. As presented in **Fig. S15**, the energy of transition state **O-TS6(R)**$^{\mathbf{HOBt}}$ with the coordination of HOBt to carbonyl oxygen is 2.8 kcal/mol higher than that of transition state **TS6(R)**$^{\mathbf{HOBt}}$ with the coordination of HOBt to tosyl oxygen. Hence, the pathway can be safely excluded.

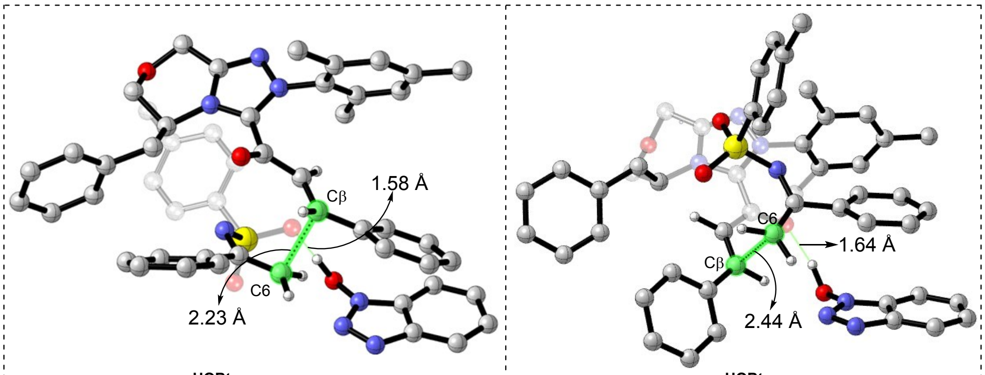

TS6(R)$^{\mathbf{HOBt}}$ **(0.0 kcal/mol)** **O-TS6(R)**$^{\mathbf{HOBt}}$ **(2.8 kcal/mol)**

Fig. S15 Optimized geometries and relative energies of the transition states **TS6(R)^{HOBt}** and **O-TS6(R)^{HOBt}**, in which HOBt coordinates to the carbonyl and tosyl oxygen, respectively; the hydrogen atoms that are not involved in the reaction have been omitted. Distances are in angstroms.

In addition, we also considered and computed the possible [3,3]-rearrangement pathway. As shown in **Fig. S16**, except for the nucleophilic addition of C6 to Cβ, the nucleophilic addition of the nitrogen atom N9 in **R2**$^-$ to the carbonyl carbon atom C2 in **M5** is likely to happen to form intermediate **M6^{CO}** *via* transition state **TS6^{CO}**, and then **M6^{CO}** might go through a [3,3]-rearrangement process. However, the energy barrier of 17.7 kcal/mol *via* transition state **TS6^{CO}** is higher than that of 13.6 kcal/mol *via* transition state **TS6(R)**, hence this pathway can be safely excluded.

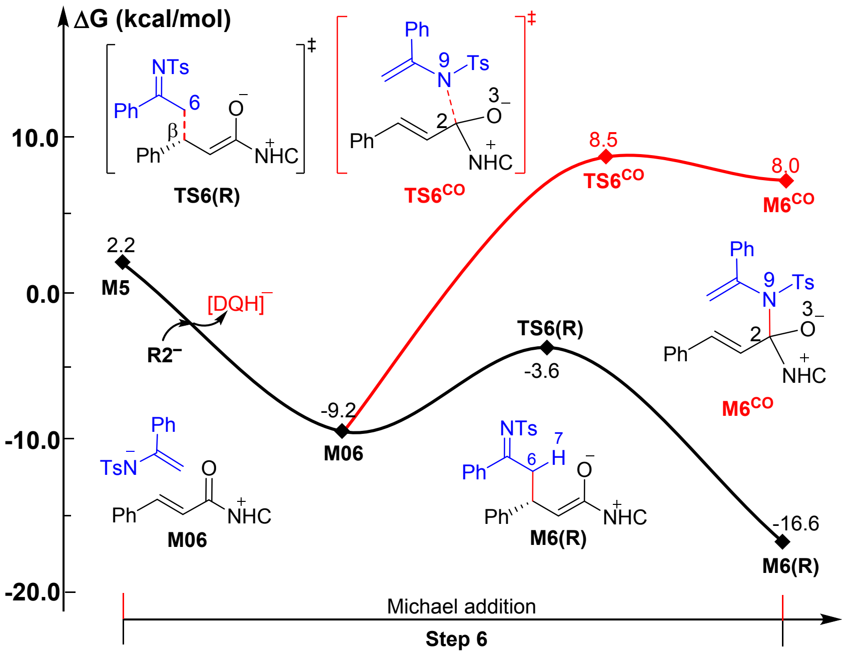

Fig. S16 Relative Gibbs free energy profiles of the possible [3,3]-rearreagenment pathway.

2.8 A conformational search of TS6

To ensure the enantioselective transition states associated with the lowest energy configurations, we have performed a conformational study *via* constructing different configurations of the **TS6(R/S)** and **TS6(R/S)^{HOBt}** by rotating the dihedral of Φ1(N−C1−C2−Cα). As presented in **Scheme S5**, the dihedral of Φ1(N−C1−C2−Cα) in **TS6(R/S)** or **TS6(R/S)^{HOBt}** was rotated 90° per time, and therefore eight (2*4) possible conformations were considered and computed for either **TS6(R/S)** or **TS6(R/S)^{HOBt}**, which can be named as **TS6(R/S)**, **TS6(R/S)** , **TS6(R/S)** , **TS6(R/S)** , and **TS6(R/S)^{HOBt}**, **TS6(R/S)^{HOBt}** , **TS6(R/S)^{HOBt}** , **TS6(R/S)^{HOBt}** ,

respectively. As summarized in **Table S3**, we confirmed that the energies of the **TS6(R/S)** and **TS6(R/S)$^{\mathbf{HOBt}}$** are the lowest among the distinctive conformers.

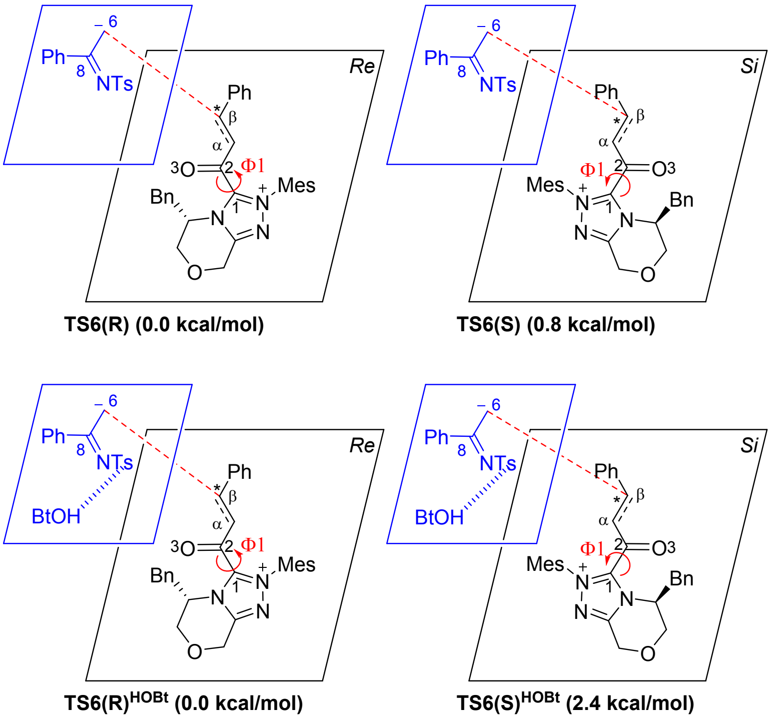

Scheme S5. Stereochemical Possibilities for the Catalytic Michael Addition Process by Rotating the Dihedral Φ1(N−C1−C2−Cα)

Table S3 Relative Energies (kcal/mol) of Different Conformers of **TS6(R/S)** and **TS6(R/S)$^{\mathbf{HOBt}}$**

	Δ*ΔG*		Δ*ΔG*		Δ*ΔG*		Δ*ΔG*
**TS6(R)**	0.0	**TS6(S)**	0.8	**TS6(R)$^{\mathbf{HOBt}}$**	0.0	**TS6(S)$^{\mathbf{HOBt}}$**	2.4
**TS6(R)**	7.2	**TS6(S)**	7.1	**TS6(R)$^{\mathbf{HOBt}}$**	1.3	**TS6(S)$^{\mathbf{HOBt}}$**	3.1
**TS6(R)**	10.7	**TS6(S)**	7.7	**TS6(R)$^{\mathbf{HOBt}}$**	3.6	**TS6(S)$^{\mathbf{HOBt}}$**	6.7
**TS6(R)**	12.6	**TS6(S)**	16.7	**TS6(R)$^{\mathbf{HOBt}}$**	9.1	**TS6(S)$^{\mathbf{HOBt}}$**	9.6

2.9 Origin of stereoselectivity

Non-covalent interaction (NCI) analysis has been performed to pursue the stereo-limiting factors in the stereochemical transition states. As shown in **Fig. S17**, the two reacting parts **Nu** and **E** of **TS6(R)$^{\mathbf{HOBt}}$/TS6(R)** present more interactions than that of **TS6(S)$^{\mathbf{HOBt}}$/TS6(R)**. Apparently, **TS6(R)$^{\mathbf{HOBt}}$** has three hydrogen bonds C−H···O (2.43/2.62 Å), C−H···N (2.62 Å), two C−H···π (2.43/2.58 Å), one π···π (3.44 Å) interactions, and **TS6(S)$^{\mathbf{HOBt}}$** has three hydrogen bonds C−H···O (2.18/2.40 Å), C−H···N (2.28 Å), one π···π (3.46 Å) interaction, indicating that **TS6(R)$^{\mathbf{HOBt}}$** is more stable than **TS6(S)$^{\mathbf{HOBt}}$**. Meanwhile, the same conclusion that **TS6(R)** is more stable than **TS6(S)** can be got from the NCI pictures of **TS6(R)** and **TS6(S)**. Hence, we can conclude that the

C−H···π/π···π interactions between **Nu** and **E** are the main limiting factors in determining the stereoselectivity.

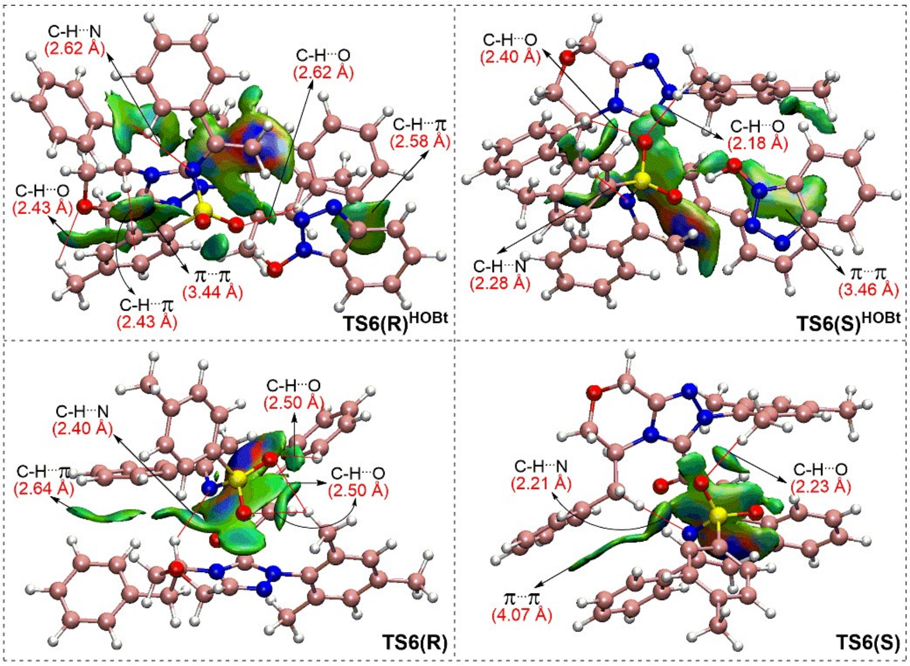

Fig. S17 Interaction analysis of stereoselective transition states **TS6(R)^{HOBt}**, **TS6(S)^{HOBt}**, **TS6(R)**, and **TS6(S)** (blue: strong attraction; green: weak interaction; red: steric effect; the δ value of isosurface is set to 0.01).

2.10 Test results of computational methods

To check whether the aforementioned calculation method (*i.e.*, M06-2X) and level (*i.e.*, L1) can obtain reliable results, we additionally performed calculations on the key transition states of the stereoselectivity-determining step by using different DFT methods. including B3LYP-D3, M06-2X(-D3) in the presence of the SMD continuum solvation model[1], and CAM-B3LYP-D3 at different levels. As summarized in **Table S4**, the energy difference between **TS6(R)^{HOBt}** and **TS6(S)^{HOBt}** calculated at the computational levels of L2-3 and L5 are close to that of computed at the L1 level. Although there is a significant difference by using the L4 computational level, more accurate MP2 result computed at the L6 level becomes closer to those results associated with the selected L1 level, and the trends obtained by all the computational methods are the same. So, we believe the conclusions based on the selected L1 computational level should be reliable, and the calculated results are in agreement with the experimental results.

Table S4 Comparisons of the Relative Gibbs Free Energies for Selected Stationary Points at Different Computational Levels Ln = L1~L6 (Unit: kcal/mol)

Level	L1	L2	L3	L4	L5	L6

**TS6(R)^{HOBt}$_{Ln}$**	0.0	0.0	0.0	0.0	0.0	0.0
**TS6(S)^{HOBt}$_{Ln}$**	2.4	2.5	3.2	6.9	1.1	4.3

L1: M06-2X-D3/6-31++G(2df, 2pd)/IEF-PCM$_{THF}$//M06-2X/6-31G(d, p)/IEF-PCM$_{THF}$;
L2: M06-2X/6-311++G(2df, 2pd)/IEF-PCM$_{THF}$//M06-2X/6-31G(d, p)/IEF-PCM$_{THF}$;
L3: B3LYP-D3/6-31++G(2df, 2pd)/IEF-PCM$_{THF}$//B3LYP/6-31G(d, p)/IEF-PCM$_{THF}$;
L4: M06-2X-D3/6-31++G(2df, 2pd)/SMD$_{THF}$//M06-2X/6-31G(d, p)/SMD$_{THF}$;
L5: ωb97X-D/6-31++G(2df, 2pd)/IEF-PCM$_{THF}$//ωb97X-D/6-31G(d, p)/IEF-PCM$_{THF}$;
L6: MP2/6-31++G(2df, 2pd)/SMD$_{THF}$//M06-2X/6-31G(d, p)/IEF-PCM$_{THF.}$

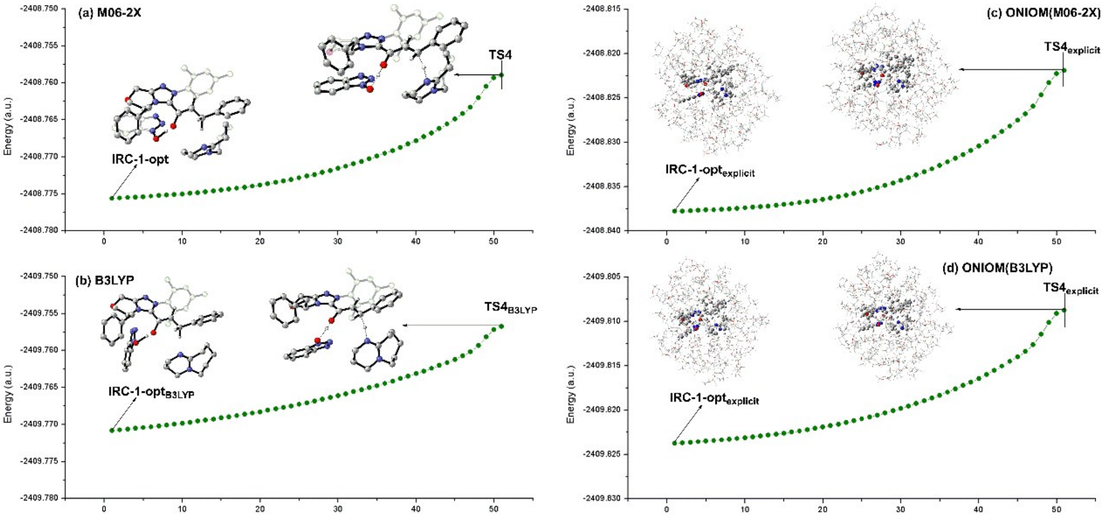

Fig. S18 The IRC results of **TS4** calculated at the different levels of (a) M06-2X/6-31G(d,p)/IEF-PCM$_{THF}$, (b) B3LYP/6-31G(d,p)/IEF-PCM$_{THF}$, (c) ONIOM(M06-2X/6-31G(d,p):UFF), (d) ONIOM(B3LYP/6-31G(d,p):UFF) (unit: a.u.)

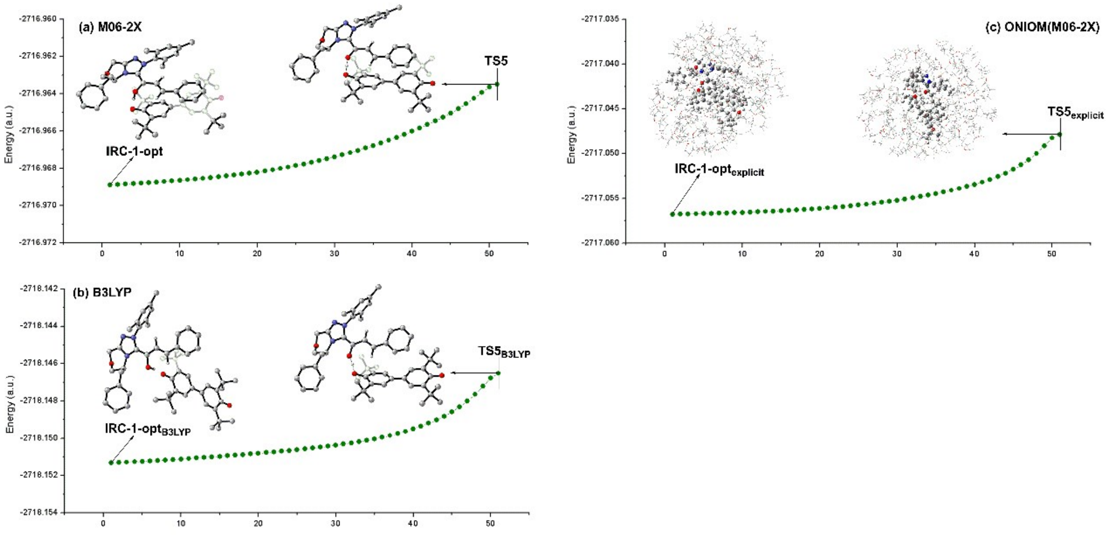

Fig. S19 The IRC results of **TS5** calculated at the different levels of (a) M06-2X/6-31G(d,p)/IEF-PCM$_{THF}$, (b) B3LYP/6-31G(d,p)/IEF-PCM$_{THF}$, (c) ONIOM(M06-2X/6-31G(d,p):UFF) (unit: a.u.)

2.11 The possible pathways for [1,3]-proton transfer of step 7

As shown in **Figs. S20-21**, in the seventh step of the [3 + 3] annulation pathway with/without

HOBt, the intermediates **M6(S/R)$^{\mathbf{HOBt}}$** or **M6(S/R)** probably undergo a [1,3]-proton transfer through the three different kinds of paths, including the direct and protic media (such as, DBU·H$^+$, and HOAr) assisted paths *via* transition states **TS7(R/S)$^{\mathbf{DT}}$**, **TS7(R/S)$^{\mathbf{DH+}}$**, and **TS7(R/S)$^{\mathbf{HOAr}}$**, respectively. Besides, intermediate **M6(S/R)$^{\mathbf{HOBt}}$** possibly proceeds through a HOBt-assisted [1,3]-proton transfer path *via* transition state **TS7(R/S)$^{\mathbf{HOBt}}$** ($\Delta G^{\ddagger}$ = 12.0/12.3 kcal/mol), affording the relevant intermediate **M7(R/S)**. By comparing these energy barriers, the [1,3]-proton transfer of the [3 + 3] annulation pathways with/without HOBt are most likely to carry out through the corresponding HOBt/HOAr-assisted [1,3]-proton transfer paths, and therefore the remaining paths can be safely excluded.

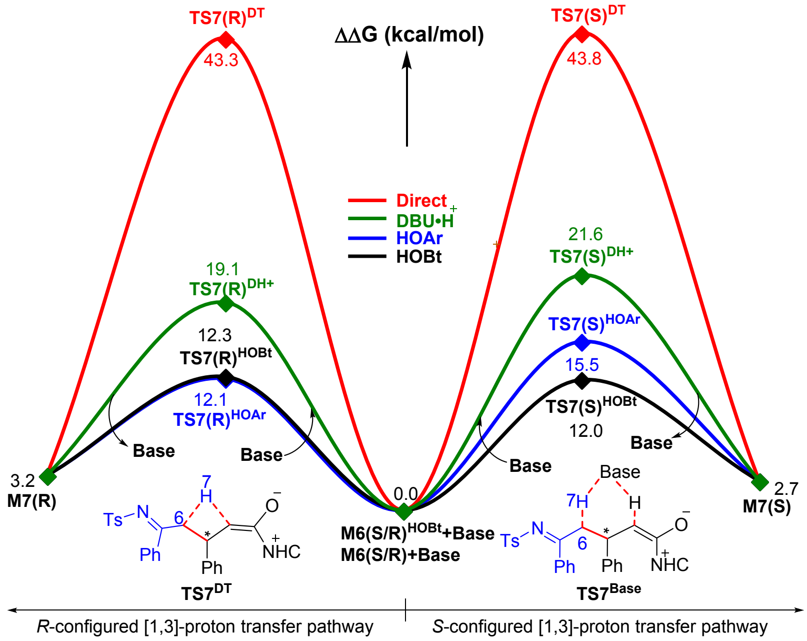

Fig. S20 Relative Gibbs free energy profiles of four possible [1,3]-proton transfer paths of step 7; the energies of the minima are relative to the energy of **M6(S/R)/M6(S/R)$^{\mathbf{HOBt}}$**+Base (0.0 kcal/mol).

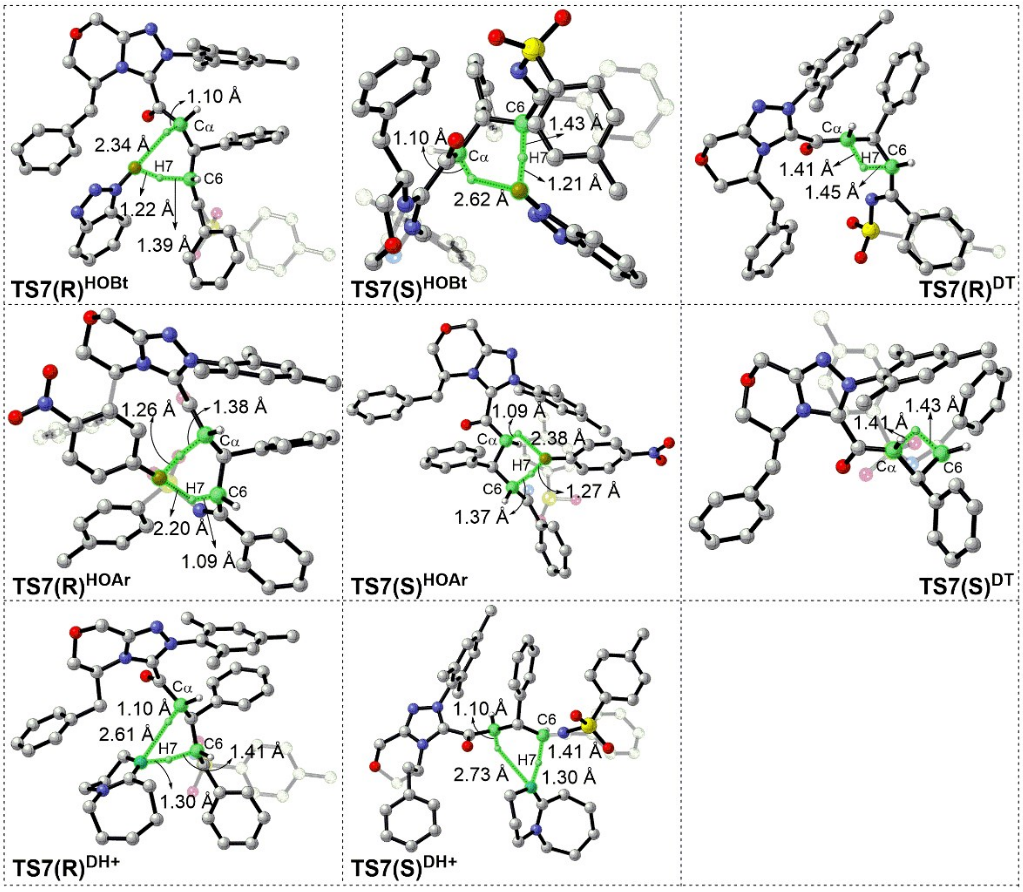

Fig. S21 Optimized geometries of all the [1,3]-proton transfer transition states; the hydrogen atoms that are not involved in the reaction have been omitted. Distances are in angstroms.

2.12 Roles of NHC and DQ

After a series of analyses, we found that NHC catalyst plays multiple roles in such type of catalytic reaction. Firstly, NHC catalyst could weaken the C2−O3/C2−O4 bonds after the absorption with substrate and thus facilitates the C2−O4 bond cleavage in the second step, since the bond orders of C2−O3/C2−O4 in **Table S5** reduce from 2.19/1.21 in **R1** to 1.70/0.80 in **M1**. Secondly, NHC catalyst could increase the acidities of Hα/Hβ atoms to advance the relevant α/β-C(sp^3)−H deprotonations, since the natural population analysis (NPA) charge values of Hα/Hβ (0.264/0.248) in **M2**/**M3** are higher than those (0.256/0.240) in **R1** as summarized in **Table S6**.

Table S5 Wiberg Bond Order Analyses on Bonds C2−O3/C2−O4

	C2−O3	C2−O4
**R1**	2.19 (1.20 **Å**)	1.21 (1.37 **Å**)
**M1**	1.70 (1.28 **Å**)	0.80 (1.55 **Å**)
**M2**	2.17 (1.21 **Å**)	0.10 (2.44 **Å**)
**M02**	2.20 (1.21 **Å**)	−

Table S6 Values of NPA Charge (unit of e) on Atoms Cα/β and Hα/β

	R1	M1	M2	M02	M3
Cα	−0.509	−0.452	−0.504	−0.514	−0.375
Hα	0.256	0.225	0.264	0.279	−
Cβ	−0.428	−0.433	−0.429	−0.423	−0.460
Hβ	0.240	0.246	0.252	0.256	0.248

Ultimately and importantly, we aim to use the global reactivity index (GRI) to uncover other possible role of NHC, which involves the parameters of electrophilicity index ω,[2] electronic chemical potential μ, chemical hardness η, one-electron energies of the frontier molecular orbital HOMO (E_L) and LUMO (E_H). The HOMO energies were obtained within the Kohn−Sham scheme,[3] and nucleophilicity index N[4] was generally utilized to judge the nucleophilicity character.

As summarized in **Table S7**, the NHC catalyst was certificated to actually realize umpolung of saturated ester **R1**, because the combination of NHC with the electrophilic saturated ester **R1** transformed to nucleophilic acylazolium intermediate **M02**→enolate **M3**→Breslow intermediate **M4**, and finally to electrophilic α,β-unsaturated acylazolium intermediate **M05**. Meanwhile, the same conclusion, namely realizing umpolung of saturated ester **R1**, can also be found in the analysis of the local reactivity index for the transformations from **R1** to **M05** as presented in **Table S8**.

Table S7 Energies of HOMO (E_H) and LUMO (E_L), Electronic Potential (μ), Chemical Hardness (η), Global Electrophilicity (ω), and Global Nucleophilicity (N) of Some Intermediates

	E_H (a.u.)	E_L (a.u.)	μ (a.u.)	η (a.u.)	ω (eV)	N (eV)
**R1**	−0.306	−0.066	−0.186	0.240	1.959	2.176
**M2**	−0.243	−0.052	−0.148	0.192	1.548	3.875
**M02**	−0.320	−0.098	−0.209	0.222	2.668	1.799
**M3**	−0.235	−0.010	−0.122	0.225	0.907	4.116
**M4**	−0.204	−0.012	−0.108	0.191	0.829	4.962
**M5**	−0.189	−0.098	−0.144	0.091	3.093	5.346
**M05**	−0.315	−0.109	−0.212	0.205	2.979	1.940
**R2**	−0.315	−0.050	−0.183	0.265	1.709	1.926
**R2^{-}**	−0.222	0.008	−0.107	0.230	0.677	4.459
**R2^{-HOBt}**	−0.240	−0.006	−0.123	0.234	0.878	3.976

$N = E_{H(SR)} - E_{H(TCNE)}$ ($E_{H(TCNE)} = -0.38586$ a.u.), $\omega = \mu^2/2\eta$, $\mu = (E_H + E_L)/2$, and $\eta = (E_L - E_H)$

As summarized in **Table S8**, the local reactivity index (electrophilic (P_k^+) and nucleophilic (P_k^-) Parr functions)[5] have been performed on the potential sites (*i.e.*, C2, Cα, Cβ atoms) of some intermediates. The C2 atom of **M2/M02** with P_k^+ value of 0.18/0.20 and the Cβ atom of **M5** with P_k^+ value of 0.24 are more electrophilic than those of the electrophilic **R1** with the P_k^+ value of 0.01/0.0, and thus promote the possible ketolation/amidation, [3 + 3] annulation pathways with the nucleophilic **R2^{-}**/**R2^{-HOBt}**. The Cα atom of **M3** with P_k^- value of 0.62 and the Cβ atom of **M4** with P_k^- value of 0.35 are much more nucleophilic than those of the electrophilic **R1** with the P_k^- value of 0.03/−0.02, which facilitates the possible [2 + 2] cycloaddition, [3 + 2] annulation reaction with

the electrophilic **R2**. Overall, the local reactivity index demonstrated that the five designed and proposed pathways of the ketolation, amidation, [2 + 2] cycloaddition, [3 + 2] annulation, and [3 + 3] annulation were reasonable and possible for the NHC-catalyzed reactions of saturated ester **R1** and imine **R2**, in which NHC can realize umpolung of the electrophilic saturated ester **R1**.

Table S8 Electrophilic and Nucleophilic Parr Functions (P_k^+ and P_k^-) For the Atoms (C2, Cα, and Cβ) of Some Intermediates

**Parr**	C2		Cα		Cβ	
**function**	P_k^+	P_k^-	P_k^+	P_k^-	P_k^+	P_k^-
**R1**	0. 01	0.0	0.0	0.03	0.0	−0.02
**M2**	0.18	0.0	0.0	0.0	0.0	0.0
**M02**	0.20	0.0	0.0	0.03	0.01	−0.01
**M3**	−0.31	−0.10	0.11	0.62	0.0	−0.03
**M4**	−0.13	0.42	0.29	−0.15	0.04	0.35
**M5**	0.22	0.0	−0.12	0.01	0.24	0.0
**M05**	0.22	−0.07	−0.09	0.37	0.25	−0.16

Table S9 Global (N and ω, unit: eV) Indexes of the Stable **Nu** and **E** Partners and the Energy Barriers ($\Delta G^‡$ and $\Delta G^‡_{total}$, unit: kcal/mol) of the Chemoselective Transition States Involved in the Ketolation, [2 + 2] Cycloaddition, [3 + 2] Annulation, and [3 + 3] Annulation Pathways

	**TS3$_B$**	**TS4$_C$(SS)**	**TS5$_D$(RS)**	**TS6(R)^{HOBt}**
**Nu**	**M0^{DBU}**	**M3**	**M4**	**R2^{-HOBt}**
N	3.887	4.116	4.962	3.976
**E**	**M2**	**R2**	**R2**	**M5**
ω	1.548	1.709	1.709	3.093
ω+N	5.44	5.83	6.67	7.07
$\Delta G^‡$	19.4	23.7	11.5	9.9
$\Delta G^‡_{total}$	31.0	28.0	24.5	9.9

"**M0^{DBU}**" is a stable nucleophile, which is composed of **R2**$^-$ anion and DBU·H$^+$ cation.
"$\Delta G^‡_{total}$" is the total energy barrier of the entire reaction.

2.13 Analyses of other cases

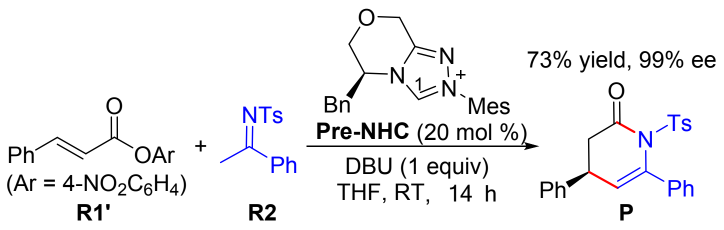

Scheme S6 NHC-catalyzed [3 + 3] annulation between unsaturated esters and imines

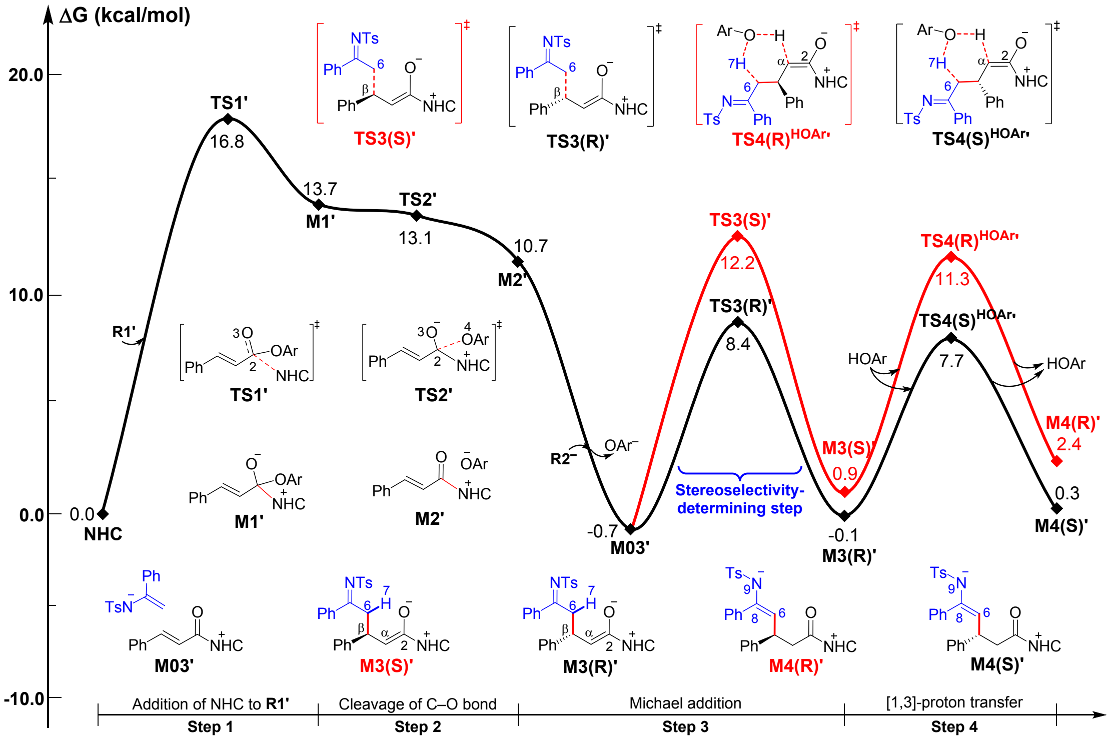

Fig. S22 Gibbs free energy profiles of the NHC-catalyzed [3 + 3] annulation of α,β-unsaturated ester with imine.

To explain the origins of reactivity or selectivity, we have additionally done modeling of the key transition states involved in the pathways for generating different products in the reaction models as depicted in **Figs. 8** and **9**. As shown in **Fig. S23**, the increasing order of the calculated Gibbs free energy barrier is (*i.e.*, $\Delta G^{\ddagger}$(**TS4b**) = 11.2 kcal/mol < $\Delta G^{\ddagger}$(**S2-TS3b**) = 18.3 kcal/mol < $\Delta G^{\ddagger}$(**S1-TS2b**) = 32.6 kcal/mol) is in agreement with that of their predicted values (*i.e.*, $\Delta G^{\ddagger}_{p}$([3+4]/[3+6] annulation pathway) = 11.0 kcal/mol < $\Delta G^{\ddagger}_{p}$([2+4] cycloaddition pathway) = 14.7 kcal/mol < $\Delta G^{\ddagger}_{p}$(amidation pathway) = 19.8 kcal/mol). Meanwhile, a similar conclusion can be obtained from the calculated results in **Fig. S24** except for the ketolation pathway. Thus, it can be seen that the predicted Gibbs free energy barriers of these distinctive pathways can be used to disclose the origin of reaction chemoselectivity in this kind of reaction.

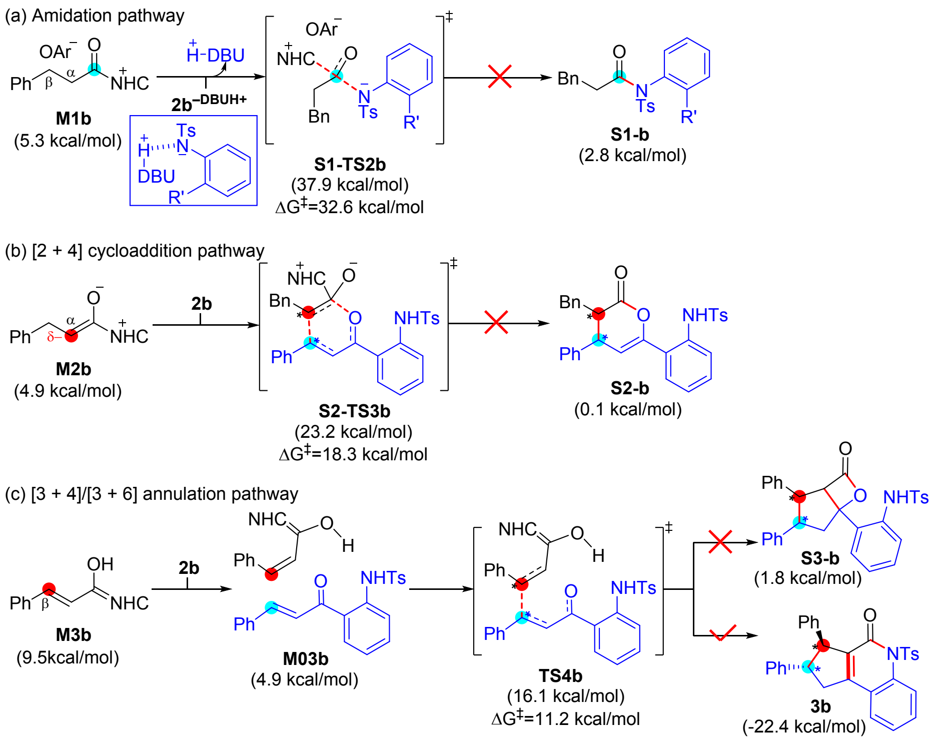

Fig. S23 Different product formation pathways of NHC-catalyzed reactions of saturated carboxylic esters with *o*-tosylamino enones.

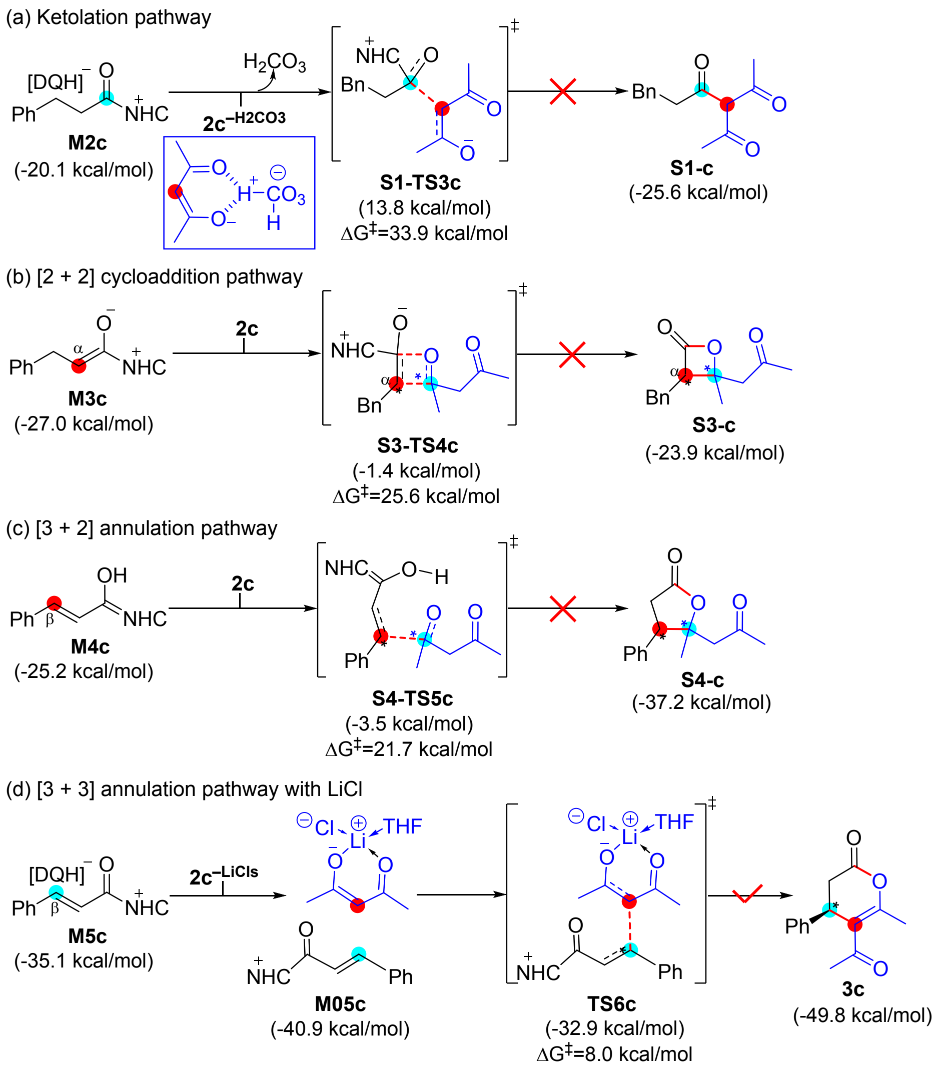

Fig. S24 Different product formation pathways of NHC-catalyzed reactions of simple aldehydes with acetylacetones.

Table S10 Energies of HOMO (E_H) and LUMO (E_L), Electronic Potential (μ), Chemical Hardness (η), Global Electrophilicity (ω), and Global Nucleophilicity (N) of Intermediates in **Fig. 7**

	E_H (a.u.)	E_L (a.u.)	μ (a.u.)	η (a.u.)	ω (eV)	N (eV)
**1a**	−0.295	−0.058	−0.176	0.237	1.783	2.618
**M2a**	−0.194	−0.009	−0.102	0.185	0.762	5.351
**M3a**	−0.339	−0.135	−0.237	0.204	3.753	1.405
**2a**	−0.335	−0.059	−0.197	0.276	1.921	1.510
**2a⁻**	−0.180	0.033	−0.073	0.213	0.346	5.727
**$2a^{-iPr2NEtH+}$**	−0.248	−0.009	−0.128	0.238	0.940	3.900

$N = E_{H(SR)} - E_{H(TCNE)}$ ($E_{H(TCNE)} = -0.39087$ a.u.), $\omega = \mu^2/2\eta$, $\mu = (E_H + E_L)/2$, and $\eta = (E_L - E_H)$
"$2a^{-iPr2NEtH+}$" is a stable nucleophile, which is composed of **2a⁻** anion and *i*$Pr_2NEt{\cdot}H^+$ cation.

Table S11 Energies of HOMO (E_H) and LUMO (E_L), Electronic Potential (μ), Chemical Hardness (η), Global Electrophilicity (ω), and Global Nucleophilicity (N) of Intermediates in **Fig. 8**

	E_H (a.u.)	E_L (a.u.)	μ (a.u.)	η (a.u.)	ω (eV)	N (eV)
**1b(R1)**	−0.306	−0.066	−0.186	0.240	1.959	2.176

**M1b**	−0.250	−0.050	−0.150	0.200	1.540	3.687
**M01b**	−0.318	−0.089	−0.204	0.229	2.471	1.845
**M2b**	−0.228	−0.025	−0.126	0.203	1.066	4.304
**M3b**	−0.204	−0.022	−0.113	0.182	0.962	4.942
**2b**	−0.294	−0.068	−0.181	0.226	1.980	2.486
**2b^{-}**	−0.225	−0.038	−0.131	0.186	1.264	4.391
**2b^{-DBUH+}**	−0.243	−0.053	−0.148	0.190	1.576	3.881

$N = E_{H(SR)} - E_{H(TCNE)}$ ($E_{H(TCNE)} = -0.38586$ a.u.), $\omega = \mu^2/2\eta$, $\mu = (E_H + E_L)/2$, and $\eta = (E_L - E_H)$
"**2b^{-DBUH+}**" is a stable nucleophile, which is composed of **2b^{-}** anion and DBU·H^{+} cation.

Table S12 Energies of HOMO (E_H) and LUMO (E_L), Electronic Potential (μ), Chemical Hardness (η), Global Electrophilicity (ω), and Global Nucleophilicity (N) of Intermediates in **Fig. 9**

	E_H (a.u.)	E_L (a.u.)	μ (a.u.)	η (a.u.)	ω (eV)	N (eV)
**1c**	−0.303	−0.0	−0.151	0.302	1.033	2.266
**M1c**	−0.216	−0.008	−0.112	0.207	0.821	4.633
**M2c**	−0.200	−0.058	−0.129	0.142	1.586	5.070
**M02c**	−0.319	−0.075	−0.197	0.244	2.174	1.809
**M3c**	−0.232	−0.018	−0.125	0.214	0.997	4.185
**M4c**	−0.204	−0.014	−0.109	0.190	0.852	4.958
**M5c**	−0.188	−-0.092	−0.140	0.096	2.790	5.382
**M05c**	−0.314	−0.108	−0.211	0.206	2.939	1.959
**2c**	−0.329	−0.003	−0.166	0.326	1.147	1.543
**2c^{-}**	−0.220	0.017	−0.102	0.237	0.593	4.511
**2c^{-H2CO3}**	−0.241	0.012	−0.115	0.252	0.707	3.949
**2c^{-LiCls}**	−0.246	0.001	−0.123	0.247	0.829	3.809

$N = E_{H(SR)} - E_{H(TCNE)}$ ($E_{H(TCNE)} = -0.38586$ a.u.), $\omega = \mu^2/2\eta$, $\mu = (E_H + E_L)/2$, and $\eta = (E_L - E_H)$
"**2c^{-LiCls}**" is a stable nucleophile, which is composed of **2c^{-}** anion, LiCl, and solvent THF.

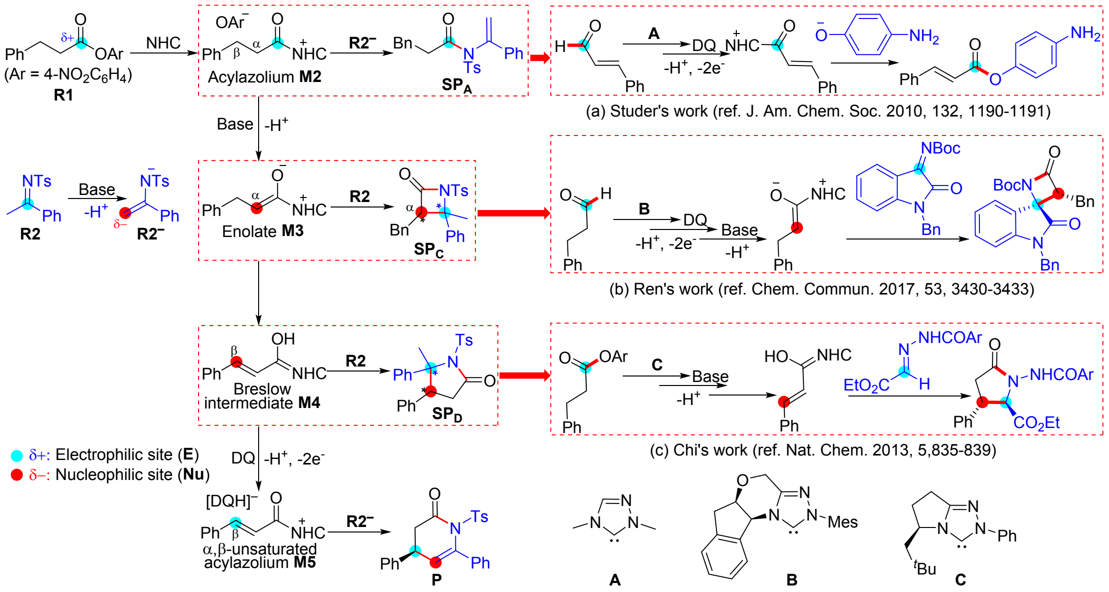

Scheme S7 NHC-catalyzed possible reactions of carbonyl compounds (**Scheme 2**) and their related experimental reports

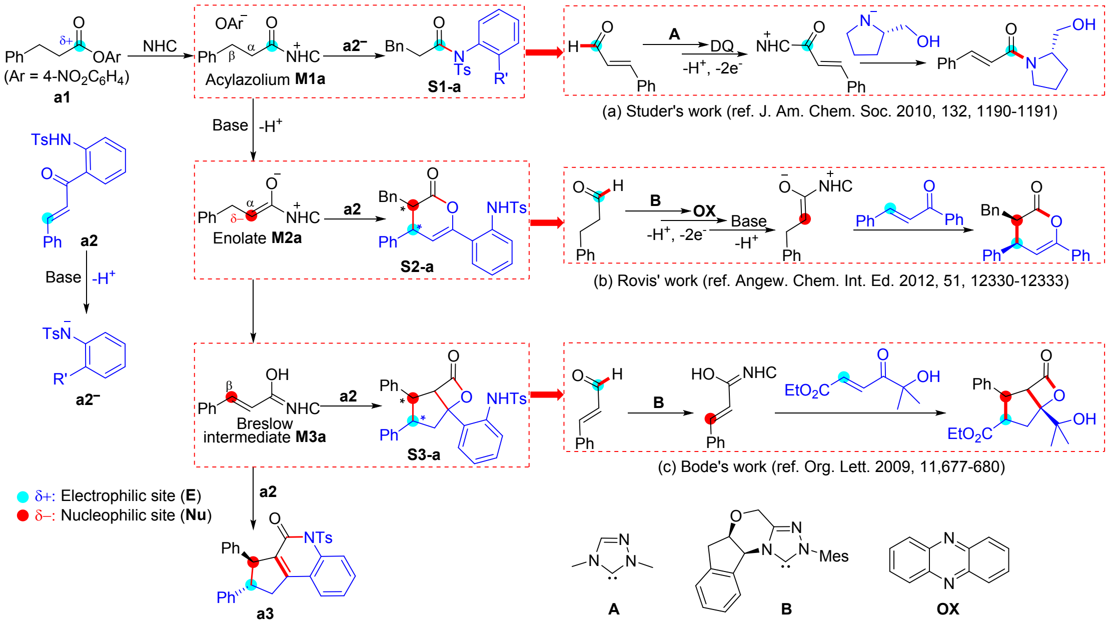

Scheme S8 NHC-catalyzed possible reactions of carbonyl compounds (**Fig. 8**) and their related experimental reports

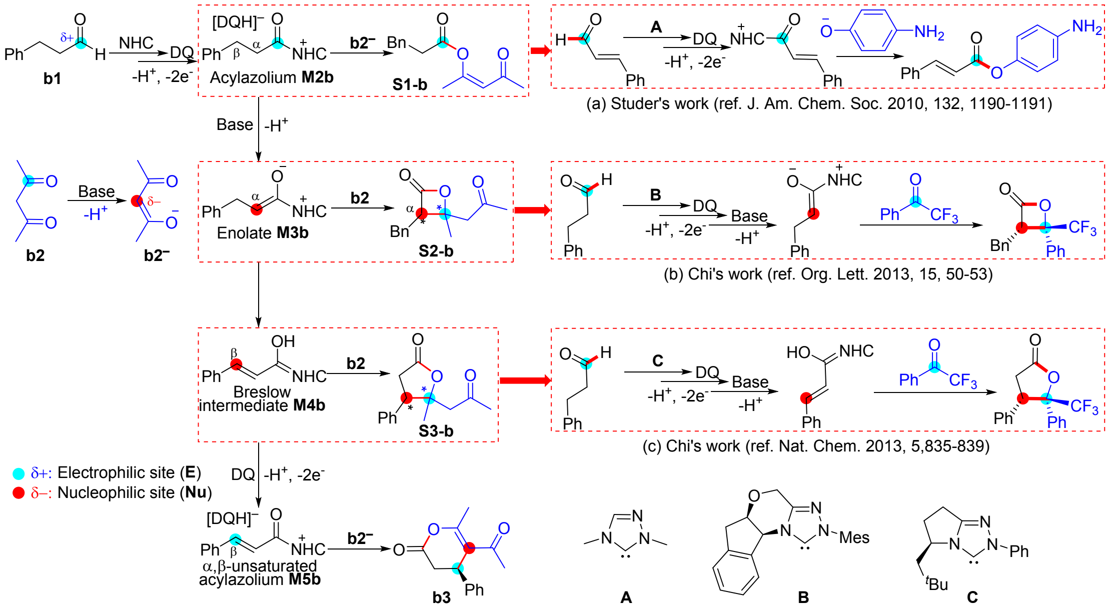

Scheme S9 NHC-catalyzed possible reactions of carbonyl compounds (**Fig. 9**) and their related experimental reports

3 Cartesian coordinates and energy values of optimized structures

3.1 Optimized geometries of the stationary points

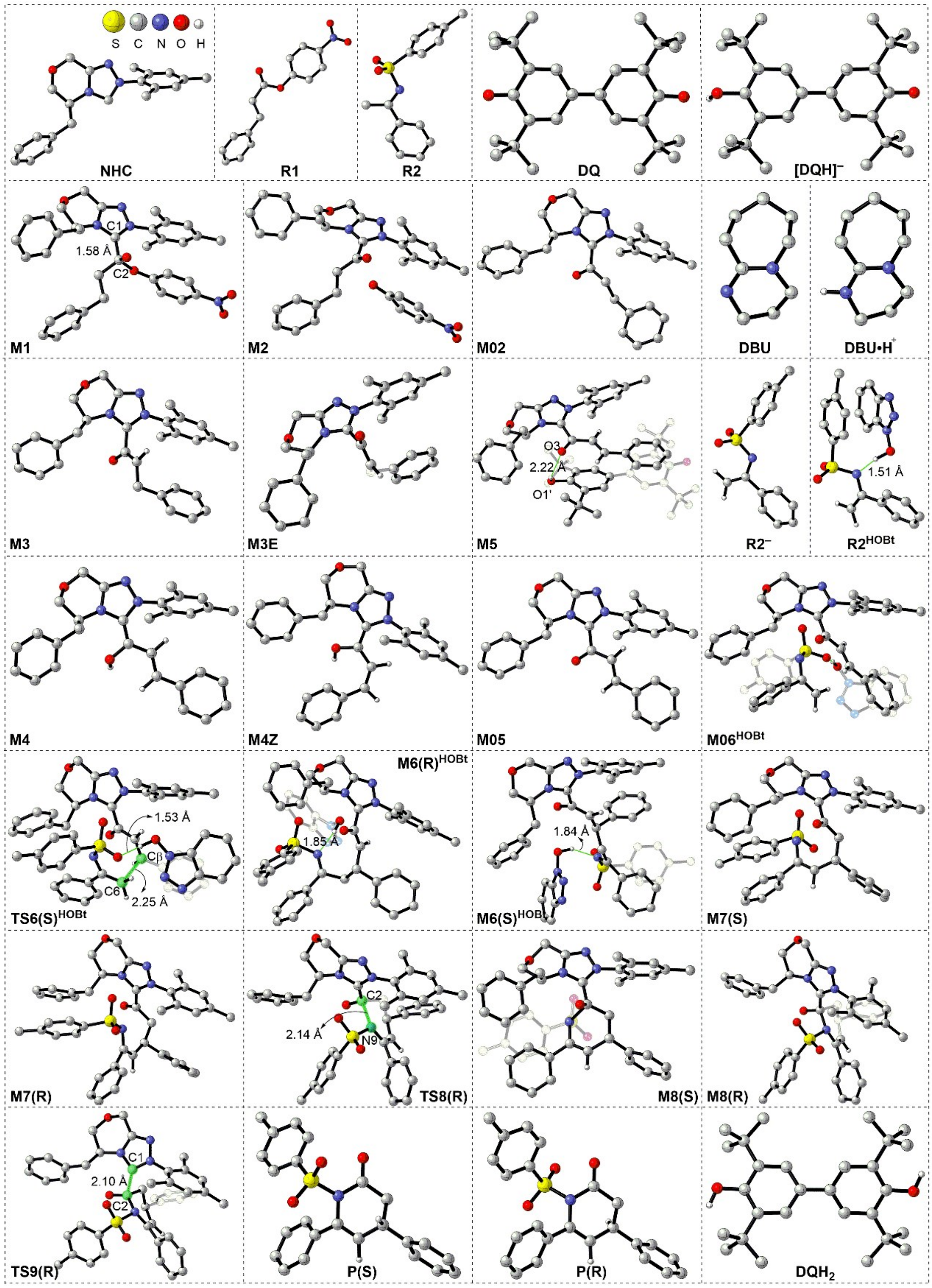

Fig. S25 Optimized geometries of the stationary points for the production of lactam **P**. Most of the hydrogen atoms are omitted for the sake of clarity.

3.2 Absolute SPE and GFE of the optimized structures by M06-2X

Table S13 The SPE, GFEC, GFE (=SPE+GFEC), and relative Gibbs free energies barriers (ΔG) of the stationary points involved in the catalytic cycle, which are computed at the L1 computational level and relative to the energy of NHC+**R1**+**R2**+DQ.

	SPE (a.u.)	GFEC (a.u.)	GFE (a.u.)
**NHC**	-1053.217708	0.351157	-1052.866551
**R1**	-934.7339669	0.208309	-934.5256579
**R2**	-1183.726653	0.221969	-1183.504684
**DBU**	-461.9467352	0.213212	-461.7335232
**DBU·H$^+$**	-462.4149822	0.228326	-462.1866562
**OAr$^-$**	-511.3709267	0.062634	-511.3082927
**HOAr**	-511.8435473	0.07611	-511.7674373
**OBt$^-$**	-470.4374549	0.067405	-470.3700499
**HOBt**	-470.9039724	0.079392	-470.8245804
**DQ**	-1241.116619	0.559151	-1240.557468
**DQ·–**	-1241.260901	0.558792	-1240.7021090
**[DQH]·**	-1241.732787	0.571984	-1241.1608031
**[DQH]$^-$**	-1241.871297	0.571707	-1241.29959
**DQH$_2$**	-1242.366799	0.585443	-1241.781356
**TS1**	-1987.956309	0.59038	-1987.365929
**M1**	-1987.965234	0.594368	-1987.370866
**TS2**	-1987.963422	0.593756	-1987.369666
**M2**	-1987.966844	0.593057	-1987.373787
**M02**	-1476.56543	0.503753	-1476.061677
**TS3**	-1987.949115	0.582358	-1987.366757
**TS3E**	-1987.945193	0.585111	-1987.360082
**TS3^{DBU}**	-1938.52505	0.737665	-1937.787385
**TS3E^{DBU}**	-1938.518867	0.739262	-1937.779605
**M3**	-1476.108415	0.490491	-1475.617924
**M3E**	-1476.101948	0.490612	-1475.611336
**TS4**	-2408.999904	0.829855	-2408.170049
**TS4Z**	-2408.988612	0.8281	-2408.160512
**TS4^{HOBt}**	-1947.012158	0.588571	-1946.423587
**TS4Z^{HOBt}**	-1947.01121	0.588992	-1946.422218
**TS4$^{DBU\text{-}DH+}$**	-2400.487954	0.968136	-2399.519818
**TS4Z$^{DBU\text{-}DH+}$**	-2400.482619	0.971242	-2399.511377
**TS4^{HOAr}**	-1987.943863	0.585691	-1987.358172
**TS4Z^{HOAr}**	-1987.937985	0.585689	-1987.352296
**TS4^{DH+}**	-1938.507469	0.736077	-1937.771392
**TS4Z^{DH+}**	-1938.502039	0.731899	-1937.77014
**TS4^{DBU}**	-1938.031578	0.720225	-1937.311353
**TS4Z^{DBU}**	-1938.023954	0.722015	-1937.301939
**M4^{com}**	-2409.021318	0.833927	-2408.187391
**M4Z^{com}**	-2409.011644	0.830659	-2408.180985
**M4**	-1476.09656	0.488059	-1475.608501
**M4Z**	-1476.089782	0.489406	-1475.600376

**$TS4\text{-}5^{O}$**	-2717.191707	1.075473	-2716.116234
**$TS4\text{-}5Z^{O}$**	-2717.182385	1.068559	-2716.113826
**$M5_{SET}$**	-1475.936729	0.491643	-1475.4450859
**$M5_{HAT}$**	-1475.496244	0.475034	-1475.0212098
**TS5**	-2717.236022	1.079245	-2716.156777
**$TS5_{HTC}$**	-2717.212135	1.081585	-2716.13055
**$TS5_{HTC}^{C2'}$**	-2717.179266	1.081183	-2716.098083
**$TS5_{HTC}^{C4'}$**	-2717.195298	1.082658	-2716.11264
**$TS5_{HTC}^{C5'}$**	-2717.216815	1.078038	-2716.138777
**M5**	-2717.262661	1.083999	-2716.178662
**M05**	-1475.34868	0.480264	-1474.868416
**$TS0^{OAr-}$**	-1695.089315	0.30355	-1694.785765
**$TS0^{DBU}$**	-1645.665105	0.453389	-1645.211716
**$TS0^{DQH-}$**	-2425.598653	0.814316	-2424.784337
**$M0^{OAr-}$**	-1695.111636	0.310708	-1694.800928
**$M0^{DBU}$**	-1645.693089	0.464054	-1645.229035
**$M0^{DQH-}$**	-2425.618406	0.820917	-2424.797489
**$R2^{-HOBt}$**	-1654.184447	0.314231	-1653.870216
**$M06^{HOBt}$**	-3129.571273	0.820569	-3128.750704
**$TS6(R)^{HOBt}$**	-3129.55728	0.822349	-3128.734931
**$TS6(S)^{HOBt}$**	-3129.557739	0.826584	-3128.731155
**$M6(R)^{HOBt}$**	-3129.584929	0.824634	-3128.760295
**$M6(S)^{HOBt}$**	-3129.581632	0.823908	-3128.757724
**$TS7(S)^{HOBt}$**	-3129.564406	0.823239	-3128.741167
**$TS7(R)^{HOBt}$**	-3129.561689	0.823561	-3128.738128
**$R2^{-}$**	-1183.238438	0.21249	-1183.025948
**M06**	-2658.638407	0.718125	-2657.920282
**TS6(R)**	-2658.630201	0.718871	-2657.91133
**TS6(S)**	-2658.629936	0.719831	-2657.910105
**M6(R)**	-2658.64391	0.716012	-2657.927898
**M6(S)**	-2658.653161	0.72276	-2657.930401
**$TS7(S)^{HOAr}$**	-3170.499781	0.821423	-3169.678358
**$TS7(R)^{HOAr}$**	-3170.500498	0.819263	-3169.681235
**$TS7(S)^{DH+}$**	-3121.056247	0.968351	-3120.087896
**$TS7(R)^{DH+}$**	-3121.061696	0.972367	-3120.089329
**$TS7(S)^{DT}$**	-2658.582998	0.71687	-2657.866128
**$TS7(R)^{DT}$**	-2658.578628	0.714537	-2657.864091
**M7(S)**	-2658.654614	0.72327	-2657.931344
**M7(R)**	-2658.651962	0.723869	-2657.928093
**TS8(S)**	-2658.651237	0.725742	-2657.925495
**TS8(R)**	-2658.645161	0.725742	-2657.919419
**M8(S)**	-2658.65489	0.728525	-2657.926365
**M8(R)**	-2658.65205	0.724622	-2657.927428
**TS9(S)**	-2658.641156	0.719862	-2657.921294
**TS9(R)**	-2658.639891	0.723422	-2657.916469
**P(S)**	-1605.41962	0.342845	-1605.076775

**P(R)**	-1605.41962	0.342845	-1605.076775
**$TS3_A$**	-2659.827371	0.747442	-2659.079929
**$M3_A$**	-2659.829738	0.746126	-2659.083612
**$TS4_A$**	-2659.823818	0.746133	-2659.077685
**SP_A**	-1606.608654	0.360273	-1606.248381
**$TS3_B$**	-2659.822492	0.742611	-2659.079881
**$M3_B$**	-2659.835414	0.744989	-2659.090425
**$TS4_B$**	-2659.828592	0.745386	-2659.083206
**SP_B**	-1606.609217	0.35714	-1606.252077
**$TS4_C$(RR)**	-2659.827022	0.74347	-2659.083552
**$TS4_C$(RS)**	-2659.819757	0.743265	-2659.076492
**$TS4_C$(SR)**	-2659.832743	0.749653	-2659.08309
**$TS4_C$(SS)**	-2659.828042	0.743175	-2659.084867
**$M4_C$(RR)**	-2659.832674	0.748723	-2659.083951
**$M4_C$(RS)**	-2659.846017	0.750485	-2659.095532
**$M4_C$(SR)**	-2659.86645	0.754297	-2659.112153
**$M4_C$(SS)**	-2659.85906	0.749065	-2659.109995
**$TS5_C$(RR)**	-2659.827152	0.74761	-2659.079542
**$TS5_C$(RS)**	-2659.837574	0.749334	-2659.08824
**$TS5_C$(SR)**	-2659.857227	0.751743	-2659.105484
**$TS5_C$(SS)**	-2659.847284	0.748235	-2659.099049
**SP_C(RR)**	-1606.623215	0.364344	-1606.258871
**SP_C(RS)**	-1606.625765	0.365215	-1606.26055
**SP_C(SR)**	-1606.625765	0.365215	-1606.26055
**SP_C(SS)**	-1606.623207	0.364347	-1606.25886
**$TS5_D$(RR)**	-2659.838801	0.744351	-2659.09445
**$TS5_D$(RS)**	-2659.838508	0.743642	-2659.094866
**$TS5_D$(SR)**	-2659.824911	0.743531	-2659.08138
**$TS5_D$(SS)**	-2659.83524	0.745358	-2659.089882
**$M5_D$(RR)**	-2659.879584	0.750002	-2659.129582
**$M5_D$(RS)**	-2659.878191	0.746103	-2659.132088
**$M5_D$(SR)**	-2659.877716	0.748315	-2659.129401
**$M5_D$(SS)**	-2659.88113	0.75284	-2659.12829
**$TS6_D$(RR)**	-2659.836104	0.743245	-2659.092859
**$TS6_D$(RS)**	-2659.833361	0.741655	-2659.091706
**$TS6_D$(SR)**	-2659.834803	0.739271	-2659.095532
**$TS6_D$(SS)**	-2659.832856	0.740135	-2659.092721
**$M6_D$(RR)**	-2659.855617	0.748273	-2659.107344
**$M6_D$(RS)**	-2659.871194	0.747598	-2659.123596
**$M6_D$(SR)**	-2659.872203	0.747885	-2659.124318
**$M6_D$(SS)**	-2659.869246	0.749012	-2659.120234
**$TS7_D$(RR)**	-2659.855922	0.753155	-2659.102767
**$TS7_D$(RS)**	-2659.864213	0.747294	-2659.116919
**$TS7_D$(SR)**	-2659.866329	0.751211	-2659.115118
**$TS7_D$(SS)**	-2659.864458	0.751233	-2659.113225
**$M7_D$(RR)**	-2659.859453	0.751799	-2659.107654

**M7$_D$(RS)**	-2659.87839	0.750653	-2659.127737
**M7$_D$(SR)**	-2659.878641	0.75164	-2659.127001
**M7$_D$(SS)**	-2659.870277	0.74882	-2659.121457
**TS8$_D$(RR)**	-2659.853934	0.755161	-2659.098773
**TS8$_D$(RS)**	-2659.866528	0.750023	-2659.116505
**TS8$_D$(SR)**	-2659.86227	0.748866	-2659.113404
**TS8$_D$(SS)**	-2659.849739	0.748277	-2659.101462
**SP$_D$(RR)**	-1606.648665	0.367004	-1606.281661
**SP$_D$(RS)**	-1606.648984	0.366628	-1606.282356
**SP$_D$(SR)**	-1606.648984	0.366628	-1606.282356
**SP$_D$(SS)**	-1606.648666	0.367003	-1606.281663
**R1^{HOBt}**	-1405.665356	0.31169	-1405.353666
**TS1^{HOBt}**	-2458.888279	0.692994	-2458.195285
**M1^{HOBt}**	-2458.892029	0.694227	-2458.197802
**TS6^{CO}**	-2658.614354	0.722395	-2657.891959
**M6^{CO}**	-2658.614402	0.721618	-2657.892784
**Pre-NHC**	-1053.684113	0.366525	-1053.317588
**M0^{DBU+R1}**	-2450.415371	0.837302	-2449.578069
**TS0^{DBU+R1}**	-2450.410489	0.83668	-2449.573809
**M0^{DBU}**	-1515.656156	0.602032	-1515.054124
**TS0^{DBU}**	-1515.65087	0.597035	-1515.053835

Table S14 The SPE, GFEC, GFE (=SPE+GFEC), and *ΔG* of different conformers of **TS6(R/S)** and **TS6(R/S)^{HOBt}** calculated at the L1 theory level

	SPE (a.u.)	GFEC (a.u.)	GFE (a.u.)	*ΔG* (kcal/mol)
**TS6(R)**	-2658.630201	0.718871	-2657.91133	0.0
**TS6(S)**	-2658.629936	0.719831	-2657.910105	0.8
**TS6(R)'**	-2658.618727	0.718887	-2657.89984	7.2
**TS6(S)'**	-2658.618567	0.718594	-2657.899973	7.1
**TS6(R)''**	-2658.617105	0.722797	-2657.894308	10.7
**TS6(S)''**	-2658.622498	0.723409	-2657.899089	7.7
**TS6(R)'''**	-2658.608335	0.717038	-2657.891297	12.6
**TS6(S)'''**	-2658.604644	0.71992	-2657.884724	16.7
**TS6(R)^{HOBt}**	-3129.55728	0.822349	-3128.734931	0.0
**TS6(S)^{HOBt}**	-3129.557739	0.826584	-3128.731155	2.4
**TS6(R)$^{HOBt'}$**	-3129.557235	0.824368	-3128.732867	1.3
**TS6(S)$^{HOBt'}$**	-3129.55253	0.822603	-3128.729927	3.1
**TS6(R)$^{HOBt''}$**	-3129.548546	0.819358	-3128.729188	3.6
**TS6(S)$^{HOBt''}$**	-3129.548819	0.824516	-3128.724303	6.7
**TS6(R)$^{HOBt'''}$**	-3129.546822	0.826435	-3128.720387	9.1
**TS6(S)$^{HOBt'''}$**	-3129.543427	0.823854	-3128.719573	9.6

3.3 Absolute SPE and GFE of the optimized structures by other DFT methods

Table S15 The SPE, GFEC, GFE (=SPE+GFEC), and *ΔG* of the stereochemical transition states for the reaction, which are calculated at the different computational levels (L2-5) and relative to the energy of $\mathbf{TS6(R/S)^{HOBt}_{Ln}}$ (n=2-5).

	SPE (a.u.)	GFEC (a.u.)	GFE (a.u.)	*ΔG* (kcal/mol)
L2	M06-2X/6-311++G(2df, 2pd)/IEF-PCM$_{THF}$// M06-2X/6-31G(d, p)/IEF-PCM$_{THF}$			
$\mathbf{TS6(R)^{HOBt}_{L2}}$	-3130.151412	0.822349	-3129.329063	0.0
$\mathbf{TS6(S)^{HOBt}_{L2}}$	-3130.151629	0.826584	-3129.325045	2.5
L3	B3LYP-D3/6-31++G(2df, 2pd)/IEF-PCM$_{THF}$// B3LYP/6-31G(d, p)/IEF-PCM$_{THF}$			
$\mathbf{TS6(R)^{HOBt}_{L3}}$	-3130.827035	0.803538	-3130.023497	0.0
$\mathbf{TS6(S)^{HOBt}_{L3}}$	-3130.819965	0.801564	-3130.018401	3.2
L4	M06-2X-D3/6-31++G(2df, 2pd)/SMD$_{THF}$// M06-2X/6-31G(d, p)/IEF-PCM$_{THF}$			
$\mathbf{TS6(R)^{HOBt}_{L4}}$	-3129.604528	0.822618	-3128.78191	0.0
$\mathbf{TS6(S)^{HOBt}_{L4}}$	-3129.593799	0.822924	-3128.770875	6.9
L5	ωB97X-D/6-311++G(2df, 2pd)/IEF-PCM$_{THF}$// M06-2X/6-31G(d, p)/IEF-PCM$_{THF}$			
$\mathbf{TS6(R)^{HOBt}_{L5}}$	-3130.358942	0.827267	-3129.531675	0.0
$\mathbf{TS6(S)^{HOBt}_{L5}}$	-3130.358836	0.828882	-3129.529954	1.1

Table S16 The SPE, GFEC, GFE (=SPE+GFEC), and *ΔG* of the stationary points involved in the proton transfer steps 4-5, which are computed at different computational levels.

computational level		SPE (a.u.)	GFEC (a.u.)	GFE (a.u.)	*ΔG* (kcal/mol)
M06-2X/6-31G(d,p)/IEF-PCM$_{THF}$	**IM3**	-2408.779175	0.828716	-2407.950459	0.0
	**TS4**	-2408.758961	0.829855	-2407.929106	13.4
	**IM4**	-2716.977927	1.082850	-2715.895077	0.0
	**TS5**	-2716.963494	1.079245	-2715.884249	6.8
B3LYP/6-31G(d,p)/IEF-PCM$_{THF}$	$\mathbf{IM3_{B3LYP}}$	-2409.778747	0.810715	-2408.968032	0.0
	$\mathbf{TS4_{B3LYP}}$	-2409.756782	0.808099	-2408.948683	12.1
	$\mathbf{IM4_{B3LYP}}$	-2718.153244	1.052253	-2717.100991	0.0
	$\mathbf{TS5_{B3LYP}}$	-2718.14653	1.056670	-2717.08986	7.0
ONIOM(M06-2X/6-31G(d, p):UFF)	$\mathbf{IM3_{explicit}}$	-2408.83893	12.453074	-2396.385856	0.0
	$\mathbf{TS4_{explicit}}$	-2408.821919	12.454003	-2396.367916	11.3
	$\mathbf{IM4_{explicit}}$	-2717.057201	12.703323	-2704.353878	0.0
	$\mathbf{TS5_{explicit}}$	-2717.047796	12.704151	-2704.343645	6.4
ONIOM(B3LYP/6-31G(d,p):UFF)	$\mathbf{IM3_{explicit}}$	-2409.829577	12.434487	-2397.39509	0.0
	$\mathbf{TS4_{explicit}}$	-2409.808733	12.431014	-2397.377719	10.9

3.4 Absolute SPE and GFE of the optimized structures for other cases

Table S17 The SPE, GFEC, GFE (=SPE+GFEC), and *ΔG* of all the active intermediates in **Scheme S6** calculated at the L1 theory level

	SPE (a.u.)	GFEC (a.u.)	GFE (a.u.)	*ΔG* (kcal/mol)
**NHC**	-1053.217708	0.351157	-1052.866551	0.0
**R1**	-933.5149469	0.18689	-933.3280569	0.0
**R2**	-1183.726653	0.221969	-1183.504684	0.0
**OAr^{-}**	-511.3709267	0.062634	-511.3082927	
**HOAr**	-511.8435473	0.076110	-511.7674373	
**TS1**	-1986.736349	0.568575	-1986.167774	16.8
**M1**	-1986.744560	0.571855	-1986.172705	13.7
**TS2**	-1986.743646	0.569843	-1986.173803	13.1
**M2**	-1986.746500	0.568877	-1986.177623	10.7
**M03**	-2658.649518	0.716531	-2657.932987	−0.7
**TS3(R)**	-2658.638904	0.720493	-2657.918411	8.4
**TS3(S)**	-2658.632558	0.720283	-2657.912275	12.2
**M3(R)**	-2658.651487	0.719479	-2657.932008	−0.1
**M3(S)**	-2658.653161	0.722760	-2657.930401	0.9
**TS4(S)^{HOAr}**	-3170.500858	0.813789	-3169.687069	7.7
**TS4(R)^{HOAr}**	-3170.500498	0.819263	-3169.681235	11.3
**M4(S)**	-2658.654614	0.723270	-2657.931344	0.3
**M4(R)**	-2658.651962	0.723869	-2657.928093	2.4

Table S18 The SPE, GFEC, GFE (=SPE+GFEC), and *ΔG* of all the active intermediates in **Scheme S4** calculated at the M06-2X-D3/6-31++G(2df,2pd)/IEF-PCM$_{toluene}$//M06-2X/6-31G(d,p)/IEF-PCM$_{toluene}$ theory level

	SPE (a.u.)	GFEC (a.u.)	GFE (a.u.)	*ΔG* (kcal/mol)
**NHCa**	-707.7282674	0.244058	-707.4842094	0.0
**1a**	-422.8423664	0.110318	-422.7320484	0.0
**2a**	-912.2395365	0.105376	-912.1341605	0.0
**2a^{-}**	-911.7335502	0.091943	-911.6416072	
**2a$^{-\ iPr_2NEtH+}$**	-1283.148955	0.358562	-1282.790393	
**^{i}Pr$_2$NEt**	-370.8945337	0.229548	-370.6649857	
**^{i}Pr$_2$NEt·H^{+}**	-371.3258858	0.24599	-371.0798958	
**DQ (toluene)**	-1241.114162	0.55964	-1240.554522	
**[DQH]$^{-}$ (toluene)**	-1241.848932	0.57289	-1241.276042	
**DQH$_2$ (toluene)**	-1242.363798	0.585894	-1241.777904	
**M2a**	-1130.61043	0.380304	-1130.230126	−8.7
**TS3(RS)a**	-2042.865333	0.515529	-2042.349804	0.4
**M3(RS)a**	-2042.89696	0.514616	-2042.382344	−20.0
**4a(RS)**	-1335.150514	0.246873	-1334.903641	−23.5
**TS3a**	-2371.757562	0.97646	-2370.781102	−6.5

**M3a**	-2371.771114	0.97846	-2370.792654	−13.7
**M04a**	-2041.666014	0.491009	-2041.175005	−30.1
**TS4(R)a**	-2041.656224	0.491048	-2041.165176	−23.9
**M4(R)a**	-2041.674778	0.493713	-2041.181065	−33.9
**3a(S)**	-1333.932698	0.222405	-1333.710293	−42.3

Table S19 The SPE, GFEC, GFE (=SPE+GFEC), and *ΔG* of all the active intermediates in **Scheme 5** calculated at the L1 theory level

	SPE (a.u.)	GFEC (a.u.)	GFE (a.u.)	*ΔG* (kcal/mol)
**NHCb**	-786.3278495	0.302948	-786.0249015	0.0
**1b(R1)**	-934.7339669	0.208309	-934.5256579	0.0
**2b**	-1528.012723	0.304797	-1527.707926	
**OAr^-**	-511.3709267	0.062634	-511.3082927	
**HOAr**	-511.8435473	0.07611	-511.7674373	
**DBU**	-461.9467352	0.213212	-461.7335232	
**$DBU{\cdot}H^+$**	-462.4149822	0.228326	-462.1866562	
**M1b**	-1721.080936	0.538895	-1720.542041	5.3
**M01b**	-1209.679854	0.45551	-1209.224344	11.2
**M2b**	-1209.214668	0.439362	-1208.775306	4.9
**M3b**	-1209.206253	0.438238	-1208.768015	9.5
**$2b^-$**	-1527.528499	0.292731	-1527.235768	
**$2b^{-DBUH+}$**	-1989.990077	0.547092	-1989.442985	
**H_2O**	-76.4105845	0.003992	-76.4065925	
**3b**	-1874.524782	0.429518	-1874.095264	−22.4
**S1-b**	-1950.908762	0.447146	-1950.461616	2.8
**S2-b**	-1950.916122	0.450166	-1950.465956	0.1
**S3-b**	-1950.91422	0.451002	-1950.463218	1.8
**S1-TS2b**	-3248.609754	0.864825	-3247.744929	
**S2-TS3b**	-2737.232506	0.778392	-2736.454114	
**M03b**	-2737.261186	0.777882	-2736.483304	
**TS4b**	-2737.242673	0.777345	-2736.465328	

Table S20 The SPE, GFEC, GFE (=SPE+GFEC), and *ΔG* of all the active intermediates in **Scheme 6** calculated at the L1 theory level

	SPE (a.u.)	GFEC (a.u.)	GFE (a.u.)	*ΔG* (kcal/mol)
**NHCc**	-1052.01911	0.334017	-1051.685093	0.0
**1c**	-424.0640509	0.132115	-423.9319359	0.0
**2c**	-345.694491	0.089002	-345.605489	
**$2c^{LiCl}$**	-813.5331311	0.088652	-813.4444791	
**DQ**	-1241.116619	0.559151	-1240.557468	
**$[DQH]^-$**	-1241.871297	0.571707	-1241.29959	

**DQH$_2$**	-1242.366799	0.585443	-1241.781356	
**HCO$_3^-$**	-264.4855664	0.001257	-264.4843094	
**H$_2$CO$_3$**	-264.9531776	0.014157	-264.9390206	
**M1c**	-1476.104505	0.490019	-1475.614486	1.6
**M2c**	-2717.289921	1.083407	-2716.206514	−20.1
**M02c**	-1475.360408	0.483135	-1474.877273	−1.5
**M3c**	-1474.906012	0.469786	-1474.436226	−27.0
**M4c**	-1474.900659	0.467368	-1474.433291	−25.2
**M5c**	-2716.069486	1.062939	-2715.006547	−35.1
**M05c**	-1474.151553	0.461752	-1473.689801	−24.3
**2c$^-$**	-345.2073628	0.07805	-345.1293128	
**2c^{-DQH2}**	-1587.593978	0.684292	-1586.909686	
**2c^{-LiCls}**	-1045.462873	0.190119	-1045.272754	
**3c**	-767.3834538	0.209027	-767.1744268	−49.8
**S1-c**	-768.5762293	0.22181	-768.3544193	−15.6
**S2-c**	-768.5793469	0.224777	-768.3545699	−25.7
**S3-c**	-768.5806289	0.229015	-768.3516139	−23.9
**S4-c**	-768.6050471	0.232186	-768.3728611	−37.2
**S1-TS3c**	-1820.592059	0.588436	-1820.003623	
**S3-TS4c**	-1820.595317	0.59451	-1820.000807	
**S4-TS5c**	-1820.589545	0.585311	-1820.004234	
**M05c**	-2519.660564	0.676259	-2518.984305	
**TS6c**	-2519.6478264	0.676319	-2518.971507	

3.5 The Cartesian coordinates of all the stationary points

NHC

0 1

C	-0.45450400	-0.27589900	0.24583200
C	-0.07710500	1.84082500	-0.44525700
C	1.94480600	0.53104800	0.20742800
H	2.13431200	0.62854400	1.28294900
N	-1.36635600	1.71705000	-0.50946800
N	-1.56332100	0.41666900	-0.08506800
N	0.50193500	0.67374800	-0.01162300
C	0.75631900	3.03502200	-0.80319400
H	0.29047100	3.94865700	-0.43098100
H	0.84828400	3.10834400	-1.89662500
C	2.63825600	1.68274200	-0.51985900
O	2.03175200	2.92338300	-0.20586700
C	-2.90192500	-0.08369400	-0.00576400
C	-3.32008500	-1.04809400	-0.92435300

C	-3.74534900	0.41298200	0.99462500
C	-4.62820900	-1.52479000	-0.81712100
C	-5.04369800	-0.08631200	1.05850800
C	-5.50153700	-1.05631400	0.16305900
H	-4.97200900	-2.27789900	-1.52207400
H	-5.71320900	0.28481100	1.83134900
H	3.68156500	1.74242400	-0.20618200
C	-2.38708300	-1.55475400	-1.99205100
H	-1.99122200	-0.72574800	-2.58720500
H	-1.52953500	-2.06581000	-1.54622500
H	-2.90460300	-2.24579300	-2.65959300
C	-6.91115100	-1.58057100	0.26586800
H	-7.08797400	-2.03408000	1.24566100
H	-7.63920400	-0.77301200	0.14541700
H	-7.10945800	-2.33450000	-0.49864100
C	-3.24918800	1.44308100	1.97391300
H	-2.30620300	1.12165400	2.42732300
H	-3.05615000	2.39661000	1.47472100
H	-3.98144000	1.60459800	2.76681400
H	2.60670400	1.50683200	-1.60788100
C	2.43267800	-0.83710400	-0.27825600
H	1.82240300	-1.60219300	0.20863800
H	2.24827600	-0.91157700	-1.35610600
C	3.89678600	-1.04399500	0.02633400
C	4.87068200	-0.88299600	-0.96233000
C	4.30436000	-1.36970100	1.32361900
C	6.22184600	-1.04481200	-0.66339500
H	4.56622000	-0.63315500	-1.97577500
C	5.65306200	-1.53165500	1.62634700
H	3.55439700	-1.50492400	2.09936300
C	6.61641900	-1.36933000	0.63203900
H	6.96578400	-0.91932300	-1.44368100
H	5.95216500	-1.78934500	2.63741000
H	7.66832500	-1.49851000	0.86541900

R1

0 1

C	-0.46670800	1.20151800	-0.00263500

O	0.06846700	2.16937500	0.46526800
C	-1.94001700	1.02004500	-0.24839200
C	-2.51498800	-0.20320200	0.48958100
H	-2.09434700	0.89813800	-1.32613200
H	-2.43883300	1.93702300	0.07043400
H	-1.99990800	-1.10349500	0.14362500
H	-2.31748100	-0.10125500	1.56183300
C	-3.99987100	-0.32532900	0.24894100
C	-4.48814100	-1.09091100	-0.81224000
C	-4.90932000	0.36219600	1.05626900
C	-5.85621000	-1.17207200	-1.06020300
H	-3.78900900	-1.63166400	-1.44562200
C	-6.27777700	0.28389600	0.81187600
H	-4.54012300	0.96013300	1.88619800
C	-6.75500900	-0.48467000	-0.24779800
H	-6.21968000	-1.77580500	-1.88586500
H	-6.97179300	0.82051800	1.45116100
H	-7.82151900	-0.54942200	-0.43767500
O	0.21634300	0.07581800	-0.37797500
C	1.58878400	0.02555400	-0.23770800
C	2.10598900	-1.11643000	0.36742100
C	2.41607200	1.02791200	-0.74224100
C	3.48062900	-1.26607100	0.47923600
H	1.42501600	-1.87190400	0.74137500
C	3.79104200	0.88424600	-0.62753400
H	1.98895300	1.90423100	-1.21211500
C	4.29668200	-0.25792600	-0.01818000
H	3.91965200	-2.13995700	0.94308300
H	4.46919700	1.63850000	-1.00539300
N	5.74938600	-0.40649900	0.10137000
O	6.45213700	0.49550300	-0.32102600
O	6.17770700	-1.42470700	0.61712500

R2

0 1

C	1.82409300	-0.81754200	0.05496300
C	2.26396600	-2.25846300	0.02341800
H	3.17038200	-2.38432800	0.61664300

H	1.49839500	-2.93222900	0.40184900
H	2.49032900	-2.53877200	-1.01062500
C	2.87597900	0.23350800	0.00217500
C	4.18788000	-0.08020200	-0.37110200
C	2.55135600	1.56162600	0.31021200
C	5.15592800	0.91706600	-0.44004300
H	4.46036600	-1.09858600	-0.62565300
C	3.52205000	2.55201400	0.25281300
H	1.53367100	1.79858100	0.59968100
C	4.82644200	2.23221700	-0.12483000
H	6.16763100	0.66426100	-0.73866600
H	3.26365300	3.57577800	0.50219800
H	5.58396600	3.00790900	-0.17300300
N	0.60611700	-0.39727100	0.10247000
S	-0.65189700	-1.51568500	0.14709000
O	-0.61880900	-2.35333900	-1.05104300
O	-0.67371300	-2.16055700	1.46062800
C	-2.04437200	-0.43021800	0.03222900
C	-2.69563200	-0.28798700	-1.18621800
C	-2.46182000	0.25698600	1.17014100
C	-3.78900000	0.57029800	-1.26377500
H	-2.35303300	-0.84419100	-2.05205500
C	-3.55154300	1.11013300	1.07128500
H	-1.94347500	0.11837200	2.11338500
C	-4.22960900	1.27782200	-0.14336700
H	-4.30726300	0.69058500	-2.21035000
H	-3.88693700	1.65484200	1.94921300
C	-5.42505900	2.18984500	-0.22291900
H	-5.69312800	2.40293800	-1.25932500
H	-6.29104100	1.72881900	0.26226100
H	-5.22976600	3.13642300	0.28714900

DBU

0 1

C	2.75251600	0.00103200	0.38814800
C	1.90132000	-1.24983200	0.61424700
C	0.89024000	-1.54824100	-0.50715600
C	1.96479500	1.30789000	0.29632500

C	-0.40265900	-0.77766400	-0.36464900
C	0.90541800	1.29077300	-0.80940100
H	3.31849700	-0.12902800	-0.54483200
H	2.57286700	-2.10947600	0.70814400
H	1.35275300	-1.35728400	-1.48398100
H	1.46777100	1.52362800	1.25117800
H	0.61288800	2.31400800	-1.06267800
C	-2.69844000	-0.67514500	0.01546900
H	-3.44127100	-1.28983800	0.53312500
C	-1.42059200	1.44085200	0.01147700
C	-2.44505300	0.62164700	0.77923100
H	-2.05298900	0.37456800	1.77196700
H	-3.36175900	1.20200000	0.91254100
H	-1.00519700	2.23046900	0.65023800
N	-0.32143800	0.59214700	-0.44617200
N	-1.47656800	-1.45273100	-0.14053600
H	1.32703000	0.85786600	-1.72418700
H	2.66257900	2.13090700	0.10556100
H	3.49299000	0.08180400	1.19135500
H	1.36128900	-1.17056700	1.56653300
H	0.60349700	-2.60033500	-0.48552100
H	-3.11941200	-0.43535900	-0.97332600
H	-1.89076400	1.93285300	-0.85130600

DBU·H$^+$

1 1

C	-2.79106400	-0.01651900	-0.38827100
C	-1.94124400	-1.27374200	-0.57263000
C	-0.91587300	-1.51086600	0.55354000
C	-2.00441800	1.29413600	-0.36107100
C	0.33108000	-0.69402900	0.37206000
C	-0.95522900	1.34875400	0.74826900
H	-3.35230000	-0.10804000	0.55064800
H	-2.60092300	-2.14466100	-0.59957800
H	-1.35782100	-1.27775300	1.52840500
H	-1.50983700	1.47176600	-1.32365500
H	-0.65126500	2.37802900	0.94335700
C	2.74438100	-0.66745900	-0.09707300

H	3.34585400	-1.30913500	-0.74056100
C	1.46224700	1.45239000	0.09028700
C	2.46431600	0.67698700	-0.74905400
H	2.06206400	0.51916500	-1.75367900
H	3.38662500	1.25285600	-0.83616400
H	1.10047800	2.32495400	-0.45816500
N	0.28414400	0.62513600	0.40794400
N	1.46455400	-1.33543600	0.13345500
H	-1.35405100	0.95240700	1.68717800
H	-2.70162500	2.12235000	-0.20340800
H	-3.53207400	0.02873300	-1.19170000
H	-1.41750400	-1.24481900	-1.53529200
H	-0.62460800	-2.56259700	0.58293700
H	3.26626000	-0.54360300	0.85698700
H	1.90673300	1.79862500	1.02918200
H	1.42792800	-2.34409400	0.09736600

OAr$^-$

-1 1

O	-3.44857300	-0.00000300	0.00003500
C	-2.19812300	0.00000100	-0.00000300
C	-1.41528500	-1.22579800	0.00000300
C	-1.41528900	1.22580100	-0.00001100
C	-0.04754300	-1.22171100	-0.00003400
H	-1.96528200	-2.16258500	-0.00001600
C	-0.04754300	1.22171200	-0.00002500
H	-1.96528100	2.16259000	0.00002200
C	0.65886700	0.00000200	-0.00003000
H	0.51691500	-2.14768100	-0.00005900
H	0.51691700	2.14768200	-0.00001300
N	2.06864200	0.00000000	-0.00000600
O	2.67464900	1.07976200	0.00001800
O	2.67464100	-1.07976600	0.00003500

HOAr

0 1

O	3.41366500	0.08765800	0.00038500
C	2.06839300	0.01473000	-0.00021400

C	1.36654100	1.22733000	0.00037700
C	1.37980500	-1.20404800	-0.00067800
C	-0.01618100	1.22239200	0.00026500
H	1.92580100	2.15558700	0.00078000
C	-0.00567500	-1.21211300	-0.00050400
H	1.93011400	-2.13960100	-0.00108800
C	-0.68530500	0.00064600	-0.00002800
H	-0.58194200	2.14545700	0.00062100
H	-0.56139000	-2.14128400	-0.00079900
N	-2.14203000	-0.00733300	0.00016200
O	-2.71150700	-1.08754000	0.00125400
O	-2.72264100	1.06680000	-0.00113300
H	3.80001900	-0.79779300	0.00000200

OBt^-

-1 1

C	0.19694000	-0.39480400	0.00001000
C	-0.22760700	0.94631300	-0.00009100
C	-1.61363600	1.23057400	0.00000500
C	-2.48579200	0.16481800	0.00020400
C	-2.02904900	-1.18575500	0.00032300
C	-0.68504100	-1.48645400	0.00023000
H	-1.96988600	2.25619800	-0.00005800
H	-3.55572600	0.35038700	0.00029000
H	-2.76114800	-1.98732900	0.00049100
H	-0.30793300	-2.50345700	0.00031300
N	0.86580500	1.74825700	-0.00023000
N	1.93462500	0.95663600	-0.00027900
N	1.55871400	-0.31788200	-0.00016500
O	2.39322500	-1.30912900	-0.00005000

HOBt

0 1

C	0.14991800	-0.40151500	0.00005700
C	-0.29570600	0.93132600	-0.00007700
C	-1.67704300	1.20951200	0.00000500
C	-2.53487800	0.13329600	0.00022900
C	-2.05839100	-1.20696800	0.00037000

C	-0.71488400	-1.50860800	0.00028400
H	-2.03485000	2.23295800	-0.00010100
H	-3.60615000	0.30239000	0.00029900
H	-2.78099800	-2.01622000	0.00053800
H	-0.34251500	-2.52565600	0.00036700
N	0.79252900	1.76517100	-0.00027400
N	1.86065300	1.02756600	-0.00007300
N	1.49222800	-0.24399800	-0.00014600
O	2.40830900	-1.24839000	-0.00032500
H	3.26607500	-0.78978300	-0.00026500

DQ

0 1

C	2.80665300	1.28429000	0.01001600
C	1.45951900	1.23582800	-0.06348900
C	0.69361100	0.00047500	-0.09145500
C	1.46139500	-1.23369800	-0.10829700
C	2.80859800	-1.28003700	-0.03314200
C	3.56785600	0.00274200	0.08161100
H	0.91905700	2.16850700	-0.09498300
H	0.92234700	-2.16725300	-0.18700900
C	-0.69357500	-0.00056700	-0.09125600
C	-1.45948000	-1.23591800	-0.06278100
C	-1.46135200	1.23361000	-0.10817700
C	-2.80660800	-1.28436200	0.01083600
H	-0.91901700	-2.16861800	-0.09394800
C	-2.80855000	1.27996900	-0.03293700
H	-0.92230200	2.16715500	-0.18699000
C	-3.56781400	-0.00278600	0.08206200
O	4.78481800	-0.00152500	0.22926900
O	-4.78476100	0.00152700	0.22984600
C	3.58729500	-2.59513600	-0.05758700
C	4.58115000	-2.59545200	-1.23541300
C	4.34624500	-2.77828800	1.27098800
C	2.65465300	-3.80194000	-0.23202400
H	4.05149100	-2.46524000	-2.18560300
H	5.31919200	-1.80014800	-1.12960700
H	5.10533300	-3.55794100	-1.26852100

H	3.65034800	-2.79211900	2.11177300
H	4.87726200	-3.73491300	1.25725100
H	5.07544000	-1.98116600	1.42807700
H	3.25418300	-4.71108200	-0.25996600
H	1.94848400	-3.89165100	0.59797000
H	2.09412700	-3.74608700	-1.17127900
C	3.58324200	2.60064900	0.04459600
C	4.33674700	2.73002700	1.37945100
C	4.57700000	2.64974600	-1.12929200
C	2.64861500	3.80577600	-0.08392300
H	3.63966200	2.69539100	2.22424000
H	5.07243400	1.93419600	1.49953900
H	4.85581000	3.69550800	1.41058000
H	4.05148700	2.56347300	-2.08737100
H	5.10470700	3.61074100	-1.11920500
H	5.31643200	1.84940000	-1.05812400
H	3.24263700	4.72631400	-0.07602300
H	2.08185100	3.78592800	-1.02321400
H	1.93821500	3.86765500	0.74713400
C	-3.58724700	2.59507000	-0.05755900
C	-4.34738900	2.77844300	1.27098900
C	-2.65461400	3.80185600	-0.23221000
C	-4.58110700	2.59517800	-1.23538800
H	-5.07536400	1.98132700	1.42423100
H	-3.65028500	2.78844000	2.11577000
H	-4.87723500	3.73505200	1.25708200
H	-2.09411800	3.74585700	-1.17147700
H	-3.25415000	4.71499400	-0.25627600
H	-1.94841800	3.89170100	0.59774900
H	-5.10529500	3.55766200	-1.26866200
H	-4.05144800	2.46880700	-2.18555900
H	-5.31914500	1.80388600	-1.13344400
C	-3.58318100	-2.60072900	0.04590300
C	-4.33661800	-2.72967500	1.38084800
C	-4.57698700	-2.65024600	-1.12793600
C	-2.64853300	-3.80589100	-0.08104400
H	-3.63949800	-2.69468100	2.22560400
H	-5.07235900	-1.93384900	1.50068200

H	-4.85561200	-3.69519100	1.41235700
H	-4.05150900	-2.56430600	-2.08607400
H	-5.10348800	-3.61124700	-1.11748700
H	-5.31641500	-1.84986800	-1.06101600
H	-3.24253700	-4.72644200	-0.07402300
H	-2.08179600	-3.78634200	-1.02036300
H	-1.93811000	-3.86747600	0.75282100

DQ·−

-1 2

C	2.83923500	1.23736600	0.19891800
C	1.46828400	1.19491800	0.18821500
C	0.71865100	0.00010800	-0.00018400
C	1.46823900	-1.19472200	-0.18865400
C	2.83920200	-1.23720200	-0.19960200
C	3.60178000	0.00010800	-0.00049700
H	0.91855900	2.10891300	0.37650200
H	0.91841300	-2.10870900	-0.37669100
C	-0.71864600	0.00006400	-0.00002400
C	-1.46816100	-1.19473800	0.18884100
C	-1.46835300	1.19479100	-0.18871800
C	-2.83911000	-1.23726600	0.19989400
H	-0.91830700	-2.10864500	0.37720200
C	-2.83931800	1.23717900	-0.19936700
H	-0.91864900	2.10875100	-0.37721400
C	-3.60177400	-0.00009300	0.00035700
O	4.86020000	0.00014100	-0.00063700
O	-4.86019500	-0.00016200	0.00051400
C	3.59978000	-2.54786900	-0.42519100
C	4.48633800	-2.43720200	-1.67904300
C	4.47592500	-2.85830800	0.80252000
C	2.65533500	-3.73817600	-0.63137700
H	3.87050600	-2.24038700	-2.56341300
H	5.21091200	-1.63112600	-1.56752700
H	5.02128600	-3.38019400	-1.84207300
H	3.85132700	-2.98027000	1.69424400
H	5.02443400	-3.79404300	0.64256000
H	5.18854700	-2.05284900	0.97815100

H	3.24949500	-4.64614200	-0.77710000
H	2.00635900	-3.89985400	0.23599800
H	2.02288800	-3.60831200	-1.51599200
C	3.59979200	2.54801100	0.42447100
C	4.48646500	2.43731700	1.67825500
C	4.47580300	2.85849900	-0.80330800
C	2.65521600	3.73819600	0.63077100
H	3.87071500	2.24042400	2.56266400
H	5.21106800	1.63128800	1.56664000
H	5.02137300	3.38032600	1.84128700
H	3.85112400	2.98043200	-1.69498100
H	5.02427200	3.79426600	-0.64339100
H	5.18846000	2.05308500	-0.97902400
H	3.24927300	4.64626300	0.77628600
H	2.00601300	3.89970400	-0.23647400
H	2.02297200	3.60832800	1.51553400
C	-3.60002800	2.54772200	-0.42516400
C	-4.47617100	2.85825600	0.80252300
C	-2.65571600	3.73809000	-0.63159600
C	-4.48662600	2.43673300	-1.67895600
H	-5.18875100	2.05278000	0.97825600
H	-3.85156500	2.98035300	1.69422600
H	-5.02473500	3.79394000	0.64246300
H	-2.02327800	3.60812500	-1.51620100
H	-3.24998200	4.64596300	-0.77747900
H	-2.00674000	3.90001800	0.23573300
H	-5.02163300	3.37965900	-1.84216400
H	-3.87081100	2.23976800	-2.56330600
H	-5.21115300	1.63064000	-1.56724900
C	-3.59953600	-2.54790300	0.42597600
C	-4.48566400	-2.43705300	1.68013300
C	-4.47606900	-2.85862200	-0.80137600
C	-2.65486000	-3.73804700	0.63205700
H	-3.86952000	-2.24009100	2.56425400
H	-5.21028600	-1.63100000	1.56877000
H	-5.02053000	-3.38002900	1.84348900
H	-3.85177300	-2.98070500	-1.69329900
H	-5.02444000	-3.79437600	-0.64105400

H	-5.18883400	-2.05327200	-0.97692800
H	-3.24885000	-4.64609400	0.77796700
H	-2.00603700	-3.89969000	-0.23544500
H	-2.02223600	-3.60803600	1.51652700

[DQH]·

0 2

C	2.85021800	-1.22823000	-0.31937200
C	1.47856200	-1.17984600	-0.30676400
C	0.75906200	-0.00007400	0.00115000
C	1.48087000	1.17819000	0.30937400
C	2.85258900	1.22423400	0.32137800
C	3.60256200	-0.00263200	0.00078900
H	0.90918500	-2.06079100	-0.57648400
H	0.91313600	2.06026800	0.57856600
C	-0.70852200	0.00257200	-0.00036300
C	-1.42780500	1.16651000	-0.30466300
C	-1.43199200	-1.15644600	0.30281500
C	-2.81669400	1.20670200	-0.31937300
H	-0.87331800	2.05648700	-0.57233100
C	-2.82343300	-1.18895200	0.31292200
H	-0.88227100	-2.04828400	0.57362600
C	-3.50150500	0.00996900	-0.00582200
O	4.85241400	-0.00377000	0.00023100
O	-4.86115300	0.07614800	-0.02857800
C	3.61598200	2.50156100	0.66782800
C	4.49056500	2.27122300	1.91478400
C	4.50021400	2.92005400	-0.52311300
C	2.66487500	3.66545700	0.97398500
H	3.87154800	1.97552000	2.76808400
H	5.23703400	1.49815100	1.73594400
H	5.00344100	3.20318600	2.17553500
H	3.88576200	3.10287000	-1.41081900
H	5.02455500	3.84964200	-0.27708700
H	5.23672700	2.15131100	-0.75513800
H	3.25653900	4.55574800	1.20567400
H	2.02368700	3.90437300	0.11916800
H	2.02829800	3.45471900	1.83937000

C	3.61157100	-2.50622000	-0.66779600
C	4.48415300	-2.27580900	-1.91616900
C	4.49761800	-2.92653600	0.52112000
C	2.65868800	-3.66884200	-0.97320400
H	3.86392000	-1.97872100	-2.76809800
H	5.23170800	-1.50363100	-1.73802900
H	4.99566500	-3.20805200	-2.17861400
H	3.88473300	-3.10942700	1.40990300
H	5.02063200	-3.85641300	0.27336900
H	5.23527700	-2.15864900	0.75230500
H	3.24895400	-4.55968500	-1.20630000
H	2.01854500	-3.90745300	-0.11749800
H	2.02109700	-3.45681200	-1.83752700
C	-3.58086100	-2.48228400	0.66505800
C	-4.43424400	-2.95513800	-0.53262200
C	-2.61591100	-3.63534900	0.97893500
C	-4.43733200	-2.28725300	1.93503700
H	-5.16951500	-2.22975400	-0.89143100
H	-3.78271600	-3.18553200	-1.38025400
H	-4.97804500	-3.86494900	-0.26107500
H	-1.98305000	-3.40982500	1.84211100
H	-3.19832900	-4.52979000	1.21679000
H	-1.97449500	-3.87201200	0.12483100
H	-4.96930500	-3.21515400	2.16571100
H	-3.78951800	-2.04420200	2.78197300
H	-5.18397000	-1.49093600	1.87252000
C	-3.57181700	2.49956500	-0.66731600
C	-4.43851300	2.29552200	-1.92342200
C	-4.44835100	2.94036200	0.52032600
C	-2.60343600	3.65229500	-0.96775600
H	-3.81410600	2.00126300	-2.77307500
H	-5.20235800	1.53353900	-1.77189300
H	-4.93577800	3.23725100	-2.17941500
H	-3.82864700	3.11527000	1.40577200
H	-4.95434500	3.87919500	0.27099500
H	-5.20519100	2.19565400	0.76561000
H	-3.18444900	4.54908000	-1.20094700
H	-1.96383200	3.88414800	-0.10999100

H	-1.96598600	3.43591400	-1.83086600
H	-5.24400100	-0.77617200	0.19929600

[DQH]$^{-}$

-1 1

C	-2.88372200	-1.19081400	0.32432000
C	-1.49729600	-1.15272800	0.32687600
C	-0.75910500	0.00244100	0.02763700
C	-1.48966800	1.15945500	-0.28288200
C	-2.87578400	1.20040700	-0.30354100
C	-3.64311400	0.00541000	0.00313100
H	-0.94454900	-2.04717200	0.59990300
H	-0.93107800	2.05202400	-0.54985400
C	0.71771200	-0.00173700	0.03038000
C	1.45112100	1.15103600	0.33764100
C	1.44247900	-1.15727900	-0.28787500
C	2.84680500	1.18247600	0.33615500
H	0.89854300	2.04255500	0.60621200
C	2.83761500	-1.20037900	-0.28634400
H	0.88201700	-2.03939500	-0.57023700
C	3.52421100	-0.01593000	0.04244300
O	-4.92077600	0.00621200	-0.01017100
O	4.91843800	-0.01183400	0.02053000
C	-3.62430600	2.49064000	-0.66083300
C	-4.49029400	2.26633500	-1.91304500
C	-4.52394000	2.91321200	0.51488300
C	-2.67699300	3.65930000	-0.95838500
H	-3.85635900	2.02359700	-2.77347700
H	-5.18274700	1.44259200	-1.74057500
H	-5.05639000	3.17487600	-2.15443400
H	-3.91285300	3.14416600	1.39499300
H	-5.09601100	3.81259900	0.25385800
H	-5.21183300	2.10454300	0.76206700
H	-3.26638600	4.55191200	-1.19531700
H	-2.04047800	3.89687000	-0.09893300
H	-2.02873600	3.45011100	-1.81618800
C	-3.64136500	-2.48140600	0.66068200
C	-4.54060600	-2.26089500	1.88980300

C	-4.50994600	-2.90068000	-0.53946900
C	-2.70192100	-3.65072100	0.98022800
H	-3.93054700	-2.01236300	2.76567700
H	-5.23438400	-1.44244600	1.69832200
H	-5.10607100	-3.17321900	2.11810600
H	-3.87616300	-3.12975200	-1.40384300
H	-5.08934200	-3.80022600	-0.29567500
H	-5.19041300	-2.09064100	-0.80239800
H	-3.29712300	-4.54355400	1.20141100
H	-2.04433100	-3.88749000	0.13660400
H	-2.07507400	-3.44249800	1.85411800
C	3.58476200	-2.48846300	-0.67646200
C	4.50947800	-2.96362600	0.46017500
C	2.61243600	-3.64042800	-0.96941100
C	4.40982600	-2.24665900	-1.95268500
H	5.36631900	-2.30262500	0.59630400
H	3.96178900	-3.03368300	1.40537000
H	4.90337000	-3.95794400	0.22354100
H	1.94466100	-3.40633400	-1.80356100
H	3.18751300	-4.53009100	-1.24317300
H	2.00110300	-3.89007100	-0.09620400
H	4.93401700	-3.16642600	-2.23650100
H	3.75335800	-1.96096600	-2.78076000
H	5.14639800	-1.45706600	-1.80250200
C	3.60534900	2.48655600	0.63941000
C	4.50416800	2.33061600	1.87918700
C	4.45798600	2.88710300	-0.57843600
C	2.64495600	3.64926400	0.92770700
H	3.92279200	1.97910400	2.73757100
H	5.32664300	1.63675200	1.70352900
H	4.94190200	3.30004900	2.14177100
H	3.81834200	3.04888200	-1.45219400
H	4.98814200	3.82311600	-0.36846600
H	5.19059600	2.11618100	-0.81878400
H	3.22983100	4.55615100	1.10934600
H	1.97753300	3.84554800	0.08302800
H	2.03312500	3.46248800	1.81591700
H	5.23650500	-0.30021300	0.88482800

DQH$_2$

0 1

C	2.84798400	-1.18749500	-0.38142700
C	1.45523000	-1.14385400	-0.36661100
C	0.74215400	-0.00302900	0.00731000
C	1.46445000	1.13045100	0.37753900
C	2.86008500	1.16326500	0.37889900
C	3.53576800	-0.01347600	-0.00760600
H	0.89183300	-2.01564500	-0.67682600
H	0.90985200	2.00551100	0.69385500
C	-0.74213400	0.00313500	0.00735700
C	-1.45525000	1.14397500	-0.36646800
C	-1.46439100	-1.13037200	0.37758800
C	-2.84800700	1.18757600	-0.38123400
H	-0.89186800	2.01579100	-0.67664300
C	-2.86004700	-1.16320800	0.37899400
H	-0.90977200	-2.00543400	0.69385000
C	-3.53573500	0.01352100	-0.00742200
O	4.90298800	-0.07975600	-0.04061900
O	-4.90299800	0.07976200	-0.04034400
C	3.61790200	2.43669100	0.79786700
C	4.48469700	2.17244900	2.04807300
C	4.46152100	2.97995200	-0.37625200
C	2.65392900	3.56862200	1.18385500
H	3.84323200	1.88438300	2.88576500
H	5.22725200	1.37838000	1.93400500
H	5.02108400	3.08516700	2.32541300
H	3.80276600	3.26088100	-1.20292800
H	5.01006300	3.87157600	-0.05707700
H	5.19095400	2.27517200	-0.78471500
H	3.23616300	4.44979400	1.46841400
H	2.00607200	3.85025500	0.34863400
H	2.02620400	3.29236100	2.03587700
C	3.59846200	-2.46207300	-0.80075000
C	4.45735800	-2.19442000	-2.05028400
C	4.48256700	-2.96696600	0.35479100
C	2.62651100	-3.59657500	-1.15491900

H	3.82703600	-1.85665200	-2.87933600
H	5.22096200	-1.43983700	-1.86374700
H	4.95351100	-3.12117800	-2.35874900
H	3.86811700	-3.19059200	1.23296600
H	4.98866500	-3.89033000	0.05241200
H	5.23886700	-2.23447900	0.63615000
H	3.20372200	-4.48232100	-1.43605400
H	1.98988800	-3.86807700	-0.30672700
H	1.98477100	-3.33455300	-2.00194000
C	-3.61779300	-2.43670000	0.79783900
C	-4.46152600	-2.97969700	-0.37632200
C	-2.65379700	-3.56867600	1.18354900
C	-4.48453200	-2.17272300	2.04819800
H	-5.19138000	-2.27497100	-0.78410900
H	-3.80292500	-3.25996100	-1.20334300
H	-5.00962200	-3.87170300	-0.05743000
H	-2.02599500	-3.29255900	2.03555400
H	-3.23604800	-4.44987400	1.46800000
H	-2.00604100	-3.85022600	0.34821900
H	-5.02099700	-3.08547500	2.32526900
H	-3.84301300	-1.88500900	2.88596900
H	-5.22698200	-1.37852300	1.93443700
C	-3.59851300	2.46206500	-0.80076500
C	-4.45719400	2.19405700	-2.05038000
C	-4.48292200	2.96699700	0.35454200
C	-2.62669500	3.59665300	-1.15495200
H	-3.82667800	1.85630900	-2.87929300
H	-5.22065100	1.43932900	-1.86382300
H	-4.95353200	3.12065400	-2.35903500
H	-3.86868000	3.19065300	1.23285800
H	-4.98891400	3.89035300	0.05197800
H	-5.23927300	2.23451200	0.63571100
H	-3.20402200	4.48217100	-1.43657100
H	-1.99043100	3.86860000	-0.30663500
H	-1.98460900	3.33451700	-2.00167500
H	-5.28253000	-0.76490200	0.21682100
H	5.28253900	0.76501200	0.21616300

TS1

0 1

C	-0.26723500	-1.27109100	0.32875900
C	-0.97481800	-3.24196800	1.08070400
C	-2.75900500	-1.79491300	0.08911600
H	-3.15408300	-1.04158000	0.77846600
N	0.30603500	-3.25929300	1.28145200
N	0.71957600	-2.03726900	0.80659200
N	-1.36304600	-2.06738900	0.48244400
C	-1.97186500	-4.30167800	1.44301500
H	-1.74159300	-4.70335200	2.43071400
H	-1.93227500	-5.11948900	0.70953800
C	-3.55939900	-3.09069200	0.26472800
O	-3.26015800	-3.72997200	1.48870900
C	2.13489000	-1.79312000	0.75845000
C	2.76899600	-1.83664500	-0.48571100
C	2.82186700	-1.58704500	1.95509800
C	4.15054800	-1.65414600	-0.50636700
C	4.20629800	-1.43370300	1.88450900
C	4.88465400	-1.46146100	0.66499400
H	4.66707200	-1.66768700	-1.46356400
H	4.76379200	-1.27150300	2.80395600
H	-4.62388000	-2.85261100	0.26569800
C	-0.14341800	0.64057700	-0.31274000
O	-0.20938200	0.67154200	-1.54277400
C	-1.24698000	1.23441700	0.55299600
C	-1.36290200	2.74330700	0.28634600
H	-1.05532300	1.03716100	1.61194900
H	-2.18983500	0.75769200	0.28061000
H	-0.44810400	3.23967100	0.62804600
H	-1.43850300	2.89629800	-0.79451900
C	-2.57034900	3.32553700	0.97823500
C	-2.54317700	3.59559900	2.34980200
C	-3.75575500	3.55700300	0.27490200
C	-3.67146800	4.08305700	3.00305700
H	-1.62475900	3.42456500	2.90674500
C	-4.88880600	4.04282600	0.92520100
H	-3.78753100	3.35969200	-0.79440400

C	-4.84954100	4.30614100	2.29223500
H	-3.63149000	4.29188700	4.06760800
H	-5.79930600	4.22387100	0.36196800
H	-5.72932500	4.68796300	2.80018000
C	2.07960500	-1.50742200	3.26183500
H	1.29951900	-0.74015600	3.20904700
H	1.58791500	-2.45560400	3.49620300
H	2.75944600	-1.25541100	4.07722500
C	6.37317800	-1.23893400	0.60086700
H	6.84172300	-1.40262000	1.57370500
H	6.84056200	-1.90845400	-0.12574500
H	6.59715600	-0.21150700	0.29030200
C	1.98558200	-2.06554100	-1.75192000
H	1.42913700	-3.00763100	-1.69950300
H	1.26116600	-1.26129000	-1.92268800
H	2.65855000	-2.11048500	-2.61020200
O	1.07987700	0.95495200	0.36729200
C	2.26065500	1.23888600	-0.23104300
C	3.28457100	1.53568500	0.68309700
C	2.52370900	1.26757800	-1.61113600
C	4.56506900	1.80869000	0.24694100
H	3.04724800	1.52086500	1.74075700
C	3.81184000	1.54056300	-2.04802800
H	1.73507900	1.06623600	-2.31824200
C	4.81823400	1.79026600	-1.12239600
H	5.36525800	2.02087200	0.94554700
H	4.04374400	1.55201700	-3.10600000
N	6.17144700	2.03694400	-1.59345200
O	6.36216500	2.11195400	-2.79712400
O	7.06001100	2.15237700	-0.75959100
H	-3.35265900	-3.77043800	-0.57711400
C	-2.84138200	-1.30605700	-1.36231200
H	-2.14436200	-0.47630900	-1.51814400
H	-2.50961600	-2.12141200	-2.01546200
C	-4.25034300	-0.88895500	-1.71762900
C	-5.03957200	-1.65810700	-2.57493600
C	-4.79354500	0.28067700	-1.17498800
C	-6.33907000	-1.26501500	-2.89036800

H	-4.62952700	-2.56811600	-3.00550800
C	-6.09022800	0.67610700	-1.48654700
H	-4.19595200	0.89952400	-0.50895300
C	-6.86774500	-0.09698700	-2.34760600
H	-6.93738200	-1.87223900	-3.56214700
H	-6.48999800	1.59086600	-1.05925500
H	-7.87860500	0.21031100	-2.59511700

M1

0 1

C	-0.30828700	-1.27826500	0.50081200
C	-1.07336500	-3.20341300	1.22066400
C	-2.85700700	-1.60005700	0.48093100
H	-3.10263600	-0.74814500	1.11916500
N	0.21754100	-3.33868400	1.21407200
N	0.67788600	-2.13941600	0.75164200
N	-1.44623400	-1.96516300	0.75645300
C	-2.09854600	-4.17539300	1.72422100
H	-1.73842400	-4.63534000	2.64524000
H	-2.27746000	-4.96136800	0.97788600
C	-3.74405400	-2.79984700	0.85972700
O	-3.28250400	-3.46845800	2.01340400
C	2.07375200	-2.02976400	0.40601600
C	2.42523900	-2.33942400	-0.91063900
C	2.99547000	-1.66320900	1.38358300
C	3.77168700	-2.23025000	-1.24971200
C	4.32682900	-1.54708600	0.98691300
C	4.72854600	-1.80996500	-0.32372700
H	4.07508300	-2.44962700	-2.27072100
H	5.06401700	-1.22656600	1.71890100
H	-4.75187000	-2.43699900	1.06402500
C	-0.15815500	0.18381900	-0.07144900
O	-0.09009800	0.18158600	-1.34539600
C	-1.19048300	1.13808300	0.56951900
C	-0.89201800	2.58634800	0.16726200
H	-1.21119100	1.03318300	1.66086100
H	-2.18032900	0.88822500	0.18256100
H	0.00797000	2.93325000	0.68402800

H	-0.68398600	2.59185400	-0.90772600
C	-2.06243300	3.48414600	0.48211800
C	-2.12329400	4.20337000	1.67759600
C	-3.14558900	3.56305500	-0.39998200
C	-3.23642900	4.98325800	1.98601200
H	-1.28774000	4.15239800	2.37144000
C	-4.26230000	4.33698400	-0.09480800
H	-3.10891900	3.00982400	-1.33652800
C	-4.31030000	5.05080700	1.10176300
H	-3.26400400	5.53977300	2.91776000
H	-5.09100100	4.38891400	-0.79464500
H	-5.17716000	5.65820600	1.34140900
C	6.15675700	-1.59789400	-0.75249400
H	6.82512300	-1.52767900	0.10826900
H	6.50264800	-2.41113100	-1.39585700
H	6.24509200	-0.66631100	-1.32415500
C	2.54902500	-1.35436600	2.78493200
H	1.84261000	-0.51809600	2.77383900
H	2.04107400	-2.21379000	3.23338800
H	3.40137200	-1.08842800	3.41206700
C	1.37762600	-2.72977400	-1.91801100
H	0.79040300	-3.58514300	-1.56657900
H	0.69398100	-1.88768300	-2.08108300
H	1.84165000	-3.00093200	-2.86761100
O	1.09995100	0.61472100	0.72713700
C	2.23806200	1.06568500	0.19352800
C	3.09575900	1.72032400	1.10422600
C	2.65829400	0.88008400	-1.13765700
C	4.36301300	2.11721900	0.72826100
H	2.74199400	1.87440500	2.11807600
C	3.93251600	1.27263900	-1.51058400
H	1.99304800	0.40290500	-1.84103700
C	4.77833600	1.86850300	-0.57896200
H	5.03724700	2.59635500	1.42735900
H	4.28767000	1.10332100	-2.52037800
N	6.12636800	2.22582600	-0.97120000
O	6.52388600	1.87144000	-2.07480500
O	6.81378300	2.85824300	-0.18186700

H	-3.79199400	-3.50239400	0.01369700
C	-3.04569500	-1.26863400	-1.00758400
H	-2.33044300	-0.50237500	-1.32224700
H	-2.80425300	-2.17003100	-1.58262500
C	-4.46906100	-0.84230000	-1.28881000
C	-5.31841200	-1.64662400	-2.05132100
C	-4.96416600	0.36312000	-0.77813600
C	-6.63047500	-1.25402600	-2.30814100
H	-4.94542900	-2.58437000	-2.45499900
C	-6.27372700	0.75785500	-1.03289800
H	-4.32249900	1.01043700	-0.18413800
C	-7.11150500	-0.05055000	-1.79992600
H	-7.27476000	-1.88887800	-2.90789300
H	-6.63721500	1.70001200	-0.63444500
H	-8.13240800	0.25724600	-2.00130200

TS2

0 1

C	-0.36632100	-1.31073500	0.15366100
C	-1.01893500	-3.15294800	1.13431600
C	-2.88415000	-1.55993400	0.58876800
H	-2.98608200	-0.63621000	1.16234300
N	0.25438600	-3.30810000	0.93085700
N	0.64459900	-2.16430700	0.30445000
N	-1.45330600	-1.94838600	0.64186200
C	-1.95871300	-4.05366400	1.87716700
H	-1.44419100	-4.48045100	2.73883500
H	-2.30757600	-4.86742600	1.22718100
C	-3.71088900	-2.67962700	1.25497400
O	-3.03706800	-3.27400700	2.34134800
C	2.02747700	-2.03228800	-0.08534400
C	2.36088600	-2.23215500	-1.42643700
C	2.97129400	-1.80034300	0.91846100
C	3.70502800	-2.08852900	-1.76893500
C	4.29771100	-1.66362300	0.51932300
C	4.67833200	-1.77919500	-0.81828700
H	3.99625300	-2.22516100	-2.80749900
H	5.04983000	-1.44359500	1.27285600

H	-4.63928600	-2.24997300	1.63161000
C	-0.34252800	0.08659300	-0.50028400
O	0.01080900	0.12997500	-1.66872900
C	-1.25073600	1.13499500	0.11512700
C	-0.87876600	2.54891900	-0.33926500
H	-1.24284500	1.05709700	1.20503500
H	-2.26668000	0.93318500	-0.23971300
H	0.05605200	2.84269900	0.14372200
H	-0.70460400	2.52694000	-1.41954900
C	-1.99182200	3.51337100	-0.00764600
C	-1.91073600	4.37206400	1.08961800
C	-3.16249800	3.51781700	-0.77519900
C	-2.97162700	5.21687700	1.41336000
H	-1.00759500	4.37908600	1.69432000
C	-4.22694100	4.35403900	-0.45239400
H	-3.23685200	2.85682200	-1.63707400
C	-4.13324700	5.20857600	0.64557000
H	-2.89023900	5.88147000	2.26787400
H	-5.12576900	4.34419400	-1.06147900
H	-4.95972800	5.86491200	0.89827000
C	6.10619100	-1.54019600	-1.23078900
H	6.79844700	-1.78102500	-0.42056400
H	6.37302100	-2.13656900	-2.10656900
H	6.25382100	-0.48447300	-1.48886300
C	2.56187200	-1.67644500	2.36003500
H	2.19580200	-2.63298000	2.74545100
H	3.41022900	-1.35756500	2.96775900
H	1.76133000	-0.93638300	2.45663800
C	1.32961900	-2.63116000	-2.44795300
H	0.76024900	-3.50114300	-2.10419900
H	0.62767800	-1.81383200	-2.62780000
H	1.81406700	-2.89350300	-3.38981900
O	1.10885800	0.66416000	0.83901200
C	2.29449900	1.07751500	0.54315200
C	3.18332700	1.45911100	1.59762900
C	2.79806300	1.15424500	-0.79195500
C	4.49285600	1.79715900	1.35137900
H	2.79811700	1.43765100	2.61201200

C	4.11075100	1.48784300	-1.03349800
H	2.14293800	0.89510600	-1.61325300
C	4.96699200	1.78966200	0.03308300
H	5.16928500	2.05538000	2.15781500
H	4.50216000	1.50934700	-2.04459000
N	6.34142400	2.09221800	-0.22470900
O	6.76016000	2.00979600	-1.38002900
O	7.06351700	2.41744500	0.71467500
H	-3.95924900	-3.44587100	0.50516200
C	-3.34738800	-1.37681700	-0.86480100
H	-2.69101300	-0.68135300	-1.39660200
H	-3.25573800	-2.34164800	-1.37466000
C	-4.78212400	-0.89603000	-0.91149900
C	-5.79187900	-1.71835100	-1.41447800
C	-5.12318600	0.37370300	-0.43225900
C	-7.11475300	-1.28087200	-1.44587900
H	-5.53989400	-2.70608500	-1.79170900
C	-6.44255800	0.81352600	-0.46524100
H	-4.35881800	1.03769700	-0.03463700
C	-7.44312700	-0.01415500	-0.97170700
H	-7.88677500	-1.93190400	-1.84292600
H	-6.68562300	1.80503500	-0.09616500
H	-8.47250300	0.32790800	-0.99728800

M2

0 1

C	-0.50450200	-1.30985800	0.04779000
C	-1.15889100	-3.01733500	1.23784100
C	-3.00687400	-1.43465300	0.60308400
H	-3.06369500	-0.46554500	1.10469300
N	0.10422100	-3.22293000	1.00679900
N	0.49700600	-2.16307300	0.25266500
N	-1.58513500	-1.85989100	0.64135700
C	-2.09139200	-3.82160800	2.09260100
H	-1.55823300	-4.16690300	2.97905700
H	-2.46811900	-4.69129100	1.53744400
C	-3.83522600	-2.47416300	1.38414100
O	-3.14412100	-2.98039400	2.50362400

C	1.88095300	-2.07496000	-0.14831500
C	2.21124700	-2.32316000	-1.48306400
C	2.83025000	-1.85149800	0.85439400
C	3.55801400	-2.21128400	-1.82718100
C	4.15922200	-1.75659700	0.45260300
C	4.53706600	-1.90050300	-0.88283500
H	3.84763700	-2.38583100	-2.86049600
H	4.91655600	-1.54477900	1.20308500
H	-4.74613700	-1.99435000	1.74246900
C	-0.46928900	0.01639000	-0.71443400
O	0.06643300	0.03064100	-1.79650500
C	-1.26933500	1.15918900	-0.14227500
C	-0.81979800	2.50509400	-0.71253900
H	-1.18635600	1.14920300	0.94692700
H	-2.32129000	1.00531100	-0.41023700
H	0.21680400	2.67365100	-0.40913800
H	-0.84894300	2.45565800	-1.80494300
C	-1.72536000	3.60100500	-0.20870600
C	-1.40673200	4.31771700	0.94679800
C	-2.93582300	3.87039500	-0.85467800
C	-2.27422000	5.28885100	1.44227500
H	-0.46963400	4.11038300	1.45772100
C	-3.80776400	4.83705100	-0.36036500
H	-3.19323400	3.31870900	-1.75661400
C	-3.47725200	5.55060000	0.79027700
H	-2.00970800	5.84249800	2.33769100
H	-4.74187200	5.03732700	-0.87607800
H	-4.15310200	6.30734200	1.17533500
C	5.97062600	-1.70473700	-1.29669600
H	6.65399100	-1.93647700	-0.47620300
H	6.22770800	-2.33228500	-2.15353100
H	6.14306600	-0.66039000	-1.58334300
C	2.43402500	-1.70790400	2.29847500
H	2.04568200	-2.65199600	2.69119300
H	3.29775600	-1.40997000	2.89457900
H	1.65927200	-0.94068300	2.39643100
C	1.18521300	-2.76368500	-2.49425400
H	0.53812800	-3.54249300	-2.07811600

H	0.55498600	-1.92963900	-2.80730300
H	1.68227500	-3.17394600	-3.37470300
O	1.14412800	0.79381000	0.93851800
C	2.34859500	1.10456800	0.66865900
C	3.30983300	1.35392200	1.71150700
C	2.83668500	1.20669500	-0.68175200
C	4.63576800	1.57686800	1.43572000
H	2.95515000	1.32555700	2.73731900
C	4.16346800	1.42489600	-0.95252400
H	2.12927200	1.05848500	-1.48950400
C	5.07798300	1.58820600	0.10207500
H	5.35824500	1.73278200	2.22876300
H	4.52754300	1.46444400	-1.97334500
N	6.45839300	1.77515300	-0.18127000
O	6.83889500	1.73501300	-1.35585900
O	7.24172800	1.96451600	0.75080800
H	-4.11635000	-3.29868100	0.71193600
C	-3.51428000	-1.35397700	-0.84496700
H	-2.88233800	-0.69089600	-1.44280400
H	-3.43281500	-2.35063300	-1.29136700
C	-4.95077200	-0.88011000	-0.88053100
C	-5.97811500	-1.75846800	-1.22953600
C	-5.27362500	0.43749800	-0.53806100
C	-7.30390500	-1.33010400	-1.24065800
H	-5.73795900	-2.78318400	-1.50133900
C	-6.59691800	0.86688100	-0.54854400
H	-4.49038000	1.14277500	-0.26683400
C	-7.61584900	-0.01720700	-0.89940500
H	-8.09157600	-2.02319900	-1.51754500
H	-6.83059800	1.89363900	-0.28580700
H	-8.64777700	0.31781900	-0.90845900

M02

1 1

C	0.51553000	-0.86061800	-0.10018700
C	1.62294000	-2.68821300	0.28324700
C	3.06576900	-0.65991900	-0.15543800
H	3.10898700	-0.36796800	-1.20742600

N	0.36362400	-3.03134700	0.33977900
N	-0.31133600	-1.88101300	0.10657600
N	1.76016600	-1.35202600	0.01868400
C	2.83343500	-3.56554900	0.39177100
H	2.61547600	-4.53705200	-0.05257400
H	3.10558200	-3.70475300	1.44690700
C	4.17026600	-1.67836600	0.17015700
O	3.87708200	-2.96342500	-0.33609600
C	-1.74724800	-1.88295700	-0.02915700
C	-2.51400100	-2.02724500	1.13064000
C	-2.28751100	-1.75359800	-1.31230700
C	-3.89772500	-2.00592500	0.97451600
C	-3.67891200	-1.73579300	-1.40713100
C	-4.49526700	-1.85398500	-0.28038900
H	-4.52481700	-2.10611100	1.85623800
H	-4.13367000	-1.63688400	-2.38905500
H	5.09779500	-1.34282900	-0.29414100
C	0.14399600	0.54029900	-0.54533700
O	0.84985400	1.03315500	-1.39380100
C	-1.03077400	1.22995800	0.09043000
C	-1.95671100	1.87109000	-0.95958500
H	-0.60532000	2.01628300	0.72827400
H	-1.58978400	0.55300800	0.74036200
H	-1.37297800	2.56168000	-1.57352600
H	-2.34453300	1.08517200	-1.61815500
C	-3.10492600	2.58168900	-0.28654000
C	-3.10049300	3.96683100	-0.11793800
C	-4.17688100	1.84072400	0.21804400
C	-4.15350600	4.60191200	0.53820100
H	-2.27087500	4.55177200	-0.50674300
C	-5.22902900	2.47144100	0.87467700
H	-4.18146600	0.76008900	0.08915900
C	-5.21917700	3.85624000	1.03549900
H	-4.14132400	5.68041500	0.65868900
H	-6.05774600	1.88367600	1.25750000
H	-6.03972500	4.35160400	1.54415700
C	-5.99283600	-1.77714500	-0.41049700
H	-6.32880000	-2.18800800	-1.36460700

H	-6.48918500	-2.31597300	0.39863200
H	-6.32100300	-0.73266800	-0.36682000
C	-1.87033800	-2.19475200	2.48110900
H	-1.34520200	-3.15194800	2.54522300
H	-1.13829500	-1.40494900	2.67941300
H	-2.62500600	-2.16289100	3.26732200
C	-1.42053900	-1.66629500	-2.54354000
H	-0.59435000	-2.38199600	-2.50212300
H	-2.01373400	-1.88662100	-3.43151500
H	-0.99186200	-0.66739000	-2.68101200
H	4.31912800	-1.72565900	1.25925600
C	3.18654500	0.56978000	0.75393300
H	2.33164700	1.23432600	0.61077700
H	3.18322700	0.24300000	1.79871400
C	4.45569600	1.32434300	0.42713400
C	5.57178300	1.26838800	1.26261700
C	4.52258300	2.07091600	-0.75325800
C	6.73916200	1.95073900	0.92435000
H	5.52614000	0.69545600	2.18523800
C	5.68774500	2.75027300	-1.09297900
H	3.65042500	2.12501000	-1.40172700
C	6.79936700	2.69022200	-0.25375800
H	7.60019400	1.90530000	1.58308000
H	5.72707400	3.33043600	-2.00904500
H	7.70765400	3.22242600	-0.51625700

TS3

0 1

C	-1.57523700	-1.27299800	-0.07406000
C	-2.56624400	-2.49102100	1.43375200
C	-3.85254100	-0.43111700	0.74189400
H	-3.47854200	0.44918100	1.27482600
N	-1.42610700	-3.07901400	1.21839600
N	-0.82271800	-2.30679300	0.27246500
N	-2.70788400	-1.37463100	0.64336400
C	-3.63456800	-2.87248800	2.41353500
H	-3.17331700	-3.25972300	3.32221800
H	-4.27849200	-3.64915500	1.97916000

C	-4.92702000	-1.12259500	1.58943300
O	-4.37650400	-1.72186500	2.74408200
C	0.51358000	-2.60212400	-0.18143200
C	0.67730000	-3.20205100	-1.42958300
C	1.56895000	-2.27830900	0.67514400
C	1.98683100	-3.46972700	-1.82676700
C	2.85309700	-2.57997900	0.23473700
C	3.07868000	-3.16969100	-1.01125300
H	2.15490400	-3.92956400	-2.79706000
H	3.70003000	-2.31596300	0.86386400
H	-5.64751600	-0.37437700	1.91921700
C	-1.21430900	-0.18975800	-1.09595100
O	-1.59032800	-0.37899000	-2.24474600
C	-0.41440800	0.88420600	-0.57970600
C	-0.45097300	2.18209600	-1.38869300
H	0.76442700	0.45501700	-0.84390100
H	-0.50862900	1.01588600	0.50218200
H	-0.26200400	1.93852800	-2.43785800
H	-1.44956100	2.63578100	-1.33633000
C	0.58760700	3.15621700	-0.88676900
C	1.80110900	3.32327100	-1.55688600
C	0.37471400	3.86216700	0.30104300
C	2.78322600	4.17016600	-1.04935300
H	1.98402400	2.75979000	-2.46720200
C	1.35401600	4.70973800	0.81280300
H	-0.56966600	3.74419200	0.82852200
C	2.56366300	4.86461900	0.13779700
H	3.72500700	4.28078100	-1.57792600
H	1.17285600	5.25090300	1.73637700
H	3.32995800	5.52197700	0.53594100
C	-0.50476200	-3.55682200	-2.29266900
H	-1.21420100	-4.18361700	-1.74290700
H	-1.03664300	-2.65935500	-2.62251500
H	-0.17669100	-4.10678900	-3.17556200
C	4.48102600	-3.49228900	-1.45437800
H	5.19005900	-2.76584100	-1.05042900
H	4.77815600	-4.48494100	-1.10074500
H	4.56135800	-3.48891200	-2.54373500

C	1.32080100	-1.62183700	2.00608600
H	0.71211300	-0.71803300	1.89041500
H	0.78915600	-2.29426000	2.68611700
H	2.26557400	-1.32948000	2.46571200
H	-5.45219000	-1.87401000	0.97976800
C	-4.40085900	-0.03454900	-0.63468300
H	-3.62150700	0.45182200	-1.22412900
H	-4.69806800	-0.94161300	-1.17155600
C	-5.57491900	0.90585600	-0.47791600
C	-6.88537000	0.46263800	-0.66655700
C	-5.35515300	2.23649100	-0.10941000
C	-7.95875100	1.33301100	-0.48838300
H	-7.06629700	-0.56775000	-0.96125300
C	-6.42579800	3.10682000	0.07136000
H	-4.33679900	2.59433800	0.02609900
C	-7.73110700	2.65551200	-0.11696500
H	-8.97211200	0.97704300	-0.64261100
H	-6.24137900	4.13828700	0.35311400
H	-8.56647200	3.33396500	0.02078000
O	1.95715900	0.16818500	-1.44400600
C	3.01887400	0.38128800	-0.74873800
C	4.30124100	0.02435100	-1.26947700
C	2.99061600	0.96843800	0.55461000
C	5.45171400	0.19658500	-0.53580300
H	4.33435600	-0.41264000	-2.26249000
C	4.14178000	1.14031000	1.28865500
H	2.03660500	1.29901200	0.95457400
C	5.37422500	0.74578200	0.75180800
H	6.41961900	-0.08822200	-0.93200700
H	4.11472600	1.58483900	2.27666100
N	6.56911700	0.91911700	1.52076300
O	6.48139000	1.39566000	2.65078900
O	7.64379900	0.58312200	1.02718300

TS3E

0 1

C	-1.48486800	-1.08748100	0.12588400
C	-2.53923600	-1.97495200	1.81348300

C	-3.83288000	-0.17316900	0.61530600
H	-3.59214200	0.79170700	1.07485100
N	-1.38831100	-2.58023100	1.77604500
N	-0.74678700	-2.01747500	0.71445100
N	-2.64406100	-1.04399500	0.80687600
C	-3.67339100	-2.20845400	2.76721300
H	-3.28701600	-2.30286000	3.78267500
H	-4.18837800	-3.13982900	2.49409100
C	-4.96987500	-0.82135600	1.40851800
O	-4.55230500	-1.10970500	2.72831700
C	0.59642300	-2.41532200	0.37089700
C	0.75913700	-3.49188300	-0.50226000
C	1.65366800	-1.71732400	0.95238600
C	2.06717200	-3.86439300	-0.80009700
C	2.94197800	-2.14026100	0.63367400
C	3.16474400	-3.20069300	-0.24416300
H	2.23402200	-4.69334200	-1.48345000
H	3.79011600	-1.60728900	1.05731800
H	-5.80067900	-0.11931200	1.47526200
C	-1.11050800	-0.37164900	-1.18294600
O	-1.21170300	-1.08313000	-2.17248500
C	-0.57498000	0.95702200	-1.08203100
C	-0.94564500	1.83125200	0.11428000
H	-0.65806300	1.46085800	-2.04810000
H	0.70336600	0.61958500	-1.15455700
H	-1.90071000	2.34564200	-0.06770900
H	-1.09563800	1.21610000	1.01188300
C	0.11400000	2.86399700	0.43618300
C	0.64294000	3.67971400	-0.56954100
C	0.61114000	2.99702700	1.73462600
C	1.65923600	4.58700600	-0.28780700
H	0.26883200	3.59191900	-1.58587500
C	1.63226600	3.90236400	2.02071700
H	0.20798300	2.37096700	2.52741800
C	2.16363300	4.69578100	1.00769400
H	2.06653300	5.20381100	-1.08257600
H	2.01582800	3.98125500	3.03306500
H	2.96501200	5.39476000	1.22410800

C	-0.43399300	-4.19739300	-1.08726700
H	-1.06775300	-4.61501000	-0.29841300
H	-1.03790900	-3.49532800	-1.67067300
H	-0.11630800	-5.01183100	-1.73953600
C	4.56586500	-3.60805000	-0.61469500
H	5.30113400	-3.13185700	0.03716300
H	4.69111300	-4.69239000	-0.55219200
H	4.78928100	-3.30741800	-1.64357100
C	1.40803300	-0.53009000	1.83996300
H	1.06648100	0.31905700	1.23615800
H	0.64777300	-0.73657100	2.60004300
H	2.32859000	-0.22255000	2.33852400
H	-5.31516000	-1.73380100	0.89796800
C	-4.16951600	0.00156800	-0.87135700
H	-3.38119800	0.58505100	-1.35691300
H	-4.19134400	-0.98364500	-1.34881700
C	-5.49691200	0.70461700	-1.04640500
C	-6.64080500	-0.01452200	-1.39962000
C	-5.60373300	2.07991000	-0.82079400
C	-7.87077800	0.62748300	-1.52315400
H	-6.56624800	-1.08294400	-1.58473400
C	-6.83247000	2.72262700	-0.94095100
H	-4.71902900	2.65530600	-0.55880200
C	-7.96948400	1.99683700	-1.29133700
H	-8.75033100	0.05696100	-1.80304000
H	-6.90087900	3.79145000	-0.76653700
H	-8.92692200	2.49792700	-1.38894300
O	1.82285200	0.08528900	-1.60765300
C	3.02040200	0.36490000	-1.21726500
C	4.12567600	-0.31860700	-1.81097300
C	3.31770300	1.31759900	-0.19876000
C	5.41702000	-0.10693700	-1.38858400
H	3.90558300	-1.03603800	-2.59514300
C	4.61204500	1.53177600	0.22283300
H	2.50474400	1.87693000	0.25053600
C	5.66208600	0.81438600	-0.36084100
H	6.24929600	-0.64096900	-1.83251800
H	4.82907400	2.25257300	1.00293900

N	7.00646900	1.03675100	0.08549100
O	7.20359500	1.85488400	0.98088200
O	7.91546500	0.39963800	-0.44141000

TS3^{DBU}

1 1

C	1.17236700	-1.93441600	-0.16368900
C	2.45839300	-3.08841100	1.16337700
C	3.57086800	-1.12143000	0.03136300
H	3.82041300	-1.26878500	-1.02158000
N	1.28603300	-3.65592500	1.25034200
N	0.49774300	-2.92045100	0.42344500
N	2.42504400	-2.01650700	0.31435300
C	3.75260400	-3.49471000	1.80390700
H	3.83407500	-4.58207600	1.80297300
H	3.78970600	-3.13324000	2.84057400
C	4.72340600	-1.56143900	0.94505000
O	4.81321700	-2.96906200	1.03959300
C	-0.90964600	-3.18707300	0.28369500
C	-1.73865700	-2.83208100	1.35337300
C	-1.38141300	-3.66609400	-0.93895900
C	-3.11223200	-2.93350000	1.14596200
C	-2.76551500	-3.75396700	-1.08916500
C	-3.64135600	-3.37669900	-0.06986500
H	-3.78603700	-2.64866500	1.95004100
H	-3.16674000	-4.11313100	-2.03338700
H	5.66298300	-1.19588400	0.52946200
C	0.73719200	-1.01992200	-1.30998700
O	1.59685500	-0.86316400	-2.17559900
C	-0.53357800	-0.38180500	-1.20225100
C	-1.10972800	0.19312100	-2.49252000
H	-0.23843600	0.66623700	-0.41227300
H	-1.26470300	-0.92286500	-0.60040800
H	-0.41188000	0.91876500	-2.92225500
H	-1.22284600	-0.60711700	-3.23705000
C	-2.45049100	0.84170100	-2.24517000
C	-2.59842400	2.22921500	-2.26990500
C	-3.57073400	0.05380900	-1.95708600

C	-3.83252300	2.82252300	-2.00464900
H	-1.73998000	2.84769000	-2.52056200
C	-4.80695000	0.63972100	-1.69961400
H	-3.46738900	-1.03016400	-1.93446600
C	-4.94078400	2.02809500	-1.71806600
H	-3.92895200	3.90378700	-2.03092900
H	-5.66684800	0.01322800	-1.48214600
H	-5.90367700	2.48671400	-1.51699700
C	-5.13177000	-3.45881500	-0.26676400
H	-5.52078300	-4.40234300	0.12875600
H	-5.64020000	-2.64589900	0.25721900
H	-5.39276800	-3.40779700	-1.32603000
C	-1.17503100	-2.35506300	2.66613800
H	-0.76361300	-3.19377800	3.23533300
H	-0.36569000	-1.63134100	2.52480500
H	-1.95637900	-1.88158800	3.26351300
C	-0.44886000	-4.03070900	-2.06428800
H	0.45292700	-4.52546300	-1.69431000
H	-0.94835900	-4.70394300	-2.76226300
H	-0.13542100	-3.14451200	-2.62814500
H	4.58838700	-1.12485000	1.94639500
C	3.18594300	0.34154000	0.29323900
H	2.22616400	0.55871300	-0.18080400
H	3.05589900	0.48864200	1.37141700
C	4.23200700	1.27710000	-0.26290500
C	5.17962300	1.88445300	0.56151300
C	4.26650500	1.51877800	-1.64024100
C	6.15253400	2.72179000	0.01835400
H	5.15362700	1.70471400	1.63335500
C	5.23664700	2.35554500	-2.18268900
H	3.52356000	1.04434500	-2.27928600
C	6.18228500	2.95781300	-1.35336900
H	6.88438100	3.19045500	0.66827300
H	5.25422400	2.54074300	-3.25179600
H	6.93778500	3.61202000	-1.77603400
C	-3.99674600	2.19802700	2.29922600
C	-3.21729900	1.22169300	1.41934400
C	-1.73556400	1.07264200	1.79816100

C	-3.46291700	3.63112900	2.28875500
C	-0.87798800	2.17236700	1.22289900
C	-2.00607500	3.74044900	2.74869100
H	-3.98690900	1.82985200	3.33424900
H	-3.68368800	0.23192800	1.48245600
H	-1.61382900	1.03202300	2.88747300
H	-3.54380100	4.06113000	1.28177800
H	-1.78313200	4.76079600	3.06658500
C	0.76636300	2.87094400	-0.37028900
H	0.97527200	2.54164400	-1.39504300
C	-0.34471500	4.55580400	1.06967400
C	0.05636700	4.21833500	-0.35677400
H	-0.84466800	4.16219900	-0.97489600
H	0.69918100	5.00469300	-0.75758000
H	-1.02683200	5.41023200	1.07876800
N	-1.03983500	3.42324000	1.69238600
N	-0.04448400	1.84523000	0.27403900
H	-1.82925200	3.09916700	3.61892200
H	-4.08109400	4.24553700	2.95124700
H	-5.04377200	2.20362300	1.97833500
H	-3.27844900	1.52661700	0.36937700
H	-1.34758400	0.13339300	1.40230500
H	1.73627800	2.95010500	0.14112400
H	0.53213400	4.82039100	1.67273400

TS3E^{DBU}

1 1

C	-1.42201000	-1.14681100	0.25181900
C	-2.61445600	-2.09506500	1.80950600
C	-3.81693300	-0.27415900	0.54518100
H	-3.63182200	0.68000100	1.04959300
N	-1.45659900	-2.68648300	1.86218100
N	-0.72896900	-2.08542500	0.87966500
N	-2.63836400	-1.13603100	0.82323400
C	-3.83207500	-2.37310300	2.64085500
H	-3.54492300	-2.49977800	3.68534200
H	-4.30376900	-3.29983400	2.28618200
C	-5.01443700	-0.95634300	1.21057900

O	-4.71746900	-1.28283800	2.55355600
C	0.63828400	-2.47117300	0.63771600
C	0.86996200	-3.57265300	-0.19039700
C	1.64729800	-1.75803300	1.28573600
C	2.19651500	-3.95999100	-0.36692400
C	2.95656600	-2.18840500	1.07622300
C	3.24689700	-3.28675600	0.26503400
H	2.41488400	-4.81255100	-1.00456600
H	3.76873800	-1.66086200	1.57161200
H	-5.85534100	-0.26339500	1.22028100
C	-0.93060100	-0.41575000	-1.00688700
O	-0.93920800	-1.13101300	-2.00288400
C	-0.43501000	0.92164700	-0.85770000
C	-0.96345000	1.76865900	0.29948800
H	-0.45750600	1.43002700	-1.82631200
H	0.92300500	0.74080000	-0.80300300
H	-1.93113900	2.21899000	0.03044600
H	-1.15379600	1.14164000	1.18084700
C	-0.02248500	2.87415500	0.72312200
C	0.53048400	3.74591600	-0.22183700
C	0.31499300	3.04095200	2.06753900
C	1.39830100	4.75943300	0.17188900
H	0.28092700	3.63024500	-1.27314300
C	1.18864100	4.05214200	2.46540800
H	-0.10597100	2.36810500	2.81104600
C	1.73013000	4.91589800	1.51792500
H	1.81999800	5.42820400	-0.57199200
H	1.44283200	4.16430200	3.51459200
H	2.40953000	5.70536400	1.82340800
C	-0.27263000	-4.28601600	-0.85994700
H	-0.96436700	-4.69743400	-0.11858000
H	-0.83142700	-3.58980900	-1.49349700
H	0.09579100	-5.10504600	-1.47844900
C	4.66645100	-3.76786600	0.11416800
H	5.37799400	-2.95082500	0.25932300
H	4.88730100	-4.53535800	0.86284900
H	4.83503800	-4.20989000	-0.87075500
C	1.33260200	-0.57293100	2.15742900

H	1.07003600	0.29915900	1.54606500
H	0.49289300	-0.77931300	2.82821500
H	2.19669100	-0.30215000	2.76671500
H	-5.30127400	-1.85420200	0.64134300
C	-4.02016100	-0.06141100	-0.95998700
H	-3.18486100	0.51974100	-1.36350100
H	-4.01822800	-1.03501700	-1.46161500
C	-5.31569500	0.67251400	-1.22363100
C	-6.44197400	-0.01159600	-1.68491300
C	-5.41159000	2.04317100	-0.96759700
C	-7.64491100	0.66144200	-1.88534800
H	-6.37550400	-1.07652800	-1.89186700
C	-6.61327000	2.71683400	-1.16475600
H	-4.53786800	2.58948100	-0.61936400
C	-7.73331000	2.02619300	-1.62353700
H	-8.51200600	0.11870700	-2.24748000
H	-6.67368900	3.78164300	-0.96499200
H	-8.67003100	2.55068800	-1.78029100
C	5.85688900	2.61627400	0.32273400
C	4.36131200	2.84401500	0.10098300
C	3.45342500	1.66803300	0.51267300
C	6.45801400	1.45591200	-0.46726300
C	3.35742600	0.61283500	-0.56116100
C	5.75410700	0.12820400	-0.18882400
H	6.02432900	2.43141600	1.39247000
H	4.05345600	3.71346600	0.68757300
H	3.81243200	1.22514000	1.45025000
H	6.41329400	1.65554000	-1.54528100
H	6.38673200	-0.70875800	-0.49552300
C	2.12150000	-0.55227500	-2.23554000
H	1.25387800	-0.28685800	-2.84407200
C	4.55880400	-0.86166300	-2.12373400
C	3.40325400	-0.54107000	-3.05200100
H	3.54383500	0.44966400	-3.49643500
H	3.36106900	-1.27464700	-3.85980800
H	5.51886800	-0.66454400	-2.61124400
N	4.49275800	-0.03119100	-0.91687000
N	2.20716300	0.41064500	-1.13861400

H	5.58133300	0.00785000	0.88718400
H	7.51571700	1.35153700	-0.20423000
H	6.39601000	3.53739000	0.07913000
H	4.16163800	3.09255400	-0.94930200
H	2.44253400	2.03737400	0.69229100
H	1.93676400	-1.55771300	-1.82927200
H	4.53633000	-1.92124500	-1.83887800

M3

0 1

C	-0.48580100	-0.87812600	-0.05521400
C	-1.48429200	-2.82432000	-0.06625400
C	-3.00741900	-0.82131500	0.13692100
H	-2.98567000	-0.37746700	1.13474500
N	-0.21474600	-3.10462400	-0.13227800
N	0.39318800	-1.87834500	-0.12888200
N	-1.69365700	-1.47214200	-0.03143300
C	-2.65089600	-3.76085500	0.03533100
H	-2.36790900	-4.63747100	0.61850700
H	-2.96693800	-4.08373600	-0.96628800
C	-4.07360100	-1.91688800	0.02484700
O	-3.69933900	-3.09877500	0.70836500
C	1.82335700	-1.76697300	-0.03478400
C	2.58551400	-1.92432600	-1.19229900
C	2.37115700	-1.49102000	1.21939400
C	3.96487200	-1.76819000	-1.07117100
C	3.75646300	-1.35132200	1.28847900
C	4.56299600	-1.46819100	0.15484600
H	4.58541500	-1.87251800	-1.95748300
H	4.21375600	-1.13018000	2.25011100
H	-5.00097700	-1.55648800	0.47226100
C	-0.24797300	0.59254500	0.18205200
O	-0.93546600	1.04851100	1.15958200
C	0.63025600	1.18419400	-0.67479500
C	1.03815800	2.62051100	-0.53028900
H	1.12589300	0.57831800	-1.42819600
H	0.76851000	3.21227500	-1.41488400
H	0.47705700	3.03841900	0.31415400

C	2.52748400	2.77697700	-0.28235700
C	3.29947600	3.68946800	-1.00379300
C	3.16051900	1.98471600	0.68023400
C	4.66924900	3.81093700	-0.77058700
H	2.82312300	4.30785000	-1.76068200
C	4.52576700	2.10223200	0.91908200
H	2.56761400	1.25793900	1.22956400
C	5.28801400	3.01703100	0.19165800
H	5.25326700	4.52315900	-1.34560600
H	4.99763900	1.47382300	1.67001800
H	6.35458200	3.10736200	0.37268200
C	6.04725000	-1.23032000	0.24215600
H	6.59382100	-1.86134300	-0.46231300
H	6.27201600	-0.18601200	-0.00174400
H	6.42218200	-1.42501700	1.24938400
C	1.92895900	-2.23530700	-2.51067400
H	1.41049500	-3.19776500	-2.47199000
H	1.18356200	-1.47537600	-2.76852400
H	2.67018100	-2.27212200	-3.30985900
C	1.49290900	-1.32561600	2.43229000
H	0.84906600	-2.20031500	2.57081000
H	2.10236700	-1.20346600	3.32883400
H	0.83873100	-0.45041400	2.33716300
H	-4.26070300	-2.14468900	-1.03576800
C	-3.23340600	0.28212200	-0.90531600
H	-2.31281200	0.85794100	-1.02455800
H	-3.46924500	-0.17615600	-1.87213600
C	-4.34379200	1.20225600	-0.45654600
C	-5.63082800	1.11669500	-0.98824700
C	-4.07464900	2.14561700	0.54147900
C	-6.64002300	1.96440300	-0.53283200
H	-5.84338600	0.38794700	-1.76679200
C	-5.08224100	2.99065400	0.99577900
H	-3.06409800	2.19681200	0.94504600
C	-6.36712200	2.90198100	0.45948200
H	-7.63721300	1.89265700	-0.95532600
H	-4.86595200	3.72313000	1.76715300
H	-7.15157800	3.56338200	0.81305700

M3E

0 1

C	-0.18857200	-0.81937000	-0.08659700
C	-0.72304000	-1.94491700	1.71402500
C	-2.59718500	-0.61671400	0.66556200
H	-2.51003100	0.46078000	0.84082500
N	0.54766800	-2.16646800	1.54700700
N	0.86056800	-1.46770800	0.41425700
N	-1.22011600	-1.14813700	0.71545300
C	-1.61005400	-2.36036000	2.84870000
H	-1.03165000	-2.39240800	3.77213000
H	-2.03818800	-3.35363400	2.65792200
C	-3.38478500	-1.28041300	1.80435700
O	-2.62597100	-1.39046300	2.99258100
C	2.21984000	-1.42037100	-0.05630100
C	2.55815800	-2.17658700	-1.18532900
C	3.13686100	-0.64264300	0.65157600
C	3.87280200	-2.08818100	-1.63444400
C	4.44189700	-0.59277000	0.15833200
C	4.82314000	-1.29731900	-0.98136500
H	4.16383700	-2.65420500	-2.51572100
H	5.16909300	0.02901100	0.67479600
H	-4.26339600	-0.67304600	2.02629900
C	-0.28317900	0.02197400	-1.35880600
O	-0.51171800	-0.68411500	-2.39421200
C	-0.21584200	1.37229700	-1.18538200
C	0.06472800	2.08171700	0.10672800
H	-0.30998200	1.97529500	-2.08581500
H	-0.65361100	2.89918700	0.25706700
H	-0.10425000	1.40462700	0.95959800
C	1.46638100	2.66103900	0.26198200
C	2.51681600	2.28471400	-0.57339300
C	1.73027400	3.55392900	1.30748800
C	3.80571900	2.77644900	-0.36288100
H	2.32273400	1.58298200	-1.37963900
C	3.01339000	4.04378100	1.52441200
H	0.91732900	3.85785200	1.96359100

C	4.06065200	3.65274400	0.68759900
H	4.61272000	2.46230500	-1.01874200
H	3.19952300	4.73137600	2.34388400
H	5.06342500	4.03394600	0.85313900
C	1.54612000	-3.06024500	-1.86315900
H	1.12355600	-3.77471800	-1.14855200
H	0.72504700	-2.45926300	-2.26883600
H	2.01715900	-3.62325200	-2.67050200
C	6.24059300	-1.23575700	-1.48802100
H	6.75044400	-0.34009700	-1.12682300
H	6.81052900	-2.10554500	-1.14596000
H	6.26783800	-1.23420400	-2.58014800
C	2.76746200	0.10312900	1.90568500
H	1.71332700	0.38778100	1.91968400
H	2.95451700	-0.52008400	2.78614000
H	3.36380300	1.01539200	1.98883000
H	-3.71932700	-2.27835900	1.48328400
C	-3.27261600	-0.87137600	-0.69092300
H	-2.55134600	-0.72099100	-1.49924800
H	-3.59230600	-1.91836300	-0.73509100
C	-4.45353800	0.05517300	-0.85840300
C	-5.76358800	-0.38113700	-0.65603700
C	-4.22754600	1.39424400	-1.19467900
C	-6.83284100	0.50358900	-0.78895100
H	-5.94802200	-1.42152200	-0.39970100
C	-5.29280700	2.27916800	-1.32612400
H	-3.20391900	1.72962000	-1.35653000
C	-6.59923400	1.83472500	-1.12325200
H	-7.84740000	0.15107400	-0.63309900
H	-5.10581500	3.31462600	-1.59221000
H	-7.43100600	2.52359300	-1.22893900

TS4

0 1

C	0.71247700	-0.04197600	1.02696000
C	2.23338800	-1.24598700	2.06663100
C	3.16061100	0.67275500	0.73858400
H	3.19945200	0.58562000	-0.35000100

N	1.10170800	-1.72195300	2.48187300
N	0.15710300	-0.96835600	1.83530100
N	2.05720300	-0.19233500	1.20882500
C	3.60970400	-1.73597000	2.40517100
H	3.62454000	-2.82634000	2.34667100
H	3.88002200	-1.42407500	3.42440500
C	4.46671000	0.16218500	1.37231200
O	4.52804900	-1.24223800	1.46209900
C	-1.22251200	-1.19524800	2.16101400
C	-1.80859600	-0.37109300	3.13090600
C	-1.88869400	-2.27436500	1.57860800
C	-3.13645900	-0.61722100	3.46898800
C	-3.20757800	-2.50195200	1.97998200
C	-3.85061100	-1.67804500	2.90229100
H	-3.61689100	0.01626500	4.21091900
H	-3.74161300	-3.34978500	1.55625200
H	5.30266900	0.48238100	0.74817400
C	0.10522800	0.88759800	0.09991000
O	0.90536000	1.35792700	-0.89292700
C	-1.14872900	1.39614800	0.28647200
C	-1.75157900	2.37196600	-0.57482800
H	-1.73173600	1.00097700	1.11008500
H	-1.02827000	2.97528500	-1.12800000
H	-2.18290500	1.61975600	-1.64725600
C	-2.95443500	3.11559800	-0.14309000
C	-3.37176200	4.22572300	-0.89944100
C	-3.77006800	2.73255500	0.93657500
C	-4.53900200	4.91711200	-0.59832400
H	-2.76109800	4.54084100	-1.74221800
C	-4.94149800	3.42401000	1.23661600
H	-3.50002600	1.87896000	1.55485300
C	-5.33737700	4.52070100	0.47426100
H	-4.82711900	5.77134100	-1.20412400
H	-5.54759700	3.10211000	2.07900600
H	-6.24934600	5.05801600	0.71239000
C	-1.01794900	0.73295800	3.78287200
H	-0.09901400	0.33871400	4.22801100
H	-0.72696000	1.49986400	3.05592300

H	-1.60458900	1.21362600	4.56679900
C	-5.28816800	-1.91318900	3.28542900
H	-5.62459500	-2.90542600	2.97661900
H	-5.42809800	-1.82059000	4.36546500
H	-5.93843300	-1.17255300	2.80685600
C	-1.21614800	-3.14814600	0.55902100
H	-1.11503300	-2.61799100	-0.39441100
H	-0.20081100	-3.41731800	0.86043900
H	-1.79694100	-4.05814400	0.39330500
H	4.58636300	0.60659200	2.37363000
C	2.93774200	2.13710000	1.14306600
H	1.93693800	2.44786800	0.84109900
H	3.01185100	2.22137300	2.23314000
C	3.96486200	3.01470900	0.46705100
C	5.00995700	3.60360500	1.17973400
C	3.88553700	3.21649400	-0.91552900
C	5.95982100	4.38687400	0.52569400
H	5.07787000	3.45227100	2.25421700
C	4.83403300	3.99584800	-1.56961700
H	3.06594800	2.75885200	-1.46583500
C	5.87412700	4.58357200	-0.84965500
H	6.76564300	4.84357900	1.09172600
H	4.76051900	4.14908100	-2.64171300
H	6.61319400	5.19303900	-1.35972600
C	-5.42489900	-1.68749700	-0.49670900
C	-4.72743400	-0.33288300	-0.37325700
C	-4.58416300	0.41325500	-1.71081900
C	-4.69752800	-2.70440600	-1.37562200
C	-3.38453600	-0.04359600	-2.50171800
C	-4.48601600	-2.22217400	-2.81368800
H	-6.42999200	-1.52474100	-0.90970800
H	-5.30214300	0.30409300	0.30776500
H	-5.49435400	0.28960300	-2.30999300
H	-3.71667400	-2.94733400	-0.94445100
H	-4.28158800	-3.06887100	-3.47297400
C	-1.19673700	0.39856200	-3.34846900
H	-0.38044200	1.03077200	-2.98598900
C	-2.10173900	-1.90420600	-3.45885300

C	-0.90217600	-1.06448600	-3.05795300
H	-0.69825600	-1.17082600	-1.98802700
H	-0.00314400	-1.40032600	-3.57750700
H	-2.02257100	-2.91060400	-3.03246900
N	-3.34980100	-1.31099500	-2.95751300
N	-2.41444000	0.81407100	-2.66195700
H	-5.39484300	-1.74240500	-3.19306200
H	-5.27656100	-3.63371900	-1.40138200
H	-5.56631700	-2.11310200	0.50214900
H	-3.73290500	-0.45444500	0.08064300
H	-4.45831000	1.48370200	-1.53633100
H	-1.31300800	0.56751400	-4.42667100
H	-2.16854500	-2.00876700	-4.54907400
C	3.57120400	-1.95606400	-1.40996600
C	3.33205600	-3.11073200	-0.64960700
C	4.41601200	-3.91298000	-0.23347000
C	5.67967800	-3.49488400	-0.58315100
C	5.89922900	-2.30749800	-1.33805700
C	4.85684000	-1.51919400	-1.77084600
H	4.24934500	-4.81887400	0.34086600
H	6.54067100	-4.08192800	-0.27988300
H	6.91887700	-2.02604200	-1.58133200
H	5.00187900	-0.61409400	-2.35075600
N	1.98898100	-3.21710400	-0.43286100
N	1.41032600	-2.20021800	-1.03108300
N	2.33149600	-1.44583200	-1.61611900
O	2.03989800	-0.33567700	-2.28032100
H	1.34352600	0.58192300	-1.45745400

TS4Z

0 1

C	1.21049500	-0.93106400	-0.63226400
C	0.76499400	-2.57342500	-2.03495600
C	-1.26388800	-1.42169000	-1.14903800
H	-1.47143400	-1.53402800	-0.08869200
N	2.05497300	-2.57403700	-1.94099800
N	2.33049100	-1.55102700	-1.07164500
N	0.19628800	-1.57340600	-1.29100800

C	-0.08830500	-3.55645200	-2.77016600
H	0.43708400	-4.50958900	-2.83306200
H	-0.30643300	-3.19632200	-3.78648600
C	-1.99112700	-2.53656400	-1.93831100
O	-1.27489100	-3.74561700	-2.03720000
C	3.70886500	-1.33060300	-0.73840100
C	4.48763300	-0.59040200	-1.63327400
C	4.21659300	-1.88994900	0.43388900
C	5.82848600	-0.39914300	-1.31384000
C	5.56697000	-1.66948200	0.71113700
C	6.38333100	-0.93250300	-0.14621400
H	6.45443900	0.18154500	-1.98715500
H	5.98796400	-2.08705300	1.62212700
H	-2.92513400	-2.75958500	-1.41666500
C	1.07970600	0.14898800	0.32279300
O	-0.17599400	0.41663700	0.77870700
C	2.15736200	0.94311700	0.63212300
C	2.18083700	2.14188400	1.41799500
H	3.07607100	0.73543700	0.09157700
H	3.17964900	2.58288200	1.46133900
H	1.55084600	2.98576800	0.47535400
C	1.44382800	2.34806300	2.69149600
C	1.38868900	3.64448200	3.23286200
C	0.83435100	1.31644600	3.41775800
C	0.73282000	3.90503100	4.43126000
H	1.86996300	4.45810400	2.69269000
C	0.16591800	1.57733900	4.61116700
H	0.88106600	0.29586900	3.05463100
C	0.10708700	2.87032000	5.12640300
H	0.70696700	4.91833200	4.82185100
H	-0.30779200	0.75531400	5.14001400
H	-0.41274800	3.06973100	6.05814600
C	3.86796100	-0.00865900	-2.87498100
H	3.53271800	-0.79946100	-3.55313700
H	2.98704600	0.59234400	-2.62138800
H	4.57907000	0.62749600	-3.40429900
C	7.84361700	-0.72639400	0.16241100
H	8.06276500	-0.94827300	1.20856900

H	8.46459900	-1.38072100	-0.45752700
H	8.14635600	0.30336300	-0.04422500
C	3.32540400	-2.66294000	1.36310600
H	2.58448200	-2.00309600	1.82568800
H	2.75361200	-3.42711200	0.82702700
H	3.91106600	-3.14278300	2.14886000
H	-2.23056700	-2.17001800	-2.94942400
C	-1.78047500	-0.05639900	-1.61545000
H	-1.15424500	0.72704600	-1.18022900
H	-1.71442000	0.01095000	-2.70821200
C	-3.21414600	0.11612700	-1.15988700
C	-4.23980200	0.33949100	-2.08020600
C	-3.53263700	0.03895200	0.20329300
C	-5.55778000	0.50014600	-1.65643800
H	-4.00177000	0.39382700	-3.13996300
C	-4.85093200	0.18688000	0.62517600
H	-2.74676400	-0.12676700	0.94029900
C	-5.86702500	0.42083900	-0.30119700
H	-6.34132000	0.67755000	-2.38634800
H	-5.08489100	0.12843000	1.68393700
H	-6.89286800	0.53888100	0.03282800
C	-3.38515600	3.52883100	-0.31776300
C	-2.17126300	3.15999800	0.53763300
C	-1.00079100	4.15747100	0.47418500
C	-3.11853900	3.65064400	-1.81811300
C	-0.13408600	3.96231100	-0.74207600
C	-2.02739100	4.66486700	-2.15310100
H	-3.78521300	4.48835600	0.03924100
H	-2.49306900	3.09613500	1.58249200
H	-1.37178300	5.18976200	0.49897500
H	-2.82589900	2.67797700	-2.23401400
H	-2.08750200	4.96879100	-3.20121500
C	1.95236800	3.36535100	-1.72205800
H	2.78426500	2.73039300	-1.39481600
C	-0.02344900	3.62007500	-3.17506100
C	1.13577000	2.70227700	-2.82429200
H	0.75712100	1.74283800	-2.44926300
H	1.73711200	2.50982300	-3.71594800

H	-0.77972400	3.07984500	-3.75566100
N	-0.67766700	4.12843500	-1.96394700
N	1.10972400	3.61153800	-0.56519000
H	-2.14382800	5.57522500	-1.55553100
H	-4.04528500	3.94430600	-2.32328600
H	-4.16862700	2.77973600	-0.15915300
H	-1.79057100	2.16548800	0.28250600
H	-0.35313700	4.02642700	1.34349400
H	2.37965200	4.30962500	-2.08282500
H	0.31942300	4.46742900	-3.78101600
C	-2.08697900	-3.04333000	1.77952800
C	-1.50290300	-4.09762200	1.05713200
C	-2.31624800	-5.13912000	0.56119400
C	-3.66757600	-5.06106400	0.81164000
C	-4.23813300	-3.97873100	1.54238600
C	-3.46559500	-2.95321200	2.03853800
H	-1.88324600	-5.95366000	-0.00891400
H	-4.32626500	-5.84012100	0.44166600
H	-5.31087600	-3.96680500	1.70561100
H	-3.87997500	-2.11738500	2.59082600
N	-0.16487400	-3.85539800	0.93709300
N	0.08354200	-2.71957000	1.54765600
N	-1.03878700	-2.22223500	2.04869600
O	-1.07818100	-1.02336200	2.61205000
H	-0.48440400	-0.17068700	1.58753000

TS4^{HOBt}

0 1

C	-1.80283200	1.06350900	-0.15445600
C	-3.40834400	2.52164300	-0.49279500
C	-4.14843100	0.11370600	-0.53653400
H	-3.89240000	-0.48446500	-1.41276400
N	-2.35920900	3.24201200	-0.24097000
N	-1.36447600	2.32846000	-0.02719200
N	-3.12890100	1.17922700	-0.42970400
C	-4.77996900	2.98750100	-0.88013300
H	-4.69856600	3.83372900	-1.56324500
H	-5.34163500	3.30010700	0.01087900

C	-5.51469200	0.79415400	-0.72219600
O	-5.43636700	1.93601600	-1.55053600
C	-0.01186300	2.75670000	0.18589000
C	0.34108800	3.19999900	1.46386100
C	0.89667500	2.63779600	-0.86825200
C	1.68207200	3.50784500	1.68408300
C	2.22413800	2.96801700	-0.59815100
C	2.63465600	3.38266300	0.66924700
H	1.99210400	3.83236300	2.67412700
H	2.96319800	2.84747200	-1.38420800
H	-6.19873200	0.08822200	-1.19399000
C	-1.05076800	-0.15947000	-0.08694000
O	-1.61036300	-1.21418000	-0.79550900
C	0.12444800	-0.30632500	0.58436600
C	0.89841200	-1.51619400	0.56465700
H	0.53276900	0.56455600	1.09152300
H	1.28282000	-1.56231500	-0.71900900
H	0.29506500	-2.42794100	0.60033600
C	2.14912900	-1.56227500	1.36505100
C	2.56282200	-2.74705800	1.98776300
C	2.99474100	-0.44381800	1.45146000
C	3.77331900	-2.81536800	2.67262300
H	1.92494000	-3.62571600	1.93021100
C	4.20087400	-0.51103500	2.13924300
H	2.72978800	0.47501700	0.92996000
C	4.60044800	-1.69766000	2.75370900
H	4.07112600	-3.74731500	3.14448400
H	4.84302000	0.36477800	2.17527600
H	5.54719100	-1.75184700	3.28152100
C	4.09212300	3.66475300	0.92411100
H	4.38281700	4.63340100	0.50507700
H	4.31376900	3.67973400	1.99339700
H	4.70848500	2.89865500	0.44483000
C	-0.68836100	3.29229400	2.55887200
H	-1.47168300	4.01189800	2.30358900
H	-1.17586900	2.32504500	2.72278500
H	-0.22417200	3.60302300	3.49581300
C	0.48791400	2.10185400	-2.21243500

H	-0.53751700	2.38219900	-2.46854800
H	1.15908800	2.47800800	-2.98620000
H	0.57818900	1.00903400	-2.21489300
H	-5.92612900	1.07271100	0.26053900
C	-4.15898400	-0.76473500	0.72250600
H	-3.16314100	-1.18072100	0.88794600
H	-4.41359000	-0.13710900	1.58372800
C	-5.15851200	-1.88719100	0.56768900
C	-6.38316800	-1.86628100	1.23693200
C	-4.87082400	-2.95484400	-0.28885800
C	-7.30709600	-2.89356100	1.05506800
H	-6.61260900	-1.04460200	1.91076500
C	-5.79308800	-3.98024200	-0.47361300
H	-3.91122300	-2.97937800	-0.80074100
C	-7.01482300	-3.95066400	0.19760100
H	-8.25384900	-2.86709400	1.58501000
H	-5.55709700	-4.80577300	-1.13749300
H	-7.73410600	-4.75054400	0.05524000
C	4.07150800	-1.64451500	-1.49438600
C	4.99254400	-0.59792500	-1.34537400
C	6.33525700	-0.88354700	-1.02777900
C	6.67718400	-2.20840600	-0.86532700
C	5.72236600	-3.25286100	-1.00797100
C	4.40583900	-2.99633900	-1.32301200
H	7.06137000	-0.08602100	-0.91105500
H	7.70053400	-2.47038900	-0.61698600
H	6.04502400	-4.27835700	-0.85991500
H	3.66224300	-3.77794100	-1.42792100
N	4.33709100	0.58906200	-1.53843800
N	3.08624200	0.31423400	-1.79393600
N	2.91045200	-1.00082700	-1.78078700
O	1.70487800	-1.54963000	-1.92616200
H	-0.96960800	-1.47795800	-1.47433500

TS4Z^{HOBt}

0 1

C	-1.79518800	1.07419400	0.41276900
C	-2.37639800	3.14685700	-0.00479900

C	0.04909000	2.83126700	0.60135000
H	0.30897900	2.64652200	1.64493500
N	-3.43158100	2.44549900	-0.28994100
N	-3.06075600	1.15525000	-0.02595700
N	-1.33985800	2.35350900	0.41106700
C	-2.21120400	4.63821000	-0.03958500
H	-3.09661800	5.11447500	0.38317900
H	-2.08346400	4.97883200	-1.07613800
C	0.06602000	4.33457800	0.28823100
O	-1.09955200	4.98784700	0.75251200
C	-4.01342400	0.09251000	-0.17391200
C	-4.21259000	-0.43604500	-1.45248700
C	-4.68675300	-0.35917000	0.96122400
C	-5.14044000	-1.46696000	-1.57609700
C	-5.60292000	-1.39582400	0.78477700
C	-5.84312900	-1.95580000	-0.47093800
H	-5.31471100	-1.90312700	-2.55632800
H	-6.14191300	-1.77246700	1.65005600
H	0.92289000	4.79024800	0.78581200
C	-1.06913200	-0.07417700	0.90559500
O	-0.22618200	0.30498600	1.92837300
C	-1.21566200	-1.31866300	0.37198300
C	-0.45946600	-2.53172800	0.58650900
H	-2.00678900	-1.40460700	-0.37023400
H	-0.00588300	-2.85021100	-0.69017300
H	-1.11264800	-3.39900000	0.72031600
C	0.76411300	-2.56992000	1.42168800
C	1.80880200	-1.64411200	1.22801600
C	0.98296200	-3.59747100	2.35508400
C	3.00265100	-1.73123100	1.94106200
H	1.70163100	-0.87038100	0.46772700
C	2.17656600	-3.68919300	3.06261300
H	0.19966400	-4.33252000	2.51983800
C	3.19381000	-2.75463500	2.86543900
H	3.79099800	-1.01049800	1.73957600
H	2.31344600	-4.49514900	3.77746700
H	4.12506900	-2.83186300	3.41657000
C	-6.85842400	-3.05532700	-0.64263000

H	-7.06369200	-3.55639900	0.30526400
H	-7.80281000	-2.64942600	-1.01877000
H	-6.51173200	-3.80064600	-1.36221300
C	-3.42144700	0.07663900	-2.62664100
H	-3.56614800	1.15268600	-2.75892900
H	-2.34821500	-0.09511600	-2.48193300
H	-3.72392300	-0.43046100	-3.54343800
C	-4.41329700	0.24226100	2.31334800
H	-4.49275400	1.33294600	2.28210100
H	-5.12162800	-0.13526100	3.05175500
H	-3.40150400	-0.00639500	2.65184900
H	0.17056100	4.48599100	-0.79677900
C	0.98146800	2.06572100	-0.34272900
H	0.78362500	0.99598800	-0.26391900
H	0.74658800	2.34165700	-1.37572000
C	2.43970200	2.31722000	-0.05348800
C	3.22837100	3.08701900	-0.91006300
C	3.02788800	1.75477700	1.08385100
C	4.58150700	3.28759300	-0.64013700
H	2.78249400	3.51766400	-1.80280900
C	4.37967500	1.94781900	1.35321800
H	2.41739700	1.14900000	1.75032400
C	5.16107200	2.71500300	0.48978900
H	5.18358500	3.88542800	-1.31705200
H	4.82399300	1.49779100	2.23592900
H	6.21563500	2.86494900	0.69702600
C	2.68709800	-2.14524500	-1.71620800
C	3.08873200	-0.81392900	-1.89544200
C	4.42395100	-0.43763400	-1.64370500
C	5.28942400	-1.43149800	-1.24195200
C	4.86325400	-2.78040500	-1.08110500
C	3.56030600	-3.16498300	-1.30673200
H	4.73469500	0.59814400	-1.74082500
H	6.32511200	-1.18584300	-1.03058900
H	5.58746000	-3.51975400	-0.75471900
H	3.21016500	-4.17957900	-1.15707600
N	1.99070000	-0.07113300	-2.23849900
N	0.95649500	-0.86887000	-2.25133800

N	1.35086000	-2.10322200	-1.95231500
O	0.47473100	-3.09542600	-1.79807800
H	0.37553800	-0.42642000	2.13696700

TS4$^{\mathbf{DBU\text{-}DH+}}$

1 1

C	0.10449200	2.27919700	-0.14272200
C	0.54825900	4.41169900	0.17609000
C	2.46253800	2.86923000	0.69476200
H	2.40939400	2.21719100	1.56665900
N	-0.64755500	4.40075600	-0.32176200
N	-0.91944200	3.07405400	-0.52541500
N	1.06859500	3.14600600	0.28179700
C	1.31536100	5.59082400	0.69439200
H	0.62884900	6.28131000	1.18511700
H	1.82200600	6.11414300	-0.12813700
C	3.13422400	4.21015600	1.04883700
O	2.24323600	5.12719500	1.64778400
C	-2.18608000	2.72137600	-1.10181500
C	-2.30723100	2.78664000	-2.49241100
C	-3.23192100	2.33230900	-0.26193800
C	-3.53779700	2.43897900	-3.04806900
C	-4.44922700	2.01664200	-0.86385700
C	-4.61814000	2.05713500	-2.25022400
H	-3.65314100	2.46619700	-4.12827900
H	-5.28653500	1.72534800	-0.23401900
H	3.94116500	4.02089100	1.75720000
C	0.16313700	0.84085700	-0.06924300
O	1.13608400	0.34481200	0.75317900
C	-0.72884000	0.04421100	-0.73102900
C	-0.84687200	-1.38165600	-0.59617400
H	-1.43883400	0.53725000	-1.38681200
H	0.08720900	-1.89008600	-0.34974200
H	-1.45033800	-1.54481200	0.62042100
C	-1.65823100	-2.09803600	-1.61104700
C	-1.27597000	-3.37115700	-2.05799300
C	-2.85553700	-1.56124500	-2.11679800
C	-2.05061500	-4.07790100	-2.97460600

H	-0.35111200	-3.80335300	-1.68055300
C	-3.62614900	-2.26352100	-3.03744500
H	-3.20147600	-0.58850600	-1.76822600
C	-3.22943000	-3.52786200	-3.47327500
H	-1.72776900	-5.06064500	-3.30499300
H	-4.54616000	-1.82203800	-3.41062300
H	-3.83255800	-4.07649600	-4.18896700
C	-1.12966200	3.17687900	-3.34467600
H	-0.76982800	4.17624700	-3.08355600
H	-0.29764400	2.47888200	-3.19778100
H	-1.39922400	3.16872400	-4.40121700
C	-5.94683500	1.70110000	-2.86367900
H	-6.41780800	0.87587300	-2.32313900
H	-6.63094300	2.55455700	-2.82360300
H	-5.83408000	1.41206000	-3.91044100
C	-3.01929900	2.17999800	1.21842800
H	-2.43364200	1.27017800	1.40789900
H	-2.46894200	3.02553500	1.64002100
H	-3.97370500	2.08691300	1.74085900
H	3.56777900	4.65658700	0.14082300
C	3.24636200	2.18137600	-0.43265600
H	2.72183700	1.27381700	-0.73495500
H	3.28984700	2.85787000	-1.29315500
C	4.63767700	1.82805500	0.03734000
C	5.75295700	2.56070000	-0.37277000
C	4.81364200	0.77308600	0.93935400
C	7.02283700	2.24979200	0.11064100
H	5.62713700	3.37716100	-1.07933000
C	6.07971500	0.46626700	1.42916500
H	3.94697000	0.19046000	1.24596000
C	7.18785200	1.20515900	1.01578200
H	7.88141700	2.82539100	-0.21964700
H	6.20367000	-0.35096000	2.13306900
H	8.17506300	0.96466200	1.39635500
C	-6.23755800	-1.01610200	1.56710000
C	-5.00441800	-1.15936700	0.67364900
C	-4.04745400	-2.28455800	1.09577100
C	-5.94533200	-0.73891700	3.04355800

C	-3.11909800	-1.84944300	2.20073300
C	-5.06555700	-1.80322300	3.70672100
H	-6.82217100	-1.94321900	1.49856500
H	-5.32963900	-1.35334200	-0.35443600
H	-4.61155500	-3.17073000	1.40728100
H	-5.45757300	0.23697900	3.16549000
H	-5.16968800	-1.75711500	4.79245200
C	-0.91853900	-1.22962100	2.91139000
H	-0.10553200	-0.70501800	2.39757900
C	-2.81981000	-1.05569400	4.50205500
C	-1.60381300	-0.33625600	3.93838100
H	-1.92002000	0.59643000	3.46065100
H	-0.92102300	-0.07980100	4.75134800
H	-3.44856700	-0.35697600	5.06143200
N	-3.63776300	-1.62816300	3.42581900
N	-1.86529300	-1.67393500	1.89826200
H	-5.38477600	-2.80689300	3.40720200
H	-6.89523900	-0.68939800	3.58560100
H	-6.87636800	-0.21818900	1.17262800
H	-4.43661200	-0.22073500	0.64334600
H	-3.42246300	-2.58098200	0.25238100
H	-0.48577900	-2.10669000	3.41085100
H	-2.51646200	-1.85505200	5.18889600
H	1.68649400	-0.33721800	0.19206100
C	4.90549700	-4.30882400	1.43789400
C	4.41466300	-2.86552100	1.56538700
C	2.95190500	-2.65471100	1.13267200
C	4.88988100	-4.86630900	0.01446600
C	2.81389600	-2.46364800	-0.35858500
C	3.50519500	-4.82159500	-0.63551800
H	4.27542800	-4.94848400	2.07050100
H	4.51046400	-2.55416000	2.61073700
H	2.33474400	-3.49768000	1.46645000
H	5.59180100	-4.31389900	-0.62286300
H	3.46538500	-5.50720400	-1.48514000
C	2.25653800	-1.12026600	-2.21846300
H	2.28223200	-0.04912200	-2.44093000
C	3.27393500	-3.33674000	-2.61296200

C	3.34656700	-1.86390100	-2.97939000
H	4.32200500	-1.45653400	-2.69352300
H	3.23154700	-1.74666500	-4.05913900
H	4.18338000	-3.85766200	-2.93064600
N	3.14324600	-3.50457400	-1.16181400
N	2.41771000	-1.30032300	-0.78016400
H	2.74129500	-5.16204900	0.07211600
H	5.22922100	-5.90710100	0.03719600
H	5.92142900	-4.37701700	1.83984400
H	5.05581600	-2.19636500	0.97751100
H	2.53951800	-1.75858900	1.60214100
H	1.26405200	-1.48757600	-2.52249800
H	2.42274200	-3.81866300	-3.11040100

TS4Z$^{DBU-DH+}$

1 1

C	1.04021100	1.40359700	-0.93225800
C	2.67995300	1.46005500	-2.40419400
C	1.73545400	-0.83115200	-1.99769100
H	1.97823000	-1.32939000	-1.05431300
N	2.47506700	2.70241300	-2.09836400
N	1.45188000	2.66493500	-1.18636200
N	1.81663400	0.62311900	-1.74278100
C	3.74907100	0.90356500	-3.29782000
H	4.67876700	1.45137200	-3.13522700
H	3.45473800	1.00331700	-4.35167100
C	2.76901000	-1.18573600	-3.08177400
O	3.96720600	-0.44782900	-2.96193400
C	0.94655100	3.90703800	-0.67325500
C	0.03267000	4.61152800	-1.46260200
C	1.39740400	4.36746100	0.56486100
C	-0.44341700	5.82327300	-0.96691100
C	0.88957200	5.58377200	1.01899300
C	-0.02541200	6.32353900	0.26859500
H	-1.15888600	6.38870300	-1.55826600
H	1.22099300	5.96209800	1.98264900
H	3.02117500	-2.24306800	-2.99018200
C	0.07895500	0.92077200	0.02959100

O	0.14560300	-0.40903500	0.31832200
C	-0.94454400	1.70731600	0.49752800
C	-2.02456900	1.26776700	1.33140600
H	-1.03026000	2.70448700	0.07426200
H	-2.73364100	2.06692400	1.55988300
H	-2.69475900	0.46371600	0.45044900
C	-1.84122100	0.34482500	2.49164300
C	-2.96805200	-0.29512700	3.02957200
C	-0.60206100	0.09328600	3.09336600
C	-2.86099000	-1.16533900	4.11040400
H	-3.94185300	-0.10836400	2.58069500
C	-0.48952900	-0.77909000	4.17208100
H	0.28687900	0.58749600	2.71224900
C	-1.61650400	-1.41862300	4.68524400
H	-3.75126400	-1.64819900	4.50294700
H	0.48702900	-0.95244100	4.61626300
H	-1.52836600	-2.09983800	5.52548200
C	-0.43007700	4.05350900	-2.78195200
H	0.41069600	3.92022700	-3.46903600
H	-0.90107200	3.07314400	-2.64727700
H	-1.15678100	4.72004200	-3.24801100
C	-0.53049300	7.65147800	0.76886400
H	0.14774800	8.45628000	0.46766900
H	-1.51700600	7.87858400	0.35922500
H	-0.59467500	7.66390600	1.85899500
C	2.36082700	3.56140000	1.38994100
H	1.84383100	2.71354000	1.85436000
H	3.17383800	3.16270000	0.77504300
H	2.79373000	4.17008800	2.18508600
H	2.33060300	-1.01755900	-4.07726700
C	0.33812400	-1.25672000	-2.47482100
H	-0.41826200	-0.82206900	-1.81800500
H	0.17617300	-0.87679000	-3.48924700
C	0.23628500	-2.76399800	-2.44035400
C	0.21324500	-3.53483100	-3.60262400
C	0.20360200	-3.40772500	-1.19805700
C	0.15324000	-4.92647300	-3.52546700
H	0.24138900	-3.04453200	-4.57256600

C	0.14527500	-4.79486100	-1.11792400
H	0.21364500	-2.79510500	-0.29941100
C	0.12040400	-5.55847200	-2.28559000
H	0.13424000	-5.51597800	-4.43648700
H	0.11647300	-5.27995000	-0.14635700
H	0.07427700	-6.64110800	-2.22772500
C	-7.63584400	0.73974100	0.01268900
C	-6.25245000	1.24250700	0.42818200
C	-5.14805500	0.99864500	-0.61557200
C	-7.73006100	-0.76910200	-0.21216000
C	-4.57045400	-0.39313300	-0.55011500
C	-6.75448300	-1.28227300	-1.27287500
H	-7.92667500	1.25060100	-0.91507300
H	-6.31308900	2.31996700	0.60789300
H	-5.53388000	1.19380500	-1.62338600
H	-7.54525800	-1.31081100	0.72409400
H	-7.06519800	-2.26729300	-1.62870300
C	-2.72162800	-1.83103200	-0.09310300
H	-1.85333800	-1.73069600	0.56543500
C	-4.96045900	-2.81499400	-0.47449800
C	-3.75016800	-2.81606700	0.44564400
H	-4.04356300	-2.50708300	1.45502400
H	-3.33461400	-3.82482800	0.50538900
H	-5.80634700	-3.32198700	0.00070300
N	-5.38390200	-1.44292100	-0.78136100
N	-3.31290400	-0.50795000	-0.23172200
H	-6.75796700	-0.62118200	-2.14648200
H	-8.74784200	-1.01822000	-0.52979500
H	-8.36549400	1.03625600	0.77298000
H	-5.95583400	0.78391200	1.38016800
H	-4.31696000	1.68853300	-0.45657500
H	-2.35183200	-2.17178800	-1.07015400
H	-4.73895400	-3.34126000	-1.41116700
H	0.93402600	-0.68712900	0.93477800
C	6.12794300	0.07099800	0.54042000
C	4.65845300	0.30643800	0.18861000
C	3.69248800	0.25230200	1.38222000
C	6.42637500	-1.30160000	1.14294200

C	3.28857400	-1.14163600	1.79382700
C	5.63732200	-1.57853300	2.42398700
H	6.44660000	0.84306300	1.25385900
H	4.57090600	1.30015900	-0.26966700
H	4.12128100	0.78698500	2.24025300
H	6.20848200	-2.09584800	0.41765100
H	6.10983300	-2.38457700	2.99106800
C	1.64012400	-2.77577300	2.16830100
H	0.63088500	-2.98499600	1.80040200
C	4.00763100	-3.44533700	2.28054800
C	2.65584800	-3.80345200	1.68219400
H	2.69993900	-3.76808200	0.58736700
H	2.38111000	-4.81907900	1.97688400
H	4.81620300	-3.95952900	1.74898400
N	4.25675700	-2.00151000	2.19196700
N	2.02756500	-1.44348300	1.72413600
H	5.64717400	-0.69571800	3.07277100
H	7.49511600	-1.36349100	1.37329200
H	6.73339500	0.20924700	-0.36136400
H	4.33892900	-0.41427600	-0.57372900
H	2.75959700	0.76470200	1.13437900
H	1.58502800	-2.79967100	3.26575900
H	4.05572500	-3.75162600	3.33283600

TS4^{HOAr}

0 1

C	-1.54061500	0.85631900	0.67869800
C	-2.68299100	2.63623600	1.25276300
C	-3.95597700	0.95071500	-0.13177600
H	-3.71109100	0.94581000	-1.19677300
N	-1.51573800	2.80886200	1.79488000
N	-0.80875900	1.69308400	1.43468000
N	-2.75495500	1.44609800	0.57203900
C	-3.85793400	3.56754900	1.25651100
H	-3.50885300	4.59765400	1.17828300
H	-4.42896000	3.45269900	2.18832200
C	-5.09622400	1.93737400	0.15851800
O	-4.65880200	3.28150500	0.13212400

C	0.62203300	1.64945200	1.56305600
C	1.19261000	1.25495500	2.77480000
C	1.36843200	1.95139100	0.42268800
C	2.57934500	1.12403400	2.81018700
C	2.75166800	1.80322400	0.50827200
C	3.36969900	1.37292200	1.68199500
H	3.05346200	0.80256100	3.73482200
H	3.35629500	2.02081500	-0.36857300
H	-5.86481900	1.82391300	-0.60654000
C	-1.11118900	-0.36979400	0.05960300
O	-1.65987700	-0.61730200	-1.18680100
C	-0.21519700	-1.20337000	0.65321300
C	0.31411400	-2.39075500	0.04465000
H	0.18683400	-0.88798700	1.61403100
H	-0.42641900	-2.97215700	-0.51297000
H	0.84015300	-1.76409100	-1.07322700
C	1.28765800	-3.19548400	0.81428500
C	1.31571900	-4.59428000	0.71309500
C	2.25189700	-2.58363800	1.63504300
C	2.25158300	-5.35041500	1.41230800
H	0.58408800	-5.09133000	0.08107000
C	3.18132300	-3.33970600	2.34220300
H	2.28878000	-1.49677100	1.69948700
C	3.18762900	-4.72956100	2.23734100
H	2.24534000	-6.43218600	1.31636200
H	3.91223800	-2.83772600	2.97005400
H	3.91582500	-5.31900200	2.78474500
C	0.32617200	0.95796500	3.96959600
H	-0.27312300	1.83072000	4.24414600
H	-0.37179000	0.14008000	3.75847300
H	0.93673200	0.67134900	4.82663300
C	4.86109800	1.16641200	1.73599600
H	5.09347700	0.13994100	2.03774600
H	5.32574900	1.34370300	0.76268000
H	5.32408300	1.83508600	2.46778000
C	0.69962900	2.38775600	-0.85300300
H	0.27460900	1.52400300	-1.37904700
H	-0.10796600	3.09959500	-0.65634100

H	1.42360500	2.85490500	-1.52181300
H	-5.54098600	1.70923300	1.13958400
C	-4.33260000	-0.46515700	0.32409400
H	-3.45715400	-1.11472500	0.25954100
H	-4.64890800	-0.42826800	1.37217800
C	-5.43208800	-1.01772600	-0.55227200
C	-6.76266200	-1.03544800	-0.13100200
C	-5.11508100	-1.49149800	-1.82899200
C	-7.76334100	-1.51930100	-0.97174400
H	-7.01571800	-0.67446400	0.86290900
C	-6.11303900	-1.97424300	-2.66971200
H	-4.07504700	-1.48431300	-2.14827600
C	-7.44024300	-1.98844100	-2.24219100
H	-8.79405500	-1.53207800	-0.63209500
H	-5.85555200	-2.34425000	-3.65704900
H	-8.21829100	-2.36752600	-2.89681400
H	-0.90640500	-0.65439400	-1.81073100
O	0.98761500	-1.04867000	-2.08068500
C	2.18934000	-0.52614000	-2.14119600
C	2.45252500	0.56347200	-3.00800600
C	3.26024300	-1.03344000	-1.36068600
C	3.70415000	1.14292500	-3.06301200
H	1.64014600	0.94018600	-3.62049600
C	4.50958600	-0.44971200	-1.40710300
H	3.08493800	-1.88761100	-0.71479700
C	4.72391700	0.64306200	-2.24919700
H	3.90717500	1.98395900	-3.71548000
H	5.32594100	-0.82582900	-0.80160900
N	6.01902300	1.28025700	-2.26555600
O	6.86209800	0.90700200	-1.45637800
O	6.22165800	2.17177200	-3.07984400

TS4Z^{HOAr}

0 1

C	-1.27912400	-0.83189700	-0.92329500
C	-1.04518100	-2.93835700	-1.49339900
C	1.02504100	-1.52758200	-1.76595800
H	1.08138900	-0.81124300	-2.58660800

N	-2.23919300	-2.86610700	-0.98908600
N	-2.37925900	-1.54999600	-0.63918800
N	-0.40761200	-1.72570200	-1.45913400
C	-0.36338100	-4.12070000	-2.11715400
H	-1.07255700	-4.66547100	-2.74166900
H	0.01685200	-4.79440100	-1.33692100
C	1.60349200	-2.89025600	-2.18274300
O	0.68167800	-3.65166200	-2.93839600
C	-3.62577200	-1.07750100	-0.11146000
C	-3.90623900	-1.33409200	1.23303600
C	-4.49558500	-0.39232200	-0.96181200
C	-5.11848000	-0.86460300	1.73340200
C	-5.69447800	0.05939800	-0.41192400
C	-6.01870400	-0.16286800	0.92778600
H	-5.36406900	-1.04935900	2.77607100
H	-6.39398400	0.59472300	-1.04894500
H	2.48547100	-2.72596500	-2.80297600
C	-1.05815900	0.58646900	-0.81254400
O	-0.24060100	1.02337000	-1.83517500
C	-1.58475400	1.33337300	0.20258500
C	-1.36071200	2.70464600	0.57477000
H	-2.25245300	0.78599000	0.86549200
H	-2.26989900	3.17548000	0.95580800
H	-0.58582400	2.61548000	1.75360300
C	-0.50823400	3.61064700	-0.24031400
C	0.86673000	3.34740000	-0.39576700
C	-1.01139900	4.78855400	-0.81243300
C	1.68733300	4.19599300	-1.13711000
H	1.29861500	2.47268700	0.08948300
C	-0.18808900	5.64792500	-1.53330900
H	-2.06545700	5.02320600	-0.69094700
C	1.16313600	5.35168500	-1.71067500
H	2.74264400	3.96034200	-1.24452500
H	-0.60575100	6.55077600	-1.96857600
H	1.80056600	6.01883500	-2.28137800
C	-2.92444000	-2.08736800	2.09049100
H	-2.85971400	-3.13482700	1.78065100
H	-1.91787300	-1.66308400	2.00151900

H	-3.22200800	-2.05337400	3.13927500
C	-7.30617200	0.36575700	1.50397100
H	-8.06412500	0.49178100	0.72824300
H	-7.70004900	-0.30588100	2.26997700
H	-7.14351600	1.34181700	1.97213900
C	-4.14236600	-0.14976800	-2.40431800
H	-3.34579600	0.59741400	-2.49006800
H	-3.78753600	-1.06748700	-2.88272500
H	-5.01105300	0.21352400	-2.95509500
H	1.90620100	-3.45517200	-1.28788000
C	1.74929500	-0.99634200	-0.52319000
H	1.20916400	-0.13722900	-0.11108300
H	1.73455500	-1.78312000	0.23964400
C	3.16949500	-0.58978700	-0.82727400
C	4.24643800	-1.40539400	-0.47650600
C	3.41816700	0.62799600	-1.46911500
C	5.55351600	-1.00295900	-0.74567100
H	4.06216500	-2.35148800	0.02821300
C	4.72130500	1.02748900	-1.74600000
H	2.57715300	1.26330800	-1.74106600
C	5.79321400	0.21247900	-1.38047700
H	6.38314500	-1.63858400	-0.45329800
H	4.90254200	1.97723800	-2.23975500
H	6.81099500	0.52711800	-1.58750600
H	0.01248500	1.94535200	-1.67514000
O	0.25400400	2.67792000	2.61720100
C	0.98319500	1.59317900	2.63481500
C	2.39044800	1.67369600	2.46616300
C	0.40324600	0.30696400	2.80162700
C	3.17481900	0.53884000	2.45723600
H	2.82820000	2.65619400	2.32426800
C	1.18274700	-0.83200700	2.78174100
H	-0.66770400	0.24421500	2.96831800
C	2.56377300	-0.70847500	2.60850300
H	4.24608000	0.58927300	2.29865400
H	0.74737400	-1.81785700	2.90270700
N	3.37052800	-1.89569300	2.51219600
O	2.80551600	-2.96717100	2.29597800

O	4.58537900	-1.79357900	2.61745800

TS4^{DH+}

1 1

C	1.94067700	-1.39419300	-0.16721200
C	3.53607300	-2.58184200	0.76586800
C	4.29169600	-0.35597700	-0.12863900
H	4.34048300	-0.18410200	-1.20388300
N	2.45419200	-3.26286400	0.98175600
N	1.46300400	-2.51323900	0.41216200
N	3.27601200	-1.41196800	0.09512000
C	4.94941000	-2.96971100	1.08320600
H	5.08041800	-4.03749300	0.90419600
H	5.18061400	-2.75210100	2.13480400
C	5.64303200	-0.87194900	0.40289800
O	5.80556800	-2.26214800	0.21697000
C	0.10072900	-2.95713600	0.48111200
C	-0.69634300	-2.46357800	1.51557000
C	-0.37980100	-3.79476300	-0.52896500
C	-2.04537400	-2.81871800	1.50668000
C	-1.73233000	-4.12737200	-0.48914300
C	-2.58029300	-3.63544400	0.50834500
H	-2.69458800	-2.43931100	2.29287300
H	-2.13867000	-4.77179600	-1.26493000
H	6.44668100	-0.36803100	-0.13454300
C	1.23633600	-0.41516000	-0.95867000
O	2.06704500	0.45477700	-1.64469000
C	-0.11948100	-0.32741700	-1.04910700
C	-0.86384200	0.71120600	-1.69736800
H	-0.70556400	-1.07337000	-0.52494600
H	-1.17443200	1.49343900	-0.66404600
H	-0.29037800	1.38207100	-2.34119600
C	-2.23028000	0.39610500	-2.20563300
C	-3.06161700	1.45766100	-2.59681100
C	-2.75636900	-0.90134700	-2.25574200
C	-4.36778100	1.23500900	-3.01699600
H	-2.66545600	2.47122200	-2.57094400
C	-4.06972800	-1.12534600	-2.66695600

H	-2.13418800	-1.75274000	-1.99036100
C	-4.88349300	-0.06155300	-3.04615400
H	-4.98698000	2.07470900	-3.31788300
H	-4.45105600	-2.14185000	-2.70657400
H	-5.90357900	-0.23891500	-3.37067600
C	-4.04984800	-3.96272900	0.48203200
H	-4.54270000	-3.65287100	1.40592700
H	-4.53778700	-3.44597000	-0.35132600
H	-4.21168300	-5.03463800	0.34303300
C	-0.12917200	-1.51998900	2.54159100
H	0.78793700	-1.91753100	2.98497000
H	0.11611100	-0.55375700	2.08134900
H	-0.85176900	-1.34006300	3.33893300
C	0.52810000	-4.25932600	-1.63475300
H	1.43049300	-4.72878700	-1.23372600
H	0.01708800	-4.97628800	-2.27816800
H	0.84265800	-3.41015700	-2.25188500
H	5.73464900	-0.62952500	1.47252000
C	3.90142500	0.93879700	0.60197400
H	2.87204900	1.20462200	0.35276400
H	3.95293900	0.76056200	1.68188600
C	4.82517700	2.06488700	0.19926900
C	5.88839600	2.46541600	1.01057600
C	4.63363300	2.70022600	-1.03182800
C	6.74692900	3.48344500	0.59921200
H	6.04095900	1.98484300	1.97395000
C	5.48906900	3.71684600	-1.44366900
H	3.79868800	2.39446000	-1.65752400
C	6.54937500	4.11019700	-0.62828800
H	7.56810000	3.78806700	1.23994600
H	5.32726200	4.20557200	-2.39913800
H	7.21626000	4.90434600	-0.94776600
C	-5.40667600	0.25578200	1.67455200
C	-4.23293300	0.04979700	0.71799600
C	-2.87966900	0.54538400	1.25739000
C	-5.70606000	1.71756300	2.00617700
C	-2.66544400	2.02358900	1.04646100
C	-4.51319700	2.44798000	2.62609700

H	-5.19751200	-0.28029900	2.61108600
H	-4.13083700	-1.01992300	0.50461300
H	-2.77994000	0.30065400	2.32347600
H	-6.01564100	2.26027200	1.10414300
H	-4.84563900	3.35311200	3.13951500
C	-1.51382300	3.79743700	-0.05192500
H	-1.01980000	3.86251900	-1.02770700
C	-3.53674400	4.30305000	1.27932900
C	-2.85777000	4.51678900	-0.06313700
H	-3.48334800	4.09703500	-0.85886100
H	-2.73533300	5.58540200	-0.25125600
H	-4.58406100	4.61748700	1.23360500
N	-3.51555300	2.88303000	1.64476100
N	-1.69147500	2.38725700	0.26168800
H	-4.03340100	1.81896300	3.38407200
H	-6.54305900	1.76171600	2.71068400
H	-6.30135000	-0.20598500	1.24440700
H	-4.43216500	0.53526700	-0.24593900
H	-2.06532200	0.03185200	0.74284200
H	-0.84230300	4.26079000	0.68222100
H	-3.04527700	4.88949700	2.06543600
H	1.81432400	0.43879100	-2.57864000

TS4Z^{DH+}

1 1

C	-2.34233400	0.64193000	-0.10088200
C	-3.79142000	2.18863100	-0.67014500
C	-1.55115800	3.07201100	0.06542500
H	-1.33712900	3.06014100	1.13548600
N	-4.39255900	1.06670100	-0.92316700
N	-3.48402300	0.10769900	-0.56720600
N	-2.52343500	1.98547300	-0.18791100
C	-4.33959200	3.58013700	-0.79369000
H	-5.37389400	3.59568900	-0.44846800
H	-4.30786000	3.90790500	-1.84179300
C	-2.21924000	4.39471900	-0.33857900
O	-3.58243300	4.43675600	0.02990400
C	-3.86717800	-1.27634300	-0.59337100
C	-3.82986800	-1.95124900	-1.81582400

C	-4.27153700	-1.87135900	0.60278500
C	-4.19939900	-3.29375700	-1.81328000
C	-4.62515800	-3.21949500	0.55328900
C	-4.59462400	-3.94233700	-0.64002800
H	-4.17618000	-3.84777000	-2.74825300
H	-4.93942000	-3.71316600	1.46908500
H	-1.70981600	5.21468700	0.16890100
C	-1.19228800	-0.02845900	0.45136300
O	-0.52326300	0.75437300	1.37779900
C	-0.78103500	-1.26596300	0.04232100
C	0.38633700	-1.97748400	0.48018900
H	-1.35401800	-1.70807100	-0.76918600
H	1.41800500	-1.35676300	-0.24352000
H	0.42251700	-2.99851800	0.09268300
C	0.81092100	-1.90750500	1.91643800
C	2.16508800	-1.99938800	2.27133000
C	-0.12513400	-1.77999100	2.95604300
C	2.56745500	-1.94280500	3.60196000
H	2.90890400	-2.11091600	1.48720600
C	0.27585500	-1.70665800	4.28986000
H	-1.18556500	-1.77018000	2.70872300
C	1.62592900	-1.78440400	4.61857100
H	3.62287600	-2.01449100	3.84704500
H	-0.47126000	-1.60244700	5.07052200
H	1.94224400	-1.73101800	5.65491000
C	-5.00651200	-5.39072300	-0.67533800
H	-4.34314300	-5.97197200	-1.32032600
H	-4.99306200	-5.83109000	0.32351200
H	-6.02119200	-5.49102300	-1.07323900
C	-3.39610300	-1.24405100	-3.07195100
H	-4.07120300	-0.41567000	-3.30606700
H	-2.39101500	-0.82228400	-2.96241700
H	-3.38678000	-1.93295800	-3.91730900
C	-4.32683800	-1.08133900	1.88254300
H	-4.95714700	-0.19414000	1.76643400
H	-4.73347100	-1.68943900	2.69146200
H	-3.33088800	-0.73659600	2.18160900
H	-2.12277700	4.54145900	-1.42540700

C	-0.26440200	2.85730700	-0.74113700
H	0.07628800	1.82775200	-0.61136600
H	-0.48482200	3.00059400	-1.80468800
C	0.82223200	3.80647500	-0.29303300
C	1.25544100	4.85279500	-1.10853400
C	1.40996000	3.64004800	0.96683700
C	2.26297500	5.71524600	-0.67874400
H	0.80588900	4.99033900	-2.08859100
C	2.41669200	4.49900000	1.39660800
H	1.07295400	2.82544400	1.60456600
C	2.84670100	5.53887200	0.57264900
H	2.59146900	6.52377300	-1.32375200
H	2.86670000	4.35713200	2.37421200
H	3.63219400	6.20875700	0.90680600
C	5.61400200	-3.75820800	-1.58766900
C	4.17908600	-3.68976700	-1.06363400
C	3.28539400	-2.66171600	-1.77996700
C	6.41662600	-2.46731300	-1.43124000
C	3.46208200	-1.25484900	-1.26720800
C	5.77061600	-1.27000400	-2.12915200
H	5.58334300	-4.02412300	-2.65281100
H	3.71681800	-4.67362000	-1.18573700
H	3.47594200	-2.68688900	-2.85949000
H	6.55318500	-2.22323600	-0.37018500
H	6.50413800	-0.47414000	-2.27519000
C	2.55985300	0.65133500	-0.14501400
H	1.77923700	0.75957100	0.61751600
C	4.97407700	0.58198700	-0.66358400
C	3.95456600	0.85084300	0.43229800
H	4.10309400	0.15307800	1.26356500
H	4.07693200	1.86824100	0.81163200
H	5.97815900	0.48323100	-0.23979800
N	4.66947600	-0.67055000	-1.36845500
N	2.43085500	-0.68130700	-0.71104000
H	5.41539700	-1.55369500	-3.12600500
H	7.41463800	-2.61533500	-1.85611900
H	6.13899500	-4.57243100	-1.07854600
H	4.18209400	-3.48045500	0.01341000

H	2.23257200	-2.91332200	-1.63961500
H	2.36935800	1.40536300	-0.92100700
H	4.99446700	1.40564900	-1.38736900
H	-0.27281000	0.20478400	2.13876700

TS4^{DBU}

0 1

C	1.29311900	-2.02166500	-0.26672200
C	2.76792000	-3.15411700	0.93023000
C	3.63828900	-1.04912700	-0.12586000
H	3.76007000	-0.97629400	-1.20579100
N	1.65167200	-3.78289400	1.12863700
N	0.73366500	-3.07543400	0.38632100
N	2.60753100	-2.07111300	0.11063800
C	4.13985900	-3.51796400	1.41585500
H	4.27533300	-4.59903200	1.36249500
H	4.27891400	-3.19279900	2.45694600
C	4.92884000	-1.50401000	0.57884100
O	5.09055300	-2.91006900	0.56927400
C	-0.64214300	-3.46199400	0.41675500
C	-1.43097400	-2.99108700	1.46818200
C	-1.15098600	-4.24521300	-0.62149700
C	-2.78413400	-3.32905300	1.46494200
C	-2.50807300	-4.55719000	-0.58492700
C	-3.33779500	-4.10478800	0.44486600
H	-3.42061000	-2.96844600	2.26926500
H	-2.93137600	-5.16107400	-1.38418900
H	5.78904200	-1.07069300	0.06634600
C	0.77299200	-1.06606500	-1.24036100
O	1.63025300	-0.48093000	-2.01598000
C	-0.58605200	-0.79674800	-1.15503500
C	-1.18975600	0.30419900	-1.84227100
H	-1.17891700	-1.33456600	-0.42466300
H	-0.74498400	1.32268500	-0.88365800
H	-0.63472700	0.67635600	-2.70409200
C	-2.63778400	0.49855100	-1.89092400
C	-3.17221200	1.63079500	-2.54444400
C	-3.56295700	-0.35449900	-1.25119100

C	-4.53486100	1.89844300	-2.55364900
H	-2.48552700	2.30761500	-3.05033900
C	-4.92761700	-0.07932400	-1.25524100
H	-3.21238600	-1.26073600	-0.76190300
C	-5.43085000	1.04847900	-1.90070300
H	-4.90328700	2.77989300	-3.07217700
H	-5.60744500	-0.76342800	-0.75259500
H	-6.49560900	1.25755100	-1.90400400
C	-4.80890600	-4.43126100	0.43044400
H	-5.29124900	-4.13206900	1.36310400
H	-5.30953900	-3.91177200	-0.39272200
H	-4.97191200	-5.50274300	0.28612000
C	-0.82961100	-2.10030700	2.52218500
H	-0.02890500	-2.61297000	3.06363900
H	-0.39095900	-1.20868300	2.05706900
H	-1.58743900	-1.77909100	3.23880500
C	-0.25803400	-4.68214700	-1.74915200
H	0.64864800	-5.16231500	-1.37005500
H	-0.77670800	-5.37915900	-2.40906000
H	0.05088300	-3.81009100	-2.33608100
H	4.92973900	-1.14602500	1.62048500
C	3.18253000	0.31456900	0.41912800
H	2.15892800	0.49498700	0.08261200
H	3.19192100	0.28212600	1.51537900
C	4.08081000	1.41319200	-0.09342500
C	5.05889200	2.00235100	0.70844500
C	3.94702600	1.82694500	-1.42476500
C	5.89395100	2.99350000	0.19341300
H	5.16515100	1.68600300	1.74358800
C	4.77991800	2.81643100	-1.93805700
H	3.17495000	1.35196600	-2.02901900
C	5.75607500	3.40144700	-1.13045900
H	6.65037700	3.44579500	0.82735100
H	4.66904600	3.13459700	-2.97028100
H	6.40572100	4.17257700	-1.53237700
C	-4.06239200	3.11909500	2.21823600
C	-3.55932900	2.10731200	1.18784000
C	-2.09908900	1.66551200	1.38142900

C	-3.30964300	4.44951000	2.24381800
C	-1.10150600	2.64941200	0.82366300
C	-1.81455000	4.28369700	2.51985700
H	-3.99458300	2.66537800	3.21674800
H	-4.18328300	1.21073900	1.24193300
H	-1.88755600	1.49555000	2.44503200
H	-3.43211900	4.98083000	1.29157600
H	-1.38125300	5.22557800	2.86410900
C	0.59014200	3.16658700	-0.79084900
H	0.76338300	2.80884300	-1.81058500
C	-0.28920500	4.95873500	0.66893800
C	0.02716700	4.58145000	-0.76963000
H	-0.88831300	4.61145100	-1.36982200
H	0.73601900	5.29786500	-1.18972700
H	-0.90230900	5.86478700	0.70137200
N	-1.04458300	3.89126100	1.33743600
N	-0.34966000	2.24964800	-0.16537800
H	-1.65286100	3.55394300	3.32074900
H	-3.73718900	5.08751000	3.02440700
H	-5.12466600	3.31133200	2.03576100
H	-3.68015500	2.49129800	0.16660000
H	-1.92627400	0.71731600	0.86522100
H	1.55204900	3.12652400	-0.26352400
H	0.63073500	5.15668400	1.23256200

TS4Z^{DBU}

0 1

C	-2.45528900	0.48195700	0.00405400
C	-4.15408200	1.84141800	-0.36849000
C	-1.99469900	2.98038200	0.22140200
H	-1.66213600	2.90778600	1.25684100
N	-4.63897500	0.65887500	-0.58547300
N	-3.57905700	-0.18618500	-0.35107500
N	-2.83225500	1.79445100	-0.02129400
C	-4.87832500	3.15403200	-0.39251600
H	-5.88640800	3.02317600	0.00258800
H	-4.94517200	3.53713500	-1.42078600
C	-2.85930900	4.22874000	-0.01273100

O	-4.19013100	4.06254100	0.43844500
C	-3.82397700	-1.59852700	-0.36772200
C	-4.04991400	-2.20907500	-1.60303000
C	-3.87486000	-2.29803800	0.84399800
C	-4.32543500	-3.57661300	-1.61014800
C	-4.14523500	-3.66270200	0.78247100
C	-4.37361400	-4.31796600	-0.43034500
H	-4.49845300	-4.07212100	-2.56223200
H	-4.18370200	-4.22989100	1.70967100
H	-2.42699500	5.06670300	0.53667100
C	-1.14637300	0.00660200	0.46323600
O	-0.54345900	0.79496000	1.30999100
C	-0.73663700	-1.17792200	-0.12600000
C	0.48563400	-1.94577500	0.00012400
H	-1.41087600	-1.55407700	-0.89315700
H	1.53760800	-1.07567500	-0.63927200
H	0.46529600	-2.82769800	-0.64522900
C	1.24108300	-2.22049300	1.22754800
C	2.14981600	-3.30191200	1.22705100
C	1.18639200	-1.44130900	2.40370500
C	2.95689400	-3.59070000	2.32051400
H	2.21005900	-3.93090700	0.33967800
C	1.99980100	-1.73506900	3.49572500
H	0.51436000	-0.58777700	2.42380000
C	2.89257100	-2.80661000	3.47281600
H	3.63703000	-4.43788400	2.27355500
H	1.93233700	-1.11308800	4.38548400
H	3.51802400	-3.02963200	4.33141200
C	-4.67210100	-5.79498600	-0.44941700
H	-4.74168300	-6.17233700	-1.47155900
H	-3.89194300	-6.35689900	0.07168300
H	-5.61920500	-6.00721200	0.05602500
C	-3.97454900	-1.41317900	-2.87867500
H	-4.77767300	-0.67218000	-2.92814200
H	-3.02731600	-0.86665500	-2.94034200
H	-4.05126300	-2.06993000	-3.74665500
C	-3.61987900	-1.61172300	2.15734800
H	-4.13468600	-0.64769800	2.20980900

H	-3.96170700	-2.23353000	2.98645900
H	-2.54842500	-1.41927200	2.28252800
H	-2.86526700	4.48238100	-1.08434200
C	-0.77000000	3.00095400	-0.70261600
H	-0.32720300	2.00314200	-0.70929000
H	-1.08933100	3.24593700	-1.72216200
C	0.24899300	4.00030400	-0.20957800
C	0.54635900	5.16197700	-0.92326000
C	0.90562400	3.76078500	1.00472900
C	1.48818900	6.07033400	-0.43996300
H	0.04087500	5.35502700	-1.86641200
C	1.84300200	4.66879600	1.48810800
H	0.67110900	2.84566600	1.54698100
C	2.13841300	5.82567600	0.76610700
H	1.71253900	6.96842400	-1.00693200
H	2.34664100	4.47350100	2.43035800
H	2.87059900	6.53223300	1.14386900
C	6.13497600	-2.82531200	-1.52705700
C	4.66178100	-2.99644200	-1.15658000
C	3.69238100	-2.06669400	-1.90844500
C	6.72315500	-1.46060700	-1.17191300
C	3.59553400	-0.69176400	-1.29864700
C	5.97060200	-0.30284000	-1.82746000
H	6.25014300	-2.98713700	-2.60759300
H	4.36550300	-4.02786600	-1.37387400
H	3.98522300	-1.98444800	-2.96257700
H	6.71906400	-1.31137600	-0.08496000
H	6.57925600	0.60445800	-1.81313600
C	2.28409400	0.99086400	-0.20631400
H	1.37528400	0.93165000	0.41153200
C	4.74449800	1.22863600	-0.29192800
C	3.53205200	1.25744700	0.62482600
H	3.61773300	0.47898300	1.39140500
H	3.47050500	2.22780500	1.12327300
H	5.67248800	1.18597600	0.28821400
N	4.71469700	0.04287000	-1.15863600
N	2.42637900	-0.28410500	-0.89465900
H	5.77098800	-0.52896200	-2.88094100

H	7.76800000	-1.42259400	-1.49768400
H	6.71782700	-3.60817200	-1.03101700
H	4.52158700	-2.85499900	-0.07839900
H	2.68139900	-2.48178200	-1.89049300
H	2.14491400	1.79488400	-0.94307800
H	4.78085500	2.12970800	-0.91666300

M4^{com}

0 1

C	0.81883700	0.07436600	1.04977600
C	2.30992900	-1.29619200	1.97597800
C	3.30576800	0.60660900	0.69312800
H	3.31896000	0.59503500	-0.40085300
N	1.18788000	-1.68120600	2.48026600
N	0.25071900	-0.81865900	1.93464300
N	2.17528600	-0.21580700	1.14018900
C	3.66035600	-1.90613300	2.21417800
H	3.59433100	-2.98852400	2.08614100
H	3.99833600	-1.68391300	3.23729200
C	4.59919900	-0.01182700	1.25188000
O	4.58045800	-1.42044100	1.26684100
C	-1.10897900	-0.95454900	2.32711700
C	-1.65379500	-0.01453700	3.21430200
C	-1.84719100	-2.04360700	1.85147100
C	-2.99432200	-0.14834200	3.56929200
C	-3.17837700	-2.15611500	2.26264700
C	-3.77286600	-1.21013800	3.09844800
H	-3.43680000	0.58028900	4.24500700
H	-3.76299100	-3.00788800	1.92036300
H	5.43616600	0.29318300	0.62106600
C	0.24793000	1.00194700	0.18109100
O	1.04825200	1.54594100	-0.80279500
C	-1.07271500	1.50798900	0.30713500
C	-1.58063300	2.54561100	-0.41741400
H	-1.71314200	1.03628800	1.04515500
H	-0.94601900	3.01336900	-1.16668000
H	-2.91577100	1.69119000	-2.29025700
C	-2.94557900	3.03849500	-0.30638200

C	-3.46056500	3.88955800	-1.30810200
C	-3.82464500	2.67499800	0.73742400
C	-4.78375600	4.32109100	-1.29231200
H	-2.80106300	4.20785100	-2.11308900
C	-5.14307900	3.11045400	0.75142400
H	-3.46897100	2.04409200	1.54877000
C	-5.64124200	3.92779600	-0.26666100
H	-5.14483100	4.96834700	-2.08594000
H	-5.79217600	2.81022200	1.56961300
H	-6.67300000	4.26192700	-0.25028200
C	-0.80472000	1.10900400	3.74517100
H	0.11997500	0.71876700	4.18082000
H	-0.51728200	1.79812500	2.94259200
H	-1.34279400	1.67338800	4.50849600
C	-5.22563500	-1.31531200	3.48455700
H	-5.60808100	-2.32610100	3.32432500
H	-5.37501800	-1.05232800	4.53471900
H	-5.83451600	-0.62696800	2.88693500
C	-1.20884800	-3.05958700	0.94472900
H	-0.74889600	-2.58149000	0.07339700
H	-0.39485500	-3.57887500	1.45859300
H	-1.94221300	-3.79942900	0.61514600
H	4.77436400	0.36753900	2.27251600
C	3.18333000	2.05685800	1.19086000
H	2.18935600	2.43072500	0.94153100
H	3.29530500	2.06857900	2.28128700
C	4.23253600	2.92128000	0.53516700
C	5.35249800	3.37818400	1.23202200
C	4.10175100	3.24222400	-0.82121800
C	6.32432200	4.14483600	0.59030500
H	5.46170700	3.13490000	2.28608400
C	5.07172700	4.00480300	-1.46391800
H	3.22322800	2.88933000	-1.35700900
C	6.18667600	4.45863800	-0.75912500
H	7.18806700	4.49631800	1.14591700
H	4.95745200	4.24980600	-2.51528500
H	6.94223700	5.05552100	-1.25980900
C	-5.61924100	-1.71112600	-0.24939500

C	-5.05245800	-0.29179600	-0.27570800
C	-4.93095000	0.31967000	-1.68646300
C	-4.77530100	-2.75393800	-0.98033600
C	-3.67850300	-0.14339200	-2.37811200
C	-4.55850900	-2.42155300	-2.45549400
H	-6.62411000	-1.69788400	-0.69237100
H	-5.70690400	0.36445600	0.30499100
H	-5.80433700	0.05944100	-2.29435700
H	-3.79283400	-2.85326500	-0.50135100
H	-4.24553100	-3.30644800	-3.01188300
C	-1.40433700	0.42615700	-3.12100400
H	-0.70527600	1.13098600	-2.66519600
C	-2.18513800	-1.93483700	-3.09617800
C	-1.07691000	-0.98721400	-2.68141000
H	-0.95688800	-0.99512300	-1.59329200
H	-0.11936400	-1.29570800	-3.09833400
H	-2.04901400	-2.90932800	-2.61867100
N	-3.49801200	-1.42457300	-2.66043600
N	-2.74727900	0.75461700	-2.63899200
H	-5.48435200	-2.06632000	-2.91836400
H	-5.26828900	-3.72876200	-0.91409500
H	-5.74419900	-2.01955200	0.79371300
H	-4.06978400	-0.26253500	0.21574600
H	-4.89726800	1.41096600	-1.61697100
H	-1.37452400	0.53389500	-4.20999700
H	-2.21044300	-2.08552600	-4.18123000
C	3.47480100	-1.95871900	-1.57776500
C	3.26698000	-3.14790900	-0.86234900
C	4.36253000	-3.98805200	-0.56949200
C	5.60547800	-3.57840600	-0.99624100
C	5.79340700	-2.35923700	-1.70810800
C	4.73835200	-1.53048100	-2.01698000
H	4.22122000	-4.91765300	-0.02715900
H	6.47323900	-4.19630500	-0.78817800
H	6.79700700	-2.08577200	-2.01821300
H	4.85708900	-0.60240100	-2.56514100
N	1.94002300	-3.24798500	-0.56378000
N	1.34223700	-2.19159100	-1.06948100

N	2.23439300	-1.41819400	-1.67567700
O	1.92043400	-0.28981700	-2.29231200
H	1.38492800	0.77646300	-1.39479900

M4Z^{com}

0 1

C	2.50945700	-0.93188600	-0.04679900
C	2.58729400	-2.25197000	1.74769800
C	0.74235300	-2.79400100	0.15681800
H	-0.05937200	-2.07897200	-0.01221300
N	3.58496000	-1.45643900	1.92007500
N	3.57213200	-0.64040000	0.79430600
N	1.91314300	-2.02666000	0.57661200
C	2.09091300	-3.29510300	2.69670700
H	2.33496000	-3.00435700	3.71893600
H	2.55328300	-4.27056200	2.48204600
C	0.33253200	-3.78542200	1.27278400
O	0.68798100	-3.37465300	2.57541900
C	4.38605700	0.53038400	0.84100600
C	5.75741500	0.38578500	0.59659000
C	3.81714700	1.76608200	1.16834100
C	6.56141000	1.52117600	0.66009700
C	4.65969700	2.87914200	1.20399400
C	6.02778500	2.77772100	0.95553300
H	7.62709200	1.42544300	0.46439000
H	4.23246200	3.85047600	1.44392600
H	-0.75497900	-3.89575300	1.25917600
C	2.13838300	-0.29364700	-1.21460100
O	0.96553300	-0.68867900	-1.84461900
C	3.03113600	0.61093800	-1.88824000
C	2.78158300	1.49201600	-2.89115500
H	4.07718100	0.51924000	-1.60091000
H	3.65057900	1.93017700	-3.37831400
H	-2.78197500	1.04119300	-0.98583500
C	1.49367300	1.97961800	-3.39023000
C	1.38724500	2.44162700	-4.71353500
C	0.35820400	2.09344400	-2.57005000
C	0.19280900	2.95214400	-5.20961700

H	2.25999700	2.38627500	-5.35968900
C	-0.83910600	2.59498100	-3.06748900
H	0.41971800	1.79773500	-1.52591500
C	-0.93315100	3.02496500	-4.39030100
H	0.13911100	3.29197000	-6.23982300
H	-1.70553900	2.65183000	-2.41333600
H	-1.86869300	3.41741400	-4.77634100
C	6.32483900	-0.96460900	0.25107700
H	6.15433300	-1.67930200	1.06129600
H	5.83640200	-1.37226300	-0.64044300
H	7.39743700	-0.89751300	0.06022400
C	6.92013700	3.99022300	1.03449800
H	6.36248600	4.90613800	0.82615700
H	7.35675700	4.08802700	2.03391500
H	7.74526400	3.91908700	0.32161100
C	2.34745000	1.89282300	1.45523400
H	1.76344700	1.85207900	0.52983900
H	1.98397300	1.06676600	2.07471400
H	2.13712200	2.83700200	1.96306100
H	0.78634400	-4.76893000	1.06875300
C	0.97686700	-3.56613900	-1.14915900
H	1.37737600	-2.87425000	-1.89180300
H	1.71210700	-4.36000600	-0.97425500
C	-0.33041000	-4.14765100	-1.63603100
C	-0.53063500	-5.52704700	-1.71155000
C	-1.38326400	-3.29101600	-1.98497900
C	-1.75240100	-6.04902000	-2.13377000
H	0.28001800	-6.19799700	-1.43814000
C	-2.60301600	-3.81258200	-2.40580900
H	-1.24517500	-2.21148200	-1.92253600
C	-2.79221700	-5.19218900	-2.48254700
H	-1.89015300	-7.12448000	-2.18889100
H	-3.40951700	-3.13884600	-2.68089600
H	-3.74421300	-5.59511300	-2.81358600
C	-2.55531100	3.69885500	3.04858300
C	-2.00203500	2.62543200	2.11096900
C	-2.17740200	2.93617000	0.61296800
C	-4.05915000	3.95024000	2.93371200

C	-3.55632800	2.58771500	0.12186100
C	-4.49272100	4.36121600	1.52658500
H	-2.02955500	4.64181200	2.84698100
H	-0.93157400	2.50344000	2.29746800
H	-1.97600600	3.99482400	0.41175800
H	-4.62517100	3.05774700	3.22840000
H	-5.47387700	4.83958800	1.54765300
C	-4.95712900	1.16112700	-1.25300700
H	-4.84535200	0.12215000	-1.56807800
C	-5.98945100	2.75179600	0.33988700
C	-5.98768800	1.31039200	-0.14408100
H	-5.72558500	0.63941000	0.68061800
H	-6.98438300	1.04410400	-0.50007400
H	-6.54949000	2.84020500	1.27493800
N	-4.62080800	3.22140900	0.60781700
N	-3.66480200	1.61548900	-0.75705800
H	-3.79472000	5.09008500	1.10300300
H	-4.34153500	4.75038900	3.62516700
H	-2.31620100	3.42427000	4.08057700
H	-2.45779500	1.64918200	2.32644500
H	-1.46542900	2.35110900	0.02479400
H	-5.24197400	1.75025400	-2.13166600
H	-6.45302700	3.41737100	-0.39657700
C	-2.17996400	-1.07874200	0.95881100
C	-1.51055400	-1.15190400	2.19220800
C	-2.01099700	-1.99076700	3.21022600
C	-3.15087300	-2.71164300	2.93208200
C	-3.80510100	-2.63100400	1.66918700
C	-3.33485100	-1.82223500	0.65884000
H	-1.49816900	-2.07150500	4.16190400
H	-3.56317800	-3.37290100	3.68702400
H	-4.68986500	-3.23605400	1.49970300
H	-3.80059400	-1.77318300	-0.31913900
N	-0.40243300	-0.35298800	2.13467800
N	-0.37144300	0.19362000	0.94695700
N	-1.41333600	-0.22459500	0.23616000
O	-1.56268000	0.13081100	-1.03839000
H	0.18330200	-0.18381400	-1.54218200

M4

0 1

C	-0.47577100	-0.83087400	0.16307700
C	-1.65876400	-2.71233200	0.03271600
C	-3.03068500	-0.63442600	0.34200800
H	-3.03510400	-0.08593200	1.28614100
N	-0.45704100	-3.09190500	-0.23343100
N	0.28996600	-1.91970500	-0.20539400
N	-1.75828600	-1.35821400	0.25304900
C	-2.87864200	-3.57340400	0.17383300
H	-2.61992300	-4.49746800	0.69247500
H	-3.29055700	-3.82386400	-0.81443700
C	-4.17628000	-1.66473300	0.31634200
O	-3.83246400	-2.88052200	0.94959300
C	1.71257200	-2.01384000	-0.15803900
C	2.42167000	-2.01953000	-1.36223800
C	2.35160300	-2.05409000	1.08611100
C	3.81495400	-2.04124900	-1.30087500
C	3.74513000	-2.06815600	1.10168600
C	4.49105900	-2.05201400	-0.07929100
H	4.38487600	-2.04008600	-2.22682300
H	4.26186000	-2.09335000	2.05840500
H	-5.04103300	-1.25273700	0.83868800
C	-0.08925200	0.46847300	0.42425400
O	-1.04597800	1.30964100	1.00467800
C	1.20574800	1.01264500	0.15812600
C	1.59508100	2.27508300	0.45535800
H	1.91574400	0.35622400	-0.33464500
H	0.87229800	2.96199700	0.88979900
C	2.92962400	2.81414600	0.19420600
C	3.13910800	4.20177800	0.26527400
C	4.03781000	2.00486300	-0.12207300
C	4.38621800	4.76168300	0.01041700
H	2.30003900	4.84540000	0.51746400
C	5.28189200	2.56596100	-0.38261800
H	3.92896500	0.92279600	-0.14715600
C	5.46714300	3.94759600	-0.32115700

H	4.51404900	5.83852800	0.06963600
H	6.11932400	1.91862500	-0.62705200
H	6.44202000	4.38097200	-0.51962100
C	5.99684700	-2.01644900	-0.03098200
H	6.43255800	-2.39270700	-0.95894900
H	6.35124800	-0.98998900	0.11223000
H	6.37959000	-2.61357000	0.80021600
C	1.68446400	-1.96247600	-2.67298900
H	0.98013600	-2.79418600	-2.76270900
H	1.10217800	-1.03766800	-2.74504100
H	2.38071500	-1.99902000	-3.51237700
C	1.54930500	-2.05089900	2.35883400
H	0.73089300	-2.77517400	2.30779400
H	2.18024800	-2.29542800	3.21495300
H	1.10493400	-1.06386100	2.52704900
H	-4.46611600	-1.86652300	-0.72692900
C	-3.19601700	0.35757800	-0.82136600
H	-2.28775300	0.95750100	-0.90603800
H	-3.31462900	-0.20954300	-1.75166200
C	-4.38464400	1.25842300	-0.58698900
C	-5.60209900	1.05578400	-1.23989500
C	-4.27931700	2.30185600	0.33913300
C	-6.69539100	1.87870100	-0.97308000
H	-5.69325900	0.25280100	-1.96740000
C	-5.36945400	3.12314800	0.60820400
H	-3.32509400	2.46324300	0.83492900
C	-6.58202700	2.91245700	-0.04741000
H	-7.63507700	1.71218200	-1.49030800
H	-5.27279600	3.93151100	1.32628200
H	-7.43255700	3.55371500	0.16059500
H	-0.81402500	1.41719500	1.93728400

M4Z

0 1

C	-0.72693600	-0.78738000	-0.63812100
C	-0.78571500	-3.01007900	-0.58285700
C	1.51795300	-2.03011500	-0.52240800
H	1.99528600	-1.54957200	-1.37739300

N	-2.01238600	-2.67114800	-0.38683900
N	-1.99441200	-1.27893300	-0.37354200
N	0.06272900	-1.93387700	-0.70082900
C	-0.23369900	-4.39775400	-0.74449200
H	-0.86014600	-4.96737400	-1.43224600
H	-0.21082000	-4.91605800	0.22469400
C	1.88970200	-3.52398200	-0.46820700
O	1.06209700	-4.31498100	-1.29693500
C	-3.11948200	-0.59222300	0.16332500
C	-3.02703700	-0.00337400	1.43236700
C	-4.29535200	-0.54292900	-0.59427700
C	-4.14453100	0.67287200	1.91917800
C	-5.39289200	0.12894900	-0.05776800
C	-5.33432700	0.74670500	1.19244800
H	-4.08748800	1.14393600	2.89783500
H	-6.31189900	0.18084800	-0.63655900
H	2.91877400	-3.65292400	-0.80688300
C	-0.31046700	0.50030900	-0.87376800
O	1.03383900	0.64102200	-1.21406600
C	-1.17094700	1.64943000	-0.87489800
C	-0.82497000	2.96295600	-0.89482700
H	-2.23165900	1.42956500	-0.96692200
H	-1.62624300	3.66915500	-1.10030400
C	0.48339300	3.58095200	-0.63737400
C	1.43073900	3.02060200	0.23827600
C	0.78842400	4.82216700	-1.22188700
C	2.64666600	3.65271000	0.47763500
H	1.19868400	2.08800800	0.74246200
C	2.00236000	5.45552300	-0.98092300
H	0.05892300	5.28512900	-1.88156600
C	2.94376900	4.86893200	-0.13582500
H	3.36227000	3.19454800	1.15462700
H	2.21523100	6.40919800	-1.45406500
H	3.89271600	5.36041200	0.05268600
C	-6.53560200	1.45617800	1.76273200
H	-7.04698300	0.82444600	2.49657500
H	-6.24401200	2.37818800	2.27207900
H	-7.25534600	1.70668600	0.98043000

C	-1.75662800	-0.08267000	2.23479100
H	-1.31789500	-1.08414000	2.17884400
H	-1.00910900	0.62073900	1.84981500
H	-1.94843100	0.15668700	3.28208700
C	-4.34833300	-1.18073000	-1.95607900
H	-4.22798600	-2.26532700	-1.88506400
H	-5.29756100	-0.96340200	-2.44858500
H	-3.53250700	-0.81101800	-2.58550400
H	1.81760200	-3.88664300	0.56924200
C	1.95399600	-1.32410300	0.77551500
H	1.43589400	-0.36466800	0.84257300
H	1.63250000	-1.93401200	1.62818100
C	3.44474700	-1.09153800	0.81477900
C	4.31380400	-2.01418000	1.40216700
C	3.97828900	0.06171400	0.22861800
C	5.68976400	-1.79219200	1.40396700
H	3.90997300	-2.90817800	1.87173100
C	5.35191200	0.28695900	0.23308200
H	3.29852400	0.78131400	-0.22184200
C	6.21180100	-0.64081400	0.81953200
H	6.35252600	-2.51588300	1.86766300
H	5.75116800	1.19037100	-0.21785600
H	7.28261800	-0.46458300	0.82559700
H	1.08397900	1.32656100	-1.89404900

TS4-5°

0 1

C	4.43341900	0.75711300	0.22881800
C	5.00743200	0.60247000	2.32996100
C	4.35285600	-1.57020900	1.21775000
H	3.30869600	-1.63739500	0.88768500
N	5.07191700	1.87435000	2.06103400
N	4.72567200	1.95084300	0.74063300
N	4.66638100	-0.12801200	1.22118500
C	5.08875500	-0.08324700	3.65959300
H	4.80202500	0.61534000	4.44558400
H	6.10393800	-0.45390100	3.85396400
C	4.50796000	-2.09794600	2.65721600

O	4.15681800	-1.14732900	3.64346800
C	4.74695000	3.19809300	0.02942300
C	5.65465700	3.33580000	-1.02659500
C	3.86955600	4.20878500	0.42871800
C	5.65611200	4.55355200	-1.70338300
C	3.91656300	5.40697200	-0.28167200
C	4.79610500	5.59456000	-1.34905500
H	6.35306300	4.69340900	-2.52544800
H	3.24341100	6.21133600	0.00370400
H	3.85541700	-2.96352900	2.78407500
C	3.74823700	0.42846100	-1.06982100
O	4.26023300	-0.43720200	-1.82646200
C	2.48667500	1.01138300	-1.15746900
C	1.50803700	0.43275300	-2.03448700
H	2.19646800	1.76511800	-0.43154100
H	1.91552900	-0.03688200	-2.93226400
H	1.19259000	-0.69637600	-1.31653200
C	0.13953200	0.93812600	-2.14695000
C	-0.57748100	0.81949500	-3.35357400
C	-0.54448100	1.42508900	-1.01779300
C	-1.92182900	1.15333600	-3.42416400
H	-0.05645100	0.47543100	-4.24276300
C	-1.89277700	1.75377000	-1.08708700
H	-0.01619500	1.52276200	-0.07405000
C	-2.59079000	1.60432600	-2.28354800
H	-2.45330500	1.05955000	-4.36628900
H	-2.41119200	2.09979700	-0.19790200
H	-3.64827200	1.84898500	-2.32744000
C	2.91473700	4.01774600	1.57759900
H	2.44882000	3.02691900	1.55341500
H	3.43450200	4.10202300	2.53617000
H	2.12625400	4.77092100	1.54391700
C	4.79693400	6.88716700	-2.12236900
H	5.77279600	7.07609800	-2.57465700
H	4.05834500	6.84910400	-2.92956700
H	4.54064800	7.73246600	-1.48004800
C	6.59975700	2.22659700	-1.41255600
H	6.07202200	1.38446100	-1.87299000

H	7.33744200	2.59520800	-2.12661200
H	7.13348000	1.84253400	-0.53725300
H	5.54827300	-2.41770900	2.81563800
C	5.24523400	-2.37034200	0.25572400
H	6.21402900	-2.55882400	0.72998100
H	5.39921800	-1.77409100	-0.64516900
C	4.56045500	-3.66431300	-0.11725700
C	3.53786400	-3.63518400	-1.07302100
C	4.90136800	-4.87858800	0.47916300
C	2.88141900	-4.80773900	-1.43331900
H	3.28400900	-2.68378800	-1.53979000
C	4.23671600	-6.05211400	0.12468400
H	5.69845000	-4.90828900	1.21858700
C	3.22684500	-6.01864900	-0.83281200
H	2.10161600	-4.77691900	-2.18877900
H	4.51255100	-6.99142100	0.59361100
H	2.71259500	-6.93197600	-1.11463500
C	-1.44202900	-1.93120300	-1.19158000
C	-2.72917700	-1.53572700	-0.95473900
C	-3.13175200	-0.85412400	0.24300900
C	-2.14877600	-0.75295200	1.28035200
C	-0.82931500	-1.04763300	1.07819700
C	-0.42576600	-1.51669300	-0.24186400
H	-3.50251200	-1.81311500	-1.65720600
H	-2.45780700	-0.37575400	2.24511800
C	-4.44369700	-0.34661400	0.39890200
C	-4.96626900	0.06617000	1.66779100
C	-5.29373300	-0.16744800	-0.73953600
C	-6.21066200	0.59728700	1.83067400
H	-4.35330600	-0.09541600	2.54535100
C	-6.53929500	0.37947900	-0.67385000
H	-4.88866400	-0.42508700	-1.70989100
C	-7.07242200	0.79212600	0.64351800
O	0.82597300	-1.60586600	-0.56035800
O	-8.20571100	1.29060200	0.74783900
C	0.19483400	-0.98263200	2.21780700
C	0.87083500	-2.36123400	2.36180600
C	1.26406500	0.09398400	1.96117200

C	-0.46830000	-0.65929300	3.56254700
H	0.12471300	-3.12926600	2.59077600
H	1.39647500	-2.65813300	1.45069300
H	1.58678300	-2.32457100	3.19094800
H	0.81678500	1.09440800	1.99851500
H	2.03523900	0.03539500	2.73990500
H	1.73248400	-0.04322500	0.98676100
H	0.29676100	-0.66649300	4.34480900
H	-0.93167300	0.33242600	3.56182800
H	-1.22930100	-1.40004600	3.82771400
C	-1.10102200	-2.94667100	-2.29586900
C	0.09694500	-2.58250300	-3.18895600
C	-0.77578300	-4.26556100	-1.56498900
C	-2.30025100	-3.18501600	-3.22260700
H	-0.07122100	-1.63486700	-3.70440600
H	1.02752300	-2.51971800	-2.62563000
H	0.21126300	-3.36131600	-3.95111400
H	-1.62768600	-4.59540600	-0.96229200
H	-0.54456300	-5.05000600	-2.29498600
H	0.09042300	-4.14018300	-0.90814800
H	-2.01827900	-3.90565400	-3.99547500
H	-3.16048600	-3.59721300	-2.68740500
H	-2.60827300	-2.25785200	-3.71916100
C	-7.39382200	0.60126100	-1.92266600
C	-7.69561200	2.10154400	-2.09350300
C	-6.68309700	0.12128500	-3.19393700
C	-8.71183500	-0.18720700	-1.80440900
H	-8.23927300	2.49109400	-1.23298700
H	-6.76391200	2.66631000	-2.20931100
H	-8.30007000	2.25665800	-2.99417900
H	-6.45584900	-0.94927200	-3.15380500
H	-7.33662100	0.29253700	-4.05462500
H	-5.74895100	0.66533500	-3.37332700
H	-9.31527100	-0.03209300	-2.70572900
H	-8.50739200	-1.25930000	-1.71388100
H	-9.28459100	0.13642500	-0.93612700
C	-6.75195300	0.99740100	3.20516600
C	-7.06440100	2.50567400	3.22573000

C	-8.02843400	0.19614500	3.52273800
C	-5.73890300	0.71873500	4.32189300
H	-6.15651000	3.08554400	3.02793800
H	-7.81414300	2.75809900	2.47624100
H	-7.44065500	2.79114100	4.21446900
H	-7.80890200	-0.87654800	3.54446700
H	-8.40848600	0.48456800	4.50909800
H	-8.80204100	0.38299100	2.77821900
H	-6.17023800	1.02447300	5.27992000
H	-5.49180900	-0.34581800	4.39174600
H	-4.81017600	1.28163700	4.17964000

TS4-5Z^{O}

0 1

C	5.03337600	0.19799200	0.26693700
C	6.87413700	-0.97257900	0.30930000
C	4.75351100	-2.32977200	0.08425000
H	4.27649700	-2.52365500	1.05018400
N	7.26261700	0.26769100	0.38101200
N	6.10151900	0.98146100	0.34337200
N	5.50560900	-1.06024300	0.22315400
C	7.71583400	-2.21403300	0.31991000
H	8.50063700	-2.12050600	1.07146100
H	8.18060300	-2.35348500	-0.66564400
C	5.79135200	-3.41933100	-0.20501500
O	6.90482000	-3.31419200	0.66085000
C	6.10193000	2.41934600	0.40932900
C	6.20002400	3.13119200	-0.78632300
C	6.00854000	3.02060400	1.66579400
C	6.21204900	4.52217400	-0.69316100
C	6.02544200	4.41255200	1.70387200
C	6.12580900	5.17528600	0.53752600
H	6.28824500	5.10776500	-1.60559600
H	5.95709600	4.91311500	2.66643500
H	5.33658900	-4.39598700	-0.04018200
C	3.60292700	0.67989600	0.11595500
O	3.36468100	1.19816300	-0.99919600
C	2.80651400	0.42207500	1.23000700

C	1.38863500	0.63128400	1.37472200
H	3.28789200	-0.14256500	2.02594700
H	0.84142200	-0.42184800	0.73125600
H	1.02575600	0.33303400	2.36250900
C	0.58584600	1.73011900	0.80426700
C	1.12621600	2.79769700	0.06575300
C	-0.80856800	1.68571200	0.98404000
C	0.29318200	3.75461300	-0.49731800
H	2.19772000	2.85911100	-0.07647800
C	-1.64279900	2.62977600	0.39293100
H	-1.24516700	0.90180500	1.59596800
C	-1.09499200	3.66596200	-0.35731100
H	0.72765700	4.57663400	-1.05824200
H	-2.71817200	2.55426000	0.52612900
H	-1.73916800	4.40938100	-0.81623300
C	5.88008600	2.18859300	2.91303500
H	4.93022100	1.64217900	2.90916400
H	6.68779900	1.45412300	2.98554300
H	5.90616200	2.82066800	3.80134600
C	6.11418000	6.67994000	0.61065300
H	5.08912400	7.05055800	0.71086500
H	6.67798300	7.03536600	1.47642800
H	6.54302000	7.12338000	-0.29014500
C	6.26720900	2.41219000	-2.10555500
H	5.35022500	1.83086700	-2.24878300
H	6.36915900	3.12349100	-2.92621300
H	7.11785200	1.72428100	-2.13623900
H	6.11409200	-3.35417100	-1.25608300
C	3.70220600	-2.24120300	-1.03210000
H	4.18932900	-1.88906900	-1.94774900
H	2.93274000	-1.51347300	-0.75587700
C	3.05184900	-3.58543100	-1.25657600
C	2.17201500	-4.09360700	-0.29591200
C	3.31304100	-4.33482600	-2.40374200
C	1.56300700	-5.33072300	-0.48099100
H	1.93900300	-3.49258100	0.57958600
C	2.70002300	-5.57227600	-2.59325900
H	3.99086200	-3.94271300	-3.15762800

C	1.82621700	-6.07287900	-1.63194000
H	0.87549400	-5.71284900	0.26762000
H	2.90540100	-6.14301900	-3.49318500
H	1.34797600	-7.03563300	-1.78003400
C	-7.31198200	0.02691400	1.02496400
C	-6.02706500	-0.42667400	1.01053900
C	-5.10087700	-0.15295100	-0.04779000
C	-5.58263600	0.65270500	-1.13037400
C	-6.84781600	1.15478300	-1.19423200
C	-7.79610500	0.85589600	-0.09974400
H	-5.69662100	-1.05611300	1.82576100
H	-4.88609000	0.90797600	-1.91791400
C	-3.76357100	-0.62840700	-0.02282900
C	-2.91189800	-0.54922900	-1.17093000
C	-3.19393400	-1.22962100	1.14111400
C	-1.58208700	-0.86234000	-1.13900600
H	-3.35209300	-0.24480700	-2.10910200
C	-1.87672000	-1.59693700	1.24131000
H	-3.82610800	-1.36654800	2.00913300
C	-1.00281500	-1.29889900	0.12211800
O	-8.96151900	1.28733500	-0.12384000
O	0.28248300	-1.40317900	0.24293300
C	-7.31835100	2.02538300	-2.36123700
C	-8.49993800	1.34267500	-3.07590300
C	-7.75103800	3.41099700	-1.84638100
C	-6.20945300	2.24013400	-3.39867600
H	-8.19331400	0.36912600	-3.47294400
H	-9.33614400	1.19769300	-2.39231700
H	-8.83046700	1.96291000	-3.91654600
H	-6.90989500	3.91713200	-1.36088000
H	-8.07989900	4.03103500	-2.68781100
H	-8.56922500	3.32359900	-1.13183700
H	-6.59758000	2.85861700	-4.21362300
H	-5.34464300	2.75763000	-2.96996200
H	-5.86774000	1.29392000	-3.83173100
C	-8.27667600	-0.30230800	2.16660500
C	-8.74959300	0.99793400	2.84369200
C	-9.49048200	-1.08039700	1.62581300

C	-7.61343200	-1.16955800	3.24350200
H	-7.89556200	1.54465400	3.25766400
H	-9.26924100	1.64124300	2.13404900
H	-9.42937900	0.75779300	3.66886100
H	-9.16573100	-2.01891300	1.16405900
H	-10.16872600	-1.32587400	2.45077100
H	-10.03387600	-0.49316000	0.88612600
H	-8.33974700	-1.36968000	4.03695500
H	-7.28207100	-2.13375000	2.84373600
H	-6.75258000	-0.66770100	3.69808600
C	-1.41174400	-2.24097100	2.56484700
C	-1.42489500	-1.18601600	3.68456700
C	-2.39615300	-3.36842100	2.94068600
C	-0.01943800	-2.88932400	2.51870200
H	-0.71226500	-0.38055300	3.48171600
H	-2.41639600	-0.73807900	3.80044800
H	-1.14473700	-1.64924700	4.63681600
H	-2.43727100	-4.12459500	2.15091400
H	-2.05861400	-3.85174000	3.86267100
H	-3.41056300	-3.00368000	3.11635800
H	0.13173200	-3.44504600	3.44989900
H	0.05485900	-3.59095700	1.68302500
H	0.78472900	-2.15852000	2.42158900
C	-0.71585600	-0.78504500	-2.40165200
C	0.33218500	0.32994100	-2.26201200
C	-0.02308800	-2.13840700	-2.64466600
C	-1.55992500	-0.46978900	-3.64480200
H	-0.15218300	1.28425500	-2.02743100
H	1.06783900	0.12367800	-1.48364200
H	0.87250000	0.44136300	-3.20917200
H	-0.76423600	-2.92969600	-2.80151900
H	0.59957700	-2.07459400	-3.54421100
H	0.61337400	-2.42013400	-1.80568700
H	-0.90794100	-0.47136900	-4.52302500
H	-2.34358600	-1.21693600	-3.80660700
H	-2.02657500	0.51877300	-3.58137900

M5$_{SET}$

1 2

C	0.49824000	-0.81974000	-0.32227700
C	1.72133400	-2.62804100	-0.17804200
C	3.04416900	-0.50157600	-0.43914600
H	3.07879400	-0.03883700	-1.42784500
N	0.49966300	-3.03653100	0.00934500
N	-0.25595300	-1.91016500	-0.07929500
N	1.77913700	-1.27244800	-0.36550800
C	2.96602700	-3.46078900	-0.26305500
H	2.75742700	-4.36359400	-0.83857900
H	3.29916800	-3.74756000	0.74373100
C	4.20114200	-1.50004800	-0.26984300
O	3.95279900	-2.71838700	-0.93858100
C	-1.68829000	-1.97857300	0.02418900
C	-2.24361200	-2.09947000	1.30081500
C	-2.44832400	-1.85508100	-1.14176300
C	-3.63414200	-2.06121900	1.39379800
C	-3.83293300	-1.81570200	-0.99157500
C	-4.43983400	-1.90049400	0.26376100
H	-4.09635500	-2.14799800	2.37353800
H	-4.45319600	-1.71318600	-1.87832600
H	5.10528600	-1.06461600	-0.69555900
C	0.03485900	0.50927600	-0.60508400
O	0.88344600	1.20682400	-1.39297300
C	-1.20965200	0.99112800	-0.17295200
C	-1.80803300	2.13233800	-0.64410000
H	-1.71825700	0.39902500	0.57735000
H	-1.31876600	2.72049800	-1.41879800
C	-3.10743000	2.61735100	-0.21534900
C	-3.54732200	3.87391400	-0.66670000
C	-3.95779200	1.86774100	0.62119800
C	-4.78476200	4.37483200	-0.28308700
H	-2.90311200	4.45859700	-1.31748500
C	-5.19276000	2.37096600	1.00029200
H	-3.66389100	0.87972200	0.96444200
C	-5.61076300	3.62653500	0.55335100
H	-5.10641400	5.34837700	-0.63717900
H	-5.83676600	1.78213100	1.64537100

H	-6.57808000	4.01527100	0.85360600
C	-5.93480000	-1.78235800	0.39398200
H	-6.28606100	-2.19168900	1.34293200
H	-6.23157600	-0.72879600	0.35039100
H	-6.44452300	-2.30062000	-0.42123000
C	-1.36987000	-2.23804500	2.51881900
H	-0.77791300	-3.15639300	2.47377200
H	-0.66815300	-1.40086300	2.60134400
H	-1.97815000	-2.26155900	3.42336700
C	-1.80097600	-1.73373300	-2.49498700
H	-0.94360900	-2.40539200	-2.58934000
H	-2.51728100	-1.97675700	-3.28078800
H	-1.44863300	-0.71074800	-2.67052700
H	4.37453000	-1.68671600	0.80121700
C	3.10057800	0.56212400	0.66686300
H	2.25378700	1.24487700	0.57079000
H	3.01493600	0.06032500	1.63678100
C	4.39402300	1.33949300	0.58526600
C	5.40866400	1.14898200	1.52485700
C	4.59858500	2.24388500	-0.46116400
C	6.60797500	1.85075500	1.42316300
H	5.25679100	0.45231200	2.34526900
C	5.79627400	2.94418200	-0.56574300
H	3.81005700	2.40329500	-1.19361100
C	6.80438100	2.74828600	0.37744900
H	7.38665100	1.69670800	2.16295400
H	5.94232800	3.64667900	-1.37977400
H	7.73783200	3.29562300	0.29772900
H	0.71352600	2.15688100	-1.31251900

M5$_{HAT}$

0 2

C	-0.49467800	-0.83893300	0.15577400
C	-1.65324900	-2.71579900	0.02062500
C	-3.04609000	-0.64489800	0.29183800
H	-3.02505600	-0.11074500	1.24312800
N	-0.42323300	-3.09294200	-0.13956800
N	0.30144500	-1.92559500	-0.07522600

N	-1.76679700	-1.36287200	0.18183000
C	-2.87171100	-3.58294900	0.11911400
H	-2.61883200	-4.51163900	0.63187400
H	-3.25892000	-3.82119100	-0.88177000
C	-4.18535000	-1.68078100	0.25672400
O	-3.84098300	-2.90041100	0.88356400
C	1.72985400	-1.99398300	-0.05457400
C	2.40442600	-2.10916700	-1.27229400
C	2.39200100	-1.91058600	1.17446900
C	3.79863500	-2.11601300	-1.24126100
C	3.78541300	-1.91730800	1.15474800
C	4.50288400	-2.00975700	-0.04023500
H	4.34579800	-2.19734400	-2.17708400
H	4.32441800	-1.84721800	2.09660300
H	-5.05092800	-1.27384700	0.78138700
C	-0.15415800	0.52966300	0.45273400
O	-1.03248400	1.27346100	0.98600500
C	1.18319100	1.00642200	0.11582700
C	1.60080800	2.24465800	0.45191400
H	1.84004000	0.35220900	-0.44657500
H	0.89879100	2.89316400	0.97243500
C	2.92370700	2.79732600	0.15477700
C	3.13473600	4.17894800	0.27976700
C	4.00706900	1.99637800	-0.24876900
C	4.37041000	4.74728300	-0.01078900
H	2.30985600	4.80967700	0.60058300
C	5.24041700	2.56514900	-0.54003300
H	3.88725900	0.91740400	-0.31575000
C	5.42921200	3.94323800	-0.42654300
H	4.50726700	5.81972600	0.08810200
H	6.06460000	1.92955700	-0.84977400
H	6.39549200	4.38320300	-0.65119200
C	6.00884700	-1.96388600	-0.02828400
H	6.42565900	-2.35376200	-0.95902800
H	6.35844000	-0.93286400	0.09101700
H	6.41404300	-2.54413400	0.80436500
C	1.63644500	-2.18641300	-2.56420000
H	0.98693600	-3.06621400	-2.58131000

H	0.99460800	-1.30719800	-2.68538400
H	2.31638300	-2.23722300	-3.41575700
C	1.62595700	-1.77839200	2.46266800
H	0.75645200	-2.44156000	2.47714700
H	2.26363000	-2.02093300	3.31426300
H	1.26222700	-0.75272400	2.59052000
H	-4.47316300	-1.87592400	-0.78812700
C	-3.22106200	0.36448000	-0.85301200
H	-2.31522300	0.96771400	-0.92590500
H	-3.35374100	-0.18209600	-1.79352300
C	-4.40216300	1.26480700	-0.58011000
C	-5.62455500	1.09906100	-1.23295000
C	-4.27544500	2.27338200	0.38163300
C	-6.70472000	1.92820300	-0.93308600
H	-5.73064600	0.32136400	-1.98544900
C	-5.35277900	3.10050800	0.68233000
H	-3.31622700	2.39875600	0.87947100
C	-6.57109800	2.92929900	0.02520200
H	-7.64968500	1.79206500	-1.44959300
H	-5.24188500	3.88233300	1.42716900
H	-7.41149300	3.57527400	0.25834200

TS5

0 1

C	2.95186100	1.61898300	-0.24368900
C	4.72443800	2.50217900	0.70515200
C	4.91583700	0.03666400	0.30161300
H	4.21186800	-0.68946200	0.71130100
N	3.92935800	3.50799000	0.50374500
N	2.84116200	2.96137000	-0.11294200
N	4.19939500	1.32962000	0.23042800
C	5.99670200	2.47493400	1.49104000
H	5.98388500	3.28015400	2.22583000
H	6.87259400	2.59270800	0.83780500
C	6.14294600	0.18109000	1.24364700
O	6.03170700	1.23900800	2.17055100
C	1.81727500	3.83007900	-0.62177700
C	1.65881000	3.93525800	-2.00883300

C	1.01133800	4.51411100	0.29275900
C	0.61249300	4.72916700	-2.47182900
C	-0.01678900	5.30213800	-0.22545700
C	-0.24178100	5.40648800	-1.59882500
H	0.46095700	4.82074700	-3.54443700
H	-0.66345700	5.83800500	0.46461800
H	6.25471200	-0.74097000	1.81493800
C	1.95858700	0.66044600	-0.69817500
O	2.41911300	-0.53516700	-1.02086400
C	0.61717300	1.04748800	-0.79809600
C	-0.39690300	0.27120200	-1.33093900
H	0.36415200	2.03511900	-0.43428500
H	-0.14781300	-0.72912300	-1.67522800
C	-1.73950900	0.72303700	-1.53773100
C	-2.68986300	-0.17132500	-2.07732300
C	-2.17840200	2.03711900	-1.23006500
C	-3.99959600	0.22141600	-2.32033700
H	-2.37280500	-1.18337400	-2.31809300
C	-3.48591400	2.41538900	-1.45611700
H	-1.48118200	2.76835700	-0.82925100
C	-4.41406500	1.50566900	-1.99052100
H	-4.70814400	-0.49051900	-2.73027800
H	-3.79777700	3.42757100	-1.21384800
H	-5.44287500	1.80853200	-2.15826400
C	-1.39584600	6.21012900	-2.13684500
H	-1.09174700	6.80655800	-3.00056200
H	-2.19955500	5.54350300	-2.46627300
H	-1.80284900	6.88014600	-1.37722700
C	2.55620400	3.20178200	-2.96930100
H	3.60194000	3.23892600	-2.65059800
H	2.26667900	2.14804600	-3.04605700
H	2.48377600	3.64349100	-3.96416300
C	1.23012400	4.39250700	1.77683400
H	2.17887600	4.84974300	2.07043800
H	0.42241300	4.88320600	2.32128500
H	1.26885600	3.34395100	2.08861700
H	7.04832300	0.32247800	0.63453100
C	5.38725900	-0.41461200	-1.08698600

H	4.52574700	-0.44140400	-1.75653600
H	6.10440800	0.31969900	-1.47131800
C	6.03001700	-1.78168900	-1.00640200
C	7.35211400	-1.97939400	-1.40667200
C	5.29791600	-2.87391200	-0.52406100
C	7.93292900	-3.24491300	-1.34261400
H	7.92922900	-1.13603900	-1.77732600
C	5.87730600	-4.13741700	-0.45934600
H	4.26989000	-2.73800700	-0.19203800
C	7.19614400	-4.32706400	-0.87008900
H	8.96122200	-3.38292400	-1.66152400
H	5.29550700	-4.97603700	-0.08882600
H	7.64656100	-5.31310200	-0.81875100
H	2.34497900	-1.29250600	-0.18019000
C	0.10521600	-2.92480000	0.07282200
C	-1.24206800	-2.64426300	-0.00274900
C	-1.86785500	-1.64037700	0.78299200
C	-1.07017400	-1.03078800	1.78692100
C	0.27412500	-1.29300300	1.93849800
C	0.90873400	-2.11987400	0.95075200
H	-1.84600400	-3.17529500	-0.72794800
H	-1.56340900	-0.37854900	2.49651500
C	-3.24637400	-1.29149500	0.60086500
C	-3.76753800	-0.06596000	1.11139600
C	-4.17090300	-2.18076500	-0.03563100
C	-5.08296200	0.29235000	1.01483800
H	-3.06577500	0.62046100	1.56951800
C	-5.49935700	-1.90582700	-0.17458200
H	-3.79461400	-3.13690300	-0.37863100
C	-6.02806500	-0.62542700	0.34412400
O	2.21039600	-2.08629600	0.79000300
O	-7.23743500	-0.34519200	0.24706400
C	1.07246500	-0.73039500	3.12311300
C	1.85112200	-1.87418800	3.79785900
C	2.05855900	0.35598800	2.66934500
C	0.15024700	-0.10528200	4.17780200
H	1.16197700	-2.64070300	4.16674700
H	2.54919500	-2.33652200	3.09791000

H	2.41657700	-1.48541300	4.65174600
H	1.52308300	1.15514800	2.14563700
H	2.57486000	0.78976700	3.53350600
H	2.80349200	-0.06582200	1.99602200
H	0.75112400	0.20420500	5.03818300
H	-0.36320700	0.78407500	3.79752800
H	-0.60345400	-0.81686000	4.52943800
C	0.74191100	-4.02759900	-0.78671000
C	1.62430500	-3.44760000	-1.90649600
C	1.58802600	-4.95145500	0.10860300
C	-0.33194300	-4.89496900	-1.45913300
H	1.06491500	-2.72540100	-2.51038300
H	2.50865800	-2.94803800	-1.51378300
H	1.95333600	-4.25971900	-2.56489600
H	0.95915500	-5.43387800	0.86420600
H	2.04807200	-5.73626500	-0.50209900
H	2.37785900	-4.39417800	0.61489500
H	0.15451000	-5.73050600	-1.97093400
H	-1.03690700	-5.30764700	-0.73024800
H	-0.89837000	-4.33538300	-2.21117900
C	-6.47189700	-2.90045900	-0.81285700
C	-7.13244200	-2.27455500	-2.05532200
C	-5.76363700	-4.18362300	-1.26353400
C	-7.55692700	-3.30157200	0.20463900
H	-7.62519300	-1.33518000	-1.80557900
H	-6.38515600	-2.09128000	-2.83514800
H	-7.87636200	-2.96676200	-2.46567100
H	-5.31032400	-4.71552500	-0.42050800
H	-6.49494000	-4.85324400	-1.72650800
H	-4.98256900	-3.97894300	-2.00382200
H	-8.23188000	-4.03747300	-0.24649700
H	-7.09944100	-3.75857000	1.08857600
H	-8.13726000	-2.43398400	0.51751500
C	-5.63004900	1.55425400	1.68899300
C	-6.39569600	2.46232500	0.70934000
C	-6.58374600	1.11932000	2.82008900
C	-4.50938400	2.39541300	2.31129600
H	-5.70992200	2.90619100	-0.01654500

H	-7.16706800	1.90560100	0.17868000
H	-6.86762500	3.27965200	1.26684000
H	-6.05843700	0.48419200	3.54103400
H	-6.95652800	2.00260900	3.35090100
H	-7.43281200	0.56619800	2.41612100
H	-4.94286600	3.29523000	2.75923400
H	-3.97816100	1.85307700	3.10074100
H	-3.78153800	2.70854800	1.55421500

TS5$_{HTC}$

0 1

C	1.81073600	1.51696600	-0.45224800
C	3.39033000	2.74264800	0.44896100
C	4.18532300	0.53578500	-0.42246900
H	3.88886500	-0.42592800	-0.00899700
N	2.36007500	3.53322000	0.37973300
N	1.38755300	2.77191500	-0.19777800
N	3.11980300	1.51158600	-0.07861900
C	4.71269100	3.00270300	1.10648700
H	4.54458300	3.53675000	2.04364200
H	5.35675600	3.61010700	0.45564600
C	5.50487300	1.01369500	0.22418100
O	5.30775100	1.76332000	1.40465600
C	0.16353200	3.41862900	-0.58866300
C	-0.00928000	3.73509200	-1.94139200
C	-0.78691400	3.69624500	0.39191600
C	-1.21593400	4.32574900	-2.30580900
C	-1.97256000	4.30562300	-0.02247600
C	-2.20876700	4.61172000	-1.36318000
H	-1.38783000	4.56587600	-3.35207500
H	-2.73255400	4.53715700	0.71898100
H	6.10121400	0.13952800	0.48832100
C	1.06705300	0.32261800	-0.90924100
O	1.77493600	-0.75992900	-0.99037000
H	1.77616800	-1.50262900	0.10949200
C	-0.29488300	0.44647500	-1.22438200
C	-3.99340500	-2.46279300	0.18160400
C	-2.67710400	-2.46199400	0.55081300

C	-1.99139500	-1.29582600	1.00295300
C	-2.77120600	-0.11896800	1.18815600
C	-4.09280600	-0.03009700	0.84265700
C	-4.73212200	-1.18000400	0.16794700
H	-2.13543400	-3.40109200	0.57927400
H	-2.28120900	0.74499600	1.62508900
C	-0.62425300	-1.37198600	1.48868400
C	-0.11533600	-0.42599100	2.45968000
C	0.25102300	-2.33030500	1.03918800
C	1.18819700	-0.33797800	2.84126700
H	-0.84534400	0.20066400	2.96319900
C	1.67241500	-2.22235000	1.20033700
H	-0.11252700	-3.07936200	0.34435700
C	2.18482300	-1.18689900	2.15048600
O	-5.87267400	-1.08902200	-0.32383600
O	3.40084000	-1.02941800	2.32675900
C	-4.96183500	1.16362300	1.25129800
C	-5.50577600	1.93229000	0.03739000
C	-6.14321900	0.64441000	2.09562700
C	-4.17769000	2.15672300	2.11426900
H	-4.68925600	2.44494300	-0.48468700
H	-5.99955900	1.25262100	-0.65774500
H	-6.22208800	2.69184500	0.37332700
H	-5.77613400	0.09560600	2.96963200
H	-6.74214400	1.49066700	2.45033100
H	-6.77969000	-0.01686400	1.50682500
H	-4.82811500	2.99315600	2.39054100
H	-3.80301900	1.69702100	3.03562400
H	-3.32697700	2.56295100	1.55984400
C	-4.76802300	-3.76401700	-0.05696700
C	-5.91618100	-3.83553100	0.96971700
C	-5.35609600	-3.84751200	-1.47449500
C	-3.88517000	-5.00026500	0.14935200
H	-5.52122800	-3.78445400	1.98996200
H	-6.61877700	-3.01386000	0.82421400
H	-6.45465200	-4.78322400	0.85874900
H	-4.55636200	-3.96270300	-2.21277500
H	-6.00835700	-4.72589400	-1.54709800

H	-5.92868900	-2.95115900	-1.71124700
H	-4.48339200	-5.90054800	-0.02346700
H	-3.04462000	-5.02024600	-0.55307300
H	-3.48613000	-5.05260000	1.16756500
C	2.55171600	-3.48381200	0.99087200
C	2.75925900	-4.16222900	2.35379400
C	1.86012500	-4.47523200	0.04528900
C	3.91681300	-3.14690000	0.37316700
H	3.29725600	-3.49894100	3.03673700
H	1.79573900	-4.42348800	2.80463000
H	3.34371800	-5.08140800	2.23524400
H	1.60613500	-4.00040500	-0.91023900
H	2.53890300	-5.30777500	-0.16269000
H	0.94714300	-4.89492300	0.47800500
H	4.47254400	-4.07208300	0.18166900
H	3.78587200	-2.63871800	-0.58626600
H	4.50794200	-2.50906400	1.02965000
C	1.65595800	0.54784900	3.99915300
C	2.41473500	-0.30819200	5.03200000
C	2.57844500	1.65913100	3.47023900
C	0.47937100	1.20235400	4.73363500
H	1.77773700	-1.12221600	5.39448100
H	3.32128500	-0.73534800	4.60423800
H	2.69017400	0.31381200	5.89039500
H	2.02271800	2.32446000	2.79835000
H	2.95084100	2.26474800	4.30497000
H	3.42961400	1.23528000	2.93289400
H	0.86628300	1.79434400	5.56862900
H	-0.09277900	1.87898800	4.09151300
H	-0.20696100	0.45269800	5.14077300
H	-0.73030600	1.43371700	-1.18505400
C	-1.11716100	-0.60510300	-1.58798500
H	-0.68765300	-1.60528700	-1.60799800
C	-2.42555100	-0.44474400	-2.16747800
C	-3.05127000	-1.55858800	-2.76195300
C	-3.10558700	0.79211900	-2.20357900
C	-4.26382700	-1.42993700	-3.42451900
H	-2.55108700	-2.52308200	-2.71861400

C	-4.32061500	0.91213900	-2.85840900
H	-2.67374000	1.66775000	-1.72454200
C	-4.90625600	-0.19535000	-3.47340500
H	-4.71673100	-2.29562600	-3.89572900
H	-4.82534400	1.87269300	-2.88128400
H	-5.85706100	-0.09405500	-3.98647300
C	1.04189900	3.39352000	-2.96281000
H	2.04175000	3.67565600	-2.61898700
H	1.04869100	2.31573000	-3.16356500
H	0.84211100	3.90856200	-3.90312700
C	-3.50936600	5.23462700	-1.79689800
H	-3.98012400	4.63607700	-2.58234500
H	-4.20782700	5.31437800	-0.96180500
H	-3.34458000	6.23618400	-2.20497500
C	-0.53468400	3.31791000	1.82387500
H	-0.24350100	2.26245500	1.89115400
H	0.28513400	3.90695200	2.24821700
H	-1.42592200	3.47548000	2.43474900
H	6.07218900	1.62320800	-0.49494100
C	4.31116300	0.48808000	-1.95744100
H	4.71477000	1.45076200	-2.29476800
H	3.31174300	0.37344900	-2.38117900
C	5.17512000	-0.65125500	-2.44422800
C	6.56722500	-0.54709500	-2.51105900
C	4.56939500	-1.84589100	-2.84298000
C	7.33847800	-1.62219300	-2.94615400
H	7.05497700	0.38523700	-2.23802800
C	5.33726500	-2.92205100	-3.27888100
H	3.48523100	-1.92361100	-2.79871300
C	6.72542100	-2.81357200	-3.32707800
H	8.41846400	-1.52588700	-2.99378700
H	4.85118200	-3.84408500	-3.58207600
H	7.32645800	-3.65096100	-3.66646300

$TS5_{HTC}^{C2'}$

0 1

C	1.86926600	1.86995300	-0.29990800
C	3.50185400	3.00395500	0.60133800

C	4.20417800	0.77937100	-0.31591300
H	3.86446500	-0.16980000	0.11847200
N	2.47462800	3.80660000	0.63821500
N	1.47213900	3.09870100	0.06333100
N	3.18798400	1.81527000	0.00659300
C	4.85670500	3.22085800	1.20303200
H	4.74697500	3.77738700	2.13479200
H	5.49563000	3.79207200	0.51491800
C	5.55232800	1.19944200	0.31573400
O	5.41142200	1.96303900	1.49520100
C	0.20790300	3.75122800	-0.16677300
C	-0.02055600	4.29900000	-1.43275600
C	-0.72180000	3.77790500	0.86966600
C	-1.26889500	4.87377900	-1.65402200
C	-1.94946600	4.38608000	0.60334400
C	-2.24344600	4.92134300	-0.65188300
H	-1.48845800	5.29150200	-2.63323000
H	-2.69641700	4.43132200	1.39114100
H	6.10136500	0.29472200	0.57771300
C	1.06020700	0.71194600	-0.80956000
O	1.68320700	-0.35591500	-0.93427700
H	1.92354000	-1.23605300	0.49267500
C	-0.32725500	0.89677600	-1.14630400
C	-4.21183600	-2.39249300	-0.15184300
C	-2.86990700	-2.50294000	0.05686500
C	-2.09525600	-1.53465900	0.77950200
C	-2.79807000	-0.37583900	1.24718900
C	-4.13635300	-0.18331000	1.08606300
C	-4.92243900	-1.19186800	0.34369800
H	-2.36291800	-3.38739000	-0.30658600
H	-2.22300300	0.38283000	1.76248200
C	-0.74798800	-1.76757900	1.14942500
C	-0.07604800	-0.91362000	2.09667000
C	-0.01057100	-2.92009100	0.71245800
C	1.25651000	-1.02589200	2.37023100
H	-0.68326200	-0.26056300	2.70987700
C	1.33016700	-3.05488700	0.92307700
H	-0.54773700	-3.72128300	0.22374700

C	2.12312800	-1.87011500	1.45952700
O	-6.14313700	-1.04420100	0.16314000
O	3.42970800	-1.94539500	1.65522100
C	-4.86880400	0.99706600	1.73106200
C	-5.56704000	1.87635500	0.67916000
C	-5.91080500	0.46000400	2.73141600
C	-3.90130800	1.89678600	2.50540200
H	-4.82419200	2.42554900	0.08921100
H	-6.18199600	1.27450300	0.00939000
H	-6.20317000	2.61501700	1.18069600
H	-5.42484000	-0.16498500	3.48809500
H	-6.39629000	1.29835000	3.24314200
H	-6.67196800	-0.13152900	2.22325500
H	-4.44795200	2.74935700	2.92236500
H	-3.41440700	1.36838200	3.33264200
H	-3.12461700	2.28731900	1.84048600
C	-5.03168900	-3.52358400	-0.78199000
C	-6.01992500	-4.06140000	0.27141000
C	-5.81162000	-3.04037300	-2.01650800
C	-4.14411100	-4.69098100	-1.22917800
H	-5.47851300	-4.43854500	1.14554000
H	-6.70684800	-3.27788400	0.59335800
H	-6.60051500	-4.88831400	-0.15286200
H	-5.11922800	-2.76449100	-2.81586100
H	-6.45196400	-3.85062200	-2.38423000
H	-6.43156900	-2.17688000	-1.77635300
H	-4.77086600	-5.46310300	-1.68604400
H	-3.40728000	-4.37267200	-1.97525100
H	-3.61146600	-5.14828400	-0.38875400
C	2.06959200	-4.34015700	0.57972000
C	2.69174800	-4.86014400	1.89051800
C	1.14487600	-5.42284900	0.01393200
C	3.18287200	-4.07120400	-0.44367000
H	3.35559400	-4.10012000	2.30650100
H	1.90814800	-5.09763600	2.61824500
H	3.26074200	-5.77511000	1.69066000
H	0.69148000	-5.11535600	-0.93440500
H	1.73123500	-6.32750400	-0.17455100

H	0.34432500	-5.68205800	0.71404400
H	3.72548900	-5.00173400	-0.64728200
H	2.75335200	-3.71691400	-1.38853500
H	3.86966800	-3.32250600	-0.04752100
C	1.89302700	-0.31400800	3.55810700
C	2.59714400	-1.38306600	4.41710300
C	2.91630800	0.71942900	3.06928200
C	0.86811000	0.39420500	4.45194600
H	1.86607100	-2.10340900	4.80016000
H	3.34283100	-1.90782900	3.81970100
H	3.07945700	-0.90052300	5.27440100
H	2.40227200	1.53182300	2.53796800
H	3.44258100	1.16360800	3.92174200
H	3.64475200	0.24223400	2.41225800
H	1.39156800	0.83450600	5.30642600
H	0.34631000	1.20542300	3.93596700
H	0.12036500	-0.30459400	4.84018000
H	-0.76937300	1.87857300	-1.06057500
C	-1.05294300	-0.12548300	-1.67144600
H	-0.56711200	-1.09197800	-1.79782500
C	-2.38788500	0.03630900	-2.23061900
C	-2.82788000	-0.86565400	-3.21149300
C	-3.21435700	1.11875100	-1.88644300
C	-4.02767500	-0.65297000	-3.87965400
H	-2.20318600	-1.71702200	-3.46863500
C	-4.42410400	1.31453400	-2.54013900
H	-2.91047600	1.79996500	-1.09467700
C	-4.82880900	0.43759000	-3.54565100
H	-4.34345500	-1.34165500	-4.65670900
H	-5.05663500	2.15197300	-2.26477200
H	-5.77052400	0.59864300	-4.06005600
C	1.01806000	4.20706200	-2.51822100
H	1.99445900	4.55329000	-2.16652300
H	1.13369400	3.17182600	-2.86019200
H	0.72719900	4.81122300	-3.37795900
C	-3.58627000	5.54273100	-0.93079000
H	-4.01258300	5.14037600	-1.85387600
H	-4.28731900	5.35395900	-0.11559200

H	-3.49247700	6.62547300	-1.05779300
C	-0.41447400	3.10904600	2.17982900
H	-0.29169000	2.02868600	2.02177600
H	0.51772100	3.48554400	2.61261000
H	-1.21949000	3.26169800	2.90046200
H	6.14294600	1.76873800	-0.41767100
C	4.35838100	0.70495900	-1.84443300
H	4.67101600	1.69174800	-2.20574000
H	3.39043600	0.46767700	-2.29087500
C	5.36991600	-0.34605100	-2.22952000
C	5.02285000	-1.69084500	-2.09005300
C	6.64520000	-0.01662600	-2.68930300
C	5.93446300	-2.69473900	-2.39647000
H	4.02677300	-1.93795100	-1.73610300
C	7.56038400	-1.02042400	-3.00337500
H	6.92184900	1.02782100	-2.81037400
C	7.20845900	-2.35944600	-2.85447800
H	5.64968300	-3.73601500	-2.27822300
H	8.54915800	-0.75447400	-3.36332200
H	7.92346500	-3.13954200	-3.09525600

TS5$_{HTC}$$^{C4'}$

0 1

C	0.57801100	0.85968100	-1.51100500
C	2.06359900	2.35671000	-2.12082400
C	3.02207100	0.07922800	-1.73455600
H	3.07806800	-0.33356800	-0.72463700
N	0.92009600	2.97003700	-2.20314400
N	0.00098500	2.03336300	-1.84503500
N	1.90940200	1.05607900	-1.73426200
C	3.42886400	2.92919400	-2.34890500
H	3.45765400	3.95414100	-1.97642400
H	3.66625300	2.92941800	-3.42235800
C	4.33543100	0.82328300	-2.06413600
O	4.35148000	2.16065400	-1.61992100
C	-1.38426200	2.41172000	-1.92832500
C	-2.14707300	1.93470700	-3.00002200
C	-1.86821300	3.32246400	-0.98712300

C	-3.48251400	2.32857600	-3.05078700
C	-3.20475500	3.70358000	-1.09780400
C	-4.03005800	3.19793200	-2.10394700
H	-4.10691300	1.95658700	-3.85889900
H	-3.61266100	4.40649600	-0.37548100
H	5.15363200	0.30329400	-1.56461700
C	0.00105600	-0.39661600	-0.98072400
O	0.85091500	-1.26825600	-0.59959500
H	1.47300400	-1.00740000	0.64209500
C	-1.40452700	-0.64275600	-1.11966100
C	-2.21193400	-2.12260000	2.00618000
C	-0.93828900	-1.63832500	2.04555800
C	-0.61306400	-0.24913500	1.99574100
C	-1.71652000	0.67332400	2.01248700
C	-3.02173600	0.27929400	1.99449000
C	-3.33465500	-1.16275200	1.87563100
H	-0.12996400	-2.34993900	2.13683500
H	-1.49319900	1.73237100	2.06967800
C	0.74518400	0.17505800	2.04769800
C	1.13537600	1.53193100	2.17080600
C	1.82719600	-0.78222900	1.82490000
C	2.43709200	1.95963400	2.05711800
H	0.37056900	2.25746500	2.41863900
C	3.20420900	-0.37655200	1.81463900
H	1.64701700	-1.78120100	2.21327100
C	3.53778300	1.01496500	1.73985000
O	-4.50096900	-1.55612000	1.72283200
O	4.70934500	1.43468400	1.53455800
C	-4.18193500	1.26037100	2.20186300
C	-5.12679900	1.28954900	0.98816600
C	-4.97444200	0.84244800	3.45621100
C	-3.67617800	2.68774100	2.43099200
H	-4.58170200	1.61499500	0.09400600
H	-5.56214400	0.30552900	0.80936700
H	-5.93433800	2.00941600	1.16767900
H	-4.32224300	0.83341100	4.33571500
H	-5.77919200	1.56335200	3.63739900
H	-5.41303100	-0.14783200	3.33239200

H	-4.53030600	3.35233700	2.59417200
H	-3.02261600	2.75502300	3.30707300
H	-3.13022900	3.05480500	1.55791500
C	-2.52655700	-3.59836200	2.27360600
C	-3.36541700	-3.68239900	3.56482500
C	-3.30382000	-4.24750300	1.11781800
C	-1.25192300	-4.42260000	2.48942000
H	-2.83002100	-3.22209900	4.40184500
H	-4.32417200	-3.17745700	3.44018300
H	-3.55213600	-4.73245900	3.81438300
H	-2.65767700	-4.36515400	0.24259500
H	-3.63694800	-5.24668600	1.42164700
H	-4.17082500	-3.65170700	0.83274000
H	-1.52859800	-5.46616800	2.66809300
H	-0.60073900	-4.39686300	1.60843700
H	-0.67881500	-4.07557000	3.35550000
C	4.32758900	-1.40991200	1.94386500
C	5.12992200	-1.08048100	3.21928700
C	3.79216000	-2.84120500	2.07376600
C	5.28677300	-1.37601800	0.73826000
H	5.55230800	-0.07651400	3.14689100
H	4.48546500	-1.13266500	4.10305000
H	5.94466600	-1.80256800	3.34605000
H	3.16999800	-3.11929500	1.21603000
H	4.63568000	-3.53843400	2.10935700
H	3.20900900	-2.98142900	2.99063000
H	6.13297300	-2.04849000	0.92395700
H	4.78677400	-1.73045400	-0.16927900
H	5.66005400	-0.36269200	0.58747900
C	2.78962100	3.43283600	2.27532100
C	3.90828800	3.56389500	3.32823300
C	3.24552400	4.02667600	0.93009700
C	1.60440700	4.26298800	2.78519800
H	3.57395600	3.15044800	4.28613100
H	4.80735700	3.04063700	3.00990500
H	4.13684200	4.62427500	3.48091000
H	2.40499300	4.02030600	0.22263400
H	3.56048700	5.06743400	1.06863300

H	4.06989300	3.44575300	0.51313400
H	1.94182300	5.28923000	2.95936000
H	0.78437200	4.30960000	2.06272900
H	1.21253600	3.87387900	3.73080400
H	-2.05517400	0.20026000	-1.31467000
C	-1.92838500	-1.89609300	-1.13990400
H	-1.25241900	-2.74163700	-1.01926200
C	-3.31166500	-2.17672500	-1.51531600
C	-3.63392400	-3.39973100	-2.12471500
C	-4.33059800	-1.23195800	-1.32818500
C	-4.93039500	-3.66040300	-2.54862600
H	-2.85408700	-4.14267000	-2.27079500
C	-5.62729000	-1.49190000	-1.75321300
H	-4.10503800	-0.30354500	-0.81505700
C	-5.93278500	-2.70631000	-2.36511500
H	-5.16228000	-4.60866500	-3.02283400
H	-6.40335500	-0.74966200	-1.59129600
H	-6.94728700	-2.91386500	-2.68988000
C	-1.55843800	1.02425800	-4.04474600
H	-0.56871600	1.36994900	-4.35797900
H	-1.45218300	0.00126800	-3.66667600
H	-2.20454000	0.99157700	-4.92289000
C	-5.49044500	3.55875800	-2.15151400
H	-5.67329300	4.53340000	-1.69436100
H	-5.86190200	3.57705400	-3.17833600
H	-6.07685000	2.81643500	-1.59822900
C	-0.95852600	3.89499600	0.06385500
H	-0.30888900	3.12085300	0.48437900
H	-0.30511900	4.65920600	-0.36999100
H	-1.52935700	4.35515300	0.87289100
C	2.76413200	-1.02786200	-2.77670900
H	2.95201000	-0.61522400	-3.77436700
H	1.71919700	-1.33640500	-2.73316500
H	4.50916200	0.80055600	-3.15084900
C	3.64626600	-2.22462000	-2.51010100
C	4.94795600	-2.29927700	-3.01185600
C	3.17822900	-3.25087000	-1.68461800
C	5.77566700	-3.36868900	-2.67611400

H	5.31844300	-1.51679600	-3.66970800
C	4.00172000	-4.32399300	-1.35462100
H	2.16790300	-3.18647300	-1.28845200
C	5.30521200	-4.38136000	-1.84354700
H	6.78678100	-3.41174500	-3.06813100
H	3.62770000	-5.11191200	-0.70803400
H	5.94954500	-5.21406900	-1.58082700

TS5$_{HTC}$$^{C5'}$

0 1

C	0.65373300	-1.38758200	-1.30118700
C	0.26409500	-3.54070400	-1.26384300
C	-1.80231800	-2.12247700	-1.50088900
H	-2.13855300	-1.45782300	-0.70262900
N	1.56064600	-3.44344900	-1.30079500
N	1.79828700	-2.09768200	-1.35697900
N	-0.34362100	-2.31484100	-1.32265400
C	-0.57141500	-4.77213300	-1.08191300
H	-0.06993300	-5.44843500	-0.38690400
H	-0.72260400	-5.28676700	-2.04038000
C	-2.48941600	-3.50056900	-1.37489300
O	-1.80356400	-4.38526800	-0.51437100
C	3.08822700	-1.61196200	-1.77461400
C	3.18616800	-1.01583900	-3.04086200
C	4.17519500	-1.73247900	-0.90680300
C	4.41943100	-0.47679100	-3.39759500
C	5.38700400	-1.17152900	-1.31338000
C	5.52373100	-0.52823700	-2.54322100
H	4.51985400	-0.00090900	-4.37003600
H	6.23971800	-1.23425000	-0.64232900
H	-3.49259200	-3.35804100	-0.97010500
C	0.49366300	0.05740400	-1.20318400
O	-0.67698400	0.52393200	-1.41451300
H	-1.28997600	0.57387700	-0.21571400
C	1.65562200	0.91440100	-1.03493100
C	2.02625300	0.30525800	2.56149800
C	0.93791300	0.82446900	1.91321900
C	-0.19372600	0.05240600	1.52579700

C	-0.14897100	-1.34046800	1.83766400
C	0.86571200	-1.92972400	2.54350300
C	2.03832700	-1.12212000	2.92418200
H	0.93255500	1.88298400	1.68462500
H	-0.98406700	-1.96130600	1.53067600
C	-1.44204700	0.67375000	1.03364100
C	-2.70496500	-0.01048700	1.34938500
C	-1.56525900	2.13727000	0.93433300
C	-3.92006600	0.57391700	1.31378200
H	-2.64244200	-1.04792600	1.65033900
C	-2.74690100	2.78395300	0.81765100
H	-0.64060800	2.68970600	0.81104100
C	-4.01075000	2.00099900	0.90149800
O	3.01138600	-1.63650800	3.51457500
O	-5.10145000	2.51884600	0.64962600
C	0.78361300	-3.38765100	3.00921300
C	1.91009000	-4.22578000	2.38418100
C	0.89184000	-3.44019200	4.54551700
C	-0.55185800	-4.03469100	2.62763100
H	1.82606900	-4.22467600	1.29035800
H	2.88589300	-3.82373900	2.66061700
H	1.84019900	-5.26277600	2.73229800
H	0.08005500	-2.86551000	5.00419200
H	0.80390900	-4.47901000	4.88316800
H	1.84463000	-3.03591000	4.88499000
H	-0.56567300	-5.06840500	2.98732900
H	-1.40078600	-3.51100200	3.08221900
H	-0.71140800	-4.05662200	1.54577700
C	3.22990600	1.17442400	2.93589800
C	3.44468100	1.15619100	4.46056800
C	4.48813800	0.63778200	2.22853800
C	3.03927200	2.63544400	2.51153600
H	2.56722000	1.56683900	4.97164500
H	3.61749300	0.14115400	4.81758700
H	4.30900600	1.77792000	4.72023600
H	4.35318800	0.65002200	1.13886700
H	5.35017400	1.27216100	2.46561500
H	4.69806900	-0.38413200	2.54610400

H	3.93198000	3.20784000	2.78309400
H	2.90134400	2.73219100	1.42979700
H	2.18138100	3.09477100	3.01491700
C	-2.83097700	4.29030400	0.55961500
C	-3.67295600	4.96939600	1.65447100
C	-1.44155900	4.93881800	0.56838500
C	-3.45987900	4.54811000	-0.82226800
H	-4.68774100	4.57142100	1.67559400
H	-3.21429400	4.81965100	2.63739000
H	-3.72372600	6.04693000	1.46459800
H	-0.80983900	4.55124800	-0.23823800
H	-1.54799400	6.01708400	0.41750900
H	-0.92355500	4.78120100	1.52046700
H	-3.45078700	5.62220200	-1.03810800
H	-2.88468700	4.03942300	-1.60433200
H	-4.49015800	4.19410400	-0.85606200
C	-5.19524900	-0.17963300	1.70251800
C	-5.93905000	0.59568700	2.80539600
C	-6.10741200	-0.35349900	0.47749400
C	-4.87834100	-1.57719300	2.24966100
H	-5.29889300	0.72152000	3.68499000
H	-6.25188300	1.57914300	2.45450900
H	-6.82840100	0.03433500	3.11069500
H	-5.59387700	-0.92375600	-0.30530300
H	-7.01119900	-0.90132300	0.76815000
H	-6.39600000	0.61228700	0.06207100
H	-5.81234000	-2.06454400	2.54434600
H	-4.39978800	-2.21271000	1.49599000
H	-4.22777400	-1.53383600	3.12958600
H	2.54617900	0.47469200	-0.59682100
C	1.63285100	2.22126800	-1.35854800
H	0.70787000	2.64552200	-1.74738100
C	2.77648100	3.12144900	-1.18760500
C	2.56489400	4.50264800	-1.08123500
C	4.09053200	2.63293800	-1.09247600
C	3.62794000	5.36766900	-0.84052500
H	1.55529200	4.89477700	-1.16961600
C	5.15026000	3.49743000	-0.85196200

H	4.28215400	1.57050300	-1.22129000
C	4.92237700	4.86780600	-0.71719100
H	3.44491500	6.43323400	-0.74658000
H	6.15842900	3.10206500	-0.77400800
H	5.75100100	5.54230000	-0.52767400
C	2.01991000	-0.93193900	-3.99159100
H	1.40004400	-1.83290400	-3.95813800
H	1.38305200	-0.07072500	-3.75992900
H	2.38218500	-0.80884700	-5.01327800
C	6.82350700	0.11574700	-2.94676600
H	7.16626800	-0.26496500	-3.91285300
H	6.69597500	1.19822600	-3.04931900
H	7.60449200	-0.06768500	-2.20674100
C	4.03491900	-2.42376700	0.41594600
H	3.15087300	-2.06821000	0.95193300
H	3.91329500	-3.50234600	0.27624600
H	4.91034000	-2.24847200	1.04232900
H	-2.57751500	-3.95968400	-2.36994700
C	-2.11915800	-1.50326800	-2.87557500
H	-1.38680600	-0.72753100	-3.09563000
H	-2.02507400	-2.28115200	-3.64108800
C	-3.50583400	-0.89833600	-2.90143500
C	-4.58354700	-1.58747400	-3.46005500
C	-3.72318700	0.37783500	-2.36471600
C	-5.85175200	-1.00886000	-3.50114200
H	-4.42645700	-2.57618100	-3.88500100
C	-4.98645300	0.95794800	-2.40973300
H	-2.89060800	0.92321000	-1.92702900
C	-6.05354700	0.26682000	-2.98282900
H	-6.67812900	-1.55337600	-3.94707900
H	-5.13942100	1.94118000	-1.97392000
H	-7.03995100	0.71859600	-3.01649400

M5

0 1

C	2.55407900	1.55454000	-0.41440400
C	3.91652100	2.88528400	0.64270900
C	4.88216800	0.62794900	0.11763800

H	4.43339600	-0.29390800	0.49049100
N	2.84841000	3.60162200	0.41944700
N	2.01644100	2.76907400	-0.25300500
N	3.79692500	1.63653400	0.10389600
C	5.10088700	3.21147200	1.49894300
H	4.78902500	3.88114500	2.30073700
H	5.89402500	3.69188100	0.91054300
C	6.00444900	1.12122400	1.06619300
O	5.54392900	2.00098600	2.06952600
C	0.79916700	3.29982000	-0.82086800
C	0.76152400	3.49861700	-2.20351300
C	-0.27647400	3.56126400	0.03050600
C	-0.43456900	3.96993600	-2.74079200
C	-1.44158200	4.05005100	-0.55862400
C	-1.54425300	4.24183600	-1.93852100
H	-0.49935600	4.12684300	-3.81415600
H	-2.29489000	4.27947900	0.07352000
H	6.44545100	0.25840400	1.56563800
C	1.90807100	0.28123100	-0.92429800
O	2.66371300	-0.67339500	-1.11286100
C	0.49092000	0.31529700	-1.13942600
C	-0.13689500	-0.67832400	-1.82277100
H	-0.07058500	1.16128900	-0.77378500
H	0.46936700	-1.51016800	-2.18135100
C	-1.53690400	-0.68170900	-2.19188600
C	-2.02104300	-1.72399000	-3.00068100
C	-2.42503500	0.32981100	-1.78089100
C	-3.34589800	-1.74191600	-3.41360800
H	-1.33909700	-2.51026200	-3.31533300
C	-3.74709100	0.31201000	-2.19791900
H	-2.08817800	1.13257000	-1.13101300
C	-4.20629200	-0.71611100	-3.01876300
H	-3.70888000	-2.54721100	-4.04406200
H	-4.43170100	1.08352500	-1.86234600
H	-5.24142000	-0.72025400	-3.34310600
C	-2.84173400	4.69204900	-2.55384700
H	-3.44189200	5.26358500	-1.84270400
H	-2.66803100	5.30470000	-3.44109900

H	-3.43017900	3.82076200	-2.86160400
C	1.93997800	3.17985100	-3.08561600
H	2.88031500	3.53129300	-2.65109900
H	2.02343300	2.09912900	-3.24963800
H	1.81889500	3.65112100	-4.06154800
C	-0.20482100	3.23133900	1.49632400
H	0.67871900	3.66723400	1.97118700
H	-1.09378800	3.58989200	2.01812100
H	-0.15394000	2.14098000	1.62656300
H	6.78556500	1.62057600	0.47522000
C	5.43283400	0.41260900	-1.30152800
H	4.59813800	0.26918400	-1.98928600
H	5.98250600	1.30877000	-1.60865200
C	6.32572000	-0.80619700	-1.32332300
C	7.71495100	-0.70230800	-1.24249400
C	5.74045500	-2.07491900	-1.38492200
C	8.50761800	-1.84896400	-1.21722300
H	8.18196700	0.27897800	-1.20798600
C	6.52975400	-3.22002100	-1.35961000
H	4.65844600	-2.15212600	-1.46671800
C	7.91697000	-3.10841600	-1.27276600
H	9.58722100	-1.75610000	-1.15726500
H	6.06294400	-4.19870800	-1.41411200
H	8.53471000	-4.00026100	-1.25488300
H	2.92067700	-2.04664100	0.61075600
C	0.48307400	-2.68173100	0.64858600
C	-0.86842900	-2.36630500	0.48749600
C	-1.49764600	-1.31828200	1.17956800
C	-0.72124900	-0.62824000	2.12442000
C	0.64266200	-0.85875300	2.29729700
C	1.24476500	-1.83509200	1.47372300
H	-1.46757000	-2.94168500	-0.20702500
H	-1.21872200	0.08950600	2.76563200
C	-2.91116800	-0.98844400	0.95732500
C	-3.41399100	0.29244500	1.25567800
C	-3.83174300	-1.93517400	0.47175700
C	-4.72899200	0.66033300	1.04325600
H	-2.71317100	1.03018100	1.63863800

C	-5.16397000	-1.64760000	0.23909400
H	-3.47878000	-2.95065100	0.31987500
C	-5.65862300	-0.29198300	0.44988800
O	2.61961000	-1.98720600	1.52900500
O	-6.84881500	0.03855100	0.15162900
C	1.42806900	-0.17258200	3.42972100
C	2.06629600	-1.24792200	4.33002400
C	2.52554600	0.74347100	2.87040400
C	0.52061400	0.68050400	4.32704300
H	1.29330600	-1.90423400	4.74211300
H	2.78439800	-1.85845100	3.78162700
H	2.58611000	-0.76752700	5.16609200
H	2.07822600	1.55495800	2.28155600
H	3.09439800	1.20235700	3.68639900
H	3.21910600	0.17768900	2.24779100
H	1.12531000	1.11457800	5.12909400
H	0.05137300	1.50721700	3.78459800
H	-0.26914700	0.07968600	4.78766300
C	1.06379800	-3.98512600	0.05550100
C	2.30140900	-3.80407900	-0.84967400
C	1.45400800	-4.89288100	1.23756100
C	0.01217100	-4.72676800	-0.78239800
H	2.17236100	-2.99619400	-1.57408900
H	3.21587000	-3.61968500	-0.27639300
H	2.47949300	-4.73347700	-1.39994300
H	0.57739000	-5.12164000	1.85091700
H	1.87041800	-5.83547900	0.86521900
H	2.20351300	-4.40580600	1.86663200
H	0.43966400	-5.66631200	-1.14386100
H	-0.87928400	-4.96891000	-0.19755500
H	-0.29708900	-4.14069600	-1.65533500
C	-6.15907900	-2.74580900	-0.15965200
C	-6.81970000	-2.44377100	-1.51624900
C	-5.49449200	-4.12253300	-0.27701000
C	-7.25046300	-2.84516300	0.92287200
H	-7.22410800	-1.43127300	-1.52003300
H	-6.08682000	-2.54611800	-2.32277700
H	-7.62898700	-3.15967700	-1.70667100

H	-5.05357200	-4.44725300	0.67149400
H	-6.24695600	-4.86349600	-0.56846400
H	-4.70850400	-4.12566000	-1.04102600
H	-7.97657600	-3.62448500	0.66108500
H	-6.80386500	-3.10632000	1.88874000
H	-7.76876100	-1.89050900	1.02011400
C	-5.22614400	2.06973900	1.38304800
C	-5.59917600	2.81147400	0.08769200
C	-6.45283700	1.99612600	2.31048400
C	-4.16255600	2.90166500	2.10696600
H	-4.70165400	2.96749700	-0.52596400
H	-6.32524300	2.22627700	-0.47977900
H	-6.02701000	3.79666800	0.31234500
H	-6.18217600	1.50947600	3.25412500
H	-6.81200800	3.00665600	2.54082100
H	-7.24965700	1.42567700	1.83480800
H	-4.56545100	3.89406700	2.33686400
H	-3.84983700	2.43354600	3.04710500
H	-3.27254500	3.03671300	1.48513000

M05

1 1

C	0.52530600	-0.91734000	-0.18532500
C	1.70788400	-2.73296900	-0.02345600
C	3.06642700	-0.61391400	-0.22649400
H	3.09800600	-0.19275100	-1.23384500
N	0.46361800	-3.12922400	0.02413000
N	-0.25783000	-1.98815800	-0.07094300
N	1.78954100	-1.37257000	-0.14224000
C	2.95032600	-3.57307300	-0.03851100
H	2.76201200	-4.48278800	-0.60971800
H	3.23451300	-3.84498600	0.98701900
C	4.20749800	-1.62025700	-0.01498600
O	3.96910900	-2.84336800	-0.67998900
C	-1.69958600	-2.01763800	-0.08838700
C	-2.35356600	-2.26804600	1.12114800
C	-2.35830500	-1.72910600	-1.28682700
C	-3.74491800	-2.18592300	1.11195700

C	-3.74894100	-1.65753300	-1.23535900
C	-4.45465700	-1.86719800	-0.04869400
H	-4.28466600	-2.36752800	2.03721700
H	-4.29396200	-1.42874500	-2.14738700
H	5.12582800	-1.19447900	-0.42004700
C	0.10788300	0.50456700	-0.50895400
O	0.87264600	1.13077100	-1.22065600
C	-1.16438300	0.97173000	0.02095800
C	-1.69585100	2.12084600	-0.44506600
H	-1.66544000	0.36656500	0.76733800
H	-1.11617000	2.68372100	-1.17612200
C	-2.98819400	2.67492000	-0.06215100
C	-3.28421800	4.00055500	-0.41135700
C	-3.94740200	1.91879700	0.63287300
C	-4.49803600	4.57144900	-0.04733400
H	-2.55021600	4.58267500	-0.96122500
C	-5.16135200	2.48878400	0.98678700
H	-3.75282700	0.87697900	0.87344600
C	-5.43623000	3.81686700	0.65292800
H	-4.71405200	5.60067000	-0.31257600
H	-5.90001800	1.89929100	1.51960100
H	-6.38727800	4.25837000	0.93236400
C	-5.95245500	-1.72097300	-0.02250300
H	-6.37652600	-2.13477100	0.89387000
H	-6.22750800	-0.66235400	-0.07676700
H	-6.41119200	-2.22240900	-0.87794700
C	-1.59068200	-2.59106900	2.37844300
H	-1.08862600	-3.55858400	2.29220800
H	-0.82185300	-1.83953400	2.58641900
H	-2.26798800	-2.62879100	3.23193900
C	-1.61583700	-1.47499100	-2.57293600
H	-0.71726600	-2.09172900	-2.65566200
H	-2.25957200	-1.69786400	-3.42489700
H	-1.31726600	-0.42373100	-2.65748500
H	4.34690600	-1.79756500	1.06244300
C	3.13681300	0.49976600	0.82675700
H	2.23294400	1.11065300	0.79069800
H	3.19554600	0.04545800	1.82131700

C	4.33888400	1.37673500	0.55775000
C	5.49837600	1.28466000	1.32818000
C	4.29905200	2.27629300	-0.51202700
C	6.60407700	2.08111500	1.03438700
H	5.53540300	0.59197000	2.16503200
C	5.40216800	3.07050500	-0.80685900
H	3.39210800	2.35405400	-1.10827600
C	6.55810800	2.97301600	-0.03359900
H	7.50010300	2.00534100	1.64178900
H	5.35954700	3.76749400	-1.63748100
H	7.41903300	3.59235000	-0.26277600

TS0^{OAr-}

-1 1

C	0.02990100	-2.02803100	-0.27386300
C	0.44379500	-2.61113500	-1.50898300
H	0.96338100	-3.56123400	-1.38929200
H	-0.29266700	-2.62428100	-2.30832900
H	1.49456200	-1.84166100	-1.94648400
C	0.90004300	-2.23339600	0.92650100
C	2.26165200	-2.52853600	0.78827500
C	0.35559200	-2.11944300	2.21019500
C	3.05809800	-2.70929000	1.91508700
H	2.70347100	-2.58495600	-0.20197800
C	1.15065100	-2.31350700	3.33407700
H	-0.69776700	-1.88206000	2.30975200
C	2.50530400	-2.60802600	3.18970000
H	4.11545700	-2.92345400	1.79580200
H	0.71388500	-2.23143200	4.32438900
H	3.12805400	-2.75342200	4.06699800
N	-1.00739700	-1.23860400	-0.04187500
S	-2.09102200	-0.99485800	-1.24915800
O	-1.50014300	-0.22317900	-2.35464100
O	-2.81368300	-2.22338100	-1.61173500
C	-3.24678100	0.07307200	-0.42079900
C	-2.90782400	1.40867000	-0.21154800
C	-4.47067000	-0.43280300	-0.00725100
C	-3.81262000	2.23923900	0.43360200

H	-1.95030500	1.78523200	-0.55791100
C	-5.36933900	0.41515000	0.63734900
H	-4.71033900	-1.47358700	-0.19615700
C	-5.05397300	1.75471800	0.86810100
H	-3.55904500	3.28214400	0.60333500
H	-6.33029800	0.02891900	0.96474500
C	-6.02120700	2.67351200	1.56763600
H	-5.58091700	3.07531600	2.48496600
H	-6.28020800	3.52476900	0.93134800
H	-6.94285500	2.15139400	1.83172700
O	2.57859800	-1.30846100	-2.27280900
C	2.75204400	-0.17932600	-1.64702200
C	4.06539900	0.24823300	-1.30842600
C	1.66231800	0.65471500	-1.26992200
C	4.28166500	1.42896900	-0.63304200
H	4.89628000	-0.38651900	-1.59757900
C	1.88007000	1.83115400	-0.58350600
H	0.65422700	0.35983800	-1.54782800
C	3.18552800	2.21676400	-0.26507100
H	5.28159400	1.75594200	-0.37401600
H	1.05416100	2.46920200	-0.29181800
N	3.40747600	3.44822600	0.44374300
O	2.43610500	4.12698100	0.76338000
O	4.56128000	3.77754000	0.70395700

TS0^{DBU}

0 1

C	0.42959200	1.09723100	-1.01462400
C	-0.45971200	0.42180300	-1.90122400
H	-1.18960800	1.07106700	-2.38186300
H	-0.01100400	-0.30486800	-2.57341100
H	-1.20188000	-0.32885900	-1.01059000
C	-0.06681400	2.33450600	-0.33584300
C	-1.43342500	2.51121500	-0.09089400
C	0.83443300	3.31339800	0.09431800
C	-1.88967100	3.64902100	0.56862100
H	-2.13776000	1.74254300	-0.39587400
C	0.37513400	4.45858300	0.73560600

H	1.89335500	3.16484900	-0.08730900
C	-0.98753400	4.62865400	0.97656400
H	-2.95066100	3.76972200	0.76342900
H	1.08160500	5.21908300	1.05270400
H	-1.34361800	5.52005500	1.48320500
N	1.62726600	0.71620200	-0.59891000
S	2.22380400	-0.70142100	-1.17874500
O	1.43402800	-1.83003600	-0.66189800
O	2.47803900	-0.67254000	-2.62694400
C	3.80606300	-0.73763200	-0.36976500
C	3.86838400	-1.00779500	0.99615300
C	4.95619500	-0.51528300	-1.11355700
C	5.10782500	-1.04424300	1.61839300
H	2.95574300	-1.18984200	1.55524500
C	6.19318800	-0.55764100	-0.47370900
H	4.87615300	-0.31670900	-2.17677200
C	6.28646100	-0.82073400	0.89355800
H	5.16887300	-1.25216600	2.68315700
H	7.09924700	-0.38403400	-1.04702400
C	7.62308400	-0.88424400	1.58511100
H	7.85716900	-1.91234900	1.87847200
H	8.42390600	-0.52857300	0.93408400
H	7.62224900	-0.27794400	2.49486400
C	-6.25941500	-0.53010900	-0.92611700
C	-4.91030500	-0.04733200	-1.46294300
C	-3.80473900	-1.11882000	-1.46425900
C	-6.24900500	-0.97227800	0.53702800
C	-3.12592100	-1.25539900	-0.12449900
C	-5.24644800	-2.09300500	0.81533400
H	-6.59606900	-1.37385300	-1.54354200
H	-5.04796600	0.29456500	-2.49294500
H	-4.21817000	-2.08618200	-1.77344600
H	-6.01789800	-0.12473800	1.19470300
H	-5.48386700	-2.59174000	1.75791900
C	-1.16751100	-0.99205800	1.22352200
H	-0.31615600	-0.30800300	1.15381500
C	-3.33135400	-1.51922300	2.30431100
C	-2.12412400	-0.59529100	2.34036900

H	-2.44352900	0.44130400	2.18389800
H	-1.64197000	-0.65859000	3.31813000
H	-4.13154200	-1.13203300	2.94342200
N	-3.86485100	-1.62461900	0.94162500
N	-1.86194200	-0.95973000	-0.05555000
H	-5.30439500	-2.85893100	0.03410300
H	-7.24896100	-1.32565400	0.80938900
H	-6.99959200	0.26576200	-1.05649400
H	-4.56774100	0.82321000	-0.88824100
H	-3.02665400	-0.86184300	-2.18576700
H	-0.75945400	-1.99530300	1.39564000
H	-3.06678600	-2.51950400	2.66889200

TS0^{DQH-}

-1 1

C	-4.34153200	1.47440000	0.41666600
C	-3.34099700	1.99432300	1.29775700
H	-3.41775800	3.07308200	1.43798100
H	-3.18481000	1.45217000	2.22663900
H	-2.16717500	1.96836800	0.60163800
C	-4.76942700	2.33072800	-0.73469300
C	-3.93858800	3.34534300	-1.22544300
C	-6.01524900	2.12078400	-1.33686300
C	-4.35302800	4.13582700	-2.29395200
H	-2.95156000	3.48734800	-0.79581400
C	-6.43051300	2.91874900	-2.39750400
H	-6.65150000	1.32994500	-0.95499700
C	-5.59992700	3.92908300	-2.87950900
H	-3.69463700	4.91063400	-2.67401700
H	-7.40364100	2.75299600	-2.84915300
H	-5.92139700	4.54903600	-3.71072100
N	-4.91248200	0.28280200	0.42331300
S	-4.66848100	-0.72171400	1.69890400
O	-3.24997300	-1.01313900	1.94467800
O	-5.47090100	-0.30224200	2.85808600
C	-5.39641900	-2.21257400	1.05810300
C	-4.69217800	-2.94452300	0.10480400
C	-6.63149400	-2.63837500	1.52618200

C	-5.24778800	-4.11596100	-0.39045600
H	-3.72053800	-2.59645700	-0.23306800
C	-7.17341400	-3.81916800	1.02204200
H	-7.14808700	-2.05394300	2.28002500
C	-6.49550000	-4.56824200	0.05882700
H	-4.70605100	-4.69548200	-1.13299500
H	-8.13640800	-4.16534400	1.38650700
C	-7.09489000	-5.83064200	-0.50405000
H	-6.33441000	-6.60541700	-0.62943600
H	-7.88135300	-6.21935800	0.14618100
H	-7.53628300	-5.64256200	-1.48810900
C	0.33018400	0.59000600	-0.92744900
C	1.60995000	0.04152800	-0.89718300
C	2.62812800	0.54509500	-0.08445100
C	2.33491100	1.65698600	0.70704700
C	1.07749100	2.25767100	0.72439600
C	0.03516200	1.70609200	-0.08340200
H	1.85181900	-0.79136600	-1.54891500
H	3.12295700	2.04435600	1.34376600
C	3.97679200	-0.07095000	-0.08054700
C	5.13091400	0.70982600	0.02537200
C	4.13466500	-1.45296100	-0.18824000
C	6.41370400	0.16356600	0.02442400
H	5.00862700	1.78490300	0.08609200
C	5.38733500	-2.06991200	-0.20159700
H	3.23852500	-2.05985200	-0.23750400
C	6.52049400	-1.23738500	-0.09654800
O	-1.16675500	2.27034000	-0.10490800
O	7.79361400	-1.75147100	-0.10024000
C	0.82764600	3.50247600	1.59135200
C	0.37838000	4.67956700	0.70682400
C	-0.24502500	3.21206700	2.65383700
C	2.09182600	3.95385300	2.33621500
H	1.16318700	4.93187600	-0.01486300
H	-0.53021300	4.42492100	0.16161700
H	0.19107300	5.56424400	1.32715100
H	0.06149100	2.37406600	3.28892500
H	-0.38790700	4.09073800	3.29440100

H	-1.19658700	2.96312600	2.18782400
H	1.86170200	4.85452200	2.91450600
H	2.44941500	3.19126600	3.03576100
H	2.90694000	4.19763700	1.64677800
C	-0.73651300	0.01512300	-1.87342800
C	-1.88548600	-0.58796800	-1.05171000
C	-1.27516200	1.11260300	-2.80968600
C	-0.18220500	-1.10438600	-2.76569700
H	-1.52598000	-1.43316000	-0.45348000
H	-2.31619800	0.13845200	-0.36707700
H	-2.68202400	-0.94857100	-1.71560200
H	-0.46365200	1.51186300	-3.42893100
H	-2.03515400	0.69135100	-3.47859400
H	-1.71713900	1.92955500	-2.24011100
H	-0.98224100	-1.46426600	-3.42084000
H	0.63887500	-0.75420400	-3.40015600
H	0.17441300	-1.95734800	-2.17893100
C	5.51059400	-3.60137300	-0.31122600
C	6.20556200	-4.18626900	0.93804900
C	4.13351600	-4.27750600	-0.38201800
C	6.24583800	-3.99845300	-1.61011900
H	7.20857300	-3.79630800	1.12946300
H	5.60694600	-3.96509500	1.82636600
H	6.28725300	-5.27363900	0.84170300
H	3.56525400	-3.94930700	-1.25715300
H	4.27201900	-5.36011400	-0.45819900
H	3.53955500	-4.07677400	0.51407600
H	6.34681800	-5.08711100	-1.66215700
H	5.66562200	-3.66657400	-2.47595100
H	7.24638600	-3.57158600	-1.72431000
C	7.65978000	1.05699500	0.13759300
C	8.46439300	0.69273100	1.39929000
C	8.54261300	0.91185400	-1.11586900
C	7.28259600	2.54030400	0.25445200
H	7.84888800	0.83112000	2.29429500
H	8.81222300	-0.33978700	1.37252600
H	9.33567100	1.35203800	1.48219000
H	7.97937700	1.19576300	-2.01079300

H	9.40806800	1.57902100	-1.03514600
H	8.90315300	-0.10895600	-1.24094200
H	8.19828700	3.13322900	0.34027900
H	6.73808800	2.89295200	-0.62708700
H	6.67188300	2.73788000	1.14111000
H	7.76196600	-2.70158400	-0.23887600

M0^{OAr-}

-1 1

C	0.16991000	-1.17942900	1.43168300
C	0.36247100	-2.49658600	1.65487600
H	1.29313300	-2.85912100	2.07172700
H	-0.41322400	-3.22523800	1.45652200
H	-0.18916600	-2.35083000	-1.50983700
C	1.25765600	-0.18914200	1.72324100
C	2.60068400	-0.57151800	1.82025900
C	0.95299400	1.17293900	1.83815000
C	3.60409900	0.37206800	2.02318200
H	2.87730000	-1.61113200	1.67925400
C	1.95437600	2.11828000	2.03789600
H	-0.08191900	1.48035400	1.74090200
C	3.28768800	1.72477900	2.12749200
H	4.64111500	0.05124300	2.06428100
H	1.69319100	3.16998000	2.11200300
H	4.07228400	2.46387000	2.25800000
N	-0.98319500	-0.55687500	0.94248400
S	-2.09580200	-1.42087700	0.24624500
O	-1.70595900	-1.90328900	-1.12548800
O	-2.72602600	-2.48695600	1.05509200
C	-3.35387700	-0.19513300	-0.05079100
C	-3.28274700	0.62813600	-1.17155600
C	-4.39588700	-0.07102800	0.85872400
C	-4.26230000	1.59361500	-1.36785800
H	-2.47657300	0.49719500	-1.88610400
C	-5.37148600	0.90106900	0.64950400
H	-4.43945000	-0.73693600	1.71399700
C	-5.31676200	1.74710800	-0.45949600
H	-4.21500000	2.23734400	-2.24230500

H	-6.18862500	1.00170700	1.35824600
C	-6.36337500	2.80784500	-0.68512900
H	-5.92433800	3.80770400	-0.61360300
H	-6.80510000	2.71648600	-1.68147900
H	-7.16497900	2.73642700	0.05267200
O	0.74489500	-2.61873000	-1.73676900
C	1.56480000	-1.57433000	-1.61497300
C	2.94764800	-1.82382300	-1.54287400
C	1.09325000	-0.25112300	-1.56761600
C	3.83441300	-0.78048300	-1.36096800
H	3.29268700	-2.85034100	-1.59581300
C	1.97970500	0.79525900	-1.39094400
H	0.02471000	-0.07238600	-1.60589000
C	3.33988800	0.52098900	-1.27799000
H	4.89855800	-0.95583000	-1.26160300
H	1.63127300	1.81701600	-1.30575900
N	4.25500000	1.60548100	-0.99352800
O	3.82510100	2.75075700	-1.00187700
O	5.42266900	1.33063400	-0.74554300

M0^{DBU}

0 1

C	0.05889600	0.52669600	1.71984800
C	0.61544200	-0.38251700	2.55066500
H	1.59919600	-0.24262100	2.97750600
H	0.07186100	-1.26817800	2.85469900
H	0.23614400	-1.18527800	-0.31763900
C	0.80114400	1.76713900	1.31871800
C	2.15640300	1.95547200	1.62134500
C	0.15099300	2.76551400	0.58272800
C	2.84358900	3.08296400	1.18296600
H	2.67891900	1.22078700	2.22329800
C	0.83368500	3.89979400	0.14912400
H	-0.89987700	2.63297600	0.35065400
C	2.18633700	4.06054400	0.43702500
H	3.89543400	3.19806900	1.42623600
H	0.30595500	4.65868000	-0.42071200
H	2.72166800	4.93947200	0.09186100

N	-1.19860800	0.45741200	1.12141600
S	-1.94941200	-0.93399300	1.14227300
O	-1.22481500	-1.96483400	0.32618600
O	-2.36666600	-1.45363700	2.46050400
C	-3.44863100	-0.53528900	0.26319400
C	-3.39473300	-0.00654200	-1.02633800
C	-4.66871800	-0.80010100	0.86945200
C	-4.57658300	0.25954100	-1.70203800
H	-2.43703900	0.20720200	-1.48899900
C	-5.84883600	-0.52931900	0.17777000
H	-4.68382300	-1.21139200	1.87265000
C	-5.82119500	0.00090700	-1.11127700
H	-4.53974000	0.67735400	-2.70464400
H	-6.80522500	-0.73212200	0.65131100
C	-7.09248400	0.29169700	-1.86543000
H	-7.15362600	-0.31655900	-2.77278700
H	-7.97266300	0.08176700	-1.25482000
H	-7.13197100	1.34034300	-2.17429900
C	4.98136300	-1.97474400	0.64513300
C	3.54249400	-1.78679000	1.12869600
C	2.46966800	-2.28833300	0.14276900
C	5.33558600	-1.15873800	-0.59924600
C	2.16443600	-1.25855400	-0.90728700
C	4.43488900	-1.46639600	-1.79421200
H	5.15102900	-3.03852700	0.43315100
H	3.40631100	-2.32329600	2.07157600
H	2.78928900	-3.22238600	-0.33346200
H	5.27852100	-0.08470000	-0.38372200
H	4.88777600	-1.11430100	-2.72278500
C	0.53373100	0.31775800	-1.81698900
H	-0.24656700	0.85593300	-1.27553700
C	2.88529800	0.32668300	-2.61765700
C	1.74414800	1.19094600	-2.10429800
H	2.04775900	1.69892600	-1.18273500
H	1.49864300	1.95263500	-2.84668400
H	3.81441800	0.90177500	-2.65346200
N	3.11856700	-0.81640200	-1.72042500
N	0.94632200	-0.76918600	-0.93990600

H	4.29704500	-2.54723800	-1.90206300
H	6.36896600	-1.37551100	-0.88813700
H	5.66518200	-1.70535800	1.45595500
H	3.37070600	-0.72464300	1.34015300
H	1.53507100	-2.48733600	0.67278400
H	0.10899500	-0.09530200	-2.73944800
H	2.67858100	-0.05230200	-3.62534700

M0^{DQH-}

-1 1

C	-4.41019000	-1.35562300	-0.66227700
C	-3.58507900	-1.99432500	-1.53794200
H	-3.55061600	-3.07775500	-1.54981200
H	-3.05026000	-1.46265900	-2.31684900
H	-1.87544800	-2.13182500	-0.18023200
C	-5.11753700	-2.16076500	0.38714400
C	-4.53743000	-3.30298100	0.95268700
C	-6.38001900	-1.75998300	0.83764300
C	-5.21311500	-4.03615000	1.92482100
H	-3.53741700	-3.60158400	0.65034500
C	-7.05889900	-2.49640100	1.80336400
H	-6.81564900	-0.86114500	0.41519800
C	-6.47876600	-3.63897000	2.35099800
H	-4.74332400	-4.91320500	2.35964900
H	-8.04221800	-2.17491400	2.13347900
H	-7.00391800	-4.20870000	3.11144100
N	-4.71962200	-0.01800800	-0.57563400
S	-4.15129700	0.96678100	-1.69300000
O	-2.67339200	1.06741400	-1.71538500
O	-4.76024200	0.79368500	-3.03146200
C	-4.75455900	2.52581000	-1.06803800
C	-4.22788200	3.03866800	0.11692000
C	-5.72156700	3.22455000	-1.77538600
C	-4.68955600	4.25672800	0.59480900
H	-3.46526300	2.48004900	0.65131400
C	-6.17679500	4.44857900	-1.28441600
H	-6.10688700	2.80690300	-2.69939300
C	-5.67225500	4.97822700	-0.09716900

H	-4.28544700	4.66073000	1.51957200
H	-6.93649900	4.99819700	-1.83350200
C	-6.16430300	6.29682300	0.44197300
H	-5.34161700	7.01118700	0.54106500
H	-6.91902800	6.73572500	-0.21374600
H	-6.60692400	6.17150400	1.43471200
C	0.25535100	-0.93348800	1.15883300
C	1.50629200	-0.31649300	1.10326000
C	2.51206300	-0.74945400	0.23806600
C	2.25653300	-1.85299400	-0.57566500
C	1.02666100	-2.51333900	-0.57154600
C	0.02231400	-2.01424400	0.28313100
H	1.72824700	0.51157200	1.76595700
H	3.03888200	-2.17910300	-1.25048100
C	3.82610000	-0.05992200	0.18887900
C	5.00815200	-0.77894800	0.00037000
C	3.91381300	1.32511100	0.32120500
C	6.25722700	-0.16515800	-0.07009000
H	4.93980300	-1.85782600	-0.07399100
C	5.13092000	2.00715000	0.27010800
H	2.99255100	1.88186300	0.44171700
C	6.29576600	1.23857700	0.06526400
O	-1.20158800	-2.65223000	0.30543500
O	7.53455900	1.81899400	-0.01257400
C	0.79204900	-3.73971900	-1.46900900
C	0.44489800	-4.96418100	-0.60266100
C	-0.34053600	-3.46953600	-2.47354000
C	2.04463200	-4.09714100	-2.28201900
H	1.26579000	-5.18880000	0.08660700
H	-0.46268900	-4.79400700	-0.02329200
H	0.29281100	-5.83898500	-1.24508500
H	-0.11918800	-2.58090700	-3.07369000
H	-0.44273700	-4.32296800	-3.15332800
H	-1.29384800	-3.31803000	-1.96941900
H	1.83348200	-4.98638200	-2.88368300
H	2.33030000	-3.29185800	-2.96614100
H	2.89945600	-4.32498500	-1.63705300
C	-0.79988700	-0.48880900	2.18625400

C	-2.13757100	-0.12693100	1.51180800
C	-1.02966100	-1.63152800	3.19217800
C	-0.33203700	0.74242400	2.97501800
H	-1.99839100	0.43388100	0.58061400
H	-2.72829200	-1.01380500	1.28048500
H	-2.74201900	0.48257500	2.19337300
H	-0.10773800	-1.85966400	3.73746100
H	-1.79426900	-1.33638800	3.91973400
H	-1.36907800	-2.53353700	2.67888100
H	-1.10639900	1.01283300	3.69891900
H	0.58950900	0.54953200	3.53295100
H	-0.16847700	1.60449800	2.31951900
C	5.18312000	3.53924200	0.42005400
C	5.72284300	4.19859900	-0.86787000
C	3.78537700	4.13454800	0.64691200
C	6.01866100	3.93706500	1.65703900
H	6.72198600	3.87263500	-1.17026400
H	5.05413900	3.97288000	-1.70332100
H	5.75816000	5.28504800	-0.74064800
H	3.32144800	3.73894900	1.55516600
H	3.87498900	5.21893500	0.76089800
H	3.11926900	3.94062400	-0.19844100
H	6.05724200	5.02750300	1.74216300
H	5.54791300	3.53988900	2.56093900
H	7.04878600	3.57119300	1.65376100
C	7.53841100	-0.98774300	-0.28060500
C	8.22889800	-0.57167000	-1.59297600
C	8.49959500	-0.80116300	0.90807300
C	7.23405100	-2.48868200	-0.38551900
H	7.55876500	-0.73776700	-2.44269800
H	8.52134300	0.47821400	-1.58078000
H	9.12638500	-1.18146100	-1.74542900
H	8.01997900	-1.12320900	1.83813400
H	9.39308100	-1.41711800	0.75733500
H	8.81029700	0.23767000	1.01836300
H	8.17246100	-3.03024600	-0.53760500
H	6.76951600	-2.87529900	0.52701700
H	6.57767000	-2.71424800	-1.23203200

H	7.45629900	2.77390500	0.06297300

R2^{-HOBt}

-1 1

C	2.75700200	1.07412900	0.47396500
C	3.26528800	2.15296800	1.10048800
H	4.32862200	2.21608600	1.29683000
H	2.64116000	2.98999700	1.38823300
H	0.93219800	-0.53209500	0.17731900
C	3.63793200	-0.08302800	0.13059100
C	4.70449200	-0.45959200	0.95531600
C	3.41473700	-0.80772500	-1.04535400
C	5.53713700	-1.51736700	0.60230400
H	4.86547800	0.07035500	1.88925400
C	4.24654300	-1.86470800	-1.40027400
H	2.58009500	-0.52687700	-1.67905900
C	5.31266600	-2.22312400	-0.57838500
H	6.35604700	-1.79970100	1.25689700
H	4.06047800	-2.41026800	-2.32025100
H	5.95817400	-3.05231300	-0.85053900
N	1.43025300	0.88930800	0.05724200
S	0.53768900	2.19893200	-0.22057300
O	0.16197500	2.94691300	0.99802200
O	1.10090600	3.03995400	-1.29585200
C	-0.95170800	1.43929300	-0.83071700
C	-2.17276300	1.84237000	-0.30553200
C	-0.88509700	0.46394900	-1.82433800
C	-3.34038400	1.23346500	-0.75715400
H	-2.19317600	2.59433100	0.47581400
C	-2.05708300	-0.13964700	-2.25877800
H	0.07865300	0.15480400	-2.21775100
C	-3.29690800	0.22509800	-1.72070900
H	-4.29615400	1.52111500	-0.32805300
H	-2.01028800	-0.93074100	-3.00226500
C	-4.53854300	-0.52200800	-2.12473900
H	-5.43960000	-0.04351600	-1.73482900
H	-4.62551000	-0.59487900	-3.21251800
H	-4.49031000	-1.54313800	-1.72907600

C	-1.49172400	-1.18162500	1.16738600
C	-2.68939100	-1.78825100	0.76347400
C	-3.90396200	-1.41628200	1.37127100
C	-3.84540600	-0.45555000	2.35748200
C	-2.61691500	0.14150900	2.75302500
C	-1.41660700	-0.20299500	2.17093800
H	-4.84096400	-1.87030800	1.06532200
H	-4.75891100	-0.13788900	2.84971400
H	-2.63167800	0.89804900	3.53095200
H	-0.47492200	0.26454300	2.43691800
N	-2.41115200	-2.68032000	-0.23967400
N	-1.13068800	-2.63604300	-0.47103400
N	-0.57544000	-1.75703200	0.35186400
O	0.75598600	-1.56526100	0.34106900

M06^{HOBt}

0 1

C	0.59248100	-2.15989100	0.99463300
O	1.31469100	-2.47536700	1.92303800
C	-0.72447600	-1.54289900	1.09113200
C	-1.23869700	-1.20920100	2.28774400
H	-1.23392500	-1.30051900	0.16626500
H	-0.64642800	-1.42085600	3.17729700
C	-2.55009200	-0.59547000	2.49754400
C	-2.89829900	-0.16660200	3.78458100
C	-3.47785600	-0.43610100	1.45489800
C	-4.13573200	0.42037200	4.02845800
H	-2.18371700	-0.28865400	4.59392000
C	-4.71773600	0.13511000	1.70313800
H	-3.23310600	-0.76855100	0.44975600
C	-5.04794400	0.57132500	2.98755700
H	-4.38725000	0.75635200	5.02905200
H	-5.43515300	0.24394600	0.89546300
H	-6.01707900	1.02617700	3.16826300
C	0.87218200	1.29242900	1.39107900
C	-0.29269500	1.86260200	1.75139500
C	2.06384900	1.46913400	2.27961300
C	2.54926500	0.40914500	3.04888900

C	2.71385800	2.70486700	2.33657000
C	3.68204000	0.57459100	3.84205000
H	2.03076600	-0.54648400	3.02376100
C	3.84204600	2.87267000	3.13646200
H	2.33300600	3.52964100	1.73968400
C	4.33307700	1.80546100	3.88484000
H	4.05470400	-0.25842200	4.43073100
H	4.34226900	3.83603700	3.16991400
H	5.21899700	1.93243400	4.49935900
N	1.15421400	0.50033200	0.26233400
S	0.37719100	0.80077600	-1.08267800
O	-1.11833200	0.85773000	-0.94434000
O	0.82493100	-0.16627900	-2.10315200
C	0.87474000	2.41595700	-1.64678100
C	0.15888100	3.54952500	-1.27632000
C	2.05933100	2.51532200	-2.37787300
C	0.64197800	4.80106500	-1.65569000
H	-0.76528000	3.46236400	-0.71505200
C	2.52123400	3.77013600	-2.74795800
H	2.59636600	1.61416500	-2.65948400
C	1.82030400	4.93038100	-2.39183200
H	0.08604300	5.69127900	-1.37562900
H	3.43838800	3.85614800	-3.32443800
C	2.32528500	6.28286900	-2.82188700
H	1.80145100	7.08616600	-2.30030000
H	2.17642500	6.42590900	-3.89687400
H	3.39600300	6.38295600	-2.62497500
H	-1.20336400	1.76032300	1.17356100
H	-0.32717300	2.45059500	2.66201500
C	-4.85674600	2.29771700	-0.97671100
C	-5.20835100	3.03316400	0.16418100
C	-6.50224000	2.92213500	0.70617300
C	-7.38001700	2.07078500	0.06994000
C	-7.00202900	1.33431400	-1.08632500
C	-5.74013100	1.42831700	-1.63521900
H	-6.78305300	3.48525700	1.58946200
H	-8.38748200	1.95415500	0.45446200
H	-7.73294100	0.67843000	-1.54767800

H	-5.44139600	0.87398200	-2.51699100
N	-4.12060600	3.76979100	0.56477600
N	-3.15384100	3.53925300	-0.26440900
N	-3.56705800	2.66125200	-1.17658100
O	-2.78012200	2.30184100	-2.21149500
H	-2.07716500	1.69589100	-1.80490400
C	1.01324200	-2.55775500	-0.41746300
C	1.94876000	-2.96852100	-2.33058500
C	3.34401600	-1.52150200	-0.85702600
H	2.96659500	-0.51013400	-1.02222700
N	0.76940000	-3.51340400	-2.41414900
N	0.20268400	-3.25409500	-1.21219900
N	2.16370900	-2.40197800	-1.09917100
C	2.98377300	-2.93514200	-3.41421300
H	2.48685400	-2.81518000	-4.37733400
H	3.53836000	-3.88464900	-3.41437800
C	4.39140000	-1.86914100	-1.91265800
O	3.84719400	-1.84469800	-3.21728700
C	-1.10665700	-3.76419500	-0.89417700
C	-1.20822300	-4.79969400	0.03552400
C	-2.20989700	-3.16954800	-1.51908300
C	-2.49530800	-5.22101800	0.37467900
C	-3.46760900	-3.62781300	-1.14088400
C	-3.62928800	-4.64029600	-0.18905500
H	-2.60834200	-6.02261400	1.09949700
H	-4.34689600	-3.17681700	-1.59467500
H	5.17593800	-1.11295900	-1.87299800
C	0.00690100	-5.44239500	0.65383700
H	0.80508800	-5.57825200	-0.08133900
H	0.40834600	-4.84014900	1.47502000
H	-0.25414900	-6.42148900	1.05804500
C	-5.01139900	-5.08061600	0.21498700
H	-5.52690800	-4.27545300	0.74727500
H	-5.61338500	-5.33072900	-0.66263200
H	-4.97565500	-5.95296300	0.86980300
C	-2.03893800	-2.07843600	-2.54152300
H	-1.30530000	-1.33110400	-2.22449200
H	-1.68509000	-2.49525700	-3.48966300

H	-2.99159300	-1.57614400	-2.72010700
H	4.83896400	-2.85321900	-1.69962300
C	3.91501000	-1.64287500	0.55237900
H	3.14089300	-1.36693300	1.26751800
H	4.20699200	-2.68022500	0.74975300
C	5.09334700	-0.71136000	0.72516900
C	6.36198000	-1.19872600	1.04103000
C	4.91311200	0.66770600	0.57360900
C	7.43472900	-0.32500900	1.21193800
H	6.50991000	-2.26915800	1.15985800
C	5.98337400	1.53969000	0.74109800
H	3.92149100	1.05561400	0.34873100
C	7.24771500	1.04628300	1.06128200
H	8.41549900	-0.71746300	1.46214300
H	5.82439800	2.60865500	0.63560700
H	8.08131500	1.72840700	1.19522100

TS6(R)^{HOBt}

0 1

C	1.27608700	1.87050500	-0.59363600
C	2.63066300	3.15306700	0.53671700
C	3.55835700	0.86824800	0.15367200
H	3.21545200	0.28821900	1.01583700
N	1.55748900	3.86879600	0.37014900
N	0.72523300	3.05860700	-0.33557500
N	2.51484100	1.91717600	-0.05571600
C	3.88552200	3.60009000	1.22661300
H	3.62595800	4.16059800	2.12655500
H	4.44173600	4.26168700	0.54714500
C	4.84966400	1.59914200	0.52359400
O	4.66091300	2.49257300	1.60202100
C	-0.51300300	3.58434500	-0.85995300
C	-0.53238300	3.95635700	-2.20569500
C	-1.61620800	3.69728200	-0.01125900
C	-1.73811200	4.43817600	-2.71298100
C	-2.79505000	4.18540700	-0.56913500
C	-2.87762700	4.54822000	-1.91550400
H	-1.78515700	4.73493100	-3.75724000

H	-3.67329000	4.27975900	0.06434800
H	5.58066700	0.85946000	0.84868400
C	0.59230400	0.82048900	-1.46470800
O	1.23400800	0.37572700	-2.42187100
C	-0.75765200	0.56889400	-1.13295000
C	-1.47906200	-0.33437500	-1.89641000
H	-1.18340500	1.03356600	-0.25564200
H	-1.01960900	-0.62298600	-2.83915100
C	-2.95343000	-0.40957700	-1.85138700
C	-3.64223900	-0.86206700	-2.98118100
C	-3.68618300	0.00298600	-0.72842300
C	-5.03417100	-0.87363700	-3.00832200
H	-3.07710500	-1.19684800	-3.84734000
C	-5.07523600	0.00301200	-0.76206100
H	-3.16157700	0.30988000	0.17363700
C	-5.75333200	-0.43219600	-1.90184100
H	-5.55413400	-1.22353200	-3.89428200
H	-5.63908500	0.32493800	0.10911700
H	-6.83865900	-0.43952400	-1.91403500
C	-4.18170800	5.03108900	-2.49353900
H	-4.70394100	5.68710300	-1.79314600
H	-4.02484800	5.57335000	-3.42784100
H	-4.84039000	4.18254700	-2.70383100
C	0.69588500	3.83665200	-3.07001200
H	1.56506100	4.29098600	-2.58372600
H	0.93925600	2.78875200	-3.27531100
H	0.53717000	4.33989500	-4.02440000
C	-1.55693200	3.27198500	1.43181900
H	-0.62781000	3.59435900	1.90973600
H	-2.39340300	3.69839500	1.98734700
H	-1.62548900	2.17992600	1.52376200
H	5.24838700	2.12810100	-0.35630100
C	3.76629800	-0.04159500	-1.05678000
H	2.85351800	-0.61480100	-1.23008200
H	3.94020800	0.57056100	-1.94682900
C	4.91869600	-0.99722800	-0.84193000
C	5.94036400	-1.10089200	-1.78604500
C	4.95545100	-1.83200900	0.28169300

C	6.96852800	-2.02900400	-1.62525700
H	5.92291200	-0.45860900	-2.66227900
C	5.98169600	-2.75624300	0.44802000
H	4.16087200	-1.76942100	1.02278200
C	6.99140300	-2.86047500	-0.50923600
H	7.75094400	-2.10076600	-2.37404400
H	5.99081900	-3.40013700	1.32171500
H	7.79015400	-3.58389000	-0.38260700
C	0.25858200	-2.24682000	-0.83157900
C	-1.10697100	-2.35393600	-1.02297500
C	1.20340700	-2.79781000	-1.84207900
C	0.85126000	-2.94543000	-3.18975300
C	2.47980600	-3.19465400	-1.43499200
C	1.76328500	-3.46203700	-4.10286700
H	-0.13129400	-2.63434000	-3.53151100
C	3.39849900	-3.70141500	-2.34904800
H	2.74526500	-3.09096700	-0.38923400
C	3.04213100	-3.83680000	-3.68839300
H	1.48059900	-3.56046300	-5.14620600
H	4.39288700	-3.97940400	-2.00896100
H	3.75372200	-4.22948800	-4.40769100
N	0.85717700	-1.55549300	0.17819900
S	0.10325000	-1.43749400	1.59504300
O	-1.15248000	-0.61338600	1.58768700
O	-0.05302100	-2.73558000	2.26474700
C	1.28061300	-0.48342300	2.51965500
C	1.10221100	0.88700400	2.66934600
C	2.37434800	-1.13248300	3.08640300
C	2.04529100	1.61952700	3.38445100
H	0.22653700	1.36301500	2.24109000
C	3.31422500	-0.38484100	3.78838600
H	2.47605700	-2.20869000	2.98381500
C	3.16601300	0.99869200	3.94692500
H	1.89867200	2.68833600	3.52297700
H	4.17002700	-0.88337000	4.23415500
C	4.19411700	1.80235100	4.69510400
H	4.69557900	1.19666500	5.45272500
H	4.94886800	2.17229000	3.99446000

H	3.73806000	2.66562900	5.18524100
H	-1.79648700	-2.13082700	-0.21834700
H	-1.48187400	-3.00825600	-1.80257600
C	-5.22005600	-1.65603700	2.08827200
C	-5.69523600	-2.49863300	1.07371700
C	-7.03302700	-2.40260200	0.64767600
C	-7.82462100	-1.45881500	1.26550200
C	-7.32126900	-0.61725200	2.29553200
C	-6.01563600	-0.69507700	2.73163600
H	-7.40939300	-3.04114900	-0.14390500
H	-8.86120200	-1.34905100	0.96551300
H	-7.98972100	0.10640700	2.75014200
H	-5.61887300	-0.05986700	3.51435500
N	-4.66108900	-3.29058200	0.64005000
N	-3.60897000	-2.99525200	1.33746900
N	-3.91988300	-2.02016200	2.18725500
O	-3.03086600	-1.55500400	3.08942400
H	-2.28001300	-1.16624700	2.54301900

TS6(S)^{HOBt}

0 1

C	0.73843900	2.30650300	0.58070200
C	1.94782000	3.84295700	-0.38106600
C	3.17543200	2.51488600	1.37576800
H	2.83289800	2.77768700	2.37742000
N	0.74859400	4.01436500	-0.85577400
N	0.00376700	3.05167700	-0.24718800
N	1.99235700	2.79851300	0.50621000
C	3.14821000	4.71297800	-0.59418100
H	2.81989000	5.72400800	-0.83635000
H	3.76765300	4.32650200	-1.41545500
C	4.31764500	3.44639500	0.94422600
O	3.87027700	4.74861100	0.61613300
C	-1.42833300	3.07213600	-0.41953300
C	-1.95024100	2.76795400	-1.67821700
C	-2.22337700	3.45163800	0.67057600
C	-3.33853400	2.80554000	-1.81409100
C	-3.60308400	3.45544400	0.47977000

C	-4.17932100	3.12387400	-0.74874800
H	-3.76689000	2.58897300	-2.78987800
H	-4.24343500	3.73620900	1.31216300
H	5.01211100	3.53585300	1.78180300
C	0.26474800	1.26440300	1.57591500
O	0.90537500	1.22799600	2.63482000
C	-0.90246900	0.52919700	1.25281000
C	-1.38219200	-0.37444600	2.18924000
H	-1.37283500	0.64977500	0.28412000
H	-0.86394400	-0.37571900	3.14695800
C	-2.79443000	-0.82080700	2.23196500
C	-3.20217800	-1.75746400	3.19069200
C	-3.76508700	-0.25429100	1.39482300
C	-4.53405400	-2.13771700	3.29831500
H	-2.46244600	-2.18896600	3.86044800
C	-5.10102800	-0.62889700	1.50876900
H	-3.47857200	0.49322000	0.65853200
C	-5.48953500	-1.57524600	2.45339700
H	-4.82832900	-2.86970900	4.04367600
H	-5.84127400	-0.18630500	0.84685200
H	-6.53112000	-1.87032000	2.53239200
C	-5.67672900	3.10409200	-0.90863700
H	-6.09095800	2.17252300	-0.50731100
H	-6.14290000	3.93276500	-0.37022600
H	-5.96462400	3.16737400	-1.96105400
C	-1.06827000	2.44127300	-2.85197900
H	-0.59478800	3.34942600	-3.23800200
H	-0.27953800	1.74146100	-2.56863100
H	-1.66274200	1.99589100	-3.65166000
C	-1.64104400	3.84269600	2.00366400
H	-0.69951500	4.38851600	1.89396700
H	-2.34205400	4.48219200	2.54236700
H	-1.44879300	2.96029800	2.62243000
H	4.85392400	3.01756900	0.08914200
C	3.61366400	1.04402800	1.39427500
H	4.15568900	0.90670200	2.33631400
H	2.72670500	0.40952000	1.46086800
C	4.48487400	0.57442100	0.25293800

C	4.08779400	0.71125400	-1.08224400
C	5.71250600	-0.03677500	0.52404800
C	4.90688700	0.26300900	-2.11489500
H	3.11667300	1.14163800	-1.31564000
C	6.52426000	-0.50572900	-0.50704600
H	6.03299200	-0.14737500	1.55745600
C	6.12522400	-0.35109500	-1.83168600
H	4.57955800	0.37105300	-3.14409100
H	7.47007100	-0.98503800	-0.27410300
H	6.75671400	-0.70911900	-2.63906000
C	0.87243800	-1.92615500	1.52667400
C	-0.44690600	-2.33609500	1.61500800
C	1.80096300	-2.11754200	2.68247800
C	1.32551000	-2.34895200	3.97945600
C	3.18673100	-2.06858900	2.48118600
C	2.20658500	-2.53918700	5.03844800
H	0.25759300	-2.36047700	4.17284400
C	4.06827100	-2.26339900	3.53957900
H	3.56732800	-1.86469800	1.48456200
C	3.58285900	-2.50167700	4.82339700
H	1.81534800	-2.70874400	6.03654800
H	5.13855500	-2.23006100	3.35858000
H	4.27008700	-2.65133200	5.64988800
N	1.42533300	-1.20826900	0.52065900
S	0.91992800	-1.11043300	-0.98059400
O	-0.32022400	-1.87918700	-1.31041700
O	0.86841900	0.29821900	-1.41089100
C	2.20159400	-1.88713700	-1.94458500
C	3.07761600	-2.80644300	-1.37930000
C	2.26819400	-1.56431400	-3.29647200
C	4.04764900	-3.39377300	-2.18395700
H	3.00623600	-3.04726100	-0.32388900
C	3.24017700	-2.16534500	-4.08771900
H	1.57832800	-0.83857600	-3.71575800
C	4.14842800	-3.07799600	-3.54225300
H	4.74377800	-4.10574300	-1.74936900
H	3.30103300	-1.91616300	-5.14348800
C	5.23365100	-3.68921200	-4.38872500

H	5.39090800	-4.73939300	-4.13101000
H	4.99208900	-3.62537200	-5.45166500
H	6.18297500	-3.16713300	-4.22885600
H	-1.07869100	-2.36186400	0.73786300
H	-0.72861100	-2.99168100	2.43014700
C	-4.79689100	-1.28866600	-1.60528800
C	-5.39426800	-2.45939700	-1.11293400
C	-6.79407200	-2.60505600	-1.15970900
C	-7.52051500	-1.55869500	-1.68488400
C	-6.89229100	-0.37838600	-2.16991500
C	-5.52351300	-0.21720900	-2.14636600
H	-7.27003900	-3.50231300	-0.77961900
H	-8.60209100	-1.62731200	-1.73077900
H	-7.51282600	0.41791000	-2.56862500
H	-5.02982700	0.67884600	-2.50292100
N	-4.41718400	-3.26669100	-0.59355900
N	-3.27902800	-2.65237300	-0.72783600
N	-3.48931100	-1.49552100	-1.34028300
O	-2.50741100	-0.58930600	-1.54101900
H	-1.63315000	-1.10479100	-1.47716000

M6(R)^{HOBt}

0 1

C	1.63403200	-1.34702400	-1.08406300
C	1.61039800	-3.50801700	-0.78574600
C	-0.52199400	-2.54488900	-1.66563300
H	-1.03885700	-1.64393200	-1.33692800
N	2.83155400	-3.15453000	-0.50880600
N	2.83608200	-1.80242200	-0.71766600
N	0.85616400	-2.43766200	-1.17230100
C	0.89900100	-4.81130500	-0.61037700
H	1.41927200	-5.42988500	0.12033000
H	0.82472100	-5.35625600	-1.56181900
C	-1.18823600	-3.80920600	-1.04625700
O	-0.38155400	-4.49419500	-0.10252100
C	4.07089200	-1.06441100	-0.68718800
C	4.54127600	-0.52980400	-1.89061600
C	4.74286800	-0.91971000	0.53100300

C	5.73587300	0.19038100	-1.84536300
C	5.93111300	-0.19170800	0.51815300
C	6.44236700	0.36788200	-0.65570000
H	6.12073400	0.62218400	-2.76553700
H	6.46922300	-0.05500800	1.45279300
H	-2.08785100	-3.50943700	-0.50843900
C	1.15141700	0.07077000	-1.25327200
O	0.33434300	0.27909700	-2.21204600
C	1.62305000	0.89366400	-0.26935100
C	1.23957000	2.32666500	-0.11798900
H	0.68929900	3.64969200	1.55865600
H	0.45108400	2.54232900	-0.85291300
C	2.39215200	3.29778000	-0.37047900
C	2.12949200	4.66600700	-0.49972800
C	3.71333400	2.86002400	-0.47543600
C	3.15766500	5.57415100	-0.72925700
H	1.10385800	5.02314900	-0.42435500
C	4.74702100	3.76760400	-0.70331200
H	3.93909200	1.80137300	-0.39386500
C	4.47486500	5.12627900	-0.83065200
H	2.93196400	6.63083400	-0.83322400
H	5.76716900	3.40260300	-0.78680900
H	5.27890600	5.83239100	-1.01160200
C	7.74231600	1.12977200	-0.63219200
H	7.86443600	1.73249500	-1.53470500
H	7.79353100	1.79057800	0.23711800
H	8.59151500	0.44195600	-0.57086600
C	3.79011100	-0.70282500	-3.18560400
H	3.47426700	-1.74164200	-3.32634600
H	2.89067200	-0.07799500	-3.21426100
H	4.42363300	-0.42202900	-4.02809900
C	4.19675200	-1.50668700	1.80489400
H	3.15924700	-1.19992700	1.96997900
H	4.20510200	-2.59957900	1.76480700
H	4.79806400	-1.18548400	2.65654100
H	-1.46435300	-4.49933600	-1.85531200
C	-0.49037600	-2.59324600	-3.20297600
H	0.13307400	-1.75878100	-3.53560400

H	-0.03348000	-3.53583100	-3.52813700
C	-1.88181600	-2.44295200	-3.76604500
C	-2.61189900	-3.52643300	-4.25623700
C	-2.47035800	-1.17411000	-3.75873900
C	-3.91271800	-3.34663200	-4.72590500
H	-2.15903800	-4.51466900	-4.27970300
C	-3.76965600	-0.99348700	-4.21947500
H	-1.88268200	-0.33673300	-3.38966600
C	-4.49489400	-2.08208500	-4.70458800
H	-4.47028500	-4.19586600	-5.10785900
H	-4.21720300	-0.00374000	-4.19870600
H	-5.50848900	-1.94391000	-5.06681100
C	-0.73000200	2.06628200	1.45182300
C	0.67667400	2.58360100	1.32457700
C	-1.82669400	3.06959200	1.51219000
C	-1.95952300	4.03195700	0.50432800
C	-2.69970800	3.07231200	2.60394200
C	-2.99554800	4.95566900	0.56438400
H	-1.28053300	4.03026200	-0.34358800
C	-3.71307700	4.02474000	2.67423400
H	-2.58356400	2.33195900	3.38754000
C	-3.87114800	4.95475300	1.65088600
H	-3.11734100	5.68022100	-0.23396000
H	-4.38359200	4.03190300	3.52691500
H	-4.67244500	5.68468900	1.70017200
N	-0.88241300	0.78530100	1.47596600
S	-2.38509500	0.01111800	1.28273400
O	-1.98777800	-1.28399800	0.73094200
O	-3.14777100	0.03564600	2.52623200
C	-3.23372700	0.92721700	0.02723100
C	-2.58645600	1.12713500	-1.18964600
C	-4.48684400	1.46292700	0.29890600
C	-3.22702100	1.89753900	-2.15316200
H	-1.58775600	0.72876800	-1.38309700
C	-5.11039300	2.22111800	-0.68545600
H	-4.94749100	1.30169400	1.26764100
C	-4.48907900	2.45479200	-1.91670300
H	-2.73270700	2.07480000	-3.10536600

H	-6.08927600	2.64928400	-0.49047200
C	-5.14933600	3.32383900	-2.95363800
H	-4.82880100	3.05089000	-3.96145300
H	-6.23734300	3.24741400	-2.89984300
H	-4.88254200	4.37382900	-2.79331900
H	0.34946400	-0.58719100	1.30655300
H	1.31950800	2.06149300	2.04140200
C	-0.42182800	-2.30643300	3.50702100
C	-0.43194700	-1.90065800	4.84825500
C	-1.23922700	-2.57643400	5.78307300
C	-1.99164300	-3.63047000	5.31392900
C	-1.95781100	-4.02666100	3.94882400
C	-1.17754000	-3.38138200	3.01429300
H	-1.25768000	-2.27219800	6.82358200
H	-2.62874700	-4.17981000	5.99867000
H	-2.56923200	-4.86701200	3.63699200
H	-1.12817700	-3.67182800	1.97124500
N	0.42594000	-0.83476400	4.99451600
N	0.95061300	-0.57814300	3.84025000
N	0.46159300	-1.44042500	2.95059300
O	0.84316800	-1.37692400	1.65727500
H	2.30271300	0.48761100	0.47405400

M6(S)^{HOBt}

0 1

C	4.14111200	-0.40122100	0.07942100
C	6.30763300	-0.15140400	0.16765300
C	4.95482800	1.98794900	0.09127300
H	4.56921100	2.20206500	-0.90773700
N	6.12377400	-1.44185600	0.14517300
N	4.76367800	-1.57917300	0.09228900
N	5.11665800	0.52023100	0.14368400
C	7.59074700	0.62566500	0.15856100
H	8.32957100	0.11069000	-0.45633600
H	7.98138300	0.72285400	1.18105200
C	6.34571200	2.59309400	0.30711100
O	7.34507500	1.89268000	-0.41251800
C	4.12504500	-2.86615400	0.00871800

C	4.07973400	-3.64982500	1.16319500
C	3.50625000	-3.22625700	-1.19176800
C	3.36818000	-4.84627600	1.09502600
C	2.79745400	-4.42622000	-1.20197100
C	2.71353700	-5.24191600	-0.07283900
H	3.31003200	-5.47314900	1.98141500
H	2.27887600	-4.72034000	-2.11104200
H	6.34532600	3.62501100	-0.04816000
C	2.70100700	-0.09865700	-0.22708100
O	2.58373600	0.74870800	-1.17579600
C	1.76255300	-0.77598500	0.49007600
C	0.32857500	-0.66982800	0.06460000
H	2.05242700	-1.51029400	1.23636500
H	0.31639700	0.10371100	-0.70720600
C	-0.10879300	-1.97280500	-0.58533400
C	-0.12460100	-2.08522900	-1.97922700
C	-0.44849900	-3.09552300	0.17676300
C	-0.48163400	-3.28243000	-2.59677800
H	0.14309400	-1.21984400	-2.58070800
C	-0.82008300	-4.29075600	-0.43599500
H	-0.40623300	-3.04598200	1.26276200
C	-0.83998000	-4.38890500	-1.82646700
H	-0.49238700	-3.34764600	-3.68056900
H	-1.08702400	-5.14966200	0.17340400
H	-1.12941900	-5.31949300	-2.30553700
C	1.94560300	-6.53682700	-0.12264800
H	1.49205600	-6.76313200	0.84564300
H	1.15345200	-6.48824700	-0.87356700
H	2.60686600	-7.37014300	-0.38168300
C	4.74188800	-3.19316300	2.43600200
H	5.82063800	-3.07663600	2.30078800
H	4.34771600	-2.22198100	2.75522000
H	4.56726400	-3.91254500	3.23724900
C	3.58389900	-2.35568000	-2.41773600
H	4.61874100	-2.06490800	-2.62579600
H	3.19849100	-2.89575300	-3.28437600
H	3.00066200	-1.43619100	-2.30060900
H	6.59002200	2.58996600	1.38027500

C	3.94848400	2.47297700	1.14884700
H	3.17103400	1.71593400	1.27894000
H	4.46382400	2.57637200	2.11066100
C	3.29351200	3.77540000	0.75295100
C	3.78682000	5.00268400	1.19872800
C	2.16811800	3.74880600	-0.07868700
C	3.16458100	6.19242000	0.82394700
H	4.65601500	5.02671300	1.85255300
C	1.54485200	4.93725300	-0.44773400
H	1.79751700	2.79003500	-0.43796000
C	2.04086000	6.16104900	0.00168000
H	3.55331300	7.14129000	1.18029200
H	0.66832400	4.90621700	-1.08911700
H	1.55113200	7.08647200	-0.28493300
C	-2.04438200	-0.07228500	0.75774400
C	-0.61034500	-0.23044700	1.19665200
C	-3.11038200	-0.37894200	1.74749500
C	-3.24668500	-1.68052900	2.24098400
C	-3.95994800	0.63912600	2.18829300
C	-4.26832400	-1.97283500	3.13617800
H	-2.58658000	-2.46464600	1.88014700
C	-4.95728000	0.34313600	3.11378700
H	-3.83081200	1.64880800	1.81185400
C	-5.12084700	-0.96019500	3.57605100
H	-4.39820200	-2.98929100	3.49319200
H	-5.61160200	1.13285600	3.46757600
H	-5.91105900	-1.18776000	4.28431800
N	-2.23653000	0.34248800	-0.44763400
S	-3.76265800	0.40130400	-1.19682400
O	-4.56394500	1.49322500	-0.65262200
O	-3.42247900	0.41943700	-2.61404200
C	-4.50957600	-1.15402300	-0.79213400
C	-5.72666400	-1.16545900	-0.12503500
C	-3.82848100	-2.32952700	-1.10992000
C	-6.27792000	-2.39244600	0.23233800
H	-6.21285200	-0.22985900	0.13037900
C	-4.39235000	-3.54017700	-0.73465600
H	-2.86623600	-2.30063500	-1.61715900

C	-5.61991500	-3.58903600	-0.05946800
H	-7.22530500	-2.41779200	0.76254600
H	-3.86481100	-4.46252500	-0.96309300
C	-6.20571900	-4.91568700	0.34511700
H	-6.43673400	-5.52179200	-0.53584100
H	-5.49635800	-5.48333100	0.95421000
H	-7.12467500	-4.78523900	0.91936200
H	-1.08074700	1.53405700	-1.24371800
H	-0.57769100	-0.91728000	2.04888300
C	-2.50217000	3.98023900	-0.84959800
C	-3.02556700	4.42855400	0.36944900
C	-4.11117600	5.32292100	0.38536300
C	-4.61518800	5.71680600	-0.83504800
C	-4.06965300	5.24450500	-2.05908000
C	-3.00593100	4.36909400	-2.09889400
H	-4.52501400	5.67867000	1.32236900
H	-5.45357900	6.40397400	-0.87131200
H	-4.51024000	5.58417000	-2.99054900
H	-2.58586600	3.99365000	-3.02420200
N	-2.31933800	3.84050000	1.39409900
N	-1.41016000	3.08041600	0.87447100
N	-1.50141800	3.15684800	-0.45219100
O	-0.67800400	2.44156500	-1.25026600
H	-0.29250900	0.75045600	1.57620800

TS7(S)^{HOBt}

0 1

C	1.15590000	2.61122300	-0.36487900
C	1.16441100	4.14919100	-1.90493700
C	-1.09549500	3.74724100	-0.87377300
H	-1.59134700	2.79458300	-1.06617400
N	2.40740600	3.76085500	-1.80127400
N	2.38742000	2.81820200	-0.83343000
N	0.36309100	3.48729500	-1.01993300
C	0.57168600	5.08571500	-2.91372100
H	1.11540100	4.98767100	-3.85381900
H	0.63696200	6.12495200	-2.56450500
C	-1.49902500	4.80397600	-1.92261800

O	-0.76514000	4.69591400	-3.12358000
C	3.59837500	2.11081600	-0.49809100
C	4.31011100	2.50915300	0.63685900
C	3.96912800	1.05124700	-1.32409800
C	5.41699600	1.74006700	0.97949200
C	5.08484500	0.31126000	-0.93112000
C	5.80271900	0.62826800	0.22124100
H	5.99043600	2.00979100	1.86244800
H	5.37311100	-0.54996000	-1.52733400
H	-2.55330000	4.66582200	-2.16319700
C	0.64976100	1.52841900	0.58253700
O	-0.55127500	1.40806700	0.63684400
C	1.62357100	0.71210900	1.37784200
C	0.91931600	-0.33762900	2.25712700
H	2.21682800	1.39914900	1.99582600
H	0.10139200	0.15749300	2.78891300
C	1.91400300	-0.86951700	3.27297300
C	1.61854100	-0.85256200	4.63653200
C	3.14188300	-1.39935900	2.85559600
C	2.52859300	-1.34888800	5.56903300
H	0.66478100	-0.45075200	4.96859600
C	4.05192300	-1.89375400	3.78518100
H	3.37957500	-1.43380500	1.79300100
C	3.74913300	-1.86790400	5.14635600
H	2.28193900	-1.32890100	6.62615200
H	4.99813800	-2.30516900	3.44669500
H	4.45912900	-2.25418500	5.87071900
C	6.96720900	-0.21719800	0.66361300
H	7.80793200	0.40550200	0.97945200
H	6.67898200	-0.83801800	1.51786100
H	7.30367000	-0.87935700	-0.13583700
C	3.89434800	3.71375600	1.44006000
H	3.81795500	4.59946800	0.80240800
H	2.92008400	3.57273600	1.92060900
H	4.62434200	3.91619800	2.22420100
C	3.19569700	0.73097700	-2.57458900
H	2.12038200	0.67979900	-2.37233900
H	3.35922000	1.50205900	-3.33377100

H	3.51403500	-0.23119300	-2.98121500
H	-1.36520200	5.80977400	-1.49800400
C	-1.43639100	4.25288400	0.53666100
H	-0.96873700	3.61294500	1.28556500
H	-1.03060800	5.26330000	0.65484800
C	-2.93505800	4.23906400	0.73494600
C	-3.71408300	5.37343800	0.49636000
C	-3.55933000	3.05167600	1.12714200
C	-5.09914100	5.31929700	0.63961100
H	-3.23601100	6.30645300	0.20654700
C	-4.94224300	2.99706500	1.27315100
H	-2.96808900	2.16474300	1.34445500
C	-5.71490700	4.13056800	1.02496100
H	-5.69472700	6.20753700	0.45372700
H	-5.39508300	2.06340300	1.59000200
H	-6.79357700	4.09071200	1.13925900
C	-1.03913200	-1.85983900	1.62911300
C	0.31314500	-1.48195400	1.44538900
C	-1.42807300	-3.17925200	1.02933800
C	-2.00930800	-4.16494300	1.83216200
C	-1.18439100	-3.44972200	-0.32001900
C	-2.33784500	-5.40289900	1.28693500
H	-2.21155500	-3.95206300	2.87592100
C	-1.52648200	-4.68164000	-0.86592800
H	-0.78151400	-2.66839200	-0.95304600
C	-2.10155800	-5.66364400	-0.06172500
H	-2.78223500	-6.16569800	1.91846500
H	-1.34550400	-4.86671500	-1.92136900
H	-2.36757500	-6.62795800	-0.48343100
N	-1.88325100	-1.05474700	2.26775200
S	-3.49912700	-1.14671000	2.04050400
O	-4.14399000	-2.26787000	2.73798300
O	-4.02334100	0.18775900	2.36173900
C	-3.69866600	-1.40791900	0.28508200
C	-4.42615700	-2.49628400	-0.18078900
C	-3.01222200	-0.56988600	-0.58906400
C	-4.44253500	-2.75980900	-1.54599400
H	-4.92950100	-3.14673200	0.52692500

C	-3.03803500	-0.84848400	-1.95115600
H	-2.42618500	0.25946400	-0.20209900
C	-3.73527400	-1.95547400	-2.44544600
H	-4.98938000	-3.62206800	-1.91838400
H	-2.49002500	-0.20793200	-2.63794100
C	-3.68820700	-2.30961700	-3.90901800
H	-4.67010300	-2.61923900	-4.27571200
H	-3.34315700	-1.46701600	-4.51285100
H	-3.00026500	-3.14710300	-4.07411800
H	0.27690500	-1.03289800	0.08893000
H	0.98944300	-2.32363700	1.28378300
C	1.24578600	-2.35613200	-2.57264100
C	2.45280300	-3.06896700	-2.62302000
C	2.65926400	-4.02649900	-3.63644300
C	1.63926100	-4.21526000	-4.54287000
C	0.42283200	-3.48065100	-4.47036000
C	0.19827400	-2.53765500	-3.49092900
H	3.58451900	-4.58978900	-3.68781800
H	1.75801800	-4.94561600	-5.33595600
H	-0.34700100	-3.67047100	-5.21157200
H	-0.72592500	-1.97364600	-3.41436000
N	3.24547200	-2.66228800	-1.58003300
N	2.59046100	-1.75495700	-0.91457400
N	1.40812300	-1.55995700	-1.48808000
O	0.54473400	-0.64728100	-1.02508100
H	2.32477100	0.23418300	0.68287700

TS7(R)^{HOBt}

0 1

C	-3.50956300	-1.58967000	0.04332300
C	-5.64816300	-1.93458000	-0.04376400
C	-4.96294700	0.47141400	0.37679600
H	-4.62458100	0.65593900	1.39966800
N	-5.10477200	-3.11038800	-0.21808700
N	-3.76887600	-2.87411400	-0.16666100
N	-4.69362300	-0.96697300	0.11583600
C	-7.09887900	-1.57150900	0.06580200
H	-7.63564400	-2.37220100	0.57513600

H	-7.52833300	-1.42954500	-0.93503100
C	-6.48117100	0.66732300	0.25792500
O	-7.20393600	-0.39973700	0.84098100
C	-2.79251300	-3.93062500	-0.26506900
C	-2.59216900	-4.50505900	-1.52663300
C	-2.03945200	-4.25842000	0.86485300
C	-1.57346100	-5.44443700	-1.63670000
C	-1.02576200	-5.20378100	0.69236800
C	-0.77730600	-5.79962600	-0.54172900
H	-1.38503100	-5.90030300	-2.60522700
H	-0.40510700	-5.46247600	1.54645200
H	-6.75784800	1.58145600	0.78412500
C	-2.16791100	-0.96490700	0.37485700
O	-2.16398000	-0.27699000	1.36693900
C	-1.02027200	-1.20542100	-0.55830500
C	0.33391900	-0.84643800	0.08181600
H	-1.04921400	-2.23160300	-0.93711400
H	0.12083700	-0.31144200	1.01125500
C	1.11143800	-2.09641900	0.45464700
C	1.26770600	-2.45863800	1.79385700
C	1.69317300	-2.90120200	-0.53067700
C	1.98633700	-3.60196800	2.14587900
H	0.84658800	-1.81884800	2.56571900
C	2.41712400	-4.03855400	-0.18515200
H	1.58412100	-2.62749200	-1.57824800
C	2.56580900	-4.39331900	1.15671200
H	2.10759500	-3.86193400	3.19291600
H	2.86722500	-4.65059200	-0.96156000
H	3.13610200	-5.27674400	1.42733100
C	0.31412200	-6.82431500	-0.69647400
H	0.83291100	-6.70061300	-1.65037100
H	1.04553000	-6.74018200	0.10957100
H	-0.10499000	-7.83543200	-0.67591200
C	-3.41409700	-4.08300100	-2.71500900
H	-4.46942300	-4.33270600	-2.57663400
H	-3.34931000	-3.00052700	-2.87016300
H	-3.05619200	-4.57738300	-3.61851600
C	-2.26113200	-3.62493200	2.21474800

H	-3.28877600	-3.27961700	2.35179900
H	-2.04052500	-4.34935400	3.00073100
H	-1.58862500	-2.77329800	2.36534400
H	-6.75647500	0.77281900	-0.80229500
C	-4.20831700	1.37091700	-0.61124400
H	-3.15926400	1.07189700	-0.69667600
H	-4.66527400	1.26882100	-1.60150800
C	-4.22984000	2.81112500	-0.15251600
C	-5.15630200	3.72517500	-0.65481300
C	-3.28528700	3.24017700	0.78500800
C	-5.13575000	5.05335800	-0.23247700
H	-5.88509000	3.40115600	-1.39421900
C	-3.25538000	4.56807500	1.19776600
H	-2.54420700	2.53153000	1.15126100
C	-4.18434900	5.47717900	0.69099300
H	-5.85563800	5.75857900	-0.63531100
H	-2.49621800	4.89677500	1.90154600
H	-4.15868500	6.51480200	1.00799700
C	2.28475800	0.68399300	-0.21638600
C	1.11772500	0.11960500	-0.79944000
C	3.32443600	1.21472900	-1.15139600
C	3.91521100	0.34015000	-2.06865800
C	3.71057100	2.55722900	-1.12342600
C	4.90882400	0.79455900	-2.92877900
H	3.61700800	-0.70507500	-2.07265000
C	4.68631600	3.01385300	-2.00591300
H	3.25121500	3.23227900	-0.40870600
C	5.29303700	2.13430100	-2.89991200
H	5.38228400	0.10490500	-3.62055900
H	4.97668000	4.05952200	-1.99074100
H	6.06248300	2.49341800	-3.57615000
N	2.36812400	0.71293200	1.10582900
S	3.74857000	1.06457700	1.91428100
O	4.13928600	2.47920000	1.84510800
O	3.56152500	0.50863700	3.25752300
C	5.00336800	0.09777800	1.09414900
C	6.14304200	0.71587800	0.60240100
C	4.77722300	-1.26656100	0.90673500

C	7.07769900	-0.04893400	-0.09290800
H	6.27293000	1.78456500	0.73752200
C	5.71411700	-2.01001200	0.20397900
H	3.86680300	-1.73166400	1.27946800
C	6.87520500	-1.41311200	-0.30656100
H	7.96850000	0.42740900	-0.49268300
H	5.53775000	-3.07041900	0.04022000
C	7.87755700	-2.23825300	-1.07078400
H	8.36568200	-2.96408800	-0.41305300
H	7.38994200	-2.80115700	-1.87175900
H	8.65201100	-1.60977500	-1.51495300
H	0.13758100	1.10681000	-0.82693200
H	1.22934400	-0.15961800	-1.84865900
C	-0.14188300	3.85748700	-0.19292100
C	-0.44850300	5.07512300	-0.81762800
C	0.09822900	6.27337000	-0.31663600
C	0.92328300	6.17899400	0.78363700
C	1.21907300	4.93039300	1.39816000
C	0.69320000	3.74450400	0.93066800
H	-0.12555600	7.22582100	-0.78513800
H	1.36603200	7.07888300	1.19782200
H	1.88422900	4.91108100	2.25516700
H	0.92119800	2.78190600	1.37883700
N	-1.29488400	4.82619000	-1.86645500
N	-1.50723000	3.54112600	-1.91269500
N	-0.83216900	2.95505600	-0.93086000
O	-0.94867100	1.64754200	-0.69420700
H	-1.24203800	-0.55352000	-1.41775700

R2⁻

-1 1

C	1.89809600	-0.97557000	0.05663000
C	2.32671700	-2.24322900	0.25710400
C	2.90118000	0.13425600	-0.04763100
C	4.20462300	-0.08857900	-0.50862500
C	2.54148900	1.43907100	0.30962600
C	5.12704000	0.95096000	-0.57958000
H	4.49130400	-1.08250200	-0.83706800

C	3.46293200	2.48024600	0.24175400
H	1.52406100	1.61763600	0.64007900
C	4.76226500	2.24133200	-0.20023300
H	6.13052000	0.75505200	-0.94579000
H	3.16398800	3.48326400	0.53196200
H	5.48034500	3.05343900	-0.26034800
N	0.59674900	-0.50533900	-0.05374000
S	-0.59999200	-1.52401600	0.16311200
O	-0.77495300	-2.52404600	-0.91801000
O	-0.66959400	-2.11176700	1.52278900
C	-1.98610400	-0.40458100	0.02868800
C	-2.93925900	-0.59761100	-0.96165900
C	-2.12065600	0.63244800	0.94978600
C	-4.03304500	0.26393400	-1.03584200
H	-2.81349600	-1.41647800	-1.66181300
C	-3.21316500	1.48489800	0.86498500
H	-1.36853600	0.76609900	1.72102100
C	-4.18336900	1.31565500	-0.13130200
H	-4.78172200	0.11563700	-1.80954900
H	-3.32168200	2.29443000	1.58202900
C	-5.34839500	2.26598600	-0.23619400
H	-6.19325800	1.80055500	-0.74887000
H	-5.68173100	2.59436000	0.75134100
H	-5.06875400	3.16059700	-0.80235700
H	1.63581400	-3.07401000	0.33182700
H	3.38005700	-2.45525200	0.39065200

M06

0 1

C	-0.99591400	-1.27971300	0.65423000
O	-0.61611300	-1.96081400	1.58608200
C	-1.91138700	-0.14053800	0.74050200
C	-2.50139200	0.15930500	1.90794500
H	-2.03949800	0.46976000	-0.14793200
H	-2.32478300	-0.50251700	2.75485800
C	-3.36893500	1.31589400	2.13430400
C	-4.13487500	1.37458800	3.30546700
C	-3.44626900	2.37368800	1.21239000

C	-4.98266500	2.45198000	3.54537700
H	-4.06629800	0.56384600	4.02580500
C	-4.29196100	3.44713200	1.45615800
H	-2.81544800	2.36712200	0.32647200
C	-5.06498200	3.48797300	2.61889500
H	-5.57459600	2.48363500	4.45410400
H	-4.34339200	4.26356600	0.74319900
H	-5.72182900	4.33177100	2.80421100
C	0.49555900	1.64860700	1.27309400
C	-0.05612200	2.86730900	1.45217900
C	0.98491700	0.90487700	2.47372400
C	0.26205200	0.90417700	3.67221700
C	2.18640700	0.19464400	2.41850800
C	0.73528200	0.21241600	4.78414700
H	-0.68458900	1.43585900	3.71907100
C	2.66808400	-0.49278500	3.52915800
H	2.74342500	0.19857200	1.48802800
C	1.93903200	-0.48770500	4.71721900
H	0.15820000	0.21285800	5.70387700
H	3.60936000	-1.03313900	3.46055800
H	2.30405000	-1.02825500	5.58489900
N	0.69350600	0.91948600	0.09446000
S	0.38837400	1.60760400	-1.30982000
O	-0.88107800	2.36729100	-1.37685000
O	0.53881300	0.56383700	-2.34584500
C	1.70114800	2.78089900	-1.61961200
C	1.50710500	4.14262000	-1.42972500
C	2.94839000	2.28351200	-2.00195500
C	2.57792200	5.01609000	-1.61996600
H	0.52495000	4.51360800	-1.15911400
C	4.00337300	3.16427000	-2.18895100
H	3.07111500	1.21746800	-2.17308200
C	3.83523300	4.54278800	-1.99463100
H	2.43018500	6.08282200	-1.47770400
H	4.97422200	2.78473900	-2.49629600
C	4.99693300	5.48214000	-2.18989300
H	4.68127300	6.52435400	-2.11332600
H	5.45953100	5.33411200	-3.16969800

H	5.76945200	5.30556400	-1.43523700
H	-0.49323000	3.43787900	0.64523100
H	-0.07725300	3.29555000	2.44723100
C	-0.61274400	-1.68669000	-0.76445400
C	0.28646900	-2.24214000	-2.65747600
C	1.96444400	-1.87436900	-0.84364400
H	2.12078400	-0.79458800	-0.75713100
N	-0.98917900	-2.17422900	-2.90416300
N	-1.53537400	-1.83256200	-1.71369100
N	0.56450200	-2.00662800	-1.33498900
C	1.36129000	-2.49542900	-3.67019000
H	1.09903600	-1.97798700	-4.59345000
H	1.43449600	-3.57375000	-3.87116600
C	2.86945400	-2.48618300	-1.91691700
O	2.58328000	-1.98353600	-3.20402200
C	-2.96594500	-1.75821000	-1.56370900
C	-3.60551800	-2.73215200	-0.79251700
C	-3.64044000	-0.71609900	-2.21061200
C	-4.98643200	-2.60854900	-0.63568600
C	-5.01643600	-0.64147100	-2.01588900
C	-5.70227600	-1.56765400	-1.22516600
H	-5.51282000	-3.35107000	-0.04193300
H	-5.56784300	0.16392100	-2.49418600
H	3.90461200	-2.23183900	-1.69479900
C	-2.85927700	-3.87960700	-0.16045300
H	-2.06428800	-4.25085100	-0.81341700
H	-2.40158300	-3.59044000	0.79065200
H	-3.54622000	-4.70313800	0.03973400
C	-7.18661200	-1.43144700	-1.01057200
H	-7.39411300	-0.65835000	-0.26409700
H	-7.69176800	-1.13942100	-1.93426700
H	-7.62354100	-2.36666500	-0.65544600
C	-2.91430500	0.27118900	-3.08256900
H	-1.97497400	0.60576700	-2.63433900
H	-2.66993900	-0.18390600	-4.04747500
H	-3.54222200	1.14504800	-3.26354800
H	2.77228800	-3.58349200	-1.90111900
C	2.16016200	-2.57941800	0.49682100

H	1.53673700	-2.08821800	1.24740400
H	1.80255300	-3.61191500	0.41056700
C	3.58759900	-2.58147500	1.00552700
C	3.98650300	-3.60717000	1.86760400
C	4.49724600	-1.55407900	0.73233300
C	5.24778700	-3.60087400	2.45607300
H	3.29168100	-4.41281900	2.08943200
C	5.76196000	-1.54544000	1.31684800
H	4.22495300	-0.73971200	0.06496500
C	6.14064600	-2.56603000	2.18508100
H	5.53296500	-4.40542500	3.12634000
H	6.44942200	-0.73615300	1.09335200
H	7.12448400	-2.55777200	2.64236100

TS6(R)

0 1

C	-0.79694600	-1.16446600	0.76501900
O	0.04702100	-1.54630800	1.58724000
C	-1.84703600	-0.24202900	1.00435300
C	-1.86171600	0.41424800	2.22653300
H	-2.51520100	0.04319000	0.20155800
H	-1.28618700	-0.05044400	3.02347000
C	-2.99174800	1.26512300	2.65579300
C	-3.30322000	1.35867400	4.01585200
C	-3.76142500	1.98418600	1.73052400
C	-4.37762700	2.13226900	4.44651400
H	-2.70057200	0.81339900	4.73762900
C	-4.83090800	2.75933200	2.16269800
H	-3.49125200	1.95849100	0.67765000
C	-5.14631000	2.83207400	3.52011300
H	-4.61257700	2.18902300	5.50461700
H	-5.41856900	3.31599100	1.43948000
H	-5.98307300	3.43821100	3.85217100
C	0.67097600	1.43550100	1.34473500
C	-0.36706400	2.05290300	2.03180300
C	1.90560200	1.03851200	2.08372200
C	1.87237500	0.67440000	3.43496700
C	3.14242500	1.10738900	1.43203700

C	3.05146200	0.41598900	4.12590600
H	0.91637200	0.58477200	3.94207400
C	4.32342600	0.85963700	2.12691900
H	3.16015600	1.38608200	0.38197800
C	4.28222100	0.52453400	3.47830500
H	3.01149300	0.13249600	5.17301700
H	5.27740600	0.93182700	1.61357200
H	5.20303600	0.34157200	4.02382500
N	0.67417200	1.07937400	0.04336700
S	-0.18540900	1.81976500	-1.10661900
O	-1.45833800	2.43721200	-0.68859100
O	-0.26375900	0.89110100	-2.24384000
C	0.88963700	3.14840400	-1.61003800
C	0.62298400	4.45156600	-1.21318000
C	2.02517800	2.83526900	-2.35858000
C	1.50963500	5.46290700	-1.57966900
H	-0.27041900	4.66787000	-0.63698000
C	2.89671500	3.85496400	-2.71322700
H	2.20390000	1.80511000	-2.65689100
C	2.65186400	5.18136300	-2.33027300
H	1.30781600	6.48702000	-1.27875200
H	3.78251000	3.62524200	-3.29938900
C	3.60141600	6.27601300	-2.74196100
H	4.63510700	6.00625900	-2.50909100
H	3.36827500	7.21446700	-2.23522000
H	3.54551000	6.45121900	-3.82089400
H	-1.11911000	2.59653900	1.47915900
H	-0.20013700	2.35114000	3.06143700
C	-0.64594700	-1.61382000	-0.66869400
C	0.23749600	-1.88910000	-2.63245100
C	1.88281100	-1.79474500	-0.73758300
H	1.93994500	-0.96333600	-0.02962100
N	-1.04737300	-1.94233100	-2.83910700
N	-1.58677200	-1.77887300	-1.59971100
N	0.52598600	-1.75364700	-1.30630100
C	1.35626400	-1.78859000	-3.62266400
H	0.99691700	-1.28114700	-4.51808200
H	1.74166200	-2.78136200	-3.89459900

C	2.90241600	-1.63040300	-1.89246000
O	2.36714200	-0.99718300	-3.03640300
C	-2.99865500	-1.95586900	-1.38890400
C	-3.41098600	-3.07441300	-0.65988500
C	-3.88774400	-1.02004300	-1.93056800
C	-4.78238700	-3.23593700	-0.45972200
C	-5.24459900	-1.23394200	-1.70364300
C	-5.70947600	-2.32957400	-0.97133400
H	-5.13009100	-4.09643900	0.10584700
H	-5.95926800	-0.51989300	-2.10526100
H	3.73565000	-1.01734400	-1.54655600
C	-2.42801500	-4.07434100	-0.10472100
H	-1.64321700	-4.31022100	-0.83020100
H	-1.94137800	-3.69980300	0.80269300
H	-2.94214700	-5.00093000	0.15468100
C	-7.18744100	-2.53034700	-0.76103900
H	-7.66501300	-1.60234800	-0.43599700
H	-7.67121500	-2.83830600	-1.69326300
H	-7.37970300	-3.29938400	-0.01050200
C	-3.39960600	0.18186900	-2.69154200
H	-2.75660300	0.81212500	-2.06883500
H	-2.79876500	-0.11404000	-3.55503400
H	-4.24786600	0.77534800	-3.03676400
H	3.29390100	-2.61937400	-2.17550300
C	2.07895400	-3.14540400	-0.03047000
H	1.31415300	-3.22866300	0.74545400
H	1.92356400	-3.94469200	-0.76501100
C	3.45541100	-3.26456200	0.57630400
C	4.40681200	-4.13505500	0.03901100
C	3.79564900	-2.49335100	1.69263200
C	5.67448800	-4.24292700	0.60644500
H	4.14758900	-4.74184400	-0.82511100
C	5.06164900	-2.60189300	2.26099100
H	3.06088000	-1.81101600	2.11890600
C	6.00354400	-3.47671700	1.72132200
H	6.40081400	-4.92798000	0.18066300
H	5.31000600	-2.00089000	3.12958500
H	6.98829800	-3.56202000	2.16968900

TS6(S)

0 1

C	-0.58899200	-2.07313000	0.43127800
C	-0.02578900	-3.48360700	1.99413800
C	1.83604600	-2.90158600	0.40586500
N	-1.29621200	-3.26773000	2.18126200
N	-1.63381700	-2.38911400	1.20140600
N	0.45158100	-2.76871700	0.92895700
C	0.85224900	-4.45754400	2.71887400
H	1.30807300	-3.98149500	3.59803300
H	0.25362200	-5.30922600	3.04291800
C	2.61087100	-3.82974700	1.35231400
O	1.83681500	-4.91574100	1.82029700
C	-3.01303700	-2.00949500	1.02759700
C	-3.69406300	-2.49080400	-0.09485200
C	-3.58473200	-1.14571200	1.96504800
C	-4.98665900	-2.01615100	-0.30341600
C	-4.88185300	-0.70261200	1.70518600
C	-5.58472600	-1.10445100	0.56897000
H	-5.53707500	-2.36124900	-1.17519000
H	-5.34682700	-0.01543900	2.40731300
H	2.98724900	-3.24770300	2.20699200
C	-0.57619800	-1.25104100	-0.85061800
O	0.24565800	-1.60588900	-1.70332000
C	-1.56934800	-0.24228300	-0.95008800
C	-1.66629400	0.55944100	-2.06931500
H	-1.04697200	0.29350800	-2.92366100
C	-2.88559200	1.34114500	-2.36397000
C	-3.18246800	1.67973400	-3.68836800
C	-3.77198800	1.73380600	-1.34986300
C	-4.34777700	2.37471500	-4.00135600
H	-2.49478800	1.38670900	-4.47788200
C	-4.93183100	2.43226600	-1.66357500
H	-3.53649700	1.51253200	-0.31192600
C	-5.22816200	2.75009000	-2.99031500
H	-4.56701300	2.62222900	-5.03517600
H	-5.60598100	2.73711100	-0.86877400

H	-6.13592100	3.29364300	-3.23131800
C	-6.95242500	-0.55097200	0.26935400
H	-7.41798400	-0.13398600	1.16457200
H	-7.61044500	-1.32091500	-0.14059900
H	-6.87667100	0.24864400	-0.47541300
C	-3.06249700	-3.45626700	-1.06371700
H	-2.42744800	-4.18562200	-0.55322000
H	-2.44714100	-2.92948900	-1.80105800
H	-3.83720500	-3.99992300	-1.60682400
C	-2.84163900	-0.71444900	3.19903400
H	-2.78681800	-1.53927200	3.91670900
H	-3.35780300	0.12096900	3.67496200
H	-1.82034700	-0.40579600	2.96187400
H	3.46383500	-4.23906300	0.80911900
C	0.70030000	1.76260100	-1.11695100
C	-0.42622500	2.42744800	-1.56019100
C	1.87346900	1.58214700	-2.02497200
C	1.73633100	1.56819400	-3.41806000
C	3.15080200	1.42692300	-1.47408200
C	2.85026100	1.40854100	-4.23654300
H	0.75111000	1.66629000	-3.86485200
C	4.26464300	1.27226300	-2.29178800
H	3.25265300	1.42387100	-0.39293700
C	4.11859300	1.26204800	-3.67663000
H	2.72581200	1.38863600	-5.31482100
H	5.24449300	1.13103100	-1.84460300
H	4.98529300	1.12752100	-4.31656200
N	0.83579500	1.10472900	0.05220300
S	-0.00240600	1.38359300	1.39392800
O	0.20278700	0.19506900	2.24360500
O	-1.39860800	1.83674100	1.24402100
C	0.87545500	2.73416300	2.16095500
C	0.33571300	4.01403700	2.13185800
C	2.12625300	2.49416700	2.72810500
C	1.06050500	5.06748800	2.68531400
H	-0.64108800	4.17367800	1.68824200
C	2.83469500	3.55404500	3.27677300
H	2.52768700	1.48541600	2.73987000

C	2.31264000	4.85418000	3.26450900
H	0.64491800	6.07110100	2.66693600
H	3.80928600	3.37667700	3.72350900
C	3.08180300	5.98983300	3.88736700
H	2.96808800	5.98078100	4.97623700
H	2.72609100	6.95550400	3.52249400
H	4.14950400	5.90846900	3.66914300
H	-0.39154900	2.96920600	-2.49870500
H	-2.21802400	-0.06898200	-0.10471200
H	-1.20710400	2.69253700	-0.86178100
C	2.55958100	-1.55949600	0.28757200
H	1.90818500	-0.81683700	-0.17518900
H	2.79230000	-1.18701000	1.29176100
C	3.81298700	-1.71984900	-0.53938600
C	3.69541300	-2.00187400	-1.90483800
C	5.08552100	-1.57563300	0.01401700
C	4.82827000	-2.13854000	-2.69849400
H	2.70112200	-2.07406100	-2.34116700
C	6.22373000	-1.70635400	-0.78162200
H	5.18627700	-1.35154800	1.07331000
C	6.09748600	-1.99015200	-2.13872900
H	4.72256600	-2.34389200	-3.75936500
H	7.20784700	-1.58654800	-0.33903300
H	6.98279100	-2.09051000	-2.75882500
H	1.72785200	-3.35576300	-0.58120000

M6(R)

0 1

C	-3.24867700	-0.70583800	-0.13445400
C	-5.35106300	-0.11555000	-0.25607100
C	-3.68916400	1.76750700	-0.51344000
H	-3.23316800	2.11212600	0.41890100
N	-5.37418700	-1.40669400	-0.09637200
N	-4.05550600	-1.75591000	-0.03600500
N	-4.06236800	0.35355000	-0.30682300
C	-6.50231500	0.84215400	-0.33114000
H	-7.28029000	0.53744000	0.36935700
H	-6.92033100	0.85331000	-1.34694500

C	-4.98444900	2.54470700	-0.79030800
O	-6.04512700	2.12281300	0.04334800
C	-3.66195000	-3.12424600	0.16829400
C	-3.45470800	-3.92474600	-0.95564000
C	-3.52358700	-3.57840700	1.47979100
C	-3.10469100	-5.25374600	-0.72796100
C	-3.17128200	-4.91568100	1.65402100
C	-2.95870600	-5.76275000	0.56469100
H	-2.93675900	-5.90589200	-1.58118600
H	-3.05703200	-5.30203200	2.66343000
H	-4.81132500	3.60328700	-0.59452400
C	-1.73313400	-0.74768700	-0.14344900
O	-1.23763600	-1.37047100	-1.12989600
C	-1.14947400	-0.07327000	0.89237800
C	0.34811600	-0.00080100	1.02794800
H	-1.77477200	0.38870800	1.65089100
H	0.76868800	-1.01329000	1.09018200
C	0.79522800	0.76499200	2.25833300
C	1.83445600	0.27530100	3.05380500
C	0.24414800	2.01158400	2.57202800
C	2.31519300	1.01703000	4.13215300
H	2.27642100	-0.68712200	2.80769200
C	0.72557200	2.75767500	3.64339500
H	-0.57245600	2.40076100	1.96871900
C	1.76670600	2.26234200	4.42718600
H	3.12281000	0.62087600	4.74040800
H	0.28931800	3.72646400	3.86676200
H	2.14420800	2.84257800	5.26324600
C	-2.54568000	-7.19591900	0.77787300
H	-1.45466200	-7.28413800	0.76811900
H	-2.90040900	-7.57109800	1.74018000
H	-2.93584900	-7.84074900	-0.01273300
C	-3.58338400	-3.34888300	-2.33831600
H	-4.57669900	-2.91646100	-2.49525200
H	-2.83773000	-2.55595700	-2.46004700
H	-3.41790100	-4.11836500	-3.09366600
C	-3.73150700	-2.64719600	2.64389500
H	-4.71787100	-2.17536100	2.60248900

H	-3.64423200	-3.18562700	3.58836100
H	-2.97933200	-1.85072300	2.62986200
H	-5.26597600	2.42581700	-1.84810500
C	-2.70328800	1.92081900	-1.68011800
H	-1.84419700	1.26343900	-1.52352200
H	-3.19993000	1.60175700	-2.60258500
C	-2.25004600	3.35801400	-1.78873800
C	-2.67068600	4.17780500	-2.83741800
C	-1.41969300	3.89898900	-0.80188100
C	-2.26806300	5.51072900	-2.90085300
H	-3.31345500	3.76797200	-3.61198900
C	-1.02212800	5.23146700	-0.85749000
H	-1.07830700	3.26629700	0.01518400
C	-1.44588100	6.04147700	-1.91009600
H	-2.59920100	6.13426900	-3.72492000
H	-0.38260100	5.63336600	-0.07800000
H	-1.13633500	7.08032900	-1.95706600
C	2.41687900	0.97557300	-0.05826800
C	0.96500000	0.64207900	-0.26908100
C	2.75844200	2.38805400	0.27562500
C	2.13743100	3.45772900	-0.37821800
C	3.72147100	2.64692500	1.25720400
C	2.49188500	4.76715000	-0.06614800
H	1.39873800	3.27690400	-1.15326300
C	4.04624600	3.95621900	1.59123700
H	4.18389600	1.80978900	1.76873900
C	3.43686400	5.01901900	0.92580700
H	2.02836000	5.58967400	-0.60131700
H	4.77586400	4.14857100	2.37099600
H	3.69845600	6.04122300	1.17961700
N	3.41914600	0.16201900	-0.02602700
S	3.25777200	-1.48280200	-0.30530900
O	3.07284800	-2.13275900	0.99466800
O	2.32819900	-1.80001000	-1.38790100
C	4.89595600	-1.84592400	-0.87749700
C	5.96288800	-1.76276800	0.01663500
C	5.08144300	-2.23694600	-2.19620000
C	7.23719300	-2.07225500	-0.43343400

H	5.79031100	-1.46434700	1.04566700
C	6.36917800	-2.54654800	-2.62927100
H	4.22966800	-2.30126500	-2.86415700
C	7.45850700	-2.46677800	-1.76089700
H	8.07787300	-2.01297100	0.25189500
H	6.52680500	-2.85712000	-3.65766600
C	8.85284400	-2.78945000	-2.22920400
H	9.32678600	-3.51903300	-1.56707700
H	8.84729500	-3.19539600	-3.24224800
H	9.47782500	-1.89135700	-2.22553100
H	0.80486700	-0.06136300	-1.08760400
H	0.41385700	1.56251100	-0.47174000

M6(S)

0 1

C	-2.38438900	2.02506400	-0.07573100
C	-3.13441800	4.02065100	-0.55018100
C	-0.70614600	3.90164100	0.15419200
H	-0.57663000	3.74783800	1.22791800
N	-4.15967000	3.23463100	-0.71782400
N	-3.67086300	1.99076500	-0.42003800
N	-2.02332600	3.31679200	-0.17161100
C	-3.06083800	5.51480600	-0.65778200
H	-3.99330900	5.95548000	-0.30411000
H	-2.89846100	5.81232000	-1.70305300
C	-0.77687300	5.38973400	-0.20545800
O	-2.01276000	5.97274400	0.16814800
C	-4.51848100	0.82944700	-0.40440400
C	-4.88116400	0.25864900	-1.62659000
C	-4.89155200	0.30290400	0.83311800
C	-5.65402100	-0.89919300	-1.58459800
C	-5.66327600	-0.85988100	0.82044500
C	-6.04956300	-1.47220900	-0.37193900
H	-5.94813600	-1.36972700	-2.51956500
H	-5.96172500	-1.29987600	1.76832800
H	0.01285000	5.92210700	0.32652000
C	-1.51878100	0.94521100	0.51361300
O	-0.95293900	1.31075900	1.59616800

C	-1.46061200	-0.21304800	-0.19996500
C	-0.57585300	-1.33097900	0.25434600
H	-2.05007400	-0.32140900	-1.10668000
H	0.12340300	-0.89575900	0.97684200
C	-1.30300100	-2.46723900	0.95705500
C	-0.64157900	-3.21937700	1.93197500
C	-2.60836500	-2.82191500	0.60424800
C	-1.26169000	-4.31435400	2.52975000
H	0.36946400	-2.93816600	2.22145400
C	-3.23133600	-3.91631000	1.20044000
H	-3.14551200	-2.22422400	-0.12905400
C	-2.55781900	-4.66963500	2.16096200
H	-0.73439400	-4.88780100	3.28579200
H	-4.24826500	-4.17570800	0.91885400
H	-3.04356400	-5.52116500	2.62682400
C	-6.88453800	-2.72668400	-0.36407800
H	-6.44196600	-3.49380100	-1.00566800
H	-6.97855400	-3.13290200	0.64494400
H	-7.89109200	-2.52561700	-0.74335600
C	-4.43025500	0.86988600	-2.92639500
H	-4.85669700	1.86825200	-3.05925800
H	-3.34046400	0.97726500	-2.95199700
H	-4.73377100	0.24764200	-3.76923300
C	-4.46748700	0.95612300	2.12204000
H	-4.72770100	2.01947800	2.12267600
H	-4.96517000	0.48087300	2.96838900
H	-3.38484800	0.88317300	2.27303000
H	-0.61859600	5.51683200	-1.28738200
C	0.42412700	3.19450500	-0.60608100
H	0.27786000	2.11209300	-0.56510500
H	0.39322800	3.50092600	-1.65800400
C	1.76967500	3.50655500	0.00405000
C	2.67980500	4.36759900	-0.60948500
C	2.11784500	2.89289100	1.21372800
C	3.92477200	4.61433700	-0.02659600
H	2.41921300	4.84088400	-1.55328600
C	3.35765200	3.13725800	1.79374700
H	1.41319100	2.19676900	1.66483800

C	4.26449100	4.00075000	1.17559100
H	4.62647500	5.28322900	-0.51534200
H	3.62469100	2.63877600	2.72041700
H	5.23546800	4.18398700	1.62498000
C	1.46968000	-2.65604200	-0.59119900
C	0.21768200	-1.89572000	-0.96042600
C	1.53320500	-4.12290300	-0.83752500
C	0.37094600	-4.90283700	-0.88056700
C	2.77976100	-4.74091700	-1.00741900
C	0.45872200	-6.27747300	-1.08109700
H	-0.60284400	-4.44959300	-0.72434400
C	2.86148500	-6.10909800	-1.22787000
H	3.67555600	-4.12992400	-0.97506500
C	1.70001000	-6.88133700	-1.26183600
H	-0.44622400	-6.87544700	-1.09629400
H	3.82998700	-6.57577600	-1.37343800
H	1.76421900	-7.95157300	-1.42956700
N	2.53791000	-2.13913500	-0.08052200
S	2.67708200	-0.51167600	0.29325800
O	1.92590800	0.36992500	-0.60160700
O	2.44936200	-0.40348100	1.73515900
C	4.39575900	-0.24425000	-0.04532100
C	4.75318800	1.00234200	-0.55156300
C	5.35435500	-1.19757000	0.29161800
C	6.10198400	1.28867700	-0.73183500
H	3.98639200	1.73494300	-0.78807100
C	6.69548100	-0.89452500	0.09225000
H	5.05032500	-2.15851400	0.69231800
C	7.08771100	0.34760900	-0.42119900
H	6.39157000	2.26255400	-1.11655700
H	7.45296100	-1.63107200	0.34365000
C	8.54310200	0.65451700	-0.65643100
H	8.74409200	1.72209900	-0.54392700
H	9.18063400	0.10454400	0.03902800
H	8.83537300	0.36790700	-1.67188300
H	0.52509800	-1.05158000	-1.57995500
H	-0.43866700	-2.53040800	-1.55930500

TS7(S)^{HOAr}

0 1

C	-2.41834300	-1.49839000	-0.49410100
C	-3.67024200	-2.88033300	-1.60730700
C	-4.94509000	-1.15068800	-0.28033000
H	-4.96266800	-1.32360800	0.79828700
N	-2.44257300	-3.25145200	-1.85804000
N	-1.67888600	-2.37202900	-1.16704000
N	-3.70012600	-1.79589300	-0.77289300
C	-4.94665700	-3.53474100	-2.04262700
H	-4.80306900	-4.61491900	-2.08012200
H	-5.23716800	-3.17505200	-3.03898300
C	-6.12852700	-1.85109800	-0.96686900
O	-5.93614100	-3.24561300	-1.08307400
C	-0.25053600	-2.53065400	-1.05390500
C	0.56252900	-2.07469200	-2.09178700
C	0.23226200	-3.09805800	0.12887400
C	1.93922400	-2.17731800	-1.89746100
C	1.61418100	-3.15036000	0.28087100
C	2.47850800	-2.67970300	-0.71142700
H	2.60482200	-1.82903100	-2.68358200
H	2.02539100	-3.56776400	1.19632400
H	-7.02390200	-1.68813300	-0.36632700
C	-1.91994000	-0.48052300	0.51951000
O	-2.62450500	-0.31725700	1.48955800
C	-0.64160100	0.25212900	0.23567800
C	-0.71154000	1.71334800	0.73745300
H	-0.42195900	0.22525000	-0.83644700
H	-0.82956900	1.67461000	1.82808300
C	-1.92072300	2.41232100	0.14479600
C	-2.93246700	2.91666900	0.96523300
C	-2.04120800	2.55578600	-1.24310000
C	-4.04491200	3.55173000	0.41517600
H	-2.84888900	2.80275100	2.04298800
C	-3.15666000	3.18324700	-1.79317100
H	-1.24526200	2.19145800	-1.89028600
C	-4.16315800	3.68048400	-0.96644300
H	-4.82965400	3.92566600	1.06573900

H	-3.23815800	3.28890100	-2.87084800
H	-5.03542600	4.16314700	-1.39576600
C	3.96369000	-2.68130700	-0.47348000
H	4.52504200	-2.60950500	-1.40841800
H	4.23134800	-1.81425900	0.14300300
H	4.27641300	-3.57975600	0.06546800
C	-0.01087900	-1.43581200	-3.32803800
H	-0.94740600	-1.90536500	-3.63685100
H	-0.19592300	-0.36930600	-3.14391900
H	0.70309000	-1.50333100	-4.15029100
C	-0.69817000	-3.61515900	1.19606200
H	-1.55785400	-4.13567000	0.76394600
H	-0.16718500	-4.31346200	1.84512000
H	-1.07525900	-2.80637900	1.83356900
H	-6.28822900	-1.40880000	-1.96184000
C	-4.96336800	0.34944700	-0.59239600
H	-4.05766600	0.83097400	-0.21583500
H	-4.98322800	0.48650700	-1.67883800
C	-6.16677700	0.99615700	0.05206400
C	-7.24432400	1.44787600	-0.70915600
C	-6.20395900	1.14653000	1.44218300
C	-8.34364100	2.04471200	-0.09338000
H	-7.21822300	1.34309600	-1.79078000
C	-7.30107000	1.73854700	2.05880500
H	-5.35315000	0.81894200	2.03680100
C	-8.37406800	2.19032500	1.29050200
H	-9.17400200	2.39691700	-0.69686600
H	-7.31651400	1.85624000	3.13753100
H	-9.22798700	2.65714200	1.77036300
C	1.73550900	2.26593000	1.17170600
C	0.56254400	2.48014000	0.38142500
C	2.82030700	3.28662200	1.02506800
C	3.09916100	3.83087900	-0.23570600
C	3.49224300	3.78405600	2.14678100
C	4.05969300	4.82613300	-0.37421400
H	2.56768600	3.46604700	-1.10923400
C	4.44183200	4.79198600	2.00403000
H	3.26623400	3.38862400	3.13007800

C	4.73459900	5.30890300	0.74528600
H	4.27920900	5.22642700	-1.35865700
H	4.95177400	5.17389200	2.88247600
H	5.48187000	6.08823500	0.63569800
N	1.79005000	1.18521000	1.93058900
S	3.17443200	0.61792900	2.59747100
O	4.28787800	0.55650200	1.63830500
O	3.45440000	1.25035900	3.89552100
C	2.70004600	-1.05751700	2.96951100
C	3.71417700	-2.00862100	2.99713500
C	1.40921100	-1.37150800	3.39303900
C	3.43165300	-3.29272700	3.45764000
H	4.71271600	-1.74194700	2.66555600
C	1.14686100	-2.65559600	3.85413400
H	0.63068500	-0.61540600	3.36214800
C	2.15041600	-3.63232700	3.89753600
H	4.22124600	-4.03845600	3.48089200
H	0.14562300	-2.90749500	4.19428900
C	1.83905500	-5.01740600	4.40104600
H	1.10210200	-5.51072900	3.75892700
H	2.73476000	-5.64041900	4.42786800
H	1.41622600	-4.97827400	5.40882400
H	0.93507400	2.02337000	-0.85869900
H	0.38128000	3.53502000	0.17190700
H	0.18675300	-0.26643700	0.74157900
O	1.04643900	1.63352900	-2.06254500
C	2.19039000	1.10729700	-2.41958800
C	2.44622700	0.86149500	-3.79501600
C	3.20597800	0.76939400	-1.48724300
C	3.63360800	0.29562900	-4.21275200
H	1.67537700	1.12502500	-4.51172000
C	4.40217300	0.22541600	-1.90458700
H	3.04568700	0.91180800	-0.42582400
C	4.60632100	-0.02845400	-3.26266600
H	3.82422200	0.09538900	-5.26050500
H	5.17366700	-0.02034600	-1.18442300
N	5.83225100	-0.65662700	-3.68889700
O	6.61171600	-1.05664700	-2.83075600

O	6.04232800	-0.77638400	-4.89034900

TS7(R)^{HOAr}

0 1

C	-2.54008700	0.10472600	-0.92795100
C	-4.27264000	-1.17859200	-1.21931300
C	-2.04636800	-2.32573700	-1.54638900
H	-1.62885900	-2.12468500	-2.53596500
N	-4.76582400	-0.00447500	-0.94161500
N	-3.67107800	0.78795900	-0.75804600
N	-2.90453200	-1.15300600	-1.22627900
C	-5.01291500	-2.44748900	-1.52341800
H	-5.90134600	-2.22178800	-2.11395800
H	-5.31273200	-2.93088800	-0.58124600
C	-2.97246600	-3.54645900	-1.57498600
O	-4.17237300	-3.29019800	-2.27818600
C	-3.82463400	2.19290200	-0.47322100
C	-4.58416300	2.56095400	0.65082200
C	-3.22351000	3.12911800	-1.32199100
C	-4.66860300	3.91859400	0.94290500
C	-3.35045600	4.47672900	-0.97687900
C	-4.04626900	4.88968900	0.15402200
H	-5.24187000	4.22642800	1.81366400
H	-2.88164600	5.21782900	-1.61915000
H	-2.45659700	-4.36731300	-2.07502200
C	-1.09467700	0.58338800	-0.95507300
O	-0.47916200	0.17010900	-1.93503600
C	-0.61164700	1.33295000	0.16591200
C	0.75399800	1.96531800	-0.09212100
H	-1.37270700	1.98124300	0.60953200
H	1.18775900	1.38194800	-0.90832000
C	0.69352800	3.40168700	-0.57724200
C	1.51361300	3.80450600	-1.63598900
C	-0.11341100	4.35890100	0.04645400
C	1.52893100	5.13040400	-2.06374800
H	2.14494600	3.06539600	-2.12477700
C	-0.10059900	5.68524600	-0.37912300
H	-0.75914300	4.07027900	0.87289300

C	0.72126000	6.07528800	-1.43535300
H	2.16978300	5.42388800	-2.88914200
H	-0.73776900	6.41460700	0.11322900
H	0.72977700	7.10861100	-1.76706300
C	-4.12853700	6.34535200	0.52936500
H	-3.49767000	6.55202400	1.39998300
H	-3.79350700	6.98467500	-0.28974900
H	-5.15128500	6.62591600	0.79373500
C	-5.33389900	1.56973200	1.50420000
H	-6.24380200	1.23952600	0.99626000
H	-4.75124400	0.67405100	1.72704200
H	-5.61528700	2.03451000	2.45009900
C	-2.45718000	2.77294000	-2.56982500
H	-2.74607000	1.80583100	-2.98727900
H	-2.63296200	3.53579400	-3.33072700
H	-1.37974800	2.75062300	-2.37074400
H	-3.20532900	-3.84810500	-0.54090600
C	-0.92848400	-2.54330700	-0.51964200
H	-0.45558400	-1.60067900	-0.23701400
H	-1.36515400	-2.98145700	0.38089700
C	0.15145700	-3.44545500	-1.06578600
C	0.32374100	-4.74231200	-0.58216800
C	1.02312200	-2.95996700	-2.04775300
C	1.35249700	-5.54868000	-1.07088100
H	-0.34495300	-5.11814900	0.18926000
C	2.04638600	-3.76390600	-2.53721800
H	0.91849400	-1.93158600	-2.38711600
C	2.21235900	-5.06180900	-2.05113200
H	1.48030300	-6.55436000	-0.68265600
H	2.73079100	-3.36878100	-3.28163600
H	3.01863600	-5.68474400	-2.42556200
C	3.16442000	1.84045600	0.75120000
C	1.70241600	1.84737600	1.13162200
C	4.03675800	2.96692300	1.18211600
C	3.51182000	4.25078000	1.37149800
C	5.40874500	2.75111700	1.36656600
C	4.35050600	5.30154000	1.73214700
H	2.45582800	4.44337700	1.20772300

C	6.23784500	3.79732100	1.74816200
H	5.80694000	1.75284800	1.21768000
C	5.71020800	5.07633800	1.92837600
H	3.93786400	6.29655900	1.86095700
H	7.29623300	3.61787000	1.90564000
H	6.35905500	5.89520400	2.22205100
N	3.76178800	0.91116100	0.08349800
S	2.98847100	-0.43684000	-0.55344000
O	1.76377100	-0.83232700	0.14329600
O	2.92040800	-0.18960100	-1.99530700
C	4.20620400	-1.68198500	-0.22738900
C	3.76526400	-2.92093200	0.22251800
C	5.55046500	-1.44576000	-0.51716500
C	4.69734800	-3.94121700	0.39340200
H	2.70927200	-3.08353000	0.41902500
C	6.46350700	-2.47323500	-0.33077700
H	5.86888100	-0.47146900	-0.87292200
C	6.05148800	-3.73299700	0.12621300
H	4.36019300	-4.91596100	0.73385200
H	7.51428700	-2.30164500	-0.54650600
C	7.06271800	-4.82938300	0.33356800
H	7.74216700	-4.90107000	-0.51964800
H	7.67167600	-4.62675600	1.22019800
H	6.57661700	-5.79680500	0.47238600
H	1.50894400	0.89834100	1.63611200
H	1.50702100	2.64925600	1.84630700
H	-0.48054100	0.46905300	1.22841700
O	-0.29409700	-0.18200200	2.29459500
C	-1.18825700	-1.11353600	2.42230900
C	-2.53751100	-0.89404600	2.02983000
C	-0.85102500	-2.39654500	2.93476500
C	-3.45711400	-1.92184500	2.00858900
H	-2.81708500	0.11399700	1.74301600
C	-1.77650600	-3.41845300	2.94870000
H	0.16645200	-2.55983700	3.27229300
C	-3.06295500	-3.19008400	2.44238300
H	-4.48151100	-1.76283300	1.68442500
H	-1.51704100	-4.40821600	3.30623700

N	-4.00088800	-4.28053600	2.36043300
O	-3.72674700	-5.33316300	2.91946300
O	-5.03460500	-4.11114000	1.71650400

TS7(S)$^{DH+}$

1 1

C	3.33970300	1.51866500	0.57465600
C	5.32243200	2.05260900	1.28356000
C	5.22195000	-0.14777800	0.07909400
N	4.55707800	3.06136100	1.60616800
N	3.32500700	2.70945700	1.16243100
N	4.60972500	1.08632100	0.62923600
C	6.77369800	1.84491700	1.61301200
H	7.33943900	2.74879200	1.38436600
H	6.85695400	1.63518000	2.68852500
C	6.49197800	-0.37369500	0.90113200
O	7.30042200	0.78975400	0.84398700
C	2.23741400	3.65414700	1.20530300
C	1.69408000	3.97121100	2.45332700
C	1.82840600	4.23761500	0.00195200
C	0.67781000	4.92197800	2.47122800
C	0.81927300	5.19435800	0.08456300
C	0.23791500	5.54795300	1.30199200
H	0.22358200	5.18504200	3.42298900
H	0.47262800	5.66687400	-0.83013500
H	7.06405800	-1.18952100	0.45688700
C	2.18091500	0.75350400	-0.02771800
O	2.39605500	0.17401000	-1.06711000
C	0.90377100	0.65035000	0.75926100
C	-0.33660100	0.49034500	-0.13995800
H	0.80381000	1.48329700	1.46205100
H	-0.01389000	0.00442700	-1.06570800
C	-0.92381500	1.84145200	-0.52550800
C	-0.80600400	2.32882700	-1.82882300
C	-1.64899000	2.59241300	0.40648000
C	-1.40422900	3.53475400	-2.19737300
H	-0.26857700	1.74117600	-2.56927600
C	-2.26674300	3.78318000	0.03829500

H	-1.75066400	2.22804200	1.42668500
C	-2.14774400	4.25885000	-1.26803300
H	-1.31173500	3.89193700	-3.21824500
H	-2.84046100	4.34332600	0.77141400
H	-2.63459000	5.18492500	-1.55941700
C	-0.83307500	6.60349500	1.35576300
H	-1.55074500	6.39835800	2.15351500
H	-1.37127300	6.66119300	0.40700600
H	-0.39047800	7.58542800	1.55171900
C	2.18794300	3.31083700	3.71289400
H	3.20481100	3.63524100	3.95130600
H	2.20720600	2.22052800	3.61188500
H	1.54091100	3.56444500	4.55300700
C	2.44922100	3.87877600	-1.32367900
H	3.53513800	3.76583600	-1.24830000
H	2.24086100	4.66080300	-2.05493500
H	2.03463200	2.94719900	-1.72392500
H	6.24831800	-0.62842100	1.94076600
C	-2.51670700	-0.73025000	-0.26078600
C	-1.37066100	-0.41042800	0.52779400
C	-3.75903900	-1.10797600	0.48194900
C	-4.33420900	-0.18089100	1.35788000
C	-4.35013700	-2.36267000	0.31403200
C	-5.50510600	-0.49551000	2.03806800
H	-3.87930000	0.80048800	1.46556800
C	-5.50789200	-2.68411000	1.01910200
H	-3.91152200	-3.07155900	-0.37937000
C	-6.09011400	-1.75091200	1.87373200
H	-5.96136400	0.23673800	2.69663400
H	-5.95792500	-3.66399600	0.89395000
H	-6.99947000	-2.00018400	2.41116000
N	-2.39226200	-0.70954000	-1.57406200
S	-3.66931000	-0.82846900	-2.60406200
O	-4.23523100	-2.18239600	-2.67192800
O	-3.19961500	-0.24834000	-3.86341300
C	-4.91221200	0.25791600	-1.93303800
C	-6.17744900	-0.23284500	-1.63639300
C	-4.56232600	1.57826700	-1.66238500

C	-7.10814000	0.61961700	-1.05203400
H	-6.40793400	-1.27403000	-1.83577200
C	-5.50394600	2.41426900	-1.07450100
H	-3.56082200	1.94087600	-1.88215800
C	-6.78516800	1.94877400	-0.76037300
H	-8.09646700	0.24339600	-0.80190200
H	-5.23446200	3.44273400	-0.84809000
C	-7.80666800	2.85988800	-0.13144400
H	-8.39331000	2.33004600	0.62335300
H	-8.50572500	3.23599500	-0.88529700
H	-7.33150400	3.72197000	0.34137500
H	-0.72319800	-1.65401600	0.65099500
H	-1.61256200	-0.14952800	1.56119700
H	1.04479700	-0.26009700	1.36582700
C	-2.28785500	-5.96489300	2.82882400
C	-2.52607600	-4.61718300	2.14874000
C	-1.44754900	-3.55356700	2.42742800
C	-1.01666000	-6.68903400	2.38772000
C	-0.24031600	-3.69479300	1.53325800
C	0.24698700	-5.85922300	2.61160500
H	-2.24100300	-5.80584300	3.91469800
H	-3.48258700	-4.21357700	2.49347700
H	-1.13780300	-3.59743600	3.47841500
H	-1.07450900	-6.96055700	1.32629000
H	1.13232900	-6.49865600	2.59515300
C	1.15143500	-2.82392700	-0.20887300
H	0.95779400	-2.16504300	-1.05968600
C	1.49431800	-5.14450100	0.57869100
C	1.32511300	-4.27015900	-0.65232700
H	0.43789800	-4.58101500	-1.21217300
H	2.19497000	-4.37924300	-1.30391100
H	1.39000400	-6.20161600	0.31541400
N	0.46933400	-4.83621400	1.58352600
N	0.03030600	-2.71495400	0.71504200
H	0.22231100	-5.38484600	3.59888500
H	-0.92140500	-7.62222800	2.95222900
H	-3.15204300	-6.61121200	2.64568200
H	-2.62211900	-4.75181300	1.06413400

H	-1.86251700	-2.55784500	2.25515500
H	2.07262400	-2.47073700	0.27792300
H	2.48722500	-5.00793000	1.02429500
C	5.52126300	0.04206700	-1.41643100
H	4.64164400	0.47826000	-1.89511900
H	6.35243400	0.74755600	-1.51040000
C	5.85693700	-1.28202800	-2.05776400
C	4.82895200	-2.15951500	-2.41810200
C	7.18246100	-1.66351800	-2.27237400
C	5.12384000	-3.39456400	-2.98726900
H	3.79406600	-1.85986400	-2.26354800
C	7.47942400	-2.90102700	-2.84002200
H	7.98571400	-0.98244900	-2.00153000
C	6.45067000	-3.76805600	-3.19806400
H	4.31858800	-4.06236900	-3.27666100
H	8.51368700	-3.18343000	-3.00767500
H	6.67965800	-4.72934900	-3.64633700
H	4.51057800	-0.96330200	0.23356100

TS7(R)$^{DH+}$

1 1

C	-2.85765200	2.37167500	-0.10479000
C	-4.84251500	3.10403200	0.36265000
C	-4.78169200	0.82425600	-0.74392100
H	-4.69253600	0.91339100	-1.83017000
N	-4.02786700	4.04486400	0.75774700
N	-2.79197600	3.56593600	0.46274600
N	-4.15436700	2.04663800	-0.17777000
C	-6.34131600	3.07264100	0.41731400
H	-6.73776600	4.05694000	0.16603100
H	-6.66663600	2.80297700	1.43115500
C	-6.25919900	0.86316400	-0.33614500
O	-6.80959400	2.14886900	-0.53683800
C	-1.58073600	4.30999800	0.70122400
C	-1.19186200	4.49769800	2.03467700
C	-0.82963400	4.74322900	-0.39487000
C	0.02086300	5.14012400	2.25180100
C	0.38213600	5.37813600	-0.11299700

C	0.82090500	5.58248600	1.19158100
H	0.35684600	5.29400600	3.27399400
H	0.99655700	5.71462400	-0.94378300
H	-6.81532500	0.16619300	-0.96347000
C	-1.68934500	1.58217600	-0.65713000
O	-1.72924100	1.31871700	-1.83458400
C	-0.66651400	1.11552900	0.33283500
C	0.60377600	0.59482800	-0.34418800
H	-0.46145700	1.90320700	1.06761200
H	0.30820800	0.18104100	-1.31409700
C	1.56745600	1.73829400	-0.62074500
C	1.73118600	2.23481600	-1.91535800
C	2.31773000	2.30312700	0.41602700
C	2.62773100	3.27266900	-2.17212400
H	1.16737800	1.78654600	-2.72982600
C	3.22135600	3.33062800	0.16282800
H	2.20122900	1.92562000	1.42977300
C	3.38060900	3.81846300	-1.13460900
H	2.75074700	3.64048900	-3.18596500
H	3.80609600	3.74993300	0.97661200
H	4.09018200	4.61571200	-1.33452400
C	2.11910800	6.29193800	1.46655600
H	2.66162800	5.81048200	2.28407500
H	2.75765300	6.29979000	0.58092300
H	1.93103800	7.32919900	1.76147700
C	-2.04325000	4.00989300	3.17671800
H	-2.95941700	4.60051000	3.26269100
H	-2.34054000	2.96552400	3.03675800
H	-1.49551300	4.08525700	4.11653600
C	-1.26615200	4.56991100	-1.82730500
H	-2.34375200	4.41999200	-1.92471000
H	-0.99842000	5.46010900	-2.39930600
H	-0.75599000	3.71883100	-2.28873700
H	-6.36621800	0.55473300	0.71521400
C	-4.08754300	-0.44912100	-0.24211700
H	-3.03187100	-0.44389300	-0.53077100
H	-4.13312500	-0.47812000	0.85315400
C	-4.74041700	-1.67881900	-0.83245600

C	-5.45142500	-2.56871800	-0.02662000
C	-4.65400400	-1.92517500	-2.20740800
C	-6.06206800	-3.69234600	-0.58186100
H	-5.52512400	-2.38242100	1.04196600
C	-5.27402600	-3.03994300	-2.76548300
H	-4.09281500	-1.24471200	-2.84465500
C	-5.97882300	-3.92671500	-1.95251900
H	-6.60852500	-4.37904700	0.05676000
H	-5.20020700	-3.21957100	-3.83283800
H	-6.45772900	-4.79817400	-2.38614600
C	2.32640300	-1.19650500	-0.13385400
C	1.21525900	-0.53691700	0.46823400
C	3.26513000	-1.91663300	0.78023000
C	3.95843300	-1.18916600	1.75273200
C	3.46021500	-3.29586200	0.67338900
C	4.86575500	-1.83056200	2.58854900
H	3.81158000	-0.11396600	1.81643000
C	4.34731600	-3.93879800	1.53226400
H	2.92941200	-3.85250900	-0.09090300
C	5.05822200	-3.20715500	2.48121700
H	5.42336900	-1.25779200	3.32291200
H	4.49133300	-5.01150700	1.45283700
H	5.76199400	-3.70909700	3.13763300
N	2.43239000	-1.15334000	-1.45010600
S	3.77000600	-1.64583000	-2.26707500
O	3.94521600	-3.10414700	-2.27383300
O	3.68400800	-0.98807000	-3.57226000
C	5.13483000	-0.92548400	-1.37766400
C	6.14128300	-1.74071500	-0.87695000
C	5.12943000	0.44910200	-1.15008400
C	7.16157300	-1.16435800	-0.12739900
H	6.10077900	-2.81052100	-1.05311200
C	6.15367600	1.00592600	-0.39396700
H	4.32653000	1.07107800	-1.54053800
C	7.17767800	0.20922700	0.13121500
H	7.95052300	-1.79320200	0.27605800
H	6.15356100	2.07630700	-0.20486700
C	8.25860400	0.81148900	0.99029200

H	8.00945200	0.70250700	2.05129700
H	9.21727600	0.31395200	0.82623300
H	8.38163600	1.87707900	0.78531900
H	0.15975800	-1.46067800	0.35787300
H	1.33556600	-0.35296900	1.53798800
H	-1.17900100	0.30622800	0.87912600
C	-1.64401400	-4.66001200	3.67881400
C	-0.68762700	-3.57174600	3.18816200
C	-1.36209200	-2.45466000	2.37050200
C	-2.35627200	-5.44209900	2.57526100
C	-1.55483000	-2.83331800	0.92364900
C	-3.16826800	-4.55013100	1.63502000
H	-2.40304600	-4.19150200	4.31986100
H	-0.21215900	-3.10889400	4.05774600
H	-2.32683100	-2.18420200	2.81784800
H	-1.63432100	-6.01443100	1.97945000
H	-3.90229500	-5.13819400	1.07888800
C	-0.96666000	-2.51680200	-1.38302000
H	-0.03986400	-2.18430500	-1.85852800
C	-2.38952000	-4.43893400	-0.72850000
C	-1.16579100	-4.01962700	-1.52594800
H	-0.27808600	-4.53599200	-1.14587100
H	-1.29787700	-4.29848200	-2.57340800
H	-2.42353100	-5.52765000	-0.62341900
N	-2.34960700	-3.87133900	0.62562500
N	-0.88967700	-2.14890200	0.02898600
H	-3.73717800	-3.80721900	2.20561000
H	-3.03635700	-6.16644000	3.03478800
H	-1.08941100	-5.35839000	4.31328400
H	0.11939000	-4.01181800	2.58899900
H	-0.73889800	-1.55789900	2.38367800
H	-1.79915000	-1.97933300	-1.85799000
H	-3.31474000	-4.11679300	-1.22048700

TS7(S)^{DT}

0 1

C	1.19937200	0.56688800	1.28468200
C	2.37761400	-0.41916400	2.84094300

C	3.59289200	-0.11877800	0.66187700
H	3.26967500	-0.71359600	-0.19274900
N	1.24460600	-0.12848300	3.41279500
N	0.52979700	0.49798600	2.44113400
N	2.40818800	0.01806000	1.54530300
C	3.51191500	-1.22164200	3.40266500
H	3.11250900	-2.00617200	4.04638200
H	4.18663200	-0.58361600	3.98894500
C	4.70021200	-0.83928300	1.45149300
O	4.19195400	-1.82744500	2.32694300
C	-0.78630100	0.98809400	2.75964900
C	-0.91519400	2.32374200	3.13917300
C	-1.85773000	0.09942000	2.66760500
C	-2.20508900	2.78321000	3.40554900
C	-3.12458100	0.60580500	2.94922700
C	-3.31644100	1.94270500	3.31138700
H	-2.34283600	3.82208700	3.69301500
H	-3.98300700	-0.05924100	2.88652200
H	5.36466000	-1.33539300	0.74385400
C	0.72904500	0.97344200	-0.10606700
O	1.44998000	0.57257000	-1.03351900
C	-0.52528700	1.62185400	-0.19168100
C	-1.18635400	1.86735800	-1.55305200
H	-0.84001700	2.27019000	0.61999300
H	-0.53077200	1.50613600	-2.34783900
C	-1.60053500	3.29698400	-1.80585100
C	-1.20438400	3.95836900	-2.96982700
C	-2.39584900	3.98704600	-0.88274800
C	-1.58983600	5.27627600	-3.20912600
H	-0.58831100	3.43325100	-3.69440300
C	-2.78302700	5.30255600	-1.11818400
H	-2.71768100	3.49084900	0.03152400
C	-2.38029800	5.95261900	-2.28413800
H	-1.27159300	5.77344100	-4.12009500
H	-3.40056300	5.82167500	-0.39181600
H	-2.68182200	6.97856300	-2.46897700
C	-4.69749600	2.45427800	3.62586500
H	-4.74809300	3.54054500	3.53071500

H	-5.44093900	2.01086500	2.95970300
H	-4.97662000	2.19420400	4.65174400
C	0.28416100	3.23143900	3.20723300
H	1.09041100	2.77786500	3.79081600
H	0.67642600	3.43791800	2.20470500
H	0.01801200	4.18410400	3.66597900
C	-1.62766800	-1.32378200	2.24685500
H	-1.25169300	-1.37438800	1.21543200
H	-0.88070700	-1.80352100	2.88552300
H	-2.55032400	-1.90471500	2.29555600
H	5.28539600	-0.10593000	2.02603100
C	4.08992500	1.25253200	0.18564000
H	3.24944900	1.81402700	-0.22356300
H	4.49259300	1.80308500	1.04275100
C	5.14342600	1.07174400	-0.88190800
C	6.49693100	1.28206200	-0.61635600
C	4.75325400	0.65166600	-2.15805300
C	7.45106200	1.07936500	-1.61229700
H	6.80466500	1.61268000	0.37253200
C	5.70516000	0.44811700	-3.15188400
H	3.69577100	0.49466800	-2.36070800
C	7.05661400	0.66151900	-2.88040300
H	8.50091500	1.24983400	-1.39660200
H	5.39277700	0.12594900	-4.13989500
H	7.79859200	0.50441000	-3.65647700
C	-2.34308300	-0.41587400	-1.87858700
C	-2.35982300	0.89531200	-1.35578800
C	-3.51566500	-1.27710100	-1.53567300
C	-4.16179300	-2.03893700	-2.51613200
C	-4.02794300	-1.27261700	-0.23322500
C	-5.27945500	-2.79928900	-2.18796900
H	-3.77934700	-2.03491300	-3.52903000
C	-5.13714100	-2.04489600	0.09694300
H	-3.54606100	-0.65883900	0.52070700
C	-5.76336900	-2.81482400	-0.88055900
H	-5.77467900	-3.38270500	-2.95727100
H	-5.51227800	-2.04333300	1.11597600
H	-6.62912600	-3.41846200	-0.62776700

N	-1.27217300	-0.79162900	-2.57713800
S	-0.75256300	-2.31965400	-2.75413500
O	-1.73918900	-3.27365100	-3.28143600
O	0.50955700	-2.22110000	-3.49197400
C	-0.34314600	-2.88025300	-1.10758900
C	-1.21739000	-3.71234600	-0.41519500
C	0.88414400	-2.50779500	-0.55867000
C	-0.84680400	-4.18569800	0.84231100
H	-2.16084700	-3.99801800	-0.87075800
C	1.23741300	-2.98810600	0.69757200
H	1.54740600	-1.86978200	-1.13359900
C	0.37975900	-3.83383600	1.41249300
H	-1.51775600	-4.84593800	1.38486200
H	2.20861600	-2.73828000	1.12240500
C	0.77474900	-4.32746100	2.78000100
H	0.23033700	-5.23443400	3.05038400
H	1.84659600	-4.53754200	2.82547900
H	0.55793800	-3.57034600	3.54304500
H	-1.57643900	0.68116700	-0.17541800
H	-3.31398100	1.31525700	-1.04541700

TS7(R)^{DT}

0 1

C	3.10724900	0.96487600	0.04800700
C	4.73083600	2.37744400	0.38429200
C	2.49501200	3.41379900	-0.19000300
H	2.24486100	3.33665300	-1.25030100
N	5.27252800	1.20062500	0.53488300
N	4.24572600	0.33080700	0.32428800
N	3.39813700	2.27484400	0.09477100
C	5.38235300	3.72695800	0.42612300
H	6.38401400	3.66175200	0.00028900
H	5.45564200	4.07960500	1.46408700
C	3.28702900	4.69402500	0.10606000
O	4.62058100	4.61421000	-0.36126400
C	4.45848400	-1.09442000	0.36531300
C	4.68343000	-1.67608200	1.61734100
C	4.36450000	-1.82741600	-0.82012100

C	4.81274800	-3.06137000	1.65944100
C	4.49110800	-3.21296700	-0.71723100
C	4.71208700	-3.84380700	0.50575600
H	4.98085700	-3.54247300	2.61970000
H	4.39298800	-3.81342100	-1.61818700
H	2.80886000	5.53103300	-0.40399900
C	1.82246000	0.37798800	-0.50776000
O	1.38341700	0.99367200	-1.48875400
C	1.29524200	-0.75324900	0.15351900
C	0.20621400	-1.58569900	-0.52532600
H	1.96258200	-1.30358600	0.81456900
H	-0.14542100	-1.03947800	-1.40510600
C	0.70390400	-2.95821900	-0.91168300
C	0.99729100	-3.24892000	-2.24718500
C	0.97986500	-3.92940000	0.05729100
C	1.54689100	-4.47767600	-2.60864000
H	0.79281500	-2.50098900	-3.00929000
C	1.52236000	-5.15980000	-0.29987300
H	0.77998400	-3.72042800	1.10542800
C	1.80834600	-5.43931200	-1.63560000
H	1.76497300	-4.68475800	-3.65198500
H	1.72536000	-5.90260900	0.46610900
H	2.23050400	-6.40000700	-1.91503400
C	4.86470600	-5.33961600	0.58408300
H	4.36143600	-5.73919900	1.46843200
H	4.44504100	-5.82175800	-0.30116800
H	5.92212200	-5.61479000	0.65390800
C	4.75008800	-0.83880900	2.86715100
H	5.62636900	-0.18490200	2.85783000
H	3.86593000	-0.19917200	2.96058600
H	4.80310000	-1.47778000	3.74939300
C	4.10543800	-1.18060200	-2.15486100
H	4.58734900	-0.20250800	-2.23666800
H	4.48382400	-1.81757800	-2.95631200
H	3.03181600	-1.04269800	-2.32159700
H	3.28209900	4.88994700	1.18920300
C	1.21661700	3.33274200	0.65789500
H	0.85485200	2.30224400	0.68551200

H	1.45666000	3.62329900	1.68711800
C	0.13095900	4.21219300	0.08438100
C	0.03188700	5.56112000	0.43508300
C	-0.78083200	3.67359200	-0.82746000
C	-0.95853900	6.36422900	-0.12412500
H	0.72474500	5.98136800	1.16098000
C	-1.78027500	4.47254000	-1.37688000
H	-0.71856400	2.61857200	-1.09269100
C	-1.86585600	5.81959300	-1.03139900
H	-1.02819600	7.41048100	0.15674400
H	-2.49843300	4.02232500	-2.05471800
H	-2.64551800	6.44278600	-1.45815300
C	-2.00615900	-0.68478000	0.43630700
C	-0.85775200	-1.49011100	0.56956400
C	-3.02985900	-0.83109200	1.52355700
C	-3.74143900	-2.03128400	1.61361900
C	-3.28080600	0.19065000	2.44234200
C	-4.72067900	-2.19535900	2.58792500
H	-3.54251100	-2.82161700	0.89515900
C	-4.24413600	0.01381300	3.43234200
H	-2.73327900	1.12329300	2.36691300
C	-4.97191400	-1.17240600	3.50033500
H	-5.28639600	-3.12069600	2.63488900
H	-4.43006100	0.80747000	4.14905100
H	-5.73162600	-1.30061800	4.26495300
N	-2.09569100	0.16530800	-0.58655700
S	-3.39850700	1.11820800	-0.83193000
O	-3.59117500	2.13203400	0.21382900
O	-3.26697400	1.61075500	-2.20799300
C	-4.82276500	0.04243700	-0.78263900
C	-5.82411200	0.26793700	0.15364400
C	-4.88574500	-1.03767600	-1.65915100
C	-6.89612500	-0.61569500	0.22234300
H	-5.73875500	1.10984100	0.83210300
C	-5.96237000	-1.91249800	-1.57654500
H	-4.09640700	-1.19540100	-2.38802900
C	-6.97564100	-1.71961800	-0.63055700
H	-7.67777400	-0.45290000	0.95951200

H	-6.01821600	-2.76024300	-2.25370800
C	-8.11355400	-2.69952400	-0.51059300
H	-9.03658200	-2.19809100	-0.21021700
H	-8.29207600	-3.21872900	-1.45472300
H	-7.88868600	-3.45712300	0.24782000
H	0.16995800	-0.56085800	0.98749300
H	-0.89709000	-2.27522400	1.32024600

M7(S)

0 1

C	1.28574300	-1.89931800	-0.10793400
C	0.66764900	-3.36928000	1.36287100
C	-1.15021200	-2.70737600	-0.21432700
H	-1.48273600	-1.69195900	0.00908000
N	1.88985400	-3.06486900	1.69004000
N	2.26151900	-2.15247700	0.76081000
N	0.26936600	-2.72156500	0.22316200
C	-0.27179000	-4.23436300	2.14345600
H	-0.00457000	-4.17916900	3.19867200
H	-0.19959200	-5.27812500	1.80598800
C	-1.92859300	-3.73676100	0.61788500
O	-1.57730500	-3.73816400	1.98266300
C	3.61529800	-1.66083800	0.74319800
C	4.47403500	-2.15799900	-0.24108000
C	3.99521000	-0.71954200	1.70353500
C	5.76977400	-1.64600700	-0.26890100
C	5.30441900	-0.24650400	1.63078100
C	6.19665400	-0.68528900	0.65039800
H	6.46244100	-2.01176100	-1.02216100
H	5.63263400	0.48987300	2.35961700
H	-2.98817400	-3.48616100	0.55208000
C	1.42366600	-0.89569300	-1.24940900
O	1.05218800	-1.21546300	-2.35511500
C	2.19394600	0.37023800	-0.96932800
C	1.80234100	1.50869000	-1.91987100
H	2.07282300	0.66958300	0.07427100
H	1.99896600	1.16232700	-2.94288100
C	2.72663800	2.68696500	-1.64309900

C	3.70484200	3.06902600	-2.56016700
C	2.61329200	3.38531500	-0.43542300
C	4.56042700	4.13492800	-2.28176600
H	3.79702100	2.52993700	-3.49975200
C	3.46935300	4.44560800	-0.15527900
H	1.84568800	3.08609200	0.27749900
C	4.44454700	4.82485700	-1.07860500
H	5.31503100	4.42521500	-3.00622900
H	3.37514900	4.98115100	0.78441500
H	5.10864800	5.65513900	-0.86020600
C	7.59021000	-0.11922600	0.57786100
H	8.27353600	-0.81182100	0.08254000
H	7.59136800	0.81576600	0.00893200
H	7.97815400	0.10130500	1.57464600
C	4.01914300	-3.19414200	-1.23712400
H	3.47435500	-4.00581100	-0.74605500
H	3.35841200	-2.76295200	-1.99727100
H	4.87900100	-3.62263900	-1.75319700
C	3.03388700	-0.20603100	2.74070200
H	2.42292600	-1.00854400	3.15829400
H	3.58423300	0.27942000	3.54844300
H	2.33763600	0.52524400	2.31378500
H	-1.78027900	-4.74183800	0.19176900
C	-1.34834700	-3.01556000	-1.70193300
H	-0.69740500	-2.37746400	-2.29809200
H	-1.07483300	-4.05714400	-1.90218500
C	-2.79359400	-2.75868400	-2.06383900
C	-3.65099700	-3.77838900	-2.47490500
C	-3.29297900	-1.45669300	-1.94930900
C	-4.98615500	-3.50121000	-2.77022500
H	-3.27356900	-4.79375500	-2.56500700
C	-4.62550300	-1.17886500	-2.23408300
H	-2.62245300	-0.66088800	-1.62800600
C	-5.47698800	-2.20412400	-2.64733000
H	-5.64337900	-4.30264900	-3.09226000
H	-4.99305600	-0.16153200	-2.13157000
H	-6.51725600	-1.99225600	-2.87196600
C	-0.62879100	1.54874800	-1.04964900

C	0.36811200	1.96620000	-1.84368400
C	-1.93836900	2.28704900	-1.10866000
C	-2.20739900	3.29773400	-0.17945300
C	-2.90783700	1.97937200	-2.06662100
C	-3.43863500	3.94628900	-0.17148200
H	-1.44636400	3.55314100	0.55304900
C	-4.13746300	2.63786000	-2.06999200
H	-2.69413600	1.22567800	-2.81957300
C	-4.41095200	3.61249500	-1.11332600
H	-3.63814700	4.71492200	0.56903700
H	-4.87832100	2.39233600	-2.82549000
H	-5.37104600	4.11904300	-1.10966500
N	-0.55378300	0.40850600	-0.20805300
S	-0.51871800	0.64615700	1.36019000
O	-0.19456800	-0.64893600	1.99867500
O	0.33894400	1.78496400	1.76648600
C	-2.16466800	1.07766600	1.93061200
C	-3.27721000	0.49588700	1.32706200
C	-2.31852800	2.02051500	2.93925800
C	-4.54975500	0.88174200	1.72498100
H	-3.14875700	-0.21997700	0.52038700
C	-3.60069100	2.39659900	3.33247900
H	-1.43951300	2.47490300	3.38393600
C	-4.73039600	1.84574400	2.72414900
H	-5.41884700	0.44302800	1.24135900
H	-3.72530800	3.14162600	4.11354300
C	-6.11623900	2.30047700	3.10090000
H	-6.80406100	1.45424400	3.17415500
H	-6.11531700	2.82950600	4.05608700
H	-6.51438800	2.98081500	2.34065300
H	0.14044800	2.79304000	-2.51398900
H	3.25718200	0.14959100	-1.13304000

M7(R)

0 1

C	0.66340400	-2.00430000	-0.05817000
C	-0.17947000	-3.55258600	-1.32120100
C	-1.68368900	-2.84750900	0.56147100

H	-1.39044100	-3.14174900	1.57121900
N	0.99804400	-3.30835800	-1.82625000
N	1.50925000	-2.34024600	-1.02987900
N	-0.42242800	-2.78343500	-0.22194300
C	-1.15432300	-4.60604100	-1.74608700
H	-0.61002100	-5.46645400	-2.13653800
H	-1.82057300	-4.21358800	-2.52629200
C	-2.59418100	-3.91129000	-0.07877700
O	-1.88082500	-5.00993300	-0.60763500
C	2.89620400	-1.97078600	-1.17992900
C	3.24981300	-1.00186000	-2.11676700
C	3.81818600	-2.62968400	-0.35809200
C	4.60415700	-0.66888000	-2.19277600
C	5.15250200	-2.25441100	-0.47171500
C	5.56024400	-1.26726900	-1.37430400
H	4.91261100	0.08782700	-2.91004000
H	5.89199800	-2.74382000	0.15670300
H	-3.27227300	-4.29264200	0.68588700
C	0.90331000	-1.08014700	1.11404700
O	0.16712500	-1.22663400	2.06629200
C	2.10382500	-0.17312700	1.13269800
C	1.85438900	1.11136400	1.94464800
H	2.90906500	-0.74989300	1.61037000
H	1.27095500	0.83151500	2.83263100
C	3.20447400	1.61862500	2.43066600
C	3.59999000	1.43757700	3.75624000
C	4.09658000	2.22207800	1.53876200
C	4.86040600	1.84940300	4.18648900
H	2.91296100	0.97141400	4.45822200
C	5.35813300	2.62896400	1.96278000
H	3.78775300	2.37794300	0.50749600
C	5.74386600	2.44432100	3.29003300
H	5.14990900	1.70676200	5.22309500
H	6.04043600	3.09472600	1.25819400
H	6.72518700	2.76669200	3.62315500
C	7.00366800	-0.84323200	-1.44008200
H	7.25206200	-0.43459600	-2.42169900
H	7.20282800	-0.06614000	-0.69476900

H	7.67208700	-1.68124800	-1.23049900
C	2.23081900	-0.28979000	-2.96055900
H	1.30987400	-0.86351000	-3.07743600
H	1.94742700	0.65707400	-2.48883200
H	2.64462400	-0.06706500	-3.94644200
C	3.37885200	-3.69018200	0.61813000
H	2.75699200	-4.44693400	0.13040100
H	4.24610700	-4.18760700	1.05351400
H	2.79477500	-3.26515800	1.44298000
H	-3.19119100	-3.44301000	-0.87497200
C	-2.40944900	-1.49981200	0.57754800
H	-1.69019800	-0.70685400	0.78565700
H	-2.80670600	-1.30793700	-0.42181200
C	-3.51361700	-1.48040400	1.60553100
C	-4.85527800	-1.43124500	1.22117900
C	-3.19954500	-1.49218600	2.96829700
C	-5.86641400	-1.39897100	2.17978700
H	-5.10523100	-1.39708500	0.16303900
C	-4.20750500	-1.46000800	3.92740300
H	-2.15451300	-1.50975300	3.27011100
C	-5.54441100	-1.41475000	3.53499500
H	-6.90540100	-1.36021700	1.86698300
H	-3.95042200	-1.46373100	4.98202900
H	-6.33106100	-1.38702900	4.28229300
C	0.37706800	2.08927600	0.04979000
C	1.13998300	2.18912600	1.16283300
C	-0.10077300	3.40217700	-0.51411500
C	-0.96087400	4.20847300	0.23797200
C	0.37243400	3.88670100	-1.73873300
C	-1.35665500	5.46081900	-0.22568200
H	-1.32017500	3.84520000	1.19662600
C	-0.01734400	5.13973600	-2.20084700
H	1.04040200	3.27125200	-2.33060100
C	-0.88682400	5.92897200	-1.44941300
H	-2.02942800	6.06916300	0.37078400
H	0.36152700	5.50340700	-3.15100400
H	-1.19329000	6.90398500	-1.81519500
N	0.05841100	0.84948200	-0.51824300

S	-0.88495300	0.67812000	-1.78376000
O	-0.53799400	1.46166700	-2.98419100
O	-0.98996400	-0.77781200	-2.04099000
C	-2.54512300	1.20397300	-1.33389600
C	-3.52583300	1.18028600	-2.32118600
C	-2.87859100	1.52026800	-0.01957000
C	-4.84855200	1.45208100	-1.98233200
H	-3.25530300	0.94485800	-3.34618000
C	-4.20145400	1.79479200	0.30586400
H	-2.11061600	1.52909300	0.74873700
C	-5.20637200	1.75339800	-0.66550100
H	-5.61596700	1.42334600	-2.75111500
H	-4.46308400	2.02305700	1.33608600
C	-6.64030200	2.00543200	-0.27981600
H	-7.30020100	1.96637900	-1.14876000
H	-6.97879700	1.25479400	0.44216100
H	-6.75428000	2.98510500	0.19306900
H	1.30734000	3.19506600	1.53703400
H	2.42748600	0.08080000	0.12458800

TS8(S)

0 1

C	0.54866300	-1.97001900	0.32855900
C	-0.10283300	-3.44282100	1.79562800
C	-1.94292100	-2.48731800	0.41268300
H	-2.09439200	-1.44963900	0.11927200
N	1.19484000	-3.44432700	1.88325700
N	1.58849200	-2.53160400	0.94347700
N	-0.53674900	-2.57921500	0.83129700
C	-1.14837900	-4.10817700	2.63197700
H	-0.73714300	-4.37472000	3.60532000
H	-1.53107800	-5.01390200	2.13986100
C	-2.85973500	-2.84883000	1.62150800
O	-2.16559500	-3.14875000	2.81875200
C	2.98498200	-2.40206200	0.61790700
C	3.42137100	-2.97999800	-0.57929800
C	3.83336600	-1.73545900	1.50407500
C	4.76463100	-2.81793100	-0.91245700

C	5.16879800	-1.60652400	1.12355600
C	5.64620100	-2.12247300	-0.08219700
H	5.13028700	-3.24864000	-1.84095200
H	5.85211100	-1.08482200	1.78851900
H	-3.49329600	-1.99424800	1.85440700
C	0.51782000	-0.94262700	-0.81093500
O	-0.04053800	-1.37950400	-1.84223600
C	1.73085000	-0.02856800	-0.93733600
C	1.44171900	1.02373100	-2.02120000
H	1.96863500	0.42470400	0.03089700
H	1.42710900	0.47969900	-2.97574000
C	2.59042100	2.01881700	-2.07783300
C	3.79329600	1.64970400	-2.68886700
C	2.50153300	3.28067600	-1.48576000
C	4.88222400	2.51572300	-2.70301400
H	3.87289100	0.67152400	-3.15837800
C	3.59011500	4.15180100	-1.49924700
H	1.56942600	3.58161800	-1.01432200
C	4.78411200	3.77204300	-2.10582200
H	5.80693400	2.21286100	-3.18437800
H	3.50210000	5.12998200	-1.03600500
H	5.63137200	4.45004900	-2.11801500
C	7.08176400	-1.91800600	-0.48944700
H	7.43787700	-2.74220900	-1.11118600
H	7.18383600	-0.99565100	-1.07051300
H	7.73236600	-1.83279100	0.38342000
C	2.47591900	-3.72992900	-1.48219600
H	1.89560100	-4.46712700	-0.91769800
H	1.76393400	-3.05483800	-1.97010400
H	3.03400900	-4.25618800	-2.25768100
C	3.33100900	-1.18850600	2.81137300
H	2.99171200	-2.00064500	3.46135800
H	4.12576500	-0.64465900	3.32405100
H	2.47875200	-0.52262900	2.65741600
H	-3.48790100	-3.70545600	1.33776900
C	-2.19226100	-3.39518000	-0.80346400
H	-1.36479200	-3.24827200	-1.50155000
H	-2.21906700	-4.44495800	-0.49001100

C	-3.48993400	-2.98910300	-1.45988600
C	-4.64498100	-3.76578300	-1.37039000
C	-3.54201900	-1.76515700	-2.13858600
C	-5.83721300	-3.32874600	-1.94770600
H	-4.61107200	-4.71996100	-0.85030000
C	-4.73217000	-1.32457300	-2.70767100
H	-2.63356000	-1.16775500	-2.21311400
C	-5.88381200	-2.10707000	-2.61313900
H	-6.72910200	-3.94280300	-1.87381100
H	-4.76154200	-0.37030300	-3.22455200
H	-6.81249600	-1.76500600	-3.05838400
C	-0.82791300	1.38957200	-0.94594400
C	0.10061500	1.68284800	-1.87210400
C	-2.11839100	2.12949500	-0.89222300
C	-2.15053800	3.50772600	-1.13627700
C	-3.30876800	1.47404200	-0.55959700
C	-3.34821700	4.21070200	-1.07022500
H	-1.22047500	4.03198400	-1.33807800
C	-4.50878000	2.17719400	-0.49977300
H	-3.28882500	0.41148100	-0.34018800
C	-4.53296400	3.54721100	-0.75280100
H	-3.35521900	5.28151600	-1.24884500
H	-5.42596400	1.65222300	-0.25119400
H	-5.46711700	4.09658100	-0.69414200
N	-0.63096400	0.32138700	-0.04007500
S	-0.77095100	0.58486300	1.56817400
O	-2.06483800	0.10683400	2.07536800
O	0.41744800	-0.00887000	2.20847800
C	-0.71407800	2.34380300	1.82070700
C	-1.85597600	3.01574100	2.24185400
C	0.46432000	3.02395600	1.52915000
C	-1.81096800	4.39914900	2.36876600
H	-2.76291100	2.45597800	2.44351600
C	0.48802000	4.40871500	1.64887000
H	1.34778400	2.47958800	1.20670700
C	-0.64811400	5.11371100	2.06278300
H	-2.69653400	4.93612100	2.69651900
H	1.40180800	4.95040000	1.41939100

C	-0.63244000	6.61696400	2.15157400
H	0.38690900	7.00020700	2.22871000
H	-1.08872400	7.05580100	1.25819100
H	-1.20223000	6.96628200	3.01573600
H	-0.15526300	2.42010500	-2.62887500
H	2.60148400	-0.61145300	-1.25587800

TS8(R)

0 1

C	-0.32766700	-1.87929500	-0.44277100
C	-1.26007000	-3.41609500	-1.67618200
C	-2.84737700	-2.30233100	-0.08434400
H	-2.66437200	-2.35001300	0.98981200
N	-0.00358200	-3.43393900	-2.01191700
N	0.56731900	-2.47446400	-1.23523700
N	-1.51026400	-2.47623100	-0.71506900
C	-2.32561500	-4.38563900	-2.07516800
H	-1.86229000	-5.31751000	-2.39936300
H	-2.94591100	-3.98475700	-2.88827500
C	-3.77592600	-3.46169600	-0.53419000
O	-3.09727800	-4.63706900	-0.92070100
C	1.99913800	-2.30589900	-1.33035400
C	2.51062700	-1.50383900	-2.35414500
C	2.81423400	-3.02507800	-0.44937500
C	3.89580500	-1.35178000	-2.41151100
C	4.19178500	-2.83650100	-0.55229300
C	4.74857300	-1.99011100	-1.51099800
H	4.31576000	-0.72271000	-3.19350400
H	4.84309100	-3.37238600	0.13419500
H	-4.42900400	-3.72213000	0.29932600
C	-0.13361600	-0.89140100	0.71047300
O	-1.16988100	-0.52988900	1.26550200
C	1.14785000	-0.99575600	1.50508800
C	1.38260300	0.25361800	2.36373800
H	1.02366900	-1.87234300	2.15494800
H	0.44663000	0.47540600	2.89487400
C	2.44747600	-0.06874900	3.39577800
C	2.11013000	-0.26174100	4.73576600

C	3.78122700	-0.23413500	3.00763700
C	3.08170600	-0.60978100	5.67249100
H	1.07621600	-0.13706500	5.04765300
C	4.75365400	-0.58473300	3.93927200
H	4.05346300	-0.07928900	1.96544600
C	4.40626500	-0.77280000	5.27664000
H	2.80301900	-0.75154700	6.71213400
H	5.78481100	-0.70831600	3.62279500
H	5.16478200	-1.04251600	6.00467500
C	6.23660100	-1.76630400	-1.56995900
H	6.56997200	-1.59635900	-2.59614700
H	6.51244900	-0.88454200	-0.98265900
H	6.78149800	-2.62072700	-1.16278000
C	1.64259100	-0.86991500	-3.40407600
H	0.58068200	-0.92955400	-3.16504600
H	1.88579200	0.18812300	-3.51786400
H	1.81133000	-1.36919400	-4.36368400
C	2.27588600	-3.97519700	0.58980600
H	2.66300100	-4.98100500	0.40614700
H	2.60496000	-3.67223600	1.58803800
H	1.18615000	-4.03427500	0.59225900
H	-4.40305900	-3.11573900	-1.36868400
C	-3.51926900	-0.96638600	-0.43131000
H	-2.82388200	-0.15359800	-0.23865700
H	-3.75830200	-0.95307100	-1.49931800
C	-4.76400500	-0.79797000	0.40848500
C	-6.04426400	-0.93544100	-0.13041600
C	-4.63148800	-0.50675700	1.77121200
C	-7.17279000	-0.78709900	0.67443500
H	-6.15918900	-1.14964500	-1.19019900
C	-5.75605100	-0.36052600	2.57668400
H	-3.63357000	-0.38652200	2.18809000
C	-7.03126100	-0.50183900	2.02955500
H	-8.16206600	-0.89309800	0.24044500
H	-5.63955400	-0.13090100	3.63128900
H	-7.90944400	-0.38609300	2.65675400
C	1.46747200	1.58281400	0.19516200
C	1.78702200	1.41493300	1.49284600

C	2.07020200	2.75107000	-0.52129400
C	1.78650000	4.05520400	-0.11596500
C	2.95446500	2.54032700	-1.58396600
C	2.38436100	5.13874900	-0.75920300
H	1.07971500	4.21248900	0.69441200
C	3.55813000	3.61832100	-2.21862400
H	3.15942500	1.52040300	-1.90071600
C	3.27197600	4.92195800	-1.80848200
H	2.15236800	6.15059400	-0.44155200
H	4.25185000	3.44653800	-3.03587700
H	3.73971800	5.76388200	-2.30924200
N	0.66028500	0.67091000	-0.51532800
S	-0.34477200	1.26102000	-1.63377100
O	0.29906100	1.89209900	-2.79470400
O	-1.26131500	0.15059700	-1.96276500
C	-1.28844100	2.57099500	-0.85785300
C	-1.55787200	3.72629900	-1.58374900
C	-1.73302500	2.42781100	0.45455500
C	-2.30180600	4.74236500	-0.99180600
H	-1.16822800	3.82367200	-2.59096700
C	-2.47750300	3.45211100	1.02972400
H	-1.48715200	1.53132900	1.01797500
C	-2.77615100	4.61852700	0.31697300
H	-2.51293100	5.64891300	-1.55223500
H	-2.82741600	3.34938600	2.05355000
C	-3.60535300	5.70785100	0.94543300
H	-3.43221000	6.66961300	0.45791900
H	-4.67217800	5.47735000	0.85795100
H	-3.37908200	5.81241400	2.00934600
H	2.45947500	2.14552000	1.93335300
H	2.01497700	-1.15359200	0.86558600

M8(S)

0 1

C	-0.95239200	-1.69435400	-0.05609400
C	-0.25595200	-3.35256100	-1.28060300
C	1.46493700	-2.39567600	0.26989400
H	1.68677400	-1.32949900	0.34237900

N	-1.52193800	-3.24350000	-1.56223000
N	-1.94733400	-2.21685100	-0.76747000
N	0.11933700	-2.46230900	-0.31762200
C	0.81362400	-4.15726400	-1.94890500
H	0.52304100	-4.37748000	-2.97577800
H	1.00467400	-5.09734400	-1.41327700
C	2.46897900	-3.12358300	-0.66855500
O	1.97375500	-3.34667000	-1.97405500
C	-3.35402300	-1.92995700	-0.66309700
C	-4.00309200	-2.37005000	0.49646200
C	-4.00373500	-1.25867700	-1.69992300
C	-5.36365400	-2.09791600	0.60981600
C	-5.36888000	-1.01876100	-1.53747000
C	-6.05960100	-1.41995600	-0.39430700
H	-5.89344700	-2.42512000	1.50080400
H	-5.90315900	-0.49884100	-2.32861700
H	3.37119300	-2.51744300	-0.77237300
C	-0.93483600	-0.48186400	0.88572300
O	-0.48034000	-0.76149200	2.02802300
C	-2.13091700	0.46915400	0.80423500
C	-1.84110800	1.67307900	1.71530600
H	-2.32644900	0.78641300	-0.22631800
H	-1.92888900	1.30955800	2.74752100
C	-2.88117300	2.75983700	1.51446100
C	-3.87894800	2.97561900	2.46514600
C	-2.86519000	3.54420300	0.35584100
C	-4.84832000	3.95806100	2.26839400
H	-3.89595900	2.36850200	3.36682300
C	-3.83119800	4.52683600	0.15889600
H	-2.09026900	3.36829300	-0.38836800
C	-4.82532600	4.73706100	1.11488800
H	-5.61829200	4.11505400	3.01744600
H	-3.81010400	5.13159500	-0.74261700
H	-5.57705100	5.50461600	0.96016600
C	-7.52449500	-1.10821900	-0.23399600
H	-8.02456800	-1.86242700	0.37755300
H	-7.65666800	-0.13982100	0.25908700
H	-8.02532400	-1.05663100	-1.20318300

C	-3.24876100	-3.08237400	1.59039300
H	-2.67914500	-3.92807500	1.19102000
H	-2.53673400	-2.41165800	2.08672700
H	-3.94078000	-3.46079600	2.34375400
C	-3.27303000	-0.77624300	-2.92279900
H	-2.53086600	-1.50098100	-3.26158500
H	-3.98147100	-0.58177700	-3.73012500
H	-2.72818700	0.14863400	-2.70718000
H	2.74947900	-4.08706800	-0.21891400
C	1.48468200	-3.02221000	1.67434400
H	0.61765000	-2.64411400	2.21807500
H	1.41580900	-4.11286900	1.58936900
C	2.75172500	-2.60477500	2.38023300
C	3.83169100	-3.47086700	2.55224400
C	2.85731800	-1.28386500	2.83181400
C	5.00241900	-3.02626500	3.16613100
H	3.75486700	-4.50023000	2.21036000
C	4.02791500	-0.83685000	3.43564600
H	2.00495400	-0.61781200	2.69795100
C	5.10383000	-1.70886500	3.60485200
H	5.83456700	-3.71044900	3.29895700
H	4.10111600	0.19236200	3.77359300
H	6.01662000	-1.36193400	4.07870200
C	0.52634400	1.70171800	0.80619900
C	-0.44297000	2.20895100	1.58029600
C	1.89823100	2.28176000	0.80685400
C	2.08066000	3.66697800	0.87917400
C	3.02793500	1.46066800	0.71845900
C	3.35988800	4.21679900	0.87720500
H	1.20922700	4.31517400	0.90314000
C	4.30552800	2.00756200	0.71873400
H	2.89827600	0.38698700	0.63313500
C	4.47758900	3.38928200	0.79686500
H	3.48232400	5.29449300	0.92377100
H	5.16913400	1.35199600	0.65270200
H	5.47535300	3.81703200	0.78692500
N	0.29972300	0.52843300	0.01593800
S	0.15687300	0.84447100	-1.60165800

O	-0.26352100	-0.40923400	-2.24067700
O	-0.65176600	2.04251100	-1.86469700
C	1.80741300	1.17173400	-2.16820100
C	2.64702600	0.08404400	-2.38412800
C	2.26145500	2.48086200	-2.29102600
C	3.98342600	0.32007800	-2.68922200
H	2.25015900	-0.92710300	-2.32628400
C	3.59837700	2.69683800	-2.59726100
H	1.57880700	3.30698100	-2.12312000
C	4.47904500	1.62439800	-2.77913600
H	4.65259500	-0.51957000	-2.85673500
H	3.97301600	3.71352400	-2.67811000
C	5.93817900	1.88539900	-3.04181400
H	6.07130000	2.61212900	-3.84779100
H	6.41218900	2.30060700	-2.14599800
H	6.46669400	0.96909900	-3.31153100
H	-0.18818700	3.03248000	2.24340900
H	-3.03146800	-0.02796400	1.18020300

M8(R)

0 1

C	-0.77416300	-1.56233000	-0.74825900
C	-1.91602300	-3.13573100	-1.75993200
C	-3.31895100	-1.57306100	-0.38546300
H	-3.14899200	-1.48053800	0.68652900
N	-0.68195800	-3.36215900	-2.09300400
N	0.01893400	-2.37692900	-1.45780500
N	-2.02524100	-2.03488400	-0.95027400
C	-3.11034500	-3.99859800	-2.01942500
H	-2.78522900	-5.02898900	-2.16354000
H	-3.65784400	-3.66346400	-2.91092900
C	-4.41404000	-2.62112200	-0.70114600
O	-3.92588800	-3.93616600	-0.87009700
C	1.44764800	-2.33137700	-1.65266600
C	1.92768800	-1.64870700	-2.77752700
C	2.28209100	-3.04779500	-0.78991000
C	3.30916100	-1.56329500	-2.93007400
C	3.65968600	-2.91891000	-0.98139700

C	4.18922100	-2.15720300	-2.02132100
H	3.70720200	-1.01749100	-3.78195500
H	4.33049200	-3.44627300	-0.30712200
H	-5.12329900	-2.64104400	0.12707200
C	-0.42019400	-0.50715600	0.36824900
O	-1.45583500	0.13986700	0.79672300
C	0.27703900	-1.33390100	1.47445300
C	0.66950000	-0.39239300	2.61175500
H	-0.43444900	-2.09310300	1.81772000
H	-0.24703300	0.08587600	2.97902200
C	1.31604500	-1.17516600	3.73544100
C	0.62607600	-1.42906500	4.92146300
C	2.60192500	-1.70556800	3.57957300
C	1.20494700	-2.19373600	5.93303900
H	-0.37351500	-1.02266400	5.05226300
C	3.18308500	-2.47035100	4.58671400
H	3.14868500	-1.50930800	2.65891600
C	2.48499500	-2.71638800	5.76852700
H	0.65556800	-2.37872800	6.85083000
H	4.18223700	-2.87266700	4.45143500
H	2.93755500	-3.31038700	6.55595400
C	5.67675000	-1.96844400	-2.15989900
H	5.96429800	-1.83565500	-3.20514700
H	5.99504900	-1.07467300	-1.61191400
H	6.22602200	-2.81929600	-1.75108900
C	1.00380400	-1.13332800	-3.84838000
H	0.06428400	-0.75366500	-3.44632800
H	1.48169000	-0.32582000	-4.40333700
H	0.77227500	-1.94845100	-4.54297300
C	1.76633500	-4.01034800	0.25218500
H	2.05238100	-5.02740800	-0.03090800
H	2.20266200	-3.80478800	1.23405000
H	0.68024300	-3.98429100	0.34946600
H	-4.95468300	-2.31787300	-1.61018100
C	-3.73910700	-0.20891600	-0.94588300
H	-2.88993100	0.46566600	-0.85072600
H	-3.99670300	-0.31752100	-2.00584300
C	-4.91364900	0.31728100	-0.15544400

C	-6.20259500	0.37299400	-0.68756300
C	-4.70335500	0.74440500	1.16199100
C	-7.26660800	0.84899900	0.07829400
H	-6.37365000	0.04891600	-1.71122400
C	-5.76462600	1.21731800	1.92706800
H	-3.69329000	0.70037700	1.56510500
C	-7.05004600	1.27084900	1.38700000
H	-8.26343800	0.89037000	-0.34942900
H	-5.59068500	1.54972000	2.94586800
H	-7.87715200	1.64201100	1.98371300
C	1.63629000	0.93488100	0.70912400
C	1.59881600	0.62624800	2.01530400
C	2.73162200	1.82136000	0.21766000
C	2.89587400	3.09828500	0.75686600
C	3.65500800	1.34594800	-0.71888400
C	3.97360500	3.89230700	0.36922600
H	2.15839900	3.47235500	1.46215700
C	4.73095200	2.13702800	-1.10238700
H	3.50759800	0.36005200	-1.15010100
C	4.89348400	3.41230500	-0.55871700
H	4.08826800	4.88727300	0.78735100
H	5.44370600	1.76122200	-1.83025300
H	5.73244500	4.03007800	-0.86303000
N	0.72401900	0.37432200	-0.23959900
S	0.25075200	1.37817200	-1.50798900
O	1.35292000	1.54528000	-2.44857200
O	-0.98289600	0.78788100	-2.03378000
C	-0.08297300	2.97904500	-0.80383500
C	0.49532500	4.09499400	-1.40025400
C	-0.88814900	3.07987300	0.32800300
C	0.24517500	5.34817300	-0.85261600
H	1.14718000	3.97261500	-2.25823400
C	-1.11762000	4.34363700	0.86097300
H	-1.29165200	2.17001900	0.77301500
C	-0.56268900	5.49009300	0.28004500
H	0.69277900	6.22884300	-1.30405600
H	-1.73535300	4.44427400	1.74909200
C	-0.84688600	6.85269700	0.85520500

H	-0.04918600	7.55863000	0.61489400
H	-1.77981000	7.25322100	0.44547600
H	-0.95754400	6.80759400	1.94095000
H	2.36410800	1.06570300	2.64790400
H	1.17883300	-1.82379300	1.10488500

TS9(S)

0 1

C	0.25972200	-0.00940900	0.55136100
O	1.42223100	0.36801600	0.67503400
C	-0.55261000	-0.46745100	1.73770900
C	-1.03867100	0.78774900	2.47693100
H	0.08946100	-1.08773000	2.36728500
H	-0.16555500	1.33956300	2.84951400
C	-1.90945900	0.40252800	3.65542800
C	-1.45867500	0.56885600	4.96496700
C	-3.16831900	-0.16976200	3.44163400
C	-2.24871700	0.17465700	6.04374100
H	-0.48171400	1.01138200	5.14070600
C	-3.95869700	-0.56477300	4.51616700
H	-3.52835000	-0.30389100	2.42335000
C	-3.50046800	-0.39277200	5.82204200
H	-1.88531200	0.31327800	7.05706700
H	-4.93379600	-1.00618200	4.33541600
H	-4.11703900	-0.69894900	6.66091100
C	-1.61142700	1.53163500	0.13210000
C	-1.78848600	1.60945900	1.46234800
C	-2.58162100	2.24615400	-0.74652000
C	-2.86487000	3.59464800	-0.52195400
C	-3.30557500	1.55008800	-1.71838000
C	-3.85421100	4.24058000	-1.26309100
H	-2.30206800	4.13777100	0.23233700
C	-4.29490200	2.19053200	-2.45124100
H	-3.08315100	0.50298100	-1.88307300
C	-4.57089700	3.54101300	-2.22849100
H	-4.06057000	5.29079900	-1.08398100
H	-4.85402200	1.63574400	-3.19877700
H	-5.34072400	4.04253100	-2.80592000

N	-0.60197800	0.68215900	-0.45392400
S	0.30224800	1.30314900	-1.76877100
O	-0.65285400	1.82969300	-2.73152800
O	1.19074900	0.22599200	-2.18187200
C	1.26563600	2.68098400	-1.19353000
C	0.65949300	3.93013400	-1.12732000
C	2.60645700	2.50125800	-0.86061000
C	1.41286100	5.01827100	-0.69302900
H	-0.37641700	4.05159600	-1.42833300
C	3.34326000	3.59980700	-0.44463200
H	3.04927900	1.51432200	-0.92509800
C	2.75707700	4.86953300	-0.34637500
H	0.95005000	5.99871300	-0.63469900
H	4.39224600	3.47448200	-0.19033600
C	3.57571400	6.04458800	0.11865300
H	2.98855400	6.96446200	0.11444400
H	4.44674800	6.18955700	-0.52656000
H	3.94673300	5.87925100	1.13426900
H	-2.61976600	2.21388700	1.81134600
H	-1.42026500	-1.04511900	1.40932500
C	0.39697300	-1.91680300	-0.44985900
C	1.32525300	-3.22905500	-1.98933200
C	2.92277000	-1.89783300	-0.59589900
H	2.96535200	-0.83969000	-0.86942000
N	0.04964400	-3.41993700	-2.13821100
N	-0.49599400	-2.60881500	-1.17034700
N	1.57704900	-2.36131500	-0.95854000
C	2.44497400	-3.78716000	-2.81319700
H	2.13240600	-3.86517900	-3.85499800
H	2.72802900	-4.78595300	-2.45064400
C	3.94311900	-2.71055100	-1.40386300
O	3.54308500	-2.90306600	-2.74628100
C	-1.91777500	-2.57199300	-1.00981000
C	-2.47973300	-3.15172000	0.13277600
C	-2.69111100	-1.99548600	-2.02106300
C	-3.86459900	-3.07043300	0.28316600
C	-4.07284800	-1.95724000	-1.83360200
C	-4.67356800	-2.46840100	-0.68191800

H	-4.32240300	-3.50441900	1.16872700
H	-4.69377700	-1.50388200	-2.60368400
H	4.89431700	-2.17677800	-1.41863100
C	-1.62926100	-3.88264500	1.14012100
H	-1.02929900	-4.65306000	0.64581900
H	-0.93337300	-3.21339400	1.65260800
H	-2.25881000	-4.36332200	1.89054400
C	-6.16315600	-2.35962600	-0.48312300
H	-6.42608200	-1.37304200	-0.08797800
H	-6.69768800	-2.48779700	-1.42729900
H	-6.52423500	-3.10895900	0.22443100
C	-2.05137200	-1.44805800	-3.26936200
H	-1.17344200	-0.84051900	-3.03120100
H	-1.71531300	-2.26299200	-3.91693800
H	-2.75974100	-0.83164100	-3.82718500
H	4.10103600	-3.68855800	-0.92022400
C	3.17914300	-2.06181900	0.90707300
H	2.35487400	-1.58998200	1.44459000
H	3.19041200	-3.13029000	1.15129000
C	4.48561800	-1.41164100	1.29916100
C	5.64149800	-2.16754800	1.50577300
C	4.55644800	-0.01865900	1.42159600
C	6.84838500	-1.54804500	1.82666500
H	5.59496800	-3.25045800	1.41846400
C	5.76119800	0.60051900	1.74242900
H	3.65156000	0.56577500	1.27420300
C	6.91117000	-0.16205200	1.94412100
H	7.73776600	-2.14926800	1.98682700
H	5.80236100	1.68101000	1.84210300
H	7.84964300	0.32152000	2.19551000

TS9(R)

0 1

C	-0.28149600	-1.86615500	-0.68767600
C	-0.82754200	-4.02857400	-0.67790200
C	-2.68927100	-2.45190800	-0.10600200
H	-2.63873700	-2.02605900	0.90010000
N	0.43347400	-4.02033800	-0.98351300

N	0.74340300	-2.68025300	-0.98565400
N	-1.30325900	-2.75142000	-0.51045000
C	-1.71018500	-5.21670600	-0.44403000
H	-1.13313600	-6.01730100	0.01996600
H	-2.12355100	-5.58120800	-1.39509000
C	-3.48310400	-3.76456500	-0.09701000
O	-2.74493900	-4.84478200	0.44071800
C	2.09552100	-2.29198800	-1.26105800
C	2.36996000	-1.64455400	-2.47151100
C	3.09321100	-2.61587100	-0.33604000
C	3.69319100	-1.29418100	-2.72914000
C	4.39826100	-2.21026100	-0.62682100
C	4.71457300	-1.54441500	-1.80950200
H	3.92943200	-0.79103600	-3.66457500
H	5.18567300	-2.43174700	0.08972400
H	-4.37480400	-3.63554700	0.51819700
C	-0.29859800	-0.06061500	0.39060500
O	-1.48231300	0.20760000	0.60097000
C	0.55446800	-0.64398900	1.49999300
C	0.96675800	0.50805200	2.42867900
H	-0.04337500	-1.39575600	2.02237600
H	0.06368400	0.96198200	2.85679400
C	1.83420400	-0.02191900	3.55168000
C	1.35548100	-0.08783500	4.86000000
C	3.11928200	-0.50358900	3.27708500
C	2.14360800	-0.62165600	5.87856600
H	0.35816500	0.28240300	5.08229200
C	3.90758000	-1.03830900	4.29122500
H	3.50157000	-0.45709600	2.25832900
C	3.42088400	-1.09832100	5.59687200
H	1.75834300	-0.66324900	6.89241400
H	4.90303000	-1.40701300	4.06369400
H	4.03539300	-1.51326900	6.38916300
C	6.11695300	-1.07007700	-2.08610600
H	6.84269600	-1.57424500	-1.44489900
H	6.39374100	-1.24402400	-3.12898600
H	6.19375900	0.00666200	-1.90066000
C	1.27286600	-1.33472000	-3.45342800

H	0.55831200	-0.62076300	-3.03187300
H	1.68980100	-0.91356600	-4.36978100
H	0.71120500	-2.23871100	-3.70986400
C	2.80721100	-3.41279200	0.91157100
H	2.88998900	-4.48356100	0.70137300
H	3.52414700	-3.16252200	1.69686300
H	1.80005000	-3.23814000	1.29576300
H	-3.80110700	-4.00826600	-1.12326500
C	-3.33716800	-1.44175900	-1.05585200
H	-2.65518300	-0.59718900	-1.16776500
H	-3.46076200	-1.90920900	-2.03975900
C	-4.67207500	-0.97472200	-0.52290300
C	-5.87166300	-1.35119400	-1.13039600
C	-4.71864300	-0.15519600	0.61291700
C	-7.09496800	-0.91539300	-0.62294100
H	-5.84670600	-1.98587900	-2.01271400
C	-5.93960800	0.28134800	1.11968200
H	-3.78390900	0.14370500	1.08196100
C	-7.13190200	-0.09714000	0.50277600
H	-8.01801700	-1.21411700	-1.10970900
H	-5.96088200	0.91995500	1.99769700
H	-8.08325900	0.24481600	0.89745100
C	1.51896300	1.61261500	0.23226300
C	1.70472400	1.48934000	1.55779300
C	2.49353200	2.42809400	-0.54627600
C	2.88318700	3.68831600	-0.08891800
C	3.11736400	1.88974600	-1.67538600
C	3.89001200	4.39621200	-0.74515000
H	2.39203200	4.11559500	0.78116300
C	4.12553300	2.59010400	-2.32294500
H	2.79753900	0.91930000	-2.03586400
C	4.51666900	3.84808300	-1.85952100
H	4.17972200	5.37754600	-0.38359200
H	4.60632300	2.15585500	-3.19469700
H	5.30079200	4.39800700	-2.36974900
N	0.50495800	0.86248800	-0.46721100
S	-0.45543900	1.71845200	-1.59185900
O	0.44492100	2.50742500	-2.41860300

O	-1.30541400	0.72744500	-2.23538600
C	-1.45893600	2.86687700	-0.68362900
C	-0.88140400	4.05451000	-0.24778200
C	-2.79725700	2.57161500	-0.44157700
C	-1.66733600	4.95681700	0.46424000
H	0.15776800	4.27500000	-0.47470400
C	-3.56641900	3.48826700	0.25978600
H	-3.21463100	1.63382900	-0.79055200
C	-3.01323800	4.68845700	0.72600600
H	-1.22924600	5.88640100	0.81489800
H	-4.61229700	3.26621400	0.45527800
C	-3.86857800	5.67587500	1.47509600
H	-3.26013700	6.44423900	1.95551700
H	-4.56603000	6.17356800	0.79419200
H	-4.46378700	5.17418500	2.24234700
H	2.54445800	2.02356400	1.99062400
H	1.46324800	-1.09895200	1.10187200

P(S)

0 1

C	-2.05561400	-1.51691700	-0.69532300
C	-2.79633100	-0.58622600	0.28204000
C	-0.59936700	-1.67707300	-0.31636100
C	-2.03744000	0.71774200	0.29344700
C	-0.70129100	0.71997300	0.28724400
H	-2.10622000	-1.09507400	-1.70680700
H	-2.75286000	-1.03797800	1.28310000
O	0.03258500	-2.69280500	-0.49685000
C	-4.24936700	-0.43791900	-0.11297600
C	-5.24896500	-1.09082900	0.60923900
C	-4.60937600	0.31978700	-1.23186700
C	-6.58447500	-0.98629100	0.22671700
H	-4.97850800	-1.68257100	1.47973000
C	-5.94257500	0.42665900	-1.61594600
H	-3.84023600	0.83240900	-1.80528900
C	-6.93445900	-0.22661900	-0.88647900
H	-7.35144100	-1.49668100	0.80048700
H	-6.20774200	1.02040400	-2.48488400

H	-7.97457100	-0.14271900	-1.18437400
H	-2.48775900	-2.51908800	-0.72705500
N	-0.01760800	-0.54556100	0.29046000
H	-2.56846300	1.65909100	0.19447400
C	0.11769900	1.94162500	0.11271800
C	1.17405200	1.95433400	-0.80301900
C	-0.19741000	3.10889000	0.81317700
C	1.90471900	3.11992200	-1.01348300
H	1.41772400	1.05045100	-1.35456000
C	0.53036900	4.27399600	0.59624300
H	-1.00205800	3.09017700	1.54207400
C	1.58469000	4.28255700	-0.31554300
H	2.72389900	3.11846900	-1.72581600
H	0.28172200	5.17382900	1.14938300
H	2.15565600	5.19070300	-0.47922400
S	1.26767400	-0.80343100	1.41269200
O	1.03091500	-2.08847900	2.04331100
O	1.29884100	0.40594600	2.21601700
C	2.74916700	-0.88701000	0.45074400
C	3.05958700	-2.05533700	-0.24311300
C	3.59787500	0.21599600	0.45512900
C	4.24749400	-2.09917100	-0.96075200
H	2.37766600	-2.89756800	-0.22016000
C	4.78201000	0.14675900	-0.27046700
H	3.33035900	1.10335100	1.01824400
C	5.12012900	-1.00418200	-0.98807500
H	4.50815300	-3.00153200	-1.50591400
H	5.45597300	0.99807200	-0.27286600
C	6.39118300	-1.06668700	-1.79216000
H	6.18339900	-0.87151500	-2.84903700
H	6.84953700	-2.05603900	-1.72465300
H	7.11370100	-0.32353200	-1.44977100

P(R)

0 1

C	2.05561400	-1.51691700	-0.69532300
C	2.79633100	-0.58622600	0.28204000
C	0.59936700	-1.67707300	-0.31636100

C	2.03744000	0.71774200	0.29344700
C	0.70129100	0.71997300	0.28724400
H	2.10622000	-1.09507400	-1.70680700
H	2.75286000	-1.03797800	1.28310000
O	-0.03258500	-2.69280500	-0.49685000
C	4.24936700	-0.43791900	-0.11297600
C	5.24896500	-1.09082900	0.60923900
C	4.60937600	0.31978700	-1.23186700
C	6.58447500	-0.98629100	0.22671700
H	4.97850800	-1.68257100	1.47973000
C	5.94257500	0.42665900	-1.61594600
H	3.84023600	0.83240900	-1.80528900
C	6.93445900	-0.22661900	-0.88647900
H	7.35144100	-1.49668100	0.80048700
H	6.20774200	1.02040400	-2.48488400
H	7.97457100	-0.14271900	-1.18437400
H	2.48775900	-2.51908800	-0.72705500
N	0.01760800	-0.54556100	0.29046000
H	2.56846300	1.65909100	0.19447400
C	-0.11769900	1.94162500	0.11271800
C	-1.17405200	1.95433400	-0.80301900
C	0.19741000	3.10889000	0.81317700
C	-1.90471900	3.11992200	-1.01348300
H	-1.41772400	1.05045100	-1.35456000
C	-0.53036900	4.27399600	0.59624300
H	1.00205800	3.09017700	1.54207400
C	-1.58469000	4.28255700	-0.31554300
H	-2.72389900	3.11846900	-1.72581600
H	-0.28172200	5.17382900	1.14938300
H	-2.15565600	5.19070300	-0.47922400
S	-1.26767400	-0.80343100	1.41269200
O	-1.03091500	-2.08847900	2.04331100
O	-1.29884100	0.40594600	2.21601700
C	-2.74916700	-0.88701000	0.45074400
C	-3.05958700	-2.05533700	-0.24311300
C	-3.59787500	0.21599600	0.45512900
C	-4.24749400	-2.09917100	-0.96075200
H	-2.37766600	-2.89756800	-0.22016000

C	-4.78201000	0.14675900	-0.27046700
H	-3.33035900	1.10335100	1.01824400
C	-5.12012900	-1.00418200	-0.98807500
H	-4.50815300	-3.00153200	-1.50591400
H	-5.45597300	0.99807200	-0.27286600
C	-6.39118300	-1.06668700	-1.79216000
H	-6.18339900	-0.87151500	-2.84903700
H	-6.84953700	-2.05603900	-1.72465300
H	-7.11370100	-0.32353200	-1.44977100

$TS3_A$

0 1

C	-0.35236800	-1.71439300	-0.33943100
C	-0.03256600	-3.56313200	-1.48002200
C	2.09344600	-2.47654400	-0.74849000
H	2.43832600	-1.58379200	-1.27283900
N	-1.31259500	-3.49496000	-1.29891100
N	-1.50104400	-2.35600700	-0.57187700
N	0.61035900	-2.51744900	-0.86827500
C	0.71100100	-4.57260100	-2.30276600
H	0.16122000	-4.75390100	-3.22722800
H	0.80829100	-5.51858600	-1.75251100
C	2.67102800	-3.71951000	-1.45069800
O	1.97349300	-4.04381200	-2.63101900
C	-2.86477800	-2.11629400	-0.15577400
C	-3.19807100	-2.33470700	1.18624100
C	-3.82100100	-1.89516600	-1.15198900
C	-4.54129100	-2.20851700	1.53668900
C	-5.14907600	-1.78082600	-0.74184300
C	-5.52494300	-1.91015000	0.59434000
H	-4.82537500	-2.36983300	2.57410400
H	-5.90974200	-1.59193700	-1.49569500
H	3.70740200	-3.51091000	-1.71755300
C	-0.05359900	-0.40152100	0.46189900
O	-0.12423300	-0.52713800	1.68949000
C	1.07980700	0.42773700	-0.14592200
C	1.32831900	1.75871000	0.57089800
H	0.95854000	0.54963500	-1.22301700

H	1.98280900	-0.16092000	0.01206700
H	0.69289900	2.53897300	0.13921800
H	1.05199500	1.64385800	1.62187500
C	2.78760200	2.13443200	0.46006500
C	3.37378200	2.35002600	-0.79301300
C	3.60361600	2.17963900	1.59279800
C	4.73900100	2.59323300	-0.91101600
H	2.75078100	2.31885700	-1.68366900
C	4.97119800	2.42610500	1.48055600
H	3.16249800	2.00607600	2.57093900
C	5.54393100	2.63080100	0.22776400
H	5.17697900	2.75331500	-1.89153300
H	5.58980400	2.45007800	2.37238700
H	6.60936900	2.81858500	0.13733500
C	-6.95908900	-1.72175100	1.01205900
H	-7.64645700	-1.99663600	0.20876500
H	-7.19814800	-2.31831400	1.89546600
H	-7.14545300	-0.67164300	1.26187100
C	-2.17925300	-2.76454400	2.20682700
H	-1.59673900	-3.61605600	1.83606100
H	-1.47830600	-1.95614800	2.42857400
H	-2.68102600	-3.07307900	3.12601000
C	-3.48276100	-1.86354500	-2.61936500
H	-3.52767600	-2.87827300	-3.02868400
H	-4.20366400	-1.24068600	-3.15118200
H	-2.48684600	-1.46105600	-2.80608600
H	2.65501000	-4.57578500	-0.75898000
C	2.53379200	-2.48721000	0.72580000
H	2.04614300	-1.68572300	1.28647100
H	2.19037400	-3.42862500	1.16836800
C	4.04041500	-2.38143400	0.83422000
C	4.80371800	-3.49186600	1.20325000
C	4.69633400	-1.17916200	0.54408700
C	6.19202500	-3.40746600	1.28421500
H	4.30479200	-4.42924300	1.43607900
C	6.08333900	-1.09362200	0.62501900
H	4.13162400	-0.29178400	0.26323100
C	6.83526300	-2.20749400	0.99403200

H	6.76867700	-4.27932500	1.57592900
H	6.57063200	-0.14887500	0.40519400
H	7.91637900	-2.13860900	1.05859700
C	-2.56027600	1.03308900	0.84613100
C	-3.89243800	1.02067300	0.67674100
C	-2.04935800	1.48013600	2.18072100
C	-2.45472700	0.80480700	3.33548500
C	-1.26802700	2.62964100	2.31153900
C	-2.06456300	1.25246400	4.59414400
H	-3.07055800	-0.08495000	3.22830700
C	-0.87916500	3.08235900	3.56964100
H	-0.97880900	3.17983700	1.42087000
C	-1.27062600	2.39139700	4.71495700
H	-2.37672000	0.71039100	5.48168000
H	-0.27661600	3.98180400	3.65548100
H	-0.96338000	2.74211800	5.69519700
N	-1.59626600	0.66267300	-0.12578500
S	-1.77049500	1.06178900	-1.67832100
O	-0.89118200	0.15863300	-2.44365200
O	-3.16441000	1.17072900	-2.12251400
C	-1.08192900	2.69979700	-1.85090300
C	0.07440900	2.90266600	-2.59116100
C	-1.70146100	3.75842200	-1.18328500
C	0.63211600	4.18025700	-2.64489600
H	0.52808600	2.06689400	-3.11475300
C	-1.13040200	5.02164300	-1.24187700
H	-2.61497900	3.58515400	-0.62049100
C	0.04698200	5.25063700	-1.96836900
H	1.53930000	4.34458600	-3.22017500
H	-1.60365300	5.84883100	-0.71966900
C	0.65179200	6.62910000	-2.01423900
H	1.55815500	6.64407900	-2.62229300
H	-0.05526400	7.35006800	-2.43465900
H	0.90898500	6.97347800	-1.00834400
H	-4.35873100	0.69777200	-0.24180100
H	-4.52240900	1.36669400	1.48936200

$M3_A$

0 1

C	-0.39719100	-1.72116200	-0.38339300
C	-0.07409000	-3.67453700	-1.34884900
C	2.04953900	-2.51819600	-0.74913400
H	2.38448900	-1.65944000	-1.33203500
N	-1.35240300	-3.59995300	-1.16964300
N	-1.54243800	-2.39635700	-0.55016900
N	0.56614300	-2.56922500	-0.84564000
C	0.66661500	-4.75154300	-2.08295100
H	0.09442000	-5.03980100	-2.96536800
H	0.80050500	-5.63230800	-1.43997700
C	2.63504200	-3.79834900	-1.37806100
O	1.90768400	-4.23705800	-2.50222800
C	-2.90160100	-2.16556300	-0.11193000
C	-3.19628200	-2.37474500	1.24255700
C	-3.88942900	-1.98756600	-1.08314400
C	-4.53284000	-2.29977500	1.62572500
C	-5.21182500	-1.90468900	-0.64041400
C	-5.55076400	-2.04621500	0.70385900
H	-4.78459400	-2.45264300	2.67238800
H	-5.99562200	-1.74324300	-1.37631500
H	3.65419300	-3.58411800	-1.70095100
C	-0.14736500	-0.32363000	0.34292300
O	-0.01390400	-0.52478700	1.60602600
C	1.04536000	0.41842000	-0.31588700
C	1.36938400	1.74081700	0.38789400
H	0.92535800	0.54586900	-1.39412300
H	1.91749100	-0.20919500	-0.16157100
H	0.72534800	2.54366100	0.01467600
H	1.16254300	1.61303000	1.45321900
C	2.82149400	2.10347500	0.18775800
C	3.33011300	2.34060600	-1.09503400
C	3.70575900	2.13177800	1.26885400
C	4.68605600	2.58617400	-1.29261500
H	2.65319100	2.32537500	-1.94621900
C	5.06391800	2.38063000	1.07737100
H	3.32478700	1.94145200	2.26875000
C	5.55922500	2.60495300	-0.20484000

H	5.06308900	2.76375900	-2.29511300
H	5.73610100	2.39039700	1.92989500
H	6.61718900	2.79446500	-0.35720600
C	-6.98065900	-1.92051400	1.16062000
H	-7.67688000	-2.07067900	0.33292000
H	-7.20972600	-2.64684500	1.94440500
H	-7.16324300	-0.92315600	1.57412600
C	-2.12070600	-2.71459700	2.23543900
H	-1.58592200	-3.61975600	1.92264600
H	-1.38541000	-1.90409100	2.30285200
H	-2.55928800	-2.90295300	3.21724500
C	-3.59006700	-1.96650000	-2.55804100
H	-3.67354100	-2.98043000	-2.96321100
H	-4.31003400	-1.32865400	-3.07415800
H	-2.58872200	-1.59409400	-2.77092600
H	2.67283700	-4.59803000	-0.62258600
C	2.50402800	-2.43489500	0.71800900
H	2.01327800	-1.60064300	1.22848600
H	2.16109400	-3.34724300	1.21913500
C	4.01207100	-2.33771800	0.80181300
C	4.76498700	-3.40147000	1.30520500
C	4.68206800	-1.19231500	0.35470800
C	6.15493100	-3.32667800	1.36675700
H	4.25548300	-4.29447200	1.65820500
C	6.07077600	-1.11581600	0.41566000
H	4.12402900	-0.34244700	-0.03437000
C	6.81157000	-2.18298200	0.92066800
H	6.72258800	-4.16192600	1.76429000
H	6.56840200	-0.21384500	0.07335400
H	7.89393300	-2.12160700	0.96854500
C	-2.26743600	0.96534700	1.16063000
C	-3.59104300	0.77990800	1.20134300
C	-1.61713300	1.67585600	2.30509000
C	-1.72144800	1.13024300	3.58839200
C	-1.02269900	2.93003600	2.15564100
C	-1.22212600	1.81020200	4.69365600
H	-2.18262900	0.15335300	3.70079700
C	-0.51917600	3.61329300	3.26094700

H	-0.95920700	3.38040700	1.17047800
C	-0.61342700	3.05455200	4.53263500
H	-1.30187200	1.36717500	5.68151800
H	-0.06074800	4.58819800	3.12649500
H	-0.22019000	3.58600100	5.39344200
N	-1.47403300	0.53707100	0.04272100
S	-1.88917900	0.97805400	-1.49358700
O	-1.10873500	0.10832200	-2.37991700
O	-3.33874900	1.02688100	-1.65556200
C	-1.31537400	2.64180800	-1.75650900
C	-0.19842200	2.87329800	-2.55199100
C	-1.99909900	3.69336900	-1.14786900
C	0.25938000	4.17846800	-2.70919600
H	0.29165600	2.04132200	-3.04738500
C	-1.52417300	4.98890000	-1.31122300
H	-2.88794600	3.49494800	-0.55605200
C	-0.38549600	5.24911500	-2.08370500
H	1.12955500	4.36858900	-3.33103300
H	-2.04785600	5.81415900	-0.83755500
C	0.13647200	6.65429600	-2.22491800
H	0.73153200	6.76602400	-3.13343100
H	-0.68165700	7.37750800	-2.25278800
H	0.77518200	6.91099300	-1.37383900
H	-4.12760300	0.26514200	0.41632000
H	-4.15348100	1.18677700	2.03529300

$TS4_A$

0 1

C	-0.25628500	-1.83072400	-0.35235900
C	0.21704300	-3.61356900	-1.59539700
C	2.21595500	-2.42732400	-0.69622900
H	2.54753300	-1.51328600	-1.19516600
N	-1.07038600	-3.63882100	-1.46857700
N	-1.34240900	-2.54295900	-0.68596500
N	0.75987300	-2.55792500	-0.90408100
C	1.06886400	-4.54453200	-2.40612700
H	0.61142800	-4.69877700	-3.38441900
H	1.15796500	-5.51554100	-1.89913700

C	2.91096700	-3.62982300	-1.35352500
O	2.33641700	-3.95960400	-2.59984500
C	-2.69877200	-2.43966400	-0.21710100
C	-2.94320900	-2.66791000	1.14483200
C	-3.72598400	-2.29680700	-1.15388300
C	-4.27106600	-2.70730900	1.56330300
C	-5.03864000	-2.32812000	-0.67702100
C	-5.33022100	-2.52922800	0.67087800
H	-4.48251600	-2.88461900	2.61519100
H	-5.85184000	-2.20248900	-1.38784000
H	3.95846800	-3.37936800	-1.52560000
C	-0.13815000	-0.09637300	0.55189500
O	-0.02167300	-0.27346900	1.77677700
C	1.05687000	0.52816100	-0.18297100
C	1.39909900	1.93202100	0.34340100
H	0.94791100	0.52037300	-1.26908800
H	1.90728300	-0.09849300	0.07276900
H	0.73256300	2.68145200	-0.09588700
H	1.24857700	1.94421200	1.42658100
C	2.83499200	2.26932700	0.01971500
C	3.25035000	2.41529700	-1.30881600
C	3.79248200	2.37044700	1.03182100
C	4.58757100	2.64557200	-1.61858000
H	2.51516800	2.34247800	-2.10706900
C	5.13226800	2.60551900	0.72719100
H	3.48510100	2.25018100	2.06728000
C	5.53484500	2.73907200	-0.59935400
H	4.89178400	2.75304000	-2.65515800
H	5.86259400	2.67838000	1.52750200
H	6.57836300	2.91860900	-0.83826900
C	-6.75333100	-2.52994800	1.16523900
H	-7.45551400	-2.73737100	0.35498700
H	-6.89760700	-3.27674000	1.94992000
H	-7.01223300	-1.55392400	1.58926000
C	-1.82005900	-2.87803200	2.12330800
H	-1.12314100	-3.64316600	1.76292800
H	-1.25128800	-1.95176200	2.26616500
H	-2.21717900	-3.20524400	3.08616900

C	-3.46379700	-2.16740300	-2.63054700
H	-3.43040900	-3.15876600	-3.09363100
H	-4.26479700	-1.59272300	-3.09976900
H	-2.51594800	-1.66747500	-2.83068300
H	2.86851800	-4.49882500	-0.67770700
C	2.54031100	-2.40547800	0.80520100
H	1.96214300	-1.62260000	1.30898400
H	2.20425400	-3.35818600	1.23052800
C	4.02465500	-2.23064100	1.03994400
C	4.77353900	-3.24292700	1.64400300
C	4.67935100	-1.06101800	0.63557000
C	6.14346600	-3.09211900	1.84967300
H	4.27619000	-4.15602000	1.96139500
C	6.04773700	-0.90882700	0.83877800
H	4.12403600	-0.25191400	0.16431100
C	6.78443900	-1.92401100	1.44663000
H	6.70723600	-3.88843700	2.32524500
H	6.53328400	0.00964000	0.52311100
H	7.85123500	-1.80366100	1.60565900
C	-2.37765000	0.86377400	1.23788400
C	-3.65546800	0.47271100	1.23870400
C	-1.86731300	1.71814600	2.34920500
C	-2.14926100	1.36085400	3.67188500
C	-1.19447400	2.91706600	2.10757100
C	-1.76739300	2.18299700	4.72683400
H	-2.65356100	0.41817300	3.86411600
C	-0.80850600	3.74061500	3.16211200
H	-0.98269100	3.21443100	1.08674400
C	-1.09299500	3.37666900	4.47590100
H	-1.98733800	1.88655200	5.74777400
H	-0.29042500	4.67177700	2.95398500
H	-0.79076300	4.01656200	5.29874500
N	-1.47993300	0.52658400	0.16308400
S	-1.90061600	0.87222400	-1.41634600
O	-1.03967600	0.05544400	-2.26960300
O	-3.34603600	0.78139700	-1.57214500
C	-1.47044600	2.57062700	-1.71152600
C	-0.38089700	2.87137300	-2.52040200

C	-2.23492300	3.57428500	-1.11775900
C	-0.03176900	4.20598700	-2.71013600
H	0.17497500	2.06811000	-2.99350500
C	-1.86884400	4.89875300	-1.31478600
H	-3.09445600	3.31469300	-0.50618300
C	-0.76216100	5.23283600	-2.10725800
H	0.82173100	4.45246300	-3.33499100
H	-2.44937800	5.68979900	-0.84876700
C	-0.39414600	6.67678400	-2.32188100
H	0.60388600	6.77226900	-2.75316100
H	-1.10432500	7.15506600	-3.00366900
H	-0.42072000	7.23069200	-1.38022500
H	-4.06856600	-0.15075000	0.45872500
H	-4.31419200	0.82466200	2.02578300

SP_A

0 1

C	-1.04894200	0.63675800	-1.05953200
O	-1.07798800	1.62537500	-1.75957100
C	-2.29424600	-0.01598900	-0.50111700
C	-3.55292800	0.75713800	-0.90089100
H	-2.20525500	-0.07078000	0.59042900
H	-2.34235700	-1.05106300	-0.85453000
H	-3.45487400	1.79710600	-0.57738900
H	-3.63202100	0.77059100	-1.99205300
C	-4.78558700	0.13510000	-0.29105700
C	-5.31510400	0.62780600	0.90380000
C	-5.39652500	-0.97070900	-0.88902000
C	-6.43163500	0.03387400	1.48764100
H	-4.84852800	1.48770500	1.37797700
C	-6.51177400	-1.56861000	-0.30877600
H	-4.99564500	-1.36195100	-1.82127100
C	-7.03323400	-1.06664500	0.88234000
H	-6.83256300	0.43222800	2.41444100
H	-6.97700700	-2.42413800	-0.78832800
H	-7.90441000	-1.52980600	1.33433000
C	1.37905500	0.79115700	-1.18372600
C	2.08213000	0.29466300	-2.20138500

C	1.70265400	2.02490800	-0.42542100
C	2.60780900	2.96405300	-0.93857900
C	1.11759600	2.26897500	0.82280300
C	2.93619300	4.10106600	-0.21129000
H	3.04399100	2.81919500	-1.92139500
C	1.44781400	3.41060500	1.54899800
H	0.40607800	1.56180300	1.23828200
C	2.36011100	4.32837200	1.03824700
H	3.63673400	4.81843800	-0.62618800
H	0.98663800	3.57984600	2.51660700
H	2.61513000	5.21891800	1.60309300
N	0.20421800	0.06147400	-0.75242900
S	0.33474600	-1.24051200	0.36558200
O	-0.04759800	-0.74648700	1.68575500
O	-0.39369100	-2.36835100	-0.19979300
C	2.06027900	-1.59848600	0.37001100
C	2.89358200	-0.89764800	1.23618000
C	2.54146800	-2.58390000	-0.48745600
C	4.25306200	-1.18473300	1.22049200
H	2.48422600	-0.14737800	1.90509700
C	3.90269800	-2.85569800	-0.48307700
H	1.85911000	-3.12300300	-1.13612600
C	4.77489200	-2.15724100	0.36079600
H	4.91942200	-0.64921100	1.88965900
H	4.29721900	-3.62331300	-1.14191200
C	6.25257700	-2.44063600	0.32814700
H	6.73718200	-2.12447700	1.25370700
H	6.44552900	-3.50500600	0.17717000
H	6.72391200	-1.89958500	-0.49861500
H	1.75448900	-0.60731400	-2.70506600
H	3.00008500	0.75969000	-2.54013400

$TS3_B$

0 1

C	0.94316400	-1.88549900	-0.03480200
C	1.87354900	-3.63899800	-0.97786000
C	3.48076300	-1.75932900	-0.61735900
H	3.42269400	-0.98674000	-1.38798500

N	0.64245000	-3.98238300	-0.76473900
N	0.07875100	-2.89427900	-0.17027400
N	2.12137700	-2.36941800	-0.51997400
C	2.91744600	-4.46039500	-1.67417600
H	2.46032100	-4.97229300	-2.52171800
H	3.32536300	-5.20913500	-0.98150500
C	4.45557600	-2.84479200	-1.09516500
O	3.92669400	-3.60813800	-2.15507300
C	-1.30859900	-3.04109500	0.20166900
C	-1.61576300	-3.31164800	1.53410300
C	-2.25948900	-2.98242900	-0.81924000
C	-2.96612300	-3.45947000	1.85210900
C	-3.59071100	-3.15380700	-0.44885400
C	-3.96364000	-3.37001900	0.88006500
H	-3.24121700	-3.65389400	2.88531100
H	-4.35642700	-3.11539600	-1.22063800
H	5.35842500	-2.35221600	-1.45702200
C	0.84758700	-0.50093100	0.69214800
O	1.12849100	-0.58399200	1.89508500
C	1.28242000	0.67410100	-0.18032800
C	1.65209500	1.90084200	0.66085100
H	0.51345400	0.90543500	-0.92175800
H	2.16332300	0.37214600	-0.76019100
H	0.76670400	2.24764800	1.20177300
H	2.38355800	1.60883300	1.42249100
C	2.21701700	3.02193600	-0.18289600
C	1.45828800	3.60281100	-1.20573400
C	3.51006500	3.50253100	0.04200800
C	1.98936500	4.62015100	-1.99302000
H	0.43790300	3.26939900	-1.37542000
C	4.04503800	4.52085900	-0.74601700
H	4.10709300	3.07088800	0.84190200
C	3.28674500	5.07988800	-1.77067900
H	1.38382400	5.05729000	-2.78116500
H	5.05293800	4.87722600	-0.55459700
H	3.69975000	5.87173200	-2.38766500
C	-5.41914300	-3.48194800	1.24912700
H	-5.97626000	-4.03649000	0.48999800

H	-5.54940400	-3.98045800	2.21155300
H	-5.86659200	-2.48525100	1.32476000
C	-0.53603100	-3.42901500	2.57645000
H	0.13870800	-4.25815500	2.33810800
H	0.06717100	-2.51638900	2.63009000
H	-0.97413700	-3.61739000	3.55752700
C	-1.86312600	-2.70542400	-2.24505800
H	-1.13443300	-3.43772300	-2.60268600
H	-2.73874000	-2.74632900	-2.89588200
H	-1.41741600	-1.70734700	-2.34388200
H	4.72625500	-3.49789800	-0.25115300
C	3.98235600	-1.18626400	0.71433400
H	3.27059600	-0.47197600	1.12752700
H	4.06295200	-2.00450000	1.43819300
C	5.32003200	-0.50884300	0.51646800
C	6.49528800	-1.03522500	1.05405400
C	5.38505300	0.67257900	-0.22780600
C	7.71355600	-0.38707200	0.85674100
H	6.45602200	-1.95210800	1.63630600
C	6.59851900	1.32398200	-0.42314800
H	4.47507800	1.09977200	-0.64474200
C	7.76779400	0.79305200	0.11949200
H	8.62041600	-0.80406500	1.28275800
H	6.62578500	2.24732000	-0.99367900
H	8.71665700	1.29777500	-0.02980500
C	-1.52279400	1.24359700	0.77865000
C	-1.36343400	-0.13025300	0.58363100
C	-1.55246300	1.78033900	2.17560100
C	-1.11045100	1.01878000	3.26590600
C	-2.02855000	3.07748000	2.40718700
C	-1.14629700	1.54553100	4.55354500
H	-0.70550200	0.02551400	3.10811600
C	-2.07209000	3.59710500	3.69572100
H	-2.36250600	3.66415400	1.55924900
C	-1.63005900	2.83279600	4.77422100
H	-0.79289300	0.94745800	5.38758200
H	-2.45183300	4.60080100	3.85891400
H	-1.66257000	3.23841200	5.78061100

N	-1.60826800	2.20229700	-0.16225700
S	-2.04449800	1.87093000	-1.68541500
O	-1.93548900	3.12910800	-2.43112300
O	-1.37689800	0.69884300	-2.28963700
C	-3.77833700	1.47117700	-1.55914900
C	-4.69530700	2.50736300	-1.38300900
C	-4.19026400	0.14454400	-1.55592900
C	-6.03698900	2.20019900	-1.20567500
H	-4.35204800	3.53701700	-1.39311300
C	-5.54005700	-0.14728200	-1.37058100
H	-3.46357200	-0.64532100	-1.70814700
C	-6.47834700	0.87017400	-1.19287000
H	-6.75869700	3.00131200	-1.07184600
H	-5.87030100	-1.18280900	-1.36717500
C	-7.94012700	0.55709200	-1.00876200
H	-8.51022100	0.83117600	-1.90190800
H	-8.09695600	-0.50727400	-0.82222300
H	-8.35826700	1.11974100	-0.16985600
H	-1.52543400	-0.52331800	-0.41044500
H	-1.62128400	-0.77656200	1.41098000

M3$_B$

0 1

C	-0.70716500	1.70516400	-0.05672900
C	-1.64059500	3.57355100	-0.76254300
C	-3.26603400	1.68497000	-0.64110100
H	-3.23377000	1.00313900	-1.49475500
N	-0.41614400	3.89748100	-0.50009300
N	0.14861900	2.73669800	-0.05341200
N	-1.88995700	2.25105300	-0.48043600
C	-2.66713700	4.48829900	-1.35907700
H	-2.19484900	5.09640300	-2.13175600
H	-3.07445100	5.15171200	-0.58315800
C	-4.22021300	2.83834000	-0.98970600
O	-3.68178700	3.71758300	-1.95078600
C	1.50631500	2.85789100	0.41977700
C	1.71246900	2.99003900	1.79492100
C	2.53011000	2.93737900	-0.52453700

C	3.03001900	3.13753000	2.22654600
C	3.82831800	3.10169900	-0.04025900
C	4.09761300	3.18253400	1.32721200
H	3.22475500	3.22725400	3.29218600
H	4.64728600	3.17022500	-0.75338500
H	-5.13034500	2.40867700	-1.40743000
C	-0.51160200	0.25613400	0.52872200
O	-1.11381500	0.29686400	1.71170100
C	-1.11296200	-0.75982500	-0.50137800
C	-1.46991100	-2.09830600	0.16881500
H	-0.42614800	-0.88740900	-1.34370200
H	-2.03139100	-0.35579400	-0.92519700
H	-0.63515900	-2.80358100	0.08949600
H	-1.63030700	-1.90560800	1.23474600
C	-2.70542100	-2.74209500	-0.41681300
C	-2.88309000	-2.83410600	-1.80265500
C	-3.70658900	-3.24998000	0.41476700
C	-4.03237200	-3.40864400	-2.33985000
H	-2.11313400	-2.44912200	-2.46706600
C	-4.85595500	-3.83040200	-0.11739800
H	-3.58666400	-3.17603500	1.49258800
C	-5.02489900	-3.90875600	-1.49740200
H	-4.15289400	-3.46808800	-3.41733100
H	-5.62657300	-4.20793600	0.54776200
H	-5.92280900	-4.35387500	-1.91476500
C	5.51501300	3.29994200	1.82332600
H	6.15293700	3.79207500	1.08553300
H	5.56238000	3.86486900	2.75685900
H	5.93450400	2.30683100	2.01566200
C	0.55291200	2.96879300	2.75447700
H	-0.11830000	3.81135600	2.55337300
H	-0.03589900	2.04756100	2.64846600
H	0.90841000	3.05404100	3.78253900
C	2.24198100	2.83209800	-1.99810900
H	1.54986800	3.61693800	-2.31637200
H	3.16499000	2.92792700	-2.57294500
H	1.78940400	1.86442400	-2.24073700
H	-4.48515000	3.39150700	-0.07457100

C	-3.78572600	0.97961400	0.62129100
H	-3.14267700	0.13867100	0.88685600
H	-3.70816600	1.68536200	1.45518100
C	-5.22291900	0.54640300	0.43297300
C	-6.25690800	1.17153100	1.13504300
C	-5.54288700	-0.47845500	-0.46368400
C	-7.58209100	0.78529300	0.94742100
H	-6.01993200	1.96618000	1.83828400
C	-6.86678900	-0.86426800	-0.65449900
H	-4.75144100	-0.99356900	-1.00497900
C	-7.89087900	-0.23286400	0.04896900
H	-8.37224100	1.28027400	1.50325000
H	-7.09145600	-1.66965700	-1.34717500
H	-8.92246200	-0.53617100	-0.09879700
C	1.48171600	-1.46803500	0.69142100
C	1.06720700	-0.02334100	0.60876500
C	1.42056500	-2.15664200	2.01243600
C	0.74270400	-1.57736000	3.09214700
C	2.06236200	-3.39307500	2.18404200
C	0.70615600	-2.23315300	4.32059600
H	0.19458400	-0.64882200	2.94284200
C	2.03114800	-4.03386700	3.41402900
H	2.58629600	-3.83495900	1.34421700
C	1.35278700	-3.45392700	4.48751000
H	0.16916200	-1.78467400	5.15027200
H	2.53518900	-4.98658900	3.53945400
H	1.32949000	-3.95624200	5.44972100
N	1.95439800	-2.19562700	-0.27134300
S	2.32513200	-1.71156900	-1.82104100
O	2.11046100	-2.88753400	-2.65687600
O	1.70265900	-0.45350000	-2.24945100
C	4.06751000	-1.42784200	-1.65551500
C	4.95589400	-2.47309200	-1.88380700
C	4.50803400	-0.16782700	-1.26076600
C	6.31642200	-2.24118800	-1.71336800
H	4.58283200	-3.44113100	-2.20133000
C	5.86996900	0.04171200	-1.08770900
H	3.79704100	0.63662700	-1.09868300

C	6.79067600	-0.98901800	-1.30879300
H	7.02381600	-3.04422200	-1.89813300
H	6.22616000	1.02144000	-0.78022200
C	8.26292900	-0.76034700	-1.09204600
H	8.86251700	-1.40850300	-1.73447500
H	8.53504100	0.27809000	-1.29292300
H	8.53358300	-0.97974100	-0.05408600
H	1.55521500	0.41873800	-0.25698700
H	1.42797400	0.46698100	1.51385100

$TS4_B$

0 1

C	-0.79279100	1.79338900	0.02433100
C	-1.61731600	3.60623500	-0.96987800
C	-3.26255300	1.74849500	-0.71255000
H	-3.18918200	0.93392900	-1.43870700
N	-0.40757900	3.93766400	-0.64724100
N	0.07762700	2.81412100	-0.02797300
N	-1.90921900	2.32829500	-0.55624500
C	-2.60069400	4.43635400	-1.73914800
H	-2.08485900	4.95013100	-2.55130300
H	-3.05995700	5.18592100	-1.07925300
C	-4.19357200	2.83060400	-1.28045300
O	-3.57843300	3.59103100	-2.29785000
C	1.38115300	2.92284700	0.56803300
C	1.46496600	3.00468400	1.95890100
C	2.49832000	2.97970300	-0.27105200
C	2.74043400	3.10170600	2.51860700
C	3.74776600	3.08530600	0.33755200
C	3.88841000	3.13677000	1.72714000
H	2.83558800	3.15215300	3.60025800
H	4.63466900	3.12528100	-0.29187000
H	-5.06841200	2.34438000	-1.71327300
C	-0.57721700	0.01757400	0.82323500
O	-1.16022400	0.01484000	1.94284300
C	-1.19786900	-0.84164600	-0.29517700
C	-1.32930100	-2.32296700	0.12974700
H	-0.64259000	-0.73026100	-1.23104700

H	-2.21004200	-0.47995100	-0.46632900
H	-0.44055500	-2.89092900	-0.16344700
H	-1.40433400	-2.36705500	1.22094500
C	-2.55466800	-2.96065900	-0.48037600
C	-2.72307800	-2.98416800	-1.87007500
C	-3.56768700	-3.48686200	0.32325200
C	-3.87948700	-3.50934200	-2.43921900
H	-1.93802900	-2.58494600	-2.50846900
C	-4.72488300	-4.01979200	-0.24234200
H	-3.45285000	-3.46590400	1.40366100
C	-4.88616900	-4.02916600	-1.62537200
H	-3.99501600	-3.51702600	-3.51868600
H	-5.50507200	-4.41703900	0.39989600
H	-5.78908300	-4.43904400	-2.06747700
C	5.25697500	3.25002100	2.34681400
H	5.67592800	4.24743600	2.18122500
H	5.21999900	3.07421300	3.42351600
H	5.94758800	2.52754900	1.90306400
C	0.22762100	2.95727500	2.81681600
H	-0.50373400	3.70041800	2.48168300
H	-0.25627100	1.97330300	2.76315000
H	0.47949300	3.16876700	3.85736000
C	2.35279500	2.90832400	-1.76727500
H	1.83445100	3.79287500	-2.14788300
H	3.33423500	2.84714400	-2.24234100
H	1.77451100	2.02701500	-2.06127100
H	-4.53058200	3.49289000	-0.46685300
C	-3.81543200	1.25527600	0.63218400
H	-3.13147200	0.52938200	1.08424600
H	-3.84778000	2.11497300	1.31180800
C	-5.20465500	0.68033200	0.46412800
C	-6.31514100	1.33679400	1.00051600
C	-5.40662500	-0.50273400	-0.25601600
C	-7.59994200	0.82630100	0.82814100
H	-6.16949400	2.25634700	1.56186300
C	-6.69026000	-1.01296400	-0.43158100
H	-4.55898700	-1.04161700	-0.67726700
C	-7.79066300	-0.35040200	0.10912100

H	-8.44972500	1.34841800	1.25657500
H	-6.82332100	-1.93679900	-0.98593700
H	-8.78980900	-0.75170200	-0.02698000
C	1.55551400	-1.46002100	0.85093100
C	0.99814500	-0.05894900	0.83906200
C	1.61740400	-2.19779800	2.14495800
C	0.96369900	-1.71351400	3.28498500
C	2.34914500	-3.39213600	2.22712000
C	1.04234400	-2.41882800	4.48359000
H	0.35838300	-0.81455200	3.22194700
C	2.43250800	-4.08328200	3.42701200
H	2.85204900	-3.76139800	1.34077000
C	1.77854100	-3.59709700	4.55997400
H	0.52487800	-2.04336000	5.36033200
H	3.00762100	-5.00173100	3.48137100
H	1.84347700	-4.13817600	5.49879400
N	2.03900400	-2.10397000	-0.16159300
S	2.19325000	-1.56862000	-1.73134800
O	1.85013700	-2.71379200	-2.56716800
O	1.53015900	-0.29390700	-2.02916600
C	3.94213200	-1.29118200	-1.79822700
C	4.76429800	-2.22490600	-2.41274500
C	4.45358800	-0.13152400	-1.21708600
C	6.13590600	-1.98333000	-2.44978700
H	4.33331400	-3.11685000	-2.85475000
C	5.82206900	0.08648800	-1.25701500
H	3.79013000	0.58682600	-0.74119700
C	6.68107800	-0.83374200	-1.87521100
H	6.79214800	-2.70198400	-2.93126800
H	6.23646000	0.98385200	-0.80471100
C	8.16334300	-0.57260200	-1.91454700
H	8.69928400	-1.39835700	-2.38557400
H	8.38001000	0.34068200	-2.47644600
H	8.56003100	-0.43570800	-0.90469600
H	1.39815100	0.46059000	-0.02645900
H	1.33591700	0.44051000	1.74858500

SP_B

0 1

C	-1.77361700	1.69642300	-1.31625200
O	-2.39321000	2.71992000	-1.50625600
C	-2.40353200	0.45984700	-0.72490900
C	-3.87884400	0.65254100	-0.37356200
H	-1.82901000	0.16316400	0.16342200
H	-2.26517800	-0.36433500	-1.43678600
H	-3.96680800	1.46798600	0.35159500
H	-4.42258700	0.96702900	-1.26910200
C	-4.47971600	-0.61409100	0.18559000
C	-4.23540600	-0.98903800	1.51024800
C	-5.25455200	-1.45506100	-0.61577600
C	-4.75582500	-2.17348400	2.02307100
H	-3.63295500	-0.34288200	2.14451200
C	-5.77836700	-2.64174400	-0.10673700
H	-5.45111500	-1.17441700	-1.64742300
C	-5.53042500	-3.00405500	1.21469600
H	-4.55874200	-2.44790200	3.05458500
H	-6.38179400	-3.28187200	-0.74275100
H	-5.93875300	-3.92704200	1.61355700
C	0.60827900	1.56770300	-0.48452200
C	-0.28894000	1.60550100	-1.70444300
C	1.03551600	2.87194300	0.09052400
C	0.36829400	4.05933700	-0.23624900
C	2.11436700	2.91198200	0.98536800
C	0.77280200	5.26458700	0.33084800
H	-0.48192800	4.04929900	-0.91031800
C	2.52256900	4.11849600	1.53547600
H	2.62677400	1.98848800	1.23141400
C	1.85073200	5.29780900	1.21072600
H	0.24389900	6.17802000	0.08142400
H	3.36567200	4.14260000	2.21763500
H	2.16941400	6.24051900	1.64347700
N	1.03587900	0.52046400	0.12971000
S	0.73472700	-1.07305800	-0.28594300
O	-0.08443200	-1.60488400	0.79997700
O	0.27229700	-1.27425100	-1.66158900
C	2.37733600	-1.71743900	-0.16096300

C	3.00598600	-1.72748300	1.08473300
C	2.99881400	-2.21737600	-1.29760300
C	4.28756700	-2.24734800	1.17850600
H	2.49521300	-1.33448300	1.95782100
C	4.28546300	-2.73786900	-1.18070000
H	2.48132600	-2.19903300	-2.25024300
C	4.94400700	-2.75969900	0.04989300
H	4.79175800	-2.26106700	2.14040700
H	4.78340800	-3.13298200	-2.06074600
C	6.33013300	-3.33249100	0.17940200
H	6.31083700	-4.25346400	0.76983900
H	6.75690000	-3.56408800	-0.79784200
H	6.99490500	-2.63124800	0.69072100
H	-0.14232900	0.70081600	-2.29498500
H	-0.04877700	2.48234800	-2.30435100

TS4$_{C}$(RR)

0 1

C	-0.02380100	-1.86688300	0.30657500
C	-0.24639700	-2.78949200	2.28385200
C	-2.20110300	-1.38020600	1.62516700
H	-2.09758500	-0.29385300	1.66787600
N	0.85223700	-3.28373100	1.80133900
N	0.97533500	-2.71944100	0.57062200
N	-0.84695800	-1.94297400	1.38901200
C	-0.80406400	-3.00183400	3.65898000
H	0.01361700	-2.99208000	4.38050400
H	-1.32231400	-3.96837900	3.71854900
C	-2.70313900	-1.88187200	2.99171200
O	-1.67565700	-1.93677300	3.95475900
C	2.07690000	-3.19368900	-0.23703100
C	1.79041800	-4.00765600	-1.33850500
C	3.37165900	-2.95893200	0.22429300
C	2.87281700	-4.53095200	-2.03928600
C	4.41887000	-3.50236300	-0.52103300
C	4.19199700	-4.27579100	-1.65691100
H	2.67925500	-5.16285400	-2.90244900
H	5.43921900	-3.31847500	-0.19417900

H	-3.46535800	-1.19135000	3.35252500
C	-0.08297400	-0.97765200	-0.93111400
O	0.67245600	-1.30508900	-1.85817300
C	-0.89085800	0.17500200	-0.90700500
C	-1.20644100	0.79190100	-2.25436200
H	-1.64197900	0.27726900	-0.13876900
H	-0.69772100	1.75590500	-2.38088000
H	-0.79969100	0.12910500	-3.02451100
C	-2.69534900	0.96131300	-2.44468100
C	-3.49675600	-0.13644900	-2.77953800
C	-3.31080500	2.19879100	-2.24366500
C	-4.87657100	-0.00377200	-2.90433000
H	-3.02497800	-1.10277100	-2.94833400
C	-4.69349200	2.33582800	-2.36427000
H	-2.69826000	3.05919500	-1.98865400
C	-5.48050600	1.23539200	-2.69393100
H	-5.48210300	-0.86665400	-3.16426900
H	-5.15382300	3.30609300	-2.20050000
H	-6.55722500	1.33962700	-2.78348600
C	5.34111600	-4.82301400	-2.46252700
H	5.10617200	-5.81151200	-2.86455400
H	5.56070700	-4.16642100	-3.31029900
H	6.24751300	-4.89976900	-1.85825500
C	0.37970700	-4.32979700	-1.75018200
H	-0.25084200	-4.55257900	-0.88314400
H	-0.05794400	-3.48034600	-2.28014000
H	0.36796600	-5.19987800	-2.40861300
C	3.66420600	-2.20909800	1.49638600
H	3.72078500	-2.90381300	2.34032800
H	4.62184800	-1.69218600	1.40846900
H	2.89800500	-1.46535100	1.71704800
H	-3.16108900	-2.87562200	2.87369500
C	-3.19305100	-1.79692900	0.52873600
H	-2.80993200	-1.50970800	-0.45351800
H	-3.28661100	-2.88809800	0.54778100
C	-4.53353400	-1.14168800	0.77487000
C	-5.60845900	-1.86989200	1.28991200
C	-4.69774900	0.22428100	0.52419100

C	-6.82532500	-1.24521300	1.55508900
H	-5.49327200	-2.93435500	1.47830200
C	-5.91231100	0.84945900	0.78926500
H	-3.88432500	0.80222500	0.08735600
C	-6.97826000	0.11723700	1.30892400
H	-7.65327800	-1.82397400	1.95152900
H	-6.02589600	1.90727700	0.57269400
H	-7.92624300	0.60409900	1.51339400
C	0.49726400	1.70146200	-0.03816300
C	0.80599300	1.08691800	1.31571400
H	-0.10155300	0.84114200	1.86858300
H	1.42965500	0.20016900	1.22325400
H	1.35494900	1.83504400	1.90168100
C	-0.55276200	2.78919900	-0.00555900
C	-1.67557000	2.72678100	0.83048000
C	-0.38034000	3.93253100	-0.79383700
C	-2.60403300	3.76079100	0.86566200
H	-1.84520400	1.86181500	1.46437000
C	-1.30271800	4.97658600	-0.75135700
H	0.49569800	3.99257000	-1.42917300
C	-2.42024700	4.89440400	0.07467000
H	-3.47155200	3.67894400	1.51300500
H	-1.14454600	5.85575700	-1.36774600
H	-3.14124000	5.70482400	0.10490500
N	1.40221400	1.88243100	-0.99524400
S	2.87174400	1.18058500	-0.99715800
O	2.93841100	-0.14175400	-0.36108800
O	3.39253500	1.30052400	-2.35932900
C	3.83327700	2.27906500	0.03157900
C	4.28434500	1.84455500	1.27134600
C	4.10576800	3.57133800	-0.41859300
C	5.01611500	2.71929700	2.07333500
H	4.06862300	0.83021600	1.59226600
C	4.83507400	4.42976900	0.39093300
H	3.75365100	3.88910300	-1.39501000
C	5.29921700	4.01763000	1.64826500
H	5.37535300	2.38480300	3.04232900
H	5.05421300	5.43684900	0.04675800

C	6.09038300	4.96634700	2.51033100
H	6.38011500	4.49802300	3.45278100
H	6.99945900	5.29208600	1.99656800
H	5.50699800	5.86306200	2.73865700

TS4$_C$(RS)

0 1

C	-1.21198200	-1.74155400	-0.24530300
C	-2.38192800	-3.48881300	0.35199300
C	-3.72375900	-1.37774900	0.04846400
H	-3.61896400	-0.60906000	0.81901900
N	-1.16309200	-3.92159700	0.22585400
N	-0.44615400	-2.82705100	-0.15611100
N	-2.46267700	-2.15524600	0.04971600
C	-3.59031800	-4.22999000	0.83769700
H	-3.29699900	-4.92714400	1.62320500
H	-4.05494300	-4.78952800	0.01465900
C	-4.86800200	-2.34178200	0.41210500
O	-4.48814600	-3.29101400	1.38583700
C	0.98763900	-2.94610500	-0.27979000
C	1.58807900	-2.87739400	-1.53333600
C	1.69536400	-3.20114000	0.90548300
C	2.98348400	-2.98108100	-1.56675300
C	3.07442300	-3.32330600	0.80718900
C	3.73796500	-3.18983100	-0.41863400
H	3.48076400	-2.89268800	-2.52936700
H	3.64965700	-3.50877700	1.71172500
H	-5.69807400	-1.76343700	0.81863700
C	-0.88781200	-0.34498500	-0.77293700
O	-0.53073200	-0.30665000	-1.95048900
C	-1.04122500	0.76278800	0.08709000
C	-1.43123500	2.04869400	-0.61990000
H	-1.50953600	0.54673600	1.04272600
H	-0.60866200	2.37176400	-1.26732500
H	-2.25944800	1.83882700	-1.31837800
C	-1.86635300	3.19224700	0.27085900
C	-1.58803100	4.50787200	-0.11599800
C	-2.58094600	2.99445400	1.45690500

C	-2.00747300	5.58929800	0.65206600
H	-1.02759600	4.67960400	-1.03190700
C	-3.00379400	4.07503600	2.23121700
H	-2.82177200	1.98774100	1.78988400
C	-2.71740700	5.37686400	1.83267200
H	-1.77434400	6.60004600	0.33169600
H	-3.55497800	3.89426200	3.14889200
H	-3.04070000	6.21871400	2.43630700
C	5.24124600	-3.28232300	-0.47583900
H	5.61252200	-3.09432700	-1.48570400
H	5.69801900	-2.55160200	0.19963100
H	5.58660500	-4.27251500	-0.16347100
C	0.83363900	-2.72938600	-2.82434600
H	-0.23720400	-2.90044300	-2.70400900
H	0.97783900	-1.71516100	-3.20149900
H	1.22356200	-3.44564800	-3.55268900
C	0.99311000	-3.33859400	2.23041000
H	0.34818500	-4.22072000	2.24491400
H	1.72484300	-3.42710600	3.03369700
H	0.37334600	-2.45850100	2.44154500
H	-5.21273500	-2.85655200	-0.49796100
C	-3.99250800	-0.73505200	-1.31999800
H	-3.13856600	-0.12653800	-1.62576700
H	-4.10385800	-1.53186200	-2.06313200
C	-5.23915100	0.11768500	-1.25838900
C	-6.44054900	-0.30894600	-1.82673300
C	-5.20364100	1.34622600	-0.59206700
C	-7.58638000	0.47845300	-1.73141000
H	-6.47832500	-1.25851200	-2.35442400
C	-6.34609500	2.13382500	-0.49461300
H	-4.27116600	1.69513100	-0.15363500
C	-7.54198800	1.70001100	-1.06446100
H	-8.51309100	0.13702000	-2.18138100
H	-6.29823400	3.08747400	0.02173300
H	-8.43400000	2.31385200	-0.99373100
C	1.08618500	1.00128400	0.53364200
C	1.14525100	2.51070800	0.73136600
H	2.16872500	2.72634100	1.06665300

H	0.96123700	3.07522400	-0.17863900
H	0.45850600	2.82968800	1.51605100
C	1.22287500	0.31782300	1.88054400
C	2.41979200	-0.35169900	2.14987400
C	0.31783400	0.51300200	2.93134600
C	2.69601900	-0.83341000	3.42743700
H	3.13359800	-0.47506900	1.34221100
C	0.58321200	0.01435000	4.20306400
H	-0.59096900	1.08431300	2.76508300
C	1.77531600	-0.66255900	4.45697000
H	3.63495200	-1.34670500	3.61521300
H	-0.13659300	0.16901600	5.00070500
H	1.98607800	-1.04342100	5.45138600
N	1.74192400	0.36661900	-0.44642000
S	2.32790000	1.07008200	-1.77178200
O	2.49578100	0.04036400	-2.80213400
O	1.66323800	2.31870500	-2.19169300
C	3.98070400	1.51838700	-1.26089900
C	4.90867300	0.49901400	-1.03948400
C	4.31846100	2.84801500	-1.05189100
C	6.18351800	0.82688300	-0.60202900
H	4.62018300	-0.53377100	-1.21976400
C	5.60492600	3.16197000	-0.61312100
H	3.58066000	3.62164100	-1.23898600
C	6.54999300	2.16246900	-0.38122500
H	6.91447800	0.03983500	-0.43359900
H	5.87715600	4.20132300	-0.45193800
C	7.94010800	2.49642100	0.09424300
H	8.13666000	2.04380600	1.07083800
H	8.69252700	2.11126500	-0.60009100
H	8.07971500	3.57538100	0.18520400

TS4$_{C}$(SR)

0 1

C	-0.22734500	-0.21778500	-0.74936800
C	-0.93777800	-1.59503600	-2.28935900
C	-2.74594200	-0.66618300	-0.82190300
H	-2.86204400	-1.08555800	0.17682800

N	0.31840700	-1.39302800	-2.56987000
N	0.74373100	-0.51817500	-1.61532500
N	-1.32676500	-0.86038900	-1.20734300
C	-1.90408900	-2.49367500	-3.00305200
H	-1.44515100	-3.47535500	-3.13988700
H	-2.14681400	-2.07148200	-3.98799500
C	-3.60988100	-1.41131100	-1.85054900
O	-3.06120400	-2.65871200	-2.22031000
C	1.99601600	0.16358900	-1.84233500
C	1.91377000	1.46741300	-2.35385300
C	3.19612500	-0.52818800	-1.69769400
C	3.11241700	2.12384300	-2.61316300
C	4.36707100	0.17140100	-1.99417400
C	4.34746100	1.49551400	-2.42291400
H	3.08198600	3.14136400	-2.99504800
H	5.32018300	-0.33545400	-1.87190000
H	-4.59062800	-1.59849100	-1.41072300
C	-0.24613100	0.54592000	0.57336100
O	-1.13681400	0.15425000	1.33960800
C	0.73101400	1.51947400	0.86968500
C	0.25185100	2.64301100	1.77967400
H	1.36967400	1.82661900	0.04981200
H	-0.19673200	2.22723200	2.68387400
H	1.09616100	3.26721600	2.09211100
C	-0.77844800	3.50240800	1.07645100
C	-2.13704700	3.37294600	1.37430800
C	-0.39548100	4.39697900	0.07051400
C	-3.09540300	4.10785900	0.67617800
H	-2.44704000	2.67499300	2.14791700
C	-1.34756200	5.13408600	-0.62714000
H	0.66105500	4.50616900	-0.16960300
C	-2.70292800	4.98859000	-0.32801600
H	-4.14806800	3.97991200	0.91411100
H	-1.03326600	5.82384700	-1.40475600
H	-3.44607900	5.56134600	-0.87365700
C	5.62806900	2.24204800	-2.68361900
H	5.58099000	2.79069500	-3.62810700
H	5.81040400	2.96952100	-1.88596900

H	6.48228400	1.56267700	-2.71652900
C	0.58691500	2.12327800	-2.64363400
H	-0.05349900	1.46499400	-3.24133900
H	0.04357800	2.38062000	-1.72727700
H	0.73951100	3.04673800	-3.20477600
C	3.22822000	-1.96018100	-1.24908900
H	2.64786500	-2.59537300	-1.92580200
H	4.25668000	-2.32531500	-1.21825600
H	2.78940000	-2.03256900	-0.24969200
H	-3.74267400	-0.78515800	-2.74615500
C	-3.10975400	0.82674900	-0.83720300
H	-2.33720800	1.40861700	-0.33110400
H	-3.15499400	1.17250000	-1.87615100
C	-4.42306500	1.07445800	-0.13635100
C	-5.53889900	1.55102400	-0.82340400
C	-4.51612000	0.84639600	1.24167000
C	-6.73065100	1.80637600	-0.14592700
H	-5.47160200	1.73431600	-1.89278000
C	-5.70438600	1.10002200	1.91846500
H	-3.63910200	0.48723500	1.77740300
C	-6.81499000	1.58286100	1.22542100
H	-7.59144000	2.18129100	-0.69063000
H	-5.76418200	0.92640400	2.98819500
H	-7.74131400	1.78376700	1.75384300
C	2.05457400	0.13820100	1.97092800
C	1.75091400	0.65043800	3.36431400
H	2.04306400	1.69568600	3.46685700
H	0.70158600	0.51061200	3.61964800
H	2.35260700	0.05895300	4.06563200
C	3.40194100	0.60033500	1.45321500
C	3.72585600	1.93446000	1.18769500
C	4.42288800	-0.35634100	1.40301500
C	5.02587200	2.29650600	0.85001700
H	2.96207900	2.70259700	1.24446300
C	5.72589200	0.00675300	1.07253600
H	4.17650100	-1.38712400	1.63751200
C	6.03195100	1.33423200	0.78666800
H	5.25374900	3.33743200	0.64003500

H	6.50243300	-0.75177100	1.03899100
H	7.04570600	1.62008400	0.52292300
N	1.76592200	-1.12178300	1.60822100
S	1.01083500	-2.21471400	2.52399600
O	2.02428800	-3.15553100	3.03754400
O	0.05111000	-1.71996800	3.52233700
C	0.07685600	-3.11584100	1.30169600
C	0.74975300	-3.91362400	0.38130900
C	-1.31338700	-3.06692300	1.32357500
C	0.01794200	-4.67142500	-0.52794100
H	1.83346500	-3.96373400	0.39728900
C	-2.03193200	-3.84046600	0.41717700
H	-1.80566500	-2.42847100	2.05006000
C	-1.38000100	-4.66142100	-0.51018700
H	0.53919900	-5.30097800	-1.24422900
H	-3.11868200	-3.81567700	0.42898900
C	-2.17218500	-5.54992700	-1.43348100
H	-1.56375700	-5.89294200	-2.27440000
H	-2.52298600	-6.43810200	-0.89800000
H	-3.04847000	-5.02418800	-1.81998900

TS4$_C$(SS)

0 1

C	0.85340400	0.29526200	-1.18232900
C	0.09525900	-0.41695600	-3.10389100
C	-1.46687300	1.13135400	-1.90498400
H	-1.95368100	0.81931600	-0.97888800
N	1.31813900	-0.85478400	-3.04425300
N	1.78141900	-0.39623700	-1.85093000
N	-0.21372800	0.35059700	-2.01454800
C	-0.94988000	-0.75740000	-4.12242500
H	-0.86661900	-1.81869800	-4.35420200
H	-0.81543600	-0.16236300	-5.03590800
C	-2.35033000	0.79878400	-3.12557100
O	-2.22293600	-0.53455600	-3.56055100
C	3.15684300	-0.62051700	-1.49535000
C	3.97480000	0.48882800	-1.23680800
C	3.61296600	-1.94247900	-1.41734300

C	5.28426600	0.23160500	-0.83538700
C	4.93271500	-2.13455200	-1.00658400
C	5.77715500	-1.06679600	-0.70307500
H	5.93162900	1.07606500	-0.61156500
H	5.30565700	-3.15185900	-0.91954000
H	-3.39482700	0.96124600	-2.85199900
C	0.87721000	0.80263100	0.24307600
O	0.05240500	1.70503300	0.52632700
C	1.69540800	0.12925900	1.14575400
C	1.87395300	0.76523400	2.49977000
H	2.49480300	-0.50037900	0.78085400
H	0.90972500	1.14408400	2.85286200
H	2.24134100	0.03611000	3.23057500
C	2.86479300	1.90146100	2.34955600
C	2.42486000	3.21872900	2.18920300
C	4.23359800	1.63250400	2.24130100
C	3.33333400	4.24313800	1.92594200
H	1.36105900	3.42800500	2.24925000
C	5.14226500	2.65340200	1.97756200
H	4.58485800	0.60809100	2.34765800
C	4.69390400	3.96426400	1.81670100
H	2.97636600	5.26118600	1.80310700
H	6.20144600	2.42610500	1.89699300
H	5.40047300	4.76174800	1.61003800
C	7.20052000	-1.30158800	-0.26981000
H	7.88780900	-1.15751100	-1.10944200
H	7.49107600	-0.59977300	0.51606500
H	7.33797300	-2.31807100	0.10405700
C	3.49667600	1.91421400	-1.33577900
H	2.75157000	2.04730600	-2.12517500
H	3.05681100	2.24444300	-0.38702200
H	4.34204400	2.57068200	-1.55080100
C	2.73977800	-3.12124200	-1.75809000
H	2.69934300	-3.26356100	-2.84181300
H	3.14902700	-4.02826800	-1.30835100
H	1.71622700	-2.97725600	-1.40322300
H	-2.10090500	1.48125000	-3.95180800
C	-1.21759200	2.64871200	-1.87488600

H	-0.38957900	2.85675800	-1.19906000
H	-0.95580600	2.99137100	-2.88134800
C	-2.46748300	3.33936100	-1.37850300
C	-3.29145700	4.07423900	-2.23121400
C	-2.82843300	3.20311500	-0.03173300
C	-4.45887300	4.66711700	-1.75043400
H	-3.01704300	4.18641900	-3.27721600
C	-3.99377100	3.79254400	0.44729700
H	-2.17415400	2.63817000	0.63011700
C	-4.81311100	4.52568000	-0.41186300
H	-5.09018900	5.23852200	-2.42339600
H	-4.26154800	3.68652000	1.49422400
H	-5.72146800	4.98647100	-0.03745000
C	0.39387400	-1.86982200	1.38251200
C	1.60552300	-2.57895600	1.94049200
H	1.25764400	-3.55956400	2.29364300
H	2.32240900	-2.75555100	1.13756200
H	2.08582200	-2.05603500	2.76766500
C	-0.56339700	-1.24497200	2.32775800
C	-0.50279700	-1.51313200	3.70274700
C	-1.54318400	-0.36053500	1.85614500
C	-1.42489700	-0.93761900	4.57170300
H	0.24695500	-2.19202600	4.09514800
C	-2.45546400	0.22237300	2.72458200
H	-1.57593600	-0.13465200	0.79974000
C	-2.40223900	-0.06985800	4.08667100
H	-1.37947200	-1.16777300	5.63104100
H	-3.21215600	0.89627000	2.33334300
H	-3.11674300	0.37926300	4.76914200
N	0.07368300	-2.21441700	0.13924600
S	-1.12082700	-3.06349600	-0.48819300
O	-1.10144500	-2.79807400	-1.93214400
O	-0.98860300	-4.46322000	-0.05719700
C	-2.71421000	-2.54031100	0.11326600
C	-3.42875300	-1.59156300	-0.60877200
C	-3.18285400	-3.02223100	1.33340100
C	-4.60512600	-1.07720600	-0.06950500
H	-3.06192300	-1.28589500	-1.58483300

C	-4.36354800	-2.50785000	1.85213500
H	-2.61977600	-3.78093800	1.86727600
C	-5.07734600	-1.51359700	1.17186400
H	-5.16768300	-0.32899500	-0.62190500
H	-4.73308200	-2.86949300	2.80780800
C	-6.31789800	-0.91867000	1.78412400
H	-6.05014600	-0.24962100	2.60894500
H	-6.88566600	-0.34056700	1.05249800
H	-6.96849200	-1.69594100	2.19296000

M4$_{C}$(RR)

0 1

C	0.18817600	1.73341600	0.21481700
C	0.78117800	2.84804400	2.01474300
C	2.37834400	1.03929900	1.41568900
H	2.17452700	-0.01625000	1.59829200
N	-0.16713600	3.55155900	1.48505100
N	-0.51410100	2.86611900	0.35823000
N	1.08037600	1.74857200	1.24643300
C	1.46334800	3.09829100	3.32648100
H	0.70721500	3.27811000	4.09191300
H	2.11816800	3.97732500	3.25630700
C	3.09205800	1.62299500	2.65182300
O	2.19038300	1.94776800	3.68339300
C	-1.40896300	3.54826600	-0.54916500
C	-0.87478400	4.00567800	-1.76391700
C	-2.68467900	3.89693500	-0.10375400
C	-1.72524300	4.71026800	-2.60850900
C	-3.49690800	4.59990700	-0.99849400
C	-3.04945400	4.98868800	-2.25700900
H	-1.33836400	5.06903100	-3.55906900
H	-4.50359100	4.86404700	-0.68353000
H	3.78785500	0.87598900	3.03391400
C	-0.18601100	0.60271200	-0.84292600
O	-0.51618200	1.06236100	-1.96772500
C	0.69387400	-0.71221000	-0.75987900
C	1.20934800	-1.14629100	-2.13184600
H	1.55219600	-0.62735200	-0.09916500

H	0.43791800	-1.69229200	-2.68075200
H	1.39578800	-0.23401600	-2.70766700
C	2.46821500	-1.97621600	-2.05038900
C	3.64508100	-1.55243600	-2.67219400
C	2.49179100	-3.17439500	-1.32546900
C	4.81733100	-2.30008100	-2.57375300
H	3.64248300	-0.62246800	-3.23525900
C	3.66260700	-3.92057000	-1.21813900
H	1.57902900	-3.52790600	-0.85074300
C	4.83082700	-3.48472000	-1.84338700
H	5.72312500	-1.94927500	-3.05835800
H	3.66214500	-4.84648200	-0.65116600
H	5.74440100	-4.06477100	-1.75923800
C	-3.95964700	5.71050800	-3.21593800
H	-3.41462900	6.46987800	-3.78218200
H	-4.38990300	5.00890700	-3.93778100
H	-4.78546600	6.19474800	-2.69040200
C	0.57602000	3.81765400	-2.11980400
H	1.22247700	4.08773900	-1.27673100
H	0.76294700	2.77431600	-2.38165000
H	0.84040200	4.45719000	-2.96383100
C	-3.17655800	3.65834000	1.30000900
H	-3.07313700	4.58540200	1.87440600
H	-4.23189700	3.38103200	1.28231800
H	-2.62895800	2.87152100	1.81411900
H	3.66820000	2.51484900	2.36083500
C	3.26810200	1.24343300	0.17660100
H	2.79242400	0.82173600	-0.71530900
H	3.36675500	2.32177700	0.00904700
C	4.63063200	0.62292900	0.39362700
C	5.75723500	1.42809100	0.57965700
C	4.77393900	-0.76675800	0.45582900
C	7.00460800	0.85873200	0.82489500
H	5.65695200	2.50921500	0.52706000
C	6.01944800	-1.33677600	0.70350500
H	3.91440900	-1.41357400	0.28610000
C	7.13722600	-0.52606800	0.89092300
H	7.87093500	1.49730200	0.96362400

H	6.11398200	-2.41749900	0.73627100
H	8.10799600	-0.97178600	1.08184700
C	-0.41763000	-1.49897100	0.01553700
C	0.06860300	-1.83982200	1.42686400
H	0.97076700	-2.45921500	1.37373700
H	0.30123100	-0.91791000	1.96593100
H	-0.68416400	-2.37625300	2.00674200
C	-1.06414200	-2.67940900	-0.70120200
C	-1.05687300	-3.97309100	-0.17810300
C	-1.73506900	-2.45034300	-1.91100700
C	-1.70998700	-5.01384700	-0.84130800
H	-0.55323600	-4.18461000	0.75976400
C	-2.37587200	-3.48691100	-2.57782400
H	-1.76638600	-1.43570800	-2.30201500
C	-2.37137700	-4.77509100	-2.04054400
H	-1.70187800	-6.01089800	-0.41165600
H	-2.89087600	-3.28857000	-3.51260000
H	-2.88109700	-5.58385700	-2.55453100
N	-1.29668100	-0.30727600	-0.07537800
S	-2.47808400	0.09445700	0.97705800
O	-1.93601500	0.54989900	2.27037200
O	-3.38410400	1.00716100	0.27763200
C	-3.31572000	-1.44710900	1.24753600
C	-3.18101700	-2.11412800	2.45829200
C	-4.05638500	-1.98910000	0.19746800
C	-3.76624100	-3.37015600	2.60295500
H	-2.61736600	-1.65554000	3.26424500
C	-4.62827800	-3.24150600	0.35658900
H	-4.15777200	-1.44158100	-0.73370800
C	-4.47676100	-3.95533100	1.55304200
H	-3.66174900	-3.90522000	3.54217100
H	-5.18726300	-3.68427700	-0.46287600
C	-5.06032500	-5.33692200	1.68171600
H	-4.96294900	-5.71899100	2.69950100
H	-6.11911800	-5.34324900	1.40909900
H	-4.54545800	-6.02778100	1.00610600

M4$_C$(RS)

0 1

C	-0.55602300	-1.53711800	0.01926000
C	-1.44485000	-3.20682800	1.13489300
C	-3.12652600	-1.53159700	0.33620400
H	-3.15059000	-0.53795500	0.79043400
N	-0.18139500	-3.48461100	1.06753600
N	0.35732000	-2.44320800	0.36432500
N	-1.73617200	-2.03811600	0.47813200
C	-2.49305300	-3.94425300	1.90927500
H	-2.04698300	-4.35260100	2.81660300
H	-2.91288500	-4.76642400	1.31353000
C	-4.07402300	-2.47152000	1.11354500
O	-3.49176000	-3.02011400	2.27428900
C	1.76412200	-2.53619000	0.06690000
C	2.13644700	-2.88059800	-1.23551100
C	2.65806400	-2.47267600	1.13717000
C	3.49614900	-3.08014900	-1.46760600
C	4.00898500	-2.67171600	0.84677800
C	4.44355500	-2.96786000	-0.44638000
H	3.82182400	-3.33642800	-2.47262300
H	4.73286100	-2.61125100	1.65581600
H	-4.95113400	-1.90048100	1.41875900
C	-0.46469500	-0.21739200	-0.81523200
O	-0.88221100	-0.42251800	-2.01104000
C	-1.02742600	0.97611000	0.05243900
C	-1.87847500	1.89733000	-0.81320100
H	-1.57979400	0.66825300	0.94268400
H	-1.28484600	2.25386700	-1.66020500
H	-2.66653100	1.28198900	-1.26465300
C	-2.49952700	3.05758600	-0.06829400
C	-2.37326400	4.36270400	-0.55203300
C	-3.23590800	2.85791500	1.10541500
C	-2.95843800	5.43642500	0.11448700
H	-1.80545600	4.53643200	-1.46270000
C	-3.82533700	3.92739000	1.77532300
H	-3.35948100	1.85332000	1.50595900
C	-3.68590300	5.22280000	1.28284900
H	-2.84421200	6.44126700	-0.28002200

H	-4.39244300	3.74694200	2.68322000
H	-4.14207800	6.05785600	1.80455900
C	5.90870400	-3.14208000	-0.75237300
H	6.08292400	-4.04494300	-1.34377300
H	6.27979000	-2.29405800	-1.33806600
H	6.50143000	-3.20748400	0.16239800
C	1.10342800	-3.10632400	-2.30605300
H	0.47944000	-3.96877500	-2.04219800
H	0.45871200	-2.23170400	-2.43659400
H	1.59306400	-3.31641600	-3.25826400
C	2.18062300	-2.29823200	2.55418400
H	1.78829000	-3.24803100	2.93072800
H	3.00305800	-1.98312300	3.19762900
H	1.38366700	-1.55277700	2.62934500
H	-4.40487700	-3.28127300	0.44528600
C	-3.57908000	-1.47069500	-1.13038600
H	-2.90469700	-0.82226400	-1.69580100
H	-3.47668100	-2.47700900	-1.55301400
C	-5.01963600	-1.01950900	-1.22421800
C	-6.00704800	-1.88454700	-1.70173200
C	-5.39668600	0.26692400	-0.82100300
C	-7.33679600	-1.47685300	-1.78307800
H	-5.72861500	-2.88618900	-2.01908500
C	-6.72459300	0.67617000	-0.89715900
H	-4.64975600	0.96363800	-0.44659100
C	-7.69981800	-0.19504100	-1.37930300
H	-8.08759600	-2.16276600	-2.16231100
H	-6.99576400	1.67909800	-0.58304900
H	-8.73482500	0.12505400	-1.44094400
C	0.41474600	1.43572900	0.36975400
C	0.71787500	2.92162200	0.14594200
H	1.78134900	3.11957900	0.30058500
H	0.43139800	3.26953300	-0.84556500
H	0.16056100	3.48567000	0.89915300
C	0.90545400	1.11212800	1.77853100
C	2.27949200	0.95773200	1.99480700
C	0.05388900	1.11813900	2.88465100
C	2.78944000	0.81717500	3.27924900

H	2.95125300	0.95229600	1.14289100
C	0.56186400	0.95055900	4.17488200
H	-1.01153700	1.28731500	2.75718900
C	1.92969300	0.80327600	4.37731200
H	3.86015300	0.70389600	3.42254200
H	-0.11716600	0.95182800	5.02167100
H	2.32466300	0.68303400	5.38096800
N	0.97475400	0.41193000	-0.60241900
S	1.73246400	0.86794200	-2.01830400
O	1.95453500	-0.34543100	-2.79863100
O	1.10097600	2.01563900	-2.67910800
C	3.32295400	1.41816100	-1.44090100
C	4.19507100	0.45866700	-0.93308400
C	3.69024900	2.75404400	-1.53245200
C	5.44524300	0.85570100	-0.47935000
H	3.87629400	-0.57714500	-0.87901200
C	4.95070400	3.13583300	-1.07823500
H	2.99400800	3.47686400	-1.94503200
C	5.84031000	2.19878200	-0.54594800
H	6.12518800	0.11635800	-0.06301100
H	5.24743900	4.17902400	-1.13684600
C	7.20908500	2.61529800	-0.07609800
H	7.51109700	2.04825100	0.80757800
H	7.95430700	2.43057100	-0.85644100
H	7.23765300	3.67939100	0.16670100

M4$_C$(SR)

0 1

C	-0.30999900	-0.37546100	-0.64906400
C	-1.30397400	-1.39716800	-2.31107100
C	-2.86426600	-0.36141900	-0.64412700
H	-2.99386600	-0.80373200	0.34173200
N	-0.04539100	-1.37342000	-2.64253200
N	0.55861300	-0.72382900	-1.60393800
N	-1.51786300	-0.75449900	-1.12167900
C	-2.43840600	-2.05871700	-3.03871400
H	-2.14245100	-3.07345800	-3.31536900
H	-2.68343400	-1.49629600	-3.95021500

C	-3.89370000	-0.88303900	-1.65854600
O	-3.55246400	-2.14997600	-2.18311700
C	1.87728800	-0.17197900	-1.80557100
C	1.91319700	1.14877900	-2.27720000
C	3.02067000	-0.94339000	-1.61354300
C	3.16403900	1.72607800	-2.47148300
C	4.24982400	-0.32319100	-1.84400600
C	4.34184300	1.00554400	-2.25026900
H	3.22028700	2.75575400	-2.81667600
H	5.15916100	-0.89477600	-1.67955400
H	-4.85943400	-0.98181900	-1.16090600
C	-0.05083800	0.08226900	0.84296500
O	-1.13540600	0.08946300	1.52473600
C	0.99700900	1.23834700	1.06439200
C	0.44350900	2.47910800	1.75923500
H	1.51991600	1.51230200	0.14620400
H	-0.36219300	2.16292500	2.42866000
H	1.23438300	2.92627400	2.37377800
C	-0.07065800	3.52118000	0.79317500
C	-1.42821700	3.84279300	0.73429000
C	0.80794100	4.18553900	-0.07156300
C	-1.90530400	4.77848000	-0.18313600
H	-2.12214400	3.34371100	1.40693500
C	0.34036900	5.13050700	-0.97925500
H	1.87028700	3.94862700	-0.04154100
C	-1.02187800	5.42461200	-1.04360500
H	-2.96788800	4.99940700	-0.22071500
H	1.03768800	5.63492300	-1.64138400
H	-1.38868400	6.15625500	-1.75629400
C	5.68463900	1.64584300	-2.48457300
H	5.95091800	1.60917200	-3.54596100
H	5.67600400	2.69603700	-2.18219800
H	6.46523900	1.13053800	-1.92071500
C	0.64163100	1.90274000	-2.57330200
H	0.01062900	1.33922600	-3.26924800
H	0.05506300	2.09486800	-1.66656700
H	0.86771300	2.87210000	-3.01958500
C	2.94317300	-2.37718200	-1.17962400

H	2.19691300	-2.92043200	-1.76701600
H	3.91150600	-2.86524300	-1.30564900
H	2.65966800	-2.43388600	-0.12433800
H	-3.99943800	-0.16128500	-2.48307600
C	-2.94448700	1.16703900	-0.55109200
H	-2.05834900	1.51362300	-0.02015200
H	-2.93963900	1.59520700	-1.56059700
C	-4.16843400	1.60095900	0.21531600
C	-5.27172100	2.17590600	-0.41643900
C	-4.19617200	1.40929200	1.60256700
C	-6.39063000	2.55585100	0.32434200
H	-5.25225800	2.33208200	-1.49227400
C	-5.31228900	1.78918600	2.34118000
H	-3.32091500	0.97113300	2.07971700
C	-6.41292000	2.36211000	1.70283300
H	-7.24271500	3.00475000	-0.17618000
H	-5.32419700	1.64280300	3.41664300
H	-7.28266500	2.65954100	2.27972200
C	1.88374300	0.26409700	1.88952700
C	1.72889100	0.41888400	3.40471700
H	2.21591900	1.34472500	3.72269600
H	0.67500600	0.43302700	3.68676800
H	2.21882900	-0.41233900	3.91990800
C	3.35886100	0.28096200	1.52767300
C	3.97448400	1.49046400	1.19631900
C	4.14415500	-0.87192800	1.61269900
C	5.34246900	1.54896700	0.93929800
H	3.38284300	2.40114100	1.14428900
C	5.50932300	-0.81713500	1.34313200
H	3.67737500	-1.81425000	1.88349800
C	6.11438600	0.39238600	1.00518100
H	5.80234900	2.49814600	0.68075000
H	6.10402700	-1.72388300	1.40208100
H	7.17960700	0.43371300	0.79897900
N	1.11983400	-0.89283900	1.30403400
S	0.72461700	-2.25632700	2.17509000
O	1.93322200	-3.09022600	2.25737600
O	0.02429500	-1.99813800	3.43318100

C	-0.41878100	-3.06177000	1.07839000
C	0.07089700	-3.88421100	0.07062800
C	-1.78785500	-2.91935000	1.29868700
C	-0.83047100	-4.55138300	-0.75562000
H	1.13891800	-4.02491200	-0.04229600
C	-2.67080100	-3.60209300	0.47277000
H	-2.13008100	-2.26756500	2.09483500
C	-2.20813100	-4.42666400	-0.56232500
H	-0.45608400	-5.19704900	-1.54485400
H	-3.74165900	-3.49861100	0.62801100
C	-3.18513700	-5.17907200	-1.42519700
H	-2.67485100	-5.71210500	-2.23045000
H	-3.73615100	-5.91332200	-0.82989400
H	-3.91220500	-4.48929100	-1.86149700

M4$_{C}$(SS)

0 1

C	-0.72474300	-1.11584200	-0.66580100
C	-0.21928800	-2.68204300	-2.10873700
C	1.64650500	-2.08195300	-0.55438500
H	2.02492800	-1.06509200	-0.49291400
N	-1.46704900	-2.40663700	-2.34604900
N	-1.77061500	-1.44334300	-1.43048800
N	0.26004400	-1.95925400	-1.05189500
C	0.71598200	-3.51849200	-2.92501600
H	0.37638800	-3.52764400	-3.96056500
H	0.76480300	-4.54966600	-2.54928700
C	2.47680400	-2.92834900	-1.54836100
O	1.98511000	-2.90207400	-2.87148200
C	-3.13975000	-1.02318300	-1.30770800
C	-3.88376000	-1.57423000	-0.25814400
C	-3.67606300	-0.15680700	-2.25875500
C	-5.19747000	-1.13831800	-0.11297800
C	-5.00087500	0.24391700	-2.07180200
C	-5.76446800	-0.21618100	-0.99886300
H	-5.79401100	-1.53533400	0.70495300
H	-5.44299800	0.93422200	-2.78568100
H	3.49486500	-2.53405200	-1.56979600

C	-0.43795500	0.11894300	0.26078700
O	0.54010900	-0.12082000	1.05673300
C	-1.64102200	0.98977100	0.80868000
C	-1.68895000	1.11077300	2.32470000
H	-2.61537700	0.67310300	0.42652800
H	-0.67549700	1.26277200	2.69951000
H	-2.27756200	2.00054800	2.58034800
C	-2.28748100	-0.10867700	2.98139300
C	-1.46166700	-1.12504700	3.47232200
C	-3.67336800	-0.25366600	3.09728500
C	-2.00965800	-2.25485800	4.07776100
H	-0.38647900	-1.01837900	3.35872100
C	-4.22379800	-1.38123400	3.70186800
H	-4.32446400	0.52881500	2.71333400
C	-3.39205100	-2.38480500	4.19680000
H	-1.35608700	-3.03342600	4.45936700
H	-5.30217000	-1.47577700	3.78976200
H	-3.81942300	-3.26231200	4.67166400
C	-7.17588800	0.26738700	-0.79137000
H	-7.83040800	-0.55113400	-0.48235400
H	-7.20827900	1.02762200	-0.00464300
H	-7.58072800	0.71173800	-1.70253000
C	-3.28357300	-2.61359700	0.64897700
H	-2.82889800	-3.41835800	0.06182400
H	-2.50896600	-2.18736900	1.29524800
H	-4.04946300	-3.04233600	1.29551200
C	-2.87221600	0.30110000	-3.44529100
H	-2.88955700	-0.46543000	-4.22704200
H	-3.29137400	1.21975700	-3.86052500
H	-1.82482400	0.47134500	-3.18388600
H	2.51999700	-3.97039300	-1.19773000
C	1.72312700	-2.70986700	0.84339700
H	0.99021100	-2.21074200	1.47795000
H	1.49358000	-3.77943300	0.78509500
C	3.12081300	-2.48643500	1.37869600
C	4.01240600	-3.54136000	1.57777400
C	3.55128300	-1.17615400	1.62702500
C	5.31271400	-3.29770000	2.02009900

H	3.68742700	-4.56100400	1.38590900
C	4.84863500	-0.93295900	2.06576600
H	2.85020000	-0.35798400	1.47086600
C	5.73462200	-1.99325100	2.26227800
H	5.99513800	-4.12799400	2.17200900
H	5.16968000	0.08760600	2.25470900
H	6.74689600	-1.80212400	2.60444700
C	-1.16252000	2.23483800	-0.02476500
C	-2.27440700	2.81780400	-0.88792900
H	-1.89671900	3.59637400	-1.55362200
H	-2.71564500	2.02350600	-1.48917900
H	-3.06186200	3.23580700	-0.25269700
C	-0.41751800	3.30520500	0.77340900
C	-0.88797500	4.61873900	0.85908100
C	0.75462100	2.97621500	1.46849700
C	-0.20701400	5.57786300	1.60933800
H	-1.78955900	4.91576500	0.33589600
C	1.43320700	3.93123400	2.21850700
H	1.12372400	1.95482100	1.40769200
C	0.95635600	5.23933400	2.29157600
H	-0.59024700	6.59243000	1.65358900
H	2.34141400	3.65189500	2.74455700
H	1.48729700	5.98586700	2.87369100
N	-0.27491600	1.30453900	-0.76150700
S	0.67500300	1.54337500	-2.05582800
O	0.51331200	0.37383800	-2.92949700
O	0.37653700	2.88075600	-2.57136600
C	2.39098800	1.51201100	-1.56691300
C	3.19264900	0.44662300	-1.97507600
C	2.91297500	2.54841700	-0.79972300
C	4.51546400	0.39528200	-1.55092900
H	2.77113700	-0.32823700	-2.60935000
C	4.23755200	2.47796100	-0.37939700
H	2.29168500	3.39520000	-0.52962600
C	5.05157200	1.39832100	-0.73584200
H	5.14584100	-0.43623600	-1.85573600
H	4.64543100	3.27645400	0.23384800
C	6.48467000	1.31717500	-0.28095200

H	6.70786000	0.32089200	0.11158300
H	7.16676000	1.50392500	-1.11618600
H	6.69570200	2.05181900	0.49878800

TS5$_C$(RR)
0 1

C	-0.24378300	1.85401800	-0.17799800
C	-0.74402600	2.92445000	-2.06061600
C	-2.38434000	1.16611000	-1.40712800
H	-2.18154100	0.10512100	-1.56891000
N	0.23232300	3.60407500	-1.55166900
N	0.52030000	2.93715800	-0.38733500
N	-1.09875700	1.87658600	-1.24365000
C	-1.41971700	3.14669700	-3.38027800
H	-0.66601500	3.27718600	-4.15805500
H	-2.05078200	4.04535700	-3.34134400
C	-3.09147900	1.73029800	-2.65326900
O	-2.18499400	2.00623600	-3.69724300
C	1.42307200	3.57695500	0.53156600
C	0.92259300	3.94131200	1.79034700
C	2.70618500	3.92705900	0.10222700
C	1.79113700	4.58745200	2.66605200
C	3.53403400	4.57290300	1.02289400
C	3.10595600	4.89243600	2.30881200
H	1.42497700	4.87449600	3.64877600
H	4.54209300	4.83981700	0.71419400
H	-3.80815800	0.99392700	-3.01754500
C	0.37041900	0.42985400	1.04107800
O	0.75994300	0.93313600	2.08938500
C	-0.65100100	-0.73732600	0.90612100
C	-1.17737800	-1.25073600	2.24293200
H	-1.49896400	-0.47506600	0.27735800
H	-0.42062100	-1.85561300	2.75048600
H	-1.34153000	-0.37161400	2.87594900
C	-2.46236100	-2.03206400	2.10791800
C	-3.63475800	-1.59213400	2.72634500
C	-2.51099000	-3.19958500	1.33653700
C	-4.82756900	-2.29859800	2.58335900
H	-3.61182500	-0.68479600	3.32447800

C	-3.70279500	-3.90375500	1.18440700
H	-1.60211000	-3.56509700	0.86365600
C	-4.86557600	-3.45557900	1.81055100
H	-5.72940200	-1.93808300	3.06804600
H	-3.72231600	-4.80756700	0.58323400
H	-5.79478700	-4.00430400	1.69370300
C	4.03529600	5.55348400	3.29346300
H	3.49520400	6.24772700	3.94191300
H	4.51019900	4.80538800	3.93643300
H	4.82903600	6.10254800	2.78205400
C	-0.51177700	3.70062400	2.18521100
H	-1.19732400	3.99813600	1.38449500
H	-0.67321100	2.64227000	2.40244500
H	-0.75477900	4.28440100	3.07522500
C	3.19826000	3.70684100	-1.30438100
H	3.02854700	4.61100900	-1.89848300
H	4.27049400	3.50105300	-1.29141900
H	2.69453600	2.87722500	-1.79671900
H	-3.64311900	2.64460600	-2.38276000
C	-3.27843800	1.36999700	-0.17178500
H	-2.78988700	0.96445400	0.72100000
H	-3.39119800	2.44811200	-0.01133500
C	-4.63093200	0.72724500	-0.38082100
C	-5.76624900	1.51287700	-0.59623400
C	-4.75619700	-0.66528000	-0.41553000
C	-7.00314400	0.92260100	-0.84486800
H	-5.67975300	2.59606400	-0.56546700
C	-5.99091100	-1.25656400	-0.66865300
H	-3.88944500	-1.29699900	-0.22404900
C	-7.11684100	-0.46486900	-0.88605400
H	-7.87599500	1.54683300	-1.00660700
H	-6.07066500	-2.33885400	-0.68345700
H	-8.07895200	-0.92728500	-1.08119500
C	0.37874300	-1.52654500	0.01199500
C	-0.16786000	-1.77701500	-1.39169000
H	-1.08988800	-2.36611700	-1.33788800
H	-0.38041300	-0.82056600	-1.87395700
H	0.54730700	-2.30996800	-2.02124200

C	1.03399900	-2.74845700	0.64595700
C	0.98929900	-4.01144900	0.05474500
C	1.74393900	-2.59564700	1.84581900
C	1.64188300	-5.09694100	0.64261200
H	0.45337700	-4.16456200	-0.87639100
C	2.38405800	-3.67698900	2.43770200
H	1.81186600	-1.60667500	2.29370200
C	2.34043700	-4.93416000	1.83332400
H	1.60309400	-6.06904000	0.16086700
H	2.92956200	-3.53704100	3.36556400
H	2.84896900	-5.77783300	2.28889000
N	1.28480200	-0.35094300	0.12556500
S	2.46075900	0.11825400	-0.92157900
O	1.89188300	0.60611100	-2.18507300
O	3.35363400	1.00708800	-0.18317600
C	3.27799600	-1.41834800	-1.25373000
C	3.09451500	-2.05189400	-2.47639500
C	4.03942900	-1.99732400	-0.23921900
C	3.65423200	-3.31265100	-2.66932900
H	2.51470000	-1.56488900	-3.25344400
C	4.58708100	-3.25323700	-0.44779800
H	4.17614200	-1.47506000	0.70204600
C	4.38674000	-3.93445900	-1.65608600
H	3.51173600	-3.82273800	-3.61726200
H	5.16335600	-3.72552700	0.34255200
C	4.93930000	-5.32289800	-1.83443600
H	4.82096300	-5.67174400	-2.86179900
H	6.00057100	-5.35995900	-1.57488300
H	4.41650600	-6.02175400	-1.17314400

TS5$_C$(RS)

0 1

C	-0.33568500	-1.71798300	-0.17610700
C	-0.91418800	-3.52761800	0.99374200
C	-2.82368200	-2.05416500	0.36276600
H	-2.96641100	-1.22045300	1.05895300
N	0.36743700	-3.63192900	0.84742000
N	0.70241700	-2.51205400	0.12254800

N	-1.38931400	-2.39982700	0.36859000
C	-1.81894400	-4.46041000	1.74228500
H	-1.33986300	-4.76965900	2.67198900
H	-2.02199800	-5.35313100	1.13423200
C	-3.60714500	-3.25759800	0.89712200
O	-3.01743700	-3.79077600	2.06428800
C	2.08474100	-2.40090800	-0.25694200
C	2.42526000	-2.51808900	-1.60904200
C	3.04190400	-2.34112200	0.76184500
C	3.78378500	-2.52375900	-1.92827500
C	4.38655400	-2.37271100	0.39002200
C	4.77540700	-2.45341100	-0.94802400
H	4.07167800	-2.59876500	-2.97377200
H	5.14558400	-2.32735400	1.16787000
H	-4.61731000	-2.93420200	1.15207200
C	-0.43252700	0.05707900	-1.21629000
O	-0.73388200	-0.19376000	-2.37752000
C	-1.22146100	0.92502500	-0.21130700
C	-2.10736000	1.89478400	-0.98671400
H	-1.82033600	0.37509800	0.51218000
H	-1.49983400	2.46892600	-1.69594300
H	-2.76931700	1.28595700	-1.61683300
C	-2.96883400	2.82292300	-0.15646800
C	-3.84369300	3.69197800	-0.82011700
C	-2.97501400	2.81943900	1.24043500
C	-4.69709600	4.53026100	-0.11242300
H	-3.85466900	3.70095200	-1.90717100
C	-3.83194800	3.65769700	1.95551900
H	-2.30379700	2.16288400	1.78867900
C	-4.69606700	4.51464600	1.28293000
H	-5.36683900	5.19512900	-0.64869200
H	-3.81830100	3.63792500	3.04064700
H	-5.36237100	5.16681800	1.83798200
C	6.23161100	-2.41129400	-1.33147400
H	6.42486800	-3.01275500	-2.22268500
H	6.53450100	-1.38269900	-1.55702900
H	6.86660400	-2.77541200	-0.52066100
C	1.37559200	-2.68239500	-2.67340900

H	0.70186900	-3.50926200	-2.42312400
H	0.77746100	-1.77392500	-2.78465800
H	1.84886500	-2.90281500	-3.63200900
C	2.64663200	-2.27982500	2.21171000
H	2.45099300	-3.28422600	2.59898700
H	3.44501200	-1.82863500	2.80419100
H	1.73819800	-1.68918600	2.35152500
H	-3.67303200	-4.03176100	0.11640100
C	-3.32033500	-1.68368200	-1.03714400
H	-2.64945600	-0.95187400	-1.49526900
H	-3.26814100	-2.57798400	-1.66821300
C	-4.73623600	-1.15400000	-0.99558500
C	-5.77776600	-1.81948600	-1.64462400
C	-5.02441700	0.02585700	-0.30106500
C	-7.07537800	-1.31126800	-1.61189100
H	-5.56886200	-2.73857800	-2.18564300
C	-6.31709500	0.53907700	-0.26911200
H	-4.23232700	0.56040200	0.21741800
C	-7.34815500	-0.12985700	-0.92690400
H	-7.87282100	-1.83916700	-2.12500400
H	-6.51209400	1.46412500	0.26507200
H	-8.35760500	0.26762000	-0.90595800
C	0.13989500	1.46362700	0.32443100
C	0.29738200	2.98685400	0.32488100
H	1.33236500	3.26823700	0.52680500
H	-0.00483900	3.44127000	-0.61657000
H	-0.31626300	3.39021200	1.13333600
C	0.58315100	0.94408900	1.69426500
C	1.89353300	1.21182600	2.11715900
C	-0.27505700	0.30617400	2.59334400
C	2.32070200	0.87726100	3.39600000
H	2.58717100	1.70354700	1.44200200
C	0.15223700	-0.03425900	3.87933000
H	-1.29997900	0.07653400	2.32167400
C	1.44734000	0.25718500	4.28934100
H	3.34010200	1.10191600	3.69474300
H	-0.53968900	-0.52248600	4.55796300
H	1.77712000	-0.00061700	5.29036400

N	0.85349200	0.68498000	-0.77978800
S	1.77771600	1.38656200	-1.98908700
O	2.13487200	0.31558800	-2.91102700
O	1.15536600	2.59722000	-2.53074700
C	3.22970300	1.86915300	-1.09729900
C	4.02503600	0.84896800	-0.58450700
C	3.54960300	3.20831600	-0.91611900
C	5.15552000	1.18143500	0.14700600
H	3.73666700	-0.18332100	-0.74384600
C	4.68727300	3.52481900	-0.17672600
H	2.91189400	3.97971700	-1.33541000
C	5.49980200	2.52312700	0.36403400
H	5.77370600	0.38973500	0.56366800
H	4.94801000	4.56664000	-0.01667000
C	6.73828900	2.87328800	1.14534300
H	6.86236000	2.20800500	2.00314200
H	7.62810700	2.76771100	0.51671500
H	6.70277100	3.90327300	1.50493600

TS5$_C$(SR)

0 1

C	-0.18829400	-0.24059200	-0.86976100
C	-0.98115000	-1.76927100	-2.27839700
C	-2.68207700	-0.62041300	-0.83539400
H	-2.80534200	-1.01873100	0.17351100
N	0.27724300	-1.67252400	-2.58867400
N	0.74212300	-0.72338000	-1.70231800
N	-1.30593300	-0.90353400	-1.26495300
C	-2.02439100	-2.65319900	-2.89579200
H	-1.65913100	-3.68183900	-2.94500400
H	-2.24800200	-2.30942200	-3.91577700
C	-3.62006800	-1.32881700	-1.81555500
O	-3.18943200	-2.64735900	-2.09961100
C	2.02408800	-0.11556300	-1.92656000
C	2.03036200	1.15251400	-2.52307200
C	3.19760400	-0.80115900	-1.61139000
C	3.26605700	1.74900000	-2.76565400
C	4.40924000	-0.17233900	-1.89392100

C	4.46326000	1.09895100	-2.46201400
H	3.29408400	2.73663500	-3.22047300
H	5.33431800	-0.68463200	-1.64249900
H	-4.61774200	-1.39771400	-1.37886500
C	-0.01299600	0.34623600	1.15353500
O	-1.14400300	0.22375100	1.60591500
C	0.90877700	1.56955800	1.16070900
C	0.34115500	2.82079700	1.81911800
H	1.28266100	1.77915500	0.15578300
H	-0.11276500	2.56152200	2.77928000
H	1.16288400	3.51822100	2.02609000
C	-0.69217600	3.49129600	0.94332500
C	-2.03443900	3.53796100	1.32255100
C	-0.32160500	4.05953200	-0.28009700
C	-2.98868300	4.13216400	0.49865700
H	-2.33933600	3.08424400	2.26171100
C	-1.27239100	4.64456400	-1.11148800
H	0.72343900	4.03825900	-0.58598000
C	-2.61173800	4.68132300	-0.72366900
H	-4.03128700	4.13973200	0.80398000
H	-0.96703700	5.07324000	-2.06144200
H	-3.35534200	5.13389300	-1.37207400
C	5.78790400	1.74815900	-2.76653900
H	6.09031700	1.54755200	-3.79978600
H	5.73258200	2.83290700	-2.64354200
H	6.57053000	1.36506100	-2.10737200
C	0.74604900	1.83389900	-2.92172400
H	0.14421200	1.18061200	-3.56311400
H	0.13206700	2.08433100	-2.05090000
H	0.95681700	2.75491400	-3.46917900
C	3.15322600	-2.15959600	-0.97428400
H	2.50131500	-2.83445900	-1.53698000
H	4.15427600	-2.59253500	-0.92041600
H	2.75933000	-2.07625600	0.04368500
H	-3.68670400	-0.74789000	-2.74978200
C	-2.93371600	0.89052800	-0.82671500
H	-2.12956400	1.37007000	-0.26149300
H	-2.89700900	1.26713300	-1.85632100

C	-4.25634500	1.22125500	-0.18243100
C	-5.33860100	1.69216900	-0.92532100
C	-4.39632500	1.07098500	1.20241600
C	-6.54090200	2.01907000	-0.29853200
H	-5.23456400	1.81834400	-2.00050900
C	-5.59509800	1.39455000	1.82911600
H	-3.53918500	0.72634300	1.77763300
C	-6.67101300	1.87224500	1.07962600
H	-7.37381000	2.39041700	-0.88755700
H	-5.69061100	1.28271800	2.90490500
H	-7.60485700	2.12894500	1.56965500
C	1.93851700	0.68450000	1.93250600
C	1.91584300	0.89181200	3.44846600
H	2.35798900	1.86413400	3.68286900
H	0.89964900	0.84474300	3.84487500
H	2.51445800	0.11807400	3.93813600
C	3.36934900	0.74775100	1.43617900
C	3.86356900	1.94254200	0.90877700
C	4.25254700	-0.31754600	1.63714100
C	5.21545800	2.07909300	0.59999500
H	3.19514400	2.78510100	0.74966500
C	5.59902700	-0.18716900	1.31110600
H	3.87779600	-1.24783600	2.05292700
C	6.08793400	1.01432600	0.80009700
H	5.58320500	3.01785500	0.19644300
H	6.27212600	-1.02504700	1.46670000
H	7.14167000	1.11832300	0.55918600
N	1.13684900	-0.52854500	1.48927000
S	0.88671600	-1.89068500	2.44120400
O	2.19182200	-2.54044100	2.58222000
O	0.13936300	-1.59685900	3.66125200
C	-0.13520000	-2.89233800	1.39448700
C	0.47080100	-3.77838900	0.51070500
C	-1.52058600	-2.84403400	1.54294900
C	-0.33229000	-4.60905000	-0.26602600
H	1.55098000	-3.83927700	0.45485000
C	-2.30295500	-3.68477500	0.76279300
H	-1.95835200	-2.14628600	2.24836200

C	-1.72377600	-4.57903800	-0.14725600
H	0.13142100	-5.30524500	-0.95917400
H	-3.38504500	-3.65283400	0.85875500
C	-2.59413900	-5.50617400	-0.95197200
H	-2.01282600	-6.04692600	-1.70233900
H	-3.07108800	-6.24417900	-0.29961000
H	-3.38400400	-4.94116200	-1.45372600

TS5$_C$(SS)

0 1

C	-0.25287600	-1.15195400	-0.72081000
C	0.78717400	-2.21267200	-2.37662300
C	2.26041600	-1.41300800	-0.51315300
H	2.47169800	-0.35051200	-0.38513700
N	-0.47259800	-2.34178200	-2.65882300
N	-1.09305700	-1.69050900	-1.61944300
N	0.96256500	-1.53523100	-1.19653200
C	1.97042200	-2.65683700	-3.18195500
H	1.79395000	-2.45351100	-4.23898900
H	2.14316500	-3.73453000	-3.05105500
C	3.33365200	-2.08291400	-1.39050900
O	3.10064500	-1.91548200	-2.77531900
C	-2.51370600	-1.80468200	-1.46903000
C	-2.99596100	-2.43903900	-0.31412400
C	-3.36280700	-1.32856800	-2.47356000
C	-4.37797600	-2.49536100	-0.13707200
C	-4.73752700	-1.42501300	-2.25322500
C	-5.26259300	-1.98215200	-1.08720800
H	-4.76894800	-2.96887300	0.76069600
H	-5.41422300	-1.04722400	-3.01589800
H	4.30466100	-1.63796800	-1.16516900
C	-0.29156600	0.54707800	0.52971400
O	0.68638900	0.44437400	1.25991500
C	-1.77084600	0.76502600	0.91389000
C	-2.05096000	0.94599500	2.40171500
H	-2.41758600	-0.01371800	0.50168200
H	-1.25216200	1.53379800	2.86132000
H	-2.98109000	1.51515100	2.51383700

C	-2.17389400	-0.38984100	3.09546300
C	-1.04728000	-1.02408500	3.62744000
C	-3.40973600	-1.03893400	3.16754100
C	-1.15649100	-2.27928400	4.22315900
H	-0.08175100	-0.53221000	3.54895000
C	-3.52202600	-2.29183600	3.76542500
H	-4.29037700	-0.55629500	2.74955900
C	-2.39408600	-2.91576800	4.29565000
H	-0.27326600	-2.75996000	4.63249400
H	-4.49021800	-2.78095100	3.81790800
H	-2.47943100	-3.89193200	4.76231800
C	-6.75058400	-2.02463200	-0.85418900
H	-7.07909500	-1.13933300	-0.30000400
H	-7.29866900	-2.04542300	-1.79861500
H	-7.03350100	-2.90273500	-0.26915200
C	-2.07019700	-3.07336800	0.69182000
H	-1.28429800	-3.64604300	0.18914400
H	-1.58109900	-2.32651300	1.32502000
H	-2.63127900	-3.74498600	1.34338100
C	-2.82992900	-0.73832800	-3.75227200
H	-2.56277200	-1.53400000	-4.45423100
H	-3.58942700	-0.10897500	-4.22109200
H	-1.92927400	-0.14528000	-3.57743700
H	3.38175200	-3.15857800	-1.15633800
C	2.21987700	-2.07624500	0.87054000
H	1.36151400	-1.67521100	1.41551000
H	2.08808400	-3.15792700	0.75054400
C	3.50339500	-1.76803600	1.60606300
C	4.49558300	-2.73315800	1.78738200
C	3.73358400	-0.46414100	2.06262800
C	5.69840700	-2.40679600	2.41307000
H	4.32479800	-3.74836000	1.43756400
C	4.93439900	-0.13706300	2.68588100
H	2.95707700	0.28339000	1.91842500
C	5.92159400	-1.10767500	2.86158400
H	6.45996300	-3.16817300	2.54893500
H	5.10187100	0.87711200	3.03689800
H	6.85756900	-0.85208700	3.34790300

C	-1.79694100	2.00850800	-0.05379700
C	-2.85312500	1.89314400	-1.14265800
H	-2.78906300	2.72690500	-1.84640700
H	-2.70158300	0.95937100	-1.68532900
H	-3.85428700	1.87101800	-0.70231900
C	-1.77964400	3.35693400	0.65315600
C	-2.84609700	4.25168500	0.55651400
C	-0.68899200	3.70322000	1.46178900
C	-2.81959100	5.46812400	1.23956600
H	-3.70630300	4.01491700	-0.05994500
C	-0.66083400	4.91411300	2.14288900
H	0.13567600	3.00287200	1.56339900
C	-1.72783500	5.80529500	2.03141500
H	-3.65622400	6.15290500	1.14469300
H	0.19485300	5.16295300	2.76244200
H	-1.70603700	6.75332900	2.55886200
N	-0.42868900	1.61376800	-0.48976400
S	0.24807500	1.96158700	-1.94685600
O	-0.04640100	0.93753900	-2.95361900
O	-0.15679600	3.33362900	-2.25289700
C	1.97774600	1.83987100	-1.59167700
C	2.78957400	1.15179900	-2.48660600
C	2.48842900	2.39366400	-0.41882400
C	4.13783600	0.99148200	-2.18073400
H	2.36126500	0.70578000	-3.37696000
C	3.83268800	2.21275900	-0.12615000
H	1.83416300	2.92839300	0.26068600
C	4.67019000	1.49328200	-0.99036800
H	4.77170500	0.42901300	-2.86011500
H	4.23954800	2.61546800	0.79716800
C	6.09918700	1.22081300	-0.60633900
H	6.13915400	0.38276700	0.09998800
H	6.70425700	0.96265800	-1.47758600
H	6.54745300	2.08550900	-0.11101300

SP_C(RR)

0 1

C	-0.93375800	-0.60802900	1.11185900

O	-1.04715300	-1.80509800	1.14318600
C	-1.78450100	0.59342400	0.69644600
C	-2.43398000	0.56434200	-0.68623100
H	-2.53917100	0.79991300	1.46331900
H	-1.71738800	0.20238300	-1.42919700
H	-2.68603300	1.59453600	-0.96130000
C	-3.67554500	-0.29812900	-0.69437500
C	-3.57975100	-1.68120900	-0.86863800
C	-4.93583100	0.27049700	-0.49659700
C	-4.72091900	-2.47842000	-0.84811600
H	-2.60222900	-2.13377700	-1.00818600
C	-6.07958500	-0.52447200	-0.47618800
H	-5.02132800	1.34616700	-0.36260100
C	-5.97448000	-1.90189600	-0.65301800
H	-4.63107900	-3.55137100	-0.98597900
H	-7.05243400	-0.06685100	-0.32641000
H	-6.86444100	-2.52279900	-0.64027400
C	-0.50301600	1.48117000	0.95383600
C	-0.68406800	2.48878100	2.07797900
H	0.25724700	2.99257500	2.30630100
H	-1.03552000	1.97083700	2.97335300
H	-1.43337600	3.23432700	1.80002800
C	0.14974100	2.04126600	-0.29810600
C	0.12713400	3.41192700	-0.56746700
C	0.75376400	1.18792000	-1.22979800
C	0.69818900	3.91737900	-1.73376000
H	-0.32948000	4.10112900	0.13409700
C	1.33019500	1.69231700	-2.39076500
H	0.78851600	0.11695300	-1.04705500
C	1.30458700	3.06133500	-2.64749100
H	0.67259400	4.98580200	-1.92161400
H	1.80287300	1.01134600	-3.09142700
H	1.75452500	3.45695400	-3.55211800
N	0.14141800	0.21819200	1.42243300
S	1.65833900	-0.09922300	2.07201100
O	1.45912700	-1.07018900	3.13411300
O	2.23504300	1.20857300	2.34073800
C	2.54018500	-0.87499600	0.75323500

C	2.17917700	-2.17162600	0.38700000
C	3.51084500	-0.15663400	0.06579400
C	2.81521600	-2.75071100	-0.70186700
H	1.40439100	-2.70003600	0.93405900
C	4.14271400	-0.76280600	-1.01585900
H	3.75304200	0.85638400	0.36865000
C	3.80408000	-2.05845600	-1.41451400
H	2.54390400	-3.75729400	-1.00626500
H	4.90555400	-0.21699800	-1.56273000
C	4.49803800	-2.71671900	-2.57688800
H	3.78297900	-3.24695200	-3.21067500
H	5.22610400	-3.45140600	-2.21882100
H	5.03068300	-1.98542700	-3.18748100

SP$_C$(RS)

0 1

C	0.66886600	-2.05392600	-1.06375300
O	0.63027900	-3.01168800	-1.78345200
C	1.64652600	-0.90386400	-0.83413400
C	3.08158700	-1.33355100	-0.57841500
H	1.59753400	-0.19417300	-1.66667600
H	3.11955000	-2.03622300	0.26183300
H	3.40326700	-1.90710600	-1.45611400
C	4.06389900	-0.20719100	-0.33359400
C	5.41749500	-0.52509400	-0.17203500
C	3.68138800	1.13303300	-0.26055100
C	6.36328400	0.46770100	0.05300400
H	5.72809000	-1.56572700	-0.22595700
C	4.62905200	2.13248500	-0.03336500
H	2.63809400	1.41521900	-0.37846600
C	5.97095700	1.80457400	0.12286500
H	7.40831300	0.20010600	0.17364000
H	4.31046600	3.16880200	0.01979900
H	6.70778800	2.58180700	0.29734800
C	0.71262300	-0.44868700	0.35271800
C	1.29248400	-0.66099000	1.74271200
H	0.54099500	-0.46138600	2.50911400
H	1.61809100	-1.69584100	1.85957600

H	2.15048500	-0.00103600	1.89834700
C	0.05403800	0.90784700	0.14293500
C	-0.66820200	1.14587400	-1.03344200
C	0.13714600	1.92530500	1.09442000
C	-1.30386000	2.36168200	-1.24536100
H	-0.76537700	0.35708300	-1.77555300
C	-0.49670900	3.14993400	0.88032700
H	0.69177700	1.77306800	2.01392300
C	-1.22140200	3.37056500	-0.28487300
H	-1.87303900	2.51952300	-2.15636800
H	-0.42161200	3.92847300	1.63263000
H	-1.71873600	4.32150200	-0.44787400
N	-0.18376500	-1.57869600	-0.06270500
S	-1.58658300	-2.18115200	0.64164700
O	-1.92379800	-3.37602700	-0.11145700
O	-1.35438800	-2.24198000	2.07749200
C	-2.76612000	-0.90698500	0.32212900
C	-3.43160800	-0.89505700	-0.89901500
C	-2.91547800	0.11896000	1.25273100
C	-4.26292400	0.18035800	-1.19182600
H	-3.29761200	-1.71068200	-1.60236200
C	-3.74319200	1.18675400	0.93658200
H	-2.38382300	0.07930200	2.19773000
C	-4.41530100	1.23809200	-0.29017900
H	-4.79331700	0.20432000	-2.13898200
H	-3.86177100	2.00030600	1.64623400
C	-5.25456700	2.43676600	-0.64018900
H	-4.60685500	3.27788500	-0.91085600
H	-5.91256800	2.23100500	-1.48640900
H	-5.86470500	2.75313000	0.20920500

SP_C(SR)

0 1

C	-0.66886600	-2.05392600	-1.06375300
O	-0.63027900	-3.01168800	-1.78345200
C	-1.64652600	-0.90386400	-0.83413400
C	-3.08158700	-1.33355100	-0.57841500
H	-1.59753400	-0.19417300	-1.66667600

H	-3.11955000	-2.03622300	0.26183300
H	-3.40326700	-1.90710600	-1.45611400
C	-4.06389900	-0.20719100	-0.33359400
C	-5.41749500	-0.52509400	-0.17203500
C	-3.68138800	1.13303300	-0.26055100
C	-6.36328400	0.46770100	0.05300400
H	-5.72809000	-1.56572700	-0.22595700
C	-4.62905200	2.13248500	-0.03336500
H	-2.63809400	1.41521900	-0.37846600
C	-5.97095700	1.80457400	0.12286500
H	-7.40831300	0.20010600	0.17364000
H	-4.31046600	3.16880200	0.01979900
H	-6.70778800	2.58180700	0.29734800
C	-0.71262300	-0.44868700	0.35271800
C	-1.29248400	-0.66099000	1.74271200
H	-0.54099500	-0.46138600	2.50911400
H	-1.61809100	-1.69584100	1.85957600
H	-2.15048500	-0.00103600	1.89834700
C	-0.05403800	0.90784700	0.14293500
C	0.66820200	1.14587400	-1.03344200
C	-0.13714600	1.92530500	1.09442000
C	1.30386000	2.36168200	-1.24536100
H	0.76537700	0.35708300	-1.77555300
C	0.49670900	3.14993400	0.88032700
H	-0.69177700	1.77306800	2.01392300
C	1.22140200	3.37056500	-0.28487300
H	1.87303900	2.51952300	-2.15636800
H	0.42161200	3.92847300	1.63263000
H	1.71873600	4.32150200	-0.44787400
N	0.18376500	-1.57869600	-0.06270500
S	1.58658300	-2.18115200	0.64164700
O	1.92379800	-3.37602700	-0.11145700
O	1.35438800	-2.24198000	2.07749200
C	2.76612000	-0.90698500	0.32212900
C	3.43160800	-0.89505700	-0.89901500
C	2.91547800	0.11896000	1.25273100
C	4.26292400	0.18035800	-1.19182600
H	3.29761200	-1.71068200	-1.60236200

C	3.74319200	1.18675400	0.93658200
H	2.38382300	0.07930200	2.19773000
C	4.41530100	1.23809200	-0.29017900
H	4.79331700	0.20432000	-2.13898200
H	3.86177100	2.00030600	1.64623400
C	5.25456700	2.43676600	-0.64018900
H	4.60685500	3.27788500	-0.91085600
H	5.91256800	2.23100500	-1.48640900
H	5.86470500	2.75313000	0.20920500

SP_C(SS)

0 1

C	0.93259500	-0.60873100	1.11187600
O	1.04550600	-1.80585700	1.14313800
C	1.78434400	0.59226900	0.69726400
C	2.43426300	0.56446000	-0.68519400
H	2.53916400	0.79747100	1.46445000
H	1.71797700	0.20350500	-1.42894800
H	2.68651000	1.59493900	-0.95901500
C	3.67571300	-0.29822100	-0.69412500
C	3.57914700	-1.68163500	-0.86544100
C	4.93653700	0.27038200	-0.49971900
C	4.72009500	-2.47915700	-0.84557000
H	2.60120300	-2.13412100	-1.00222300
C	6.08006700	-0.52494600	-0.47983100
H	5.02261200	1.34625200	-0.36776700
C	5.97421800	-1.90264800	-0.65390400
H	4.62967300	-3.55234900	-0.98118100
H	7.05331300	-0.06737500	-0.33252200
H	6.86398900	-2.52382700	-0.64148200
C	0.50338300	1.48069400	0.95466500
C	0.68418300	2.48753600	2.07940700
H	-0.25722100	2.99132400	2.30747000
H	1.03515100	1.96912800	2.97470800
H	1.43364800	3.23323500	1.80235500
C	-0.14903800	2.04200600	-0.29707900
C	-0.12478700	3.41282400	-0.56578700
C	-0.75485600	1.19006000	-1.22882700

C	-0.69584500	3.91964800	-1.73145300
H	0.33301400	4.10113400	0.13587000
C	-1.33148500	1.69585300	-2.38910200
H	-0.79074500	0.11902500	-1.04684900
C	-1.30407500	3.06492100	-2.64521500
H	-0.66885900	4.98811500	-1.91885100
H	-1.80561500	1.01580700	-3.08968500
H	-1.75396900	3.46162200	-3.54938400
N	-0.14202500	0.21797600	1.42247900
S	-1.65901100	-0.10003300	2.07155800
O	-1.45979100	-1.07128400	3.13339200
O	-2.23604900	1.20761500	2.34054400
C	-2.54041400	-0.87574000	0.75241100
C	-2.17933300	-2.17228600	0.38591400
C	-3.51051300	-0.15699800	0.06452400
C	-2.81494800	-2.75097500	-0.70337400
H	-1.40507800	-2.70107400	0.93331400
C	-4.14199400	-0.76281000	-1.01758100
H	-3.75254700	0.85608700	0.36732300
C	-3.80337700	-2.05841200	-1.41636900
H	-2.54371600	-3.75757300	-1.00780100
H	-4.90459000	-0.21678700	-1.56456700
C	-4.49679800	-2.71650700	-2.57916000
H	-3.78099200	-3.24423300	-3.21424300
H	-5.22286300	-3.45337700	-2.22152400
H	-5.03154100	-1.98556100	-3.18830500

TS5$_D$(RR)

0 1

C	-0.60901800	1.92863600	-0.43612900
C	-0.19173800	4.04678700	-0.00780700
C	1.77759500	2.52286100	0.28999000
H	1.71461800	1.65991800	0.95535900
N	-1.45396900	4.02087900	-0.29364900
N	-1.70368100	2.71022700	-0.60337800
N	0.39090200	2.80765600	-0.11957300
C	0.59017500	5.16805000	0.59425000
H	-0.09499700	5.88843400	1.04029500

H	1.21796100	5.67331000	-0.15256300
C	2.35843200	3.75058800	1.04689500
O	1.38397300	4.59687300	1.61595500
C	-3.01656200	2.32465400	-1.03014500
C	-3.21010200	1.95803300	-2.36316900
C	-4.03547300	2.29027600	-0.07219800
C	-4.48225900	1.51546600	-2.72538000
C	-5.28906500	1.84681300	-0.48574100
C	-5.52531000	1.44182600	-1.80221100
H	-4.65870200	1.21568000	-3.75527200
H	-6.09578400	1.79645900	0.24169500
H	2.99250200	3.39270400	1.85905400
C	-0.53205100	0.50362500	-0.50092400
O	0.72253600	-0.00956400	-0.70714100
C	-1.65102300	-0.27871500	-0.32361700
C	-1.70273600	-1.68637800	-0.33300300
H	-2.56054000	0.24840800	-0.06147300
H	-0.95021400	-2.21919700	-0.90634500
C	-2.99334600	-2.36835100	-0.21523500
C	-3.12522000	-3.67864000	-0.71149800
C	-4.11137300	-1.79538800	0.42020100
C	-4.31905000	-4.37913900	-0.58887800
H	-2.26491800	-4.13807300	-1.19323000
C	-5.30582800	-2.49849300	0.53927700
H	-4.04847600	-0.79404100	0.83910500
C	-5.42016600	-3.79348300	0.03580500
H	-4.39255200	-5.38756700	-0.98507500
H	-6.15307400	-2.03076200	1.03261500
H	-6.35364000	-4.33867800	0.12998700
C	-6.87277700	0.90116000	-2.20242500
H	-6.94916100	-0.15757200	-1.93332900
H	-7.68000200	1.43086200	-1.69127200
H	-7.02934900	0.98331400	-3.27993400
C	-2.08153200	2.00239500	-3.35693700
H	-1.52126000	2.93857000	-3.27950900
H	-1.37958100	1.17932000	-3.18162400
H	-2.46457800	1.90975500	-4.37416100
C	-3.75697000	2.66475500	1.35833500

H	-3.53429100	3.73173700	1.45182800
H	-4.61519200	2.42951000	1.99030800
H	-2.88788700	2.11739700	1.74261200
H	2.98346200	4.33373600	0.35463600
C	2.70014200	2.18723600	-0.89328500
H	2.26314900	1.36885300	-1.46616900
H	2.78319800	3.06901300	-1.53849100
C	4.05620700	1.78834200	-0.35540000
C	5.17556900	2.60980700	-0.50754400
C	4.18495000	0.59737000	0.36749100
C	6.39974300	2.25458900	0.05630500
H	5.08744300	3.53522800	-1.07155500
C	5.40362500	0.24601400	0.94020700
H	3.32671800	-0.06871800	0.46203500
C	6.51440400	1.07406800	0.78707300
H	7.26106800	2.90257800	-0.07210700
H	5.48640200	-0.68366900	1.49413500
H	7.46511300	0.79824800	1.23216300
H	0.84028500	-0.81522200	-0.13378000
C	-0.40452200	-2.27255900	1.44115500
C	-1.04804600	-3.58969100	1.79632600
H	-2.09447500	-3.47537000	2.07356600
H	-0.99731800	-4.29336100	0.96431000
H	-0.49935500	-4.01471100	2.64456100
C	-0.71925900	-1.10374300	2.30909400
C	-1.95328800	-0.98829700	2.96998400
C	0.20763800	-0.06277000	2.45352700
C	-2.25111900	0.13872100	3.72467000
H	-2.70044500	-1.76818400	2.87874000
C	-0.09531900	1.07178700	3.20453900
H	1.18442700	-0.16977500	1.99271700
C	-1.32664000	1.17927400	3.84232200
H	-3.21326400	0.20966900	4.22263700
H	0.63540700	1.87137000	3.29387400
H	-1.56738800	2.06036300	4.42845500
N	0.81854900	-2.22585600	0.85519400
S	1.39999100	-3.54985400	0.09579200
O	1.82842800	-4.58930400	1.04173400

O	0.50488400	-3.99005300	-0.99198100
C	2.85500100	-2.86513500	-0.66009400
C	4.10540100	-3.15615800	-0.12880000
C	2.71621900	-2.05615800	-1.78639100
C	5.23497100	-2.60241300	-0.72541900
H	4.18178600	-3.80927200	0.73412800
C	3.85299000	-1.49991600	-2.35776800
H	1.73181900	-1.85651300	-2.19664900
C	5.12347000	-1.75324100	-1.82844900
H	6.21784300	-2.81500400	-0.31383100
H	3.75642400	-0.85305900	-3.22566200
C	6.33411500	-1.06516200	-2.39724000
H	6.41916900	-0.05990700	-1.96703300
H	7.25116400	-1.60847600	-2.15936600
H	6.26033100	-0.95944100	-3.48231300

TS5$_D$(RS)

0 1

C	-0.37134800	1.69760500	0.29890100
C	-1.82097200	3.22223000	-0.35087900
C	-2.87583500	1.14348000	0.56902400
H	-2.79254000	0.18278500	0.05800900
N	-0.67056600	3.79743200	-0.48558100
N	0.23057900	2.86298500	-0.04871400
N	-1.70881100	1.95569000	0.16910300
C	-3.15606100	3.75270400	-0.77311400
H	-3.03959400	4.33027200	-1.69078100
H	-3.58344800	4.39941800	0.00609200
C	-4.16110500	1.88397100	0.13329500
O	-4.00072000	2.65602100	-1.03689200
C	1.59988300	3.25434400	0.11519200
C	2.09051700	3.41459800	1.41767500
C	2.38716800	3.44596400	-1.02268400
C	3.44178700	3.71656600	1.56123600
C	3.73367400	3.75729500	-0.82318100
C	4.28024800	3.87514300	0.45480600
H	3.85127900	3.83155700	2.56184200
H	4.37057800	3.90174300	-1.69226900

H	-4.93820100	1.14315600	-0.06084300
C	0.24426900	0.45480900	0.64539900
O	-0.60445900	-0.57071100	1.03192700
C	1.60381200	0.26004700	0.57497500
C	2.23791200	-0.96503400	0.84127500
H	2.21273300	1.07755000	0.21272300
H	1.66016800	-1.68101000	1.41904800
C	3.69122900	-1.04072600	1.02260000
C	4.24060800	-2.12154800	1.73271700
C	4.58379300	-0.09271600	0.48523900
C	5.61472600	-2.24908500	1.90822200
H	3.56830900	-2.86935300	2.14694900
C	5.95669100	-0.22292200	0.65998500
H	4.20764700	0.75502600	-0.08388500
C	6.48292900	-1.29969400	1.37392500
H	6.00760300	-3.09515600	2.46434500
H	6.62252600	0.52016600	0.23083000
H	7.55525400	-1.39701900	1.50906800
C	5.74770200	4.15353300	0.64911800
H	6.26377800	3.25104800	0.99240900
H	6.21736000	4.47846400	-0.28132600
H	5.90467700	4.92663600	1.40569500
C	1.20206800	3.22652800	2.61773500
H	0.22633500	3.69850900	2.46832400
H	1.02983400	2.16167100	2.81004200
H	1.66427400	3.66082500	3.50540900
C	1.80716600	3.31043300	-2.40329300
H	1.09260600	4.11564200	-2.59940400
H	2.59823600	3.36164000	-3.15348500
H	1.27260300	2.36278700	-2.51159700
H	-4.50213800	2.53578800	0.95267300
C	-2.92674600	0.91987900	2.08871000
H	-1.97732000	0.50074300	2.42410300
H	-3.08104700	1.88293000	2.58825900
C	-4.05727400	-0.03465700	2.39931600
C	-5.25905500	0.40511400	2.95714000
C	-3.92394400	-1.38476800	2.05758900
C	-6.31087500	-0.48504200	3.16895900

H	-5.37203200	1.45147100	3.22984300
C	-4.97228500	-2.27544400	2.26743100
H	-2.98719200	-1.72819600	1.62377500
C	-6.17064900	-1.82642200	2.82273600
H	-7.23898800	-0.12892300	3.60465400
H	-4.85421300	-3.32187300	2.00251200
H	-6.98813300	-2.52091800	2.98730400
H	-0.46920700	-1.26463100	0.35106900
C	1.72854500	-2.05274100	-1.05551500
C	2.75132700	-1.47244800	-2.00121300
H	2.75403900	-2.05748300	-2.92769600
H	2.49152700	-0.44036700	-2.24589700
H	3.75216500	-1.48584500	-1.57023900
C	2.03492600	-3.37226900	-0.42605600
C	3.28906200	-3.97560600	-0.56911100
C	1.05643200	-4.04191400	0.32658300
C	3.55759300	-5.21005500	0.02223900
H	4.06965800	-3.49069100	-1.14388300
C	1.32954800	-5.26124500	0.92798500
H	0.07735000	-3.58764000	0.44205600
C	2.58549000	-5.85490300	0.77673600
H	4.53686700	-5.66021100	-0.10629000
H	0.56013000	-5.75727600	1.51122700
H	2.79693900	-6.81199100	1.24250800
N	0.40788700	-1.80830200	-1.22799500
S	-0.14431000	-0.94968000	-2.48478700
O	0.17987000	0.49073200	-2.46905800
O	0.14898000	-1.62535000	-3.76114800
C	-1.89355400	-1.06016000	-2.15596700
C	-2.70455400	-0.01879000	-2.59484800
C	-2.44107500	-2.17009200	-1.51116300
C	-4.07744700	-0.08221900	-2.36378100
H	-2.26092100	0.84481200	-3.08024300
C	-3.80688900	-2.20270600	-1.26310600
H	-1.79438600	-2.97901300	-1.18971300
C	-4.64236900	-1.15578700	-1.67225400
H	-4.70691700	0.74371700	-2.68144700
H	-4.23532400	-3.04809900	-0.73095500

C	-6.10562300	-1.17958000	-1.31778800
H	-6.64369000	-0.35477100	-1.78980600
H	-6.57183200	-2.11919900	-1.62684400
H	-6.23004500	-1.09799000	-0.23062400

TS5$_D$(SR)

0 1

C	1.16105600	1.91558100	0.01277300
C	2.11366300	3.25782300	1.48207100
C	3.29122200	1.07317900	1.22592000
H	2.90089500	0.16446900	1.68916000
N	1.23658100	3.99557500	0.88629000
N	0.65892300	3.17152500	-0.04256800
N	2.15825300	1.98797300	0.95843800
C	2.97557700	3.64528700	2.64772100
H	2.36063000	4.13716700	3.40280800
H	3.76498200	4.33801500	2.32475500
C	4.23251800	1.75969500	2.23225000
O	3.52575100	2.48018600	3.21638500
C	-0.31309600	3.76502000	-0.91819900
C	0.02758300	3.96269000	-2.26123800
C	-1.49747000	4.24300300	-0.35380200
C	-0.90867300	4.59928400	-3.07123900
C	-2.40012400	4.87493400	-1.20998300
C	-2.13223900	5.04694600	-2.56691200
H	-0.67100900	4.76336300	-4.11954300
H	-3.33627900	5.24514400	-0.79908500
H	4.82324700	0.99423000	2.73664100
C	0.70734700	0.76601500	-0.70965000
O	-0.06401300	1.01706600	-1.82376700
C	1.03344700	-0.52167500	-0.34467400
C	0.59882600	-1.66082800	-1.04613400
H	1.58748300	-0.66117500	0.57701100
H	0.24133800	-1.46830900	-2.05410900
C	1.19766200	-2.98206800	-0.84796300
C	1.17762300	-3.90805900	-1.90560800
C	1.77127000	-3.40046600	0.36827300
C	1.72249500	-5.17967000	-1.76640400

H	0.72267900	-3.61313900	-2.84835700
C	2.31872700	-4.67184200	0.50667200
H	1.79065900	-2.72450000	1.22007700
C	2.30167300	-5.56981400	-0.56035000
H	1.69417900	-5.86859800	-2.60522600
H	2.75870700	-4.96316200	1.45596600
H	2.72943500	-6.56085100	-0.44972200
C	-3.14357600	5.69922900	-3.47362200
H	-3.73901800	6.43927800	-2.93375700
H	-2.65760500	6.19392000	-4.31780500
H	-3.83432100	4.95320400	-3.87983300
C	1.36473600	3.53471600	-2.80486300
H	2.17496000	3.81002000	-2.12168200
H	1.39615000	2.45130300	-2.94773200
H	1.55325200	4.01462400	-3.76697600
C	-1.77071200	4.14555200	1.12271500
H	-1.28447200	4.97326200	1.64920800
H	-2.84472500	4.19423500	1.30727300
H	-1.39780100	3.20650800	1.53564100
H	4.91766500	2.43496600	1.69659800
C	4.05799000	0.75603200	-0.07095300
H	3.35493000	0.44707900	-0.84723000
H	4.53931200	1.67998900	-0.41159600
C	5.07705700	-0.33715700	0.14899300
C	6.39009400	-0.05461000	0.53329400
C	4.69109500	-1.67044500	-0.00498900
C	7.29429300	-1.08749800	0.77136700
H	6.71118300	0.97897500	0.63841200
C	5.59001600	-2.70511400	0.23548900
H	3.67902300	-1.89988300	-0.32911200
C	6.89533900	-2.41471000	0.62746300
H	8.31284700	-0.85431100	1.06492300
H	5.26575800	-3.73406500	0.11017800
H	7.60171800	-3.21748800	0.81251400
H	-0.92557400	0.58576100	-1.65848800
C	-1.54120400	-1.79701300	-0.31943900
C	-1.32807800	-2.42790700	1.03560900
H	-0.63577400	-3.26704100	0.97793600

H	-0.93417000	-1.69654900	1.74255400
H	-2.28691900	-2.80338300	1.41270200
C	-1.91253900	-2.71069400	-1.44312400
C	-1.84453200	-4.10222400	-1.30727600
C	-2.35239500	-2.18140100	-2.66753500
C	-2.20392500	-4.93976400	-2.36256400
H	-1.50744000	-4.54878900	-0.37853200
C	-2.69357300	-3.01621000	-3.72184600
H	-2.42745600	-1.10482900	-2.77622800
C	-2.62307400	-4.40336800	-3.57404000
H	-2.14575300	-6.01579300	-2.23218500
H	-3.02712600	-2.58622900	-4.66114300
H	-2.89708000	-5.05528600	-4.39725900
N	-2.00412000	-0.53598100	-0.46045500
S	-2.34592900	0.43594800	0.79236300
O	-1.23352100	0.67884600	1.73026300
O	-2.96887900	1.62293200	0.19845800
C	-3.60807200	-0.42007500	1.72448100
C	-4.68277500	-0.99569400	1.04641700
C	-3.52015600	-0.47934000	3.10860800
C	-5.67217000	-1.64148400	1.77436300
H	-4.72647800	-0.95224600	-0.03802100
C	-4.52429900	-1.12762400	3.82527700
H	-2.66728900	-0.03346400	3.60907200
C	-5.60880100	-1.71552400	3.17265600
H	-6.50843700	-2.10084500	1.25438200
H	-4.45967500	-1.18125200	4.90829800
C	-6.69494300	-2.42011100	3.94272400
H	-6.80669600	-3.45335100	3.60168900
H	-6.47767900	-2.43385800	5.01238500
H	-7.65886800	-1.92373200	3.79658000

TS5$_D$(SS)

0 1

C	-1.28571600	1.87432900	0.04638100
C	-1.44770800	4.03460800	0.41337300
C	0.89319300	3.12647900	0.57369400
H	1.20390700	2.58399500	1.46867300

N	-2.62468700	3.68191600	-0.00577600
N	-2.51155600	2.34051200	-0.25014100
N	-0.57512700	2.97475000	0.43323100
C	-1.00074600	5.39062600	0.87674400
H	-1.74111500	5.80832700	1.56027700
H	-0.88984500	6.06548500	0.01694400
C	1.18319400	4.62475900	0.75926200
O	0.21551400	5.25259800	1.57474100
C	-3.68320300	1.56281800	-0.53963500
C	-4.01669000	1.30437400	-1.86968900
C	-4.45072500	1.13170600	0.54381100
C	-5.17523900	0.56634200	-2.10280600
C	-5.60597300	0.40416300	0.25790200
C	-5.97598200	0.10588300	-1.05496700
H	-5.46056700	0.34729700	-3.12848700
H	-6.23436700	0.06859400	1.07977400
H	2.15353500	4.74002500	1.24325700
C	-0.81282400	0.52509300	-0.04794200
O	-1.24685500	-0.12178800	-1.17232800
C	0.11460300	0.08215900	0.87521700
C	0.98695100	-1.01988000	0.80711300
H	0.15891300	0.68600300	1.78309000
H	1.33091400	-1.31675700	-0.17698600
C	1.88122900	-1.31590900	1.91506500
C	3.15454500	-1.87292400	1.67172200
C	1.51728500	-1.08721400	3.25673500
C	4.02276500	-2.17036300	2.71407600
H	3.45619900	-2.05136000	0.64080900
C	2.39272600	-1.38245200	4.29796400
H	0.52770500	-0.69823500	3.48095300
C	3.64965500	-1.92464400	4.03734800
H	4.99915700	-2.59273800	2.49491800
H	2.08518500	-1.19640000	5.32289000
H	4.32783300	-2.15698700	4.85169600
C	-7.20590900	-0.71671200	-1.33782600
H	-7.67645400	-0.41305000	-2.27555100
H	-6.94393200	-1.77571500	-1.42803300
H	-7.93895800	-0.62305600	-0.53377000

C	-3.14691400	1.80061200	-2.99093800
H	-2.97257500	2.87741300	-2.90643100
H	-2.17667400	1.29686000	-2.95358100
H	-3.61106200	1.59872600	-3.95725300
C	-4.03449500	1.45328400	1.95470100
H	-3.94245900	2.53387500	2.10030500
H	-4.76323500	1.06796000	2.67008200
H	-3.06400600	0.99675000	2.18073500
H	1.22131100	5.11961300	-0.22344200
C	1.63286500	2.58865000	-0.66456900
H	1.27729700	1.58203700	-0.89455600
H	1.39438100	3.23250600	-1.51855500
C	3.12403100	2.54009400	-0.42528700
C	3.98524100	3.50387200	-0.95358600
C	3.65630700	1.51103300	0.35987000
C	5.35535100	3.44445700	-0.69678700
H	3.58382400	4.30205000	-1.57325900
C	5.02125500	1.45284300	0.62027800
H	2.99339000	0.74588500	0.75775700
C	5.87503500	2.42168000	0.09274800
H	6.01483100	4.19777300	-1.11559200
H	5.41478100	0.64353100	1.22831600
H	6.94099500	2.37586100	0.29092300
H	-1.18474700	-1.10835900	-1.04810100
C	-0.33070800	-2.99793200	0.38785400
C	0.77089900	-3.98250100	0.67513200
H	0.38000200	-4.99625700	0.54007500
H	1.60505300	-3.85385000	-0.01404800
H	1.15750000	-3.87492700	1.68525500
C	-1.29933900	-2.68892600	1.47164600
C	-1.03458100	-3.03271200	2.80836800
C	-2.53206500	-2.08032900	1.17522500
C	-1.96109000	-2.76855200	3.81097400
H	-0.10044000	-3.50451500	3.08438600
C	-3.46158200	-1.83873900	2.17987000
H	-2.77623000	-1.82362400	0.14983200
C	-3.18134400	-2.17210400	3.50324800
H	-1.72892900	-3.04101200	4.83556800

H	-4.41495300	-1.39039900	1.91954800
H	-3.90982400	-1.97766900	4.28382900
N	-0.75454600	-2.72973100	-0.85270100
S	-0.02004100	-3.26593300	-2.22172100
O	0.35982900	-4.67938300	-2.13539500
O	-0.90897600	-2.85265500	-3.30664000
C	1.48420900	-2.32306700	-2.40453000
C	2.71634200	-2.92465000	-2.16764800
C	1.39606600	-0.96684800	-2.72181800
C	3.87186700	-2.14858700	-2.21845100
H	2.76357600	-3.98709800	-1.95127400
C	2.55908600	-0.20796700	-2.76382000
H	0.42637100	-0.51218700	-2.89741400
C	3.81006200	-0.78136400	-2.50340700
H	4.83623300	-2.61199800	-2.02977800
H	2.50262100	0.85211500	-2.99844800
C	5.06170300	0.05156200	-2.56932600
H	5.80222400	-0.29259400	-1.84365500
H	5.51204900	-0.01669300	-3.56526100
H	4.84477300	1.10312700	-2.36393900

M5$_D$(RR)

0 1

C	-0.40384200	1.84581800	0.41949500
C	-1.68135500	3.37257000	-0.49905400
C	-2.94267700	1.43651700	0.48121200
H	-2.78427900	0.42868700	0.09323700
N	-0.47160600	3.81886000	-0.65565000
N	0.31204500	2.87390400	-0.06485700
N	-1.69673000	2.18451000	0.18258600
C	-2.94020400	3.91112700	-1.10421100
H	-2.69674700	4.43858700	-2.02675800
H	-3.45281700	4.59918500	-0.41857700
C	-4.12492900	2.13088000	-0.22975600
O	-3.75843400	2.80415200	-1.41691400
C	1.74071900	2.98606200	-0.16279000
C	2.47914700	3.26793900	0.98546800
C	2.32062400	2.70769300	-1.40548200

C	3.86919000	3.22672200	0.87190600
C	3.71064600	2.67458400	-1.46448900
C	4.49761200	2.91593900	-0.33390500
H	4.47217300	3.42202400	1.75478600
H	4.19220700	2.43922200	-2.41060800
H	-4.86061300	1.36908800	-0.49449900
C	0.03601500	0.52424500	0.97335400
O	-0.92122800	-0.23012800	1.40633300
C	1.34867700	0.16670100	0.87278900
C	1.79085500	-1.19313800	1.34905200
H	2.08993400	0.81628500	0.43792900
H	1.12190300	-1.48261200	2.17114100
C	3.20825600	-1.16007600	1.88804800
C	3.49743400	-1.75674700	3.11816400
C	4.24865200	-0.52403600	1.19751100
C	4.78491800	-1.72487600	3.65027600
H	2.69836700	-2.24876200	3.66752700
C	5.53501200	-0.48695800	1.72745000
H	4.05675800	-0.05964200	0.23247100
C	5.80854300	-1.08667300	2.95575200
H	4.98364000	-2.19313000	4.60925300
H	6.32793400	0.01132000	1.17761500
H	6.81205700	-1.05406100	3.36818200
C	5.99855500	2.83763900	-0.43068600
H	6.46331800	2.89255300	0.55568100
H	6.30512900	1.90151200	-0.90694900
H	6.39261800	3.65749100	-1.03906800
C	1.80497900	3.51954600	2.30611900
H	0.96330600	4.20950700	2.20221600
H	1.42205800	2.57816800	2.71590000
H	2.51259100	3.93867800	3.02261400
C	1.46986000	2.43527100	-2.61674600
H	0.87857800	3.31549900	-2.88300100
H	2.09405800	2.16893500	-3.47038500
H	0.76510100	1.61586300	-2.43291100
H	-4.59992700	2.84631900	0.45800200
C	-3.23016000	1.37248300	1.98745700
H	-2.32601000	1.03482400	2.49327400

H	-3.50162300	2.37104400	2.34806300
C	-4.35271400	0.38931600	2.22823700
C	-5.63599300	0.80540200	2.58563000
C	-4.10723800	-0.97602700	2.04065200
C	-6.66091300	-0.12502700	2.75434400
H	-5.83441000	1.86370300	2.73754000
C	-5.12954500	-1.90486900	2.20755200
H	-3.10293500	-1.29153500	1.76403100
C	-6.41025100	-1.48113100	2.56323900
H	-7.65389200	0.21111100	3.03576600
H	-4.92727600	-2.96236100	2.06474900
H	-7.20670500	-2.20650900	2.69476500
H	-0.41184200	-1.56272400	0.52251700
C	1.57038500	-2.32348200	0.25857100
C	1.74696600	-3.69775600	0.91070300
H	2.73005200	-3.79626700	1.37582700
H	0.98582100	-3.82869400	1.68417300
H	1.62948200	-4.49324700	0.16878900
C	2.51250000	-2.09760100	-0.92122900
C	3.71822400	-2.79153100	-1.03784500
C	2.22730500	-1.10073700	-1.86097400
C	4.62213700	-2.49018000	-2.05563100
H	3.97146000	-3.56891400	-0.32523600
C	3.12818600	-0.79537600	-2.87578500
H	1.28885500	-0.56396700	-1.77234900
C	4.33470700	-1.48734100	-2.97537600
H	5.55400300	-3.04278500	-2.12453100
H	2.88445700	-0.02288400	-3.59909900
H	5.03753200	-1.25268700	-3.76864800
N	0.16859300	-2.10613400	-0.15918200
S	-0.72086700	-3.21503800	-0.95412200
O	0.05682200	-3.70002000	-2.09628600
O	-1.32214400	-4.23334100	-0.08512900
C	-2.04478300	-2.15307400	-1.50041300
C	-1.76170300	-1.11098500	-2.38500200
C	-3.33382000	-2.36997900	-1.03428400
C	-2.79650600	-0.29364700	-2.81766400
H	-0.74380900	-0.96075300	-2.73296100

C	-4.36346500	-1.54066100	-1.47951500
H	-3.52234300	-3.17675300	-0.33378600
C	-4.11535300	-0.51069200	-2.38859800
H	-2.58995100	0.51862400	-3.50912100
H	-5.37309400	-1.70252600	-1.11180800
C	-5.23598300	0.33699600	-2.93190600
H	-4.96248000	1.39555300	-2.92789400
H	-5.45570900	0.05389900	-3.96624400
H	-6.15129700	0.20535600	-2.34982400

M5$_D$(RS)

0 1

C	0.76746500	2.03357700	-0.37227800
C	2.32180900	3.50354100	0.09623700
C	3.19907700	1.29375600	-0.72703100
H	3.07962800	0.45363500	-0.04334700
N	1.20252600	4.11082800	0.36029400
N	0.24476100	3.19093500	0.05450600
N	2.10671300	2.23812100	-0.38237600
C	3.71334300	3.98768200	0.37015800
H	3.70546300	4.62015300	1.25808700
H	4.09911800	4.56658000	-0.48007200
C	4.53970300	2.01102800	-0.50206200
O	4.52469400	2.86279800	0.62649500
C	-1.13541700	3.48857600	0.32310000
C	-1.88314500	4.11750100	-0.67214100
C	-1.66469600	3.07543800	1.54676900
C	-3.23818700	4.32039400	-0.41511900
C	-3.02545100	3.29592300	1.75325100
C	-3.82541900	3.90477600	0.78258300
H	-3.85035400	4.80378900	-1.17212100
H	-3.47160500	2.97670900	2.69201900
H	5.31061600	1.25632200	-0.33536000
C	0.10782500	0.70934000	-0.59210400
O	0.92644000	-0.29499000	-0.51826200
C	-1.24327500	0.63815500	-0.73977300
C	-1.91809700	-0.70463300	-0.88194600
H	-1.84544400	1.53255100	-0.78693000

H	-1.23116800	-1.36099300	-1.43570100
C	-3.21323200	-0.61944900	-1.66634700
C	-3.51511200	-1.61187600	-2.60398700
C	-4.13677700	0.41469600	-1.47605800
C	-4.70256800	-1.58083700	-3.32814300
H	-2.81090500	-2.42671100	-2.75295500
C	-5.32834100	0.44922000	-2.19802900
H	-3.93143100	1.20253500	-0.75404000
C	-5.61529300	-0.54770400	-3.12712900
H	-4.91377600	-2.36317800	-4.05045600
H	-6.03199200	1.25993600	-2.03440900
H	-6.54192400	-0.51880000	-3.69150000
C	-5.30446400	4.07684300	1.01082100
H	-5.69795400	4.92147800	0.44141200
H	-5.84474800	3.17897600	0.69197700
H	-5.52512400	4.23559300	2.06854600
C	-1.24995400	4.50002600	-1.98255700
H	-0.35688100	5.11188100	-1.82720400
H	-0.94543200	3.60441500	-2.53559400
H	-1.95234700	5.05928300	-2.60167300
C	-0.80855600	2.36382100	2.55733400
H	0.13210300	2.89653500	2.72865200
H	-1.33259500	2.26491400	3.50910400
H	-0.57135900	1.34953600	2.21176300
H	4.81039100	2.59211100	-1.39703000
C	3.10483400	0.77967700	-2.16822100
H	2.07177700	0.49895500	-2.37411900
H	3.40433600	1.56977000	-2.86563900
C	3.99196500	-0.43722100	-2.31249700
C	5.27562700	-0.34975700	-2.85573900
C	3.53432000	-1.67263600	-1.84002100
C	6.09130300	-1.47802000	-2.93161400
H	5.63803900	0.60600200	-3.22731400
C	4.34576900	-2.80062200	-1.92425700
H	2.53959200	-1.72591300	-1.40248700
C	5.62664500	-2.70664300	-2.46799700
H	7.08638000	-1.39624400	-3.35736400
H	3.97743800	-3.75642100	-1.56367600

H	6.25870100	-3.58676700	-2.53003700
H	0.04009900	-1.15209700	0.43757100
C	-2.11509800	-1.44646700	0.50976800
C	-2.98798900	-0.61489800	1.45161100
H	-3.13029300	-1.12574900	2.40720200
H	-2.50554300	0.34739400	1.63875300
H	-3.96914800	-0.42715500	1.01188200
C	-2.69911700	-2.83668200	0.23876400
C	-4.06650400	-3.10254400	0.33821200
C	-1.85673800	-3.85620600	-0.21772100
C	-4.57904500	-4.35135200	-0.00989500
H	-4.75088900	-2.33263800	0.67840800
C	-2.36556900	-5.10074200	-0.57321200
H	-0.78976800	-3.66398800	-0.28237300
C	-3.73246500	-5.35422600	-0.47097500
H	-5.64535700	-4.53559500	0.07751300
H	-1.69290400	-5.87684000	-0.92548400
H	-4.13162600	-6.32586900	-0.74460300
N	-0.73976400	-1.57229900	1.02280900
S	-0.32165600	-1.88117800	2.56234900
O	-0.71029600	-0.81989800	3.50274200
O	-0.71485400	-3.24207600	2.94484500
C	1.44020000	-1.78814800	2.33499000
C	2.08293900	-0.57610400	2.55569900
C	2.11764200	-2.88487000	1.81045700
C	3.43072500	-0.45872400	2.22748700
H	1.52831300	0.25852500	2.97345000
C	3.46099400	-2.74902300	1.48233600
H	1.59167800	-3.82229600	1.65672400
C	4.12826400	-1.53067700	1.66218800
H	3.93898500	0.49125100	2.37740700
H	4.00132500	-3.59401800	1.06483500
C	5.55325300	-1.36952400	1.20409400
H	6.02561400	-0.50177600	1.67081000
H	6.14836100	-2.25643700	1.43624400
H	5.58241700	-1.23658200	0.11432900

$M5_D$(SR)

0 1

C	2.27826400	1.72707300	0.00613600
C	4.10635800	2.64998700	0.76452800
C	4.27025000	0.16168400	0.37260800
H	3.82723000	-0.45465100	1.15957500
N	3.26492000	3.63334200	0.62686600
N	2.13752700	3.04088900	0.14284400
N	3.54814200	1.45699900	0.37539100
C	5.50284700	2.69480200	1.30858000
H	5.54229500	3.37712200	2.15826400
H	6.19829500	3.04779500	0.53480100
C	5.73637800	0.46273600	0.71124400
O	5.85469800	1.40579600	1.75697100
C	0.93492500	3.79724400	-0.09269500
C	0.77434400	4.39898000	-1.33883800
C	-0.01857600	3.85073600	0.92477800
C	-0.40522200	5.10865000	-1.55185600
C	-1.18191800	4.56763000	0.66027900
C	-1.39341000	5.19189900	-0.56981000
H	-0.56749600	5.58066500	-2.51747900
H	-1.95882300	4.59772500	1.41851000
H	6.21898700	-0.45826200	1.03887500
C	1.18882500	0.80257900	-0.45602100
O	0.58147600	1.14962400	-1.52730400
C	0.95887800	-0.26643700	0.35832100
C	-0.04996200	-1.30582700	-0.05282600
H	1.53386700	-0.38876800	1.27000300
H	-0.06712100	-1.29147100	-1.15043700
C	0.38552200	-2.69858500	0.36860000
C	0.37026300	-3.74369300	-0.56086700
C	0.87981200	-2.96515400	1.65181600
C	0.83778700	-5.01204000	-0.22656600
H	-0.00701200	-3.55448900	-1.56205900
C	1.34375100	-4.23345300	1.99341100
H	0.91957900	-2.17139200	2.39237400
C	1.32620600	-5.26219200	1.05391900
H	0.82489900	-5.80364900	-0.96906700
H	1.72592700	-4.41499700	2.99302500

H	1.69362600	-6.24928800	1.31673300
C	-2.70614500	5.87124600	-0.85077200
H	-3.05429300	6.43948600	0.01541200
H	-2.63476200	6.54653900	-1.70604200
H	-3.45970000	5.10940500	-1.07523700
C	1.80916700	4.21493800	-2.41340800
H	2.80230000	4.52466100	-2.07435600
H	1.85425500	3.15347700	-2.67976900
H	1.55100800	4.79099700	-3.30312000
C	0.19620200	3.13494300	2.23109800
H	1.12571700	3.45593100	2.71245700
H	-0.63787000	3.32140800	2.90747300
H	0.25605500	2.05368500	2.06387800
H	6.25430900	0.83006400	-0.18842900
C	4.15882000	-0.54703900	-0.98461400
H	3.10474600	-0.64319400	-1.25326800
H	4.64927700	0.07162700	-1.74377700
C	4.79095500	-1.91889500	-0.91798200
C	6.07441300	-2.15066800	-1.41786300
C	4.09281700	-2.97488500	-0.32248200
C	6.65405600	-3.41426800	-1.32304700
H	6.62041200	-1.33850500	-1.89160500
C	4.67134200	-4.23728600	-0.22686400
H	3.08610300	-2.81003100	0.05820400
C	5.95403300	-4.45955700	-0.72564900
H	7.65045900	-3.58151700	-1.71926800
H	4.11145900	-5.04586300	0.23260700
H	6.40377400	-5.44452700	-0.65374300
H	-1.04910800	0.76427900	-0.87423700
C	-1.52778500	-0.95692900	0.37026100
C	-1.62508600	-0.84547300	1.89108500
H	-1.34788300	-1.79541600	2.35230900
H	-0.94824500	-0.06332800	2.24132600
H	-2.63540100	-0.59299500	2.21728400
C	-2.47180800	-2.02125500	-0.20804200
C	-3.26017700	-2.84957200	0.58864400
C	-2.55124000	-2.16866600	-1.59950600
C	-4.10930800	-3.79764000	0.01366600

H	-3.23674000	-2.75580500	1.66889100
C	-3.38268800	-3.12051000	-2.17536800
H	-1.97438100	-1.50353100	-2.23706900
C	-4.17082800	-3.94129900	-1.36729500
H	-4.72374700	-4.42289500	0.65466800
H	-3.42698200	-3.21490300	-3.25606400
H	-4.82933600	-4.68011100	-1.81327100
N	-1.80842500	0.33510000	-0.30873000
S	-2.93309600	1.42338600	0.16319500
O	-2.73492200	1.92645700	1.52605200
O	-3.00121500	2.40461500	-0.92128600
C	-4.42764900	0.46217500	0.19446000
C	-4.89339700	-0.08813300	-0.99880600
C	-5.07342700	0.22154200	1.39951900
C	-6.00056100	-0.92040000	-0.96826700
H	-4.37155900	0.11350500	-1.92895100
C	-6.18787500	-0.61531300	1.41322500
H	-4.69630900	0.67629700	2.30943000
C	-6.65217300	-1.20852300	0.23889300
H	-6.35656100	-1.37525500	-1.88837500
H	-6.69401500	-0.81896900	2.35229600
C	-7.81124100	-2.16840000	0.25456600
H	-7.46275100	-3.17991900	0.02024800
H	-8.29701500	-2.19345500	1.23177800
H	-8.55781800	-1.89840600	-0.49714600

M5$_D$(SS)

0 1

C	-1.20716100	1.94144200	0.01282800
C	-1.30572600	4.06827900	0.51416500
C	1.00641400	3.07471800	0.62209700
H	1.21949100	2.46969600	1.50659100
N	-2.52418000	3.73918600	0.20109400
N	-2.44128900	2.41774000	-0.12286500
N	-0.45463500	2.99949100	0.38863800
C	-0.80224500	5.38876600	1.01559200
H	-1.53478500	5.82185200	1.69738900
H	-0.64304600	6.07892000	0.17564200

C	1.35453200	4.54427200	0.90233400
O	0.39459800	5.17092500	1.72763100
C	-3.64069800	1.69643300	-0.46045600
C	-4.02471000	1.63530100	-1.79851900
C	-4.38524500	1.15375300	0.58737400
C	-5.22912700	0.99170700	-2.08144300
C	-5.58919600	0.53775400	0.25319900
C	-6.02331200	0.44665700	-1.07215500
H	-5.55286600	0.91683800	-3.11609400
H	-6.20368800	0.11779300	1.04655700
H	2.31429700	4.58409200	1.41888800
C	-0.78769800	0.52179700	-0.25467300
O	-1.19915300	0.06462900	-1.38568100
C	-0.05574100	-0.04559100	0.74510500
C	0.68714800	-1.36653500	0.69673500
H	0.09951600	0.55300400	1.63811300
H	1.42799300	-1.30075700	-0.11281600
C	1.48748700	-1.55958300	1.97265400
C	2.83972500	-1.91181700	1.89419200
C	0.91517100	-1.39429300	3.23800900
C	3.60025500	-2.10204000	3.04499000
H	3.29828100	-2.03131100	0.91298800
C	1.67113200	-1.59013100	4.39198300
H	-0.13181300	-1.11288900	3.31672300
C	3.01517400	-1.94448000	4.29970800
H	4.64846800	-2.37274600	2.96137600
H	1.20926800	-1.46084200	5.36588800
H	3.60361100	-2.09187100	5.19970500
C	-7.33445600	-0.22213700	-1.39348100
H	-7.44735000	-0.37667200	-2.46811300
H	-7.40960100	-1.19276400	-0.89622900
H	-8.17450000	0.38879600	-1.04862400
C	-3.15328500	2.22059600	-2.87339800
H	-2.97137700	3.28549900	-2.69650800
H	-2.18982600	1.70165100	-2.86990900
H	-3.61855500	2.10677400	-3.85333400
C	-3.88220100	1.22403800	2.00456700
H	-3.72922000	2.26136600	2.31891600

H	-4.59033100	0.75384200	2.68890500
H	-2.92496900	0.69642400	2.09201900
H	1.44457700	5.09041000	-0.04919600
C	1.80229200	2.55179600	-0.58191400
H	1.39914000	1.58268000	-0.88785600
H	1.68371600	3.24967700	-1.41835600
C	3.25647400	2.38748700	-0.20579500
C	4.22483600	3.33232600	-0.54819500
C	3.63330300	1.27080500	0.54739300
C	5.54726200	3.16872100	-0.13359700
H	3.94525800	4.20024300	-1.14055700
C	4.95007900	1.10439400	0.96035400
H	2.88053500	0.53026600	0.81213200
C	5.91101500	2.05738100	0.62276300
H	6.29201500	3.91040600	-0.40439100
H	5.22259000	0.23022900	1.54468100
H	6.94004600	1.93115300	0.94356000
H	-0.98478100	-1.55103600	-1.28919200
C	-0.16210800	-2.65556300	0.31916400
C	0.71135000	-3.90391700	0.46414500
H	0.16666700	-4.78833600	0.13102800
H	1.62021600	-3.81177100	-0.13441600
H	1.02711500	-4.03564700	1.49742500
C	-1.41516900	-2.75537100	1.19814500
C	-1.41689400	-3.49734400	2.38448100
C	-2.59063800	-2.07770700	0.84683600
C	-2.55212100	-3.57299300	3.18965000
H	-0.52757000	-4.02852800	2.70197800
C	-3.72945700	-2.16862900	1.64196700
H	-2.62964100	-1.47844000	-0.05998000
C	-3.71817000	-2.91400000	2.81825900
H	-2.52138200	-4.16017500	4.10217600
H	-4.63267500	-1.65328600	1.33188100
H	-4.60943400	-2.98114500	3.43427300
N	-0.63482800	-2.52541600	-1.08623900
S	0.19262100	-2.98541900	-2.42861300
O	0.49810100	-4.41286400	-2.38437500
O	-0.60231000	-2.46585600	-3.53931300

C	1.74971600	-2.11715100	-2.46066300
C	2.93665400	-2.80133900	-2.22577600
C	1.73560300	-0.73527400	-2.65628600
C	4.13074600	-2.08591700	-2.16762800
H	2.91727100	-3.87810900	-2.09136800
C	2.93805300	-0.04210900	-2.60637400
H	0.78955900	-0.21947000	-2.80422900
C	4.14788400	-0.70128400	-2.35346400
H	5.06257700	-2.61152700	-1.97837700
H	2.94779500	1.03445500	-2.75737900
C	5.43947300	0.06921400	-2.31350800
H	6.13433400	-0.36153900	-1.58860500
H	5.92568300	0.05102300	-3.29474700
H	5.26158300	1.11220200	-2.03941800

TS6$_D$(RR)

0 1

C	2.04971600	-1.34902200	0.36793400
C	3.33584900	-3.10991800	0.44677700
C	4.48519400	-1.03319500	-0.38360900
H	4.18304200	-0.63240800	-1.35142000
N	2.18061000	-3.51389300	0.89605900
N	1.39649200	-2.40791300	0.85113500
N	3.30829600	-1.78012700	0.13170000
C	4.55205600	-3.93507600	0.15696700
H	4.24124100	-4.93064800	-0.16103900
H	5.18300100	-4.02280400	1.05190300
C	5.65949000	-2.02267100	-0.52828100
O	5.24761800	-3.32041700	-0.90208000
C	0.01564900	-2.51345300	1.25537800
C	-0.29242100	-2.26690600	2.59606900
C	-0.92875600	-2.84506800	0.28669000
C	-1.63583600	-2.32047600	2.95341200
C	-2.26096700	-2.89300200	0.69977700
C	-2.63039600	-2.62095400	2.01705800
H	-1.91293300	-2.11941600	3.98520200
H	-3.02486900	-3.15047800	-0.02980700
H	6.32953300	-1.65194800	-1.30447400

C	1.52647600	0.04257700	0.00099400
O	2.25576100	0.67703600	-0.75712600
C	0.25107700	0.42337200	0.52724000
C	-0.15217400	1.89847800	0.50835500
H	-0.00736800	-0.09019400	1.44776000
H	0.72701700	2.54919200	0.43918000
C	-0.90517800	2.21378700	1.78514800
C	-0.53977100	3.30291800	2.57745600
C	-1.97788400	1.41354900	2.20259300
C	-1.22214500	3.59250200	3.75792500
H	0.28772400	3.93416900	2.26292300
C	-2.66066100	1.70060400	3.38035200
H	-2.28079100	0.56692700	1.58986500
C	-2.28527600	2.79128500	4.16407100
H	-0.92137500	4.44489600	4.35931700
H	-3.49044800	1.07065400	3.68775900
H	-2.81813300	3.01416800	5.08300800
C	-4.08006200	-2.62410700	2.42324700
H	-4.49047600	-1.60952200	2.36668000
H	-4.67449200	-3.25543500	1.75857400
H	-4.20351200	-2.97641800	3.45002800
C	0.78306000	-1.91167600	3.58755000
H	1.58700600	-2.65352200	3.58497900
H	1.22926200	-0.93820500	3.35180500
H	0.36906300	-1.85333400	4.59472300
C	-0.53867800	-3.10781800	-1.14209000
H	0.41428700	-3.63971200	-1.20697400
H	-1.30479800	-3.71238900	-1.63145400
H	-0.45663900	-2.16943300	-1.70596900
H	6.21807800	-2.07236100	0.41872700
C	4.88308500	0.10751400	0.56287000
H	4.00766600	0.71565100	0.79211800
H	5.25645900	-0.32107500	1.49884000
C	5.93814500	0.96745100	-0.09428200
C	7.28007900	0.89527500	0.28153000
C	5.56498200	1.82961900	-1.13055100
C	8.23731500	1.67378500	-0.36700400
H	7.57763200	0.23265200	1.09042100

C	6.51961300	2.60657800	-1.77856800
H	4.51703900	1.88796200	-1.41714700
C	7.85900500	2.52894100	-1.39821000
H	9.27772900	1.61235400	-0.06425200
H	6.21895600	3.27426100	-2.57958100
H	8.60420700	3.13499500	-1.90316400
H	-0.71662700	0.12230000	-0.28264200
C	-1.02859100	2.13282000	-0.80890300
C	-0.07886300	2.38439400	-1.99555100
H	0.57027000	3.24082600	-1.79664300
H	0.55010500	1.51120300	-2.17166200
H	-0.66211100	2.59242600	-2.89605000
C	-1.93225100	3.35073100	-0.62605100
C	-1.35530200	4.60957200	-0.41534400
C	-3.32271500	3.26083300	-0.65402800
C	-2.14310100	5.74132400	-0.23504300
H	-0.27312000	4.70872900	-0.37770600
C	-4.11688300	4.39292000	-0.47670100
H	-3.78053100	2.29585800	-0.83319400
C	-3.53268600	5.63780700	-0.26605900
H	-1.67135700	6.70539600	-0.07046900
H	-5.19825100	4.29831900	-0.50774800
H	-4.15108700	6.51948200	-0.12925300
N	-1.76851000	0.86796000	-0.91954100
S	-2.20795700	0.31685500	-2.35493900
O	-2.90462400	1.33172200	-3.17033100
O	-1.13838600	-0.41077800	-3.07565800
C	-3.41245500	-0.92400900	-1.89873800
C	-4.34002000	-0.69291300	-0.88346800
C	-3.46845600	-2.08769000	-2.65720100
C	-5.32994100	-1.63756400	-0.63973300
H	-4.26605000	0.20660200	-0.28043200
C	-4.45986900	-3.03131300	-2.39521100
H	-2.72852800	-2.25065900	-3.43404800
C	-5.40665100	-2.81813100	-1.39023500
H	-6.05448800	-1.46343100	0.15166300
H	-4.49832400	-3.94521800	-2.98148400
C	-6.49825500	-3.82107200	-1.12203600

H	-6.64085700	-3.97185200	-0.04836200
H	-7.45130600	-3.47043000	-1.53068300
H	-6.27200600	-4.78612200	-1.57970600

TS6$_D$(RS)

0 1

C	-1.86134800	-1.53696000	0.78808700
C	-3.39317900	-2.25541300	2.16134300
C	-4.06370900	-0.21828800	0.83693400
H	-3.57481200	0.71139200	1.13054700
N	-2.38912700	-3.07437300	2.31445900
N	-1.45070500	-2.61902500	1.44663100
N	-3.12080200	-1.30519400	1.21584500
C	-4.67173700	-2.20793200	2.94317800
H	-4.47382400	-2.50936200	3.97232700
H	-5.41460600	-2.88834900	2.50522700
C	-5.36556500	-0.43399800	1.62549700
O	-5.13529700	-0.87771900	2.94691600
C	-0.16990300	-3.27263800	1.34401700
C	-0.09184000	-4.41516400	0.54685400
C	0.92576900	-2.68253400	1.97637300
C	1.17272200	-4.97347100	0.37004500
C	2.16670700	-3.28117400	1.76736400
C	2.30710000	-4.41342000	0.96118100
H	1.27288600	-5.85607700	-0.25624900
H	3.04258300	-2.81772000	2.20952700
H	-5.89538700	0.51799500	1.68266100
C	-1.08487200	-0.60482100	-0.14055200
O	-1.54795500	0.52969900	-0.19806400
C	0.10351700	-1.09196500	-0.77087800
C	0.64884400	-0.29004400	-1.95334500
H	0.15861800	-2.17270600	-0.87453300
H	-0.16194300	0.16169100	-2.53424000
C	1.44068800	-1.21566000	-2.85047800
C	1.28043300	-1.16543300	-4.23634700
C	2.36547000	-2.12329500	-2.31549900
C	2.01921400	-1.99874500	-5.07421100
H	0.56666300	-0.46391600	-4.66233300

C	3.10671200	-2.95413200	-3.15006500
H	2.50669400	-2.17264900	-1.23776200
C	2.93579500	-2.89623600	-4.53282300
H	1.87645100	-1.94715400	-6.14926000
H	3.82111900	-3.64938400	-2.71928300
H	3.51351100	-3.54636600	-5.18220000
C	3.67206600	-5.00868200	0.73383100
H	3.64003500	-5.80155300	-0.01610400
H	4.37355600	-4.24007800	0.39723900
H	4.07109200	-5.43097100	1.66091600
C	-1.31427800	-4.98167200	-0.12485600
H	-2.07403100	-5.26589100	0.60892800
H	-1.76708000	-4.24759700	-0.80092100
H	-1.05278200	-5.86334900	-0.71095400
C	0.77838100	-1.43146600	2.80086100
H	-0.13336100	-1.45608000	3.40475000
H	1.64022600	-1.31316200	3.45752300
H	0.75091800	-0.54416900	2.15546700
H	-6.00348900	-1.15869100	1.09752900
C	-4.35791100	-0.20250200	-0.67219500
H	-3.43675000	-0.36905100	-1.23024900
H	-5.04857700	-1.01826500	-0.91087200
C	-4.94294000	1.13369700	-1.07250100
C	-6.31894200	1.30528600	-1.23104200
C	-4.09161600	2.22888600	-1.25941300
C	-6.84146000	2.55197600	-1.57154700
H	-6.98585500	0.45705300	-1.09478700
C	-4.61389100	3.47235900	-1.60019400
H	-3.01770600	2.09865500	-1.14235800
C	-5.98987700	3.63780000	-1.75621100
H	-7.91304700	2.67245000	-1.69543000
H	-3.94012900	4.31171500	-1.74619100
H	-6.39589900	4.60817900	-2.02368200
H	1.08401900	-0.68784800	-0.01658300
C	1.55670100	0.90130700	-1.36978800
C	2.65442900	1.28290400	-2.37615200
H	3.19232700	2.16779700	-2.02592700
H	3.36697600	0.47210700	-2.51593100

H	2.19695100	1.52260000	-3.34048300
C	0.67942900	2.14977500	-1.16123800
C	0.01205900	2.72610900	-2.24851400
C	0.53801400	2.75235100	0.08780300
C	-0.78121400	3.85864600	-2.09041000
H	0.10168500	2.28313500	-3.23770100
C	-0.24551900	3.89277300	0.25120300
H	1.03126300	2.30413900	0.94116900
C	-0.91385200	4.45069400	-0.83489300
H	-1.29350300	4.28176900	-2.94999900
H	-0.33641300	4.34254600	1.23599200
H	-1.52678900	5.33838000	-0.70868700
N	2.02012400	0.35195200	-0.09818100
S	3.47892500	0.41953500	0.52808200
O	3.50517900	-0.53969600	1.64475100
O	4.58524200	0.32045200	-0.43862100
C	3.64635900	2.03619700	1.27645300
C	3.08135300	2.26180100	2.53073300
C	4.25445200	3.07205600	0.57803400
C	3.11152500	3.53966700	3.07335400
H	2.62630900	1.43391500	3.06700900
C	4.27751400	4.34958900	1.13359000
H	4.71955100	2.87132100	-0.38174100
C	3.69934500	4.60433600	2.37838000
H	2.67563100	3.71854900	4.05284400
H	4.75617300	5.16091000	0.59218100
C	3.69193900	5.99293500	2.96274300
H	3.89619500	5.97011800	4.03618200
H	4.43782000	6.62971000	2.48251400
H	2.71282200	6.46331800	2.82526600

TS6$_D$(SR)

0 1

C	0.87859500	-2.13563900	-0.74529700
C	1.80310800	-2.87831800	-2.56984300
C	3.25610200	-1.31140700	-1.22601200
H	3.02356800	-0.27799400	-1.50440500
N	0.60494400	-3.38835000	-2.56471900

N	0.04703900	-2.91865200	-1.41579400
N	2.01694600	-2.09739800	-1.45815400
C	2.88159000	-3.05370100	-3.59705400
H	2.44243600	-3.04209500	-4.59493000
H	3.38418400	-4.01774500	-3.43892700
C	4.31037200	-1.86232800	-2.19053400
O	3.79104400	-1.98280900	-3.49970200
C	-1.33893800	-3.14706700	-1.08988700
C	-1.66209200	-4.25275300	-0.30540800
C	-2.27246600	-2.21871900	-1.56035600
C	-3.00913200	-4.42630100	0.00828400
C	-3.60202200	-2.42986900	-1.20374700
C	-3.98412100	-3.52509400	-0.42462700
H	-3.29914400	-5.27521000	0.62191000
H	-4.34514000	-1.70328600	-1.51816500
H	5.14714200	-1.16515000	-2.23220800
C	0.52980300	-1.45945900	0.58155900
O	0.30109400	-2.20360300	1.52159700
C	0.46026200	-0.03134300	0.48413200
C	0.39708200	0.79155500	1.77082000
H	1.12160600	0.36431300	-0.29089600
H	0.67195600	0.17137100	2.63036700
C	1.35512100	1.95850900	1.69429100
C	2.27612600	2.18336500	2.71944800
C	1.36001400	2.82136100	0.58858400
C	3.19043200	3.23355300	2.64415900
H	2.28387600	1.52047400	3.58101900
C	2.26803000	3.87335300	0.51281000
H	0.63424300	2.66657300	-0.20620700
C	3.19015000	4.08112800	1.53977600
H	3.90663300	3.38379500	3.44592200
H	2.25637000	4.53347700	-0.34930800
H	3.90135300	4.89893800	1.47807900
C	-5.43026500	-3.70721500	-0.04312500
H	-6.05473800	-3.83662300	-0.93168600
H	-5.56509700	-4.57864200	0.60054300
H	-5.79908400	-2.82510800	0.48867800
C	-0.59287800	-5.18439500	0.19549900

H	0.00576100	-5.58141800	-0.62987400
H	0.07715200	-4.64635800	0.87356500
H	-1.03585200	-6.02251800	0.73475900
C	-1.85700900	-1.05134700	-2.41565700
H	-1.33879500	-1.39294500	-3.31737200
H	-2.72995800	-0.46366400	-2.69678600
H	-1.18196100	-0.38118800	-1.87177200
H	4.67662400	-2.83546300	-1.82803200
C	3.72587400	-1.37716800	0.23126300
H	2.92710200	-1.03638600	0.89633700
H	3.95395700	-2.41706400	0.48805500
C	4.93725600	-0.49192500	0.42614100
C	6.22017400	-1.03643300	0.51273100
C	4.77968000	0.89615800	0.49248100
C	7.33084900	-0.20856700	0.66147800
H	6.34923000	-2.11491000	0.46987000
C	5.88922800	1.72326700	0.64180200
H	3.78401300	1.33523300	0.44355100
C	7.16712900	1.17310700	0.72453500
H	8.32227200	-0.64405900	0.73141800
H	5.74792000	2.79774500	0.70243700
H	8.03133700	1.81841000	0.84343200
H	-0.69381000	0.38290000	0.08914900
C	-1.13528000	1.21938000	1.97016200
C	-1.23616000	2.51702700	2.77982700
H	-0.62367900	2.47011500	3.68498300
H	-0.86710200	3.35189100	2.18010100
H	-2.27399200	2.70829900	3.05334500
C	-1.82361300	0.06070600	2.70955800
C	-1.65352000	-0.08711600	4.09288500
C	-2.56924000	-0.90741100	2.03202900
C	-2.22232400	-1.15409600	4.78011500
H	-1.06463800	0.63705600	4.64779900
C	-3.14404300	-1.97556500	2.72101600
H	-2.71523600	-0.82719700	0.95903500
C	-2.97637200	-2.10498900	4.09534800
H	-2.07815200	-1.24057400	5.85288000
H	-3.72265700	-2.71152800	2.17104000

H	-3.42614100	-2.93633300	4.62912000
N	-1.57654400	1.37488200	0.58697200
S	-3.04927200	1.74656300	0.12105100
O	-3.73856600	0.64340600	-0.58024600
O	-3.83409800	2.39621800	1.18951100
C	-2.74846400	2.96607400	-1.15693200
C	-3.41502800	2.86974900	-2.37126500
C	-1.88720100	4.03373800	-0.90382700
C	-3.21120000	3.84838400	-3.34397500
H	-4.07848200	2.03010600	-2.54742100
C	-1.69431100	5.00222800	-1.87876000
H	-1.36287600	4.08699900	0.04580300
C	-2.35335600	4.92395800	-3.11369300
H	-3.72727400	3.77224700	-4.29692700
H	-1.02061200	5.83317800	-1.68605700
C	-2.13007800	5.98420700	-4.16071200
H	-2.43603300	6.96704500	-3.79035500
H	-1.07152800	6.05298700	-4.42797500
H	-2.69806200	5.77075900	-5.06820900

TS6$_D$(SS)

0 1

C	-0.72385200	-2.42663100	-0.24135900
C	-1.43359300	-3.92485600	1.16844900
C	-2.94325100	-1.91416900	0.93520700
H	-2.61566000	-1.08328900	1.56802300
N	-0.31421300	-4.40009300	0.70163000
N	0.10733400	-3.45758200	-0.18206800
N	-1.72998300	-2.70640100	0.60608700
C	-2.34243900	-4.53529300	2.19268000
H	-1.74673400	-4.98014200	2.99056300
H	-2.95439000	-5.31856800	1.72469400
C	-3.86422900	-2.83164900	1.74834400
O	-3.14725400	-3.52327700	2.75009500
C	1.36920900	-3.57820300	-0.87006400
C	1.39070500	-4.25483600	-2.08790400
C	2.48741000	-2.98395800	-0.28259200
C	2.62135700	-4.34154200	-2.73537300

C	3.68873700	-3.08591000	-0.97914000
C	3.77271800	-3.76184300	-2.19847900
H	2.67741400	-4.86099400	-3.68838000
H	4.57236500	-2.60905300	-0.56443500
H	-4.61892000	-2.22088700	2.24347400
C	-0.49929200	-1.21708400	-1.14342100
O	-0.32297700	-1.46465600	-2.32777400
C	-0.44999200	0.04190800	-0.45754100
C	-0.57254400	1.31282800	-1.29837300
H	-1.01706000	0.04363800	0.47556100
H	-0.92212500	1.05878900	-2.30546700
C	-1.56575800	2.26390900	-0.67064300
C	-2.65961700	2.72786600	-1.40352400
C	-1.42279700	2.68353500	0.65833200
C	-3.59351100	3.58868100	-0.82959300
H	-2.77979100	2.41017600	-2.43623800
C	-2.35429200	3.54259100	1.23500000
H	-0.56356100	2.33988600	1.22964600
C	-3.44326200	3.99911400	0.49193100
H	-4.44142000	3.93218100	-1.41412300
H	-2.22781500	3.86265400	2.26479600
H	-4.16789400	4.67146000	0.94099300
C	5.09345600	-3.85865600	-2.91675900
H	5.53615200	-2.86770300	-3.04726000
H	5.80317700	-4.46085400	-2.34147500
H	4.97759800	-4.31685400	-3.90070500
C	0.12895900	-4.82283400	-2.67647300
H	-0.36549000	-5.50469600	-1.97797900
H	-0.56666400	-4.00914500	-2.90663800
H	0.34438900	-5.36708000	-3.59683300
C	2.38802000	-2.27747000	1.04183200
H	1.96136800	-2.93597100	1.80554500
H	3.37210000	-1.94267600	1.36794700
H	1.75591300	-1.38536000	0.96970300
H	-4.36986200	-3.54249000	1.07658300
C	-3.64277200	-1.39189200	-0.32593100
H	-2.96454800	-0.74059000	-0.88629600
H	-3.89708500	-2.24366600	-0.96593100

C	-4.88294100	-0.61168300	0.04836300
C	-6.15495100	-1.16867100	-0.10092200
C	-4.75958800	0.67497400	0.58112100
C	-7.28836100	-0.45327700	0.27847100
H	-6.25907800	-2.16449400	-0.52442400
C	-5.89220300	1.38993700	0.95993700
H	-3.77534400	1.12975400	0.68397800
C	-7.15844700	0.82703200	0.81178900
H	-8.27169700	-0.89493000	0.15312600
H	-5.77963600	2.39178400	1.36234600
H	-8.04086800	1.38668200	1.10458800
H	0.72798300	0.36003200	-0.05874000
C	0.90913800	1.90502500	-1.45488000
C	1.57648100	1.19372000	-2.64686200
H	2.59504500	1.56795500	-2.78018100
H	1.61941600	0.11726000	-2.46369800
H	1.02021800	1.36289900	-3.57190100
C	0.83614000	3.40899400	-1.70476700
C	0.24761700	3.88750700	-2.88150200
C	1.29590100	4.33782100	-0.77177800
C	0.12935100	5.25277100	-3.12205200
H	-0.14120400	3.18756700	-3.61656500
C	1.18207600	5.70621600	-1.01003100
H	1.75660800	3.97655100	0.13972300
C	0.59999600	6.17100200	-2.18551400
H	-0.33186100	5.59968600	-4.04187800
H	1.55376800	6.41115200	-0.27232100
H	0.51275000	7.23691400	-2.37153900
N	1.53480600	1.54899700	-0.17778300
S	3.12227400	1.36389400	-0.07593100
O	3.62573200	0.06763500	-0.57908000
O	3.86201000	2.53759600	-0.58357300
C	3.33325300	1.29721000	1.69888600
C	4.37838600	0.52500600	2.20028500
C	2.53585300	2.04829500	2.55728400
C	4.61700800	0.50095700	3.57004400
H	4.98330900	-0.06141300	1.51582200
C	2.78295400	2.01146400	3.92637700

H	1.72075100	2.63781900	2.15109000
C	3.82553300	1.24309700	4.45213500
H	5.42698000	-0.10766300	3.96300600
H	2.15425600	2.58866900	4.59889800
C	4.10776300	1.23089300	5.93198100
H	4.46239600	0.25047800	6.25874700
H	4.88480300	1.96103700	6.18096800
H	3.21589100	1.48697700	6.50784600

M6$_D$(RR)

0 1

C	0.56606200	1.46198500	-0.78019300
C	1.90038300	3.15774500	-0.48757200
C	3.09388300	0.97294300	-0.81680000
H	2.94727100	0.16393400	-0.09723700
N	0.69703900	3.66281200	-0.45008300
N	-0.11755300	2.60281000	-0.64603400
N	1.87032000	1.81340900	-0.72086600
C	3.19541700	3.84692200	-0.19557100
H	3.01472000	4.67242600	0.49324700
H	3.64755300	4.23636200	-1.11775800
C	4.32448400	1.83711900	-0.43915800
O	4.03387800	2.90763300	0.43356700
C	-1.54649900	2.79479100	-0.67481900
C	-2.19343300	2.77435400	-1.90876700
C	-2.20349300	2.92311900	0.55211100
C	-3.58862500	2.80438300	-1.88493400
C	-3.59377200	2.94779000	0.51904100
C	-4.29954600	2.85922700	-0.68497200
H	-4.12976700	2.76683500	-2.82666200
H	-4.14057800	3.00647400	1.45619700
H	5.04991400	1.19497700	0.06264900
C	0.03835500	0.03610800	-0.96163000
O	0.86436000	-0.79333700	-1.25285900
C	-1.44201700	-0.20251400	-0.90114300
C	-1.81036300	-1.66292600	-0.62721100
H	-1.82975700	0.11796400	-1.87810300
H	-0.97611200	-2.29361000	-0.95490700

C	-3.04411000	-2.07513700	-1.40419000
C	-3.18330200	-3.39695500	-1.83928400
C	-4.07349200	-1.17279200	-1.70160000
C	-4.32058600	-3.81319000	-2.52765600
H	-2.38639100	-4.10746500	-1.63424900
C	-5.21052400	-1.58233600	-2.39207700
H	-4.00071500	-0.14025700	-1.36714800
C	-5.34087200	-2.90646400	-2.80515000
H	-4.40486500	-4.84497300	-2.85445400
H	-5.99759700	-0.86455600	-2.60361000
H	-6.22657800	-3.22684100	-3.34452900
C	-5.80440400	2.81847200	-0.66681800
H	-6.21187800	2.66755400	-1.66805700
H	-6.14853700	2.00218600	-0.02430000
H	-6.21524800	3.74905100	-0.26461000
C	-1.42727300	2.68627500	-3.20289900
H	-0.56851400	3.36324400	-3.20380600
H	-1.05046900	1.67331400	-3.38565100
H	-2.07267600	2.94976800	-4.04156600
C	-1.42744500	2.99705400	1.83771500
H	-0.82842300	3.91137400	1.87841600
H	-2.10244800	2.98259700	2.69327300
H	-0.74262400	2.14731800	1.92940800
H	4.78251200	2.23504500	-1.35614200
C	3.30705800	0.40092900	-2.22697500
H	2.36850800	0.01577000	-2.62082300
H	3.65465200	1.19819200	-2.89218800
C	4.32688300	-0.71414500	-2.14758900
C	5.60810100	-0.56437100	-2.67956500
C	3.99196100	-1.90686500	-1.49377900
C	6.54620600	-1.58965700	-2.56667900
H	5.87266500	0.35830000	-3.19036100
C	4.93041300	-2.92791000	-1.37992400
H	2.99594200	-2.03604600	-1.07063900
C	6.20941400	-2.77237700	-1.91484700
H	7.53828800	-1.46169300	-2.98797000
H	4.65972200	-3.84939300	-0.87331900
H	6.93880400	-3.57083400	-1.82395000

H	-1.89990900	0.45175000	-0.15507300
C	-1.85109200	-1.88308200	0.94483300
C	-2.06901200	-3.37904400	1.24183400
H	-3.04235300	-3.71946800	0.88324800
H	-1.29264500	-3.97685900	0.75633900
H	-2.00906100	-3.54718800	2.31896300
C	-3.00640600	-1.09776600	1.59367500
C	-4.34787900	-1.42683200	1.34547600
C	-2.75259900	-0.05873400	2.49333100
C	-5.38830100	-0.71397400	1.93481800
H	-4.59556800	-2.25225900	0.68732800
C	-3.79173300	0.64330200	3.10346400
H	-1.71868300	0.16334000	2.72968400
C	-5.11671700	0.33092200	2.81618500
H	-6.41580500	-0.98948300	1.71607100
H	-3.56338400	1.43175600	3.81602500
H	-5.92785100	0.87725400	3.28799800
N	-0.59137100	-1.33671400	1.45050800
S	0.62118200	-2.30147500	1.68718600
O	0.55619500	-3.10932600	2.93410100
O	1.05414400	-3.12434800	0.52232000
C	1.95742100	-1.13804700	1.95369600
C	1.74977700	0.17641700	2.36540200
C	3.25253000	-1.62860900	1.81008300
C	2.84348800	0.99419300	2.63739800
H	0.73185900	0.53829700	2.46759300
C	4.33924700	-0.80793500	2.10024500
H	3.39986900	-2.64570000	1.46004500
C	4.15276500	0.50770600	2.53314900
H	2.68322200	2.02446000	2.94577300
H	5.34806100	-1.19634100	1.98513500
C	5.32704100	1.38109700	2.89134600
H	5.20694600	2.38228500	2.47102100
H	5.41377400	1.48139600	3.97805000
H	6.26318800	0.95501000	2.52196600

M6$_{D}$(RS)

0 1

C	0.88245800	2.12877500	0.24046900
C	0.25873300	3.90468100	-0.84699700
C	-1.58698200	2.70874300	0.32715700
H	-1.70149500	1.62555900	0.30125300
N	1.53321500	3.79238100	-1.09824600
N	1.90886000	2.68628600	-0.40198000
N	-0.17164400	2.92282800	-0.00670500
C	-0.76476200	4.80503500	-1.45472000
H	-0.35464300	5.29053600	-2.33963700
H	-1.09430800	5.57107200	-0.73817300
C	-2.49580200	3.37784300	-0.75650900
O	-1.82482600	3.96654000	-1.85050200
C	3.27866800	2.24768400	-0.42582800
C	4.07423200	2.49585300	0.69192700
C	3.71021200	1.53580300	-1.54937400
C	5.36498600	1.96683100	0.67905000
C	5.00648600	1.02759300	-1.50886300
C	5.83944500	1.22201400	-0.40185100
H	6.01081600	2.13824000	1.53601300
H	5.37242200	0.45866300	-2.35965900
H	-3.14725400	2.61684000	-1.18626400
C	0.85669400	0.92910700	1.16848500
O	-0.01228500	0.92867800	2.01953600
C	1.98720700	-0.06765200	1.17054000
C	1.40637000	-1.48370100	1.13166100
H	2.54358800	0.08367900	2.10394000
H	0.57319100	-1.49856400	1.84103700
C	2.39922800	-2.54256200	1.56168900
C	1.93459700	-3.73955200	2.11817900
C	3.77924100	-2.37661900	1.40064900
C	2.82129200	-4.74356300	2.49778200
H	0.86492700	-3.88045300	2.25102800
C	4.66899700	-3.37830500	1.78177900
H	4.17024700	-1.45774600	0.96953400
C	4.19307400	-4.56650000	2.33053100
H	2.43980700	-5.66350600	2.92957000
H	5.73611800	-3.22831200	1.64956000
H	4.88584800	-5.34662300	2.62926900

C	7.21720500	0.61411500	-0.37255000
H	7.85751200	1.11789000	0.35388700
H	7.16014500	-0.44313400	-0.09353700
H	7.69243200	0.66902500	-1.35449000
C	3.54922500	3.26985100	1.87291200
H	3.01564800	4.16927300	1.55378300
H	2.85535400	2.66766700	2.47086900
H	4.37002800	3.56887000	2.52581100
C	2.78888500	1.29626700	-2.71392800
H	2.47310400	2.24064000	-3.16617100
H	3.28597600	0.69260600	-3.47464800
H	1.88584400	0.76581000	-2.39321100
H	-3.11134400	4.14090800	-0.25936100
C	-1.93941200	3.22847300	1.72888800
H	-1.17987800	2.88704000	2.43370500
H	-1.95219300	4.32377800	1.73021400
C	-3.29444000	2.67648900	2.11320800
C	-4.42296600	3.49121000	2.20805900
C	-3.43266700	1.29947900	2.32886900
C	-5.66965600	2.94371900	2.51003700
H	-4.32629200	4.56193500	2.04687100
C	-4.67604800	0.75092800	2.62639100
H	-2.55154500	0.66340700	2.26934600
C	-5.79970500	1.57330200	2.71526800
H	-6.53868900	3.58979200	2.58121700
H	-4.76780200	-0.31823500	2.79148200
H	-6.77018300	1.14665900	2.94679700
H	2.66709400	0.09229800	0.33659900
C	0.79870600	-1.73705000	-0.30040100
C	1.92787100	-2.06288500	-1.28955200
H	1.52615900	-2.19114800	-2.29436600
H	2.64450300	-1.23533700	-1.32095400
H	2.47058100	-2.96340500	-0.99304200
C	-0.20384500	-2.89425500	-0.16784100
C	-0.04453700	-4.12148200	-0.80929700
C	-1.33069400	-2.71573800	0.64730200
C	-0.98049500	-5.14539500	-0.63887400
H	0.81001700	-4.29507300	-1.45381900

C	-2.26326800	-3.72836400	0.81939500
H	-1.48401600	-1.74764800	1.11885600
C	-2.08901100	-4.95587900	0.17604400
H	-0.83508300	-6.09265500	-1.14928100
H	-3.13616000	-3.55808400	1.44311600
H	-2.81739500	-5.75051200	0.30598900
N	0.13049500	-0.46193000	-0.61598300
S	-0.69564400	-0.25523400	-1.94946400
O	-1.01823400	1.18583000	-2.03334400
O	-0.08549500	-0.83716700	-3.16438000
C	-2.28455000	-1.08319000	-1.80395400
C	-3.26311600	-0.55719100	-0.96573800
C	-2.49720200	-2.29449300	-2.45437800
C	-4.44773200	-1.25642200	-0.76174400
H	-3.10380100	0.39186100	-0.46335600
C	-3.68410000	-2.98696100	-2.24271800
H	-1.72606300	-2.68785600	-3.10750900
C	-4.66909700	-2.48732400	-1.38580600
H	-5.20645600	-0.84187400	-0.10257500
H	-3.84361700	-3.94093900	-2.73882500
C	-5.91944100	-3.28287200	-1.11597300
H	-6.69910500	-2.66207100	-0.66945600
H	-6.31359800	-3.72644600	-2.03383900
H	-5.70558900	-4.10372400	-0.42245500

M6$_D$(SR)

0 1

C	1.25343100	-2.00129800	0.21285900
C	1.00739300	-3.87945600	-0.85253200
C	-0.84987200	-3.36390900	0.76210700
H	-0.58823600	-3.43208100	1.81961000
N	2.15977400	-3.41754700	-1.24774200
N	2.30006900	-2.24687500	-0.57567300
N	0.41982000	-3.04704500	0.05756000
C	0.39395500	-5.20491500	-1.18017400
H	1.18441000	-5.92690500	-1.38531300
H	-0.25692600	-5.11869500	-2.06052500
C	-1.36463600	-4.71743400	0.23789700

O	-0.32987400	-5.63539000	-0.04912000
C	3.56151900	-1.55226800	-0.61995500
C	3.88514800	-0.82378200	-1.76856000
C	4.39568900	-1.64832200	0.49687200
C	5.08084900	-0.10891000	-1.74043600
C	5.57948300	-0.91133600	0.47038900
C	5.92950000	-0.12825400	-0.62997000
H	5.35692400	0.47838100	-2.61249100
H	6.24455900	-0.95777700	1.32840900
H	-2.00075800	-5.16320100	1.00371300
C	1.07817300	-0.91325700	1.24960100
O	0.33003300	-1.17890100	2.16899100
C	1.90308100	0.34638300	1.23222400
C	0.97506400	1.57151000	1.24380200
H	2.50365100	0.33161300	2.14943800
H	0.12889900	1.32169400	1.89355900
C	1.64214500	2.80521500	1.81499700
C	0.87061000	3.76378600	2.48103100
C	3.01757800	3.03007100	1.69077200
C	1.45382000	4.91474300	3.00396400
H	-0.19933200	3.60174100	2.58494800
C	3.60483000	4.17988600	2.21359100
H	3.64173300	2.30253300	1.17696200
C	2.82432600	5.12700800	2.87187800
H	0.83691600	5.64405300	3.51946900
H	4.67403500	4.33394000	2.10707400
H	3.28125500	6.02182000	3.28213400
C	7.21552100	0.65554300	-0.64579400
H	7.93800400	0.19378300	-1.32563800
H	7.04487900	1.67694600	-0.99521500
H	7.66589800	0.69927100	0.34740400
C	2.97855700	-0.79954500	-2.96654700
H	2.70727000	-1.81271600	-3.27421300
H	2.05024100	-0.25789600	-2.75938500
H	3.47678600	-0.30528000	-3.80233900
C	4.03920400	-2.50091900	1.68835600
H	3.62797800	-3.46651300	1.38049200
H	4.92758200	-2.68757200	2.29300500

H	3.30185200	-2.01637500	2.33803300
H	-1.96832100	-4.55055900	-0.66608700
C	-1.91818500	-2.29133600	0.53509100
H	-1.47476800	-1.29529200	0.61844200
H	-2.25705400	-2.39142200	-0.49684600
C	-3.06369700	-2.45431600	1.50433600
C	-4.29994300	-2.94845300	1.08236100
C	-2.89589800	-2.10845500	2.84982900
C	-5.35126400	-3.09690100	1.98586900
H	-4.44067100	-3.21357900	0.03700200
C	-3.94331900	-2.25786300	3.75341200
H	-1.93753500	-1.71141900	3.17621700
C	-5.17410600	-2.75358800	3.32329800
H	-6.30747000	-3.47933700	1.64325900
H	-3.80259700	-1.98248200	4.79384200
H	-5.99143800	-2.86768900	4.02796300
H	2.57793300	0.38215900	0.37969000
C	0.38099800	1.77479600	-0.19978600
C	1.45848600	2.34637500	-1.13507300
H	1.89396800	3.26188500	-0.72856900
H	2.26175000	1.61587300	-1.27328000
H	1.04017500	2.55249000	-2.12011000
C	-0.80931800	2.73797500	-0.05369200
C	-0.78104200	4.05492200	-0.51288900
C	-1.96513000	2.29083900	0.60009300
C	-1.87754900	4.90079900	-0.33291300
H	0.09634500	4.43828300	-1.02194000
C	-3.06025500	3.12600300	0.77615600
H	-2.00726900	1.25805100	0.93817400
C	-3.02058100	4.44143100	0.30961900
H	-1.83197800	5.92128900	-0.70119400
H	-3.95285300	2.74824300	1.26635700
H	-3.87582700	5.09662800	0.44492000
N	-0.04318200	0.41401900	-0.57159400
S	-0.64774500	0.08944900	-1.99717500
O	0.06446000	0.70066800	-3.14395100
O	-0.80306300	-1.38056400	-2.07846000
C	-2.30322900	0.78405200	-2.11585400

C	-2.46572400	2.04374400	-2.68893000
C	-3.38798200	0.14085500	-1.53077900
C	-3.70707600	2.66547700	-2.64396600
H	-1.61446100	2.53104300	-3.15201800
C	-4.63001800	0.76810500	-1.49887600
H	-3.26724700	-0.84333000	-1.09584800
C	-4.80310800	2.04515700	-2.03595600
H	-3.82635200	3.65673200	-3.07380300
H	-5.47343100	0.26164000	-1.03661800
C	-6.12237300	2.76336200	-1.92901200
H	-6.08269600	3.50716000	-1.12530500
H	-6.36282300	3.29455900	-2.85351200
H	-6.93722900	2.07163900	-1.70484600

M6$_{D}$(SS)

0 1

C	-0.13760400	2.06353700	0.49454100
C	0.85401200	3.63591000	-0.63198200
C	2.37316700	2.40453600	0.93776800
H	2.24359300	2.62809800	1.99826400
N	-0.39121200	3.68865300	-1.00641300
N	-0.99752500	2.70451600	-0.29677700
N	1.05420400	2.66422600	0.30495200
C	1.96443800	4.55160100	-1.04221000
H	1.56136800	5.54620800	-1.23412900
H	2.44503200	4.17283700	-1.95449800
C	3.40573100	3.35204900	0.30377100
O	2.88307200	4.63388600	0.02310200
C	-2.42808300	2.58746700	-0.38335500
C	-2.98153900	2.23843700	-1.61988500
C	-3.19492200	2.91303000	0.74098100
C	-4.37098700	2.15072800	-1.68391700
C	-4.57815600	2.78504400	0.62326400
C	-5.18070800	2.39308600	-0.57282300
H	-4.83241400	1.88421100	-2.63193900
H	-5.19939000	3.01944700	1.48343400
H	4.23322000	3.47599700	1.00347000
C	-0.39474900	1.01722200	1.55953400

O	0.38944900	1.02805000	2.48711800
C	-1.58600700	0.09374600	1.54469700
C	-1.10638700	-1.37550700	1.56185600
H	-2.13615800	0.29369400	2.47085100
H	-0.18679400	-1.39733400	2.15986900
C	-2.10566400	-2.29185600	2.23417700
C	-1.65864800	-3.29540500	3.09748900
C	-3.48292100	-2.16886400	2.01427700
C	-2.55601200	-4.16349700	3.71703600
H	-0.59272500	-3.39406500	3.28834600
C	-4.38295500	-3.02865200	2.63542000
H	-3.85345700	-1.40558800	1.33374200
C	-3.92242600	-4.03144800	3.48758700
H	-2.18625800	-4.93623600	4.38357000
H	-5.44679000	-2.91911800	2.44965600
H	-4.62517000	-4.70260100	3.97088100
C	-6.67413600	2.22875000	-0.67018100
H	-6.94239200	1.16854700	-0.62025900
H	-7.18478000	2.74314500	0.14602100
H	-7.05138800	2.61821900	-1.61866400
C	-2.13196400	1.98434900	-2.83790400
H	-1.97688200	2.91902600	-3.38607700
H	-1.14769200	1.58144600	-2.58495400
H	-2.63258900	1.27671000	-3.50274800
C	-2.57776800	3.41940000	2.02084900
H	-1.73650800	4.08960200	1.82268500
H	-3.32235100	3.97324100	2.59439200
H	-2.21683800	2.60931200	2.66234000
H	3.79251900	2.89989800	-0.62136000
C	2.82375400	0.95811800	0.73396000
H	2.00062300	0.28150200	0.96910900
H	3.04357000	0.81928300	-0.32735400
C	4.02866900	0.63592300	1.58412400
C	5.27536600	0.39239400	1.00394700
C	3.90401900	0.56808300	2.97621700
C	6.38034100	0.09037200	1.79877600
H	5.37511900	0.42875300	-0.07809900
C	5.00640100	0.27087400	3.77140600

H	2.92975700	0.73748400	3.42959800
C	6.24855100	0.03234100	3.18361400
H	7.34293200	-0.09972400	1.33475500
H	4.89643500	0.21855900	4.84991600
H	7.10794500	-0.20194000	3.80340600
H	-2.24881900	0.28250800	0.70113400
C	-0.67387900	-1.82016600	0.10867200
C	0.21232400	-3.06727100	0.24809700
H	0.49401300	-3.45624200	-0.73510200
H	1.12191100	-2.79840700	0.78948700
H	-0.28519300	-3.86370800	0.80626200
C	-1.93826400	-2.13443500	-0.70854300
C	-2.48042300	-3.42326100	-0.75258900
C	-2.65181900	-1.11275500	-1.34077700
C	-3.69443000	-3.67678000	-1.38921600
H	-1.97068400	-4.25033100	-0.27134900
C	-3.87104000	-1.35406000	-1.96454500
H	-2.21993000	-0.12094200	-1.36386600
C	-4.40250800	-2.64190000	-1.99077400
H	-4.08854500	-4.68832900	-1.40421600
H	-4.40193100	-0.53367300	-2.43918000
H	-5.35123900	-2.83620600	-2.48107000
N	0.09433800	-0.65651600	-0.34863600
S	0.68146100	-0.51258900	-1.80129100
O	-0.17968600	-0.97844800	-2.90666400
O	1.14458600	0.89381800	-1.93702900
C	2.18167100	-1.50586500	-2.00173000
C	2.50743800	-1.94405700	-3.28301500
C	3.03551400	-1.78930300	-0.93729100
C	3.68888900	-2.64936700	-3.49627300
H	1.82435900	-1.74145700	-4.10063400
C	4.21447900	-2.49229600	-1.16035300
H	2.77178800	-1.48190900	0.06784800
C	4.56281200	-2.92840100	-2.44294500
H	3.93382900	-2.99310000	-4.49758200
H	4.87277100	-2.70871800	-0.32260400
C	5.85376500	-3.66988200	-2.67594700
H	5.84718400	-4.18309700	-3.63972700

H	6.70356200	-2.97965800	-2.67040400
H	6.02999000	-4.41033400	-1.89149600

TS7$_D$(RR)

0 1

C	0.71639600	-0.59962000	1.06359800
C	2.28403200	-2.10334600	1.29404000
C	3.14129200	0.17083200	0.72625600
H	2.78498600	0.81423700	-0.08100900
N	1.18248600	-2.72975300	1.58944600
N	0.21575300	-1.77996000	1.45264500
N	2.05015800	-0.79195500	0.99562800
C	3.66934800	-2.64414600	1.15537000
H	3.62201600	-3.71030700	0.92943900
H	4.24369200	-2.49300700	2.08110200
C	4.43149900	-0.60108200	0.30068500
O	4.25725100	-1.98195900	0.06298900
C	-1.11409500	-2.05616500	1.93402200
C	-1.47652900	-1.45998500	3.15021900
C	-1.96931400	-2.87733900	1.20021000
C	-2.79787400	-1.59773400	3.55723000
C	-3.28847100	-2.97667100	1.65206200
C	-3.72736000	-2.31903800	2.79888500
H	-3.11343400	-1.13335200	4.48795800
H	-3.98347100	-3.59608600	1.09024400
H	4.80805900	-0.17904400	-0.63218500
C	0.01828900	0.76381700	0.85795600
O	0.73410300	1.73486900	1.11995400
C	-1.48782000	0.83658500	1.06304300
C	-1.97569600	1.93960300	0.14365000
H	-1.68915600	1.08736800	2.10914300
H	-1.36453500	2.82272700	0.36116900
C	-3.42598000	2.31203700	0.35005600
C	-3.81372400	3.65263100	0.28008700
C	-4.41163800	1.34903000	0.60382500
C	-5.14803300	4.02576900	0.43157800
H	-3.05702500	4.41500500	0.11203300
C	-5.74382400	1.71753200	0.75907800

H	-4.14174900	0.29732800	0.66288800
C	-6.11861200	3.05737500	0.66840900
H	-5.42513000	5.07362300	0.37143600
H	-6.49357200	0.95509200	0.94774800
H	-7.15882000	3.34317100	0.78815300
C	-5.16846400	-2.38648300	3.22903100
H	-5.25120700	-2.52755200	4.30941900
H	-5.68015700	-1.45091100	2.98023100
H	-5.69522000	-3.20117600	2.72888300
C	-0.46955100	-0.70768300	3.98445200
H	0.45113100	-1.28829200	4.10003400
H	-0.19439600	0.25711500	3.54398800
H	-0.87689200	-0.51233300	4.97718300
C	-1.51959200	-3.67442400	0.00224200
H	-0.43275100	-3.73375000	-0.05844200
H	-1.90864300	-4.69231800	0.08123800
H	-1.89904300	-3.25708200	-0.93541200
H	5.19422300	-0.46087300	1.07990300
C	3.43562200	1.01055000	1.98006300
H	2.49304300	1.38821900	2.37692100
H	3.91194600	0.37701500	2.73738400
C	4.33656600	2.16658200	1.61060600
C	5.61416700	2.30512000	2.15403700
C	3.88043800	3.11742200	0.68893000
C	6.42583900	3.37975100	1.79130100
H	5.97500700	1.57063700	2.87005300
C	4.69125300	4.18746900	0.32575800
H	2.88747900	3.00596800	0.25757500
C	5.96594100	4.32234000	0.87659000
H	7.41666700	3.47774100	2.22367700
H	4.32779200	4.91877400	-0.38925400
H	6.59647000	5.15859100	0.59203000
H	-1.98498400	-0.10415700	0.82835400
C	-1.59042200	1.50294300	-1.32529000
C	-1.37209200	2.81014800	-2.10862100
H	-2.19310400	3.50222800	-1.90171800
H	-0.44222700	3.27568200	-1.77101000
H	-1.31137800	2.64028600	-3.18226600

C	-2.71783600	0.66293200	-1.93043200
C	-3.74310600	1.24013500	-2.68579000
C	-2.80372200	-0.70318800	-1.64919000
C	-4.81686200	0.47712200	-3.13853900
H	-3.71977500	2.29988200	-2.91712500
C	-3.88101600	-1.46853800	-2.08574300
H	-2.00671900	-1.15779300	-1.07198100
C	-4.89545400	-0.87972300	-2.83666600
H	-5.59887200	0.94953400	-3.72512200
H	-3.92951700	-2.52697300	-1.84249700
H	-5.73615000	-1.47175200	-3.18406500
N	-0.38165800	0.65344200	-1.13535800
S	0.78397800	0.79137800	-2.22656900
O	0.25290000	0.93275700	-3.59514000
O	1.82349700	1.78223500	-1.86473000
C	1.61899300	-0.78696100	-2.18969600
C	0.96329500	-1.96496600	-1.85942000
C	2.92664800	-0.81438500	-2.67709900
C	1.64394800	-3.17759700	-1.96346000
H	-0.06769800	-1.92185900	-1.52594300
C	3.57862100	-2.03117000	-2.81081000
H	3.41804800	0.11680800	-2.94229600
C	2.95560300	-3.22735400	-2.43758700
H	1.14100800	-4.10232300	-1.69122700
H	4.59927400	-2.05624700	-3.18328000
C	3.71044000	-4.52697300	-2.51461400
H	3.05384700	-5.38226400	-2.34283300
H	4.18875400	-4.64815600	-3.49018600
H	4.50249600	-4.54601100	-1.75823000

TS7$_D$(RS)

0 1

C	-1.09493900	-1.84766300	0.25411200
C	-2.55036100	-2.45277100	1.75521700
C	-3.35808700	-0.67130200	0.23042300
H	-2.80518800	0.18146500	-0.16066500
N	-1.49586200	-3.17349200	2.01163600
N	-0.60169200	-2.79287300	1.05655300

N	-2.35301500	-1.64730500	0.67529300
C	-3.81158000	-2.26877900	2.53175000
H	-3.70876100	-2.70899400	3.52319400
H	-4.66637700	-2.73083300	2.01724500
C	-4.21810500	-0.20804400	1.44814900
O	-3.97210900	-0.87629000	2.67273100
C	0.69438900	-3.41241100	1.00030900
C	0.89282600	-4.43948000	0.07759500
C	1.69987700	-2.90511100	1.82684100
C	2.18700500	-4.94465200	-0.04093300
C	2.97619400	-3.44184200	1.66662200
C	3.23955700	-4.44625700	0.73010300
H	2.37754900	-5.73882000	-0.75779000
H	3.78633100	-3.05975600	2.28264400
H	-4.00539600	0.84330100	1.64188000
C	-0.48418500	-1.12784400	-0.94582900
O	-1.30291900	-0.68487700	-1.75494200
C	0.93333500	-1.45357500	-1.38136800
C	1.55603400	-0.13768100	-1.82784700
H	0.87767500	-2.16211000	-2.21573800
H	0.86965300	0.29957000	-2.55857100
C	2.90994300	-0.27242900	-2.48507300
C	3.33878800	0.71750000	-3.37683800
C	3.76973000	-1.34202900	-2.21361600
C	4.59470100	0.64760800	-3.97302900
H	2.67530200	1.54905100	-3.60103400
C	5.02565400	-1.41637100	-2.81216800
H	3.46186300	-2.12862800	-1.52842500
C	5.44395300	-0.42075500	-3.69146500
H	4.90778600	1.42490400	-4.66299400
H	5.67751700	-2.25613300	-2.59130300
H	6.42173000	-0.47960300	-4.15862800
C	4.64264000	-4.96084300	0.54086800
H	4.64356200	-5.94855300	0.07601600
H	5.21069700	-4.28507300	-0.10718600
H	5.17177700	-5.02331000	1.49440200
C	-0.23884500	-4.94484700	-0.77773200
H	-1.11684200	-5.18089400	-0.16976800

H	-0.54391300	-4.19673900	-1.51830200
H	0.06163200	-5.84394100	-1.31691200
C	1.40370200	-1.81083100	2.81448400
H	0.88723800	-0.98137800	2.31976700
H	0.75102700	-2.17767900	3.61305200
H	2.32859100	-1.44172000	3.26223500
H	-5.27708500	-0.31958000	1.17598900
C	-4.20533900	-1.28608600	-0.89384700
H	-3.51839000	-1.71350600	-1.62916200
H	-4.83089200	-2.09231500	-0.49305700
C	-5.06120600	-0.22728200	-1.54737900
C	-6.45262800	-0.24321200	-1.44573600
C	-4.44312000	0.80884900	-2.25709300
C	-7.21878600	0.75553400	-2.04565400
H	-6.94012000	-1.04519900	-0.89685900
C	-5.20587400	1.80626800	-2.85487400
H	-3.35802500	0.81843100	-2.33723800
C	-6.59686300	1.78187300	-2.75047300
H	-8.30046400	0.73031100	-1.96037300
H	-4.71600400	2.60421900	-3.40375500
H	-7.19157000	2.56062300	-3.21684800
H	1.51694100	-1.89728900	-0.57778000
C	1.55774000	0.81564500	-0.56854400
C	2.80165700	0.55754800	0.29215400
H	2.76211600	1.16857400	1.19842300
H	2.81948300	-0.49484700	0.59427300
H	3.72937600	0.77258300	-0.24301300
C	1.49128300	2.25470900	-1.08738100
C	2.52813400	3.17047500	-0.90295500
C	0.36198300	2.66243200	-1.80882500
C	2.43472700	4.46678900	-1.41025500
H	3.41355200	2.89062700	-0.34336200
C	0.26390900	3.95391300	-2.31250100
H	-0.45674900	1.95978600	-1.94001000
C	1.30242200	4.86499100	-2.11248900
H	3.25028800	5.16499700	-1.24913300
H	-0.62541000	4.25293700	-2.85849900
H	1.22714100	5.87459700	-2.50389300

N	0.38035300	0.36864200	0.21031300
S	-0.56515300	1.29485500	1.10561900
O	-1.54183700	2.11264700	0.35487100
O	-1.17877600	0.40319000	2.11434900
C	0.44460300	2.46100600	2.01118900
C	0.58339500	3.76758900	1.55629900
C	1.08101100	2.03683000	3.17672400
C	1.38621600	4.65185700	2.27123600
H	0.07867400	4.07795100	0.64598700
C	1.87982000	2.93082700	3.87863300
H	0.93788100	1.01979500	3.52817200
C	2.04469600	4.24905400	3.43564000
H	1.50683000	5.67079000	1.91376600
H	2.37973500	2.60705500	4.78733600
C	2.88964200	5.21809200	4.22083400
H	2.30937600	5.66024900	5.03718100
H	3.75323600	4.71807900	4.66573100
H	3.24783700	6.03339200	3.58879600

TS7$_D$(SR)

0 1

C	0.54108300	-2.10612900	0.26199500
C	-0.06198100	-4.08420300	-0.44083700
C	-1.82191900	-2.91546700	0.90500200
H	-1.63339400	-2.74641000	1.96634700
N	1.15353800	-3.93025700	-0.87789200
N	1.51537600	-2.69779300	-0.43506800
N	-0.48121500	-2.99278500	0.26559100
C	-0.90530800	-5.31924100	-0.52013500
H	-0.25636900	-6.19506600	-0.53478600
H	-1.52183500	-5.31093800	-1.42898200
C	-2.56251400	-4.24671100	0.67297300
O	-1.70689600	-5.37022500	0.63836200
C	2.88849400	-2.30038500	-0.62427400
C	3.26513700	-1.65872900	-1.79991400
C	3.78559000	-2.62037500	0.40524400
C	4.60178300	-1.25195500	-1.89350500
C	5.09796300	-2.18921200	0.26357100

C	5.51776200	-1.48294600	-0.87200900
H	4.92242800	-0.73384700	-2.79423400
H	5.81356900	-2.40799600	1.05215400
H	-3.27011100	-4.39371400	1.49027100
C	0.56222300	-0.81180500	1.09011800
O	-0.35430800	-0.70618600	1.90460100
C	1.88293400	-0.10150300	1.33080200
C	1.63314900	1.40277000	1.27328300
H	2.23429700	-0.39031400	2.32785600
H	0.82007000	1.60040600	1.97482700
C	2.82639000	2.23396700	1.69690500
C	2.62609200	3.53372700	2.17495900
C	4.13847100	1.75555000	1.60951800
C	3.70215400	4.33614300	2.54442700
H	1.61307200	3.91866100	2.25556900
C	5.21695000	2.55498900	1.98199100
H	4.33147300	0.74903500	1.24519100
C	5.00384900	3.84926200	2.44855400
H	3.52248400	5.34114400	2.91351700
H	6.22653200	2.16225600	1.90727600
H	5.84443300	4.47118000	2.73920600
C	6.93243200	-0.97584500	-0.96905700
H	7.20615900	-0.76001900	-2.00350000
H	7.04256900	-0.05053800	-0.39363600
H	7.64103800	-1.70194200	-0.56373100
C	2.30776700	-1.38954700	-2.92623800
H	1.34958000	-1.89045700	-2.78868700
H	2.09558400	-0.31934700	-3.00429400
H	2.75054900	-1.71643800	-3.87095900
C	3.32832600	-3.37872300	1.62327600
H	2.80692900	-4.29808800	1.34006500
H	4.18104100	-3.64459200	2.24904200
H	2.63802200	-2.78514400	2.23295700
H	-3.12686600	-4.18915500	-0.26947100
C	-2.63197500	-1.76049200	0.32077300
H	-1.97543700	-0.89859300	0.27727800
H	-2.91197300	-1.99517800	-0.71044700
C	-3.84139100	-1.42576500	1.15677400

C	-5.13548400	-1.67936000	0.70087800
C	-3.66813100	-0.80010600	2.39694700
C	-6.24143400	-1.31030300	1.46549600
H	-5.27799600	-2.15170600	-0.26780600
C	-4.77094800	-0.43200200	3.16201200
H	-2.65739400	-0.58912400	2.73996400
C	-6.06147800	-0.68466300	2.69637800
H	-7.24298700	-1.50678800	1.09564200
H	-4.62537300	0.05805100	4.11976200
H	-6.92204200	-0.39369400	3.29030200
H	2.63450400	-0.38250800	0.59928100
C	1.12511400	1.71935500	-0.17886800
C	2.33519600	1.83139500	-1.11988200
H	2.91784300	2.72987700	-0.89910900
H	2.98850400	0.96243600	-0.99357800
H	2.01007600	1.85537300	-2.15894900
C	0.28239600	3.00834100	-0.19638700
C	0.36039800	3.90926300	-1.26288700
C	-0.64520400	3.28448700	0.81606900
C	-0.45333700	5.04047100	-1.31518000
H	1.05288500	3.72835200	-2.07634100
C	-1.45456300	4.41435100	0.77339400
H	-0.76645100	2.59716400	1.64767300
C	-1.36282700	5.30138500	-0.29649300
H	-0.37191400	5.71815800	-2.15939500
H	-2.16740200	4.59341200	1.57249400
H	-1.99534200	6.18248100	-0.33530400
N	0.31009500	0.52483600	-0.49331500
S	-0.53640800	0.39618100	-1.83024300
O	0.04797400	1.07553200	-3.00339300
O	-0.83509200	-1.04204400	-2.01116100
C	-2.13898000	1.18393500	-1.60966000
C	-2.85475400	1.55109900	-2.74613000
C	-2.70947500	1.32005100	-0.34828400
C	-4.15567900	2.02778100	-2.61267900
H	-2.39322600	1.45854800	-3.72379100
C	-4.01375500	1.78439800	-0.22711100
H	-2.13466000	1.05719500	0.53491800

C	-4.75720600	2.13974900	-1.35592400
H	-4.71728500	2.30839100	-3.49955300
H	-4.46297000	1.85903400	0.76085000
C	-6.16815800	2.64752600	-1.20885600
H	-6.70990500	2.59444700	-2.15560500
H	-6.71531900	2.06420900	-0.46318500
H	-6.17376200	3.69110500	-0.87785300

TS7$_D$(SS)

0 1

C	0.09300700	1.91675500	0.52350600
C	0.91335800	3.89365200	0.06325500
C	2.60531500	2.31398600	1.00267000
H	2.49453600	2.03777000	2.05144200
N	-0.34750600	3.97443500	-0.23874600
N	-0.84720900	2.74426500	0.05103600
N	1.23426700	2.65203100	0.53149600
C	1.91027000	5.01237500	0.06747600
H	1.39145600	5.94901400	0.27353200
H	2.41418200	5.08529900	-0.90591400
C	3.50692000	3.55233200	0.85483200
O	2.83396800	4.76862900	1.10280100
C	-2.26621000	2.59043400	-0.16786700
C	-2.69570200	2.54499200	-1.50072200
C	-3.14369500	2.64846100	0.91558300
C	-4.06690100	2.48763600	-1.72367200
C	-4.50809900	2.53957700	0.63425900
C	-4.98480100	2.44758900	-0.67090400
H	-4.42847700	2.45533000	-2.74878500
H	-5.21113400	2.54702000	1.46361200
H	4.31921400	3.46705000	1.57836200
C	-0.02499300	0.54741600	1.22131200
O	0.95377400	0.25789100	1.90598400
C	-1.38446600	-0.03545800	1.55345000
C	-1.26179400	-1.55550400	1.44115000
H	-1.62330200	0.25243600	2.58270800
H	-0.34280600	-1.83930900	1.96770700
C	-2.41064000	-2.30042800	2.07637900

C	-2.17381800	-3.51191200	2.73136100
C	-3.72411400	-1.82022200	2.01855100
C	-3.21899000	-4.23746500	3.29942100
H	-1.15562500	-3.88796600	2.79824100
C	-4.76944300	-2.53614600	2.59330700
H	-3.93612000	-0.88763700	1.49989900
C	-4.52117700	-3.75002700	3.23216100
H	-3.01357400	-5.17763100	3.80165800
H	-5.78193900	-2.14873800	2.53664900
H	-5.33744700	-4.30851900	3.67926600
C	-6.45350700	2.28134100	-0.95426100
H	-6.66995100	1.23479000	-1.19492600
H	-7.06158900	2.56125400	-0.09195700
H	-6.76075800	2.88584400	-1.81110000
C	-1.71744600	2.56374600	-2.64410500
H	-1.34765000	3.58061700	-2.81225700
H	-0.84867900	1.92688000	-2.44662700
H	-2.20368300	2.21980300	-3.55897600
C	-2.69557800	2.85052600	2.34002000
H	-3.01541200	3.83683800	2.68822000
H	-3.15509200	2.10513100	2.99433400
H	-1.61297600	2.79160100	2.46387300
H	3.94086800	3.57272600	-0.15561400
C	3.19058200	1.15445400	0.20231300
H	2.41767700	0.40003800	0.10039300
H	3.42919300	1.49319600	-0.81049400
C	4.39843100	0.54927400	0.87284900
C	5.65358200	0.57582300	0.26503200
C	4.25062100	-0.11419200	2.09670000
C	6.74436100	-0.05585500	0.86130900
H	5.77254300	1.07667900	-0.69233200
C	5.33907500	-0.74106900	2.69563200
H	3.26591800	-0.15772400	2.55682200
C	6.58916200	-0.71677500	2.07710000
H	7.71398100	-0.03265100	0.37338000
H	5.21136700	-1.25691300	3.64232600
H	7.43683100	-1.21026000	2.54175500
H	-2.16326300	0.32893000	0.88296600

C	-0.99899000	-1.87257500	-0.08072800
C	-0.28407300	-3.22765200	-0.18439100
H	-0.09262400	-3.48427000	-1.23041600
H	0.66658400	-3.19078100	0.34983200
H	-0.88295800	-4.02439600	0.26181200
C	-2.33050200	-1.91936600	-0.85101200
C	-3.13789000	-3.06592800	-0.83117600
C	-2.80721100	-0.80402700	-1.53868600
C	-4.36821400	-3.09465800	-1.47986500
H	-2.82512400	-3.94876000	-0.28377000
C	-4.03965800	-0.82620900	-2.18840700
H	-2.18793700	0.08338100	-1.57767200
C	-4.82757200	-1.97247400	-2.16449500
H	-4.96987300	-3.99788200	-1.44603000
H	-4.37780400	0.05471000	-2.72492900
H	-5.78485200	-1.99344900	-2.67624600
N	-0.14492000	-0.73793800	-0.46331400
S	0.62675900	-0.66496700	-1.84213300
O	-0.09424900	-1.24332400	-2.98844700
O	1.06983600	0.73955600	-2.00364500
C	2.14780200	-1.62139400	-1.72999300
C	2.77882500	-1.98755200	-2.91528800
C	2.74333800	-1.90151400	-0.50255700
C	4.02210600	-2.61222400	-2.86735800
H	2.29586800	-1.78278200	-3.86542700
C	3.98909600	-2.51673300	-0.46690900
H	2.23970300	-1.61712100	0.41970900
C	4.64969100	-2.87338300	-1.64659300
H	4.51761900	-2.89199800	-3.79301700
H	4.46798500	-2.69616100	0.49349400
C	6.00894400	-3.51982800	-1.58388600
H	6.44554800	-3.62818000	-2.57870200
H	6.68921200	-2.92065600	-0.97094900
H	5.94915000	-4.51343300	-1.12907300

M7$_D$(RR)

0 1

C	-0.78412400	-0.58578300	-0.85147300

C	-2.40172600	-1.99955300	-1.30137600
C	-3.20797600	0.23076500	-0.56954700
H	-2.87151300	0.84629600	0.26519000
N	-1.31793600	-2.62952800	-1.64223400
N	-0.32015600	-1.73630600	-1.37064200
N	-2.12812800	-0.74408700	-0.83802600
C	-3.81630500	-2.48075900	-1.29656900
H	-3.83598200	-3.56798200	-1.21360300
H	-4.33193400	-2.18108200	-2.22095700
C	-4.53523600	-0.52335300	-0.22717700
O	-4.43630000	-1.93229000	-0.15869300
C	0.97807600	-1.99000300	-1.94600900
C	1.23517200	-1.36897000	-3.17791000
C	1.89457100	-2.82221300	-1.30967700
C	2.51611200	-1.49191000	-3.69962500
C	3.17308600	-2.90605700	-1.87213100
C	3.50956700	-2.22599000	-3.03921500
H	2.75012700	-1.00507300	-4.64295000
H	3.91485700	-3.53427400	-1.38464800
H	-4.89817500	-0.20091000	0.74942100
C	-0.06027900	0.77314600	-0.45657800
O	-0.83600500	1.76478700	-0.71488500
C	1.37078200	0.87263000	-1.04913500
C	1.97476500	1.95529000	-0.18160200
H	1.32830000	1.16707100	-2.09897000
H	1.39509900	2.86476200	-0.36778800
C	3.43342100	2.26872600	-0.41699800
C	3.87282300	3.59043300	-0.30256900
C	4.37543500	1.28162800	-0.73215500
C	5.21595000	3.92137100	-0.47077400
H	3.14845700	4.37229000	-0.08751700
C	5.71733500	1.60817800	-0.90219600
H	4.06519300	0.24494900	-0.83201600
C	6.14411000	2.92839000	-0.76812300
H	5.53342900	4.95509300	-0.37612400
H	6.43358500	0.82725700	-1.13960500
H	7.19151000	3.17981500	-0.90164600
C	4.90667500	-2.28244500	-3.59769600

H	4.89217200	-2.46410300	-4.67527600
H	5.41863000	-1.32874600	-3.43328300
H	5.49419400	-3.06859800	-3.12007000
C	0.15499100	-0.60667000	-3.90333800
H	-0.71575800	-1.24705600	-4.07906700
H	-0.19004500	0.26628100	-3.33840600
H	0.52247700	-0.25581200	-4.86844700
C	1.54106800	-3.65541100	-0.10562900
H	0.46155400	-3.70943000	0.03657600
H	1.91698200	-4.67168900	-0.24607600
H	1.99588000	-3.26867500	0.81198800
H	-5.28795900	-0.25052900	-0.98045700
C	-3.41243600	1.11813600	-1.81000300
H	-2.42888000	1.45714600	-2.13702200
H	-3.88481600	0.52868900	-2.60546300
C	-4.26628400	2.31104500	-1.45233100
C	-5.53064100	2.50743900	-2.00860700
C	-3.77342300	3.24247700	-0.52888300
C	-6.29504500	3.61975400	-1.65637100
H	-5.91717000	1.78984900	-2.72845000
C	-4.53775100	4.34971500	-0.17613000
H	-2.78328400	3.08206800	-0.10496600
C	-5.80018800	4.54203500	-0.73911700
H	-7.27562200	3.76321100	-2.09948700
H	-4.14750000	5.06819900	0.53804500
H	-6.39340000	5.40820400	-0.46358600
H	1.94167600	-0.05042200	-0.95189800
C	1.61273400	1.51271700	1.28721600
C	1.32321800	2.80107700	2.07146700
H	2.13380500	3.51449100	1.89744800
H	0.39607400	3.23699400	1.69131300
H	1.23003200	2.62752500	3.14272300
C	2.73436700	0.68045000	1.89961900
C	3.71374500	1.26076500	2.70907900
C	2.87268200	-0.66973400	1.56556100
C	4.79641700	0.51312400	3.16762900
H	3.64601200	2.30855900	2.98176700
C	3.95632900	-1.41858800	2.01296800

H	2.12059100	-1.12735300	0.93167500
C	4.92629800	-0.82829000	2.81972000
H	5.54387100	0.98541300	3.79755700
H	4.04666400	-2.46384500	1.72890600
H	5.77300900	-1.40767000	3.17357700
N	0.40793000	0.62716600	1.06182500
S	-0.66626500	0.60778200	2.31828400
O	0.12015000	0.59507900	3.55484600
O	-1.72259400	1.62020900	2.20633600
C	-1.47522400	-0.96986300	2.20720300
C	-0.73867400	-2.12695300	1.98631300
C	-2.83262200	-1.02464400	2.51354800
C	-1.39234200	-3.35565700	2.00944800
H	0.32868800	-2.05776200	1.80240000
C	-3.46424900	-2.25981000	2.54989500
H	-3.37300000	-0.10471700	2.71270400
C	-2.76160300	-3.43778000	2.27875400
H	-0.83061100	-4.26796900	1.82795300
H	-4.52792100	-2.31138200	2.76488200
C	-3.48402300	-4.75781300	2.26787900
H	-2.79905400	-5.58769500	2.08556400
H	-3.99295000	-4.93241900	3.21991100
H	-4.24851900	-4.76271000	1.48421900

M7$_D$(RS)

0 1

C	-0.12454200	-1.99246900	-0.06569900
C	0.60349600	-3.86404400	-0.93802000
C	2.37689000	-2.51721300	0.19755900
H	2.60615300	-1.52311000	-0.17934300
N	-0.66818400	-3.85331100	-1.20108400
N	-1.11219400	-2.69433200	-0.63565800
N	0.97305600	-2.77246200	-0.20166200
C	1.63268300	-4.81452800	-1.46328400
H	1.28957700	-5.22076200	-2.41481600
H	1.80848000	-5.64002500	-0.75951900
C	3.29598400	-3.57656200	-0.45685500
O	2.81336200	-4.07717300	-1.68469500

C	-2.53357200	-2.45601000	-0.67484400
C	-3.28882800	-2.93258200	0.40974500
C	-3.10696300	-1.87147300	-1.79923800
C	-4.65899600	-2.70903800	0.38524900
C	-4.49471700	-1.67873400	-1.77771600
C	-5.27679400	-2.06315500	-0.69493700
H	-5.26443700	-3.05409600	1.21969800
H	-4.96443000	-1.20890300	-2.63856000
H	4.26652200	-3.11759100	-0.64888600
C	-0.13355400	-0.54403300	0.56887200
O	1.00993500	-0.24575600	1.10406300
C	-1.36835800	-0.35950600	1.48605200
C	-1.57605600	1.14527400	1.46571100
H	-1.13033100	-0.73485400	2.48406000
H	-0.73366200	1.58326400	2.00286800
C	-2.84395600	1.67371800	2.09531200
C	-2.86295700	2.99341400	2.56158400
C	-4.01322500	0.91416000	2.19938500
C	-4.01815100	3.54352700	3.10920200
H	-1.95737200	3.59110700	2.48843200
C	-5.17019700	1.46106600	2.75120000
H	-4.03196600	-0.11328200	1.84446800
C	-5.17803400	2.77713400	3.20596800
H	-4.01136700	4.56898200	3.46502900
H	-6.06768400	0.85459900	2.82586900
H	-6.07946200	3.20143400	3.63627200
C	-6.75843500	-1.79530100	-0.66852700
H	-7.30871000	-2.66309800	-0.29643600
H	-6.98087200	-0.95517000	-0.00262200
H	-7.13433300	-1.54463600	-1.66213600
C	-2.62858300	-3.65856000	1.55167000
H	-2.06528400	-4.52316900	1.18730700
H	-1.92559000	-3.01233400	2.08732800
H	-3.37623500	-4.00891400	2.26425500
C	-2.30981900	-1.45718100	-3.00324300
H	-1.26596100	-1.76585300	-2.93845500
H	-2.75060800	-1.89298000	-3.90406000
H	-2.31952400	-0.36987000	-3.11240500

H	3.44222100	-4.41522600	0.24063700
C	2.54942700	-2.56477400	1.72388600
H	1.74197900	-1.98508300	2.16866400
H	2.48657900	-3.60605400	2.05955100
C	3.87756900	-1.95564600	2.11046500
C	5.00058700	-2.74220300	2.37358300
C	3.99485300	-0.56117000	2.17309600
C	6.22368100	-2.15100700	2.68862200
H	4.91701500	-3.82594500	2.34022600
C	5.21508900	0.02867300	2.49051000
H	3.10857500	0.04047900	1.98307600
C	6.33414000	-0.76451200	2.74519600
H	7.08787800	-2.77509900	2.89310700
H	5.29247100	1.11039000	2.54933400
H	7.28476100	-0.30347800	2.99325900
H	-2.24988900	-0.86972300	1.10067300
C	-1.42112100	1.53838700	-0.05118000
C	-2.76762600	1.50225400	-0.77241100
H	-2.61827400	1.62196100	-1.84724600
H	-3.24921000	0.53655600	-0.59479400
H	-3.43199000	2.28822800	-0.40503900
C	-0.72615000	2.90054600	-0.15099000
C	-1.23882900	3.94297200	-0.92172700
C	0.46466100	3.11814100	0.55809200
C	-0.59092700	5.17921700	-0.97120000
H	-2.14784800	3.80344100	-1.49574500
C	1.10928300	4.34669100	0.51285400
H	0.90971600	2.28721400	1.10183000
C	0.57840100	5.38775500	-0.25008400
H	-1.00854100	5.97796500	-1.57613400
H	2.03828500	4.48666100	1.05748200
H	1.08113000	6.34917700	-0.28877200
N	-0.58173300	0.40641200	-0.57662600
S	0.34509200	0.58150700	-1.92581300
O	0.91248200	-0.74927800	-2.17999900
O	-0.48897700	1.21198800	-2.95081400
C	1.74208800	1.67709300	-1.71115000
C	2.75300000	1.35064300	-0.81184200

C	1.81104700	2.82535100	-2.49215000
C	3.84272900	2.20251200	-0.68836300
H	2.63646000	0.48349300	-0.17158600
C	2.91130700	3.66519200	-2.35492800
H	1.00682500	3.05794500	-3.18032300
C	3.93556300	3.37259300	-1.45063600
H	4.63238200	1.95580300	0.01703600
H	2.97001900	4.56972200	-2.95409900
C	5.09292700	4.31979800	-1.27260200
H	5.97092600	3.80462000	-0.87736300
H	5.36460300	4.79574900	-2.21764900
H	4.82842700	5.11549600	-0.56781400

$M7_D$(SR)

0 1

C	-0.03485500	-1.78985000	0.38151100
C	0.51614700	-3.65864300	-0.61351200
C	2.23248000	-1.89126400	-0.82990000
H	2.12427900	-0.81179000	-0.96260300
N	-0.65931800	-3.88164300	-0.10643200
N	-0.98794200	-2.71509000	0.51911100
N	0.95455500	-2.40301300	-0.29888000
C	1.26196900	-4.55562200	-1.56411400
H	0.60411600	-4.73304900	-2.42558600
H	1.48375500	-5.51454200	-1.09163700
C	2.39720700	-2.58247600	-2.18332400
O	2.48503800	-3.98461500	-1.97545600
C	-2.27448100	-2.68091800	1.17861200
C	-2.32386000	-2.64351800	2.57520000
C	-3.41353000	-2.79555600	0.38047100
C	-3.57918000	-2.69131200	3.17259900
C	-4.64956700	-2.82400000	1.03176600
C	-4.75306800	-2.77518600	2.41930900
H	-3.64313000	-2.65373500	4.25757600
H	-5.55239700	-2.89204000	0.42946300
H	1.54932700	-2.32572100	-2.83201600
C	-0.17317300	-0.31327300	0.85058300
O	-1.32301700	-0.09940700	1.38971700

C	1.05445800	0.14857300	1.67720100
C	0.94538300	1.66050300	1.54767000
H	0.97345200	-0.19991400	2.70948900
H	0.00972200	1.93936900	2.03803200
C	2.05623500	2.48458600	2.15456400
C	1.90328200	3.87429800	2.24869500
C	3.24013300	1.91452200	2.62642900
C	2.91662800	4.66998800	2.77162400
H	0.97899100	4.32998200	1.90174800
C	4.25747800	2.70930100	3.15517500
H	3.37152000	0.83640300	2.60194800
C	4.10201000	4.08972200	3.22331100
H	2.77988100	5.74518800	2.83248100
H	5.16927200	2.24456000	3.51761500
H	4.89257400	4.71008200	3.63305400
C	-6.09473000	-2.83431700	3.10258700
H	-6.25450400	-3.81676400	3.55818800
H	-6.16256500	-2.08991300	3.90024500
H	-6.90715200	-2.65663600	2.39498800
C	-1.08069000	-2.49602000	3.40550000
H	-0.26326200	-3.12254500	3.03514300
H	-0.76015300	-1.45108500	3.36052100
H	-1.27655700	-2.76496100	4.44489800
C	-3.34418300	-2.90013600	-1.12096800
H	-3.16039400	-3.93652600	-1.42197800
H	-4.29191600	-2.58018200	-1.55983800
H	-2.54217200	-2.28662300	-1.53797200
H	3.32907000	-2.27076700	-2.65522100
C	3.37252400	-2.25271600	0.14087100
H	3.13602600	-1.86932200	1.13740800
H	3.41326600	-3.34368800	0.21609200
C	4.70470400	-1.71787400	-0.32466200
C	5.52853200	-2.49279400	-1.14662900
C	5.12707000	-0.43644000	0.03744300
C	6.74691400	-1.99517400	-1.60080200
H	5.20667700	-3.49277300	-1.42878000
C	6.34538500	0.06434100	-0.41498800
H	4.50523200	0.17349500	0.68955400

C	7.15761400	-0.71452400	-1.23623600
H	7.37712400	-2.60930500	-2.23601300
H	6.66029400	1.06041000	-0.12079100
H	8.10805700	-0.32684200	-1.58810900
H	1.99549600	-0.20961000	1.24867900
C	0.74809100	1.89038300	0.00041600
C	2.10097300	2.08023000	-0.68910400
H	2.56951600	3.01653300	-0.37735000
H	2.77589300	1.26593300	-0.40633300
H	1.98319300	2.07172500	-1.77260600
C	-0.22610100	3.04490500	-0.24977900
C	0.08444400	4.12032600	-1.08161700
C	-1.48671100	3.01070400	0.36341300
C	-0.83700000	5.14935900	-1.28605900
H	1.04508400	4.16598600	-1.58307600
C	-2.40472100	4.03187300	0.15697000
H	-1.75446900	2.13965300	0.96006400
C	-2.08050200	5.11086500	-0.66613800
H	-0.57609900	5.98021100	-1.93426200
H	-3.38320300	3.97618400	0.62489900
H	-2.79806500	5.90910900	-0.82924500
N	0.17048900	0.56881600	-0.42474000
S	-0.59157000	0.40557400	-1.86821900
O	0.14252300	1.18683400	-2.86358800
O	-0.71747300	-1.04313300	-2.08651000
C	-2.24993500	1.05612200	-1.82584800
C	-2.59072500	2.07825000	-2.70216800
C	-3.17183500	0.52733700	-0.92391600
C	-3.88794600	2.58200800	-2.67231400
H	-1.84472000	2.48063000	-3.37828200
C	-4.46046300	1.04325000	-0.91315500
H	-2.85650200	-0.21139600	-0.19094200
C	-4.83801100	2.07326900	-1.78408400
H	-4.16236100	3.39150700	-3.34294300
H	-5.18456400	0.64835700	-0.20506300
C	-6.24493000	2.61075300	-1.76635600
H	-6.29028100	3.61670300	-2.18907500
H	-6.90922500	1.97115700	-2.35673000

H	-6.64085200	2.64332300	-0.74835300

M7$_D$(SS)

0 1

C	0.73876800	1.50578900	0.49797200
C	0.66190200	3.55750600	-0.26459900
C	-1.46400900	2.32823700	-0.53263300
H	-1.63205000	1.28350500	-0.79381900
N	1.87474700	3.40806600	0.17662700
N	1.90868600	2.13361500	0.65873700
N	-0.07698000	2.42870000	-0.05011000
C	0.13379700	4.72525200	-1.05265400
H	0.75906400	4.82305900	-1.95039800
H	0.21309400	5.64520700	-0.47056000
C	-1.51974700	3.21354100	-1.77762600
O	-1.22176200	4.54932400	-1.39943500
C	3.17167700	1.70717300	1.22226100
C	3.28621800	1.58001700	2.61030600
C	4.25006200	1.56329300	0.35139100
C	4.53613600	1.24630800	3.11879500
C	5.48156900	1.21294700	0.91434500
C	5.64305100	1.04808000	2.28686800
H	4.65000800	1.13245100	4.19428000
H	6.33425900	1.07591200	0.25368000
H	-0.81163300	2.84240400	-2.52998300
C	0.50124000	0.01556500	0.87858800
O	1.56620700	-0.49172600	1.39644800
C	-0.79196300	-0.20911100	1.70192100
C	-0.90124300	-1.72472300	1.59533800
H	-0.66817600	0.13544800	2.73161100
H	-0.01220100	-2.12009500	2.09306100
C	-2.11095300	-2.37017700	2.22193000
C	-1.98258000	-3.63508200	2.80325000
C	-3.37091000	-1.76455600	2.22458100
C	-3.08478400	-4.29287100	3.34438200
H	-1.00404400	-4.10877300	2.83027000
C	-4.47451800	-2.41350900	2.76973200
H	-3.49636900	-0.77448100	1.79704500

C	-4.33713800	-3.68450100	3.32494600
H	-2.96281500	-5.27645600	3.78693800
H	-5.44432400	-1.92596200	2.75920100
H	-5.19833700	-4.19201400	3.74737800
C	6.97869000	0.67661800	2.87748300
H	6.90429600	-0.24600200	3.46018600
H	7.72920900	0.52921500	2.09846800
H	7.33747800	1.45930600	3.55249600
C	2.09735400	1.74857400	3.51316200
H	1.49784200	2.62153700	3.23643200
H	1.46798500	0.85821600	3.42711700
H	2.41398200	1.86417600	4.55101800
C	4.12459800	1.79197400	-1.13221800
H	4.22179600	2.85835800	-1.36034800
H	4.91524700	1.25532500	-1.66097400
H	3.15959100	1.45579500	-1.51953500
H	-2.52871300	3.21676300	-2.19034400
C	-2.43854300	2.81907800	0.55147600
H	-2.30351300	2.22276200	1.45846900
H	-2.17739300	3.85200100	0.79807800
C	-3.86902800	2.74700900	0.07450800
C	-4.56189600	3.90598600	-0.28243600
C	-4.50723900	1.51120800	-0.05982400
C	-5.87244400	3.83207300	-0.75005300
H	-4.06603500	4.86938000	-0.19685100
C	-5.81550000	1.43308200	-0.52881300
H	-3.97544900	0.59709900	0.19349600
C	-6.50284600	2.59597200	-0.87238300
H	-6.40003500	4.74108200	-1.02032000
H	-6.29461000	0.46333200	-0.62373200
H	-7.52361300	2.53843800	-1.23572700
H	-1.66131400	0.27875500	1.25036500
C	-0.68201500	-2.01961500	0.04619200
C	0.27879000	-3.20749800	-0.08286800
H	0.40460300	-3.51262600	-1.12586800
H	1.24460200	-2.91644200	0.33625200
H	-0.09769300	-4.06336700	0.48285900
C	-2.01784800	-2.28933600	-0.65035700

C	-2.60089200	-3.56072000	-0.56682800
C	-2.74073800	-1.28996600	-1.30051700
C	-3.86650700	-3.81042100	-1.08834700
H	-2.07905000	-4.36939100	-0.06753800
C	-4.01062400	-1.53127900	-1.82211300
H	-2.29036700	-0.31665500	-1.43912100
C	-4.58490400	-2.79312900	-1.71223000
H	-4.29263900	-4.80488900	-1.00101700
H	-4.54266400	-0.72611600	-2.32031700
H	-5.57339200	-2.98564200	-2.11662500
N	-0.05064700	-0.73971300	-0.41805800
S	0.68509600	-0.65203900	-1.88487100
O	-0.17724200	-1.30388600	-2.86833900
O	0.99438500	0.77476900	-2.07264100
C	2.24848700	-1.52102300	-1.94733200
C	2.50765100	-2.29503200	-3.07449800
C	3.20634000	-1.34487600	-0.95105600
C	3.75565300	-2.89786400	-3.20549600
H	1.74041200	-2.42519400	-3.82940300
C	4.44647600	-1.95291100	-1.10499100
H	2.95464600	-0.78873600	-0.04854200
C	4.74268800	-2.73022500	-2.23121100
H	3.96449400	-3.50829200	-4.07926900
H	5.19771700	-1.82419600	-0.32952000
C	6.10196000	-3.35965900	-2.38873200
H	6.08588100	-4.15778300	-3.13349500
H	6.83538800	-2.61407100	-2.71214800
H	6.45453000	-3.77541500	-1.44163800

TS8$_D$(RR)

0 1

C	0.86350700	-0.54584800	0.70548700
C	2.40881400	-1.92790200	1.49698900
C	3.29498300	0.25866300	0.68844700
H	3.29645300	0.53968100	-0.36234900
N	1.28524400	-2.50346800	1.79364100
N	0.34086900	-1.62910300	1.31026100
N	2.20402900	-0.72305500	0.87602400

C	3.79195000	-2.46201400	1.71591300
H	3.81477700	-3.52233100	1.45500700
H	4.08411300	-2.34933400	2.76979400
C	4.63240500	-0.39022800	1.10522200
O	4.68782500	-1.78155000	0.86985800
C	-1.00655800	-1.80956200	1.78761400
C	-1.42484300	-0.96522400	2.82999300
C	-1.78372700	-2.88151500	1.34684600
C	-2.72835300	-1.10977100	3.29864300
C	-3.06737700	-3.01008300	1.88165200
C	-3.57503000	-2.11037000	2.81451900
H	-3.07883800	-0.44826300	4.08708700
H	-3.68861400	-3.83840500	1.54833200
H	5.43965400	0.06954000	0.53325200
C	0.23957400	0.93297800	-0.39020900
O	1.27087300	1.52865300	-0.76457600
C	-0.81043900	1.57702800	0.51165800
C	-1.74324800	2.22379900	-0.49694900
H	-0.34938600	2.29532300	1.19311800
H	-1.19251000	3.04174500	-0.97569100
C	-3.01640300	2.79220700	0.07764800
C	-3.50354300	4.01489000	-0.39151700
C	-3.74647600	2.11436000	1.06056300
C	-4.69818500	4.54377700	0.09433300
H	-2.93977600	4.55659200	-1.14726400
C	-4.93540800	2.64215400	1.55243900
H	-3.39972200	1.15229200	1.42995300
C	-5.41807300	3.85735200	1.06803600
H	-5.06197300	5.49336700	-0.28519900
H	-5.49005900	2.09988300	2.31208400
H	-6.34743700	4.26695200	1.45067800
C	-4.99813100	-2.22077100	3.29208600
H	-5.12553400	-1.76675800	4.27708300
H	-5.66756500	-1.70425900	2.59573300
H	-5.31855100	-3.26396600	3.34381000
C	-0.46801700	-0.01680400	3.50936300
H	0.36542300	-0.58087700	3.94291700
H	-0.03831600	0.72240200	2.83091200

H	-0.97411100	0.51629000	4.31557300
C	-1.29223600	-3.89670000	0.34946700
H	-0.20466200	-3.89826500	0.27356900
H	-1.61765200	-4.89536400	0.64992900
H	-1.71163800	-3.70776100	-0.64546100
H	4.81601600	-0.19833200	2.17420900
C	3.05392400	1.50180000	1.55847800
H	2.04758500	1.87046000	1.35774900
H	3.12475500	1.21794200	2.61540300
C	4.06004400	2.57740300	1.22310400
C	5.09641500	2.91614500	2.09434200
C	3.96503800	3.23398800	-0.00980500
C	6.02411300	3.89654200	1.74417700
H	5.17469100	2.41504200	3.05628800
C	4.89181800	4.21004900	-0.36017500
H	3.14627800	2.97141600	-0.67581100
C	5.92482300	4.54384200	0.51581600
H	6.82328600	4.15368000	2.43238200
H	4.80653400	4.71564500	-1.31712600
H	6.64644700	5.30716400	0.24229200
H	-1.34762300	0.81237700	1.07540000
C	-1.90848700	1.12368600	-1.61771500
C	-2.01334600	1.86350900	-2.96287300
H	-2.85060300	2.56394600	-2.90494700
H	-1.10210000	2.43123400	-3.15635000
H	-2.19229500	1.17417300	-3.78996100
C	-3.12772900	0.21575700	-1.44651700
C	-4.42060600	0.72314200	-1.64885500
C	-2.99637000	-1.13529900	-1.13792400
C	-5.53810700	-0.09420000	-1.53312400
H	-4.56664500	1.77338900	-1.87994500
C	-4.11497800	-1.96172800	-1.03606900
H	-2.00064200	-1.53739700	-0.99233600
C	-5.39093500	-1.44679500	-1.22913600
H	-6.52664400	0.32739800	-1.68630300
H	-3.98439100	-3.01627300	-0.81139500
H	-6.26163200	-2.09036800	-1.15203700
N	-0.63480500	0.32436700	-1.45116000

S	0.09166900	-0.20722800	-2.86420900
O	-0.92343600	-1.02313000	-3.53491900
O	0.72546000	0.85100300	-3.64851200
C	1.37888300	-1.28322000	-2.29866700
C	1.03631100	-2.55437900	-1.85591700
C	2.70670000	-0.88529600	-2.41992700
C	2.04890500	-3.44009400	-1.50073100
H	-0.00757600	-2.84597400	-1.81772700
C	3.70525600	-1.78630800	-2.07300700
H	2.93257800	0.11371200	-2.77733100
C	3.39316100	-3.07225200	-1.61443400
H	1.79402300	-4.43842300	-1.15472300
H	4.74816700	-1.49200800	-2.15438500
C	4.49573600	-4.04191600	-1.28377400
H	4.10363300	-4.94478200	-0.80995200
H	5.02464900	-4.34298100	-2.19334900
H	5.22307700	-3.57364400	-0.61480600

TS8$_D$(RS)

0 1

C	0.49846400	-2.12857700	-0.12898400
C	1.65349700	-3.41643900	-1.52514300
C	3.02262300	-1.99549300	0.01119200
H	3.03675000	-0.93333900	-0.24881700
N	0.41370600	-3.67331600	-1.81096900
N	-0.27610600	-2.87828700	-0.92741500
N	1.74735400	-2.52544000	-0.48879300
C	2.88170800	-3.91974200	-2.21862900
H	2.68064100	-4.03434900	-3.28438000
H	3.18738900	-4.89262300	-1.80690100
C	4.16645900	-2.74206100	-0.69004700
O	3.91184500	-2.96768600	-2.06190800
C	-1.70703600	-2.92380800	-0.92724500
C	-2.35282000	-3.48196300	0.18659300
C	-2.40400100	-2.45797500	-2.04323200
C	-3.74513600	-3.47350200	0.19713300
C	-3.80047400	-2.49631600	-1.99341000
C	-4.48528300	-2.96957100	-0.87753700

H	-4.26733300	-3.88845800	1.05681600
H	-4.36202000	-2.12116000	-2.84597500
H	5.07722200	-2.14556300	-0.61890900
C	0.18288200	-0.24326700	0.76121300
O	1.30960200	0.13602000	1.09368200
C	-0.94352900	-0.55691300	1.73603500
C	-1.74751800	0.73953500	1.70536300
H	-0.54343100	-0.80377700	2.72112600
H	-1.16544300	1.49973100	2.23137800
C	-3.12214500	0.71612300	2.33115600
C	-3.69852500	1.92848100	2.72675800
C	-3.86112500	-0.45861700	2.49138300
C	-4.98360100	1.96840100	3.25856300
H	-3.12957000	2.84814700	2.61002900
C	-5.14744000	-0.42166400	3.02601000
H	-3.44111800	-1.41467000	2.19113700
C	-5.71395600	0.79095000	3.40890700
H	-5.41365300	2.91843900	3.55998000
H	-5.70578300	-1.34569800	3.14262800
H	-6.71564200	0.81927700	3.82565800
C	-5.98935700	-2.92617800	-0.80852400
H	-6.39180000	-3.84283300	-0.36977000
H	-6.31552900	-2.09014500	-0.18060900
H	-6.42928600	-2.79310800	-1.79888000
C	-1.57186200	-4.11498000	1.31118000
H	-0.85487400	-4.84113000	0.91591900
H	-0.99859500	-3.38160600	1.88583800
H	-2.24713300	-4.63298200	1.99447700
C	-1.69862900	-1.94232100	-3.26839100
H	-0.71412500	-1.53551100	-3.03347900
H	-1.56090000	-2.75333900	-3.99114400
H	-2.28959400	-1.15637000	-3.74305000
H	4.34242800	-3.70562400	-0.18420600
C	3.13000500	-2.17080300	1.53137300
H	2.22819300	-1.75414100	1.98366200
H	3.17401100	-3.24217700	1.76006900
C	4.35226500	-1.46425400	2.06881900
C	5.51000900	-2.16798100	2.40752100

C	4.34522600	-0.07059100	2.19951100
C	6.64168100	-1.49675600	2.86770000
H	5.52333300	-3.25143200	2.31529200
C	5.47456900	0.60059200	2.65885900
H	3.43704700	0.47143000	1.94870000
C	6.62684200	-0.11018600	2.99299800
H	7.53288400	-2.05820300	3.12995000
H	5.45544100	1.68148500	2.76094000
H	7.50676000	0.41429400	3.35188400
H	-1.53878600	-1.38421600	1.35234800
C	-1.77006000	1.13379400	0.17483200
C	-2.99413500	0.54256000	-0.51665100
H	-2.89688400	0.62667600	-1.60070500
H	-3.07457600	-0.51621200	-0.25823300
H	-3.90779800	1.04188700	-0.18498500
C	-1.64916500	2.65262300	0.03207300
C	-2.52799600	3.39478000	-0.75483300
C	-0.62156500	3.32969000	0.70602000
C	-2.40012100	4.78191000	-0.85017200
H	-3.31900600	2.89733100	-1.30478600
C	-0.49415100	4.70887300	0.61685500
H	0.11525500	2.76043700	1.26841600
C	-1.39106500	5.44304900	-0.16000800
H	-3.09611200	5.34244800	-1.46632200
H	0.31709300	5.20961100	1.13648400
H	-1.29402300	6.52174800	-0.23390500
N	-0.53684000	0.43401600	-0.33080100
S	0.20672400	0.85010200	-1.75278300
O	1.16201200	-0.21915900	-2.03701700
O	-0.85903600	1.14054700	-2.70767200
C	1.14742900	2.35345500	-1.58149300
C	2.28030900	2.37849400	-0.77332400
C	0.73048500	3.47671000	-2.28866100
C	2.98926700	3.56868500	-0.65756500
H	2.57060400	1.48973600	-0.22355800
C	1.45881100	4.65308900	-2.16533700
H	-0.16061100	3.42575800	-2.90398900
C	2.58718800	4.72022100	-1.34241400

H	3.87122800	3.60398800	-0.02402400
H	1.13859600	5.53936400	-2.70650000
C	3.33215100	6.01908100	-1.18085700
H	4.31544800	5.86195900	-0.73336300
H	3.46438800	6.51884000	-2.14378200
H	2.77208600	6.70104400	-0.53231900

TS8$_D$(SR)

0 1

C	0.52177900	-2.02256900	-0.01234800
C	1.46487900	-3.20107800	-1.64817600
C	2.40204600	-0.92504500	-1.32519800
H	1.88480300	0.02764200	-1.19280100
N	0.58511400	-4.00840300	-1.13703500
N	0.02417700	-3.25911400	-0.12861900
N	1.47017400	-2.00346700	-0.98172400
C	2.31917100	-3.42982500	-2.86337000
H	1.66599700	-3.45650600	-3.74671900
H	2.84442900	-4.38353100	-2.78872500
C	2.72910200	-1.11963900	-2.80130800
O	3.28978300	-2.41101900	-2.99619500
C	-0.94694900	-3.87722800	0.72519100
C	-0.55663800	-4.25573000	2.00895600
C	-2.23424700	-4.09576400	0.22742600
C	-1.51215300	-4.87477600	2.81830200
C	-3.15022900	-4.72033500	1.06810400
C	-2.80725200	-5.11616200	2.36477200
H	-1.23132700	-5.18073300	3.82310900
H	-4.16096900	-4.89625400	0.70633300
H	1.81095100	-0.99813000	-3.39341500
C	-0.70656900	-0.54703600	0.80575400
O	-1.81877200	-1.06839400	0.81128500
C	0.08508100	-0.16419200	2.05497000
C	-0.14130800	1.34294200	2.12674700
H	-0.30053600	-0.70247400	2.92282200
H	-1.18102800	1.49780700	2.42620100
C	0.71989100	2.13603700	3.08160900
C	0.26188500	3.38450600	3.51851700

C	1.97215200	1.69547400	3.51676500
C	1.03705700	4.17499000	4.36142300
H	-0.71335400	3.73516500	3.18893300
C	2.74908900	2.48350700	4.36408700
H	2.35312100	0.72891500	3.19996700
C	2.28593600	3.72617600	4.78697200
H	0.66351900	5.13931000	4.69101400
H	3.71716200	2.12176300	4.69591600
H	2.89078700	4.33865000	5.44776400
C	-3.82329000	-5.80298900	3.24107300
H	-4.06788200	-6.79488800	2.84823500
H	-3.44998600	-5.92610400	4.25968700
H	-4.75462900	-5.23139700	3.28295800
C	0.84762100	-4.01650200	2.50108800
H	1.58376200	-4.38634200	1.78055500
H	1.04365700	-2.94926800	2.64410200
H	1.01086500	-4.52353100	3.45362500
C	-2.59896600	-3.64141800	-1.15835800
H	-2.06472400	-4.22499500	-1.91466100
H	-3.67173000	-3.75158800	-1.32941900
H	-2.31401400	-2.59315800	-1.29376900
H	3.46787500	-0.39092100	-3.13442300
C	3.62627400	-1.02872400	-0.39139600
H	3.28027600	-0.86389700	0.63659300
H	3.98993900	-2.06058200	-0.44608600
C	4.75313300	-0.08186900	-0.72951100
C	5.65498400	-0.41774300	-1.74616600
C	4.93176900	1.12456200	-0.04864500
C	6.69413000	0.44051600	-2.09016600
H	5.53151800	-1.36410800	-2.26808600
C	5.97315100	1.98628700	-0.39000700
H	4.26468800	1.38750600	0.76846800
C	6.85349000	1.64907000	-1.41422500
H	7.38315500	0.16334500	-2.88172600
H	6.09833000	2.91947500	0.14999200
H	7.66488000	2.31925600	-1.67917600
H	1.13824900	-0.41006400	1.90841000
C	-0.01322800	1.82430100	0.62869400

C	1.43395900	2.18129500	0.29822900
H	1.75675300	3.06853500	0.84787600
H	2.07922000	1.35092000	0.59886700
H	1.55669900	2.34723200	-0.77353400
C	-1.00033100	2.96188300	0.36184000
C	-0.60503300	4.17189200	-0.20517100
C	-2.35306800	2.78753700	0.68878200
C	-1.53335700	5.19201700	-0.42502600
H	0.43022400	4.33351500	-0.48426400
C	-3.27791000	3.79883600	0.47178200
H	-2.69084200	1.82820100	1.07399900
C	-2.86791100	5.01202100	-0.08332800
H	-1.20384700	6.12825700	-0.86444500
H	-4.32220900	3.63461200	0.71949400
H	-3.58919300	5.80492100	-0.25454000
N	-0.40170700	0.57754900	-0.11067600
S	-0.83646100	0.57495900	-1.70938900
O	0.06694600	1.50188300	-2.39077700
O	-0.86342800	-0.82240500	-2.13275700
C	-2.48209700	1.21291100	-1.89643400
C	-2.67090800	2.39681600	-2.59909900
C	-3.54589500	0.50470400	-1.34389400
C	-3.96363600	2.88875800	-2.73714100
H	-1.81602400	2.92200800	-3.01026100
C	-4.82845000	1.01928500	-1.48847900
H	-3.35271200	-0.40530000	-0.78181600
C	-5.05409800	2.21610700	-2.17844700
H	-4.12796100	3.81792000	-3.27575600
H	-5.67071800	0.48659500	-1.05601800
C	-6.44111500	2.79256900	-2.28743900
H	-6.59271000	3.27660700	-3.25504500
H	-7.20361900	2.02153100	-2.16027200
H	-6.59966300	3.55000800	-1.51231800

TS8$_D$(SS)

0 1

C	0.43335800	1.75139200	0.25164700
C	-0.02330400	3.70986300	-0.70912500

C	-1.96439300	2.18532800	-0.47010300
H	-2.01224700	1.13476500	-0.75157500
N	1.22391000	3.80296000	-0.37090100
N	1.48075600	2.59399000	0.23254000
N	-0.54288100	2.50069300	-0.32976800
C	-0.86522900	4.61566100	-1.54923300
H	-0.23184800	5.22619200	-2.19267000
H	-1.49469400	5.27381700	-0.93318100
C	-2.59999200	3.06205500	-1.59350200
O	-1.66106800	3.77772900	-2.36621900
C	2.79628900	2.38983200	0.77115500
C	2.92724000	2.18840800	2.15280300
C	3.90225600	2.47261500	-0.08001000
C	4.20556200	1.97741100	2.65934700
C	5.16391700	2.24860300	0.48126300
C	5.33497300	1.98376800	1.83683400
H	4.32494700	1.81013600	3.72752400
H	6.03470600	2.28740300	-0.16910800
H	-3.15419700	2.42194300	-2.28229500
C	0.63143400	-0.22948300	0.75814300
O	1.74440400	-0.32227200	1.27809700
C	-0.66227300	-0.51354700	1.50907900
C	-0.85175600	-1.98731200	1.17375300
H	-0.54821500	-0.31231300	2.57571100
H	0.01743800	-2.52201700	1.57760600
C	-2.08899200	-2.64485500	1.73049500
C	-2.08011200	-4.01868400	1.99676000
C	-3.26202500	-1.92767900	1.97920600
C	-3.22719900	-4.66791200	2.44545200
H	-1.16321900	-4.58378600	1.84836400
C	-4.41132800	-2.57209400	2.42827500
H	-3.29228200	-0.85574700	1.81446200
C	-4.40146800	-3.94684700	2.65195200
H	-3.20161300	-5.73538700	2.63988200
H	-5.31493000	-1.99524800	2.60119200
H	-5.29749000	-4.45097800	2.99957500
C	6.70241100	1.73006000	2.41757400
H	6.76421400	0.72450900	2.84486200

H	7.47956100	1.82474800	1.65637700
H	6.92372500	2.43803800	3.22172300
C	1.73890800	2.20172700	3.07640700
H	1.01783200	2.97441400	2.79462500
H	1.23413000	1.23375900	3.03852100
H	2.06150600	2.38808500	4.10280600
C	3.79935500	2.83261200	-1.53948100
H	3.88678900	3.91678900	-1.66298600
H	4.61357800	2.36137300	-2.09516400
H	2.85472300	2.51420100	-1.97369900
H	-3.30891100	3.76580800	-1.13236500
C	-2.71067100	2.41383500	0.84942300
H	-2.19922300	1.87927200	1.65823000
H	-2.65987300	3.47974200	1.10232200
C	-4.15917300	1.97785000	0.75617400
C	-5.17211300	2.78304500	1.28336800
C	-4.51506200	0.76368400	0.15656200
C	-6.50445700	2.38069400	1.23311500
H	-4.91190900	3.73376900	1.74149800
C	-5.84656600	0.35752100	0.10838700
H	-3.76000300	0.11018900	-0.27611600
C	-6.84596500	1.16378100	0.64738100
H	-7.27555300	3.02096100	1.64956700
H	-6.09268800	-0.59136000	-0.35924600
H	-7.88386600	0.84950400	0.60677000
H	-1.49316800	0.06667100	1.10520200
C	-0.63440000	-2.02591900	-0.39269700
C	-0.11847500	-3.41863800	-0.77667900
H	0.02107400	-3.51093000	-1.85341400
H	0.82302900	-3.63343400	-0.26205000
H	-0.85750400	-4.15976000	-0.46026500
C	-1.87746500	-1.70965400	-1.23827500
C	-3.02451900	-2.51409900	-1.15585400
C	-1.87219900	-0.68248100	-2.18771600
C	-4.14183000	-2.25665200	-1.94647800
H	-3.05744800	-3.35372700	-0.47045100
C	-2.98528100	-0.43129300	-2.98979500
H	-0.98769300	-0.06656200	-2.30501500

C	-4.13279400	-1.20629200	-2.86172200
H	-5.01941100	-2.88864400	-1.84969300
H	-2.94312600	0.36752200	-3.72423100
H	-5.00340700	-1.00635900	-3.47804100
N	0.37893500	-0.93012000	-0.53985500
S	1.58485500	-0.93531600	-1.67890300
O	1.04978300	-1.63330700	-2.84563000
O	2.00515900	0.45517600	-1.80885200
C	3.01577000	-1.85090600	-1.13697900
C	3.21013500	-3.14108700	-1.62120600
C	3.96348300	-1.22896200	-0.32672800
C	4.36331400	-3.83031600	-1.26093000
H	2.47495200	-3.58812000	-2.28156000
C	5.11196300	-1.93208800	0.01453300
H	3.78705900	-0.22299400	0.03793400
C	5.32800100	-3.23740700	-0.44151600
H	4.52101000	-4.83922700	-1.63123100
H	5.85694300	-1.45647100	0.64720200
C	6.59193000	-3.97588300	-0.08761200
H	6.41433800	-5.05121200	-0.01516200
H	7.35550500	-3.81824000	-0.85628900
H	7.00098500	-3.62523900	0.86233900

SP$_D$(RR)

0 1

C	-0.75903300	-0.15935500	0.42201400
C	-1.29551400	-1.32757000	-0.49570900
C	-0.82339200	-0.95342500	-1.89782200
C	0.49394900	-0.25465100	-1.64980100
H	-0.72141200	-2.20899100	-0.18599800
H	-0.69469200	-1.80399100	-2.56752900
H	-1.48924700	-0.23023100	-2.38271800
O	1.39353800	-0.05466600	-2.43382500
C	-2.75980400	-1.65377800	-0.33682300
C	-3.13441100	-2.75089800	0.44360900
C	-3.76412900	-0.88564800	-0.93642900
C	-4.47674700	-3.07504300	0.62841400
H	-2.36312300	-3.36258500	0.90546800

C	-5.10501600	-1.20835200	-0.75684700
H	-3.50249700	-0.01593200	-1.53295000
C	-5.46608500	-2.30355100	0.02674300
H	-4.74594700	-3.93301000	1.23593600
H	-5.87102800	-0.60088000	-1.22809800
H	-6.51311000	-2.55355700	0.16447700
C	-1.69564500	1.04543500	0.42545100
C	-2.74142100	1.12072700	1.35162800
C	-1.58927100	2.05059700	-0.54110500
C	-3.65782800	2.16671300	1.30841000
H	-2.85863600	0.34783300	2.10443200
C	-2.50792500	3.09723400	-0.58573300
H	-0.77455600	2.04286900	-1.25845400
C	-3.54762100	3.15810100	0.33622400
H	-4.46238000	2.20384300	2.03590100
H	-2.40135300	3.86827300	-1.34178900
H	-4.26238700	3.97385800	0.30271500
C	-0.48804200	-0.71788400	1.81899700
H	0.36265300	-1.40343800	1.77298300
H	-0.27827700	0.07069600	2.53997900
H	-1.35988200	-1.28681400	2.15442300
S	1.66000300	1.27599900	0.21460500
O	1.29118600	1.58907600	1.58643000
O	1.75810800	2.34064600	-0.76991400
C	3.16676200	0.35085200	0.21445800
C	3.50087900	-0.37773300	1.35302100
C	3.99381900	0.38958200	-0.90448700
C	4.69327500	-1.09118300	1.35953800
H	2.84899300	-0.36763000	2.22065200
C	5.18260700	-0.32838300	-0.87368300
H	3.69897900	0.96678500	-1.77266500
C	5.54555600	-1.07931300	0.25005300
H	4.97183100	-1.65884500	2.24217200
H	5.84257700	-0.30443100	-1.73570000
C	6.82333500	-1.87519400	0.25351700
H	7.61334400	-1.35359900	-0.29118700
H	7.16856300	-2.06618600	1.27151800
H	6.66967700	-2.84306700	-0.23445000

N	0.49848800	0.15412900	-0.31373900

SP_D(RS)

0 1

C	1.10282900	-0.31604300	0.46221400
C	1.82827800	-0.74277900	-0.87022100
C	1.34598400	-2.16916800	-1.12258000
C	-0.06849300	-2.16230800	-0.58471700
H	1.39201900	-0.11861100	-1.65701400
H	1.36167700	-2.46517300	-2.17174100
H	1.90058300	-2.91889300	-0.54777400
O	-0.96996700	-2.92258500	-0.85430000
C	3.31488300	-0.48267700	-0.85431000
C	3.77180100	0.79877500	-1.18200100
C	4.25018000	-1.45295300	-0.48674300
C	5.12843300	1.10527900	-1.13938400
H	3.05250000	1.55991300	-1.47492900
C	5.60922500	-1.14906100	-0.44584700
H	3.92803600	-2.45691000	-0.22704900
C	6.05242000	0.13033300	-0.77015500
H	5.46353000	2.10400500	-1.40029100
H	6.32193200	-1.91648500	-0.16102900
H	7.11155800	0.36470500	-0.74051800
S	-1.46305900	-0.99978800	1.40297100
O	-1.09761200	0.04860700	2.34304400
O	-1.74654000	-2.34055600	1.88509200
C	-2.82134000	-0.43806400	0.41934900
C	-3.11842300	0.92418400	0.41330700
C	-3.56936100	-1.35906600	-0.30742700
C	-4.18537500	1.36692300	-0.35610200
H	-2.52249100	1.61145700	1.00413000
C	-4.63412600	-0.89069700	-1.07034200
H	-3.30850800	-2.41077500	-0.28010500
C	-4.95383900	0.46940800	-1.10816600
H	-4.43257200	2.42464700	-0.37016000
H	-5.22894700	-1.59464000	-1.64431900
C	-6.10489300	0.97363100	-1.93720400
H	-6.62158500	0.15468900	-2.44030900

H	-6.82793400	1.50517900	-1.31214000
H	-5.75300600	1.67676300	-2.69775400
N	-0.16091600	-1.09812800	0.31406900
C	0.83053500	1.18323900	0.43696600
C	1.48525300	2.06864800	1.29186000
C	-0.05439300	1.70086500	-0.51594800
C	1.25982800	3.44167900	1.19747300
H	2.17523400	1.69867600	2.04163500
C	-0.28632500	3.06851600	-0.60663400
H	-0.58157000	1.02326000	-1.18374900
C	0.37411000	3.94660600	0.25124700
H	1.77893100	4.11542700	1.87158800
H	-0.98621700	3.44731200	-1.34498200
H	0.19745500	5.01500400	0.18222700
C	1.85812100	-0.79251800	1.70176400
H	1.98565600	-1.87834000	1.66317700
H	2.85032200	-0.34051100	1.75171600
H	1.29983900	-0.53746000	2.60427300

SP_D(SR)

0 1

C	-1.10282900	-0.31604300	0.46221400
C	-1.82827800	-0.74277900	-0.87022100
C	-1.34598400	-2.16916800	-1.12258000
C	0.06849300	-2.16230800	-0.58471700
H	-1.39201900	-0.11861100	-1.65701400
H	-1.36167700	-2.46517300	-2.17174100
H	-1.90058300	-2.91889300	-0.54777400
O	0.96996700	-2.92258500	-0.85430000
C	-3.31488300	-0.48267700	-0.85431000
C	-3.77180100	0.79877500	-1.18200100
C	-4.25018000	-1.45295300	-0.48674300
C	-5.12843300	1.10527900	-1.13938400
H	-3.05250000	1.55991300	-1.47492900
C	-5.60922500	-1.14906100	-0.44584700
H	-3.92803600	-2.45691000	-0.22704900
C	-6.05242000	0.13033300	-0.77015500
H	-5.46353000	2.10400500	-1.40029100

H	-6.32193200	-1.91648500	-0.16102900
H	-7.11155800	0.36470500	-0.74051800
S	1.46305900	-0.99978800	1.40297100
O	1.09761200	0.04860700	2.34304400
O	1.74654000	-2.34055600	1.88509200
C	2.82134000	-0.43806400	0.41934900
C	3.11842300	0.92418400	0.41330700
C	3.56936100	-1.35906600	-0.30742700
C	4.18537500	1.36692300	-0.35610200
H	2.52249100	1.61145700	1.00413000
C	4.63412600	-0.89069700	-1.07034200
H	3.30850800	-2.41077500	-0.28010500
C	4.95383900	0.46940800	-1.10816600
H	4.43257200	2.42464700	-0.37016000
H	5.22894700	-1.59464000	-1.64431900
C	6.10489300	0.97363100	-1.93720400
H	6.62158500	0.15468900	-2.44030900
H	6.82793400	1.50517900	-1.31214000
H	5.75300600	1.67676300	-2.69775400
N	0.16091600	-1.09812800	0.31406900
C	-0.83053500	1.18323900	0.43696600
C	-1.48525300	2.06864800	1.29186000
C	0.05439300	1.70086500	-0.51594800
C	-1.25982800	3.44167900	1.19747300
H	-2.17523400	1.69867600	2.04163500
C	0.28632500	3.06851600	-0.60663400
H	0.58157000	1.02326000	-1.18374900
C	-0.37411000	3.94660600	0.25124700
H	-1.77893100	4.11542700	1.87158800
H	0.98621700	3.44731200	-1.34498200
H	-0.19745500	5.01500400	0.18222700
C	-1.85812100	-0.79251800	1.70176400
H	-1.98565600	-1.87834000	1.66317700
H	-2.85032200	-0.34051100	1.75171600
H	-1.29983900	-0.53746000	2.60427300

SP_D(SS)

0 1

C	-0.75903100	-0.15924800	-0.42209400
C	-1.29558200	-1.32748900	0.49560300
C	-0.82339800	-0.95344000	1.89773000
C	0.49396200	-0.25465200	1.64970000
H	-0.72149500	-2.20890500	0.18581700
H	-0.69480500	-1.80400300	2.56746500
H	-1.48921700	-0.23018700	2.38252800
O	1.39352400	-0.05461200	2.43373500
C	-2.75988500	-1.65374500	0.33675400
C	-3.13440400	-2.75105700	-0.44345700
C	-3.76429400	-0.88557000	0.93615000
C	-4.47670600	-3.07535500	-0.62822600
H	-2.36306400	-3.36276600	-0.90520700
C	-5.10516000	-1.20841800	0.75659400
H	-3.50277400	-0.01577700	1.53255700
C	-5.46612000	-2.30382200	-0.02674400
H	-4.74583000	-3.93346500	-1.23558200
H	-5.87125200	-0.60091800	1.22768700
H	-6.51312000	-2.55397500	-0.16442000
C	-1.69568000	1.04554500	-0.42550700
C	-2.74174500	1.12066000	-1.35135900
C	-1.58897900	2.05091100	0.54079100
C	-3.65813100	2.16668700	-1.30806000
H	-2.85919100	0.34765200	-2.10400800
C	-2.50762100	3.09752300	0.58554600
H	-0.77405300	2.04328800	1.25788600
C	-3.54760100	3.15825200	-0.33609200
H	-4.46290300	2.20367400	-2.03531100
H	-2.40084700	3.86864800	1.34149200
H	-4.26237300	3.97400100	-0.30245000
C	-0.48807500	-0.71773000	-1.81911100
H	0.36256100	-1.40335400	-1.77308400
H	-0.27822600	0.07088500	-2.54001200
H	-1.35992900	-1.28655900	-2.15466100
S	1.66010900	1.27608100	-0.21452200
O	1.29137000	1.58945300	-1.58627500
O	1.75841100	2.34057300	0.77012400
C	3.16674400	0.35074900	-0.21446300

C	3.50086000	-0.37780000	-1.35306500
C	3.99370100	0.38920600	0.90456100
C	4.69320600	-1.09133400	-1.35956400
H	2.84906600	-0.36754700	-2.22076200
C	5.18250500	-0.32871300	0.87372700
H	3.69881000	0.96627700	1.77281800
C	5.54549900	-1.07951700	-0.25007400
H	4.97176300	-1.65895400	-2.24222300
H	5.84245900	-0.30482700	1.73575700
C	6.82337000	-1.87521200	-0.25346700
H	7.61318800	-1.35329500	0.29130000
H	7.16899400	-2.06598000	-1.27135400
H	6.67006400	-2.84310500	0.23455400
N	0.49848100	0.15428500	0.31366600

R1^{HOBt}

0 1

C	1.51001500	-1.30264700	1.01773300
O	1.00378100	-1.12768600	2.10357800
C	2.97926900	-1.23537500	0.72547400
C	3.30577400	0.04919000	-0.06799800
H	3.27076400	-2.11234900	0.14044000
H	3.51179600	-1.24393300	1.67771800
H	2.74740100	0.03582900	-1.00904400
H	2.96050100	0.91751900	0.50133800
C	4.78763100	0.14438200	-0.33413000
C	5.34711000	-0.46555800	-1.45969700
C	5.63081100	0.80311200	0.56355000
C	6.71985200	-0.41552200	-1.68668400
H	4.69848800	-0.97906200	-2.16557400
C	7.00443800	0.85554800	0.34013700
H	5.20430100	1.28159400	1.44179200
C	7.55235100	0.24580100	-0.78616900
H	7.13947400	-0.88874300	-2.56884400
H	7.64647600	1.37594500	1.04389000
H	8.62218700	0.28844200	-0.96318300
O	0.81279800	-1.54455700	-0.11412700
C	-0.56768000	-1.50749700	-0.17356800

C	-1.07718600	-1.04644400	-1.38480900
C	-1.40290100	-1.95384300	0.85306500
C	-2.45006000	-0.98343200	-1.57182400
H	-0.38798100	-0.71683600	-2.15348300
C	-2.77738900	-1.87193600	0.67306800
H	-0.99465100	-2.35127500	1.77146400
C	-3.27393200	-1.37909800	-0.52735100
H	-2.87949000	-0.60385300	-2.48997800
H	-3.46140400	-2.18011700	1.45363100
N	-4.72091700	-1.21878400	-0.67746800
O	-5.42434800	-1.46046600	0.28877300
O	-5.14344600	-0.83270900	-1.75335100
C	-2.04165700	1.53590100	0.77064700
C	-1.58936500	1.99256400	-0.47529100
C	-2.51641600	2.36063800	-1.46893900
C	-3.85291300	2.25261500	-1.15134300
C	-4.28743600	1.80449600	0.12612800
C	-3.39937100	1.43797000	1.11433700
H	-2.18078300	2.70186900	-2.44192400
H	-4.60235000	2.51481700	-1.89025300
H	-5.35316100	1.73501400	0.31957800
H	-3.71740100	1.07969500	2.08626900
N	-0.21616100	1.93541300	-0.49424800
N	0.18601900	1.50068100	0.65788300
N	-0.88350200	1.24992500	1.41166400
O	-0.76263900	0.83610900	2.69401400
H	-0.27706300	-0.01592900	2.63222300

TS1^{HOBt}

0 1

C	1.22103300	1.64204700	-0.08790400
C	2.49936200	3.45897900	-0.16024100
C	3.45533900	1.44902800	-1.30667800
H	3.92310700	0.73665200	-0.62091300
N	1.43984500	3.83294600	0.48921500
N	0.66632100	2.69910300	0.52308600
N	2.39768200	2.14725300	-0.55137100
C	3.73198400	4.26280800	-0.44915800

H	3.97648400	4.88206800	0.41453200
H	3.56200400	4.91459200	-1.31774000
C	4.49697800	2.49031800	-1.73068800
O	4.81082700	3.38462300	-0.68274200
C	-0.67325200	2.82754300	1.03655900
C	-1.64953700	3.28557100	0.14126100
C	-0.95050400	2.53025500	2.36833800
C	-2.95488500	3.38734400	0.60732800
C	-2.27501800	2.66796300	2.79430500
C	-3.28737500	3.07281000	1.92827100
H	-3.73555300	3.71257500	-0.07685900
H	-2.51789300	2.42810300	3.82689300
H	5.41544600	1.97415600	-2.01534500
C	0.88993000	-0.27208200	0.19158100
O	0.89803300	-0.93425400	-0.85439000
C	1.97037000	-0.49395700	1.24781400
C	1.87639100	-1.92444600	1.79872100
H	1.88754000	0.23431500	2.05919100
H	2.94507400	-0.36412800	0.76926600
H	0.89181700	-2.06716700	2.25714200
H	1.95565300	-2.62299400	0.95913700
C	2.97159500	-2.19213700	2.79955900
C	2.76762700	-1.96997700	4.16358000
C	4.23258300	-2.61657100	2.37058700
C	3.79672100	-2.17289800	5.08001100
H	1.79117500	-1.63738500	4.50813300
C	5.26439900	-2.82048200	3.28275600
H	4.40275500	-2.78912700	1.30968500
C	5.04839900	-2.59973700	4.64182700
H	3.62014600	-2.00040700	6.13715700
H	6.23599600	-3.15556100	2.93332800
H	5.84985500	-2.76099900	5.35552900
C	0.10575200	2.02269300	3.31153900
H	0.12843100	0.92735500	3.28779000
H	1.10004600	2.38839800	3.04356900
H	-0.11313700	2.33414100	4.33495700
C	-4.72295800	3.14992500	2.37618800
H	-4.84067300	2.80006200	3.40365400

H	-5.09904700	4.17569300	2.31803200
H	-5.35449300	2.53449000	1.72670900
C	-1.29142900	3.65818600	-1.27258000
H	-0.64486200	4.54140800	-1.28702700
H	-0.75330300	2.84897900	-1.77549900
H	-2.19136200	3.87945300	-1.84882300
O	-0.34232100	-0.14900900	0.91962400
C	-1.52096300	-0.03180200	0.27688400
C	-2.65370700	-0.15731300	1.09271000
C	-1.65753700	0.25272000	-1.09242900
C	-3.91870200	0.02069800	0.56666300
H	-2.51046500	-0.38859200	2.14210500
C	-2.92547700	0.40792000	-1.62754400
H	-0.78160000	0.31720600	-1.72308600
C	-4.03527100	0.29697300	-0.79273500
H	-4.80796000	-0.08175400	1.17782200
H	-3.05776600	0.63181200	-2.68011300
N	-5.35867800	0.42541400	-1.36212300
O	-5.49183800	0.20682600	-2.57054800
O	-6.28259800	0.73592900	-0.63773200
H	4.12413900	3.05018100	-2.60238100
C	2.89761100	0.70626100	-2.52981900
H	1.94740300	0.24342300	-2.25365400
H	2.71730400	1.42877000	-3.33387600
C	3.86106200	-0.37160800	-2.96910100
C	4.81039200	-0.15427000	-3.96955700
C	3.82812700	-1.61104100	-2.31997400
C	5.71657400	-1.15598300	-4.31429000
H	4.83739900	0.80226000	-4.48690400
C	4.73240200	-2.61098500	-2.66339300
H	3.06675000	-1.77879700	-1.56010800
C	5.68112100	-2.38469600	-3.66030500
H	6.44877300	-0.97658000	-5.09523300
H	4.69339900	-3.57139500	-2.15831600
H	6.38556500	-3.16520900	-3.92956500
C	-3.20202700	-2.93809800	-0.99901100
C	-3.83326100	-3.24795800	0.21385200
C	-3.06931000	-3.63847100	1.32936900

C	-1.70186400	-3.69122100	1.16480900
C	-1.08327800	-3.36227600	-0.07083700
C	-1.81163200	-2.98444300	-1.17752500
H	-3.54818200	-3.87653100	2.27275300
H	-1.07324200	-3.98482600	1.99923200
H	-0.00150900	-3.37668000	-0.14256900
H	-1.34215100	-2.70092300	-2.11149900
N	-5.18705000	-3.04999500	0.07170500
N	-5.41482800	-2.66480300	-1.14294100
N	-4.25098900	-2.59254800	-1.78700700
O	-4.19915300	-2.22935100	-3.09139500
H	-4.54935500	-1.31228000	-3.10804800

M1[HOBt]

0 1

C	2.19688200	-1.63575000	-0.39887600
C	3.92317800	-2.93389200	-0.76157800
C	4.50858000	-0.53189200	-0.30067700
H	4.30853900	0.21544400	-1.07232100
N	2.89885300	-3.73172100	-0.76538200
N	1.83353700	-2.91144600	-0.52661700
N	3.54351700	-1.63814500	-0.50787500
C	5.35440900	-3.25935200	-1.06766700
H	5.39618200	-3.98792900	-1.87795300
H	5.84896800	-3.67990400	-0.18130600
C	5.93045700	-1.09609600	-0.48442600
O	5.99556900	-2.07914300	-1.49467100
C	0.54449400	-3.50865200	-0.27157100
C	0.22449700	-3.78284900	1.05802900
C	-0.29416600	-3.80652800	-1.34566300
C	-1.02118400	-4.36116000	1.30406700
C	-1.53080900	-4.37139500	-1.04512300
C	-1.91368000	-4.64497400	0.27104200
H	-1.30246400	-4.58088000	2.33109200
H	-2.21555800	-4.59630400	-1.85907200
H	6.59498400	-0.27992700	-0.76982800
C	1.25524800	-0.43022900	-0.06334700
O	1.09309600	-0.30260800	1.19548600

C	1.64217200	0.84693200	-0.84127500
C	0.51957700	1.88448700	-0.70143900
H	1.85143800	0.63508100	-1.89610700
H	2.54582600	1.26102900	-0.38804100
H	-0.28910400	1.65204700	-1.40123900
H	0.11403400	1.78661500	0.31331300
C	1.01700300	3.29170000	-0.92103300
C	0.68523500	4.01901200	-2.06545600
C	1.86112500	3.88396100	0.02631800
C	1.18848200	5.30485600	-2.26442200
H	0.02749600	3.57312700	-2.80783600
C	2.37110600	5.16284900	-0.17053500
H	2.11951600	3.32899000	0.92671900
C	2.03559600	5.87815200	-1.32064600
H	0.91913300	5.85672500	-3.15956500
H	3.02611200	5.60375200	0.57500900
H	2.43041100	6.87702500	-1.47591700
C	-3.28238800	-5.19703700	0.57182100
H	-3.65761100	-5.80299300	-0.25584100
H	-3.27344700	-5.80914600	1.47640100
H	-3.99240700	-4.37851600	0.73248900
C	0.11555000	-3.47875500	-2.75398200
H	0.24239500	-2.39640300	-2.85892400
H	1.06832200	-3.95314700	-3.00813400
H	-0.64013200	-3.81665200	-3.46468600
C	1.17949100	-3.44341100	2.17099700
H	2.15215600	-3.92152800	2.01280100
H	1.33447500	-2.35848300	2.20867000
H	0.78462300	-3.78168900	3.13024100
O	0.02929600	-0.88151400	-0.91391300
C	-1.22556800	-0.85881600	-0.46979600
C	-2.21270000	-0.70598000	-1.46706300
C	-1.61585000	-1.02518300	0.87579800
C	-3.55320300	-0.70795200	-1.14420300
H	-1.88621700	-0.57796700	-2.49348000
C	-2.95929100	-1.00984700	1.20140800
H	-0.85832700	-1.15041500	1.63398500
C	-3.91328600	-0.85603100	0.19568000

H	-4.31872500	-0.57389300	-1.89880100
H	-3.28207300	-1.12009100	2.22972000
N	-5.30571900	-0.78446900	0.54729300
O	-5.60751700	-0.75292200	1.74607100
O	-6.14236900	-0.71806200	-0.33795200
H	6.28431400	-1.51577600	0.46952700
C	4.36192000	0.05536400	1.11092900
H	3.32193400	0.33978400	1.29993800
H	4.59868600	-0.73869300	1.82838700
C	5.29774600	1.22660500	1.31193500
C	6.35371500	1.14616300	2.22179400
C	5.12430400	2.41140000	0.58755300
C	7.21297600	2.22622600	2.41379900
H	6.49787500	0.23180500	2.79159600
C	5.98003100	3.49211000	0.77785900
H	4.30738200	2.50311600	-0.12479300
C	7.02763600	3.40248000	1.69304700
H	8.02518100	2.14716600	3.12926600
H	5.82359000	4.40588900	0.21291400
H	7.69444100	4.24519400	1.84366500
C	-5.38090000	2.19905200	0.71479500
C	-5.24409600	2.59106400	-0.62285400
C	-3.99048700	2.99576000	-1.11640900
C	-2.93001800	2.96901200	-0.23567100
C	-3.08995300	2.55426500	1.11312200
C	-4.31193500	2.16673900	1.62212800
H	-3.87550600	3.31063100	-2.14773500
H	-1.94572300	3.28136000	-0.57112800
H	-2.21916900	2.54256800	1.76083500
H	-4.44470000	1.83829500	2.64621900
N	-6.47082700	2.48474700	-1.24060700
N	-7.32380400	2.04563600	-0.37649600
N	-6.70024300	1.88223500	0.78954800
O	-7.36651400	1.37810700	1.85466300
H	-7.10791900	0.43153500	1.87296600

TS6^{CO}

0 1

C	5.12943700	1.60698100	2.65324800
C	3.93763700	1.22053600	2.05159200
C	2.82713500	0.86823600	2.83344300
C	2.93939600	0.93764100	4.22778000
C	4.13296000	1.32344300	4.82975800
C	5.23314400	1.65758600	4.04321400
H	5.97752200	1.87810500	2.03198500
H	3.86749300	1.20869800	0.96605200
H	2.07968500	0.67979000	4.84039300
H	4.20304400	1.36735800	5.91193500
H	6.16378600	1.96522800	4.50935200
C	1.55144200	0.44609500	2.23425100
H	0.66116200	0.48678300	2.86105800
C	1.39357500	0.02460000	0.97825600
H	2.24398100	-0.04420500	0.30546700
C	0.00875800	-0.30056800	0.45183300
O	-0.97855000	-0.19654600	1.22894100
C	1.99070200	-1.45996600	-1.92126700
C	1.99509900	-1.80570800	-3.44531500
H	1.84950100	-0.38465200	-1.80110500
H	1.69460700	-0.92898900	-4.02134100
O	1.09722800	-2.83004300	-3.83034500
N	0.82769700	-2.08498700	-1.29460000
C	1.00243900	-3.89950000	-2.91098900
C	0.38751600	-3.31509900	-1.68171900
H	0.34490500	-4.65700000	-3.33624400
H	1.98240900	-4.34928300	-2.70108700
N	-0.62598200	-3.69707800	-0.96421400
N	-0.83424200	-2.65886300	-0.10072600
C	0.01889700	-1.65285100	-0.31347900
C	-1.87084500	-2.82365600	0.89168600
C	-3.19524300	-2.88866300	0.46175300
C	-1.48808700	-3.01652800	2.22356300
C	-4.17227700	-3.09282700	1.44114800
C	-2.49888300	-3.22710800	3.15453900
C	-3.84693600	-3.25511300	2.78485800
H	-5.21440100	-3.13275800	1.13361000
H	-2.22827100	-3.37648300	4.19711600

C	-0.04263700	-2.99219400	2.63584000
H	0.57760800	-3.59020400	1.95992100
H	0.33888700	-1.96721500	2.62191800
H	0.07280600	-3.39038500	3.64531600
C	-3.57853800	-2.78279700	-0.99104700
H	-2.93655500	-2.08724800	-1.53597400
H	-3.49294000	-3.76141300	-1.47462400
H	-4.61463500	-2.44883400	-1.08100700
C	-4.91546000	-3.45880000	3.82761500
H	-5.89293300	-3.61821900	3.36821300
H	-4.68594000	-4.32142500	4.45913100
H	-4.98726800	-2.58513100	4.48275100
C	-5.12914000	2.31905600	-0.43101500
C	-4.45951100	2.99035100	-1.45840900
C	-3.26270900	2.50309200	-1.97098700
C	-2.73619500	1.32758100	-1.44641700
C	-3.38336300	0.63078400	-0.43031400
C	-4.58011000	1.13287100	0.06712700
H	-4.87654600	3.91142900	-1.85622300
H	-2.73094500	3.02562500	-2.75822800
H	-2.92646500	-0.25270400	0.00439000
H	-5.09039100	0.59915900	0.86458600
C	-6.39481200	2.88850400	0.15396000
H	-6.99393300	2.11347600	0.63672000
H	-6.15672200	3.64474900	0.90978500
H	-7.00349300	3.37225000	-0.61372100
S	-1.18198400	0.72263200	-2.07104500
O	-1.36139000	-0.70843600	-2.34879400
O	-0.80979400	1.59603500	-3.18537500
C	0.47465900	2.16152000	-0.69269200
C	-0.46821900	3.19737600	-0.18160400
C	-1.36156500	2.90219200	0.85470100
C	-0.47438800	4.47543400	-0.74871500
C	-2.23458700	3.87974900	1.32030600
H	-1.37080900	1.89686400	1.27047200
C	-1.35301400	5.45042100	-0.28233100
H	0.19473800	4.69178300	-1.57670300
C	-2.23496500	5.15411000	0.75387100

H	-2.92599800	3.64316400	2.12390400
H	-1.35645400	6.43624300	-0.73709400
H	-2.92626400	5.91016800	1.11363600
N	-0.00806300	0.82487100	-0.91899100
C	1.75814000	2.45475900	-0.92424900
H	2.44850200	1.72215300	-1.32969700
H	2.15116100	3.43679800	-0.68351000
H	3.02204700	-2.08162900	-3.72300700
C	3.25733100	-1.93538500	-1.18683600
H	2.99664200	-2.16168900	-0.14741000
H	3.60139800	-2.88032100	-1.62728100
C	4.39102800	-0.93270200	-1.17835000
C	4.48883400	0.11836200	-2.09352400
C	5.37861000	-1.05818200	-0.19496600
C	5.53927600	1.03397600	-2.01441900
H	3.74559000	0.24521800	-2.87724100
C	6.43299800	-0.15592100	-0.12238200
H	5.30721500	-1.86496800	0.52987100
C	6.51220000	0.90077200	-1.02899100
H	5.59238500	1.85026100	-2.72740000
H	7.18419900	-0.26746700	0.65308500
H	7.32725100	1.61437800	-0.96671800

M6^{CO}

0 1

C	5.06054200	1.68601900	2.70449900
C	3.88493800	1.29669500	2.07349100
C	2.76209800	0.92126500	2.82640100
C	2.84618900	0.97144100	4.22351400
C	4.02312500	1.36124700	4.85529800
C	5.13588100	1.71780400	4.09679400
H	5.91832000	1.97264400	2.10366900
H	3.83866600	1.29925100	0.98679900
H	1.97720500	0.69588800	4.81500700
H	4.07050300	1.39000700	5.93923300
H	6.05376700	2.02711700	4.58650000
C	1.50320300	0.49271800	2.19537800
H	0.60073000	0.50353000	2.80577200

C	1.37312900	0.09649100	0.92876700
H	2.23643900	0.06176900	0.26927200
C	-0.00479200	-0.24241200	0.36501300
O	-0.99939700	-0.18797900	1.15868100
C	2.05716600	-1.41520400	-1.93510800
C	2.11884100	-1.80509500	-3.44710700
H	1.88784400	-0.34076000	-1.84866700
H	1.82404700	-0.95125400	-4.05902300
O	1.24867100	-2.85466600	-3.82772600
N	0.89279500	-2.05423300	-1.32569200
C	1.15599500	-3.90620600	-2.88743500
C	0.50435200	-3.31113000	-1.68181300
H	0.52362500	-4.68617000	-3.31054600
H	2.14054700	-4.33147600	-2.64916500
N	-0.49529600	-3.71391900	-0.95684200
N	-0.74692700	-2.66264200	-0.11914900
C	0.06676800	-1.62900700	-0.35451800
C	-1.77409500	-2.85167600	0.87965100
C	-3.09820100	-2.97670500	0.46021600
C	-1.37654500	-3.02230600	2.20934300
C	-4.05949400	-3.21029500	1.44771100
C	-2.37178600	-3.26588200	3.14998200
C	-3.71971300	-3.34739400	2.79128000
H	-5.10096400	-3.29678500	1.14714800
H	-2.08702300	-3.39866800	4.19095400
C	0.06968700	-2.94255300	2.60979800
H	0.70374500	-3.53584700	1.94231600
H	0.41520900	-1.90609700	2.56692900
H	0.20567500	-3.31301000	3.62729800
C	-3.49622600	-2.90669600	-0.99055600
H	-2.89132100	-2.18831700	-1.54707700
H	-3.36890300	-3.88609900	-1.46330500
H	-4.54771500	-2.62347800	-1.07673700
C	-4.77696400	-3.56973500	3.84152800
H	-5.69588800	-3.96390300	3.40260100
H	-4.43046400	-4.26902300	4.60655200
H	-5.02346600	-2.62918300	4.34471900
C	-5.19916200	2.24877300	-0.39808000

C	-4.53520100	2.92846700	-1.42374500
C	-3.33993900	2.44694300	-1.94370000
C	-2.80913600	1.26930600	-1.42710300
C	-3.45121800	0.56143800	-0.41518900
C	-4.64705400	1.05955000	0.08900100
H	-4.95487900	3.85174600	-1.81344100
H	-2.81185900	2.97625700	-2.72878600
H	-2.98946000	-0.32347900	0.01145300
H	-5.15454400	0.51963500	0.88394400
C	-6.46243100	2.81329000	0.19646000
H	-7.05459200	2.03635300	0.68458900
H	-6.22067100	3.57106100	0.94962000
H	-7.07897400	3.29433200	-0.56664600
S	-1.25764300	0.67955300	-2.06899100
O	-1.41376500	-0.75447400	-2.33299400
O	-0.90058600	1.55565900	-3.18362200
C	0.40048600	2.16860800	-0.73132100
C	-0.55693200	3.18563900	-0.21086300
C	-1.42752900	2.87965600	0.84188100
C	-0.60162200	4.45773600	-0.79038900
C	-2.31515600	3.84218800	1.31143200
H	-1.41028100	1.87606800	1.26249300
C	-1.49490400	5.41688600	-0.31931900
H	0.04722500	4.68149100	-1.63244200
C	-2.35329800	5.11071900	0.73373500
H	-2.98908800	3.59705600	2.12719500
H	-1.52915200	6.39741900	-0.78405300
H	-3.05624400	5.85427800	1.09702700
N	-0.05415200	0.81179600	-0.93344500
C	1.66613200	2.48940100	-1.01247200
H	2.36138500	1.76869200	-1.42990300
H	2.04146500	3.48493300	-0.80114600
H	3.15808500	-2.07278200	-3.68361600
C	3.31020400	-1.84137300	-1.14948900
H	3.02287200	-2.03470500	-0.11030200
H	3.68008400	-2.79581600	-1.54588100
C	4.43055200	-0.82402800	-1.14339300
C	4.52639700	0.21871900	-2.06788300

C	5.40853600	-0.92842800	-0.14800600
C	5.56577400	1.14704800	-1.98619100
H	3.79264000	0.32739100	-2.86322700
C	6.45178400	-0.01399100	-0.07278800
H	5.33776400	-1.72879100	0.58401400
C	6.52896300	1.03478200	-0.98894200
H	5.61833400	1.95625000	-2.70725700
H	7.19586400	-0.10996500	0.71158600
H	7.33536100	1.75798200	-0.92499500

TS6(R)'

0 1

C	2.26971100	-0.78464300	1.05429200
C	3.76644800	-1.99447100	2.07050800
C	4.65832500	-0.47558900	0.25523500
H	4.49195700	-0.90645600	-0.73661300
N	2.62931000	-2.35522200	2.59511500
N	1.70681400	-1.58615600	1.95128200
N	3.58697100	-1.02240900	1.11842300
C	5.14165400	-2.52527100	2.34437400
H	5.08721900	-3.59455300	2.55013800
H	5.57402500	-2.01317100	3.21494900
C	5.99086600	-0.96689000	0.83292300
O	5.93435000	-2.33264600	1.19478100
C	0.28863100	-1.80557900	2.10041400
C	-0.39423000	-1.15763700	3.13146900
C	-0.32780200	-2.64786300	1.16958900
C	-1.77752800	-1.33157800	3.17725000
C	-1.71006400	-2.78624400	1.26089600
C	-2.45119100	-2.11177100	2.23409300
H	-2.34558400	-0.81598500	3.94752400
H	-2.22407100	-3.39870600	0.52304800
H	6.76585800	-0.85818800	0.07384300
C	1.52546700	0.06244700	0.03165100
O	1.61096000	-0.34896800	-1.13124800
C	0.81427500	1.16922800	0.54484600
C	-0.10905900	1.81464300	-0.26479700
H	-0.10745600	1.56418400	-1.32396300

C	-0.83058500	3.03852800	0.09325800
C	-1.67299300	3.61822600	-0.86777600
C	-0.70680300	3.65285800	1.34779400
C	-2.38471800	4.77490800	-0.57454700
H	-1.77660000	3.12866500	-1.83228100
C	-1.41185500	4.81552400	1.63488400
H	-0.05506600	3.22527000	2.10408200
C	-2.25456400	5.37712800	0.67639100
H	-3.04191200	5.20755600	-1.32162600
H	-1.30562100	5.28420500	2.60796600
H	-2.80813600	6.28224000	0.90479600
C	-3.95414700	-2.15625700	2.20514100
H	-4.38419700	-1.86190500	3.16478800
H	-4.31635800	-1.45203500	1.44614000
H	-4.32037100	-3.15124000	1.94079500
C	0.33310400	-0.31892800	4.14779200
H	0.94105500	-0.94862700	4.80477600
H	1.00963600	0.39875400	3.67413700
H	-0.37596300	0.23547600	4.76424400
C	0.46472700	-3.33831100	0.09094900
H	1.35194900	-3.82981400	0.50254800
H	-0.15039700	-4.09234600	-0.40276000
H	0.79609700	-2.62150000	-0.66933300
H	6.26183700	-0.35403700	1.70637000
C	4.60761600	1.05488700	0.18754200
H	3.59735700	1.38364900	-0.07254800
H	4.84117200	1.46270500	1.17685800
C	5.58209600	1.55997800	-0.85113600
C	6.81155000	2.11135700	-0.48783800
C	5.26123400	1.44184600	-2.20654600
C	7.70959300	2.53714400	-1.46465600
H	7.06449900	2.21387700	0.56450600
C	6.15717400	1.86541900	-3.18277600
H	4.29820500	1.02354500	-2.49229300
C	7.38465100	2.41317400	-2.81272300
H	8.66129600	2.96784800	-1.17038000
H	5.89557500	1.77351500	-4.23186200
H	8.08291800	2.74698300	-3.57330400

C	-2.98088500	0.77442500	-0.46556100
C	-1.78129900	0.15910900	-0.17800300
C	-3.79943000	1.29238300	0.67121300
C	-3.24237500	1.51754700	1.93808400
C	-5.16559400	1.54445400	0.48993500
C	-4.03208300	1.96075700	2.99415400
H	-2.18093900	1.36141000	2.10112900
C	-5.95564700	1.98306100	1.54745900
H	-5.59295400	1.38411900	-0.49348900
C	-5.39306700	2.19081100	2.80575000
H	-3.57908800	2.13405800	3.96600500
H	-7.01433600	2.16375400	1.38887700
H	-6.00916700	2.53352500	3.63113400
N	-3.53764500	1.01052800	-1.68362800
S	-2.80497700	0.43362000	-2.98994600
O	-3.69092000	0.66721400	-4.13747100
O	-1.41457600	0.91377500	-3.14399700
C	-2.68375100	-1.34262500	-2.76239100
C	-3.77499900	-2.04323300	-2.25164700
C	-1.50466200	-1.99918900	-3.08824200
C	-3.67532300	-3.41647400	-2.06910100
H	-4.68127400	-1.50872800	-1.98170200
C	-1.42075900	-3.37854200	-2.90634200
H	-0.65704600	-1.42465000	-3.44937700
C	-2.49726600	-4.10353600	-2.39239000
H	-4.51979000	-3.96790600	-1.66308000
H	-0.49801500	-3.89645000	-3.15433200
C	-2.39760700	-5.58951600	-2.16190100
H	-3.21051900	-6.11998600	-2.66562800
H	-1.44958800	-5.98522400	-2.53220600
H	-2.46986000	-5.82396900	-1.09453400
H	-1.54991100	-0.07902100	0.84731500
H	0.89849400	1.38147200	1.60519500
H	-1.19296900	-0.34661000	-0.93263800

TS6(S)'

0 1

C	-2.35949200	-1.56558400	0.00017000

C	-3.17343600	-3.51421200	0.52623800
C	-0.64552300	-3.36410600	0.53630400
H	-0.16649100	-3.36541300	-0.44595600
N	-4.21503600	-2.75360400	0.33396900
N	-3.68674100	-1.53934100	0.01366300
N	-2.00970500	-2.81709100	0.33982200
C	-3.12316900	-4.97859600	0.84486700
H	-3.28555200	-5.13735200	1.91952100
H	-3.90076300	-5.50049500	0.28650700
C	-0.81474700	-4.79097900	1.07832600
O	-1.87078700	-5.47823800	0.43664400
C	-4.53293800	-0.42607700	-0.33073000
C	-4.65661600	-0.08146900	-1.67830000
C	-5.18814900	0.24114800	0.70634900
C	-5.47717200	1.00540900	-1.97582700
C	-5.99730500	1.31784700	0.35259800
C	-6.14702900	1.71603400	-0.97820100
H	-5.59814400	1.30014800	-3.01467900
H	-6.52113800	1.85974600	1.13551700
H	-0.99595100	-4.75462100	2.16338000
C	-1.39725400	-0.51186000	-0.50589000
O	-0.59942000	-0.94235300	-1.35183200
C	-1.47177100	0.77847700	0.05843200
C	-0.58806900	1.74288600	-0.40851800
H	-0.12737400	1.57039400	-1.37755800
C	-0.53023000	3.11876900	0.08596300
C	-0.02061000	4.11093400	-0.76389500
C	-0.95022700	3.47232700	1.37671600
C	0.02300200	5.43779500	-0.34590600
H	0.33970000	3.82041000	-1.74684700
C	-0.88694300	4.79401900	1.79690300
H	-1.30551700	2.70427600	2.05789200
C	-0.41062700	5.78120400	0.93211700
H	0.40826900	6.20149800	-1.01382900
H	-1.20439800	5.05851300	2.80040500
H	-0.36580300	6.81428300	1.26220700
C	-6.99940400	2.90745500	-1.32755700
H	-6.40547100	3.82579700	-1.28321200

H	-7.83082200	3.01675000	-0.62803400
H	-7.40244900	2.82177700	-2.33864900
C	-3.94540100	-0.85426300	-2.75817800
H	-4.10447500	-1.93069800	-2.64240300
H	-2.86532800	-0.67570000	-2.73933500
H	-4.31577100	-0.55752500	-3.73994500
C	-5.02334500	-0.19361100	2.13829600
H	-5.43952800	-1.19299600	2.29446800
H	-5.53078100	0.50088800	2.80845600
H	-3.96661200	-0.23269900	2.42339500
H	0.10237500	-5.35163600	0.89810900
C	0.13252700	-2.47741800	1.52042000
H	-0.44297600	-2.41557100	2.45140000
H	0.21120400	-1.46935700	1.10635900
C	1.51995700	-3.00950100	1.79518400
C	2.58169300	-2.67919300	0.94730200
C	1.76174900	-3.83727600	2.89560700
C	3.85932400	-3.17653800	1.19655300
H	2.41431500	-2.01677400	0.09953300
C	3.03707100	-4.33942400	3.14045400
H	0.94797200	-4.07981500	3.57448400
C	4.08885200	-4.01057000	2.28884200
H	4.67663300	-2.90036600	0.53773400
H	3.20969400	-4.97879000	4.00005900
H	5.08570700	-4.39363400	2.48103500
C	2.35134800	1.92674100	0.21838900
C	1.49366900	0.85008400	0.30413900
C	2.52360500	2.78174700	1.43369300
C	2.43350900	2.25341200	2.72775000
C	2.75736300	4.15302400	1.28439500
C	2.54992500	3.07869200	3.84179500
H	2.29884300	1.18508600	2.86519900
C	2.85976000	4.98071100	2.39769500
H	2.83693500	4.55622600	0.28158400
C	2.75486000	4.44786700	3.68081800
H	2.48852700	2.65058100	4.83746600
H	3.02080700	6.04596500	2.26283500
H	2.84055900	5.09253900	4.54995900

N	3.02836600	2.40156400	-0.85817700
S	2.83055400	1.76811400	-2.31965300
O	3.80071700	2.42448300	-3.20513800
O	1.42484500	1.78584000	-2.78032100
C	3.29384600	0.03732100	-2.26678800
C	4.58142000	-0.31038600	-1.87014000
C	2.37984300	-0.93586800	-2.66147200
C	4.95356300	-1.65132000	-1.87128800
H	5.28473900	0.45964100	-1.56796500
C	2.76601100	-2.27253900	-2.65516900
H	1.37421000	-0.63994700	-2.93943200
C	4.05385200	-2.64975600	-2.26045800
H	5.95971800	-1.92893600	-1.56818700
H	2.05493200	-3.03724900	-2.95743600
C	4.44289500	-4.10422200	-2.22003500
H	3.98283700	-4.59612000	-1.35578300
H	5.52516500	-4.22431400	-2.13636700
H	4.10409400	-4.62850700	-3.11718100
H	1.07416000	0.60185400	1.26998100
H	-2.15595200	0.95803000	0.88151200
H	1.37889400	0.12090800	-0.48929800

TS6(R)''

0 1

C	-0.12406500	-2.00721800	0.13775600
C	-0.70588700	-3.28866200	-1.53033000
C	-2.63583200	-2.22789200	-0.41634000
H	-2.80497800	-1.98710600	0.63433600
N	0.58773800	-3.39560200	-1.46268900
N	0.93825500	-2.59378000	-0.42023200
N	-1.18799800	-2.45940800	-0.55797700
C	-1.69315300	-4.10542500	-2.29644900
H	-1.19644000	-4.96232500	-2.75029800
H	-2.19766100	-3.52133900	-3.07740500
C	-3.40575200	-3.54451400	-0.78035000
O	-2.60356700	-4.57593000	-1.32137200
C	2.32385900	-2.60336500	-0.01118000
C	3.26709200	-1.91165100	-0.77053300

C	2.66463700	-3.42106700	1.07502000
C	4.60018600	-2.00296500	-0.35992500
C	4.00416900	-3.46738200	1.44486100
C	4.98468000	-2.75955300	0.74294500
H	5.35370600	-1.45750800	-0.92321700
H	4.29249200	-4.08174500	2.29403100
H	-3.86578400	-3.96501400	0.11395000
C	-0.09108100	-1.07786200	1.34069500
O	0.81609200	-1.25417700	2.15670300
C	-1.12776400	-0.10641100	1.44212800
C	-1.06809800	0.83544000	2.45369100
H	-0.36328600	0.63965900	3.25792000
C	-2.21797800	1.70269100	2.78758900
C	-2.44731700	2.05904400	4.12069900
C	-3.10624200	2.15506200	1.80129300
C	-3.55950000	2.82143800	4.46922300
H	-1.75492600	1.72271000	4.88785500
C	-4.21725200	2.91397800	2.15130200
H	-2.90390300	1.92772300	0.75689000
C	-4.45091800	3.24491900	3.48661000
H	-3.73042000	3.08185400	5.50895500
H	-4.90065600	3.25264900	1.37865600
H	-5.31882200	3.83751100	3.75739600
C	6.43253700	-2.84741200	1.15111300
H	6.97980300	-1.94863800	0.85747300
H	6.53026600	-2.97621300	2.23188500
H	6.91767200	-3.70378200	0.67153700
C	2.90866300	-1.05611500	-1.95221200
H	1.94301700	-1.32688900	-2.37888900
H	2.83455800	-0.01324900	-1.62739100
H	3.68270900	-1.13428800	-2.71950900
C	1.62735300	-4.22767600	1.80743200
H	0.99315200	-4.78375900	1.10927700
H	2.10688600	-4.94505000	2.47512600
H	0.98891300	-3.56813000	2.40089500
H	-4.19882000	-3.27957200	-1.49181800
C	-3.09826100	-1.06252600	-1.30915800
H	-2.45123500	-0.19217800	-1.16842000

H	-2.98461300	-1.35331300	-2.35967700
C	-4.53993700	-0.70744200	-1.02121700
C	-5.51571400	-0.78866500	-2.01652500
C	-4.91742900	-0.27834700	0.25581500
C	-6.83808100	-0.44092400	-1.74715600
H	-5.23456300	-1.12053300	-3.01272300
C	-6.23528600	0.07587700	0.52672200
H	-4.17492900	-0.20216100	1.04747200
C	-7.20068900	-0.00592200	-0.47533900
H	-7.58350700	-0.50962600	-2.53299000
H	-6.50447000	0.41990600	1.52062000
H	-8.22959300	0.26749600	-0.26497400
C	1.23841300	1.76427000	1.03804400
C	0.22626600	2.51509300	1.61505500
C	2.56109400	1.68541500	1.73580600
C	3.09224400	2.82716000	2.34686600
C	3.31003400	0.50337900	1.75410400
C	4.33319900	2.78711600	2.97774200
H	2.54158900	3.76185200	2.30568300
C	4.54749000	0.46324200	2.38737500
H	2.89429300	-0.38629600	1.29564300
C	5.06424300	1.60277200	3.00336000
H	4.73132400	3.68540000	3.43929000
H	5.10740200	-0.46756900	2.40382700
H	6.03121800	1.56750400	3.49566100
N	1.13762800	0.97317300	-0.05688600
S	0.07366200	1.21159200	-1.24605300
O	-0.10983300	-0.07267200	-1.94527100
O	-1.17465900	1.91739000	-0.89213200
C	0.93736600	2.29460700	-2.37194600
C	1.49434600	1.78314700	-3.53711200
C	1.06018100	3.64515800	-2.04735900
C	2.18758600	2.64017600	-4.38906700
H	1.37089400	0.73099000	-3.77181900
C	1.75766300	4.48426000	-2.90546100
H	0.60836400	4.02943100	-1.13754200
C	2.33346900	3.99482300	-4.08509000
H	2.61808300	2.24973500	-5.30688300

H	1.85490500	5.53904600	-2.66321600
C	3.10711600	4.91558800	-4.99221800
H	2.60048700	5.87774400	-5.10200900
H	4.10166900	5.11460300	-4.58014200
H	3.23729600	4.47742200	-5.98376900
H	0.44458800	3.07903900	2.51518900
H	-1.86646000	-0.01363300	0.66070300
H	-0.63833400	2.81065000	1.04067000

TS6(S)''

0 1

C	-0.27802900	-1.90843800	0.35054700
C	-0.53112300	-2.52196500	2.42987600
C	-2.58025600	-1.49811700	1.42942300
H	-2.55948500	-0.40518300	1.37848400
N	0.67065400	-2.90000200	2.11166600
N	0.81273500	-2.52390600	0.81486300
N	-1.17416100	-1.95786500	1.36378000
C	-1.16908300	-2.59072200	3.78416300
H	-1.51925700	-3.61173000	3.99064600
H	-0.43001100	-2.30788000	4.53485500
C	-3.14441600	-1.91396600	2.79531200
O	-2.23135500	-1.66868100	3.84042100
C	2.03164900	-2.90120000	0.14055100
C	3.22814000	-2.28825200	0.50412100
C	1.95850900	-3.95593700	-0.78226800
C	4.38803500	-2.72981400	-0.14477400
C	3.13866700	-4.35014500	-1.39714900
C	4.36322200	-3.74007300	-1.09843700
H	5.33245500	-2.25239000	0.10644100
H	3.10780700	-5.15858600	-2.12366500
H	-3.41927300	-2.98037700	2.77663700
C	-0.33766200	-1.20242600	-1.00184700
O	0.43383300	-1.61262200	-1.87280900
C	-1.23907200	-0.10933800	-1.12232100
C	-1.14288700	0.74874400	-2.20983200
H	-0.52631600	0.40913600	-3.03910500
C	-2.23599200	1.68578400	-2.56118300

C	-2.52389700	1.93139400	-3.90743100
C	-3.01005500	2.31681600	-1.57617800
C	-3.57928900	2.76610100	-4.26685100
H	-1.92127700	1.45481100	-4.67596000
C	-4.06108000	3.15220600	-1.93665600
H	-2.76555900	2.16258600	-0.52719200
C	-4.35373600	3.37541800	-3.28324600
H	-3.79552900	2.94000100	-5.31613500
H	-4.65199000	3.63511900	-1.16437300
H	-5.17611800	4.02646800	-3.56165000
C	5.62129400	-4.18618700	-1.79696300
H	6.48079000	-3.58947200	-1.48523800
H	5.51532500	-4.09412100	-2.88206600
H	5.83697800	-5.23658800	-1.57968000
C	3.32196600	-1.16169500	1.49645300
H	2.40085500	-1.02421800	2.06251400
H	3.50696200	-0.22552600	0.95896700
H	4.15297700	-1.34106300	2.18422700
C	0.65245600	-4.63191000	-1.09920600
H	0.09688800	-4.87519300	-0.18789800
H	0.82777800	-5.55818100	-1.64860000
H	0.03079600	-3.97428700	-1.71320500
H	-4.04141200	-1.32570000	2.99260000
C	-3.41013700	-2.12399400	0.29540400
H	-3.51499100	-3.19512200	0.50168000
H	-2.87161200	-2.02410100	-0.64990000
C	-4.76348300	-1.46394500	0.16353000
C	-4.91508500	-0.37795900	-0.70175700
C	-5.86702200	-1.89749700	0.90312900
C	-6.13802900	0.27668400	-0.81459900
H	-4.06983100	-0.05085800	-1.30180200
C	-7.09332800	-1.24629800	0.79034200
H	-5.77099300	-2.75539100	1.56462800
C	-7.23038900	-0.15555500	-0.06569300
H	-6.23110500	1.12040300	-1.49211000
H	-7.94336300	-1.59395000	1.36871400
H	-8.18684900	0.34966500	-0.15328600
C	1.28379700	1.55267100	-1.08881200

C	0.27182600	2.35366600	-1.59992100
C	2.48229100	1.28018200	-1.94101700
C	3.06044300	2.31196400	-2.68870200
C	3.07263300	0.01295700	-1.97101100
C	4.20092400	2.08028200	-3.45454600
H	2.63266400	3.30910600	-2.64787500
C	4.20955800	-0.21852500	-2.73696200
H	2.60641300	-0.79103200	-1.41471200
C	4.77886300	0.81406600	-3.48183200
H	4.64297900	2.89404000	-4.02102000
H	4.64712100	-1.21306200	-2.75400700
H	5.66772000	0.63220300	-4.07801200
N	1.24999700	0.85838400	0.06812800
S	0.37780200	1.32920400	1.35037200
O	0.40531200	0.21155400	2.30909400
O	-0.95765300	1.90260200	1.08754200
C	1.35104100	2.64970900	2.04877800
C	2.47338400	2.33231000	2.81160700
C	1.01920000	3.97142300	1.77437500
C	3.26641800	3.35881900	3.30571700
H	2.70683500	1.29218900	3.01741600
C	1.82814300	4.98888700	2.27480000
H	0.13347400	4.19643900	1.19007000
C	2.95721600	4.69982700	3.04387300
H	4.14095100	3.12042500	3.90483800
H	1.57558800	6.02472000	2.06644400
C	3.81600600	5.80207300	3.60646900
H	3.62799700	6.74981000	3.09798300
H	4.87770300	5.56137100	3.50906300
H	3.60957000	5.94641300	4.67182200
H	0.43031000	2.85436500	-2.54896500
H	-1.89783800	0.14742200	-0.30793600
H	-0.48643300	2.76280200	-0.94827900

TS6(R)'''

0 1

C	2.81164400	1.11843300	-0.24310500
C	3.78669100	2.90616000	-1.01988100

C	1.31736100	2.75451000	-1.51533000
H	1.09554800	2.15065700	-2.40032500
N	4.72090200	2.25994900	-0.38200600
N	4.09227200	1.15465000	0.10293500
N	2.58937100	2.24021500	-0.95119500
C	3.89512600	4.20526500	-1.76237400
H	4.80806000	4.21167000	-2.35886200
H	3.93165000	5.03635000	-1.04470200
C	1.57321000	4.20486700	-1.93450100
O	2.79587200	4.33172700	-2.63401600
C	4.82539500	0.16035600	0.84557000
C	4.90432600	0.30302800	2.23202400
C	5.42139700	-0.87881200	0.13400900
C	5.64297900	-0.65441700	2.92008400
C	6.14935200	-1.81274000	0.87155000
C	6.27353700	-1.71215200	2.25735100
H	5.72312000	-0.57801200	4.00152400
H	6.62718300	-2.63713300	0.34917900
H	0.77367300	4.52548300	-2.60315100
C	1.85436500	-0.00522100	0.13320800
O	1.81626900	-0.29520400	1.32689600
C	1.20128500	-0.61367000	-0.97065300
C	0.36856500	-1.70664200	-0.76777800
H	0.31424700	-2.07482400	0.25610400
C	0.10231900	-2.69511100	-1.83046700
C	0.05058600	-4.05242700	-1.49646100
C	-0.06565000	-2.32124300	-3.17087400
C	-0.16321300	-5.01657600	-2.47746200
H	0.16824700	-4.35122600	-0.45928200
C	-0.28433100	-3.28292600	-4.14882000
H	-0.05085100	-1.26803900	-3.44131200
C	-0.33176500	-4.63545100	-3.80495500
H	-0.20902400	-6.06431700	-2.19809800
H	-0.42266100	-2.97999400	-5.18182600
H	-0.50541900	-5.38480700	-4.57048100
C	7.08053300	-2.71686200	3.03730800
H	6.53111800	-3.05767100	3.91853100
H	7.32999300	-3.58670700	2.42692200

H	8.01605600	-2.26980500	3.38775900
C	4.18703000	1.42396000	2.93129300
H	4.48054800	2.39713400	2.52580100
H	3.10726500	1.30670600	2.79278100
H	4.40664200	1.41459500	3.99948600
C	5.25965700	-0.98945600	-1.35896000
H	5.58107700	-0.07255800	-1.86231700
H	5.84873700	-1.82070300	-1.74797300
H	4.21033600	-1.16456700	-1.62289700
H	1.57019700	4.85333800	-1.04518900
C	0.19784800	2.65598000	-0.47894100
H	0.07271700	1.62297200	-0.14531400
H	0.48222800	3.23885900	0.40384400
C	-1.11775500	3.15158700	-1.02629000
C	-1.73839500	4.27437900	-0.47632200
C	-1.75129600	2.47179500	-2.07306200
C	-2.97343300	4.70775800	-0.95328400
H	-1.25697500	4.80099100	0.34291000
C	-2.98445300	2.90391500	-2.55333200
H	-1.28633200	1.58498400	-2.49983000
C	-3.59745200	4.02482500	-1.99451500
H	-3.44851700	5.57639700	-0.50895200
H	-3.46992200	2.36351400	-3.35949000
H	-4.55990400	4.36111600	-2.36706600
C	-2.21476200	-1.69592000	0.40620200
C	-1.81098700	-0.87269800	-0.63602600
C	-2.59222600	-3.10606100	0.08137500
C	-3.20824800	-3.44307200	-1.12923700
C	-2.29393400	-4.12754400	0.99118200
C	-3.49488600	-4.76971200	-1.43369300
H	-3.47868900	-2.66402200	-1.83467900
C	-2.56835700	-5.45569700	0.68073400
H	-1.83134300	-3.86118900	1.93541200
C	-3.16718200	-5.78214600	-0.53457500
H	-3.97485800	-5.01252200	-2.37665000
H	-2.31619800	-6.23792800	1.39019900
H	-3.38488900	-6.81807000	-0.77558100
N	-2.19127600	-1.45020800	1.73578300

S	-2.09176200	0.05382000	2.30957100
O	-1.95879600	-0.05536600	3.76653200
O	-1.11779100	0.93017000	1.63176600
C	-3.70634900	0.75594800	1.99636300
C	-4.76117000	0.38508200	2.83081900
C	-3.91790900	1.60866900	0.91856900
C	-6.03456400	0.87546400	2.57943800
H	-4.57186100	-0.27550100	3.67099200
C	-5.20443100	2.09029000	0.67833000
H	-3.08804600	1.90791100	0.28573700
C	-6.27501600	1.73430100	1.49913800
H	-6.85876300	0.59196000	3.22863300
H	-5.37099100	2.75932600	-0.16164600
C	-7.65864800	2.27756900	1.25285600
H	-7.74404000	2.70685800	0.25242400
H	-8.41357400	1.49413000	1.35843900
H	-7.90040700	3.06267000	1.97667000
H	-2.01918600	-1.16157300	-1.65695700
H	1.43327900	-0.25944500	-1.97112300
H	-1.56535800	0.16349300	-0.44984400

TS6(S)'''

0 1

C	-2.54976500	-1.06633800	0.75481700
C	-4.14049500	-2.24126000	1.66632300
C	-4.93859800	-0.42939500	0.09842200
H	-4.89468400	0.56484400	0.55409000
N	-3.03931700	-2.71678200	2.17266900
N	-2.06024100	-1.97390200	1.59009100
N	-3.88401700	-1.23501600	0.76530700
C	-5.55135000	-2.61797200	2.00221600
H	-5.86535800	-3.48109800	1.39977100
H	-5.61532800	-2.87850300	3.05899400
C	-6.28809200	-1.08961500	0.41653600
O	-6.37800100	-1.50203900	1.76476700
C	-0.66416800	-2.21139800	1.85776700
C	-0.13910300	-1.75624100	3.07224600
C	0.08148600	-2.88103100	0.88758400

C	1.21825400	-1.96738900	3.28773200
C	1.43737200	-3.07237000	1.15981900
C	2.01950500	-2.61442500	2.34023700
H	1.66255300	-1.62218200	4.21808200
H	2.04885700	-3.57983200	0.41843000
H	-6.44455000	-1.94782700	-0.25496300
C	-1.66724600	-0.08028700	-0.01147600
O	-1.54600100	-0.26538900	-1.22336800
C	-1.07969400	0.91164700	0.80902100
C	-0.11657300	1.76182500	0.28819900
H	0.03445300	1.71970400	-0.78920100
C	0.31155300	2.99586500	0.96220900
C	0.75021700	4.07663600	0.18792000
C	0.29841700	3.12555800	2.35828400
C	1.14815100	5.26567100	0.78992500
H	0.78487000	3.97402400	-0.89303000
C	0.69908200	4.31189100	2.95951500
H	-0.00367900	2.28359600	2.97600000
C	1.12010600	5.38744500	2.17653100
H	1.49243700	6.09106700	0.17538200
H	0.68951400	4.39847800	4.04140100
H	1.43711000	6.31203700	2.64870400
C	3.49294300	-2.79611600	2.59574300
H	3.68142500	-3.08782400	3.63192200
H	4.03050700	-1.85755400	2.41628300
H	3.91498700	-3.55891100	1.93736000
C	-1.01288900	-1.09913000	4.10688400
H	-1.70275400	-1.82459500	4.54854700
H	-1.62259000	-0.29864800	3.67629600
H	-0.40435000	-0.67258800	4.90547200
C	-0.54774900	-3.37503000	-0.38623700
H	-1.47104300	-3.92567800	-0.17858400
H	0.14246900	-4.03473400	-0.91260000
H	-0.77451900	-2.53576800	-1.05035000
H	-7.08101900	-0.36242000	0.24176600
C	-4.72255500	-0.33932800	-1.41770600
H	-4.73788000	-1.35278700	-1.83277200
H	-3.73803700	0.08210700	-1.62862000

C	-5.80488500	0.50988100	-2.04487800
C	-5.78100600	1.89859500	-1.88385800
C	-6.85496600	-0.06925800	-2.76017600
C	-6.78941600	2.69160200	-2.42245400
H	-4.95933200	2.36173300	-1.34246000
C	-7.86449200	0.72291100	-3.30290900
H	-6.87841600	-1.14721200	-2.89851700
C	-7.83523100	2.10446400	-3.13274400
H	-6.75584100	3.76858000	-2.29333900
H	-8.67226200	0.25889300	-3.85967500
H	-8.62040900	2.72258800	-3.55568800
C	2.63579000	1.15137500	-0.39132000
C	1.85616700	0.53792300	0.57583800
C	3.36498700	2.41299900	-0.04493600
C	3.65778000	2.76242200	1.27993300
C	3.74891700	3.29596500	-1.06326300
C	4.28521400	3.96641100	1.57817000
H	3.40241600	2.09080200	2.09210300
C	4.37406600	4.50175700	-0.76530100
H	3.53556600	3.02118800	-2.08983900
C	4.63990100	4.84631300	0.55808900
H	4.49641600	4.21808500	2.61304700
H	4.65263200	5.17567400	-1.56978500
H	5.12643800	5.78821700	0.79213100
N	2.72842200	0.83709400	-1.71043300
S	2.42259600	-0.62322900	-2.29622900
O	2.26123400	-0.49230900	-3.74973200
O	1.38711100	-1.40151400	-1.59119000
C	3.94951000	-1.51971500	-2.03822900
C	4.79279400	-1.78996200	-3.10762100
C	4.28798600	-1.91720400	-0.74439700
C	5.98778200	-2.46994800	-2.87877400
H	4.50258100	-1.47510700	-4.10434300
C	5.48745700	-2.58298800	-0.52993800
H	3.60864300	-1.70389200	0.07709200
C	6.35435900	-2.86973100	-1.59269400
H	6.64578700	-2.69337800	-3.71412400
H	5.76166900	-2.89024600	0.47642100

C	7.66170600	-3.57541700	-1.34081400
H	8.40335500	-2.88056800	-0.93370700
H	8.06899700	-3.99534500	-2.26286800
H	7.53975000	-4.38450100	-0.61615600
H	1.93342600	0.84158900	1.61124200
H	-1.36880900	0.95662400	1.85323900
H	1.36440300	-0.40238400	0.36785100

TS6(R)$^{HOBt'}$

0 1

C	1.31745800	1.90117800	-0.51024000
C	2.69066300	3.11042700	0.67565700
C	3.55404500	0.80818100	0.23004800
H	3.15095300	0.17741100	1.02774600
N	1.64089300	3.86156600	0.51436300
N	0.79702400	3.09633200	-0.22638400
N	2.54962100	1.89702200	0.04396700
C	3.93522200	3.48724100	1.42243300
H	3.66229900	4.03944500	2.32368800
H	4.55168400	4.13516200	0.78306500
C	4.84898200	1.47119100	0.70571900
O	4.63883200	2.33465000	1.80409600
C	-0.42113800	3.67069400	-0.74650800
C	-0.41604800	4.08252300	-2.08269600
C	-1.52711300	3.79295800	0.09411500
C	-1.59932000	4.61648500	-2.58490200
C	-2.68568800	4.33653200	-0.45969200
C	-2.74166000	4.74676500	-1.79143300
H	-1.62942000	4.94062200	-3.62200900
H	-3.56751800	4.43523900	0.16808700
H	5.53002700	0.68964500	1.04172600
C	0.61137600	0.89377500	-1.41201900
O	1.24119400	0.46611500	-2.38493900
C	-0.74197900	0.65578900	-1.08506900
C	-1.48313400	-0.19012500	-1.89390900
H	-1.14973300	1.07583700	-0.17656300
H	-1.04155800	-0.41930700	-2.86127500
C	-2.95605000	-0.27076500	-1.82714100

C	-3.66038000	-0.69552000	-2.95820300
C	-3.67335700	0.10363300	-0.68083200
C	-5.05204100	-0.71579200	-2.96400000
H	-3.10670500	-1.00154200	-3.84237500
C	-5.06261400	0.09406100	-0.69226400
H	-3.13678800	0.38921300	0.22044200
C	-5.75582400	-0.31058700	-1.83418700
H	-5.58444400	-1.04217600	-3.85167400
H	-5.61531000	0.38481100	0.19705200
H	-6.84116300	-0.32333400	-1.83003800
C	-3.99866000	5.34985900	-2.36090500
H	-4.16234400	5.01635300	-3.38825300
H	-4.87226900	5.07966800	-1.76468200
H	-3.92664700	6.44206000	-2.37607700
C	0.81689300	3.94912400	-2.93838200
H	1.68738900	4.39109400	-2.44282100
H	1.04993700	2.89947200	-3.14662000
H	0.67215100	4.45866000	-3.89168900
C	-1.50146100	3.32148800	1.52386200
H	-0.54869200	3.54983400	2.00890000
H	-2.29886000	3.79981400	2.09461700
H	-1.66336500	2.23720400	1.58386400
H	5.31849000	2.01403900	-0.12983500
C	3.80225700	-0.01966400	-1.03108600
H	2.88431800	-0.54750900	-1.29784100
H	4.04855100	0.64887500	-1.86221100
C	4.91040100	-1.02741500	-0.82062700
C	5.96669700	-1.12618800	-1.72615800
C	4.86663400	-1.91746200	0.25908500
C	6.94873600	-2.10434400	-1.57171400
H	6.01300200	-0.44093200	-2.56821900
C	5.84646300	-2.89146000	0.41935900
H	4.04255600	-1.86131200	0.96793000
C	6.89070500	-2.99075800	-0.50033700
H	7.75871500	-2.17220100	-2.29111700
H	5.79212100	-3.57784600	1.25846700
H	7.65359800	-3.75279500	-0.37885500
C	0.27284000	-2.18338000	-0.97025100

C	-1.08928800	-2.27605100	-1.18986500
C	1.23030500	-2.67469800	-1.99988500
C	0.91666900	-2.68423900	-3.36466700
C	2.47670400	-3.15625500	-1.59366200
C	1.83950700	-3.14820200	-4.29471500
H	-0.04251900	-2.30366300	-3.70276000
C	3.40750600	-3.60961100	-2.52356300
H	2.71013800	-3.16184400	-0.53537400
C	3.09112200	-3.60610500	-3.87941700
H	1.58828300	-3.13916700	-5.35077200
H	4.38029200	-3.95322800	-2.18073100
H	3.81220600	-3.95681000	-4.61108100
N	0.85845700	-1.56860000	0.09606800
S	0.07473500	-1.53723600	1.50149100
O	-1.19888400	-0.74093200	1.51332900
O	-0.07007500	-2.87051800	2.10124300
C	1.21412700	-0.60762100	2.49533000
C	1.01580400	0.75464100	2.68293300
C	2.30185100	-1.26280500	3.06738200
C	1.93370500	1.47520700	3.44231600
H	0.14364600	1.23152800	2.24846900
C	3.21508500	-0.52757100	3.81467000
H	2.41779400	-2.33407400	2.93158000
C	3.04683700	0.84934400	4.01176900
H	1.77435800	2.53839100	3.60822200
H	4.06573800	-1.02981600	4.26660400
C	4.04860300	1.63256300	4.81519900
H	3.62472400	2.57861000	5.15970000
H	4.38461600	1.06495400	5.68616300
H	4.92057700	1.85991500	4.19520200
H	-1.79331500	-2.11730200	-0.38286100
H	-1.44692600	-2.86752800	-2.02611100
C	-5.25879300	-1.82043800	1.97886400
C	-5.78446800	-2.53397700	0.89367100
C	-7.13060500	-2.35963700	0.52318200
C	-7.87793100	-1.47036400	1.26499600
C	-7.32311800	-0.76049000	2.36509500
C	-6.00835000	-0.91773100	2.74866300

H	-7.54763800	-2.89708500	-0.32160100
H	-8.91930700	-1.30265900	1.01160500
H	-7.95841800	-0.07627300	2.91796300
H	-5.57096600	-0.38357000	3.58367900
N	-4.78325000	-3.29547900	0.34247800
N	-3.70414500	-3.10242200	1.03416900
N	-3.96573600	-2.22030600	1.99582500
O	-3.04314300	-1.88268700	2.92077000
H	-2.31252800	-1.41635500	2.40931700

TS6(S)$^{HOBt'}$

0 1

C	-2.52407200	-1.94636900	-0.64208300
C	-3.09711700	-3.99072500	-0.17201600
C	-0.62391800	-3.50712600	0.04654000
H	-0.07179300	-3.38669500	-0.88640100
N	-4.21559300	-3.38909800	-0.47019400
N	-3.83980000	-2.11270600	-0.75262000
N	-2.03207200	-3.13626200	-0.25725200
C	-2.87156200	-5.44042000	0.13760500
H	-3.55363000	-6.04859200	-0.45745900
H	-3.05321200	-5.63367700	1.20378500
C	-0.63006900	-4.97143800	0.50496800
O	-1.55013600	-5.76390500	-0.22507500
C	-4.80197600	-1.11986400	-1.14934200
C	-5.65514400	-0.61212800	-0.16544600
C	-4.79378400	-0.67563600	-2.47307300
C	-6.52085200	0.41050200	-0.54649300
C	-5.68133800	0.34824700	-2.79983200
C	-6.53750600	0.90984400	-1.85145200
H	-7.19418500	0.83132700	0.19577300
H	-5.69912000	0.71832900	-3.82152000
H	0.36526900	-5.38703200	0.34177800
C	-1.66615700	-0.74444100	-0.98704700
O	-0.60553000	-1.03691000	-1.55037300
C	-2.15369000	0.53182100	-0.62232000
C	-1.31468400	1.63477700	-0.73742000
H	-3.15519800	0.61450400	-0.21624500

H	-0.33279300	1.44197200	-1.16855000
C	-1.78736000	3.02482500	-0.77729200
C	-0.91383300	4.01691500	-1.24312900
C	-3.08026200	3.39710000	-0.38129500
C	-1.31587700	5.34504900	-1.31228800
H	0.09479300	3.73437000	-1.53461300
C	-3.48239900	4.72554900	-0.45099900
H	-3.77877900	2.64753400	-0.01995700
C	-2.60210400	5.70299100	-0.91553200
H	-0.62001000	6.10169400	-1.65983000
H	-4.48567100	5.00109500	-0.14151600
H	-2.91769300	6.74050300	-0.96226500
C	-7.44339300	2.05461600	-2.22072800
H	-6.93438000	3.00939900	-2.05227500
H	-7.72517700	2.01177600	-3.27482500
H	-8.35224300	2.05182000	-1.61539000
C	-5.62941900	-1.14501800	1.24228000
H	-6.06353100	-2.14812500	1.28706600
H	-4.60637000	-1.22143900	1.62567600
H	-6.19855600	-0.49344800	1.90662600
C	-3.85834300	-1.25676800	-3.50053900
H	-3.77651900	-2.34260900	-3.40082300
H	-4.21674300	-1.03054900	-4.50569600
H	-2.85210700	-0.83484200	-3.40316300
H	-0.86872300	-5.03117100	1.57485900
C	0.00376800	-2.58275400	1.10703200
H	1.01987900	-2.95702100	1.27453900
H	0.14056600	-1.58909200	0.67640500
C	-0.76114300	-2.49846900	2.40696300
C	-1.79058700	-1.56344600	2.56823400
C	-0.46570700	-3.34650600	3.47919700
C	-2.51704500	-1.49252500	3.75471500
H	-2.00439200	-0.85672400	1.76786100
C	-1.19111200	-3.28191600	4.66649300
H	0.35244000	-4.05656800	3.38708600
C	-2.22419200	-2.35835200	4.80549600
H	-3.30309100	-0.75109700	3.86247500
H	-0.94224800	-3.94802300	5.48635700

H	-2.78707200	-2.30371900	5.73155700
C	0.69726800	2.57824500	1.33425600
C	-0.37600800	1.70474900	1.39489100
C	0.47071100	4.04424900	1.49623900
C	-0.69790600	4.55587400	2.07479500
C	1.42483800	4.94884900	1.00946600
C	-0.92081900	5.92643700	2.13301500
H	-1.44429400	3.88523300	2.48513700
C	1.20402000	6.31971200	1.07173400
H	2.33187800	4.55349300	0.56661800
C	0.02512700	6.81466500	1.62559300
H	-1.83886600	6.30213000	2.57403700
H	1.95014100	7.00416500	0.68003900
H	-0.15265400	7.88475300	1.66821100
N	1.98772100	2.26224500	1.03729900
S	2.33925700	0.74565500	0.66691900
O	2.02461100	-0.25591000	1.70055500
O	1.80402500	0.41003600	-0.69102300
C	4.10340000	0.80007800	0.50377400
C	4.89348900	0.18322500	1.46224800
C	4.66591400	1.43368100	-0.60433600
C	6.27945200	0.21548200	1.31451100
H	4.42509100	-0.32510900	2.29800700
C	6.04512100	1.45443800	-0.73588000
H	4.02436100	1.88379700	-1.35543500
C	6.87013100	0.84874900	0.22221100
H	6.90787100	-0.27205100	2.05437600
H	6.49643400	1.92943800	-1.60291800
C	8.36371600	0.85129900	0.03429500
H	8.87333500	0.39209300	0.88376300
H	8.63038700	0.29095300	-0.86832300
H	8.74489200	1.86908500	-0.08813200
H	-0.19511100	0.63839300	1.41709500
H	-1.33467500	2.03938200	1.76594800
C	4.24350200	-1.74230800	-1.79362200
C	5.35557900	-2.16938700	-1.05731300
C	6.65456100	-1.92639000	-1.53944400
C	6.76583800	-1.26375600	-2.74330900

C	5.62270000	-0.83266400	-3.46791000
C	4.34050500	-1.05768100	-3.01370900
H	7.52473700	-2.24678000	-0.97567900
H	7.74969300	-1.05941100	-3.15362700
H	5.76789400	-0.31205700	-4.40893100
H	3.45874100	-0.73394800	-3.55411100
N	4.91103200	-2.81219300	0.07523700
N	3.61689700	-2.79528800	0.06675200
N	3.20432800	-2.16224900	-1.02999900
O	1.89103200	-2.09144700	-1.33417900
H	1.63113300	-1.14926000	-1.09812300

TS6(R)$^{HOBt''}$

0 1

C	-2.55472600	-1.86865800	-0.51689700
C	-3.26356100	-3.89661100	-0.16678800
C	-0.82557700	-3.54235900	0.37154700
H	-0.15225900	-3.41362500	-0.47609600
N	-4.30719800	-3.24590300	-0.60025100
N	-3.85055800	-1.98427900	-0.80703600
N	-2.16397000	-3.08519900	-0.09026700
C	-3.14968500	-5.36098200	0.12925000
H	-3.79356300	-5.91768500	-0.55206200
H	-3.45635100	-5.56572100	1.16426100
C	-0.95026400	-5.02393700	0.75966700
O	-1.81447900	-5.74638100	-0.09540900
C	-4.73178700	-0.96146500	-1.30563400
C	-5.65163600	-0.41029500	-0.40994300
C	-4.58921300	-0.54174200	-2.62926900
C	-6.44204800	0.63645400	-0.87995900
C	-5.40631100	0.50706600	-3.04787600
C	-6.32243600	1.11478800	-2.18746000
H	-7.16279900	1.09282700	-0.20660400
H	-5.31789900	0.86200800	-4.07116600
H	0.03977500	-5.47629200	0.68690700
C	-1.62730500	-0.69052800	-0.72112100
O	-0.44405800	-0.99363600	-0.91920500
C	-2.16665400	0.61544200	-0.63058400

C	-1.28885200	1.67849700	-0.79729000
H	-3.20391300	0.75750200	-0.35680700
H	-0.32166800	1.43860400	-1.23677300
C	-1.69618400	3.08591100	-0.87722700
C	-0.75157400	4.02482300	-1.31582200
C	-2.98091000	3.52723600	-0.52951100
C	-1.07872600	5.37277600	-1.40139400
H	0.24945300	3.68048800	-1.56647300
C	-3.30585200	4.87636500	-0.61287600
H	-3.73069100	2.81746600	-0.19126400
C	-2.35631400	5.80173500	-1.04717300
H	-0.33521900	6.08928700	-1.73465000
H	-4.30236200	5.20861200	-0.33974800
H	-2.61338400	6.85432300	-1.10861100
C	-7.14526500	2.28710600	-2.65245300
H	-6.59541700	3.22053800	-2.49312400
H	-7.36965800	2.21423000	-3.71860600
H	-8.08517800	2.35553000	-2.10111900
C	-5.76603500	-0.92197500	1.00096400
H	-6.21728000	-1.91837300	1.01835700
H	-4.78334300	-1.00652100	1.47718700
H	-6.38361400	-0.25297800	1.60156500
C	-3.57680400	-1.16405700	-3.55289900
H	-3.53455800	-2.24987200	-3.43276100
H	-3.82598900	-0.94225500	-4.59132900
H	-2.57565200	-0.76381000	-3.35820700
H	-1.30669800	-5.11333200	1.79403700
C	-0.28808200	-2.71833600	1.56104900
H	0.60336000	-3.25324700	1.90784200
H	0.05803900	-1.75189700	1.18913000
C	-1.26517800	-2.53037900	2.69840800
C	-2.12546700	-1.42583300	2.71565500
C	-1.33070300	-3.43679900	3.76173400
C	-3.04326600	-1.24874200	3.74869700
H	-2.05674900	-0.67648000	1.92868400
C	-2.24891100	-3.26508300	4.79519100
H	-0.64043000	-4.27594100	3.79326900
C	-3.11375300	-2.17383600	4.78778400

H	-3.69453000	-0.37996800	3.74743300
H	-2.27986800	-3.97964100	5.61123200
H	-3.82610700	-2.03572200	5.59430400
C	0.81444300	2.56476200	1.09182900
C	-0.16403000	1.59901500	1.22331100
C	0.57984800	3.94731200	1.60776700
C	-0.69067300	4.38349400	2.00772400
C	1.64087500	4.86139600	1.65467600
C	-0.89273100	5.68929900	2.43950400
H	-1.54362300	3.71768700	1.94685900
C	1.44119600	6.16452500	2.09739100
H	2.62230400	4.53419000	1.33123000
C	0.17236100	6.58619300	2.48908500
H	-1.88936000	6.00934300	2.72690600
H	2.27804800	6.85509400	2.13002800
H	0.01375600	7.60585800	2.82549900
N	2.01118400	2.43655500	0.45619600
S	2.41830000	1.10078700	-0.30781500
O	2.15629600	-0.15084200	0.47710000
O	1.91239800	1.03683100	-1.69114300
C	4.18535500	1.24097000	-0.39916400
C	4.91472000	1.75717800	0.66602600
C	4.81917500	0.68582500	-1.50762800
C	6.30429500	1.71274500	0.61488600
H	4.39541000	2.18846200	1.51553300
C	6.20695200	0.64789300	-1.54055800
H	4.22590800	0.28377000	-2.32351700
C	6.96695000	1.15257400	-0.48068800
H	6.88419400	2.11105700	1.44270200
H	6.71026200	0.20201600	-2.39401900
C	8.47023700	1.08801300	-0.53238900
H	8.79906300	0.11417100	-0.90604800
H	8.87123800	1.85154100	-1.20648900
H	8.90916300	1.24719000	0.45481900
H	0.06582800	0.56813400	0.99004800
H	-1.02683500	1.78894200	1.84723200
C	4.42211900	-2.40769400	-0.49916000
C	5.33102900	-2.75265000	-1.50856400

C	6.71648700	-2.70918200	-1.25977300
C	7.11566900	-2.29963100	-0.00698700
C	6.17605500	-1.92245000	0.99128800
C	4.81631900	-1.96897400	0.77519300
H	7.42761600	-2.98256400	-2.03168700
H	8.17349500	-2.24913000	0.22980100
H	6.54700800	-1.58187700	1.95252100
H	4.08795000	-1.66133500	1.51568700
N	4.62576100	-3.09523200	-2.63740000
N	3.36229400	-2.97889400	-2.37546200
N	3.22319900	-2.57081900	-1.11658900
O	1.98742700	-2.50010300	-0.57314900
H	1.86323100	-1.53693200	-0.31998400

TS6(S)$^{HOBt''}$

0 1

C	2.46479500	1.09534000	-1.11765200
C	3.40350200	3.07184600	-1.15070300
C	0.94002900	3.11231100	-1.58852000
H	0.52367900	2.67665700	-2.50056700
N	4.35266400	2.25228500	-0.80421300
N	3.75768500	1.03433100	-0.77900500
N	2.21747100	2.41130900	-1.32670200
C	3.51443800	4.53317900	-1.46177000
H	4.47967100	4.72672500	-1.93038900
H	3.43192400	5.13441800	-0.54637300
C	1.23205300	4.61472400	-1.80218100
O	2.49542700	4.85639200	-2.37941500
C	4.55146800	-0.09833600	-0.35807300
C	4.40766700	-0.53872600	0.95514100
C	5.42287900	-0.67168900	-1.28088800
C	5.19118300	-1.62264600	1.34427800
C	6.17923600	-1.75689200	-0.84415800
C	6.06616000	-2.25108900	0.45743800
H	5.09608300	-1.99898200	2.35878600
H	6.86122700	-2.23620300	-1.54208500
H	0.47587100	5.02457500	-2.47175800
C	1.66520300	-0.16953200	-1.40184700

O	2.30152200	-1.21441400	-1.23640300
C	0.34058800	-0.13259400	-1.90542800
C	-0.18852900	-1.36300500	-2.28237000
H	-0.25362500	0.76736600	-1.92009800
H	0.54057700	-2.14239900	-2.48370100
C	-1.48707900	-1.51002800	-2.96995300
C	-1.73668600	-2.66502700	-3.72238500
C	-2.48675900	-0.53257000	-2.88995200
C	-2.94020600	-2.82381000	-4.40325700
H	-0.97547900	-3.43916900	-3.77461500
C	-3.68736800	-0.68579600	-3.57487300
H	-2.33295400	0.33126600	-2.25534200
C	-3.91597100	-1.82942800	-4.33943400
H	-3.11594600	-3.72226700	-4.98582400
H	-4.45323200	0.07820800	-3.48949300
H	-4.85447300	-1.95238000	-4.86980300
C	6.83945700	-3.47097900	0.88387600
H	6.98828100	-3.48790000	1.96587000
H	6.28865900	-4.37794700	0.61179300
H	7.81599600	-3.51587800	0.39607200
C	3.40815600	0.09818100	1.87982300
H	3.52441300	1.18716400	1.91285700
H	2.38727100	-0.13004400	1.54600000
H	3.52404700	-0.29213600	2.89338000
C	5.48901400	-0.16632400	-2.69617100
H	5.76562400	0.89127900	-2.73157800
H	6.21934000	-0.73415500	-3.27403700
H	4.51228200	-0.27077500	-3.18088200
H	1.15884400	5.13675400	-0.83661400
C	-0.00126500	2.93744400	-0.39016800
H	-0.17763900	1.87466300	-0.20607000
H	0.49517500	3.34538300	0.49876400
C	-1.31781600	3.64104700	-0.61924000
C	-1.67084900	4.77135700	0.12074800
C	-2.21042900	3.16406700	-1.58321000
C	-2.89988800	5.39666900	-0.07962600
H	-0.98689800	5.14822000	0.87728100
C	-3.43998900	3.78286100	-1.78555800

H	-1.94186800	2.29876500	-2.18285100
C	-3.78952700	4.90022500	-1.02897400
H	-3.16332200	6.26859200	0.51038200
H	-4.12573500	3.39023100	-2.52941700
H	-4.75031100	5.38101100	-1.17977100
C	0.21229900	-2.10043700	0.51890600
C	-0.69002800	-2.53346400	-0.44267400
C	1.36171400	-2.95493200	0.93341700
C	1.95927300	-3.87194000	0.05993700
C	1.81585700	-2.88928400	2.25417800
C	2.98604600	-4.69883800	0.49849200
H	1.64047600	-3.90554400	-0.97700700
C	2.83534500	-3.72840400	2.69783800
H	1.34537600	-2.18485000	2.93262900
C	3.42547800	-4.63497800	1.82182100
H	3.45000600	-5.39420300	-0.19438200
H	3.16725400	-3.67273600	3.73039000
H	4.22325800	-5.28715100	2.16423900
N	0.13554000	-0.90054900	1.15190200
S	-1.31353500	-0.23765900	1.34962600
O	-1.89836500	0.34537100	0.09575100
O	-2.25750100	-1.10770200	2.06644200
C	-0.92915000	1.16702900	2.36737900
C	-1.82915700	2.22965900	2.34729100
C	0.18548300	1.18335100	3.20078800
C	-1.59700200	3.32619000	3.16977600
H	-2.68429400	2.20753200	1.67751100
C	0.40808600	2.29491100	4.00702900
H	0.87021500	0.34324500	3.19871300
C	-0.47764500	3.37743500	4.00675800
H	-2.29026300	4.16259400	3.14895600
H	1.28396900	2.32162100	4.64916100
C	-0.24740500	4.56321500	4.90653000
H	-0.59873300	5.48682700	4.44058400
H	-0.79350300	4.44111400	5.84735300
H	0.81110200	4.67624700	5.15008800
H	-1.66676000	-2.07010400	-0.49850200
H	-0.62863100	-3.55313800	-0.80813100

C	-4.69003600	-2.07068700	0.30408500
C	-5.16941200	-2.57317800	1.52127400
C	-5.23185100	-3.96150900	1.73832500
C	-4.80129600	-4.77967500	0.71572700
C	-4.31787700	-4.24887300	-0.50915600
C	-4.25300900	-2.89192500	-0.74709600
H	-5.60111600	-4.35935300	2.67710200
H	-4.82869900	-5.85657900	0.84275400
H	-3.98881600	-4.93410600	-1.28405200
H	-3.88621100	-2.48105400	-1.68070100
N	-5.51196800	-1.51537800	2.33028800
N	-5.26511100	-0.42104100	1.68670300
N	-4.78912100	-0.72984500	0.48403400
O	-4.43902100	0.24636300	-0.38273000
H	-3.45726400	0.36860900	-0.21409000

TS6(R)$^{HOBt'''}$

0 1

C	1.81680800	1.00817100	-1.29921700
C	1.45704200	3.15763000	-1.44868200
C	-0.51524600	1.76099400	-2.08633900
H	-0.48344100	0.91836700	-2.78112100
N	2.69791400	3.05212100	-1.07389600
N	2.91442900	1.71110300	-0.98161200
N	0.87369800	1.92897900	-1.60139000
C	0.70996100	4.36608100	-1.90790200
H	1.40954300	5.17087800	-2.13122500
H	-0.01143900	4.70351300	-1.14905200
C	-0.97460300	3.05871700	-2.82840000
O	0.05937000	3.97849800	-3.10000900
C	4.27910400	1.25711400	-0.78388000
C	4.82866400	1.26422400	0.49681900
C	4.99745900	0.88169800	-1.92198700
C	6.14015900	0.80776200	0.62768900
C	6.30176900	0.43355400	-1.73555900
C	6.88005200	0.36552000	-0.46662100
H	6.58474700	0.78494200	1.61892900
H	6.87604700	0.12038000	-2.60382100

H	-1.41580100	2.78645900	-3.78691700
C	1.79139600	-0.50153100	-1.32077800
O	2.90364300	-1.03895200	-1.22911900
C	0.58156300	-1.22786000	-1.37718200
C	0.72349600	-2.61228400	-1.25523200
H	-0.39130100	-0.76617900	-1.36917100
H	1.70275600	-2.99738600	-1.52426200
C	-0.39845000	-3.54987500	-1.47365200
C	-0.11986100	-4.81044700	-2.01538500
C	-1.72929800	-3.21139300	-1.19084600
C	-1.14788300	-5.70192900	-2.30553900
H	0.91192600	-5.08346200	-2.22080300
C	-2.75760800	-4.10052400	-1.48711300
H	-1.95785300	-2.26083800	-0.71613300
C	-2.46971600	-5.34395000	-2.05022900
H	-0.91728800	-6.67261300	-2.73259400
H	-3.78206800	-3.82051300	-1.26451100
H	-3.27409500	-6.03580900	-2.27874800
C	8.26250300	-0.19893300	-0.27513400
H	8.78711100	0.30395700	0.54067500
H	8.19902700	-1.26244100	-0.01979400
H	8.86136600	-0.10814600	-1.18393100
C	4.05493100	1.72615400	1.69851600
H	3.62026100	2.71800900	1.53649100
H	3.25115600	1.01818300	1.92145500
H	4.70818300	1.77687900	2.57168400
C	4.37226100	0.90983600	-3.28982200
H	3.82803600	1.84231900	-3.46941500
H	5.13717000	0.80994400	-4.06127900
H	3.66945700	0.07748000	-3.39552500
H	-1.74504100	3.54839300	-2.21651100
C	-1.46930300	1.48156600	-0.91880500
H	-1.10744700	0.64353400	-0.32237400
H	-1.47318500	2.36231300	-0.26649700
C	-2.87060600	1.18944100	-1.39948000
C	-3.93094100	2.02979600	-1.05369000
C	-3.12976900	0.06620200	-2.19286000
C	-5.22221800	1.76552700	-1.50425400

H	-3.73937400	2.89322600	-0.42027800
C	-4.41979300	-0.20431200	-2.63865700
H	-2.32231100	-0.61033500	-2.46533700
C	-5.46673800	0.65173700	-2.30267100
H	-6.03938900	2.42032000	-1.21935400
H	-4.60660000	-1.08560100	-3.24366000
H	-6.47414200	0.44095100	-2.64653300
C	1.93431100	-1.91058100	1.28841100
C	1.19778300	-2.99956600	0.82114000
C	3.39949000	-2.03765700	1.53746700
C	4.23553000	-2.79719700	0.71067400
C	3.94027400	-1.43747500	2.67843600
C	5.58512900	-2.93470900	1.01063400
H	3.82717400	-3.24039100	-0.19157400
C	5.29153800	-1.58325300	2.98403200
H	3.28509900	-0.86812700	3.33073600
C	6.11905400	-2.32858500	2.14902900
H	6.22682100	-3.51080700	0.35095800
H	5.69530900	-1.11468400	3.87650400
H	7.17415800	-2.43823400	2.38106600
N	1.41916100	-0.67702900	1.54369000
S	-0.07920500	-0.54621300	2.09362700
O	-1.14618100	-1.09064000	1.18479600
O	-0.22950400	-1.03601400	3.47110200
C	-0.36694100	1.21218500	2.09443600
C	-1.65069200	1.61475700	2.47001600
C	0.57689200	2.14076400	1.68529500
C	-1.97710000	2.96272900	2.42864000
H	-2.38916400	0.87707900	2.77202500
C	0.23668100	3.49408400	1.65803800
H	1.55953500	1.80719300	1.38273600
C	-1.04042600	3.92446500	2.02120800
H	-2.97699400	3.28044000	2.71168600
H	0.98252700	4.22352400	1.35028400
C	-1.41801400	5.38145300	1.97823400
H	-2.29068100	5.53729200	1.33713800
H	-1.67882300	5.74350000	2.97672700
H	-0.59760300	5.99293000	1.59769400

H	0.12990800	-3.02645300	0.99463800
H	1.69835900	-3.96029200	0.75938900
C	-5.37740600	-0.54024800	1.41221900
C	-6.17805900	-1.14212400	0.43113200
C	-7.38933300	-0.54090100	0.04141200
C	-7.73014200	0.64436500	0.65513900
C	-6.89836100	1.24174000	1.64048300
C	-5.71177400	0.66784400	2.04360900
H	-8.01938600	-1.00209500	-0.71131300
H	-8.65668800	1.14047400	0.38660800
H	-7.21622100	2.17437000	2.09489100
H	-5.08359500	1.10464800	2.81087300
N	-5.57581700	-2.30990300	0.02880700
N	-4.48994700	-2.46061000	0.71924200
N	-4.34785600	-1.41499200	1.52799600
O	-3.37104800	-1.38819000	2.46077800
H	-2.50119900	-1.32762900	1.95352300

TS6(S)$^{HOBt'''}$

0 1

C	-2.85699000	-2.08109800	0.06935800
C	-4.70673200	-3.20408800	0.32404800
C	-5.03671000	-0.86452600	-0.55178200
H	-5.00926600	-0.09085800	0.22023300
N	-3.75632900	-4.02972300	0.65845100
N	-2.61190400	-3.32066200	0.48186500
N	-4.19721400	-1.99078400	-0.06353700
C	-6.18739700	-3.43876900	0.36558900
H	-6.49876400	-4.01727500	-0.51478600
H	-6.44073100	-3.99840800	1.26656700
C	-6.47176400	-1.39212300	-0.68593000
O	-6.84281200	-2.19299900	0.41547500
C	-1.33473100	-3.93700200	0.75975200
C	-0.66319000	-3.59199100	1.93024700
C	-0.85228600	-4.86010400	-0.16955200
C	0.55400400	-4.22933200	2.16918500
C	0.35746900	-5.47799800	0.12590100
C	1.07346300	-5.17474900	1.28659500

H	1.11306700	-3.97192200	3.06413800
H	0.76075800	-6.20268600	-0.57737600
H	-6.56945100	-1.96463900	-1.62099800
C	-1.76117300	-1.04793600	-0.13143800
O	-0.70962300	-1.47291500	-0.60237100
C	-2.06505800	0.28305600	0.27373700
C	-1.12812800	1.27373700	0.06246500
H	-0.23959600	1.00074100	-0.50571000
C	-1.44088900	2.70251300	0.15383000
C	-0.82328400	3.57946400	-0.74537000
C	-2.37193200	3.20908900	1.07243500
C	-1.16200900	4.92968500	-0.75745100
H	-0.08766600	3.19059300	-1.44379900
C	-2.68811400	4.56083100	1.07719300
H	-2.82456200	2.54199000	1.80165600
C	-2.09285700	5.42207500	0.15290100
H	-0.68911500	5.59905600	-1.46884600
H	-3.39483300	4.94834700	1.80389200
H	-2.34571600	6.47763800	0.15505800
C	2.38606400	-5.85897800	1.56116000
H	2.83753100	-5.49101100	2.48353400
H	3.09259900	-5.66908800	0.74832100
H	2.25026100	-6.94196800	1.64207800
C	-1.22233900	-2.57134000	2.88605800
H	-2.26810700	-2.78242400	3.13091200
H	-1.17591300	-1.56086100	2.46263100
H	-0.64709300	-2.56419000	3.81268500
C	-1.59977500	-5.14114300	-1.44505700
H	-2.58771900	-5.56763100	-1.24846100
H	-1.04059000	-5.83996600	-2.06845900
H	-1.74197500	-4.21602600	-2.01398400
H	-7.15221000	-0.54079400	-0.72394900
C	-4.53554700	-0.31290200	-1.89444300
H	-4.72977800	-1.05532400	-2.67565500
H	-3.45654900	-0.15151000	-1.84930100
C	-5.21533200	1.00108400	-2.20718600
C	-4.73017500	2.17691000	-1.62704700
C	-6.33519800	1.06601000	-3.03804900

C	-5.35444800	3.39616800	-1.86936400
H	-3.84777700	2.13891100	-0.99218500
C	-6.96241000	2.28648100	-3.28229500
H	-6.71465000	0.15857800	-3.50167000
C	-6.47440000	3.45265900	-2.69769500
H	-4.95846600	4.29952500	-1.41511200
H	-7.83067400	2.32472600	-3.93209700
H	-6.96192600	4.40282400	-2.89020500
C	1.08209100	2.14123300	1.79704500
C	0.31112600	1.01811800	1.99137700
C	0.67620700	3.41917000	2.45522000
C	-0.02371400	3.42783300	3.66870000
C	0.99474000	4.64415200	1.85945400
C	-0.41547200	4.62608500	4.25610700
H	-0.24103400	2.49172600	4.17266100
C	0.59177900	5.84217800	2.43955800
H	1.54635600	4.63885600	0.92619400
C	-0.11708000	5.83902900	3.63889800
H	-0.94877600	4.61220300	5.20163600
H	0.83069900	6.78248200	1.95176200
H	-0.42648300	6.77464600	4.09457600
N	2.15992800	2.27849800	0.96804000
S	2.78948400	0.93846900	0.35758500
O	1.86500300	0.24188200	-0.58999200
O	3.38427400	0.04823600	1.36967300
C	4.11477600	1.56480600	-0.64554200
C	3.82543200	2.16080900	-1.86978900
C	5.42401600	1.38641600	-0.22120800
C	4.87305700	2.58524200	-2.67654000
H	2.79382100	2.27393400	-2.18798900
C	6.46242600	1.82010200	-1.04039300
H	5.61648800	0.89340900	0.72585900
C	6.20404100	2.41468100	-2.27668800
H	4.66057400	3.04960300	-3.63570700
H	7.49134800	1.67601500	-0.72154200
C	7.33686000	2.82288000	-3.18223400
H	8.20705700	3.14666500	-2.60661500
H	7.03989500	3.63497400	-3.84967600

H	7.65008000	1.97847500	-3.80599100
H	-0.52008900	1.04819000	2.68379500
H	-3.01957600	0.49520600	0.74359100
H	0.60285700	0.04612500	1.60964000
C	4.12085500	-2.05605700	-0.84556000
C	5.23399900	-2.32560800	-0.04006900
C	6.52916500	-2.03679500	-0.50981500
C	6.63319900	-1.47716300	-1.76525700
C	5.48815200	-1.19346600	-2.55633600
C	4.21171000	-1.47493800	-2.11937900
H	7.40085600	-2.24541400	0.10113500
H	7.61248100	-1.22772500	-2.16158600
H	5.62661500	-0.72736600	-3.52689600
H	3.32535300	-1.24202700	-2.69735200
N	4.79539400	-2.86927300	1.14423000
N	3.50184100	-2.92805900	1.10499000
N	3.08865900	-2.46653200	-0.06851700
O	1.77381200	-2.45136900	-0.37513700
H	1.51779500	-1.49512600	-0.35988500

TS6(R)^{HOBt}$_{L3}$

0 1

C	1.55036300	0.07844000	1.67052800
C	-0.12641800	0.31792200	3.05862400
C	0.09490100	-1.99857900	2.08631300
H	0.05122000	-2.16459000	1.00770900
N	0.48836400	1.47031400	3.08239200
N	1.54850100	1.30364500	2.22187300
N	0.52582000	-0.58660100	2.25824100
C	-1.46122700	-0.02822400	3.64514400
H	-2.09405200	0.86089100	3.65786100
H	-1.35890800	-0.42443100	4.66656100
C	-1.30784300	-2.17255600	2.73590500
O	-2.07461000	-0.98013600	2.78727700
C	2.54975800	2.34057400	2.12704500
C	3.80739800	2.10342000	2.70676000
C	2.20828400	3.56084000	1.51966000
C	4.75252200	3.13084600	2.63257800

C	3.19335400	4.55121200	1.48036400
C	4.46955600	4.35567000	2.02023000
H	5.73158800	2.96951700	3.07549000
H	2.95309000	5.50239200	1.01297600
H	-1.87957200	-2.89362600	2.15102400
C	2.39927100	-0.43832300	0.52881100
O	2.72381800	-1.64535200	0.54002100
C	2.70390600	0.50270500	-0.48567100
C	3.34279700	0.07568800	-1.65602900
H	2.38659100	1.52997300	-0.37617900
H	3.77577800	-0.91853900	-1.60452700
C	4.01671900	1.00701100	-2.58685600
C	5.16107500	0.57443100	-3.27815600
C	3.56349100	2.32201100	-2.79897200
C	5.84598500	1.43271300	-4.13810800
H	5.51984900	-0.44059900	-3.12903300
C	4.24486800	3.17698400	-3.66175900
H	2.66017800	2.66531600	-2.30444900
C	5.39122700	2.73832500	-4.33194700
H	6.73326500	1.08125600	-4.65649600
H	3.87876700	4.18776200	-3.81633200
H	5.92066000	3.40763500	-5.00343000
C	5.51930600	5.43597100	1.92688700
H	5.07485900	6.43215300	2.00668600
H	6.27281400	5.33052600	2.71194800
H	6.04051800	5.38893100	0.96302000
C	4.14431700	0.80639000	3.40338000
H	3.36495000	0.51757800	4.11611000
H	4.26491000	-0.02069000	2.69535300
H	5.08195800	0.90630300	3.95383700
C	0.84583200	3.80766300	0.92186000
H	0.05635500	3.74086600	1.67523400
H	0.80551700	4.80126000	0.47045700
H	0.60298000	3.07198200	0.14860500
H	-1.19041000	-2.57135000	3.75379700
C	1.11560000	-2.94815800	2.75925100
H	2.08829500	-2.75144100	2.30584100
H	1.17034400	-2.69236500	3.82341600

C	0.75538600	-4.41182200	2.60269100
C	0.28112800	-5.16180800	3.68776500
C	0.90870200	-5.05030200	1.36251800
C	-0.03701400	-6.51384700	3.54091300
H	0.17001900	-4.68700500	4.65940800
C	0.58930600	-6.40000500	1.21292500
H	1.28841200	-4.48907300	0.51358300
C	0.11447900	-7.13659700	2.30120900
H	-0.39935300	-7.07843000	4.39520300
H	0.71571200	-6.87443100	0.24453600
H	-0.13072500	-8.18816400	2.18423700
C	0.88764900	-1.37553300	-2.27315400
C	1.73401900	-0.56764400	-3.04055500
C	0.99440700	-2.86161600	-2.37096100
C	2.18488200	-3.50005600	-2.76270400
C	-0.13896900	-3.65625200	-2.12825600
C	2.23608300	-4.88403600	-2.91390600
H	3.08145200	-2.91484600	-2.93671100
C	-0.09081300	-5.04023400	-2.29346300
H	-1.06122700	-3.17117000	-1.82885300
C	1.09650500	-5.66119900	-2.68703200
H	3.16812800	-5.35747300	-3.20835700
H	-0.98330600	-5.63361000	-2.11729700
H	1.13483000	-6.73874500	-2.81730100
N	-0.01271100	-0.94637600	-1.35635500
S	-0.75920000	0.49719200	-1.38210600
O	-1.00284400	0.84674800	0.05755100
O	-0.17500900	1.58043800	-2.20844700
C	-2.37876400	0.12848900	-2.06436600
C	-3.34326800	-0.48053300	-1.25492200
C	-2.64576800	0.41881400	-3.40203300
C	-4.58527900	-0.79255400	-1.80017700
H	-3.12636600	-0.68759600	-0.21236000
C	-3.89616400	0.09762100	-3.93072400
H	-1.88967600	0.89795800	-4.01387100
C	-4.88369800	-0.50837800	-3.14274900
H	-5.33945700	-1.25841900	-1.17189200
H	-4.10769100	0.32687200	-4.97145000

C	-6.24722900	-0.82077100	-3.70889800
H	-6.22972400	-0.85247100	-4.80135500
H	-6.62113700	-1.78158700	-3.34241800
H	-6.97528200	-0.05642400	-3.41124300
H	1.46612600	0.46670000	-3.19080200
H	2.31014800	-1.03268200	-3.83173200
C	-4.76177800	2.74211300	0.49351000
C	-5.75353500	2.30516300	1.39396200
C	-7.10813900	2.31172200	1.01095900
C	-7.40421600	2.76412200	-0.26322700
C	-6.38836600	3.20357500	-1.15394000
C	-5.04944700	3.20249200	-0.79965300
H	-7.88051100	1.97635400	1.69497700
H	-8.43679300	2.78800800	-0.59685100
H	-6.67385200	3.55142100	-2.14188800
H	-4.26802300	3.53369700	-1.47334200
N	-5.14853100	1.92916100	2.57482700
N	-3.86289000	2.11443300	2.45147200
N	-3.61491300	2.58065100	1.21072900
O	-2.36147200	2.96020100	0.84279400
H	-1.89144300	2.13413800	0.51872500

TS6(S)^{HOBt}$_{L3}$

0 1

C	2.42426000	2.03134400	0.16808300
C	4.34410500	2.48465500	-0.79877100
C	4.50591100	0.80711000	1.08248400
H	4.19781700	1.12730500	2.07579500
N	3.47872000	3.25460400	-1.40318900
N	2.28042700	2.95711200	-0.79963700
N	3.74634900	1.70728300	0.16043200
C	5.83610000	2.50971700	-0.92051200
H	6.15467500	3.48566600	-1.28935300
H	6.19564400	1.73316900	-1.61066000
C	6.02833500	1.03334400	0.87493800
O	6.36293200	2.32852900	0.38545900
C	1.12025100	3.73512300	-1.17847300
C	0.48232900	3.43785900	-2.39444200

C	0.73876700	4.80719700	-0.35548700
C	-0.61518800	4.22959800	-2.74588500
C	-0.36459600	5.56256900	-0.76071100
C	-1.06149600	5.28322800	-1.94130400
H	-1.12896800	4.01708100	-3.67946100
H	-0.68130300	6.39486900	-0.13812100
H	6.52232000	0.92831000	1.84250100
C	1.44180900	1.59418000	1.24320000
O	1.97035200	1.26745100	2.32709600
C	0.05153900	1.67799800	0.98344800
C	-0.84554900	1.46697500	2.03518700
H	-0.29607600	1.92729000	-0.00909500
H	-0.39683800	1.42572100	3.02240300
C	-2.25166400	1.91103000	2.00212700
C	-2.87309500	2.29025300	3.20509500
C	-2.99437600	1.99380100	0.80947000
C	-4.18689100	2.75666400	3.21772600
H	-2.31390600	2.22516300	4.13487000
C	-4.30875600	2.45405100	0.82425500
H	-2.54782100	1.66706300	-0.12381600
C	-4.90986100	2.84125200	2.02633800
H	-4.64578900	3.05099600	4.15710000
H	-4.87248200	2.50009000	-0.10260200
H	-5.93562200	3.19752400	2.03333600
C	-2.27442000	6.09190200	-2.33121200
H	-3.18519600	5.65004000	-1.90899100
H	-2.20781500	7.11785100	-1.95893900
H	-2.40067600	6.12543200	-3.41680800
C	0.95105200	2.32412200	-3.29840700
H	1.99832200	2.46113800	-3.58644100
H	0.86640500	1.34782700	-2.80980000
H	0.34944900	2.30158900	-4.20973800
C	1.47406100	5.14576600	0.91795400
H	2.55863000	5.15344700	0.77156200
H	1.17596200	6.13272100	1.27765700
H	1.25197800	4.42385700	1.71163500
H	6.43085400	0.27505700	0.19335100
C	4.11118500	-0.68424900	0.92995800

H	4.52797700	-1.18736900	1.80949100
H	3.02449100	-0.76321300	1.02201300
C	4.59068900	-1.38984700	-0.32350400
C	3.93956500	-1.20415200	-1.55294900
C	5.69486900	-2.25466200	-0.27834000
C	4.39336200	-1.85060100	-2.70500600
H	3.04933900	-0.58524500	-1.59975700
C	6.14767500	-2.90542800	-1.42787200
H	6.20120600	-2.42563800	0.66879900
C	5.50050900	-2.70075700	-2.64769300
H	3.87422500	-1.69539500	-3.64671200
H	7.00247700	-3.57300000	-1.36824300
H	5.85001300	-3.20547900	-3.54361300
C	-0.04717900	-1.35183200	1.85388500
C	-1.28358500	-0.77915300	2.14707900
C	0.81245700	-1.88891400	2.95430900
C	0.66994000	-1.46421000	4.28684300
C	1.77083700	-2.87724800	2.67190300
C	1.45576100	-2.01060800	5.29961400
H	-0.04418300	-0.68655400	4.53467400
C	2.55347900	-3.42821200	3.68646500
H	1.88513800	-3.21269300	1.64749200
C	2.40069600	-2.99745400	5.00576600
H	1.33397000	-1.66030300	6.32062300
H	3.28043100	-4.19897300	3.44563900
H	3.01022300	-3.42450600	5.79685800
N	0.53557800	-1.41643800	0.63267500
S	-0.21851800	-1.36538800	-0.79957600
O	-1.62429100	-0.82769200	-0.78957500
O	0.68726400	-0.71835400	-1.77573400
C	-0.34027100	-3.09729700	-1.26932400
C	-1.43562000	-3.86153800	-0.85464300
C	0.69564500	-3.67488400	-2.00517300
C	-1.48552300	-5.21206800	-1.19117800
H	-2.24878800	-3.41369100	-0.29463500
C	0.62728000	-5.02959900	-2.33070600
H	1.53898600	-3.07141300	-2.32142700
C	-0.45850400	-5.81978300	-1.93015900

H	-2.34228000	-5.80262800	-0.87751300
H	1.43109500	-5.47789300	-2.90829700
C	-0.52565500	-7.28826500	-2.27329300
H	0.27610400	-7.57632200	-2.95808700
H	-0.43537900	-7.90686200	-1.37236100
H	-1.48252400	-7.54312800	-2.74135100
H	-2.00198400	-0.64199600	1.35526700
H	-1.68188000	-0.87847100	3.14949600
C	-5.84497500	-0.91133800	-0.98472600
C	-6.50005700	-1.43127900	0.15048300
C	-7.72492400	-0.88109600	0.57345100
C	-8.23989400	0.16671700	-0.16989800
C	-7.56592200	0.67209900	-1.31444000
C	-6.35845500	0.14849100	-1.74759400
H	-8.23696300	-1.27164600	1.44662100
H	-9.18347500	0.61743300	0.12125500
H	-8.01535200	1.49231200	-1.86581400
H	-5.84032200	0.52431800	-2.62222600
N	-5.74843500	-2.46589000	0.66427900
N	-4.69201500	-2.61488100	-0.08777400
N	-4.72806000	-1.68541800	-1.06405000
O	-3.78694200	-1.65659700	-2.04711600
H	-2.94435700	-1.33137800	-1.61170700

TS6(R)$^{HOBt}{}_{L4}$

0 1

C	-1.49914500	2.08311200	-0.45709500
C	-2.64333100	2.07597400	-2.30577300
C	-3.60321600	0.64814700	-0.48747700
N	-1.66392200	2.90843600	-2.52084200
N	-0.95842000	2.90537400	-1.35571500
N	-2.59461900	1.57557200	-1.03683400
C	-3.64246700	1.54994800	-3.29472400
H	-4.08304100	2.36979400	-3.86474900
H	-3.10326800	0.88666000	-3.98675200
C	-4.17924200	-0.08074800	-1.69478800
O	-4.68045900	0.86042600	-2.63717600
C	0.11939200	3.83977900	-1.16512400

C	1.29173400	3.66850700	-1.90659500
C	-0.07377100	4.89333800	-0.26440500
C	2.30991400	4.60118100	-1.70919900
C	0.97696700	5.79467800	-0.10491300
C	2.17391700	5.66134900	-0.81167400
H	3.23436300	4.49058000	-2.27030500
H	0.85252500	6.62535200	0.58526700
H	-5.01802700	-0.70638000	-1.38455600
C	-0.98538000	1.63998500	0.88989500
O	-1.83114100	1.36080000	1.75135900
C	0.41922000	1.49513200	0.98931300
C	0.92946900	0.82606000	2.09815600
H	1.05107100	1.77231400	0.15440800
H	0.25960400	0.74761900	2.95142100
C	2.37411700	0.83499900	2.42240800
C	2.77195000	0.79554700	3.76207500
C	3.35539800	0.90301100	1.42232100
C	4.12080200	0.85855900	4.10417800
H	2.01457700	0.72659000	4.53896100
C	4.70034800	0.97046000	1.76573700
H	3.05842400	0.89021200	0.37560900
C	5.08721700	0.95343700	3.10709100
H	4.41412900	0.83467400	5.14913700
H	5.45593900	1.02175500	0.98614500
H	6.14021900	1.00082000	3.36773500
C	3.29881400	6.63696100	-0.59279600
H	3.85737100	6.38242400	0.31426000
H	2.91888500	7.65441500	-0.46888500
H	4.00061500	6.62766300	-1.42974500
C	1.45271800	2.52070900	-2.86410000
H	0.73531900	2.59088500	-3.68702000
H	1.27498900	1.56205000	-2.36719700
H	2.46152100	2.51922100	-3.28141100
C	-1.36285100	5.06516000	0.49462900
H	-2.23054200	4.94268500	-0.16138200
H	-1.40750600	6.06206800	0.93648700
H	-1.45270400	4.33739300	1.30861000
H	-3.40464300	-0.71344200	-2.15165600

C	-0.50946800	-1.39566800	1.12707500
C	0.77049700	-1.28541900	1.65799700
C	-1.62503100	-1.90589500	1.97342000
C	-1.60920000	-1.77328100	3.36766900
C	-2.68977500	-2.58715900	1.37038900
C	-2.62262600	-2.33014000	4.14039700
H	-0.80524700	-1.22396000	3.84854700
C	-3.69721200	-3.15393300	2.14606000
H	-2.70188000	-2.68700100	0.28872200
C	-3.66317300	-3.03378300	3.53362400
H	-2.59957600	-2.21682200	5.21994300
H	-4.51083000	-3.68922100	1.66591200
H	-4.44600900	-3.47960900	4.13967400
N	-0.89903200	-0.98743600	-0.10282500
S	0.03867600	-0.93126800	-1.39896100
O	1.47412800	-0.60022100	-1.13794500
O	-0.61679900	-0.07011500	-2.39062700
C	0.03296800	-2.59224200	-2.04080000
C	0.75736400	-3.58235300	-1.37472900
C	-0.73127500	-2.88437900	-3.16371100
C	0.70400800	-4.88369000	-1.85420100
H	1.36033700	-3.34105900	-0.50323600
C	-0.76743300	-4.19620600	-3.63139900
H	-1.27843900	-2.09294600	-3.66529800
C	-0.05624300	-5.20972800	-2.98574300
H	1.26545100	-5.66286700	-1.34572000
H	-1.35746900	-4.43457100	-4.51177500
C	-0.08978300	-6.62730800	-3.48980700
H	-0.77606000	-6.73350900	-4.33256700
H	-0.40594200	-7.31460700	-2.69943300
H	0.90477700	-6.94592700	-3.81773600
H	1.61616900	-1.18993200	0.99268300
H	0.97195300	-1.74106000	2.62196000
C	5.39288900	-1.61155500	-0.34631700
C	5.57726700	-2.03124400	0.97881000
C	6.77312200	-1.72894100	1.65700900
C	7.72521600	-1.01570000	0.96198200
C	7.51663000	-0.59954100	-0.38188200

C	6.35418800	-0.88409900	-1.06624500
H	6.92146800	-2.04151700	2.68515100
H	8.66224500	-0.76006100	1.44551000
H	8.30158100	-0.04107000	-0.88169200
H	6.18595000	-0.57244000	-2.09039800
N	4.44622200	-2.69083300	1.39211500
N	3.61109800	-2.71489000	0.40253000
N	4.14771700	-2.06614400	-0.62780500
O	3.52594800	-2.01601300	-1.82577100
H	2.71513800	-1.44691600	-1.66968400
C	-4.64062800	1.47453700	0.28488500
H	-4.09327800	2.06740000	1.02313500
H	-5.13561400	2.15537200	-0.41497600
C	-5.66201700	0.60776300	0.97636900
C	-5.33018100	-0.06362200	2.15709200
C	-6.94753200	0.45587800	0.44873300
C	-6.27649800	-0.85079500	2.80896900
H	-4.32546300	0.03845700	2.56523800
C	-7.89213600	-0.33814800	1.09519500
H	-7.20787300	0.97115900	-0.47283200
C	-7.55925100	-0.98896000	2.28127600
H	-6.00968600	-1.35757000	3.73115700
H	-8.88821400	-0.44305800	0.67609200
H	-8.29586100	-1.60090200	2.79263800
H	-3.07656800	-0.04845800	0.16934000

TS6(S)$^{HOBt}{}_{L4}$

0 1

C	1.63190800	2.45237300	0.12648200
C	3.34857300	3.27455100	-0.93845400
C	3.96731400	1.86596100	1.05258700
H	3.77856900	2.42058300	1.97283300
N	2.30906000	3.79450800	-1.52352500
N	1.24855000	3.27589800	-0.85202300
N	2.98180100	2.43034900	0.07761800
C	4.78983300	3.59104900	-1.19758600
H	4.86978400	4.61421300	-1.56740100
H	5.20353700	2.90421500	-1.94884700

C	5.37319600	2.17573200	0.52794400
O	5.48805500	3.49320600	0.02239100
C	-0.07947200	3.73671000	-1.16494400
C	-0.63150000	3.39018800	-2.40141200
C	-0.75190300	4.51624000	-0.21492800
C	-1.95697500	3.76489500	-2.62924300
C	-2.07059200	4.86322100	-0.49687100
C	-2.69750600	4.47232300	-1.68239700
H	-2.41928400	3.49075300	-3.57408000
H	-2.62172100	5.45430800	0.23051200
H	6.07336600	2.07357600	1.36015000
C	0.77527100	1.83145300	1.21961100
O	1.33146500	1.71180600	2.31810000
C	-0.57795500	1.53212600	0.91882700
C	-1.38891100	1.05244500	1.93863800
H	-0.94569800	1.64785300	-0.09181600
H	-1.00200400	1.15869700	2.94946100
C	-2.86403200	1.03358100	1.81503400
C	-3.64837800	1.17521100	2.96345700
C	-3.49988600	0.89467800	0.57126000
C	-5.03823300	1.21545700	2.87462900
H	-3.16127600	1.26353700	3.93124800
C	-4.88616000	0.93487500	0.48504800
H	-2.90405400	0.73496600	-0.32373000
C	-5.66025600	1.10514900	1.63488800
H	-5.63257900	1.33345200	3.77542600
H	-5.36958800	0.81420700	-0.48123000
H	-6.74328800	1.13249100	1.56172300
C	-4.14453700	4.80912100	-1.92142800
H	-4.78810200	4.16899900	-1.30839600
H	-4.35963300	5.84576500	-1.64797100
H	-4.42018900	4.66116800	-2.96779100
C	0.16421400	2.69021500	-3.46886900
H	0.79949600	3.40915400	-3.99676900
H	0.80183300	1.91071600	-3.05171300
H	-0.50886500	2.23494100	-4.19839100
C	-0.10270600	4.97019900	1.06570200
H	0.96261500	5.17826200	0.92967900

H	-0.58329100	5.88527500	1.41761200
H	-0.20644100	4.21909200	1.85579300
H	5.65275700	1.45906000	-0.25453100
C	3.79318200	0.36820700	1.34858200
H	4.21678500	0.21367200	2.34652200
H	2.72948900	0.14389900	1.42771300
C	4.43719100	-0.59894400	0.38465700
C	3.92096800	-0.78997400	-0.90244300
C	5.54651600	-1.35241100	0.78066500
C	4.50467000	-1.71227400	-1.76861200
H	3.04554400	-0.22706500	-1.21843200
C	6.12862600	-2.27883200	-0.08292400
H	5.95090300	-1.21535600	1.78091900
C	5.60820600	-2.46082900	-1.36182400
H	4.08542900	-1.85749900	-2.76004400
H	6.98579400	-2.85917700	0.24495200
H	6.05532100	-3.18462900	-2.03626900
C	0.24871300	-1.26517700	1.72828000
C	-1.10741600	-1.13524800	1.98242900
C	1.18820400	-1.63933600	2.82613200
C	0.92062700	-1.32049000	4.16267400
C	2.36441300	-2.33421300	2.51895500
C	1.80773500	-1.69193600	5.16746000
H	0.02659600	-0.75821600	4.41496200
C	3.24842100	-2.71089500	3.52623400
H	2.58112900	-2.56859300	1.48023800
C	2.97377600	-2.39029300	4.85430300
H	1.59234500	-1.42814300	6.19834800
H	4.15297800	-3.25504200	3.27033700
H	3.66341100	-2.68018200	5.64105300
N	0.87890000	-0.98203700	0.56616600
S	0.23688500	-0.90683300	-0.88966900
O	-1.25470000	-1.00164100	-0.96971100
O	0.78986400	0.24525800	-1.61620200
C	0.85975200	-2.36546100	-1.70739600
C	0.95120500	-3.57014100	-1.01312600
C	1.19944200	-2.28074700	-3.05281300
C	1.40030000	-4.70037200	-1.68412100

H	0.68893300	-3.62171400	0.03996200
C	1.64776400	-3.42421400	-3.70961300
H	1.12702800	-1.32969200	-3.57073400
C	1.75920300	-4.64367600	-3.03678800
H	1.48158500	-5.64283700	-1.14958300
H	1.92034100	-3.36627700	-4.75980000
C	2.27798700	-5.87161700	-3.73518000
H	2.29811900	-5.73354800	-4.81837400
H	3.29690100	-6.09965800	-3.40483400
H	1.65901000	-6.74387300	-3.50828400
H	-1.80941300	-1.14912800	1.16593300
H	-1.49230500	-1.45417600	2.94515300
C	-4.82270400	-2.37678100	-0.57911100
C	-5.73794100	-2.17934100	0.46245000
C	-7.04679500	-1.74579000	0.18345900
C	-7.36570000	-1.52482000	-1.13934800
C	-6.41825200	-1.72356600	-2.18023800
C	-5.13187500	-2.15288900	-1.92923400
H	-7.76180100	-1.59013200	0.98443000
H	-8.36344900	-1.18917900	-1.40225900
H	-6.72165800	-1.53251500	-3.20458700
H	-4.40140000	-2.30678400	-2.71508100
N	-5.10991800	-2.45109500	1.65502300
N	-3.89257400	-2.80549800	1.39937200
N	-3.69987800	-2.75767600	0.08190200
O	-2.54544300	-3.20694900	-0.45823800
H	-2.00439500	-2.38884700	-0.66067400

TS6(R)^{HOBt}$_{L5}$

0 1

C	1.20944400	1.83325000	-0.84359400
C	2.50550300	3.33894000	0.05250000
C	3.55814100	1.07645200	-0.03712800
H	3.26113200	0.59301300	0.89569500
N	1.38608900	3.96119100	-0.17565700
N	0.58905400	3.00940400	-0.73387100
N	2.45337800	2.02759600	-0.35479200
C	3.74569800	3.94535100	0.63322300

H	3.47577400	4.62046500	1.44594900
H	4.25207600	4.52684400	-0.15107400
C	4.81215200	1.91664600	0.21349500
O	4.58605500	2.94906100	1.15012300
C	-0.73958700	3.32507700	-1.19377300
C	-0.93398000	3.47727400	-2.56774600
C	-1.78019600	3.37104400	-0.26251500
C	-2.24633700	3.62275200	-3.01271500
C	-3.07019500	3.52561300	-0.76037200
C	-3.32490800	3.61902600	-2.12920400
H	-2.42891200	3.72256000	-4.07896900
H	-3.90187900	3.54170700	-0.06091500
H	5.57787600	1.26884100	0.63772600
C	0.59707800	0.64283500	-1.57593000
O	1.25190200	0.18293100	-2.52064700
C	-0.71222900	0.32242800	-1.17722800
C	-1.42444200	-0.67106900	-1.82621300
H	-1.13550600	0.83686800	-0.33081700
H	-0.95904500	-1.07913200	-2.71916800
C	-2.90015700	-0.72399300	-1.80335600
C	-3.56802200	-1.40629300	-2.82527700
C	-3.65866100	-0.04371200	-0.84152600
C	-4.95676100	-1.39101200	-2.90454800
H	-2.98853700	-1.94406000	-3.57095100
C	-5.04393100	-0.01720200	-0.92820200
H	-3.16573800	0.46981600	-0.02267700
C	-5.69837100	-0.68794000	-1.96049200
H	-5.45743800	-1.92368200	-3.70665800
H	-5.62036100	0.51194300	-0.17601000
H	-6.78199800	-0.67194000	-2.01466700
C	-4.74285300	3.67046200	-2.63146600
H	-5.35842100	4.33523300	-2.01957000
H	-4.79101500	4.00923600	-3.66862900
H	-5.18790200	2.67021000	-2.58118800
C	0.22139600	3.44596900	-3.53349500
H	1.03601600	4.09452700	-3.19798500
H	0.62469900	2.43344800	-3.63796200
H	-0.09783700	3.78337800	-4.52081100

C	-1.54891100	3.17419000	1.21168200
H	-0.62204700	3.64445700	1.54891200
H	-2.37382200	3.59863200	1.78682300
H	-1.49793800	2.10499000	1.45366600
H	5.18750000	2.32711700	-0.73647200
C	3.79875300	0.02872800	-1.12440900
H	2.91791600	-0.60963100	-1.20272300
H	3.91621100	0.52840900	-2.08935900
C	5.00382800	-0.83742900	-0.83471100
C	6.00143900	-1.00119600	-1.79553400
C	5.11440800	-1.53916200	0.37026600
C	7.07721200	-1.85703200	-1.56865200
H	5.92914600	-0.46442400	-2.73723900
C	6.18904000	-2.38982700	0.60338300
H	4.34416300	-1.43082600	1.12798400
C	7.17401800	-2.55535600	-0.36903000
H	7.83948900	-1.97718200	-2.33194400
H	6.25375800	-2.92990300	1.54268900
H	8.01071600	-3.22296700	-0.19064100
C	0.30627400	-2.40624600	-0.48774200
C	-1.05805800	-2.55991500	-0.65131800
C	1.26204200	-3.02566800	-1.44853300
C	0.89027100	-3.37364600	-2.75241900
C	2.57869800	-3.26455200	-1.04490500
C	1.81610700	-3.93116500	-3.62658300
H	-0.12134600	-3.18978200	-3.09896600
C	3.50896100	-3.81440900	-1.91914300
H	2.86853200	-2.99923800	-0.03540900
C	3.13032800	-4.14913700	-3.21619500
H	1.51346700	-4.18439700	-4.63781600
H	4.53093800	-3.96729500	-1.58438500
H	3.85303600	-4.57459000	-3.90533400
N	0.90084700	-1.58562700	0.41550200
S	0.18924500	-1.27273300	1.82438800
O	-1.02644000	-0.40156300	1.75033300
O	0.00123800	-2.47610400	2.64420200
C	1.44308100	-0.26914600	2.58819100
C	1.27231700	1.10594200	2.69966600

C	2.59424100	-0.88299900	3.07217100
C	2.27758500	1.87364500	3.27568200
H	0.35951500	1.56405800	2.34097800
C	3.59258400	-0.10258200	3.64416000
H	2.70658400	-1.95986500	3.00180400
C	3.45428700	1.28507800	3.74979400
H	2.14384900	2.94828000	3.36319400
H	4.49361800	-0.58061200	4.01653700
C	4.54800400	2.13401300	4.33715800
H	5.27786900	1.52908600	4.87952900
H	5.06652500	2.66635000	3.53379300
H	4.14065100	2.88128700	5.02372700
H	-1.74238000	-2.24169000	0.12526400
H	-1.43431000	-3.32108900	-1.32523100
C	-5.20117700	-1.17629200	2.24229300
C	-5.73043200	-2.18018100	1.42258500
C	-7.05234900	-2.08524300	0.95443800
C	-7.77801000	-0.97756400	1.33567600
C	-7.22069600	0.02978600	2.16700000
C	-5.92676200	-0.04403000	2.63804200
H	-7.47023600	-2.85259700	0.31285000
H	-8.80193400	-0.86435600	0.99587000
H	-7.83571500	0.88138400	2.43870700
H	-5.49173400	0.71752000	3.27362200
N	-4.76365300	-3.13212700	1.20878900
N	-3.69812700	-2.77813900	1.86085100
N	-3.93732500	-1.61041700	2.46428600
O	-3.03656600	-1.04298700	3.29338600
H	-2.25946100	-0.79357600	2.71840600

TS6(S)$^{HOBt}{}_{L5}$

0 1

C	1.42028100	2.60238300	0.07515500
C	3.06252000	3.51908800	-1.02360000
C	3.78692800	2.14943700	0.96009100
H	3.55898300	2.67207900	1.88979200
N	1.98583000	3.98009200	-1.59152700
N	0.96861000	3.39798200	-0.89682300

N	2.76500700	2.66164600	0.00240900
C	4.48064500	3.89971400	-1.30896400
H	4.51165900	4.91436400	-1.70570900
H	4.91634000	3.21406200	-2.04990300
C	5.17431600	2.56297000	0.44724600
O	5.19688900	3.86858600	-0.09696100
C	-0.40135000	3.74632800	-1.16851200
C	-1.00466800	3.24342200	-2.32522400
C	-1.08461600	4.51456900	-0.22146100
C	-2.37427500	3.45546800	-2.46692600
C	-2.45172900	4.69607100	-0.41492200
C	-3.11811600	4.14427500	-1.50818800
H	-2.87446700	3.05593200	-3.34484300
H	-3.01184500	5.27085300	0.31748700
H	5.86343000	2.54917100	1.29281700
C	0.61146100	1.95566400	1.18743400
O	1.14600700	1.97469900	2.30501900
C	-0.68738200	1.51487800	0.85212500
C	-1.54715100	1.03123600	1.82407000
H	-1.00196500	1.56309600	-0.17977700
H	-1.24384000	1.17179500	2.85780000
C	-3.00309900	0.95489700	1.57774100
C	-3.89007300	1.14313500	2.64071600
C	-3.52327900	0.73842500	0.29376900
C	-5.26578700	1.14552100	2.42661800
H	-3.49622600	1.29745300	3.64158400
C	-4.89423100	0.74059100	0.08159300
H	-2.85130600	0.53758300	-0.53500900
C	-5.77144100	0.95020200	1.14572500
H	-5.94160300	1.29751500	3.26219400
H	-5.28431200	0.55080500	-0.91318600
H	-6.84318500	0.93963700	0.97545400
C	-4.61310100	4.26370300	-1.63496100
H	-5.09461200	3.42951200	-1.11170200
H	-4.98113400	5.19077100	-1.18864600
H	-4.93094600	4.22933300	-2.67986900
C	-0.21973300	2.50155400	-3.37170700
H	0.52718000	3.15662200	-3.82987800

H	0.30031000	1.64624700	-2.93427900
H	-0.88562500	2.13886400	-4.15700600
C	-0.39908500	5.10115300	0.98453200
H	0.61197300	5.44561800	0.75093300
H	-0.96728100	5.95272800	1.36287400
H	-0.32468000	4.36745500	1.79349200
H	5.53023600	1.84383100	-0.30093800
C	3.70506700	0.64375900	1.24134900
H	4.17973000	0.50021700	2.21680100
H	2.65909700	0.36244900	1.37700500
C	4.33049300	-0.30558200	0.24631200
C	4.02090800	-0.26426200	-1.11611300
C	5.20000400	-1.30150600	0.69897800
C	4.56498500	-1.19224700	-1.99767100
H	3.31054100	0.46675800	-1.48591300
C	5.73330200	-2.24240900	-0.17845900
H	5.45057900	-1.34860300	1.75581900
C	5.41744700	-2.18961100	-1.53265300
H	4.30200900	-1.14963700	-3.04939900
H	6.39779100	-3.01422400	0.19749500
H	5.82638300	-2.92265000	-2.22084600
C	0.18235400	-1.21666900	1.82831700
C	-1.19618000	-1.18313900	1.92679900
C	1.02417700	-1.41660200	3.04527700
C	0.54419000	-1.12538900	4.32730800
C	2.32498500	-1.91660300	2.91723600
C	1.33949700	-1.33670000	5.44793800
H	-0.44863800	-0.70687000	4.45414800
C	3.11886100	-2.13291800	4.03810000
H	2.71654400	-2.12186800	1.92665100
C	2.62900400	-1.84534600	5.30934800
H	0.95141500	-1.09771000	6.43300700
H	4.12197800	-2.53047600	3.91574300
H	3.24694500	-2.01229600	6.18587200
N	0.90699800	-0.94507100	0.71978900
S	0.43426500	-1.02213100	-0.79758800
O	-1.01564200	-1.29684800	-1.03655800
O	0.92569000	0.15137700	-1.53145800

C	1.31238800	-2.42741900	-1.45900400
C	1.74954700	-3.45707100	-0.63460000
C	1.52831100	-2.47450200	-2.83282700
C	2.43228200	-4.53023800	-1.19377900
H	1.57259800	-3.41304900	0.43383300
C	2.20665300	-3.55678500	-3.37784200
H	1.19065500	-1.66064800	-3.46517600
C	2.67974000	-4.59251900	-2.56735500
H	2.78519600	-5.32916200	-0.54835900
H	2.38273400	-3.59159900	-4.44904900
C	3.47017900	-5.73342700	-3.15172500
H	3.26933700	-5.85615900	-4.21859100
H	4.54412100	-5.55175500	-3.03383300
H	3.24150700	-6.67552400	-2.64679900
H	-1.79424300	-1.25872100	1.03408500
H	-1.67512800	-1.49391700	2.84733500
C	-4.46028500	-2.73913700	-0.76120700
C	-5.40966800	-2.65097000	0.26243800
C	-6.70529000	-2.18239400	-0.01392500
C	-6.97939300	-1.81969100	-1.31515800
C	-5.99745700	-1.90793800	-2.33611000
C	-4.72055800	-2.36496000	-2.08594500
H	-7.44879500	-2.10820400	0.77137000
H	-7.96710000	-1.45194200	-1.57220200
H	-6.26330900	-1.60762200	-3.34437300
H	-3.96303400	-2.43640600	-2.85678300
N	-4.83794300	-3.09033700	1.43401500
N	-3.61473500	-3.43881800	1.18177300
N	-3.37105200	-3.22857200	-0.11560400
O	-2.19858400	-3.61385600	-0.66422400
H	-1.67359300	-2.77616200	-0.77915200

NHCb

0 1

C	-0.28536600	-0.26082900	0.05316200
C	-0.06325200	1.98546800	0.08389200
C	2.05048800	0.92743500	0.09849700
H	2.37123800	0.85790800	1.14501100

N	-1.34656100	1.81933400	0.06848200
N	-1.45665400	0.43632900	0.05267500
N	0.58683800	0.77984300	0.07805000
C	0.90990500	3.11940000	0.08962700
H	1.02142900	3.51171200	1.10559100
H	0.61156300	3.93962400	-0.56353800
C	2.19201600	2.39076000	-0.38128500
C	-2.75485400	-0.14348500	0.01473000
C	-2.88377200	-1.53289000	-0.01924300
C	-3.88517700	0.67270400	0.01218900
C	-4.15198600	-2.09998400	-0.05718700
C	-5.14851300	0.08873100	-0.02579400
C	-5.29055200	-1.29536200	-0.06098300
H	-4.24895900	-3.18056800	-0.08460100
H	-6.02662500	0.72641000	-0.02793300
H	3.10892900	2.84772400	-0.00587400
H	2.22843100	2.39528400	-1.47561500
C	2.78326000	-0.10649000	-0.76118800
H	2.05904000	-0.52634900	-1.46971800
H	3.54494100	0.41420500	-1.35609300
H	-3.76600900	1.74833500	0.03923900
H	-1.98930400	-2.14330900	-0.01588900
H	-6.27788000	-1.74381100	-0.09182600
C	3.48023000	-1.24877100	0.00446800
C	4.65313700	-0.69191800	0.82034100
C	2.50031000	-1.97235000	0.93300100
C	4.02260000	-2.24398100	-1.02708900
H	5.38350300	-0.19989600	0.16859600
H	4.32275700	0.03610200	1.56904600
H	5.16462100	-1.50038400	1.35285500
H	1.64354400	-2.35919100	0.37523400
H	3.00366500	-2.80499400	1.43596600
H	2.10655800	-1.30339600	1.70626100
H	4.56708300	-3.05475400	-0.53215600
H	3.20638300	-2.68757800	-1.60670800
H	4.70887800	-1.75223500	-1.72512100

2b

0 1

C	1.25986500	0.61012700	-0.73421800
O	0.58338600	-0.31430300	-1.18225000
C	2.65331700	0.38476400	-0.29151900
C	3.24411400	-0.79561900	-0.53878000
H	3.15773000	1.17564900	0.25054800
H	2.65837100	-1.53247800	-1.08697100
C	4.59644600	-1.20593700	-0.15608100
C	5.05319400	-2.46490100	-0.56911500
C	5.45095100	-0.39517400	0.60748100
C	6.32967700	-2.90482800	-0.23474100
H	4.39658700	-3.09870500	-1.15843700
C	6.72421400	-0.83457900	0.94188600
H	5.11824700	0.58081900	0.94578000
C	7.16784600	-2.08994700	0.52150900
H	6.66849200	-3.88179000	-0.56277500
H	7.37475300	-0.19961700	1.53404600
H	8.16386400	-2.42968600	0.78581400
C	0.69436400	1.98861100	-0.60675000
C	1.54554800	3.10127000	-0.61192100
C	-0.69535900	2.20733300	-0.50116100
C	1.04762100	4.39068600	-0.48059200
H	2.61266400	2.95679100	-0.74027500
C	-1.19225600	3.50241500	-0.35291700
C	-0.32380700	4.58715300	-0.33437500
H	1.72526000	5.23686600	-0.49423500
H	-2.26360400	3.63894200	-0.26571600
H	-0.72287100	5.58995600	-0.22543900
N	-1.62857700	1.12768100	-0.54745400
H	-1.17323900	0.28811400	-0.92115800
S	-2.33772300	0.73849600	0.93268800
O	-1.32818800	0.25837700	1.87436900
O	-3.17270900	1.86841400	1.32497300
C	-3.35395500	-0.62116500	0.44207300
C	-4.57493300	-0.36011500	-0.17779900
C	-2.91246700	-1.91773000	0.67790900
C	-5.36224000	-1.43218100	-0.57103900
H	-4.89576100	0.66356100	-0.34008600

C	-3.71802400	-2.97889000	0.27487600
H	-1.96061100	-2.08586300	1.17023600
C	-4.94624300	-2.75251200	-0.35078200
H	-6.31681700	-1.24778300	-1.05498000
H	-3.38755400	-3.99769800	0.45174900
C	-5.82607400	-3.90016900	-0.76913600
H	-6.70364200	-3.96563100	-0.11872700
H	-6.18668500	-3.76232200	-1.79159100
H	-5.29228600	-4.85039800	-0.71420300

M1b

0 1

C	-0.99469700	-1.75419300	0.13908400
C	-1.08100000	-3.55014700	-1.08379600
C	1.20684400	-3.01586000	-0.67579600
H	1.53219400	-2.30349800	-1.43521700
N	-2.32965100	-3.22732100	-0.90756800
N	-2.25876600	-2.11104700	-0.12809500
N	-0.23623300	-2.69132800	-0.44017700
C	-0.33378200	-4.58569400	-1.84896900
H	-0.34514000	-4.32276100	-2.91075000
H	-0.76671800	-5.57908500	-1.73474100
C	1.07824900	-4.45399700	-1.23425700
C	-3.47024200	-1.50919900	0.34658500
C	-3.59131800	-1.17174500	1.69016000
C	-4.50054500	-1.29860400	-0.56579800
C	-4.77021100	-0.56791600	2.12053900
C	-5.67854100	-0.71426300	-0.11529400
C	-5.81050500	-0.34167100	1.22269000
H	-4.87752200	-0.29241400	3.16356400
H	-6.48991400	-0.54199400	-0.81355800
H	1.85640000	-4.65442800	-1.97046100
C	-0.58502600	-0.60124000	1.05354000
O	0.00437000	-0.91079300	2.06473600
C	-1.11468300	0.79124200	0.80303900
C	-1.60252700	1.14090000	-0.60603400
H	-0.30220500	1.46376400	1.10291100
H	-1.92388200	0.94554300	1.52838200

H	-0.75811900	1.08416500	-1.29552600
H	-2.35312400	0.41919500	-0.94440200
C	-2.21721900	2.51881800	-0.61240500
C	-1.43835100	3.64533100	-0.88491400
C	-3.56666600	2.68989500	-0.29287400
C	-1.99979700	4.91968400	-0.84753200
H	-0.38511400	3.51447100	-1.12599600
C	-4.13103300	3.96199400	-0.25545500
H	-4.17598200	1.81423400	-0.07319600
C	-3.34782900	5.08062000	-0.53424400
H	-1.38499600	5.78726900	-1.06604000
H	-5.18214500	4.08120900	-0.01123400
H	-3.78631600	6.07310900	-0.50746700
O	1.05189200	-0.22074300	-0.68132300
C	1.78056800	0.80072500	-0.45444100
C	2.03698500	1.78046700	-1.47430400
C	2.37645500	1.05024300	0.83092500
C	2.82531900	2.88296000	-1.24101400
H	1.59497700	1.61349000	-2.45226900
C	3.16209400	2.15633200	1.06138800
H	2.18160400	0.34132200	1.63026900
C	3.39337700	3.07356900	0.02732800
H	3.01794300	3.61293700	-2.01879600
H	3.60905200	2.33396300	2.03281600
N	4.21545500	4.21883100	0.26778000
O	4.70681400	4.37083700	1.38504000
O	4.40511000	5.01496100	-0.65037400
H	1.18912200	-5.16820800	-0.41326700
C	2.04238600	-2.85164800	0.58709900
H	1.66413100	-3.53368300	1.36069600
H	1.87960700	-1.82916400	0.93261500
H	-2.78784100	-1.38583200	2.38844600
H	-6.72953600	0.12019400	1.56684300
H	-4.36856200	-1.58593900	-1.60312100
C	3.56588800	-3.04785800	0.42149300
C	3.95858800	-4.52282900	0.26924300
C	4.08682700	-2.24295600	-0.77458800
C	4.21591800	-2.50473400	1.70149700

H	3.55135100	-5.12457500	1.08931800
H	3.61423000	-4.95262700	-0.67487800
H	5.04909800	-4.62072600	0.29042400
H	3.76299000	-1.19805100	-0.71051500
H	5.18117400	-2.26423400	-0.79502600
H	3.73355600	-2.65392400	-1.72707000
H	5.30025400	-2.65178700	1.67380500
H	4.01954600	-1.43279600	1.80986500
H	3.82560800	-3.01653500	2.58797400

M01b

1 1

C	0.49288400	0.71959600	0.11130100
C	1.79235900	2.41024000	0.53636000
C	3.02486700	0.36729600	0.70445200
H	2.83846000	-0.42568000	1.42995200
N	0.62672500	2.94513800	0.27868200
N	-0.17206500	1.87547700	0.01979100
N	1.73533800	1.05487300	0.46521300
C	3.17564700	2.86464400	0.85596300
H	3.18980300	3.63961200	1.62194000
H	3.64048700	3.26718800	-0.04823000
C	3.83929400	1.53998400	1.32257300
C	-1.57800100	2.05546600	-0.19113700
C	-1.99760600	3.04636100	-1.07158100
C	-2.46842500	1.23817800	0.49796700
C	-3.36325600	3.20750400	-1.27484200
C	-3.83099000	1.40484900	0.27092000
C	-4.27537800	2.38642800	-0.61187700
H	-3.71498200	3.97228400	-1.95801300
H	-4.53494300	0.76818000	0.79460200
H	3.79619600	1.47393500	2.41117400
C	0.06956500	-0.71152900	-0.13753100
O	0.56423300	-1.53582200	0.59504900
C	-0.76728300	-1.02289600	-1.34826100
C	-1.50889800	-2.36010800	-1.22465900
H	-1.45015800	-0.20025800	-1.57616900
H	-1.81526100	-2.67006400	-2.22647600

H	-0.81347600	-3.11345800	-0.84430200
C	-2.72125200	-2.25292300	-0.32905700
C	-3.98130200	-2.00264400	-0.87842700
C	-2.60260400	-2.36065200	1.06068900
C	-5.10136600	-1.86860500	-0.06187500
H	-4.08615000	-1.92132000	-1.95746600
C	-3.71859000	-2.20973500	1.88144500
H	-1.63152100	-2.57367600	1.50004500
C	-4.97208400	-1.96726000	1.32209800
H	-6.07476600	-1.68646400	-0.50646800
H	-3.61153300	-2.29905600	2.95793800
H	-5.84344200	-1.86452800	1.96091600
H	4.88360800	1.48803700	1.01820500
C	3.56880000	-0.20198000	-0.61187300
H	2.72578500	-0.53804600	-1.23048900
H	4.06332700	0.60728300	-1.16473700
H	-1.26729300	3.66588700	-1.57971900
H	-5.33862300	2.51813100	-0.78035300
H	-2.11163800	0.49899400	1.20992500
C	4.53008600	-1.39757500	-0.44737000
C	5.67797900	-1.07299800	0.51463300
C	3.76187000	-2.62317700	0.06243500
C	5.10909200	-1.71480100	-1.83069100
H	6.25063800	-0.20296900	0.17506400
H	5.31911200	-0.87655800	1.53132600
H	6.36504200	-1.92268500	0.57041100
H	2.94199800	-2.88140200	-0.61633700
H	4.43351800	-3.48482400	0.12588900
H	3.33685100	-2.46668700	1.05899100
H	5.77105800	-2.58428700	-1.77575800
H	4.31267500	-1.94024000	-2.54766400
H	5.68849900	-0.87001200	-2.21711400
H	-0.03956200	-1.06112500	-2.17322000

M2b

0 1

C	0.53872600	0.71687000	0.09029600
C	1.86488400	2.42410900	0.44646600

C	3.05820500	0.35977400	0.64327200
H	2.85964600	-0.38034000	1.41863300
N	0.70559700	2.96965600	0.21384200
N	-0.11812400	1.88808200	0.00983400
N	1.79133000	1.06803100	0.40267900
C	3.26603000	2.86613100	0.70876500
H	3.32166500	3.64284600	1.47155100
H	3.69969200	3.26309400	-0.21323100
C	3.94470300	1.53756200	1.14898000
C	-1.52357900	2.09892700	-0.17173800
C	-1.94365100	3.09097100	-1.04865500
C	-2.42821100	1.30968700	0.53375400
C	-3.31077300	3.28527500	-1.23230400
C	-3.78808700	1.49532700	0.31971800
C	-4.23008600	2.48349000	-0.56049800
H	-3.65405200	4.05694100	-1.91276300
H	-4.49592800	0.85810000	0.84080900
H	4.00623100	1.50240000	2.23843600
C	0.09631500	-0.71697600	-0.04120300
O	0.66956000	-1.50041700	0.78823100
C	-0.81291200	-0.95467700	-1.03078200
C	-1.49585200	-2.28873900	-1.14318800
H	-1.70291700	-2.55601500	-2.18521500
H	-0.83748800	-3.05525900	-0.72024100
C	-2.79415700	-2.24840200	-0.35816500
C	-4.03992800	-2.21495700	-0.98337900
C	-2.74181800	-2.15680500	1.03961200
C	-5.21244700	-2.09674000	-0.23464500
H	-4.09473200	-2.27835000	-2.06755200
C	-3.90717700	-2.04104700	1.78885500
H	-1.76479600	-2.16228200	1.52019000
C	-5.15101000	-2.01155800	1.15279900
H	-6.17404100	-2.07237800	-0.73899200
H	-3.85003500	-1.97577400	2.87159700
H	-6.06224000	-1.92486000	1.73658700
H	4.95662900	1.46210100	0.75225800
C	3.52045700	-0.33284200	-0.64101800
H	2.63077500	-0.71870700	-1.15391300

H	3.97888300	0.41624600	-1.30242700
H	-1.20947900	3.69158300	-1.57413400
H	-5.29302700	2.62910000	-0.72177600
H	-2.07265000	0.54524400	1.21552000
C	4.48772700	-1.51537400	-0.43693400
C	5.76475000	-1.08550900	0.29298200
C	3.79874100	-2.63457500	0.35518800
C	4.85944600	-2.04339200	-1.82750900
H	6.27887400	-0.27840800	-0.24147300
H	5.55207200	-0.74427400	1.31216000
H	6.45571000	-1.93153400	0.36852000
H	2.81091700	-2.85992200	-0.05685000
H	4.41453100	-3.53987500	0.33386700
H	3.65490700	-2.36150700	1.40515600
H	5.55155300	-2.88748200	-1.74461600
H	3.96922300	-2.38532100	-2.36576900
H	5.34342500	-1.26504800	-2.42738700
H	-1.20321900	-0.12654400	-1.61328500

M3b

0 1

C	0.68837400	0.78899000	0.21720900
C	2.32547300	2.22397600	-0.23174700
C	3.24208700	0.15984600	0.50778200
H	3.14852600	-0.35400800	1.46582600
N	1.27631100	2.88415900	-0.57880600
N	0.21862700	2.00804100	-0.27236000
N	2.05078400	0.99359600	0.29923500
C	3.80304500	2.42796700	-0.32196300
H	4.11577500	3.41168700	0.02894500
H	4.12008000	2.32227000	-1.36389700
C	4.32858300	1.26924500	0.55974900
C	-1.05868300	2.59028100	-0.08632500
C	-1.51135800	3.52433600	-1.01990400
C	-1.83953400	2.27715200	1.02940400
C	-2.75094100	4.13003700	-0.84125300
C	-3.08491300	2.87600600	1.18673100
C	-3.54743200	3.80297100	0.25439600

H	-3.09933900	4.85480600	-1.56990200
H	-3.68911500	2.62353300	2.05210800
H	4.43829100	1.61742900	1.58992900
C	0.03143100	-0.38847700	0.48156000
O	0.81391400	-1.40852300	1.02242400
C	-1.33440500	-0.66680300	0.16202800
C	-1.96068900	-1.83999000	0.41066100
C	-3.35942900	-2.12681200	0.09059300
C	-3.80744400	-3.45797600	0.08328800
C	-4.29796600	-1.12073400	-0.20253900
C	-5.12513700	-3.77637800	-0.22730300
H	-3.10128000	-4.25079800	0.31682600
C	-5.61257700	-1.43968800	-0.51919200
H	-3.99946400	-0.07633400	-0.15949900
C	-6.03620900	-2.76902000	-0.53705600
H	-5.44087300	-4.81538100	-0.22925300
H	-6.31706200	-0.64359600	-0.74124300
H	-7.06532700	-3.01346900	-0.77942800
H	5.29634000	0.90505100	0.21744900
C	3.41254300	-0.85250300	-0.63300200
H	2.41578700	-1.19084000	-0.93928800
H	3.85526100	-0.33199100	-1.49479800
H	-0.88409800	3.76917700	-1.86967900
H	-4.51836300	4.26910000	0.38324900
H	-1.47299400	1.56270000	1.75852400
C	4.25442000	-2.10445800	-0.30800500
C	5.63988900	-1.73936200	0.23567600
C	3.53062400	-2.99341200	0.71118200
C	4.42587000	-2.89467600	-1.61090300
H	6.19280000	-1.10920600	-0.47019900
H	5.57400200	-1.21028900	1.19291000
H	6.22701200	-2.64875400	0.40208300
H	2.53183700	-3.26099800	0.35561300
H	4.10406100	-3.91182300	0.87766700
H	3.41351900	-2.49644800	1.67939900
H	4.98986200	-3.81586600	-1.43096300
H	3.45271200	-3.16914200	-2.03169600
H	4.96670700	-2.30582000	-2.35942900

H	-1.88182500	0.12507300	-0.34072100
H	-1.39043200	-2.65426300	0.85275900
H	0.55251600	-1.53727600	1.94442600

2b$^-$

-1 1

C	1.32043800	0.63478100	-0.73603900
O	0.75901000	-0.25702600	-1.35578100
C	2.71057700	0.41923800	-0.21570800
C	3.39226500	-0.67995300	-0.56363200
H	3.12169400	1.15551500	0.46715500
H	2.89293700	-1.36668300	-1.24635300
C	4.73842700	-1.06981300	-0.12693300
C	5.28193500	-2.26512300	-0.61591600
C	5.50645000	-0.29957600	0.75997300
C	6.55321100	-2.68305700	-0.23432900
H	4.69521500	-2.86922000	-1.30246300
C	6.77507300	-0.71579300	1.14154200
H	5.10918100	0.63017200	1.15465000
C	7.30388100	-1.90885400	0.64612400
H	6.95620800	-3.61219100	-0.62401400
H	7.35598300	-0.10912300	1.82866100
H	8.29558000	-2.23071700	0.94695700
C	0.69993600	1.95824400	-0.45796100
C	1.53419700	3.08583700	-0.45658900
C	-0.70037600	2.09990500	-0.20916200
C	1.04669300	4.36401600	-0.23791800
H	2.59259800	2.95106700	-0.66334800
C	-1.16915400	3.42125600	0.02905500
C	-0.32089200	4.51310000	0.01210300
H	1.70856400	5.22248000	-0.25964600
H	-2.22743400	3.57362000	0.20958500
H	-0.73354100	5.50206700	0.19295400
N	-1.48685800	0.97970300	-0.19894800
S	-2.96816000	1.05989000	0.36946000
O	-3.03677600	1.35981500	1.82037100
O	-3.91566500	1.86764100	-0.43593100
C	-3.47394900	-0.63978200	0.16342700

C	-4.81031400	-0.90084400	-0.11323700
C	-2.57047300	-1.68395400	0.35441800
C	-5.24746400	-2.22138500	-0.19702100
H	-5.49166300	-0.07236800	-0.27568700
C	-3.01981300	-2.99517300	0.26527400
H	-1.52689000	-1.45392700	0.53784400
C	-4.36295300	-3.28399000	-0.00727200
H	-6.29053200	-2.42850700	-0.41973000
H	-2.31748800	-3.81336000	0.40279700
C	-4.83011800	-4.71436200	-0.09333700
H	-4.72866500	-5.21656700	0.87375200
H	-5.87767100	-4.77188000	-0.39605600
H	-4.23352900	-5.27829200	-0.81622800

2b$^{\mathbf{-DBUH+}}$

0 1

C	-1.83411600	1.38776400	0.64291700
O	-1.15057800	0.52530400	1.18750200
C	-3.19651900	1.04709400	0.13759500
C	-3.74520000	-0.13496300	0.45172700
H	-3.69233400	1.75832000	-0.51515700
H	-3.15825800	-0.78558700	1.09875400
C	-5.03486300	-0.66702400	0.00078500
C	-5.32668100	-2.01448300	0.25137000
C	-5.98382900	0.11265200	-0.67679500
C	-6.52643900	-2.57585300	-0.17379600
H	-4.59795000	-2.62287600	0.78128800
C	-7.18340600	-0.44608900	-1.09717500
H	-5.78715900	1.16348100	-0.86505900
C	-7.45776300	-1.79218000	-0.84989300
H	-6.73513700	-3.62194900	0.02523500
H	-7.91068700	0.16898600	-1.61706200
H	-8.39684900	-2.22421600	-1.18028200
C	-1.36960200	2.78407500	0.47002500
C	-2.33080400	3.79585500	0.62149100
C	-0.00476800	3.11366500	0.21571600
C	-2.00025000	5.13835300	0.56702800
H	-3.35946800	3.50945700	0.82283500

C	0.30198700	4.49928800	0.16621900
C	-0.66433300	5.47451200	0.33731800
H	-2.75571100	5.90332300	0.70535000
H	1.32678600	4.80909300	0.00127300
H	-0.36719600	6.51861200	0.29356900
N	0.91906900	2.10366900	0.00566200
S	2.34887800	2.54812100	-0.59102700
O	2.24891300	3.20992500	-1.90754300
O	3.21232500	3.24484100	0.38650200
C	3.11427500	0.96460400	-0.87942300
C	4.14381400	0.54350400	-0.04474800
C	2.69285100	0.17722100	-1.94416300
C	4.70700800	-0.71362700	-0.23814200
H	4.48303100	1.20155600	0.74872200
C	3.27167500	-1.07538500	-2.13413700
H	1.91255200	0.53728000	-2.60803200
C	4.26611000	-1.54804900	-1.27184500
H	5.50514800	-1.05239900	0.41834900
H	2.94458400	-1.69814300	-2.96316900
C	4.85674900	-2.92310900	-1.44408100
H	4.46035800	-3.41785800	-2.33333700
H	5.94557600	-2.87540000	-1.53305900
H	4.63082700	-3.55396200	-0.57683000
C	-0.89167700	-3.78558500	-0.73222400
C	-1.02916800	-2.31042300	-0.35229300
C	0.23781400	-1.48293600	-0.64245700
C	0.19877300	-4.54922900	0.02214100
C	1.22375500	-1.60424700	0.48398500
C	1.59593900	-3.94276000	-0.12288000
H	-0.68253900	-3.85013900	-1.80830700
H	-1.85411000	-1.86965600	-0.92068900
H	0.70011700	-1.81621300	-1.57613100
H	-0.04054400	-4.60725100	1.09124200
H	2.35597400	-4.67118500	0.16633600
C	2.18169300	-0.56418200	2.48654800
H	1.68347600	0.10927600	3.18643500
C	2.67827800	-2.92782700	1.91183900
C	2.23715500	-1.98578400	3.02241800

H	1.24740300	-2.28670600	3.37969900
H	2.93564100	-2.04776300	3.85844200
H	2.60820600	-3.96743700	2.23903500
N	1.80900900	-2.77265400	0.73884700
N	1.41484700	-0.55324900	1.24779700
H	1.80295500	-3.66921200	-1.16460600
H	0.23597500	-5.57686700	-0.35310700
H	-1.85146700	-4.28746100	-0.57213900
H	-1.29007700	-2.20138400	0.70716900
H	-0.00034500	-0.42305600	-0.74830300
H	3.18778500	-0.17684700	2.29780300
H	3.71681900	-2.72730800	1.61853200
H	0.95806700	0.32753800	0.96339400

H_2O

0 1

O	0.00000000	0.00000000	0.11876900
H	0.00000000	0.75799400	-0.47507600
H	0.00000000	-0.75799400	-0.47507600

3b

0 1

C	-0.94565100	0.19513500	-0.11336400
C	-1.48896300	0.99981000	0.81965600
C	0.16285500	0.60550300	-0.95273500
C	-1.01461100	2.34996000	1.01090700
C	0.11066400	2.77702700	0.26916500
O	0.62806900	-0.06269900	-1.85938200
C	-1.66700100	3.25502500	1.86070100
N	0.75526500	1.83894900	-0.57773000
C	-1.23657000	4.56497100	1.96550200
C	0.50649600	4.11928400	0.33850800
C	-0.15808500	4.99215400	1.18687300
H	-2.52762200	2.91173500	2.42645200
H	-1.74611400	5.25867400	2.62480100
H	0.16404800	6.02721500	1.22803000
H	1.32259200	4.48283400	-0.26844900
C	-2.60580000	0.30504300	1.55054800

H	-2.33169600	0.14521700	2.60035700
H	-3.52628100	0.89850700	1.55073300
C	-1.51962800	-1.19686000	-0.12449700
H	-1.80287000	-1.49644800	-1.13916100
C	-2.79360300	-1.03626800	0.77657100
H	-2.85251000	-1.87310500	1.47734400
C	-0.47667000	-2.17740700	0.38874500
C	0.27490900	-2.93318100	-0.51395800
C	-0.20447700	-2.29572100	1.75489400
C	1.25813200	-3.80886100	-0.06048300
H	0.09080500	-2.82231500	-1.57835300
C	0.77509400	-3.17382300	2.21123800
H	-0.76360800	-1.69957300	2.47224800
C	1.50513200	-3.93854700	1.30410800
H	1.82963200	-4.39283600	-0.77619000
H	0.96612700	-3.26195500	3.27630200
H	2.26260800	-4.63044600	1.65948600
C	-4.05403100	-1.03712700	-0.06284800
C	-4.98128200	-2.07392700	0.04811300
C	-4.30241900	-0.01110700	-0.98271300
C	-6.13180200	-2.09016300	-0.73933400
H	-4.80112300	-2.87592400	0.75905400
C	-5.44779500	-0.02564300	-1.77176500
H	-3.58974200	0.80454700	-1.08452400
C	-6.36768700	-1.06673700	-1.65222100
H	-6.84336900	-2.90346800	-0.63715400
H	-5.62293900	0.77629000	-2.48200700
H	-7.26197900	-1.07760800	-2.26679800
S	2.42795000	1.98566200	-1.03342400
O	2.51131600	2.19355000	-2.46637800
O	2.99756800	2.97012300	-0.12796700
C	3.07977500	0.40022500	-0.59433700
C	3.57579700	-0.43187500	-1.59087800
C	3.11240600	0.04769800	0.75102200
C	4.13519000	-1.64628200	-1.21801600
H	3.50942300	-0.12962000	-2.62988200
C	3.66154500	-1.18047700	1.09955800
H	2.71444500	0.71623500	1.50871200

C	4.18456900	-2.03611500	0.12531800
H	4.53075400	-2.31041400	-1.98104700
H	3.68194400	-1.48106100	2.14252700
C	4.79997000	-3.35572000	0.50514200
H	5.87226600	-3.35642800	0.28705100
H	4.66711000	-3.56385200	1.56842900
H	4.34967300	-4.17319800	-0.06580400

S1-b

0 1

C	-0.90866500	-0.01546500	-1.14809400
O	-1.26206900	-0.49842000	-2.19985500
C	0.52810300	0.36704000	-0.87001700
C	1.47870800	-0.25451900	-1.89787700
H	0.81014700	0.05813100	0.14261300
H	0.61549800	1.45950400	-0.90378400
H	1.38979000	-1.34532600	-1.85255200
H	1.17351300	0.05389900	-2.90138600
C	2.89969300	0.16898500	-1.61470500
C	3.73529600	-0.63117100	-0.83266300
C	3.38405600	1.39599000	-2.07729300
C	5.02620100	-0.21559400	-0.51600500
H	3.37095600	-1.58829700	-0.46987400
C	4.67494400	1.81405100	-1.76475700
H	2.74348900	2.02614100	-2.69041200
C	5.50005800	1.00904900	-0.98064400
H	5.65900500	-0.85323200	0.09453300
H	5.03898600	2.76705500	-2.13593800
H	6.50661200	1.33380700	-0.73688000
N	-1.88138800	0.18298200	-0.12977600
C	-3.17929200	-0.42748900	-0.30597900
C	-3.30617500	-1.81564800	-0.46783600
C	-4.30453700	0.39051800	-0.36288200
C	-4.58048700	-2.34707900	-0.68533900
C	-5.56769500	-0.16062400	-0.54212400
H	-4.18700700	1.46189500	-0.25026500
C	-5.70767000	-1.53631800	-0.70252800
H	-4.66316800	-3.41716700	-0.84105700

H	-6.43686900	0.48750700	-0.56721700
H	-6.68883800	-1.97370300	-0.85204700
C	-2.16250500	-2.79676100	-0.53232100
O	-2.27207300	-3.76688600	-1.26472900
C	-0.95418300	-2.59667700	0.30506000
H	-1.00371900	-1.90637400	1.14006300
C	0.16831600	-3.26524600	-0.00482300
H	0.12123200	-3.93581900	-0.86234000
C	1.46544200	-3.17594000	0.67286200
C	2.50387200	-4.01517300	0.24597900
C	1.71318000	-2.26626800	1.71385300
C	3.75976500	-3.95412000	0.84306100
H	2.32013600	-4.71761500	-0.56232700
C	2.96904800	-2.20295000	2.30391300
H	0.92686600	-1.59766100	2.05138100
C	3.99515700	-3.04545700	1.87249200
H	4.55326600	-4.61076200	0.50187900
H	3.15196000	-1.48956900	3.10084800
H	4.97490400	-2.98952100	2.33623100
S	-1.56102300	0.92309500	1.38434700
O	-2.86305100	1.10621200	2.00003300
O	-0.54822000	0.15814200	2.10314100
C	-0.86620400	2.48698200	0.93938200
C	0.33114000	2.87515200	1.53293500
C	-1.54370700	3.30885300	0.04308400
C	0.86101500	4.11719300	1.20552300
H	0.83853800	2.20829500	2.22156900
C	-0.99393600	4.54378900	-0.27281300
H	-2.47337400	2.98612200	-0.41519000
C	0.21325600	4.96309100	0.29912500
H	1.79643500	4.43363200	1.65702900
H	-1.50769000	5.19263200	-0.97526800
C	0.82134600	6.28783600	-0.07407700
H	1.57634300	6.15131900	-0.85487100
H	0.06633100	6.97740700	-0.45580500
H	1.31480700	6.74875400	0.78428000

S2-b

0 1

C	1.72989000	-0.68328200	0.57921500
C	2.13134500	0.16594800	-0.65052600
C	0.22675600	-0.79635900	0.77105600
C	-0.03136800	1.35765900	-0.24708400
H	2.07115200	-0.14058500	1.47401600
H	2.12814500	-0.47970000	-1.54088700
O	-0.32504900	-1.76537500	1.22279800
C	3.52841900	0.75949000	-0.50373800
C	4.54365900	0.45069700	-1.40633800
C	3.79668700	1.65337400	0.53696100
C	5.81772800	0.99128800	-1.25016400
H	4.34486000	-0.24283800	-2.21842400
C	5.06739600	2.19726100	0.69417600
H	3.00416200	1.92709800	1.23069700
C	6.08463400	1.86233900	-0.19730100
H	6.60404000	0.72354200	-1.94918000
H	5.26247500	2.88560400	1.51054800
O	-0.54514200	0.28719000	0.48256700
H	7.07798300	2.28173000	-0.07362500
C	1.15833500	1.29773700	-0.84217100
H	1.46403700	2.13516500	-1.45929300
C	-0.94165300	2.52418100	-0.29248400
C	-2.33441700	2.41892200	-0.46021400
C	-0.37705500	3.80250500	-0.19545600
C	-3.12070700	3.57139300	-0.52835400
C	-1.16122700	4.94673900	-0.25176200
H	0.69458400	3.88425500	-0.04346900
C	-2.53910100	4.82751200	-0.41369300
H	-4.18781000	3.46202200	-0.68250000
H	-0.69957800	5.92365900	-0.16010000
H	-3.16412800	5.71285000	-0.46024700
N	-2.98254100	1.15493000	-0.62053300
H	-2.31713600	0.38769600	-0.69932400
S	-4.06741300	0.73975500	0.61858400
O	-3.39366700	0.75158300	1.91336400
O	-5.25750500	1.56571800	0.44687100
C	-4.42376200	-0.92896800	0.16047600

C	-5.57289900	-1.19834200	-0.57559600
C	-3.53265500	-1.93106000	0.53709600
C	-5.83190700	-2.51377100	-0.94206000
H	-6.24441800	-0.39116800	-0.84768800
C	-3.80823000	-3.23694800	0.15038300
H	-2.63846700	-1.69407600	1.10752000
C	-4.95764400	-3.54621800	-0.58621400
H	-6.72533500	-2.74424500	-1.51457300
H	-3.12124300	-4.03107400	0.42816300
C	-5.25915300	-4.97139300	-0.96612100
H	-5.79607500	-5.47537900	-0.15617000
H	-5.88433400	-5.01884200	-1.85999600
H	-4.34034600	-5.53198200	-1.15165400
C	2.31559900	-2.10800900	0.59112700
H	1.82033400	-2.68606900	-0.19651200
H	2.03713900	-2.57568500	1.53991400
C	3.81032500	-2.17190700	0.39410000
C	4.34809800	-2.80092200	-0.72818600
C	4.68207000	-1.58908200	1.32019100
C	5.72662500	-2.83192100	-0.93785000
H	3.67977300	-3.26171400	-1.45159500
C	6.05509500	-1.60645400	1.10959800
H	4.27765600	-1.09886800	2.20287100
C	6.58226600	-2.22733500	-0.02354400
H	6.12819700	-3.32237900	-1.81927900
H	6.71773400	-1.13366500	1.82788700
H	7.65501100	-2.23930800	-0.18863900

S3-b

0 1

O	-0.86571400	-1.55143600	-3.65852500
C	-0.55396900	-1.62197000	-2.51012500
C	-1.03836900	-2.31663800	-1.24907900
C	0.22384800	-1.68146600	-0.61938900
H	-1.05571900	-3.40165600	-1.35551300
C	-2.27316400	-1.77008300	-0.48551100
O	0.51887500	-1.02023700	-1.91497400
C	-1.62112900	-1.31257900	0.86713200

H	-1.39328200	-2.24114300	1.40982100
C	-0.27425000	-0.70541400	0.45568700
H	-0.38473200	0.28798200	0.01120000
H	0.42118500	-0.62433500	1.29444800
C	-2.52465700	-0.48146300	1.74406200
C	-3.45019500	-1.13320500	2.56445000
C	-2.51389000	0.91554600	1.72868000
C	-4.34623300	-0.41274800	3.34814600
H	-3.46531400	-2.22053100	2.58724700
C	-3.40683400	1.64046600	2.51499800
H	-1.80829000	1.45026800	1.09936600
C	-4.32629500	0.98013000	3.32543800
H	-5.05592500	-0.93830400	3.97928400
H	-3.38248700	2.72550000	2.49303500
H	-5.02009800	1.54720700	3.93763300
C	1.38319100	-2.53474800	-0.18907400
C	2.62780800	-1.90714400	-0.01475300
C	1.26562400	-3.89045200	0.10164800
C	3.72818200	-2.62722100	0.43898900
C	2.35999900	-4.61143200	0.57778700
H	0.30870200	-4.38812100	-0.01965800
C	3.58616400	-3.97824200	0.74740500
H	4.68912000	-2.13397000	0.54554800
H	2.25108200	-5.66462700	0.81205600
H	4.44354800	-4.53614300	1.10845500
N	2.69247600	-0.51487800	-0.29833600
S	3.70004700	0.51212600	0.53586800
O	3.58982100	0.13428700	1.93976500
O	5.02721300	0.57792300	-0.06975200
C	2.87330600	2.04850100	0.24344800
C	3.34665300	2.89882600	-0.74875700
C	1.74068500	2.35694700	0.99606200
C	2.65789400	4.08237600	-0.99746900
H	4.23665300	2.63567500	-1.31005800
C	1.06988600	3.54293300	0.73315100
H	1.40131400	1.68230400	1.77611100
C	1.51634500	4.41884000	-0.26559700
H	3.01454300	4.75513400	-1.77146000

H	0.18749300	3.79873000	1.31265400
C	0.79267600	5.71409600	-0.51951800
H	1.13391500	6.48287200	0.18116100
H	0.97734000	6.07948600	-1.53147900
H	-0.28437100	5.59667100	-0.38059400
H	2.46125600	-0.21931800	-1.24399400
H	-2.95500100	-2.59849900	-0.27815100
C	-3.09169700	-0.67616600	-1.14258400
C	-4.48451600	-0.74790700	-1.03819300
C	-2.53543500	0.45290100	-1.75571100
C	-5.29999500	0.26703700	-1.52890100
H	-4.93489300	-1.61110100	-0.55518900
C	-3.34852400	1.46919200	-2.25191800
H	-1.46027600	0.56227600	-1.85683600
C	-4.73306000	1.38220300	-2.13985200
H	-6.37810700	0.18481700	-1.43391900
H	-2.89302200	2.33223900	-2.72683600
H	-5.36456300	2.17570700	-2.52589900

NHCc

0 1

C	0.10060000	0.00673700	0.81381400
C	-0.53590400	-1.74171900	-0.46752100
C	-2.30563800	-0.72600500	0.98171200
H	-2.26747400	-0.99753400	2.03931200
N	0.72279500	-1.59167200	-0.74643100
N	1.07812700	-0.51824500	0.04755500
N	-0.94295300	-0.80530000	0.44674900
C	-1.48400200	-2.75316200	-1.03378200
H	-1.00119800	-3.73072200	-1.08101800
H	-1.77192700	-2.46904400	-2.05420100
C	-3.22847700	-1.67093800	0.16890100
O	-2.61548500	-2.89007200	-0.19652800
C	-3.78430600	0.57255200	-0.37267000
C	-2.93250500	0.62450800	0.73318900
C	-2.74172900	1.80469200	1.44173300
C	-3.43052300	2.94545400	1.03292000
C	-4.29126800	2.89613200	-0.06578700

C	-4.47375800	1.71090000	-0.77712100
C	-3.78898300	-0.80696400	-0.98214800
H	-2.06549200	1.83311300	2.28992900
H	-4.82587900	3.79118700	-0.36802300
H	-5.14338900	1.67845900	-1.63138100
H	-3.13122100	-0.83147800	-1.85905500
H	-4.77646500	-1.14518900	-1.30360100
C	2.42978700	-0.05081800	-0.00282300
C	2.69827000	1.16463000	-0.63732400
C	3.43396000	-0.82973000	0.57932900
C	4.02341000	1.59948000	-0.67414300
C	4.74409600	-0.35853900	0.51271200
C	5.05707500	0.85029500	-0.11170600
H	4.25252800	2.54477100	-1.16047200
H	5.53872100	-0.94702900	0.96582000
H	-4.05650300	-1.95449800	0.82323300
H	-3.30083900	3.87766700	1.57297500
C	1.58897300	1.97215600	-1.25743300
H	1.02698500	1.36913800	-1.97767700
H	0.87679900	2.30439400	-0.49710900
H	1.99080900	2.84592300	-1.77332800
C	6.48663000	1.32040800	-0.19778600
H	7.04699100	1.04791400	0.69988000
H	6.99374000	0.86145000	-1.05286700
H	6.54044300	2.40407300	-0.32326700
C	3.09751300	-2.12627300	1.26631500
H	2.28715300	-1.98295600	1.98778600
H	2.75659700	-2.87448300	0.54518400
H	3.96837000	-2.52083300	1.79269900

1c

0 1

C	3.28904600	-0.29159600	-0.07451600
O	4.18243000	0.46620000	-0.37260300
H	3.50250100	-1.20669600	0.51762100
C	1.84366100	-0.09517600	-0.44373400
C	0.93379700	-0.18009200	0.79498300
H	1.56417700	-0.89571600	-1.14158300

H	1.73213600	0.86386800	-0.95644800
H	1.11551900	-1.12945600	1.31141800
H	1.19672800	0.62295500	1.49140000
C	-0.52318100	-0.07411300	0.41561300
C	-1.27050500	-1.21852800	0.12680100
C	-1.13676800	1.17569700	0.30099400
C	-2.60322100	-1.11741100	-0.26466500
H	-0.80361400	-2.19663200	0.21536700
C	-2.46888600	1.28140400	-0.09035200
H	-0.56423000	2.07247200	0.52532100
C	-3.20620300	0.13364700	-0.37383800
H	-3.17169700	-2.01638300	-0.48157500
H	-2.93205800	2.25974800	-0.17189300
H	-4.24534600	0.21325500	-0.67652800

2c

0 1

C	-1.18692400	0.12853400	-0.11014000
O	-0.92852100	1.04094100	-0.86646900
C	-0.08720000	-0.70696800	0.53601000
C	1.29263800	-0.29919700	0.04583900
H	-0.25866100	-1.76094200	0.29715400
H	-0.16785000	-0.59352000	1.62390000
O	1.89492900	-0.99080900	-0.74682400
C	1.85116000	0.98939100	0.59061700
H	2.81948500	1.19763600	0.13685900
H	1.15129100	1.80201700	0.37868300
H	1.95720200	0.91508900	1.67733500
C	-2.60216000	-0.25688900	0.23864300
H	-2.79735900	-1.27944900	-0.09775600
H	-2.73999000	-0.24035400	1.32344300
H	-3.30046800	0.42923900	-0.23909800

2c^{LiCl}

0 1

C	0.80188200	-1.26840000	-0.09288900
O	0.05718500	-1.38822500	-1.05172700
C	0.81249700	0.00158200	0.73846400

C	0.79666300	1.27086900	-0.09409300
H	-0.14594800	-0.00003700	1.28574100
O	0.05311800	1.38637900	-1.05428000
C	1.70670700	2.37973500	0.34050900
H	1.57232600	3.25202900	-0.29708400
H	1.48896000	2.63499000	1.38237400
H	2.74519500	2.03688000	0.30419600
C	1.71836900	-2.37231400	0.34066000
H	2.75489400	-2.02362100	0.30390200
H	1.50268200	-2.62920200	1.38255200
H	1.58854900	-3.24509400	-0.29721000
Li	-1.32323900	-0.00489600	-1.35945400
Cl	-2.65107200	-0.00406200	0.47107500
H	1.63214800	0.00372400	1.45776500

HCO_3^-

-1 1

C	0.14000300	0.06409300	0.00006300
O	-0.10294000	1.28922900	0.00016300
O	-1.00053500	-0.76622200	-0.00004600
H	-1.73889900	-0.14535400	0.00009400
O	1.21583500	-0.55290700	-0.00017700

H_2CO_3

0 1

C	-0.05216600	0.12678000	-0.00000800
O	-0.54012800	-1.12224900	0.00015400
O	1.27868500	0.12671500	-0.00002900
H	1.61082100	-0.78252100	0.00008700
O	-0.71245500	1.13117300	-0.00014800
H	-1.50663800	-1.06327700	0.00013800

M1c

0 1

C	-0.58400000	-0.13464300	-0.69512300
C	-2.23849600	1.22110500	-1.35009200
C	-3.00093100	-1.07130600	-0.85778800
H	-2.59275800	-2.05177300	-1.09798700

N	-1.23333500	2.01566200	-1.27988600
N	-0.15632800	1.19294300	-0.89998900
N	-1.94080400	-0.08925700	-1.06295600
C	-3.64019200	1.52944800	-1.75818400
H	-3.66806000	2.42787800	-2.37459600
H	-4.27386300	1.68715400	-0.87269200
C	-4.23878300	-0.75514700	-1.78527400
O	-4.10442700	0.44138000	-2.53444700
C	-4.93968400	-0.81986200	0.54450300
C	-3.56444200	-1.04057400	0.54656800
C	-2.86147600	-1.19579900	1.73918700
C	-3.55796700	-1.09668800	2.94126600
C	-4.93574500	-0.86120000	2.94443200
C	-5.63627100	-0.72879600	1.74775900
C	-5.48088300	-0.73147300	-0.85863300
H	-1.79601100	-1.40223800	1.72004800
H	-5.46529500	-0.78615000	3.88907200
H	-6.70843200	-0.55485100	1.75333700
H	-6.06425200	0.17822300	-1.03694000
H	-6.13897100	-1.57752100	-1.07950800
C	0.87206300	1.84235700	-0.14837200
C	0.77720800	1.95149900	1.24227400
C	1.95909800	2.37652700	-0.85453400
C	1.81943900	2.58487700	1.92525100
C	2.97061600	3.00688400	-0.13649500
C	2.92056600	3.11404100	1.25688400
H	1.75898000	2.67296600	3.00780100
H	3.82137900	3.42202200	-0.67277600
H	-4.32891500	-1.53508800	-2.54191600
H	-3.02979300	-1.20939900	3.88281200
C	0.14301900	-1.20018900	-0.29479100
O	-0.53097100	-2.40332400	-0.04845700
H	-0.30761100	-3.03059800	-0.74889300
C	1.61229700	-1.20881000	0.00117400
C	2.34592700	-2.36187000	-0.71402700
H	1.79737500	-1.30564600	1.08241800
H	2.07068900	-0.26658800	-0.30889400
H	1.94252500	-3.32015100	-0.36836600

H	2.15386800	-2.28943800	-1.79129200
C	3.82937300	-2.31173900	-0.45016200
C	4.38519200	-2.97540000	0.64662600
C	4.66910000	-1.54303800	-1.26151900
C	5.74671300	-2.87881100	0.92426800
H	3.74256000	-3.57590200	1.28610000
C	6.03102400	-1.44512900	-0.98986200
H	4.24658700	-1.01860300	-2.11587800
C	6.57437000	-2.11392700	0.10531900
H	6.16261600	-3.40337700	1.77905000
H	6.66870700	-0.84707300	-1.63352400
H	7.63608300	-2.04041400	0.31810800
C	2.02080800	2.24004500	-2.35220200
H	1.14689400	2.69977100	-2.82226500
H	2.92316300	2.70935100	-2.74864100
H	2.01800000	1.18428800	-2.64379400
C	4.03561900	3.79672900	2.00709000
H	3.84489400	3.80139800	3.08216100
H	4.99041100	3.29175400	1.83284600
H	4.15171400	4.83335500	1.67692000
C	-0.39969000	1.38791000	1.99278200
H	-1.33034900	1.50578400	1.42924900
H	-0.26584500	0.31561700	2.17163800
H	-0.51306000	1.88323900	2.95949300

M2c

0 1

C	3.26415500	-0.32992600	0.43448900
C	3.89893000	-1.92816800	1.75157900
C	2.80632400	-2.72527400	-0.31822600
H	2.01310100	-2.29656200	-0.93437900
N	4.33947200	-0.83743400	2.31719700
N	3.93905400	0.15733900	1.47388700
N	3.25424900	-1.65518800	0.57961600
C	3.89399800	-3.35711900	2.19268500
H	4.05065100	-3.42257300	3.26900000
H	4.68451200	-3.91900500	1.67702500
C	2.25445200	-3.92553600	0.52491700

O	2.60368300	-3.85788400	1.90475600
C	3.98292700	-4.69642800	-0.98426600
C	3.97383700	-3.30663300	-1.08576000
C	4.94018500	-2.62638500	-1.82013600
C	5.93578100	-3.36789400	-2.45242800
C	5.95180000	-4.76125200	-2.35349800
C	4.97482500	-5.43504900	-1.62320400
C	2.82382300	-5.18828800	-0.15503300
H	4.90823800	-1.54408400	-1.90562400
H	6.73520300	-5.32411000	-2.85074400
H	4.99089700	-6.51809600	-1.54773200
H	3.10728100	-5.92783900	0.59953800
H	2.06048300	-5.64718800	-0.79248700
C	4.48023000	1.49239400	1.57827700
C	5.48312400	1.84833400	0.66629900
C	4.02422400	2.34517600	2.58763200
C	6.00649400	3.13482200	0.76962300
C	4.58241100	3.62011100	2.64011600
C	5.56597900	4.03263900	1.74087800
H	6.77774600	3.44216200	0.06885700
H	4.23797400	4.30715600	3.40893600
H	1.16443500	-3.89427700	0.51879200
H	6.70297200	-2.85979600	-3.02720300
C	2.71696200	0.45240600	-0.73684300
O	3.08421000	0.10228100	-1.83577800
H	-9.59118200	0.81653300	0.69245800
C	1.75573500	1.56499900	-0.44821900
C	1.68943900	2.58281300	-1.58894100
H	2.00960400	2.03959600	0.50751800
H	0.79590300	1.04520000	-0.30529000
H	1.56056000	2.04378300	-2.53198900
H	0.79765200	3.19763900	-1.43968000
C	2.91345700	3.46794100	-1.65044600
C	3.99206900	3.16246300	-2.48600900
C	2.98041000	4.62218200	-0.86411000
C	5.10597100	3.99926600	-2.54276900
H	3.94807600	2.27006900	-3.10531700
C	4.08543300	5.46558500	-0.92708800

H	2.14625500	4.87036800	-0.21179500
C	5.15110700	5.15794400	-1.77086900
H	5.93246400	3.75175500	-3.20211000
H	4.11242300	6.36677400	-0.32157200
H	6.01187500	5.81774400	-1.82607500
C	-1.66905300	-1.13526500	-1.25845400
C	-3.01909400	-0.80505100	-1.22385800
C	-3.68695400	-0.44105500	-0.05026800
C	-2.94416000	-0.46739600	1.13388200
C	-1.59249800	-0.79068900	1.17806000
C	-0.87819500	-1.07372700	-0.04683700
H	-3.60207600	-0.85709900	-2.13824300
H	-3.45464000	-0.19914100	2.05311700
C	-5.11909400	-0.06560200	-0.05691800
C	-5.95293700	-0.36108700	1.02383500
C	-5.68839500	0.60649900	-1.14354400
C	-7.30233300	-0.00353100	1.06023200
H	-5.52654500	-0.90898900	1.85500100
C	-7.03149500	0.97915200	-1.18553500
H	-5.04060300	0.86322000	-1.97317300
C	-7.82664800	0.66705700	-0.06348100
O	0.40303600	-1.27951600	-0.04918300
O	-9.14381900	1.05319800	-0.12394800
C	-0.86686600	-0.87003200	2.52785300
C	-0.26592400	-2.27287200	2.71066100
C	0.26865400	0.15780600	2.60503400
C	-1.80081500	-0.60666300	3.71664500
H	-1.05030900	-3.03692200	2.67279400
H	0.46011300	-2.46396700	1.92153100
H	0.24024400	-2.34954500	3.68102100
H	-0.11538000	1.17438000	2.45503900
H	0.74680200	0.11302900	3.59048700
H	1.01342700	-0.05370800	1.83481200
H	-1.23479200	-0.71167100	4.64819000
H	-2.21903400	0.40530800	3.69411000
H	-2.63118400	-1.31990800	3.74665400
C	-1.02487900	-1.59525800	-2.57298800
C	0.07934200	-0.61229200	-2.98869900

C	-0.44157000	-3.00860400	-2.38989900
C	-2.02378400	-1.66932700	-3.73542300
H	-0.34957800	0.38215600	-3.16032900
H	0.82781300	-0.53983400	-2.20378800
H	0.56466900	-0.93947300	-3.91648800
H	-1.25029300	-3.73327200	-2.24627100
H	0.13247100	-3.30931700	-3.27468700
H	0.20485200	-3.04040700	-1.51240600
H	-1.50347100	-2.02451900	-4.63151700
H	-2.84413300	-2.36376500	-3.52715800
H	-2.45379000	-0.68898600	-3.96658500
C	-7.61418500	1.71860500	-2.40106200
C	-8.13960100	3.10526700	-1.98493300
C	-6.55371600	1.94397900	-3.48778600
C	-8.74608300	0.89091600	-3.03794600
H	-8.93985400	3.03013900	-1.24867700
H	-7.32842700	3.70530200	-1.55937600
H	-8.52445200	3.63267600	-2.86499800
H	-6.14503900	0.99922900	-3.86016200
H	-7.01615500	2.46438500	-4.33192100
H	-5.72544400	2.56182400	-3.12736600
H	-9.13847000	1.41718900	-3.91535000
H	-8.36458000	-0.08053700	-3.36905100
H	-9.56532700	0.72312800	-2.33915400
C	-8.17312800	-0.33892200	2.28591900
C	-8.69329500	0.95519900	2.94996600
C	-9.33064300	-1.28550000	1.90093600
C	-7.37006500	-1.07405700	3.36986600
H	-7.84630900	1.55536300	3.29483000
H	-9.28831200	1.60030300	2.29761600
H	-9.31247800	0.70555000	3.81737800
H	-8.92325600	-2.22685000	1.52117400
H	-9.93784700	-1.50696200	2.78432500
H	-10.00276200	-0.90072000	1.12933400
H	-8.02518500	-1.27937600	4.22173700
H	-6.98232500	-2.03010200	3.00680600
H	-6.53053700	-0.47173900	3.72900300
C	6.01084500	0.89554800	-0.37630000

H	6.04234000	-0.13594500	-0.01106000
H	5.39900300	0.92062200	-1.28422800
H	7.02546100	1.18162900	-0.65764100
C	6.16744500	5.40862100	1.83942900
H	5.42414600	6.14024100	2.16526700
H	6.98373500	5.41864900	2.56902300
H	6.57327200	5.72776900	0.87680500
C	2.95419800	1.93514900	3.56005600
H	1.96102800	2.10481800	3.13267100
H	3.02576200	0.87813100	3.82263000
H	3.02906300	2.52686300	4.47393000

M02c

1 1

C	-0.58253600	-0.42549400	0.09340300
C	-1.62662300	-1.26770000	1.80113900
C	-2.68886900	0.84296900	0.89212700
H	-2.16299400	1.73013500	1.24965800
N	-0.58356400	-2.03050700	1.62901300
N	0.05559700	-1.48951900	0.56448500
N	-1.67520000	-0.26507600	0.86072200
C	-2.67843600	-1.42099800	2.85692700
H	-2.19957000	-1.67561000	3.80301200
H	-3.35444200	-2.23943900	2.58087600
C	-3.82286200	0.38454100	1.84539200
O	-3.36281500	-0.20555600	3.03696000
C	-4.62865700	0.28306800	-0.36340000
C	-3.44626700	1.02986800	-0.39784200
C	-3.16177400	1.88392800	-1.45681100
C	-4.05540400	1.93732400	-2.52377400
C	-5.21704100	1.16267300	-2.51235300
C	-5.51999900	0.34011800	-1.42789900
C	-4.76329600	-0.44857200	0.94818400
H	-2.26793000	2.50014200	-1.45522000
H	-5.90517500	1.21986200	-3.34931200
H	-6.44312600	-0.22998100	-1.40728400
H	-4.42252100	-1.48621500	0.85009400
H	-5.78348900	-0.46879400	1.33485700

C	1.28553500	-2.04280500	0.05655100
C	1.26936700	-2.69474700	-1.17668500
C	2.44120100	-1.86220400	0.83059100
C	2.50046700	-3.14215700	-1.66535000
C	3.63555200	-2.32448400	0.29125000
C	3.68506100	-2.95734400	-0.95773900
H	2.52346900	-3.65420700	-2.62319700
H	4.55288400	-2.19246500	0.85915300
H	-4.34625500	1.29225400	2.15278600
H	-3.85296100	2.59320100	-3.36329500
C	-0.13584400	0.34616700	-1.15218600
O	-0.85866600	0.29070500	-2.11325300
C	1.17495300	1.07472200	-1.07578500
C	1.24593100	2.05047400	0.11216500
H	1.31052300	1.59517800	-2.02602600
H	1.97093500	0.32376900	-0.98574000
H	0.48977800	2.83175600	-0.01384700
H	1.00198500	1.52263600	1.04423600
C	2.61841400	2.67382000	0.23035900
C	2.79513600	4.04836200	0.07280000
C	3.73577600	1.87381400	0.49001000
C	4.06355400	4.61689700	0.17720100
H	1.93445400	4.67911200	-0.13165300
C	5.00266600	2.43811600	0.59488900
H	3.61691800	0.79790300	0.60655600
C	5.16953100	3.81367000	0.43897800
H	4.18522600	5.68793900	0.05255200
H	5.86016100	1.80507000	0.79873700
H	6.15733300	4.25489000	0.52021600
C	5.00167900	-3.44151900	-1.50329000
H	5.44154000	-4.18986600	-0.83815900
H	4.88195000	-3.88791400	-2.49146200
H	5.71359900	-2.61527700	-1.58119300
C	0.00165800	-2.93084900	-1.95715200
H	-0.86356900	-3.05404200	-1.30135800
H	-0.20913900	-2.10426900	-2.64191800
H	0.10337100	-3.83695200	-2.55606000
C	2.39005200	-1.21450500	2.18967600

H	1.87654600	-1.86118500	2.90648200
H	3.39928300	-1.02477700	2.55761300
H	1.85168600	-0.26125100	2.17144400

M3c

0 1

C	-0.16823200	-0.29629200	0.16989000
C	-1.65791200	-1.26065500	1.45741100
C	-2.43572800	0.86610300	0.47295300
H	-1.91437200	1.78172600	0.19858500
N	-0.65597500	-2.08690100	1.44709300
N	0.26054900	-1.48041200	0.62975400
N	-1.40426100	-0.15740000	0.69171300
C	-2.92738700	-1.27220000	2.24252100
H	-2.84782100	-1.96060800	3.08333500
H	-3.77678800	-1.56920300	1.61240800
C	-3.32491700	1.02814800	1.76296400
O	-3.07961500	0.03860600	2.75375600
C	-4.71795800	0.47779300	-0.14881800
C	-3.40288400	0.42135500	-0.60152900
C	-3.08869400	-0.00800000	-1.88747200
C	-4.13180000	-0.40286300	-2.72139000
C	-5.45539900	-0.35701400	-2.27336300
C	-5.75890200	0.08833100	-0.98881200
C	-4.78873200	1.00132200	1.26297700
H	-2.05256600	0.00058700	-2.21455600
H	-6.25667200	-0.66958400	-2.93561700
H	-6.78891600	0.12550200	-0.64674700
H	-5.39893100	0.38103300	1.92696700
H	-5.21438400	2.00959500	1.28364600
C	1.46349400	-2.19278400	0.29784400
C	1.56717400	-2.76537900	-0.97053100
C	2.45588700	-2.30153700	1.27482800
C	2.73505200	-3.47286900	-1.25437200
C	3.60201600	-3.01899900	0.94171700
C	3.75649300	-3.61201600	-0.31413500
H	2.84667700	-3.92695300	-2.23565600
H	4.39488900	-3.11464200	1.67929700

H	-3.08479900	1.96785300	2.26026400
H	-3.91776700	-0.74396900	-3.72917500
C	0.47284900	0.71207200	-0.74071400
O	-0.35922800	1.42455200	-1.40505700
C	1.83644800	0.77070300	-0.70400700
C	2.56853000	1.79403500	-1.52775900
H	2.40331200	0.13312500	-0.03564800
H	3.52059900	1.38982800	-1.88918700
H	1.95054000	2.02608800	-2.40288500
C	2.82858800	3.08059700	-0.76708700
C	4.11926500	3.51516400	-0.46774500
C	1.74115900	3.84894600	-0.33114400
C	4.32747300	4.69394700	0.24984200
H	4.97119900	2.92560700	-0.79793100
C	1.94581500	5.02608300	0.37953500
H	0.73847200	3.49790800	-0.56707700
C	3.24194100	5.45328700	0.67476700
H	5.33949200	5.01682300	0.47574100
H	1.09389900	5.61599400	0.70524100
H	3.40184700	6.37258500	1.22957600
C	4.99562600	-4.40741700	-0.63321600
H	5.88334700	-3.94565800	-0.19444200
H	4.91676000	-5.42045700	-0.22584500
H	5.14528900	-4.49213300	-1.71146100
C	2.29036400	-1.64325300	2.61860900
H	2.05946600	-0.57868000	2.50479600
H	1.46832500	-2.09691100	3.17972300
H	3.20501100	-1.73567700	3.20562200
C	0.47985500	-2.59657400	-1.99698800
H	-0.51161500	-2.74910700	-1.55954100
H	0.50664600	-1.58444200	-2.41656200
H	0.61052000	-3.30871600	-2.81300800

M4c

0 1

C	-0.68624000	0.18456900	-0.73253600
C	-2.38055300	1.63042200	-0.67710500
C	-3.10140100	-0.69181300	-1.05446800

H	-2.70029800	-1.51413200	-1.64319300
N	-1.36178000	2.35916400	-0.38283500
N	-0.28844400	1.47428200	-0.41524200
N	-2.05533800	0.32061800	-0.93474800
C	-3.81263700	2.01995700	-0.83397300
H	-3.89695500	3.08711100	-1.03818400
H	-4.37917400	1.78604800	0.07908000
C	-4.38257000	-0.09096700	-1.74813800
O	-4.31746500	1.31204100	-1.95029100
C	-4.96826300	-1.03471600	0.41685200
C	-3.58663200	-1.16019900	0.29997900
C	-2.81082100	-1.64194100	1.35146800
C	-3.44744100	-1.99234100	2.53983700
C	-4.83412600	-1.86707100	2.66402900
C	-5.60362400	-1.39144300	1.60437500
C	-5.57930200	-0.50276700	-0.85442900
H	-1.73525500	-1.74498900	1.23794700
H	-5.31601000	-2.14283900	3.59661400
H	-6.68056000	-1.29460400	1.70559400
H	-6.23583600	0.35763900	-0.68875100
H	-6.17949200	-1.27072700	-1.35226500
C	0.93481900	1.86516800	0.20318000
C	1.21957800	1.42884600	1.50280600
C	1.83208800	2.64515300	-0.53437200
C	2.46032800	1.76488000	2.04403500
C	3.05555500	2.96744900	0.05111900
C	3.38973600	2.52763900	1.33428700
H	2.70683200	1.42115800	3.04603500
H	3.77005800	3.56254600	-0.51253700
H	-4.47874000	-0.50125200	-2.75346600
H	-2.86415900	-2.36738600	3.37451600
C	0.07557900	-0.95739100	-0.82123100
O	-0.61059400	-2.16576600	-0.94959700
C	1.50168200	-0.99666200	-0.72053900
C	2.25068900	-2.12284100	-0.65887400
H	2.01102200	-0.03905500	-0.73777200
H	1.75817700	-3.09279100	-0.67058500
C	3.71128700	-2.13894200	-0.56501400

C	4.40557100	-3.34407600	-0.76282400
C	4.47045800	-0.99245400	-0.26251200
C	5.79317700	-3.40257400	-0.68469900
H	3.83948000	-4.24472600	-0.98671400
C	5.85637000	-1.05090500	-0.18941000
H	3.97087000	-0.04813400	-0.05997500
C	6.53005400	-2.25446100	-0.40230600
H	6.30074100	-4.34889400	-0.84585400
H	6.41611900	-0.15066000	0.04764700
H	7.61245100	-2.29674800	-0.33982000
C	0.24284000	0.58014300	2.27090900
H	-0.79040700	0.88233200	2.07565400
H	0.33859900	-0.47081600	1.97483600
H	0.43198200	0.65225700	3.34354700
C	4.71855900	2.88332800	1.95008400
H	4.62637800	3.76814900	2.58815400
H	5.09417100	2.06689400	2.57157700
H	5.46267900	3.10433000	1.18168000
C	1.48331700	3.07610200	-1.93340600
H	1.31319300	2.20342100	-2.57252200
H	0.56114800	3.66364500	-1.94278300
H	2.28663700	3.67370500	-2.36724100
H	-0.30872700	-2.59068400	-1.76507600

M5c

0 1

C	2.71465000	0.89523800	-0.19689700
C	4.31925200	1.72945900	1.00970000
C	4.80655300	-0.54688100	0.16853700
H	4.15803100	-1.40633900	-0.01335700
N	3.42097900	2.67471700	0.94842900
N	2.43113900	2.14330100	0.18335900
N	3.92725100	0.62708100	0.30756100
C	5.58488500	1.62576700	1.79505600
H	5.59882100	2.37100300	2.58962400
H	6.45829900	1.77156300	1.14516600
C	5.66458500	-0.74116800	1.47965400
O	5.56549200	0.34345300	2.39000700

C	7.11962500	-0.53389300	-0.44999900
C	5.82898100	-0.31183900	-0.92337000
C	5.59745700	0.09081200	-2.23621700
C	6.69309000	0.27259900	-3.07534400
C	7.99080200	0.05045600	-2.60689400
C	8.21289700	-0.35651000	-1.29400900
C	7.11343400	-0.96573200	0.99214600
H	4.58570500	0.24749800	-2.59610200
H	8.83387400	0.19964500	-3.27372600
H	9.22194300	-0.52493300	-0.93043100
H	7.80882800	-0.39779600	1.61769600
H	7.38468400	-2.02180100	1.08550200
C	1.33546700	2.96516800	-0.27322400
C	1.36560400	3.40806200	-1.59760400
C	0.28616300	3.23091500	0.60926800
C	0.26397900	4.13543100	-2.04568800
C	-0.78041000	3.97794600	0.11476800
C	-0.81826700	4.41768100	-1.21124000
H	0.25135900	4.48433600	-3.07484800
H	-1.61052800	4.21159500	0.77581300
H	5.28420100	-1.59695200	2.03850800
H	6.53785500	0.58826700	-4.10162800
C	1.92586000	-0.01066700	-1.11488600
O	2.59101200	-0.80131900	-1.77475900
H	2.47936600	-3.49649400	0.67242400
C	0.50275000	0.20312800	-1.17089000
C	-0.23471800	-0.38294300	-2.14475200
H	0.04422000	0.83843600	-0.42364000
H	0.28477100	-1.04900800	-2.83432600
C	-1.64417900	-0.15819600	-2.39320400
C	-2.36905300	0.84334700	-1.72214300
C	-2.29796600	-0.93772600	-3.36220500
C	-3.70197000	1.06798300	-2.02976200
H	-1.88573900	1.46576300	-0.97375000
C	-3.63788200	-0.72303200	-3.65516800
H	-1.74026900	-1.70834900	-3.88866100
C	-4.33647600	0.28861200	-2.99440700
H	-4.25588400	1.83633800	-1.50198200

H	-4.13587000	-1.33180200	-4.40307600
H	-5.38009600	0.47107400	-3.22710100
C	-5.35583900	-1.17402500	0.00506700
C	-4.05945000	-1.64061700	0.13475900
C	-3.03429900	-0.90819900	0.75734600
C	-3.38360300	0.34392200	1.29428200
C	-4.65510400	0.88072000	1.20394700
C	-5.70101300	0.15263300	0.49943500
H	-3.82359700	-2.64019300	-0.21750300
H	-2.59861600	0.92366200	1.77393500
C	-1.67072000	-1.44179800	0.89605100
C	-0.83479100	-1.03706200	1.94851200
C	-1.16095500	-2.41675400	0.02772500
C	0.47389500	-1.48924400	2.09980300
H	-1.25200300	-0.38325900	2.70480100
C	0.13636400	-2.92603100	0.12641300
H	-1.80379200	-2.76584000	-0.77058200
C	0.97198400	-2.37967000	1.12019800
O	-6.86500400	0.64306200	0.34275100
O	2.30575800	-2.72319300	1.21804600
C	-4.98810500	2.24417800	1.81985800
C	-5.36076900	3.24173900	0.70950800
C	-6.16160000	2.10322000	2.80648600
C	-3.80869200	2.83621500	2.59924000
H	-4.48143000	3.46254100	0.09010200
H	-6.14721500	2.82019100	0.08119200
H	-5.71055000	4.18847200	1.13969700
H	-5.88901300	1.42184300	3.62026900
H	-6.40614600	3.07764900	3.24679400
H	-7.03570100	1.70585700	2.29183600
H	-4.09778600	3.80406400	3.02314900
H	-3.49438700	2.18403600	3.42189100
H	-2.94359900	2.99987200	1.94823400
C	-6.46766500	-2.06359700	-0.56900800
C	-7.54126300	-2.28044400	0.51407200
C	-7.12261800	-1.41927400	-1.80366200
C	-5.95154100	-3.44156900	-0.99986300
H	-7.10314000	-2.77330800	1.38900900

H	-7.95851000	-1.32053100	0.82102600
H	-8.34671600	-2.91865200	0.13036000
H	-6.42985200	-1.43469600	-2.65114200
H	-8.01564300	-1.98713200	-2.09332700
H	-7.40544600	-0.38977200	-1.58232500
H	-6.78021900	-4.02449400	-1.41663900
H	-5.17963700	-3.35535700	-1.77350400
H	-5.53394300	-4.00556600	-0.15896000
C	0.58417100	-4.10390700	-0.76429400
C	0.87889000	-5.32479900	0.13487400
C	-0.53100000	-4.53528500	-1.72810400
C	1.79972500	-3.77321300	-1.65267100
H	1.66008800	-5.15189600	0.88201600
H	-0.02431900	-5.60897500	0.68209600
H	1.19337000	-6.17515800	-0.47853400
H	-0.79831300	-3.72733900	-2.41702400
H	-0.18101200	-5.38311200	-2.32430900
H	-1.43260800	-4.84744100	-1.19383000
H	2.07577700	-4.66052800	-2.23155300
H	1.55339400	-2.96777800	-2.34750600
H	2.69229700	-3.44124900	-1.11605800
C	1.28070300	-1.15298000	3.36779600
C	1.66301900	-2.45891100	4.09227800
C	2.55053500	-0.36269400	3.02577200
C	0.46882600	-0.31891600	4.36943400
H	0.76190900	-3.02216200	4.35439600
H	2.30260300	-3.09066400	3.47628600
H	2.19699600	-2.22111600	5.01870200
H	2.29187600	0.61648100	2.60191500
H	3.14741200	-0.18394100	3.92636900
H	3.16630100	-0.91009800	2.31308100
H	1.08818700	-0.12880500	5.25130500
H	0.16803200	0.65204200	3.96437200
H	-0.43061100	-0.84758500	4.69859000
C	-2.02862200	5.13833100	-1.74044500
H	-2.73444300	4.41500100	-2.16348200
H	-2.54623600	5.68104900	-0.94676200
H	-1.75904900	5.84143100	-2.53125200

C	0.27521800	2.65278200	1.99716500
H	-0.58630700	3.01593700	2.56118400
H	0.20422600	1.55770200	1.94347200
H	1.18559400	2.90093100	2.55093300
C	2.50944000	3.08000000	-2.52136000
H	2.39306000	2.07515900	-2.94494000
H	2.53463100	3.78310500	-3.35491900
H	3.47549400	3.12325800	-2.00979600

M05c

1 1

C	0.11284500	0.61741900	-1.02293100
O	0.55933100	1.19954400	-1.99503300
C	-1.14933800	0.88953300	-0.35001900
C	-2.04664100	1.69750700	-0.95151100
H	-1.35158400	0.39796800	0.59516800
H	-1.74913600	2.15858100	-1.89312000
C	-3.38910700	2.00121100	-0.47397000
C	-4.09769800	3.03922400	-1.09623300
C	-4.00056700	1.28202600	0.56732500
C	-5.37774500	3.37471900	-0.67186800
H	-3.63183400	3.58650000	-1.91066800
C	-5.28111600	1.61556700	0.98456100
H	-3.48443500	0.44656300	1.03325000
C	-5.96940000	2.66451300	0.36971200
H	-5.91373500	4.18415400	-1.15527700
H	-5.75056600	1.05636300	1.78712200
H	-6.97117800	2.91978600	0.69919900
C	0.94200300	-0.55014600	-0.53327300
C	2.58279100	-1.95080000	-0.29551400
C	3.28139500	0.37459400	-1.03027800
H	3.09956800	0.60807900	-2.07897500
N	1.50890400	-2.62004100	0.03537800
N	0.49967100	-1.73228500	-0.11404600
N	2.27533700	-0.66436600	-0.64487900
C	3.98787200	-2.46731300	-0.35603200
H	3.98522900	-3.43681700	-0.85703600
H	4.38144600	-2.60616000	0.65755500

C	4.68246800	-0.23724200	-0.76047500
O	4.78038100	-1.59570700	-1.12638300
C	4.24781200	1.40310900	0.89032800
C	3.26784800	1.56446500	-0.09542900
C	2.49298500	2.72105600	-0.15077200
C	2.68617000	3.69813200	0.82464300
C	3.64485900	3.52646800	1.82397000
C	4.44044900	2.38262700	1.85723100
C	5.00451800	0.11424700	0.70673200
H	1.76704800	2.87208300	-0.94099000
H	3.78512200	4.29998300	2.57186600
H	5.20533300	2.26267200	2.61796200
H	4.63636300	-0.64701800	1.40392600
H	6.07899600	0.21368400	0.87149700
C	-0.87204800	-2.09877900	0.13659800
C	-1.26544000	-2.24254000	1.47069800
C	-1.74230700	-2.21052500	-0.95017300
C	-2.61947600	-2.47290300	1.70435300
C	-3.08569900	-2.43798900	-0.65615800
C	-3.54254600	-2.55468800	0.65769800
H	-2.96262000	-2.58087500	2.72962400
H	-3.79280900	-2.52289800	-1.47722900
H	5.38030900	0.29064000	-1.41355900
H	2.09324000	4.60599200	0.79735400
C	-0.27463500	-2.13576400	2.59936100
H	0.43484600	-2.96745600	2.57395300
H	0.30589100	-1.20912900	2.54038800
H	-0.79131100	-2.15239500	3.55927800
C	-5.01089800	-2.73162900	0.93758800
H	-5.52578600	-1.76819300	0.85546000
H	-5.47009900	-3.41166600	0.21665000
H	-5.18081500	-3.12089500	1.94294100
C	-1.27843500	-2.06302400	-2.37569300
H	-1.33663300	-1.01889000	-2.70340900
H	-0.25023900	-2.40710900	-2.51257000
H	-1.92120600	-2.64584700	-3.03701000

2c⁻

-1 1

C	-1.26444400	-0.07299700	-0.01766000
O	-1.49580400	-1.29545500	-0.07541800
C	0.00000100	0.57046200	-0.00009800
C	1.26444200	-0.07300800	0.01746000
H	0.00000700	1.65526900	-0.00009300
O	1.49579200	-1.29545400	0.07548500
C	2.49123800	0.84927300	-0.03996100
H	3.05934800	0.62693100	-0.94933800
H	2.24383300	1.91304200	-0.03221500
H	3.14793900	0.62699200	0.80671600
C	-2.49122900	0.84927900	0.04008300
H	-3.05914800	0.62689500	0.94957000
H	-2.24382500	1.91304800	0.03234400
H	-3.14810800	0.62704100	-0.80646500

2c^{-H2CO3}

-1 1

C	2.06985700	-1.03944200	0.01092000
O	0.98447900	-1.62920500	-0.06126900
C	2.24711500	0.37918300	0.04813000
C	1.22035300	1.31962000	-0.00796400
H	3.26136300	0.75731200	0.10381500
O	-0.03431200	1.11965900	-0.07145100
C	1.60897700	2.78928100	0.00193700
H	1.11532700	3.28561200	0.84253600
H	2.68656400	2.94542800	0.07331700
H	1.23655200	3.26219900	-0.91184000
C	3.34076000	-1.88984100	0.03561700
H	3.49023500	-2.33430500	-0.95367100
H	4.23463400	-1.32165400	0.30008700
H	3.20804500	-2.71177200	0.74354300
C	-2.82418900	-0.15955100	0.01365300
O	-1.64443300	-0.70862500	-0.03457000
O	-3.81416000	-1.09153900	0.02625500
H	-3.39039400	-1.95977200	-0.00301700
O	-3.11430500	1.01868800	0.04589400
H	-0.83772300	0.00962800	-0.04740200

2c^{-LiCls}

-1 1

C	2.34763100	0.36860600	0.72553200
O	1.38918300	1.16312700	0.91489500
C	2.39832400	-0.64048300	-0.26044000
C	1.38096200	-0.87372400	-1.20997600
O	0.28910000	-0.24898700	-1.28844500
C	1.59905200	-1.97661100	-2.23103000
H	1.48723900	-1.55868000	-3.23518900
H	2.57771300	-2.45100700	-2.14331700
H	0.82108200	-2.73682800	-2.10838400
C	3.54459800	0.52429000	1.64698300
H	3.20700200	0.45311200	2.68482900
H	4.32131800	-0.22057700	1.46757700
H	3.96881400	1.52368200	1.51313500
Li	-0.20144900	1.11579700	-0.08243500
Cl	-1.20933200	3.01696500	-0.85926500
H	3.28866800	-1.25519700	-0.31037300
C	-2.70456000	-0.08907500	0.23315200
O	-1.51715300	0.10408400	1.02318300
C	-0.95676000	-1.17003900	1.38607900
C	-1.66767700	-2.19515200	0.51018800
C	-3.05630700	-1.56637700	0.37219200
H	-3.47615100	0.58967600	0.60377100
H	-2.47636800	0.17168300	-0.80700300
H	0.12317800	-1.12903800	1.22080900
H	-1.15621100	-1.34776800	2.45043100
H	-1.17830300	-2.24018500	-0.46735300
H	-1.68004300	-3.19184400	0.95614800
H	-3.62208700	-1.93928300	-0.48379200
H	-3.64347200	-1.73792400	1.28038600

3c

0 1

C	-0.77478500	-1.79512200	0.20376600
C	-0.12669800	-0.61161700	-0.52933300
C	-2.26427200	-1.83889000	0.02277900

C	-0.90637000	0.64686500	-0.18886300
C	-2.21508200	0.56678600	0.11683900
H	-0.58979100	-1.68919600	1.28063500
H	-0.18227000	-0.79660500	-1.61109100
O	-2.93000900	-2.82686400	-0.11839000
C	1.33860000	-0.53779000	-0.13856400
C	2.32932900	-0.96644900	-1.02121500
C	1.71146200	-0.05768400	1.12043800
C	3.67356400	-0.90718700	-0.65970700
H	2.04619800	-1.33726400	-2.00316800
C	3.05358200	0.00553200	1.48248400
H	0.94653300	0.28506300	1.81423600
C	4.03876200	-0.41746900	0.59146200
H	4.43466000	-1.23914300	-1.35861600
H	3.33133200	0.38834500	2.45927600
O	-2.90280300	-0.63327300	0.08140100
H	5.08569900	-0.36477200	0.87182400
H	-0.36071700	-2.75066400	-0.11886800
C	-3.17732000	1.66011300	0.44633400
H	-3.39803700	1.63972400	1.51807400
H	-2.77697400	2.63892700	0.19856900
H	-4.11174700	1.47687600	-0.08881300
C	-0.24836800	1.98213200	-0.29184700
O	-0.54864200	2.91307900	0.43518000
C	0.80289200	2.17306800	-1.36844400
H	1.79817300	2.10570900	-0.91900100
H	0.73589200	1.42964100	-2.16477800
H	0.68091000	3.17608800	-1.78059100

S1-c

0 1

C	-1.10789200	-0.44722400	0.36451800
O	-1.36579000	-0.89409300	1.46207500
C	0.30296300	-0.21253300	-0.11568400
C	1.35221900	-0.44798100	0.97051100
H	0.36957100	0.80705400	-0.52015800
H	0.47264200	-0.87517800	-0.97580100
H	1.15056100	0.22340800	1.81127600

H	1.24567500	-1.46905100	1.34883600
C	2.74819800	-0.22372800	0.44319200
C	3.35709200	1.02946000	0.54365400
C	3.43914000	-1.25547300	-0.19817900
C	4.62853400	1.24812400	0.01865200
H	2.83039800	1.83890200	1.04328100
C	4.70984600	-1.04152800	-0.72522300
H	2.97610100	-2.23602500	-0.28034900
C	5.30850200	0.21228600	-0.61801900
H	5.08928600	2.22670000	0.11064500
H	5.23443100	-1.85471500	-1.21703600
H	6.30014800	0.37934200	-1.02605400
C	-2.23155900	-0.03930100	-0.59022900
H	-1.92703800	-0.22099600	-1.62689400
C	-3.51075900	-0.83487200	-0.29878000
O	-4.52898700	-0.26889000	0.03189200
C	-2.51354400	1.46875400	-0.46240000
O	-2.59347000	2.14992300	-1.45805400
C	-3.42418900	-2.32792600	-0.47099200
H	-2.99039600	-2.57351700	-1.44430500
H	-2.76746200	-2.73862300	0.30096200
H	-4.41762400	-2.76487000	-0.37802300
C	-2.66129200	2.02174800	0.93000300
H	-1.67328500	2.07247600	1.40209700
H	-3.08406200	3.02433600	0.87910800
H	-3.28651800	1.36638400	1.53896700

S2-c

0 1

C	0.99121800	0.88457500	-0.33083600
O	1.34502000	0.90717200	-1.47761500
C	-0.40970300	0.68254700	0.17168900
C	-1.34395300	0.19878900	-0.93748700
H	-0.76172900	1.62945100	0.59832400
H	-0.36141600	-0.03756000	0.99339800
H	-1.33912900	0.92566800	-1.75496500
H	-0.94765600	-0.73928300	-1.33956100
C	-2.74684000	-0.00210800	-0.41922500

C	-3.69896100	1.01473300	-0.52042000
C	-3.10382500	-1.19397000	0.21739000
C	-4.98043700	0.84574300	-0.00087400
H	-3.43243900	1.94547500	-1.01534100
C	-4.38324800	-1.36758000	0.73840800
H	-2.36994200	-1.99211900	0.30142000
C	-5.32575100	-0.34671500	0.63071000
H	-5.71019100	1.64416500	-0.09262100
H	-4.64565800	-2.30093700	1.22677000
H	-6.32396000	-0.48127600	1.03484800
O	1.84888900	1.10475200	0.71510200
C	3.20284300	0.97100300	0.49936700
C	3.75069200	-0.20887600	0.17322800
H	4.82852300	-0.24383700	0.05672000
C	3.00756100	-1.47746700	0.00521100
O	1.79163100	-1.56347700	0.09022700
C	3.94638900	2.24243700	0.72518900
H	3.61428700	2.98784100	-0.00459200
H	3.71726200	2.63353700	1.72076300
H	5.02206100	2.09614000	0.63105400
C	3.85898500	-2.69334000	-0.27476300
H	4.39729500	-2.55366900	-1.21760400
H	4.60945500	-2.81999400	0.51106700
H	3.22909100	-3.57980400	-0.33690600

S3-c

0 1

C	-1.56154200	0.22537100	0.68087800
C	-0.02538700	0.35001100	0.47464000
H	0.47751400	0.57562100	1.42051200
C	-0.41412000	1.63333700	-0.24467900
O	0.12875400	2.49461500	-0.87633700
C	0.75770900	-0.71454500	-0.29875400
H	0.47701400	-1.69899600	0.09074000
H	0.46633100	-0.69929100	-1.35531900
C	2.25780200	-0.54804900	-0.18529200
C	2.87093600	0.66798600	-0.50603100
C	3.05475800	-1.61245700	0.24023100

C	4.25207000	0.80990300	-0.40396400
H	2.26037800	1.50526200	-0.83413500
C	4.43757700	-1.47222800	0.34034400
H	2.58864100	-2.56039200	0.49579400
C	5.04025100	-0.25939800	0.01829200
H	4.71439800	1.75918100	-0.65572000
H	5.04193200	-2.31023100	0.67289900
H	6.11670900	-0.14682100	0.09705500
C	-2.24397900	-0.85238900	-0.14338800
H	-1.79989300	-0.91007000	-1.14465200
H	-2.07684900	-1.83410100	0.31759200
O	-1.73773500	1.53269900	0.01666700
C	-2.01275100	0.28760900	2.12439900
H	-3.09161300	0.42726500	2.18442300
H	-1.74035500	-0.64403100	2.62871800
H	-1.51426900	1.11574200	2.63419800
C	-3.74382400	-0.66389200	-0.33245300
O	-4.35735200	0.24718100	0.17962800
C	-4.41578200	-1.68103500	-1.22391700
H	-5.49328200	-1.52143700	-1.21799300
H	-4.03585200	-1.57905700	-2.24510300
H	-4.18245500	-2.69595700	-0.89050600

S4-c

0 1

C	0.33607700	0.00325300	1.01429300
C	-0.74950300	-0.34378000	-0.06172700
C	-0.01618900	1.45898200	1.33437600
H	0.19150000	-0.64990700	1.87949700
H	0.83389900	2.07808700	1.62229300
H	-0.77018000	1.53906600	2.12338600
C	-0.63097000	1.96762500	0.04506600
O	-0.78834200	3.10747300	-0.30631100
C	1.75436800	-0.16686000	0.51344600
C	2.46323900	-1.32911400	0.82716400
C	2.36663100	0.79126900	-0.30143400
C	3.75087400	-1.53514800	0.33903800
H	1.99929800	-2.07968100	1.46254100

C	3.65292400	0.58684200	-0.79343300
H	1.84589700	1.71019100	-0.55883300
C	4.34882600	-0.57733400	-0.47594400
H	4.28712600	-2.44270000	0.59732000
H	4.11169500	1.34028900	-1.42561500
H	5.35185300	-0.73444300	-0.85894900
C	-0.30322500	-1.34203700	-1.11434700
H	0.57157300	-0.96355500	-1.64590500
H	-0.02953800	-2.28387300	-0.62922700
H	-1.11231200	-1.52124200	-1.82085900
C	-2.03985800	-0.77526900	0.64275100
H	-2.23946500	-0.13174700	1.50892900
H	-1.90423700	-1.78455700	1.05363800
C	-3.30293100	-0.80191500	-0.20867000
O	-3.28844900	-0.78348100	-1.42014500
C	-4.59671800	-0.85337700	0.56997400
H	-5.42510000	-1.07574700	-0.10169900
H	-4.54177300	-1.59909500	1.36783100
H	-4.76495600	0.11918600	1.04409700
O	-0.99902900	0.92362000	-0.72376500

R1

0 1

C	0.49921900	-0.27176600	-0.12184400
O	0.19510900	-1.36300400	-0.53534600
C	1.85925200	0.24882700	0.08284300
C	2.90662600	-0.53443800	-0.21277800
H	1.94725400	1.25852800	0.46627200
H	2.68431500	-1.52978700	-0.59552600
C	4.32568700	-0.19889400	-0.07931000
C	5.27562600	-1.15475100	-0.46260300
C	4.77042500	1.03695100	0.41517200
C	6.63664100	-0.88691100	-0.35819500
H	4.93786500	-2.11355900	-0.84565000
C	6.12840200	1.30365300	0.51962800
H	4.05310400	1.79204500	0.72027200
C	7.06526200	0.34317600	0.13318000
H	7.36033400	-1.63674800	-0.65946100

H	6.46092900	2.26229000	0.90357400
H	8.12591500	0.55610700	0.21658000
O	-0.42083000	0.68524100	0.21869800
C	-1.76834900	0.40977400	0.13721800
C	-2.32304800	-0.75349400	0.67140300
C	-2.56104600	1.40027900	-0.43831800
C	-3.69827000	-0.92758600	0.61694500
H	-1.68840000	-1.50760000	1.11712100
C	-3.93657900	1.23083200	-0.49087200
H	-2.08846600	2.29069700	-0.83556400
C	-4.47864500	0.06532000	0.03640900
H	-4.16794200	-1.81538400	1.02038900
H	-4.58303400	1.97902400	-0.93086900
N	-5.93010000	-0.12235400	-0.01749900
O	-6.60126700	0.76167000	-0.52215100
O	-6.39030900	-1.15202500	0.44530900

TS1

0 1

C	-0.16598300	-1.07522000	0.22226000
C	-0.97480000	-3.04620100	0.84626400
C	-2.67276900	-1.45749100	-0.08484900
H	-3.03525500	-0.70876600	0.62738800
N	0.30209600	-3.13771400	1.05646200
N	0.77977500	-1.91172200	0.66153500
N	-1.30047900	-1.82023900	0.31891900
C	-2.02642100	-4.07666400	1.13062000
H	-1.83170600	-4.54612700	2.09589100
H	-2.01210800	-4.85110800	0.35058200
C	-3.53587100	-2.72105700	0.00577600
O	-3.28657800	-3.44790700	1.19212000
C	2.20414600	-1.72517300	0.64021100
C	2.84186400	-1.66525600	-0.60185300
C	2.89098200	-1.66530600	1.85245100
C	4.22927700	-1.53661000	-0.60247000
C	4.28070800	-1.55620800	1.79882600
C	4.96373800	-1.48795400	0.58410500
H	4.74978300	-1.47277600	-1.55537700

H	4.83941200	-1.50418900	2.73025500
H	-4.58764900	-2.43303200	0.00900800
C	-0.02014700	0.85857600	-0.15307300
O	-0.11386800	1.09406600	-1.36092800
C	-1.07476100	1.29012600	0.83169100
C	-2.12565300	2.00527800	0.42433300
H	-0.90905400	1.01471300	1.87034100
H	-2.16877100	2.26226100	-0.63445600
C	-3.23577400	2.48271400	1.26602900
C	-3.43647700	2.02508200	2.57616900
C	-4.14952800	3.40083200	0.73051600
C	-4.50760800	2.48628300	3.33108900
H	-2.75717900	1.29368800	3.00356700
C	-5.22348100	3.86200600	1.48586600
H	-4.01236600	3.75070100	-0.28949800
C	-5.40455600	3.40748800	2.78970800
H	-4.64959700	2.11950700	4.34252100
H	-5.91976800	4.57455000	1.05552300
H	-6.24377900	3.76120400	3.37968500
C	6.45873500	-1.30791900	0.54305100
H	6.71405900	-0.27460700	0.27948600
H	6.91274000	-1.52813100	1.51156300
H	6.91386000	-1.95828900	-0.20854300
C	2.14827800	-1.68594500	3.16118800
H	1.36940500	-0.91571700	3.16857900
H	1.65599300	-2.64885900	3.32218200
H	2.82939400	-1.49813100	3.99272700
C	2.05530300	-1.73034700	-1.88558100
H	1.47238400	-2.65637600	-1.93766400
H	1.35344600	-0.89264600	-1.96686900
H	2.72906800	-1.69841200	-2.74365500
O	1.22318700	1.05343400	0.54636600
C	2.40227700	1.37330100	-0.03362000
C	3.44762600	1.53553200	0.89089900
C	2.64952900	1.54780500	-1.40622300
C	4.73149500	1.81053300	0.46623000
H	3.22499500	1.40973000	1.94445800
C	3.94065500	1.82499500	-1.83122300

H	1.84570800	1.45263600	-2.11837500
C	4.96718300	1.93485000	-0.90063700
H	5.54738800	1.91539900	1.17098700
H	4.15937800	1.94646400	-2.88509600
N	6.32216200	2.18383400	-1.36411000
O	6.49950700	2.39045200	-2.55453800
O	7.22608200	2.16846000	-0.53868900
H	-3.35047400	-3.35727000	-0.87453500
C	-2.68547400	-0.90203600	-1.51302200
H	-2.03717700	-0.02113700	-1.56498000
H	-2.24987800	-1.65926000	-2.17547700
C	-4.08800500	-0.56494400	-1.96688800
C	-4.67268600	-1.21886500	-3.05313400
C	-4.83078300	0.41110400	-1.29392400
C	-5.96455500	-0.89701200	-3.46670400
H	-4.10923900	-1.98243500	-3.58325600
C	-6.12111300	0.73272400	-1.70149100
H	-4.39652400	0.92759900	-0.44274600
C	-6.69226400	0.08007700	-2.79270700
H	-6.40205200	-1.41173600	-4.31632700
H	-6.67672100	1.49744500	-1.16709600
H	-7.69775100	0.33135300	-3.11436600

M1

0 1

C	-0.19215900	-1.21497800	0.46732400
C	-0.94219400	-3.14137100	1.19088200
C	-2.73256200	-1.53130900	0.46920200
H	-2.95465200	-0.65180400	1.07843800
N	0.34998000	-3.27175900	1.17150800
N	0.80108200	-2.06960300	0.70731100
N	-1.32321600	-1.90371500	0.73472700
C	-1.96553900	-4.10932800	1.70488000
H	-1.59152500	-4.58379600	2.61289400
H	-2.17052900	-4.88464400	0.95401700
C	-3.62423800	-2.71562300	0.88807600
O	-3.13371900	-3.38995200	2.02722100
C	2.19477200	-1.93677500	0.36405500

C	2.55151300	-2.21835600	-0.95734300
C	3.10946100	-1.56886300	1.34812100
C	3.89602500	-2.08018200	-1.29376700
C	4.43893400	-1.42515200	0.95470200
C	4.84523400	-1.65984000	-0.35985300
H	4.20391800	-2.27799600	-2.31781300
H	5.17053900	-1.10484000	1.69236100
H	-4.61883700	-2.33811800	1.12710500
C	-0.07708200	0.25242700	-0.09028300
O	0.00470700	0.27730300	-1.35878000
C	-1.11382000	1.14814000	0.58614000
C	-1.81909500	2.01959800	-0.13639400
H	-1.18340700	1.09372500	1.67160500
H	-1.67702200	1.98421800	-1.21680600
C	-2.78209100	3.00682200	0.38135600
C	-2.83393900	3.37221900	1.73377900
C	-3.69898400	3.58983400	-0.50392300
C	-3.78651100	4.27725200	2.18695700
H	-2.11311000	2.95778500	2.43207700
C	-4.65525800	4.49363000	-0.05056300
H	-3.66624700	3.31501600	-1.55516800
C	-4.70335600	4.83855500	1.29810300
H	-3.81014600	4.55228600	3.23667300
H	-5.36047700	4.92920500	-0.75122500
H	-5.44453700	5.54607500	1.65535600
C	6.27014800	-1.41676900	-0.78274600
H	6.93984900	-1.37541900	0.07887200
H	6.62225200	-2.19896500	-1.46016600
H	6.34836900	-0.46128700	-1.31518200
C	2.65715800	-1.29021600	2.75391700
H	1.92938800	-0.47237800	2.75471600
H	2.17273500	-2.16863300	3.19151500
H	3.50341900	-1.01016600	3.38311800
C	1.51102800	-2.61220200	-1.97076900
H	0.93442900	-3.47853400	-1.62885200
H	0.81601000	-1.77840300	-2.12726500
H	1.98036100	-2.86727500	-2.92218400
O	1.16300000	0.70789600	0.74597200

C	2.30186300	1.18352000	0.23763500
C	3.14074500	1.82836600	1.17291100
C	2.74386700	1.02625900	-1.09038800
C	4.40778700	2.24712900	0.82183700
H	2.77118800	1.95956400	2.18411000
C	4.01703800	1.44332300	-1.43910600
H	2.09721400	0.55034100	-1.81173500
C	4.84273000	2.03198600	-0.48516600
H	5.06737800	2.71991000	1.53899600
H	4.38794000	1.29776000	-2.44688800
N	6.19002100	2.41608000	-0.85331600
O	6.60272000	2.09768900	-1.96245000
O	6.86209900	3.03397200	-0.03956500
H	-3.71386700	-3.41995700	0.04671500
C	-2.93011600	-1.23433300	-1.02369500
H	-2.25221800	-0.43404800	-1.33341400
H	-2.64648300	-2.13186400	-1.58579700
C	-4.36854800	-0.87303900	-1.31528500
C	-5.17789900	-1.69995200	-2.09663900
C	-4.91454700	0.30265700	-0.79026500
C	-6.50271900	-1.35438200	-2.35803200
H	-4.76551700	-2.61670300	-2.51023300
C	-6.23569200	0.65152200	-1.05101500
H	-4.29694600	0.95632200	-0.18071600
C	-7.03461600	-0.17767400	-1.83729300
H	-7.11796000	-2.00476800	-2.97160000
H	-6.63599400	1.57520400	-0.64408400
H	-8.06506100	0.09253300	-2.04433900

TS2

0 1

C	-0.25387500	-1.24957600	0.20096700
C	-0.90651100	-3.09638200	1.16883700
C	-2.77596800	-1.52549900	0.55582200
H	-2.91203700	-0.60724400	1.13267500
N	0.37461100	-3.23397400	0.99966000
N	0.76556100	-2.08559400	0.38102700
N	-1.34372300	-1.89979200	0.66128800

C	-1.85794400	-4.01088500	1.87983500
H	-1.37368600	-4.41785900	2.76830300
H	-2.15785100	-4.83909400	1.22339000
C	-3.60641100	-2.66901900	1.17008500
O	-2.97356900	-3.25281000	2.28777800
C	2.14808400	-1.94142000	-0.00443200
C	2.48337200	-2.15222400	-1.34353800
C	3.08809400	-1.67518600	0.99376800
C	3.82490800	-1.99496200	-1.68967300
C	4.41136700	-1.52060800	0.59044500
C	4.79337700	-1.65365700	-0.74522700
H	4.11713000	-2.14127800	-2.72666700
H	5.15965800	-1.27241000	1.33902400
H	-4.56424300	-2.26754600	1.50104200
C	-0.27380700	0.14216200	-0.45379700
O	0.02518200	0.19605400	-1.63960500
C	-1.15381600	1.14116800	0.21584900
C	-1.80278700	2.05105500	-0.51948600
H	-1.21187800	1.10317800	1.29942200
H	-1.69189000	1.99357600	-1.60216300
C	-2.68820600	3.10234400	0.00141300
C	-2.63504500	3.53489000	1.33371400
C	-3.64064200	3.66999800	-0.85552700
C	-3.52536400	4.49597800	1.79765600
H	-1.88005600	3.13222600	2.00206900
C	-4.53588600	4.62727000	-0.38872400
H	-3.68419200	3.34273500	-1.89100300
C	-4.48136700	5.04094500	0.94045300
H	-3.46976500	4.82735900	2.82949700
H	-5.27194200	5.05204300	-1.06345400
H	-5.17460600	5.79105400	1.30676300
C	6.21599100	-1.39651200	-1.16500900
H	6.91306000	-1.59183900	-0.34684700
H	6.49720200	-2.01759400	-2.01892600
H	6.33861900	-0.34784000	-1.46217000
C	2.67642300	-1.53031500	2.43245400
H	1.87625300	-0.78830200	2.51493500
H	2.30802300	-2.48057700	2.83120600

H	3.52375800	-1.20418400	3.03786100
C	1.45355400	-2.56399900	-2.36164100
H	0.87204400	-3.42023500	-2.00386100
H	0.76079800	-1.74307700	-2.56237300
H	1.93995700	-2.85031500	-3.29544100
O	1.19461000	0.73596000	0.85751900
C	2.35770400	1.18500500	0.52499800
C	3.24857300	1.63501600	1.54974500
C	2.83863000	1.22153900	-0.81936000
C	4.54333000	2.00284900	1.26880500
H	2.87917500	1.64115900	2.57016900
C	4.13590800	1.58590200	-1.09561100
H	2.17820000	0.91298400	-1.61899000
C	4.99756600	1.95710400	-0.05559500
H	5.22397200	2.31350500	2.05293500
H	4.51152300	1.57645800	-2.11274000
N	6.35827900	2.28991700	-0.34740400
O	6.76388000	2.16602200	-1.50366100
O	7.08219200	2.68141600	0.56483100
H	-3.79550300	-3.43571000	0.40356500
C	-3.17539500	-1.31750900	-0.91221400
H	-2.54733200	-0.54637700	-1.36594700
H	-2.99101900	-2.25147200	-1.45443700
C	-4.63154600	-0.92411200	-1.01580500
C	-5.58395600	-1.80574500	-1.52955200
C	-5.04374400	0.33733700	-0.57489500
C	-6.92426000	-1.43180700	-1.60832700
H	-5.27331600	-2.78696500	-1.87916700
C	-6.38017400	0.71430400	-0.65633800
H	-4.31139100	1.03367700	-0.17485800
C	-7.32492700	-0.17124400	-1.17323300
H	-7.65358900	-2.12570200	-2.01358800
H	-6.68015700	1.70206500	-0.32023200
H	-8.36786500	0.12132900	-1.23806200

M2

0 1

C	-0.28406700	-1.29697000	-0.00472400

C	-0.84854700	-3.13620900	1.02308100
C	-2.77039400	-1.57616000	0.56918300
H	-2.85649100	-0.67146700	1.17592500
N	0.41821400	-3.26498300	0.75902000
N	0.75485200	-2.12235300	0.10493400
N	-1.33214600	-1.94690200	0.54403100
C	-1.73238700	-4.05902700	1.80667100
H	-1.17499600	-4.45690400	2.65550000
H	-2.07085700	-4.89259000	1.17657100
C	-3.54150000	-2.73182000	1.23429200
O	-2.82228600	-3.31424500	2.29884200
C	2.12832700	-1.92444900	-0.29179500
C	2.45999300	-2.03161800	-1.64422600
C	3.07398200	-1.73634300	0.72252300
C	3.79381200	-1.80252800	-1.98365600
C	4.38886400	-1.51672500	0.32464100
C	4.76003400	-1.51065200	-1.02091600
H	4.08277600	-1.86383800	-3.02994400
H	5.13950000	-1.32516300	1.08723300
H	-4.47590900	-2.34051500	1.63675700
C	-0.34521500	0.07754200	-0.66544100
O	0.12163700	0.19178600	-1.77941500
C	-1.18075700	1.09073500	0.00713600
C	-1.75833600	2.05360500	-0.72593300
H	-1.28982600	1.01013500	1.08323300
H	-1.59157100	2.03356900	-1.80252000
C	-2.64707500	3.10477300	-0.22045800
C	-2.72877300	3.42879100	1.14189200
C	-3.47782100	3.77452300	-1.12799800
C	-3.63276100	4.38726600	1.58158900
H	-2.06925100	2.94371300	1.85502600
C	-4.38559200	4.73121300	-0.68572100
H	-3.41562100	3.53044200	-2.18483100
C	-4.46673600	5.03697800	0.67070700
H	-3.68349600	4.63512600	2.63669200
H	-5.02737300	5.23783700	-1.39885600
H	-5.17121100	5.78512900	1.01919700
C	6.17090700	-1.17398300	-1.42185900

H	6.88410800	-1.47629300	-0.65106300
H	6.44516200	-1.65974600	-2.36135600
H	6.27498100	-0.09087700	-1.56023700
C	2.69101700	-1.76798100	2.17675000
H	2.37881700	-2.77358300	2.47313300
H	3.54099600	-1.47045500	2.79258900
H	1.86397200	-1.07566100	2.36062300
C	1.45545600	-2.44512900	-2.68798700
H	0.85408800	-3.29002100	-2.33725700
H	0.77777100	-1.62531400	-2.93117700
H	1.97198700	-2.75360900	-3.59845700
O	1.18897100	0.72665900	1.06115600
C	2.35736200	1.17043600	0.81594400
C	3.29516200	1.41657100	1.88014100
C	2.83103300	1.42191800	-0.51945000
C	4.59800400	1.76857300	1.63054300
H	2.94531500	1.27421600	2.89804700
C	4.13522400	1.76877000	-0.76408600
H	2.13891100	1.28347400	-1.34185400
C	5.03532500	1.92153200	0.30446300
H	5.30630700	1.91933500	2.43735300
H	4.49395300	1.91908000	-1.77652800
N	6.39577700	2.23996100	0.04336300
O	6.77989700	2.30875100	-1.12875600
O	7.15948500	2.43145000	0.99103600
H	-3.77880400	-3.49565000	0.47826500
C	-3.30624500	-1.34021900	-0.84990200
H	-2.71121300	-0.57642700	-1.35642300
H	-3.20334300	-2.27043000	-1.41871300
C	-4.75269300	-0.90191000	-0.79637400
C	-5.78061300	-1.74818200	-1.21449000
C	-5.07529100	0.36432200	-0.29800500
C	-7.10982700	-1.33563900	-1.13907500
H	-5.53989300	-2.73244800	-1.60802300
C	-6.40126000	0.77819200	-0.22339700
H	-4.28246500	1.03511000	0.02465300
C	-7.42255800	-0.07286600	-0.64347600
H	-7.89982800	-2.00182600	-1.47035500

H	-6.63402000	1.76748500	0.15813500
H	-8.45732100	0.24891600	-0.58720200

M03

0 1

C	-0.45492600	-1.76910300	-0.54568800
C	0.46452300	-2.17072800	-2.46632100
C	2.08621100	-1.67146200	-0.62909700
N	-0.81433500	-2.33684800	-2.66661100
N	-1.37658000	-2.07965000	-1.45700200
N	0.72575200	-1.86883900	-1.16310400
C	1.55851200	-2.12285000	-3.49263800
H	1.49770800	-2.98059600	-4.16361400
H	1.40882300	-1.19906500	-4.06921900
C	2.90848400	-1.18625900	-1.81616700
O	2.82500000	-2.13479500	-2.87470800
C	-2.78615900	-2.27169500	-1.24691800
C	-3.67945100	-1.49655900	-1.99857600
C	-3.19756600	-3.22776700	-0.31111200
C	-5.03724000	-1.68229500	-1.75106000
C	-4.57065600	-3.36877000	-0.10841300
C	-5.50091400	-2.60142600	-0.80707400
H	-5.75330900	-1.08850700	-2.31341100
H	-4.91750600	-4.10584600	0.61100700
H	3.95969300	-1.11059400	-1.53329600
C	-0.63999600	-1.33714300	0.89008100
O	0.19397900	-1.70720200	1.70002300
C	-1.80763600	-0.50836900	1.15828700
C	-2.02633600	-0.04724400	2.40252400
H	-2.41814400	-0.20500500	0.31586600
H	-1.36400300	-0.38817600	3.19723600
C	-3.11029000	0.86035400	2.78056600
C	-3.46552800	0.97751600	4.13015300
C	-3.79866500	1.62284400	1.82260200
C	-4.50704300	1.81465300	4.51764000
H	-2.92354700	0.40086900	4.87467400
C	-4.83308400	2.46242400	2.21406300
H	-3.48975500	1.58244000	0.78072300

C	-5.19460100	2.55572100	3.55958000
H	-4.77833600	1.89191500	5.56531200
H	-5.35560100	3.05540000	1.47055400
H	-6.00347900	3.21398200	3.85964600
C	-6.97651300	-2.74712300	-0.54420100
H	-7.32356200	-1.95766500	0.12988600
H	-7.20275200	-3.70803000	-0.07802100
H	-7.55089400	-2.66480100	-1.46979400
C	-3.20903800	-0.51583200	-3.03813100
H	-2.94492000	-1.04073500	-3.96114000
H	-2.32583400	0.03436400	-2.70674500
H	-4.00581800	0.19517900	-3.26337200
C	-2.23166800	-4.09504700	0.45699100
H	-1.34555300	-4.34655800	-0.13204500
H	-2.72180900	-5.02681200	0.74345800
H	-1.89704000	-3.60662400	1.37808800
H	2.53104500	-0.20607900	-2.13942700
C	0.66510900	1.65642600	1.10430300
C	-0.25809400	2.46625900	1.66593200
C	1.96075200	1.41758400	1.80968500
C	2.01481100	1.26892700	3.19932300
C	3.15621000	1.37358400	1.08111800
C	3.23731500	1.12043000	3.85049700
H	1.08775800	1.26432700	3.76509400
C	4.37823700	1.23137000	1.73043500
H	3.10660400	1.47518900	0.00027400
C	4.42313700	1.11449000	3.11914300
H	3.26335300	1.00726400	4.92995600
H	5.29829700	1.20845600	1.15423900
H	5.37694600	1.00673900	3.62644500
N	0.58848400	0.97433200	-0.10739300
S	-0.30905400	1.53458500	-1.29372200
O	-1.74278100	1.74790800	-0.98515400
O	-0.05498300	0.65691500	-2.45878400
C	0.33750600	3.14758400	-1.71435700
C	-0.33740000	4.29903700	-1.32984900
C	1.55875800	3.22651100	-2.38450000
C	0.21764700	5.54418200	-1.62383100

H	-1.29144100	4.21411500	-0.82168100
C	2.09799300	4.47192300	-2.67237200
H	2.06743000	2.31562000	-2.68580100
C	1.43674300	5.64882000	-2.29377200
H	-0.30757300	6.44789500	-1.32805800
H	3.04470100	4.54010900	-3.20160100
C	2.04064300	6.99187200	-2.61148200
H	1.39323900	7.80598100	-2.28010700
H	2.20481700	7.10142500	-3.68735500
H	3.01171500	7.10741200	-2.12108500
H	-1.22198000	2.63481400	1.20185300
H	-0.03632100	2.98264400	2.59265100
C	2.57536200	-3.00403500	-0.04803400
H	1.84382600	-3.32611300	0.69924500
H	2.59788600	-3.74687500	-0.85174200
C	3.94013900	-2.87304100	0.57944700
C	5.06744500	-3.41249900	-0.04563000
C	4.09594800	-2.19309000	1.79057300
C	6.32695800	-3.29595900	0.53746800
H	4.95191800	-3.92979900	-0.99484200
C	5.35333200	-2.08220300	2.37804100
H	3.22764400	-1.74620200	2.27302800
C	6.47072400	-2.63545800	1.75554100
H	7.19329500	-3.72370900	0.04299000
H	5.45812900	-1.55756900	3.32208800
H	7.44938500	-2.54945200	2.21667000
H	2.01870500	-0.88959600	0.13006800

TS3(R)

0 1

C	-0.51991100	-1.77739800	-0.51072200
C	0.37707800	-2.29537200	-2.42124000
C	2.02264500	-1.73341700	-0.62384200
N	-0.90384400	-2.45008800	-2.60403900
N	-1.45148600	-2.12402000	-1.39959000
N	0.65381600	-1.92243100	-1.13804500
C	1.46625000	-2.34385800	-3.45284100
H	1.35250000	-1.45102600	-4.08448600

H	1.37005800	-3.23573700	-4.07365700
C	2.85868700	-1.36102400	-1.84117000
O	2.73487300	-2.36876900	-2.83909300
C	-2.85909800	-2.29394800	-1.16594500
C	-3.26220200	-3.24624400	-0.22269400
C	-3.75493300	-1.50311700	-1.89103700
C	-4.63055500	-3.37547800	0.00427400
C	-5.11231100	-1.67517100	-1.62187300
C	-5.56661800	-2.59521600	-0.67641500
H	-4.97377100	-4.10960300	0.72882100
H	-5.83176800	-1.06863400	-2.16613200
H	2.52181400	-0.38883800	-2.22724300
C	-0.66357600	-1.19314200	0.87251700
O	0.23379100	-1.44919000	1.68934900
C	-1.76622800	-0.32534600	1.05215100
C	-1.78818000	0.48579400	2.18068200
H	-1.15340300	0.17005100	3.00545100
C	-2.97859300	1.28299600	2.55026500
C	-3.25619100	1.52422600	3.89938200
C	-3.84041800	1.80295300	1.57444400
C	-4.38694400	2.24628900	4.27262200
H	-2.58297000	1.13386600	4.65835800
C	-4.96608000	2.52707800	1.94863100
H	-3.60404100	1.65977600	0.52291100
C	-5.24634500	2.74669200	3.29800300
H	-4.59469000	2.41922100	5.32377200
H	-5.62664300	2.92780500	1.18625300
H	-6.12655900	3.31246700	3.58588800
C	-7.03688100	-2.73141300	-0.37902100
H	-7.29057700	-3.75851400	-0.10694800
H	-7.31797900	-2.08812800	0.46098500
H	-7.64313400	-2.43912900	-1.23892600
C	-2.27345800	-4.10879300	0.51928100
H	-1.49257100	-4.48865200	-0.14683000
H	-1.78186400	-3.55669000	1.32727500
H	-2.78451400	-4.96272600	0.96629600
C	-3.28055000	-0.51128200	-2.91808300
H	-3.02013000	-1.02129000	-3.85065700

H	-4.06865000	0.21329600	-3.13079900
H	-2.39056800	0.02447600	-2.57892100
H	3.91331400	-1.30910300	-1.56625300
C	0.61042300	1.57191100	1.13356100
C	-0.48948800	2.19311900	1.71563700
C	1.83415400	1.32310800	1.94981700
C	1.77007600	1.15274100	3.33781700
C	3.08673200	1.30580700	1.32533000
C	2.93144500	0.98612800	4.08403400
H	0.80590300	1.13869800	3.83674300
C	4.24946100	1.14909100	2.07402900
H	3.13159400	1.43840900	0.24810500
C	4.17643900	0.99401200	3.45623500
H	2.86504600	0.84972200	5.15888200
H	5.21462400	1.14418800	1.57678100
H	5.08356900	0.87626100	4.04120200
N	0.67607500	1.06678900	-0.11483800
S	-0.23508800	1.58942600	-1.34540100
O	-0.02233700	0.62445500	-2.43912300
O	-1.63850100	1.91788600	-1.03838000
C	0.54205300	3.12128800	-1.82480800
C	1.72551400	3.08406100	-2.55863600
C	-0.00172500	4.32942400	-1.40062200
C	2.36554500	4.27664600	-2.87149600
H	2.12742300	2.13065200	-2.88718600
C	0.65405300	5.51479100	-1.72004500
H	-0.93004800	4.33561000	-0.83998400
C	1.84146600	5.50650000	-2.45666800
H	3.28618800	4.25681700	-3.44783800
H	0.23628800	6.46263100	-1.39224800
C	2.52695700	6.79643200	-2.82402100
H	2.36421800	7.56102700	-2.06117500
H	3.60255300	6.65131400	-2.94670400
H	2.13519200	7.18428400	-3.77000500
H	-0.37157500	2.65233100	2.69108000
H	-2.47475600	-0.18366600	0.24662200
H	-1.27322500	2.58740500	1.08433100
C	2.46160800	-3.03972000	0.05086400

H	1.72937700	-3.25672200	0.83415300
H	2.42835300	-3.84288500	-0.69268100
C	3.84456900	-2.92952100	0.63935800
C	4.94446700	-3.49418800	-0.01222500
C	4.04728200	-2.23596800	1.83531600
C	6.22342000	-3.38561900	0.52814900
H	4.79158300	-4.02291000	-0.95011600
C	5.32500700	-2.13036200	2.37899100
H	3.19781300	-1.77635700	2.33842200
C	6.41495000	-2.70672800	1.72950800
H	7.06765300	-3.83402400	0.01385500
H	5.46752000	-1.59505800	3.31239100
H	7.40914900	-2.62502900	2.15726000
H	1.98857400	-0.90577500	0.08795700

TS3(S)

0 1

C	0.59374900	-2.09751700	-0.19773800
C	-0.11364500	-3.37967800	-1.81029800
C	-1.82927200	-2.91461700	-0.04300500
N	1.14695300	-3.17847200	-2.07159500
N	1.57846800	-2.38930400	-1.05404500
N	-0.48324800	-2.76693500	-0.64724000
C	-1.08264700	-4.21847500	-2.59242900
H	-1.05230400	-3.93003100	-3.64407500
H	-0.78991000	-5.27425800	-2.50618000
C	-2.45635800	-4.13889200	-0.70795100
O	-2.39292700	-4.01459700	-2.11979800
C	2.97901500	-2.07549300	-0.93656000
C	3.67113900	-2.56755900	0.17770700
C	3.57529700	-1.27629200	-1.91674700
C	4.99477400	-2.16529600	0.33436500
C	4.90383900	-0.90357800	-1.70479000
C	5.61831200	-1.31387400	-0.57996400
H	5.55094500	-2.52316500	1.19698400
H	5.38675400	-0.26707300	-2.44172800
H	-3.51134000	-4.18802200	-0.43217400
C	0.60400800	-1.20901500	1.03733300

O	-0.25670500	-1.44980800	1.89225400
C	1.62764700	-0.22708800	1.07851700
C	1.69124500	0.69695000	2.10369500
H	2.31212600	-0.15325400	0.24759000
H	1.03912400	0.53144300	2.95892300
C	2.91131600	1.49309800	2.35849400
C	3.16420100	1.96909400	3.64919600
C	3.83686100	1.77079000	1.34118800
C	4.32362800	2.68796500	3.92834900
H	2.44612700	1.76563600	4.43962100
C	4.99145600	2.49184600	1.62051900
H	3.63721900	1.44165300	0.32440500
C	5.24253300	2.94878900	2.91544300
H	4.50784700	3.04425300	4.93677200
H	5.69695100	2.70505700	0.82341100
H	6.14602600	3.51026400	3.12969400
C	7.02567700	-0.83542200	-0.33989900
H	7.49923100	-0.51086500	-1.26854700
H	7.63909300	-1.61971100	0.10969000
H	7.02068400	0.01509700	0.34994100
C	3.02924100	-3.47626600	1.19444900
H	2.33482000	-4.18196600	0.73039300
H	2.47671200	-2.90178100	1.94540400
H	3.79717000	-4.04926300	1.71653300
C	2.84704300	-0.84911200	-3.16122400
H	2.88779500	-1.64792300	-3.90883600
H	3.32062700	0.03905900	-3.58330500
H	1.79894300	-0.62503900	-2.95954700
H	-1.95802000	-5.06326700	-0.38416900
C	-0.63726200	1.80035200	0.98273400
C	0.50835900	2.48428400	1.34661700
C	-1.81445400	1.77571000	1.90104400
C	-1.67490700	1.90909800	3.28788100
C	-3.09956600	1.63689600	1.36411900
C	-2.79408400	1.90639600	4.11472700
H	-0.68473900	1.99968200	3.72520200
C	-4.21851800	1.64582400	2.18987000
H	-3.20377400	1.52378400	0.28925100

C	-4.07021300	1.77824800	3.56845200
H	-2.66871300	1.99858500	5.18902600
H	-5.20752700	1.52691800	1.75740100
H	-4.94233500	1.77209600	4.21472100
N	-0.79155000	1.01354900	-0.10076800
S	0.03221800	1.14525800	-1.47514800
O	-0.22068000	-0.10977300	-2.20893700
O	1.44202400	1.56914400	-1.38269800
C	-0.82526700	2.44253000	-2.34809800
C	-2.11359700	2.19453600	-2.82320900
C	-0.23336800	3.68942800	-2.49647700
C	-2.80655800	3.21395500	-3.45892700
H	-2.55359900	1.20989800	-2.69611600
C	-0.94416700	4.70381100	-3.13729300
H	0.77162200	3.85366400	-2.12324500
C	-2.23258700	4.48274100	-3.62463500
H	-3.80875500	3.02998100	-3.83645600
H	-0.48736000	5.68149500	-3.26078900
C	-2.99979700	5.57386800	-4.32483300
H	-2.45383000	6.51882800	-4.30340700
H	-3.97428500	5.72940000	-3.85353500
H	-3.18257000	5.30999000	-5.37083900
H	1.29271900	2.64884300	0.62135500
H	0.48245400	3.14165000	2.20850200
C	-2.66549700	-1.64755200	-0.23471500
H	-2.05199200	-0.77408600	0.01172000
H	-2.94440100	-1.57010800	-1.29148600
C	-3.88735800	-1.69717700	0.65026700
C	-3.73272400	-1.65031200	2.04073700
C	-5.17420200	-1.79613500	0.11834500
C	-4.84168200	-1.70162100	2.87802500
H	-2.73206000	-1.54103100	2.45535400
C	-6.28878200	-1.84035900	0.95595500
H	-5.30343900	-1.83429800	-0.96051400
C	-6.12430600	-1.79603300	2.33785400
H	-4.70708000	-1.65048800	3.95411300
H	-7.28355200	-1.91210400	0.52735800
H	-6.99041800	-1.83141100	2.99110000

H	-1.66763400	-3.09692000	1.01926500

M3(R)

0 1

C	3.24480900	-0.11233100	-0.19252200
C	5.36400900	-0.03236900	-0.72269000
C	4.26530400	2.19765500	-0.27596700
H	3.63961100	2.51859300	-1.11214700
N	5.05343900	-1.29479100	-0.79544400
N	3.72735500	-1.32802300	-0.46103400
N	4.28675500	0.72312400	-0.34559200
C	6.66654900	0.63823900	-1.04012800
H	7.15747200	0.11856700	-1.86329400
H	7.32533700	0.61894400	-0.16083900
C	5.71199300	2.67737600	-0.43605000
O	6.40199700	1.96338300	-1.44576500
C	2.98038400	-2.55562900	-0.55237100
C	3.15252700	-3.51374800	0.44480700
C	2.11161200	-2.71998100	-1.63759900
C	2.39518100	-4.68386300	0.34750300
C	1.37238300	-3.89947800	-1.68213800
C	1.49232700	-4.88473100	-0.69566700
H	2.50251500	-5.44277800	1.11815400
H	0.68597300	-4.05847000	-2.51107000
H	5.70746500	3.73029100	-0.72155900
C	1.80420400	0.30627600	-0.02517500
O	1.47473200	1.25975100	-0.80872700
C	1.09755600	-0.44107700	0.86654900
C	-0.40304300	-0.40318600	0.97715400
H	1.62420500	-1.20468700	1.42941600
H	-0.75816300	0.52696200	1.44061800
C	-0.92605800	-1.57742300	1.78469300
C	-1.93474600	-1.41460300	2.73506400
C	-0.45915300	-2.86713200	1.51028100
C	-2.46180900	-2.52157700	3.40179500
H	-2.31883400	-0.41713000	2.93499600
C	-0.98281600	-3.97153700	2.17230200
H	0.31279300	-3.00022700	0.75661600

C	-1.98974300	-3.80132800	3.12311400
H	-3.24485300	-2.38124500	4.14098100
H	-0.60330700	-4.96484900	1.94622700
H	-2.40281200	-4.66085900	3.64208100
C	0.61908700	-6.11031700	-0.74982600
H	-0.42119900	-5.83730700	-0.53830500
H	0.64052100	-6.56702900	-1.74330400
H	0.93289300	-6.85721900	-0.01773400
C	4.10242400	-3.27630700	1.58769100
H	5.13375600	-3.20313400	1.23076900
H	3.86933700	-2.33806200	2.10257400
H	4.04147200	-4.08882200	2.31272000
C	1.98844800	-1.66687000	-2.70813900
H	2.96564600	-1.45706400	-3.15604300
H	1.31892500	-2.00897600	-3.49953600
H	1.60143200	-0.72140800	-2.31132200
H	6.24360800	2.57458000	0.52255300
C	3.65282900	2.69504700	1.03994500
H	2.77309000	2.09364600	1.27969600
H	4.37978100	2.56239500	1.84899400
C	3.24311400	4.14272300	0.91035100
C	4.04835900	5.17763400	1.38725200
C	2.03899600	4.45017100	0.26755500
C	3.65938200	6.50638200	1.22555100
H	4.98089100	4.94291700	1.89539800
C	1.65106600	5.77680900	0.10758900
H	1.41891800	3.63234200	-0.09769000
C	2.46032900	6.80750800	0.58520500
H	4.29147200	7.30395000	1.60315100
H	0.71271400	6.00771500	-0.38683700
H	2.15466600	7.84159900	0.46153000
C	-2.46754200	-0.77472600	-0.43958400
C	-0.99789300	-0.45928100	-0.47390000
C	-2.86415800	-2.18706000	-0.70558300
C	-2.19510200	-2.95966900	-1.66155400
C	-3.88783200	-2.76687500	0.05077600
C	-2.54569700	-4.29117500	-1.85763900
H	-1.41313600	-2.51823700	-2.27124000

C	-4.21699100	-4.10578100	-0.12690200
H	-4.38601600	-2.16478000	0.80306900
C	-3.54702800	-4.87066900	-1.08055300
H	-2.03227800	-4.87813100	-2.61330400
H	-4.99266300	-4.55572100	0.48418000
H	-3.80512200	-5.91575200	-1.21940600
N	-3.43288500	0.01171700	-0.09652300
S	-3.17065000	1.60254700	0.36633500
O	-3.16667900	1.61982800	1.83120200
O	-2.06830900	2.25576700	-0.33878000
C	-4.68243300	2.33598000	-0.19738800
C	-5.87681000	2.01296500	0.44453100
C	-4.64227900	3.24749700	-1.24423800
C	-7.04984100	2.61641100	0.01549100
H	-5.87993000	1.30875700	1.27036200
C	-5.82999200	3.84428900	-1.65898600
H	-3.69480300	3.48690100	-1.71443500
C	-7.04356900	3.53718300	-1.04102800
H	-7.98751300	2.37828400	0.50942300
H	-5.81109400	4.56286100	-2.47306200
C	-8.32928700	4.16843900	-1.50565600
H	-8.13757400	5.06129500	-2.10389800
H	-8.90054600	3.46681800	-2.12180700
H	-8.95874300	4.44724900	-0.65711200
H	-0.77767900	0.48613300	-0.96848600
H	-0.46868800	-1.26284700	-0.98922200

M3(S)

0 1

C	-2.38438900	2.02506400	-0.07573100
C	-3.13441800	4.02065100	-0.55018100
C	-0.70614600	3.90164100	0.15419200
H	-0.57663000	3.74783800	1.22791800
N	-4.15967000	3.23463100	-0.71782400
N	-3.67086300	1.99076500	-0.42003800
N	-2.02332600	3.31679200	-0.17161100
C	-3.06083800	5.51480600	-0.65778200
H	-3.99330900	5.95548000	-0.30411000

H	-2.89846100	5.81232000	-1.70305300
C	-0.77687300	5.38973400	-0.20545800
O	-2.01276000	5.97274400	0.16814800
C	-4.51848100	0.82944700	-0.40440400
C	-4.88116400	0.25864900	-1.62659000
C	-4.89155200	0.30290400	0.83311800
C	-5.65402100	-0.89919300	-1.58459800
C	-5.66327600	-0.85988100	0.82044500
C	-6.04956300	-1.47220900	-0.37193900
H	-5.94813600	-1.36972700	-2.51956500
H	-5.96172500	-1.29987600	1.76832800
H	0.01285000	5.92210700	0.32652000
C	-1.51878100	0.94521100	0.51361300
O	-0.95293900	1.31075900	1.59616800
C	-1.46061200	-0.21304800	-0.19996500
C	-0.57585300	-1.33097900	0.25434600
H	-2.05007400	-0.32140900	-1.10668000
H	0.12340300	-0.89575900	0.97684200
C	-1.30300100	-2.46723900	0.95705500
C	-0.64157900	-3.21937700	1.93197500
C	-2.60836500	-2.82191500	0.60424800
C	-1.26169000	-4.31435400	2.52975000
H	0.36946400	-2.93816600	2.22145400
C	-3.23133600	-3.91631000	1.20044000
H	-3.14551200	-2.22422400	-0.12905400
C	-2.55781900	-4.66963500	2.16096200
H	-0.73439400	-4.88780100	3.28579200
H	-4.24826500	-4.17570800	0.91885400
H	-3.04356400	-5.52116500	2.62682400
C	-6.88453800	-2.72668400	-0.36407800
H	-6.44196600	-3.49380100	-1.00566800
H	-6.97855400	-3.13290200	0.64494400
H	-7.89109200	-2.52561700	-0.74335600
C	-4.43025500	0.86988600	-2.92639500
H	-4.85669700	1.86825200	-3.05925800
H	-3.34046400	0.97726500	-2.95199700
H	-4.73377100	0.24764200	-3.76923300
C	-4.46748700	0.95612300	2.12204000

H	-4.72770100	2.01947800	2.12267600
H	-4.96517000	0.48087300	2.96838900
H	-3.38484800	0.88317300	2.27303000
H	-0.61859600	5.51683200	-1.28738200
C	0.42412700	3.19450500	-0.60608100
H	0.27786000	2.11209300	-0.56510500
H	0.39322800	3.50092600	-1.65800400
C	1.76967500	3.50655500	0.00405000
C	2.67980500	4.36759900	-0.60948500
C	2.11784500	2.89289100	1.21372800
C	3.92477200	4.61433700	-0.02659600
H	2.41921300	4.84088400	-1.55328600
C	3.35765200	3.13725800	1.79374700
H	1.41319100	2.19676900	1.66483800
C	4.26449100	4.00075000	1.17559100
H	4.62647500	5.28322900	-0.51534200
H	3.62469100	2.63877600	2.72041700
H	5.23546800	4.18398700	1.62498000
C	1.46968000	-2.65604200	-0.59119900
C	0.21768200	-1.89572000	-0.96042600
C	1.53320500	-4.12290300	-0.83752500
C	0.37094600	-4.90283700	-0.88056700
C	2.77976100	-4.74091700	-1.00741900
C	0.45872200	-6.27747300	-1.08109700
H	-0.60284400	-4.44959300	-0.72434400
C	2.86148500	-6.10909800	-1.22787000
H	3.67555600	-4.12992400	-0.97506500
C	1.70001000	-6.88133700	-1.26183600
H	-0.44622400	-6.87544700	-1.09629400
H	3.82998700	-6.57577600	-1.37343800
H	1.76421900	-7.95157300	-1.42956700
N	2.53791000	-2.13913500	-0.08052200
S	2.67708200	-0.51167600	0.29325800
O	1.92590800	0.36992500	-0.60160700
O	2.44936200	-0.40348100	1.73515900
C	4.39575900	-0.24425000	-0.04532100
C	4.75318800	1.00234200	-0.55156300
C	5.35435500	-1.19757000	0.29161800

C	6.10198400	1.28867700	-0.73183500
H	3.98639200	1.73494300	-0.78807100
C	6.69548100	-0.89452500	0.09225000
H	5.05032500	-2.15851400	0.69231800
C	7.08771100	0.34760900	-0.42119900
H	6.39157000	2.26255400	-1.11655700
H	7.45296100	-1.63107200	0.34365000
C	8.54310200	0.65451700	-0.65643100
H	8.74409200	1.72209900	-0.54392700
H	9.18063400	0.10454400	0.03902800
H	8.83537300	0.36790700	-1.67188300
H	0.52509800	-1.05158000	-1.57995500
H	-0.43866700	-2.53040800	-1.55930500

TS4(S)^{HOAr}

0 1

C	2.56407200	0.49951000	-0.94348900
C	4.59242200	-0.24924200	-0.71576400
C	2.82736600	-2.01571500	-1.08955500
N	4.69151200	1.04507700	-0.58389000
N	3.41225600	1.49911300	-0.72303100
N	3.29957400	-0.61884900	-0.96422000
C	5.65473700	-1.29818300	-0.54717800
H	6.54560200	-1.02980300	-1.11630400
H	5.91704800	-1.35944700	0.52034200
C	3.92288900	-2.87520700	-0.45865900
O	5.17912600	-2.53329100	-1.02965500
C	3.11454200	2.90948800	-0.71251400
C	3.47193700	3.65447400	0.42504200
C	2.50162000	3.47857700	-1.83284700
C	3.15566900	5.00784500	0.42292000
C	2.21876500	4.84686500	-1.77730400
C	2.52347100	5.62133500	-0.66436500
H	3.41359200	5.60332600	1.29487400
H	1.73730700	5.30851300	-2.63546500
H	3.73250200	-3.92497000	-0.68985100
C	1.04933100	0.51348000	-1.09445400
O	0.60946100	-0.02339100	-2.10568000

C	0.32770400	0.97015300	0.05435700
C	-1.15414400	1.19592200	-0.19318900
H	0.84723900	1.74114400	0.63020300
H	-1.47896700	0.39155700	-0.86031700
C	-1.47336200	2.50917800	-0.88953500
C	-2.56558300	2.56609800	-1.76323700
C	-0.75065700	3.68045100	-0.64427000
C	-2.94097000	3.76778200	-2.35708000
H	-3.11891300	1.65346000	-1.97572100
C	-1.12501400	4.88420100	-1.23970100
H	0.11566300	3.66512400	0.01369000
C	-2.22459300	4.93357000	-2.09253600
H	-3.79185400	3.79268800	-3.03062000
H	-0.54793600	5.78255500	-1.03742700
H	-2.51640200	5.87115600	-2.55491300
C	2.18326300	7.08727800	-0.61625100
H	1.44206800	7.28454800	0.16470900
H	1.77573700	7.43124200	-1.56886800
H	3.06795700	7.68588400	-0.38283700
C	4.17952800	3.04494000	1.60690800
H	5.19059600	2.72836300	1.34011800
H	3.65318400	2.16572400	1.98723100
H	4.24410000	3.77227800	2.41690800
C	2.12372000	2.70922000	-3.07198200
H	2.74146800	1.82190800	-3.22857400
H	2.22995100	3.35396000	-3.94618600
H	1.07796400	2.38800500	-3.02229500
H	3.94618100	-2.74310700	0.62946100
C	-3.43534600	1.13288100	0.97512400
C	-1.93285600	1.05893500	1.13924900
C	-4.17043000	2.30265200	1.53169900
C	-3.55404400	3.55200800	1.66472400
C	-5.51469900	2.15917100	1.90037200
C	-4.27423200	4.63954400	2.14968200
H	-2.52373400	3.69233600	1.35577200
C	-6.22339700	3.24098400	2.40505100
H	-5.98636200	1.18833300	1.79283200
C	-5.60501700	4.48546000	2.52757000

H	-3.79261200	5.60817600	2.23254300
H	-7.25916800	3.11593300	2.70286300
H	-6.16090200	5.33256600	2.91667300
N	-4.18296500	0.26074500	0.38761700
S	-3.61109100	-1.14307700	-0.32706800
O	-2.40811300	-1.69479400	0.29650900
O	-3.58864200	-0.88057700	-1.76718100
C	-4.95895900	-2.23193900	0.04040000
C	-4.75621200	-3.27577700	0.93312800
C	-6.18970200	-2.03035700	-0.58389100
C	-5.81680500	-4.13486300	1.21073100
H	-3.78426300	-3.40888100	1.39539300
C	-7.23321900	-2.89631200	-0.29345200
H	-6.31972300	-1.21221800	-1.28495400
C	-7.06236800	-3.95808200	0.60619700
H	-5.67196500	-4.95425700	1.90822000
H	-8.19850500	-2.75255400	-0.77055700
C	-8.20740300	-4.89148700	0.89707900
H	-8.46459900	-5.47429900	0.00750400
H	-9.10026400	-4.33236000	1.18967400
H	-7.95760300	-5.58780000	1.69946600
H	-1.68290100	0.08676900	1.57524100
H	-1.60734100	1.83484300	1.83617000
H	0.45597000	-0.11434000	0.82944700
O	0.51671000	-1.19751700	1.55372400
C	1.67455600	-1.43307200	2.08459100
C	2.70778700	-0.45170200	2.14452400
C	1.97476700	-2.72176600	2.61607700
C	3.96324500	-0.74979200	2.63271400
H	2.47994500	0.55303900	1.80696700
C	3.21835000	-3.01295400	3.13381500
H	1.19237900	-3.47238000	2.59205000
C	4.22030100	-2.03508800	3.11519200
H	4.75037200	-0.00244600	2.65835100
H	3.44573100	-3.99682800	3.52733700
N	5.54495500	-2.36287800	3.57514000
O	5.72123500	-3.43815100	4.13275300
O	6.44607600	-1.55284100	3.37642100

C	2.57548200	-2.38419000	-2.55921100
H	2.08323000	-1.54197100	-3.05063200
H	3.54127300	-2.56103900	-3.04318900
C	1.69162000	-3.60711300	-2.63146900
C	2.19464100	-4.85114200	-3.01348400
C	0.34231700	-3.49872600	-2.27407600
C	1.36447900	-5.97130700	-3.04364900
H	3.24135000	-4.94231200	-3.29334600
C	-0.48546400	-4.61588000	-2.30023000
H	-0.05767200	-2.53059700	-1.97751400
C	0.02433800	-5.85555200	-2.68629100
H	1.76687700	-6.93288700	-3.34638500
H	-1.52939200	-4.51715700	-2.01926900
H	-0.62164800	-6.72735200	-2.70769300
H	1.90008900	-2.08250000	-0.50934900

TS4(R)^{HOAr}

0 1

C	-2.54008700	0.10472600	-0.92795100
C	-4.27264000	-1.17859200	-1.21931300
C	-2.04636800	-2.32573700	-1.54638900
H	-1.62885900	-2.12468500	-2.53596500
N	-4.76582400	-0.00447500	-0.94161500
N	-3.67107800	0.78795900	-0.75804600
N	-2.90453200	-1.15300600	-1.22627900
C	-5.01291500	-2.44748900	-1.52341800
H	-5.90134600	-2.22178800	-2.11395800
H	-5.31273200	-2.93088800	-0.58124600
C	-2.97246600	-3.54645900	-1.57498600
O	-4.17237300	-3.29019800	-2.27818600
C	-3.82463400	2.19290200	-0.47322100
C	-4.58416300	2.56095400	0.65082200
C	-3.22351000	3.12911800	-1.32199100
C	-4.66860300	3.91859400	0.94290500
C	-3.35045600	4.47672900	-0.97687900
C	-4.04626900	4.88968900	0.15402200
H	-5.24187000	4.22642800	1.81366400
H	-2.88164600	5.21782900	-1.61915000

H	-2.45659700	-4.36731300	-2.07502200
C	-1.09467700	0.58338800	-0.95507300
O	-0.47916200	0.17010900	-1.93503600
C	-0.61164700	1.33295000	0.16591200
C	0.75399800	1.96531800	-0.09212100
H	-1.37270700	1.98124300	0.60953200
H	1.18775900	1.38194800	-0.90832000
C	0.69352800	3.40168700	-0.57724200
C	1.51361300	3.80450600	-1.63598900
C	-0.11341100	4.35890100	0.04645400
C	1.52893100	5.13040400	-2.06374800
H	2.14494600	3.06539600	-2.12477700
C	-0.10059900	5.68524600	-0.37912300
H	-0.75914300	4.07027900	0.87289300
C	0.72126000	6.07528800	-1.43535300
H	2.16978300	5.42388800	-2.88914200
H	-0.73776900	6.41460700	0.11322900
H	0.72977700	7.10861100	-1.76706300
C	-4.12853700	6.34535200	0.52936500
H	-3.49767000	6.55202400	1.39998300
H	-3.79350700	6.98467500	-0.28974900
H	-5.15128500	6.62591600	0.79373500
C	-5.33389900	1.56973200	1.50420000
H	-6.24380200	1.23952600	0.99626000
H	-4.75124400	0.67405100	1.72704200
H	-5.61528700	2.03451000	2.45009900
C	-2.45718000	2.77294000	-2.56982500
H	-2.74607000	1.80583100	-2.98727900
H	-2.63296200	3.53579400	-3.33072700
H	-1.37974800	2.75062300	-2.37074400
H	-3.20532900	-3.84810500	-0.54090600
C	-0.92848400	-2.54330700	-0.51964200
H	-0.45558400	-1.60067900	-0.23701400
H	-1.36515400	-2.98145700	0.38089700
C	0.15145700	-3.44545500	-1.06578600
C	0.32374100	-4.74231200	-0.58216800
C	1.02312200	-2.95996700	-2.04775300
C	1.35249700	-5.54868000	-1.07088100

H	-0.34495300	-5.11814900	0.18926000
C	2.04638600	-3.76390600	-2.53721800
H	0.91849400	-1.93158600	-2.38711600
C	2.21235900	-5.06180900	-2.05113200
H	1.48030300	-6.55436000	-0.68265600
H	2.73079100	-3.36878100	-3.28163600
H	3.01863600	-5.68474400	-2.42556200
C	3.16442000	1.84045600	0.75120000
C	1.70241600	1.84737600	1.13162200
C	4.03675800	2.96692300	1.18211600
C	3.51182000	4.25078000	1.37149800
C	5.40874500	2.75111700	1.36656600
C	4.35050600	5.30154000	1.73214700
H	2.45582800	4.44337700	1.20772300
C	6.23784500	3.79732100	1.74816200
H	5.80694000	1.75284800	1.21768000
C	5.71020800	5.07633800	1.92837600
H	3.93786400	6.29655900	1.86095700
H	7.29623300	3.61787000	1.90564000
H	6.35905500	5.89520400	2.22205100
N	3.76178800	0.91116100	0.08349800
S	2.98847100	-0.43684000	-0.55344000
O	1.76377100	-0.83232700	0.14329600
O	2.92040800	-0.18960100	-1.99530700
C	4.20620400	-1.68198500	-0.22738900
C	3.76526400	-2.92093200	0.22251800
C	5.55046500	-1.44576000	-0.51716500
C	4.69734800	-3.94121700	0.39340200
H	2.70927200	-3.08353000	0.41902500
C	6.46350700	-2.47323500	-0.33077700
H	5.86888100	-0.47146900	-0.87292200
C	6.05148800	-3.73299700	0.12621300
H	4.36019300	-4.91596100	0.73385200
H	7.51428700	-2.30164500	-0.54650600
C	7.06271800	-4.82938300	0.33356800
H	7.74216700	-4.90107000	-0.51964800
H	7.67167600	-4.62675600	1.22019800
H	6.57661700	-5.79680500	0.47238600

H	1.50894400	0.89834100	1.63611200
H	1.50702100	2.64925600	1.84630700
H	-0.48054100	0.46905300	1.22841700
O	-0.29409700	-0.18200200	2.29459500
C	-1.18825700	-1.11353600	2.42230900
C	-2.53751100	-0.89404600	2.02983000
C	-0.85102500	-2.39654500	2.93476500
C	-3.45711400	-1.92184500	2.00858900
H	-2.81708500	0.11399700	1.74301600
C	-1.77650600	-3.41845300	2.94870000
H	0.16645200	-2.55983700	3.27229300
C	-3.06295500	-3.19008400	2.44238300
H	-4.48151100	-1.76283300	1.68442500
H	-1.51704100	-4.40821600	3.30623700
N	-4.00088800	-4.28053600	2.36043300
O	-3.72674700	-5.33316300	2.91946300
O	-5.03460500	-4.11114000	1.71650400

M4(S)

0 1

C	1.28574300	-1.89931800	-0.10793400
C	0.66764900	-3.36928000	1.36287100
C	-1.15021200	-2.70737600	-0.21432700
H	-1.48273600	-1.69195900	0.00908000
N	1.88985400	-3.06486900	1.69004000
N	2.26151900	-2.15247700	0.76081000
N	0.26936600	-2.72156500	0.22316200
C	-0.27179000	-4.23436300	2.14345600
H	-0.00457000	-4.17916900	3.19867200
H	-0.19959200	-5.27812500	1.80598800
C	-1.92859300	-3.73676100	0.61788500
O	-1.57730500	-3.73816400	1.98266300
C	3.61529800	-1.66083800	0.74319800
C	4.47403500	-2.15799900	-0.24108000
C	3.99521000	-0.71954200	1.70353500
C	5.76977400	-1.64600700	-0.26890100
C	5.30441900	-0.24650400	1.63078100
C	6.19665400	-0.68528900	0.65039800

H	6.46244100	-2.01176100	-1.02216100
H	5.63263400	0.48987300	2.35961700
H	-2.98817400	-3.48616100	0.55208000
C	1.42366600	-0.89569300	-1.24940900
O	1.05218800	-1.21546300	-2.35511500
C	2.19394600	0.37023800	-0.96932800
C	1.80234100	1.50869000	-1.91987100
H	2.07282300	0.66958300	0.07427100
H	1.99896600	1.16232700	-2.94288100
C	2.72663800	2.68696500	-1.64309900
C	3.70484200	3.06902600	-2.56016700
C	2.61329200	3.38531500	-0.43542300
C	4.56042700	4.13492800	-2.28176600
H	3.79702100	2.52993700	-3.49975200
C	3.46935300	4.44560800	-0.15527900
H	1.84568800	3.08609200	0.27749900
C	4.44454700	4.82485700	-1.07860500
H	5.31503100	4.42521500	-3.00622900
H	3.37514900	4.98115100	0.78441500
H	5.10864800	5.65513900	-0.86020600
C	7.59021000	-0.11922600	0.57786100
H	8.27353600	-0.81182100	0.08254000
H	7.59136800	0.81576600	0.00893200
H	7.97815400	0.10130500	1.57464600
C	4.01914300	-3.19414200	-1.23712400
H	3.47435500	-4.00581100	-0.74605500
H	3.35841200	-2.76295200	-1.99727100
H	4.87900100	-3.62263900	-1.75319700
C	3.03388700	-0.20603100	2.74070200
H	2.42292600	-1.00854400	3.15829400
H	3.58423300	0.27942000	3.54844300
H	2.33763600	0.52524400	2.31378500
H	-1.78027900	-4.74183800	0.19176900
C	-1.34834700	-3.01556000	-1.70193300
H	-0.69740500	-2.37746400	-2.29809200
H	-1.07483300	-4.05714400	-1.90218500
C	-2.79359400	-2.75868400	-2.06383900
C	-3.65099700	-3.77838900	-2.47490500

C	-3.29297900	-1.45669300	-1.94930900
C	-4.98615500	-3.50121000	-2.77022500
H	-3.27356900	-4.79375500	-2.56500700
C	-4.62550300	-1.17886500	-2.23408300
H	-2.62245300	-0.66088800	-1.62800600
C	-5.47698800	-2.20412400	-2.64733000
H	-5.64337900	-4.30264900	-3.09226000
H	-4.99305600	-0.16153200	-2.13157000
H	-6.51725600	-1.99225600	-2.87196600
C	-0.62879100	1.54874800	-1.04964900
C	0.36811200	1.96620000	-1.84368400
C	-1.93836900	2.28704900	-1.10866000
C	-2.20739900	3.29773400	-0.17945300
C	-2.90783700	1.97937200	-2.06662100
C	-3.43863500	3.94628900	-0.17148200
H	-1.44636400	3.55314100	0.55304900
C	-4.13746300	2.63786000	-2.06999200
H	-2.69413600	1.22567800	-2.81957300
C	-4.41095200	3.61249500	-1.11332600
H	-3.63814700	4.71492200	0.56903700
H	-4.87832100	2.39233600	-2.82549000
H	-5.37104600	4.11904300	-1.10966500
N	-0.55378300	0.40850600	-0.20805300
S	-0.51871800	0.64615700	1.36019000
O	-0.19456800	-0.64893600	1.99867500
O	0.33894400	1.78496400	1.76648600
C	-2.16466800	1.07766600	1.93061200
C	-3.27721000	0.49588700	1.32706200
C	-2.31852800	2.02051500	2.93925800
C	-4.54975500	0.88174200	1.72498100
H	-3.14875700	-0.21997700	0.52038700
C	-3.60069100	2.39659900	3.33247900
H	-1.43951300	2.47490300	3.38393600
C	-4.73039600	1.84574400	2.72414900
H	-5.41884700	0.44302800	1.24135900
H	-3.72530800	3.14162600	4.11354300
C	-6.11623900	2.30047700	3.10090000
H	-6.80406100	1.45424400	3.17415500

H	-6.11531700	2.82950600	4.05608700
H	-6.51438800	2.98081500	2.34065300
H	0.14044800	2.79304000	-2.51398900
H	3.25718200	0.14959100	-1.13304000

M4(R)

0 1

C	0.66340400	-2.00430000	-0.05817000
C	-0.17947000	-3.55258600	-1.32120100
C	-1.68368900	-2.84750900	0.56147100
H	-1.39044100	-3.14174900	1.57121900
N	0.99804400	-3.30835800	-1.82625000
N	1.50925000	-2.34024600	-1.02987900
N	-0.42242800	-2.78343500	-0.22194300
C	-1.15432300	-4.60604100	-1.74608700
H	-0.61002100	-5.46645400	-2.13653800
H	-1.82057300	-4.21358800	-2.52629200
C	-2.59418100	-3.91129000	-0.07877700
O	-1.88082500	-5.00993300	-0.60763500
C	2.89620400	-1.97078600	-1.17992900
C	3.24981300	-1.00186000	-2.11676700
C	3.81818600	-2.62968400	-0.35809200
C	4.60415700	-0.66888000	-2.19277600
C	5.15250200	-2.25441100	-0.47171500
C	5.56024400	-1.26726900	-1.37430400
H	4.91261100	0.08782700	-2.91004000
H	5.89199800	-2.74382000	0.15670300
H	-3.27227300	-4.29264200	0.68588700
C	0.90331000	-1.08014700	1.11404700
O	0.16712500	-1.22663400	2.06629200
C	2.10382500	-0.17312700	1.13269800
C	1.85438900	1.11136400	1.94464800
H	2.90906500	-0.74989300	1.61037000
H	1.27095500	0.83151500	2.83263100
C	3.20447400	1.61862500	2.43066600
C	3.59999000	1.43757700	3.75624000
C	4.09658000	2.22207800	1.53876200
C	4.86040600	1.84940300	4.18648900

H	2.91296100	0.97141400	4.45822200
C	5.35813300	2.62896400	1.96278000
H	3.78775300	2.37794300	0.50749600
C	5.74386600	2.44432100	3.29003300
H	5.14990900	1.70676200	5.22309500
H	6.04043600	3.09472600	1.25819400
H	6.72518700	2.76669200	3.62315500
C	7.00366800	-0.84323200	-1.44008200
H	7.25206200	-0.43459600	-2.42169900
H	7.20282800	-0.06614000	-0.69476900
H	7.67208700	-1.68124800	-1.23049900
C	2.23081900	-0.28979000	-2.96055900
H	1.30987400	-0.86351000	-3.07743600
H	1.94742700	0.65707400	-2.48883200
H	2.64462400	-0.06706500	-3.94644200
C	3.37885200	-3.69018200	0.61813000
H	2.75699200	-4.44693400	0.13040100
H	4.24610700	-4.18760700	1.05351400
H	2.79477500	-3.26515800	1.44298000
H	-3.19119100	-3.44301000	-0.87497200
C	-2.40944900	-1.49981200	0.57754800
H	-1.69019800	-0.70685400	0.78565700
H	-2.80670600	-1.30793700	-0.42181200
C	-3.51361700	-1.48040400	1.60553100
C	-4.85527800	-1.43124500	1.22117900
C	-3.19954500	-1.49218600	2.96829700
C	-5.86641400	-1.39897100	2.17978700
H	-5.10523100	-1.39708500	0.16303900
C	-4.20750500	-1.46000800	3.92740300
H	-2.15451300	-1.50975300	3.27011100
C	-5.54441100	-1.41475000	3.53499500
H	-6.90540100	-1.36021700	1.86698300
H	-3.95042200	-1.46373100	4.98202900
H	-6.33106100	-1.38702900	4.28229300
C	0.37706800	2.08927600	0.04979000
C	1.13998300	2.18912600	1.16283300
C	-0.10077300	3.40217700	-0.51411500
C	-0.96087400	4.20847300	0.23797200

C	0.37243400	3.88670100	-1.73873300
C	-1.35665500	5.46081900	-0.22568200
H	-1.32017500	3.84520000	1.19662600
C	-0.01734400	5.13973600	-2.20084700
H	1.04040200	3.27125200	-2.33060100
C	-0.88682400	5.92897200	-1.44941300
H	-2.02942800	6.06916300	0.37078400
H	0.36152700	5.50340700	-3.15100400
H	-1.19329000	6.90398500	-1.81519500
N	0.05841100	0.84948200	-0.51824300
S	-0.88495300	0.67812000	-1.78376000
O	-0.53799400	1.46166700	-2.98419100
O	-0.98996400	-0.77781200	-2.04099000
C	-2.54512300	1.20397300	-1.33389600
C	-3.52583300	1.18028600	-2.32118600
C	-2.87859100	1.52026800	-0.01957000
C	-4.84855200	1.45208100	-1.98233200
H	-3.25530300	0.94485800	-3.34618000
C	-4.20145400	1.79479200	0.30586400
H	-2.11061600	1.52909300	0.74873700
C	-5.20637200	1.75339800	-0.66550100
H	-5.61596700	1.42334600	-2.75111500
H	-4.46308400	2.02305700	1.33608600
C	-6.64030200	2.00543200	-0.27981600
H	-7.30020100	1.96637900	-1.14876000
H	-6.97879700	1.25479400	0.44216100
H	-6.75428000	2.98510500	0.19306900
H	1.30734000	3.19506600	1.53703400
H	2.42748600	0.08080000	0.12458800

NHCa

0 1

C	-1.25256100	0.55771300	1.10578300
C	-2.42902000	-0.38275300	-0.58566100
C	-3.86236300	0.47611200	1.07369300
H	-4.10265400	-0.25614000	1.85015200
N	-1.19959000	-0.56397200	-0.94851400
N	-0.50567100	0.02161200	0.10638900

N	-2.49392600	0.27629100	0.61267100
C	-3.79840800	-0.70508400	-1.09329400
H	-4.02739700	-1.75808000	-0.90045800
H	-3.90924400	-0.52607800	-2.16296100
C	-4.67073700	0.23677100	-0.22288200
C	0.92360300	0.02191900	0.05716000
C	1.59906600	1.23174600	-0.11710000
C	1.59984000	-1.19694000	0.18447400
C	2.99422200	1.19978100	-0.15794100
C	2.99120600	-1.18065700	0.13036000
C	3.70533600	0.00739300	-0.04178000
H	3.53579600	2.13356900	-0.28981600
H	3.53252200	-2.11866900	0.23408900
C	0.83784500	-2.47909900	0.39077600
H	0.27335700	-2.74560500	-0.50605100
H	0.11384800	-2.36837700	1.20434100
H	1.51877600	-3.29531700	0.63716500
C	5.21306100	-0.01302700	-0.11802800
H	5.54343000	-0.40571700	-1.08401800
H	5.63408000	-0.65300000	0.66074500
H	5.62503900	0.99126400	-0.00307900
C	0.84475500	2.52742800	-0.25343100
H	0.29955200	2.75513900	0.66596600
H	0.10402800	2.46155700	-1.05648600
H	1.52822600	3.34868200	-0.47680900
H	-5.65913600	-0.17574500	-0.01910200
H	-3.99031900	1.47829100	1.48306100
H	-4.80066800	1.18888700	-0.74332700

1a

0 1

C	3.40996700	0.38321300	0.00000200
O	3.84671500	-0.74969300	-0.00000400
H	4.10228500	1.24738200	-0.00000400
C	1.97301700	0.72072700	0.00000100
C	1.05322800	-0.25737100	0.00000300
H	1.71182300	1.77477800	-0.00000400
H	1.43374000	-1.27883600	0.00000600

C	-0.40423300	-0.11161900	0.00000200
C	-1.04207900	1.13790300	0.00000100
C	-1.19403300	-1.26887100	0.00000100
C	-2.42708500	1.22209000	-0.00000100
H	-0.45219800	2.04884200	0.00000200
C	-2.58213600	-1.18520900	-0.00000100
H	-0.70779300	-2.24055000	0.00000200
C	-3.20171400	0.06103200	-0.00000200
H	-2.90782600	2.19491800	-0.00000100
H	-3.17874400	-2.09136300	-0.00000100
H	-4.28459300	0.13100200	-0.00000300

2a

0 1

C	0.30944300	-0.67178700	0.00013200
C	0.77011100	0.63741100	0.00022800
C	2.13459900	0.89547400	0.00007500
C	3.00808900	-0.19365200	-0.00005200
C	2.52590900	-1.50302400	-0.00007500
C	1.15257400	-1.76508100	-0.00000600
C	-0.37225400	1.59803100	0.00043600
H	2.51367400	1.91253300	0.00012400
H	4.07860000	-0.01993700	-0.00012500
H	3.22603600	-2.33139800	-0.00020300
H	0.76979000	-2.77965300	-0.00009300
S	-1.47341400	-0.60425600	-0.00003800
O	-2.01135600	-1.09735400	1.25554200
O	-2.01114600	-1.09701400	-1.25583100
N	-1.55663100	1.10122300	0.00019000
C	-0.14603100	3.07241900	-0.00023400
H	0.43334700	3.35783600	0.88303700
H	0.43357300	3.35709600	-0.88360100
H	-1.09860000	3.59925300	-0.00058000

2a$^-$

-1 1

C	0.28963300	-0.59657000	0.00177100
C	0.79432300	0.69552400	0.00053900

C	2.17628400	0.88212900	-0.00100800
C	3.00712500	-0.23583800	-0.00085500
C	2.47663500	-1.53038900	0.00087800
C	1.09705700	-1.72310100	0.00193300
C	-0.32013000	1.70001200	0.00183900
H	2.60278500	1.88060200	-0.00237700
H	4.08470700	-0.10096000	-0.00201900
H	3.14408900	-2.38651100	0.00120200
H	0.66436000	-2.71900000	0.00296600
S	-1.49424700	-0.47243900	-0.00036700
O	-2.02193600	-1.09500100	1.22868000
O	-2.01523600	-1.08577500	-1.23696600
N	-1.57365200	1.12465100	0.00585300
C	-0.07466400	3.02709500	0.00025300
H	0.93900400	3.40777500	-0.00170500
H	-0.89163300	3.73759500	0.00101300

2a^{-} ^{i}Pr_2NEtH+

0 1

C	2.57467100	-0.60780800	0.05503200
C	2.60833300	0.76129300	-0.16308700
C	3.83092400	1.42570600	-0.07030300
C	4.97142700	0.69156200	0.24152800
C	4.91028300	-0.68956100	0.45772200
C	3.69638200	-1.36316500	0.36035900
C	1.25014700	1.30394100	-0.46755200
H	3.89849300	2.49599600	-0.23606000
H	5.92732600	1.19969100	0.31892400
H	5.81483200	-1.23733900	0.69950900
H	3.62565900	-2.43446600	0.51709200
S	0.90402600	-1.15654100	-0.18543400
O	0.35151400	-1.76879500	1.03852900
O	0.84027500	-2.02539300	-1.37008800
N	0.26793000	0.31520300	-0.43287500
C	1.04223500	2.60992000	-0.71205300
H	1.86826700	3.30889600	-0.69876500
H	0.05756100	3.00405200	-0.92734800
N	-2.42437900	0.31097300	0.04238900

C	-3.20444300	1.02130000	-1.03059200
C	-2.34574900	1.36527500	-2.23768600
H	-3.63587400	1.92286300	-0.59369600
H	-4.04009800	0.38283300	-1.31943100
H	-2.96603500	1.86008900	-2.98844900
H	-1.52622900	2.03395000	-1.96673900
H	-1.90438100	0.47158100	-2.68248700
C	-2.53739900	1.01518200	1.38007100
C	-1.84536800	2.37270800	1.28824100
C	-1.90327800	0.19853500	2.50130400
H	-3.61044300	1.14930900	1.56074200
H	-2.19413400	2.98117600	0.44979700
H	-2.04349700	2.93158900	2.20551700
H	-0.76446900	2.23801300	1.18999500
H	-2.46521100	-0.70451900	2.74549100
H	-0.88204400	-0.09115900	2.23563000
H	-1.87009300	0.82131500	3.39834900
C	-2.73877200	-1.17268000	0.10145700
C	-2.49526600	-1.82333900	-1.25557900
C	-4.14774000	-1.44170000	0.61567000
H	-1.99836100	-1.57657400	0.79526300
H	-2.57891600	-2.90530900	-1.12878000
H	-3.23711200	-1.51920700	-2.00061300
H	-1.48984500	-1.61896900	-1.63128000
H	-4.33258700	-1.00598900	1.59987000
H	-4.91412300	-1.07704300	-0.07468800
H	-4.27706300	-2.52300600	0.70311500
H	-1.36353200	0.36415100	-0.20535300

^{i}Pr$_2$NEt

0 1

N	-0.01785200	0.02874900	-0.64121200
C	0.68547800	1.28332900	-0.85352500
C	1.27588700	2.01539800	0.36221900
H	-0.02297500	1.95329100	-1.35555100
H	1.49903400	1.12001300	-1.57256600
H	1.63074900	3.00536200	0.05795000
H	0.54177000	2.14531800	1.16083200

H	2.13117000	1.47706300	0.77908200
C	-1.46577300	0.15148000	-0.46747000
C	-1.93420400	0.99308200	0.73263200
C	-2.16004200	-1.20900100	-0.46879200
H	-1.82026000	0.68044800	-1.36550000
H	-1.57244400	2.02199100	0.65744600
H	-3.02830500	1.03086600	0.75770200
H	-1.58718400	0.57769100	1.68301300
H	-1.84456400	-1.80857900	-1.32682500
H	-1.95777500	-1.77530600	0.44610300
H	-3.24138700	-1.06190300	-0.53123600
C	0.69422700	-1.07212600	0.00350600
C	0.73135000	-1.05989200	1.54247400
C	2.10742700	-1.20620100	-0.56704500
H	0.16709400	-1.98967900	-0.28507300
H	-0.27473400	-1.12425200	1.96509300
H	1.29664600	-1.92430000	1.90755900
H	1.20544700	-0.15600100	1.93294100
H	2.07936000	-1.22492100	-1.65974300
H	2.76003300	-0.38639700	-0.25145000
H	2.55718300	-2.13836400	-0.21528500

$^{i}Pr_2NEt·H^+$

1 1

N	-0.09775300	0.24269700	0.19389300
C	0.51466100	1.37415700	-0.60428200
C	1.83275200	1.90951100	-0.07285700
H	-0.23322000	2.16631000	-0.60300500
H	0.60579600	1.01016300	-1.62849100
H	2.00467700	2.88345700	-0.53493400
H	1.80763800	2.05816400	1.01007100
H	2.67984600	1.27352500	-0.32637900
C	-1.56187000	0.07211500	-0.26815500
C	-2.40988900	1.13701100	0.42277800
C	-2.13744500	-1.31114800	-0.00118400
H	-1.52876000	0.24952500	-1.34684600
H	-2.02431100	2.15121100	0.30597800
H	-3.41279900	1.11932900	-0.00692200

H	-2.50275800	0.91503000	1.49059800
H	-1.65081200	-2.10109600	-0.57486200
H	-2.11126200	-1.56309700	1.06296400
H	-3.18588800	-1.28677800	-0.30544300
C	0.72728000	-1.04635600	0.22585300
C	1.88571800	-0.95905800	1.21402400
C	1.15061700	-1.45828000	-1.17387700
H	0.03488000	-1.78433300	0.62645000
H	1.57239900	-0.53632900	2.17338100
H	2.22766700	-1.97814300	1.40760900
H	2.73517000	-0.39238800	0.83746200
H	0.31083700	-1.46670600	-1.87525100
H	1.93643300	-0.80914800	-1.56859300
H	1.55447000	-2.47150800	-1.12541900
H	-0.14668800	0.55622400	1.17058000

DQ (toluene)

0 1

C	2.80665300	1.28429000	0.01001600
C	1.45951900	1.23582800	-0.06348900
C	0.69361100	0.00047500	-0.09145500
C	1.46139500	-1.23369800	-0.10829700
C	2.80859800	-1.28003700	-0.03314200
C	3.56785600	0.00274200	0.08161100
H	0.91905700	2.16850700	-0.09498300
H	0.92234700	-2.16725300	-0.18700900
C	-0.69357500	-0.00056700	-0.09125600
C	-1.45948000	-1.23591800	-0.06278100
C	-1.46135200	1.23361000	-0.10817700
C	-2.80660800	-1.28436200	0.01083600
H	-0.91901700	-2.16861800	-0.09394800
C	-2.80855000	1.27996900	-0.03293700
H	-0.92230200	2.16715500	-0.18699000
C	-3.56781400	-0.00278600	0.08206200
O	4.78481800	-0.00152500	0.22926900
O	-4.78476100	0.00152700	0.22984600
C	3.58729500	-2.59513600	-0.05758700
C	4.58115000	-2.59545200	-1.23541300

C	4.34624500	-2.77828800	1.27098800
C	2.65465300	-3.80194000	-0.23202400
H	4.05149100	-2.46524000	-2.18560300
H	5.31919200	-1.80014800	-1.12960700
H	5.10533300	-3.55794100	-1.26852100
H	3.65034800	-2.79211900	2.11177300
H	4.87726200	-3.73491300	1.25725100
H	5.07544000	-1.98116600	1.42807700
H	3.25418300	-4.71108200	-0.25996600
H	1.94848400	-3.89165100	0.59797000
H	2.09412700	-3.74608700	-1.17127900
C	3.58324200	2.60064900	0.04459600
C	4.33674700	2.73002700	1.37945100
C	4.57700000	2.64974600	-1.12929200
C	2.64861500	3.80577600	-0.08392300
H	3.63966200	2.69539100	2.22424000
H	5.07243400	1.93419600	1.49953900
H	4.85581000	3.69550800	1.41058000
H	4.05148700	2.56347300	-2.08737100
H	5.10470700	3.61074100	-1.11920500
H	5.31643200	1.84940000	-1.05812400
H	3.24263700	4.72631400	-0.07602300
H	2.08185100	3.78592800	-1.02321400
H	1.93821500	3.86765500	0.74713400
C	-3.58724700	2.59507000	-0.05755900
C	-4.34738900	2.77844300	1.27098900
C	-2.65461400	3.80185600	-0.23221000
C	-4.58110700	2.59517800	-1.23538800
H	-5.07536400	1.98132700	1.42423100
H	-3.65028500	2.78844000	2.11577000
H	-4.87723500	3.73505200	1.25708200
H	-2.09411800	3.74585700	-1.17147700
H	-3.25415000	4.71499400	-0.25627600
H	-1.94841800	3.89170100	0.59774900
H	-5.10529500	3.55766200	-1.26866200
H	-4.05144800	2.46880700	-2.18555900
H	-5.31914500	1.80388600	-1.13344400
C	-3.58318100	-2.60072900	0.04590300

C	-4.33661800	-2.72967500	1.38084800
C	-4.57698700	-2.65024600	-1.12793600
C	-2.64853300	-3.80589100	-0.08104400
H	-3.63949800	-2.69468100	2.22560400
H	-5.07235900	-1.93384900	1.50068200
H	-4.85561200	-3.69519100	1.41235700
H	-4.05150900	-2.56430600	-2.08607400
H	-5.10348800	-3.61124700	-1.11748700
H	-5.31641500	-1.84986800	-1.06101600
H	-3.24253700	-4.72644200	-0.07402300
H	-2.08179600	-3.78634200	-1.02036300
H	-1.93811000	-3.86747600	0.75282100

[DQH]⁻ (toluene)

-1 1

C	-2.87967600	-1.19275600	0.31871900
C	-1.49504800	-1.15390800	0.31926900
C	-0.75771200	0.00412400	0.02566200
C	-1.48987500	1.16290100	-0.27760500
C	-2.87431200	1.20221100	-0.29772200
C	-3.64140800	0.00457900	0.00342400
H	-0.94102200	-2.04851600	0.58989000
H	-0.93196600	2.05666600	-0.54278700
C	0.71595300	0.00045800	0.02736100
C	1.45226700	1.15363500	0.33006900
C	1.44295600	-1.15579100	-0.28701000
C	2.84737300	1.18366100	0.32962500
H	0.89917800	2.04546400	0.59587200
C	2.83737600	-1.19952500	-0.28262500
H	0.88147600	-2.03696400	-0.56952100
C	3.52492400	-0.01569700	0.04340100
O	-4.91573600	0.00404500	-0.00952500
O	4.92263000	-0.01577000	0.03164400
C	-3.62907200	2.48950800	-0.64894200
C	-4.49578500	2.26462800	-1.90062600
C	-4.53048000	2.90177100	0.52918500
C	-2.68671200	3.66289600	-0.94221800
H	-3.86122800	2.02378300	-2.76112300

H	-5.18487300	1.43813000	-1.72704200
H	-5.06494500	3.17244300	-2.13999000
H	-3.91954600	3.12754300	1.41082700
H	-5.10518000	3.80158600	0.27317800
H	-5.21547800	2.08847800	0.76964100
H	-3.27919700	4.55499900	-1.17507400
H	-2.05055600	3.89794500	-0.08165600
H	-2.03804000	3.45850700	-1.80098500
C	-3.64050100	-2.48165800	0.65059700
C	-4.53956500	-2.26127800	1.87994300
C	-4.51118900	-2.89331400	-0.55072700
C	-2.70324400	-3.65358600	0.96572700
H	-3.92822200	-2.01320700	2.75505400
H	-5.23131900	-1.44112200	1.68763000
H	-5.10646800	-3.17360000	2.10732900
H	-3.87798000	-3.11570600	-1.41731100
H	-5.09042000	-3.79474900	-0.31111900
H	-5.19135800	-2.08053000	-0.80626000
H	-3.29944500	-4.54686600	1.18428000
H	-2.04644600	-3.88736000	0.12049100
H	-2.07545200	-3.44802800	1.83970100
C	3.58471800	-2.48929200	-0.66591200
C	4.49619300	-2.96632500	0.48045200
C	2.61252600	-3.63883700	-0.96803500
C	4.42383800	-2.25093900	-1.93336100
H	5.34885900	-2.30199500	0.62812200
H	3.93583900	-3.03948000	1.41804000
H	4.89666800	-3.95921900	0.24727900
H	1.95171400	-3.40033600	-1.80623100
H	3.18719000	-4.53013900	-1.23888000
H	1.99285500	-3.88727700	-0.10056300
H	4.94779900	-3.17265500	-2.21291600
H	3.77616700	-1.96179900	-2.76691100
H	5.16065200	-1.46317700	-1.77554900
C	3.60730500	2.48847900	0.62588900
C	4.50457100	2.34035200	1.86782000
C	4.46316300	2.87876200	-0.59281700
C	2.64848600	3.65442900	0.90506900

H	3.92277400	1.98858700	2.72582700
H	5.33004900	1.64930100	1.69356200
H	4.93950100	3.31170600	2.12929000
H	3.82458600	3.03500500	-1.46812600
H	4.99627000	3.81502300	-0.38929900
H	5.19280500	2.10302400	-0.82677200
H	3.23368500	4.56301900	1.07917700
H	1.98087000	3.84282200	0.05905400
H	2.03592300	3.47463600	1.79407300
H	5.22671200	-0.28905500	0.90515800

DQH_2 (toluene)

0 1

C	2.84797400	-1.18444700	-0.38920100
C	1.45554000	-1.14093000	-0.37539300
C	0.74191900	-0.00288900	0.00483400
C	1.46303000	1.12833600	0.38240300
C	2.85846800	1.16074300	0.38592100
C	3.53446500	-0.01321100	-0.00658400
H	0.89185400	-2.01004200	-0.69253500
H	0.90675800	2.00049600	0.70380400
C	-0.74192700	0.00270500	0.00480600
C	-1.45547500	1.14077600	-0.37546700
C	-1.46309500	-1.12847700	0.38237600
C	-2.84791000	1.18438000	-0.38929900
H	-0.89172800	2.00982400	-0.69266800
C	-2.85853600	-1.16080000	0.38588800
H	-0.90688100	-2.00067600	0.70377600
C	-3.53445300	0.01318200	-0.00666300
O	4.90246900	-0.07889300	-0.03662400
O	-4.90246900	0.07894400	-0.03668200
C	3.61640100	2.43083000	0.81431600
C	4.48044600	2.15808600	2.06471700
C	4.46270300	2.98157300	-0.35448200
C	2.65287800	3.56120300	1.20553500
H	3.83632700	1.86873200	2.89978400
H	5.21773900	1.35939600	1.94856300
H	5.02095800	3.06687300	2.34771400

H	3.80589500	3.26532900	-1.18160500
H	5.00985800	3.87241000	-0.03011500
H	5.19309900	2.27939600	-0.76603700
H	3.23439000	4.44058500	1.49753000
H	2.00725000	3.84850600	0.37056600
H	2.02262300	3.27949000	2.05380900
C	3.60026200	-2.45476800	-0.81725300
C	4.45998200	-2.17602000	-2.06379100
C	4.48407100	-2.96731300	0.33506000
C	2.62948900	-3.58712700	-1.18093300
H	3.83022400	-1.83109200	-2.89019700
H	5.22281100	-1.42258900	-1.86969100
H	4.95731800	-3.09965800	-2.37978600
H	3.86978400	-3.19484000	1.21225600
H	4.98934300	-3.88941600	0.02732100
H	5.24112100	-2.23716200	0.62011200
H	3.20706100	-4.47049900	-1.46881500
H	1.99303500	-3.86570200	-0.33485700
H	1.98788800	-3.31878700	-2.02607800
C	-3.61656000	-2.43082500	0.81429700
C	-4.46304300	-2.98142200	-0.35446100
C	-2.65313600	-3.56136200	1.20533300
C	-4.48039600	-2.15798200	2.06480700
H	-5.19319300	-2.27904800	-0.76610500
H	-3.80627800	-3.26541300	-1.18153400
H	-5.01042700	-3.87208600	-0.03001200
H	-2.02290600	-3.27987200	2.05369900
H	-3.23474400	-4.44074800	1.49713300
H	-2.00749400	-3.84853400	0.37033400
H	-5.02107000	-3.06662900	2.34791100
H	-3.83612900	-1.86876100	2.89982400
H	-5.21748900	-1.35908100	1.94873900
C	-3.60012600	2.45478400	-0.81727300
C	-4.45998400	2.17622900	-2.06375700
C	-4.48370900	2.96740200	0.33516800
C	-2.62930200	3.58707600	-1.18103900
H	-3.83032100	1.83125900	-2.89022100
H	-5.22291800	1.42290300	-1.86969000

H	-4.95717700	3.09997200	-2.37966500
H	-3.86925300	3.19487600	1.21226400
H	-4.98897300	3.88954600	0.02753400
H	-5.24076800	2.23732400	0.62038500
H	-3.20683500	4.47050400	-1.46883200
H	-1.99273500	3.86559700	-0.33503100
H	-1.98781900	3.31868400	-2.02625600
H	-5.27769200	-0.76655400	0.22203200
H	5.27764700	0.76668600	0.22189300

M2a

0 1

C	-1.85014900	-0.68270900	0.01232700
C	-3.90226700	0.18744800	-0.00526600
C	-4.03129700	-2.17668700	0.02808300
H	-3.66913900	-2.93175900	-0.66785300
N	-3.15044000	1.23397600	0.00550800
N	-1.85904400	0.70361000	0.02424000
N	-3.19524100	-0.98433600	-0.01444300
C	-5.37054400	-0.09697700	0.03582700
H	-5.94354100	0.54500000	-0.63340500
H	-5.74274800	0.05373700	1.05390400
C	-5.40750800	-1.59208000	-0.36909300
C	-0.73123000	1.56729700	0.08367900
C	-0.06763800	1.73216400	1.30575400
C	-0.28153100	2.16852900	-1.09456700
C	1.09379900	2.50043000	1.31799700
C	0.87975000	2.93933500	-1.03541300
C	1.58326000	3.10678900	0.15774500
H	1.63437300	2.62442300	2.25369600
H	1.25222400	3.40328700	-1.94557200
H	-5.52774700	-1.66955500	-1.45246100
C	-0.81092900	-1.58461600	0.04143500
O	-1.19093000	-2.92245300	0.17496400
C	0.57755900	-1.26766900	-0.04336100
C	1.59033400	-2.16107000	0.06804200
H	0.81993000	-0.23156600	-0.25498000
H	1.35670400	-3.20245800	0.28010600

C	3.01029000	-1.83341600	-0.05336400
C	3.95961000	-2.86877300	-0.03845900
C	3.49174500	-0.51600800	-0.17680400
C	5.32089900	-2.60957000	-0.15226400
H	3.61306200	-3.89429300	0.06287000
C	4.85106700	-0.25851200	-0.29338300
H	2.79798700	0.32096200	-0.16867100
C	5.77812900	-1.30096200	-0.28430600
H	6.02716700	-3.43448300	-0.13809600
H	5.19241800	0.76864300	-0.38742700
H	6.83961800	-1.09439400	-0.37425100
C	2.84678900	3.92701600	0.20552900
H	3.20542200	4.16090000	-0.79904000
H	2.67884300	4.87194300	0.73184700
H	3.63874500	3.39253700	0.73774600
C	-1.01815800	1.93599100	-2.38568800
H	-1.04032100	0.86740000	-2.62520700
H	-2.05632300	2.27072400	-2.30716600
H	-0.53679100	2.46525700	-3.20983100
C	-0.57632700	1.04565800	2.54306900
H	-1.64959400	1.21485300	2.66803700
H	-0.42000200	-0.03673600	2.46981000
H	-0.05593400	1.40961100	3.43067100
H	-0.81257400	-3.40150800	-0.57550700
H	-4.02641000	-2.60342500	1.03595900
H	-6.22876600	-2.13188100	0.10286000

TS3(RS)a

0 1

C	2.21522100	-0.71329300	-0.75068000
C	4.15261500	-1.65711200	-0.30423100
C	2.71271800	-3.22812100	-1.35180900
H	1.74110300	-3.59348800	-1.01996000
N	4.27836800	-0.45907700	0.17556100
N	3.06549300	0.12927000	-0.11437600
N	2.92710700	-1.85657400	-0.87322300
C	4.98545700	-2.89289000	-0.40632500
H	5.55483400	-3.08657100	0.50248400

H	5.68873500	-2.79325800	-1.23868900
C	3.90143800	-3.96527100	-0.68899200
C	2.76849400	1.45092000	0.34414000
C	2.89219800	2.51242200	-0.55138100
C	2.26443300	1.59458400	1.64147500
C	2.47876700	3.77153100	-0.11513300
C	1.86538200	2.87113500	2.03126400
C	1.95926000	3.96590300	1.16550700
H	2.55169900	4.61484600	-0.79698800
H	1.45648000	3.01221800	3.02921300
H	3.56683400	-4.39753200	0.25642800
C	0.86773400	-0.54691000	-1.20276000
O	0.42387300	-1.57820900	-1.99060000
C	0.07651500	0.52750000	-0.88570200
C	-1.32629100	0.50066100	-1.01894300
H	0.53164000	1.37203400	-0.38095000
H	-1.72309700	-0.33411600	-1.59401800
C	-2.11862100	1.73556700	-1.10902000
C	-3.38689200	1.69633700	-1.71077500
C	-1.67368800	2.97472600	-0.61056500
C	-4.18542500	2.83287500	-1.79438600
H	-3.74307600	0.75376600	-2.11824300
C	-2.47227100	4.10835600	-0.69297900
H	-0.69207600	3.05768000	-0.14879100
C	-3.73551400	4.04658600	-1.28158400
H	-5.16060900	2.76887500	-2.26789300
H	-2.10294200	5.05049800	-0.29816000
H	-4.35466600	4.93535900	-1.34604800
C	1.50317700	5.32909300	1.61718100
H	1.46315700	6.03060000	0.78159000
H	0.50968000	5.27619700	2.07129400
H	2.18568100	5.73670300	2.36919200
C	2.09954800	0.39374000	2.53217900
H	1.36056500	-0.30109300	2.11034600
H	3.04567900	-0.14476800	2.63993800
H	1.75505200	0.69586900	3.52291600
C	3.38573400	2.27680700	-1.95268900
H	4.32306300	1.71366900	-1.95047700

H	2.65011100	1.69555000	-2.52036100
H	3.54635600	3.22326000	-2.47080900
H	-0.06373400	-2.24018000	-1.43802900
H	2.75043000	-3.23763700	-2.44273600
H	4.27248100	-4.77311400	-1.31930500
C	-2.94614500	-2.34118900	0.48663700
C	-3.00471800	-0.96272500	0.66344900
C	-4.23550300	-0.31791600	0.55539500
C	-5.35857700	-1.08043100	0.23298800
C	-5.26921500	-2.45965200	0.02972800
C	-4.04549000	-3.11639700	0.16488800
C	-1.65464900	-0.40604300	0.98122000
H	-4.32188200	0.75235200	0.70915400
H	-6.32341900	-0.59178000	0.14181800
H	-6.16008800	-3.02637400	-0.21856900
H	-3.96087500	-4.19087800	0.03964200
S	-1.27119500	-2.83256200	0.82213600
O	-0.65309000	-3.37256900	-0.42154700
O	-1.19293900	-3.75320800	1.95326400
N	-0.71003200	-1.35521300	1.18763100
C	-1.54977200	0.82883700	1.83184000
H	-2.27598000	1.58973700	1.54404600
H	-1.73127400	0.54010200	2.87282500
H	-0.54557600	1.25772400	1.76724900

M3(RS)a

0 1

C	2.42279300	-1.33842300	0.00088400
C	4.45963500	-2.14341400	-0.11351300
C	2.89490100	-3.91969800	0.02551200
H	2.14579000	-4.13928900	0.78084400
N	4.62833900	-0.85467200	-0.15326500
N	3.34743300	-0.36280900	-0.07372000
N	3.14126300	-2.47106100	-0.00621100
C	5.30727900	-3.37232100	-0.17806700
H	6.20675400	-3.29283400	0.43214600
H	5.61023100	-3.55065100	-1.21430000
C	4.31453500	-4.45246100	0.32824900

C	3.15426700	1.06091600	-0.02347200
C	3.24088000	1.77659700	-1.22133400
C	2.88338400	1.65785200	1.20669100
C	3.03345700	3.15075700	-1.15992200
C	2.69128700	3.04052600	1.21613300
C	2.75334900	3.79723600	0.04711000
H	3.07956400	3.73119300	-2.07804500
H	2.47415100	3.53237200	2.16146800
H	4.42936200	-4.56958400	1.40821800
C	0.92917300	-1.34292100	0.11908300
O	0.49006500	-2.45483600	0.59505700
C	0.29676700	-0.20960800	-0.29791900
C	-1.18502600	0.08103300	-0.36863300
H	0.91582200	0.57956400	-0.70579000
H	-1.57826000	-0.30888700	-1.31919400
C	-1.40188200	1.58668600	-0.38554600
C	-2.35208300	2.15498700	-1.23874900
C	-0.67094000	2.43425600	0.45168700
C	-2.57580700	3.52874900	-1.24719000
H	-2.92983300	1.50650200	-1.89343400
C	-0.89347200	3.80912800	0.45117600
H	0.09266600	2.01142600	1.09975400
C	-1.84947200	4.36160600	-0.39801900
H	-3.31747700	3.94924800	-1.91913800
H	-0.31245000	4.44674400	1.11231200
H	-2.02369800	5.43309800	-0.40311000
C	2.49671900	5.28103200	0.06488300
H	2.41039100	5.65812500	1.08617900
H	3.30175100	5.82533100	-0.43626400
H	1.56498800	5.50996200	-0.46246200
C	2.73717900	0.83505600	2.45707400
H	1.78808200	0.28536100	2.43305700
H	3.54467600	0.10368000	2.55485000
H	2.73947000	1.47372100	3.34157400
C	3.50565700	1.06487400	-2.52030500
H	4.47481100	0.55818100	-2.50048700
H	2.73864300	0.30457400	-2.70451300
H	3.49603100	1.76849800	-3.35357000

H	-1.12875000	-2.49129100	0.41444100
H	2.51858500	-4.23072700	-0.95149500
H	4.48625900	-5.42207600	-0.13819300
C	-4.28845200	-1.15591700	-0.08837900
C	-3.51891800	-0.21785600	0.58406300
C	-4.13223900	0.94858700	1.03401100
C	-5.49121100	1.13458300	0.78827300
C	-6.24198900	0.17644600	0.10218100
C	-5.64080200	-0.99490500	-0.34975900
C	-2.06070200	-0.63128500	0.73633500
H	-3.55954800	1.70784600	1.55895800
H	-5.97554600	2.04029700	1.13890800
H	-7.29985600	0.34237800	-0.07148200
H	-6.20370500	-1.76326100	-0.86957900
S	-3.27136200	-2.53312500	-0.54838900
O	-2.86921800	-2.39500100	-1.95358300
O	-3.86389400	-3.81270800	-0.17014500
N	-2.09958800	-2.09173400	0.51992000
C	-1.53532500	-0.38579400	2.14758900
H	-1.42747800	0.68448200	2.34235100
H	-2.23038000	-0.81379700	2.87329800
H	-0.56466800	-0.87572400	2.25845100

4a(RS)

0 1

C	-0.80818800	-2.39187400	-0.43113400
O	-1.56327500	-3.26298900	-0.77280400
C	0.70001200	-2.31486100	-0.61908700
C	0.98892700	-0.80885600	-0.56514100
H	0.98396700	-2.78537900	-1.56085600
H	0.65661200	-0.38517400	-1.52061600
C	2.42088300	-0.38646600	-0.34316000
C	2.91805800	0.71852300	-1.04137800
C	3.27280100	-1.05172300	0.54396900
C	4.22402200	1.16043100	-0.84930400
H	2.27056400	1.23464700	-1.74619800
C	4.58007400	-0.61420000	0.73643500
H	2.92390100	-1.92215100	1.09171200

C	5.05916900	0.49455700	0.04321500
H	4.58971400	2.01864300	-1.40392900
H	5.22680600	-1.14530200	1.42734900
H	6.07961100	0.83200100	0.19206300
H	1.18077900	-2.86772600	0.19483000
C	-1.95219900	1.13024700	-0.02368500
C	-0.58850300	1.08509500	0.22785600
C	0.12996800	2.27859400	0.24751500
C	-0.53892300	3.47099200	-0.01982300
C	-1.91047600	3.48899400	-0.28744800
C	-2.64277200	2.30643900	-0.28372600
C	-0.04442300	-0.30199600	0.49680200
H	1.19438700	2.27698800	0.46121300
H	0.01556100	4.40346600	-0.01688900
H	-2.40899600	4.43015600	-0.49106100
H	-3.71030800	2.29773900	-0.47709700
S	-2.69675300	-0.47581700	0.08839800
O	-3.31962600	-0.87365400	-1.16043800
O	-3.44831800	-0.59883800	1.32498700
N	-1.17828300	-1.22119000	0.24688700
C	0.42882100	-0.42596300	1.94472000
H	-0.40028600	-0.19096000	2.61642400
H	0.76656300	-1.44369900	2.15871100
H	1.25340300	0.26440800	2.13806200

TS3a

0 1

C	-3.49566300	-0.71558500	-0.71351400
C	-5.33357800	-1.61737500	0.08370700
C	-5.74592400	0.64261300	-0.49375900
H	-5.28033400	1.56196600	-0.14238600
N	-4.46945400	-2.58686200	0.12489100
N	-3.32204100	-2.02039900	-0.38011100
N	-4.79311100	-0.47509700	-0.42589500
C	-6.77365600	-1.41855400	0.42701200
H	-7.03707100	-1.86842400	1.38412600
H	-7.39948600	-1.86963900	-0.34884700
C	-6.87431200	0.12845000	0.42913200

C	-2.19632200	-2.86881800	-0.66142300
C	-2.03896300	-3.33644800	-1.96990200
C	-1.29909800	-3.16435500	0.36562800
C	-0.91647100	-4.11477300	-2.24119700
C	-0.20181900	-3.96547400	0.04882700
C	0.01341200	-4.43224900	-1.24891000
H	-0.76263800	-4.47982800	-3.25346300
H	0.50714800	-4.22399400	0.83135500
H	-6.69896800	0.49938100	1.44200700
C	-2.59202600	0.28475800	-1.25339600
O	-3.19028400	1.40623500	-1.59930700
H	-3.22879500	2.13401600	-0.66788000
C	-1.24031200	-0.03978100	-1.41963400
C	-0.28347100	0.72385100	-2.05743300
H	-0.92531800	-0.98948600	-1.00903100
H	-0.58898000	1.65573700	-2.52668600
C	1.07814400	0.30391400	-2.24628000
C	1.61211600	-0.85854400	-1.64899900
C	1.94508500	1.09753500	-3.03013300
C	2.92818800	-1.23379500	-1.87504400
H	0.99650000	-1.48192200	-1.00631900
C	3.26004000	0.72460500	-3.24566000
H	1.55473600	2.00735000	-3.48101900
C	3.75862700	-0.44914300	-2.66982900
H	3.32281000	-2.12863600	-1.40337500
H	3.90549600	1.34646200	-3.85912300
H	4.78868500	-0.74640500	-2.83505300
C	4.44043500	0.98036100	0.25677400
C	3.17612300	1.49576900	0.24218700
C	2.04854400	0.83070100	0.81499200
C	2.29434100	-0.42735200	1.45744100
C	3.51456300	-1.03069000	1.48540100
C	4.65666800	-0.37303000	0.81101800
H	3.02499200	2.49123300	-0.15595300
H	1.44308600	-0.93100900	1.90285500
C	0.74971000	1.42081400	0.83792500
C	-0.24553800	1.02752200	1.77808200
C	0.40931900	2.50931900	-0.02092400

C	-1.52313500	1.53554100	1.76183900
H	0.04488300	0.35517000	2.57581300
C	-0.83873100	3.09770400	-0.03997200
H	1.17675900	2.88491300	-0.68590900
C	-1.87297400	2.46437200	0.70832700
O	5.76352600	-0.93399800	0.73553900
O	-3.12910100	2.70959900	0.40404700
C	3.73564800	-2.38700500	2.15674000
C	4.07210400	-3.44048500	1.08633200
C	4.87772200	-2.29871800	3.18657500
C	2.48357400	-2.86233000	2.90150500
H	3.21625500	-3.57553700	0.41224100
H	4.94222400	-3.13530900	0.50306300
H	4.28326800	-4.40663800	1.55931100
H	4.64287100	-1.54944800	3.95009300
H	4.99674300	-3.26639700	3.68679500
H	5.81735700	-2.02866000	2.70703100
H	2.68604600	-3.82913300	3.37283500
H	2.18305800	-2.15803700	3.68499900
H	1.63979100	-2.99451500	2.21703100
C	5.66028400	1.80226000	-0.17328700
C	6.58391700	1.97026000	1.04943300
C	6.45159800	1.13125600	-1.31082500
C	5.25942500	3.20008000	-0.65895100
H	6.04765100	2.45768200	1.87028300
H	6.94919700	1.00161900	1.39331700
H	7.44306400	2.59607400	0.78311800
H	5.88112800	1.16607600	-2.24303300
H	7.38843300	1.67726000	-1.47226600
H	6.68428000	0.09486900	-1.06719800
H	6.15745800	3.74516600	-0.96611300
H	4.58722200	3.14494400	-1.52295300
H	4.76753000	3.78136100	0.12796300
C	-1.11274200	4.38790100	-0.83219800
C	-1.64403400	5.43334900	0.16691500
C	0.18151500	4.93691400	-1.44825200
C	-2.13595100	4.23943800	-1.97301200
H	-2.57357200	5.08557900	0.62340200

H	-0.91056600	5.61960500	0.95782000
H	-1.84161200	6.37928200	-0.34947700
H	0.57690400	4.27124000	-2.22444100
H	-0.02574600	5.90313900	-1.91740500
H	0.96033400	5.08863300	-0.69422900
H	-2.16912800	5.17740300	-2.53893400
H	-1.86787400	3.43460300	-2.66182500
H	-3.13576400	4.03619900	-1.59125800
C	-2.51919600	1.26312500	2.89957900
C	-2.99607300	2.61824800	3.45706200
C	-3.74338200	0.47101700	2.41667400
C	-1.87316500	0.48411100	4.05169400
H	-2.14483900	3.20559100	3.81568100
H	-3.51784200	3.19193900	2.68854900
H	-3.67874000	2.45574000	4.29838000
H	-3.44850900	-0.50761800	2.01976000
H	-4.42983300	0.29960400	3.25443500
H	-4.26487900	1.03384100	1.64283600
H	-2.60678500	0.36316300	4.85446700
H	-1.55145400	-0.51701500	3.74633900
H	-1.00805000	1.01422000	4.46129800
C	1.24725800	-5.22715300	-1.58535700
H	2.00714700	-4.57247700	-2.02603500
H	1.67937000	-5.68757700	-0.69431200
H	1.02797100	-6.01251800	-2.31256400
C	-1.48032400	-2.56538500	1.73188000
H	-0.74308300	-2.95948600	2.43465200
H	-1.35178400	-1.47662500	1.67177300
H	-2.48131900	-2.75971600	2.12892900
C	-3.02457700	-2.96782400	-3.04495500
H	-2.93991300	-1.90416000	-3.29514700
H	-2.83698100	-3.54410700	-3.95187000
H	-4.05268400	-3.15522400	-2.72066600
H	-7.85131600	0.48178000	0.10054300
H	-6.05894500	0.77298700	-1.53176000

M3a

0 1

C	-3.56904200	-0.72985700	-0.70109400
C	-5.38077700	-1.54846800	0.19807000
C	-5.75925000	0.70062600	-0.44646900
H	-5.24870000	1.61345700	-0.14497900
N	-4.53024900	-2.53758800	0.22686900
N	-3.40396800	-2.01353500	-0.34129000
N	-4.83386100	-0.44147500	-0.37352800
C	-6.79691000	-1.29991500	0.60019500
H	-7.01850600	-1.69585900	1.59106300
H	-7.46987700	-1.77572300	-0.11887600
C	-6.86707900	0.24887600	0.53263200
C	-2.28513700	-2.87807300	-0.62996000
C	-2.17136100	-3.37611900	-1.93037200
C	-1.35844900	-3.13945800	0.38041100
C	-1.05472600	-4.15866400	-2.21451600
C	-0.26941500	-3.94396800	0.04779400
C	-0.09186500	-4.44265100	-1.24450700
H	-0.93142800	-4.55078800	-3.22054100
H	0.46721400	-4.17593000	0.81214400
H	-6.65706500	0.66366000	1.52108800
C	-2.64393000	0.26698100	-1.34738200
O	-3.21443900	1.28040300	-1.77976500
H	-3.30778300	2.72838200	-0.37349600
C	-1.26615000	-0.08705300	-1.41460300
C	-0.35893800	0.63442200	-2.13659800
H	-0.94301000	-0.96549100	-0.87510800
H	-0.71938900	1.51023700	-2.67570300
C	1.02886500	0.28256600	-2.31033100
C	1.61642300	-0.81609200	-1.65226900
C	1.82957600	1.06749600	-3.16197200
C	2.94721800	-1.13631600	-1.87250400
H	1.03404900	-1.42702700	-0.96835800
C	3.16217100	0.75145400	-3.37188900
H	1.38278800	1.92258600	-3.66402300
C	3.72018400	-0.35705300	-2.72939500
H	3.40002500	-1.97254400	-1.35076200
H	3.77075000	1.36351600	-4.02993200
H	4.76252400	-0.61110000	-2.88806100

C	4.51497700	0.94578600	0.33361700
C	3.25669800	1.50721600	0.35980800
C	2.12296200	0.84363500	0.87562500
C	2.33214000	-0.43323400	1.44586100
C	3.55390300	-1.06665300	1.45134500
C	4.70292200	-0.42493400	0.81184600
H	3.13078900	2.52919300	0.01649200
H	1.46771500	-0.94219000	1.86500600
C	0.80053100	1.46286000	0.87796900
C	-0.18261000	1.11282500	1.82212900
C	0.46264700	2.47668000	-0.03805900
C	-1.47864800	1.61768800	1.78350100
H	0.10429100	0.45469800	2.63281800
C	-0.80396000	3.06039900	-0.09460900
H	1.22873800	2.80478300	-0.72909000
C	-1.79669400	2.51686100	0.73800900
O	5.80936300	-1.02022600	0.69871700
O	-3.11570300	2.90330000	0.56381300
C	3.73816400	-2.45147800	2.07878900
C	4.05405200	-3.47923700	0.97863200
C	4.88264200	-2.42308100	3.10862700
C	2.47906100	-2.92391500	2.81294100
H	3.19442600	-3.57819700	0.30153600
H	4.92714700	-3.15835800	0.40763100
H	4.25278600	-4.46575900	1.41564600
H	4.64945600	-1.71391600	3.91041600
H	5.00790000	-3.41559000	3.55821000
H	5.81433900	-2.12078900	2.63211900
H	2.65898400	-3.91023100	3.25377300
H	2.19567500	-2.23632500	3.61754400
H	1.62977500	-3.01179900	2.12790000
C	5.74845000	1.75327600	-0.09028000
C	6.72030900	1.82695200	1.10292600
C	6.47258600	1.11016700	-1.28666600
C	5.38927100	3.18793600	-0.49388600
H	6.23529700	2.31160600	1.95701000
H	7.03215600	0.82359300	1.39595700
H	7.60669100	2.41391000	0.83352800

H	5.86944100	1.21146900	-2.19473000
H	7.42629900	1.62292800	-1.46278300
H	6.66427400	0.05487300	-1.09087700
H	6.29973900	3.71828500	-0.79267900
H	4.69719300	3.20279300	-1.34398900
H	4.93301300	3.74304900	0.33257500
C	-1.05374200	4.32442200	-0.94705000
C	-1.43295300	5.46123100	0.02186000
C	0.22103400	4.74754300	-1.69074100
C	-2.16199200	4.19326400	-2.00961800
H	-2.34327600	5.21206900	0.57252700
H	-0.62735300	5.63581300	0.74129100
H	-1.60455200	6.38884700	-0.53585600
H	0.53300500	3.99372100	-2.42286500
H	0.02761000	5.67871700	-2.23108100
H	1.05377500	4.92391700	-1.00434800
H	-2.12875000	5.07097600	-2.66364800
H	-2.04155700	3.29985800	-2.62582000
H	-3.16335700	4.17226900	-1.57276800
C	-2.47393000	1.33796800	2.92506900
C	-2.92328400	2.68122200	3.53141100
C	-3.70918200	0.56971300	2.43240600
C	-1.83842500	0.51778700	4.05564000
H	-2.05585000	3.24044500	3.89493500
H	-3.44890600	3.29489100	2.79805700
H	-3.59309800	2.50089900	4.37973600
H	-3.42345400	-0.41140100	2.03341800
H	-4.40231700	0.40025100	3.26501100
H	-4.22427600	1.14228900	1.66089500
H	-2.57718900	0.37965100	4.85101600
H	-1.52148400	-0.47540900	3.72208200
H	-0.97180700	1.02785400	4.48563700
C	1.14064900	-5.23176300	-1.59629100
H	1.90327000	-4.56421100	-2.01224100
H	1.56870400	-5.71450800	-0.71533800
H	0.92420400	-5.99613200	-2.34591200
C	-1.48967200	-2.50032300	1.73458400
H	-0.74800300	-2.90195500	2.42789300

H	-1.32089200	-1.41771600	1.65051800
H	-2.48532800	-2.65360800	2.16175400
C	-3.18764100	-3.04237400	-2.98936700
H	-3.05946800	-2.01175700	-3.33977900
H	-3.07082500	-3.70162400	-3.85034600
H	-4.20912400	-3.14796500	-2.61248000
H	-7.84635300	0.60402900	0.21404600
H	-6.09831400	0.80172100	-1.47892300

M04a

0 1

C	-1.98405300	-0.63467600	0.76838000
C	-3.34521200	-1.92512100	-0.31973300
C	-2.23227700	-3.17911400	1.35739100
H	-1.17149900	-3.28557500	1.58510400
N	-3.51167500	-0.75309000	-0.86530600
N	-2.65308700	0.04570100	-0.16618300
N	-2.45608400	-1.87513200	0.71306800
C	-3.82447600	-3.32477500	-0.52793300
H	-3.87115900	-3.58315800	-1.58548400
H	-4.82089300	-3.44839300	-0.09227600
C	-2.74251200	-4.12365300	0.24810200
C	-2.61624000	1.46898300	-0.36983800
C	-3.04887500	2.29063400	0.67840500
C	-2.16528500	1.96732100	-1.59696500
C	-2.97027500	3.66820700	0.48373700
C	-2.11101200	3.35318300	-1.73450200
C	-2.49330800	4.21600400	-0.70678700
H	-3.30051200	4.32860000	1.28147400
H	-1.75663700	3.76766000	-2.67495000
H	-1.90631900	-4.34846700	-0.42091700
C	-0.87974100	-0.11315200	1.65910800
O	-0.92854000	-0.37973100	2.84654200
C	0.13878600	0.66835700	0.96865300
C	1.19751000	1.12137300	1.66431300
H	0.07514100	0.70921800	-0.11456800
H	1.18417200	0.97536800	2.74236100
C	2.39666500	1.73692800	1.09511000

C	3.50057600	1.95321300	1.92966600
C	2.50538400	2.05959400	-0.26559100
C	4.68853400	2.46651600	1.41971400
H	3.42355000	1.69757000	2.98330700
C	3.68897900	2.57631300	-0.77314400
H	1.66221900	1.90225400	-0.93170100
C	4.78549800	2.77825500	0.06607200
H	5.53702800	2.62287200	2.07814400
H	3.76212300	2.81519500	-1.82929900
H	5.71139400	3.17780300	-0.33545100
C	-2.38263100	5.70867500	-0.87296900
H	-1.42944400	6.06868600	-0.47254800
H	-2.42973100	5.99441500	-1.92590100
H	-3.18177900	6.22608700	-0.33723100
C	-1.76744300	1.05789100	-2.72700700
H	-1.12809000	0.24041600	-2.38381800
H	-2.65409700	0.60128600	-3.17724800
H	-1.24586300	1.62656100	-3.49900300
C	-3.61041600	1.72998000	1.96130300
H	-4.26420300	0.87439300	1.76733700
H	-2.82433100	1.39889800	2.64705700
H	-4.19591000	2.49450600	2.47427200
H	-2.83855000	-3.21348100	2.26600900
H	-3.12861400	-5.05640700	0.65877100
C	2.18984800	-1.72692300	-1.14476400
C	2.79546200	-1.50750800	0.08592000
C	4.08690600	-0.98349600	0.11884600
C	4.71693800	-0.67962000	-1.08390600
C	4.08354400	-0.90112000	-2.31216400
C	2.80024400	-1.43941800	-2.35619100
C	1.87461000	-1.85660000	1.20924300
H	4.58606300	-0.79058100	1.06316800
H	5.71437400	-0.25133600	-1.06782100
H	4.59760200	-0.65318600	-3.23507900
H	2.29392900	-1.62455600	-3.29812400
S	0.57731000	-2.41111300	-0.82382300
O	0.50241800	-3.80856400	-1.28137600
O	-0.47607900	-1.55528000	-1.41904800

N	0.62364200	-2.25800900	0.77571500
C	2.20462400	-1.69271600	2.50589800
H	3.18652100	-1.33155500	2.78741500
H	1.49247500	-1.92621200	3.28793800

TS4(R)a

0 1

C	-1.97892100	-0.56708500	0.84737900
C	-3.26406800	-1.97133000	-0.20744600
C	-2.17320000	-3.06335200	1.59506800
H	-1.11527100	-3.12997700	1.84492400
N	-3.43234400	-0.84816600	-0.84431500
N	-2.61846200	0.02296000	-0.16797300
N	-2.41690300	-1.82347200	0.84949000
C	-3.71031000	-3.39272100	-0.32204700
H	-3.70309800	-3.73795400	-1.35549900
H	-4.72449600	-3.49953800	0.07469600
C	-2.65531700	-4.11042900	0.56458300
C	-2.60916900	1.42301400	-0.49109100
C	-3.11225400	2.32099200	0.45663100
C	-2.11449200	1.82206500	-1.73630500
C	-3.08340800	3.67551500	0.13003200
C	-2.11281600	3.18847200	-2.01050600
C	-2.58215900	4.12701000	-1.09084700
H	-3.46969900	4.39460000	0.84776900
H	-1.73020300	3.52652800	-2.97032700
H	-1.80356900	-4.40654500	-0.05344300
C	-0.92158300	-0.03451100	1.78204400
O	-0.99362400	-0.40919400	2.96082200
C	0.07764200	0.73636800	1.15424400
C	1.24967600	1.00752300	1.86174600
H	0.01872600	0.86078300	0.07872100
H	1.14304800	1.08473700	2.94099300
C	2.37558000	1.77713500	1.28471000
C	3.37734800	2.26444000	2.13548800
C	2.49787000	2.01438000	-0.08983300
C	4.46898300	2.96237300	1.63263200
H	3.29401900	2.08377000	3.20456200

C	3.58734900	2.71757500	-0.59482000
H	1.74844100	1.63355500	-0.77699000
C	4.57794800	3.19137400	0.26145400
H	5.23322400	3.33070700	2.30952300
H	3.66635800	2.88614600	-1.66415200
H	5.42857000	3.73621100	-0.13510500
C	-2.52880700	5.59984600	-1.40259300
H	-1.56316900	6.01993600	-1.10347700
H	-2.65154600	5.78216300	-2.47255400
H	-3.30789900	6.14621200	-0.86657600
C	-1.61005100	0.82205100	-2.74050600
H	-0.96196900	0.07436500	-2.27515900
H	-2.44434200	0.27579900	-3.19064400
H	-1.06212400	1.33105700	-3.53556000
C	-3.68002100	1.85729000	1.77385400
H	-4.34549600	0.99920100	1.63812000
H	-2.89404100	1.55803100	2.47473900
H	-4.25275100	2.66167500	2.23786700
H	-2.77719600	-3.04027200	2.50484900
H	-3.05941300	-4.99721400	1.05254900
C	2.23320300	-1.95703900	-1.21167800
C	2.83181200	-1.40728400	-0.08374000
C	4.07871300	-0.80047900	-0.20027700
C	4.69014100	-0.77571000	-1.45295100
C	4.07374700	-1.34183700	-2.57185600
C	2.81949300	-1.94453000	-2.46460300
C	1.94237500	-1.53252600	1.10623000
H	4.55471900	-0.33057300	0.65550900
H	5.65819800	-0.29806000	-1.56247100
H	4.57243900	-1.30781900	-3.53461500
H	2.32409400	-2.38036900	-3.32562000
S	0.65145700	-2.60048700	-0.69922600
O	0.61780300	-4.06239500	-0.79786100
O	-0.44831800	-1.90377700	-1.38479600
N	0.75959100	-2.10996000	0.86384100
C	2.19179000	-0.88759000	2.30856600
H	3.19134600	-0.53410700	2.53174800
H	1.53714400	-1.12786000	3.13926300

M4(R)a

0 1

C	0.93143100	1.20232700	-1.33724400
C	2.66546600	2.53651500	-1.28753400
C	3.04803200	0.75928700	-2.80939300
H	3.00722500	-0.31655300	-2.66812300
N	1.83089900	3.07796000	-0.44775100
N	0.74722000	2.23213000	-0.49331700
N	2.14732500	1.41179900	-1.85134300
C	4.04068700	2.79779700	-1.81385200
H	4.73159300	3.11964600	-1.03478200
H	3.99820300	3.57794700	-2.58049000
C	4.39156600	1.41089500	-2.41727900
C	-0.38628600	2.47546200	0.35147100
C	-1.59514500	2.87573100	-0.22478900
C	-0.22952600	2.25345200	1.72076300
C	-2.68062900	3.04778100	0.63163500
C	-1.34656600	2.43900000	2.53450700
C	-2.57712700	2.83037800	2.00774500
H	-3.63541100	3.35521500	0.21053000
H	-1.25178900	2.26432700	3.60345100
H	4.86100400	0.79145700	-1.64904000
C	0.10935000	0.00654900	-1.74170800
O	0.49855200	-0.50069500	-2.83086100
C	-0.86680400	-0.40830100	-0.86756100
C	-1.55186300	-1.71307200	-1.15392600
H	-1.05065400	0.11118500	0.06656200
H	-1.14760200	-2.05587200	-2.11310700
C	-3.06773500	-1.66032600	-1.25005600
C	-3.76802200	-2.75945700	-1.75943600
C	-3.79735700	-0.55336500	-0.81228000
C	-5.15708100	-2.75563600	-1.82908100
H	-3.21219900	-3.62419500	-2.11587200
C	-5.18988700	-0.54642100	-0.87820400
H	-3.26777800	0.31595600	-0.43122500
C	-5.87511900	-1.64663100	-1.38417300
H	-5.68016300	-3.61571200	-2.23609200

H	-5.73989000	0.32605600	-0.53681400
H	-6.95915100	-1.63958200	-1.43897200
C	-3.77898700	3.02135200	2.89589500
H	-3.55237400	2.76368200	3.93227300
H	-4.11905400	4.06091200	2.87120100
H	-4.61281000	2.39692800	2.56217600
C	1.09042600	1.79589100	2.27710100
H	1.42081000	0.87654400	1.77730100
H	1.86587100	2.55012700	2.12013000
H	1.00712700	1.59312000	3.34544900
C	-1.74030100	3.05324700	-1.71075300
H	-0.87247100	3.55863500	-2.14284600
H	-1.83441500	2.07279500	-2.19119000
H	-2.63297700	3.63686000	-1.94151800
H	2.71525700	1.01175100	-3.81771700
H	5.07036300	1.48725700	-3.26653300
C	2.02588100	-1.89255300	1.76990900
C	0.64758400	-1.99597300	1.64643300
C	-0.16520200	-1.64929500	2.71996800
C	0.44788300	-1.23179200	3.90301100
C	1.83728600	-1.15523500	4.01091400
C	2.65727700	-1.47626900	2.92585500
C	0.28092600	-2.55605000	0.30617400
H	-1.24639300	-1.70459700	2.65138000
H	-0.16741900	-0.96577900	4.75614600
H	2.28637500	-0.83572200	4.94542800
H	3.73799600	-1.41268900	2.99278000
S	2.72057500	-2.35087500	0.19407500
O	3.60765300	-3.49603300	0.30941600
O	3.20179300	-1.15134000	-0.48625100
N	1.24922400	-2.83155700	-0.49605800
C	-1.14726100	-2.78014800	-0.09030000
H	-1.79114900	-2.69124000	0.78911300
H	-1.26137100	-3.79309300	-0.48679200

3a(S)

0 1

C	0.02174300	-2.02250400	0.13538500

O	-0.36763600	-3.16057800	0.24393400
C	1.46215500	-1.57669900	0.27130400
C	1.83741400	-0.36808400	-0.60664000
H	2.09809600	-2.43805300	0.06136300
H	1.84212200	-0.69723000	-1.65633900
C	3.23422000	0.10838100	-0.26340000
C	4.32280100	-0.27622900	-1.04668100
C	3.45877900	0.89629100	0.86832800
C	5.61418900	0.12041500	-0.70872500
H	4.15602000	-0.88898700	-1.92905400
C	4.74793900	1.29525200	1.20765500
H	2.61574900	1.19876700	1.48570400
C	5.82930800	0.90775400	0.41879200
H	6.45169600	-0.18363300	-1.32835200
H	4.90938100	1.90877100	2.08837800
H	6.83479400	1.21916500	0.68235500
C	-2.85356600	0.53001700	0.01493300
C	-1.67247600	1.24517000	-0.15425900
C	-1.73465100	2.63583600	-0.23886600
C	-2.97478600	3.25762500	-0.14868500
C	-4.14906000	2.51690300	0.02250600
C	-4.10126000	1.12940800	0.10576700
C	-0.48229900	0.38553300	-0.23212500
H	-0.83148700	3.22232600	-0.37041600
H	-3.03237400	4.33896800	-0.21186700
H	-5.10353200	3.02675800	0.09028800
H	-5.00048300	0.53673100	0.23642800
S	-2.56620800	-1.21666100	0.08064600
O	-3.08120800	-1.88113200	-1.10108500
O	-2.87003900	-1.75789200	1.39122300
N	-0.87166400	-0.96949900	-0.06672500
C	0.79021900	0.70930600	-0.47068600
H	1.06651900	1.74757100	-0.61879100
H	1.60152900	-1.30752300	1.32685100

Pre-NHC

1 1

C	-0.49242500	-0.15750400	0.32144700

C	-0.05082100	1.78514200	-0.53175800
C	1.97009700	0.51127300	0.28931900
H	2.12641400	0.71681300	1.35218600
N	-1.34648900	1.65917400	-0.61546800
N	-1.60278900	0.43813700	-0.07870300
N	0.51621000	0.67312400	0.03724700
C	0.80891000	2.93005500	-0.98102700
H	0.34184400	3.87397400	-0.69889000
H	0.91280100	2.89633200	-2.07401600
C	2.67191100	1.58741500	-0.54220400
O	2.06070600	2.84723300	-0.34267000
C	-2.94787800	-0.07286300	0.00860400
C	-3.35651300	-1.01824400	-0.93153800
C	-3.77227600	0.43115400	1.01968900
C	-4.66583000	-1.48847400	-0.82190100
C	-5.06791800	-0.07121800	1.08122600
C	-5.52875300	-1.03034000	0.17319900
H	-5.01820100	-2.22536800	-1.53811900
H	-5.73644800	0.29567600	1.85558500
H	3.70801900	1.66997100	-0.21462000
C	-2.43306200	-1.50518500	-2.01761800
H	-1.93065100	-0.67108300	-2.51608400
H	-1.66039000	-2.17064100	-1.61934000
H	-2.99484000	-2.06216800	-2.76806800
C	-6.93971200	-1.54660900	0.27414800
H	-7.11175000	-2.01252600	1.24838000
H	-7.65793000	-0.72827600	0.17273600
H	-7.14865900	-2.28488300	-0.50157400
C	-3.27664000	1.47628200	1.98306800
H	-2.35762900	1.15528700	2.48320200
H	-3.05727400	2.41295900	1.46253200
H	-4.02824400	1.67590200	2.74716100
H	2.65675600	1.31019200	-1.60719200
C	2.42476200	-0.90751600	-0.06597500
H	1.89585000	-1.62785200	0.56664800
H	2.15518800	-1.11477600	-1.10703500
C	3.91644400	-1.05683800	0.13336000
C	4.78515200	-1.05480400	-0.95986700

C	4.44586700	-1.16193800	1.42268200
C	6.16137800	-1.15624500	-0.76851100
H	4.38239000	-0.98058600	-1.96659700
C	5.82048600	-1.26144700	1.61545500
H	3.77726700	-1.17491100	2.27992300
C	6.68122400	-1.25793800	0.51914200
H	6.82568900	-1.15834400	-1.62633800
H	6.21956400	-1.34641400	2.62080800
H	7.75278700	-1.33827600	0.66870800
H	-0.43314600	-1.12685100	0.79419000

M0^{DBU+R1}

1 1

C	1.25263000	0.54725700	-1.22926200
C	3.37851200	0.43601500	-0.76341800
C	2.27827800	-1.77203000	-1.30198500
H	1.83860100	-2.18386800	-0.38937700
N	3.07204700	1.69884600	-0.64139200
N	1.74242200	1.74375500	-0.93662500
N	2.28184100	-0.30291500	-1.13242300
C	4.69390400	-0.23996000	-0.50449300
H	5.13589600	0.15753700	0.41360300
H	5.37692200	-0.05341800	-1.34385900
C	3.74268200	-2.19158800	-1.39736400
O	4.47501100	-1.62237200	-0.32258200
C	1.00469600	2.97909900	-0.98396800
C	1.30060500	3.87074600	-2.01670900
C	-0.00113300	3.20031600	-0.03779400
C	0.54632100	5.04247000	-2.08211600
C	-0.72780700	4.38236000	-0.15408900
C	-0.46893300	5.31264500	-1.16505600
H	0.75415500	5.75454400	-2.87604400
H	-1.51649200	4.58428300	0.56615900
H	3.81487600	-3.27557000	-1.30562800
C	0.69103700	-0.90644600	1.99596400
O	0.20847600	-1.46727900	1.04054100
C	2.12571000	-0.46634300	2.06318100
C	2.76268900	-0.18749700	3.42492000

H	2.15341400	0.46569600	1.48219000
H	2.72047300	-1.19914600	1.50707100
H	2.18527600	0.57374200	3.95729100
H	2.76590200	-1.09410900	4.03590600
C	4.17326000	0.29104800	3.16507800
C	4.39993700	1.61530200	2.77633900
C	5.24960600	-0.59842500	3.18915700
C	5.67792800	2.04529800	2.42843500
H	3.56538900	2.31290900	2.74299000
C	6.53098000	-0.17150400	2.84387400
H	5.08197100	-1.63265000	3.47776000
C	6.74796400	1.15110400	2.46243200
H	5.83878300	3.07660700	2.13173400
H	7.35850600	-0.87292200	2.87087100
H	7.74479600	1.48422400	2.19311900
C	-0.29868100	2.21759400	1.06418700
H	-0.61641200	1.24799200	0.66184400
H	0.57527700	2.06342500	1.70800300
H	-1.11017100	2.59799600	1.68606300
C	-1.28788000	6.57308600	-1.26002000
H	-2.32672500	6.33897500	-1.51160500
H	-1.29535300	7.10463600	-0.30472800
H	-0.89510600	7.24440100	-2.02532200
C	2.37524700	3.57297500	-3.02828100
H	3.36761000	3.61065000	-2.57143600
H	2.24906500	2.57274100	-3.45598900
H	2.34162000	4.29866300	-3.84140000
O	-0.02724200	-0.52204500	3.07748500
C	-1.40403400	-0.69502100	3.07494500
C	-2.15463200	0.41966700	3.43594500
C	-1.99665000	-1.92170900	2.78134100
C	-3.53908100	0.32579900	3.47137900
H	-1.64698200	1.34346100	3.68749000
C	-3.38154500	-2.01927000	2.81700600
H	-1.38879000	-2.77767500	2.51884100
C	-4.12471200	-0.89189400	3.14898700
H	-4.15781100	1.17255300	3.73884200
H	-3.88499700	-2.95058600	2.58832800

N	-5.58854400	-0.98770600	3.15507500
O	-6.08947700	-2.05718700	2.85223700
O	-6.22199800	0.00842000	3.45421700
H	4.17165300	-1.88528300	-2.36318600
C	1.42355000	-2.17462500	-2.50480600
H	0.42971100	-1.72300300	-2.39773700
H	1.87241500	-1.77895700	-3.42203900
C	1.28785700	-3.67792800	-2.57084200
C	2.01811700	-4.42958400	-3.49241000
C	0.44284900	-4.33570300	-1.67160500
C	1.90356000	-5.81834100	-3.51930500
H	2.67529700	-3.92530400	-4.19610000
C	0.32806100	-5.72204200	-1.69662900
H	-0.12879500	-3.75272900	-0.95195000
C	1.05885000	-6.46628200	-2.62196900
H	2.47268800	-6.39201400	-4.24323000
H	-0.33449900	-6.22224300	-0.99788700
H	0.96726400	-7.54711500	-2.64408400
H	0.17776600	0.38253100	-1.48760100
C	-4.88962900	-1.83987400	-0.59048000
C	-3.36787800	-1.85149600	-0.73928800
C	-2.66575900	-0.55143400	-0.31105900
C	-5.60971800	-0.75272000	-1.38879200
C	-2.67845100	0.51371200	-1.37859200
C	-5.12721900	0.66126800	-1.05319400
H	-5.13624000	-1.71188500	0.47250300
H	-2.96580700	-2.66596800	-0.12597800
H	-3.11238800	-0.16697000	0.61588000
H	-5.48119500	-0.91703000	-2.46636000
H	-5.87449900	1.39763000	-1.35941900
C	-1.56586500	1.86270700	-2.95048200
H	-0.65099200	1.76871500	-3.54764600
C	-4.03636500	1.84785500	-2.94045200
C	-2.80650300	1.72575200	-3.82593600
H	-2.79521300	0.74372700	-4.31049400
H	-2.83561600	2.48945000	-4.60648500
H	-4.92782400	1.49985200	-3.47394100
N	-3.88585200	1.03515600	-1.73028800

N	-1.54299400	0.84054900	-1.91119400
H	-5.01158600	0.77305500	0.03177900
H	-6.68307000	-0.81517600	-1.18155900
H	-5.28441200	-2.81990100	-0.87796000
H	-3.08945500	-2.07625800	-1.77714800
H	-1.61796700	-0.76256900	-0.09547100
H	-1.54372500	2.86331800	-2.49165700
H	-4.20792500	2.89479800	-2.65885200

TS0^{DBU+R1}

1 1

C	-1.20467400	-0.47751500	-1.20481200
C	-3.32213900	-0.04788100	-0.75843500
C	-1.88918000	1.95967100	-1.25484400
H	-1.40598600	2.30185100	-0.33473500
N	-3.22056000	-1.34269200	-0.65185700
N	-1.90410600	-1.58015400	-0.93257700
N	-2.11527200	0.51090000	-1.10235200
C	-4.52272600	0.81908700	-0.51494200
H	-5.04066300	0.48730400	0.38931800
H	-5.20997200	0.74848900	-1.36921600
C	-3.26661100	2.60760400	-1.36800300
O	-4.10182700	2.15227600	-0.31389800
C	-1.37577700	-2.91317900	-1.02563000
C	-1.79317100	-3.70894100	-2.09590200
C	-0.42270800	-3.32868500	-0.09140200
C	-1.21845400	-4.97370100	-2.21650100
C	0.12616900	-4.59854400	-0.26173400
C	-0.25571900	-5.43137100	-1.31639300
H	-1.52598300	-5.61163300	-3.04082800
H	0.86666400	-4.94976700	0.45257300
H	-3.16911000	3.68894100	-1.26427700
C	-0.56919900	1.01486400	2.14342500
O	-0.01115300	1.70434400	1.32443900
C	-2.03213300	0.67925100	2.11054600
C	-2.74433000	0.38329200	3.43256400
H	-2.09813500	-0.22036000	1.48205700
H	-2.54077800	1.48479200	1.57106700

H	-2.26326800	-0.46262800	3.93179300
H	-2.67713200	1.24739900	4.09957700
C	-4.18813000	0.07041300	3.11216000
C	-4.54283400	-1.19482300	2.63432100
C	-5.16787100	1.06331500	3.18196600
C	-5.85154000	-1.46566500	2.24336300
H	-3.78535100	-1.97256900	2.56094500
C	-6.47993600	0.79523000	2.79559400
H	-4.89978800	2.05378700	3.54041800
C	-6.82482800	-0.47023900	2.32542500
H	-6.11161800	-2.45312700	1.87573800
H	-7.23145700	1.57540400	2.86179200
H	-7.84601400	-0.68051200	2.02460800
C	-0.00528900	-2.45703900	1.06455800
H	0.48619900	-1.53765200	0.72323700
H	-0.86566200	-2.17608300	1.68284600
H	0.70179700	-2.99783100	1.69527100
C	0.37919600	-6.78695900	-1.48517700
H	1.36020400	-6.69445600	-1.96195200
H	0.52878800	-7.27323300	-0.51840700
H	-0.23643500	-7.43695000	-2.10969200
C	-2.81301600	-3.21532800	-3.08747000
H	-3.80461900	-3.15016100	-2.63156100
H	-2.55834200	-2.21500600	-3.45322700
H	-2.86907400	-3.88998200	-3.94272200
O	0.08437400	0.37488200	3.14783600
C	1.46976300	0.39462000	3.17173300
C	2.08546500	-0.83594000	3.38339700
C	2.20379600	1.57098900	3.02788000
C	3.47125400	-0.91337200	3.39361100
H	1.47129900	-1.71615200	3.53282800
C	3.59010500	1.49624100	3.03398000
H	1.70430700	2.52112900	2.89595800
C	4.19583800	0.25548800	3.19614300
H	3.98482300	-1.85502200	3.53988200
H	4.20012600	2.38259500	2.90921200
N	5.65903100	0.17715600	3.14569600
O	6.27325600	1.19801500	2.88558800

O	6.17860900	-0.90451400	3.35306500
H	-3.72221500	2.38269300	-2.34453400
C	-0.96969200	2.24035200	-2.44408500
H	-0.05943400	1.63788000	-2.33114900
H	-1.46080800	1.92218100	-3.37030700
C	-0.60795500	3.70574600	-2.49967100
C	-1.11691400	4.53974000	-3.49557800
C	0.22841400	4.24928700	-1.51896400
C	-0.79090900	5.89479600	-3.51777400
H	-1.76951400	4.12525700	-4.25972600
C	0.55414500	5.60164000	-1.53907900
H	0.62336300	3.60336300	-0.73660600
C	0.04519900	6.42754200	-2.54055700
H	-1.19007500	6.53294700	-4.29948000
H	1.20813800	6.01155200	-0.77613400
H	0.30072700	7.48196600	-2.55824700
H	0.07589300	-0.57438100	-1.49525500
C	4.63594500	1.69713200	-0.45132100
C	3.10884400	1.68608100	-0.53080100
C	2.45230100	0.32936700	-0.22945600
C	5.33960200	0.73457200	-1.40759000
C	2.46659800	-0.63352600	-1.38759900
C	4.92188000	-0.72291800	-1.20496400
H	4.93962100	1.44547300	0.57442800
H	2.71509900	2.40237900	0.19935600
H	2.92869600	-0.13859600	0.64324500
H	5.14605300	1.01214500	-2.45136000
H	5.66873600	-1.39643300	-1.63054900
C	1.28008600	-1.93669700	-2.99290300
H	0.33052600	-1.80034300	-3.52315300
C	3.75004600	-1.83535700	-3.10072800
C	2.47124200	-1.71662500	-3.91552900
H	2.40391200	-0.71679600	-4.35613200
H	2.48547000	-2.44602000	-4.72802100
H	4.60156300	-1.44400900	-3.66654000
N	3.65267200	-1.06502200	-1.85495100
N	1.31588000	-0.98578500	-1.88987700
H	4.86507000	-0.95830500	-0.13549400

H	6.42045200	0.80917200	-1.25125600
H	4.99316000	2.71534800	-0.63578600
H	2.77705200	2.03094100	-1.51921600
H	1.40581600	0.49609100	0.03294300
H	1.28352500	-2.96269000	-2.59653600
H	3.96386100	-2.88330100	-2.85875900

M0^{DBU}

1 1

C	-0.63155800	-1.34446600	-0.21315400
C	-0.43619000	-3.51446900	-0.22646000
C	1.73858200	-2.24031000	-0.28590700
H	1.99534200	-2.06191700	-1.33494700
N	-1.72052700	-3.29087300	-0.22016500
N	-1.81893500	-1.93161200	-0.21077100
N	0.26597300	-2.33635300	-0.22264000
C	0.29458900	-4.82622100	-0.20255600
H	-0.17288300	-5.52771200	-0.89429200
H	0.24870100	-5.24383200	0.81274900
C	2.27184900	-3.61027200	0.12983800
O	1.62879700	-4.63009100	-0.61271700
C	-3.08540000	-1.25114900	-0.22989400
C	-3.94783400	-1.42593300	0.85908900
C	-3.36753700	-0.40965000	-1.30842300
C	-5.14440400	-0.71691200	0.83901900
C	-4.58284200	0.27697800	-1.27495100
C	-5.47879000	0.13668600	-0.21698300
H	-5.83252700	-0.82684800	1.67332200
H	-4.83118700	0.93577100	-2.10275000
H	3.33832400	-3.66568300	-0.09117000
C	-2.41021700	-0.21519500	-2.45622600
H	-1.61415300	0.48481800	-2.17647900
H	-1.94578800	-1.15684900	-2.76243800
H	-2.93728300	0.20029600	-3.31629000
C	-6.79321700	0.87130600	-0.20842900
H	-6.97601200	1.33215700	0.76564300
H	-6.81781500	1.65111800	-0.97165200
H	-7.61918700	0.18051300	-0.40349600

C	-3.58676700	-2.32195300	2.01422700
H	-3.60187300	-3.37360600	1.71668700
H	-2.58000000	-2.10323800	2.38528700
H	-4.29258500	-2.18317400	2.83391300
H	2.12172300	-3.76019200	1.20987600
C	2.24796300	-1.09722900	0.59413100
H	1.72694800	-0.16854800	0.32587600
H	2.00192100	-1.31428900	1.63926500
C	3.73999700	-0.92290300	0.42560700
C	4.62013800	-1.24014600	1.46072400
C	4.25987400	-0.46683800	-0.79018300
C	5.99574200	-1.09503600	1.28964300
H	4.22596500	-1.60006100	2.40747800
C	5.63260800	-0.32109500	-0.96362500
H	3.58403200	-0.21729900	-1.60599300
C	6.50414600	-0.63503900	0.07822600
H	6.66851400	-1.34204600	2.10437400
H	6.02183400	0.03917600	-1.91033400
H	7.57484600	-0.52082900	-0.05541900
H	-0.45995700	-0.23604500	-0.19842500
C	2.91473500	4.22276900	-0.56899100
C	2.56487300	2.73308400	-0.55519000
C	1.22066500	2.38841600	-1.22330400
C	1.92502200	5.12757700	0.16404800
C	0.04430500	2.57723600	-0.29413100
C	0.49666100	5.01080700	-0.37257900
H	2.97815700	4.55281400	-1.61488100
H	3.35923800	2.19065000	-1.07684100
H	1.09227100	2.98407800	-2.13496800
H	1.91342400	4.89698200	1.23699100
H	-0.09200300	5.88150900	-0.07353200
C	-1.71146000	1.69257500	1.00885400
H	-1.87585700	0.74094900	1.53021500
C	-1.15581700	4.08968800	1.23137700
C	-1.42617300	2.80990900	2.00434300
H	-0.54445300	2.54372500	2.59700300
H	-2.26304600	2.95996200	2.69023600
H	-0.70952000	4.84498400	1.88737300

N	-0.22233300	3.84082800	0.12991800
N	-0.60382300	1.51735500	0.07630100
H	0.50112800	5.00422500	-1.46812000
H	2.25357300	6.16737600	0.06359700
H	3.91282400	4.35671700	-0.13963200
H	2.55819700	2.35483700	0.47637300
H	1.20210700	1.33825500	-1.52749100
H	-2.63770200	1.90609400	0.45430400
H	-2.08935400	4.50719200	0.83298300

TS0^{DBU}

1 1

C	-0.36784100	-1.26406400	-0.27716800
C	0.13270000	-3.38158400	-0.63762600
C	2.09559600	-1.82419200	-0.43313500
H	2.34690700	-1.41366800	-1.41701200
N	-1.16845900	-3.35337700	-0.60198600
N	-1.44957600	-2.03454700	-0.37782600
N	0.65447300	-2.12745400	-0.43976100
C	1.03995200	-4.56270500	-0.82229800
H	0.67400700	-5.19520200	-1.63215400
H	1.06046800	-5.15191600	0.10526600
C	2.82317700	-3.15588400	-0.25019600
O	2.33266000	-4.11640700	-1.16731100
C	-2.79768600	-1.56035800	-0.23551900
C	-3.51043400	-1.92840000	0.91195800
C	-3.31078100	-0.70287600	-1.21007900
C	-4.78988300	-1.40336000	1.06583900
C	-4.59802900	-0.20092400	-1.00571400
C	-5.34889900	-0.53895400	0.11919100
H	-5.36382100	-1.67003800	1.94985700
H	-5.02227800	0.46809000	-1.74981800
H	3.88600900	-3.02161800	-0.45378000
C	-2.51292500	-0.31324200	-2.42706100
H	-1.77212200	0.45379800	-2.17486400
H	-1.97854900	-1.16899500	-2.84785600
H	-3.17110900	0.09515700	-3.19546700
C	-6.74342800	-0.00276100	0.31138700

H	-6.87299000	0.39905000	1.31967800
H	-6.96654100	0.78784500	-0.40736200
H	-7.48304500	-0.79824800	0.17968600
C	-2.90363300	-2.83993400	1.94520400
H	-2.78129900	-3.85236600	1.55121100
H	-1.91128800	-2.48808700	2.24699000
H	-3.53666700	-2.88567700	2.83226600
H	2.70210900	-3.51084200	0.78526000
C	2.41979600	-0.80897900	0.66606600
H	1.82872700	0.09979300	0.49689400
H	2.10655400	-1.22381100	1.63055100
C	3.89385600	-0.47903300	0.68280300
C	4.72527500	-0.95835200	1.69648800
C	4.45246800	0.28354500	-0.34693900
C	6.08986600	-0.67647200	1.68516800
H	4.30058500	-1.55272600	2.50141500
C	5.81474800	0.56631600	-0.36126400
H	3.81210800	0.65877700	-1.14194800
C	6.63674300	0.08645800	0.65702500
H	6.72458500	-1.05207100	2.48113900
H	6.23480600	1.16261800	-1.16481700
H	7.69886400	0.30796200	0.64872800
H	-0.48277600	0.03701600	-0.04788600
C	1.84743000	4.51784900	-1.25512400
C	1.83304700	2.99715500	-1.08929000
C	0.47948400	2.32879900	-1.39035200
C	0.88511900	5.27778500	-0.34366700
C	-0.49667800	2.40210500	-0.24129200
C	-0.57137500	4.85009400	-0.52478700
H	1.59990100	4.75457000	-2.29857000
H	2.57119500	2.57509300	-1.77789200
H	0.03024200	2.77326700	-2.28693100
H	1.16194200	5.14496900	0.70958000
H	-1.24534900	5.61407500	-0.13148600
C	-1.80846100	1.29361300	1.41760600
H	-1.67092800	0.36591300	1.98486500
C	-1.68009800	3.76396000	1.42211900
C	-1.57083700	2.51943900	2.28855000

H	-0.56985300	2.45975400	2.72763000
H	-2.29651700	2.57528700	3.10242400
H	-1.28174000	4.63520500	1.95154200
N	-0.90942300	3.61071200	0.18149900
N	-0.86706900	1.27865900	0.30585400
H	-0.80904200	4.74358900	-1.58883700
H	0.96095500	6.34774400	-0.56222800
H	2.86626200	4.88047300	-1.08711900
H	2.15341200	2.72098700	-0.07585700
H	0.63018700	1.26740900	-1.60896100
H	-2.83814100	1.28505200	1.03199000
H	-2.72631100	3.97482200	1.17053500

IM3 (M06-2X/6-31G(d,p)/IEF-PCM$_{THF}$)

0 1

C	0.76505600	0.74307500	0.94925900
C	2.26274200	0.26089000	2.46447900
C	3.21455400	0.95914300	0.23878000
H	3.20328500	0.20119800	-0.54843300
N	1.12104200	0.16611200	3.08043800
N	0.19504700	0.46600700	2.12795200
N	2.09556500	0.64527300	1.16031200
C	3.63079400	-0.00477200	3.01666500
H	3.60662100	-0.91869400	3.61294600
H	3.94326700	0.83529100	3.65334800
C	4.51544600	0.88014300	1.05305700
O	4.53042100	-0.20413800	1.95526100
C	-1.19128500	0.57924900	2.51456600
C	-1.58091700	1.77595500	3.12655900
C	-2.06471900	-0.48330300	2.28573700
C	-2.91790900	1.90351000	3.49510600
C	-3.39189400	-0.30676400	2.68341800
C	-3.83610800	0.87211000	3.28170600
H	-3.24993400	2.82974700	3.95688900
H	-4.09658800	-1.11783800	2.51461800
H	5.34661700	0.73693400	0.36092600
C	0.09959200	1.04350300	-0.33443300
O	0.69583200	0.62733000	-1.45393200

C	-1.00108500	1.81484800	-0.32670700
C	-1.76280400	2.16301200	-1.56693600
H	-1.34620000	2.22054700	0.61692300
H	-1.22977800	2.93755500	-2.13502100
H	-1.80026100	1.26965800	-2.21035400
C	-3.17383600	2.62761400	-1.28368800
C	-3.81384100	3.51382600	-2.15534400
C	-3.88610100	2.15648900	-0.17667500
C	-5.12718700	3.91716900	-1.93129900
H	-3.27305300	3.89042400	-3.02007900
C	-5.19912900	2.56239200	0.05453100
H	-3.41855300	1.45380100	0.51017300
C	-5.82616400	3.44392700	-0.82207400
H	-5.60346700	4.60697300	-2.62132500
H	-5.73073000	2.18272000	0.92343800
H	-6.84861100	3.76107100	-0.64312500
C	-0.58860900	2.88620500	3.35260100
H	0.21105800	2.56351900	4.02599700
H	-0.11811300	3.19744100	2.41245000
H	-1.07980300	3.75638900	3.78926000
C	-5.27353800	1.02002600	3.70738100
H	-5.40970400	0.66325500	4.73341200
H	-5.58908800	2.06545100	3.67534700
H	-5.93536900	0.43470200	3.06392500
C	-1.61079000	-1.74864600	1.61721600
H	-1.41149400	-1.57411800	0.55290600
H	-0.67947700	-2.12561100	2.04913700
H	-2.38400600	-2.51523300	1.70405000
H	4.66711300	1.82703300	1.59582800
C	3.06279100	2.35191100	-0.38483400
H	2.08580800	2.43215600	-0.86511100
H	3.12608400	3.11242900	0.40113600
C	4.14607500	2.55539000	-1.41909500
C	5.16933200	3.48427500	-1.23004900
C	4.14078600	1.77197300	-2.57900000
C	6.17211200	3.63596900	-2.18661600
H	5.17887500	4.09483300	-0.33082500
C	5.14366900	1.91961700	-3.53174900

H	3.34320900	1.04603100	-2.72716500
C	6.16144400	2.85345900	-3.33778900
H	6.96009100	4.36583800	-2.03079900
H	5.12986400	1.30876600	-4.42867300
H	6.94140200	2.97069000	-4.08309400
C	-5.30453600	-2.46749400	-0.14427000
C	-4.65622300	-1.13893000	-0.53767200
C	-4.44207400	-0.93772200	-2.04757700
C	-4.52067300	-3.71879300	-0.53925100
C	-3.17259900	-1.59040100	-2.54833900
C	-4.22460000	-3.78412500	-2.03986500
H	-6.29761600	-2.51978200	-0.61239200
H	-5.28992600	-0.32447300	-0.16896200
H	-5.31648400	-1.30199700	-2.60204200
H	-3.56558900	-3.76206700	0.00026200
H	-3.97875400	-4.81023300	-2.32948100
C	-1.00784100	-1.48673800	-3.41672900
H	-0.20024600	-0.74965600	-3.40980000
C	-1.80773500	-3.64952100	-2.52134700
C	-0.66213600	-2.65316300	-2.49919600
H	-0.50994500	-2.26561300	-1.48673600
H	0.27100100	-3.13847500	-2.79494400
H	-1.73617000	-4.33364700	-1.66536000
N	-3.09680200	-2.96002900	-2.45196600
N	-2.23647100	-0.82551000	-2.99291500
H	-5.11904000	-3.50949000	-2.61066100
H	-5.09380100	-4.60587100	-0.24723500
H	-5.47671200	-2.47648600	0.93900500
H	-3.68735000	-1.02173300	-0.03356200
H	-4.33455300	0.12733100	-2.26607000
H	-1.11965800	-1.85153900	-4.45005900
H	-1.77320100	-4.26395100	-3.43210200
C	3.39519600	-2.41016300	0.07067400
C	3.01676300	-2.87909200	1.33843000
C	3.99825500	-3.38971100	2.21497800
C	5.30205200	-3.38195600	1.77378500
C	5.66157300	-2.88993000	0.48661300
C	4.72204600	-2.39999900	-0.39129100

H	3.72605800	-3.76787200	3.19517100
H	6.08620400	-3.76258100	2.42018400
H	6.70686400	-2.90792500	0.19542800
H	4.97307200	-2.02147200	-1.37617600
N	1.66988700	-2.70150200	1.48412400
N	1.22264100	-2.16143200	0.37390800
N	2.22690300	-1.98737800	-0.47158100
O	2.06842800	-1.38354800	-1.64364400
H	1.23897800	-0.30009900	-1.44885700

IM4 (M06-2X/6-31G(d,p)/IEF-PCM_{THF})

0 1

C	2.42827300	1.49325700	-0.37521700
C	3.83134700	2.86805800	0.66135400
C	4.82884200	0.65952600	0.01166600
H	4.46514500	-0.27269100	0.45334500
N	2.75430100	3.56793700	0.54362700
N	1.88080200	2.74014500	-0.14605800
N	3.73319000	1.63120500	0.07269200
C	5.05929600	3.21111000	1.44640500
H	4.78081800	3.82737800	2.30162900
H	5.78485900	3.75919100	0.82830000
C	6.01854100	1.19822600	0.83847300
O	5.62287600	2.00781300	1.92570700
C	0.71616800	3.31449600	-0.73674100
C	0.67227600	3.49515700	-2.12559400
C	-0.35539700	3.65720800	0.09326100
C	-0.50520200	3.99536900	-2.67837700
C	-1.50151900	4.18616300	-0.50163800
C	-1.60202900	4.34088700	-1.88539300
H	-0.56433100	4.12682000	-3.75636600
H	-2.33880500	4.47866000	0.12734700
H	6.57984000	0.35542100	1.24421000
C	1.79800300	0.34482900	-0.83762900
O	2.57350100	-0.78021300	-1.08254500
C	0.41658600	0.31404000	-1.18508700
C	-0.22287400	-0.70623100	-1.81169400
H	-0.16367000	1.18726500	-0.90976700

H	0.34364500	-1.59672600	-2.07560500
C	-1.62658000	-0.68457100	-2.19467000
C	-2.18428500	-1.81272400	-2.82635200
C	-2.46886200	0.42613300	-1.98594400
C	-3.50314100	-1.82090500	-3.26314600
H	-1.55317300	-2.68314400	-2.99326100
C	-3.78777500	0.41331100	-2.41753500
H	-2.08990500	1.31906900	-1.49324700
C	-4.31272700	-0.70245600	-3.06807900
H	-3.89976500	-2.70136600	-3.76161300
H	-4.41558800	1.28386300	-2.24824800
H	-5.34002500	-0.69608600	-3.41642100
C	-2.87397000	4.84365100	-2.51734800
H	-2.66479000	5.58110400	-3.29657500
H	-3.41884300	4.01754800	-2.98694000
H	-3.53262800	5.30036900	-1.77524000
C	1.84443300	3.12312500	-2.99226100
H	2.78718900	3.44657700	-2.54015900
H	1.89893400	2.03612000	-3.12080800
H	1.75353000	3.58143500	-3.97837300
C	-0.27583500	3.38637900	1.57049800
H	0.59861600	3.86437700	2.02122600
H	-1.17367100	3.73252400	2.08665400
H	-0.16947700	2.30593600	1.73446100
H	6.68712800	1.77746600	0.18302300
C	5.26991000	0.41501000	-1.44329800
H	4.37848300	0.26444700	-2.05439100
H	5.78671000	1.31148300	-1.80493200
C	6.16517000	-0.79783200	-1.53552900
C	7.55662900	-0.68951700	-1.48684900
C	5.59018900	-2.07008600	-1.62538700
C	8.35892700	-1.82902100	-1.51804800
H	8.01705900	0.29410700	-1.43016900
C	6.38882000	-3.20924700	-1.65569200
H	4.50775800	-2.15295000	-1.68480900
C	7.77675800	-3.09143200	-1.59902100
H	9.43915700	-1.72885600	-1.48197600
H	5.92709700	-4.18935300	-1.72894100

H	8.40102900	-3.97879600	-1.62320300
H	2.63948100	-1.30211700	-0.26018000
C	0.51824700	-2.78719900	0.87588900
C	-0.77990700	-2.47414600	0.64853600
C	-1.42044800	-1.28868100	1.18350700
C	-0.64668000	-0.50452500	2.12385300
C	0.65283100	-0.76353800	2.40888900
C	1.34242600	-1.80255100	1.60673000
H	-1.36796100	-3.11473100	0.00508900
H	-1.16309800	0.25946200	2.69141600
C	-2.74848300	-0.98831300	0.89625900
C	-3.31477800	0.29743000	1.25444300
C	-3.64372700	-1.95796400	0.30080400
C	-4.60149300	0.63626800	1.01795300
H	-2.64392700	1.03270100	1.67996000
C	-4.96244800	-1.72962500	0.09999300
H	-3.25430600	-2.94723400	0.10759200
C	-5.49890800	-0.36435900	0.36658100
O	2.58381700	-1.84106300	1.53197800
O	-6.65787800	-0.07099700	0.07563700
C	1.42229100	-0.05810200	3.52657300
C	2.09375400	-1.11588700	4.42372500
C	2.49466200	0.87482200	2.94076500
C	0.48732700	0.77475400	4.41221900
H	1.34816600	-1.80305700	4.83744500
H	2.83553400	-1.69420100	3.87104400
H	2.59672200	-0.61793600	5.25877700
H	2.02857000	1.65773700	2.33149200
H	3.04801400	1.36056600	3.75248300
H	3.20022200	0.31803700	2.32317700
H	1.06804700	1.21157900	5.22981000
H	0.02277700	1.60184300	3.86581100
H	-0.30515500	0.15922600	4.85026600
C	1.15214300	-4.09720800	0.39819300
C	2.30293000	-3.88512800	-0.60437100
C	1.69520600	-4.83882600	1.63442300
C	0.10915900	-4.99364300	-0.27953200
H	1.98430800	-3.27201700	-1.45322300

H	3.16262900	-3.41052300	-0.12898800
H	2.62322200	-4.85954200	-0.98885900
H	0.88847500	-5.05675400	2.34155000
H	2.14663400	-5.78805000	1.32724200
H	2.45757000	-4.24471800	2.14361700
H	0.57437000	-5.94653500	-0.54710500
H	-0.73653200	-5.20618800	0.38234700
H	-0.27396200	-4.54019900	-1.20052700
C	-5.94156700	-2.84748900	-0.27078300
C	-6.71590900	-2.56624900	-1.57149100
C	-5.21907200	-4.18742000	-0.45266100
C	-6.94404400	-2.99904000	0.89189000
H	-7.18339800	-1.58242100	-1.55672600
H	-6.04959500	-2.63355700	-2.43427900
H	-7.49659200	-3.32580400	-1.68970400
H	-4.72519100	-4.51684500	0.46734700
H	-5.95087800	-4.95158300	-0.73122000
H	-4.47134100	-4.13100700	-1.25178900
H	-7.61799500	-3.83724700	0.68657900
H	-6.41859600	-3.20540600	1.83002300
H	-7.54216000	-2.09461300	1.01478500
C	-5.16028900	2.01638600	1.36123000
C	-5.51713700	2.76017700	0.06093600
C	-6.40358700	1.88935900	2.26213100
C	-4.12838300	2.86251300	2.11353200
H	-4.61409400	2.91640600	-0.54212700
H	-6.24642000	2.20087200	-0.52716600
H	-5.93837800	3.74295500	0.30007000
H	-6.15665100	1.34374700	3.17881400
H	-6.74612900	2.88964400	2.54709700
H	-7.21531800	1.37208300	1.75277900
H	-4.56133400	3.84057000	2.34367200
H	-3.82506500	2.39426300	3.05632100
H	-3.23409200	3.02771400	1.50601700

$IM3_{B3LYP}$ (B3LYP/6-31G(d,p)/IEF-PCM_{THF})

0 1

C	-1.32408600	-1.39945200	0.84827700

C	-2.82584300	-1.30229800	2.44139300
C	-3.76649900	-0.94254800	0.12978100
H	-3.59211400	0.08176900	-0.21397400
N	-1.70425500	-1.55433400	3.05863100
N	-0.76887600	-1.61509200	2.05601700
N	-2.65289000	-1.23585500	1.08130400
C	-4.16697300	-1.06917400	3.07186600
H	-4.04241500	-0.46708000	3.97357700
H	-4.62140200	-2.03179500	3.35319800
C	-5.08172100	-1.01849500	0.92457600
O	-4.99711100	-0.35345000	2.17755900
C	0.60061900	-1.91976200	2.40655700
C	0.88340300	-3.23821000	2.81341600
C	1.57023100	-0.90789500	2.38348900
C	2.19287900	-3.52827000	3.19572200
C	2.86824800	-1.26067900	2.77893500
C	3.20017500	-2.55385300	3.18662600
H	2.43221800	-4.54092800	3.50952200
H	3.63562300	-0.49121400	2.77215900
H	-5.86267300	-0.52076700	0.34937900
C	-0.62226400	-1.28076200	-0.44161400
O	-1.04694700	-0.32948000	-1.29358400
C	0.35753300	-2.14820700	-0.77053700
C	1.12147000	-2.04645800	-2.06418100
H	0.59905600	-2.95265000	-0.08570900
H	0.45165200	-2.25270900	-2.91005100
H	1.43843300	-0.99892200	-2.18683300
C	2.32946800	-2.95848000	-2.14245500
C	2.53906300	-3.77867500	-3.25856900
C	3.28111900	-2.98433500	-1.11065400
C	3.66562500	-4.59977100	-3.34725500
H	1.81189400	-3.77374500	-4.06681500
C	4.40681700	-3.80549500	-1.19426500
H	3.13828400	-2.35719900	-0.23447300
C	4.60414900	-4.61684200	-2.31422600
H	3.80663400	-5.22825200	-4.22214100
H	5.13076500	-3.81074700	-0.38408800
H	5.47974500	-5.25608000	-2.38009900

C	-0.17990800	-4.30977600	2.84289700
H	-0.94224600	-4.09274700	3.59767700
H	-0.69654500	-4.39833800	1.88082700
H	0.26376700	-5.27990500	3.07524700
C	4.60286400	-2.89877700	3.62512800
H	4.63733200	-3.10754800	4.70065000
H	4.96863800	-3.79471300	3.11289400
H	5.29819900	-2.08091900	3.42113300
C	1.28180400	0.49884800	1.92614900
H	1.46292500	0.59574500	0.84814600
H	0.25197700	0.80364700	2.12146600
H	1.94874200	1.20190300	2.43222100
H	-5.37630200	-2.06900800	1.07339500
C	-3.78930800	-1.92411400	-1.05897200
H	-2.80714900	-1.92903700	-1.53415000
H	-3.98650600	-2.93557100	-0.68759400
C	-4.83040200	-1.53214800	-2.09156900
C	-5.95080900	-2.33730400	-2.33151300
C	-4.67590700	-0.35164300	-2.83521300
C	-6.89771100	-1.97548800	-3.29290000
H	-6.08048000	-3.25676000	-1.76632900
C	-5.62279100	0.01292000	-3.79214500
H	-3.81026800	0.28187000	-2.66106200
C	-6.73666300	-0.79842600	-4.02481700
H	-7.75876400	-2.61391100	-3.46819500
H	-5.48864700	0.92907500	-4.35979700
H	-7.47171900	-0.51512800	-4.77233500
C	6.58585200	1.91686700	-0.18426900
C	5.45385000	0.88146900	-0.23255600
C	4.68549700	0.81944000	-1.57072200
C	6.15097500	3.37017500	-0.41062900
C	3.60357500	1.87059800	-1.73189400
C	5.41152500	3.59626400	-1.73979300
H	7.33092400	1.65331600	-0.94878000
H	5.88613400	-0.10846000	-0.04415800
H	5.39893200	0.87626200	-2.40441600
H	5.50780100	3.71131600	0.41102900
H	5.42279900	4.66165300	-1.98621300

C	1.34077500	2.46699300	-2.00151500
H	0.38309500	2.04552500	-1.68193100
C	3.01598100	4.27511900	-1.66995500
C	1.65754600	3.74618600	-1.22474700
H	1.68384100	3.51715000	-0.15282200
H	0.89106400	4.51280800	-1.37548200
H	3.38045800	5.03407700	-0.96698700
N	4.00386900	3.19004900	-1.73430400
N	2.38110300	1.45175200	-1.83457100
H	5.94743800	3.09255200	-2.55412300
H	7.04219400	4.01047000	-0.39857500
H	7.10059600	1.84201000	0.78156100
H	4.73920900	1.06479200	0.58072700
H	4.17475600	-0.14061800	-1.65851700
H	1.23169300	2.70871600	-3.07148800
H	2.93405500	4.76179300	-2.65331600
C	-2.03113800	3.75507000	0.65038100
C	-1.91834500	3.91956900	2.04857300
C	-1.83214700	5.21903300	2.59378700
C	-1.86492200	6.28588200	1.71391600
C	-1.98186200	6.09601800	0.30763200
C	-2.06791600	4.83278500	-0.25038900
H	-1.74511900	5.36652400	3.66574800
H	-1.80195500	7.29931700	2.09908500
H	-2.00629800	6.96845900	-0.33853800
H	-2.16279700	4.67121800	-1.31833800
N	-1.91453800	2.68671300	2.64106600
N	-2.02529600	1.78214900	1.67733700
N	-2.08863600	2.40340600	0.48690400
O	-2.22719100	1.75329300	-0.66359000
H	-1.46383500	0.53296500	-0.87256400

$TS4_{B3LYP}$ (B3LYP/6-31G(d,p)/IEF-PCM_{THF})

0 1

C	-0.89611100	-1.14932000	1.10614200
C	-2.45751200	-0.99186300	2.67059100
C	-3.37774200	-1.21137000	0.33402800
H	-3.30195100	-0.34046500	-0.32194100

N	-1.34129700	-1.02375300	3.33525100
N	-0.36449400	-1.13308000	2.36265200
N	-2.25864200	-1.10150400	1.31344800
C	-3.83337100	-0.79092100	3.23512900
H	-3.79193100	-0.06186500	4.04650500
H	-4.22730800	-1.73755800	3.63647800
C	-4.70857100	-1.17069400	1.11676600
O	-4.67911400	-0.27767200	2.22190600
C	1.00663700	-1.19414700	2.79500600
C	1.55565100	-2.45206500	3.09471300
C	1.71814900	0.00331900	2.96757000
C	2.87703200	-2.49096400	3.54918700
C	3.03625200	-0.09324300	3.42524900
C	3.63283200	-1.32462800	3.71924200
H	3.32209900	-3.45489600	3.78134300
H	3.60698400	0.82125500	3.56395500
H	-5.49577900	-0.82613000	0.44571600
C	-0.22715200	-1.13256300	-0.16863600
O	-0.95455500	-0.74070900	-1.25999500
C	1.05165900	-1.60572900	-0.34795600
C	1.73248200	-1.62258700	-1.61477900
H	1.57492200	-1.96925500	0.52802200
H	1.03973200	-1.64956000	-2.46024400
H	2.15303300	-0.33493400	-1.84364800
C	2.92421000	-2.48829500	-1.81873500
C	3.23660300	-2.93975300	-3.11717500
C	3.81532500	-2.84275700	-0.78478100
C	4.37007500	-3.71019300	-3.37102800
H	2.56906000	-2.68336300	-3.93649800
C	4.94784100	-3.61831000	-1.03743100
H	3.62811700	-2.51000200	0.23256700
C	5.23579100	-4.05915800	-2.33101500
H	4.57460500	-4.04442900	-4.38488200
H	5.61024900	-3.87789900	-0.21555100
H	6.11777200	-4.66231300	-2.52499300
C	0.75082400	-3.71845900	2.93148200
H	-0.16335400	-3.68725400	3.53324600
H	0.44705900	-3.87160600	1.88975700

H	1.33376700	-4.58823400	3.24127600
C	5.04681500	-1.39132600	4.24460000
H	5.52331800	-2.34196000	3.99011700
H	5.66053200	-0.57991700	3.84332200
H	5.06143400	-1.30051400	5.33758000
C	1.08418800	1.34089800	2.68703000
H	0.72658700	1.42368200	1.65643100
H	0.21452000	1.50556900	3.33188300
H	1.79582300	2.14944700	2.86872700
H	-4.97092400	-2.18105900	1.46685800
C	-3.29099000	-2.51731200	-0.48683600
H	-2.30227700	-2.57207300	-0.94339300
H	-3.40154300	-3.37137800	0.19066100
C	-4.34545600	-2.57341000	-1.57590000
C	-5.40449400	-3.48829900	-1.51666200
C	-4.26869800	-1.70435100	-2.67615500
C	-6.36638000	-3.53672800	-2.52879200
H	-5.47372300	-4.17343100	-0.67545200
C	-5.23030000	-1.74853000	-3.68539700
H	-3.44813900	-0.99504700	-2.73803100
C	-6.28293900	-2.66538600	-3.61549300
H	-7.17819900	-4.25578000	-2.46686100
H	-5.15514500	-1.06980600	-4.53012600
H	-7.02949200	-2.70116200	-4.40344300
C	5.77365000	2.72301300	0.17661700
C	4.90214800	1.46048700	0.15075800
C	4.59000800	0.92030700	-1.26266000
C	5.17742300	3.93912600	-0.54269400
C	3.45363400	1.63026700	-1.96619700
C	4.84517500	3.68366300	-2.02029700
H	6.74466400	2.48688900	-0.28103000
H	5.42282100	0.66837000	0.70031600
H	5.49152500	0.97474000	-1.88638600
H	4.26888000	4.28175400	-0.03183000
H	4.73567200	4.63559300	-2.54452000
C	1.25169800	1.55888500	-2.93231300
H	0.34958300	1.01005200	-2.64877300
C	2.46511600	3.73080000	-2.79346000

C	1.13468500	3.03065100	-2.54426100
H	0.85695600	3.09949500	-1.48784800
H	0.34843900	3.52338400	-3.12361900
H	2.47413300	4.71464000	-2.31371800
N	3.58805800	2.95143700	-2.24251600
N	2.38769600	0.93296500	-2.25788200
H	5.67302500	3.15756300	-2.51009400
H	5.89667800	4.76564900	-0.49514300
H	5.98371700	2.99084800	1.21891900
H	3.96029500	1.63631300	0.68492700
H	4.32041000	-0.13458300	-1.20520100
H	1.37184100	1.45638400	-4.02041600
H	2.62948000	3.88887100	-3.86802600
C	-3.21712600	3.15781500	0.01066000
C	-2.61476700	3.96402100	1.00194500
C	-3.38212700	4.95072500	1.65890600
C	-4.70951200	5.08040100	1.29133700
C	-5.29513600	4.25612800	0.28868000
C	-4.56366100	3.28327400	-0.36886200
H	-2.93867500	5.58083600	2.42381100
H	-5.32833600	5.82955400	1.77659100
H	-6.34204700	4.39951300	0.03800200
H	-4.99265500	2.64869500	-1.13640800
N	-1.30557100	3.59510600	1.13713500
N	-1.07936100	2.60884800	0.27611000
N	-2.20821600	2.33703300	-0.40068500
O	-2.28405100	1.40439800	-1.33932000
H	-1.39485300	0.18135300	-1.16299900

IM4$_{\mathbf{B3LYP}}$ (B3LYP/6-31G(d,p)/IEF-PCM$_{THF}$)

0 1

C	4.36280700	-0.11339100	0.65957700
C	5.38156100	-0.85068900	2.50940200
C	4.19757700	-2.64378100	1.21396500
H	3.11344800	-2.69465800	1.11893900
N	5.79611900	0.37063300	2.40029000
N	5.21027400	0.83501700	1.22071700
N	4.55265200	-1.23425600	1.47916800

C	5.66119100	-1.80107900	3.63691800
H	5.61839100	-1.27016000	4.59008100
H	6.66252700	-2.24782000	3.53382800
C	4.66993900	-3.50839500	2.41114100
O	4.66098100	-2.80775500	3.64816400
C	5.53441900	2.15942600	0.79102100
C	6.47787600	2.33274700	-0.23669700
C	4.94107900	3.25357500	1.44604300
C	6.79518300	3.63924100	-0.62325900
C	5.30092400	4.53876100	1.03272200
C	6.21796800	4.75318800	-0.00394700
H	7.52042300	3.78839800	-1.41917600
H	4.85073200	5.39391900	1.53098000
H	3.99997400	-4.36187600	2.52196500
C	3.45740100	-0.00394500	-0.39325600
O	2.62113500	-1.10210700	-0.61456000
C	3.32762900	1.11691800	-1.26540400
C	2.44321100	1.19660200	-2.30352700
H	3.98113600	1.95883400	-1.06961400
H	1.80677300	0.33719600	-2.49735100
C	2.26886200	2.32516700	-3.20741100
C	1.31594100	2.22923500	-4.24712500
C	2.99901200	3.53378000	-3.11565000
C	1.10280900	3.27397300	-5.14306500
H	0.73837300	1.31295300	-4.34355900
C	2.78440400	4.57619600	-4.01200300
H	3.74313700	3.65913400	-2.33465100
C	1.83542000	4.45873100	-5.03391400
H	0.36147800	3.16287400	-5.93009600
H	3.36279000	5.49123900	-3.91341400
H	1.67175400	5.27555500	-5.73045900
C	6.56154600	6.15518600	-0.44804200
H	7.48689900	6.17519300	-1.02966400
H	5.76683300	6.57377500	-1.07727400
H	6.68012700	6.82627400	0.40830700
C	7.12270100	1.14534600	-0.90704600
H	7.58458400	0.47869300	-0.17159800
H	6.38614600	0.55203300	-1.46070900

H	7.89372000	1.46763200	-1.61050500
C	3.94619500	3.04486200	2.56046600
H	4.40162700	2.51393800	3.40246800
H	3.56271300	4.00188900	2.92179400
H	3.09679000	2.44048900	2.22462000
H	5.68344700	-3.89168800	2.21853900
C	4.84822000	-3.14114000	-0.10001400
H	4.60666600	-2.42724400	-0.88959500
H	5.93655400	-3.13743500	0.03027200
C	4.37343500	-4.52155200	-0.50493700
C	5.16697600	-5.65847900	-0.30040900
C	3.11157600	-4.68318200	-1.09831700
C	4.71148700	-6.92614400	-0.67083900
H	6.15371900	-5.55084300	0.14297700
C	2.65437100	-5.94803200	-1.46861500
H	2.49717000	-3.80545300	-1.27735300
C	3.45227900	-7.07506300	-1.25405600
H	5.34306500	-7.79472900	-0.50774300
H	1.67806100	-6.05406300	-1.93326600
H	3.09790300	-8.05936200	-1.54570000
H	1.73515100	-0.90658600	-0.24790400
C	-2.09117500	-1.53898700	-0.30814400
C	-3.43413900	-1.31148200	-0.30207900
C	-4.05867400	-0.18517600	0.34937300
C	-3.17561900	0.73273100	1.02803000
C	-1.82150600	0.59457400	1.07071300
C	-1.20323000	-0.57028400	0.38580600
H	-4.07131700	-2.02272600	-0.80311700
H	-3.61468500	1.58513700	1.52134100
C	-5.45597600	0.00749000	0.32564700
C	-6.08283400	1.10479600	1.02749800
C	-6.33434700	-0.88131700	-0.40115700
C	-7.42335000	1.33539200	1.03132100
H	-5.44957800	1.77281200	1.58986300
C	-7.68511900	-0.73114800	-0.45957400
H	-5.88917500	-1.70480000	-0.93723100
C	-8.30918100	0.41238000	0.26601000
O	0.03342400	-0.73553700	0.40962700

O	-9.53633500	0.59119800	0.23420400
C	-0.92797400	1.61346500	1.80191700
C	-0.11062700	0.90664000	2.91256900
C	0.02473800	2.29437500	0.78912300
C	-1.75583300	2.72580600	2.47728900
H	-0.77638600	0.43853300	3.64621400
H	0.54513600	0.14050700	2.50054900
H	0.50293200	1.64419100	3.44159200
H	-0.54448300	2.82978600	0.02148800
H	0.65254200	3.02644300	1.30951400
H	0.67586100	1.57520300	0.29362300
H	-1.07563900	3.41506200	2.98690200
H	-2.33068400	3.31207500	1.75260800
H	-2.44638700	2.33021600	3.22978400
C	-1.48508900	-2.76876700	-1.00844700
C	-0.53603400	-2.31673100	-2.14681700
C	-0.71359400	-3.63500200	0.01876300
C	-2.56878900	-3.66372800	-1.64390000
H	-1.07481700	-1.70751600	-2.88067800
H	0.30693200	-1.73943600	-1.77003300
H	-0.14221000	-3.19625900	-2.66840600
H	-1.38647300	-3.99262400	0.80585600
H	-0.28870600	-4.51179900	-0.48176500
H	0.09792500	-3.07630900	0.48425500
H	-2.08601900	-4.52321100	-2.11853800
H	-3.27289500	-4.05273500	-0.90073300
H	-3.13743000	-3.13956400	-2.41948300
C	-8.58202100	-1.69787200	-1.25186200
C	-9.33124500	-0.92620100	-2.36778800
C	-7.76686100	-2.81962700	-1.92545900
C	-9.60392900	-2.36936100	-0.29942000
H	-9.96561800	-0.14398300	-1.95151500
H	-8.62233800	-0.46744400	-3.06576900
H	-9.95968500	-1.62066100	-2.93616400
H	-7.22954800	-3.43453100	-1.19554700
H	-8.44968000	-3.47909700	-2.46974500
H	-7.04319200	-2.42899300	-2.64880000
H	-10.22557200	-3.07282000	-0.86441700

H	-9.08994900	-2.93377100	0.48639100
H	-10.25349100	-1.63089600	0.16988600
C	-8.04760800	2.51313900	1.79864000
C	-8.78420300	3.45412300	0.81098200
C	-9.04344000	1.98346000	2.86164300
C	-6.98317800	3.35109300	2.53435200
H	-8.08849200	3.85867600	0.06765300
H	-9.58681100	2.93307200	0.28945300
H	-9.21482100	4.29829600	1.36073600
H	-8.53307300	1.33569300	3.58290900
H	-9.47318800	2.82639500	3.41385000
H	-9.85500800	1.42040900	2.40111900
H	-7.47718000	4.17298500	3.06115900
H	-6.43856400	2.76368400	3.28130500
H	-6.25627100	3.79468800	1.84556000

TS5$_{\mathbf{B3LYP}}$ (B3LYP/6-31G(d,p)/IEF-PCM$_{THF}$)

0 1

C	3.63957800	1.20248500	-0.05957900
C	5.58002600	1.45574900	0.98618400
C	5.04490100	-0.93728900	0.45195600
H	4.18189000	-1.44115200	0.88574500
N	5.15857500	2.67140000	0.79719200
N	3.96688000	2.52543400	0.11698600
N	4.73298600	0.51675900	0.45152900
C	6.77115800	1.03586400	1.79200600
H	6.93169900	1.74955000	2.60168200
H	7.67887400	0.99865600	1.17062200
C	6.30682800	-1.18819100	1.32633600
O	6.49094500	-0.23276100	2.36021900
C	3.32326700	3.72666600	-0.34309400
C	3.43159500	4.07809600	-1.69941700
C	2.66156700	4.53635800	0.59640600
C	2.82066200	5.26672200	-2.10972100
C	2.07329700	5.71582500	0.13195100
C	2.13983900	6.09823800	-1.21350400
H	2.88678500	5.55187200	-3.15628600
H	1.54995400	6.35154400	0.84131000

H	6.21115800	-2.16312700	1.80489500
C	2.42415100	0.61637500	-0.55155500
O	2.40968600	-0.70109600	-0.67653800
C	1.29377300	1.41638200	-0.90120800
C	0.11322100	0.90003000	-1.36638400
H	1.38185500	2.48556500	-0.76493000
H	0.04325100	-0.17878300	-1.47502800
C	-1.07510900	1.64913300	-1.72719500
C	-2.22939500	0.93839100	-2.13084400
C	-1.15224200	3.06263300	-1.69943300
C	-3.40033200	1.60089200	-2.48705500
H	-2.19538700	-0.14687800	-2.15247600
C	-2.32381200	3.72006200	-2.05336000
H	-0.28807600	3.64925400	-1.40395000
C	-3.45659200	2.99635700	-2.44904100
H	-4.27300800	1.02788100	-2.78569500
H	-2.35858100	4.80538100	-2.02428200
H	-4.36857300	3.51614100	-2.72613500
C	1.52109700	7.39469400	-1.67719700
H	0.60297600	7.61819900	-1.12634200
H	2.20867500	8.23379600	-1.51556500
H	1.28467700	7.36616700	-2.74409000
C	4.16600200	3.20651000	-2.68758000
H	5.16678900	2.94739100	-2.32728400
H	3.63048700	2.26753600	-2.86754700
H	4.27184200	3.71873600	-3.64600400
C	2.58117900	4.15197200	2.05286200
H	3.57463200	4.13659600	2.51228700
H	1.95994000	4.86124200	2.60358000
H	2.15319700	3.15266400	2.18345900
H	7.20003200	-1.20682100	0.68504600
C	5.29560000	-1.45749600	-0.98170100
H	4.41218600	-1.23553000	-1.58214300
H	6.14391800	-0.90905600	-1.40586600
C	5.57544900	-2.94814900	-1.01098200
C	6.81767100	-3.44237500	-1.42958800
C	4.58387700	-3.86414700	-0.62487700
C	7.06659000	-4.81654700	-1.46553000

H	7.59437400	-2.74677900	-1.73728100
C	4.83199800	-5.23637500	-0.65634400
H	3.61264800	-3.50046300	-0.30151000
C	6.07458200	-5.71780100	-1.07730300
H	8.03489400	-5.18022600	-1.79707400
H	4.05191900	-5.92993500	-0.35611800
H	6.26602000	-6.78642800	-1.10404300
H	1.84628500	-1.37279900	0.08514200
C	-0.78448600	-2.85938700	0.00177700
C	-2.12605300	-2.52839900	-0.04334700
C	-2.72091400	-1.56871400	0.82098400
C	-1.90736300	-1.08185100	1.88246200
C	-0.56668300	-1.38700200	2.00653100
C	0.05129600	-2.15989900	0.95312900
H	-2.74490400	-2.98275600	-0.80363900
H	-2.37769100	-0.47292200	2.64128700
C	-4.07547300	-1.11339300	0.63478100
C	-4.53512500	0.09611300	1.24033600
C	-5.00867900	-1.82920700	-0.17697500
C	-5.79778700	0.60155800	1.06275900
H	-3.82449000	0.66126400	1.82731000
C	-6.29760600	-1.41384000	-0.39427700
H	-4.68692400	-2.76847700	-0.60507100
C	-6.75324400	-0.14532100	0.21692200
O	1.36126200	-2.20478000	0.85645900
O	-7.91679100	0.28295100	0.02286200
C	0.24800400	-0.91695600	3.23152900
C	0.96821100	-2.12914400	3.87192300
C	1.29315100	0.15197000	2.83228200
C	-0.65528300	-0.29307200	4.31574400
H	0.24236000	-2.87494800	4.21414300
H	1.64739300	-2.60495400	3.16366600
H	1.54761500	-1.80100200	4.74242800
H	0.80909700	0.99997800	2.33669200
H	1.80431000	0.52678200	3.72690300
H	2.04101600	-0.26219000	2.15769600
H	-0.04451600	-0.04246600	5.18880400
H	-1.13363200	0.63209900	3.97758300

H	-1.43779300	-0.98413600	4.64595300
C	-0.20499500	-3.93849400	-0.94150400
C	0.68847100	-3.30985400	-2.03718100
C	0.60863600	-4.96566900	-0.11602900
C	-1.32135800	-4.72559400	-1.66188900
H	0.11015000	-2.60932200	-2.65018400
H	1.53707400	-2.77047100	-1.62046600
H	1.07031400	-4.09462400	-2.70071300
H	-0.03554600	-5.47293100	0.61084400
H	1.02762300	-5.72894800	-0.78151500
H	1.42511200	-4.48762500	0.42505100
H	-0.86888300	-5.53305300	-2.24625500
H	-2.02488600	-5.17950400	-0.95616400
H	-1.88831200	-4.10175800	-2.36050800
C	-7.28049400	-2.23493000	-1.24833700
C	-7.73869600	-1.39989900	-2.47072200
C	-6.64548900	-3.53398600	-1.78354400
C	-8.51404300	-2.63442800	-0.39987200
H	-8.22963800	-0.48090700	-2.15113800
H	-6.88295700	-1.13857400	-3.10381500
H	-8.44006900	-1.98245000	-3.07956000
H	-6.32572500	-4.20056700	-0.97530100
H	-7.38512900	-4.07543900	-2.38208600
H	-5.78216900	-3.33814100	-2.42868300
H	-9.21548900	-3.21357200	-1.01165100
H	-8.21436200	-3.25954100	0.44877900
H	-9.02730900	-1.75161100	-0.01905700
C	-6.23610100	1.93015800	1.70458900
C	-6.62825400	2.94658200	0.60255200
C	-7.44342800	1.68775900	2.64525700
C	-5.11271200	2.56929300	2.54511200
H	-5.77629100	3.14560200	-0.05713500
H	-7.45532800	2.56996600	0.00064300
H	-6.92964100	3.89622900	1.06022900
H	-7.17170500	1.00052900	3.45448300
H	-7.75777400	2.63416300	3.10073400
H	-8.28528800	1.26496600	2.09717700
H	-5.48008300	3.50198900	2.98529800

H	-4.79251100	1.92127600	3.36827000
H	-4.23382400	2.81544500	1.93970900

IM3$_{explicit}$ (ONIOM(M06-2X/6-31G(d, p):UFF))

0 1 0 1 0 1

C-C_3	0	-5.40277000	7.22999700	-4.73228200	L
O-O_3	0	-6.33183700	6.83673900	-3.74821500	L
C-C_3	0	-6.99328300	7.99557800	-3.30364000	L
C-C_3	0	-6.14622600	9.19666400	-3.65218900	L
C-C_3	0	-5.43073600	8.73975300	-4.88446200	L
H-H_	0	-4.39128700	6.90227800	-4.41482900	L
H-H_	0	-5.64736800	6.73494900	-5.69666700	L
H-H_	0	-7.98109100	8.06505800	-3.81006500	L
H-H_	0	-7.16301500	7.93631300	-2.20908000	L
H-H_	0	-6.76309700	10.10240100	-3.83787600	L
H-H_	0	-5.41416900	9.39281700	-2.83885500	L
H-H_	0	-6.01240200	9.02163100	-5.78993600	L
H-H_	0	-4.40985200	9.16820900	-4.94740500	L
C-C_3	0	-8.44112000	3.03236400	-11.83363500	L
O-O_3	0	-9.21076100	4.21228800	-11.86301300	L
C-C_3	0	-8.31471400	5.28571700	-12.00454600	L
C-C_3	0	-6.97317100	4.86940200	-11.44852000	L
C-C_3	0	-6.97564900	3.37988000	-11.62401100	L
H-H_	0	-8.80462500	2.38695500	-11.00645400	L
H-H_	0	-8.57467800	2.48117300	-12.78953700	L
H-H_	0	-8.22015200	5.54238800	-13.08284900	L
H-H_	0	-8.70190000	6.17264900	-11.45947400	L
H-H_	0	-6.13627200	5.35070900	-11.99939200	L
H-H_	0	-6.91213100	5.12903900	-10.36892200	L
H-H_	0	-6.37928900	3.09456800	-12.51862900	L
H-H_	0	-6.56321200	2.87232400	-10.72748000	L
C-C_3	0	7.33268600	-3.38808200	8.11303100	L
O-O_3	0	6.14910000	-2.96335000	7.48503500	L
C-C_3	0	5.44289200	-2.21841000	8.44307800	L
C-C_3	0	6.44157700	-1.57370400	9.38125700	L
C-C_3	0	7.69054400	-2.39529600	9.20440800	L
H-H_	0	7.17433000	-4.40055900	8.54566200	L
H-H_	0	8.14949700	-3.44890700	7.36368400	L

H-H_	0	4.82665600	-1.44760100	7.93576800	L
H-H_	0	4.76690000	-2.89443200	9.01054700	L
H-H_	0	6.62756800	-0.52093800	9.07992800	L
H-H_	0	6.07832500	-1.60070200	10.43175000	L
H-H_	0	8.53809800	-1.75027500	8.88484800	L
H-H_	0	7.96117100	-2.92352500	10.14464500	L
C-C_3	0	-5.61911500	-7.12718700	5.81119500	L
O-O_3	0	-6.43429800	-7.64011600	4.78931700	L
C-C_3	0	-7.67052800	-6.97229100	4.88673800	L
C-C_3	0	-7.78158900	-6.30433300	6.24961800	L
C-C_3	0	-6.51127400	-6.69112600	6.94707400	L
H-H_	0	-4.90486000	-7.90478400	6.15476300	L
H-H_	0	-5.04627000	-6.25872700	5.41885400	L
H-H_	0	-7.74458800	-6.21654400	4.07526900	L
H-H_	0	-8.48988900	-7.71034500	4.75796200	L
H-H_	0	-7.83421300	-5.20052400	6.13765900	L
H-H_	0	-8.67277500	-6.66312400	6.80942300	L
H-H_	0	-6.07079900	-5.84210400	7.50852400	L
H-H_	0	-6.69597700	-7.54646800	7.63376200	L
C-C_3	0	-3.79220200	3.05581600	5.91277800	L
O-O_3	0	-3.74934200	1.82298600	6.58992700	L
C-C_3	0	-4.16850900	0.82384500	5.69057800	L
C-C_3	0	-4.30638000	1.41544600	4.30222300	L
C-C_3	0	-4.46969400	2.87451900	4.57377800	L
H-H_	0	-4.33400800	3.80216100	6.52657000	L
H-H_	0	-2.75576600	3.41707500	5.76385300	L
H-H_	0	-3.43490100	-0.00887100	5.69168000	L
H-H_	0	-5.14698900	0.42658500	6.03059200	L
H-H_	0	-3.38050100	1.23835200	3.71438000	L
H-H_	0	-5.18237100	1.00276800	3.75623600	L
H-H_	0	-3.99385300	3.49931000	3.78908100	L
H-H_	0	-5.55011100	3.12007000	4.65560600	L
C-C_3	0	0.17778200	-6.04843100	-5.95441400	L
O-O_3	0	-0.93315800	-5.19474900	-5.87468400	L
C-C_3	0	-0.76219700	-4.46957300	-4.68587200	L
C-C_3	0	-0.06345100	-5.36689900	-3.68521300	L
C-C_3	0	0.60041100	-6.40891300	-4.54361100	L
H-H_	0	-0.08998500	-6.95942000	-6.52697500	L

H-H_	0	1.00318100	-5.52448800	-6.48462400	L
H-H_	0	-0.14228200	-3.57173800	-4.89480200	L
H-H_	0	-1.74703200	-4.13658200	-4.30001900	L
H-H_	0	0.67285700	-4.80004000	-3.07823500	L
H-H_	0	-0.80241000	-5.84609500	-3.00988000	L
H-H_	0	1.70549800	-6.37846900	-4.44057800	L
H-H_	0	0.22249200	-7.41755500	-4.27396100	L
C-C_3	0	-6.03681400	-9.75677600	-0.42074300	L
O-O_3	0	-5.77824000	-10.05745000	0.93146100	L
C-C_3	0	-6.01838800	-11.43075400	1.11823700	L
C-C_3	0	-6.02224600	-12.11681900	-0.22800400	L
C-C_3	0	-6.42758300	-11.02109100	-1.16228000	L
H-H_	0	-6.84601800	-9.00062200	-0.48240600	L
H-H_	0	-5.12259300	-9.31921800	-0.87278200	L
H-H_	0	-5.23215200	-11.86382900	1.77156200	L
H-H_	0	-7.00362900	-11.56101800	1.61619200	L
H-H_	0	-4.99820800	-12.47207200	-0.47794100	L
H-H_	0	-6.73570800	-12.96891500	-0.25472500	L
H-H_	0	-5.90241700	-11.10536600	-2.13770400	L
H-H_	0	-7.52773200	-11.04698300	-1.32209800	L
C-C_3	0	7.68426800	-5.15269100	-7.55415700	L
O-O_3	0	7.71635700	-5.11966000	-8.96328000	L
C-C_3	0	8.86124400	-4.39331300	-9.34662600	L
C-C_3	0	9.41445100	-3.66681800	-8.14129100	L
C-C_3	0	8.94064600	-4.50963400	-7.00534700	L
H-H_	0	6.79050800	-4.59426300	-7.20113300	L
H-H_	0	7.60406800	-6.20438200	-7.20685300	L
H-H_	0	9.62096600	-5.10223400	-9.74194900	L
H-H_	0	8.60083800	-3.67327900	-10.14969800	L
H-H_	0	10.52352600	-3.59954400	-8.16835500	L
H-H_	0	8.97032200	-2.65058900	-8.06809500	L
H-H_	0	9.70086600	-5.28847400	-6.77741900	L
H-H_	0	8.73272300	-3.90247900	-6.10011000	L
C-C_3	0	9.34838700	4.40088600	-1.17682000	L
O-O_3	0	9.42813700	3.50771400	-2.26496000	L
C-C_3	0	8.17937100	3.50945500	-2.91490600	L
C-C_3	0	7.16048200	4.19644500	-2.03369000	L
C-C_3	0	7.99593300	5.08263700	-1.16839300	L

H-H_	0	9.49127600	3.82887700	-0.23675000	L
H-H_	0	10.15694300	5.15616300	-1.25225900	L
H-H_	0	8.27719200	4.05053300	-3.87935100	L
H-H_	0	7.86546900	2.46620900	-3.13067800	L
H-H_	0	6.42087800	4.77194600	-2.62656500	L
H-H_	0	6.63882900	3.44849600	-1.40404800	L
H-H_	0	8.07202200	6.09434400	-1.61999200	L
H-H_	0	7.58310000	5.16539900	-0.14032200	L
C-C_3	0	9.76703900	-5.04756500	-2.42449100	L
O-O_3	0	9.71319400	-5.16339600	-3.82162800	L
C-C_3	0	8.90427700	-6.27978900	-4.10706700	L
C-C_3	0	8.74081900	-7.12755200	-2.85808800	L
C-C_3	0	9.56121100	-6.41799500	-1.82456000	L
H-H_	0	10.75071900	-4.63575500	-2.12009700	L
H-H_	0	8.96836600	-4.35234900	-2.08654900	L
H-H_	0	7.91782700	-5.92676200	-4.47008300	L
H-H_	0	9.37923200	-6.87892700	-4.91230500	L
H-H_	0	7.67568300	-7.15420000	-2.54827400	L
H-H_	0	9.10649200	-8.16474600	-3.02018500	L
H-H_	0	9.03249600	-6.36108900	-0.85204100	L
H-H_	0	10.53912900	-6.93004700	-1.69272600	L
C-C_3	0	-3.82374800	-9.75203000	3.80145300	L
O-O_3	0	-2.91966000	-10.09588200	2.77716700	L
C-C_3	0	-2.34200900	-11.33644900	3.11091200	L
C-C_3	0	-2.70247600	-11.69124800	4.53777600	L
C-C_3	0	-3.94829700	-10.90066100	4.77931100	L
H-H_	0	-3.44527700	-8.84872000	4.32146800	L
H-H_	0	-4.81474500	-9.51244600	3.36429200	L
H-H_	0	-2.73265200	-12.11239700	2.41762300	L
H-H_	0	-1.23933700	-11.28128000	2.98976900	L
H-H_	0	-2.87424100	-12.78245300	4.66210800	L
H-H_	0	-1.90161700	-11.35488600	5.23052800	L
H-H_	0	-4.84261300	-11.51481300	4.53207900	L
H-H_	0	-4.01303900	-10.54294900	5.83012300	L
C-C_3	0	-4.80679100	-7.63816900	-6.49728500	L
O-O_3	0	-5.59511800	-8.21913100	-5.48427000	L
C-C_3	0	-4.88867600	-9.32867600	-4.99018700	L
C-C_3	0	-3.41830200	-9.12918000	-5.26847300	L

C-C_3	0	-3.41228400	-8.24188200	-6.47559900	L
H-H_	0	-5.28936800	-7.81077000	-7.48329300	L
H-H_	0	-4.74962800	-6.54283100	-6.32537500	L
H-H_	0	-5.06622900	-9.43094900	-3.89886500	L
H-H_	0	-5.25309800	-10.24802100	-5.49895100	L
H-H_	0	-2.93421700	-8.60766900	-4.41554800	L
H-H_	0	-2.90669100	-10.09697000	-5.45922600	L
H-H_	0	-2.63159100	-7.45602800	-6.38882100	L
H-H_	0	-3.23652800	-8.84300000	-7.39486300	L
C-C_3	0	5.24061800	8.63858700	4.94767200	L
O-O_3	0	6.58878100	8.55104200	4.55369300	L
C-C_3	0	7.31057500	9.51996300	5.27976700	L
C-C_3	0	6.36166200	10.31134500	6.15824600	L
C-C_3	0	5.15185000	9.43636400	6.22875300	L
H-H_	0	4.66023900	9.14175700	4.14533200	L
H-H_	0	4.82759300	7.61990200	5.09209100	L
H-H_	0	8.06787900	9.00418300	5.90822700	L
H-H_	0	7.83935200	10.19719900	4.57629200	L
H-H_	0	6.79115200	10.50113700	7.16597600	L
H-H_	0	6.10440000	11.27685500	5.66881400	L
H-H_	0	5.22577100	8.75973800	7.10703900	L
H-H_	0	4.21368200	10.02714800	6.28793000	L
C-C_3	0	-8.06932200	-5.97369000	-6.21701300	L
O-O_3	0	-8.11083000	-5.37601700	-7.49341000	L
C-C_3	0	-9.29862000	-4.62453700	-7.57356200	L
C-C_3	0	-9.85070000	-4.42450500	-6.18053500	L
C-C_3	0	-9.31904900	-5.60843300	-5.43851200	L
H-H_	0	-7.98989700	-7.07543300	-6.32914200	L
H-H_	0	-7.16999900	-5.60712400	-5.67806800	L
H-H_	0	-9.09105000	-3.64512900	-8.05364400	L
H-H_	0	-10.03407800	-5.17757500	-8.19766300	L
H-H_	0	-9.43920000	-3.49056500	-5.74424400	L
H-H_	0	-10.96180600	-4.39193300	-6.17355600	L
H-H_	0	-9.09177700	-5.36217900	-4.37805100	L
H-H_	0	-10.05282600	-6.44351000	-5.48261700	L
C-C_3	0	-6.72209800	-0.37039800	-11.30402200	L
O-O_3	0	-5.72009200	0.30780800	-10.58611800	L
C-C_3	0	-4.73888100	-0.63715600	-10.22400800	L

C-C_3	0	-5.15156600	-2.01414600	-10.70799200	L
C-C_3	0	-6.60303100	-1.85134400	-11.03064700	L
H-H_	0	-7.72246800	0.00045400	-10.99696800	L
H-H_	0	-6.58701300	-0.16940500	-12.38931200	L
H-H_	0	-3.76421400	-0.34371600	-10.66723900	L
H-H_	0	-4.63322200	-0.64290800	-9.11857300	L
H-H_	0	-4.58341200	-2.28121600	-11.62649600	L
H-H_	0	-4.99711300	-2.78985200	-9.92910800	L
H-H_	0	-6.90354600	-2.46030800	-11.91067300	L
H-H_	0	-7.22269900	-2.12666000	-10.14894700	L
C-C_3	0	-9.05801400	-3.12902500	4.25247400	L
O-O_3	0	-9.56176700	-3.17941000	5.56665200	L
C-C_3	0	-10.09414600	-1.90984200	5.86957400	L
C-C_3	0	-10.00855600	-1.02025000	4.64702900	L
C-C_3	0	-8.96089600	-1.68747900	3.81519100	L
H-H_	0	-8.06317400	-3.61752900	4.20997000	L
H-H_	0	-9.75051500	-3.67728400	3.57984700	L
H-H_	0	-11.14861800	-2.01428000	6.20217900	L
H-H_	0	-9.50747800	-1.46550000	6.69968900	L
H-H_	0	-10.98111400	-1.01914400	4.11146800	L
H-H_	0	-9.72552500	0.02058800	4.90625000	L
H-H_	0	-9.15436000	-1.57360100	2.72699800	L
H-H_	0	-7.95939300	-1.27843800	4.07145000	L
C-C_3	0	10.29830500	-2.65855900	4.83886100	L
O-O_3	0	9.23496000	-3.57788200	4.74407800	L
C-C_3	0	9.74452900	-4.85141400	5.06373900	L
C-C_3	0	11.14776000	-4.70931700	5.61160000	L
C-C_3	0	11.59887600	-3.40715700	5.03294700	L
H-H_	0	10.34399700	-2.04007500	3.91945400	L
H-H_	0	10.11246600	-1.99340300	5.70671800	L
H-H_	0	9.08307400	-5.34737500	5.80546400	L
H-H_	0	9.76795300	-5.46512100	4.14028100	L
H-H_	0	11.11728900	-4.64270400	6.72185100	L
H-H_	0	11.80234800	-5.55134600	5.29765700	L
H-H_	0	12.29197000	-2.86835200	5.71346100	L
H-H_	0	12.09208300	-3.57574000	4.05030400	L
C-C_3	0	-5.11667100	6.01407200	8.67895200	L
O-O_3	0	-6.31762300	6.56517500	9.16332000	L

C-C_3	0	-6.54033300	6.03900100	10.45169600	L
C-C_3	0	-5.40552800	5.10524400	10.82768700	L
C-C_3	0	-4.74926500	4.80993200	9.51601800	L
H-H_	0	-5.24148300	5.72808500	7.61446400	L
H-H_	0	-4.31625000	6.78082700	8.75158200	L
H-H_	0	-6.61678200	6.87009600	11.18496500	L
H-H_	0	-7.50312800	5.48459800	10.44929100	L
H-H_	0	-4.69001700	5.62737000	11.50088800	L
H-H_	0	-5.77989400	4.18210500	11.31994100	L
H-H_	0	-3.64974700	4.69504200	9.61944000	L
H-H_	0	-5.17881800	3.88834800	9.06995200	L
C-C_3	0	-6.29450400	6.08907800	4.43615400	L
O-O_3	0	-6.32244900	7.46948700	4.69296800	L
C-C_3	0	-7.29792400	7.68685300	5.68612800	L
C-C_3	0	-8.13264100	6.42949300	5.87061400	L
C-C_3	0	-7.64087400	5.50707700	4.79693600	L
H-H_	0	-6.06612200	5.91093400	3.36467700	L
H-H_	0	-5.49810800	5.62357600	5.05463900	L
H-H_	0	-6.79783200	7.96372400	6.63894700	L
H-H_	0	-7.94698700	8.53259200	5.37406200	L
H-H_	0	-7.94245400	5.98687800	6.87255100	L
H-H_	0	-9.21878200	6.63798800	5.75587200	L
H-H_	0	-7.55615300	4.46096000	5.15937100	L
H-H_	0	-8.32041000	5.54645600	3.91869900	L
C-C_3	0	2.21802500	-10.28533900	7.17978100	L
O-O_3	0	1.17226700	-9.68138400	7.90274800	L
C-C_3	0	0.15088400	-10.64228400	8.05332700	L
C-C_3	0	0.55802900	-11.93320000	7.36674600	L
C-C_3	0	1.68080000	-11.50867400	6.47421100	L
H-H_	0	2.63498400	-9.56593700	6.44614300	L
H-H_	0	3.02556600	-10.57614000	7.88672400	L
H-H_	0	-0.03750300	-10.81901800	9.13386700	L
H-H_	0	-0.78156300	-10.25061400	7.59501600	L
H-H_	0	0.92767300	-12.66435000	8.11960200	L
H-H_	0	-0.27937300	-12.38101400	6.78974600	L
H-H_	0	2.45379700	-12.30085300	6.37167600	L
H-H_	0	1.28770300	-11.22852300	5.47183400	L
C-C_3	0	4.06516200	5.67076900	-7.11538700	L

O-O_3	0	5.03046200	6.60849200	-7.52276000	L
C-C_3	0	6.03645100	6.63402500	-6.53570700	L
C-C_3	0	5.62385000	5.77328200	-5.35445300	L
C-C_3	0	4.18605800	5.46089900	-5.62346900	L
H-H_	0	3.05012400	6.03973400	-7.37251600	L
H-H_	0	4.24475200	4.71593600	-7.65428200	L
H-H_	0	6.99239200	6.26912500	-6.96688800	L
H-H_	0	6.18736900	7.68361700	-6.20779700	L
H-H_	0	6.21717100	4.83503400	-5.33245000	L
H-H_	0	5.74944900	6.31296200	-4.39280800	L
H-H_	0	3.92698100	4.42250000	-5.32653400	L
H-H_	0	3.53355400	6.17946300	-5.08332100	L
C-C_3	0	-6.53691400	10.23999900	0.12891600	L
O-O_3	0	-6.74598000	10.83921600	1.38727100	L
C-C_3	0	-5.96071300	10.14396100	2.32842800	L
C-C_3	0	-5.42836000	8.87514700	1.69853300	L
C-C_3	0	-5.44453800	9.19809300	0.24132900	L
H-H_	0	-7.48327700	9.76471700	-0.20501300	L
H-H_	0	-6.25965800	11.01488000	-0.61463200	L
H-H_	0	-5.11471200	10.79498000	2.63879200	L
H-H_	0	-6.57296200	9.90583100	3.22364300	L
H-H_	0	-4.40622800	8.62854700	2.05505200	L
H-H_	0	-6.11292600	8.02552700	1.90604700	L
H-H_	0	-4.46927100	9.63646700	-0.05393300	L
H-H_	0	-5.65985000	8.30382800	-0.37776200	L
C-C_3	0	-0.20668800	5.33959800	9.89421200	L
O-O_3	0	-0.58656300	4.00977000	9.64839700	L
C-C_3	0	-0.93907300	3.93427900	8.28803800	L
C-C_3	0	-1.22107600	5.33059300	7.75782900	L
C-C_3	0	-0.94605500	6.23214500	8.92723500	L
H-H_	0	-0.45157300	5.61602100	10.94058900	L
H-H_	0	0.89044000	5.44264600	9.75152700	L
H-H_	0	-0.10655300	3.46792500	7.72007600	L
H-H_	0	-1.83888000	3.29390400	8.18174200	L
H-H_	0	-0.53733300	5.56670500	6.91459200	L
H-H_	0	-2.27457700	5.43155400	7.41679600	L
H-H_	0	-0.33179800	7.11233300	8.63865500	L
H-H_	0	-1.90084600	6.57337700	9.38294800	L

C-C_3	0	12.76128000	3.79189900	-2.50424400	L
O-O_3	0	14.05442800	3.92954900	-3.04700600	L
C-C_3	0	14.96981600	3.96699000	-1.97616900	L
C-C_3	0	14.25955600	3.59828500	-0.69151600	L
C-C_3	0	12.82974300	3.90816700	-0.99608300	L
H-H_	0	12.09593700	4.57609000	-2.92410400	L
H-H_	0	12.35418700	2.79774100	-2.78836300	L
H-H_	0	15.80494000	3.26127900	-2.17279300	L
H-H_	0	15.38569500	4.99481300	-1.89617700	L
H-H_	0	14.38106400	2.51315500	-0.48773400	L
H-H_	0	14.63374100	4.18687500	0.17424800	L
H-H_	0	12.13744100	3.19665100	-0.49676400	L
H-H_	0	12.59647700	4.95063200	-0.68602400	L
C-C_3	0	10.17255100	-0.09048500	-2.99659100	L
O-O_3	0	10.26631300	0.81620900	-4.07222200	L
C-C_3	0	10.38204800	0.06309700	-5.25578100	L
C-C_3	0	10.74730400	-1.36102500	-4.90233300	L
C-C_3	0	10.19975300	-1.51315900	-3.51912800	L
H-H_	0	11.03033400	0.07685700	-2.31186600	L
H-H_	0	9.23329100	0.09428800	-2.43697400	L
H-H_	0	9.40907100	0.08448200	-5.79144600	L
H-H_	0	11.15743900	0.51145900	-5.91253900	L
H-H_	0	10.30033000	-2.08996500	-5.61012400	L
H-H_	0	11.85207600	-1.47855700	-4.88834200	L
H-H_	0	9.16943200	-1.92557500	-3.56075700	L
H-H_	0	10.83632600	-2.17158000	-2.89123400	L
C-C_3	0	-8.03546900	1.37351100	6.85565200	L
O-O_3	0	-9.15818600	1.05475600	7.64168300	L
C-C_3	0	-9.21530100	1.97744700	8.70521500	L
C-C_3	0	-8.04924400	2.94162700	8.61286700	L
C-C_3	0	-7.12165700	2.28155700	7.64282700	L
H-H_	0	-7.50915600	0.44099400	6.56350600	L
H-H_	0	-8.37218800	1.88905000	5.93276800	L
H-H_	0	-10.17860200	2.52948300	8.66712800	L
H-H_	0	-9.16917300	1.42046800	9.66539500	L
H-H_	0	-8.39234000	3.91918300	8.20887900	L
H-H_	0	-7.56516200	3.09196500	9.60155700	L
H-H_	0	-6.61683000	3.02386200	6.99041400	L

H-H_	0	-6.36498500	1.67808200	8.18659700	L
C-C_3	0	8.88441000	0.91655300	4.65477400	L
O-O_3	0	8.77629400	1.63475800	5.86226100	L
C-C_3	0	7.70124900	1.08634400	6.58737300	L
C-C_3	0	7.27953300	-0.21457800	5.94522500	L
C-C_3	0	7.71859700	-0.04042800	4.52956500	L
H-H_	0	8.90504400	1.62036900	3.80120000	L
H-H_	0	9.83796300	0.35155000	4.65916900	L
H-H_	0	8.00385700	0.92102400	7.64207600	L
H-H_	0	6.85677700	1.80435600	6.57092800	L
H-H_	0	7.83222800	-1.05965000	6.40696900	L
H-H_	0	6.18495100	-0.38231500	6.02637900	L
H-H_	0	8.02095400	-1.00341600	4.06830600	L
H-H_	0	6.90179100	0.42056500	3.93494800	L
C-C_3	0	-11.06845200	3.97722400	5.98254900	L
O-O_3	0	-11.38520500	4.72312600	4.83023000	L
C-C_3	0	-12.53344800	4.13834100	4.26992800	L
C-C_3	0	-12.55718500	2.67640800	4.65037300	L
C-C_3	0	-11.81198500	2.65012100	5.95198000	L
H-H_	0	-9.97289300	3.80326800	6.01175600	L
H-H_	0	-11.35049900	4.55922900	6.88651000	L
H-H_	0	-13.43639300	4.65335400	4.66621300	L
H-H_	0	-12.51624200	4.25309200	3.16617800	L
H-H_	0	-13.59650400	2.29716400	4.75846000	L
H-H_	0	-12.01505700	2.07475900	3.88906300	L
H-H_	0	-12.52300700	2.58719100	6.80497500	L
H-H_	0	-11.11276000	1.78869200	5.98902900	L
C-C_3	0	0.29543800	-7.19410800	10.22701400	L
O-O_3	0	-0.91266300	-6.92660600	10.90340500	L
C-C_3	0	-0.65296700	-5.92632700	11.86118800	L
C-C_3	0	0.84156900	-5.78642000	12.03505500	L
C-C_3	0	1.36732900	-6.23858800	10.71072200	L
H-H_	0	0.60004100	-8.24166600	10.43909700	L
H-H_	0	0.14469000	-7.08618600	9.13235600	L
H-H_	0	-1.08155300	-4.96384900	11.50564100	L
H-H_	0	-1.13362000	-6.19481300	12.82569000	L
H-H_	0	1.13607900	-4.74170500	12.26983900	L
H-H_	0	1.19611300	-6.46981800	12.83826900	L

H-H_	0	1.44291100	-5.37443900	10.01540000	L
H-H_	0	2.35646000	-6.73625900	10.80808100	L
C-C_3	0	11.04038600	-1.08614500	-10.38151300	L
O-O_3	0	9.96127700	-0.74891300	-11.21970400	L
C-C_3	0	8.91788800	-0.32961900	-10.37796800	L
C-C_3	0	9.52244400	0.25811000	-9.12266300	L
C-C_3	0	10.88902400	-0.36396100	-9.05244300	L
H-H_	0	11.05291700	-2.18668700	-10.22689700	L
H-H_	0	11.99221800	-0.79185900	-10.87270000	L
H-H_	0	8.29461700	0.42703800	-10.90030800	L
H-H_	0	8.27654200	-1.20043000	-10.12558600	L
H-H_	0	9.61617800	1.35997900	-9.23042200	L
H-H_	0	8.90809500	0.01372700	-8.22904000	L
H-H_	0	11.67030400	0.41887900	-8.93430500	L
H-H_	0	10.95895200	-1.07827700	-8.20400100	L
C-C_3	0	6.30162300	-2.86803600	-4.40365500	L
O-O_3	0	5.67906800	-2.48881400	-3.20362500	L
C-C_3	0	6.12142800	-1.18418000	-2.92589500	L
C-C_3	0	6.43955400	-0.49368100	-4.23541900	L
C-C_3	0	6.55972000	-1.62121800	-5.22691700	L
H-H_	0	7.25608200	-3.38737800	-4.16990500	L
H-H_	0	5.64568400	-3.57162500	-4.95890800	L
H-H_	0	5.32916600	-0.63196700	-2.37806800	L
H-H_	0	7.02789900	-1.23029300	-2.28348700	L
H-H_	0	5.61069800	0.19000300	-4.51664200	L
H-H_	0	7.38353300	0.08598100	-4.16249600	L
H-H_	0	5.79079200	-1.51991400	-6.02125500	L
H-H_	0	7.56831700	-1.65101000	-5.69211100	L
C-C_3	0	2.36427000	5.37029400	4.88009200	L
O-O_3	0	3.08095300	5.53047900	6.07638700	L
C-C_3	0	2.50714100	6.63488500	6.73366300	L
C-C_3	0	1.77548500	7.50029700	5.71730400	L
C-C_3	0	1.89574200	6.73458600	4.43057700	L
H-H_	0	3.01171400	4.90546500	4.10817200	L
H-H_	0	1.49324200	4.70425700	5.06465500	L
H-H_	0	1.80371300	6.27055300	7.51119400	L
H-H_	0	3.30623500	7.21752200	7.23639000	L
H-H_	0	0.70822600	7.61904700	6.00342800	L

H-H_	0	2.24470200	8.50299000	5.62517700	L
H-H_	0	0.92694900	6.67797200	3.89389600	L
H-H_	0	2.66208200	7.20289100	3.77459000	L
C-C_3	0	2.24551500	-1.12950900	9.83557700	L
O-O_3	0	1.56437800	0.04527700	10.21348700	L
C-C_3	0	0.44026000	-0.34034700	10.96552100	L
C-C_3	0	0.63710500	-1.75785100	11.45049900	L
C-C_3	0	1.53288100	-2.34162500	10.40498500	L
H-H_	0	2.30024500	-1.19039700	8.72756700	L
H-H_	0	3.27925700	-1.08462800	10.23838300	L
H-H_	0	0.31143900	0.34610400	11.82709400	L
H-H_	0	-0.46080700	-0.27865400	10.31924900	L
H-H_	0	1.15446300	-1.75396300	12.43534600	L
H-H_	0	-0.32589400	-2.30737900	11.53038200	L
H-H_	0	2.25197000	-3.07071600	10.83666600	L
H-H_	0	0.92098500	-2.83630600	9.62069700	L
C-C_3	0	5.86707800	1.86107100	-7.00053500	L
O-O_3	0	6.98695000	2.15691400	-6.19822200	L
C-C_3	0	7.87791800	2.92683200	-6.97315400	L
C-C_3	0	7.24324200	3.24157600	-8.31379700	L
C-C_3	0	6.17902700	2.20002800	-8.44058400	L
H-H_	0	5.60809900	0.78671500	-6.90920400	L
H-H_	0	5.00428200	2.46205300	-6.64476200	L
H-H_	0	8.13402200	3.86372200	-6.43580000	L
H-H_	0	8.81438600	2.34911400	-7.11858100	L
H-H_	0	6.77965700	4.25080500	-8.29111400	L
H-H_	0	7.97669100	3.18713100	-9.14649000	L
H-H_	0	5.28527100	2.58248400	-8.97970200	L
H-H_	0	6.58342500	1.30546900	-8.96307300	L
C-C_3	0	-2.85403800	7.62880400	-1.60083200	L
O-O_3	0	-2.59677700	8.99691700	-1.79753700	L
C-C_3	0	-1.69947800	9.09320500	-2.87129600	L
C-C_3	0	-0.88998300	7.81715800	-2.94427200	L
C-C_3	0	-1.74318800	6.80754300	-2.22903400	L
H-H_	0	-2.91346000	7.42305000	-0.51116000	L
H-H_	0	-3.83226000	7.37296900	-2.05919000	L
H-H_	0	-2.27213100	9.24575900	-3.80884900	L
H-H_	0	-1.03369000	9.96701600	-2.71719700	L

H-H_	0	-0.69291700	7.51972400	-3.99616800	L
H-H_	0	0.07585700	7.95163700	-2.41261500	L
H-H_	0	-2.16531300	6.06510400	-2.93953800	L
H-H_	0	-1.15728300	6.27452000	-1.45017700	L
C-C_3	0	-4.85710900	-3.02611700	5.55998400	L
O-O_3	0	-3.62399500	-3.70766600	5.57337800	L
C-C_3	0	-3.09320300	-3.63255600	4.27516600	L
C-C_3	0	-3.72115900	-2.45619400	3.57170400	L
C-C_3	0	-5.07773300	-2.39862200	4.19747000	L
H-H_	0	-5.67651900	-3.73798200	5.79278700	L
H-H_	0	-4.83364400	-2.23539900	6.33857600	L
H-H_	0	-1.99252100	-3.52361600	4.31869900	L
H-H_	0	-3.33007500	-4.57433800	3.73718400	L
H-H_	0	-3.15486200	-1.52765100	3.79651400	L
H-H_	0	-3.76889300	-2.61079300	2.47778900	L
H-H_	0	-5.45753700	-1.35992700	4.27155300	L
H-H_	0	-5.79385900	-3.01157300	3.61241600	L
C-C_3	0	-12.86953000	3.77452000	-1.78519200	L
O-O_3	0	-12.38540900	4.91468900	-1.11478600	L
C-C_3	0	-12.17910500	5.92406000	-2.07766900	L
C-C_3	0	-12.67036000	5.44639600	-3.42881700	L
C-C_3	0	-12.69287500	3.95932600	-3.27638000	L
H-H_	0	-12.32132200	2.87496600	-1.43780800	L
H-H_	0	-13.94700700	3.64582100	-1.54402900	L
H-H_	0	-12.71810800	6.84634400	-1.77321100	L
H-H_	0	-11.09301000	6.15387900	-2.13121700	L
H-H_	0	-13.69982700	5.82460000	-3.61576600	L
H-H_	0	-11.99480300	5.76783300	-4.25049800	L
H-H_	0	-13.52078800	3.49400000	-3.85398800	L
H-H_	0	-11.72072800	3.53167200	-3.60029400	L
C-C_3	0	-5.38274700	3.80572200	-7.93090400	L
O-O_3	0	-5.42019200	3.79242200	-6.52248400	L
C-C_3	0	-4.51621100	2.80576900	-6.08180800	L
C-C_3	0	-4.07816300	1.96785800	-7.26143200	L
C-C_3	0	-4.27386000	2.89584400	-8.41292900	L
H-H_	0	-6.35715400	3.44338100	-8.31505700	L
H-H_	0	-5.22461900	4.84265100	-8.29577800	L
H-H_	0	-3.63765400	3.31022700	-5.62732100	L

H-H_	0	-4.99745100	2.16415000	-5.31405100	L
H-H_	0	-3.02509500	1.63132200	-7.17432400	L
H-H_	0	-4.74443600	1.08715600	-7.36773200	L
H-H_	0	-3.34727200	3.48323000	-8.58704200	L
H-H_	0	-4.55572400	2.35079100	-9.33637400	L
C-C_3	0	-10.34568000	-10.66865300	-3.11007600	L
O-O_3	0	-9.35552900	-11.30533100	-3.88521000	L
C-C_3	0	-8.42800100	-10.32150900	-4.28427700	L
C-C_3	0	-8.72947500	-9.02575000	-3.56241100	L
C-C_3	0	-10.17526900	-9.16924900	-3.21785600	L
H-H_	0	-11.35377300	-10.97078000	-3.46525100	L
H-H_	0	-10.23224400	-10.98756000	-2.05129400	L
H-H_	0	-7.39838900	-10.66451600	-4.05638500	L
H-H_	0	-8.51414900	-10.17011400	-5.38226600	L
H-H_	0	-8.12247400	-8.95176500	-2.63479800	L
H-H_	0	-8.54387700	-8.14071700	-4.20313900	L
H-H_	0	-10.42798600	-8.65248600	-2.26964400	L
H-H_	0	-10.80367900	-8.77431300	-4.04641500	L
C-C_3	0	-0.38578800	-7.66617600	3.54576900	L
O-O_3	0	-1.50184800	-6.83723700	3.74713700	L
C-C_3	0	-1.57293000	-6.61297100	5.13061500	L
C-C_3	0	-1.00963300	-7.82334300	5.84236200	L
C-C_3	0	-0.14956600	-8.48437200	4.80053900	L
H-H_	0	-0.57198300	-8.33259500	2.67773500	L
H-H_	0	0.50267900	-7.03377500	3.33088600	L
H-H_	0	-0.98381100	-5.70543600	5.38260700	L
H-H_	0	-2.62758900	-6.44822000	5.42709400	L
H-H_	0	-0.41912400	-7.52611100	6.73453000	L
H-H_	0	-1.83294700	-8.50285500	6.15323200	L
H-H_	0	0.92115700	-8.45822100	5.08705100	L
H-H_	0	-0.45990800	-9.53784900	4.64720000	L
C-C_3	0	3.22730800	-6.78808200	7.44642100	L
O-O_3	0	2.29761600	-6.61851500	6.40682600	L
C-C_3	0	1.77795800	-5.32173000	6.54948400	L
C-C_3	0	2.83195600	-4.44726300	7.19458500	L
C-C_3	0	3.76936700	-5.42887500	7.84639900	L
H-H_	0	2.72617000	-7.27684700	8.30886100	L
H-H_	0	4.05176800	-7.44814500	7.10249100	L

H-H_	0	1.49759800	-4.92328600	5.55220200	L
H-H_	0	0.86913800	-5.35966700	7.18748800	L
H-H_	0	3.36476100	-3.84951000	6.42383900	L
H-H_	0	2.37871400	-3.76046800	7.93854100	L
H-H_	0	4.80319800	-5.29756400	7.46201600	L
H-H_	0	3.77112200	-5.31221700	8.95177800	L
C-C_3	0	2.22240900	-10.34443100	2.09098000	L
O-O_3	0	2.91853400	-11.26903500	2.89599700	L
C-C_3	0	3.96214100	-10.57298600	3.53879300	L
C-C_3	0	3.77651300	-9.08726200	3.32400000	L
C-C_3	0	2.98003800	-9.03461300	2.06235000	L
H-H_	0	2.11000700	-10.74872900	1.06401000	L
H-H_	0	1.21017400	-10.18697800	2.52118000	L
H-H_	0	3.96587400	-10.81246700	4.62244400	L
H-H_	0	4.93121000	-10.89885100	3.10405600	L
H-H_	0	3.19477100	-8.64791700	4.16289400	L
H-H_	0	4.74502800	-8.55942400	3.21922800	L
H-H_	0	2.29645200	-8.16089200	2.03434200	L
H-H_	0	3.66638000	-9.00679800	1.18769200	L
C-C_3	0	-0.03443500	1.56898700	-7.69306800	L
O-O_3	0	0.70230300	2.37138100	-6.80930500	L
C-C_3	0	1.76281000	2.90591200	-7.55708400	L
C-C_3	0	2.10440300	1.94284200	-8.67556400	L
C-C_3	0	0.90352700	1.04044000	-8.76018800	L
H-H_	0	-0.50190800	0.73471000	-7.13122300	L
H-H_	0	-0.84113500	2.17895000	-8.15183700	L
H-H_	0	1.46073800	3.89031300	-7.97486300	L
H-H_	0	2.63686600	3.05318400	-6.89336200	L
H-H_	0	2.27170400	2.48625000	-9.63111000	L
H-H_	0	3.01344400	1.35752500	-8.41574000	L
H-H_	0	0.43211600	1.08070000	-9.76534900	L
H-H_	0	1.19474700	-0.00503100	-8.53242100	L
C-C_3	0	-3.73846000	9.47006100	5.26498900	L
O-O_3	0	-2.78635900	10.50480400	5.33263700	L
C-C_3	0	-1.89340100	10.19775900	6.37921800	L
C-C_3	0	-2.30066200	8.89314500	7.03532900	L
C-C_3	0	-3.69633100	8.67263600	6.54663900	L
H-H_	0	-4.74993600	9.90157300	5.11364300	L

H-H_	0	-3.49897400	8.81166200	4.40263600	L
H-H_	0	-0.86042600	10.12098400	5.97811300	L
H-H_	0	-1.92037000	11.02095800	7.12503600	L
H-H_	0	-1.64241800	8.07195600	6.68277900	L
H-H_	0	-2.26099500	8.95774100	8.14437700	L
H-H_	0	-3.90620400	7.59652900	6.36650000	L
H-H_	0	-4.42647000	9.08629000	7.27692700	L
C-C_3	0	-1.56871600	10.43659100	2.10535300	L
O-O_3	0	-1.60307200	9.88134400	0.81226900	L
C-C_3	0	-0.41890700	9.13954300	0.63281500	L
C-C_3	0	0.50127100	9.36033900	1.81338600	L
C-C_3	0	-0.43325600	9.81381700	2.88700500	L
H-H_	0	-1.41461700	11.53405800	2.02272000	L
H-H_	0	-2.53793100	10.25689800	2.61189400	L
H-H_	0	-0.67864300	8.06453500	0.54965300	L
H-H_	0	0.07799300	9.44808000	-0.30921700	L
H-H_	0	1.04026600	8.43241800	2.09841600	L
H-H_	0	1.23147000	10.16421300	1.57670500	L
H-H_	0	-0.79989200	8.94124100	3.46732900	L
H-H_	0	0.04405700	10.54204200	3.57251200	L
C-C_3	0	5.81330100	-8.10506800	-5.37033600	L
O-O_3	0	5.52785500	-6.72843100	-5.26959500	L
C-C_3	0	4.95389000	-6.49824500	-4.00582200	L
C-C_3	0	4.53974700	-7.81922800	-3.39944300	L
C-C_3	0	5.43317000	-8.80542100	-4.08098600	L
H-H_	0	5.23158900	-8.52927800	-6.21549000	L
H-H_	0	6.89438900	-8.24648900	-5.58324900	L
H-H_	0	5.70182200	-5.99726600	-3.35641800	L
H-H_	0	4.07812800	-5.82512600	-4.10923000	L
H-H_	0	4.67887400	-7.82678500	-2.29678100	L
H-H_	0	3.48032400	-8.03543200	-3.65402500	L
H-H_	0	6.33897400	-8.99190300	-3.46476800	L
H-H_	0	4.90931900	-9.76674600	-4.27358200	L
C-C_3	0	-1.03062800	3.70541700	-11.01725100	L
O-O_3	0	-1.94783000	4.77159000	-10.97535600	L
C-C_3	0	-3.13302000	4.33768300	-11.60423900	L
C-C_3	0	-2.99300800	2.88348400	-12.01897600	L
C-C_3	0	-1.78684800	2.41917700	-11.26393200	L

H-H_	0	-0.30569500	3.88480800	-11.84114900	L
H-H_	0	-0.46919500	3.65499900	-10.06143200	L
H-H_	0	-3.97671600	4.44957300	-10.89139800	L
H-H_	0	-3.33893900	4.97554100	-12.49027100	L
H-H_	0	-3.89472600	2.28834000	-11.75832100	L
H-H_	0	-2.80509000	2.81607100	-13.11340000	L
H-H_	0	-2.09393400	1.96282100	-10.29774300	L
H-H_	0	-1.18477000	1.69101100	-11.84958600	L
C-C_3	0	12.37430900	0.31020400	2.89272100	L
O-O_3	0	13.20075300	-0.65787000	3.49731800	L
C-C_3	0	14.28525400	-0.89891000	2.63152500	L
C-C_3	0	14.01893400	-0.23989700	1.29505100	L
C-C_3	0	13.03902800	0.83527600	1.63774600	L
H-H_	0	11.40467100	-0.16111500	2.63496000	L
H-H_	0	12.18313100	1.13805600	3.60709700	L
H-H_	0	15.20682200	-0.47425100	3.08547200	L
H-H_	0	14.42550300	-1.99328600	2.50370000	L
H-H_	0	14.94693800	0.17447700	0.84477000	L
H-H_	0	13.55354900	-0.96779600	0.59601400	L
H-H_	0	13.57637300	1.78325300	1.86123800	L
H-H_	0	12.30680300	0.99875300	0.81788700	L
C-C_3	0	-11.03378200	6.87837300	1.22863100	L
O-O_3	0	-9.93572400	6.40055300	0.48589400	L
C-C_3	0	-8.77119000	6.98863200	1.01869400	L
C-C_3	0	-9.15874500	8.03790600	2.03889200	L
C-C_3	0	-10.52855300	7.60831500	2.45388100	L
H-H_	0	-11.62188400	7.57373800	0.59130100	L
H-H_	0	-11.68760700	6.03035700	1.52190200	L
H-H_	0	-8.16280400	6.20107500	1.50955700	L
H-H_	0	-8.17049100	7.43648400	0.20026500	L
H-H_	0	-8.45673800	8.06115900	2.89828000	L
H-H_	0	-9.20341400	9.03991300	1.55841000	L
H-H_	0	-10.46312800	6.90730100	3.31487100	L
H-H_	0	-11.17302100	8.47482800	2.71714400	L
C-C_3	0	2.15069700	8.96240400	-5.56457700	L
O-O_3	0	3.51714000	8.79549300	-5.85175200	L
C-C_3	0	4.24420100	9.37690200	-4.79228900	L
C-C_3	0	3.28794200	10.02892200	-3.81298000	L

C-C_3	0	2.00268300	10.08948300	-4.57281500	L
H-H_	0	1.59462700	9.19005700	-6.49751900	L
H-H_	0	1.75235000	8.02015500	-5.12926500	L
H-H_	0	4.84614000	8.59890400	-4.28005900	L
H-H_	0	4.93752800	10.13828500	-5.20946600	L
H-H_	0	3.16173000	9.38786100	-2.91411700	L
H-H_	0	3.63751400	11.03859000	-3.50888900	L
H-H_	0	1.12351500	9.94335700	-3.91186700	L
H-H_	0	1.92507900	11.05974500	-5.11066600	L
C-C_3	0	-0.93070200	-11.19641200	-0.47180400	L
O-O_3	0	-0.21578100	-10.80648400	-1.61639800	L
C-C_3	0	-1.16700500	-10.38023300	-2.56288600	L
C-C_3	0	-2.48241700	-10.08074900	-1.86221600	L
C-C_3	0	-2.21587900	-10.40501300	-0.42155500	L
H-H_	0	-1.15067700	-12.28537200	-0.52928200	L
H-H_	0	-0.32256500	-11.00328900	0.43623200	L
H-H_	0	-0.78944900	-9.46981100	-3.07400100	L
H-H_	0	-1.30810300	-11.17687300	-3.32488600	L
H-H_	0	-2.76974700	-9.01393500	-1.98039400	L
H-H_	0	-3.29081000	-10.72963900	-2.26501800	L
H-H_	0	-2.06076000	-9.47121300	0.16008100	L
H-H_	0	-3.04135400	-10.99483000	0.02974500	L
C-C_3	0	11.71530400	-1.40697900	8.48403600	L
O-O_3	0	13.08260000	-1.51851400	8.15770400	L
C-C_3	0	13.41826400	-0.42582900	7.33404900	L
C-C_3	0	12.15072800	0.24226700	6.85105300	L
C-C_3	0	11.15681700	-0.12163100	7.90726700	L
H-H_	0	11.17278500	-2.27934500	8.05992800	L
H-H_	0	11.59187900	-1.42146900	9.58792900	L
H-H_	0	14.02583300	0.29549200	7.92278600	L
H-H_	0	14.02293300	-0.77811900	6.47111300	L
H-H_	0	12.27018300	1.34317900	6.75343100	L
H-H_	0	11.84631700	-0.19275700	5.87615200	L
H-H_	0	11.12511200	0.66829600	8.68998000	L
H-H_	0	10.14353200	-0.27160400	7.48186200	L
C-C_3	0	8.06098100	4.41790300	8.44741900	L
O-O_3	0	8.86057500	4.85161700	7.37705200	L
C-C_3	0	8.34626900	6.09872000	6.97111200	L

C-C_3	0	6.93070300	6.26093200	7.50506700	L
C-C_3	0	6.67591400	4.99126800	8.26311500	L
H-H_	0	8.02545300	3.30963100	8.46841900	L
H-H_	0	8.50180000	4.77671000	9.40392400	L
H-H_	0	9.00124300	6.90913400	7.35842100	L
H-H_	0	8.34856500	6.15192700	5.86220800	L
H-H_	0	6.87386800	7.13826700	8.18615500	L
H-H_	0	6.19625500	6.38055800	6.68156900	L
H-H_	0	6.17568800	5.18041900	9.23762500	L
H-H_	0	6.05835200	4.30003600	7.65021900	L
C-C_3	0	6.78445300	-5.19218400	1.14061900	L
O-O_3	0	6.95838500	-3.80751300	0.94703600	L
C-C_3	0	6.24431500	-3.46181000	-0.21209600	L
C-C_3	0	5.17682200	-4.50081600	-0.44454100	L
C-C_3	0	5.79897700	-5.73627500	0.12120800	L
H-H_	0	7.76419000	-5.70494000	1.06194100	L
H-H_	0	6.38888400	-5.35890700	2.15811400	L
H-H_	0	5.78360900	-2.46100000	-0.09152500	L
H-H_	0	6.94804800	-3.43628700	-1.07068600	L
H-H_	0	4.26732900	-4.23142100	0.13296800	L
H-H_	0	4.92688000	-4.61140400	-1.52050300	L
H-H_	0	5.04206500	-6.40292700	0.58943500	L
H-H_	0	6.33875400	-6.28493300	-0.67986600	L
C-C_3	0	-4.04198800	-3.40307500	-6.99614500	L
O-O_3	0	-5.40202800	-3.74569900	-7.11873600	L
C-C_3	0	-6.14099400	-2.60470700	-6.76531300	L
C-C_3	0	-5.32024800	-1.77942300	-5.80266200	L
C-C_3	0	-3.90639200	-2.15319400	-6.14193500	L
H-H_	0	-3.49531100	-4.24874900	-6.52813500	L
H-H_	0	-3.61316000	-3.23002300	-8.00574900	L
H-H_	0	-6.36410100	-2.02109700	-7.68272000	L
H-H_	0	-7.09782800	-2.90566500	-6.29216700	L
H-H_	0	-5.50623800	-0.69265700	-5.93502300	L
H-H_	0	-5.55577500	-2.06900100	-4.75518400	L
H-H_	0	-3.41788800	-1.33773800	-6.71909300	L
H-H_	0	-3.31964700	-2.36132600	-5.22171100	L
C-C_3	0	-10.46005600	-1.49591600	-3.14007400	L
O-O_3	0	-9.70261800	-0.33022300	-2.94234900	L

C-C_3	0	-10.62755300	0.68479100	-2.65600600	L
C-C_3	0	-11.91297000	0.37747000	-3.39541800	L
C-C_3	0	-11.82747700	-1.10073500	-3.66793900	L
H-H_	0	-9.93780400	-2.15944600	-3.85793000	L
H-H_	0	-10.55918200	-2.03599800	-2.17459200	L
H-H_	0	-10.80520800	0.71814500	-1.56085300	L
H-H_	0	-10.21991400	1.66430900	-2.97362600	L
H-H_	0	-12.80133500	0.62897500	-2.77609500	L
H-H_	0	-11.94987400	0.94448300	-4.35187000	L
H-H_	0	-12.63224400	-1.65260500	-3.13630300	L
H-H_	0	-11.89923600	-1.30210300	-4.75928000	L
C-C_3	0	-5.75980900	3.57875200	1.05107000	L
O-O_3	0	-6.31892000	4.68277000	0.38257300	L
C-C_3	0	-5.26595200	5.33030100	-0.27976500	L
C-C_3	0	-4.20871700	4.30358600	-0.60445700	L
C-C_3	0	-4.39021600	3.27144100	0.47105700	L
H-H_	0	-5.68345900	3.81201800	2.13165200	L
H-H_	0	-6.42982800	2.70538500	0.93383700	L
H-H_	0	-5.64472000	5.80697600	-1.20784600	L
H-H_	0	-4.84641500	6.11443700	0.38641000	L
H-H_	0	-4.40769900	3.86226000	-1.60316000	L
H-H_	0	-3.19128700	4.74491100	-0.59298500	L
H-H_	0	-4.34462000	2.24351700	0.05014400	L
H-H_	0	-3.61274200	3.38512000	1.25677900	L
C-C_3	0	4.68191000	1.84865300	8.98643600	L
O-O_3	0	4.13528200	2.91085400	9.72882600	L
C-C_3	0	2.91274900	3.26791200	9.12505500	L
C-C_3	0	2.67552400	2.41479900	7.89318400	L
C-C_3	0	4.01135700	1.79546900	7.63341200	L
H-H_	0	5.77630900	1.99365100	8.87432600	L
H-H_	0	4.50766000	0.89969700	9.53556500	L
H-H_	0	2.08642800	3.13056900	9.85440700	L
H-H_	0	2.95957300	4.33954500	8.83719400	L
H-H_	0	1.92421200	1.62553500	8.10850700	L
H-H_	0	2.33258900	3.02555100	7.03120100	L
H-H_	0	3.92187000	0.75308200	7.26202400	L
H-H_	0	4.57402100	2.41244500	6.90113900	L
C-C_3	0	-7.19405300	2.49996400	-2.53470700	L

O-O_3	0	-8.01284600	2.96840300	-1.48741400	L
C-C_3	0	-8.69435500	4.10968700	-1.95544600	L
C-C_3	0	-8.49464100	4.23111900	-3.44757600	L
C-C_3	0	-7.21164700	3.49950900	-3.66896700	L
H-H_	0	-6.15897500	2.34975400	-2.16643100	L
H-H_	0	-7.58402800	1.52137300	-2.88680400	L
H-H_	0	-9.77373700	4.02619500	-1.71818300	L
H-H_	0	-8.28837900	5.00979100	-1.44641800	L
H-H_	0	-9.31805400	3.71311300	-3.98268700	L
H-H_	0	-8.44167800	5.28925400	-3.77804600	L
H-H_	0	-7.19480400	3.00028700	-4.65783200	L
H-H_	0	-6.34766000	4.19298300	-3.57688600	L
C-C_3	0	1.85691500	-5.82049200	-0.33402800	L
O-O_3	0	2.29935000	-6.61591000	-1.40250800	L
C-C_3	0	1.62688100	-7.84612900	-1.28304200	L
C-C_3	0	0.34998000	-7.64095200	-0.48741600	L
C-C_3	0	0.44119700	-6.22302500	0.00083700	L
H-H_	0	2.51823800	-5.98506700	0.54461500	L
H-H_	0	1.89777200	-4.74931900	-0.61910400	L
H-H_	0	1.39254300	-8.24073600	-2.29279800	L
H-H_	0	2.28859400	-8.57764600	-0.77411000	L
H-H_	0	-0.54435100	-7.77215000	-1.13343000	L
H-H_	0	0.30260300	-8.35322700	0.36377900	L
H-H_	0	-0.28059300	-5.58719900	-0.55325000	L
H-H_	0	0.24862800	-6.14046100	1.09055500	L
C-C_3	0	6.18615500	4.11187600	4.55073000	L
O-O_3	0	6.43522300	5.41248600	4.08182200	L
C-C_3	0	5.88598300	5.50038800	2.78868600	L
C-C_3	0	5.51176200	4.11355800	2.29417900	L
C-C_3	0	6.01689800	3.19703000	3.36302900	L
H-H_	0	7.03436600	3.77672300	5.18133400	L
H-H_	0	5.26210800	4.11530500	5.16706800	L
H-H_	0	4.99303200	6.15993500	2.81452300	L
H-H_	0	6.63724600	5.95414300	2.10874800	L
H-H_	0	4.40842200	4.01994500	2.19389200	L
H-H_	0	5.99813400	3.88923700	1.32264600	L
H-H_	0	5.31234800	2.36622600	3.56931600	L
H-H_	0	6.99768000	2.77767400	3.06555100	L

C-C_3	0	-2.22941300	3.91487200	12.82514700	L
O-O_3	0	-1.49171100	4.89480700	13.51972900	L
C-C_3	0	-0.23383400	4.34439600	13.83358100	L
C-C_3	0	-0.02776000	3.07669500	13.03457300	L
C-C_3	0	-1.42509100	2.63311400	12.73774500	L
H-H_	0	-3.19310800	3.73337500	13.34734000	L
H-H_	0	-2.45100100	4.29072600	11.80429300	L
H-H_	0	0.56515600	5.08087600	13.60288500	L
H-H_	0	-0.20285500	4.11588100	14.92123400	L
H-H_	0	0.51168000	3.30392200	12.08912300	L
H-H_	0	0.53461500	2.31144800	13.61254800	L
H-H_	0	-1.49999300	2.16816300	11.73151000	L
H-H_	0	-1.76947500	1.91352500	13.51305600	L
C-C_3	0	2.02756000	13.08369700	-2.09693800	L
O-O_3	0	1.34486000	14.26800300	-1.77431600	L
C-C_3	0	0.10665300	13.86908100	-1.24209700	L
C-C_3	0	0.26592000	12.49772100	-0.61124500	L
C-C_3	0	1.58385400	11.99854800	-1.13736500	L
H-H_	0	1.78535700	12.79743100	-3.14392100	L
H-H_	0	3.12319600	13.24829000	-2.01753300	L
H-H_	0	-0.22751500	14.60809100	-0.48320100	L
H-H_	0	-0.64819600	13.83502600	-2.05864800	L
H-H_	0	0.29410400	12.57737000	0.49716100	L
H-H_	0	-0.56531000	11.82686900	-0.91440800	L
H-H_	0	2.31729000	11.89575700	-0.30859400	L
H-H_	0	1.47201000	11.02308900	-1.65627400	L
C-C_3	0	-2.09665600	6.05815300	4.13348100	L
O-O_3	0	-1.22999400	4.96834800	4.35502300	L
C-C_3	0	-0.94959400	4.39606200	3.10239300	L
C-C_3	0	-1.23061500	5.42306200	2.03257900	L
C-C_3	0	-2.33506100	6.22598600	2.64391100	L
H-H_	0	-3.05548000	5.88729400	4.66546400	L
H-H_	0	-1.62492800	6.97293500	4.54520800	L
H-H_	0	0.11070800	4.07024900	3.06139800	L
H-H_	0	-1.60515900	3.51033500	2.96175700	L
H-H_	0	-0.33220700	6.05833100	1.87497100	L
H-H_	0	-1.53159400	4.95721200	1.07334200	L
H-H_	0	-2.29308300	7.29032100	2.33157300	L

H-H_	0	-3.32100100	5.79213300	2.36657700	L
C-C_3	0	-3.57512000	12.72296300	-0.33949900	L
O-O_3	0	-4.71358000	13.50447000	-0.06520500	L
C-C_3	0	-5.28152700	13.88131500	-1.29958900	L
C-C_3	0	-4.43428300	13.34644400	-2.43794300	L
C-C_3	0	-3.61365100	12.28060900	-1.78555900	L
H-H_	0	-2.66662300	13.33449700	-0.15206600	L
H-H_	0	-3.55414800	11.84459400	0.33715500	L
H-H_	0	-6.30857400	13.46170600	-1.36387500	L
H-H_	0	-5.35067000	14.98870600	-1.35557100	L
H-H_	0	-5.05978400	12.93715700	-3.26104800	L
H-H_	0	-3.77097500	14.14908700	-2.83012000	L
H-H_	0	-4.12851100	11.30085800	-1.87370300	L
H-H_	0	-2.59815400	12.20971300	-2.22873300	L
C-C_3	0	-7.85852800	0.81379400	-8.01766700	L
O-O_3	0	-7.87110700	1.50811200	-6.79552700	L
C-C_3	0	-8.60475100	0.71888900	-5.89595200	L
C-C_3	0	-9.61772400	-0.08487500	-6.67937400	L
C-C_3	0	-9.02813400	-0.15438200	-8.05983300	L
H-H_	0	-7.92941600	1.53970300	-8.85272000	L
H-H_	0	-6.89951700	0.26554200	-8.11427000	L
H-H_	0	-7.91202600	0.04250100	-5.34812600	L
H-H_	0	-9.11708900	1.37327000	-5.16225800	L
H-H_	0	-9.75602100	-1.09583300	-6.24153400	L
H-H_	0	-10.59287400	0.44964100	-6.70325000	L
H-H_	0	-8.67442000	-1.18288200	-8.28566000	L
H-H_	0	-9.77413700	0.15395800	-8.82472700	L
C-C_3	0	5.39918900	10.09814300	-0.85855400	L
O-O_3	0	4.15828700	9.73198300	-0.31240200	L
C-C_3	0	4.33854400	9.70538000	1.08226400	L
C-C_3	0	5.80451200	9.44918700	1.38606200	L
C-C_3	0	6.48777000	9.57684000	0.05231000	L
H-H_	0	5.50844300	9.66846700	-1.87542500	L
H-H_	0	5.45402900	11.20694000	-0.93482200	L
H-H_	0	4.02336900	10.68164600	1.50824100	L
H-H_	0	3.70650300	8.90691400	1.52526300	L
H-H_	0	6.19284600	10.20228700	2.10651100	L
H-H_	0	5.94827800	8.42964300	1.80277200	L

H-H_	0	7.34701200	10.28063300	0.09541300	L
H-H_	0	6.83434800	8.57945200	-0.29915100	L
C-C_3	0	9.72516300	-8.58548000	3.85867200	L
O-O_3	0	8.95466700	-7.80506000	2.97463600	L
C-C_3	0	7.69757100	-7.59422700	3.57311200	L
C-C_3	0	7.55276800	-8.49719100	4.77692400	L
C-C_3	0	8.97250700	-8.76821900	5.16144000	L
H-H_	0	9.91504300	-9.57478900	3.38896200	L
H-H_	0	10.70146600	-8.08748300	4.04100700	L
H-H_	0	7.62406700	-6.53440400	3.89060200	L
H-H_	0	6.89603900	-7.79331300	2.83506300	L
H-H_	0	6.98807500	-8.00380600	5.59795500	L
H-H_	0	7.04624200	-9.44364800	4.48574400	L
H-H_	0	9.31452900	-8.01606100	5.90668400	L
H-H_	0	9.09935200	-9.79317800	5.57273300	L
C-C_3	0	5.53593200	-2.28000500	-8.88843200	L
O-O_3	0	4.98499600	-0.99026300	-9.00374600	L
C-C_3	0	4.22441100	-0.95682300	-10.19058500	L
C-C_3	0	4.26670600	-2.31607600	-10.86131500	L
C-C_3	0	5.45752300	-2.96598400	-10.23266600	L
H-H_	0	6.58843800	-2.20846900	-8.54565600	L
H-H_	0	4.95654600	-2.85510400	-8.13362500	L
H-H_	0	3.17666300	-0.67639100	-9.95668200	L
H-H_	0	4.65356400	-0.18651800	-10.86698300	L
H-H_	0	3.34558900	-2.89014900	-10.61727300	L
H-H_	0	4.37764100	-2.23160900	-11.96414800	L
H-H_	0	5.33211800	-4.06522200	-10.13431500	L
H-H_	0	6.36861900	-2.74037200	-10.82865200	L
C-C_3	0	-8.86963400	2.52741500	2.93557900	L
O-O_3	0	-8.86202800	1.21106200	2.45192400	L
C-C_3	0	-9.99326000	1.08369500	1.62432000	L
C-C_3	0	-10.45050900	2.46255100	1.17985200	L
C-C_3	0	-9.52763900	3.40434800	1.89812300	L
H-H_	0	-9.44402100	2.56424500	3.88482300	L
H-H_	0	-7.83068900	2.86203100	3.13455400	L
H-H_	0	-9.73159200	0.46267600	0.74359300	L
H-H_	0	-10.80319900	0.57069100	2.18460300	L
H-H_	0	-10.35653300	2.57670900	0.08083000	L

H-H_	0	-11.50662600	2.63949900	1.47747500	L
H-H_	0	-8.76473900	3.79648100	1.19372800	L
H-H_	0	-10.07455200	4.24704600	2.36950200	L
C-C_3	0	-3.65665700	-4.97767000	9.61902400	L
O-O_3	0	-3.43574900	-3.59986000	9.81739100	L
C-C_3	0	-2.44287000	-3.18842200	8.90674900	L
C-C_3	0	-1.79128300	-4.40645900	8.29423300	L
C-C_3	0	-2.85037900	-5.45450400	8.42817600	L
H-H_	0	-3.34534200	-5.52622700	10.53277700	L
H-H_	0	-4.73881500	-5.16321300	9.45798300	L
H-H_	0	-2.91507900	-2.56807600	8.11428300	L
H-H_	0	-1.68491100	-2.57129100	9.43151100	L
H-H_	0	-1.50538800	-4.23069900	7.23606900	L
H-H_	0	-0.89420500	-4.69164900	8.88517500	L
H-H_	0	-3.49241400	-5.46891800	7.52234200	L
H-H_	0	-2.41624100	-6.46393000	8.59332400	L
C-C_3	0	-3.83576300	-5.83667500	-3.23651100	L
O-O_3	0	-4.55976000	-6.93083800	-2.72556800	L
C-C_3	0	-5.88076700	-6.50243400	-2.49807200	L
C-C_3	0	-5.95476000	-4.99826800	-2.63623300	L
C-C_3	0	-4.77427300	-4.67858400	-3.49236800	L
H-H_	0	-3.06989700	-5.53792900	-2.49328400	L
H-H_	0	-3.32026600	-6.13239800	-4.17355400	L
H-H_	0	-6.54660200	-6.98026200	-3.24610500	L
H-H_	0	-6.20260500	-6.81520900	-1.48365000	L
H-H_	0	-6.90674300	-4.66913200	-3.10619900	L
H-H_	0	-5.83899700	-4.52148800	-1.64173000	L
H-H_	0	-5.07858200	-4.66866200	-4.55949900	L
H-H_	0	-4.31408000	-3.70488600	-3.21509000	L
C-C_3	0	9.67639300	4.40457100	2.76687700	L
O-O_3	0	9.61464700	4.25924800	4.16686000	L
C-C_3	0	10.92764300	4.06892600	4.63986900	L
C-C_3	0	11.91137600	4.36417400	3.52917100	L
C-C_3	0	11.09442700	4.17546700	2.29127600	L
H-H_	0	8.99090300	3.68201100	2.28182100	L
H-H_	0	9.34283800	5.42659500	2.49857400	L
H-H_	0	11.11215500	4.73681400	5.50789700	L
H-H_	0	11.04153600	3.01589700	4.97510700	L

H-H_	0	12.26044000	5.41690400	3.60110200	L
H-H_	0	12.78493700	3.67742900	3.55638100	L
H-H_	0	11.38095400	4.89553800	1.49620100	L
H-H_	0	11.20338400	3.13477400	1.91697600	L
C-C_3	0	10.30311000	8.16153800	3.30642700	L
O-O_3	0	11.44127400	7.75262400	2.58377500	L
C-C_3	0	11.20295600	8.05402100	1.23211000	L
C-C_3	0	9.71100300	8.07408900	0.99877700	L
C-C_3	0	9.15063500	8.41097300	2.34770000	L
H-H_	0	10.03063600	7.36771700	4.03466700	L
H-H_	0	10.54077000	9.08732700	3.87383300	L
H-H_	0	11.64126400	9.04903900	0.99720200	L
H-H_	0	11.68611100	7.29128600	0.58502700	L
H-H_	0	9.43202500	8.83377000	0.23881400	L
H-H_	0	9.36078300	7.07006300	0.67492500	L
H-H_	0	8.84430600	9.47940300	2.38104500	L
H-H_	0	8.27902700	7.76615600	2.58769900	L
C-C_3	0	-1.91295800	-5.78688700	-11.40099600	L
O-O_3	0	-1.08402400	-5.87000800	-10.26592300	L
C-C_3	0	-1.91029700	-6.09579500	-9.14663800	L
C-C_3	0	-3.34406700	-6.27841300	-9.59992300	L
C-C_3	0	-3.35244100	-5.65261000	-10.95738400	L
H-H_	0	-1.61547900	-4.91415700	-12.02036100	L
H-H_	0	-1.78722400	-6.71118300	-12.00585600	L
H-H_	0	-1.55782300	-6.99660400	-8.60227500	L
H-H_	0	-1.84156900	-5.21984100	-8.46840100	L
H-H_	0	-3.58341700	-7.36202700	-9.67891000	L
H-H_	0	-4.06352000	-5.78202900	-8.91343100	L
H-H_	0	-4.05123800	-6.17061500	-11.64945900	L
H-H_	0	-3.62267900	-4.57843300	-10.87826700	L
C-C_3	0	-4.90386300	-6.90123800	1.91214500	L
O-O_3	0	-4.02065000	-7.64367100	1.11321100	L
C-C_3	0	-3.10508700	-6.72628300	0.56479900	L
C-C_3	0	-3.65881000	-5.31643500	0.69086400	L
C-C_3	0	-4.99768300	-5.51137000	1.33904900	L
H-H_	0	-5.90306300	-7.38508300	1.92400100	L
H-H_	0	-4.50885500	-6.85918400	2.95052100	L
H-H_	0	-2.14109700	-6.81206000	1.10557400	L

H-H_	0	-2.93145500	-6.97891100	-0.50047500	L
H-H_	0	-2.99359700	-4.70039300	1.33288900	L
H-H_	0	-3.76671600	-4.82534500	-0.29816100	L
H-H_	0	-5.19734500	-4.76112900	2.12944400	L
H-H_	0	-5.80077600	-5.47367400	0.57390600	L
C-C_3	0	2.10154600	6.85540600	-0.55819800	L
O-O_3	0	2.60891300	6.91025800	-1.86790100	L
C-C_3	0	3.96008600	6.51665100	-1.81726500	L
C-C_3	0	4.31582300	6.07228600	-0.41218400	L
C-C_3	0	2.99315900	5.97511800	0.28208200	L
H-H_	0	2.08173100	7.88337400	-0.14261300	L
H-H_	0	1.06861800	6.45167800	-0.56994200	L
H-H_	0	4.11355700	5.68097100	-2.52928800	L
H-H_	0	4.60773500	7.36105900	-2.13009100	L
H-H_	0	4.84178600	5.09716600	-0.42054600	L
H-H_	0	4.95442600	6.83448600	0.08199600	L
H-H_	0	2.62308500	4.92783100	0.26146400	L
H-H_	0	3.04473100	6.33204800	1.33266500	L
C-C_3	0	-13.33514600	-5.43658900	-1.29117300	L
O-O_3	0	-13.18435600	-4.31058100	-2.12558300	L
C-C_3	0	-12.71092700	-4.75855300	-3.37513900	L
C-C_3	0	-12.32193500	-6.21677300	-3.27359200	L
C-C_3	0	-13.11317500	-6.69819600	-2.09910000	L
H-H_	0	-12.59155600	-5.37794400	-0.46754500	L
H-H_	0	-14.35183900	-5.43638000	-0.84389100	L
H-H_	0	-13.51621300	-4.63216100	-4.13069600	L
H-H_	0	-11.83669400	-4.14888100	-3.68577200	L
H-H_	0	-12.57252300	-6.77742100	-4.20015800	L
H-H_	0	-11.23422200	-6.30388000	-3.06410100	L
H-H_	0	-14.08935400	-7.10751200	-2.44140100	L
H-H_	0	-12.56421000	-7.46944300	-1.51781200	L
C-C_3	0	-6.22890800	-2.20554100	0.69755000	L
O-O_3	0	-6.77277800	-2.36893600	-0.58931100	L
C-C_3	0	-7.35755100	-1.14642400	-0.94852500	L
C-C_3	0	-6.67211600	-0.04222400	-0.18220600	L
C-C_3	0	-6.16600500	-0.72942100	1.04960400	L
H-H_	0	-6.85533000	-2.74980200	1.43391800	L
H-H_	0	-5.21207000	-2.64340400	0.70519100	L

H-H_	0	-7.24366100	-0.98728900	-2.04170800	L
H-H_	0	-8.43981600	-1.17492900	-0.69547200	L
H-H_	0	-5.82025500	0.35572500	-0.77163600	L
H-H_	0	-7.38114300	0.77526800	0.05930200	L
H-H_	0	-5.12825300	-0.41086000	1.29175800	L
H-H_	0	-6.82696900	-0.51082800	1.91509700	L
C-C_3	0	0.42243900	-10.13863600	-7.50654700	L
O-O_3	0	1.77256200	-10.49936400	-7.69737900	L
C-C_3	0	2.33476500	-10.73549100	-6.42791300	L
C-C_3	0	1.43915700	-10.12980700	-5.37228000	L
C-C_3	0	0.09495000	-10.14834200	-6.02664000	L
H-H_	0	0.25890600	-9.12214100	-7.92180400	L
H-H_	0	-0.23432100	-10.84850200	-8.05328700	L
H-H_	0	2.42072500	-11.83260800	-6.26962500	L
H-H_	0	3.35004100	-10.28879100	-6.37754000	L
H-H_	0	1.44364200	-10.71850300	-4.43088500	L
H-H_	0	1.75406700	-9.08458100	-5.16753200	L
H-H_	0	-0.44313000	-11.08554700	-5.76300000	L
H-H_	0	-0.51545800	-9.26947300	-5.73257500	L
C-C_3	0	-9.12144100	-9.19577500	2.03085600	L
O-O_3	0	-10.09985000	-8.34900900	2.58930500	L
C-C_3	0	-11.25608300	-8.44029600	1.78934600	L
C-C_3	0	-11.10327900	-9.59229200	0.82051400	L
C-C_3	0	-9.61981700	-9.73829600	0.70763900	L
H-H_	0	-8.17470700	-8.63289400	1.89022700	L
H-H_	0	-8.92996600	-10.03387500	2.73518400	L
H-H_	0	-12.14716600	-8.59195200	2.43472200	L
H-H_	0	-11.38427700	-7.48764700	1.23128500	L
H-H_	0	-11.54336000	-10.51644300	1.25640200	L
H-H_	0	-11.57622700	-9.37542900	-0.16090600	L
H-H_	0	-9.31929100	-10.79704200	0.55272000	L
H-H_	0	-9.24119900	-9.11116100	-0.12758500	L
C-C_3	0	2.65172300	-5.82822100	-9.79696300	L
O-O_3	0	3.22949400	-5.07546900	-8.76239600	L
C-C_3	0	3.73774600	-6.00400400	-7.83427200	L
C-C_3	0	3.04800500	-7.34446200	-8.03416300	L
C-C_3	0	2.12704300	-7.11050200	-9.19752900	L
H-H_	0	1.83063300	-5.25362500	-10.27335100	L

H-H_	0	3.42375800	-6.04839600	-10.56761400	L
H-H_	0	4.83397300	-6.10766300	-7.98351600	L
H-H_	0	3.55573500	-5.63090000	-6.80513300	L
H-H_	0	3.79392500	-8.13089900	-8.28271200	L
H-H_	0	2.48045600	-7.64551500	-7.12761200	L
H-H_	0	2.15499400	-7.94842500	-9.92731800	L
H-H_	0	1.08790400	-6.95572800	-8.83595900	L
C-C_3	0	1.01170800	12.92347300	5.00919500	L
O-O_3	0	1.04653400	13.13708200	3.61831200	L
C-C_3	0	2.32716600	12.75293100	3.18830300	L
C-C_3	0	2.83668700	11.67006400	4.11340500	L
C-C_3	0	2.08174500	11.91216900	5.39060100	L
H-H_	0	1.18852200	13.88881800	5.53199200	L
H-H_	0	0.00642300	12.54841700	5.29671200	L
H-H_	0	2.27598300	12.38198300	2.14448300	L
H-H_	0	3.00432000	13.63516800	3.21813000	L
H-H_	0	2.58669000	10.66622200	3.70575400	L
H-H_	0	3.93565400	11.74922800	4.25980000	L
H-H_	0	1.62585300	10.96956800	5.76475500	L
H-H_	0	2.75421100	12.33217200	6.17059200	L
C-C_3	0	-3.36605300	-0.55755200	11.61854000	L
O-O_3	0	-3.11347900	-0.41311500	10.24435800	L
C-C_3	0	-3.64629600	0.82889900	9.85187400	L
C-C_3	0	-4.57141300	1.35553200	10.93569300	L
C-C_3	0	-4.57611400	0.27380800	11.97446800	L
H-H_	0	-3.54683600	-1.62708900	11.85621400	L
H-H_	0	-2.47790500	-0.21390700	12.19188900	L
H-H_	0	-2.81641100	1.54346600	9.66595500	L
H-H_	0	-4.20749500	0.69326900	8.90639300	L
H-H_	0	-4.16348600	2.29608900	11.36117200	L
H-H_	0	-5.59601300	1.53939600	10.54626200	L
H-H_	0	-4.49903100	0.68823000	13.00327600	L
H-H_	0	-5.49883100	-0.34058000	11.88217600	L
C-C_3	0	10.10029800	8.31288300	-2.62268200	L
O-O_3	0	8.86118000	8.97061600	-2.50808400	L
C-C_3	0	8.01483100	8.46672100	-3.51658900	L
C-C_3	0	8.77833500	7.48427500	-4.38665200	L
C-C_3	0	10.20747400	7.69666300	-3.99742200	L

H-H_	0	10.92605000	9.03641200	-2.45501900	L
H-H_	0	10.16484100	7.52051300	-1.84809500	L
H-H_	0	7.13707700	7.97115200	-3.04963200	L
H-H_	0	7.64855300	9.31465800	-4.13423600	L
H-H_	0	8.46815000	6.44405400	-4.15080300	L
H-H_	0	8.62339400	7.68472000	-5.46847700	L
H-H_	0	10.77729900	6.74194200	-3.97779900	L
H-H_	0	10.69067400	8.41382800	-4.69734000	L
C-C_3	0	-9.00330800	5.00386500	-7.03667300	L
O-O_3	0	-10.13520300	5.00886100	-6.20603500	L
C-C_3	0	-10.97246300	3.97802600	-6.67244600	L
C-C_3	0	-10.64264000	3.68736600	-8.12796200	L
C-C_3	0	-9.43083500	4.53174000	-8.40742000	L
H-H_	0	-8.57071800	6.02535200	-7.09161800	L
H-H_	0	-8.23916100	4.31692600	-6.61221400	L
H-H_	0	-10.81731600	3.06932700	-6.05104900	L
H-H_	0	-12.03285400	4.29315200	-6.57236500	L
H-H_	0	-10.41207400	2.60938900	-8.26734600	L
H-H_	0	-11.48489900	3.97392600	-8.79516500	L
H-H_	0	-8.62978200	3.94757400	-8.90446400	L
H-H_	0	-9.70790400	5.40366000	-9.03986000	L
C-C_3	0	8.77123500	-0.77306700	0.95171200	L
O-O_3	0	7.43570300	-0.79101500	1.38197500	L
C-C_3	0	6.79750000	0.29874600	0.76052400	L
C-C_3	0	7.84231900	1.26020100	0.22105900	L
C-C_3	0	9.14660700	0.65711500	0.64783500	L
H-H_	0	9.42339000	-1.18845100	1.74558200	L
H-H_	0	8.86930300	-1.40003300	0.03744200	L
H-H_	0	6.16318600	-0.07945200	-0.06576200	L
H-H_	0	6.14543200	0.81208600	1.49798200	L
H-H_	0	7.78213700	1.30180700	-0.88699300	L
H-H_	0	7.71451000	2.27945500	0.64093600	L
H-H_	0	9.92012300	0.72453500	-0.14509100	L
H-H_	0	9.51523000	1.15777600	1.56735600	L
C-C_3	0	12.62847700	-4.01571500	0.24709600	L
O-O_3	0	12.30659200	-5.36539700	0.49868400	L
C-C_3	0	11.18131200	-5.37248700	1.34121300	L
C-C_3	0	10.43369100	-4.07413400	1.15048100	L

C-C_3	0	11.52016500	-3.11682000	0.76890000	L
H-H_	0	13.59399800	-3.76679200	0.73729100	L
H-H_	0	12.74568900	-3.87271500	-0.84708300	L
H-H_	0	10.53107700	-6.23848800	1.09820300	L
H-H_	0	11.51997500	-5.47023200	2.39544200	L
H-H_	0	9.70472900	-4.16969500	0.31914900	L
H-H_	0	9.90426300	-3.76197900	2.07432900	L
H-H_	0	11.17930600	-2.39749700	-0.00743500	L
H-H_	0	11.86627600	-2.56195700	1.66726700	L
C-C_3	0	7.41329100	-10.90845100	1.40045600	L
O-O_3	0	6.70945900	-9.77585900	0.95047300	L
C-C_3	0	7.55386900	-9.06677000	0.07271600	L
C-C_3	0	8.89500900	-9.76521800	-0.03048400	L
C-C_3	0	8.61948800	-11.12999700	0.51577200	L
H-H_	0	7.73814400	-10.73803600	2.44909600	L
H-H_	0	6.74703800	-11.79674100	1.37676300	L
H-H_	0	7.07251100	-9.01461100	-0.92611800	L
H-H_	0	7.69210000	-8.03411300	0.44696700	L
H-H_	0	9.25718700	-9.81065900	-1.08023500	L
H-H_	0	9.64696600	-9.24432900	0.60279700	L
H-H_	0	8.36209900	-11.82657100	-0.31217800	L
H-H_	0	9.48443900	-11.52780800	1.08981700	L
C-C_3	0	-8.84615900	-5.33019600	1.25793800	L
O-O_3	0	-8.49456300	-6.33254700	0.33054400	L
C-C_3	0	-9.23596500	-6.08353300	-0.83662900	L
C-C_3	0	-9.53753400	-4.60715100	-0.89375400	L
C-C_3	0	-9.63840300	-4.23958200	0.55444500	L
H-H_	0	-9.44707800	-5.77761100	2.07679300	L
H-H_	0	-7.92280400	-4.90622700	1.70228100	L
H-H_	0	-8.66351700	-6.40099200	-1.73157400	L
H-H_	0	-10.18164800	-6.66521900	-0.79680800	L
H-H_	0	-8.69318800	-4.06580100	-1.37117900	L
H-H_	0	-10.47725100	-4.39728900	-1.44299700	L
H-H_	0	-9.22306000	-3.22817800	0.74929700	L
H-H_	0	-10.69974300	-4.27449100	0.87959300	L
C-C_3	0	-1.23926100	-1.89655900	-9.91075600	L
O-O_3	0	-1.34008400	-1.44231300	-8.58591300	L
C-C_3	0	-0.51698800	-2.28050800	-7.80997100	L

C-C_3	0	0.49031600	-2.97608300	-8.71067100	L
C-C_3	0	0.12243500	-2.52502600	-10.09491200	L
H-H_	0	-2.03933800	-2.64489000	-10.10421700	L
H-H_	0	-1.36813700	-1.04366500	-10.61019200	L
H-H_	0	0.00691400	-1.67679300	-7.04025000	L
H-H_	0	-1.14997600	-3.02665800	-7.29017500	L
H-H_	0	1.53089400	-2.67529900	-8.46759400	L
H-H_	0	0.39495400	-4.07851200	-8.61447000	L
H-H_	0	0.84611200	-1.76042900	-10.45365500	L
H-H_	0	0.08872800	-3.37188500	-10.81272700	L
C-C_3	0	13.02898700	-4.57301000	-4.58385800	L
O-O_3	0	12.33134700	-5.66537400	-5.13690500	L
C-C_3	0	12.68393800	-6.81533300	-4.40075800	L
C-C_3	0	13.52744600	-6.41353000	-3.20862800	L
C-C_3	0	14.07756100	-5.08676200	-3.62100900	L
H-H_	0	13.50178000	-3.97911400	-5.39475300	L
H-H_	0	12.30909600	-3.92201100	-4.04403400	L
H-H_	0	11.76756800	-7.34490100	-4.06551800	L
H-H_	0	13.26334700	-7.49743700	-5.05986600	L
H-H_	0	12.88561500	-6.29424100	-2.30821200	L
H-H_	0	14.33431400	-7.15005900	-3.00328100	L
H-H_	0	14.21538000	-4.40982000	-2.75154000	L
H-H_	0	15.04583400	-5.22366600	-4.15156700	L
C-C_3	0	-2.07729600	7.38972400	-6.70491700	L
O-O_3	0	-0.69067300	7.54174100	-6.89524500	L
C-C_3	0	-0.13054900	6.25425300	-7.01254100	L
C-C_3	0	-1.23392900	5.21731800	-7.02104300	L
C-C_3	0	-2.38666800	5.94114900	-6.40963900	L
H-H_	0	-2.41188600	8.03114100	-5.86637800	L
H-H_	0	-2.60743900	7.70990300	-7.62753300	L
H-H_	0	0.46472100	6.18802200	-7.94502100	L
H-H_	0	0.55068800	6.08186500	-6.15564600	L
H-H_	0	-1.47405900	4.93012700	-8.06731600	L
H-H_	0	-0.96603100	4.31687400	-6.43002700	L
H-H_	0	-3.35434400	5.63235300	-6.85446000	L
H-H_	0	-2.39987300	5.76675000	-5.31265200	L
C-C_3	0	1.49779600	8.46083400	-9.71786300	L
O-O_3	0	0.61123300	9.38025300	-9.12149100	L

C-C_3	0	-0.61117000	9.28285700	-9.80837300	L
C-C_3	0	-0.70574100	7.90836000	-10.42931800	L
C-C_3	0	0.72984600	7.53489300	-10.64929200	L
H-H_	0	2.27876400	9.01553900	-10.28136000	L
H-H_	0	1.99846800	7.87299600	-8.91958300	L
H-H_	0	-1.45270700	9.45182100	-9.10399200	L
H-H_	0	-0.64781300	10.06408300	-10.59929300	L
H-H_	0	-1.18041100	7.20004000	-9.71643000	L
H-H_	0	-1.28101000	7.92509500	-11.38042100	L
H-H_	0	0.91050800	6.46448000	-10.40736400	L
H-H_	0	1.01829200	7.72926800	-11.70593200	L
C-C_3	0	-0.85169600	12.29449600	-5.11542300	L
O-O_3	0	-2.09678800	12.93200000	-4.94510900	L
C-C_3	0	-3.06409000	12.10923400	-5.54665300	L
C-C_3	0	-2.39233200	11.30070400	-6.63047800	L
C-C_3	0	-0.98851800	11.16256000	-6.12353800	L
H-H_	0	-0.11204700	13.03952700	-5.47849900	L
H-H_	0	-0.50201900	11.90620400	-4.13551700	L
H-H_	0	-3.49766700	11.43465300	-4.77660500	L
H-H_	0	-3.87771600	12.73322000	-5.97305800	L
H-H_	0	-2.88544200	10.31612000	-6.77529900	L
H-H_	0	-2.39756700	11.87031800	-7.58598000	L
H-H_	0	-0.85701500	10.18029100	-5.62095300	L
H-H_	0	-0.24995100	11.25639800	-6.94933400	L
C-C_3	0	14.76256100	-0.69236200	-4.45472600	L
O-O_3	0	14.58439800	-1.81993200	-3.62969300	L
C-C_3	0	14.16099400	-1.36596500	-2.36444900	L
C-C_3	0	14.23541300	0.14520200	-2.31915700	L
C-C_3	0	14.19754500	0.52904700	-3.76357300	L
H-H_	0	15.84976100	-0.55265100	-4.63987700	L
H-H_	0	14.25657600	-0.85646200	-5.42989400	L
H-H_	0	13.11491500	-1.69735900	-2.19572900	L
H-H_	0	14.80166400	-1.81100500	-1.57360900	L
H-H_	0	13.38740600	0.58429900	-1.75113900	L
H-H_	0	15.20018700	0.46464100	-1.86668700	L
H-H_	0	13.14722100	0.69784800	-4.08554500	L
H-H_	0	14.80674000	1.43546300	-3.96770000	L
C-C_3	0	-11.88457700	-5.60196200	6.11454200	L

O-O_3	0	-12.70876200	-4.47911200	5.89483400	L
C-C_3	0	-12.82761600	-4.30154000	4.50334600	L
C-C_3	0	-12.38199000	-5.56451500	3.80223500	L
C-C_3	0	-11.43434300	-6.17643300	4.78505300	L
H-H_	0	-11.00627600	-5.30616700	6.72737400	L
H-H_	0	-12.46410600	-6.36497900	6.67741500	L
H-H_	0	-13.88045800	-4.06300300	4.24224400	L
H-H_	0	-12.18643600	-3.44960900	4.19494000	L
H-H_	0	-13.25257200	-6.23834700	3.64252700	L
H-H_	0	-11.88988700	-5.34570400	2.83049800	L
H-H_	0	-11.49223900	-7.28631100	4.77641100	L
H-H_	0	-10.39497000	-5.85451500	4.56194700	L
C-C_3	0	-13.59966800	-1.28414400	2.26894000	L
O-O_3	0	-13.58286100	-0.00075700	1.69813500	L
C-C_3	0	-13.24827500	-0.17863600	0.34412600	L
C-C_3	0	-12.43799600	-1.45522800	0.20046000	L
C-C_3	0	-12.61416700	-2.15651100	1.51872500	L
H-H_	0	-13.32688800	-1.21926400	3.34320500	L
H-H_	0	-14.62557500	-1.70745600	2.19023800	L
H-H_	0	-14.18076100	-0.24368100	-0.25872200	L
H-H_	0	-12.66458400	0.69611500	-0.00872300	L
H-H_	0	-12.82571300	-2.07430400	-0.63599000	L
H-H_	0	-11.36518800	-1.22646200	0.02427800	L
H-H_	0	-13.01139200	-3.18656400	1.38508300	L
H-H_	0	-11.64495000	-2.19845800	2.06081800	L
C-C_3	0	5.57808300	-11.81527500	-1.78046200	L
O-O_3	0	5.06990300	-12.40092200	-2.95540800	L
C-C_3	0	3.67437800	-12.45110400	-2.80709700	L
C-C_3	0	3.25044800	-11.30990300	-1.91162100	L
C-C_3	0	4.46823600	-11.05447100	-1.07153000	L
H-H_	0	5.98207600	-12.61277500	-1.11966800	L
H-H_	0	6.40822200	-11.12854400	-2.04714300	L
H-H_	0	3.18912500	-12.36917000	-3.80166600	L
H-H_	0	3.38860100	-13.42478500	-2.35075500	L
H-H_	0	3.01455800	-10.41305000	-2.52433600	L
H-H_	0	2.37116500	-11.58600000	-1.29171800	L
H-H_	0	4.69074800	-9.96729200	-1.02027000	L
H-H_	0	4.32551900	-11.45102500	-0.04233800	L

C-C_3	0	-8.50033000	-1.70984900	9.79027200	L
O-O_3	0	-7.40564300	-0.83038900	9.66102200	L
C-C_3	0	-6.39890300	-1.50347600	8.94345600	L
C-C_3	0	-6.98822200	-2.73476200	8.29691900	L
C-C_3	0	-8.15392800	-3.05231400	9.17596200	L
H-H_	0	-8.75686400	-1.82969000	10.86441600	L
H-H_	0	-9.37671900	-1.27088200	9.26723700	L
H-H_	0	-5.97286800	-0.83097400	8.17118400	L
H-H_	0	-5.58934400	-1.79504100	9.64632200	L
H-H_	0	-7.33802600	-2.49356400	7.26959700	L
H-H_	0	-6.25610100	-3.56776500	8.26403000	L
H-H_	0	-9.00256800	-3.47560500	8.59516500	L
H-H_	0	-7.84583000	-3.76780300	9.97043300	L
C-C_R	0	2.14167500	0.15449900	1.08976700	H
C-C_R	0	2.32041600	1.36116500	2.91025900	H
C-C_3	0	2.90697300	-1.06426700	3.21023300	H
H-H_	0	2.08066600	-1.77363100	3.27946500	H
N-N_R	0	2.05576500	2.21727600	1.97169400	H
N-N_R	0	1.95095600	1.45777800	0.84019300	H
N-N_R	0	2.43588700	0.09088500	2.41175700	H
C-C_3	0	2.39355900	1.61993400	4.38551100	H
H-H_	0	1.51379000	2.19944800	4.67196500	H
H-H_	0	3.29418700	2.19154000	4.64280700	H
C-C_3	0	3.28568300	-0.52107500	4.59308700	H
O-O_3	0	2.34334100	0.40227900	5.08460100	H
C-C_R	0	1.67828000	2.15595400	-0.39155900	H
C-C_R	0	2.75068800	2.47313400	-1.23169700	H
C-C_R	0	0.37561400	2.59080200	-0.63227900	H
C-C_R	0	2.47909300	3.23310600	-2.36548400	H
C-C_R	0	0.17100900	3.41101600	-1.74135000	H
C-C_R	0	1.20034100	3.73250500	-2.62145500	H
H-H_	0	3.29263200	3.45526200	-3.05174900	H
H-H_	0	-0.83427200	3.77852800	-1.93228200	H
H-H_	0	3.33200900	-1.35229700	5.29899700	H
C-C_R	0	2.01029500	-1.04237100	0.21795200	H
O-O_R	0	1.66649300	-2.15123500	0.83199700	H
C-C_R	0	2.37443100	-0.92377800	-1.07767800	H
C-C_3	0	2.40433900	-2.07910300	-2.02074800	H

H-H_	0	2.68791000	0.04413700	-1.42997300	H
H-H_	0	3.19363300	-2.77809300	-1.71346700	H
H-H_	0	1.45487200	-2.62034900	-1.94548800	H
C-C_R	0	2.60392800	-1.72654900	-3.47843100	H
C-C_R	0	2.67282700	-2.77533100	-4.40308300	H
C-C_R	0	2.69923100	-0.42255600	-3.96529600	H
C-C_R	0	2.81192200	-2.53335800	-5.76163700	H
H-H_	0	2.61175200	-3.79704000	-4.04123900	H
C-C_R	0	2.84854600	-0.17431100	-5.32964500	H
H-H_	0	2.63123000	0.42985400	-3.29637700	H
C-C_R	0	2.89958300	-1.22497700	-6.23357500	H
H-H_	0	2.85097600	-3.36483800	-6.45553300	H
H-H_	0	2.92432300	0.85087200	-5.67871400	H
H-H_	0	3.01062000	-1.03108300	-7.29281200	H
C-C_3	0	4.15345700	2.03480200	-0.91377200	H
H-H_	0	4.60656800	2.70857900	-0.18420900	H
H-H_	0	4.17449600	1.03123300	-0.48892600	H
H-H_	0	4.76629700	2.03008800	-1.81673800	H
C-C_3	0	0.94617400	4.58329500	-3.83501600	H
H-H_	0	-0.11054300	4.83929200	-3.92727600	H
H-H_	0	1.51365500	5.51581900	-3.79343200	H
H-H_	0	1.24945300	4.05702800	-4.74408700	H
C-C_3	0	-0.76954800	2.14655700	0.22409100	H
H-H_	0	-1.01375800	1.10083600	0.00472100	H
H-H_	0	-0.54401100	2.19410500	1.28947500	H
H-H_	0	-1.64843000	2.75221200	0.00995200	H
H-H_	0	4.28081200	-0.04925100	4.53747400	H
C-C_3	0	4.11700300	-1.75133400	2.56567700	H
H-H_	0	3.92601800	-1.89518100	1.50137500	H
H-H_	0	4.99753300	-1.10996300	2.67884200	H
C-C_R	0	4.34861100	-3.09508900	3.21449700	H
C-C_R	0	5.41046500	-3.31808200	4.08978100	H
C-C_R	0	3.45484700	-4.13568900	2.94206800	H
C-C_R	0	5.59231800	-4.56868000	4.67575000	H
H-H_	0	6.10776600	-2.51407600	4.30463400	H
C-C_R	0	3.63693300	-5.38250200	3.52710000	H
H-H_	0	2.63086400	-3.95249800	2.25473300	H
C-C_R	0	4.70811300	-5.60359100	4.39048700	H

H-H_	0	6.42887100	-4.73708700	5.34591300	H
H-H_	0	2.93796900	-6.18147600	3.31000200	H
H-H_	0	4.85419900	-6.57933600	4.84026700	H
C-C_3	0	-1.54886100	1.79551500	-4.05745000	H
C-C_3	0	-0.40003700	0.80525200	-3.87504600	H
C-C_3	0	-0.79351500	-0.65591900	-4.14210300	H
C-C_3	0	-2.70418700	1.62265400	-3.07285000	H
C-C_R	0	-1.37551100	-1.31027800	-2.91120400	H
C-C_3	0	-3.32288900	0.22311100	-3.14006200	H
H-H_	0	-1.93720200	1.66634700	-5.07487300	H
H-H_	0	0.41093900	1.07560600	-4.55828100	H
H-H_	0	-1.48332200	-0.70534700	-4.99324300	H
H-H_	0	-2.36180700	1.80050600	-2.04462000	H
H-H_	0	-4.32388400	0.23111700	-2.69921900	H
C-C_3	0	-1.19871000	-2.80555200	-1.11467200	H
H-H_	0	-0.34825900	-3.20201400	-0.55011800	H
C-C_3	0	-3.02976500	-1.13198000	-1.09135500	H
C-C_3	0	-1.91244900	-1.75776700	-0.27472500	H
H-H_	0	-1.17969900	-0.99414100	0.00662700	H
H-H_	0	-2.30037000	-2.17821500	0.65479700	H
H-H_	0	-3.37913600	-0.21092200	-0.60623100	H
N-N_R	0	-2.56384600	-0.79516000	-2.43400200	H
N-N_R	0	-0.69425500	-2.24548500	-2.35837300	H
H-H_	0	-3.45724300	-0.05806800	-4.19033100	H
H-H_	0	-3.47884600	2.36735000	-3.28685700	H
H-H_	0	-1.16923000	2.82058300	-4.00523600	H
H-H_	0	0.02073100	0.87640400	-2.86182700	H
H-H_	0	0.09194400	-1.23719200	-4.39921000	H
H-H_	0	-1.87181300	-3.64550100	-1.33688400	H
H-H_	0	-3.88801900	-1.81500100	-1.16313500	H
C-C_R	0	-0.31582200	-1.03172900	4.38178500	H
C-C_R	0	-0.58156600	0.34019000	4.48757300	H
C-C_R	0	-0.63607400	0.95382400	5.75325000	H
C-C_R	0	-0.41554600	0.15680000	6.85211300	H
C-C_R	0	-0.12914000	-1.22901400	6.72064100	H
C-C_R	0	-0.07097500	-1.85180400	5.49475300	H
H-H_	0	-0.84457100	2.01372500	5.83866900	H
H-H_	0	-0.45832400	0.58901600	7.84625000	H

H-H_	0	0.04254600	-1.81287500	7.61708600	H
H-H_	0	0.14142600	-2.90758700	5.38122600	H
N-N_R	0	-0.70894300	0.85913600	3.22918700	H
N-N_R	0	-0.56739500	-0.12035300	2.37877100	H
N-N_R	0	-0.33274500	-1.24390800	3.04292400	H
O-O_R	0	-0.12760400	-2.41082000	2.41679200	H
H-H_	0	0.67029100	-2.22806100	1.64637100	H

TS4$_{explicit}$ (ONIOM(M06-2X/6-31G(d, p):UFF))

0 1 0 1 0 1

C-C_3	0	-5.34573700	7.30481800	-4.73711700	L
O-O_3	0	-6.29527100	6.88810100	-3.78274100	L
C-C_3	0	-6.95210200	8.03775200	-3.30761500	L
C-C_3	0	-6.09823400	9.24500700	-3.61744800	L
C-C_3	0	-5.37071800	8.81745800	-4.85308100	L
H-H_	0	-4.34090700	6.96877600	-4.40679800	L
H-H_	0	-5.57022700	6.83410400	-5.71831500	L
H-H_	0	-7.93730500	8.12690500	-3.81589400	L
H-H_	0	-7.12699600	7.94770400	-2.21583500	L
H-H_	0	-6.71100200	10.15664500	-3.78724500	L
H-H_	0	-5.37473500	9.41996000	-2.79176900	L
H-H_	0	-5.94318800	9.12172500	-5.75717200	L
H-H_	0	-4.34876400	9.24565200	-4.89582900	L
C-C_3	0	-8.28541600	3.24879000	-11.83636000	L
O-O_3	0	-9.05766300	4.42658500	-11.88625400	L
C-C_3	0	-8.16100300	5.50773800	-11.94094100	L
C-C_3	0	-6.85074800	5.07911300	-11.32334300	L
C-C_3	0	-6.83288800	3.59772800	-11.55362500	L
H-H_	0	-8.68448900	2.59139600	-11.03515100	L
H-H_	0	-8.37198800	2.70936200	-12.80424600	L
H-H_	0	-8.00665400	5.80010200	-13.00309900	L
H-H_	0	-8.58381900	6.37471000	-11.39023100	L
H-H_	0	-5.98633600	5.58473000	-11.80571000	L
H-H_	0	-6.85546900	5.29855100	-10.23327000	L
H-H_	0	-6.19891700	3.35144200	-12.43384800	L
H-H_	0	-6.45314100	3.05949000	-10.66078300	L
C-C_3	0	7.25468000	-3.46918400	8.13422500	L
O-O_3	0	6.07876400	-3.03614800	7.49814600	L

C-C_3	0	5.36246200	-2.30181900	8.45708000	L
C-C_3	0	6.34920800	-1.67827000	9.42309900	L
C-C_3	0	7.60882300	-2.47941600	9.22844300	L
H-H_	0	7.08655400	-4.48083200	8.56523200	L
H-H_	0	8.07709300	-3.53418800	7.39146900	L
H-H_	0	4.75990000	-1.51887300	7.95210700	L
H-H_	0	4.67218100	-2.98210200	9.00163800	L
H-H_	0	6.52544900	-0.61338300	9.16116300	L
H-H_	0	5.97936600	-1.74638500	10.46945200	L
H-H_	0	8.44186200	-1.81899200	8.90222500	L
H-H_	0	7.89943300	-3.00880700	10.16199000	L
C-C_3	0	-5.65926500	-7.18620300	5.66885000	L
O-O_3	0	-6.47556700	-7.70419500	4.65066800	L
C-C_3	0	-7.70736500	-7.02831400	4.74307600	L
C-C_3	0	-7.83045700	-6.38506600	6.11689900	L
C-C_3	0	-6.55005500	-6.75373800	6.80746600	L
H-H_	0	-4.94022500	-7.95999000	6.01091800	L
H-H_	0	-5.09204100	-6.31524000	5.27372600	L
H-H_	0	-7.76271000	-6.25626700	3.94543500	L
H-H_	0	-8.53138800	-7.75608300	4.58834500	L
H-H_	0	-7.91136200	-5.28127000	6.02366100	L
H-H_	0	-8.71159900	-6.77515700	6.67176000	L
H-H_	0	-6.11482300	-5.89668500	7.36096600	L
H-H_	0	-6.72002800	-7.60674800	7.50081300	L
C-C_3	0	-3.85394800	2.99604400	5.89613200	L
O-O_3	0	-3.84905500	1.76291400	6.57362900	L
C-C_3	0	-4.25294500	0.76961100	5.66070900	L
C-C_3	0	-4.36979500	1.36981100	4.27370500	L
C-C_3	0	-4.52048900	2.82938000	4.55026700	L
H-H_	0	-4.38493300	3.75558500	6.50341000	L
H-H_	0	-2.80703800	3.33134100	5.75841400	L
H-H_	0	-3.51904100	-0.06262000	5.66825800	L
H-H_	0	-5.23590000	0.36872700	5.98261400	L
H-H_	0	-3.43953800	1.18539300	3.69477700	L
H-H_	0	-5.24460000	0.96934900	3.71683500	L
H-H_	0	-4.03055400	3.45298000	3.77350500	L
H-H_	0	-5.59888600	3.08622200	4.62351400	L
C-C_3	0	0.22479300	-5.97076300	-5.94666600	L

O-O_3	0	-0.87660400	-5.10643800	-5.84967100	L
C-C_3	0	-0.69376200	-4.40197400	-4.64996700	L
C-C_3	0	-0.00130300	-5.32282100	-3.66581500	L
C-C_3	0	0.64350600	-6.36174600	-4.54280600	L
H-H_	0	-0.05343500	-6.86748700	-6.53663200	L
H-H_	0	1.05657800	-5.44671000	-6.46757900	L
H-H_	0	-0.06587500	-3.50739000	-4.84733300	L
H-H_	0	-1.67321900	-4.06370300	-4.25526400	L
H-H_	0	0.74612000	-4.77436900	-3.05481800	L
H-H_	0	-0.74262000	-5.80161200	-2.99266900	L
H-H_	0	1.74878500	-6.35075100	-4.44152800	L
H-H_	0	0.24973100	-7.36882400	-4.28948200	L
C-C_3	0	-6.03982700	-9.78685600	-0.59586800	L
O-O_3	0	-5.80058100	-10.08662100	0.76007300	L
C-C_3	0	-6.03850200	-11.46082600	0.94394000	L
C-C_3	0	-6.03462700	-12.14622800	-0.40281500	L
C-C_3	0	-6.43140800	-11.04951800	-1.33940800	L
H-H_	0	-6.84121700	-9.02354700	-0.66934300	L
H-H_	0	-5.11663700	-9.35796800	-1.03750500	L
H-H_	0	-5.25437100	-11.89277500	1.60052900	L
H-H_	0	-7.02573300	-11.59369400	1.43720200	L
H-H_	0	-5.00965900	-12.50344700	-0.64607200	L
H-H_	0	-6.74955600	-12.99691000	-0.43486300	L
H-H_	0	-5.90150600	-11.13576900	-2.31211000	L
H-H_	0	-7.53081400	-11.07146000	-1.50486400	L
C-C_3	0	7.77325600	-5.07186000	-7.57038700	L
O-O_3	0	7.82379000	-5.03451000	-8.97883600	L
C-C_3	0	8.97453300	-4.30839700	-9.34526400	L
C-C_3	0	9.51981600	-3.59419100	-8.12870000	L
C-C_3	0	9.02827200	-4.44156000	-7.00371500	L
H-H_	0	6.87959600	-4.50707800	-7.22737200	L
H-H_	0	7.67931400	-6.12388400	-7.22734800	L
H-H_	0	9.73538800	-5.01629400	-9.74016600	L
H-H_	0	8.72335100	-3.58046400	-10.14422900	L
H-H_	0	10.62953700	-3.53344500	-8.14353000	L
H-H_	0	9.08181200	-2.57568700	-8.05267100	L
H-H_	0	9.78059900	-5.22736300	-6.77367600	L
H-H_	0	8.81546200	-3.83932900	-6.09626900	L

C-C_3	0	9.34228900	4.42199200	-1.05676200	L
O-O_3	0	9.41703500	3.55150200	-2.16340800	L
C-C_3	0	8.16276500	3.55999700	-2.80293700	L
C-C_3	0	7.15138700	4.24269300	-1.90910800	L
C-C_3	0	7.99451000	5.11217600	-1.03508000	L
H-H_	0	9.48073100	3.82944700	-0.12876100	L
H-H_	0	10.15546300	5.17361800	-1.11587900	L
H-H_	0	8.25380900	4.10715500	-3.76451300	L
H-H_	0	7.84551000	2.51895400	-3.02438900	L
H-H_	0	6.41424400	4.83076300	-2.49250200	L
H-H_	0	6.62653300	3.49146600	-1.28571400	L
H-H_	0	8.07562200	6.12922800	-1.47366500	L
H-H_	0	7.58455100	5.18288700	-0.00484300	L
C-C_3	0	9.77930100	-5.00519300	-2.39098100	L
O-O_3	0	9.72568000	-5.08713400	-3.79069000	L
C-C_3	0	8.93249700	-6.20743800	-4.10474900	L
C-C_3	0	8.75588800	-7.07430200	-2.87092300	L
C-C_3	0	9.58060200	-6.39056000	-1.82428800	L
H-H_	0	10.76084900	-4.59595600	-2.07663300	L
H-H_	0	8.97692800	-4.32260300	-2.03626800	L
H-H_	0	7.94924800	-5.85949300	-4.48069100	L
H-H_	0	9.42770000	-6.79120800	-4.90900600	L
H-H_	0	7.68984700	-7.09308600	-2.56364400	L
H-H_	0	9.10974600	-8.11280200	-3.04939800	L
H-H_	0	9.05799900	-6.36043300	-0.84738100	L
H-H_	0	10.56037800	-6.90354200	-1.71196000	L
C-C_3	0	-3.85628800	-9.79307600	3.64932300	L
O-O_3	0	-2.92415200	-10.13899000	2.65131700	L
C-C_3	0	-2.37821100	-11.39312000	2.98774400	L
C-C_3	0	-2.79032200	-11.75902600	4.39776200	L
C-C_3	0	-4.02992900	-10.95029600	4.60948900	L
H-H_	0	-3.48188300	-8.90137000	4.19167600	L
H-H_	0	-4.82873900	-9.53415000	3.18276100	L
H-H_	0	-2.75920700	-12.15396200	2.27269200	L
H-H_	0	-1.27139200	-11.35518000	2.90252700	L
H-H_	0	-2.98437000	-12.84856500	4.50242600	L
H-H_	0	-2.00668600	-11.44523000	5.12026600	L
H-H_	0	-4.92612800	-11.54661600	4.32785900	L

H-H_	0	-4.12157800	-10.60314200	5.66183100	L
C-C_3	0	-4.76220100	-7.60680800	-6.58406000	L
O-O_3	0	-5.55660400	-8.19748600	-5.58140500	L
C-C_3	0	-4.84706900	-9.30302600	-5.08268200	L
C-C_3	0	-3.37622800	-9.09737200	-5.35424300	L
C-C_3	0	-3.36879800	-8.21260600	-6.56293800	L
H-H_	0	-5.24036800	-7.76704000	-7.57426700	L
H-H_	0	-4.70357600	-6.51389100	-6.39894000	L
H-H_	0	-5.02989100	-9.40516600	-3.99223500	L
H-H_	0	-5.20447100	-10.22449700	-5.59246300	L
H-H_	0	-2.89835000	-8.57186500	-4.50031900	L
H-H_	0	-2.85948100	-10.06318900	-5.54075800	L
H-H_	0	-2.58599500	-7.42810000	-6.47962100	L
H-H_	0	-3.19588100	-8.81643200	-7.48099400	L
C-C_3	0	5.19000000	8.57228700	5.07783800	L
O-O_3	0	6.54191300	8.48411300	4.69737400	L
C-C_3	0	7.25729600	9.44950800	5.43456800	L
C-C_3	0	6.30227800	10.23055700	6.31594400	L
C-C_3	0	5.09112300	9.35603400	6.36671900	L
H-H_	0	4.61993300	9.08671700	4.27528600	L
H-H_	0	4.77154800	7.55370700	5.20617900	L
H-H_	0	8.01393700	8.93120400	6.06167800	L
H-H_	0	7.78647200	10.13449000	4.73896100	L
H-H_	0	6.72439100	10.40723000	7.32917200	L
H-H_	0	6.04954500	11.20251900	5.83707600	L
H-H_	0	5.15731200	8.66992900	7.23812300	L
H-H_	0	4.15297200	9.94696200	6.42460200	L
C-C_3	0	-8.01156400	-5.93428000	-6.37265700	L
O-O_3	0	-8.07251700	-5.30131800	-7.63115100	L
C-C_3	0	-9.25479000	-4.53743400	-7.66730500	L
C-C_3	0	-9.78826600	-4.38622300	-6.26084300	L
C-C_3	0	-9.25442400	-5.59967900	-5.57056000	L
H-H_	0	-7.92555700	-7.03189300	-6.51615500	L
H-H_	0	-7.10908800	-5.57551600	-5.83357900	L
H-H_	0	-9.04507100	-3.54205200	-8.11234600	L
H-H_	0	-10.00261300	-5.06026400	-8.30260600	L
H-H_	0	-9.36461800	-3.47219700	-5.79505600	L
H-H_	0	-10.89892400	-4.34753400	-6.23860700	L

H-H_	0	-9.01877300	-5.39680100	-4.50280900	L
H-H_	0	-9.99004300	-6.43108100	-5.64340200	L
C-C_3	0	-6.52152900	-0.14783200	-11.38118700	L
O-O_3	0	-5.55790000	0.50495400	-10.59167900	L
C-C_3	0	-4.58442300	-0.44829400	-10.23072900	L
C-C_3	0	-4.98602100	-1.81433100	-10.75484300	L
C-C_3	0	-6.41981900	-1.63582900	-11.14345200	L
H-H_	0	-7.53594200	0.21769700	-11.11636800	L
H-H_	0	-6.32859900	0.08356600	-12.45164400	L
H-H_	0	-3.60077600	-0.14501000	-10.64674000	L
H-H_	0	-4.50151300	-0.47861400	-9.12370600	L
H-H_	0	-4.37626100	-2.07525000	-11.64811900	L
H-H_	0	-4.87604100	-2.60134700	-9.98026900	L
H-H_	0	-6.67904100	-2.21885200	-12.05366500	L
H-H_	0	-7.08284700	-1.93229400	-10.30102400	L
C-C_3	0	-9.08623000	-3.16838300	4.15481000	L
O-O_3	0	-9.61150600	-3.23451800	5.45986200	L
C-C_3	0	-10.15552600	-1.97076400	5.76651400	L
C-C_3	0	-10.05550200	-1.06905800	4.55400900	L
C-C_3	0	-8.99022300	-1.72201500	3.73330100	L
H-H_	0	-8.08803200	-3.65091700	4.12425500	L
H-H_	0	-9.76420300	-3.71390800	3.46532700	L
H-H_	0	-11.21454300	-2.08397500	6.08128700	L
H-H_	0	-9.58461900	-1.53132900	6.61017900	L
H-H_	0	-11.01909900	-1.06899500	4.00247900	L
H-H_	0	-9.78356600	-0.02893200	4.82753500	L
H-H_	0	-9.16658200	-1.59838400	2.64322800	L
H-H_	0	-7.99538400	-1.30997500	4.01027000	L
C-C_3	0	10.28039600	-2.68616200	4.88650300	L
O-O_3	0	9.22806000	-3.61788700	4.79052800	L
C-C_3	0	9.74993000	-4.88389400	5.12041100	L
C-C_3	0	11.15010800	-4.72365900	5.67117800	L
C-C_3	0	11.58851700	-3.41961100	5.08703000	L
H-H_	0	10.32191500	-2.06941200	3.96572200	L
H-H_	0	10.08420000	-2.02083100	5.75192600	L
H-H_	0	9.09113100	-5.38145700	5.86331500	L
H-H_	0	9.78279600	-5.50418300	4.20166900	L
H-H_	0	11.11609800	-4.65203700	6.78097800	L

H-H_	0	11.81472000	-5.55998300	5.36307300	L
H-H_	0	12.27416400	-2.86988600	5.76641900	L
H-H_	0	12.08540800	-3.58799600	4.10614300	L
C-C_3	0	-5.20907300	5.92077700	8.70274400	L
O-O_3	0	-6.42111700	6.45349000	9.17948200	L
C-C_3	0	-6.64500100	5.92140100	10.46526700	L
C-C_3	0	-5.51150300	4.98420100	10.83794900	L
C-C_3	0	-4.84084300	4.70964500	9.52885200	L
H-H_	0	-5.31831900	5.64700300	7.63339000	L
H-H_	0	-4.41704500	6.69421200	8.79472800	L
H-H_	0	-6.72077300	6.74933300	11.20221900	L
H-H_	0	-7.60863900	5.36854100	10.46000500	L
H-H_	0	-4.80398600	5.49708600	11.52655200	L
H-H_	0	-5.88844800	4.05268900	11.31225700	L
H-H_	0	-3.74173700	4.60202400	9.64196700	L
H-H_	0	-5.25864100	3.79029700	9.06717800	L
C-C_3	0	-6.34259800	6.05491500	4.45597500	L
O-O_3	0	-6.37659100	7.43298500	4.72390400	L
C-C_3	0	-7.35998900	7.63879700	5.71153500	L
C-C_3	0	-8.19956300	6.38114300	5.87054800	L
C-C_3	0	-7.69145200	5.46687800	4.79722200	L
H-H_	0	-6.10232500	5.88662600	3.38557100	L
H-H_	0	-5.55184800	5.58574500	5.07884800	L
H-H_	0	-6.86722200	7.90027700	6.67252300	L
H-H_	0	-8.00399900	8.49086800	5.40627500	L
H-H_	0	-8.02795200	5.92960700	6.87182200	L
H-H_	0	-9.28315300	6.59283300	5.73883700	L
H-H_	0	-7.60751700	4.41881800	5.15451000	L
H-H_	0	-8.36029000	5.50932200	3.91095000	L
C-C_3	0	2.18510300	-10.39971700	7.14632900	L
O-O_3	0	1.13595700	-9.78900800	7.85821400	L
C-C_3	0	0.10120800	-10.73895600	7.98501600	L
C-C_3	0	0.49215200	-12.02052500	7.27130000	L
C-C_3	0	1.63925800	-11.59960600	6.40811700	L
H-H_	0	2.63019800	-9.67543600	6.43415500	L
H-H_	0	2.97232300	-10.71832900	7.86416800	L
H-H_	0	-0.09230900	-10.93651700	9.06106700	L
H-H_	0	-0.82473300	-10.32454900	7.53372300	L

H-H_	0	0.83204500	-12.77942800	8.01056500	L
H-H_	0	-0.34495000	-12.43388400	6.66869700	L
H-H_	0	2.39990300	-12.40357100	6.30490000	L
H-H_	0	1.27049800	-11.29397100	5.40397400	L
C-C_3	0	4.10891500	5.77039100	-7.03128300	L
O-O_3	0	5.08009900	6.71692700	-7.40269800	L
C-C_3	0	6.07098500	6.72023900	-6.40026700	L
C-C_3	0	5.64108000	5.83304400	-5.24513300	L
C-C_3	0	4.20754000	5.52575000	-5.54279400	L
H-H_	0	3.09809600	6.14579500	-7.29516800	L
H-H_	0	4.29645900	4.82819400	-7.58955000	L
H-H_	0	7.03344600	6.36550300	-6.82507400	L
H-H_	0	6.21694100	7.76209600	-6.04669300	L
H-H_	0	6.23509700	4.89475200	-5.23572400	L
H-H_	0	5.75210500	6.35105800	-4.26999400	L
H-H_	0	3.94781100	4.47988900	-5.27522200	L
H-H_	0	3.54345100	6.22829600	-4.99465700	L
C-C_3	0	-6.55409400	10.24960500	0.18520300	L
O-O_3	0	-6.76963900	10.83237500	1.45019500	L
C-C_3	0	-5.98736200	10.12659000	2.38598800	L
C-C_3	0	-5.45200100	8.86549500	1.74333800	L
C-C_3	0	-5.46360600	9.20494000	0.28973600	L
H-H_	0	-7.49903200	9.77994600	-0.16056200	L
H-H_	0	-6.27167700	11.03401700	-0.54641400	L
H-H_	0	-5.14287300	10.77433300	2.70719100	L
H-H_	0	-6.60281200	9.87767300	3.27604900	L
H-H_	0	-4.43074800	8.61584700	2.10051100	L
H-H_	0	-6.13637600	8.01291100	1.93906700	L
H-H_	0	-4.48695300	9.64523500	0.00232100	L
H-H_	0	-5.67851500	8.31807500	-0.34008100	L
C-C_3	0	-0.32429700	5.20641600	9.94054700	L
O-O_3	0	-0.70974200	3.88066400	9.68009200	L
C-C_3	0	-1.06808500	3.81836100	8.32012600	L
C-C_3	0	-1.29825500	5.22335400	7.78759800	L
C-C_3	0	-1.04139000	6.11220100	8.96972300	L
H-H_	0	-0.58278300	5.47738300	10.98511500	L
H-H_	0	0.77538400	5.30233200	9.81528100	L
H-H_	0	-0.25742300	3.31743100	7.74977800	L

H-H_	0	-1.99294000	3.21404000	8.21818900	L
H-H_	0	-0.57873500	5.44643200	6.97089600	L
H-H_	0	-2.33629100	5.35218700	7.41028600	L
H-H_	0	-0.41861000	6.99268900	8.70115500	L
H-H_	0	-2.00294900	6.45292600	9.41116100	L
C-C_3	0	12.76915000	3.83344000	-2.35835400	L
O-O_3	0	14.06603100	3.97953200	-2.88994900	L
C-C_3	0	14.97295100	4.01100500	-1.81172600	L
C-C_3	0	14.25425200	3.62739200	-0.53621000	L
C-C_3	0	12.82588900	3.93603500	-0.84870000	L
H-H_	0	12.10484400	4.61963600	-2.77610100	L
H-H_	0	12.36698700	2.84081400	-2.65461900	L
H-H_	0	15.81267600	3.31075200	-2.00822700	L
H-H_	0	15.38368100	5.03983300	-1.71899000	L
H-H_	0	14.37756800	2.54063300	-0.34228500	L
H-H_	0	14.62004900	4.20852100	0.33813300	L
H-H_	0	12.13185800	3.21805900	-0.36126300	L
H-H_	0	12.58738200	4.97495700	-0.53077600	L
C-C_3	0	10.19521000	-0.03513000	-2.91504400	L
O-O_3	0	10.30391500	0.88017600	-3.98188300	L
C-C_3	0	10.45677100	0.13791600	-5.16835300	L
C-C_3	0	10.80678400	-1.29143900	-4.81863000	L
C-C_3	0	10.22981800	-1.45328600	-3.44844800	L
H-H_	0	11.04320100	0.12679000	-2.21679900	L
H-H_	0	9.24794600	0.14480500	-2.36735400	L
H-H_	0	9.50280000	0.16866500	-5.73675700	L
H-H_	0	11.25460500	0.59074500	-5.79444500	L
H-H_	0	10.37137400	-2.01263900	-5.54181200	L
H-H_	0	11.91036900	-1.41487000	-4.78250700	L
H-H_	0	9.19973000	-1.86306500	-3.51433600	L
H-H_	0	10.85135400	-2.11824700	-2.81240800	L
C-C_3	0	-8.10660400	1.29042300	6.81253300	L
O-O_3	0	-9.23913300	0.98142600	7.58840500	L
C-C_3	0	-9.29170800	1.89702600	8.65818200	L
C-C_3	0	-8.11790900	2.85238900	8.57561600	L
C-C_3	0	-7.19026300	2.18884400	7.60805900	L
H-H_	0	-7.58710700	0.35297500	6.52398000	L
H-H_	0	-8.43047000	1.81022700	5.88745100	L

H-H_	0	-10.25047200	2.45698100	8.62155800	L
H-H_	0	-9.25248200	1.33295400	9.61453500	L
H-H_	0	-8.45113200	3.83415400	8.17362200	L
H-H_	0	-7.63828800	2.99480000	9.56754200	L
H-H_	0	-6.67609500	2.92967600	6.96101600	L
H-H_	0	-6.44079400	1.57778300	8.15338400	L
C-C_3	0	8.83212100	0.88579400	4.73551000	L
O-O_3	0	8.68687600	1.57479500	5.95605400	L
C-C_3	0	7.60472100	0.99414700	6.64465300	L
C-C_3	0	7.22000200	-0.29972600	5.96641500	L
C-C_3	0	7.68575500	-0.08705600	4.56451300	L
H-H_	0	8.85812800	1.60881500	3.89841300	L
H-H_	0	9.79454700	0.33609600	4.74747900	L
H-H_	0	7.88582600	0.81169600	7.70240800	L
H-H_	0	6.74876200	1.69846300	6.62285300	L
H-H_	0	7.77772900	-1.14502000	6.42178800	L
H-H_	0	6.12711700	-0.48808600	6.02063800	L
H-H_	0	8.01424600	-1.03394700	4.08832400	L
H-H_	0	6.87405200	0.37330200	3.96279200	L
C-C_3	0	-11.11723800	3.91785500	5.94292100	L
O-O_3	0	-11.42032900	4.67940200	4.79715300	L
C-C_3	0	-12.56517800	4.10598400	4.21847500	L
C-C_3	0	-12.59825600	2.63968200	4.58087800	L
C-C_3	0	-11.86723400	2.59511600	5.88990800	L
H-H_	0	-10.02288400	3.73776100	5.97917900	L
H-H_	0	-11.40388300	4.49028200	6.85153000	L
H-H_	0	-13.47002200	4.61970500	4.61208900	L
H-H_	0	-12.53664800	4.23407800	3.11645800	L
H-H_	0	-13.64000400	2.26296100	4.67320900	L
H-H_	0	-12.04989100	2.04520300	3.81838900	L
H-H_	0	-12.58773700	2.52673500	6.73448700	L
H-H_	0	-11.17305000	1.72955200	5.92485300	L
C-C_3	0	0.23066900	-7.31977000	10.17065200	L
O-O_3	0	-0.98676500	-7.05443400	10.83101700	L
C-C_3	0	-0.73949100	-6.06021800	11.79827600	L
C-C_3	0	0.75263300	-5.92390300	11.99452700	L
C-C_3	0	1.29661200	-6.36830500	10.67487400	L
H-H_	0	0.53092500	-8.36883000	10.38144900	L

H-H_	0	0.09592500	-7.20555500	9.07469900	L
H-H_	0	-1.16143800	-5.09507600	11.44208400	L
H-H_	0	-1.23449900	-6.33352700	12.75412300	L
H-H_	0	1.04540400	-4.88136400	12.24064500	L
H-H_	0	1.09447900	-6.61323100	12.79817900	L
H-H_	0	1.38260600	-5.50010700	9.98583400	L
H-H_	0	2.28389600	-6.86744400	10.78283400	L
C-C_3	0	11.18703700	-0.93690800	-10.26496900	L
O-O_3	0	10.13928500	-0.60768700	-11.14509000	L
C-C_3	0	9.05158300	-0.22343000	-10.34379100	L
C-C_3	0	9.58917900	0.36017800	-9.05647900	L
C-C_3	0	10.96252100	-0.23865800	-8.93336900	L
H-H_	0	11.21464400	-2.03933900	-10.12611300	L
H-H_	0	12.15346700	-0.61646000	-10.70901200	L
H-H_	0	8.43400600	0.52636700	-10.88253200	L
H-H_	0	8.42040000	-1.11185100	-10.13101100	L
H-H_	0	9.66785700	1.46442300	-9.14999600	L
H-H_	0	8.94097300	0.09642600	-8.19246000	L
H-H_	0	11.72314800	0.55577800	-8.76754100	L
H-H_	0	11.00710700	-0.96561800	-8.09403800	L
C-C_3	0	6.35543600	-2.79527300	-4.45528600	L
O-O_3	0	5.67736200	-2.41346200	-3.28635100	L
C-C_3	0	6.12773500	-1.11860700	-2.97783700	L
C-C_3	0	6.51050300	-0.42329800	-4.26692700	L
C-C_3	0	6.67231600	-1.54727800	-5.25685900	L
H-H_	0	7.29009300	-3.32834100	-4.17609400	L
H-H_	0	5.71985000	-3.48765300	-5.04698300	L
H-H_	0	5.32217600	-0.55927400	-2.45835800	L
H-H_	0	7.00654300	-1.18350500	-2.29938600	L
H-H_	0	5.69809900	0.26444700	-4.58255700	L
H-H_	0	7.45158000	0.15295200	-4.14681200	L
H-H_	0	5.95157000	-1.43268300	-6.09324900	L
H-H_	0	7.70451400	-1.58661500	-5.66535400	L
C-C_3	0	2.30510900	5.30509200	4.96922300	L
O-O_3	0	3.01490100	5.44960300	6.17164700	L
C-C_3	0	2.44503200	6.55222700	6.83555700	L
C-C_3	0	1.71804200	7.42797800	5.82474600	L
C-C_3	0	1.84462200	6.67576800	4.53093300	L

H-H_	0	2.95575900	4.84649700	4.19651800	L
H-H_	0	1.43086700	4.63977900	5.14092800	L
H-H_	0	1.73937100	6.18619700	7.61020800	L
H-H_	0	3.24619900	7.12784700	7.34289300	L
H-H_	0	0.64924300	7.54269500	6.10687900	L
H-H_	0	2.18675700	8.43200700	5.74536200	L
H-H_	0	0.87945300	6.62883900	3.98705900	L
H-H_	0	2.61711200	7.14817700	3.88515500	L
C-C_3	0	2.15199100	-1.24853600	9.84661400	L
O-O_3	0	1.45849000	-0.08143000	10.22578900	L
C-C_3	0	0.33067300	-0.47973600	10.96578000	L
C-C_3	0	0.52759800	-1.90293800	11.43426600	L
C-C_3	0	1.43723700	-2.47009000	10.39157800	L
H-H_	0	2.22495000	-1.29758300	8.73909000	L
H-H_	0	3.17878100	-1.20277800	10.26683600	L
H-H_	0	0.19468700	0.19548700	11.83516800	L
H-H_	0	-0.56659400	-0.41173800	10.31480900	L
H-H_	0	1.03375500	-1.91017500	12.42490200	L
H-H_	0	-0.43417200	-2.45693200	11.49611800	L
H-H_	0	2.15411800	-3.20212200	10.82182800	L
H-H_	0	0.83660400	-2.95633800	9.59366500	L
C-C_3	0	5.96252600	1.96874300	-7.00905800	L
O-O_3	0	7.07421900	2.23591900	-6.18609400	L
C-C_3	0	7.96286100	3.04979600	-6.91743300	L
C-C_3	0	7.34784500	3.39688700	-8.25990500	L
C-C_3	0	6.29052400	2.35469000	-8.43316300	L
H-H_	0	5.70161800	0.89209200	-6.95645400	L
H-H_	0	5.09716800	2.55970300	-6.64269600	L
H-H_	0	8.18812300	3.97297900	-6.34358200	L
H-H_	0	8.91417900	2.49665900	-7.06301500	L
H-H_	0	6.87897300	4.40306500	-8.21877400	L
H-H_	0	8.09539500	3.36763800	-9.08141000	L
H-H_	0	5.40106000	2.74966700	-8.97050500	L
H-H_	0	6.70548100	1.47913100	-8.97891700	L
C-C_3	0	-2.85357900	7.67956000	-1.55510100	L
O-O_3	0	-2.59103600	9.05023100	-1.72588600	L
C-C_3	0	-1.67847300	9.16281600	-2.78505700	L
C-C_3	0	-0.87219900	7.88569800	-2.86972600	L

C-C_3	0	-1.74096300	6.86566400	-2.18935700	L
H-H_	0	-2.92115200	7.45527800	-0.46959100	L
H-H_	0	-3.82918200	7.43444000	-2.02474100	L
H-H_	0	-2.23722100	9.33418800	-3.72763700	L
H-H_	0	-1.01226000	10.03145400	-2.60576100	L
H-H_	0	-0.65749700	7.60802300	-3.92353400	L
H-H_	0	0.08451800	8.00646500	-2.31886800	L
H-H_	0	-2.15986400	6.14407300	-2.92266100	L
H-H_	0	-1.16870000	6.30958500	-1.41684300	L
C-C_3	0	-4.92953300	-3.08746600	5.49968500	L
O-O_3	0	-3.70714100	-3.78728700	5.53661700	L
C-C_3	0	-3.13641600	-3.69796200	4.25614400	L
C-C_3	0	-3.73708900	-2.51001300	3.54799500	L
C-C_3	0	-5.10976800	-2.44880700	4.13688400	L
H-H_	0	-5.76450700	-3.78833000	5.70922400	L
H-H_	0	-4.91222500	-2.30170100	6.28353300	L
H-H_	0	-2.03718300	-3.59450000	4.33491600	L
H-H_	0	-3.35994600	-4.63209700	3.69936200	L
H-H_	0	-3.17021100	-1.58794200	3.79697000	L
H-H_	0	-3.75722500	-2.65356700	2.45193800	L
H-H_	0	-5.48594600	-1.40868000	4.20796700	L
H-H_	0	-5.81330200	-3.05353400	3.52848000	L
C-C_3	0	-12.88114900	3.77322900	-1.83282100	L
O-O_3	0	-12.42114900	4.91255400	-1.14418700	L
C-C_3	0	-12.22067800	5.93621300	-2.09313000	L
C-C_3	0	-12.69181300	5.46728300	-3.45451000	L
C-C_3	0	-12.69226300	3.97838000	-3.31978000	L
H-H_	0	-12.32274500	2.87803200	-1.49070200	L
H-H_	0	-13.95889900	3.62533800	-1.60420000	L
H-H_	0	-12.77618600	6.84679400	-1.78313400	L
H-H_	0	-11.13763500	6.18253100	-2.13346000	L
H-H_	0	-13.72536900	5.83150100	-3.64662300	L
H-H_	0	-12.01386600	5.80892300	-4.26606600	L
H-H_	0	-13.50661500	3.50682800	-3.91144800	L
H-H_	0	-11.70998800	3.57018300	-3.63823100	L
C-C_3	0	-5.32634800	3.95006100	-7.83595100	L
O-O_3	0	-5.33835500	3.93288300	-6.42741900	L
C-C_3	0	-4.44618800	2.92691100	-6.00515500	L

C-C_3	0	-4.00094000	2.11605000	-7.20199200	L
C-C_3	0	-4.21123600	3.06010200	-8.33776300	L
H-H_	0	-6.30113400	3.57267700	-8.20482500	L
H-H_	0	-5.19118800	4.99011800	-8.20091100	L
H-H_	0	-3.56874200	3.41044300	-5.52542100	L
H-H_	0	-4.94168600	2.26847100	-5.26121000	L
H-H_	0	-2.94301000	1.79320600	-7.12367800	L
H-H_	0	-4.65492000	1.22761900	-7.32201000	L
H-H_	0	-3.29165000	3.65910700	-8.50698900	L
H-H_	0	-4.49146800	2.52768700	-9.26863900	L
C-C_3	0	-10.32689000	-10.61303700	-3.32550200	L
O-O_3	0	-9.35426600	-11.27757600	-4.09930600	L
C-C_3	0	-8.39135800	-10.32325800	-4.48600000	L
C-C_3	0	-8.65636800	-9.02063800	-3.76195900	L
C-C_3	0	-10.10780500	-9.11942500	-3.42500900	L
H-H_	0	-11.34256100	-10.88074300	-3.68665200	L
H-H_	0	-10.22866300	-10.94073000	-2.26791700	L
H-H_	0	-7.37559500	-10.70090400	-4.25029300	L
H-H_	0	-8.46254500	-10.16514000	-5.58409800	L
H-H_	0	-8.05254100	-8.96778600	-2.83118000	L
H-H_	0	-8.43874300	-8.14036100	-4.39916600	L
H-H_	0	-10.34858800	-8.60000200	-2.47504800	L
H-H_	0	-10.71926500	-8.70042000	-4.25438700	L
C-C_3	0	-0.37554200	-7.75484800	3.52331300	L
O-O_3	0	-1.49441400	-6.92169200	3.68633300	L
C-C_3	0	-1.61408000	-6.70279500	5.06720000	L
C-C_3	0	-1.08176000	-7.91874800	5.79425500	L
C-C_3	0	-0.20071600	-8.58974500	4.77645600	L
H-H_	0	-0.52696100	-8.40863000	2.63917100	L
H-H_	0	0.52554100	-7.12521500	3.35772700	L
H-H_	0	-1.03025800	-5.79909000	5.34313000	L
H-H_	0	-2.67782600	-6.53414900	5.32633700	L
H-H_	0	-0.51110200	-7.62621800	6.70068900	L
H-H_	0	-1.91927800	-8.58993800	6.08495200	L
H-H_	0	0.86006000	-8.59193200	5.09952700	L
H-H_	0	-0.53057500	-9.63386200	4.60111700	L
C-C_3	0	3.21176000	-6.90126300	7.42832800	L
O-O_3	0	2.28344000	-6.75407200	6.38420900	L

C-C_3	0	1.73848300	-5.46688400	6.51884400	L
C-C_3	0	2.77149200	-4.56937200	7.16679200	L
C-C_3	0	3.72584900	-5.53024300	7.82484300	L
H-H_	0	2.71634600	-7.39587600	8.29090700	L
H-H_	0	4.05022600	-7.54665200	7.09030900	L
H-H_	0	1.45668600	-5.07882000	5.51822100	L
H-H_	0	0.82663900	-5.51932300	7.15137700	L
H-H_	0	3.29566400	-3.96204500	6.39732000	L
H-H_	0	2.30068700	-3.89066200	7.90767500	L
H-H_	0	4.75788600	-5.38021700	7.44246600	L
H-H_	0	3.72199900	-5.40981400	8.92986700	L
C-C_3	0	2.23467300	-10.38472900	2.03357900	L
O-O_3	0	2.93324000	-11.31925000	2.82454700	L
C-C_3	0	3.95246900	-10.62329600	3.50591400	L
C-C_3	0	3.75993100	-9.13542900	3.30926000	L
C-C_3	0	2.98326300	-9.06960100	2.03578700	L
H-H_	0	2.13045800	-10.76954100	0.99808800	L
H-H_	0	1.21883800	-10.24354900	2.46099600	L
H-H_	0	3.92952900	-10.87996600	4.58529100	L
H-H_	0	4.93578900	-10.93396400	3.09307400	L
H-H_	0	3.16129700	-8.71178600	4.14473100	L
H-H_	0	4.72696400	-8.59984700	3.22786300	L
H-H_	0	2.29395300	-8.20052800	2.01074100	L
H-H_	0	3.68259100	-9.02432300	1.17204500	L
C-C_3	0	0.05863700	1.61280200	-7.60500800	L
O-O_3	0	0.79599100	2.42410100	-6.73038500	L
C-C_3	0	1.83454500	2.98036300	-7.49447900	L
C-C_3	0	2.15520500	2.04527600	-8.64473900	L
C-C_3	0	0.99449500	1.08910200	-8.67487200	L
H-H_	0	-0.39998700	0.77707800	-7.03751400	L
H-H_	0	-0.75351000	2.21614000	-8.06298700	L
H-H_	0	1.51789600	3.97291900	-7.88068800	L
H-H_	0	2.72411700	3.11753900	-6.85013700	L
H-H_	0	2.23778300	2.60817900	-9.60015800	L
H-H_	0	3.10516100	1.50036700	-8.45112200	L
H-H_	0	0.50104200	1.07342800	-9.67018500	L
H-H_	0	1.33723800	0.06624100	-8.41612400	L
C-C_3	0	-3.79038100	9.41353500	5.33854400	L

O-O_3	0	-2.83603900	10.44471800	5.42656400	L
C-C_3	0	-1.95325500	10.12331500	6.47745200	L
C-C_3	0	-2.36842100	8.81127700	7.11345600	L
C-C_3	0	-3.76040300	8.59985400	6.61038000	L
H-H_	0	-4.79944900	9.84983300	5.18471500	L
H-H_	0	-3.54618200	8.76545600	4.46968300	L
H-H_	0	-0.91666600	10.05008100	6.08506000	L
H-H_	0	-1.98605400	10.93720000	7.23319400	L
H-H_	0	-1.70878900	7.99327200	6.75608300	L
H-H_	0	-2.33784600	8.86169100	8.22352900	L
H-H_	0	-3.97086000	7.52648700	6.41509500	L
H-H_	0	-4.49576800	9.00582500	7.33973100	L
C-C_3	0	-1.58915200	10.41486900	2.21400100	L
O-O_3	0	-1.61760300	9.87706100	0.91350500	L
C-C_3	0	-0.43509100	9.13329600	0.73141500	L
C-C_3	0	0.47714000	9.33136100	1.92217200	L
C-C_3	0	-0.46286900	9.77495000	2.99511600	L
H-H_	0	-1.42812500	11.51241300	2.14706500	L
H-H_	0	-2.56301100	10.23410800	2.71115200	L
H-H_	0	-0.69888800	8.06100400	0.62915600	L
H-H_	0	0.07041400	9.45476400	-0.20159000	L
H-H_	0	1.00853500	8.39617300	2.19780300	L
H-H_	0	1.21381900	10.13423400	1.70285200	L
H-H_	0	-0.83840800	8.89650200	3.56059400	L
H-H_	0	0.01341600	10.49078000	3.69416600	L
C-C_3	0	5.84379600	-8.03578100	-5.40644900	L
O-O_3	0	5.57614300	-6.65722900	-5.28671200	L
C-C_3	0	4.98232300	-6.43952200	-4.02968600	L
C-C_3	0	4.56521500	-7.76689700	-3.43769800	L
C-C_3	0	5.46148600	-8.74689300	-4.12421600	L
H-H_	0	5.25227500	-8.44211300	-6.25366100	L
H-H_	0	6.92199100	-8.18787200	-5.62661500	L
H-H_	0	5.71858100	-5.93951400	-3.36646900	L
H-H_	0	4.10557400	-5.76864400	-4.14100800	L
H-H_	0	4.69907200	-7.78598100	-2.33460900	L
H-H_	0	3.50702200	-7.98133300	-3.69840300	L
H-H_	0	6.36633900	-8.93630200	-3.50764500	L
H-H_	0	4.93960100	-9.70754800	-4.32563900	L

C-C_3	0	-0.88354200	3.78636300	-10.92449100	L
O-O_3	0	-1.75168700	4.89444000	-10.93141100	L
C-C_3	0	-2.95909700	4.48755100	-11.53561700	L
C-C_3	0	-2.86440600	3.02755100	-11.93929000	L
C-C_3	0	-1.68889500	2.52779200	-11.16000200	L
H-H_	0	-0.13120700	3.91287100	-11.73333400	L
H-H_	0	-0.34867300	3.73434300	-9.95369200	L
H-H_	0	-3.78707000	4.62756400	-10.80908500	L
H-H_	0	-3.16400200	5.12282000	-12.42365700	L
H-H_	0	-3.79100600	2.46682000	-11.69118300	L
H-H_	0	-2.65901700	2.94670000	-13.02966700	L
H-H_	0	-2.02724500	2.10144900	-10.19065000	L
H-H_	0	-1.11002100	1.76521600	-11.72488800	L
C-C_3	0	12.30554900	0.29634900	2.97378800	L
O-O_3	0	13.12506500	-0.66773000	3.59385100	L
C-C_3	0	14.22640900	-0.90423800	2.74828500	L
C-C_3	0	13.98555600	-0.24023200	1.40934000	L
C-C_3	0	12.99495000	0.83004300	1.73608800	L
H-H_	0	11.34519400	-0.18078200	2.69234100	L
H-H_	0	12.09258600	1.12037500	3.68629200	L
H-H_	0	15.13847400	-0.48044200	3.22177200	L
H-H_	0	14.37031100	-1.99798000	2.61907700	L
H-H_	0	14.92119800	0.17936000	0.98018700	L
H-H_	0	13.53728700	-0.96661900	0.69767300	L
H-H_	0	13.52425400	1.77854900	1.97584400	L
H-H_	0	12.27967500	0.99523500	0.90173000	L
C-C_3	0	-11.05024000	6.86886400	1.20801500	L
O-O_3	0	-9.94551900	6.39935500	0.47003000	L
C-C_3	0	-8.78607900	6.98393000	1.01775200	L
C-C_3	0	-9.18305600	8.01958500	2.04835800	L
C-C_3	0	-10.55603000	7.58397200	2.44634900	L
H-H_	0	-11.63219500	7.57191900	0.57345600	L
H-H_	0	-11.70693200	6.01768900	1.48524600	L
H-H_	0	-8.17841200	6.19191700	1.50255200	L
H-H_	0	-8.18147500	7.44357100	0.20870800	L
H-H_	0	-8.48829000	8.03221700	2.91384900	L
H-H_	0	-9.22446200	9.02775800	1.58068300	L
H-H_	0	-10.49742700	6.87243100	3.29910500	L

H-H_	0	-11.20343000	8.44670900	2.71476100	L
C-C_3	0	2.19483400	9.04978200	-5.45834700	L
O-O_3	0	3.56664400	8.87678300	-5.71409100	L
C-C_3	0	4.27071500	9.42421200	-4.62156700	L
C-C_3	0	3.29495100	10.06120000	-3.65173900	L
C-C_3	0	2.03122100	10.15381600	-4.44355500	L
H-H_	0	1.66521600	9.30537700	-6.39938700	L
H-H_	0	1.77729900	8.10116600	-5.05624000	L
H-H_	0	4.85104600	8.62747000	-4.11332800	L
H-H_	0	4.98221000	10.18862100	-5.00093200	L
H-H_	0	3.13873000	9.39878800	-2.77309100	L
H-H_	0	3.64577200	11.05939100	-3.31317100	L
H-H_	0	1.13371900	10.00029400	-3.80982200	L
H-H_	0	1.97679700	11.13773100	-4.95908600	L
C-C_3	0	-0.93476100	-11.24021800	-0.58072600	L
O-O_3	0	-0.20604500	-10.83768000	-1.71221600	L
C-C_3	0	-1.14479100	-10.39479900	-2.66346900	L
C-C_3	0	-2.46774600	-10.10230600	-1.97429000	L
C-C_3	0	-2.21918000	-10.44695100	-0.53524800	L
H-H_	0	-1.15626800	-12.32787300	-0.65448900	L
H-H_	0	-0.33600000	-11.06014700	0.33655500	L
H-H_	0	-0.75877300	-9.47775700	-3.15624900	L
H-H_	0	-1.27855900	-11.17950600	-3.43906300	L
H-H_	0	-2.75148500	-9.03320800	-2.08062900	L
H-H_	0	-3.27255000	-10.74390700	-2.39561300	L
H-H_	0	-2.07031900	-9.52123000	0.06085000	L
H-H_	0	-3.05119500	-11.04134400	-0.10236000	L
C-C_3	0	11.62608800	-1.45761500	8.56249700	L
O-O_3	0	12.99841800	-1.55892000	8.25442300	L
C-C_3	0	13.34252900	-0.44995800	7.45651100	L
C-C_3	0	12.07995500	0.21925900	6.96244800	L
C-C_3	0	11.06994700	-0.16689900	7.99530100	L
H-H_	0	11.09364100	-2.32638900	8.11886100	L
H-H_	0	11.48693400	-1.48777700	9.66420900	L
H-H_	0	13.93502300	0.26423400	8.06885600	L
H-H_	0	13.96472300	-0.78346400	6.59857300	L
H-H_	0	12.19509800	1.32221500	6.88477100	L
H-H_	0	11.79491400	-0.20140800	5.97547000	L

H-H_	0	11.01921900	0.61080400	8.78917500	L
H-H_	0	10.06536700	-0.31655600	7.54967100	L
C-C_3	0	7.96308400	4.30141100	8.54046800	L
O-O_3	0	8.76644800	4.76129100	7.48391000	L
C-C_3	0	8.24001200	6.00779400	7.09165600	L
C-C_3	0	6.82039400	6.14767700	7.62100600	L
C-C_3	0	6.57324300	4.86204500	8.35376700	L
H-H_	0	7.93992200	3.19261300	8.54348900	L
H-H_	0	8.39192600	4.64975600	9.50623200	L
H-H_	0	8.88427800	6.82036500	7.49210900	L
H-H_	0	8.24633300	6.07509700	5.98354400	L
H-H_	0	6.75261200	7.01123900	8.31848100	L
H-H_	0	6.08895200	6.27626700	6.79618600	L
H-H_	0	6.06181000	5.02782400	9.32671300	L
H-H_	0	5.96962400	4.17524200	7.72242200	L
C-C_3	0	6.70236900	-5.15108800	1.05034700	L
O-O_3	0	6.89042600	-3.76764100	0.86107900	L
C-C_3	0	6.17807500	-3.41240700	-0.29634500	L
C-C_3	0	5.08993900	-4.43179700	-0.51820500	L
C-C_3	0	5.70583600	-5.67865900	0.03265800	L
H-H_	0	7.67601400	-5.67367800	0.96377500	L
H-H_	0	6.31068300	-5.31868800	2.06986100	L
H-H_	0	5.73828100	-2.40215200	-0.17963100	L
H-H_	0	6.87857300	-3.40666200	-1.15806700	L
H-H_	0	4.19388200	-4.15201900	0.07562700	L
H-H_	0	4.81945800	-4.53002100	-1.59065200	L
H-H_	0	4.94896200	-6.34469500	0.50047700	L
H-H_	0	6.23776100	-6.22464400	-0.77560400	L
C-C_3	0	-3.96502100	-3.38957600	-7.09510800	L
O-O_3	0	-5.32773600	-3.71237900	-7.24148800	L
C-C_3	0	-6.05734500	-2.56561200	-6.88681900	L
C-C_3	0	-5.23866100	-1.75855300	-5.90736700	L
C-C_3	0	-3.82532200	-2.14528100	-6.23399300	L
H-H_	0	-3.43850600	-4.24452200	-6.62071900	L
H-H_	0	-3.51607500	-3.21969800	-8.09644900	L
H-H_	0	-6.26249900	-1.97102200	-7.80151400	L
H-H_	0	-7.02351600	-2.85903700	-6.42780400	L
H-H_	0	-5.41056500	-0.66856300	-6.03215400	L

H-H_	0	-5.48996800	-2.05472800	-4.86556400	L
H-H_	0	-3.32359800	-1.33129700	-6.80149000	L
H-H_	0	-3.24831400	-2.36504700	-5.31049200	L
C-C_3	0	-10.39919500	-1.45095900	-3.23110100	L
O-O_3	0	-9.65602000	-0.28050600	-3.00963100	L
C-C_3	0	-10.59507000	0.72486600	-2.73500800	L
C-C_3	0	-11.86554000	0.41015200	-3.49711800	L
C-C_3	0	-11.75764300	-1.06334400	-3.78641100	L
H-H_	0	-9.85580300	-2.10540600	-3.94138200	L
H-H_	0	-10.51492300	-1.99827600	-2.27155200	L
H-H_	0	-10.79117800	0.75231500	-1.64283500	L
H-H_	0	-10.19095800	1.70902700	-3.04232600	L
H-H_	0	-12.76565000	0.64253400	-2.88726000	L
H-H_	0	-11.89673500	0.98866500	-4.44686500	L
H-H_	0	-12.56918300	-1.63050700	-3.28185500	L
H-H_	0	-11.80017500	-1.25090500	-4.88177300	L
C-C_3	0	-5.76871800	3.59307600	1.02777500	L
O-O_3	0	-6.32000200	4.69385400	0.34769200	L
C-C_3	0	-5.25746200	5.34439400	-0.29617500	L
C-C_3	0	-4.19173400	4.32067500	-0.60281900	L
C-C_3	0	-4.39034900	3.28675200	0.46825100	L
H-H_	0	-5.70814700	3.83005000	2.10867200	L
H-H_	0	-6.43541200	2.71809300	0.90404400	L
H-H_	0	-5.62134200	5.82084800	-1.23039300	L
H-H_	0	-4.85143000	6.12875300	0.37809000	L
H-H_	0	-4.36961600	3.88010100	-1.60611600	L
H-H_	0	-3.17612600	4.76521200	-0.57142000	L
H-H_	0	-4.33777700	2.25937400	0.04685800	L
H-H_	0	-3.62547900	3.39906400	1.26615300	L
C-C_3	0	4.55695400	1.73033500	9.01020000	L
O-O_3	0	3.99121100	2.77372400	9.76472700	L
C-C_3	0	2.77133100	3.12816700	9.15420400	L
C-C_3	0	2.55322600	2.29019300	7.90854200	L
C-C_3	0	3.89832600	1.69034600	7.65083000	L
H-H_	0	5.65046200	1.88995600	8.91013200	L
H-H_	0	4.38980200	0.77101400	9.54277300	L
H-H_	0	1.93947900	2.97366700	9.87368300	L
H-H_	0	2.81185600	4.20377600	8.88127200	L

H-H_	0	1.80953600	1.48918400	8.10680700	L
H-H_	0	2.20954900	2.90921000	7.05262700	L
H-H_	0	3.82406200	0.65293300	7.26283400	L
H-H_	0	4.46012000	2.32476600	6.93307000	L
C-C_3	0	-7.19470800	2.54802500	-2.53742900	L
O-O_3	0	-8.04004400	2.99768600	-1.50307100	L
C-C_3	0	-8.70763700	4.14882600	-1.96655000	L
C-C_3	0	-8.48475200	4.28711200	-3.45347200	L
C-C_3	0	-7.19845200	3.55784200	-3.66300300	L
H-H_	0	-6.16632800	2.40487000	-2.14834300	L
H-H_	0	-7.56648000	1.56886600	-2.90671300	L
H-H_	0	-9.79093000	4.06908300	-1.74635500	L
H-H_	0	-8.30375200	5.04010100	-1.44080100	L
H-H_	0	-9.29974700	3.77515600	-4.00684200	L
H-H_	0	-8.42631900	5.34912400	-3.77065200	L
H-H_	0	-7.16802900	3.06722000	-4.65605600	L
H-H_	0	-6.33643900	4.25112000	-3.55305600	L
C-C_3	0	1.84998200	-5.89127800	-0.28610400	L
O-O_3	0	2.31411100	-6.64909200	-1.37271100	L
C-C_3	0	1.64918700	-7.88701200	-1.30324200	L
C-C_3	0	0.35992600	-7.71710400	-0.51974900	L
C-C_3	0	0.43574000	-6.31779000	0.02073200	L
H-H_	0	2.50354000	-6.07049500	0.59491500	L
H-H_	0	1.87911600	-4.81315700	-0.53919700	L
H-H_	0	1.43172100	-8.24975100	-2.32872000	L
H-H_	0	2.30895200	-8.62993100	-0.80863100	L
H-H_	0	-0.52372100	-7.82858000	-1.18380800	L
H-H_	0	0.30382000	-8.45969900	0.30454600	L
H-H_	0	-0.28554300	-5.66695900	-0.51496100	L
H-H_	0	0.23297500	-6.27602500	1.11038100	L
C-C_3	0	6.11176500	4.02911300	4.62526800	L
O-O_3	0	6.34979600	5.33919300	4.17706900	L
C-C_3	0	5.82530700	5.43702000	2.87417800	L
C-C_3	0	5.43461100	4.05778800	2.37106800	L
C-C_3	0	5.95070500	3.13022200	3.42430500	L
H-H_	0	6.96205300	3.69170200	5.25185700	L
H-H_	0	5.18715900	4.01581300	5.24037100	L
H-H_	0	4.94431400	6.11267200	2.88444700	L

H-H_	0	6.59761200	5.87721700	2.20914600	L
H-H_	0	4.32920200	3.97219000	2.28983900	L
H-H_	0	5.90056400	3.83854400	1.38801800	L
H-H_	0	5.25501700	2.28903500	3.61925100	L
H-H_	0	6.93594000	2.72700700	3.11897200	L
C-C_3	0	-2.34953500	3.75307400	12.86121400	L
O-O_3	0	-1.62088400	4.72943000	13.57018400	L
C-C_3	0	-0.35781900	4.18624600	13.87602600	L
C-C_3	0	-0.14255100	2.92818800	13.06411300	L
C-C_3	0	-1.53652900	2.47775100	12.76225500	L
H-H_	0	-3.31414000	3.55910700	13.37721400	L
H-H_	0	-2.56953800	4.13958900	11.84393900	L
H-H_	0	0.43457100	4.93164000	13.65125500	L
H-H_	0	-0.32281200	3.94719400	14.96127600	L
H-H_	0	0.39551500	3.16885800	12.12121900	L
H-H_	0	0.42506400	2.16096800	13.63430300	L
H-H_	0	-1.60750100	2.02192200	11.75170600	L
H-H_	0	-1.87639400	1.74844900	13.53046400	L
C-C_3	0	2.03700000	13.11298400	-1.91632500	L
O-O_3	0	1.35514700	14.29416700	-1.57991600	L
C-C_3	0	0.10806000	13.89059500	-1.07322600	L
C-C_3	0	0.25824300	12.51374700	-0.45373400	L
C-C_3	0	1.56887000	12.00735300	-0.99131500	L
H-H_	0	1.81428900	12.85279100	-2.97438600	L
H-H_	0	3.13175700	13.26922400	-1.81177200	L
H-H_	0	-0.23972300	14.62189100	-0.31299100	L
H-H_	0	-0.63250900	13.86376200	-1.90308000	L
H-H_	0	0.29497000	12.58665600	0.65488500	L
H-H_	0	-0.58111600	11.85241100	-0.75509700	L
H-H_	0	2.29720300	11.86249300	-0.16433400	L
H-H_	0	1.43922000	11.05161000	-1.54167300	L
C-C_3	0	-2.14729500	6.01232600	4.17742500	L
O-O_3	0	-1.28133500	4.92136800	4.39616600	L
C-C_3	0	-0.98124000	4.36751100	3.13984200	L
C-C_3	0	-1.24936100	5.40919900	2.08080700	L
C-C_3	0	-2.36504600	6.20029600	2.68712500	L
H-H_	0	-3.11307800	5.83221200	4.69339100	L
H-H_	0	-1.68345500	6.92226000	4.60830200	L

H-H_	0	0.08050300	4.04505500	3.10944400	L
H-H_	0	-1.63186700	3.48168800	2.97764400	L
H-H_	0	-0.35058700	6.04907000	1.94599300	L
H-H_	0	-1.53425500	4.95714200	1.11027900	L
H-H_	0	-2.32210300	7.26898500	2.39029800	L
H-H_	0	-3.34567800	5.76782700	2.38952600	L
C-C_3	0	-3.58423400	12.73297900	-0.21537600	L
O-O_3	0	-4.72469300	13.51006700	0.06298200	L
C-C_3	0	-5.27821700	13.91399300	-1.16943300	L
C-C_3	0	-4.42029300	13.39984300	-2.30944000	L
C-C_3	0	-3.60979100	12.31861900	-1.66995100	L
H-H_	0	-2.67716600	13.34019000	-0.00790800	L
H-H_	0	-3.56996700	11.84180200	0.44453400	L
H-H_	0	-6.30569900	13.49892600	-1.25330200	L
H-H_	0	-5.34350500	15.02251100	-1.20338500	L
H-H_	0	-5.03792500	13.00913500	-3.14738500	L
H-H_	0	-3.75050300	14.20822700	-2.67812800	L
H-H_	0	-4.12753100	11.34278500	-1.78210000	L
H-H_	0	-2.59016800	12.25261100	-2.10436500	L
C-C_3	0	-7.74189900	0.89779400	-8.06307200	L
O-O_3	0	-7.79273800	1.59498800	-6.84400600	L
C-C_3	0	-8.51043800	0.78216500	-5.95256400	L
C-C_3	0	-9.49031200	-0.05304700	-6.74697300	L
C-C_3	0	-8.90663800	-0.07364900	-8.13249100	L
H-H_	0	-7.79120200	1.62004400	-8.90304500	L
H-H_	0	-6.77898800	0.35123000	-8.12918400	L
H-H_	0	-7.80224300	0.12773700	-5.39772100	L
H-H_	0	-9.05024000	1.41970700	-5.22354100	L
H-H_	0	-9.57885400	-1.07665300	-6.32577500	L
H-H_	0	-10.48962100	0.43542400	-6.75638700	L
H-H_	0	-8.55165300	-1.09198900	-8.39863400	L
H-H_	0	-9.65760200	0.26063900	-8.88144100	L
C-C_3	0	5.39191800	10.06184100	-0.71022200	L
O-O_3	0	4.14329300	9.69973600	-0.17928800	L
C-C_3	0	4.31274400	9.64840600	1.21600100	L
C-C_3	0	5.77389200	9.37434500	1.52633900	L
C-C_3	0	6.46804100	9.51432600	0.19970100	L
H-H_	0	5.50455100	9.64775500	-1.73298600	L

H-H_	0	5.45875000	11.17111200	-0.76786200	L
H-H_	0	4.00253200	10.62012000	1.65594400	L
H-H_	0	3.67089700	8.84818500	1.64116700	L
H-H_	0	6.16378200	10.11399500	2.25974300	L
H-H_	0	5.90514000	8.34790000	1.92955700	L
H-H_	0	7.33427000	10.20817700	0.25939100	L
H-H_	0	6.80614400	8.51843600	-0.16425300	L
C-C_3	0	9.70303700	-8.56745500	3.72870600	L
O-O_3	0	8.92109000	-7.78268400	2.85864000	L
C-C_3	0	7.68238900	-7.54943800	3.48602000	L
C-C_3	0	7.55626500	-8.43796100	4.70302800	L
C-C_3	0	8.98064500	-8.73380900	5.05055300	L
H-H_	0	9.87007600	-9.56155200	3.26064800	L
H-H_	0	10.68948300	-8.08046000	3.88421700	L
H-H_	0	7.63085200	-6.48482800	3.79144800	L
H-H_	0	6.85906200	-7.74631400	2.77100100	L
H-H_	0	7.02542800	-7.92597300	5.53511100	L
H-H_	0	7.02351400	-9.37726000	4.43640900	L
H-H_	0	9.35584300	-7.98789400	5.78575500	L
H-H_	0	9.09942700	-9.76029700	5.46050300	L
C-C_3	0	5.69152900	-2.18451400	-8.96787700	L
O-O_3	0	5.18870400	-0.87643800	-9.09009300	L
C-C_3	0	4.37221200	-0.84332600	-10.23949400	L
C-C_3	0	4.39076500	-2.20082000	-10.91808300	L
C-C_3	0	5.58167400	-2.87042100	-10.30877400	L
H-H_	0	6.74647300	-2.15040100	-8.62882000	L
H-H_	0	5.09438000	-2.73542800	-8.20870900	L
H-H_	0	3.33498200	-0.56927700	-9.95416900	L
H-H_	0	4.76386100	-0.06772400	-10.93219600	L
H-H_	0	3.46583600	-2.76561400	-10.66630200	L
H-H_	0	4.48940200	-2.11184700	-12.02175400	L
H-H_	0	5.44039300	-3.96741200	-10.20664500	L
H-H_	0	6.48747100	-2.65941900	-10.91840300	L
C-C_3	0	-8.89160300	2.50655600	2.89614100	L
O-O_3	0	-8.88270700	1.19413600	2.40183000	L
C-C_3	0	-10.00500200	1.07580000	1.56071100	L
C-C_3	0	-10.46172000	2.45939500	1.13065200	L
C-C_3	0	-9.54154000	3.39336000	1.86213500	L

H-H_	0	-9.47204400	2.53632900	3.84191700	L
H-H_	0	-7.85333300	2.83795800	3.10424600	L
H-H_	0	-9.73218600	0.46797500	0.67400500	L
H-H_	0	-10.81932500	0.55273700	2.10509000	L
H-H_	0	-10.36562800	2.58625700	0.03329200	L
H-H_	0	-11.51862000	2.63240600	1.42765100	L
H-H_	0	-8.77338700	3.78987800	1.16594100	L
H-H_	0	-10.08986700	4.23317100	2.33704400	L
C-C_3	0	-3.71566600	-5.07125000	9.53062300	L
O-O_3	0	-3.48455000	-3.69637100	9.73862200	L
C-C_3	0	-2.49088800	-3.28559000	8.82883600	L
C-C_3	0	-1.83272800	-4.50461200	8.22624200	L
C-C_3	0	-2.89236200	-5.55324400	8.35305300	L
H-H_	0	-3.42711700	-5.62625900	10.44801200	L
H-H_	0	-4.79658200	-5.24478600	9.34840900	L
H-H_	0	-2.96320700	-2.67168300	8.03148200	L
H-H_	0	-1.73713500	-2.66179500	9.35166600	L
H-H_	0	-1.53738900	-4.33039500	7.17050800	L
H-H_	0	-0.94104200	-4.78782200	8.82638100	L
H-H_	0	-3.52215500	-5.57569000	7.43892000	L
H-H_	0	-2.45874600	-6.56082700	8.52990700	L
C-C_3	0	-3.77847500	-5.79910600	-3.32112400	L
O-O_3	0	-4.49721700	-6.90109000	-2.81990800	L
C-C_3	0	-5.81977100	-6.47999700	-2.58637600	L
C-C_3	0	-5.90665600	-4.97729700	-2.73879900	L
C-C_3	0	-4.72830200	-4.65558000	-3.59664500	L
H-H_	0	-3.03083400	-5.48675800	-2.56448700	L
H-H_	0	-3.24258500	-6.09024200	-4.24824500	L
H-H_	0	-6.48736000	-6.97000900	-3.32492700	L
H-H_	0	-6.13230800	-6.78638200	-1.56692400	L
H-H_	0	-6.86073100	-4.66065100	-3.21309400	L
H-H_	0	-5.79502000	-4.48919000	-1.74917500	L
H-H_	0	-5.02994000	-4.66352900	-4.66468300	L
H-H_	0	-4.27951700	-3.67357000	-3.33135900	L
C-C_3	0	9.62147500	4.38293500	2.88536000	L
O-O_3	0	9.55052400	4.23344900	4.28446200	L
C-C_3	0	10.85947600	4.03464200	4.76497200	L
C-C_3	0	11.85184900	4.33197500	3.66259700	L

C-C_3	0	11.04250600	4.15547200	2.41780400	L
H-H_	0	8.93884300	3.66216400	2.39363900	L
H-H_	0	9.28951600	5.40549300	2.61796100	L
H-H_	0	11.04106800	4.69685700	5.63795300	L
H-H_	0	10.96659100	2.97935300	5.09525200	L
H-H_	0	12.20605500	5.38222000	3.74373300	L
H-H_	0	12.72182400	3.64079500	3.69133000	L
H-H_	0	11.33558400	4.88223900	1.63118100	L
H-H_	0	11.15208400	3.11805800	2.03467300	L
C-C_3	0	10.23738200	8.12615000	3.48874600	L
O-O_3	0	11.39902500	7.73860900	2.79217400	L
C-C_3	0	11.19475100	8.05555500	1.43847800	L
C-C_3	0	9.70962300	8.06631600	1.16474600	L
C-C_3	0	9.10986600	8.38054700	2.50209600	L
H-H_	0	9.95198700	7.31872200	4.19675600	L
H-H_	0	10.45062400	9.04491000	4.07688500	L
H-H_	0	11.63064800	9.05713900	1.22835500	L
H-H_	0	11.70182000	7.30524000	0.79515600	L
H-H_	0	9.44469300	8.83359600	0.40742700	L
H-H_	0	9.37723000	7.06385900	0.81836300	L
H-H_	0	8.79543900	9.44637700	2.54137000	L
H-H_	0	8.23630500	7.72671700	2.70915700	L
C-C_3	0	-1.84691000	-5.61783900	-11.42759200	L
O-O_3	0	-1.00342800	-5.76706400	-10.30985300	L
C-C_3	0	-1.81531600	-6.05747800	-9.19487300	L
C-C_3	0	-3.25131400	-6.23232900	-9.64340800	L
C-C_3	0	-3.28243300	-5.52643900	-10.96039700	L
H-H_	0	-1.56460400	-4.70402700	-11.99235300	L
H-H_	0	-1.72035600	-6.49951500	-12.09282500	L
H-H_	0	-1.44906400	-6.98084800	-8.69907400	L
H-H_	0	-1.74742800	-5.21603800	-8.47393300	L
H-H_	0	-3.47742400	-7.31248100	-9.78461700	L
H-H_	0	-3.96954400	-5.78753200	-8.92177000	L
H-H_	0	-3.98417600	-6.00963200	-11.67432400	L
H-H_	0	-3.56320400	-4.46217700	-10.81313300	L
C-C_3	0	-4.95311300	-6.92322600	1.75076900	L
O-O_3	0	-4.07763400	-7.66314200	0.94128600	L
C-C_3	0	-3.10653200	-6.75964500	0.47191200	L

C-C_3	0	-3.61964600	-5.33696400	0.62661100	L
C-C_3	0	-4.98061500	-5.50776400	1.23601400	L
H-H_	0	-5.96909000	-7.36967700	1.71858000	L
H-H_	0	-4.58262400	-6.93888900	2.79886200	L
H-H_	0	-2.17383100	-6.89960500	1.05519100	L
H-H_	0	-2.88890800	-6.97857400	-0.59285600	L
H-H_	0	-2.95233700	-4.76235200	1.30376800	L
H-H_	0	-3.68665900	-4.81372000	-0.35010800	L
H-H_	0	-5.17078600	-4.78516500	2.05371300	L
H-H_	0	-5.76435300	-5.40566600	0.45710600	L
C-C_3	0	2.06530500	6.84828700	-0.46727200	L
O-O_3	0	2.57508100	6.88777500	-1.77595700	L
C-C_3	0	3.92501900	6.49273000	-1.71583700	L
C-C_3	0	4.25470400	5.99027400	-0.32325000	L
C-C_3	0	2.92856100	5.93907400	0.37127100	L
H-H_	0	2.07504200	7.87595900	-0.05047800	L
H-H_	0	1.02110100	6.47547400	-0.48045900	L
H-H_	0	4.09349000	5.68797600	-2.45932700	L
H-H_	0	4.57680900	7.35043300	-1.98088800	L
H-H_	0	4.72608600	4.98746900	-0.36139700	L
H-H_	0	4.93371100	6.70294700	0.19185500	L
H-H_	0	2.52308800	4.90493100	0.35172800	L
H-H_	0	2.99203300	6.29418100	1.42180400	L
C-C_3	0	-13.28859800	-5.44262500	-1.43054400	L
O-O_3	0	-13.14616500	-4.29821900	-2.24089700	L
C-C_3	0	-12.66766300	-4.71616900	-3.49896200	L
C-C_3	0	-12.27597000	-6.17563300	-3.43035200	L
C-C_3	0	-13.06682300	-6.68492300	-2.26757700	L
H-H_	0	-12.54045600	-5.39958100	-0.61014500	L
H-H_	0	-14.30275500	-5.45590200	-0.97776300	L
H-H_	0	-13.47060300	-4.57363900	-4.25412300	L
H-H_	0	-11.79350000	-4.09792400	-3.79213000	L
H-H_	0	-12.52471100	-6.71582600	-4.36948800	L
H-H_	0	-11.18828400	-6.26512200	-3.22183300	L
H-H_	0	-14.04303900	-7.08620600	-2.61915200	L
H-H_	0	-12.51735000	-7.46924700	-1.70489900	L
C-C_3	0	-6.22137600	-2.19137100	0.64825100	L
O-O_3	0	-6.71806000	-2.33939500	-0.65939100	L

C-C_3	0	-7.31182800	-1.11961000	-1.01299000	L
C-C_3	0	-6.65696300	-0.01928100	-0.21478100	L
C-C_3	0	-6.17323900	-0.71924200	1.01923200	L
H-H_	0	-6.87346100	-2.74466200	1.35500900	L
H-H_	0	-5.20558900	-2.62870700	0.68888800	L
H-H_	0	-7.17771500	-0.94197600	-2.10106600	L
H-H_	0	-8.39868700	-1.16578200	-0.78269900	L
H-H_	0	-5.79535100	0.39659600	-0.77730900	L
H-H_	0	-7.38053300	0.78703700	0.02113800	L
H-H_	0	-5.14249200	-0.39866900	1.28699600	L
H-H_	0	-6.85254100	-0.51439600	1.87359800	L
C-C_3	0	0.46582600	-10.02442900	-7.59510300	L
O-O_3	0	1.80978600	-10.40660700	-7.78736100	L
C-C_3	0	2.36865300	-10.65764000	-6.51899200	L
C-C_3	0	1.47114400	-10.06464300	-5.45741300	L
C-C_3	0	0.13023500	-10.06457500	-6.11801100	L
H-H_	0	0.32466200	-8.99476700	-7.98542900	L
H-H_	0	-0.20162500	-10.70736200	-8.16273300	L
H-H_	0	2.45385600	-11.75656100	-6.37369700	L
H-H_	0	3.38413400	-10.21225600	-6.46082800	L
H-H_	0	1.46733900	-10.66968000	-4.52653400	L
H-H_	0	1.79038700	-9.02463400	-5.23303800	L
H-H_	0	-0.41174300	-11.00572700	-5.87742600	L
H-H_	0	-0.47861000	-9.19015200	-5.80810300	L
C-C_3	0	-9.14174500	-9.22145600	1.84526400	L
O-O_3	0	-10.12311300	-8.37137000	2.39320600	L
C-C_3	0	-11.26921600	-8.45458600	1.57783400	L
C-C_3	0	-11.10633100	-9.59971300	0.60229300	L
C-C_3	0	-9.62207000	-9.75065000	0.51026700	L
H-H_	0	-8.18876200	-8.66476700	1.72422200	L
H-H_	0	-8.96821000	-10.06594400	2.54663300	L
H-H_	0	-12.16872600	-8.60927600	2.21067900	L
H-H_	0	-11.38851400	-7.49750300	1.02550900	L
H-H_	0	-11.55640700	-10.52551800	1.02425100	L
H-H_	0	-11.56316300	-9.37375800	-0.38464700	L
H-H_	0	-9.32333700	-10.80920300	0.35078700	L
H-H_	0	-9.22890400	-9.11751400	-0.31363600	L
C-C_3	0	2.75899300	-5.73404800	-9.84434400	L

O-O_3	0	3.37469600	-5.00804500	-8.81246800	L
C-C_3	0	3.83688600	-5.95719100	-7.88065400	L
C-C_3	0	3.10145400	-7.27127200	-8.09075900	L
C-C_3	0	2.18014000	-6.99154400	-9.24269100	L
H-H_	0	1.96243200	-5.12323400	-10.31716800	L
H-H_	0	3.51704600	-5.98855000	-10.61825400	L
H-H_	0	4.93070300	-6.09991800	-8.01513300	L
H-H_	0	3.65364200	-5.58097300	-6.85325500	L
H-H_	0	3.81902100	-8.07831400	-8.35645300	L
H-H_	0	2.53177300	-7.56526400	-7.18306500	L
H-H_	0	2.15958800	-7.82804100	-9.97434300	L
H-H_	0	1.15445600	-6.78825500	-8.86734300	L
C-C_3	0	0.96819700	12.86316600	5.14268900	L
O-O_3	0	1.02074200	13.08777500	3.75403200	L
C-C_3	0	2.30672300	12.70679200	3.33734500	L
C-C_3	0	2.80368000	11.61609200	4.25985500	L
C-C_3	0	2.03461500	11.85021400	5.53005400	L
H-H_	0	1.13681400	13.82457300	5.67534300	L
H-H_	0	-0.04022300	12.48454000	5.41419400	L
H-H_	0	2.26930000	12.34451800	2.28989900	L
H-H_	0	2.98368500	13.58848400	3.38344900	L
H-H_	0	2.55615700	10.61590800	3.84184200	L
H-H_	0	3.90103400	11.69199700	4.41921200	L
H-H_	0	1.57540800	10.90514200	5.89384400	L
H-H_	0	2.69830500	12.26619700	6.31966200	L
C-C_3	0	-3.48376600	-0.69522800	11.59943600	L
O-O_3	0	-3.23074700	-0.56960500	10.22377100	L
C-C_3	0	-3.71661800	0.68998000	9.82762300	L
C-C_3	0	-4.65180600	1.23514400	10.89367300	L
C-C_3	0	-4.67337900	0.16833000	11.94853300	L
H-H_	0	-3.68981200	-1.75801600	11.84694800	L
H-H_	0	-2.58670300	-0.36755300	12.16839100	L
H-H_	0	-2.86101300	1.38034800	9.66847300	L
H-H_	0	-4.25778000	0.57884300	8.86733300	L
H-H_	0	-4.24729200	2.18163300	11.30884900	L
H-H_	0	-5.67140400	1.41506900	10.48977000	L
H-H_	0	-4.58114500	0.59605600	12.97065200	L
H-H_	0	-5.60988500	-0.42702100	11.87078300	L

C-C_3	0	10.11112000	8.34644600	-2.45292000	L
O-O_3	0	8.86215700	8.98466500	-2.33251800	L
C-C_3	0	8.03233000	8.50049600	-3.36415200	L
C-C_3	0	8.80947800	7.53390400	-4.23920000	L
C-C_3	0	10.23359100	7.75415500	-3.83703400	L
H-H_	0	10.92573700	9.07874800	-2.26956700	L
H-H_	0	10.18375000	7.54249300	-1.69105900	L
H-H_	0	7.14659700	7.99696600	-2.92147800	L
H-H_	0	7.67718700	9.36021800	-3.97197000	L
H-H_	0	8.50628000	6.48889000	-4.01596100	L
H-H_	0	8.66048600	7.74450800	-5.31989800	L
H-H_	0	10.81288400	6.80498000	-3.82953000	L
H-H_	0	10.71396800	8.48761500	-4.52185800	L
C-C_3	0	-8.99562700	5.07307600	-7.02823100	L
O-O_3	0	-10.14082500	5.06155400	-6.21600600	L
C-C_3	0	-10.94772900	4.00907300	-6.68696500	L
C-C_3	0	-10.59928000	3.72672100	-8.13941500	L
C-C_3	0	-9.39059400	4.58137500	-8.40219900	L
H-H_	0	-8.58319000	6.10278600	-7.08457800	L
H-H_	0	-8.22434800	4.40560000	-6.58577500	L
H-H_	0	-10.77277500	3.10481700	-6.06420000	L
H-H_	0	-12.01679400	4.29597100	-6.59454000	L
H-H_	0	-10.36038600	2.65092300	-8.28097800	L
H-H_	0	-11.43627600	4.01103400	-8.81419800	L
H-H_	0	-8.57309600	4.00004800	-8.87518300	L
H-H_	0	-9.66280200	5.44304900	-9.05056500	L
C-C_3	0	8.71372700	-0.77577100	0.98842500	L
O-O_3	0	7.36849700	-0.79864700	1.38792200	L
C-C_3	0	6.74831100	0.31356900	0.78756500	L
C-C_3	0	7.80852400	1.27520400	0.28020700	L
C-C_3	0	9.10062200	0.65887700	0.72323400	L
H-H_	0	9.34698600	-1.20988200	1.78796500	L
H-H_	0	8.83061100	-1.38378000	0.06365700	L
H-H_	0	6.11471900	-0.03514700	-0.05213700	L
H-H_	0	6.09640300	0.81618200	1.53216500	L
H-H_	0	7.76969300	1.32943400	-0.82806600	L
H-H_	0	7.67918700	2.29019100	0.70977600	L
H-H_	0	9.89193000	0.74013200	-0.05072200	L

H-H_	0	9.44952500	1.13894400	1.66118600	L
C-C_3	0	12.60722800	-3.98663400	0.30730600	L
O-O_3	0	12.29602100	-5.34280600	0.53541800	L
C-C_3	0	11.16816900	-5.37287600	1.37382200	L
C-C_3	0	10.40710900	-4.08034100	1.19731200	L
C-C_3	0	11.48396000	-3.10648700	0.82946500	L
H-H_	0	13.56447900	-3.73582600	0.81255300	L
H-H_	0	12.73608500	-3.82634400	-0.78318000	L
H-H_	0	10.52833500	-6.24221400	1.11635300	L
H-H_	0	11.50402900	-5.48087500	2.42801100	L
H-H_	0	9.68031900	-4.17366100	0.36397700	L
H-H_	0	9.87243900	-3.78540200	2.12377700	L
H-H_	0	11.13852700	-2.38571400	0.05654900	L
H-H_	0	11.81774500	-2.55404200	1.73380300	L
C-C_3	0	7.35667000	-10.86512600	1.33650200	L
O-O_3	0	6.64672800	-9.73845700	0.88127700	L
C-C_3	0	7.48478800	-9.03101900	-0.00371400	L
C-C_3	0	8.82996700	-9.72206300	-0.10540600	L
C-C_3	0	8.56680300	-11.08308400	0.45615400	L
H-H_	0	7.67691200	-10.68965600	2.38566200	L
H-H_	0	6.69592800	-11.75756600	1.31356400	L
H-H_	0	7.00123500	-8.98864700	-1.00165200	L
H-H_	0	7.61612600	-7.99442100	0.36181300	L
H-H_	0	9.18724200	-9.77701500	-1.15628300	L
H-H_	0	9.58212500	-9.18941500	0.51745100	L
H-H_	0	8.31877500	-11.79187000	-0.36417500	L
H-H_	0	9.43477500	-11.46547700	1.03610900	L
C-C_3	0	-8.82973700	-5.34034400	1.13217700	L
O-O_3	0	-8.46709200	-6.33300200	0.19856600	L
C-C_3	0	-9.19222000	-6.07003200	-0.97577400	L
C-C_3	0	-9.49252000	-4.59282700	-1.01986500	L
C-C_3	0	-9.61449500	-4.24277800	0.43097400	L
H-H_	0	-9.43988500	-5.79644100	1.93934300	L
H-H_	0	-7.91151800	-4.92076100	1.59126200	L
H-H_	0	-8.60785400	-6.37807100	-1.86629700	L
H-H_	0	-10.13861600	-6.65147900	-0.95563500	L
H-H_	0	-8.64086900	-4.04582900	-1.47777300	L
H-H_	0	-10.42423200	-4.37608900	-1.58013600	L

H-H_	0	-9.20457800	-3.23277800	0.64349300	L
H-H_	0	-10.68016300	-4.28362700	0.74090800	L
C-C_3	0	-1.11122900	-1.79746600	-9.90353000	L
O-O_3	0	-1.22356000	-1.36954900	-8.57091500	L
C-C_3	0	-0.41677300	-2.23091200	-7.80307100	L
C-C_3	0	0.59311100	-2.91836500	-8.70721500	L
C-C_3	0	0.24887700	-2.42972600	-10.08467600	L
H-H_	0	-1.91321500	-2.53717000	-10.12065800	L
H-H_	0	-1.22795300	-0.93026000	-10.58722300	L
H-H_	0	0.10441900	-1.64789400	-7.01525800	L
H-H_	0	-1.06263700	-2.98143900	-7.30587700	L
H-H_	0	1.63424600	-2.63769100	-8.44430700	L
H-H_	0	0.48154700	-4.02141800	-8.63885000	L
H-H_	0	0.98234900	-1.66041500	-10.41222000	L
H-H_	0	0.22065200	-3.25752400	-10.82462200	L
C-C_3	0	13.05624000	-4.49458800	-4.51331400	L
O-O_3	0	12.36262100	-5.57819400	-5.08818300	L
C-C_3	0	12.71669500	-6.74076800	-4.37296300	L
C-C_3	0	13.55495900	-6.35891500	-3.17061300	L
C-C_3	0	14.10274100	-5.02320300	-3.55627100	L
H-H_	0	13.53027300	-3.88515000	-5.31186000	L
H-H_	0	12.33327700	-3.85515000	-3.96409300	L
H-H_	0	11.80102500	-7.27968800	-4.05105000	L
H-H_	0	13.30057700	-7.40822700	-5.04303200	L
H-H_	0	12.90980700	-6.25799800	-2.27032500	L
H-H_	0	14.36317100	-7.09693700	-2.97620100	L
H-H_	0	14.23521000	-4.36213500	-2.67377800	L
H-H_	0	15.07338600	-5.14744000	-4.08561000	L
C-C_3	0	-1.98821800	7.50204000	-6.64995100	L
O-O_3	0	-0.60144200	7.65220100	-6.84159800	L
C-C_3	0	-0.04749500	6.36481900	-6.98743100	L
C-C_3	0	-1.15676300	5.33542600	-7.02576600	L
C-C_3	0	-2.30270400	6.04799000	-6.38877300	L
H-H_	0	-2.31782500	8.12500300	-5.79557200	L
H-H_	0	-2.51985000	7.84617800	-7.56303000	L
H-H_	0	0.55158900	6.31685100	-7.91869400	L
H-H_	0	0.62842900	6.16772100	-6.13160900	L
H-H_	0	-1.40165100	5.08556700	-8.08013500	L

H-H_	0	-0.89280900	4.41338400	-6.46689400	L
H-H_	0	-3.27416200	5.75505100	-6.83522800	L
H-H_	0	-2.31048400	5.84644500	-5.29651200	L
C-C_3	0	1.57499100	8.65353800	-9.62072500	L
O-O_3	0	0.67872400	9.55808000	-9.01602200	L
C-C_3	0	-0.53804900	9.46625600	-9.71376600	L
C-C_3	0	-0.61825600	8.10471600	-10.36458300	L
C-C_3	0	0.82153600	7.74722300	-10.58264800	L
H-H_	0	2.36248500	9.22177500	-10.16114500	L
H-H_	0	2.06601200	8.04886600	-8.82895100	L
H-H_	0	-1.38583700	9.61419100	-9.01220600	L
H-H_	0	-0.57476600	10.26385500	-10.48815000	L
H-H_	0	-1.09227000	7.37764700	-9.67030700	L
H-H_	0	-1.18721400	8.13754500	-11.31904800	L
H-H_	0	1.00819200	6.67203100	-10.36812100	L
H-H_	0	1.11686200	7.97224700	-11.63129100	L
C-C_3	0	-0.80984100	12.38506700	-4.97806300	L
O-O_3	0	-2.05701100	13.01784500	-4.80541000	L
C-C_3	0	-3.01935000	12.20276600	-5.42510500	L
C-C_3	0	-2.34000500	11.41201400	-6.51740600	L
C-C_3	0	-0.93810900	11.27019100	-6.00609600	L
H-H_	0	-0.06881300	13.13714400	-5.32334700	L
H-H_	0	-0.46623600	11.98074100	-4.00253100	L
H-H_	0	-3.45645300	11.51595500	-4.66798900	L
H-H_	0	-3.83154000	12.83191800	-5.84663200	L
H-H_	0	-2.82943800	10.42812000	-6.67889300	L
H-H_	0	-2.34243300	11.99543200	-7.46455100	L
H-H_	0	-0.80554800	10.27980100	-5.52012800	L
H-H_	0	-0.19594300	11.38085200	-6.82654600	L
C-C_3	0	14.81292500	-0.62231500	-4.33308100	L
O-O_3	0	14.62930100	-1.76018000	-3.52337400	L
C-C_3	0	14.18164000	-1.32325200	-2.26058200	L
C-C_3	0	14.25717500	0.18683500	-2.19319500	L
C-C_3	0	14.24032900	0.59043900	-3.63255800	L
H-H_	0	15.90147500	-0.47907700	-4.50709300	L
H-H_	0	14.31569500	-0.77539900	-5.31454900	L
H-H_	0	13.13197000	-1.65518100	-2.11716300	L
H-H_	0	14.80609000	-1.78009300	-1.46354800	L

H-H_	0	13.40150700	0.61906700	-1.63144300	L
H-H_	0	15.21575500	0.49866900	-1.72262800	L
H-H_	0	13.19493200	0.76594000	-3.96656400	L
H-H_	0	14.85382200	1.49845700	-3.81551100	L
C-C_3	0	-11.92839500	-5.67612700	5.94014100	L
O-O_3	0	-12.75042700	-4.55072500	5.72548600	L
C-C_3	0	-12.85555800	-4.35684600	4.33503100	L
C-C_3	0	-12.40128600	-5.61083800	3.62348400	L
C-C_3	0	-11.46285200	-6.23310400	4.60858100	L
H-H_	0	-11.05716500	-5.38822400	6.56671500	L
H-H_	0	-12.51407800	-6.44651200	6.48636800	L
H-H_	0	-13.90600200	-4.11636600	4.06617500	L
H-H_	0	-12.21221800	-3.50075800	4.04316100	L
H-H_	0	-13.26933700	-6.28375600	3.44703600	L
H-H_	0	-11.89962700	-5.37995000	2.65942900	L
H-H_	0	-11.51819400	-7.34290200	4.58556700	L
H-H_	0	-10.42199500	-5.90596400	4.40064300	L
C-C_3	0	-13.61471500	-1.32338300	2.13043300	L
O-O_3	0	-13.59524400	-0.02846300	1.58637700	L
C-C_3	0	-13.24273500	-0.17741600	0.23344600	L
C-C_3	0	-12.42565000	-1.44775300	0.07432200	L
C-C_3	0	-12.61615300	-2.17667500	1.37550100	L
H-H_	0	-13.35633700	-1.27959600	3.20929800	L
H-H_	0	-14.63789500	-1.74858400	2.02952900	L
H-H_	0	-14.16719000	-0.23353900	-0.38253300	L
H-H_	0	-12.65793800	0.70634400	-0.09406700	L
H-H_	0	-12.79992100	-2.05055300	-0.77989000	L
H-H_	0	-11.35157800	-1.21144400	-0.08282200	L
H-H_	0	-13.00752800	-3.20522900	1.21563100	L
H-H_	0	-11.65385900	-2.22616300	1.92925700	L
C-C_3	0	5.57575100	-11.80332300	-1.88004000	L
O-O_3	0	5.07244500	-12.38288300	-3.06015900	L
C-C_3	0	3.67707200	-12.44265800	-2.91393800	L
C-C_3	0	3.24555300	-11.31136100	-2.00975100	L
C-C_3	0	4.45993800	-11.05699700	-1.16507700	L
H-H_	0	5.98543300	-12.60328700	-1.22575800	L
H-H_	0	6.40078700	-11.10818100	-2.14059400	L
H-H_	0	3.19225300	-12.35540500	-3.90833600	L

H-H_	0	3.39660300	-13.42167600	-2.46579800	L
H-H_	0	3.00759900	-10.41029900	-2.61549300	L
H-H_	0	2.36668500	-11.59670300	-1.39347800	L
H-H_	0	4.67415800	-9.96884700	-1.10227300	L
H-H_	0	4.31802600	-11.46521900	-0.14037200	L
C-C_3	0	-8.60225400	-1.80008000	9.71626900	L
O-O_3	0	-7.49806900	-0.92911500	9.61223300	L
C-C_3	0	-6.48820200	-1.60269500	8.89953300	L
C-C_3	0	-7.08147400	-2.81808000	8.22716000	L
C-C_3	0	-8.26086600	-3.13701200	9.08724200	L
H-H_	0	-8.87267100	-1.93246400	10.78552400	L
H-H_	0	-9.46829800	-1.34590500	9.18904800	L
H-H_	0	-6.04403300	-0.92396900	8.14297700	L
H-H_	0	-5.69214500	-1.91330600	9.60976200	L
H-H_	0	-7.41653900	-2.55762100	7.19963100	L
H-H_	0	-6.35727400	-3.65781200	8.19043300	L
H-H_	0	-9.10615300	-3.54406300	8.49020200	L
H-H_	0	-7.96929300	-3.86649400	9.87519400	L
C-C_R	0	2.16348000	0.13129700	1.03910200	H
C-C_R	0	2.28406500	1.31303200	2.89316100	H
C-C_3	0	2.90187200	-1.10251400	3.17724800	H
H-H_	0	2.08326800	-1.82428500	3.22467500	H
N-N_R	0	2.03629700	2.18600800	1.97096400	H
N-N_R	0	1.97227200	1.45202000	0.81381500	H
N-N_R	0	2.43440300	0.05107300	2.37570900	H
C-C_3	0	2.31840900	1.55571900	4.37304700	H
H-H_	0	1.42035400	2.11389000	4.64574500	H
H-H_	0	3.20193100	2.14422700	4.65299900	H
C-C_3	0	3.24148000	-0.56956300	4.57455200	H
O-O_3	0	2.28044600	0.33337500	5.06268800	H
C-C_R	0	1.67704900	2.17745600	-0.39065600	H
C-C_R	0	2.72549100	2.49735100	-1.26111400	H
C-C_R	0	0.37032400	2.62057800	-0.59691600	H
C-C_R	0	2.42791400	3.27082400	-2.37915000	H
C-C_R	0	0.13937600	3.45624600	-1.69073500	H
C-C_R	0	1.14570500	3.78133400	-2.59562400	H
H-H_	0	3.22277500	3.49186100	-3.08706100	H
H-H_	0	-0.86927700	3.83087800	-1.84973000	H

H-H_	0	3.28529000	-1.41050100	5.26941100	H
C-C_R	0	2.04488700	-1.00020600	0.14845200	H
O-O_R	0	1.87866400	-2.21788200	0.72024000	H
C-C_R	0	2.20765800	-0.85436700	-1.20007200	H
C-C_R	0	2.14490100	-1.90010800	-2.17393000	H
H-H_	0	2.32926200	0.15603900	-1.56485300	H
H-H_	0	2.40565100	-2.88999200	-1.79896100	H
H-H_	0	0.77675700	-2.08450100	-2.29902800	H
C-C_R	0	2.57103900	-1.62830700	-3.56307700	H
C-C_R	0	2.70516600	-2.69862900	-4.46287900	H
C-C_R	0	2.74358600	-0.33293300	-4.08221300	H
C-C_R	0	2.95107300	-2.48770200	-5.81208600	H
H-H_	0	2.60703100	-3.71236000	-4.08734500	H
C-C_R	0	2.99215900	-0.12327000	-5.43402700	H
H-H_	0	2.66976300	0.53788000	-3.43586200	H
C-C_R	0	3.08534500	-1.19553600	-6.31279100	H
H-H_	0	3.03633800	-3.33711800	-6.47946000	H
H-H_	0	3.12268400	0.89101200	-5.79777100	H
H-H_	0	3.26734100	-1.02750700	-7.36587600	H
C-C_3	0	4.12120100	2.00451400	-1.00323600	H
H-H_	0	4.54924900	2.49477400	-0.12514800	H
H-H_	0	4.12798500	0.92896100	-0.81364100	H
H-H_	0	4.75857000	2.19766100	-1.86684900	H
C-C_3	0	0.86671800	4.64768300	-3.79340900	H
H-H_	0	-0.20475200	4.80339900	-3.93338200	H
H-H_	0	1.33290900	5.63100000	-3.69126700	H
H-H_	0	1.26830300	4.19170800	-4.70183900	H
C-C_3	0	-0.75568200	2.15359200	0.27290500	H
H-H_	0	-0.98524800	1.10418000	0.05284900	H
H-H_	0	-0.51679900	2.18712300	1.33596400	H
H-H_	0	-1.64733800	2.74742300	0.07826000	H
H-H_	0	4.23144500	-0.08371800	4.54489400	H
C-C_3	0	4.14322000	-1.77237400	2.57354200	H
H-H_	0	3.99630200	-1.90379400	1.50126000	H
H-H_	0	5.01364900	-1.12722500	2.73220000	H
C-C_R	0	4.36288200	-3.12144700	3.21806200	H
C-C_R	0	5.42882500	-3.35524800	4.08577000	H
C-C_R	0	3.46193500	-4.15844100	2.95242400	H

C-C_R	0	5.60837300	-4.60754000	4.66871900	H
H-H_	0	6.13171400	-2.55614500	4.29810300	H
C-C_R	0	3.64282300	-5.40829000	3.53158100	H
H-H_	0	2.62782200	-3.97322100	2.27970500	H
C-C_R	0	4.71848600	-5.63811200	4.38675600	H
H-H_	0	6.44731000	-4.77980200	5.33507600	H
H-H_	0	2.93794200	-6.20338200	3.31765600	H
H-H_	0	4.86081100	-6.61532500	4.83462200	H
C-C_3	0	-1.61755800	1.73606000	-3.94215500	H
C-C_3	0	-0.40073700	0.83115200	-3.76803400	H
C-C_3	0	-0.70287800	-0.65001800	-4.03916600	H
C-C_3	0	-2.76293000	1.46436000	-2.96628700	H
C-C_R	0	-1.27164600	-1.33443700	-2.82372300	H
C-C_3	0	-3.30482400	0.03607900	-3.07532900	H
H-H_	0	-1.99084900	1.59649300	-4.96389600	H
H-H_	0	0.38843400	1.14943000	-4.45525800	H
H-H_	0	-1.39160900	-0.74727400	-4.88639300	H
H-H_	0	-2.43723300	1.64217700	-1.93290200	H
H-H_	0	-4.30666300	-0.03183700	-2.64591800	H
C-C_3	0	-0.98790800	-2.85954100	-1.00738200	H
H-H_	0	-0.10466800	-3.16206200	-0.43752300	H
C-C_3	0	-2.97394800	-1.38199100	-1.05559200	H
C-C_3	0	-1.83288600	-1.89526600	-0.19523000	H
H-H_	0	-1.19982800	-1.06801800	0.13794200	H
H-H_	0	-2.20683700	-2.37359300	0.70979900	H
H-H_	0	-3.43938100	-0.51144700	-0.58160800	H
N-N_R	0	-2.49206900	-0.95796600	-2.37807700	H
N-N_R	0	-0.52810300	-2.22010600	-2.23170400	H
H-H_	0	-3.40478100	-0.23210800	-4.13126700	H
H-H_	0	-3.58212800	2.16185400	-3.16878300	H
H-H_	0	-1.31206600	2.78344200	-3.86611000	H
H-H_	0	0.01801400	0.93214800	-2.75702200	H
H-H_	0	0.21204000	-1.17734100	-4.30983100	H
H-H_	0	-1.56064400	-3.75741600	-1.26957200	H
H-H_	0	-3.74764900	-2.14795200	-1.19289900	H
C-C_R	0	-0.30849500	-1.14795300	4.29039000	H
C-C_R	0	-0.63321000	0.20852700	4.43741900	H
C-C_R	0	-0.72006800	0.77385800	5.72591100	H

C-C_R	0	-0.46997000	-0.04973100	6.79822500	H
C-C_R	0	-0.12262600	-1.41833600	6.62412300	H
C-C_R	0	-0.03345600	-1.99305600	5.37749400	H
H-H_	0	-0.97087600	1.82091700	5.85035400	H
H-H_	0	-0.53298000	0.34835600	7.80578500	H
H-H_	0	0.07277700	-2.02392000	7.50138400	H
H-H_	0	0.22711700	-3.03297700	5.22242200	H
N-N_R	0	-0.76471400	0.76123900	3.19856200	H
N-N_R	0	-0.55788200	-0.19177800	2.31908500	H
N-N_R	0	-0.28983900	-1.32906400	2.94547100	H
O-O_R	0	0.00013000	-2.45077200	2.30012400	H
H-H_	0	1.04154400	-2.25244600	1.37347300	H

IM4$_{explicit}$ (ONIOM(M06-2X/6-31G(d, p):UFF))

0 1 0 1 0 1

C-C_3	0	4.35672000	5.97200100	-9.05399000	L
O-O_3	0	5.06087300	7.09754400	-8.58293700	L
C-C_3	0	4.19080800	7.82749800	-7.74806900	L
C-C_3	0	2.81106600	7.20506500	-7.78696100	L
C-C_3	0	3.08134600	5.81257700	-8.25650100	L
H-H_	0	4.11478100	6.12293600	-10.12851100	L
H-H_	0	4.99064500	5.06689500	-8.95833400	L
H-H_	0	4.58209500	7.80190100	-6.70946900	L
H-H_	0	4.15138000	8.88514300	-8.08459000	L
H-H_	0	2.32527300	7.21648200	-6.78942700	L
H-H_	0	2.17416400	7.74055700	-8.52493300	L
H-H_	0	3.25829100	5.14618100	-7.38454900	L
H-H_	0	2.25005200	5.41266800	-8.87615300	L
C-C_3	0	2.37117400	-4.41854400	10.89199300	L
O-O_3	0	1.94235200	-3.97263200	9.62921900	L
C-C_3	0	2.42695800	-2.66073200	9.45703100	L
C-C_3	0	3.10965300	-2.18835400	10.72773800	L
C-C_3	0	2.72004300	-3.21836400	11.74157000	L
H-H_	0	1.56563300	-5.01186800	11.37400700	L
H-H_	0	3.26297800	-5.07008500	10.76244100	L
H-H_	0	3.13482100	-2.63822800	8.60418100	L
H-H_	0	1.57503900	-1.99350600	9.21768000	L
H-H_	0	4.21286100	-2.18204700	10.59207300	L

H-H_	0	2.76924900	-1.17288500	11.02557900	L
H-H_	0	3.54976900	-3.44016900	12.44734900	L
H-H_	0	1.82267300	-2.87873200	12.30459900	L
C-C_3	0	-8.47971000	3.99406600	4.22997000	L
O-O_3	0	-9.76224000	4.57273000	4.31031100	L
C-C_3	0	-10.62350900	3.81830600	3.49082400	L
C-C_3	0	-9.92653400	2.53789300	3.09153900	L
C-C_3	0	-8.48382200	2.91075300	3.17551900	L
H-H_	0	-7.73490800	4.77517300	3.98007000	L
H-H_	0	-8.21647600	3.55699500	5.21681700	L
H-H_	0	-11.56249500	3.59615800	4.03998200	L
H-H_	0	-10.87212700	4.41362200	2.58551300	L
H-H_	0	-10.15743600	1.74130700	3.82913100	L
H-H_	0	-10.20676000	2.20098700	2.07298500	L
H-H_	0	-7.85143900	2.04568500	3.46252200	L
H-H_	0	-8.14385200	3.32896500	2.20301400	L
C-C_3	0	-5.84204800	-0.92982100	-4.79044400	L
O-O_3	0	-5.19751000	-2.17880600	-4.89240200	L
C-C_3	0	-6.06584300	-3.15170500	-4.36265000	L
C-C_3	0	-7.17124600	-2.46590100	-3.59401600	L
C-C_3	0	-7.24380700	-1.12619100	-4.25011400	L
H-H_	0	-5.26147900	-0.28008400	-4.10147900	L
H-H_	0	-5.87046700	-0.44490700	-5.78842900	L
H-H_	0	-6.49763000	-3.73918600	-5.19845300	L
H-H_	0	-5.49987300	-3.84414600	-3.70743800	L
H-H_	0	-8.13230300	-3.01645100	-3.67296200	L
H-H_	0	-6.88162100	-2.35176100	-2.52624200	L
H-H_	0	-7.97259800	-1.15181900	-5.08793400	L
H-H_	0	-7.52776100	-0.33205200	-3.52911700	L
C-C_3	0	-13.60444100	1.81199100	2.26051500	L
O-O_3	0	-14.24414400	2.68888000	3.16023300	L
C-C_3	0	-14.34316100	2.02857600	4.39872200	L
C-C_3	0	-13.34095600	0.89886100	4.43466800	L
C-C_3	0	-13.16655700	0.55386200	2.98876500	L
H-H_	0	-14.29943200	1.55767400	1.43165500	L
H-H_	0	-12.72256100	2.32707500	1.82751700	L
H-H_	0	-14.14598200	2.74627400	5.22342000	L
H-H_	0	-15.37397200	1.62738100	4.51381200	L

H-H_	0	-12.38025600	1.25889100	4.86312200	L
H-H_	0	-13.71789700	0.03427800	5.02197100	L
H-H_	0	-12.11160600	0.29133500	2.76197600	L
H-H_	0	-13.83136000	-0.29473900	2.71451800	L
C-C_3	0	-4.04483100	-9.96456400	-3.24091900	L
O-O_3	0	-3.71638700	-11.28011700	-3.62447800	L
C-C_3	0	-4.80473000	-11.80131700	-4.35007900	L
C-C_3	0	-5.75431200	-10.67772300	-4.70318000	L
C-C_3	0	-5.46725500	-9.65321500	-3.65349000	L
H-H_	0	-3.93230200	-9.85907600	-2.14379600	L
H-H_	0	-3.34345200	-9.26067500	-3.73349600	L
H-H_	0	-4.43617000	-12.30196100	-5.27031800	L
H-H_	0	-5.32643100	-12.55636100	-3.72318700	L
H-H_	0	-5.50753900	-10.27216300	-5.70917800	L
H-H_	0	-6.81535200	-11.00860200	-4.67734300	L
H-H_	0	-5.56976600	-8.62183400	-4.04799500	L
H-H_	0	-6.15017400	-9.79117600	-2.78969500	L
C-C_3	0	4.57128100	-3.83097200	-2.66009100	L
O-O_3	0	4.27195200	-4.74410900	-3.68876800	L
C-C_3	0	5.46118300	-5.42452300	-4.01659800	L
C-C_3	0	6.58829900	-4.95069800	-3.11949700	L
C-C_3	0	6.06918400	-3.66786600	-2.55431300	L
H-H_	0	4.08897200	-2.85583100	-2.87292300	L
H-H_	0	4.16787100	-4.22181500	-1.70392600	L
H-H_	0	5.30554400	-6.51553000	-3.90394900	L
H-H_	0	5.70906100	-5.22196100	-5.07921500	L
H-H_	0	6.76588900	-5.68197300	-2.30186600	L
H-H_	0	7.52644700	-4.79167500	-3.68914200	L
H-H_	0	6.39290200	-3.51496200	-1.50402200	L
H-H_	0	6.40914300	-2.81204700	-3.17530900	L
C-C_3	0	-12.16240700	-0.97713000	7.43838100	L
O-O_3	0	-12.52540300	-2.00892700	6.55089800	L
C-C_3	0	-12.19813900	-3.23582100	7.16351800	L
C-C_3	0	-11.69410600	-2.98262400	8.57029400	L
C-C_3	0	-11.28211800	-1.54644600	8.52735600	L
H-H_	0	-11.62735600	-0.17694500	6.88644100	L
H-H_	0	-13.08427100	-0.54302600	7.88280500	L
H-H_	0	-13.09226900	-3.89458800	7.18269600	L

H-H_	0	-11.40791300	-3.73499200	6.56484700	L
H-H_	0	-12.52169900	-3.11824900	9.30137800	L
H-H_	0	-10.84429900	-3.64833800	8.83501600	L
H-H_	0	-11.44255400	-1.03725600	9.50226200	L
H-H_	0	-10.21596900	-1.46730000	8.22750100	L
C-C_3	0	-13.12306600	-0.06076200	-5.99157600	L
O-O_3	0	-13.09940300	-1.38043000	-6.48856100	L
C-C_3	0	-11.75918600	-1.70476800	-6.77166600	L
C-C_3	0	-10.85567300	-0.72542500	-6.06025700	L
C-C_3	0	-11.71256500	0.49473600	-5.94362600	L
H-H_	0	-13.76544500	0.57109700	-6.64150600	L
H-H_	0	-13.56022600	-0.07091500	-4.97013500	L
H-H_	0	-11.54520000	-2.74208800	-6.43965300	L
H-H_	0	-11.59615900	-1.64292300	-7.86988200	L
H-H_	0	-10.59891100	-1.10987200	-5.05047100	L
H-H_	0	-9.92696700	-0.52599500	-6.63516000	L
H-H_	0	-11.51903600	1.04566200	-4.99801300	L
H-H_	0	-11.53507000	1.16649000	-6.81263300	L
C-C_3	0	12.31920500	-3.46221400	2.06510300	L
O-O_3	0	11.67201600	-4.08879300	3.14406800	L
C-C_3	0	12.29249100	-3.63477600	4.32516600	L
C-C_3	0	13.25018200	-2.49997900	4.00215600	L
C-C_3	0	12.97278600	-2.19520700	2.56228900	L
H-H_	0	13.08761800	-4.15092000	1.65156900	L
H-H_	0	11.58048800	-3.23179900	1.27044400	L
H-H_	0	11.50998300	-3.28124500	5.02901100	L
H-H_	0	12.83783100	-4.47742500	4.80168000	L
H-H_	0	13.06773000	-1.61252200	4.64677800	L
H-H_	0	14.30219200	-2.84029300	4.12463100	L
H-H_	0	12.26055100	-1.34806500	2.48450100	L
H-H_	0	13.90202200	-1.96042100	1.99910700	L
C-C_3	0	-7.15149100	8.37669000	-9.32966300	L
O-O_3	0	-6.54294900	9.42629700	-8.61211500	L
C-C_3	0	-5.94005700	8.86902400	-7.46633700	L
C-C_3	0	-6.31928000	7.40691800	-7.35876700	L
C-C_3	0	-6.69865400	7.05040500	-8.75848900	L
H-H_	0	-8.25536000	8.47117300	-9.23676700	L
H-H_	0	-6.87870200	8.44914900	-10.40396400	L

H-H_	0	-4.83746300	8.96881000	-7.55627000	L
H-H_	0	-6.26774400	9.42504000	-6.56310500	L
H-H_	0	-5.47782900	6.78560500	-6.98983700	L
H-H_	0	-7.19705300	7.29067500	-6.68733500	L
H-H_	0	-5.80677700	6.68257500	-9.31276500	L
H-H_	0	-7.50597200	6.28634500	-8.78598300	L
C-C_3	0	-0.33253500	5.51836800	5.38498800	L
O-O_3	0	0.86434900	5.89292700	6.01331800	L
C-C_3	0	1.59917600	6.63720500	5.07351100	L
C-C_3	0	0.66101100	7.15811000	3.99966300	L
C-C_3	0	-0.67414800	6.57075100	4.35801100	L
H-H_	0	-0.19564700	4.53025800	4.89539400	L
H-H_	0	-1.13992600	5.43067600	6.14058500	L
H-H_	0	2.09008600	7.48625800	5.59161200	L
H-H_	0	2.38531400	5.99043500	4.62627700	L
H-H_	0	0.61852600	8.26760300	4.01125200	L
H-H_	0	0.99019400	6.81113200	2.99747400	L
H-H_	0	-1.32201400	7.34865500	4.81243500	L
H-H_	0	-1.18487500	6.12851800	3.47848900	L
C-C_3	0	0.59702200	-6.99580900	8.74971300	L
O-O_3	0	-0.36345900	-8.01034700	8.57831700	L
C-C_3	0	0.32635500	-9.22317500	8.37755100	L
C-C_3	0	1.82249800	-8.97439800	8.41137500	L
C-C_3	0	1.93634500	-7.62745400	9.05253500	L
H-H_	0	0.66163400	-6.39630800	7.81611700	L
H-H_	0	0.28719000	-6.32825900	9.58016800	L
H-H_	0	0.04390400	-9.93188300	9.18545700	L
H-H_	0	0.02706200	-9.66419500	7.40310500	L
H-H_	0	2.35922100	-9.75296700	8.99592000	L
H-H_	0	2.22568000	-8.94060100	7.37702400	L
H-H_	0	2.06349700	-7.73835700	10.15213700	L
H-H_	0	2.77804300	-7.03547300	8.63341200	L
C-C_3	0	5.06270500	8.22079700	5.48296500	L
O-O_3	0	4.41221500	9.04013300	6.42188200	L
C-C_3	0	5.27106600	10.12385700	6.69841400	L
C-C_3	0	6.46273100	10.09120400	5.75530300	L
C-C_3	0	6.08926900	9.04760900	4.74921800	L
H-H_	0	5.55343100	7.38141700	6.01762500	L

H-H_	0	4.32701300	7.80193900	4.76669500	L
H-H_	0	4.70776300	11.07183600	6.56492500	L
H-H_	0	5.61069500	10.06670100	7.75477100	L
H-H_	0	6.63247000	11.07782900	5.27129300	L
H-H_	0	7.37918900	9.78405400	6.30497800	L
H-H_	0	5.62110900	9.52838800	3.86388200	L
H-H_	0	6.96029500	8.43555800	4.43359100	L
C-C_3	0	7.75733200	-3.44409100	-6.79734500	L
O-O_3	0	7.25817600	-2.41286700	-5.98583200	L
C-C_3	0	5.87144000	-2.34746500	-6.21723400	L
C-C_3	0	5.53620000	-3.05291900	-7.51972800	L
C-C_3	0	6.85538600	-3.58348400	-8.00189400	L
H-H_	0	8.79435400	-3.20374400	-7.11360900	L
H-H_	0	7.77538500	-4.39207500	-6.21748700	L
H-H_	0	5.33941900	-2.82379000	-5.37218700	L
H-H_	0	5.55433200	-1.28663400	-6.26092700	L
H-H_	0	4.82011500	-3.88376900	-7.33660400	L
H-H_	0	5.10282200	-2.34445600	-8.25764500	L
H-H_	0	6.78011400	-4.64185700	-8.32886900	L
H-H_	0	7.23740900	-2.95558100	-8.83701200	L
C-C_3	0	8.50661900	11.75670600	0.08991700	L
O-O_3	0	8.97386900	11.41013800	1.37413500	L
C-C_3	0	7.91379800	11.59784600	2.28331700	L
C-C_3	0	6.78559200	12.33527500	1.59575400	L
C-C_3	0	7.01958600	12.03535300	0.14930400	L
H-H_	0	9.04702500	12.66468900	-0.25458500	L
H-H_	0	8.71700100	10.93038400	-0.62201000	L
H-H_	0	7.55833800	10.60560700	2.63014100	L
H-H_	0	8.27223500	12.17001500	3.16521100	L
H-H_	0	5.79022300	11.98025900	1.93882600	L
H-H_	0	6.87941500	13.42882100	1.77703800	L
H-H_	0	6.45529600	11.12749200	-0.14998400	L
H-H_	0	6.73027800	12.88936800	-0.50088200	L
C-C_3	0	8.26009700	4.85451300	7.90332000	L
O-O_3	0	9.11982900	5.71959900	7.19869400	L
C-C_3	0	8.70443800	7.04202100	7.45866000	L
C-C_3	0	7.56691400	7.03232500	8.45971700	L
C-C_3	0	7.05178600	5.63096000	8.37826400	L

H-H_	0	7.94916100	4.01615500	7.24635300	L
H-H_	0	8.80744100	4.43469200	8.77514000	L
H-H_	0	9.56047200	7.63336100	7.84771400	L
H-H_	0	8.36424400	7.50405000	6.50868600	L
H-H_	0	7.95832800	7.23428400	9.48140300	L
H-H_	0	6.78164700	7.77575300	8.20485500	L
H-H_	0	6.68833500	5.26986700	9.36435200	L
H-H_	0	6.23577100	5.56371700	7.62564800	L
C-C_3	0	4.41450600	-12.58481700	-0.52480900	L
O-O_3	0	3.38948000	-13.52458700	-0.29501900	L
C-C_3	0	3.10416400	-13.51683900	1.08545200	L
C-C_3	0	3.84765000	-12.37657100	1.74567100	L
C-C_3	0	4.97630500	-12.11995000	0.80110300	L
H-H_	0	5.21264900	-13.04178300	-1.14762800	L
H-H_	0	3.98818700	-11.72021400	-1.07383300	L
H-H_	0	2.01120000	-13.40786900	1.24490900	L
H-H_	0	3.43048100	-14.48456800	1.52418900	L
H-H_	0	3.19354600	-11.48086300	1.79914100	L
H-H_	0	4.20747000	-12.64760400	2.76175600	L
H-H_	0	5.26053200	-11.04655500	0.77947700	L
H-H_	0	5.85684200	-12.73933700	1.08150700	L
C-C_3	0	-4.05355500	-9.69206700	1.62812000	L
O-O_3	0	-2.74997500	-9.45480400	1.16016200	L
C-C_3	0	-2.79911100	-8.32077400	0.32618000	L
C-C_3	0	-4.23020900	-7.83417400	0.20452300	L
C-C_3	0	-5.03193600	-8.98429000	0.72058700	L
H-H_	0	-4.25365200	-10.78400800	1.64327000	L
H-H_	0	-4.13752400	-9.29777600	2.66273200	L
H-H_	0	-2.14786000	-7.52632200	0.74367200	L
H-H_	0	-2.41338800	-8.59941700	-0.67483800	L
H-H_	0	-4.38921000	-6.94611000	0.85318900	L
H-H_	0	-4.49392700	-7.58972000	-0.84738600	L
H-H_	0	-5.94017100	-8.64614200	1.26305700	L
H-H_	0	-5.31994500	-9.65175300	-0.11859300	L
C-C_3	0	1.65812900	12.60761500	5.33662500	L
O-O_3	0	2.74072600	13.01647600	4.53278300	L
C-C_3	0	3.17811500	11.89328500	3.80147000	L
C-C_3	0	2.24189300	10.72930000	4.05328700	L

C-C_3	0	1.58815300	11.09751400	5.34471000	L
H-H_	0	1.78922400	12.99487700	6.36940500	L
H-H_	0	0.71819000	13.02696700	4.91548300	L
H-H_	0	3.20756900	12.14037300	2.72118000	L
H-H_	0	4.20662700	11.63117800	4.12758200	L
H-H_	0	1.47972300	10.67346400	3.24516200	L
H-H_	0	2.78670100	9.76423400	4.12827200	L
H-H_	0	0.54265500	10.73077300	5.40083700	L
H-H_	0	2.17664400	10.69633400	6.19735500	L
C-C_3	0	5.27926300	-3.58595000	6.71619400	L
O-O_3	0	5.77195700	-2.42937000	7.34245500	L
C-C_3	0	6.55599200	-2.86258800	8.42521000	L
C-C_3	0	6.07451000	-4.23084400	8.86914400	L
C-C_3	0	5.21091400	-4.70573900	7.73447900	L
H-H_	0	4.27139800	-3.37759100	6.30238800	L
H-H_	0	5.95503300	-3.86929400	5.88009600	L
H-H_	0	7.61936000	-2.91368500	8.10830600	L
H-H_	0	6.47260600	-2.13211600	9.25774100	L
H-H_	0	6.93166400	-4.91821700	9.03762000	L
H-H_	0	5.47897900	-4.15236000	9.80400200	L
H-H_	0	5.59911700	-5.64960600	7.30157000	L
H-H_	0	4.16587300	-4.86054300	8.07884100	L
C-C_3	0	1.93722100	12.87741800	-0.14049900	L
O-O_3	0	1.38372600	12.12019700	-1.18680100	L
C-C_3	0	2.34384800	12.07999100	-2.21716600	L
C-C_3	0	3.68222200	12.57524200	-1.69234000	L
C-C_3	0	3.44142600	12.77680300	-0.22688800	L
H-H_	0	1.61645400	13.93722600	-0.24728000	L
H-H_	0	1.57416400	12.49456000	0.83632000	L
H-H_	0	2.44038700	11.03523600	-2.57814200	L
H-H_	0	2.00215900	12.71618000	-3.06151800	L
H-H_	0	4.49188200	11.83568200	-1.86961300	L
H-H_	0	3.95104700	13.54085500	-2.17457300	L
H-H_	0	3.79293400	11.88873300	0.33962600	L
H-H_	0	3.94062900	13.69285100	0.15680900	L
C-C_3	0	-0.92631700	-12.29635100	-5.44870700	L
O-O_3	0	-1.66523000	-13.06352500	-6.37169700	L
C-C_3	0	-2.02146200	-12.21285700	-7.43312500	L

C-C_3	0	-1.99845900	-10.78540700	-6.93997900	L
C-C_3	0	-1.00081000	-10.82575800	-5.82388400	L
H-H_	0	-1.34844100	-12.45205400	-4.43316200	L
H-H_	0	0.12900200	-12.64278000	-5.44227300	L
H-H_	0	-1.29575000	-12.34418500	-8.26494700	L
H-H_	0	-3.03507000	-12.47798400	-7.80189200	L
H-H_	0	-1.69707900	-10.07680300	-7.74167100	L
H-H_	0	-2.99975000	-10.50854600	-6.54609800	L
H-H_	0	-0.01119000	-10.46534300	-6.18000300	L
H-H_	0	-1.33573800	-10.20771300	-4.96585900	L
C-C_3	0	12.73993500	2.34883700	5.48758900	L
O-O_3	0	12.53918500	3.54969200	4.78032700	L
C-C_3	0	11.22886200	3.52687500	4.26096100	L
C-C_3	0	10.54550500	2.23086400	4.64919400	L
C-C_3	0	11.67362300	1.35406900	5.08942500	L
H-H_	0	13.74968900	1.94462500	5.26232200	L
H-H_	0	12.67561000	2.55788000	6.57781400	L
H-H_	0	10.65921000	4.39324600	4.65696700	L
H-H_	0	11.27780600	3.61505600	3.15430900	L
H-H_	0	9.85308400	2.40860900	5.50144100	L
H-H_	0	9.99100300	1.78790600	3.79456400	L
H-H_	0	11.38203100	0.69883900	5.93884700	L
H-H_	0	12.02747200	0.73142200	4.24036600	L
C-C_3	0	5.05085600	-3.77362700	-11.67464900	L
O-O_3	0	3.78543200	-4.32546600	-11.40926100	L
C-C_3	0	4.01138700	-5.40950800	-10.54527300	L
C-C_3	0	5.37736700	-5.98905500	-10.85544400	L
C-C_3	0	6.08937000	-4.87622100	-11.57777500	L
H-H_	0	5.05495000	-3.32323300	-12.68987200	L
H-H_	0	5.26721200	-2.97353200	-10.93206300	L
H-H_	0	3.96991300	-5.05292500	-9.49274800	L
H-H_	0	3.22166600	-6.17627300	-10.69070600	L
H-H_	0	5.91272900	-6.27806100	-9.92660400	L
H-H_	0	5.27701500	-6.87811500	-11.51636300	L
H-H_	0	6.98165900	-4.53155900	-11.01104500	L
H-H_	0	6.40047100	-5.20912500	-12.59245300	L
C-C_3	0	7.15319900	4.51997500	-3.85309700	L
O-O_3	0	6.89911700	4.26665600	-5.21208100	L

C-C_3	0	5.55234900	4.59198400	-5.46236900	L
C-C_3	0	4.96044000	5.30883800	-4.26328700	L
C-C_3	0	6.11554400	5.48154700	-3.32412000	L
H-H_	0	7.11211900	3.56313000	-3.29136700	L
H-H_	0	8.16929000	4.95171900	-3.73751100	L
H-H_	0	5.50334800	5.24321500	-6.35777600	L
H-H_	0	4.98003500	3.66702000	-5.68376700	L
H-H_	0	4.53291800	6.29182300	-4.55438100	L
H-H_	0	4.17033800	4.68419700	-3.79360400	L
H-H_	0	6.50106800	6.52335500	-3.37504800	L
H-H_	0	5.83324100	5.23563300	-2.27711300	L
C-C_3	0	-2.80762300	3.65475000	3.10730900	L
O-O_3	0	-3.50433300	4.28742100	4.15195700	L
C-C_3	0	-4.45988300	3.36901200	4.63181700	L
C-C_3	0	-4.41693000	2.10230800	3.79632900	L
C-C_3	0	-3.64988400	2.51704300	2.58382300	L
H-H_	0	-2.59214600	4.38498900	2.29952200	L
H-H_	0	-1.84960500	3.26085400	3.50693600	L
H-H_	0	-4.25240100	3.14280300	5.69746300	L
H-H_	0	-5.46637300	3.82951800	4.56153800	L
H-H_	0	-3.86801400	1.30262000	4.34085900	L
H-H_	0	-5.43262500	1.75157100	3.52433400	L
H-H_	0	-3.03728200	1.68930700	2.17100100	L
H-H_	0	-4.34726900	2.88984900	1.80525900	L
C-C_3	0	1.75538200	6.65906000	-3.65469600	L
O-O_3	0	0.43666200	6.31295500	-3.31925600	L
C-C_3	0	-0.39411700	7.34706400	-3.79310700	L
C-C_3	0	0.45614300	8.51723900	-4.25401100	L
C-C_3	0	1.83527300	8.15688900	-3.80026400	L
H-H_	0	2.44979100	6.29908400	-2.87032700	L
H-H_	0	2.02605900	6.17813300	-4.61636300	L
H-H_	0	-1.02326600	6.96274500	-4.62178500	L
H-H_	0	-1.06574100	7.67306200	-2.97578100	L
H-H_	0	0.42841900	8.58947900	-5.36108500	L
H-H_	0	0.12328000	9.47690500	-3.80320000	L
H-H_	0	2.60561900	8.45788900	-4.54186800	L
H-H_	0	2.04744300	8.62102800	-2.81340100	L
C-C_3	0	-10.36487000	3.47698800	-0.59816800	L

O-O_3	0	-9.56597600	4.57868400	-0.24213600	L
C-C_3	0	-9.19917700	5.20458000	-1.44423800	L
C-C_3	0	-10.29832000	4.97043500	-2.45628600	L
C-C_3	0	-10.97830900	3.72298100	-1.96523900	L
H-H_	0	-9.73670900	2.56073700	-0.61417000	L
H-H_	0	-11.16638700	3.34637800	0.15759500	L
H-H_	0	-9.05367700	6.29233300	-1.27237200	L
H-H_	0	-8.24215300	4.76911700	-1.80543700	L
H-H_	0	-11.01017600	5.82427300	-2.44701000	L
H-H_	0	-9.88485200	4.83764000	-3.47798900	L
H-H_	0	-12.07521100	3.88148900	-1.88327600	L
H-H_	0	-10.78100400	2.86557200	-2.64493200	L
C-C_3	0	-7.34750600	-0.79825700	4.60506600	L
O-O_3	0	-7.69362200	-1.25189100	3.31684600	L
C-C_3	0	-9.09612800	-1.24191200	3.22687900	L
C-C_3	0	-9.67588800	-1.20705100	4.62104100	L
C-C_3	0	-8.60164700	-0.53282200	5.41408000	L
H-H_	0	-6.72983800	-1.57241900	5.10364900	L
H-H_	0	-6.74059400	0.12332900	4.52151900	L
H-H_	0	-9.41321700	-0.34397500	2.65593400	L
H-H_	0	-9.44361500	-2.14493200	2.68613800	L
H-H_	0	-10.63387700	-0.64774300	4.65425300	L
H-H_	0	-9.82943200	-2.24217400	4.99328300	L
H-H_	0	-8.79373900	0.56040300	5.47191800	L
H-H_	0	-8.52401100	-0.95008800	6.43997900	L
C-C_3	0	-1.48194900	-1.55920600	11.85839200	L
O-O_3	0	-0.31892800	-1.44738500	11.07110300	L
C-C_3	0	-0.30274200	-0.15365900	10.51082000	L
C-C_3	0	-1.42111000	0.66930700	11.11498400	L
C-C_3	0	-2.38232000	-0.37265500	11.58954500	L
H-H_	0	-2.00672900	-2.50898400	11.62239000	L
H-H_	0	-1.18812700	-1.57557700	12.93041500	L
H-H_	0	0.68023300	0.32741100	10.70086400	L
H-H_	0	-0.44447500	-0.23871600	9.41226000	L
H-H_	0	-1.03739900	1.25469200	11.97975700	L
H-H_	0	-1.88475500	1.35358800	10.37405400	L
H-H_	0	-2.92902200	-0.04918500	12.50171700	L
H-H_	0	-3.10488600	-0.61894200	10.78125900	L

C-C_3	0	10.11264300	8.79878200	4.15546800	L
O-O_3	0	11.15773700	9.44248100	3.46477000	L
C-C_3	0	12.00756400	8.44504700	2.94433300	L
C-C_3	0	11.41821100	7.07616100	3.22082700	L
C-C_3	0	10.45680600	7.33683500	4.33484400	L
H-H_	0	9.95690100	9.28550700	5.14168900	L
H-H_	0	9.17935800	8.89705000	3.56304500	L
H-H_	0	12.13809300	8.59917600	1.85234900	L
H-H_	0	13.00195600	8.53231000	3.43293000	L
H-H_	0	10.86940600	6.70376700	2.32918000	L
H-H_	0	12.20018400	6.34193000	3.51252400	L
H-H_	0	9.55687800	6.69020700	4.26123100	L
H-H_	0	10.95963200	7.18071500	5.31476000	L
C-C_3	0	8.66420000	1.82940200	0.46225700	L
O-O_3	0	7.71191200	2.63601800	1.10355600	L
C-C_3	0	8.12866100	3.96289100	0.90462600	L
C-C_3	0	8.93099400	4.03695800	-0.38115200	L
C-C_3	0	9.22899900	2.60029400	-0.71262400	L
H-H_	0	9.47258500	1.57388300	1.18195600	L
H-H_	0	8.18438600	0.88897000	0.12068900	L
H-H_	0	7.23716900	4.62299600	0.84728200	L
H-H_	0	8.75714600	4.28236800	1.76495800	L
H-H_	0	8.33015700	4.50519300	-1.18854700	L
H-H_	0	9.86582500	4.61911500	-0.23138000	L
H-H_	0	8.71205200	2.30702400	-1.65181200	L
H-H_	0	10.31947000	2.42365500	-0.81699200	L
C-C_3	0	-8.76723500	-7.78953200	-3.58373600	L
O-O_3	0	-7.77077600	-6.84274800	-3.89288100	L
C-C_3	0	-7.20627200	-6.40187500	-2.67953900	L
C-C_3	0	-7.73498300	-7.24867700	-1.54202200	L
C-C_3	0	-9.01710600	-7.78527700	-2.09111400	L
H-H_	0	-9.69958400	-7.54609500	-4.13447000	L
H-H_	0	-8.41968600	-8.79483800	-3.90624300	L
H-H_	0	-6.09975200	-6.47306100	-2.73436000	L
H-H_	0	-7.48492600	-5.33856300	-2.51990800	L
H-H_	0	-7.03331000	-8.08253400	-1.32951800	L
H-H_	0	-7.89690800	-6.65410300	-0.62087000	L
H-H_	0	-9.24049900	-8.80309700	-1.70501500	L

H-H_	0	-9.85430900	-7.09464000	-1.84696700	L
C-C_3	0	1.92501400	0.29360200	-10.85704800	L
O-O_3	0	3.01613200	-0.53016500	-10.51221500	L
C-C_3	0	2.53339000	-1.47758400	-9.59449000	L
C-C_3	0	1.06260800	-1.68616000	-9.86343800	L
C-C_3	0	0.62986700	-0.34634400	-10.37829200	L
H-H_	0	2.05763400	1.29540200	-10.39342200	L
H-H_	0	1.90225500	0.41703400	-11.96083700	L
H-H_	0	3.08487600	-2.43216700	-9.70400300	L
H-H_	0	2.69274700	-1.09300400	-8.56487400	L
H-H_	0	0.92958500	-2.45900800	-10.65259500	L
H-H_	0	0.50881800	-1.98532700	-8.94904000	L
H-H_	0	-0.10812700	-0.45030300	-11.20340100	L
H-H_	0	0.18694600	0.25656700	-9.55596000	L
C-C_3	0	-3.23606600	-3.06928000	7.29949400	L
O-O_3	0	-3.27823800	-4.42953300	6.95443400	L
C-C_3	0	-2.18988000	-5.03039500	7.60792800	L
C-C_3	0	-1.87195200	-4.23635700	8.86146800	L
C-C_3	0	-2.60650500	-2.93789200	8.66985800	L
H-H_	0	-2.63901100	-2.51262200	6.54659100	L
H-H_	0	-4.26216600	-2.65686000	7.30261800	L
H-H_	0	-2.44260000	-6.07893100	7.86675600	L
H-H_	0	-1.31677800	-5.04116000	6.92227200	L
H-H_	0	-2.24449000	-4.76521900	9.76565100	L
H-H_	0	-0.77810500	-4.06982300	8.96050500	L
H-H_	0	-3.39056300	-2.82067100	9.44747300	L
H-H_	0	-1.91639600	-2.06945200	8.70658500	L
C-C_3	0	7.07190600	10.96547800	-3.62966300	L
O-O_3	0	6.44596900	9.81359700	-3.11681200	L
C-C_3	0	5.29830900	9.56516600	-3.89536500	L
C-C_3	0	5.10857500	10.68321100	-4.89925200	L
C-C_3	0	6.46541200	11.30759300	-4.97303600	L
H-H_	0	6.91617200	11.80569700	-2.91923400	L
H-H_	0	8.16327800	10.78488600	-3.73030200	L
H-H_	0	5.42815900	8.59988200	-4.42565400	L
H-H_	0	4.41198200	9.48709700	-3.23496400	L
H-H_	0	4.77638800	10.30061600	-5.88825100	L
H-H_	0	4.36988900	11.42070700	-4.51525100	L

H-H_	0	7.05793500	10.83696000	-5.78805600	L
H-H_	0	6.40663400	12.40613500	-5.13282900	L
C-C_3	0	10.23264200	-0.07308100	-6.14545800	L
O-O_3	0	9.38441300	1.04867400	-6.17263500	L
C-C_3	0	8.36394500	0.79705200	-5.24193700	L
C-C_3	0	8.91969600	-0.09101300	-4.15339100	L
C-C_3	0	10.07727500	-0.78666200	-4.81229200	L
H-H_	0	9.96260300	-0.75418600	-6.98222900	L
H-H_	0	11.28341900	0.25861900	-6.28529800	L
H-H_	0	8.00231200	1.75311400	-4.81500800	L
H-H_	0	7.51345400	0.29842100	-5.75114500	L
H-H_	0	9.27663200	0.53105700	-3.30618500	L
H-H_	0	8.15773700	-0.81318200	-3.79398600	L
H-H_	0	10.99606600	-0.68892300	-4.19413000	L
H-H_	0	9.85554300	-1.86483900	-4.96976700	L
C-C_3	0	-10.41577400	-0.42809100	-2.18029200	L
O-O_3	0	-9.99731500	-0.24015600	-0.84921700	L
C-C_3	0	-11.13827200	-0.29068200	-0.02637800	L
C-C_3	0	-12.32953800	-0.74665000	-0.83832000	L
C-C_3	0	-11.92854800	-0.41823100	-2.24112300	L
H-H_	0	-9.99715200	0.37672900	-2.82054300	L
H-H_	0	-10.02635000	-1.40321600	-2.54174100	L
H-H_	0	-10.95873800	-0.98834000	0.81649700	L
H-H_	0	-11.32755700	0.72303800	0.38309400	L
H-H_	0	-12.46748100	-1.84362500	-0.72441800	L
H-H_	0	-13.26012400	-0.21705100	-0.53897900	L
H-H_	0	-12.31834000	-1.16408400	-2.96742900	L
H-H_	0	-12.29016200	0.59830600	-2.50961500	L
C-C_3	0	9.90960500	-6.13565500	5.71629700	L
O-O_3	0	11.17318400	-6.34417200	6.30459200	L
C-C_3	0	11.23289500	-5.56819300	7.47877400	L
C-C_3	0	9.84984000	-5.05145700	7.80897600	L
C-C_3	0	9.16143400	-5.06316300	6.48219700	L
H-H_	0	10.03825400	-5.83586600	4.65568800	L
H-H_	0	9.34005400	-7.08856300	5.74799400	L
H-H_	0	11.62619400	-6.18409200	8.31528000	L
H-H_	0	11.92558200	-4.71493100	7.31151100	L
H-H_	0	9.34036400	-5.75082700	8.50816000	L

H-H_	0	9.88445500	-4.03153700	8.24941500	L
H-H_	0	8.08166900	-5.30363200	6.58190600	L
H-H_	0	9.28197400	-4.07759500	5.98059000	L
C-C_3	0	-4.92062700	2.93698900	-8.34705500	L
O-O_3	0	-5.16131800	1.99639400	-7.32491200	L
C-C_3	0	-4.38318300	2.36993100	-6.21467200	L
C-C_3	0	-3.97861900	3.81733200	-6.36716300	L
C-C_3	0	-3.94806000	3.99003200	-7.85210400	L
H-H_	0	-4.50876800	2.42101300	-9.24041200	L
H-H_	0	-5.88449200	3.41309100	-8.62878100	L
H-H_	0	-4.96195000	2.22300800	-5.28342400	L
H-H_	0	-3.48458400	1.72078100	-6.17255100	L
H-H_	0	-4.75180700	4.47788500	-5.92004000	L
H-H_	0	-2.99408300	4.02965900	-5.90100800	L
H-H_	0	-4.25957900	5.01083100	-8.15435600	L
H-H_	0	-2.92720300	3.77668600	-8.23835900	L
C-C_3	0	-2.84094700	12.88054300	-4.14489500	L
O-O_3	0	-2.13257100	11.86959600	-3.46323800	L
C-C_3	0	-2.91886500	10.70222100	-3.49685700	L
C-C_3	0	-4.00517900	10.86613100	-4.53519100	L
C-C_3	0	-4.18947900	12.34813400	-4.58987800	L
H-H_	0	-2.97102600	13.76014200	-3.47951400	L
H-H_	0	-2.25035200	13.19651500	-5.03099900	L
H-H_	0	-2.28227800	9.82856800	-3.74271500	L
H-H_	0	-3.37174100	10.54136300	-2.49422800	L
H-H_	0	-3.65047600	10.48893800	-5.52018200	L
H-H_	0	-4.94017600	10.34312500	-4.24343100	L
H-H_	0	-4.45388100	12.69138100	-5.61371200	L
H-H_	0	-4.97981500	12.65921800	-3.87105400	L
C-C_3	0	5.43501500	2.49779400	9.08862800	L
O-O_3	0	5.53806900	2.39601800	7.68603400	L
C-C_3	0	5.09552600	1.11076600	7.32631200	L
C-C_3	0	4.21252200	0.58021000	8.43020400	L
C-C_3	0	4.75616000	1.25872600	9.64685100	L
H-H_	0	6.44938400	2.61434200	9.52698700	L
H-H_	0	4.83823300	3.40053600	9.33866700	L
H-H_	0	4.53554900	1.15735200	6.36976500	L
H-H_	0	5.97836600	0.44832400	7.18851700	L

H-H_	0	3.15871200	0.89175700	8.26370800	L
H-H_	0	4.27499500	-0.52333900	8.50815500	L
H-H_	0	3.94625900	1.51768400	10.36358800	L
H-H_	0	5.50411600	0.60299500	10.14561700	L
C-C_3	0	-5.27382700	-0.15574400	-0.51029900	L
O-O_3	0	-5.79918300	0.66138500	-1.52945800	L
C-C_3	0	-6.88692900	1.37724300	-0.99048500	L
C-C_3	0	-6.97865200	1.11836200	0.49674500	L
C-C_3	0	-6.21961600	-0.15635600	0.66906300	L
H-H_	0	-5.12904500	-1.18581600	-0.89646500	L
H-H_	0	-4.28694400	0.24542400	-0.19726500	L
H-H_	0	-6.76007600	2.46288000	-1.18685200	L
H-H_	0	-7.81668600	1.04130700	-1.49155400	L
H-H_	0	-6.47415300	1.93746900	1.05280700	L
H-H_	0	-8.03216300	1.01940700	0.83647400	L
H-H_	0	-5.67338000	-0.17787500	1.63549600	L
H-H_	0	-6.91074400	-1.02374600	0.60072800	L
C-C_3	0	7.66648500	-5.60684500	1.14786300	L
O-O_3	0	8.31841100	-5.63023300	2.39560800	L
C-C_3	0	7.45996700	-5.02620000	3.33566600	L
C-C_3	0	6.14464900	-4.68613000	2.66990200	L
C-C_3	0	6.50349200	-4.64878600	1.22053900	L
H-H_	0	8.37700800	-5.29776500	0.35714800	L
H-H_	0	7.29755400	-6.62739500	0.91189100	L
H-H_	0	7.29440200	-5.71401700	4.19048500	L
H-H_	0	7.94252500	-4.10130100	3.71620700	L
H-H_	0	5.40593300	-5.49600000	2.84972400	L
H-H_	0	5.74037800	-3.71635900	3.02684700	L
H-H_	0	5.66278700	-4.97201500	0.57288400	L
H-H_	0	6.82996400	-3.62546800	0.93604000	L
C-C_3	0	10.91755600	3.36286500	-3.94433200	L
O-O_3	0	11.45025800	2.22644600	-3.30789200	L
C-C_3	0	12.76102300	2.03728000	-3.79008600	L
C-C_3	0	13.09292300	3.10335600	-4.81565100	L
C-C_3	0	11.76220800	3.69842800	-5.15168400	L
H-H_	0	9.87130700	3.16433300	-4.24994300	L
H-H_	0	10.91280000	4.21232700	-3.22963300	L
H-H_	0	13.47909200	2.08230700	-2.94421400	L

H-H_	0	12.83069200	1.03093000	-4.25465800	L
H-H_	0	13.75409100	3.87462100	-4.36377000	L
H-H_	0	13.58471100	2.67103900	-5.71426200	L
H-H_	0	11.83344500	4.79585800	-5.31134100	L
H-H_	0	11.34208900	3.20791100	-6.05758500	L
C-C_3	0	4.05481000	7.53620200	-0.59971200	L
O-O_3	0	4.62566100	6.82910000	0.47272100	L
C-C_3	0	4.40936700	7.61037700	1.61962400	L
C-C_3	0	4.33918000	9.06468200	1.20631200	L
C-C_3	0	4.02039200	9.01304800	-0.26054600	L
H-H_	0	4.64904900	7.36403900	-1.52102500	L
H-H_	0	3.02622700	7.16509300	-0.77020100	L
H-H_	0	3.45562400	7.30492400	2.10213000	L
H-H_	0	5.23961100	7.44930400	2.33819600	L
H-H_	0	3.55315300	9.60673300	1.77061000	L
H-H_	0	5.32028700	9.55714700	1.37050700	L
H-H_	0	3.01829100	9.44409000	-0.47177000	L
H-H_	0	4.78454100	9.56687200	-0.84233000	L
C-C_3	0	13.61707500	6.99214100	-0.98971400	L
O-O_3	0	13.44727400	6.64300000	0.36359200	L
C-C_3	0	13.13017800	5.27027500	0.41404200	L
C-C_3	0	13.14162100	4.69119400	-0.98732300	L
C-C_3	0	13.03081200	5.90238700	-1.85802400	L
H-H_	0	13.11806200	7.96283700	-1.19374900	L
H-H_	0	14.70353000	7.10113100	-1.19976200	L
H-H_	0	13.86423200	4.74470600	1.05910300	L
H-H_	0	12.12108900	5.15026900	0.86201800	L
H-H_	0	14.10653600	4.17181300	-1.17871100	L
H-H_	0	12.29861300	3.98718500	-1.15012900	L
H-H_	0	13.59330900	5.78531200	-2.80876000	L
H-H_	0	11.96240000	6.12246100	-2.07436500	L
C-C_3	0	-2.16728900	7.38037700	-7.33147500	L
O-O_3	0	-2.47716500	7.93951800	-8.58348600	L
C-C_3	0	-2.06444600	7.00333700	-9.54571600	L
C-C_3	0	-0.89745900	6.21790600	-8.98352400	L
C-C_3	0	-1.01457600	6.40689300	-7.49595700	L
H-H_	0	-1.89748500	8.19041500	-6.62236600	L
H-H_	0	-3.05902400	6.85134700	-6.93500300	L

H-H_	0	-2.91130800	6.32305400	-9.78419000	L
H-H_	0	-1.76441100	7.53188800	-10.47534800	L
H-H_	0	-0.96008700	5.14477200	-9.26677100	L
H-H_	0	0.06019800	6.64494300	-9.35217400	L
H-H_	0	-1.23546100	5.44593600	-6.98413300	L
H-H_	0	-0.07757100	6.83474800	-7.08223500	L
C-C_3	0	10.30082400	-1.19822700	-0.62483200	L
O-O_3	0	11.40332700	-1.82303500	-1.24147600	L
C-C_3	0	10.95672800	-3.04228000	-1.78592300	L
C-C_3	0	9.44688700	-3.09586100	-1.72254300	L
C-C_3	0	9.12017100	-2.14467200	-0.61721900	L
H-H_	0	10.56706500	-0.90479900	0.41164600	L
H-H_	0	10.04865000	-0.28087300	-1.19499000	L
H-H_	0	11.30531100	-3.13280700	-2.83647400	L
H-H_	0	11.38880500	-3.87858400	-1.19569100	L
H-H_	0	9.01341800	-2.73742700	-2.67917300	L
H-H_	0	9.07768700	-4.11872400	-1.51035600	L
H-H_	0	8.15969300	-1.61453300	-0.79679500	L
H-H_	0	9.07888900	-2.68902000	0.35171500	L
C-C_3	0	-8.84853500	-8.99392100	4.96707800	L
O-O_3	0	-8.00415200	-8.87930600	6.09013700	L
C-C_3	0	-6.71566000	-8.54453400	5.62712200	L
C-C_3	0	-6.68594400	-8.62236200	4.11631300	L
C-C_3	0	-8.12587900	-8.48380000	3.73894400	L
H-H_	0	-9.11828800	-10.06349400	4.82886900	L
H-H_	0	-9.77976100	-8.41232500	5.13745000	L
H-H_	0	-6.47203700	-7.51216800	5.95446300	L
H-H_	0	-5.96723400	-9.23744000	6.06717900	L
H-H_	0	-6.06322500	-7.81679900	3.67040400	L
H-H_	0	-6.30566500	-9.61794500	3.80102800	L
H-H_	0	-8.37630400	-7.41441700	3.57015600	L
H-H_	0	-8.37548300	-9.07654100	2.83211200	L
C-C_3	0	-8.82482900	-5.04083900	-6.56739700	L
O-O_3	0	-7.75809300	-5.88151100	-6.94276000	L
C-C_3	0	-7.19741500	-5.34050400	-8.11233800	L
C-C_3	0	-7.49222400	-3.86048300	-8.14777200	L
C-C_3	0	-8.76096600	-3.74489000	-7.35790400	L
H-H_	0	-9.78812600	-5.56202400	-6.75462100	L

H-H_	0	-8.74921800	-4.82582200	-5.48161300	L
H-H_	0	-6.10212300	-5.51454200	-8.12134700	L
H-H_	0	-7.64530400	-5.84302100	-8.99778600	L
H-H_	0	-6.67423100	-3.29748600	-7.64678200	L
H-H_	0	-7.62079700	-3.49703300	-9.18998600	L
H-H_	0	-8.73691200	-2.86013400	-6.68698500	L
H-H_	0	-9.63415800	-3.66987100	-8.04262000	L
C-C_3	0	-1.74076300	8.44881800	1.25766500	L
O-O_3	0	-0.69672900	9.24029600	1.77568700	L
C-C_3	0	0.38615000	9.15856500	0.87798300	L
C-C_3	0	-0.05785600	8.44179800	-0.37668700	L
C-C_3	0	-1.21951000	7.63222000	0.09897300	L
H-H_	0	-2.14176700	7.78689200	2.05109700	L
H-H_	0	-2.55852700	9.11330300	0.90899800	L
H-H_	0	0.74855000	10.17742600	0.63545500	L
H-H_	0	1.21389000	8.59708000	1.35961100	L
H-H_	0	-0.39139000	9.18253200	-1.13660800	L
H-H_	0	0.74259600	7.80052800	-0.79814500	L
H-H_	0	-1.98697200	7.50399500	-0.69144100	L
H-H_	0	-0.87146400	6.63967500	0.46070500	L
C-C_3	0	-6.08464300	1.30396500	7.39887800	L
O-O_3	0	-5.62323800	2.30018700	8.28345700	L
C-C_3	0	-4.33640600	1.92545800	8.71533100	L
C-C_3	0	-3.79889100	0.85511500	7.79434500	L
C-C_3	0	-5.04302900	0.20988500	7.27614400	L
H-H_	0	-7.03906700	0.88250900	7.77744000	L
H-H_	0	-6.27601100	1.76484600	6.40674700	L
H-H_	0	-3.66491600	2.80984700	8.71353100	L
H-H_	0	-4.40482900	1.53385200	9.75344500	L
H-H_	0	-3.23586700	1.32246400	6.95788700	L
H-H_	0	-3.14784700	0.13250600	8.33064900	L
H-H_	0	-4.91584900	-0.13715300	6.22869900	L
H-H_	0	-5.32502000	-0.64384500	7.92706500	L
C-C_3	0	-7.67905500	2.72692700	-4.17801200	L
O-O_3	0	-7.35412400	4.04180600	-4.56223400	L
C-C_3	0	-7.91289900	4.26749100	-5.83668500	L
C-C_3	0	-8.79582400	3.09846700	-6.21541500	L
C-C_3	0	-8.27180900	1.99327900	-5.35776900	L

H-H_	0	-6.77209100	2.20543200	-3.81128100	L
H-H_	0	-8.41526700	2.76340600	-3.34911400	L
H-H_	0	-8.49813000	5.20892200	-5.82764700	L
H-H_	0	-7.09266200	4.37547000	-6.57682400	L
H-H_	0	-9.85206900	3.31730500	-5.94978800	L
H-H_	0	-8.72160800	2.85530000	-7.29732500	L
H-H_	0	-9.07196500	1.29550600	-5.04055000	L
H-H_	0	-7.47671100	1.43854000	-5.89806100	L
C-C_3	0	0.02426300	-6.67535600	-9.74747400	L
O-O_3	0	-0.00048700	-7.92849200	-9.10298500	L
C-C_3	0	0.59958000	-7.77438600	-7.83755300	L
C-C_3	0	1.22241100	-6.39975200	-7.73788600	L
C-C_3	0	0.48350200	-5.61257600	-8.77183200	L
H-H_	0	0.72626100	-6.72911300	-10.60727600	L
H-H_	0	-0.98927400	-6.43352000	-10.13298600	L
H-H_	0	-0.17889100	-7.89643800	-7.05600100	L
H-H_	0	1.36946900	-8.55854300	-7.68488200	L
H-H_	0	1.09810100	-5.97109300	-6.72308900	L
H-H_	0	2.30089900	-6.44842400	-7.99913400	L
H-H_	0	-0.39763500	-5.11316600	-8.31573100	L
H-H_	0	1.13559700	-4.85444700	-9.25774700	L
C-C_3	0	5.44569700	-10.04705400	7.90810400	L
O-O_3	0	6.59033900	-10.54853100	7.26197100	L
C-C_3	0	7.56307800	-9.53828900	7.34303100	L
C-C_3	0	6.85968700	-8.19848300	7.37414600	L
C-C_3	0	5.47531200	-8.52914700	7.86174800	L
H-H_	0	4.53631700	-10.43184900	7.39899800	L
H-H_	0	5.43514800	-10.40088700	8.96244700	L
H-H_	0	8.16260000	-9.67968500	8.26959300	L
H-H_	0	8.24418800	-9.60217800	6.46871000	L
H-H_	0	7.37662100	-7.48906200	8.05628600	L
H-H_	0	6.81252700	-7.76409700	6.35196800	L
H-H_	0	5.29435100	-8.10010300	8.87172900	L
H-H_	0	4.70975900	-8.14353500	7.15516800	L
C-C_3	0	12.49741200	-7.53668600	2.32447300	L
O-O_3	0	13.32629600	-7.12853400	1.25924100	L
C-C_3	0	12.49542900	-6.82564000	0.16202400	L
C-C_3	0	11.05715400	-6.77060400	0.62638500	L

C-C_3	0	11.06738300	-7.64704400	1.83811200	L
H-H_	0	12.84865000	-8.51349800	2.71986000	L
H-H_	0	12.56237400	-6.78266800	3.13866800	L
H-H_	0	12.79828900	-5.85379000	-0.28173300	L
H-H_	0	12.61323900	-7.61869000	-0.60841300	L
H-H_	0	10.79044300	-5.72960400	0.90991300	L
H-H_	0	10.35616500	-7.14188800	-0.15054700	L
H-H_	0	10.34396400	-7.30541900	2.60754400	L
H-H_	0	10.84233800	-8.69777600	1.54962900	L
C-C_3	0	-3.04069500	-2.08892000	-10.21979700	L
O-O_3	0	-2.73573900	-0.77225100	-9.81807600	L
C-C_3	0	-2.75182200	-0.74266300	-8.41137500	L
C-C_3	0	-3.48957400	-1.95791300	-7.90154700	L
C-C_3	0	-3.28727900	-2.95434400	-8.99824800	L
H-H_	0	-3.95112400	-2.06631500	-10.85652100	L
H-H_	0	-2.20009700	-2.49798400	-10.82062400	L
H-H_	0	-1.70655000	-0.75075800	-8.03852800	L
H-H_	0	-3.24512900	0.18635000	-8.06268800	L
H-H_	0	-3.07560200	-2.31860400	-6.93806400	L
H-H_	0	-4.57039300	-1.72794400	-7.78307700	L
H-H_	0	-2.38838800	-3.57403700	-8.78589500	L
H-H_	0	-4.17645400	-3.61004100	-9.12511500	L
C-C_3	0	-4.02381300	3.42228000	-2.65318500	L
O-O_3	0	-2.77090800	3.78836400	-3.17657600	L
C-C_3	0	-2.10871400	4.53615700	-2.18965000	L
C-C_3	0	-2.68112000	4.19092100	-0.83378700	L
C-C_3	0	-4.03036000	3.61909700	-1.14769900	L
H-H_	0	-4.81322400	4.03742600	-3.13286700	L
H-H_	0	-4.22048200	2.35828100	-2.89868300	L
H-H_	0	-1.02462600	4.30759200	-2.22602900	L
H-H_	0	-2.24835000	5.61437700	-2.39451700	L
H-H_	0	-2.04705000	3.42959500	-0.33249500	L
H-H_	0	-2.76274400	5.08834800	-0.18452100	L
H-H_	0	-4.17218400	2.65404700	-0.61959600	L
H-H_	0	-4.83676000	4.32285500	-0.85122100	L
C-C_3	0	0.38581500	-7.69170500	-3.99222400	L
O-O_3	0	0.90098800	-7.05762600	-2.85250000	L
C-C_3	0	-0.20765400	-6.63292600	-2.09907600	L

C-C_3	0	-1.42071200	-6.51583500	-3.00795000	L
C-C_3	0	-0.95024200	-7.06752200	-4.32309200	L
H-H_	0	1.09303700	-7.56917700	-4.83724100	L
H-H_	0	0.26457900	-8.77452900	-3.78121200	L
H-H_	0	-0.39833000	-7.36673700	-1.28763100	L
H-H_	0	0.02276600	-5.65062900	-1.63633000	L
H-H_	0	-2.26834800	-7.11415300	-2.61003200	L
H-H_	0	-1.73878400	-5.45578700	-3.11654900	L
H-H_	0	-1.65655700	-7.81572500	-4.73645200	L
H-H_	0	-0.81563300	-6.24586000	-5.05693300	L
C-C_3	0	4.77922100	1.42027600	-3.68036600	L
O-O_3	0	4.57325000	2.30810400	-2.60905000	L
C-C_3	0	5.28333400	1.79489900	-1.50355100	L
C-C_3	0	5.82855900	0.41794700	-1.84419100	L
C-C_3	0	5.11854200	0.06690100	-3.11416300	L
H-H_	0	3.87224000	1.36404000	-4.31368100	L
H-H_	0	5.61887500	1.78752800	-4.30575800	L
H-H_	0	6.10889900	2.48847700	-1.23932800	L
H-H_	0	4.59811300	1.72593700	-0.63327900	L
H-H_	0	6.92307800	0.47694600	-2.02464100	L
H-H_	0	5.62314400	-0.32226700	-1.04397100	L
H-H_	0	5.75572500	-0.52067900	-3.80597700	L
H-H_	0	4.18440000	-0.48921200	-2.88948400	L
C-C_3	0	-7.27881100	10.72237900	-2.08374600	L
O-O_3	0	-6.42471000	10.93662600	-0.99037300	L
C-C_3	0	-7.24094600	11.38424700	0.06436400	L
C-C_3	0	-8.66221300	10.89567700	-0.16511600	L
C-C_3	0	-8.63431200	10.31304500	-1.55152200	L
H-H_	0	-7.36142300	11.66063400	-2.67608800	L
H-H_	0	-6.86214600	9.92909000	-2.73612900	L
H-H_	0	-6.84754700	10.99625700	1.02719400	L
H-H_	0	-7.21562600	12.49542700	0.09927200	L
H-H_	0	-8.94470000	10.12100200	0.58014500	L
H-H_	0	-9.38000500	11.74302900	-0.10608100	L
H-H_	0	-8.70640600	9.20447000	-1.50288800	L
H-H_	0	-9.45552900	10.71432300	-2.18402400	L
C-C_3	0	-12.64272500	-4.63694500	-2.03075200	L
O-O_3	0	-11.49614000	-3.85374600	-2.27312800	L

C-C_3	0	-10.93351000	-4.28459000	-3.48936300	L
C-C_3	0	-11.94278900	-5.13549300	-4.22488200	L
C-C_3	0	-12.77491700	-5.68965600	-3.11319600	L
H-H_	0	-12.56619700	-5.11512100	-1.03133400	L
H-H_	0	-13.53482000	-3.97403200	-2.03709600	L
H-H_	0	-10.64524900	-3.40651700	-4.10313500	L
H-H_	0	-10.02128400	-4.88037100	-3.27118600	L
H-H_	0	-12.56900300	-4.49608500	-4.88559100	L
H-H_	0	-11.45752700	-5.93718700	-4.82103100	L
H-H_	0	-13.83242700	-5.83633400	-3.42297600	L
H-H_	0	-12.34514900	-6.65402800	-2.76153700	L
C-C_3	0	-9.78606000	7.75040100	3.79840400	L
O-O_3	0	-9.94457200	9.03256400	3.23431000	L
C-C_3	0	-8.99737800	9.88625500	3.83412800	L
C-C_3	0	-8.35818900	9.18076800	5.01030600	L
C-C_3	0	-8.55832700	7.73473300	4.68605100	L
H-H_	0	-10.68924100	7.50929300	4.39981200	L
H-H_	0	-9.69090600	6.99557000	2.98919100	L
H-H_	0	-8.22051000	10.14697200	3.08348600	L
H-H_	0	-9.49404100	10.82247000	4.16647200	L
H-H_	0	-7.28328900	9.44131500	5.10845000	L
H-H_	0	-8.89786200	9.43801800	5.94853600	L
H-H_	0	-7.68224700	7.34549900	4.12287300	L
H-H_	0	-8.71842000	7.12418600	5.60113500	L
C-C_3	0	14.96438100	1.71654500	0.92166600	L
O-O_3	0	14.40512800	1.29425500	-0.30174600	L
C-C_3	0	13.09671100	0.85986700	-0.02855000	L
C-C_3	0	12.61809100	1.54589000	1.22703000	L
C-C_3	0	13.88847100	1.74226000	1.99512900	L
H-H_	0	15.78471100	1.02536700	1.21207400	L
H-H_	0	15.39315400	2.73250700	0.78812500	L
H-H_	0	12.43022900	1.10017200	-0.88176600	L
H-H_	0	13.10726600	-0.24146300	0.11968200	L
H-H_	0	12.16598200	2.52866300	0.97504000	L
H-H_	0	11.88168000	0.92880200	1.78177700	L
H-H_	0	13.88436900	2.70440200	2.55003800	L
H-H_	0	14.03894800	0.89956100	2.70563000	L
C-C_3	0	9.69735300	-6.74700700	-5.23506700	L

O-O_3	0	10.06440000	-8.02264800	-4.76209200	L
C-C_3	0	10.69767500	-7.84681400	-3.51378700	L
C-C_3	0	10.64420500	-6.38387400	-3.12377300	L
C-C_3	0	10.42994900	-5.69605300	-4.43358000	L
H-H_	0	9.94311000	-6.65748200	-6.31443900	L
H-H_	0	8.60184600	-6.61814900	-5.10591700	L
H-H_	0	10.19698800	-8.47283200	-2.74478500	L
H-H_	0	11.75603600	-8.17495000	-3.59995400	L
H-H_	0	9.77440900	-6.20599700	-2.45438100	L
H-H_	0	11.58225900	-6.04939800	-2.63166700	L
H-H_	0	9.83806000	-4.76231400	-4.32691400	L
H-H_	0	11.40970100	-5.46916600	-4.90927600	L
C-C_3	0	-0.57359800	11.21033000	-6.78740900	L
O-O_3	0	0.13761800	12.19401900	-6.08005700	L
C-C_3	0	1.45634800	11.72012700	-5.96274000	L
C-C_3	0	1.74035500	10.75883000	-7.10195200	L
C-C_3	0	0.38999800	10.49431800	-7.71178500	L
H-H_	0	-1.02582300	10.49585800	-6.06612300	L
H-H_	0	-1.39179400	11.68524500	-7.36926100	L
H-H_	0	2.16304100	12.57654400	-5.99158600	L
H-H_	0	1.57306400	11.20095200	-4.98797700	L
H-H_	0	2.42511600	11.21922300	-7.84757700	L
H-H_	0	2.19413500	9.82390100	-6.71216600	L
H-H_	0	0.33729800	10.93236100	-8.73274900	L
H-H_	0	0.16876500	9.40648000	-7.76342500	L
C-C_3	0	2.79424900	-5.23576000	4.30599300	L
O-O_3	0	2.65256800	-5.88691200	3.07062700	L
C-C_3	0	2.30761500	-7.22578800	3.33991500	L
C-C_3	0	2.37421000	-7.48329300	4.83398200	L
C-C_3	0	3.06530800	-6.27008700	5.36846400	L
H-H_	0	3.62925100	-4.50948700	4.25552900	L
H-H_	0	1.85669900	-4.68841700	4.52997900	L
H-H_	0	1.28535300	-7.42840900	2.95596400	L
H-H_	0	3.02161700	-7.89035300	2.80958100	L
H-H_	0	1.35036500	-7.55385000	5.25832800	L
H-H_	0	2.93858800	-8.41080000	5.06694300	L
H-H_	0	2.66251100	-5.95987700	6.35537400	L
H-H_	0	4.15874500	-6.45557800	5.43872800	L

C-C_3	0	0.25026900	-10.46528800	2.31442900	L
O-O_3	0	1.30344300	-10.21142000	3.21262300	L
C-C_3	0	1.16374100	-11.09641900	4.29972900	L
C-C_3	0	-0.03509700	-11.99777200	4.07278800	L
C-C_3	0	-0.81219500	-11.28060800	3.01437500	L
H-H_	0	-0.16744400	-9.50459200	1.94769600	L
H-H_	0	0.64792800	-11.03276200	1.44664700	L
H-H_	0	2.08983300	-11.70034900	4.40914500	L
H-H_	0	1.02191600	-10.50178100	5.22715000	L
H-H_	0	0.30054600	-12.98771700	3.69110600	L
H-H_	0	-0.63280100	-12.13225700	5.00057000	L
H-H_	0	-1.31841500	-11.98513400	2.31959400	L
H-H_	0	-1.56478600	-10.60853100	3.48089700	L
C-C_3	0	-7.32121700	-4.68949800	5.00373700	L
O-O_3	0	-6.25901200	-4.39936000	5.87403500	L
C-C_3	0	-6.83367800	-3.93439000	7.07284900	L
C-C_3	0	-8.31924400	-4.25570000	7.08919900	L
C-C_3	0	-8.53129100	-5.04493400	5.83269900	L
H-H_	0	-7.54063900	-3.79336800	4.38832700	L
H-H_	0	-7.03991000	-5.52476800	4.33105700	L
H-H_	0	-6.32465700	-4.42221700	7.92769500	L
H-H_	0	-6.67748000	-2.83892400	7.15374200	L
H-H_	0	-8.60539700	-4.84145000	7.98889500	L
H-H_	0	-8.91229200	-3.31764300	7.05855000	L
H-H_	0	-8.53320900	-6.13114800	6.06206500	L
H-H_	0	-9.47535600	-4.76803400	5.31853800	L
C-C_3	0	-4.20708600	7.23252200	-4.05114400	L
O-O_3	0	-3.74555200	7.67917900	-2.79931600	L
C-C_3	0	-4.80710900	7.56200900	-1.88119700	L
C-C_3	0	-6.01502800	6.95977100	-2.57084000	L
C-C_3	0	-5.71331000	7.14647800	-4.01997900	L
H-H_	0	-3.87727900	7.93457400	-4.84393600	L
H-H_	0	-3.77179800	6.23337500	-4.26478000	L
H-H_	0	-4.49235100	6.92703200	-1.02653500	L
H-H_	0	-5.05328000	8.57284000	-1.49453900	L
H-H_	0	-6.09140800	5.87817700	-2.33448900	L
H-H_	0	-6.95970300	7.46838900	-2.28685100	L
H-H_	0	-6.08870600	6.30050500	-4.62900800	L

H-H_	0	-6.15364400	8.10031700	-4.37885200	L
C-C_3	0	-7.33318900	-11.85179000	1.22516100	L
O-O_3	0	-6.27648400	-12.51480400	0.56900600	L
C-C_3	0	-6.51199000	-12.43120800	-0.81799100	L
C-C_3	0	-7.89089400	-11.85261500	-1.06162200	L
C-C_3	0	-8.17561200	-11.11340500	0.20672300	L
H-H_	0	-7.95524500	-12.60536300	1.75500100	L
H-H_	0	-6.92284600	-11.14437300	1.97482100	L
H-H_	0	-5.73781900	-11.77796100	-1.27281600	L
H-H_	0	-6.42918900	-13.44082400	-1.27354100	L
H-H_	0	-7.91403300	-11.18169400	-1.94666500	L
H-H_	0	-8.62864900	-12.67469300	-1.19457300	L
H-H_	0	-7.83365000	-10.05929200	0.12025200	L
H-H_	0	-9.25618900	-11.13750900	0.46645300	L
C-C_3	0	7.09553300	2.73354100	-8.46304500	L
O-O_3	0	5.71809300	2.58724600	-8.23291100	L
C-C_3	0	5.47830300	1.20621300	-8.11306300	L
C-C_3	0	6.60429800	0.43873100	-8.78883100	L
C-C_3	0	7.58594200	1.50300400	-9.19263300	L
H-H_	0	7.28051600	3.64529100	-9.06950600	L
H-H_	0	7.62196200	2.84220100	-7.48952400	L
H-H_	0	5.41429700	0.94398100	-7.03558400	L
H-H_	0	4.50778300	0.96068200	-8.59250900	L
H-H_	0	7.07393800	-0.27670800	-8.08057800	L
H-H_	0	6.23294200	-0.11694800	-9.67806300	L
H-H_	0	8.62526900	1.24149600	-8.89728800	L
H-H_	0	7.53784600	1.66984000	-10.29138300	L
C-C_3	0	9.44818800	6.05295000	-6.59918700	L
O-O_3	0	9.62143600	6.73258100	-5.37584600	L
C-C_3	0	8.80987100	7.88018400	-5.41584100	L
C-C_3	0	7.70506900	7.65678500	-6.41930600	L
C-C_3	0	8.31094400	6.68428300	-7.38413200	L
H-H_	0	10.39258000	6.09757400	-7.18310400	L
H-H_	0	9.21998700	4.98631000	-6.39007900	L
H-H_	0	8.38080200	8.07139400	-4.41119400	L
H-H_	0	9.42798400	8.75550200	-5.71330100	L
H-H_	0	6.83168200	7.19802900	-5.91289800	L
H-H_	0	7.39876400	8.60153400	-6.91779800	L

H-H_	0	7.56956100	5.92485600	-7.71467200	L
H-H_	0	8.71053300	7.22449700	-8.27062500	L
C-C_3	0	-1.96946000	-12.78439300	-0.89109100	L
O-O_3	0	-2.02210000	-11.38475000	-0.99894200	L
C-C_3	0	-0.76115300	-10.94987700	-1.45454100	L
C-C_3	0	0.21523100	-12.11346500	-1.45432000	L
C-C_3	0	-0.52575400	-13.19993100	-0.73691300	L
H-H_	0	-2.56591000	-13.11523800	-0.01437600	L
H-H_	0	-2.40317000	-13.23649600	-1.81032100	L
H-H_	0	-0.86389900	-10.52853100	-2.47638500	L
H-H_	0	-0.39540500	-10.14744500	-0.78029000	L
H-H_	0	0.43533200	-12.43006500	-2.49722500	L
H-H_	0	1.16320500	-11.85398500	-0.93497100	L
H-H_	0	-0.33328200	-14.19936100	-1.18379400	L
H-H_	0	-0.24968300	-13.20608800	0.33952900	L
C-C_3	0	-4.08371200	10.87092000	5.95164300	L
O-O_3	0	-4.63212900	10.35443500	4.75979200	L
C-C_3	0	-3.56617500	9.87829400	3.97299600	L
C-C_3	0	-2.26874400	10.43155800	4.51311000	L
C-C_3	0	-2.58368000	10.65049000	5.95777300	L
H-H_	0	-4.30484500	11.95877600	6.00406100	L
H-H_	0	-4.55347900	10.37249300	6.82633600	L
H-H_	0	-3.55234800	8.76743900	4.01872900	L
H-H_	0	-3.71045900	10.19022000	2.91844500	L
H-H_	0	-1.42755300	9.72184100	4.37494800	L
H-H_	0	-2.03247800	11.40090800	4.02035200	L
H-H_	0	-2.33902300	9.73672000	6.54178100	L
H-H_	0	-2.03411900	11.52357000	6.37207100	L
C-C_3	0	-1.69490200	13.46663400	-0.35130600	L
O-O_3	0	-2.92566200	14.09780800	-0.62495000	L
C-C_3	0	-3.94505600	13.15577600	-0.39639500	L
C-C_3	0	-3.40533500	12.05507500	0.48591300	L
C-C_3	0	-1.93988700	12.06563300	0.17998300	L
H-H_	0	-1.08451300	13.42925500	-1.27870000	L
H-H_	0	-1.14560800	14.06638900	0.40585900	L
H-H_	0	-4.81220700	13.65131400	0.08909300	L
H-H_	0	-4.27658200	12.73612500	-1.37040400	L
H-H_	0	-3.57317000	12.31150400	1.55523300	L

H-H_	0	-3.87290300	11.07507900	0.25074100	L
H-H_	0	-1.33355700	11.86017600	1.08885500	L
H-H_	0	-1.70694200	11.31232600	-0.60402900	L
C-C_3	0	-12.14434900	-5.23669400	3.68098900	L
O-O_3	0	-11.65514800	-3.92618100	3.80092900	L
C-C_3	0	-12.57582500	-3.09160300	3.14134800	L
C-C_3	0	-13.39042100	-3.91658100	2.15800300	L
C-C_3	0	-12.93881300	-5.33060600	2.39771300	L
H-H_	0	-11.29688900	-5.95496800	3.66818200	L
H-H_	0	-12.79292700	-5.46661400	4.55533600	L
H-H_	0	-13.24198400	-2.61500900	3.89283800	L
H-H_	0	-12.02383700	-2.29232000	2.60617200	L
H-H_	0	-14.47694100	-3.81021900	2.37027200	L
H-H_	0	-13.18762000	-3.60753300	1.10918400	L
H-H_	0	-13.79907600	-6.02680600	2.50290400	L
H-H_	0	-12.28129900	-5.67087500	1.56788400	L
C-C_3	0	1.80592300	7.60836900	8.81860000	L
O-O_3	0	1.59300600	8.75917900	8.03285700	L
C-C_3	0	0.20440600	8.90281900	7.85680000	L
C-C_3	0	-0.50982700	8.09307700	8.91328700	L
C-C_3	0	0.47092700	7.00582100	9.22012800	L
H-H_	0	2.40960500	6.87457300	8.24374200	L
H-H_	0	2.37783300	7.89713600	9.72638100	L
H-H_	0	-0.07449900	9.97495100	7.93560800	L
H-H_	0	-0.07254500	8.53761900	6.84411300	L
H-H_	0	-0.68248000	8.71744700	9.81763500	L
H-H_	0	-1.47455200	7.68763400	8.54157100	L
H-H_	0	0.44996200	6.72640300	10.29596200	L
H-H_	0	0.24846000	6.11186100	8.59922900	L
C-C_3	0	5.88661800	-9.18527900	-8.41935000	L
O-O_3	0	6.75087900	-8.98049900	-7.33171000	L
C-C_3	0	6.37045100	-7.76199100	-6.73935600	L
C-C_3	0	4.94516200	-7.42398800	-7.14353500	L
C-C_3	0	4.57100300	-8.50463200	-8.11844400	L
H-H_	0	6.34322500	-8.75496900	-9.33783000	L
H-H_	0	5.73470600	-10.27397600	-8.57465000	L
H-H_	0	6.44362900	-7.85829900	-5.63666800	L
H-H_	0	7.06495700	-6.96034700	-7.07239000	L

H-H_	0	4.26411600	-7.42541300	-6.26524700	L
H-H_	0	4.90805400	-6.42526200	-7.62753100	L
H-H_	0	3.87506200	-9.22678900	-7.64346000	L
H-H_	0	4.10692800	-8.09061500	-9.03873500	L
C-C_3	0	2.44241300	-8.68762600	-0.19055300	L
O-O_3	0	1.97461800	-7.41439500	0.19000900	L
C-C_3	0	2.91532100	-6.46293900	-0.25413700	L
C-C_3	0	3.95919100	-7.15046500	-1.10582700	L
C-C_3	0	3.87405900	-8.57092800	-0.65588200	L
H-H_	0	2.37531000	-9.38524200	0.66810100	L
H-H_	0	1.80769000	-9.07332200	-1.01658900	L
H-H_	0	2.40029900	-5.66685700	-0.83121800	L
H-H_	0	3.40208800	-5.99518100	0.62584000	L
H-H_	0	3.67718400	-7.07438300	-2.17703700	L
H-H_	0	4.97432300	-6.73030800	-0.94713600	L
H-H_	0	4.10585900	-9.28144800	-1.47547800	L
H-H_	0	4.56195800	-8.73829900	0.19892200	L
C-C_3	0	-9.21700300	9.19793700	-5.08399000	L
O-O_3	0	-9.43424600	9.06385200	-6.46936000	L
C-C_3	0	-10.25464800	7.93369200	-6.66563400	L
C-C_3	0	-10.66532600	7.36529200	-5.32213500	L
C-C_3	0	-9.63601500	7.92106500	-4.39037400	L
H-H_	0	-8.14671600	9.41429600	-4.88986700	L
H-H_	0	-9.82267500	10.05019700	-4.70634700	L
H-H_	0	-11.15104100	8.22000800	-7.25585600	L
H-H_	0	-9.68462800	7.17153300	-7.23951500	L
H-H_	0	-11.67376400	7.74195500	-5.04095700	L
H-H_	0	-10.66780000	6.25489400	-5.32659900	L
H-H_	0	-10.05506800	8.11547000	-3.37978100	L
H-H_	0	-8.77141600	7.22569100	-4.31556600	L
C-C_3	0	-5.71948600	8.24425600	1.72049200	L
O-O_3	0	-4.79848100	7.19307000	1.86581200	L
C-C_3	0	-5.55500700	6.01263300	1.92902300	L
C-C_3	0	-6.83506300	6.21561200	1.14683300	L
C-C_3	0	-6.97911100	7.70981200	1.06664400	L
H-H_	0	-5.95646500	8.66554600	2.72156800	L
H-H_	0	-5.26807000	9.04390500	1.09702800	L
H-H_	0	-4.96988000	5.17512000	1.50038300	L

H-H_	0	-5.78262500	5.78208800	2.99085800	L
H-H_	0	-6.73364000	5.78097300	0.12977400	L
H-H_	0	-7.70191900	5.75212500	1.66405400	L
H-H_	0	-7.04538800	8.04380700	0.01191700	L
H-H_	0	-7.88215200	8.05510900	1.60873700	L
C-C_3	0	-3.67251100	-5.13773200	-5.98792800	L
O-O_3	0	-2.72570400	-5.61471900	-6.90884400	L
C-C_3	0	-3.28339800	-6.77358900	-7.47481400	L
C-C_3	0	-4.23422300	-7.39720300	-6.46997200	L
C-C_3	0	-4.45018500	-6.31706600	-5.44505300	L
H-H_	0	-3.15892600	-4.59793200	-5.16515800	L
H-H_	0	-4.36027800	-4.43292400	-6.50295400	L
H-H_	0	-3.82424300	-6.50172700	-8.40760700	L
H-H_	0	-2.47594500	-7.48752900	-7.73576900	L
H-H_	0	-5.19018300	-7.69104100	-6.95566300	L
H-H_	0	-3.77075000	-8.28876000	-5.99874800	L
H-H_	0	-5.52694200	-6.07014200	-5.33704600	L
H-H_	0	-4.04154900	-6.62739800	-4.45917200	L
C-C_3	0	-3.98619900	5.71733500	7.72921200	L
O-O_3	0	-5.30979300	5.23482200	7.70198300	L
C-C_3	0	-6.04889400	6.06095600	6.83169200	L
C-C_3	0	-5.10937900	7.00899200	6.11765000	L
C-C_3	0	-3.92945400	7.05900100	7.03185100	L
H-H_	0	-3.64157500	5.81113000	8.78094800	L
H-H_	0	-3.32901600	4.98986800	7.20640100	L
H-H_	0	-6.59981700	5.43644500	6.09981200	L
H-H_	0	-6.78823000	6.63843800	7.42794500	L
H-H_	0	-4.81100000	6.58673600	5.13354100	L
H-H_	0	-5.56188900	8.01190300	5.97719600	L
H-H_	0	-2.98085000	7.19821600	6.47469800	L
H-H_	0	-4.05659700	7.87909900	7.77285100	L
C-C_3	0	8.42806800	-0.81989700	5.34059500	L
O-O_3	0	9.59223200	-1.28425600	4.70353200	L
C-C_3	0	9.34765600	-1.27402500	3.31568800	L
C-C_3	0	7.88818700	-0.95861500	3.04964800	L
C-C_3	0	7.25575600	-1.00120800	4.40714300	L
H-H_	0	8.26654300	-1.38538000	6.28100400	L
H-H_	0	8.55184800	0.25475700	5.59273100	L

H-H_	0	9.99766000	-0.51619700	2.83162400	L
H-H_	0	9.59982400	-2.27158200	2.89902900	L
H-H_	0	7.78650600	0.05624900	2.61137100	L
H-H_	0	7.43188100	-1.70242700	2.36271700	L
H-H_	0	6.49678600	-0.19946500	4.53904900	L
H-H_	0	6.78773200	-1.99342600	4.57642500	L
C-C_3	0	4.58472000	-11.38701400	-5.06395700	L
O-O_3	0	4.59500700	-9.99963900	-4.83124900	L
C-C_3	0	3.26868300	-9.63245400	-4.55386100	L
C-C_3	0	2.57058500	-10.81230800	-3.92081600	L
C-C_3	0	3.34404700	-11.99542300	-4.42922700	L
H-H_	0	4.59144200	-11.57202300	-6.15918500	L
H-H_	0	5.50031500	-11.83945200	-4.62854900	L
H-H_	0	3.25730900	-8.75863500	-3.87123600	L
H-H_	0	2.75686800	-9.35063500	-5.49929800	L
H-H_	0	2.64335900	-10.74873000	-2.81522700	L
H-H_	0	1.50321200	-10.85963100	-4.21873600	L
H-H_	0	3.61352700	-12.67874500	-3.59427300	L
H-H_	0	2.75387000	-12.55368400	-5.18876000	L
C-C_3	0	-6.95402400	-0.03399100	11.13202800	L
O-O_3	0	-5.86920300	-0.90778600	10.91150100	L
C-C_3	0	-6.38909300	-2.10449800	10.38108400	L
C-C_3	0	-7.80908600	-1.86395200	9.92370300	L
C-C_3	0	-8.25229100	-0.74871000	10.81350700	L
H-H_	0	-6.94885800	0.31047800	12.18800000	L
H-H_	0	-6.84085900	0.85192900	10.47131400	L
H-H_	0	-5.76276700	-2.44438500	9.53240200	L
H-H_	0	-6.37120500	-2.88709600	11.17059400	L
H-H_	0	-7.81252100	-1.52256500	8.86579800	L
H-H_	0	-8.44401200	-2.76932100	10.03736600	L
H-H_	0	-8.98159300	-0.08181900	10.30778200	L
H-H_	0	-8.70030200	-1.16094300	11.74472600	L
C-C_3	0	8.20986500	7.85251100	0.86936800	L
O-O_3	0	9.46653300	8.47441500	0.81444100	L
C-C_3	0	10.04767300	8.05701500	-0.39372800	L
C-C_3	0	8.93976000	7.79784600	-1.39553500	L
C-C_3	0	7.70076300	7.68887500	-0.54762900	L
H-H_	0	8.31384900	6.86336400	1.36401200	L

H-H_	0	7.51107900	8.47122100	1.46725500	L
H-H_	0	10.73548500	8.84449100	-0.76764500	L
H-H_	0	10.63034600	7.12778700	-0.21509700	L
H-H_	0	8.85218500	8.65387200	-2.09884100	L
H-H_	0	9.12785800	6.86216200	-1.96627700	L
H-H_	0	6.99478600	8.50911600	-0.79481200	L
H-H_	0	7.19765700	6.70836300	-0.68110100	L
C-C_3	0	-7.20078000	-0.10365600	-10.95953200	L
O-O_3	0	-8.59470500	-0.17914400	-10.76389000	L
C-C_3	0	-8.82732500	-0.30471300	-9.37917700	L
C-C_3	0	-7.52502500	-0.11592600	-8.63095900	L
C-C_3	0	-6.49437100	-0.43806300	-9.66445800	L
H-H_	0	-6.89499600	-0.80951700	-11.76095200	L
H-H_	0	-6.93813700	0.92921000	-11.27640400	L
H-H_	0	-9.57433100	0.45077800	-9.05386900	L
H-H_	0	-9.23750300	-1.31684000	-9.17545200	L
H-H_	0	-7.42474000	0.94495300	-8.31789200	L
H-H_	0	-7.45087100	-0.78317200	-7.74594200	L
H-H_	0	-5.56868000	0.16008300	-9.53004300	L
H-H_	0	-6.24852300	-1.52116500	-9.63676500	L
C-C_3	0	1.84430300	-10.53525900	-10.12600700	L
O-O_3	0	1.03202700	-11.00743200	-9.07992500	L
C-C_3	0	1.87698300	-11.71968500	-8.21216800	L
C-C_3	0	3.03585800	-12.27744500	-9.01347700	L
C-C_3	0	3.04297900	-11.45350600	-10.27264400	L
H-H_	0	1.25683200	-10.51028300	-11.06825300	L
H-H_	0	2.17907900	-9.50098500	-9.89505500	L
H-H_	0	2.24652100	-11.03748700	-7.41662300	L
H-H_	0	1.30858000	-12.54306700	-7.73096200	L
H-H_	0	3.99258900	-12.17853600	-8.45767500	L
H-H_	0	2.85639600	-13.34845900	-9.25447100	L
H-H_	0	3.98154000	-10.86461000	-10.36477400	L
H-H_	0	2.92759300	-12.10690800	-11.16538300	L
C-C_3	0	-2.99641100	-6.41972000	4.08651200	L
O-O_3	0	-3.74364100	-7.09107500	5.07558700	L
C-C_3	0	-2.96692100	-8.17290700	5.52797900	L
C-C_3	0	-1.51799800	-7.89486900	5.20961800	L
C-C_3	0	-1.60106200	-7.01475900	4.00354500	L

H-H_	0	-3.51405400	-6.50554500	3.10807100	L
H-H_	0	-2.93572300	-5.34410300	4.35418900	L
H-H_	0	-3.10326400	-8.30380900	6.62170600	L
H-H_	0	-3.30078000	-9.10124700	5.01542400	L
H-H_	0	-1.04489400	-7.34219500	6.04778100	L
H-H_	0	-0.94955300	-8.82685000	5.00864600	L
H-H_	0	-0.81464000	-6.23123500	4.02466400	L
H-H_	0	-1.49647200	-7.62485100	3.08141200	L
C-C_3	0	-13.99557300	5.54466300	-4.24967000	L
O-O_3	0	-13.30892500	5.21622500	-5.43096000	L
C-C_3	0	-12.92352700	3.87223900	-5.29541300	L
C-C_3	0	-13.93604500	3.16637900	-4.41546400	L
C-C_3	0	-14.63957100	4.28663700	-3.69667000	L
H-H_	0	-13.27738000	5.96759500	-3.51339900	L
H-H_	0	-14.76768600	6.31220800	-4.46948800	L
H-H_	0	-12.87600300	3.39442900	-6.29671800	L
H-H_	0	-11.91513900	3.82622100	-4.83176000	L
H-H_	0	-14.65729300	2.59385500	-5.03918500	L
H-H_	0	-13.43328700	2.47784600	-3.70272800	L
H-H_	0	-15.72737200	4.27266800	-3.92859100	L
H-H_	0	-14.49478400	4.21682900	-2.59648500	L
C-C_3	0	-7.53799200	-4.51061200	1.33618200	L
O-O_3	0	-7.48835900	-3.93483800	0.05493800	L
C-C_3	0	-8.78201000	-3.46430000	-0.21154200	L
C-C_3	0	-9.76868600	-4.35589800	0.50806200	L
C-C_3	0	-8.95645900	-4.97622100	1.61198100	L
H-H_	0	-6.83021600	-5.36533000	1.39030200	L
H-H_	0	-7.22778000	-3.74972100	2.08216300	L
H-H_	0	-8.87213900	-2.41911500	0.15235100	L
H-H_	0	-8.96686900	-3.47817300	-1.30436600	L
H-H_	0	-10.62163600	-3.76528600	0.90321200	L
H-H_	0	-10.14266900	-5.14516500	-0.17938700	L
H-H_	0	-9.29648400	-4.62451600	2.60866400	L
H-H_	0	-9.02532100	-6.08497800	1.56968600	L
C-C_3	0	5.36314700	-9.76299300	3.90192100	L
O-O_3	0	6.23504900	-10.86224900	3.77056700	L
C-C_3	0	7.45858900	-10.38381000	3.26298900	L
C-C_3	0	7.47028900	-8.87213400	3.31883500	L

C-C_3	0	6.01705800	-8.52028400	3.33704100	L
H-H_	0	4.41042700	-9.97259300	3.37315900	L
H-H_	0	5.13844500	-9.61334100	4.97795100	L
H-H_	0	8.29874700	-10.79869300	3.85939900	L
H-H_	0	7.56939700	-10.72616100	2.21139600	L
H-H_	0	7.95865100	-8.52711400	4.25441900	L
H-H_	0	7.99369100	-8.43390700	2.44413000	L
H-H_	0	5.81494900	-7.63374000	3.97287100	L
H-H_	0	5.65677600	-8.33091600	2.30323700	L
C-C_3	0	-8.81759000	3.85849000	8.46549700	L
O-O_3	0	-8.89314500	2.57102300	9.02799000	L
C-C_3	0	-9.95259800	1.89230200	8.39059900	L
C-C_3	0	-10.61254600	2.80763800	7.37638600	L
C-C_3	0	-10.12745400	4.16552900	7.77815300	L
H-H_	0	-8.60957700	4.60610100	9.25992800	L
H-H_	0	-7.98629400	3.88169700	7.72833400	L
H-H_	0	-9.56276100	0.97994600	7.89417500	L
H-H_	0	-10.69418200	1.58533800	9.15899800	L
H-H_	0	-10.25724100	2.55926800	6.35292700	L
H-H_	0	-11.72156800	2.73802000	7.41365600	L
H-H_	0	-9.98988600	4.84060800	6.90766600	L
H-H_	0	-10.83681700	4.62305400	8.50280200	L
C-C_3	0	7.09171000	2.92792800	4.41339500	L
O-O_3	0	7.88029500	4.09446400	4.38621500	L
C-C_3	0	7.01040900	5.19538900	4.25811000	L
C-C_3	0	5.57687400	4.71738200	4.32983000	L
C-C_3	0	5.68890300	3.26999300	3.97207900	L
H-H_	0	7.53158400	2.16252300	3.74416100	L
H-H_	0	7.07961700	2.52256600	5.44679000	L
H-H_	0	7.21572600	5.92461700	5.06552900	L
H-H_	0	7.19512400	5.69171700	3.28216600	L
H-H_	0	5.19590100	4.82621300	5.36813200	L
H-H_	0	4.92072900	5.27171300	3.62712300	L
H-H_	0	4.92373300	2.65167600	4.48718200	L
H-H_	0	5.60380200	3.14082500	2.87118800	L
C-C_3	0	7.79154000	-9.18654000	-0.53561900	L
O-O_3	0	7.52213700	-8.18981700	-1.49050300	L
C-C_3	0	7.04908500	-8.83478200	-2.65145900	L

C-C_3	0	7.10922200	-10.33960500	-2.46576300	L
C-C_3	0	7.95639100	-10.50936400	-1.24431700	L
H-H_	0	8.71335500	-8.93243100	0.02622300	L
H-H_	0	6.94437300	-9.23995100	0.17995900	L
H-H_	0	6.00780400	-8.51525500	-2.85706500	L
H-H_	0	7.68061500	-8.53312000	-3.51197000	L
H-H_	0	6.08923300	-10.74157100	-2.28065300	L
H-H_	0	7.55834900	-10.84596200	-3.34766700	L
H-H_	0	7.61593200	-11.36067200	-0.61605800	L
H-H_	0	9.02055600	-10.65240200	-1.53557900	L
C-C_3	0	-5.08361800	-5.83752200	10.44070800	L
O-O_3	0	-4.31761100	-6.79550100	9.75509800	L
C-C_3	0	-5.14509100	-7.29362700	8.73512100	L
C-C_3	0	-6.58366900	-7.22172600	9.20740400	L
C-C_3	0	-6.54791400	-6.22255900	10.33437900	L
H-H_	0	-4.76704100	-5.79836500	11.50449200	L
H-H_	0	-4.91163100	-4.83845200	9.98484000	L
H-H_	0	-5.00745300	-6.67860700	7.81840200	L
H-H_	0	-4.86408100	-8.34282500	8.50397600	L
H-H_	0	-7.25654300	-6.89198300	8.38734900	L
H-H_	0	-6.91634400	-8.21490400	9.58162600	L
H-H_	0	-7.17756500	-5.33384200	10.11158200	L
H-H_	0	-6.89535900	-6.69320900	11.28031700	L
C-C_R	0	-0.76739100	-1.44123500	3.36403800	H
C-C_R	0	-0.19897100	-2.61554500	5.15863600	H
C-C_3	0	0.55757900	-0.23213100	5.20184500	H
H-H_	0	1.11528300	0.22192500	4.38351900	H
N-N_R	0	-0.77251000	-3.48556600	4.40078100	H
N-N_R	0	-1.16525900	-2.75955400	3.29346400	H
N-N_R	0	-0.19904800	-1.34771400	4.62447000	H
C-C_3	0	0.59522900	-2.85170400	6.39573200	H
H-H_	0	0.81100700	-3.91214400	6.51789100	H
H-H_	0	0.06803100	-2.48174900	7.28634800	H
C-C_3	0	1.56205600	-0.76430100	6.27096200	H
O-O_3	0	1.80540200	-2.14974900	6.21904500	H
C-C_R	0	-2.04160000	-3.33043500	2.32720700	H
C-C_R	0	-3.35933300	-2.85634100	2.26646900	H
C-C_R	0	-1.54538300	-4.26080300	1.41097600	H

C-C_R	0	-4.14964400	-3.26451800	1.19820000	H
C-C_R	0	-2.38798700	-4.65860500	0.37368400	H
C-C_R	0	-3.67217500	-4.14454000	0.22652400	H
H-H_	0	-5.15386800	-2.86288700	1.10967200	H
H-H_	0	-2.01573500	-5.35857600	-0.36614000	H
H-H_	0	2.52460100	-0.27652200	6.12268300	H
C-C_R	0	-0.83629600	-0.45802900	2.38541800	H
O-O_R	0	-0.64740900	0.85315100	2.78874300	H
C-C_R	0	-1.14969600	-0.74132900	1.02733000	H
C-C_R	0	-1.28728800	0.16523600	0.02453900	H
H-H_	0	-1.24574600	-1.79014700	0.76948900	H
H-H_	0	-1.15688400	1.22123200	0.24751600	H
C-C_R	0	-1.65708600	-0.17781700	-1.33951200	H
C-C_R	0	-1.62222300	0.81847300	-2.32683400	H
C-C_R	0	-2.08216000	-1.46333800	-1.73862100	H
C-C_R	0	-1.98556200	0.56289200	-3.64184600	H
H-H_	0	-1.27587200	1.80709900	-2.04177800	H
C-C_R	0	-2.44117100	-1.71956400	-3.05298400	H
H-H_	0	-2.16517000	-2.26035900	-1.00555200	H
C-C_R	0	-2.38933500	-0.71060400	-4.02032200	H
H-H_	0	-1.92460400	1.35923700	-4.37586900	H
H-H_	0	-2.77355600	-2.71799700	-3.32742600	H
H-H_	0	-2.66634100	-0.91008500	-5.04926600	H
C-C_3	0	-4.51413800	-4.50389300	-0.96716100	H
H-H_	0	-5.32121300	-5.18819600	-0.69555800	H
H-H_	0	-4.96883200	-3.60690400	-1.39189700	H
H-H_	0	-3.91699500	-4.98719900	-1.74327800	H
C-C_3	0	-3.88339800	-1.88411100	3.28584600	H
H-H_	0	-3.51095700	-2.11986100	4.28538600	H
H-H_	0	-3.56732200	-0.86400500	3.04367000	H
H-H_	0	-4.97274300	-1.91536500	3.30632600	H
C-C_3	0	-0.12320500	-4.75227600	1.46061600	H
H-H_	0	0.30745500	-4.64282700	2.45511200	H
H-H_	0	-0.07685200	-5.80514500	1.17313500	H
H-H_	0	0.49999700	-4.18941500	0.75546200	H
H-H_	0	1.18845400	-0.49500000	7.27067600	H
C-C_3	0	-0.36811300	0.82129900	5.82313200	H
H-H_	0	-0.99388200	1.24320600	5.03168100	H

H-H_	0	-1.02270800	0.31594800	6.54371600	H
C-C_R	0	0.43062500	1.88942900	6.53799900	H
C-C_R	0	0.04767500	2.31182200	7.81143300	H
C-C_R	0	1.59381300	2.42909900	5.97590300	H
C-C_R	0	0.81940900	3.21767900	8.53137800	H
H-H_	0	-0.86740400	1.91688500	8.24150100	H
C-C_R	0	2.36927900	3.33243000	6.69656700	H
H-H_	0	1.90797600	2.13658000	4.97491700	H
C-C_R	0	1.99397600	3.71917800	7.98020800	H
H-H_	0	0.50809700	3.52168500	9.52571300	H
H-H_	0	3.27337500	3.73346500	6.25386700	H
H-H_	0	2.61062300	4.41037600	8.54420700	H
H-H_	0	0.27925800	1.11257300	2.63961500	H
C-C_R	0	1.86634400	2.26011700	0.40382700	H
C-C_R	0	1.61632500	2.01916500	-0.90687400	H
C-C_R	0	1.67960400	0.69875400	-1.48225200	H
C-C_R	0	2.20270700	-0.35252900	-0.64293200	H
C-C_R	0	2.53942600	-0.17601300	0.65713800	H
C-C_R	0	2.18779400	1.11252500	1.28034000	H
H-H_	0	1.29987400	2.83914500	-1.53900400	H
H-H_	0	2.41878600	-1.30174600	-1.11249800	H
C-C_R	0	1.31643000	0.44026700	-2.79970600	H
C-C_R	0	1.11900700	-0.92231200	-3.23925300	H
C-C_R	0	1.18147400	1.48903800	-3.79224100	H
C-C_R	0	0.90999800	-1.26676500	-4.52701200	H
H-H_	0	1.10087800	-1.68846700	-2.47698600	H
C-C_R	0	0.99522800	1.23315000	-5.10694300	H
H-H_	0	1.31884800	2.51217000	-3.46724400	H
C-C_R	0	0.86173000	-0.18792700	-5.55192900	H
O-O_R	0	2.12590500	1.23292200	2.52109400	H
O-O_R	0	0.73123000	-0.45932100	-6.74065100	H
C-C_3	0	3.23913500	-1.25780300	1.47637500	H
C-C_3	0	4.50144500	-0.66198400	2.12088800	H
C-C_3	0	2.33219500	-1.80882700	2.58459900	H
C-C_3	0	3.64649200	-2.44703200	0.60367900	H
H-H_	0	5.20899500	-0.31238300	1.36348400	H
H-H_	0	4.25087900	0.17228000	2.77930100	H
H-H_	0	4.98978400	-1.43514400	2.71811700	H

H-H_	0	1.44247400	-2.28394200	2.15923500	H
H-H_	0	2.88105400	-2.55107400	3.17066000	H
H-H_	0	2.02286200	-1.01156900	3.25326400	H
H-H_	0	4.11625900	-3.19757100	1.23951200	H
H-H_	0	2.77786600	-2.92482200	0.13838200	H
H-H_	0	4.35463700	-2.16537900	-0.18005900	H
C-C_3	0	1.72613600	3.65016000	1.01849900	H
C-C_3	0	0.49636700	3.69480600	1.94101600	H
H-H_	0	0.68853200	3.19825600	2.89317700	H
H-H_	0	0.23583500	4.73498900	2.14550700	H
H-H_	0	-0.36592900	3.21737200	1.46847900	H
C-C_3	0	2.98197100	4.01006200	1.82380800	H
H-H_	0	2.84079500	4.99155200	2.28402400	H
H-H_	0	3.15189500	3.28179200	2.61575300	H
H-H_	0	3.86493900	4.05604000	1.17806500	H
C-C_3	0	1.54177200	4.72447300	-0.05734800	H
H-H_	0	2.34914800	4.70363400	-0.79638800	H
H-H_	0	0.58520100	4.62373400	-0.57545800	H
H-H_	0	1.54604600	5.70345800	0.42841200	H
C-C_3	0	0.95982100	2.32745700	-6.17165400	H
C-C_3	0	0.85281400	-2.72187500	-4.98057200	H
C-C_3	0	2.12217500	2.13254000	-7.16210200	H
H-H_	0	2.05428700	1.17018900	-7.66625600	H
H-H_	0	2.09079000	2.92328100	-7.91824300	H
H-H_	0	3.08194100	2.19864100	-6.64133100	H
C-C_3	0	1.10757900	3.71289100	-5.54270800	H
H-H_	0	0.30089500	3.92495600	-4.83249500	H
H-H_	0	2.06818400	3.81408700	-5.02801200	H
H-H_	0	1.06688600	4.47172600	-6.32817300	H
C-C_3	0	-0.37890000	2.29644100	-6.92810500	H
H-H_	0	-0.37928500	3.07442200	-7.69829200	H
H-H_	0	-0.54756900	1.33303700	-7.40436400	H
H-H_	0	-1.20159300	2.50513100	-6.24050000	H
C-C_3	0	2.12248900	-2.98746800	-5.81213900	H
H-H_	0	2.22175700	-4.05467300	-6.02577800	H
H-H_	0	2.08957800	-2.44205500	-6.75347500	H
H-H_	0	3.00647900	-2.67235400	-5.25280500	H
C-C_3	0	-0.39681300	-3.03217900	-5.81943000	H

H-H_	0	-0.44911500	-2.40473300	-6.70644900	H
H-H_	0	-0.36679800	-4.07916400	-6.13116100	H
H-H_	0	-1.29849900	-2.88655600	-5.22129400	H
C-C_3	0	0.84922700	-3.67369100	-3.78311100	H
H-H_	0	1.74133600	-3.56703900	-3.16044400	H
H-H_	0	-0.03463800	-3.51441100	-3.15514600	H
H-H_	0	0.83612500	-4.69825500	-4.15758200	H

TS5$_{explicit}$ (ONIOM(M06-2X/6-31G(d, p):UFF))

0 1 0 1 0 1

C-C_3	0	4.56079100	5.92183700	-9.07680200	L
O-O_3	0	5.27625200	7.02019300	-8.56171700	L
C-C_3	0	4.40395600	7.74352600	-7.72319700	L
C-C_3	0	3.02318500	7.12200000	-7.76785100	L
C-C_3	0	3.28517200	5.74428800	-8.28441300	L
H-H_	0	4.31953800	6.11782600	-10.14423300	L
H-H_	0	5.18556200	5.00697800	-9.01747500	L
H-H_	0	4.79378900	7.71183000	-6.68440200	L
H-H_	0	4.36547800	8.80350400	-8.05248600	L
H-H_	0	2.54786800	7.10157700	-6.76509300	L
H-H_	0	2.37845200	7.68127200	-8.48094900	L
H-H_	0	3.45868500	5.04732100	-7.43616600	L
H-H_	0	2.45090200	5.37081800	-8.91664400	L
C-C_3	0	2.24028400	-4.52752800	10.92993200	L
O-O_3	0	1.83697800	-4.06459600	9.66479000	L
C-C_3	0	2.34094500	-2.75750100	9.51225200	L
C-C_3	0	3.02070300	-2.31001000	10.79320100	L
C-C_3	0	2.59911400	-3.34032000	11.79368700	L
H-H_	0	1.41773200	-5.10991100	11.39630000	L
H-H_	0	3.12202000	-5.19395400	10.80734900	L
H-H_	0	3.05582200	-2.73372800	8.66530300	L
H-H_	0	1.49951400	-2.07647400	9.27542800	L
H-H_	0	4.12514400	-2.32626600	10.66817400	L
H-H_	0	2.69907400	-1.29001100	11.09631400	L
H-H_	0	3.41510900	-3.58228800	12.50877000	L
H-H_	0	1.70037000	-2.98870100	12.34712200	L
C-C_3	0	-8.43955900	4.16505600	4.21212200	L
O-O_3	0	-9.71927400	4.75189800	4.27786800	L

C-C_3	0	-10.56944800	4.01749300	3.42982100	L
C-C_3	0	-9.88235100	2.72831700	3.04272300	L
C-C_3	0	-8.43631500	3.08097700	3.15767900	L
H-H_	0	-7.68791700	4.94199600	3.97144400	L
H-H_	0	-8.19035900	3.72657400	5.20179300	L
H-H_	0	-11.52677700	3.80790600	3.95130600	L
H-H_	0	-10.78264300	4.62235800	2.52177400	L
H-H_	0	-10.14038300	1.93468300	3.77441700	L
H-H_	0	-10.14651200	2.39757200	2.01774500	L
H-H_	0	-7.82009000	2.20815800	3.45780000	L
H-H_	0	-8.06978100	3.49285300	2.19228500	L
C-C_3	0	-5.80017300	-0.77802000	-4.80616700	L
O-O_3	0	-5.17773200	-2.04018700	-4.87985800	L
C-C_3	0	-6.08397000	-2.99174200	-4.37580000	L
C-C_3	0	-7.18437800	-2.27894000	-3.62552600	L
C-C_3	0	-7.21459000	-0.93968300	-4.28583500	L
H-H_	0	-5.21714200	-0.12973300	-4.11798300	L
H-H_	0	-5.80452800	-0.30751600	-5.81141900	L
H-H_	0	-6.51545700	-3.56029000	-5.22491200	L
H-H_	0	-5.55202200	-3.70449000	-3.71386200	L
H-H_	0	-8.15686000	-2.80697700	-3.71800500	L
H-H_	0	-6.90808700	-2.16883400	-2.55378900	L
H-H_	0	-7.93180300	-0.94994500	-5.13409200	L
H-H_	0	-7.49076500	-0.13740200	-3.57099500	L
C-C_3	0	-13.57697300	2.09315300	2.16870200	L
O-O_3	0	-14.21304500	2.97975700	3.06143900	L
C-C_3	0	-14.34078500	2.31923200	4.29721900	L
C-C_3	0	-13.36173900	1.16969300	4.34370300	L
C-C_3	0	-13.17493600	0.82462600	2.89943700	L
H-H_	0	-14.26409700	1.85498300	1.32857900	L
H-H_	0	-12.67854500	2.59199800	1.75085100	L
H-H_	0	-14.14001100	3.03138200	5.12583600	L
H-H_	0	-15.38073100	1.93837400	4.39836400	L
H-H_	0	-12.39965600	1.50917500	4.78584500	L
H-H_	0	-13.76378900	0.31152000	4.92366000	L
H-H_	0	-12.12256500	0.54119800	2.68647700	L
H-H_	0	-13.85320800	-0.00945000	2.61385200	L
C-C_3	0	-4.23045200	-9.86280200	-3.28284000	L

O-O_3	0	-3.94590900	-11.18807600	-3.66770500	L
C-C_3	0	-5.04684600	-11.66909200	-4.40234200	L
C-C_3	0	-5.96441100	-10.51527500	-4.74327400	L
C-C_3	0	-5.64497700	-9.50818900	-3.68615400	L
H-H_	0	-4.10736000	-9.76001500	-2.18674800	L
H-H_	0	-3.51090000	-9.18113800	-3.78031700	L
H-H_	0	-4.69023600	-12.16832500	-5.32802500	L
H-H_	0	-5.59088300	-12.41724300	-3.78636800	L
H-H_	0	-5.70892600	-10.10871000	-5.74668400	L
H-H_	0	-7.03454000	-10.81539400	-4.71702500	L
H-H_	0	-5.72007900	-8.47092500	-4.07110900	L
H-H_	0	-6.32769000	-9.63487400	-2.82047800	L
C-C_3	0	4.49217800	-3.90146500	-2.66526400	L
O-O_3	0	4.17405400	-4.79901500	-3.70183600	L
C-C_3	0	5.34433100	-5.51454100	-4.02253600	L
C-C_3	0	6.47867200	-5.07801900	-3.11549700	L
C-C_3	0	5.99327300	-3.78367900	-2.54667300	L
H-H_	0	4.04101600	-2.91117000	-2.87632900	L
H-H_	0	4.06973600	-4.28601300	-1.71481600	L
H-H_	0	5.15397000	-6.60055600	-3.91402700	L
H-H_	0	5.60646900	-5.31619800	-5.08255000	L
H-H_	0	6.62967800	-5.81805500	-2.30062800	L
H-H_	0	7.42501600	-4.94350100	-3.67794100	L
H-H_	0	6.31257500	-3.64671700	-1.49280000	L
H-H_	0	6.36371300	-2.93477300	-3.15963500	L
C-C_3	0	-12.23056800	-0.69800800	7.36937500	L
O-O_3	0	-12.61888100	-1.71632700	6.47705400	L
C-C_3	0	-12.34145900	-2.95410800	7.09220400	L
C-C_3	0	-11.84624400	-2.71944800	8.50530100	L
C-C_3	0	-11.38511000	-1.29804000	8.46954400	L
H-H_	0	-11.66040200	0.08246700	6.82427700	L
H-H_	0	-13.14194900	-0.23115200	7.80204600	L
H-H_	0	-13.25789500	-3.58172100	7.09976300	L
H-H_	0	-11.56179300	-3.47975600	6.50244400	L
H-H_	0	-12.68677500	-2.82761500	9.22612500	L
H-H_	0	-11.02281300	-3.41400600	8.77925800	L
H-H_	0	-11.54122200	-0.78444800	9.44284900	L
H-H_	0	-10.31288600	-1.25501700	8.18463300	L

C-C_3	0	-13.07699100	0.24909700	-6.07018100	L
O-O_3	0	-13.08352800	-1.07084500	-6.56695500	L
C-C_3	0	-11.75010000	-1.42888200	-6.84111100	L
C-C_3	0	-10.82725300	-0.47541700	-6.11953400	L
C-C_3	0	-11.65265200	0.76676400	-6.00774600	L
H-H_	0	-13.69557500	0.89770600	-6.72667300	L
H-H_	0	-13.52475100	0.25088200	-5.05330500	L
H-H_	0	-11.56547400	-2.47237900	-6.51099400	L
H-H_	0	-11.57720400	-1.36776500	-7.93785600	L
H-H_	0	-10.58946000	-0.86862000	-5.10854100	L
H-H_	0	-9.88842300	-0.29961200	-6.68553200	L
H-H_	0	-11.45329800	1.31022700	-5.05902400	L
H-H_	0	-11.44930900	1.43555000	-6.87336900	L
C-C_3	0	12.17505100	-3.70901500	2.10884900	L
O-O_3	0	11.52426300	-4.32128000	3.19389600	L
C-C_3	0	12.16732500	-3.88356900	4.36912600	L
C-C_3	0	13.15529200	-2.77811400	4.03616900	L
C-C_3	0	12.86748500	-2.46100600	2.60120600	L
H-H_	0	12.92024000	-4.41651200	1.68472900	L
H-H_	0	11.43343700	-3.45728200	1.32347400	L
H-H_	0	11.40120900	-3.50570900	5.07840000	L
H-H_	0	12.69159800	-4.74091600	4.84297600	L
H-H_	0	13.00973200	-1.88737400	4.68561100	L
H-H_	0	14.19775300	-3.15157600	4.14281200	L
H-H_	0	12.17606100	-1.59577900	2.53564500	L
H-H_	0	13.79521300	-2.24833500	2.02690000	L
C-C_3	0	-6.88737700	8.57357100	-9.34961700	L
O-O_3	0	-6.25919800	9.60555800	-8.62357000	L
C-C_3	0	-5.68292200	9.03046400	-7.47287400	L
C-C_3	0	-6.09581700	7.57662200	-7.37584300	L
C-C_3	0	-6.46983400	7.23490500	-8.78066100	L
H-H_	0	-7.98935800	8.69368700	-9.26380200	L
H-H_	0	-6.60544900	8.64373500	-10.42170500	L
H-H_	0	-4.57736800	9.10607100	-7.54889600	L
H-H_	0	-6.00890900	9.59032100	-6.57144500	L
H-H_	0	-5.27183600	6.93509800	-7.00228700	L
H-H_	0	-6.98211600	7.47653400	-6.71298900	L
H-H_	0	-5.58112100	6.84962200	-9.32819700	L

H-H_	0	-7.29384300	6.48936700	-8.81928700	L
C-C_3	0	-0.18339700	5.54623200	5.45050800	L
O-O_3	0	1.03210100	5.90342900	6.05304100	L
C-C_3	0	1.75245500	6.65339500	5.10590200	L
C-C_3	0	0.80767400	7.14433600	4.02351200	L
C-C_3	0	-0.53117500	6.60195100	4.43022000	L
H-H_	0	-0.06978500	4.55686700	4.95803300	L
H-H_	0	-0.97604000	5.47047000	6.22294100	L
H-H_	0	2.22312800	7.51829000	5.61707400	L
H-H_	0	2.55343200	6.01937200	4.66773600	L
H-H_	0	0.78835700	8.25330800	3.98153100	L
H-H_	0	1.11148800	6.74133300	3.03425300	L
H-H_	0	-1.13578300	7.40256500	4.90442400	L
H-H_	0	-1.08698800	6.17206400	3.57205600	L
C-C_3	0	0.40662900	-7.04035000	8.75107400	L
O-O_3	0	-0.57443100	-8.03239000	8.56556100	L
C-C_3	0	0.09042700	-9.25746600	8.35527200	L
C-C_3	0	1.59140800	-9.04197600	8.40385100	L
C-C_3	0	1.72959700	-7.70352600	9.05799800	L
H-H_	0	0.49053800	-6.43553600	7.82250500	L
H-H_	0	0.10595500	-6.37238900	9.58456500	L
H-H_	0	-0.21396300	-9.96962800	9.15208100	L
H-H_	0	-0.21060800	-9.67986600	7.37315600	L
H-H_	0	2.10588500	-9.83729000	8.98585400	L
H-H_	0	2.00407200	-9.00812800	7.37328100	L
H-H_	0	1.84593100	-7.82695100	10.15746000	L
H-H_	0	2.58702200	-7.12607800	8.65057500	L
C-C_3	0	5.24061800	8.11537500	5.53117400	L
O-O_3	0	4.61017600	8.93257400	6.48561500	L
C-C_3	0	5.47790700	10.01180800	6.75210500	L
C-C_3	0	6.65909100	9.97181400	5.79633500	L
C-C_3	0	6.26215200	8.93952000	4.78782500	L
H-H_	0	5.73276400	7.26884800	6.05259300	L
H-H_	0	4.49208200	7.70686800	4.82276700	L
H-H_	0	4.91831000	10.96266900	6.62360400	L
H-H_	0	5.82855500	9.95390600	7.80478700	L
H-H_	0	6.83558900	10.95964100	5.31728100	L
H-H_	0	7.57742600	9.64946500	6.33417800	L

H-H_	0	5.78419600	9.43109400	3.91359600	L
H-H_	0	7.12304800	8.32279800	4.45404800	L
C-C_3	0	7.70926100	-3.57772500	-6.77129500	L
O-O_3	0	7.23148600	-2.53878500	-5.95661000	L
C-C_3	0	5.84898600	-2.43275200	-6.19887200	L
C-C_3	0	5.50224300	-3.13015300	-7.50282000	L
C-C_3	0	6.81076800	-3.68909500	-7.98128400	L
H-H_	0	8.75353800	-3.36138100	-7.08094000	L
H-H_	0	7.70095200	-4.52882600	-6.19644600	L
H-H_	0	5.29640200	-2.89129600	-5.35706700	L
H-H_	0	5.56429500	-1.36286400	-6.24667600	L
H-H_	0	4.76843200	-3.94601900	-7.32207900	L
H-H_	0	5.08588000	-2.41248600	-8.24169600	L
H-H_	0	6.71195700	-4.74363800	-8.31437400	L
H-H_	0	7.21187500	-3.06602100	-8.81105600	L
C-C_3	0	8.72793200	11.58233500	0.14801200	L
O-O_3	0	9.19613200	11.23503700	1.43166200	L
C-C_3	0	8.13930500	11.43077000	2.34299100	L
C-C_3	0	7.01272200	12.17132200	1.65609000	L
C-C_3	0	7.24206200	11.86610600	0.21003300	L
H-H_	0	9.27081800	12.48826400	-0.19802300	L
H-H_	0	8.93417900	10.75489200	-0.56380700	L
H-H_	0	7.77995100	10.44133100	2.69358600	L
H-H_	0	8.50271800	12.00393300	3.22219700	L
H-H_	0	6.01645500	11.82197700	2.00259900	L
H-H_	0	7.11195300	13.26495900	1.83392600	L
H-H_	0	6.67397100	10.95909700	-0.08478700	L
H-H_	0	6.95438100	12.71918600	-0.44204200	L
C-C_3	0	8.31103800	4.67138100	7.90740500	L
O-O_3	0	9.18748100	5.51858600	7.20148800	L
C-C_3	0	8.80854300	6.84958300	7.47258200	L
C-C_3	0	7.69257200	6.86325500	8.49709200	L
C-C_3	0	7.13521200	5.47841800	8.41254300	L
H-H_	0	7.96347700	3.85307500	7.24368300	L
H-H_	0	8.85797400	4.22163600	8.76438700	L
H-H_	0	9.68588600	7.42005300	7.84492500	L
H-H_	0	8.45934300	7.32154800	6.53078200	L
H-H_	0	8.11114600	7.04285000	9.51217000	L

H-H_	0	6.92452800	7.63205800	8.26612700	L
H-H_	0	6.78010600	5.11874800	9.40218500	L
H-H_	0	6.30337500	5.44216100	7.67517700	L
C-C_3	0	4.15064900	-12.65448700	-0.54790300	L
O-O_3	0	3.10763500	-13.57720200	-0.33023900	L
C-C_3	0	2.81387400	-13.57265300	1.04848300	L
C-C_3	0	3.57318300	-12.44960800	1.72007500	L
C-C_3	0	4.71237100	-12.20798900	0.78430500	L
H-H_	0	4.94432900	-13.12179000	-1.16875500	L
H-H_	0	3.74326800	-11.77888200	-1.09393700	L
H-H_	0	1.72204900	-13.44533400	1.20191600	L
H-H_	0	3.12036600	-14.54856900	1.48336300	L
H-H_	0	2.93490800	-11.54260600	1.77420400	L
H-H_	0	3.92135300	-12.73264400	2.73693900	L
H-H_	0	5.01610700	-11.13984800	0.77107500	L
H-H_	0	5.57965900	-12.84503000	1.06643800	L
C-C_3	0	-4.24866500	-9.59327900	1.57804900	L
O-O_3	0	-2.93666400	-9.37127700	1.12599000	L
C-C_3	0	-2.96201700	-8.23813000	0.28970800	L
C-C_3	0	-4.38388800	-7.72808100	0.15935500	L
C-C_3	0	-5.20772900	-8.86750000	0.66402400	L
H-H_	0	-4.46371600	-10.68251100	1.58497800	L
H-H_	0	-4.33873700	-9.20348500	2.61385400	L
H-H_	0	-2.30029100	-7.45347500	0.70943200	L
H-H_	0	-2.57509200	-8.52505400	-0.70865000	L
H-H_	0	-4.53316500	-6.84095800	0.81162400	L
H-H_	0	-4.63565700	-7.47424200	-0.89325800	L
H-H_	0	-6.11548100	-8.51696700	1.19925900	L
H-H_	0	-5.49885800	-9.52778500	-0.17972500	L
C-C_3	0	1.84561300	12.50060300	5.41100700	L
O-O_3	0	2.92412900	12.93968900	4.61736500	L
C-C_3	0	3.38517600	11.83440100	3.87329800	L
C-C_3	0	2.46408900	10.65355400	4.09936500	L
C-C_3	0	1.79893500	10.98964500	5.39369400	L
H-H_	0	1.96761500	12.87250700	6.45052100	L
H-H_	0	0.90041700	12.91223300	4.99399100	L
H-H_	0	3.42078100	12.09793200	2.79693600	L
H-H_	0	4.41452200	11.58246500	4.20512400	L

H-H_	0	1.70707400	10.60207000	3.28604100	L
H-H_	0	3.02154400	9.69503400	4.16067100	L
H-H_	0	0.75933100	10.60544200	5.43895600	L
H-H_	0	2.38936900	10.58296600	6.24240400	L
C-C_3	0	5.17273500	-3.69809200	6.74872100	L
O-O_3	0	5.68299100	-2.55845100	7.39156600	L
C-C_3	0	6.45527300	-3.01931300	8.47129600	L
C-C_3	0	5.94672300	-4.38371200	8.89671500	L
C-C_3	0	5.07909800	-4.82869200	7.75297300	L
H-H_	0	4.17103500	-3.46635800	6.33257600	L
H-H_	0	5.84766900	-3.98321700	5.91260600	L
H-H_	0	7.51866000	-3.08601100	8.15695300	L
H-H_	0	6.38242600	-2.29774200	9.31254400	L
H-H_	0	6.79021400	-5.08886500	9.06029300	L
H-H_	0	5.34872100	-4.30541200	9.83002800	L
H-H_	0	5.45106700	-5.77449300	7.31012300	L
H-H_	0	4.02996700	-4.96788300	8.09149800	L
C-C_3	0	2.18921500	12.81868400	-0.08529500	L
O-O_3	0	1.61848000	12.08946700	-1.14240900	L
C-C_3	0	2.59210700	12.00400300	-2.15733900	L
C-C_3	0	3.93915900	12.45718800	-1.61760000	L
C-C_3	0	3.68922600	12.65992300	-0.15413600	L
H-H_	0	1.91155800	13.89077700	-0.18979600	L
H-H_	0	1.79973800	12.44523500	0.88506100	L
H-H_	0	2.65686000	10.95228100	-2.50531800	L
H-H_	0	2.28610300	12.64218800	-3.01370300	L
H-H_	0	4.72864200	11.69523900	-1.79063400	L
H-H_	0	4.24049900	13.41681200	-2.09255600	L
H-H_	0	3.99891500	11.75581600	0.41154500	L
H-H_	0	4.21907700	13.55380500	0.24059600	L
C-C_3	0	-1.17091400	-12.24095700	-5.47798900	L
O-O_3	0	-1.92433300	-12.98998500	-6.40426400	L
C-C_3	0	-2.26383200	-12.12879000	-7.46280100	L
C-C_3	0	-2.20485900	-10.70321400	-6.96731800	L
C-C_3	0	-1.20298400	-10.76977400	-5.85655100	L
H-H_	0	-1.60373400	-12.38299100	-4.46502400	L
H-H_	0	-0.12552900	-12.61581100	-5.46376500	L
H-H_	0	-1.54417200	-12.27579000	-8.29727800	L

H-H_	0	-3.28472500	-12.36883400	-7.82864900	L
H-H_	0	-1.89089800	-10.00043200	-7.76920700	L
H-H_	0	-3.19725700	-10.40369700	-6.56768900	L
H-H_	0	-0.20498900	-10.44126900	-6.21969300	L
H-H_	0	-1.51311500	-10.13850500	-4.99877300	L
C-C_3	0	12.72174300	2.04988000	5.52633000	L
O-O_3	0	12.55416700	3.25915500	4.82460900	L
C-C_3	0	11.24848200	3.26866700	4.29325600	L
C-C_3	0	10.52979400	1.99047700	4.67622500	L
C-C_3	0	11.63328000	1.08366300	5.11769500	L
H-H_	0	13.72241700	1.62214900	5.30403300	L
H-H_	0	12.65700800	2.25473200	6.61731300	L
H-H_	0	10.69695200	4.14926600	4.68339300	L
H-H_	0	11.30967100	3.35447500	3.18702700	L
H-H_	0	9.83990900	2.18472300	5.52693900	L
H-H_	0	9.96634000	1.56406900	3.81910400	L
H-H_	0	11.32101200	0.43147400	5.96209000	L
H-H_	0	11.97608600	0.45699700	4.26707900	L
C-C_3	0	5.04909000	-3.88042900	-11.67840800	L
O-O_3	0	3.76705400	-4.39541800	-11.41857400	L
C-C_3	0	3.95621700	-5.47512900	-10.54046000	L
C-C_3	0	5.30895800	-6.09426700	-10.83175500	L
C-C_3	0	6.05706100	-5.00882300	-11.55918400	L
H-H_	0	5.07433500	-3.44219700	-12.69863900	L
H-H_	0	5.28004300	-3.07777900	-10.94298200	L
H-H_	0	3.91471000	-5.10568400	-9.49230100	L
H-H_	0	3.14759300	-6.22216200	-10.68471100	L
H-H_	0	5.82821400	-6.38721600	-9.89510000	L
H-H_	0	5.19075500	-6.98747800	-11.48407600	L
H-H_	0	6.95256900	-4.68132900	-10.98729300	L
H-H_	0	6.36908500	-5.36119800	-12.56697900	L
C-C_3	0	7.28675800	4.38545800	-3.84604400	L
O-O_3	0	7.04014700	4.14195200	-5.20817900	L
C-C_3	0	5.70193500	4.49307000	-5.46910500	L
C-C_3	0	5.11374700	5.21918100	-4.27367000	L
C-C_3	0	6.26298100	5.36485200	-3.32259900	L
H-H_	0	7.22247300	3.42751600	-3.28837400	L
H-H_	0	8.30986100	4.79720000	-3.71982200	L

H-H_	0	5.67301100	5.14652200	-6.36378700	L
H-H_	0	5.11400500	3.57928900	-5.69686600	L
H-H_	0	4.70888700	6.21146200	-4.56549100	L
H-H_	0	4.30611600	4.61028100	-3.81383500	L
H-H_	0	6.66932100	6.39905200	-3.36440900	L
H-H_	0	5.96604400	5.11964700	-2.27952900	L
C-C_3	0	-2.73752800	3.69453400	3.23475300	L
O-O_3	0	-3.51717600	4.31551800	4.22621400	L
C-C_3	0	-4.48539400	3.38061800	4.64381600	L
C-C_3	0	-4.35501600	2.10786800	3.82760600	L
C-C_3	0	-3.50700400	2.52509200	2.67110100	L
H-H_	0	-2.49693300	4.42416600	2.43355200	L
H-H_	0	-1.79420600	3.33440700	3.69667000	L
H-H_	0	-4.35090100	3.16863500	5.72388000	L
H-H_	0	-5.49345100	3.81830500	4.49571300	L
H-H_	0	-3.83354600	1.32481600	4.42063900	L
H-H_	0	-5.34123400	1.73472400	3.48399500	L
H-H_	0	-2.83923700	1.70920900	2.32438300	L
H-H_	0	-4.15189600	2.86438400	1.83425300	L
C-C_3	0	1.92763800	6.58008800	-3.61615300	L
O-O_3	0	0.60038600	6.25680100	-3.29126500	L
C-C_3	0	-0.20852600	7.30577100	-3.77037300	L
C-C_3	0	0.66574400	8.46041600	-4.22587600	L
C-C_3	0	2.03506900	8.07608700	-3.76237500	L
H-H_	0	2.60987900	6.20809400	-2.82663700	L
H-H_	0	2.19725200	6.09504900	-4.57560400	L
H-H_	0	-0.83913500	6.93298300	-4.60341800	L
H-H_	0	-0.87879100	7.64408500	-2.95686200	L
H-H_	0	0.64700600	8.53287600	-5.33319100	L
H-H_	0	0.34735400	9.42613100	-3.77776600	L
H-H_	0	2.81548700	8.36239400	-4.49935700	L
H-H_	0	2.24904500	8.53754700	-2.77465900	L
C-C_3	0	-10.27532800	3.69576500	-0.65252300	L
O-O_3	0	-9.45121900	4.77545000	-0.28682900	L
C-C_3	0	-9.06611200	5.40058900	-1.48362100	L
C-C_3	0	-10.16783700	5.20002000	-2.50012100	L
C-C_3	0	-10.87967700	3.96649100	-2.01899700	L
H-H_	0	-9.66915600	2.76494700	-0.67389900	L

H-H_	0	-11.08124400	3.57854600	0.10074800	L
H-H_	0	-8.89475700	6.48327100	-1.30376600	L
H-H_	0	-8.11881300	4.94478200	-1.84536800	L
H-H_	0	-10.85850700	6.07105800	-2.48729800	L
H-H_	0	-9.75487500	5.06358300	-3.52156600	L
H-H_	0	-11.97246800	4.15170800	-1.93794400	L
H-H_	0	-10.70235700	3.10923000	-2.70431500	L
C-C_3	0	-7.40369200	-0.64036700	4.59673600	L
O-O_3	0	-7.73204900	-1.05029600	3.28927800	L
C-C_3	0	-9.13381200	-1.04649500	3.18132300	L
C-C_3	0	-9.73548500	-1.01818500	4.56696700	L
C-C_3	0	-8.66793500	-0.36340200	5.38440700	L
H-H_	0	-6.81771300	-1.44365300	5.08773300	L
H-H_	0	-6.77209600	0.26712400	4.55247600	L
H-H_	0	-9.44692400	-0.14924500	2.60767100	L
H-H_	0	-9.47004500	-1.94912900	2.63307700	L
H-H_	0	-10.68787300	-0.44867300	4.59022700	L
H-H_	0	-9.90679900	-2.05481200	4.92698200	L
H-H_	0	-8.84651200	0.73189200	5.44545000	L
H-H_	0	-8.61400900	-0.78799100	6.40882300	L
C-C_3	0	-1.61274300	-1.60160600	11.88714900	L
O-O_3	0	-0.43675200	-1.53586200	11.11379600	L
C-C_3	0	-0.37118000	-0.24887100	10.54180900	L
C-C_3	0	-1.46250600	0.61883600	11.13189300	L
C-C_3	0	-2.46617300	-0.38410300	11.60283300	L
H-H_	0	-2.16900300	-2.53265400	11.64866400	L
H-H_	0	-1.33267200	-1.62389700	12.96269800	L
H-H_	0	0.62714600	0.19893000	10.73291700	L
H-H_	0	-0.51049100	-0.33926300	9.44341200	L
H-H_	0	-1.06435300	1.19358300	11.99730600	L
H-H_	0	-1.89253000	1.31657200	10.38318800	L
H-H_	0	-3.00911600	-0.03509300	12.50779200	L
H-H_	0	-3.18991800	-0.60852100	10.78915100	L
C-C_3	0	10.28381800	8.56970800	4.20555700	L
O-O_3	0	11.35541700	9.18850900	3.53327300	L
C-C_3	0	12.17333000	8.17104500	3.00050400	L
C-C_3	0	11.54328000	6.81750100	3.26387000	L
C-C_3	0	10.58345600	7.09702800	4.37463700	L

H-H_	0	10.13071800	9.05157900	5.19455700	L
H-H_	0	9.36099700	8.70045800	3.60317400	L
H-H_	0	12.30632400	8.33285900	1.90993800	L
H-H_	0	13.17090400	8.22289800	3.48759900	L
H-H_	0	10.98901400	6.46728500	2.36667300	L
H-H_	0	12.30220800	6.05899500	3.55437300	L
H-H_	0	9.66517500	6.47800100	4.29044600	L
H-H_	0	11.07595600	6.91821800	5.35588900	L
C-C_3	0	8.69745900	1.66696200	0.48933900	L
O-O_3	0	7.75212800	2.49515300	1.11305000	L
C-C_3	0	8.19897000	3.81236000	0.91487700	L
C-C_3	0	9.02369800	3.86442200	-0.35785300	L
C-C_3	0	9.29089900	2.42038000	-0.68267500	L
H-H_	0	9.49178800	1.40154800	1.22094800	L
H-H_	0	8.20540800	0.73298200	0.14793500	L
H-H_	0	7.32202400	4.48984700	0.84021000	L
H-H_	0	8.82025600	4.12300400	1.78353000	L
H-H_	0	8.44859700	4.34668500	-1.17563900	L
H-H_	0	9.97047400	4.42272800	-0.19308500	L
H-H_	0	8.77504500	2.13750500	-1.62540100	L
H-H_	0	10.37757500	2.21750600	-0.77753200	L
C-C_3	0	-8.89800900	-7.58438100	-3.64130900	L
O-O_3	0	-7.88239300	-6.65443300	-3.93917500	L
C-C_3	0	-7.32395500	-6.22309700	-2.71962500	L
C-C_3	0	-7.87596100	-7.06411900	-1.58901700	L
C-C_3	0	-9.16106500	-7.57982700	-2.15092800	L
H-H_	0	-9.82096300	-7.32333700	-4.19961000	L
H-H_	0	-8.56549700	-8.59473800	-3.96361600	L
H-H_	0	-6.21803300	-6.30964300	-2.76335400	L
H-H_	0	-7.58913900	-5.15613400	-2.56123100	L
H-H_	0	-7.18905600	-7.90891800	-1.37181000	L
H-H_	0	-8.03747400	-6.46918200	-0.66825100	L
H-H_	0	-9.40320900	-8.59511000	-1.76939800	L
H-H_	0	-9.98984100	-6.87708500	-1.91236500	L
C-C_3	0	1.95263300	0.27147300	-10.90799600	L
O-O_3	0	3.04509700	-0.54724200	-10.55533600	L
C-C_3	0	2.56077800	-1.49980600	-9.64356900	L
C-C_3	0	1.09034200	-1.70831100	-9.91531600	L

C-C_3	0	0.65791800	-0.36755400	-10.42752000	L
H-H_	0	2.08198900	1.27696400	-10.45162200	L
H-H_	0	1.93181600	0.38673400	-12.01271300	L
H-H_	0	3.11322100	-2.45320000	-9.75714700	L
H-H_	0	2.71879800	-1.12120200	-8.61176100	L
H-H_	0	0.95899400	-2.47921100	-10.70666700	L
H-H_	0	0.53479000	-2.01002600	-9.00254600	L
H-H_	0	-0.08218500	-0.46934900	-11.25097500	L
H-H_	0	0.21787800	0.23498800	-9.60347600	L
C-C_3	0	-3.38786500	-3.07861400	7.32406600	L
O-O_3	0	-3.44888000	-4.43840400	6.98030800	L
C-C_3	0	-2.37640300	-5.05529500	7.64486900	L
C-C_3	0	-2.05974600	-4.26594300	8.90179400	L
C-C_3	0	-2.77119500	-2.95588100	8.70116200	L
H-H_	0	-2.77346900	-2.53290700	6.57706400	L
H-H_	0	-4.40684700	-2.64953500	7.31520900	L
H-H_	0	-2.64648700	-6.10031400	7.90022300	L
H-H_	0	-1.49707000	-5.07831100	6.96816600	L
H-H_	0	-2.45162600	-4.78835400	9.80158400	L
H-H_	0	-0.96431200	-4.11704500	9.01369900	L
H-H_	0	-3.56132600	-2.82486200	9.47036100	L
H-H_	0	-2.06777300	-2.09863500	8.74457600	L
C-C_3	0	7.30355800	10.83889000	-3.58211100	L
O-O_3	0	6.66217500	9.69645600	-3.06697300	L
C-C_3	0	5.51403600	9.45890900	-3.84808800	L
C-C_3	0	5.33277400	10.58373400	-4.84551400	L
C-C_3	0	6.69704100	11.19098900	-4.92304300	L
H-H_	0	7.16324200	11.68103700	-2.87072400	L
H-H_	0	8.39167400	10.64177200	-3.68701900	L
H-H_	0	5.63934800	8.49624800	-4.38430000	L
H-H_	0	4.62631000	9.38221600	-3.18922600	L
H-H_	0	4.99047500	10.21004800	-5.83450300	L
H-H_	0	4.60602100	11.32868700	-4.45337500	L
H-H_	0	7.28067600	10.71405400	-5.74084900	L
H-H_	0	6.65158800	12.29043400	-5.08095700	L
C-C_3	0	10.27918800	-0.27206400	-6.11678400	L
O-O_3	0	9.44961600	0.86352600	-6.15637600	L
C-C_3	0	8.42705300	0.64205900	-5.22039200	L

C-C_3	0	8.96973300	-0.24230900	-4.12282600	L
C-C_3	0	10.10020500	-0.97938700	-4.78288300	L
H-H_	0	10.00695000	-0.95139400	-6.95419800	L
H-H_	0	11.33635000	0.04265900	-6.24732000	L
H-H_	0	8.08414000	1.60963800	-4.80432300	L
H-H_	0	7.56627600	0.15286200	-5.72135600	L
H-H_	0	9.35369800	0.38477400	-3.29146200	L
H-H_	0	8.19325800	-0.93715200	-3.74156800	L
H-H_	0	11.02276300	-0.91692400	-4.16574000	L
H-H_	0	9.83748900	-2.04828200	-4.94058400	L
C-C_3	0	-10.40786900	-0.19184000	-2.24029400	L
O-O_3	0	-9.99401900	-0.02949400	-0.90437400	L
C-C_3	0	-11.14219800	-0.05048300	-0.09057400	L
C-C_3	0	-12.33990100	-0.46664100	-0.91410100	L
C-C_3	0	-11.91946800	-0.14045500	-2.31173800	L
H-H_	0	-9.96285600	0.60792900	-2.86912300	L
H-H_	0	-10.04205400	-1.17313300	-2.60949100	L
H-H_	0	-10.98980600	-0.75745500	0.74997200	L
H-H_	0	-11.30458500	0.96627300	0.32267900	L
H-H_	0	-12.50993700	-1.55983900	-0.80863100	L
H-H_	0	-13.25716500	0.08729000	-0.61777800	L
H-H_	0	-12.32404500	-0.87093000	-3.04553900	L
H-H_	0	-12.25149600	0.88711200	-2.57636000	L
C-C_3	0	9.73493300	-6.35189200	5.73651900	L
O-O_3	0	10.99563900	-6.58802400	6.32040700	L
C-C_3	0	11.07166400	-5.82317500	7.50103100	L
C-C_3	0	9.70099000	-5.27527600	7.83316300	L
C-C_3	0	9.01268800	-5.26489200	6.50634800	L
H-H_	0	9.86630400	-6.05329300	4.67602100	L
H-H_	0	9.14532500	-7.29245400	5.76893500	L
H-H_	0	11.44822600	-6.45520700	8.33320200	L
H-H_	0	11.78530400	-4.98579000	7.34203000	L
H-H_	0	9.17499700	-5.96540700	8.52928000	L
H-H_	0	9.75946400	-4.25835500	8.27799800	L
H-H_	0	7.92756000	-5.47986700	6.60484800	L
H-H_	0	9.15709700	-4.28058200	6.00864000	L
C-C_3	0	-4.79809400	3.02220200	-8.31448900	L
O-O_3	0	-5.04050700	2.08614700	-7.28841700	L

C-C_3	0	-4.25472000	2.45770200	-6.18280200	L
C-C_3	0	-3.84771200	3.90404400	-6.33892500	L
C-C_3	0	-3.82355400	4.07550600	-7.82426300	L
H-H_	0	-4.38694000	2.50221200	-9.20574700	L
H-H_	0	-5.76100500	3.49866500	-8.59865100	L
H-H_	0	-4.82843200	2.31306400	-5.24770600	L
H-H_	0	-3.35755000	1.80563800	-6.14498300	L
H-H_	0	-4.61765500	4.56652500	-5.88927500	L
H-H_	0	-2.86071300	4.11489300	-5.87727200	L
H-H_	0	-4.13643200	5.09594800	-8.12647700	L
H-H_	0	-2.80444400	3.86222400	-8.21499200	L
C-C_3	0	-2.52135200	12.93100500	-4.08606400	L
O-O_3	0	-1.83808300	11.90542900	-3.40054400	L
C-C_3	0	-2.64970000	10.75570600	-3.43661600	L
C-C_3	0	-3.72105700	10.93907900	-4.48698900	L
C-C_3	0	-3.87480000	12.42441400	-4.54655500	L
H-H_	0	-2.64234800	13.81073500	-3.41913800	L
H-H_	0	-1.91615200	13.23919600	-4.96500200	L
H-H_	0	-2.03033900	9.86681100	-3.67142000	L
H-H_	0	-3.11644900	10.60994800	-2.43806200	L
H-H_	0	-3.36364800	10.55264500	-5.46735600	L
H-H_	0	-4.66934200	10.43571700	-4.20423400	L
H-H_	0	-4.11978600	12.77064200	-5.57421900	L
H-H_	0	-4.66759900	12.75271200	-3.83814500	L
C-C_3	0	5.47988900	2.36819700	9.10371600	L
O-O_3	0	5.55185000	2.25448600	7.70010700	L
C-C_3	0	5.07664900	0.97572200	7.36008300	L
C-C_3	0	4.19634800	0.47750300	8.48145400	L
C-C_3	0	4.77241100	1.15520300	9.68374200	L
H-H_	0	6.50606200	2.45439200	9.52118800	L
H-H_	0	4.91786900	3.29134200	9.35950500	L
H-H_	0	4.50608600	1.02472600	6.40970900	L
H-H_	0	5.94281500	0.29242700	7.21877000	L
H-H_	0	3.14812500	0.81256100	8.32530400	L
H-H_	0	4.23270400	-0.62658900	8.57123300	L
H-H_	0	3.97779200	1.44552700	10.40553500	L
H-H_	0	5.50682000	0.48470700	10.18300000	L
C-C_3	0	-5.23501300	-0.01183200	-0.55492500	L

O-O_3	0	-5.77740200	0.79723800	-1.57146200	L
C-C_3	0	-6.84943500	1.52559600	-1.01752200	L
C-C_3	0	-6.91951300	1.27300500	0.47240400	L
C-C_3	0	-6.16523300	-0.00584300	0.63692100	L
H-H_	0	-5.09441800	-1.04451200	-0.93566500	L
H-H_	0	-4.24290700	0.39268600	-0.26056500	L
H-H_	0	-6.71626700	2.60931900	-1.22046800	L
H-H_	0	-7.78973200	1.19695200	-1.50291800	L
H-H_	0	-6.40174100	2.09126100	1.01872000	L
H-H_	0	-7.96852500	1.18077200	0.82794900	L
H-H_	0	-5.60632700	-0.02983000	1.59561500	L
H-H_	0	-6.86308700	-0.86900200	0.57842100	L
C-C_3	0	7.53441700	-5.74293500	1.18685400	L
O-O_3	0	8.15847000	-5.77933800	2.44851900	L
C-C_3	0	7.29013700	-5.16272100	3.37131100	L
C-C_3	0	5.99344500	-4.80282400	2.67943300	L
C-C_3	0	6.38162800	-4.77126300	1.23741900	L
H-H_	0	8.26578400	-5.43834600	0.41340100	L
H-H_	0	7.15899800	-6.75801600	0.93791700	L
H-H_	0	7.09769100	-5.84787600	4.22266300	L
H-H_	0	7.77857900	-4.24536000	3.76219300	L
H-H_	0	5.23849400	-5.60086500	2.84491400	L
H-H_	0	5.59679300	-3.82649400	3.02762700	L
H-H_	0	5.54972500	-5.08405400	0.57377000	L
H-H_	0	6.72597700	-3.75250500	0.95815500	L
C-C_3	0	11.02880200	3.16647700	-3.91329900	L
O-O_3	0	11.53935800	2.03241200	-3.25411900	L
C-C_3	0	12.83926400	1.79615800	-3.74460100	L
C-C_3	0	13.20663700	2.85750000	-4.76197600	L
C-C_3	0	11.89170200	3.47653500	-5.11505700	L
H-H_	0	9.98381900	2.97664200	-4.22875200	L
H-H_	0	11.02600200	4.02552500	-3.21012500	L
H-H_	0	13.56294400	1.80531700	-2.90238100	L
H-H_	0	12.86682700	0.79228200	-4.21884200	L
H-H_	0	13.87407300	3.61577900	-4.29752200	L
H-H_	0	13.70425700	2.41841600	-5.65403000	L
H-H_	0	11.98681900	4.57109200	-5.28183500	L
H-H_	0	11.47056000	2.98798800	-6.02159800	L

C-C_3	0	4.20678700	7.40320300	-0.55063100	L
O-O_3	0	4.77594700	6.69124100	0.51929700	L
C-C_3	0	4.56759200	7.47382000	1.66695000	L
C-C_3	0	4.50370300	8.92935400	1.25423800	L
C-C_3	0	4.20394300	8.88113000	-0.21696100	L
H-H_	0	4.78770300	7.21637500	-1.47763800	L
H-H_	0	3.16883800	7.05151300	-0.70749700	L
H-H_	0	3.61450200	7.17144100	2.15312200	L
H-H_	0	5.39998200	7.30964400	2.38236700	L
H-H_	0	3.71040900	9.47088900	1.80878500	L
H-H_	0	5.48218100	9.42219600	1.43235200	L
H-H_	0	3.21679100	9.33697400	-0.44491300	L
H-H_	0	4.99048000	9.41273300	-0.78947700	L
C-C_3	0	13.77487600	6.71511900	-0.91309700	L
O-O_3	0	13.59323100	6.35744800	0.43641600	L
C-C_3	0	13.24389900	4.99217200	0.47330200	L
C-C_3	0	13.24690700	4.42556100	-0.93312700	L
C-C_3	0	13.16731600	5.64673400	-1.79325200	L
H-H_	0	13.29814500	7.69850500	-1.10950000	L
H-H_	0	14.86412200	6.80191000	-1.11896500	L
H-H_	0	13.96305400	4.44375900	1.11611300	L
H-H_	0	12.23063500	4.89186000	0.91661800	L
H-H_	0	14.20006200	3.88533000	-1.12586900	L
H-H_	0	12.38813400	3.74313000	-1.10500400	L
H-H_	0	13.73069700	5.52533900	-2.74290800	L
H-H_	0	12.10508800	5.89304600	-2.01165300	L
C-C_3	0	-1.96129100	7.41018500	-7.31905900	L
O-O_3	0	-2.27076200	7.97812200	-8.56723100	L
C-C_3	0	-1.87674800	7.03960500	-9.53492100	L
C-C_3	0	-0.71534900	6.23892200	-8.98307600	L
C-C_3	0	-0.82388200	6.42074700	-7.49395600	L
H-H_	0	-1.67542500	8.21346600	-6.60864300	L
H-H_	0	-2.85802500	6.89263900	-6.91893600	L
H-H_	0	-2.73357100	6.36993600	-9.76819700	L
H-H_	0	-1.57728800	7.56670300	-10.46555000	L
H-H_	0	-0.79082600	5.16838300	-9.27256900	L
H-H_	0	0.24529200	6.65793100	-9.35349500	L
H-H_	0	-1.05789000	5.46030600	-6.98717600	L

H-H_	0	0.12110400	6.83171400	-7.08123300	L
C-C_3	0	10.25880800	-1.40120000	-0.60410200	L
O-O_3	0	11.35172200	-2.05745100	-1.20493800	L
C-C_3	0	10.88130000	-3.26974900	-1.74455700	L
C-C_3	0	9.36987800	-3.28340400	-1.70323600	L
C-C_3	0	9.05139300	-2.31354700	-0.61168900	L
H-H_	0	10.51928200	-1.11398000	0.43560300	L
H-H_	0	10.04060700	-0.47756600	-1.17789000	L
H-H_	0	11.24260700	-3.37905700	-2.78900200	L
H-H_	0	11.28282100	-4.11145600	-1.14059500	L
H-H_	0	8.96014200	-2.92325900	-2.66974600	L
H-H_	0	8.97100000	-4.29442900	-1.48672300	L
H-H_	0	8.11014000	-1.75745700	-0.81326800	L
H-H_	0	8.97616000	-2.84820600	0.36039900	L
C-C_3	0	-9.07501200	-8.81555000	4.85649800	L
O-O_3	0	-8.24174200	-8.72153700	5.98971700	L
C-C_3	0	-6.94397900	-8.39966200	5.54383100	L
C-C_3	0	-6.89794100	-8.46703500	4.03307100	L
C-C_3	0	-8.33147800	-8.30668400	3.64035900	L
H-H_	0	-9.35846600	-9.88006200	4.70710600	L
H-H_	0	-9.99965600	-8.22188700	5.02101600	L
H-H_	0	-6.69117800	-7.37280100	5.88127000	L
H-H_	0	-6.20936400	-9.10512100	5.98720700	L
H-H_	0	-6.25939900	-7.66679500	3.60002100	L
H-H_	0	-6.52737100	-9.46538300	3.71536100	L
H-H_	0	-8.56571200	-7.23295300	3.47639800	L
H-H_	0	-8.57846400	-8.88969500	2.72653100	L
C-C_3	0	-8.87362000	-4.81588000	-6.60799900	L
O-O_3	0	-7.83554800	-5.68539500	-6.99803000	L
C-C_3	0	-7.25879800	-5.14707900	-8.16117600	L
C-C_3	0	-7.51632400	-3.65991300	-8.18382400	L
C-C_3	0	-8.78383800	-3.52057600	-7.39663200	L
H-H_	0	-9.85343300	-5.30939000	-6.78367600	L
H-H_	0	-8.77804900	-4.60527100	-5.52293200	L
H-H_	0	-6.16822100	-5.34831300	-8.16959500	L
H-H_	0	-7.71701400	-5.62993500	-9.05222200	L
H-H_	0	-6.68628000	-3.12187000	-7.67528600	L
H-H_	0	-7.63308000	-3.28399500	-9.22292300	L

H-H_	0	-8.74477700	-2.63695900	-6.72505900	L
H-H_	0	-9.65368200	-3.42907400	-8.08361800	L
C-C_3	0	-1.56253900	8.48487900	1.29081600	L
O-O_3	0	-0.49512500	9.25944500	1.78648900	L
C-C_3	0	0.58314200	9.12250000	0.89054900	L
C-C_3	0	0.10670900	8.41642600	-0.35752900	L
C-C_3	0	-1.07587300	7.64639700	0.13218800	L
H-H_	0	-1.96573800	7.83895100	2.09635300	L
H-H_	0	-2.37079200	9.16342500	0.94779300	L
H-H_	0	0.99071000	10.12211100	0.64011500	L
H-H_	0	1.38427500	8.52728400	1.37684300	L
H-H_	0	-0.21019000	9.16499000	-1.11703800	L
H-H_	0	0.88359000	7.74876500	-0.78280800	L
H-H_	0	-1.85302000	7.53738800	-0.65128500	L
H-H_	0	-0.75590500	6.64585600	0.49691600	L
C-C_3	0	-6.10184300	1.35275000	7.45605300	L
O-O_3	0	-5.61976200	2.35081000	8.32749400	L
C-C_3	0	-4.33090000	1.96684100	8.74476400	L
C-C_3	0	-3.81147300	0.89357700	7.81668000	L
C-C_3	0	-5.06688100	0.25382100	7.31827800	L
H-H_	0	-7.05107100	0.93699900	7.85371200	L
H-H_	0	-6.31044100	1.81065100	6.46618100	L
H-H_	0	-3.65315100	2.84638100	8.73623200	L
H-H_	0	-4.39125600	1.57467800	9.78320600	L
H-H_	0	-3.25720600	1.35748500	6.97221600	L
H-H_	0	-3.15623500	0.16805200	8.34386300	L
H-H_	0	-4.95852300	-0.09592400	6.26958800	L
H-H_	0	-5.34234700	-0.59709800	7.97559000	L
C-C_3	0	-7.59282800	2.90445200	-4.21951700	L
O-O_3	0	-7.24674700	4.21802400	-4.58999700	L
C-C_3	0	-7.77863900	4.45688300	-5.87341300	L
C-C_3	0	-8.67601900	3.30517400	-6.27012600	L
C-C_3	0	-8.18295700	2.18711300	-5.41071300	L
H-H_	0	-6.69671800	2.36819700	-3.84759400	L
H-H_	0	-8.33681600	2.94413700	-3.39764400	L
H-H_	0	-8.34650800	5.40887000	-5.87246900	L
H-H_	0	-6.94369300	4.55168000	-6.59873700	L
H-H_	0	-9.73196000	3.54096800	-6.01801000	L

H-H_	0	-8.59091500	3.06607800	-7.35210600	L
H-H_	0	-8.99958800	1.50261100	-5.10700500	L
H-H_	0	-7.39105200	1.61996100	-5.94288900	L
C-C_3	0	-0.07106600	-6.68090400	-9.76504800	L
O-O_3	0	-0.14677600	-7.92261200	-9.10321500	L
C-C_3	0	0.46007500	-7.77543200	-7.83996200	L
C-C_3	0	1.10809000	-6.41175200	-7.74480300	L
C-C_3	0	0.39680900	-5.61870600	-8.79364100	L
H-H_	0	0.64838800	-6.76758300	-10.60765700	L
H-H_	0	-1.06820400	-6.41539900	-10.17672700	L
H-H_	0	-0.31800500	-7.88128700	-7.05562300	L
H-H_	0	1.21598700	-8.57308800	-7.68765300	L
H-H_	0	0.98034300	-5.97265800	-6.73488200	L
H-H_	0	2.18845400	-6.48282900	-7.99295100	L
H-H_	0	-0.48008700	-5.09877800	-8.35242800	L
H-H_	0	1.06906100	-4.87761500	-9.27840000	L
C-C_3	0	5.19067800	-10.17629100	7.89884300	L
O-O_3	0	6.32713700	-10.69775700	7.25394600	L
C-C_3	0	7.32027600	-9.70836000	7.34379900	L
C-C_3	0	6.64451200	-8.35467100	7.37933500	L
C-C_3	0	5.25378100	-8.65893700	7.86565700	L
H-H_	0	4.27564500	-10.53665600	7.38205900	L
H-H_	0	5.16692700	-10.53873000	8.95002500	L
H-H_	0	7.91321200	-9.86683400	8.27183600	L
H-H_	0	8.00322200	-9.78177000	6.47167100	L
H-H_	0	7.17571900	-7.65806000	8.06368700	L
H-H_	0	6.60601000	-7.91633000	6.35848300	L
H-H_	0	5.08382300	-8.23503800	8.87971700	L
H-H_	0	4.49614500	-8.24997100	7.16374600	L
C-C_3	0	12.30159700	-7.78618900	2.35511500	L
O-O_3	0	13.14859400	-7.40373700	1.29460700	L
C-C_3	0	12.33390100	-7.08352200	0.19015300	L
C-C_3	0	10.89366100	-6.99303100	0.64288500	L
C-C_3	0	10.87354300	-7.86550700	1.85722400	L
H-H_	0	12.62633700	-8.76877200	2.75875800	L
H-H_	0	12.37758600	-7.02927200	3.16566400	L
H-H_	0	12.66352100	-6.12021800	-0.25320100	L
H-H_	0	12.43875700	-7.88087700	-0.57770200	L

H-H_	0	10.64966400	-5.94528000	0.92158700	L
H-H_	0	10.19005500	-7.34976600	-0.13846900	L
H-H_	0	10.15159900	-7.50472000	2.61920000	L
H-H_	0	10.62709300	-8.91179400	1.57013800	L
C-C_3	0	-3.04096200	-2.02306300	-10.22341400	L
O-O_3	0	-2.72079300	-0.70031700	-9.85477000	L
C-C_3	0	-2.73006500	-0.63922700	-8.44935000	L
C-C_3	0	-3.50960300	-1.81587300	-7.91313500	L
C-C_3	0	-3.32764400	-2.84706700	-8.98149600	L
H-H_	0	-3.93764600	-2.00407100	-10.87938900	L
H-H_	0	-2.19562800	-2.46427400	-10.79421800	L
H-H_	0	-1.68468400	-0.67847000	-8.07999500	L
H-H_	0	-3.18674100	0.31445400	-8.11870800	L
H-H_	0	-3.11668500	-2.16129300	-6.93503900	L
H-H_	0	-4.58376300	-1.54947800	-7.81281300	L
H-H_	0	-2.45099700	-3.48864300	-8.74308600	L
H-H_	0	-4.23574600	-3.47762000	-9.10111200	L
C-C_3	0	-3.94209200	3.47799500	-2.61264700	L
O-O_3	0	-2.71366800	3.90098300	-3.15410900	L
C-C_3	0	-2.00131000	4.55412400	-2.13438800	L
C-C_3	0	-2.57157100	4.15823100	-0.79243300	L
C-C_3	0	-3.97118600	3.74692100	-1.11950100	L
H-H_	0	-4.77136400	4.01213000	-3.12137800	L
H-H_	0	-4.06045300	2.39172500	-2.80490000	L
H-H_	0	-0.93003500	4.27906800	-2.20342600	L
H-H_	0	-2.09396800	5.64816900	-2.27260000	L
H-H_	0	-2.00994900	3.29675400	-0.37494900	L
H-H_	0	-2.55185800	5.00190300	-0.07016500	L
H-H_	0	-4.26027200	2.84230900	-0.54829000	L
H-H_	0	-4.67941400	4.56952900	-0.88981000	L
C-C_3	0	0.23136700	-7.65348100	-3.98293100	L
O-O_3	0	0.75992700	-7.01708400	-2.85059000	L
C-C_3	0	-0.33955800	-6.55713700	-2.10437800	L
C-C_3	0	-1.54860400	-6.42451900	-3.01664200	L
C-C_3	0	-1.08740600	-6.99985600	-4.32496900	L
H-H_	0	0.94286500	-7.56006500	-4.82816000	L
H-H_	0	0.08170200	-8.73005100	-3.75752800	L
H-H_	0	-0.54816000	-7.27576000	-1.28380000	L

H-H_	0	-0.08726300	-5.57446100	-1.65380500	L
H-H_	0	-2.40903700	-7.00144800	-2.61440300	L
H-H_	0	-1.84461700	-5.35935700	-3.13665000	L
H-H_	0	-1.81025800	-7.73433900	-4.73442900	L
H-H_	0	-0.92925200	-6.18842300	-5.06541600	L
C-C_3	0	4.84627500	1.33103400	-3.69591100	L
O-O_3	0	4.62853300	2.24361500	-2.64766400	L
C-C_3	0	5.31302500	1.74850800	-1.51780500	L
C-C_3	0	5.86888400	0.36840000	-1.82577000	L
C-C_3	0	5.17093100	-0.01048300	-3.09422600	L
H-H_	0	3.94930200	1.26415700	-4.34238700	L
H-H_	0	5.69690700	1.68086200	-4.31647200	L
H-H_	0	6.13095400	2.44730000	-1.24291100	L
H-H_	0	4.60607400	1.68988800	-0.66386500	L
H-H_	0	6.96392000	0.42995500	-2.00176100	L
H-H_	0	5.66333300	-0.35527100	-1.01056600	L
H-H_	0	5.81160700	-0.61826800	-3.76485600	L
H-H_	0	4.23099600	-0.55500600	-2.86668700	L
C-C_3	0	-7.03931000	10.86631500	-2.08727500	L
O-O_3	0	-6.16749300	11.04718800	-1.00185600	L
C-C_3	0	-6.96297100	11.47094400	0.07873100	L
C-C_3	0	-8.39959300	11.03008900	-0.15432100	L
C-C_3	0	-8.39093300	10.45943900	-1.54516800	L
H-H_	0	-7.12025900	11.81843900	-2.65723500	L
H-H_	0	-6.64071800	10.08312900	-2.76281800	L
H-H_	0	-6.57034800	11.03200000	1.01970300	L
H-H_	0	-6.90757000	12.57839500	0.16079300	L
H-H_	0	-8.70922200	10.25977400	0.58450100	L
H-H_	0	-9.08863000	11.90066700	-0.08992400	L
H-H_	0	-8.46724900	9.35086900	-1.50433300	L
H-H_	0	-9.21721700	10.86957000	-2.16528700	L
C-C_3	0	-12.72605100	-4.32071300	-2.12462200	L
O-O_3	0	-11.55229800	-3.57627100	-2.35904400	L
C-C_3	0	-10.98864600	-4.03507800	-3.56458800	L
C-C_3	0	-12.01238000	-4.86702700	-4.30215100	L
C-C_3	0	-12.87274300	-5.38421500	-3.19427600	L
H-H_	0	-12.68031800	-4.78655800	-1.11756900	L
H-H_	0	-13.59733600	-3.63129800	-2.15510300	L

H-H_	0	-10.67055000	-3.17166200	-4.18438400	L
H-H_	0	-10.09488600	-4.65224600	-3.33024400	L
H-H_	0	-12.61282500	-4.21899200	-4.97815500	L
H-H_	0	-11.54277300	-5.68919500	-4.88260600	L
H-H_	0	-13.92983600	-5.50720300	-3.51557200	L
H-H_	0	-12.47288900	-6.35459000	-2.82429600	L
C-C_3	0	-9.60059100	7.93227300	3.77133800	L
O-O_3	0	-9.72550500	9.21632700	3.20315100	L
C-C_3	0	-8.76766000	10.05098400	3.81282700	L
C-C_3	0	-8.15813000	9.33447100	4.99792200	L
C-C_3	0	-8.38293200	7.89258000	4.67194000	L
H-H_	0	-10.51500600	7.71200400	4.36369800	L
H-H_	0	-9.51317800	7.17376000	2.96475600	L
H-H_	0	-7.97596200	10.29285400	3.07147300	L
H-H_	0	-9.24805100	10.99843700	4.13713900	L
H-H_	0	-7.07958300	9.57341700	5.10959800	L
H-H_	0	-8.70477500	9.60345300	5.92879600	L
H-H_	0	-7.50859600	7.48697600	4.11771900	L
H-H_	0	-8.56428800	7.28529300	5.58523000	L
C-C_3	0	14.98474800	1.39041400	0.98656000	L
O-O_3	0	14.42859900	0.98710700	-0.24465200	L
C-C_3	0	13.10627800	0.58609800	0.01213400	L
C-C_3	0	12.63157500	1.27981900	1.26498500	L
C-C_3	0	13.89782600	1.44024200	2.04804100	L
H-H_	0	15.78358300	0.67718300	1.28349300	L
H-H_	0	15.44114100	2.39538600	0.86164500	L
H-H_	0	12.45609100	0.84673800	-0.84763900	L
H-H_	0	13.08632000	-0.51561800	0.15613200	L
H-H_	0	12.20843200	2.27504100	1.01180200	L
H-H_	0	11.87291100	0.68032900	1.80902700	L
H-H_	0	13.91245800	2.40023100	2.60654000	L
H-H_	0	14.01820500	0.59139900	2.75696000	L
C-C_3	0	9.56809600	-6.92914200	-5.22242100	L
O-O_3	0	9.90345700	-8.21250400	-4.74675400	L
C-C_3	0	10.53331200	-8.05041400	-3.49489300	L
C-C_3	0	10.50805000	-6.58688800	-3.10441200	L
C-C_3	0	10.31694200	-5.89413300	-4.41519900	L
H-H_	0	9.82406100	-6.84508600	-6.29985900	L

H-H_	0	8.47478100	-6.77638800	-5.10174200	L
H-H_	0	10.01547600	-8.66620100	-2.72901000	L
H-H_	0	11.58509300	-8.40042900	-3.57538300	L
H-H_	0	9.63781700	-6.39090900	-2.44064900	L
H-H_	0	11.44969700	-6.27253700	-2.60591500	L
H-H_	0	9.74363700	-4.94845900	-4.31182700	L
H-H_	0	11.30435400	-5.68725000	-4.88412700	L
C-C_3	0	-0.30048600	11.20151700	-6.72460100	L
O-O_3	0	0.43796400	12.15684700	-6.00652600	L
C-C_3	0	1.74622200	11.65093900	-5.90524400	L
C-C_3	0	1.99826200	10.69228000	-7.05443000	L
C-C_3	0	0.64031300	10.47913700	-7.66682300	L
H-H_	0	-0.76395300	10.48583700	-6.01178900	L
H-H_	0	-1.11142000	11.70436500	-7.29299800	L
H-H_	0	2.47229100	12.49100300	-5.93388300	L
H-H_	0	1.85958500	11.12143200	-4.93567200	L
H-H_	0	2.69905500	11.13593300	-7.79527800	L
H-H_	0	2.41910300	9.73834700	-6.67367700	L
H-H_	0	0.59821900	10.94169200	-8.67742000	L
H-H_	0	0.38875000	9.39936400	-7.74221300	L
C-C_3	0	2.63869800	-5.28944800	4.33919900	L
O-O_3	0	2.47957000	-5.92405200	3.09703500	L
C-C_3	0	2.12138200	-7.26226300	3.35259900	L
C-C_3	0	2.18422800	-7.53511800	4.84390700	L
C-C_3	0	2.89861000	-6.34012100	5.38829100	L
H-H_	0	3.48516300	-4.57594800	4.29233500	L
H-H_	0	1.71115000	-4.72959200	4.57507800	L
H-H_	0	1.09763800	-7.45146700	2.96590100	L
H-H_	0	2.82962000	-7.92843900	2.81647500	L
H-H_	0	1.15952800	-7.58950600	5.26854700	L
H-H_	0	2.73071500	-8.47541000	5.06785600	L
H-H_	0	2.50654100	-6.03467700	6.38092100	L
H-H_	0	3.98919200	-6.54471800	5.44983900	L
C-C_3	0	0.02007800	-10.46366800	2.29369900	L
O-O_3	0	1.07708500	-10.24042500	3.19564200	L
C-C_3	0	0.91305100	-11.12717500	4.27788200	L
C-C_3	0	-0.30252100	-12.00273500	4.03984300	L
C-C_3	0	-1.06130000	-11.26180100	2.98459000	L

H-H_	0	-0.37577300	-9.49087400	1.93479400	L
H-H_	0	0.40666100	-11.03224600	1.42161000	L
H-H_	0	1.82594300	-11.75040300	4.38991300	L
H-H_	0	0.77783300	-10.53383900	5.20710800	L
H-H_	0	0.01482500	-12.99603700	3.65127200	L
H-H_	0	-0.90601300	-12.13270800	4.96450800	L
H-H_	0	-1.58002100	-11.95052500	2.28324700	L
H-H_	0	-1.80126400	-10.57773800	3.45379500	L
C-C_3	0	-7.50292800	-4.53433000	4.96259900	L
O-O_3	0	-6.43754000	-4.26671300	5.83635400	L
C-C_3	0	-7.00638700	-3.81176000	7.04190400	L
C-C_3	0	-8.49838200	-4.10147300	7.04830600	L
C-C_3	0	-8.71865400	-4.88038400	5.78708800	L
H-H_	0	-7.70934800	-3.62961600	4.35502200	L
H-H_	0	-7.23247200	-5.36746300	4.28293100	L
H-H_	0	-6.51085900	-4.32683100	7.88886300	L
H-H_	0	-6.82702900	-2.72148400	7.14343500	L
H-H_	0	-8.80296900	-4.68435700	7.94379600	L
H-H_	0	-9.07064400	-3.15055600	7.01729900	L
H-H_	0	-8.73398900	-5.96793700	6.00968000	L
H-H_	0	-9.65853900	-4.58894000	5.27353500	L
C-C_3	0	-3.99904000	7.30963300	-4.04894400	L
O-O_3	0	-3.54363100	7.73812900	-2.78861100	L
C-C_3	0	-4.62716700	7.66820600	-1.89197500	L
C-C_3	0	-5.83625700	7.08803200	-2.59772200	L
C-C_3	0	-5.50783200	7.26722800	-4.04154500	L
H-H_	0	-3.63667500	8.00456000	-4.83371900	L
H-H_	0	-3.58953800	6.29885700	-4.25825700	L
H-H_	0	-4.34866000	7.04173300	-1.01896900	L
H-H_	0	-4.85273500	8.69266900	-1.52973300	L
H-H_	0	-5.93632400	6.00784700	-2.36402400	L
H-H_	0	-6.77492300	7.61504000	-2.32749500	L
H-H_	0	-5.89844900	6.43299200	-4.65720300	L
H-H_	0	-5.91528800	8.23383200	-4.40521900	L
C-C_3	0	-7.56736700	-11.69389600	1.15498100	L
O-O_3	0	-6.52080200	-12.38112900	0.50749000	L
C-C_3	0	-6.74310700	-12.29230200	-0.88130500	L
C-C_3	0	-8.10938600	-11.68904600	-1.13550400	L

C-C_3	0	-8.38796300	-10.94122900	0.12909000	L
H-H_	0	-8.20834900	-12.43249300	1.68333300	L
H-H_	0	-7.14678500	-10.99291800	1.90488700	L
H-H_	0	-5.95372000	-11.65240700	-1.32890500	L
H-H_	0	-6.67458000	-13.30284400	-1.33715200	L
H-H_	0	-8.11464400	-11.02038400	-2.02253100	L
H-H_	0	-8.86140000	-12.49772800	-1.27072100	L
H-H_	0	-8.02423700	-9.89444300	0.04246100	L
H-H_	0	-9.47056400	-10.94301300	0.38140700	L
C-C_3	0	7.22666600	2.61227900	-8.46494900	L
O-O_3	0	5.84422600	2.49246000	-8.24993200	L
C-C_3	0	5.57860900	1.11659500	-8.12349900	L
C-C_3	0	6.69853400	0.32456000	-8.78074500	L
C-C_3	0	7.70204300	1.36899000	-9.18275700	L
H-H_	0	7.43547400	3.51744700	-9.07356800	L
H-H_	0	7.74407200	2.71580400	-7.48597300	L
H-H_	0	5.49811700	0.86305100	-7.04503300	L
H-H_	0	4.60946200	0.88450100	-8.61261300	L
H-H_	0	7.14818100	-0.39270200	-8.06131300	L
H-H_	0	6.32738100	-0.23237600	-9.66926400	L
H-H_	0	8.73419500	1.09140400	-8.87724000	L
H-H_	0	7.66587200	1.52984200	-10.28285500	L
C-C_3	0	9.63446300	5.89947500	-6.55411100	L
O-O_3	0	9.80554500	6.57209700	-5.32659000	L
C-C_3	0	9.01227100	7.73227400	-5.37093600	L
C-C_3	0	7.91402500	7.52886000	-6.38574600	L
C-C_3	0	8.51464100	6.55050900	-7.34801100	L
H-H_	0	10.58519200	5.93214700	-7.12845800	L
H-H_	0	9.38829300	4.83562300	-6.35125700	L
H-H_	0	8.57629900	7.92726600	-4.37001000	L
H-H_	0	9.64672400	8.59877200	-5.65970400	L
H-H_	0	7.02903900	7.08157500	-5.88929800	L
H-H_	0	7.62681100	8.47983200	-6.88380800	L
H-H_	0	7.76528400	5.80339000	-7.68863700	L
H-H_	0	8.93109700	7.08763800	-8.22859700	L
C-C_3	0	-2.24697600	-12.73259100	-0.92020800	L
O-O_3	0	-2.26425000	-11.33180100	-1.02328800	L
C-C_3	0	-0.99410200	-10.92827500	-1.48209300	L

C-C_3	0	-0.04529000	-12.11456500	-1.47869300	L
C-C_3	0	-0.81425800	-13.18415300	-0.76508200	L
H-H_	0	-2.85295400	-13.05137200	-0.04556100	L
H-H_	0	-2.69029100	-13.17066400	-1.84167200	L
H-H_	0	-1.08825100	-10.50847700	-2.50548000	L
H-H_	0	-0.60879300	-10.13207600	-0.81141600	L
H-H_	0	0.17214700	-12.43540900	-2.52088600	L
H-H_	0	0.90615900	-11.87746200	-0.95504800	L
H-H_	0	-0.64608500	-14.18695300	-1.21421900	L
H-H_	0	-0.54001400	-13.19984100	0.31174900	L
C-C_3	0	-3.83377000	10.90843500	6.00845600	L
O-O_3	0	-4.41920100	10.41781600	4.82322600	L
C-C_3	0	-3.38000500	9.93900600	4.00303000	L
C-C_3	0	-2.06257700	10.46925200	4.51692300	L
C-C_3	0	-2.33721200	10.66815800	5.97262800	L
H-H_	0	-4.03865400	11.99817900	6.08304200	L
H-H_	0	-4.28777300	10.40275300	6.88727100	L
H-H_	0	-3.37886100	8.82752900	4.03466900	L
H-H_	0	-3.54719000	10.26610100	2.95640500	L
H-H_	0	-1.23390800	9.75241900	4.34532200	L
H-H_	0	-1.82818200	11.44371200	4.03348400	L
H-H_	0	-2.09054200	9.74126800	6.53476100	L
H-H_	0	-1.76525600	11.52672200	6.38697500	L
C-C_3	0	-1.41001400	13.50726400	-0.28907900	L
O-O_3	0	-2.62805600	14.16238200	-0.56318600	L
C-C_3	0	-3.66447700	13.23612800	-0.34792100	L
C-C_3	0	-3.15020500	12.12395800	0.53507500	L
C-C_3	0	-1.68242800	12.11018100	0.23980000	L
H-H_	0	-0.79964800	13.45981300	-1.21596200	L
H-H_	0	-0.85006200	14.09522200	0.46950800	L
H-H_	0	-4.52791800	13.74374900	0.13160700	L
H-H_	0	-3.99363800	12.82543300	-1.32658100	L
H-H_	0	-3.32127900	12.38066800	1.60378800	L
H-H_	0	-3.63326900	11.15293500	0.29395200	L
H-H_	0	-1.08625500	11.89432600	1.15302100	L
H-H_	0	-1.45588900	11.35302700	-0.54243000	L
C-C_3	0	-12.30623700	-4.98435300	3.59070300	L
O-O_3	0	-11.77946100	-3.69152500	3.73995000	L

C-C_3	0	-12.65739400	-2.81875900	3.07135400	L
C-C_3	0	-13.48808000	-3.60555000	2.07023000	L
C-C_3	0	-13.07815800	-5.03485000	2.29159000	L
H-H_	0	-11.48105000	-5.72819900	3.58283600	L
H-H_	0	-12.97838700	-5.20677800	4.44901800	L
H-H_	0	-13.31612700	-2.32081900	3.81540500	L
H-H_	0	-12.06646300	-2.03860600	2.54971200	L
H-H_	0	-14.57250000	-3.47241300	2.27819000	L
H-H_	0	-13.27069100	-3.28653300	1.02739100	L
H-H_	0	-13.95790100	-5.71018100	2.36754000	L
H-H_	0	-12.41357600	-5.37683100	1.46817100	L
C-C_3	0	1.96618200	7.52121500	8.89442200	L
O-O_3	0	1.76848800	8.69736000	8.14294500	L
C-C_3	0	0.38345600	8.84855700	7.94741200	L
C-C_3	0	-0.34976700	8.01743000	8.97379700	L
C-C_3	0	0.62343100	6.92117300	9.27467800	L
H-H_	0	2.56338300	6.79834300	8.29912300	L
H-H_	0	2.53901600	7.77601100	9.81169900	L
H-H_	0	0.10581700	9.91953000	8.04475600	L
H-H_	0	0.12209100	8.50658700	6.92284600	L
H-H_	0	-0.53731800	8.62270400	9.88809400	L
H-H_	0	-1.30867600	7.62213800	8.57635400	L
H-H_	0	0.59066800	6.62800600	10.34657600	L
H-H_	0	0.40248700	6.03572600	8.64110300	L
C-C_3	0	5.71586500	-9.29237000	-8.40632600	L
O-O_3	0	6.58052500	-9.10214000	-7.31639800	L
C-C_3	0	6.22429600	-7.87345300	-6.73022000	L
C-C_3	0	4.80630100	-7.50922200	-7.13687900	L
C-C_3	0	4.41384900	-8.58290500	-8.11244400	L
H-H_	0	6.18469500	-8.87531100	-9.32473700	L
H-H_	0	5.54150100	-10.37815200	-8.55829400	L
H-H_	0	6.29492400	-7.96581700	-5.62705600	L
H-H_	0	6.93468500	-7.08738800	-7.06701800	L
H-H_	0	4.12398700	-7.49856000	-6.25972300	L
H-H_	0	4.78812500	-6.50949400	-7.62004700	L
H-H_	0	3.70010400	-9.28929100	-7.64016100	L
H-H_	0	3.96333300	-8.16007000	-9.03543900	L
C-C_3	0	2.24456000	-8.71206100	-0.19323400	L

O-O_3	0	1.80689100	-7.43090400	0.19699200	L
C-C_3	0	2.77420200	-6.49963500	-0.23229400	L
C-C_3	0	3.79672500	-7.20318300	-1.09676300	L
C-C_3	0	3.67749700	-8.62583000	-0.66190700	L
H-H_	0	2.16376800	-9.41355700	0.66106100	L
H-H_	0	1.59897600	-9.07742800	-1.02005600	L
H-H_	0	2.28188300	-5.67954600	-0.79540500	L
H-H_	0	3.27487900	-6.06138900	0.65527400	L
H-H_	0	3.51271600	-7.10802900	-2.16607900	L
H-H_	0	4.82277300	-6.81098100	-0.93795500	L
H-H_	0	3.88976600	-9.33305700	-1.48952500	L
H-H_	0	4.36361100	-8.81956100	0.18870400	L
C-C_3	0	-8.96298900	9.40795500	-5.10870200	L
O-O_3	0	-9.18589600	9.29286700	-6.49499200	L
C-C_3	0	-10.02815800	8.18085800	-6.70153800	L
C-C_3	0	-10.45734200	7.61452200	-5.36317000	L
C-C_3	0	-9.41637400	8.13716000	-4.42533600	L
H-H_	0	-7.88676500	9.59220900	-4.91476000	L
H-H_	0	-9.54370000	10.27350000	-4.72220900	L
H-H_	0	-10.91541000	8.48846700	-7.29483400	L
H-H_	0	-9.47075400	7.41008800	-7.27637700	L
H-H_	0	-11.45630700	8.01574600	-5.08216800	L
H-H_	0	-10.48875900	6.50463500	-5.37496300	L
H-H_	0	-9.83276500	8.33485100	-3.41429500	L
H-H_	0	-8.57002100	7.41937900	-4.35355100	L
C-C_3	0	-5.49218500	8.27553600	1.70943300	L
O-O_3	0	-4.62587900	7.18021200	1.86944900	L
C-C_3	0	-5.44350700	6.04188400	1.94374800	L
C-C_3	0	-6.69936100	6.29920400	1.13899300	L
C-C_3	0	-6.77632000	7.79828100	1.05802800	L
H-H_	0	-5.71102300	8.71918800	2.70502700	L
H-H_	0	-4.99994900	9.04489100	1.07860000	L
H-H_	0	-4.89871800	5.16417600	1.54161200	L
H-H_	0	-5.69800700	5.84618200	3.00651200	L
H-H_	0	-6.59731700	5.86217700	0.12295000	L
H-H_	0	-7.59489600	5.87364500	1.63920800	L
H-H_	0	-6.83193400	8.13469500	0.00331300	L
H-H_	0	-7.66101300	8.18358800	1.60357600	L

C-C_3	0	-3.74928800	-5.02562400	-6.00776700	L
O-O_3	0	-2.80748900	-5.52621600	-6.92120900	L
C-C_3	0	-3.38994000	-6.67006900	-7.49259400	L
C-C_3	0	-4.36360400	-7.27016200	-6.49549000	L
C-C_3	0	-4.56059400	-6.18529000	-5.47175600	L
H-H_	0	-3.22909900	-4.49917400	-5.18054600	L
H-H_	0	-4.41452100	-4.30314400	-6.52777400	L
H-H_	0	-3.91652700	-6.38396600	-8.42924700	L
H-H_	0	-2.59884900	-7.40391900	-7.74805200	L
H-H_	0	-5.32271000	-7.54040200	-6.98859900	L
H-H_	0	-3.92559700	-8.17276100	-6.02115200	L
H-H_	0	-5.63158800	-5.91166100	-5.37213300	L
H-H_	0	-4.16756600	-6.50605400	-4.48284600	L
C-C_3	0	-3.87059800	5.72684500	7.69936000	L
O-O_3	0	-5.20301500	5.27021800	7.67159100	L
C-C_3	0	-5.93063400	6.12364800	6.81786600	L
C-C_3	0	-4.98257800	7.09107400	6.14107500	L
C-C_3	0	-3.79874500	7.09208000	7.05189500	L
H-H_	0	-3.51134800	5.77539800	8.74924800	L
H-H_	0	-3.23345800	5.00751000	7.14139300	L
H-H_	0	-6.47445400	5.52181600	6.06206100	L
H-H_	0	-6.67558000	6.68453900	7.42280200	L
H-H_	0	-4.69228800	6.70786900	5.13850500	L
H-H_	0	-5.42523700	8.10366500	6.04485300	L
H-H_	0	-2.85053900	7.23902500	6.49639200	L
H-H_	0	-3.91209000	7.88586200	7.82316200	L
C-C_3	0	8.33469700	-1.01098900	5.34968300	L
O-O_3	0	9.48067900	-1.51550100	4.71026700	L
C-C_3	0	9.24011500	-1.48113300	3.32216900	L
C-C_3	0	7.79209200	-1.11546500	3.05658400	L
C-C_3	0	7.15728100	-1.13888100	4.41370000	L
H-H_	0	8.14992000	-1.57738000	6.28534200	L
H-H_	0	8.49998100	0.05590000	5.61069800	L
H-H_	0	9.91570800	-0.73902700	2.84859000	L
H-H_	0	9.46134000	-2.48131800	2.89440800	L
H-H_	0	7.72546400	-0.09762300	2.61868400	L
H-H_	0	7.31026600	-1.84175100	2.36858500	L
H-H_	0	6.43179100	-0.30732900	4.54901900	L

H-H_	0	6.64776800	-2.11138700	4.57775500	L
C-C_3	0	4.33415900	-11.46131800	-5.05527700	L
O-O_3	0	4.38015200	-10.07407900	-4.82524100	L
C-C_3	0	3.06296600	-9.67085600	-4.55424200	L
C-C_3	0	2.33034000	-10.83082700	-3.92375200	L
C-C_3	0	3.07056700	-12.03452400	-4.43294100	L
H-H_	0	4.34786600	-11.64911500	-6.15001000	L
H-H_	0	5.23220500	-11.93755400	-4.60878900	L
H-H_	0	3.07209700	-8.79625100	-3.87240800	L
H-H_	0	2.56327900	-9.37650200	-5.50235900	L
H-H_	0	2.40355700	-10.77107200	-2.81802200	L
H-H_	0	1.26236900	-10.84711700	-4.22270400	L
H-H_	0	3.31199000	-12.73090900	-3.60023200	L
H-H_	0	2.46920000	-12.56960900	-5.20024300	L
C-C_3	0	-7.01853400	0.03916000	11.15349900	L
O-O_3	0	-5.97192700	-0.87787800	10.92389100	L
C-C_3	0	-6.54189300	-2.04425900	10.37790200	L
C-C_3	0	-7.94468400	-1.73365700	9.91031800	L
C-C_3	0	-8.34514900	-0.60882200	10.80846300	L
H-H_	0	-7.00796700	0.36057300	12.21669700	L
H-H_	0	-6.85878100	0.93308000	10.51360300	L
H-H_	0	-5.92449400	-2.40500000	9.53141100	L
H-H_	0	-6.56751800	-2.83407200	11.16001200	L
H-H_	0	-7.92248500	-1.38156400	8.85618700	L
H-H_	0	-8.62127700	-2.61025900	10.00808100	L
H-H_	0	-9.03462700	0.09836800	10.30147600	L
H-H_	0	-8.82547600	-1.00996300	11.72834200	L
C-C_3	0	8.36493600	7.69368700	0.90120500	L
O-O_3	0	9.63585400	8.28721000	0.85835400	L
C-C_3	0	10.21937100	7.85660500	-0.34398500	L
C-C_3	0	9.11593200	7.62459700	-1.35700500	L
C-C_3	0	7.86747100	7.53546700	-0.52077300	L
H-H_	0	8.44113700	6.70466900	1.40102000	L
H-H_	0	7.67359300	8.33060200	1.48862700	L
H-H_	0	10.92953200	8.62761200	-0.71035300	L
H-H_	0	10.77796900	6.91364100	-0.16020900	L
H-H_	0	9.05156100	8.48654600	-2.05561200	L
H-H_	0	9.29085800	6.68889400	-1.93185600	L

H-H_	0	7.17605700	8.36514800	-0.77603500	L
H-H_	0	7.35077900	6.56264700	-0.65824000	L
C-C_3	0	-7.16759000	0.08667600	-11.01662000	L
O-O_3	0	-8.55977900	0.04760000	-10.79937000	L
C-C_3	0	-8.77416400	-0.07263000	-9.41130600	L
C-C_3	0	-7.45647600	0.08444200	-8.68295900	L
C-C_3	0	-6.44976000	-0.26291600	-9.73179200	L
H-H_	0	-6.89259900	-0.62857500	-11.82086800	L
H-H_	0	-6.88348800	1.11170500	-11.34029800	L
H-H_	0	-9.49764900	0.70064900	-9.07484800	L
H-H_	0	-9.20541300	-1.07466500	-9.20137300	L
H-H_	0	-7.32526500	1.14257700	-8.37207400	L
H-H_	0	-7.38529000	-0.58412000	-7.79855400	L
H-H_	0	-5.50852800	0.31366400	-9.61231400	L
H-H_	0	-6.22857300	-1.35137900	-9.70611600	L
C-C_3	0	1.65659600	-10.54778900	-10.14261200	L
O-O_3	0	0.82767200	-11.00719600	-9.10391800	L
C-C_3	0	1.65242800	-11.73781200	-8.23203500	L
C-C_3	0	2.80544100	-12.31648700	-9.02678000	L
C-C_3	0	2.83905000	-11.48795700	-10.28253400	L
H-H_	0	1.07686500	-10.50957700	-11.08919900	L
H-H_	0	2.00877100	-9.52047700	-9.90649600	L
H-H_	0	2.03020000	-11.06464600	-7.43262700	L
H-H_	0	1.06454600	-12.55075800	-7.75643800	L
H-H_	0	3.75974000	-12.23977700	-8.46327500	L
H-H_	0	2.60538900	-13.38254300	-9.27346800	L
H-H_	0	3.78925500	-10.91645600	-10.36407400	L
H-H_	0	2.71935500	-12.13558200	-11.17890400	L
C-C_3	0	-3.17779700	-6.37244700	4.07197400	L
O-O_3	0	-3.93248700	-7.04621500	5.05365100	L
C-C_3	0	-3.16592100	-8.13846300	5.49803500	L
C-C_3	0	-1.71381100	-7.86982100	5.18540100	L
C-C_3	0	-1.78614000	-6.97619900	3.98834700	L
H-H_	0	-3.69314300	-6.44728700	3.09137800	L
H-H_	0	-3.11118600	-5.29941400	4.34812500	L
H-H_	0	-3.30641700	-8.27797600	6.59032500	L
H-H_	0	-3.50645700	-9.05925000	4.97631800	L
H-H_	0	-1.23730000	-7.33091500	6.03052300	L

H-H_	0	-1.15335100	-8.80470000	4.97552900	L
H-H_	0	-0.99420200	-6.19823500	4.02004200	L
H-H_	0	-1.68227700	-7.57729800	3.06020100	L
C-C_3	0	-13.82772600	5.86837500	-4.33223000	L
O-O_3	0	-13.14157600	5.52279800	-5.50889900	L
C-C_3	0	-12.78992200	4.16988800	-5.37039000	L
C-C_3	0	-13.82516800	3.48934200	-4.49693000	L
C-C_3	0	-14.50639500	4.62678500	-3.78366200	L
H-H_	0	-13.10417500	6.27337100	-3.59113200	L
H-H_	0	-14.57904800	6.65483900	-4.55716200	L
H-H_	0	-12.74752700	3.69062600	-6.37123100	L
H-H_	0	-11.78595500	4.09948500	-4.90014600	L
H-H_	0	-14.55575400	2.93387600	-5.12518700	L
H-H_	0	-13.34404100	2.78946500	-3.78034100	L
H-H_	0	-15.59244500	4.63935800	-4.02373500	L
H-H_	0	-14.37159000	4.55397800	-2.68241200	L
C-C_3	0	-7.65900200	-4.35428500	1.29937600	L
O-O_3	0	-7.57020400	-3.77218700	0.02315200	L
C-C_3	0	-8.84246400	-3.25435800	-0.25786300	L
C-C_3	0	-9.86982100	-4.11837200	0.43836000	L
C-C_3	0	-9.09583600	-4.77717500	1.54757000	L
H-H_	0	-6.97879000	-5.23073500	1.35947200	L
H-H_	0	-7.33850600	-3.60799800	2.05584600	L
H-H_	0	-8.90088900	-2.20989300	0.11497100	L
H-H_	0	-9.01183300	-3.25182900	-1.35340200	L
H-H_	0	-10.70797600	-3.50277600	0.82697300	L
H-H_	0	-10.25995000	-4.88794400	-0.26237500	L
H-H_	0	-9.44053800	-4.42671700	2.54306200	L
H-H_	0	-9.19945700	-5.88254700	1.49054800	L
C-C_3	0	5.12336300	-9.87115700	3.89422500	L
O-O_3	0	5.97285500	-10.98752500	3.76090500	L
C-C_3	0	7.21052400	-10.53115900	3.26724600	L
C-C_3	0	7.25020400	-9.01991300	3.32551500	L
C-C_3	0	5.80383800	-8.64062600	3.33451800	L
H-H_	0	4.16752000	-10.05936900	3.36299000	L
H-H_	0	4.89998400	-9.72024900	4.97038400	L
H-H_	0	8.03633800	-10.96267100	3.87184100	L
H-H_	0	7.32615100	-10.87408300	2.21637400	L

H-H_	0	7.73871100	-8.68542200	4.26475400	L
H-H_	0	7.78768100	-8.59031600	2.45513600	L
H-H_	0	5.61404600	-7.75090500	3.96964700	L
H-H_	0	5.45391700	-8.44399700	2.29854300	L
C-C_3	0	-8.74624500	4.01955400	8.44298700	L
O-O_3	0	-8.86910800	2.73931400	9.01302000	L
C-C_3	0	-9.94441000	2.09193000	8.36963200	L
C-C_3	0	-10.57946000	3.03003700	7.35973400	L
C-C_3	0	-10.04560500	4.37103000	7.75735300	L
H-H_	0	-8.50838300	4.76338900	9.23256500	L
H-H_	0	-7.91651300	4.00717400	7.70378000	L
H-H_	0	-9.57666400	1.17307000	7.86834100	L
H-H_	0	-10.69483600	1.79901700	9.13485900	L
H-H_	0	-10.24123100	2.76943400	6.33342700	L
H-H_	0	-11.68992200	2.99877300	7.40523900	L
H-H_	0	-9.88580400	5.03888800	6.88505100	L
H-H_	0	-10.73678500	4.85514200	8.48228400	L
C-C_3	0	7.10214700	2.77463000	4.41589800	L
O-O_3	0	7.91717500	3.92271900	4.37887600	L
C-C_3	0	7.07232300	5.04272600	4.24995400	L
C-C_3	0	5.62945900	4.59946300	4.34800800	L
C-C_3	0	5.70119100	3.14846000	3.99437500	L
H-H_	0	7.51538900	2.00053700	3.74012600	L
H-H_	0	7.09481900	2.36820300	5.44901000	L
H-H_	0	7.30723100	5.77603000	5.04554200	L
H-H_	0	7.25426600	5.52336000	3.26586100	L
H-H_	0	5.27145100	4.72222900	5.39276400	L
H-H_	0	4.97337900	5.16520600	3.65439700	L
H-H_	0	4.93015700	2.54976800	4.52382100	L
H-H_	0	5.59571700	3.01728000	2.89532100	L
C-C_3	0	7.58672700	-9.33268600	-0.52433200	L
O-O_3	0	7.34326900	-8.32123900	-1.47059500	L
C-C_3	0	6.85976900	-8.94449900	-2.63910100	L
C-C_3	0	6.88456000	-10.45187600	-2.46694900	L
C-C_3	0	7.72450000	-10.65203600	-1.24515700	L
H-H_	0	8.51186000	-9.10476400	0.04313600	L
H-H_	0	6.73589100	-9.37370800	0.18765100	L
H-H_	0	5.82684200	-8.59926900	-2.84542300	L

H-H_	0	7.50137600	-8.64963000	-3.49449000	L
H-H_	0	5.85508000	-10.83140700	-2.28739400	L
H-H_	0	7.32371900	-10.96070000	-3.35245500	L
H-H_	0	7.36378900	-11.50160300	-0.62594600	L
H-H_	0	8.78603800	-10.81562300	-1.53517400	L
C-C_3	0	-5.33173900	-5.80768100	10.40852300	L
O-O_3	0	-4.57927300	-6.77691500	9.72386800	L
C-C_3	0	-5.40176200	-7.23985700	8.68347400	L
C-C_3	0	-6.84590600	-7.13648600	9.13272100	L
C-C_3	0	-6.80311400	-6.15485900	10.27482400	L
H-H_	0	-5.03116800	-5.78818500	11.47747400	L
H-H_	0	-5.12796300	-4.80834500	9.96662200	L
H-H_	0	-5.23275000	-6.61659500	7.77767500	L
H-H_	0	-5.14374400	-8.29275900	8.44247200	L
H-H_	0	-7.49570100	-6.77687200	8.30667100	L
H-H_	0	-7.21116900	-8.12571600	9.48641700	L
H-H_	0	-7.40737900	-5.24769400	10.05612800	L
H-H_	0	-7.17607100	-6.63061200	11.20841100	L
C-C_R	0	-0.77797800	-1.46815500	3.40043200	H
C-C_R	0	-0.30517200	-2.63411000	5.20564800	H
C-C_3	0	0.59450000	-0.30430300	5.24741700	H
H-H_	0	1.21454600	0.11849800	4.46070100	H
N-N_R	0	-0.97510300	-3.45760000	4.46262400	H
N-N_R	0	-1.28580400	-2.72691000	3.35079600	H
N-N_R	0	-0.18116200	-1.39802000	4.62975400	H
C-C_3	0	0.45090800	-2.91912900	6.45973800	H
H-H_	0	0.61468600	-3.99024900	6.56882500	H
H-H_	0	-0.08468800	-2.54086900	7.34117300	H
C-C_3	0	1.51253600	-0.87833200	6.36798600	H
O-O_3	0	1.69291900	-2.27465300	6.32155400	H
C-C_R	0	-2.15286400	-3.27700100	2.35268300	H
C-C_R	0	-3.45935500	-2.77741400	2.28994900	H
C-C_R	0	-1.65066500	-4.19943800	1.43463300	H
C-C_R	0	-4.24548400	-3.16493200	1.21289000	H
C-C_R	0	-2.49037100	-4.56841600	0.38321900	H
C-C_R	0	-3.76736500	-4.03823200	0.23371100	H
H-H_	0	-5.24368700	-2.75104300	1.12041400	H
H-H_	0	-2.11988000	-5.26091600	-0.36387900	H

H-H_	0	2.50105700	-0.43478300	6.26503700	H
C-C_R	0	-0.75001000	-0.42711200	2.38975600	H
O-O_R	0	-0.37979800	0.76251400	2.80998300	H
C-C_R	0	-1.09790400	-0.73503600	1.06276600	H
C-C_R	0	-1.11198300	0.17200200	0.02112900	H
H-H_	0	-1.34844700	-1.76249600	0.83821700	H
H-H_	0	-0.82237800	1.19684000	0.23706900	H
C-C_R	0	-1.52314100	-0.12671900	-1.31739200	H
C-C_R	0	-1.42864900	0.88036100	-2.30067400	H
C-C_R	0	-2.02452000	-1.38977900	-1.73016400	H
C-C_R	0	-1.84444600	0.66876400	-3.60873300	H
H-H_	0	-1.02430800	1.84294600	-2.00936900	H
C-C_R	0	-2.40367600	-1.60579600	-3.03815100	H
H-H_	0	-2.13067800	-2.19491100	-1.00914100	H
C-C_R	0	-2.30382900	-0.57980700	-3.99599300	H
H-H_	0	-1.75000400	1.46784800	-4.33503700	H
H-H_	0	-2.78055800	-2.58208100	-3.33151500	H
H-H_	0	-2.59506900	-0.75594400	-5.02545100	H
C-C_3	0	-4.60446500	-4.37633100	-0.96890200	H
H-H_	0	-5.41493900	-5.06043900	-0.70788700	H
H-H_	0	-5.05491900	-3.47230400	-1.38218300	H
H-H_	0	-4.00624000	-4.85140600	-1.74858000	H
C-C_3	0	-3.96715100	-1.80116000	3.31426600	H
H-H_	0	-3.66060700	-2.08567300	4.32355300	H
H-H_	0	-3.58067200	-0.79667300	3.11257700	H
H-H_	0	-5.05523500	-1.76207700	3.28589500	H
C-C_3	0	-0.23900300	-4.71821700	1.48912200	H
H-H_	0	0.21392300	-4.56768800	2.46820800	H
H-H_	0	-0.22249900	-5.78625200	1.26104400	H
H-H_	0	0.38281900	-4.21140400	0.74282600	H
H-H_	0	1.10221800	-0.59126800	7.34732800	H
C-C_3	0	-0.32876000	0.78351200	5.80907700	H
H-H_	0	-0.89867300	1.21156100	4.98042000	H
H-H_	0	-1.02857600	0.31618500	6.51302800	H
C-C_R	0	0.48891800	1.83194000	6.53024500	H
C-C_R	0	0.14648900	2.22388800	7.82445400	H
C-C_R	0	1.63456800	2.37874700	5.93988100	H
C-C_R	0	0.94186200	3.11587500	8.53718900	H

H-H_	0	-0.75594800	1.82210400	8.27475800	H
C-C_R	0	2.43191400	3.26683800	6.65449400	H
H-H_	0	1.91075600	2.11058800	4.92073800	H
C-C_R	0	2.09711300	3.62864800	7.95691100	H
H-H_	0	0.66278100	3.40127400	9.54634800	H
H-H_	0	3.31832400	3.68019700	6.18976400	H
H-H_	0	2.73020200	4.31210600	8.51179000	H
H-H_	0	0.79656500	0.99435900	2.70115000	H
C-C_R	0	1.83193000	2.19474900	0.42950300	H
C-C_R	0	1.61433600	1.98912400	-0.91795700	H
C-C_R	0	1.70926600	0.70694400	-1.51331500	H
C-C_R	0	2.21525400	-0.33702900	-0.69954600	H
C-C_R	0	2.48133100	-0.18542200	0.64217500	H
C-C_R	0	2.12195800	1.05630000	1.24792100	H
H-H_	0	1.31025600	2.82607100	-1.53582700	H
H-H_	0	2.45563700	-1.27611500	-1.17721500	H
C-C_R	0	1.34032200	0.45156100	-2.87225800	H
C-C_R	0	1.11688300	-0.89026500	-3.30043900	H
C-C_R	0	1.27080200	1.48526500	-3.86279000	H
C-C_R	0	0.88517500	-1.23729000	-4.59533600	H
H-H_	0	1.09973300	-1.65741700	-2.53805300	H
C-C_R	0	1.05313000	1.22856400	-5.18287100	H
H-H_	0	1.46370200	2.50305300	-3.54316300	H
C-C_R	0	0.86174800	-0.17528700	-5.62322200	H
O-O_R	0	1.96496900	1.13938600	2.56000700	H
O-O_R	0	0.71233800	-0.45109500	-6.81998600	H
C-C_3	0	3.15724300	-1.30068600	1.44546400	H
C-C_3	0	4.39001000	-0.72169300	2.15718300	H
C-C_3	0	2.22551500	-1.90994300	2.49850100	H
C-C_3	0	3.60745600	-2.45570600	0.54653000	H
H-H_	0	5.12048900	-0.34591400	1.43515700	H
H-H_	0	4.10546100	0.09559000	2.82352900	H
H-H_	0	4.86270400	-1.50700500	2.75278300	H
H-H_	0	1.33058700	-2.32648600	2.02528300	H
H-H_	0	2.74482700	-2.71153700	3.03174200	H
H-H_	0	1.93187000	-1.15550000	3.22173500	H
H-H_	0	4.08210300	-3.21430100	1.16922000	H
H-H_	0	2.75852100	-2.93517600	0.04756500	H

H-H_	0	4.32331600	-2.13878600	-0.21520600	H
C-C_3	0	1.70157600	3.58441300	1.06062100	H
C-C_3	0	0.45265000	3.66807000	1.95180800	H
H-H_	0	0.59407100	3.14358500	2.89676400	H
H-H_	0	0.22844500	4.71561500	2.16622700	H
H-H_	0	-0.41549700	3.23086800	1.45253300	H
C-C_3	0	2.94940000	3.89547600	1.89974800	H
H-H_	0	2.82540200	4.86807600	2.38504600	H
H-H_	0	3.09304600	3.13915200	2.67120700	H
H-H_	0	3.84238000	3.93862300	1.26793900	H
C-C_3	0	1.58777300	4.68032700	-0.00401000	H
H-H_	0	2.40952100	4.63401600	-0.72529300	H
H-H_	0	0.64128700	4.62935800	-0.54659200	H
H-H_	0	1.62326700	5.65201100	0.49553700	H
C-C_3	0	1.06103700	2.32359400	-6.24838800	H
C-C_3	0	0.80694000	-2.69710300	-5.03445900	H
C-C_3	0	2.21704300	2.08184300	-7.23523200	H
H-H_	0	2.11551000	1.11401700	-7.72278600	H
H-H_	0	2.21657600	2.86330300	-8.00230600	H
H-H_	0	3.17567800	2.12092100	-6.70938000	H
C-C_3	0	1.26049000	3.70499400	-5.62568000	H
H-H_	0	0.46740400	3.94566700	-4.90911800	H
H-H_	0	2.22726100	3.77365400	-5.11754800	H
H-H_	0	1.24044100	4.46358100	-6.41228800	H
C-C_3	0	-0.27513300	2.34129400	-7.00927800	H
H-H_	0	-0.24307200	3.10744900	-7.79083300	H
H-H_	0	-0.48003400	1.37710100	-7.46928400	H
H-H_	0	-1.08975800	2.59442300	-6.32607100	H
C-C_3	0	2.06766900	-2.99241000	-5.86843000	H
H-H_	0	2.13727700	-4.05903700	-6.09815300	H
H-H_	0	2.05031400	-2.42987200	-6.79954900	H
H-H_	0	2.95794500	-2.70917800	-5.30209200	H
C-C_3	0	-0.45085500	-2.99345900	-5.86523900	H
H-H_	0	-0.49607300	-2.36044000	-6.74856200	H
H-H_	0	-0.44139400	-4.04084700	-6.17810500	H
H-H_	0	-1.34682700	-2.83424400	-5.25997900	H
C-C_3	0	0.79595900	-3.64640000	-3.83381100	H
H-H_	0	1.69101800	-3.54796800	-3.21457100	H

H-H_	0	-0.08157600	-3.47577700	-3.20003900	H
H-H_	0	0.76845300	-4.67279000	-4.20445800	H

IM3$_{\textbf{explicit}}$ (ONIOM(B3LYP/6-31G(d, p):UFF))

0 1 0 1 0 1

C-C_3	0	-4.57486200	8.10341000	-4.43242300	L
O-O_3	0	-5.81068600	7.74092500	-3.86413000	L
C-C_3	0	-6.20800100	8.78241200	-3.00073300	L
C-C_3	0	-5.13399900	9.85264600	-2.96261200	L
C-C_3	0	-4.33130000	9.57612700	-4.19303200	L
H-H_	0	-3.77021800	7.50053000	-3.95816200	L
H-H_	0	-4.58577200	7.88897800	-5.52139100	L
H-H_	0	-7.15869200	9.21570000	-3.37938300	L
H-H_	0	-6.38966900	8.37508300	-1.98364700	L
H-H_	0	-5.56606500	10.87692000	-2.97311800	L
H-H_	0	-4.49801500	9.72514000	-2.06149200	L
H-H_	0	-4.72370700	10.17151000	-5.04693300	L
H-H_	0	-3.25498500	9.79619300	-4.04159700	L
C-C_3	0	-7.61968100	5.23137200	-12.03444100	L
O-O_3	0	-7.86698500	6.53435200	-12.51272400	L
C-C_3	0	-6.85570600	7.37588000	-12.00701700	L
C-C_3	0	-6.07460100	6.63583800	-10.94341900	L
C-C_3	0	-6.28940300	5.20325100	-11.31219500	L
H-H_	0	-8.43542200	4.94512700	-11.33551400	L
H-H_	0	-7.61463100	4.51460900	-12.88314900	L
H-H_	0	-6.17890500	7.66499800	-12.84037100	L
H-H_	0	-7.31066700	8.29662000	-11.58361100	L
H-H_	0	-4.99669900	6.90810200	-10.95067100	L
H-H_	0	-6.51263200	6.84405500	-9.94274400	L
H-H_	0	-5.48681000	4.86532000	-12.00439400	L
H-H_	0	-6.32131200	4.54482200	-10.41963700	L
C-C_3	0	7.37832000	-4.56489900	8.37007400	L
O-O_3	0	6.20079300	-4.41216000	7.61623600	L
C-C_3	0	5.29400900	-3.71514100	8.43188200	L
C-C_3	0	6.07776200	-2.83282400	9.37751300	L
C-C_3	0	7.43758100	-3.47457400	9.42692200	L
H-H_	0	7.37796200	-5.56874400	8.84853500	L
H-H_	0	8.25883600	-4.49805600	7.69673400	L

H-H_	0	4.61470200	-3.10486300	7.79868100	L
H-H_	0	4.68527400	-4.44370200	9.01111600	L
H-H_	0	6.15082400	-1.80540000	8.96451000	L
H-H_	0	5.60221100	-2.79588000	10.38186500	L
H-H_	0	8.22931900	-2.73230400	9.18699200	L
H-H_	0	7.63446800	-3.91236600	10.42997500	L
C-C_3	0	-6.69756500	-6.97892800	5.05174700	L
O-O_3	0	-7.41763100	-7.61358100	4.02112400	L
C-C_3	0	-8.76369600	-7.25285300	4.18945600	L
C-C_3	0	-9.00194200	-6.99045400	5.65682500	L
C-C_3	0	-7.65800600	-6.53557300	6.14450400	L
H-H_	0	-5.95135100	-7.69070300	5.46493400	L
H-H_	0	-6.15606300	-6.10351700	4.63344200	L
H-H_	0	-8.97106100	-6.33511700	3.59950000	L
H-H_	0	-9.42178500	-8.07065500	3.82865300	L
H-H_	0	-9.78346900	-6.21698300	5.81351200	L
H-H_	0	-9.29506700	-7.93440300	6.16710400	L
H-H_	0	-7.64132300	-5.42978700	6.24782500	L
H-H_	0	-7.40869200	-7.00517800	7.12057800	L
C-C_3	0	-3.98574300	2.93389800	5.95820000	L
O-O_3	0	-4.07062000	1.63097100	6.48575400	L
C-C_3	0	-4.51655700	0.78074100	5.45649000	L
C-C_3	0	-4.40480800	1.49976300	4.13074800	L
C-C_3	0	-4.48094800	2.93832400	4.52714500	L
H-H_	0	-4.58866700	3.62582000	6.57935100	L
H-H_	0	-2.92827900	3.26528500	5.99276400	L
H-H_	0	-3.90794300	-0.14694200	5.44692400	L
H-H_	0	-5.57533100	0.50809500	5.65136200	L
H-H_	0	-3.42202600	1.28619000	3.65860000	L
H-H_	0	-5.22444700	1.21675600	3.43681700	L
H-H_	0	-3.85300300	3.58256800	3.87639100	L
H-H_	0	-5.53638000	3.28107800	4.49134400	L
C-C_3	0	-0.07910400	-5.54837500	-6.30355900	L
O-O_3	0	-1.01217900	-4.50856500	-6.42788900	L
C-C_3	0	-1.01366300	-3.84785700	-5.19062500	L
C-C_3	0	-0.70752000	-4.86253900	-4.10936500	L
C-C_3	0	-0.05128200	-5.99309100	-4.85432600	L
H-H_	0	-0.37001100	-6.38824600	-6.96671800	L

H-H_	0	0.92299600	-5.17924300	-6.61522800	L
H-H_	0	-0.23600600	-3.05623100	-5.20596100	L
H-H_	0	-2.00082200	-3.37354200	-5.01749700	L
H-H_	0	-0.02756300	-4.43486700	-3.34134300	L
H-H_	0	-1.64813600	-5.20820100	-3.62660400	L
H-H_	0	0.99459600	-6.14392000	-4.51331800	L
H-H_	0	-0.63311000	-6.93156900	-4.72238900	L
C-C_3	0	-6.90618600	-9.02643800	-1.49593600	L
O-O_3	0	-6.89295900	-9.53251500	-0.18207500	L
C-C_3	0	-7.04750100	-10.93103600	-0.25464300	L
C-C_3	0	-7.00948500	-11.37175500	-1.70315100	L
C-C_3	0	-7.33552500	-10.11849900	-2.45019200	L
H-H_	0	-7.59497100	-8.15755800	-1.55448900	L
H-H_	0	-5.88522100	-8.68004300	-1.75739900	L
H-H_	0	-6.23969500	-11.42692700	0.32330900	L
H-H_	0	-8.02213700	-11.20809500	0.20058200	L
H-H_	0	-5.98774800	-11.72042100	-1.97087800	L
H-H_	0	-7.74778500	-12.17697000	-1.90936000	L
H-H_	0	-6.79017100	-10.06153500	-3.41650600	L
H-H_	0	-8.43022300	-10.05882400	-2.62705100	L
C-C_3	0	7.60597000	-5.10435000	-7.89773700	L
O-O_3	0	7.71834100	-4.94898100	-9.29450400	L
C-C_3	0	8.95312500	-4.32438600	-9.55748500	L
C-C_3	0	9.50100200	-3.74674200	-8.27244800	L
C-C_3	0	8.86867000	-4.60284900	-7.22644400	L
H-H_	0	6.73070100	-4.52099400	-7.53979200	L
H-H_	0	7.43711800	-6.17487500	-7.65427200	L
H-H_	0	9.65958200	-5.08055800	-9.96391500	L
H-H_	0	8.81747100	-3.52470000	-10.31548500	L
H-H_	0	10.61052900	-3.80013200	-8.23239700	L
H-H_	0	9.16552100	-2.69464000	-8.15122400	L
H-H_	0	9.53849600	-5.45701700	-6.98468700	L
H-H_	0	8.64387900	-4.02255600	-6.30744600	L
C-C_3	0	9.96527500	3.60010500	-0.54320300	L
O-O_3	0	10.06063500	3.14106700	-1.86847000	L
C-C_3	0	8.74945400	3.07241600	-2.37897600	L
C-C_3	0	7.79563400	3.78583700	-1.43579400	L
C-C_3	0	8.68750800	4.39010700	-0.39639800	L

H-H_	0	9.95972100	2.72627600	0.14377800	L
H-H_	0	10.83975300	4.23809900	-0.30099900	L
H-H_	0	8.72638200	3.54977600	-3.38027800	L
H-H_	0	8.45356900	2.00914000	-2.50090200	L
H-H_	0	7.20480600	4.56383100	-1.96263000	L
H-H_	0	7.11350300	3.05505400	-0.95913200	L
H-H_	0	8.86968400	5.46097200	-0.62741500	L
H-H_	0	8.26203700	4.29511700	0.62566600	L
C-C_3	0	9.32454600	-5.43215200	-2.59502200	L
O-O_3	0	9.24095900	-5.39012700	-3.99652100	L
C-C_3	0	8.30019300	-6.36228800	-4.39157900	L
C-C_3	0	7.86859200	-7.18192100	-3.19184000	L
C-C_3	0	8.82951000	-6.77723300	-2.12090000	L
H-H_	0	10.37460800	-5.27133200	-2.27594000	L
H-H_	0	8.69849800	-4.61793500	-2.17100600	L
H-H_	0	7.42471100	-5.85986200	-4.84976200	L
H-H_	0	8.76213700	-7.02358900	-5.15482500	L
H-H_	0	6.83183700	-6.90918800	-2.89738600	L
H-H_	0	7.92888800	-8.27249000	-3.39786000	L
H-H_	0	8.33083100	-6.70528800	-1.13554400	L
H-H_	0	9.67216400	-7.50058100	-2.06738000	L
C-C_3	0	-5.06144400	-9.64669100	2.85624900	L
O-O_3	0	-4.05438200	-9.99840600	1.93498100	L
C-C_3	0	-3.62252900	-11.30004700	2.25163900	L
C-C_3	0	-4.08477500	-11.64572500	3.64926800	L
C-C_3	0	-5.30127600	-10.79225900	3.81903800	L
H-H_	0	-4.73459700	-8.74617100	3.41426100	L
H-H_	0	-5.99675600	-9.39905900	2.31361900	L
H-H_	0	-4.05873500	-12.01336300	1.51902500	L
H-H_	0	-2.51588000	-11.35781200	2.18061000	L
H-H_	0	-4.31813800	-12.72758300	3.75255200	L
H-H_	0	-3.31137800	-11.35209600	4.39131800	L
H-H_	0	-6.20955000	-11.35946900	3.51659100	L
H-H_	0	-5.41041600	-10.43506000	4.86645500	L
C-C_3	0	-5.29280600	-6.43182900	-6.96224100	L
O-O_3	0	-6.08323600	-7.10686800	-6.01079300	L
C-C_3	0	-5.22047900	-7.86393400	-5.19502000	L
C-C_3	0	-3.79337900	-7.42512400	-5.43509000	L

C-C_3	0	-3.84502800	-6.85281100	-6.81483900	L
H-H_	0	-5.66270100	-6.66063400	-7.98430600	L
H-H_	0	-5.38484700	-5.33902600	-6.79225300	L
H-H_	0	-5.49661100	-7.72761400	-4.12836800	L
H-H_	0	-5.33288800	-8.93943400	-5.45298200	L
H-H_	0	-3.50214100	-6.63914200	-4.70513500	L
H-H_	0	-3.08944300	-8.27937700	-5.36677500	L
H-H_	0	-3.15402600	-5.99169800	-6.92749400	L
H-H_	0	-3.59660800	-7.63708100	-7.56387300	L
C-C_3	0	5.84197500	7.65516000	5.81688800	L
O-O_3	0	7.18878100	7.46316400	5.45578600	L
C-C_3	0	7.97640400	8.28140100	6.29067500	L
C-C_3	0	7.07893400	9.09264900	7.20407900	L
C-C_3	0	5.78427300	8.34718800	7.15983600	L
H-H_	0	5.34543400	8.28079600	5.04496800	L
H-H_	0	5.32396300	6.67582400	5.86025700	L
H-H_	0	8.64696600	7.63390000	6.89537700	L
H-H_	0	8.60341100	8.95554500	5.66994900	L
H-H_	0	7.48450400	9.15175200	8.23752500	L
H-H_	0	6.94179500	10.11713900	6.79229600	L
H-H_	0	5.75360000	7.59482800	7.97703600	L
H-H_	0	4.90987200	9.02655900	7.24171800	L
C-C_3	0	-8.47079800	-4.56196200	-7.06198800	L
O-O_3	0	-8.43819900	-4.07496000	-8.38446700	L
C-C_3	0	-9.51014400	-3.17254000	-8.53389000	L
C-C_3	0	-10.09021800	-2.85701600	-7.17223000	L
C-C_3	0	-9.72373200	-4.06469200	-6.37102100	L
H-H_	0	-8.44437000	-5.67190700	-7.07338100	L
H-H_	0	-7.57379400	-4.19276800	-6.52223300	L
H-H_	0	-9.15356200	-2.24554400	-9.02984500	L
H-H_	0	-10.28712900	-3.64121900	-9.17619800	L
H-H_	0	-9.59541000	-1.95470100	-6.75228700	L
H-H_	0	-11.19056800	-2.70353900	-7.21140600	L
H-H_	0	-9.54217600	-3.81613300	-5.30376100	L
H-H_	0	-10.52780000	-4.82925800	-6.44920800	L
C-C_3	0	-5.46678900	1.72870900	-11.95681100	L
O-O_3	0	-4.63391600	2.18443100	-10.92074200	L
C-C_3	0	-3.75902100	1.12810400	-10.59766300	L

C-C_3	0	-4.23003200	-0.15093100	-11.27044400	L
C-C_3	0	-5.54939600	0.22370000	-11.87395800	L
H-H_	0	-6.47683000	2.17797200	-11.85567500	L
H-H_	0	-5.03540600	2.03823300	-12.93435400	L
H-H_	0	-2.73338700	1.38640200	-10.93425200	L
H-H_	0	-3.74110300	0.99537800	-9.49564500	L
H-H_	0	-3.51463900	-0.45166900	-12.06761200	L
H-H_	0	-4.34393100	-0.97960700	-10.54148400	L
H-H_	0	-5.69487000	-0.23700700	-12.87506600	L
H-H_	0	-6.37914900	-0.07269900	-11.19468600	L
C-C_3	0	-9.50332500	-3.30446900	4.45027200	L
O-O_3	0	-10.15380400	-3.41389000	5.69564000	L
C-C_3	0	-10.68921000	-2.14923900	6.01158100	L
C-C_3	0	-10.58363500	-1.24556100	4.80345900	L
C-C_3	0	-9.44895000	-1.84796000	4.03877700	L
H-H_	0	-8.48129200	-3.72921700	4.52501700	L
H-H_	0	-10.07208100	-3.88973200	3.69684200	L
H-H_	0	-11.74832600	-2.25589000	6.32708900	L
H-H_	0	-10.11255400	-1.71638400	6.85612000	L
H-H_	0	-11.52015600	-1.29761400	4.20668000	L
H-H_	0	-10.38016500	-0.19341700	5.09090600	L
H-H_	0	-9.57278300	-1.71790500	2.94292400	L
H-H_	0	-8.48808500	-1.39410000	4.36728400	L
C-C_3	0	9.90928500	-3.93302700	4.92433200	L
O-O_3	0	8.73159900	-4.69287700	4.77787400	L
C-C_3	0	9.06445000	-6.04088800	5.01213800	L
C-C_3	0	10.45994500	-6.11915000	5.59065300	L
C-C_3	0	11.09361400	-4.86021500	5.09295700	L
H-H_	0	10.05084200	-3.28271100	4.03673000	L
H-H_	0	9.80402400	-3.29098900	5.82224900	L
H-H_	0	8.32638100	-6.49704300	5.70565200	L
H-H_	0	9.03394600	-6.58589100	4.04664100	L
H-H_	0	10.41183800	-6.10802900	6.70226200	L
H-H_	0	11.00342500	-7.02367700	5.24062200	L
H-H_	0	11.83842800	-4.45749200	5.81224200	L
H-H_	0	11.57976300	-5.04105500	4.10893500	L
C-C_3	0	-4.89041800	5.65837600	9.05692400	L
O-O_3	0	-6.03100300	6.27716800	9.60316800	L

C-C_3	0	-6.39362900	5.56177800	10.76173700	L
C-C_3	0	-5.37269400	4.47385200	11.02998500	L
C-C_3	0	-4.69193200	4.30757900	9.70841600	L
H-H_	0	-5.01601800	5.54782200	7.96095300	L
H-H_	0	-4.00824300	6.30452000	9.25007500	L
H-H_	0	-6.45415100	6.25727900	11.62585000	L
H-H_	0	-7.39677700	5.11096700	10.60333300	L
H-H_	0	-4.64060500	4.81940400	11.79310700	L
H-H_	0	-5.85526300	3.53248300	11.36973300	L
H-H_	0	-3.61617100	4.05719500	9.82625800	L
H-H_	0	-5.19696500	3.51714100	9.11202100	L
C-C_3	0	-5.87262700	6.29422200	4.90407200	L
O-O_3	0	-5.89234700	7.67320400	5.16415800	L
C-C_3	0	-6.78304500	7.86743500	6.23599100	L
C-C_3	0	-7.73267600	6.68378500	6.32300000	L
C-C_3	0	-7.21134700	5.71471300	5.30079400	L
H-H_	0	-5.67297300	6.11590600	3.82632900	L
H-H_	0	-5.06000300	5.82462900	5.49656000	L
H-H_	0	-6.20406200	7.96849000	7.17937600	L
H-H_	0	-7.35256400	8.80680700	6.07169300	L
H-H_	0	-7.70536700	6.23429000	7.33979600	L
H-H_	0	-8.77513200	6.98745800	6.08417500	L
H-H_	0	-7.10021700	4.69743300	5.72939800	L
H-H_	0	-7.88810900	5.68144400	4.41989400	L
C-C_3	0	1.15202600	-10.73913300	6.37278100	L
O-O_3	0	0.08837000	-10.11231900	7.04557600	L
C-C_3	0	-0.97712700	-11.03455900	7.09602900	L
C-C_3	0	-0.63926400	-12.25667800	6.25834400	L
C-C_3	0	0.59121300	-11.84518000	5.51093700	L
H-H_	0	1.69362200	-9.99798200	5.75031100	L
H-H_	0	1.85986400	-11.15772700	7.12155700	L
H-H_	0	-1.16361400	-11.32659800	8.15184000	L
H-H_	0	-1.89413600	-10.54808100	6.70264200	L
H-H_	0	-0.41157900	-13.12167800	6.91995000	L
H-H_	0	-1.46483800	-12.52531800	5.56461100	L
H-H_	0	1.30990200	-12.68561500	5.39806400	L
H-H_	0	0.31536900	-11.44502500	4.51063300	L
C-C_3	0	4.80718400	5.97544100	-6.49212600	L

O-O_3	0	5.88835100	6.81283400	-6.82133300	L
C-C_3	0	6.86057300	6.67040800	-5.81071800	L
C-C_3	0	6.33791300	5.76115400	-4.71347500	L
C-C_3	0	4.88044700	5.62853100	-5.02227200	L
H-H_	0	3.85138200	6.49170600	-6.71995600	L
H-H_	0	4.86778500	5.05283200	-7.10856500	L
H-H_	0	7.79395600	6.25641200	-6.24623900	L
H-H_	0	7.09293500	7.67324100	-5.39515800	L
H-H_	0	6.82831900	4.76644200	-4.76757100	L
H-H_	0	6.50379400	6.19932500	-3.70760000	L
H-H_	0	4.51305300	4.60062700	-4.82258900	L
H-H_	0	4.29326900	6.35888500	-4.42458400	L
C-C_3	0	-5.74551700	10.65690900	0.84708400	L
O-O_3	0	-5.68183800	11.26400300	2.11571500	L
C-C_3	0	-5.04159200	10.36310800	2.99127100	L
C-C_3	0	-4.72908700	9.07172300	2.26097100	L
C-C_3	0	-4.82350800	9.46000300	0.82176700	L
H-H_	0	-6.79146200	10.33559800	0.65312400	L
H-H_	0	-5.45310300	11.38737400	0.06421200	L
H-H_	0	-4.10014600	10.82783500	3.35540700	L
H-H_	0	-5.69825300	10.16481000	3.86476500	L
H-H_	0	-3.71953200	8.68431300	2.51601900	L
H-H_	0	-5.49504300	8.30222100	2.49491600	L
H-H_	0	-3.82237900	9.75488200	0.44430800	L
H-H_	0	-5.23694100	8.64242700	0.19755300	L
C-C_3	0	-0.14536700	4.47145600	10.30249700	L
O-O_3	0	-0.63209700	3.21756900	9.89739700	L
C-C_3	0	-0.90403900	3.31573600	8.52016800	L
C-C_3	0	-1.03922000	4.77891800	8.13282800	L
C-C_3	0	-0.73741800	5.52684800	9.40007600	L
H-H_	0	-0.42770500	4.66393800	11.35812600	L
H-H_	0	0.96323000	4.47902500	10.22888300	L
H-H_	0	-0.07683800	2.84162700	7.94977400	L
H-H_	0	-1.84509500	2.77158800	8.29543200	L
H-H_	0	-0.30234200	5.03759700	7.34255500	L
H-H_	0	-2.06506300	5.00897400	7.77032500	L
H-H_	0	-0.02233400	6.36063500	9.23018000	L
H-H_	0	-1.67526800	5.92174600	9.84684300	L

C-C_3	0	13.37758300	2.89045300	-1.76140000	L
O-O_3	0	14.68380700	2.86522200	-2.29014300	L
C-C_3	0	15.58016500	2.70035900	-1.21609500	L
C-C_3	0	14.81043100	2.30122800	0.02347100	L
C-C_3	0	13.43727100	2.82534800	-0.24868600	L
H-H_	0	12.86199800	3.81847900	-2.08884100	L
H-H_	0	12.81289100	2.01812300	-2.15499900	L
H-H_	0	16.33159800	1.92311000	-1.47166500	L
H-H_	0	16.10974900	3.66180800	-1.03953700	L
H-H_	0	14.78635400	1.19462400	0.11635100	L
H-H_	0	15.24705300	2.74560200	0.94423200	L
H-H_	0	12.65281700	2.15546900	0.16407400	L
H-H_	0	13.33071000	3.84642100	0.17995700	L
C-C_3	0	10.29959800	-0.65806400	-2.74246500	L
O-O_3	0	10.60778900	0.37259100	-3.65337700	L
C-C_3	0	10.56123000	-0.18045700	-4.94521000	L
C-C_3	0	10.72893300	-1.67829300	-4.83985200	L
C-C_3	0	10.19322700	-1.98246700	-3.47574000	L
H-H_	0	11.10124800	-0.71157000	-1.97655400	L
H-H_	0	9.34166300	-0.42600900	-2.23380500	L
H-H_	0	9.58045300	0.06284000	-5.40704200	L
H-H_	0	11.36726200	0.25768400	-5.57113100	L
H-H_	0	10.16880900	-2.21177900	-5.63598600	L
H-H_	0	11.80565600	-1.94587100	-4.89450300	L
H-H_	0	9.13050000	-2.29684700	-3.54313100	L
H-H_	0	10.78166600	-2.77874100	-2.97335300	L
C-C_3	0	-8.38527800	1.03928800	6.91990500	L
O-O_3	0	-9.47451700	0.85488300	7.79205800	L
C-C_3	0	-9.35077200	1.79004600	8.83908600	L
C-C_3	0	-8.14680700	2.67753700	8.59514800	L
C-C_3	0	-7.34884000	1.91195500	7.58951800	L
H-H_	0	-7.95292900	0.05477900	6.64430800	L
H-H_	0	-8.74786700	1.53128900	5.99599000	L
H-H_	0	-10.27556100	2.40192700	8.90420100	L
H-H_	0	-9.22541200	1.23948500	9.79633000	L
H-H_	0	-8.46656800	3.65322900	8.16706500	L
H-H_	0	-7.57145800	2.84602700	9.52967500	L
H-H_	0	-6.84847700	2.58387500	6.86218200	L

H-H_	0	-6.59129300	1.28244600	8.10264900	L
C-C_3	0	8.79160000	-0.28077800	4.93953100	L
O-O_3	0	8.69122200	0.36917200	6.18528600	L
C-C_3	0	7.57995800	-0.17348800	6.85922000	L
C-C_3	0	7.07474800	-1.38077600	6.10187800	L
C-C_3	0	7.55633200	-1.12370100	4.71237100	L
H-H_	0	8.90733800	0.46849100	4.13360100	L
H-H_	0	9.69420600	-0.92413900	4.94373800	L
H-H_	0	7.86861500	-0.45310400	7.89323700	L
H-H_	0	6.78375000	0.59629100	6.91307100	L
H-H_	0	7.55134700	-2.30186800	6.49830200	L
H-H_	0	5.96902900	-1.47204100	6.15423500	L
H-H_	0	7.78924000	-2.06454300	4.17181400	L
H-H_	0	6.79438900	-0.54549600	4.14863300	L
C-C_3	0	-10.79164900	4.16916900	6.05229800	L
O-O_3	0	-11.28718700	5.18307300	5.20852800	L
C-C_3	0	-12.40397700	4.66520200	4.52166200	L
C-C_3	0	-12.76498100	3.30912300	5.08995400	L
C-C_3	0	-11.49523000	2.86561500	5.74138600	L
H-H_	0	-9.69666000	4.06208200	5.90526000	L
H-H_	0	-10.97859400	4.45795000	7.10916100	L
H-H_	0	-13.25988500	5.36659800	4.61746500	L
H-H_	0	-12.15117500	4.56529200	3.44494500	L
H-H_	0	-13.56644600	3.41741700	5.85396700	L
H-H_	0	-13.08994800	2.60069000	4.29650100	L
H-H_	0	-11.68831400	2.26709400	6.65754800	L
H-H_	0	-10.89166100	2.27284500	5.02394200	L
C-C_3	0	-0.84011100	-8.13059300	9.70768900	L
O-O_3	0	-2.07311300	-7.92605100	10.35950400	L
C-C_3	0	-1.87278700	-6.95596700	11.36228300	L
C-C_3	0	-0.38968400	-6.71968400	11.54072500	L
C-C_3	0	0.17268400	-7.12569800	10.21687400	L
H-H_	0	-0.48837800	-9.16298900	9.92167800	L
H-H_	0	-0.97486000	-8.02480400	8.61012700	L
H-H_	0	-2.36872400	-6.01032100	11.05492300	L
H-H_	0	-2.32868900	-7.30080500	12.31453500	L
H-H_	0	-0.16519000	-5.65833600	11.78055400	L
H-H_	0	0.00735500	-7.38194400	12.34172600	L

H-H_	0	0.21046100	-6.24908300	9.53475400	L
H-H_	0	1.18634500	-7.57058700	10.31829200	L
C-C_3	0	11.48549700	-1.06976400	-10.08506300	L
O-O_3	0	10.48005200	-0.54822900	-10.92072100	L
C-C_3	0	9.43571400	-0.13405500	-10.07779900	L
C-C_3	0	10.02733500	0.26099600	-8.74381800	L
C-C_3	0	11.32342100	-0.49799600	-8.68560600	L
H-H_	0	11.40456300	-2.17814700	-10.06383100	L
H-H_	0	12.48100200	-0.80000000	-10.49743400	L
H-H_	0	8.90035800	0.72476900	-10.53566500	L
H-H_	0	8.71622900	-0.96972100	-9.94762600	L
H-H_	0	10.23093800	1.35326100	-8.73230700	L
H-H_	0	9.34828800	-0.00943800	-7.90613400	L
H-H_	0	12.16603600	0.18258900	-8.43275400	L
H-H_	0	11.27434500	-1.31212100	-7.93090300	L
C-C_3	0	6.25709500	-2.87030400	-4.62138600	L
O-O_3	0	5.56353800	-2.61272900	-3.42839200	L
C-C_3	0	6.06849800	-1.39505000	-2.94178800	L
C-C_3	0	6.51635100	-0.55534000	-4.12055300	L
C-C_3	0	6.63410200	-1.54457100	-5.25115300	L
H-H_	0	7.16764600	-3.46195900	-4.39016400	L
H-H_	0	5.61693400	-3.46208400	-5.30922800	L
H-H_	0	5.27820800	-0.86859800	-2.36796000	L
H-H_	0	6.92596800	-1.59645200	-2.26191500	L
H-H_	0	5.75100300	0.21640500	-4.35280900	L
H-H_	0	7.48748400	-0.05918800	-3.91063700	L
H-H_	0	5.92088700	-1.29098500	-6.06259300	L
H-H_	0	7.66466800	-1.57664700	-5.66451200	L
C-C_3	0	2.66515800	4.70871000	5.40930000	L
O-O_3	0	3.34294100	4.70383800	6.63861200	L
C-C_3	0	2.84916900	5.80424300	7.36400800	L
C-C_3	0	2.25206800	6.81632700	6.39667400	L
C-C_3	0	2.34362800	6.14190200	5.05752000	L
H-H_	0	3.29960200	4.24849600	4.62379600	L
H-H_	0	1.72849900	4.11758900	5.50780100	L
H-H_	0	2.07834100	5.45181300	8.08119100	L
H-H_	0	3.67703700	6.26236300	7.94336500	L
H-H_	0	1.19265600	7.02666100	6.65760200	L

H-H_	0	2.82656000	7.76705000	6.39902700	L
H-H_	0	1.39222500	6.21834700	4.49241800	L
H-H_	0	3.17310500	6.58156600	4.46118400	L
C-C_3	0	1.68315000	-2.17926100	9.44435100	L
O-O_3	0	1.49860400	-1.01839200	10.21900900	L
C-C_3	0	0.29937100	-1.19553000	10.92588000	L
C-C_3	0	0.10872300	-2.67480100	11.16613100	L
C-C_3	0	0.84514300	-3.30953000	10.02177200	L
H-H_	0	1.38787700	-1.96938800	8.39364500	L
H-H_	0	2.75773000	-2.45642200	9.45628000	L
H-H_	0	0.34903800	-0.65078700	11.89113500	L
H-H_	0	-0.54280600	-0.78698900	10.32580400	L
H-H_	0	0.58005600	-2.96501600	12.13104000	L
H-H_	0	-0.96633800	-2.95433300	11.17502200	L
H-H_	0	1.48838400	-4.14368600	10.37774400	L
H-H_	0	0.12725200	-3.69193500	9.26475100	L
C-C_3	0	6.36050800	2.08384500	-6.69889000	L
O-O_3	0	7.48659400	2.18033100	-5.85900100	L
C-C_3	0	8.45799000	2.94626900	-6.53609600	L
C-C_3	0	7.91490700	3.38444700	-7.88394100	L
C-C_3	0	6.75951200	2.46242200	-8.10589500	L
H-H_	0	5.95889500	1.05106000	-6.68082700	L
H-H_	0	5.57469200	2.77263600	-6.32496700	L
H-H_	0	8.73648000	3.82946700	-5.92407900	L
H-H_	0	9.36798300	2.32649900	-6.67359500	L
H-H_	0	7.55255300	4.43357500	-7.82966800	L
H-H_	0	8.67489700	3.29344000	-8.68946900	L
H-H_	0	5.92871300	2.95891900	-8.65285000	L
H-H_	0	7.09625800	1.55957800	-8.66134000	L
C-C_3	0	-1.93015200	8.03318500	-1.06610500	L
O-O_3	0	-1.54971000	9.38579600	-1.08713100	L
C-C_3	0	-0.58366300	9.52343600	-2.09451700	L
C-C_3	0	0.10848300	8.19304500	-2.29497800	L
C-C_3	0	-0.86240200	7.19103400	-1.73776400	L
H-H_	0	-2.06110600	7.71254600	-0.01175800	L
H-H_	0	-2.89793000	7.92042000	-1.59710900	L
H-H_	0	-1.08014200	9.84910900	-3.03062600	L
H-H_	0	0.14859500	10.30015500	-1.79564600	L

H-H_	0	0.32224500	8.00532500	-3.36870100	L
H-H_	0	1.05907200	8.17001700	-1.72109800	L
H-H_	0	-1.30837700	6.57342300	-2.54583000	L
H-H_	0	-0.36638500	6.52218500	-1.00273400	L
C-C_3	0	-5.02526900	-3.13260500	5.25378900	L
O-O_3	0	-3.81514000	-3.78520900	4.94387000	L
C-C_3	0	-3.61901700	-3.65546400	3.55891800	L
C-C_3	0	-4.39571600	-2.45537000	3.08426400	L
C-C_3	0	-5.56590400	-2.44740300	4.01301400	L
H-H_	0	-5.75726500	-3.87440100	5.63018800	L
H-H_	0	-4.83433000	-2.38319500	6.04990100	L
H-H_	0	-2.54035400	-3.54199900	3.33233000	L
H-H_	0	-3.99017600	-4.57340300	3.05575100	L
H-H_	0	-3.79036000	-1.53096100	3.21379500	L
H-H_	0	-4.71096300	-2.56468000	2.02934700	L
H-H_	0	-5.92394600	-1.41797300	4.21935500	L
H-H_	0	-6.39275300	-3.04891600	3.57840100	L
C-C_3	0	-12.49589900	4.52476200	-1.62637300	L
O-O_3	0	-12.04408100	5.57201900	-0.79854700	L
C-C_3	0	-11.68341900	6.65198100	-1.62950300	L
C-C_3	0	-12.12630800	6.36698900	-3.04832500	L
C-C_3	0	-12.23099400	4.87617300	-3.07537500	L
H-H_	0	-11.97466800	3.58363700	-1.35496300	L
H-H_	0	-13.58634200	4.38372600	-1.46410000	L
H-H_	0	-12.15612400	7.58537600	-1.25678800	L
H-H_	0	-10.57969100	6.77861200	-1.59674700	L
H-H_	0	-13.12455400	6.82145800	-3.23461100	L
H-H_	0	-11.39284800	6.74488100	-3.79270500	L
H-H_	0	-13.04846600	4.52932500	-3.74403800	L
H-H_	0	-11.26548500	4.43646400	-3.40291200	L
C-C_3	0	-4.88811600	5.50660800	-7.68095800	L
O-O_3	0	-4.93814500	5.24241700	-6.29896800	L
C-C_3	0	-4.17164400	4.08612200	-6.05538000	L
C-C_3	0	-3.72980700	3.48185300	-7.37151700	L
C-C_3	0	-3.83981300	4.62667300	-8.32376200	L
H-H_	0	-5.88180900	5.28721100	-8.11933400	L
H-H_	0	-4.65578100	6.57898900	-7.85271200	L
H-H_	0	-3.28434300	4.36948300	-5.45035500	L

H-H_	0	-4.77432500	3.35277800	-5.48087500	L
H-H_	0	-2.69353400	3.08509300	-7.32120900	L
H-H_	0	-4.42978300	2.67346300	-7.67191400	L
H-H_	0	-2.87247800	5.16668400	-8.38281100	L
H-H_	0	-4.14300900	4.29283900	-9.33665500	L
C-C_3	0	-10.86873400	-9.58152900	-4.46683600	L
O-O_3	0	-9.79930100	-9.95604800	-5.30576900	L
C-C_3	0	-9.01483400	-8.80827700	-5.53729900	L
C-C_3	0	-9.43784600	-7.71355500	-4.58275700	L
C-C_3	0	-10.85136300	-8.08083200	-4.26920000	L
H-H_	0	-11.83071000	-9.90116700	-4.92113500	L
H-H_	0	-10.75578400	-10.09417200	-3.48749400	L
H-H_	0	-7.94317000	-9.05532100	-5.39604200	L
H-H_	0	-9.16566900	-8.47360900	-6.58676100	L
H-H_	0	-8.81899900	-7.74886400	-3.66086900	L
H-H_	0	-9.36614500	-6.70604800	-5.04147300	L
H-H_	0	-11.13083200	-7.79210300	-3.23523400	L
H-H_	0	-11.53826700	-7.59904300	-4.99882000	L
C-C_3	0	-1.32278100	-7.90191900	2.77091300	L
O-O_3	0	-2.42996200	-7.04987700	2.92414000	L
C-C_3	0	-2.60215800	-6.88087600	4.30748000	L
C-C_3	0	-2.12067700	-8.13343100	5.00710100	L
C-C_3	0	-1.19190900	-8.76868000	4.00906500	L
H-H_	0	-1.46575300	-8.53449300	1.86991800	L
H-H_	0	-0.40554800	-7.28740800	2.63823600	L
H-H_	0	-2.01601400	-5.99890200	4.64386100	L
H-H_	0	-3.67309100	-6.69905700	4.53128400	L
H-H_	0	-1.59863700	-7.89060400	5.95738900	L
H-H_	0	-2.97984400	-8.80673600	5.21856500	L
H-H_	0	-0.14450900	-8.77026900	4.37560100	L
H-H_	0	-1.50191800	-9.81209700	3.79538800	L
C-C_3	0	2.12562300	-7.20744100	6.94772600	L
O-O_3	0	1.30145300	-7.12351600	5.81280700	L
C-C_3	0	0.74133000	-5.83646600	5.82877400	L
C-C_3	0	1.72049000	-4.89736700	6.49676200	L
C-C_3	0	2.57222900	-5.80927300	7.33707200	L
H-H_	0	1.55838000	-7.68197100	7.77526300	L
H-H_	0	3.00488200	-7.84660200	6.72076200	L

H-H_	0	0.52946100	-5.50749000	4.78946300	L
H-H_	0	-0.21123400	-5.85985600	6.39975200	L
H-H_	0	2.34098200	-4.38066800	5.73347000	L
H-H_	0	1.19150500	-4.14133300	7.11269000	L
H-H_	0	3.64699700	-5.66671600	7.09877700	L
H-H_	0	2.40485300	-5.62853400	8.42068100	L
C-C_3	0	1.26730800	-10.67280300	1.32214200	L
O-O_3	0	1.94849700	-11.68281200	2.03184800	L
C-C_3	0	3.00788900	-11.06995000	2.73162600	L
C-C_3	0	2.85672700	-9.56734200	2.64678000	L
C-C_3	0	2.06335200	-9.38812800	1.39574600	L
H-H_	0	1.12759800	-10.98272700	0.26598900	L
H-H_	0	0.26681600	-10.52422200	1.78299100	L
H-H_	0	3.00523500	-11.40173200	3.79089100	L
H-H_	0	3.96948200	-11.37888800	2.26823200	L
H-H_	0	2.28099600	-9.18937600	3.51936400	L
H-H_	0	3.83737200	-9.05433000	2.58666200	L
H-H_	0	1.40728000	-8.49377900	1.43936300	L
H-H_	0	2.75300400	-9.31284000	0.52673900	L
C-C_3	0	0.44646200	2.15233300	-7.45036800	L
O-O_3	0	1.18366800	2.78471200	-6.43743000	L
C-C_3	0	2.32384900	3.32348500	-7.05769300	L
C-C_3	0	2.63242700	2.52420000	-8.31053100	L
C-C_3	0	1.41103600	1.67029000	-8.51380900	L
H-H_	0	-0.12367900	1.30168400	-7.02267000	L
H-H_	0	-0.27603200	2.87717300	-7.88119900	L
H-H_	0	2.13486600	4.38768600	-7.31523300	L
H-H_	0	3.17688000	3.27724900	-6.35184500	L
H-H_	0	2.79813300	3.19989400	-9.17797400	L
H-H_	0	3.53272000	1.88929600	-8.15885500	L
H-H_	0	0.98508800	1.79609100	-9.53253900	L
H-H_	0	1.66201900	0.60170100	-8.34596700	L
C-C_3	0	-3.11507900	9.25557500	5.98989100	L
O-O_3	0	-2.07508000	10.19920700	6.09740500	L
C-C_3	0	-1.23073400	9.79212600	7.14987500	L
C-C_3	0	-1.71560500	8.47222200	7.71309300	L
C-C_3	0	-3.12330400	8.37740000	7.21978700	L
H-H_	0	-4.08699600	9.78208400	5.88651200	L

H-H_	0	-2.94977200	8.63456800	5.08308200	L
H-H_	0	-0.18951200	9.69172400	6.77674700	L
H-H_	0	-1.24706300	10.57061900	7.94269300	L
H-H_	0	-1.11168100	7.63971100	7.29601000	L
H-H_	0	-1.66762300	8.45354900	8.82349900	L
H-H_	0	-3.40505400	7.33120500	6.97462800	L
H-H_	0	-3.82208900	8.79159600	7.97981100	L
C-C_3	0	-0.63969400	10.17058800	2.99775400	L
O-O_3	0	-0.65334500	9.76394800	1.65033300	L
C-C_3	0	0.49436000	8.97903900	1.42717300	L
C-C_3	0	1.39734000	9.04829800	2.63777600	L
C-C_3	0	0.45913800	9.43543800	3.73415800	L
H-H_	0	-0.45794400	11.26613500	3.04139100	L
H-H_	0	-1.62644800	9.95944800	3.45570000	L
H-H_	0	0.18132000	7.93083900	1.24452800	L
H-H_	0	1.02813800	9.34280000	0.52637200	L
H-H_	0	1.89353500	8.07578000	2.83858200	L
H-H_	0	2.16280100	9.84077300	2.49216800	L
H-H_	0	0.05147400	8.52753200	4.22632500	L
H-H_	0	0.94984400	10.07965600	4.49078200	L
C-C_3	0	5.34391400	-8.06165000	-5.98289800	L
O-O_3	0	5.18396400	-6.66491600	-5.92102000	L
C-C_3	0	4.31001500	-6.37184900	-4.85632000	L
C-C_3	0	3.83235300	-7.65707500	-4.21092600	L
C-C_3	0	4.77589100	-8.69387000	-4.73180300	L
H-H_	0	4.81159100	-8.44474000	-6.87866400	L
H-H_	0	6.42212300	-8.30838700	-6.08464500	L
H-H_	0	4.84988600	-5.74955700	-4.11137100	L
H-H_	0	3.44649100	-5.78929900	-5.24010500	L
H-H_	0	3.86817700	-7.59667800	-3.10150000	L
H-H_	0	2.79782900	-7.88764900	-4.54121300	L
H-H_	0	5.58477800	-8.87856100	-3.99379300	L
H-H_	0	4.25336800	-9.64824900	-4.95819000	L
C-C_3	0	0.13505400	4.79175400	-10.51008900	L
O-O_3	0	-0.34074300	6.11281900	-10.62367400	L
C-C_3	0	-1.61184300	6.06222700	-11.23055100	L
C-C_3	0	-1.90081600	4.64483500	-11.68075000	L
C-C_3	0	-0.98723300	3.82628600	-10.82545900	L

H-H_	0	0.97067900	4.64816300	-11.22891300	L
H-H_	0	0.52234300	4.62036100	-9.48455200	L
H-H_	0	-2.37487800	6.38995900	-10.49229700	L
H-H_	0	-1.64069600	6.75756000	-12.09626300	L
H-H_	0	-2.96661800	4.37057300	-11.53094100	L
H-H_	0	-1.62787000	4.52275500	-12.75233900	L
H-H_	0	-1.50923000	3.52722100	-9.89081400	L
H-H_	0	-0.61920800	2.92270100	-11.35826800	L
C-C_3	0	12.22899300	-1.01532500	3.16774000	L
O-O_3	0	12.92998100	-2.10649800	3.71985300	L
C-C_3	0	14.04613200	-2.35882500	2.89987500	L
C-C_3	0	13.88577500	-1.61100700	1.59490100	L
C-C_3	0	12.97763300	-0.48203400	1.96322400	L
H-H_	0	11.22080200	-1.35894800	2.86130100	L
H-H_	0	12.10963000	-0.22155900	3.93452100	L
H-H_	0	14.96288500	-2.01275100	3.42505500	L
H-H_	0	14.13448800	-3.45021600	2.71335200	L
H-H_	0	14.86070100	-1.24822900	1.20336500	L
H-H_	0	13.39447100	-2.26245900	0.84048000	L
H-H_	0	13.57655000	0.40926000	2.25343800	L
H-H_	0	12.28987800	-0.22095100	1.12981700	L
C-C_3	0	-10.44507500	7.15883000	1.74575700	L
O-O_3	0	-9.33114200	6.78569900	0.96792500	L
C-C_3	0	-8.19208500	7.39248000	1.53288400	L
C-C_3	0	-8.62526500	8.38838200	2.58647500	L
C-C_3	0	-9.97089000	7.87762500	2.99035100	L
H-H_	0	-11.09096700	7.83497400	1.14536100	L
H-H_	0	-11.03260500	6.25659300	2.01499000	L
H-H_	0	-7.56083200	6.60733300	2.00014400	L
H-H_	0	-7.60341400	7.89435800	0.73691700	L
H-H_	0	-7.92056400	8.42071400	3.44380000	L
H-H_	0	-8.72115300	9.40126000	2.13677900	L
H-H_	0	-9.86874000	7.15499400	3.82845500	L
H-H_	0	-10.65691300	8.70223300	3.28173400	L
C-C_3	0	3.25851900	9.32445800	-4.72917200	L
O-O_3	0	4.61781600	9.04353400	-4.95763400	L
C-C_3	0	5.32969500	9.43312000	-3.80386700	L
C-C_3	0	4.37390700	10.03623000	-2.79357600	L

C-C_3	0	3.15528600	10.32782100	-3.60730700	L
H-H_	0	2.79583900	9.72868700	-5.65276100	L
H-H_	0	2.73542500	8.38465000	-4.44773900	L
H-H_	0	5.84637300	8.55401600	-3.36728800	L
H-H_	0	6.09716800	10.18263200	-4.09328500	L
H-H_	0	4.12933800	9.29205400	-2.00467000	L
H-H_	0	4.79113800	10.95514900	-2.32865200	L
H-H_	0	2.22462200	10.19366400	-3.02053100	L
H-H_	0	3.20719000	11.36103300	-4.01597300	L
C-C_3	0	-1.97594200	-10.99705400	-1.16954400	L
O-O_3	0	-1.12934000	-10.69471100	-2.24883900	L
C-C_3	0	-1.94162000	-10.14919300	-3.26078600	L
C-C_3	0	-3.24900600	-9.66446100	-2.65373200	L
C-C_3	0	-3.15188400	-10.04974400	-1.20601800	L
H-H_	0	-2.32573300	-12.04917500	-1.26041300	L
H-H_	0	-1.42136200	-10.88743400	-0.21435700	L
H-H_	0	-1.40451900	-9.30567100	-3.74369700	L
H-H_	0	-2.13671600	-10.92492600	-4.03289500	L
H-H_	0	-3.35984400	-8.56382600	-2.75858600	L
H-H_	0	-4.11209700	-10.17134500	-3.13807500	L
H-H_	0	-2.93786700	-9.15466000	-0.58286700	L
H-H_	0	-4.07945200	-10.54116700	-0.84567400	L
C-C_3	0	11.40567500	-3.01600900	8.63573700	L
O-O_3	0	12.75706900	-3.25551700	8.31251400	L
C-C_3	0	13.21434400	-2.16795500	7.54228800	L
C-C_3	0	12.03069700	-1.34057200	7.09380500	L
C-C_3	0	10.99409400	-1.64966700	8.12571400	L
H-H_	0	10.77917000	-3.80191300	8.16189500	L
H-H_	0	11.26787800	-3.07577300	9.73639300	L
H-H_	0	13.89567500	-1.54772600	8.16445500	L
H-H_	0	13.77978900	-2.54284900	6.66242100	L
H-H_	0	12.26997400	-0.25559700	7.05597500	L
H-H_	0	11.68767700	-1.68664000	6.09660200	L
H-H_	0	11.03979700	-0.90281200	8.94894200	L
H-H_	0	9.97520700	-1.66716100	7.68791400	L
C-C_3	0	8.07208100	2.88960900	9.06229800	L
O-O_3	0	8.97004100	3.31488400	8.06906100	L
C-C_3	0	8.59980000	4.63118700	7.72912600	L

C-C_3	0	7.18363200	4.90161900	8.21637800	L
C-C_3	0	6.76142200	3.60947800	8.85130300	L
H-H_	0	7.92932000	1.79172200	8.99957100	L
H-H_	0	8.48922300	3.13582900	10.06398000	L
H-H_	0	9.31261100	5.34506600	8.19599500	L
H-H_	0	8.65889500	4.75421300	6.62738800	L
H-H_	0	7.18553500	5.72069500	8.96873600	L
H-H_	0	6.50677500	5.16825100	7.37831900	L
H-H_	0	6.22243600	3.77262700	9.80968000	L
H-H_	0	6.11993300	3.03159900	8.15077900	L
C-C_3	0	6.14090000	-5.74647300	0.85997800	L
O-O_3	0	6.48889300	-4.41057900	0.58012700	L
C-C_3	0	5.77493400	-4.02834000	-0.56774200	L
C-C_3	0	4.57351200	-4.92838100	-0.71097200	L
C-C_3	0	5.04086500	-6.20320300	-0.08309300	L
H-H_	0	7.03773900	-6.39007500	0.77049400	L
H-H_	0	5.78549900	-5.80851700	1.90362400	L
H-H_	0	5.45300200	-2.97096200	-0.47752900	L
H-H_	0	6.43612500	-4.13362100	-1.45386500	L
H-H_	0	3.72375900	-4.50696400	-0.13293400	L
H-H_	0	4.28163800	-5.06627300	-1.77412900	L
H-H_	0	4.21909300	-6.72087900	0.45811000	L
H-H_	0	5.45571100	-6.87742300	-0.86223900	L
C-C_3	0	-3.99310000	-2.50164800	-7.74870800	L
O-O_3	0	-5.35268800	-2.79216000	-7.94672600	L
C-C_3	0	-6.04054100	-1.57127500	-7.83375100	L
C-C_3	0	-5.21671300	-0.60954900	-6.99674000	L
C-C_3	0	-3.88509000	-1.28823700	-6.85189300	L
H-H_	0	-3.48943100	-3.37444900	-7.28434200	L
H-H_	0	-3.51102700	-2.30131100	-8.72923900	L
H-H_	0	-6.21133000	-1.15345500	-8.84888100	L
H-H_	0	-7.02651600	-1.74684600	-7.35645300	L
H-H_	0	-5.10756500	0.36479200	-7.51471500	L
H-H_	0	-5.68484500	-0.44496900	-6.00207400	L
H-H_	0	-3.05378800	-0.62194800	-7.16723100	L
H-H_	0	-3.73055600	-1.60790200	-5.80014700	L
C-C_3	0	-10.10612200	-0.51047200	-3.76281200	L
O-O_3	0	-9.25202400	0.60433800	-3.74286100	L

C-C_3	0	-10.07717000	1.72607600	-3.55528400	L
C-C_3	0	-11.44871300	1.42610200	-4.12644300	L
C-C_3	0	-11.46318600	-0.07071500	-4.27840100	L
H-H_	0	-9.67463100	-1.29679200	-4.41551100	L
H-H_	0	-10.19754100	-0.92422100	-2.73530100	L
H-H_	0	-10.15312500	1.94925600	-2.47041200	L
H-H_	0	-9.62964100	2.60517400	-4.06093900	L
H-H_	0	-12.24897500	1.77112900	-3.43688300	L
H-H_	0	-11.57867400	1.92081500	-5.11372600	L
H-H_	0	-12.28303400	-0.52481900	-3.68093900	L
H-H_	0	-11.57947500	-0.35170000	-5.34850200	L
C-C_3	0	-5.45499400	4.13526900	0.98330800	L
O-O_3	0	-5.71713900	5.42090700	0.47371200	L
C-C_3	0	-4.52976500	5.88626600	-0.11082400	L
C-C_3	0	-3.67303700	4.70023000	-0.47698300	L
C-C_3	0	-4.08088800	3.67093200	0.53275000	L
H-H_	0	-5.51783900	4.16029900	2.09027300	L
H-H_	0	-6.22965900	3.44199400	0.60401300	L
H-H_	0	-4.76813800	6.48683100	-1.01321400	L
H-H_	0	-3.99830400	6.52576700	0.62489200	L
H-H_	0	-3.92445100	4.35737000	-1.50237500	L
H-H_	0	-2.59115300	4.94146600	-0.42097900	L
H-H_	0	-4.11446800	2.65313500	0.08708100	L
H-H_	0	-3.37659800	3.67676900	1.39244400	L
C-C_3	0	4.44971600	0.63359100	9.28021000	L
O-O_3	0	3.99460700	1.75575300	9.99304800	L
C-C_3	0	2.78230500	2.16531400	9.40349000	L
C-C_3	0	2.55872200	1.41627700	8.10122900	L
C-C_3	0	3.82833900	0.64475600	7.90317000	L
H-H_	0	5.55682800	0.66149300	9.20674300	L
H-H_	0	4.15617600	-0.28784500	9.82770600	L
H-H_	0	1.94574300	1.97214400	10.10890100	L
H-H_	0	2.83454200	3.25642800	9.20629300	L
H-H_	0	1.69301200	0.72486300	8.19234100	L
H-H_	0	2.37677000	2.11801400	7.25962400	L
H-H_	0	3.63734700	-0.38707100	7.53699700	L
H-H_	0	4.48792600	1.18427400	7.18996900	L
C-C_3	0	-6.83979300	3.20314300	-2.56436200	L

O-O_3	0	-7.88238700	3.17237400	-1.61758100	L
C-C_3	0	-8.44786300	4.45804400	-1.56649500	L
C-C_3	0	-8.08426500	5.20168400	-2.82817600	L
C-C_3	0	-6.79018700	4.56652100	-3.22924100	L
H-H_	0	-5.87987600	2.98272100	-2.05545100	L
H-H_	0	-7.02024100	2.41436300	-3.32557300	L
H-H_	0	-9.54811200	4.37688200	-1.46498900	L
H-H_	0	-8.05185900	4.99486600	-0.67976800	L
H-H_	0	-8.85357200	5.01938100	-3.60834200	L
H-H_	0	-7.97788000	6.29269500	-2.64736700	L
H-H_	0	-6.70690600	4.48428500	-4.33276000	L
H-H_	0	-5.93512600	5.15493200	-2.83254900	L
C-C_3	0	1.16825900	-5.84268200	-0.65841800	L
O-O_3	0	1.70889600	-6.55472900	-1.74115300	L
C-C_3	0	0.96243800	-7.74351500	-1.84477700	L
C-C_3	0	-0.38257300	-7.55073100	-1.16638400	L
C-C_3	0	-0.29749500	-6.18210200	-0.55695000	L
H-H_	0	1.69582800	-6.13649400	0.27525300	L
H-H_	0	1.29891600	-4.75344000	-0.81862500	L
H-H_	0	0.82084600	-8.00303400	-2.91370000	L
H-H_	0	1.52339900	-8.57147300	-1.36570100	L
H-H_	0	-1.21145500	-7.59346900	-1.90396900	L
H-H_	0	-0.53394600	-8.32345700	-0.38302100	L
H-H_	0	-0.89423400	-5.46600200	-1.15886300	L
H-H_	0	-0.64924400	-6.16596500	0.49552200	L
C-C_3	0	6.37557400	3.10442400	5.06006400	L
O-O_3	0	6.73380600	4.42734600	4.74965100	L
C-C_3	0	6.29260800	4.68494900	3.43770800	L
C-C_3	0	5.80881100	3.39670500	2.79583800	L
C-C_3	0	6.20311800	2.33642400	3.77294000	L
H-H_	0	7.16788300	2.64366900	5.68356200	L
H-H_	0	5.42530400	3.10772100	5.63582200	L
H-H_	0	5.47647400	5.43696400	3.46733400	L
H-H_	0	7.13594600	5.10609300	2.85079000	L
H-H_	0	4.70445800	3.41637000	2.67302100	L
H-H_	0	6.29475700	3.22764900	1.81348900	L
H-H_	0	5.43749100	1.53909900	3.85747300	L
H-H_	0	7.16758200	1.88531100	3.46824100	L

C-C_3	0	-2.31407700	2.88450600	13.03275000	L
O-O_3	0	-1.58377900	3.79281500	13.82563000	L
C-C_3	0	-0.34874600	3.19320000	14.13916200	L
C-C_3	0	-0.13082500	1.99674900	13.24005700	L
C-C_3	0	-1.52232600	1.60352900	12.85702900	L
H-H_	0	-3.29265200	2.67262600	13.51426600	L
H-H_	0	-2.50544200	3.34813200	12.04210200	L
H-H_	0	0.47051800	3.93140600	14.00560300	L
H-H_	0	-0.36571500	2.86994900	15.20286700	L
H-H_	0	0.44514200	2.29630200	12.33725300	L
H-H_	0	0.40020100	1.17554400	13.76868700	L
H-H_	0	-1.56709300	1.23257000	11.81086200	L
H-H_	0	-1.90246900	0.82186200	13.55130600	L
C-C_3	0	3.23029000	13.02941800	-0.80862600	L
O-O_3	0	2.58507900	14.20756200	-0.39597900	L
C-C_3	0	1.30686800	13.81863700	0.03929700	L
C-C_3	0	1.38468500	12.39555200	0.55676700	L
C-C_3	0	2.67577400	11.86765300	-0.00734300	L
H-H_	0	3.04612200	12.87428300	-1.89410900	L
H-H_	0	4.32485200	13.12547500	-0.64567900	L
H-H_	0	0.96053100	14.50447500	0.84138800	L
H-H_	0	0.59431600	13.88278600	-0.81277800	L
H-H_	0	1.41353700	12.38649800	1.66816300	L
H-H_	0	0.51724600	11.80040000	0.20306100	L
H-H_	0	3.37502300	11.59392900	0.81188300	L
H-H_	0	2.50219900	10.98220900	-0.65465400	L
C-C_3	0	-1.69287200	5.83486300	4.60779000	L
O-O_3	0	-0.99690500	4.61654700	4.74545900	L
C-C_3	0	-0.70392400	4.15139100	3.45144300	L
C-C_3	0	-0.82018500	5.30434000	2.48329700	L
C-C_3	0	-1.85039400	6.16919700	3.13663600	L
H-H_	0	-2.68456700	5.75518600	5.09752600	L
H-H_	0	-1.11417900	6.63237200	5.11598300	L
H-H_	0	0.31996500	3.72284700	3.42559200	L
H-H_	0	-1.43473800	3.35833800	3.18467300	L
H-H_	0	0.15118200	5.84059000	2.41903100	L
H-H_	0	-1.13258500	4.97379300	1.47237600	L
H-H_	0	-1.67521300	7.24584600	2.93150100	L

H-H_	0	-2.86720800	5.88113000	2.78930900	L
C-C_3	0	-2.48851300	12.68580100	0.78994900	L
O-O_3	0	-3.52692800	13.55664100	1.17206700	L
C-C_3	0	-3.98619300	14.21036500	0.01021100	L
C-C_3	0	-3.14053400	13.79458200	-1.17743800	L
C-C_3	0	-2.50351500	12.52461800	-0.71401600	L
H-H_	0	-1.51923900	13.11952100	1.11700700	L
H-H_	0	-2.62121600	11.70404100	1.28822100	L
H-H_	0	-5.04666200	13.92713200	-0.16392800	L
H-H_	0	-3.93516300	15.31049600	0.15582400	L
H-H_	0	-3.75656000	13.63681500	-2.08949200	L
H-H_	0	-2.35826400	14.56050400	-1.37639100	L
H-H_	0	-3.13760000	11.65861000	-1.00145000	L
H-H_	0	-1.48191200	12.39727800	-1.13014300	L
C-C_3	0	-7.49414100	2.08779300	-8.82315700	L
O-O_3	0	-7.24068200	2.96834600	-7.75906400	L
C-C_3	0	-7.61780000	2.30056500	-6.58128300	L
C-C_3	0	-8.62140900	1.21367600	-6.92094500	L
C-C_3	0	-8.60561100	1.14273000	-8.42178100	L
H-H_	0	-7.79002100	2.66712800	-9.72253900	L
H-H_	0	-6.56913600	1.51922100	-9.05561700	L
H-H_	0	-6.71645900	1.86194100	-6.10276000	L
H-H_	0	-8.06432000	3.03031200	-5.87563200	L
H-H_	0	-8.31775400	0.24482400	-6.46912300	L
H-H_	0	-9.63530300	1.48803500	-6.56058600	L
H-H_	0	-8.40449800	0.11171000	-8.78155700	L
H-H_	0	-9.57673400	1.49421700	-8.83470400	L
C-C_3	0	6.41436100	9.62044900	0.17442800	L
O-O_3	0	5.11881100	9.32929200	0.63169500	L
C-C_3	0	5.22281700	9.17419000	2.02554800	L
C-C_3	0	6.63942400	8.74986600	2.37048500	L
C-C_3	0	7.40469900	8.93320300	1.08847800	L
H-H_	0	6.53746800	9.26241900	-0.86835700	L
H-H_	0	6.57036200	10.72205900	0.19097100	L
H-H_	0	4.98081100	10.13990900	2.51826800	L
H-H_	0	4.49556000	8.40970500	2.37114300	L
H-H_	0	7.05527200	9.39031200	3.17912500	L
H-H_	0	6.66339700	7.68651800	2.69097800	L

H-H_	0	8.31326000	9.55606200	1.23726200	L
H-H_	0	7.69219500	7.94420800	0.66734300	L
C-C_3	0	8.81311200	-9.58002400	3.29531800	L
O-O_3	0	8.12610800	-8.67524700	2.46226600	L
C-C_3	0	6.86847400	-8.42587700	3.04407800	L
C-C_3	0	6.61710700	-9.42787600	4.14770100	L
C-C_3	0	7.99956200	-9.83420200	4.54866400	L
H-H_	0	8.96337200	-10.53190100	2.74162700	L
H-H_	0	9.80915100	-9.16304000	3.55692400	L
H-H_	0	6.86354900	-7.39857400	3.46165700	L
H-H_	0	6.07945400	-8.48995000	2.26878300	L
H-H_	0	6.05599800	-8.97787200	4.99536300	L
H-H_	0	6.06050700	-10.30390900	3.74791500	L
H-H_	0	8.36201700	-9.18387700	5.37560900	L
H-H_	0	8.04113200	-10.90051500	4.86021900	L
C-C_3	0	5.83813100	-1.93272100	-8.96276100	L
O-O_3	0	5.37208600	-0.60664600	-8.89656300	L
C-C_3	0	4.66563000	-0.34466900	-10.08837500	L
C-C_3	0	4.67627600	-1.57944700	-10.96954100	L
C-C_3	0	5.79498400	-2.39270200	-10.40124000	L
H-H_	0	6.87163000	-1.98631600	-8.56438900	L
H-H_	0	5.18396100	-2.57909300	-8.33749500	L
H-H_	0	3.62241900	-0.05177200	-9.84830600	L
H-H_	0	5.15725900	0.50140500	-10.61514000	L
H-H_	0	3.71557300	-2.13076100	-10.86615700	L
H-H_	0	4.85283100	-1.32583600	-12.03736700	L
H-H_	0	5.60429000	-3.48361100	-10.48530900	L
H-H_	0	6.74761700	-2.13474700	-10.91299200	L
C-C_3	0	-8.28387600	2.62524100	3.19187400	L
O-O_3	0	-8.99892700	1.41731500	3.27220700	L
C-C_3	0	-10.00085500	1.44101200	2.28215800	L
C-C_3	0	-10.01227400	2.78913600	1.58467600	L
C-C_3	0	-9.09144300	3.63427400	2.40952600	L
H-H_	0	-8.07903700	3.00517300	4.21262500	L
H-H_	0	-7.31121800	2.43862800	2.69286300	L
H-H_	0	-9.81418700	0.62647700	1.55028100	L
H-H_	0	-10.98397800	1.25484800	2.76343100	L
H-H_	0	-9.62070300	2.68828400	0.55009400	L

H-H_	0	-11.03531200	3.22270300	1.54708400	L
H-H_	0	-8.43994100	4.27027100	1.77465300	L
H-H_	0	-9.67584500	4.27294000	3.10517100	L
C-C_3	0	-4.72264200	-5.93552300	8.96270100	L
O-O_3	0	-4.41093900	-4.67529800	9.51221500	L
C-C_3	0	-3.34412700	-4.13476800	8.76725000	L
C-C_3	0	-2.76691800	-5.20540800	7.87011400	L
C-C_3	0	-3.91020800	-6.15383600	7.70321900	L
H-H_	0	-4.48332300	-6.72206100	9.70926700	L
H-H_	0	-5.80853700	-5.98944000	8.74058300	L
H-H_	0	-3.72389100	-3.29050000	8.15277600	L
H-H_	0	-2.56426100	-3.74858400	9.45552500	L
H-H_	0	-2.42149000	-4.79222800	6.90013100	L
H-H_	0	-1.92162300	-5.71070900	8.38162900	L
H-H_	0	-4.51052900	-5.86982800	6.81218100	L
H-H_	0	-3.56494700	-7.20617100	7.60922400	L
C-C_3	0	-4.85673600	-3.74722100	-4.03053300	L
O-O_3	0	-5.30412400	-5.01457000	-3.60848700	L
C-C_3	0	-6.67912900	-4.90844500	-3.33521500	L
C-C_3	0	-7.01971800	-3.45112200	-3.13478400	L
C-C_3	0	-6.00672600	-2.75905000	-3.98695500	L
H-H_	0	-4.04222000	-3.41020600	-3.35545000	L
H-H_	0	-4.45142700	-3.82737300	-5.05874900	L
H-H_	0	-7.25409100	-5.31997600	-4.19265700	L
H-H_	0	-6.92511300	-5.49977700	-2.42999100	L
H-H_	0	-8.05775200	-3.21665400	-3.45212200	L
H-H_	0	-6.87062200	-3.17316000	-2.06984500	L
H-H_	0	-6.41724700	-2.60728500	-5.00771300	L
H-H_	0	-5.70304900	-1.78200800	-3.55480700	L
C-C_3	0	9.92823900	3.28932500	3.27947700	L
O-O_3	0	9.77181800	3.00761100	4.65077400	L
C-C_3	0	11.04078400	2.69415900	5.17640200	L
C-C_3	0	12.10588400	2.99256300	4.14345400	L
C-C_3	0	11.34744400	2.98421100	2.85500000	L
H-H_	0	9.21478800	2.68404400	2.68504600	L
H-H_	0	9.69845900	4.35948500	3.10647600	L
H-H_	0	11.21875800	3.28710900	6.09863700	L
H-H_	0	11.06524800	1.61581800	5.44183500	L

H-H_	0	12.54072900	3.99870000	4.32570300	L
H-H_	0	12.91464300	2.22999200	4.15045200	L
H-H_	0	11.73522300	3.74573400	2.14575500	L
H-H_	0	11.39289600	1.97506300	2.39156400	L
C-C_3	0	10.88264700	6.88966700	4.32868900	L
O-O_3	0	12.07482800	6.52724400	3.67013800	L
C-C_3	0	11.96967600	6.96584400	2.33858500	L
C-C_3	0	10.50810600	7.11263000	1.98872700	L
C-C_3	0	9.85862900	7.36810100	3.31377400	L
H-H_	0	10.49114100	6.00755200	4.87937200	L
H-H_	0	11.09855000	7.69164200	5.06711600	L
H-H_	0	12.48748600	7.94481400	2.23623000	L
H-H_	0	12.45784900	6.23279300	1.66142500	L
H-H_	0	10.34240300	7.95332200	1.28254000	L
H-H_	0	10.12478200	6.16777200	1.54756500	L
H-H_	0	9.66843200	8.45626600	3.44222100	L
H-H_	0	8.90366900	6.80879400	3.40582500	L
C-C_3	0	-2.07691000	-4.54833300	-11.87026500	L
O-O_3	0	-1.24448800	-5.00865100	-10.82977800	L
C-C_3	0	-2.06661600	-5.31208100	-9.72711100	L
C-C_3	0	-3.51045000	-5.33900100	-10.17559000	L
C-C_3	0	-3.50243300	-4.43759400	-11.36823600	L
H-H_	0	-1.71543900	-3.56276800	-12.23283000	L
H-H_	0	-2.02898600	-5.27251600	-12.71201200	L
H-H_	0	-1.77359200	-6.29314300	-9.29686200	L
H-H_	0	-1.92939200	-4.52886600	-8.95281700	L
H-H_	0	-3.79521500	-6.37116600	-10.47769600	L
H-H_	0	-4.19906800	-4.97256500	-9.38539000	L
H-H_	0	-4.23948100	-4.76101500	-12.13495200	L
H-H_	0	-3.71585500	-3.39173900	-11.05632900	L
C-C_3	0	-5.79012600	-6.57464700	1.18347600	L
O-O_3	0	-4.85312600	-7.28263400	0.40565100	L
C-C_3	0	-4.22262200	-6.35980000	-0.45202100	L
C-C_3	0	-4.62934400	-4.95467800	-0.06551700	L
C-C_3	0	-5.92436100	-5.16749200	0.64959000	L
H-H_	0	-6.77197500	-7.09308200	1.15904900	L
H-H_	0	-5.43004200	-6.54493700	2.23361500	L
H-H_	0	-3.12339400	-6.47808600	-0.37894500	L

H-H_	0	-4.53108000	-6.57101500	-1.49777300	L
H-H_	0	-3.87389300	-4.52265900	0.62468700	L
H-H_	0	-4.75037200	-4.29495800	-0.95027000	L
H-H_	0	-6.08037000	-4.43602300	1.46803900	L
H-H_	0	-6.76832700	-5.10930700	-0.07008200	L
C-C_3	0	2.85093900	6.63896900	0.02704300	L
O-O_3	0	3.40245100	6.70010700	-1.26383500	L
C-C_3	0	4.73505500	6.25506800	-1.17313600	L
C-C_3	0	4.99774900	5.69124500	0.21011100	L
C-C_3	0	3.64579700	5.65996800	0.85404300	L
H-H_	0	2.89730300	7.64955500	0.48303500	L
H-H_	0	1.79144500	6.31666500	-0.02994800	L
H-H_	0	4.90109500	5.47039100	-1.93827400	L
H-H_	0	5.42456000	7.09790500	-1.38424000	L
H-H_	0	5.43993400	4.67722000	0.15008400	L
H-H_	0	5.67967800	6.35964700	0.77630500	L
H-H_	0	3.20328900	4.64418700	0.77106100	L
H-H_	0	3.68211100	5.96560400	1.92121300	L
C-C_3	0	-13.87325600	-4.40108100	-2.23441800	L
O-O_3	0	-13.51818100	-3.18663700	-2.85422900	L
C-C_3	0	-12.80734400	-3.49282100	-4.03262500	L
C-C_3	0	-12.59078600	-4.98736400	-4.12537400	L
C-C_3	0	-13.61406200	-5.54686300	-3.18825800	L
H-H_	0	-13.26700100	-4.52873800	-1.31167200	L
H-H_	0	-14.94545000	-4.37322100	-1.94586600	L
H-H_	0	-13.39298300	-3.13820100	-4.90801500	L
H-H_	0	-11.82922600	-2.96840500	-4.02710500	L
H-H_	0	-12.73667100	-5.35977700	-5.16247800	L
H-H_	0	-11.56706900	-5.24183000	-3.77532800	L
H-H_	0	-14.54414300	-5.79430800	-3.74623300	L
H-H_	0	-13.24128800	-6.44944900	-2.65730300	L
C-C_3	0	-7.10829600	-1.66365200	0.64081200	L
O-O_3	0	-8.12788700	-1.39298000	-0.28419800	L
C-C_3	0	-7.81831900	-0.15156800	-0.86291500	L
C-C_3	0	-6.95057300	0.63777500	0.09661600	L
C-C_3	0	-6.58130700	-0.34799100	1.16757100	L
H-H_	0	-7.51084000	-2.28326500	1.46886600	L
H-H_	0	-6.29635400	-2.22856000	0.13578900	L

H-H_	0	-7.28483700	-0.31647100	-1.82499000	L
H-H_	0	-8.75907800	0.40098800	-1.06391000	L
H-H_	0	-6.04989600	1.02935100	-0.42044700	L
H-H_	0	-7.52238400	1.48177700	0.52762300	L
H-H_	0	-5.48289900	-0.37907100	1.32655700	L
H-H_	0	-7.08779800	-0.09410100	2.12325600	L
C-C_3	0	-0.17663500	-9.51572900	-8.44531600	L
O-O_3	0	1.18366800	-9.87604500	-8.54115000	L
C-C_3	0	1.65455700	-10.10927100	-7.23494800	L
C-C_3	0	0.69152300	-9.49110200	-6.24896300	L
C-C_3	0	-0.60564100	-9.52081900	-6.99109900	L
H-H_	0	-0.30998100	-8.49898500	-8.87236100	L
H-H_	0	-0.79381800	-10.22734500	-9.03419600	L
H-H_	0	1.72119800	-11.20600100	-7.06616200	L
H-H_	0	2.66628400	-9.66959100	-7.11538100	L
H-H_	0	0.63805900	-10.06494700	-5.29962300	L
H-H_	0	0.98828500	-8.44077900	-6.04502700	L
H-H_	0	-1.15860200	-10.45725500	-6.75615400	L
H-H_	0	-1.23497300	-8.64098900	-6.74815400	L
C-C_3	0	-10.18089000	-9.08728000	1.01772900	L
O-O_3	0	-11.19836200	-8.32940100	1.63292300	L
C-C_3	0	-12.25520000	-8.20239400	0.71026000	L
C-C_3	0	-12.03922500	-9.17209100	-0.42963600	L
C-C_3	0	-10.55633900	-9.36364900	-0.42453400	L
H-H_	0	-9.21816100	-8.53606200	1.07211000	L
H-H_	0	-10.06720100	-10.04586900	1.56838100	L
H-H_	0	-13.22252300	-8.40506300	1.21701500	L
H-H_	0	-12.27124300	-7.16213100	0.32058100	L
H-H_	0	-12.54760700	-10.13677100	-0.20903300	L
H-H_	0	-12.40537600	-8.76386000	-1.39579300	L
H-H_	0	-10.27349600	-10.39141000	-0.73903700	L
H-H_	0	-10.07390000	-8.61849000	-1.09348900	L
C-C_3	0	2.42463800	-5.39746300	-10.49024000	L
O-O_3	0	3.32647500	-4.68014700	-9.68902200	L
C-C_3	0	3.86010000	-5.60460400	-8.77167000	L
C-C_3	0	2.91189500	-6.78753200	-8.64263600	L
C-C_3	0	1.83180000	-6.50234100	-9.64807000	L
H-H_	0	1.62941300	-4.72122000	-10.86523900	L

H-H_	0	2.96703800	-5.82488000	-11.36286500	L
H-H_	0	4.85597700	-5.94439400	-9.13069800	L
H-H_	0	3.99593700	-5.10774700	-7.78998500	L
H-H_	0	3.43784300	-7.73368200	-8.89684900	L
H-H_	0	2.49332700	-6.86263300	-7.61542000	L
H-H_	0	1.58836100	-7.39615500	-10.26228300	L
H-H_	0	0.91754200	-6.13503000	-9.13425200	L
C-C_3	0	2.01542000	12.30336400	6.12081600	L
O-O_3	0	2.13160400	12.62308000	4.75513700	L
C-C_3	0	3.40203500	12.18136000	4.35119900	L
C-C_3	0	3.78449400	10.98907400	5.20010600	L
C-C_3	0	2.98088600	11.17732100	6.45701100	L
H-H_	0	2.24715000	13.20532500	6.72856600	L
H-H_	0	0.97073700	11.99418700	6.33701100	L
H-H_	0	3.37835400	11.90472800	3.27772900	L
H-H_	0	4.13790500	13.00440600	4.48879200	L
H-H_	0	3.48743400	10.04489800	4.69362400	L
H-H_	0	4.87690900	10.97370200	5.40470500	L
H-H_	0	2.43227600	10.24613500	6.71825600	L
H-H_	0	3.63920400	11.46820000	7.30491700	L
C-C_3	0	-3.56843200	-1.31623600	11.32163000	L
O-O_3	0	-3.28932800	-1.02884200	9.97494600	L
C-C_3	0	-3.75524000	0.27602000	9.72574300	L
C-C_3	0	-4.70329700	0.70266300	10.83307700	L
C-C_3	0	-4.74304700	-0.47391300	11.76355200	L
H-H_	0	-3.80257000	-2.39610900	11.43310700	L
H-H_	0	-2.67403500	-1.08384700	11.93889400	L
H-H_	0	-2.89042400	0.97071000	9.66955200	L
H-H_	0	-4.27881200	0.29056900	8.74852500	L
H-H_	0	-4.30241800	1.59550400	11.35637000	L
H-H_	0	-5.71588700	0.93276300	10.43636600	L
H-H_	0	-4.64261600	-0.16214500	12.82610200	L
H-H_	0	-5.69043300	-1.03997700	11.62685700	L
C-C_3	0	11.00685200	7.51274600	-1.61732400	L
O-O_3	0	9.80835400	8.22320400	-1.41407100	L
C-C_3	0	8.92890000	7.89255900	-2.46501300	L
C-C_3	0	9.61184800	6.93783500	-3.42633900	L
C-C_3	0	11.05600900	7.01956200	-3.04458100	L

H-H_	0	11.87418400	8.17295500	-1.40434900	L
H-H_	0	11.04273700	6.65094600	-0.91802000	L
H-H_	0	8.00933200	7.42996000	-2.04718500	L
H-H_	0	8.64021800	8.82418300	-2.99748500	L
H-H_	0	9.23373900	5.90574100	-3.27066100	L
H-H_	0	9.45771500	7.23734000	-4.48494300	L
H-H_	0	11.56065200	6.03167700	-3.12228200	L
H-H_	0	11.57773100	7.76454600	-3.68529700	L
C-C_3	0	-8.54850500	6.22721100	-6.79473400	L
O-O_3	0	-9.64044900	6.17990200	-5.91362200	L
C-C_3	0	-10.50768600	5.18799600	-6.40931100	L
C-C_3	0	-10.23701200	4.98011000	-7.89104100	L
C-C_3	0	-9.03966300	5.84418800	-8.17292700	L
H-H_	0	-8.11493700	7.24968200	-6.80447800	L
H-H_	0	-7.76919300	5.51463700	-6.44830200	L
H-H_	0	-10.34137600	4.24223400	-5.85002900	L
H-H_	0	-11.55942600	5.50797000	-6.25046000	L
H-H_	0	-10.00909200	3.91222400	-8.09737700	L
H-H_	0	-11.10692700	5.29763400	-8.50671900	L
H-H_	0	-8.26351800	5.29705600	-8.74772000	L
H-H_	0	-9.34662600	6.75484400	-8.73311600	L
C-C_3	0	8.60121000	-1.63903400	1.08703200	L
O-O_3	0	7.25472100	-1.60849600	1.48136600	L
C-C_3	0	6.70196700	-0.42653800	0.95247500	L
C-C_3	0	7.81584200	0.49025800	0.47635300	L
C-C_3	0	9.06867400	-0.21681300	0.89574400	L
H-H_	0	9.20415100	-2.14747100	1.86574700	L
H-H_	0	8.68945600	-2.20455300	0.13259700	L
H-H_	0	6.03196400	-0.69092600	0.11051300	L
H-H_	0	6.09891300	0.07938200	1.73543000	L
H-H_	0	7.77835300	0.58714500	-0.62944500	L
H-H_	0	7.74852600	1.49546000	0.94201300	L
H-H_	0	9.87063400	-0.14092200	0.13304600	L
H-H_	0	9.43575400	0.19620300	1.85844600	L
C-C_3	0	12.16641600	-5.16800900	0.30516900	L
O-O_3	0	11.66590700	-6.48371300	0.38579200	L
C-C_3	0	10.50913200	-6.44049500	1.18360700	L
C-C_3	0	9.94518600	-5.04017500	1.13639600	L

C-C_3	0	11.15994700	-4.19713800	0.89863900	L
H-H_	0	13.13329300	-5.10504800	0.84913500	L
H-H_	0	12.34900300	-4.91788000	-0.76076000	L
H-H_	0	9.76558300	-7.17416700	0.80903900	L
H-H_	0	10.77848500	-6.71086600	2.22799200	L
H-H_	0	9.24194600	-4.93688400	0.28447200	L
H-H_	0	9.42553000	-4.77274900	2.07985700	L
H-H_	0	10.94609200	-3.35669900	0.20272000	L
H-H_	0	11.53660500	-3.79627200	1.86379800	L
C-C_3	0	6.48736500	-11.49708800	0.55169900	L
O-O_3	0	5.91583900	-10.25746400	0.20901700	L
C-C_3	0	6.84951400	-9.55949200	-0.58319900	L
C-C_3	0	8.11709100	-10.37679500	-0.72742800	L
C-C_3	0	7.68680700	-11.75393400	-0.33317100	L
H-H_	0	6.80098900	-11.46714300	1.61713200	L
H-H_	0	5.73392700	-12.30363700	0.42826400	L
H-H_	0	6.40302900	-9.36857100	-1.58117600	L
H-H_	0	7.07941600	-8.58473800	-0.11204200	L
H-H_	0	8.50900300	-10.35001800	-1.76752400	L
H-H_	0	8.89401100	-10.00332000	-0.02381900	L
H-H_	0	7.37944300	-12.32894600	-1.23427300	L
H-H_	0	8.49011100	-12.29905600	0.20851300	L
C-C_3	0	-9.69612400	-5.19521500	0.95639200	L
O-O_3	0	-9.38047600	-6.05261900	-0.11806800	L
C-C_3	0	-9.97152900	-5.50527300	-1.26985600	L
C-C_3	0	-10.15791400	-4.02546100	-1.04656200	L
C-C_3	0	-10.38010800	-3.94708900	0.43008100	L
H-H_	0	-10.35924300	-5.72439600	1.67044800	L
H-H_	0	-8.76025000	-4.91789500	1.48580300	L
H-H_	0	-9.32812800	-5.68997700	-2.15376600	L
H-H_	0	-10.95525500	-5.99293300	-1.43963100	L
H-H_	0	-9.22901600	-3.48627900	-1.31771300	L
H-H_	0	-11.01164200	-3.61521600	-1.62297800	L
H-H_	0	-9.94457200	-3.02353300	0.86425200	L
H-H_	0	-11.46812100	-3.98528100	0.64854100	L
C-C_3	0	-0.76329800	-0.97655100	-10.23884000	L
O-O_3	0	-0.91709700	-0.61933500	-8.89007700	L
C-C_3	0	-0.30598100	-1.63767300	-8.13354500	L

C-C_3	0	0.64387900	-2.42807800	-9.01993500	L
C-C_3	0	0.50362800	-1.78693000	-10.36981700	L
H-H_	0	-1.64080700	-1.57951600	-10.56013300	L
H-H_	0	-0.70422600	-0.06247400	-10.86632700	L
H-H_	0	0.24308400	-1.18669600	-7.28063600	L
H-H_	0	-1.09266100	-2.30169000	-7.72516300	L
H-H_	0	1.69197100	-2.36306200	-8.66264100	L
H-H_	0	0.33819900	-3.49518500	-9.05641700	L
H-H_	0	1.36397100	-1.10907800	-10.56203600	L
H-H_	0	0.42652300	-2.53905600	-11.18365600	L
C-C_3	0	12.59782200	-5.37359900	-4.72897000	L
O-O_3	0	11.77508400	-6.35207400	-5.32217900	L
C-C_3	0	11.95644900	-7.55536000	-4.61047000	L
C-C_3	0	12.78611100	-7.29006700	-3.37201000	L
C-C_3	0	13.51419900	-6.03164600	-3.71873300	L
H-H_	0	13.19082600	-4.85644700	-5.51305900	L
H-H_	0	11.95555500	-4.62197200	-4.22336100	L
H-H_	0	10.96955800	-7.98068700	-4.33120100	L
H-H_	0	12.48057900	-8.28325000	-5.26695500	L
H-H_	0	12.12330200	-7.11687400	-2.49634000	L
H-H_	0	13.48601200	-8.12596900	-3.15492000	L
H-H_	0	13.67764900	-5.39293700	-2.82494500	L
H-H_	0	14.49056200	-6.27492900	-4.19336700	L
C-C_3	0	-1.02825100	8.24034300	-6.08894900	L
O-O_3	0	0.36724100	8.25606100	-6.27806700	L
C-C_3	0	0.77819800	6.93460300	-6.54352700	L
C-C_3	0	-0.43765000	6.04400300	-6.68459400	L
C-C_3	0	-1.50266500	6.81087000	-5.97313600	L
H-H_	0	-1.28898000	8.80540500	-5.17281200	L
H-H_	0	-1.51691100	8.73172800	-6.95765500	L
H-H_	0	1.38231900	6.90723400	-7.47340700	L
H-H_	0	1.41392500	6.58439100	-5.70544600	L
H-H_	0	-0.70088300	5.93566300	-7.75818800	L
H-H_	0	-0.28052900	5.04357300	-6.22914000	L
H-H_	0	-2.49733000	6.67375500	-6.44248800	L
H-H_	0	-1.54242500	6.50391400	-4.90657600	L
C-C_3	0	2.66657800	9.44950200	-8.79589000	L
O-O_3	0	1.84166900	10.39918400	-8.15875700	L

C-C_3	0	0.63562000	10.46230000	-8.87963200	L
C-C_3	0	0.47694300	9.18800100	-9.67616600	L
C-C_3	0	1.89268600	8.75435900	-9.90354700	L
H-H_	0	3.56351600	9.95778800	-9.21034500	L
H-H_	0	3.00605700	8.70590400	-8.04381300	L
H-H_	0	-0.21520300	10.59107900	-8.17804700	L
H-H_	0	0.66749400	11.33869200	-9.56348100	L
H-H_	0	-0.06461200	8.42711500	-9.07368500	L
H-H_	0	-0.06243000	9.36297000	-10.63234300	L
H-H_	0	1.99493100	7.64859900	-9.84679600	L
H-H_	0	2.24482900	9.11041700	-10.89695500	L
C-C_3	0	0.45085800	12.83535200	-3.95085300	L
O-O_3	0	-0.76907800	13.48861700	-3.68471300	L
C-C_3	0	-1.76699400	12.80521100	-4.40028200	L
C-C_3	0	-1.12445900	12.12788400	-5.58710500	L
C-C_3	0	0.27392300	11.86873600	-5.11242800	L
H-H_	0	1.21963500	13.59643500	-4.20323700	L
H-H_	0	0.78322000	12.29517000	-3.03982800	L
H-H_	0	-2.24001200	12.04993500	-3.73579400	L
H-H_	0	-2.54699600	13.52098500	-4.73565100	L
H-H_	0	-1.65503700	11.19142300	-5.86149400	L
H-H_	0	-1.10869100	12.82187200	-6.45634800	L
H-H_	0	0.37271200	10.81919900	-4.76117200	L
H-H_	0	1.01553800	12.05808600	-5.91873500	L
C-C_3	0	14.71257800	-1.66200800	-4.13020400	L
O-O_3	0	14.19456800	-2.78004600	-3.45235100	L
C-C_3	0	13.98297600	-2.40199300	-2.11052100	L
C-C_3	0	14.28825800	-0.92505200	-1.93949800	L
C-C_3	0	14.37091500	-0.41821400	-3.34521200	L
H-H_	0	15.81639600	-1.76890400	-4.21429400	L
H-H_	0	14.28239300	-1.60755800	-5.15268200	L
H-H_	0	12.92470400	-2.60298500	-1.84263900	L
H-H_	0	14.63131000	-3.01244400	-1.44591400	L
H-H_	0	13.49487800	-0.40355600	-1.36198500	L
H-H_	0	15.26926600	-0.79562900	-1.43128200	L
H-H_	0	13.38278800	-0.02520800	-3.66842700	L
H-H_	0	15.14887200	0.36688900	-3.45971000	L
C-C_3	0	-13.01494100	-5.35461600	5.23015800	L

O-O_3	0	-13.32317200	-4.02421900	4.87822400	L
C-C_3	0	-13.37030700	-3.95563200	3.47209900	L
C-C_3	0	-13.40351400	-5.35883200	2.91019000	L
C-C_3	0	-12.72086500	-6.15349500	3.97655600	L
H-H_	0	-12.14224000	-5.36909300	5.91610900	L
H-H_	0	-13.88675800	-5.79464000	5.76086800	L
H-H_	0	-14.26791900	-3.38543900	3.15162700	L
H-H_	0	-12.46417300	-3.42631400	3.10832600	L
H-H_	0	-14.45669900	-5.69894300	2.79690000	L
H-H_	0	-12.87803900	-5.42890600	1.93619500	L
H-H_	0	-13.11787500	-7.18992800	4.03870100	L
H-H_	0	-11.62504400	-6.17814900	3.79036400	L
C-C_3	0	-13.48143200	-0.05571000	1.41204500	L
O-O_3	0	-13.00931100	1.10357500	0.77507600	L
C-C_3	0	-12.39649600	0.68445800	-0.42044600	L
C-C_3	0	-12.02106800	-0.78263700	-0.31010000	L
C-C_3	0	-12.60562800	-1.21808000	1.00251900	L
H-H_	0	-13.45114900	0.08312600	2.51392800	L
H-H_	0	-14.53447000	-0.23992800	1.10414500	L
H-H_	0	-13.09999600	0.84176700	-1.26647100	L
H-H_	0	-11.48795100	1.29465300	-0.59692600	L
H-H_	0	-12.46123200	-1.36175000	-1.14979100	L
H-H_	0	-10.91755900	-0.91309600	-0.31109600	L
H-H_	0	-13.19791600	-2.15305100	0.89841300	L
H-H_	0	-11.79540400	-1.36445100	1.74839100	L
C-C_3	0	4.61026000	-11.85476100	-2.71023300	L
O-O_3	0	4.02893400	-12.34089800	-3.89675200	L
C-C_3	0	2.63796600	-12.24644600	-3.72819400	L
C-C_3	0	2.35122300	-11.09448300	-2.79386600	L
C-C_3	0	3.59993900	-10.99463600	-1.96649600	L
H-H_	0	4.92394700	-12.71426700	-2.07890400	L
H-H_	0	5.51235700	-11.25963800	-2.96411900	L
H-H_	0	2.15048000	-12.08125700	-4.71141700	L
H-H_	0	2.25491100	-13.19742500	-3.29580700	L
H-H_	0	2.20419400	-10.16059200	-3.37745900	L
H-H_	0	1.45661200	-11.29357800	-2.16786600	L
H-H_	0	3.94057700	-9.93901500	-1.89272200	L
H-H_	0	3.42665300	-11.39956900	-0.94528900	L

C-C_3	0	-8.56749600	-1.98289700	9.70884100	L
O-O_3	0	-7.49134900	-1.11483300	9.43167400	L
C-C_3	0	-6.56821900	-1.82719800	8.64533900	L
C-C_3	0	-7.27748400	-2.99245600	8.00231500	L
C-C_3	0	-8.36428800	-3.30071600	8.98209800	L
H-H_	0	-8.63570000	-2.15072300	10.80501300	L
H-H_	0	-9.51051400	-1.50503500	9.36798000	L
H-H_	0	-6.13433800	-1.16207800	7.87045200	L
H-H_	0	-5.74830200	-2.19579500	9.29715400	L
H-H_	0	-7.71215800	-2.68090900	7.02726800	L
H-H_	0	-6.59615000	-3.85540900	7.85941000	L
H-H_	0	-9.29087200	-3.64227600	8.47185100	L
H-H_	0	-8.02028100	-4.08170100	9.69622200	L
C-C_R	0	2.30820500	-0.07843700	1.09127700	H
C-C_R	0	2.50552900	0.97978100	3.01003000	H
C-C_3	0	2.82804400	-1.51299400	3.18904200	H
H-H_	0	1.90766900	-2.09960900	3.18175600	H
N-N_R	0	2.35948500	1.92597300	2.12770500	H
N-N_R	0	2.24224000	1.26229800	0.92731700	H
N-N_R	0	2.55276900	-0.26105000	2.42156200	H
C-C_3	0	2.47043800	1.14197200	4.50211700	H
H-H_	0	1.60063400	1.75368900	4.74731600	H
H-H_	0	3.37426200	1.64597900	4.87224500	H
C-C_3	0	3.18108000	-1.08226400	4.62157400	H
O-O_3	0	2.28607300	-0.11059100	5.12657300	H
C-C_R	0	2.01461800	2.08313300	-0.24179000	H
C-C_R	0	3.10790200	2.41464400	-1.05858900	H
C-C_R	0	0.73429600	2.61985200	-0.44598400	H
C-C_R	0	2.88571100	3.29444300	-2.11886800	H
C-C_R	0	0.58225200	3.53338500	-1.49528700	H
C-C_R	0	1.63601600	3.88169500	-2.34156900	H
H-H_	0	3.71717200	3.53394900	-2.77480900	H
H-H_	0	-0.40005200	3.96679800	-1.66114000	H
H-H_	0	3.12017500	-1.95160200	5.27714200	H
C-C_R	0	2.03950400	-1.15789100	0.12504700	H
O-O_R	0	1.55445400	-2.30479700	0.62418600	H
C-C_R	0	2.35369700	-1.01184000	-1.18315700	H
C-C_3	0	2.07848000	-2.06374700	-2.20844500	H

H-H_	0	2.81197300	-0.08725100	-1.49648100	H
H-H_	0	2.54912900	-3.00643800	-1.90336200	H
H-H_	0	0.98954400	-2.26106700	-2.20828800	H
C-C_R	0	2.47645200	-1.74458300	-3.63476900	H
C-C_R	0	2.55331200	-2.80142900	-4.55429200	H
C-C_R	0	2.70843600	-0.44672500	-4.10776000	H
C-C_R	0	2.83766000	-2.57226800	-5.89722700	H
H-H_	0	2.37175300	-3.81413500	-4.20493700	H
C-C_R	0	3.01359100	-0.21502900	-5.45046600	H
H-H_	0	2.64218700	0.40490600	-3.43877600	H
C-C_R	0	3.07395600	-1.27371300	-6.35118500	H
H-H_	0	2.87431100	-3.40647100	-6.58936100	H
H-H_	0	3.20847100	0.79785100	-5.78766500	H
H-H_	0	3.29808600	-1.08735600	-7.39461600	H
C-C_3	0	4.48524700	1.85948500	-0.81030400	H
H-H_	0	4.92845300	2.28366800	0.09483000	H
H-H_	0	4.46425300	0.77736000	-0.68028600	H
H-H_	0	5.13874100	2.07691600	-1.65651400	H
C-C_3	0	1.43852000	4.86738700	-3.46439600	H
H-H_	0	0.38128600	4.98358100	-3.71380300	H
H-H_	0	1.82440400	5.85774700	-3.19955800	H
H-H_	0	1.96802600	4.54969600	-4.36606400	H
C-C_3	0	-0.45028200	2.20144700	0.37743300	H
H-H_	0	-0.75690000	1.18301500	0.12037300	H
H-H_	0	-0.24417200	2.18732700	1.44691800	H
H-H_	0	-1.29486400	2.86437000	0.18796800	H
H-H_	0	4.21341500	-0.69679700	4.64910700	H
C-C_3	0	3.98058000	-2.33973400	2.58344200	H
H-H_	0	3.85869600	-2.38843500	1.50070200	H
H-H_	0	4.92940800	-1.83679000	2.79449700	H
C-C_R	0	4.00605000	-3.74775300	3.14423600	H
C-C_R	0	4.98442700	-4.15328000	4.05932700	H
C-C_R	0	3.03289700	-4.67194300	2.73733400	H
C-C_R	0	5.00445500	-5.46085000	4.54756400	H
H-H_	0	5.74680500	-3.44997100	4.37969200	H
C-C_R	0	3.05472500	-5.97698000	3.22209000	H
H-H_	0	2.26568400	-4.36011600	2.03550900	H
C-C_R	0	4.04348700	-6.37742400	4.12302200	H

H-H_	0	5.77639300	-5.76638800	5.24713800	H
H-H_	0	2.29216700	-6.67682500	2.89799300	H
H-H_	0	4.06383100	-7.39634200	4.49527800	H
C-C_3	0	-1.93256300	2.09785200	-4.04606700	H
C-C_3	0	-0.78313000	1.10927000	-3.83514500	H
C-C_3	0	-1.15910400	-0.36477200	-4.07199300	H
C-C_3	0	-3.12126900	1.91194600	-3.09625900	H
C-C_R	0	-1.81113100	-1.03888700	-2.88139300	H
C-C_3	0	-3.75411300	0.51207800	-3.15592200	H
H-H_	0	-2.29009800	1.99331100	-5.08015900	H
H-H_	0	0.03267400	1.36348600	-4.51871100	H
H-H_	0	-1.78925700	-0.44272600	-4.96639200	H
H-H_	0	-2.81576800	2.11013000	-2.06125100	H
H-H_	0	-4.75328400	0.54683500	-2.71126500	H
C-C_3	0	-1.74142100	-2.64891200	-1.15685100	H
H-H_	0	-0.92806700	-3.07527900	-0.56260100	H
C-C_3	0	-3.58573400	-0.97141500	-1.16101200	H
C-C_3	0	-2.53525000	-1.66549800	-0.30280900	H
H-H_	0	-1.84845300	-0.92928900	0.12667000	H
H-H_	0	-3.00846600	-2.17260700	0.53835900	H
H-H_	0	-3.97207700	-0.09153000	-0.63502100	H
N-N_R	0	-3.01723700	-0.52664000	-2.43928400	H
N-N_R	0	-1.16621700	-2.01497700	-2.33667800	H
H-H_	0	-3.90237300	0.22539400	-4.20408400	H
H-H_	0	-3.88965000	2.65201400	-3.34668800	H
H-H_	0	-1.55625400	3.12500400	-3.95517800	H
H-H_	0	-0.37073000	1.21591600	-2.82563900	H
H-H_	0	-0.25343800	-0.93483800	-4.27052100	H
H-H_	0	-2.38333000	-3.48643700	-1.46980700	H
H-H_	0	-4.43860600	-1.64113800	-1.34583600	H
C-C_R	0	-0.75783900	-1.36698900	4.38123700	H
C-C_R	0	-0.89052200	0.01762800	4.62038500	H
C-C_R	0	-1.09824200	0.48703600	5.93189100	H
C-C_R	0	-1.18466200	-0.45086800	6.94248300	H
C-C_R	0	-1.07154100	-1.84315500	6.67782400	H
C-C_R	0	-0.85115900	-2.32701300	5.40128400	H
H-H_	0	-1.18476700	1.55069700	6.12177400	H
H-H_	0	-1.33724000	-0.12360900	7.96564600	H

H-H_	0	-1.15248400	-2.53972600	7.50531500	H
H-H_	0	-0.75885600	-3.38429700	5.18547700	H
N-N_R	0	-0.71840700	0.69141200	3.44429700	H
N-N_R	0	-0.48981700	-0.21123900	2.50100800	H
N-N_R	0	-0.50290500	-1.43772100	3.04202600	H
O-O_R	0	-0.20307400	-2.52509100	2.33544100	H
H-H_	0	0.75406700	-2.26082700	1.34384200	H

TS4$_{\mathbf{explicit}}$ (ONIOM(B3LYP/6-31G(d, p):UFF))

0 1 0 1 0 1

C-C_3	0	-5.26120500	7.60113200	-5.03144700	L
O-O_3	0	-6.45392500	7.17839900	-4.41839600	L
C-C_3	0	-6.83348200	8.17445600	-3.49455200	L
C-C_3	0	-5.85378100	9.33276800	-3.55924500	L
C-C_3	0	-5.12016500	9.09254400	-4.84045700	L
H-H_	0	-4.40626900	7.07234200	-4.55708400	L
H-H_	0	-5.28386200	7.34885600	-6.11268200	L
H-H_	0	-7.85452000	8.52934800	-3.75271400	L
H-H_	0	-6.86204000	7.74235600	-2.47215400	L
H-H_	0	-6.37140000	10.31664000	-3.55967100	L
H-H_	0	-5.14616000	9.28384400	-2.70460500	L
H-H_	0	-5.61993100	9.63245900	-5.67466000	L
H-H_	0	-4.05712900	9.39967100	-4.77346400	L
C-C_3	0	-8.36857200	3.44080400	-11.89257700	L
O-O_3	0	-9.17847300	4.57566700	-12.10413600	L
C-C_3	0	-8.36304200	5.71318300	-11.95302400	L
C-C_3	0	-7.12049700	5.32658800	-11.18579800	L
C-C_3	0	-6.96538700	3.87429000	-11.50998900	L
H-H_	0	-8.81326600	2.83023000	-11.07785000	L
H-H_	0	-8.34403200	2.82395600	-12.81667100	L
H-H_	0	-8.08565800	6.09544700	-12.95953000	L
H-H_	0	-8.92223500	6.50618500	-11.41227400	L
H-H_	0	-6.23510000	5.91986900	-11.50261000	L
H-H_	0	-7.29573900	5.46064200	-10.09607000	L
H-H_	0	-6.27480900	3.74688100	-12.37318300	L
H-H_	0	-6.58529000	3.30208700	-10.63991600	L
C-C_3	0	7.19549300	-3.64839400	8.16610600	L
O-O_3	0	6.02559100	-3.23145000	7.50916000	L

C-C_3	0	5.28968000	-2.49461700	8.45081200	L
C-C_3	0	6.25849700	-1.84701500	9.41870000	L
C-C_3	0	7.52290300	-2.64933600	9.26099100	L
H-H_	0	7.03090800	-4.65957100	8.59957200	L
H-H_	0	8.03009600	-3.70940700	7.43639000	L
H-H_	0	4.68205600	-1.72541100	7.93056400	L
H-H_	0	4.60304700	-3.17658900	8.99758200	L
H-H_	0	6.43575500	-0.78808700	9.13507300	L
H-H_	0	5.87239700	-1.89261800	10.46073800	L
H-H_	0	8.36449400	-1.99188600	8.95184100	L
H-H_	0	7.78920900	-3.17214400	10.20568400	L
C-C_3	0	-5.84484000	-7.16969100	5.57101000	L
O-O_3	0	-6.64828000	-7.70781300	4.55333400	L
C-C_3	0	-7.87449300	-7.01940700	4.61198200	L
C-C_3	0	-8.03650600	-6.39785700	5.99133400	L
C-C_3	0	-6.75005600	-6.72336200	6.69382800	L
H-H_	0	-5.12519200	-7.93356400	5.93216400	L
H-H_	0	-5.27764100	-6.30191600	5.16787000	L
H-H_	0	-7.89089000	-6.23259500	3.82659800	L
H-H_	0	-8.70168400	-7.73313500	4.41541600	L
H-H_	0	-8.16387600	-5.29759800	5.91067100	L
H-H_	0	-8.90638700	-6.83195600	6.53121900	L
H-H_	0	-6.33404900	-5.84212400	7.22249500	L
H-H_	0	-6.90459300	-7.55888000	7.41157600	L
C-C_3	0	-4.00719800	3.04905500	5.91246800	L
O-O_3	0	-4.01223600	1.80848700	6.57720800	L
C-C_3	0	-4.43428300	0.82886500	5.65818700	L
C-C_3	0	-4.47478800	1.42449300	4.26657500	L
C-C_3	0	-4.60092300	2.88888600	4.53103100	L
H-H_	0	-4.58267000	3.79053700	6.50048300	L
H-H_	0	-2.96268700	3.40886400	5.83348500	L
H-H_	0	-3.74219400	-0.03780500	5.69387800	L
H-H_	0	-5.44585600	0.47771500	5.94830400	L
H-H_	0	-3.52509000	1.21409100	3.72998400	L
H-H_	0	-5.33405900	1.04166400	3.67432900	L
H-H_	0	-4.05005600	3.49367900	3.78059200	L
H-H_	0	-5.67401300	3.17578200	4.54146100	L
C-C_3	0	0.17688700	-5.88126500	-5.96528000	L

O-O_3	0	-0.90513900	-4.99025200	-5.88392100	L
C-C_3	0	-0.73932000	-4.30697300	-4.66949200	L
C-C_3	0	-0.08304400	-5.25218900	-3.68276600	L
C-C_3	0	0.55109600	-6.29976700	-4.55699400	L
H-H_	0	-0.11053800	-6.76348400	-6.57324000	L
H-H_	0	1.03434700	-5.37273400	-6.45947500	L
H-H_	0	-0.09872700	-3.41477900	-4.83689300	L
H-H_	0	-1.72362100	-3.96416500	-4.29159600	L
H-H_	0	0.66765400	-4.72517900	-3.05690500	L
H-H_	0	-0.84610000	-5.71857700	-3.02426600	L
H-H_	0	1.65440600	-6.32237800	-4.43020700	L
H-H_	0	0.12336100	-7.29806300	-4.32495200	L
C-C_3	0	-6.22571400	-9.68732900	-0.74073500	L
O-O_3	0	-6.05264000	-9.98496400	0.62593800	L
C-C_3	0	-6.26279500	-11.36583000	0.79702800	L
C-C_3	0	-6.23987800	-12.04384300	-0.55448800	L
C-C_3	0	-6.62453400	-10.94296600	-1.49061800	L
H-H_	0	-6.99869100	-8.90024300	-0.85322000	L
H-H_	0	-5.27170600	-9.29092700	-1.14595700	L
H-H_	0	-5.47297300	-11.78669900	1.45431000	L
H-H_	0	-7.24882800	-11.52259300	1.28468000	L
H-H_	0	-5.21189600	-12.39962900	-0.78633200	L
H-H_	0	-6.95358500	-12.89494100	-0.60188200	L
H-H_	0	-6.09162300	-11.02998900	-2.46150900	L
H-H_	0	-7.72352700	-10.95500200	-1.65918600	L
C-C_3	0	7.74413600	-5.18901700	-7.56994900	L
O-O_3	0	7.81507500	-5.14634100	-8.97731200	L
C-C_3	0	8.98757100	-4.44596500	-9.32444800	L
C-C_3	0	9.53680900	-3.75527600	-8.09594500	L
C-C_3	0	9.00741200	-4.59550600	-6.98274200	L
H-H_	0	6.86116500	-4.60218500	-7.23661300	L
H-H_	0	7.61746400	-6.23976800	-7.23349500	L
H-H_	0	9.73512000	-5.16923100	-9.71695100	L
H-H_	0	8.76328300	-3.70549500	-10.11997500	L
H-H_	0	10.64789900	-3.72540100	-8.09609400	L
H-H_	0	9.12671600	-2.72539500	-8.01877400	L
H-H_	0	9.73511800	-5.40209700	-6.74726400	L
H-H_	0	8.79792700	-3.99221000	-6.07470400	L

C-C_3	0	9.59461800	4.31839800	-0.89256900	L
O-O_3	0	9.71653800	3.58983300	-2.09144200	L
C-C_3	0	8.44359000	3.53708300	-2.69365400	L
C-C_3	0	7.43290700	4.24562600	-1.81350300	L
C-C_3	0	8.27661100	5.05752800	-0.88635500	L
H-H_	0	9.63997000	3.61085600	-0.03816400	L
H-H_	0	10.43520900	5.03629900	-0.80056300	L
H-H_	0	8.49691800	4.03042500	-3.68628900	L
H-H_	0	8.14658100	2.47866300	-2.85269400	L
H-H_	0	6.74350400	4.88161900	-2.40604400	L
H-H_	0	6.85423300	3.50499300	-1.22723800	L
H-H_	0	8.40867300	6.08274300	-1.29152700	L
H-H_	0	7.83935900	5.11202800	0.13393500	L
C-C_3	0	9.76090100	-5.18482100	-2.34219100	L
O-O_3	0	9.66239700	-5.22493900	-3.74198600	L
C-C_3	0	8.87186300	-6.34411700	-4.06933000	L
C-C_3	0	8.66035400	-7.20462600	-2.83686600	L
C-C_3	0	9.54835400	-6.58041100	-1.80696900	L
H-H_	0	10.76000800	-4.80480400	-2.04645700	L
H-H_	0	8.98420500	-4.49602700	-1.94459000	L
H-H_	0	7.90014300	-5.99572100	-4.47275300	L
H-H_	0	9.38623300	-6.93416100	-4.85612100	L
H-H_	0	7.60167600	-7.14940600	-2.50757900	L
H-H_	0	8.93709900	-8.26423100	-3.02499600	L
H-H_	0	9.07049200	-6.56826600	-0.80717100	L
H-H_	0	10.51718100	-7.12322300	-1.75582900	L
C-C_3	0	-4.03496700	-9.74558900	3.52994600	L
O-O_3	0	-3.07307200	-10.10683700	2.56566200	L
C-C_3	0	-2.58539900	-11.38427100	2.90222600	L
C-C_3	0	-3.03275400	-11.73701100	4.30426300	L
C-C_3	0	-4.25463800	-10.89510800	4.49099600	L
H-H_	0	-3.67011900	-8.85501100	4.08024500	L
H-H_	0	-4.98815500	-9.47913400	3.02864600	L
H-H_	0	-2.98842400	-12.12512200	2.17817200	L
H-H_	0	-1.47683300	-11.39232100	2.83340200	L
H-H_	0	-3.25647300	-12.82106800	4.40701500	L
H-H_	0	-2.25462700	-11.44091300	5.04032300	L
H-H_	0	-5.16095500	-11.46938400	4.19619500	L

H-H_	0	-4.35531200	-10.54279200	5.54076600	L
C-C_3	0	-4.88474800	-7.53662700	-6.65626000	L
O-O_3	0	-5.68657800	-8.14210800	-5.66787500	L
C-C_3	0	-4.95515400	-9.21374500	-5.12863900	L
C-C_3	0	-3.48686600	-8.99110500	-5.40357800	L
C-C_3	0	-3.49331200	-8.14551200	-6.63893100	L
H-H_	0	-5.35800000	-7.67688200	-7.65179000	L
H-H_	0	-4.82406500	-6.44818900	-6.45013800	L
H-H_	0	-5.13984700	-9.28148600	-4.03623000	L
H-H_	0	-5.29234600	-10.15952500	-5.60585800	L
H-H_	0	-3.01937800	-8.43053500	-4.56618500	L
H-H_	0	-2.95399000	-9.95325800	-5.55975400	L
H-H_	0	-2.70501100	-7.36237400	-6.59554100	L
H-H_	0	-3.33917900	-8.78051100	-7.53931600	L
C-C_3	0	5.32277800	8.43828900	5.15632600	L
O-O_3	0	6.67427200	8.30369100	4.78786900	L
C-C_3	0	7.41398000	9.25132400	5.52398600	L
C-C_3	0	6.47827800	10.06267000	6.39909400	L
C-C_3	0	5.23979300	9.22677900	6.44328800	L
H-H_	0	4.77759000	8.97045100	4.34799300	L
H-H_	0	4.86870500	7.43490400	5.28326600	L
H-H_	0	8.15364400	8.71435100	6.15564100	L
H-H_	0	7.96444800	9.91845400	4.82791200	L
H-H_	0	6.89948300	10.22661600	7.41478600	L
H-H_	0	6.25896000	11.04155700	5.91827400	L
H-H_	0	5.27818900	8.53979000	7.31596000	L
H-H_	0	4.32031200	9.84696800	6.49399500	L
C-C_3	0	-8.10406900	-5.78992200	-6.58325700	L
O-O_3	0	-8.23347200	-5.16595700	-7.84168100	L
C-C_3	0	-9.39605500	-4.37146300	-7.80669900	L
C-C_3	0	-9.85978700	-4.23321000	-6.37406000	L
C-C_3	0	-9.31131800	-5.46440100	-5.72752800	L
H-H_	0	-8.01020800	-6.88718000	-6.72294400	L
H-H_	0	-7.18266800	-5.41514400	-6.08912500	L
H-H_	0	-9.18318100	-3.37355800	-8.24404100	L
H-H_	0	-10.18646600	-4.86400200	-8.41441600	L
H-H_	0	-9.40044400	-3.33196700	-5.91593800	L
H-H_	0	-10.96755600	-4.17960700	-6.29794800	L

H-H_	0	-9.03492200	-5.28602300	-4.66578800	L
H-H_	0	-10.05590300	-6.28856200	-5.79159300	L
C-C_3	0	-6.05105600	0.31937500	-11.47701900	L
O-O_3	0	-5.19508400	0.89309000	-10.51979100	L
C-C_3	0	-4.25835300	-0.08958700	-10.14146600	L
C-C_3	0	-4.56733800	-1.39262700	-10.85521300	L
C-C_3	0	-5.95275100	-1.18434700	-11.37970100	L
H-H_	0	-7.09541800	0.64988100	-11.29655100	L
H-H_	0	-5.74053400	0.65918500	-12.48922400	L
H-H_	0	-3.23537300	0.25815700	-10.39327000	L
H-H_	0	-4.31562200	-0.23807700	-9.04231100	L
H-H_	0	-3.85747800	-1.54495400	-11.69788600	L
H-H_	0	-4.52327500	-2.25974300	-10.16511200	L
H-H_	0	-6.10084000	-1.67394500	-12.36708700	L
H-H_	0	-6.70025200	-1.56651500	-10.64968000	L
C-C_3	0	-9.22662700	-3.09239400	4.10701600	L
O-O_3	0	-9.75309300	-3.18343800	5.41034300	L
C-C_3	0	-10.28785100	-1.92290300	5.74540500	L
C-C_3	0	-10.19472300	-1.00111300	4.54834200	L
C-C_3	0	-9.13056100	-1.63761500	3.71379300	L
H-H_	0	-8.22879700	-3.57436800	4.06808200	L
H-H_	0	-9.90286000	-3.62467700	3.40601600	L
H-H_	0	-11.34425400	-2.03731700	6.06870200	L
H-H_	0	-9.70599500	-1.50130800	6.59164100	L
H-H_	0	-11.16058700	-0.99509600	4.00058800	L
H-H_	0	-9.92556900	0.03577400	4.83836000	L
H-H_	0	-9.30749300	-1.49163700	2.62667900	L
H-H_	0	-8.13514600	-1.23081800	3.99737900	L
C-C_3	0	10.26660700	-2.88303100	4.92475100	L
O-O_3	0	9.20777500	-3.80646600	4.82026500	L
C-C_3	0	9.71936100	-5.07871500	5.14270100	L
C-C_3	0	11.12398200	-4.93434000	5.68714600	L
C-C_3	0	11.56984500	-3.62765700	5.11449400	L
H-H_	0	10.31086800	-2.25592200	4.01092300	L
H-H_	0	10.07679700	-2.22624000	5.79800900	L
H-H_	0	9.05942100	-5.57230800	5.88739800	L
H-H_	0	9.74112400	-5.69669000	4.22176800	L
H-H_	0	11.09680000	-4.87421900	6.79779800	L

H-H_	0	11.78047000	-5.77241800	5.36683600	L
H-H_	0	12.26104500	-3.08942600	5.79743400	L
H-H_	0	12.06314100	-3.78940600	4.13074400	L
C-C_3	0	-5.18280700	5.96497200	8.74005100	L
O-O_3	0	-6.34995800	6.55593400	9.26024200	L
C-C_3	0	-6.62002200	5.94957400	10.50335600	L
C-C_3	0	-5.51727400	4.96597700	10.84226100	L
C-C_3	0	-4.86817200	4.70749400	9.51977000	L
H-H_	0	-5.33100500	5.73266900	7.66570500	L
H-H_	0	-4.34445600	6.68708500	8.83570500	L
H-H_	0	-6.69753500	6.73019100	11.29003900	L
H-H_	0	-7.59446200	5.41980200	10.43782900	L
H-H_	0	-4.78755100	5.43729400	11.53734200	L
H-H_	0	-5.92171100	4.03515800	11.29461400	L
H-H_	0	-3.77451400	4.54301600	9.62071900	L
H-H_	0	-5.33389000	3.82637100	9.02947900	L
C-C_3	0	-6.33876800	6.15743500	4.50684100	L
O-O_3	0	-6.34861200	7.53348600	4.78775400	L
C-C_3	0	-7.31938000	7.74626700	5.78694000	L
C-C_3	0	-8.16298100	6.49328300	5.95925200	L
C-C_3	0	-7.68835500	5.58352900	4.86799800	L
H-H_	0	-6.12045600	5.99506300	3.43083700	L
H-H_	0	-5.54289200	5.67249600	5.11013700	L
H-H_	0	-6.81408900	8.00841400	6.74124800	L
H-H_	0	-7.96264500	8.60012900	5.48580800	L
H-H_	0	-7.96840600	6.03303600	6.95262100	L
H-H_	0	-9.24812900	6.71294100	5.85738100	L
H-H_	0	-7.61181800	4.53096700	5.21349200	L
H-H_	0	-8.37280800	5.64396200	3.99404900	L
C-C_3	0	1.95352700	-10.48697600	7.07282700	L
O-O_3	0	0.90784400	-9.86905800	7.78386500	L
C-C_3	0	-0.14688300	-10.80040300	7.88076300	L
C-C_3	0	0.23456700	-12.08179800	7.16207700	L
C-C_3	0	1.39566800	-11.66866000	6.31430100	L
H-H_	0	2.41631300	-9.76059900	6.37419100	L
H-H_	0	2.72923600	-10.82733100	7.79318500	L
H-H_	0	-0.36570100	-11.00478400	8.95065100	L
H-H_	0	-1.05554400	-10.36419200	7.41506900	L

H-H_	0	0.55752000	-12.84995200	7.89879300	L
H-H_	0	-0.60127000	-12.48045000	6.54745600	L
H-H_	0	2.14426700	-12.48337300	6.20745300	L
H-H_	0	1.04036800	-11.34429300	5.31111600	L
C-C_3	0	4.33594700	5.71707100	-7.00486400	L
O-O_3	0	5.27966600	6.69113300	-7.37441100	L
C-C_3	0	6.29370700	6.68849600	-6.39544400	L
C-C_3	0	5.88616600	5.80287100	-5.23011800	L
C-C_3	0	4.45995100	5.45480300	-5.52155100	L
H-H_	0	3.31293800	6.07261200	-7.25009300	L
H-H_	0	4.53613500	4.78633400	-7.57823400	L
H-H_	0	7.24352600	6.32728800	-6.84337400	L
H-H_	0	6.45453200	7.72953000	-6.04505700	L
H-H_	0	6.50524700	4.88176000	-5.20672300	L
H-H_	0	5.98481200	6.33532100	-4.26142600	L
H-H_	0	4.23747600	4.39613900	-5.27087900	L
H-H_	0	3.77871600	6.12673300	-4.95612000	L
C-C_3	0	-6.41966500	10.29030200	0.15728400	L
O-O_3	0	-6.54232400	10.90190800	1.42130800	L
C-C_3	0	-5.76595900	10.16195900	2.33522600	L
C-C_3	0	-5.32491800	8.86585600	1.69005100	L
C-C_3	0	-5.39918800	9.17447300	0.23065100	L
H-H_	0	-7.40805500	9.88299900	-0.14354300	L
H-H_	0	-6.11574800	11.04574000	-0.59640000	L
H-H_	0	-4.87635500	10.76495000	2.61927300	L
H-H_	0	-6.36170300	9.95599500	3.24945400	L
H-H_	0	-4.29784500	8.57752800	1.99844800	L
H-H_	0	-6.03401800	8.04926900	1.94096800	L
H-H_	0	-4.41327500	9.53733800	-0.12576300	L
H-H_	0	-5.71291200	8.29196200	-0.36382000	L
C-C_3	0	-0.31711100	5.13033300	9.98258200	L
O-O_3	0	-0.71741900	3.81547900	9.69149700	L
C-C_3	0	-1.05064100	3.78420200	8.32430400	L
C-C_3	0	-1.27187000	5.20139500	7.82134500	L
C-C_3	0	-1.01189000	6.06436900	9.02215100	L
H-H_	0	-0.58344600	5.38398100	11.02943700	L
H-H_	0	0.78503000	5.21335300	9.87124500	L
H-H_	0	-0.22795800	3.29743100	7.75779400	L

H-H_	0	-1.97269300	3.18129600	8.19046200	L
H-H_	0	-0.54998300	5.43714400	7.01039400	L
H-H_	0	-2.30903400	5.34341200	7.44613400	L
H-H_	0	-0.37352400	6.93971300	8.77468900	L
H-H_	0	-1.97186000	6.41137900	9.46185900	L
C-C_3	0	13.05190500	3.84317800	-2.12737700	L
O-O_3	0	14.35636900	3.94150400	-2.65091600	L
C-C_3	0	15.26196800	3.83351500	-1.57653000	L
C-C_3	0	14.51530400	3.42973700	-0.32314600	L
C-C_3	0	13.11130500	3.85023400	-0.61458700	L
H-H_	0	12.43779100	4.69274300	-2.49517400	L
H-H_	0	12.59072900	2.89670300	-2.48297200	L
H-H_	0	16.04314500	3.08183200	-1.82028500	L
H-H_	0	15.75359500	4.81819200	-1.42224400	L
H-H_	0	14.56399800	2.32823500	-0.18930800	L
H-H_	0	14.91765300	3.93539400	0.58151100	L
H-H_	0	12.37112800	3.15001400	-0.17131300	L
H-H_	0	12.93874600	4.88098700	-0.23347600	L
C-C_3	0	10.13659100	-0.22477200	-2.85283300	L
O-O_3	0	10.24483600	0.70946800	-3.90309500	L
C-C_3	0	10.40899600	-0.01093200	-5.10145200	L
C-C_3	0	10.75663400	-1.44612400	-4.77531100	L
C-C_3	0	10.17225100	-1.63267300	-3.41149500	L
H-H_	0	10.98512000	-0.07507700	-2.15252700	L
H-H_	0	9.18903600	-0.05591200	-2.30192400	L
H-H_	0	9.46014100	0.02934500	-5.67722500	L
H-H_	0	11.21181500	0.45397000	-5.71224700	L
H-H_	0	10.32573500	-2.15430700	-5.51370700	L
H-H_	0	11.85969100	-1.57012300	-4.73549800	L
H-H_	0	9.14187100	-2.03928500	-3.48960200	L
H-H_	0	10.78971200	-2.30981500	-2.78438700	L
C-C_3	0	-8.27431700	1.31929300	6.85937000	L
O-O_3	0	-9.41136300	1.05069900	7.64403400	L
C-C_3	0	-9.41971600	1.96223900	8.71865600	L
C-C_3	0	-8.22883200	2.89372300	8.61179300	L
C-C_3	0	-7.32959100	2.20496300	7.63624600	L
H-H_	0	-7.78076000	0.36470900	6.58152300	L
H-H_	0	-8.58888300	1.83408800	5.92840000	L

H-H_	0	-10.36716000	2.54224800	8.70601000	L
H-H_	0	-9.36931200	1.39273600	9.67119700	L
H-H_	0	-8.54752900	3.87975300	8.20804600	L
H-H_	0	-7.73143700	3.03324800	9.59545900	L
H-H_	0	-6.81139500	2.93046400	6.97552400	L
H-H_	0	-6.58410500	1.58352500	8.17520300	L
C-C_3	0	8.85998900	0.70943800	4.80897600	L
O-O_3	0	8.70724700	1.39435200	6.03091600	L
C-C_3	0	7.63024700	0.80136600	6.71796500	L
C-C_3	0	7.23764100	-0.47964500	6.01896900	L
C-C_3	0	7.70312000	-0.24671100	4.61997400	L
H-H_	0	8.90674800	1.43517800	3.97481000	L
H-H_	0	9.81469200	0.14657300	4.83150600	L
H-H_	0	7.92002200	0.59980200	7.76990200	L
H-H_	0	6.77446400	1.50641500	6.71760300	L
H-H_	0	7.79077000	-1.33557300	6.45976400	L
H-H_	0	6.14430900	-0.66383900	6.07260800	L
H-H_	0	8.02025500	-1.18809000	4.12534900	L
H-H_	0	6.89694000	0.23596100	4.02814000	L
C-C_3	0	-11.11691000	4.10125700	5.98127900	L
O-O_3	0	-11.34922700	4.86012800	4.81900800	L
C-C_3	0	-12.53274700	4.36481600	4.24726700	L
C-C_3	0	-12.66026600	2.90332100	4.61029000	L
C-C_3	0	-11.89471100	2.79656000	5.89817000	L
H-H_	0	-10.03012900	3.89688300	6.07222200	L
H-H_	0	-11.43512300	4.68649700	6.87169000	L
H-H_	0	-13.39951200	4.93803100	4.64466100	L
H-H_	0	-12.50105200	4.48935100	3.14502900	L
H-H_	0	-13.72388200	2.60480700	4.73422500	L
H-H_	0	-12.18172900	2.27269400	3.83029900	L
H-H_	0	-12.58958300	2.70404600	6.76091300	L
H-H_	0	-11.21394700	1.91986100	5.87575400	L
C-C_3	0	-0.02146500	-7.45827200	10.11382900	L
O-O_3	0	-1.23173100	-7.19129800	10.78641700	L
C-C_3	0	-0.97544600	-6.19441300	11.74853800	L
C-C_3	0	0.51902000	-6.04472700	11.91830200	L
C-C_3	0	1.04594700	-6.49653700	10.59414200	L
H-H_	0	0.28630700	-8.50354600	10.33204400	L

H-H_	0	-0.17023000	-7.35556000	9.01853600	L
H-H_	0	-1.41208500	-5.23326700	11.39991300	L
H-H_	0	-1.45074700	-6.47145500	12.71308700	L
H-H_	0	0.80846400	-4.99777200	12.14968300	L
H-H_	0	0.87977500	-6.72381900	12.72239800	L
H-H_	0	1.11613500	-5.63362600	9.89671100	L
H-H_	0	2.03775400	-6.98905000	10.69096500	L
C-C_3	0	11.32565500	-1.11693300	-10.16893700	L
O-O_3	0	10.32073500	-0.73716200	-11.07771200	L
C-C_3	0	9.21438300	-0.34680000	-10.30539300	L
C-C_3	0	9.71937200	0.19250300	-8.98542600	L
C-C_3	0	11.08108000	-0.42688500	-8.83660200	L
H-H_	0	11.30699100	-2.22134300	-10.04442900	L
H-H_	0	12.31790900	-0.82701000	-10.57580100	L
H-H_	0	8.63369100	0.43043500	-10.84632100	L
H-H_	0	8.55594200	-1.22507500	-10.13722900	L
H-H_	0	9.81286100	1.29801900	-9.04280600	L
H-H_	0	9.04111400	-0.08836100	-8.15043900	L
H-H_	0	11.84814000	0.35565400	-8.64844900	L
H-H_	0	11.09792000	-1.16113400	-8.00252100	L
C-C_3	0	6.31417900	-2.93839500	-4.46806400	L
O-O_3	0	5.60972700	-2.56238800	-3.31278000	L
C-C_3	0	6.04740800	-1.26632200	-2.99186400	L
C-C_3	0	6.45037900	-0.56513200	-4.27105700	L
C-C_3	0	6.65019800	-1.68685900	-5.25667000	L
H-H_	0	7.24149200	-3.47426900	-4.17067500	L
H-H_	0	5.69168400	-3.62708400	-5.07746600	L
H-H_	0	5.22944100	-0.71329600	-2.48562900	L
H-H_	0	6.91463000	-1.32862100	-2.29843500	L
H-H_	0	5.63457800	0.10982600	-4.60559800	L
H-H_	0	7.38008300	0.02517000	-4.12815200	L
H-H_	0	5.95751300	-1.57405100	-6.11646100	L
H-H_	0	7.69504900	-1.72084900	-5.63135700	L
C-C_3	0	2.37728200	5.24579700	5.04370000	L
O-O_3	0	3.09206200	5.37454700	6.24504000	L
C-C_3	0	2.52489400	6.46883800	6.92396000	L
C-C_3	0	1.81923000	7.37049100	5.92117900	L
C-C_3	0	1.91922300	6.62274600	4.62186400	L

H-H_	0	3.02430000	4.79410600	4.26318100	L
H-H_	0	1.50084300	4.58213100	5.21133500	L
H-H_	0	1.80468100	6.09453000	7.68152600	L
H-H_	0	3.32495300	7.02708800	7.45253500	L
H-H_	0	0.75565200	7.51633700	6.20897200	L
H-H_	0	2.31889900	8.35957500	5.84526000	L
H-H_	0	0.94382900	6.58462900	4.09535600	L
H-H_	0	2.68389700	7.09283800	3.96501900	L
C-C_3	0	2.01052200	-1.40443500	9.73274900	L
O-O_3	0	1.34294500	-0.23469700	10.14866700	L
C-C_3	0	0.23152200	-0.63104900	10.91468500	L
C-C_3	0	0.43357300	-2.05763400	11.36921400	L
C-C_3	0	1.30520300	-2.62492600	10.29417300	L
H-H_	0	2.04084000	-1.44302700	8.62278100	L
H-H_	0	3.05276600	-1.37177600	10.11441900	L
H-H_	0	0.12061100	0.03997200	11.79091900	L
H-H_	0	-0.68051600	-0.55596300	10.28674400	L
H-H_	0	0.97190300	-2.07329600	12.34264900	L
H-H_	0	-0.52935800	-2.60539000	11.45982900	L
H-H_	0	2.02949000	-3.36490800	10.69772800	L
H-H_	0	0.67577800	-3.10296800	9.51323600	L
C-C_3	0	6.04824200	1.86441000	-6.99680900	L
O-O_3	0	7.10353100	2.16772400	-6.11260000	L
C-C_3	0	8.07172600	2.89404000	-6.83350100	L
C-C_3	0	7.50930000	3.27500000	-8.18733200	L
C-C_3	0	6.43808200	2.25616500	-8.40582200	L
H-H_	0	5.81794900	0.78048600	-6.95327700	L
H-H_	0	5.14561200	2.42786800	-6.68106300	L
H-H_	0	8.36500000	3.80179000	-6.26617200	L
H-H_	0	8.97467300	2.26056200	-6.95747900	L
H-H_	0	7.05930600	4.28965000	-8.14340800	L
H-H_	0	8.28237000	3.24227600	-8.98470700	L
H-H_	0	5.57780200	2.67382600	-8.97282400	L
H-H_	0	6.85510400	1.37718000	-8.94460600	L
C-C_3	0	-2.64062700	7.65602700	-1.59875700	L
O-O_3	0	-2.35500400	9.02684200	-1.71424800	L
C-C_3	0	-1.40618200	9.15986600	-2.73923100	L
C-C_3	0	-0.62526800	7.86761100	-2.85890200	L

C-C_3	0	-1.48005700	6.85269900	-2.15047200	L
H-H_	0	-2.80246400	7.40894200	-0.52869300	L
H-H_	0	-3.57094900	7.42669100	-2.16114700	L
H-H_	0	-1.92893200	9.38878800	-3.68958900	L
H-H_	0	-0.72562400	10.00255400	-2.50117200	L
H-H_	0	-0.46219100	7.59345500	-3.92255200	L
H-H_	0	0.35816400	7.96745900	-2.35261200	L
H-H_	0	-1.84399900	6.06994100	-2.84976500	L
H-H_	0	-0.91507200	6.36847000	-1.32594400	L
C-C_3	0	-5.09564100	-3.06101400	5.46558700	L
O-O_3	0	-3.88194400	-3.77618100	5.50280000	L
C-C_3	0	-3.30058300	-3.67578600	4.22740700	L
C-C_3	0	-3.88042000	-2.46917100	3.53359900	L
C-C_3	0	-5.25782400	-2.40340200	4.10926000	L
H-H_	0	-5.94088600	-3.75314100	5.66124000	L
H-H_	0	-5.07327900	-2.28466800	6.25849800	L
H-H_	0	-2.20068000	-3.58888600	4.31367600	L
H-H_	0	-3.53417700	-4.59816100	3.65545100	L
H-H_	0	-3.30603700	-1.55767000	3.80437100	L
H-H_	0	-3.89180300	-2.59422000	2.43526200	L
H-H_	0	-5.62558400	-1.36066600	4.18988500	L
H-H_	0	-5.95898900	-2.99538800	3.48519800	L
C-C_3	0	-12.97126900	3.93672100	-1.63497700	L
O-O_3	0	-12.52545700	5.07531400	-0.93645400	L
C-C_3	0	-12.35106100	6.11330100	-1.87542100	L
C-C_3	0	-12.77299600	5.63537300	-3.25090500	L
C-C_3	0	-12.75251200	4.14660000	-3.11602200	L
H-H_	0	-12.41688800	3.04115300	-1.28654600	L
H-H_	0	-14.05294600	3.78685500	-1.42754300	L
H-H_	0	-12.95160200	6.99683200	-1.57033400	L
H-H_	0	-11.27963900	6.40735200	-1.89230900	L
H-H_	0	-13.80553500	5.98237000	-3.47540900	L
H-H_	0	-12.07553600	5.98631400	-4.04150300	L
H-H_	0	-13.54482200	3.66140600	-3.72647400	L
H-H_	0	-11.75654500	3.75491000	-3.40885300	L
C-C_3	0	-5.52644400	4.47193900	-7.85597500	L
O-O_3	0	-5.49155700	4.43345700	-6.45058500	L
C-C_3	0	-4.59901100	3.41068300	-6.07138000	L

C-C_3	0	-3.98083500	2.77849100	-7.30346800	L
C-C_3	0	-4.33477300	3.72435000	-8.40536500	L
H-H_	0	-6.46434800	3.99350600	-8.20501200	L
H-H_	0	-5.51587600	5.52698500	-8.20141500	L
H-H_	0	-3.80725200	3.85159800	-5.42990000	L
H-H_	0	-5.14015900	2.64229100	-5.48096900	L
H-H_	0	-2.88009700	2.67267500	-7.20119300	L
H-H_	0	-4.43665900	1.78191800	-7.49072100	L
H-H_	0	-3.49716600	4.42731500	-8.59355100	L
H-H_	0	-4.58235300	3.18651100	-9.34240800	L
C-C_3	0	-10.47798000	-10.40826400	-3.50372600	L
O-O_3	0	-9.54309800	-11.12393900	-4.27919000	L
C-C_3	0	-8.53791600	-10.21954700	-4.67809200	L
C-C_3	0	-8.74009600	-8.89692100	-3.96956800	L
C-C_3	0	-10.19144600	-8.92739700	-3.61864400	L
H-H_	0	-11.50791400	-10.63208500	-3.85459500	L
H-H_	0	-10.38583600	-10.73074600	-2.44460500	L
H-H_	0	-7.53993900	-10.64147900	-4.43929300	L
H-H_	0	-8.60368500	-10.07154700	-5.77808000	L
H-H_	0	-8.12614400	-8.85740200	-3.04497600	L
H-H_	0	-8.48934200	-8.03588200	-4.62091100	L
H-H_	0	-10.39935200	-8.38963700	-2.67111100	L
H-H_	0	-10.79165200	-8.48760200	-4.44632500	L
C-C_3	0	-0.51454300	-7.75529700	3.46554800	L
O-O_3	0	-1.62738300	-6.90971600	3.60222900	L
C-C_3	0	-1.78228200	-6.69520500	4.98067300	L
C-C_3	0	-1.26730400	-7.91208500	5.72055700	L
C-C_3	0	-0.39394800	-8.60740200	4.71231100	L
H-H_	0	-0.64483700	-8.39663000	2.56920100	L
H-H_	0	0.40035100	-7.13606900	3.33809100	L
H-H_	0	-1.20856500	-5.79056400	5.27418400	L
H-H_	0	-2.85288300	-6.52860200	5.21235100	L
H-H_	0	-0.69252700	-7.61653900	6.62302000	L
H-H_	0	-2.11382500	-8.56822000	6.01916100	L
H-H_	0	0.66042900	-8.65613100	5.05250000	L
H-H_	0	-0.76257900	-9.63520700	4.51964400	L
C-C_3	0	2.94491600	-6.95024300	7.32489800	L
O-O_3	0	2.04579700	-6.74913800	6.26391600	L

C-C_3	0	1.56197300	-5.43805200	6.39960400	L
C-C_3	0	2.63260200	-4.59761100	7.06046400	L
C-C_3	0	3.50223600	-5.60731600	7.76071300	L
H-H_	0	2.41342900	-7.44272400	8.16627600	L
H-H_	0	3.76573000	-7.62036200	6.99228400	L
H-H_	0	1.30865500	-5.03140700	5.39841100	L
H-H_	0	0.64342100	-5.44862400	7.02579500	L
H-H_	0	3.21688100	-4.04327700	6.29455500	L
H-H_	0	2.18844600	-3.87419400	7.77426400	L
H-H_	0	4.56100800	-5.50316300	7.43988500	L
H-H_	0	3.43954400	-5.49348200	8.86463700	L
C-C_3	0	2.04430500	-10.45673000	2.00438800	L
O-O_3	0	2.72839000	-11.40905700	2.78663800	L
C-C_3	0	3.76785700	-10.73826400	3.46262100	L
C-C_3	0	3.61022600	-9.24592000	3.26868500	L
C-C_3	0	2.82251100	-9.15895700	2.00292600	L
H-H_	0	1.92261100	-10.83633700	0.96899000	L
H-H_	0	1.03564700	-10.29388500	2.44103000	L
H-H_	0	3.74479600	-10.99531100	4.54201400	L
H-H_	0	4.74102400	-11.07209900	3.04401600	L
H-H_	0	3.02972800	-8.80906700	4.11049900	L
H-H_	0	4.59031700	-8.73474200	3.17812800	L
H-H_	0	2.15241700	-8.27451100	1.98653200	L
H-H_	0	3.51407400	-9.12680300	1.13250700	L
C-C_3	0	0.14225100	1.65542800	-7.63003000	L
O-O_3	0	0.86987900	2.47356900	-6.75237100	L
C-C_3	0	1.91093100	3.03515800	-7.51000600	L
C-C_3	0	2.23980000	2.10486600	-8.66266500	L
C-C_3	0	1.08287000	1.14445400	-8.70267400	L
H-H_	0	-0.30790800	0.81226500	-7.06672900	L
H-H_	0	-0.67590400	2.24967100	-8.08710600	L
H-H_	0	1.59256000	4.02702900	-7.89518900	L
H-H_	0	2.79779000	3.17521900	-6.86120700	L
H-H_	0	2.32511100	2.67226300	-9.61574000	L
H-H_	0	3.19079700	1.56180000	-8.46811400	L
H-H_	0	0.59034400	1.13725000	-9.69897700	L
H-H_	0	1.42912400	0.11964600	-8.45375300	L
C-C_3	0	-3.69527400	9.42609100	5.36718400	L

O-O_3	0	-2.69571800	10.41571400	5.42951400	L
C-C_3	0	-1.83014100	10.08741700	6.49314700	L
C-C_3	0	-2.28392900	8.79676200	7.14457200	L
C-C_3	0	-3.68298400	8.62250300	6.64661000	L
H-H_	0	-4.68644100	9.90530600	5.22359200	L
H-H_	0	-3.49460800	8.76068100	4.50015600	L
H-H_	0	-0.79279300	9.98118400	6.11050500	L
H-H_	0	-1.84766500	10.91329800	7.23633500	L
H-H_	0	-1.64961700	7.95506700	6.79683700	L
H-H_	0	-2.24910800	8.85931900	8.25394100	L
H-H_	0	-3.92481100	7.55407300	6.46107800	L
H-H_	0	-4.40399200	9.05468000	7.37523900	L
C-C_3	0	-1.38060800	10.31043300	2.26676400	L
O-O_3	0	-1.38745700	9.82086700	0.94597700	L
C-C_3	0	-0.22144600	9.05257800	0.76768500	L
C-C_3	0	0.69228700	9.23770600	1.95796200	L
C-C_3	0	-0.24779500	9.66580900	3.03712500	L
H-H_	0	-1.24220400	11.41279100	2.24109900	L
H-H_	0	-2.35460500	10.09065000	2.74669000	L
H-H_	0	-0.50826100	7.98555300	0.67055900	L
H-H_	0	0.29189200	9.36056600	-0.16582300	L
H-H_	0	1.22616000	8.30005900	2.21992600	L
H-H_	0	1.42679200	10.04516800	1.74758800	L
H-H_	0	-0.61686400	8.77976200	3.59425600	L
H-H_	0	0.22711300	10.37607800	3.74278000	L
C-C_3	0	5.75814700	-8.11937200	-5.39599500	L
O-O_3	0	5.52694500	-6.73425200	-5.27902300	L
C-C_3	0	4.90933500	-6.50252200	-4.03585800	L
C-C_3	0	4.46071700	-7.82069900	-3.44647700	L
C-C_3	0	5.35315800	-8.81688200	-4.11381500	L
H-H_	0	5.15814100	-8.51075900	-6.24477900	L
H-H_	0	6.83253000	-8.30064900	-5.61262800	L
H-H_	0	5.63883400	-6.01006500	-3.35952400	L
H-H_	0	4.04459300	-5.81958600	-4.16923700	L
H-H_	0	4.57483400	-7.83811300	-2.34123500	L
H-H_	0	3.40373500	-8.01842500	-3.72458200	L
H-H_	0	6.24843400	-9.01023100	-3.48545600	L
H-H_	0	4.82228800	-9.77308000	-4.31425000	L

C-C_3	0	-0.76519000	4.01956000	-10.84447700	L
O-O_3	0	-1.52401600	5.20066400	-10.93981600	L
C-C_3	0	-2.71182400	4.88423800	-11.63260900	L
C-C_3	0	-2.75626900	3.39393600	-11.92069500	L
C-C_3	0	-1.68452000	2.83654300	-11.03644700	L
H-H_	0	0.01784900	4.02663100	-11.63450500	L
H-H_	0	-0.26475200	3.97087900	-9.85549700	L
H-H_	0	-3.58180400	5.17560700	-11.00619400	L
H-H_	0	-2.75536300	5.46365900	-12.57978600	L
H-H_	0	-3.75060600	2.95569800	-11.68929200	L
H-H_	0	-2.50467700	3.20428900	-12.98781500	L
H-H_	0	-2.11806600	2.53025500	-10.05987500	L
H-H_	0	-1.16160700	1.97577300	-11.50668700	L
C-C_3	0	12.34202900	0.12582500	3.07925700	L
O-O_3	0	13.14089200	-0.86683300	3.68088300	L
C-C_3	0	14.23080900	-1.11913000	2.82516100	L
C-C_3	0	13.99948600	-0.42935300	1.49761700	L
C-C_3	0	13.03839000	0.66095600	1.84614200	L
H-H_	0	11.36923800	-0.32406700	2.79598200	L
H-H_	0	12.15254800	0.94440700	3.80466700	L
H-H_	0	15.15623400	-0.72599000	3.29916400	L
H-H_	0	14.34572900	-2.21417100	2.67917900	L
H-H_	0	14.94326600	-0.02766100	1.06925100	L
H-H_	0	13.52893000	-1.13350200	0.77797300	L
H-H_	0	13.59296500	1.59178900	2.09800000	L
H-H_	0	12.32322600	0.85776700	1.01848600	L
C-C_3	0	-11.00071300	7.11421600	1.32273900	L
O-O_3	0	-9.93892800	6.57736000	0.56876100	L
C-C_3	0	-8.73960300	7.12396300	1.06825400	L
C-C_3	0	-9.05979600	8.17401500	2.11220400	L
C-C_3	0	-10.44201000	7.80650300	2.54579500	L
H-H_	0	-11.55344300	7.84535900	0.69368400	L
H-H_	0	-11.69888800	6.30385200	1.61848200	L
H-H_	0	-8.13588000	6.31164000	1.52624500	L
H-H_	0	-8.15558000	7.56398100	0.23347800	L
H-H_	0	-8.34351700	8.15022200	2.95927500	L
H-H_	0	-9.06365200	9.18501500	1.64902200	L
H-H_	0	-10.39689200	7.09478900	3.39908700	L

H-H_	0	-11.04008900	8.70017900	2.82711100	L
C-C_3	0	2.44793700	9.00115100	-5.32405500	L
O-O_3	0	3.81275400	8.79619500	-5.59125300	L
C-C_3	0	4.54335100	9.38755100	-4.53963500	L
C-C_3	0	3.59001100	10.05399300	-3.56732800	L
C-C_3	0	2.31389200	10.13681600	-4.33985100	L
H-H_	0	1.91092700	9.23975500	-6.26542000	L
H-H_	0	2.01826600	8.07109100	-4.89169700	L
H-H_	0	5.14453500	8.61434100	-4.01964300	L
H-H_	0	5.23741500	10.14274400	-4.96683500	L
H-H_	0	3.44483500	9.41255100	-2.67155200	L
H-H_	0	3.95306000	11.05723500	-3.25736100	L
H-H_	0	1.42521900	10.01335500	-3.68760500	L
H-H_	0	2.26198500	11.10566700	-4.88339200	L
C-C_3	0	-1.11899800	-11.21645400	-0.65557700	L
O-O_3	0	-0.37489200	-10.81672900	-1.77830500	L
C-C_3	0	-1.29925900	-10.35733900	-2.73660200	L
C-C_3	0	-2.62996900	-10.06724700	-2.06155700	L
C-C_3	0	-2.39568200	-10.41075300	-0.62024000	L
H-H_	0	-1.34984200	-12.30148400	-0.73540900	L
H-H_	0	-0.52766600	-11.04478400	0.26845000	L
H-H_	0	-0.90252600	-9.43582200	-3.21246900	L
H-H_	0	-1.42635100	-11.13188100	-3.52373600	L
H-H_	0	-2.91589700	-8.99918000	-2.17272500	L
H-H_	0	-3.42852200	-10.71203900	-2.48999600	L
H-H_	0	-2.24032300	-9.48539500	-0.02586400	L
H-H_	0	-3.23670500	-10.99507400	-0.19100600	L
C-C_3	0	11.60681600	-1.72558100	8.62364300	L
O-O_3	0	12.97811400	-1.84412900	8.31695900	L
C-C_3	0	13.34339100	-0.72479500	7.54343200	L
C-C_3	0	12.09364500	-0.02942100	7.05318100	L
C-C_3	0	11.07187500	-0.41861100	8.07323200	L
H-H_	0	11.06179300	-2.57972800	8.16708800	L
H-H_	0	11.46488700	-1.76875700	9.72457600	L
H-H_	0	13.94065100	-0.03072200	8.17392700	L
H-H_	0	13.96760100	-1.05007000	6.68381100	L
H-H_	0	12.22483000	1.07295000	6.99472700	L
H-H_	0	11.80864100	-0.42936700	6.05763500	L

H-H_	0	11.02569300	0.34710000	8.87894900	L
H-H_	0	10.06876700	-0.54805100	7.61829000	L
C-C_3	0	7.93858900	4.08814100	8.65698900	L
O-O_3	0	8.77620400	4.53989700	7.62384800	L
C-C_3	0	8.29080200	5.80424500	7.23651300	L
C-C_3	0	6.86373300	5.97397200	7.73674000	L
C-C_3	0	6.56760200	4.68593400	8.44703900	L
H-H_	0	7.88760300	2.98028100	8.64597100	L
H-H_	0	8.35466000	4.41382800	9.63606200	L
H-H_	0	8.94679500	6.59503400	7.66166700	L
H-H_	0	8.32447900	5.88529600	6.12977500	L
H-H_	0	6.80263800	6.83011300	8.44394700	L
H-H_	0	6.15418500	6.13227500	6.89849700	L
H-H_	0	6.03927900	4.85289600	9.41070000	L
H-H_	0	5.96093100	4.02222200	7.79406200	L
C-C_3	0	6.65922000	-5.27752700	1.03961900	L
O-O_3	0	6.87637000	-3.90260800	0.82274500	L
C-C_3	0	6.14525400	-3.54913600	-0.32306300	L
C-C_3	0	5.01934500	-4.53728300	-0.49454700	L
C-C_3	0	5.60512000	-5.79146400	0.07360100	L
H-H_	0	7.61365100	-5.82748300	0.91719900	L
H-H_	0	6.31009800	-5.42098300	2.07802800	L
H-H_	0	5.74143300	-2.52287800	-0.21549100	L
H-H_	0	6.82204700	-3.58473600	-1.20312700	L
H-H_	0	4.14521600	-4.21397300	0.10960700	L
H-H_	0	4.72167800	-4.65240800	-1.55808400	L
H-H_	0	4.83883500	-6.41051400	0.58919800	L
H-H_	0	6.08301900	-6.38398000	-0.73435200	L
C-C_3	0	-3.93931700	-3.45890500	-7.32593500	L
O-O_3	0	-5.29921600	-3.73233900	-7.56375200	L
C-C_3	0	-6.00882500	-2.55672300	-7.26872100	L
C-C_3	0	-5.22728000	-1.76329900	-6.24895700	L
C-C_3	0	-3.81214300	-2.21428800	-6.46565500	L
H-H_	0	-3.47882700	-4.32812800	-6.80936900	L
H-H_	0	-3.41660200	-3.31464800	-8.29493400	L
H-H_	0	-6.13882700	-1.96602000	-8.19925900	L
H-H_	0	-7.00959300	-2.81453800	-6.86669500	L
H-H_	0	-5.33953400	-0.66889100	-6.40459800	L

H-H_	0	-5.56552500	-2.02677700	-5.22318500	L
H-H_	0	-3.23396500	-1.42810100	-6.99782600	L
H-H_	0	-3.31536400	-2.45070600	-5.50167700	L
C-C_3	0	-10.43433700	-1.25060200	-3.35525500	L
O-O_3	0	-9.64610600	-0.09799200	-3.20794800	L
C-C_3	0	-10.53595100	0.93842300	-2.88649200	L
C-C_3	0	-11.86811000	0.64380700	-3.54528500	L
C-C_3	0	-11.81596900	-0.83425900	-3.82497600	L
H-H_	0	-9.96045000	-1.93290700	-4.08902700	L
H-H_	0	-10.49864400	-1.77799800	-2.37957400	L
H-H_	0	-10.64786800	0.99413500	-1.78235300	L
H-H_	0	-10.13041000	1.90370200	-3.24798100	L
H-H_	0	-12.71354000	0.90326800	-2.87133800	L
H-H_	0	-11.96054700	1.21316100	-4.49656200	L
H-H_	0	-12.60725800	-1.37737200	-3.26521600	L
H-H_	0	-11.93281900	-1.03032000	-4.91321500	L
C-C_3	0	-5.67524800	3.69544100	1.01168400	L
O-O_3	0	-6.20715000	4.74702000	0.24414000	L
C-C_3	0	-5.11687500	5.37291600	-0.37630300	L
C-C_3	0	-4.04767400	4.33171200	-0.61237100	L
C-C_3	0	-4.30667400	3.31809100	0.46733200	L
H-H_	0	-5.59878500	4.02569800	2.06692800	L
H-H_	0	-6.36078300	2.82627300	0.96917700	L
H-H_	0	-5.43949600	5.82842100	-1.33606400	L
H-H_	0	-4.72970100	6.17083100	0.29315000	L
H-H_	0	-4.17966400	3.87436100	-1.61671600	L
H-H_	0	-3.03112800	4.76900400	-0.54069600	L
H-H_	0	-4.30764400	2.28693800	0.05360600	L
H-H_	0	-3.54006700	3.39129200	1.26834400	L
C-C_3	0	4.51808300	1.58499300	9.10206100	L
O-O_3	0	3.97100700	2.66522900	9.81511800	L
C-C_3	0	2.75052500	3.00659200	9.19812700	L
C-C_3	0	2.55335600	2.17694300	7.94243100	L
C-C_3	0	3.88673100	1.53164800	7.73083400	L
H-H_	0	5.61798000	1.70810600	9.01963600	L
H-H_	0	4.30993100	0.64509900	9.65551200	L
H-H_	0	1.91602900	2.83345300	9.91030400	L
H-H_	0	2.77638800	4.08497200	8.93575000	L

H-H_	0	1.77297000	1.40266800	8.10650900	L
H-H_	0	2.26505200	2.81002600	7.07639200	L
H-H_	0	3.79025900	0.48722500	7.36677600	L
H-H_	0	4.48342900	2.13118500	7.01048500	L
C-C_3	0	-7.25651700	2.83161100	-2.49056000	L
O-O_3	0	-8.18001700	3.16696500	-1.48057800	L
C-C_3	0	-8.81018900	4.36609300	-1.86158400	L
C-C_3	0	-8.56020300	4.60890800	-3.32875700	L
C-C_3	0	-7.26335900	3.90341500	-3.56154100	L
H-H_	0	-6.24516200	2.72970200	-2.04863700	L
H-H_	0	-7.54371700	1.85326100	-2.92988000	L
H-H_	0	-9.89812700	4.29872900	-1.66166300	L
H-H_	0	-8.38928800	5.20334300	-1.26487800	L
H-H_	0	-9.36078400	4.13293000	-3.93191400	L
H-H_	0	-8.50259900	5.69153300	-3.56677800	L
H-H_	0	-7.21973800	3.46790400	-4.57966600	L
H-H_	0	-6.41137600	4.60164000	-3.40898100	L
C-C_3	0	1.74029000	-5.90405900	-0.29140000	L
O-O_3	0	2.20300400	-6.65021200	-1.38642800	L
C-C_3	0	1.52150900	-7.88033700	-1.34181400	L
C-C_3	0	0.22719600	-7.70477300	-0.56764200	L
C-C_3	0	0.31668200	-6.31490500	-0.00494700	L
H-H_	0	2.38291400	-6.10968600	0.59259000	L
H-H_	0	1.79002700	-4.82159400	-0.52480100	L
H-H_	0	1.30889900	-8.22567000	-2.37427300	L
H-H_	0	2.16699700	-8.63853900	-0.85047300	L
H-H_	0	-0.65244100	-7.79436700	-1.24071900	L
H-H_	0	0.15438300	-8.45914900	0.24451600	L
H-H_	0	-0.38893000	-5.64607400	-0.53769500	L
H-H_	0	0.10174900	-6.28632200	1.08261300	L
C-C_3	0	6.16842700	3.86679200	4.70685900	L
O-O_3	0	6.42989600	5.19491100	4.32914800	L
C-C_3	0	5.97623700	5.35264800	3.00543100	L
C-C_3	0	5.57593200	4.00416900	2.43416000	L
C-C_3	0	6.04632700	3.02457000	3.46082600	L
H-H_	0	6.99406400	3.49565500	5.34711100	L
H-H_	0	5.22246400	3.83187000	5.28784800	L
H-H_	0	5.11422400	6.05227000	2.99423900	L

H-H_	0	6.79387900	5.79313300	2.39679700	L
H-H_	0	4.47170600	3.94435400	2.32302300	L
H-H_	0	6.06370200	3.81868400	1.45509200	L
H-H_	0	5.33896000	2.18086800	3.58935700	L
H-H_	0	7.03876000	2.62586800	3.17465600	L
C-C_3	0	-2.37540300	3.63608600	12.84715600	L
O-O_3	0	-1.62892100	4.58851400	13.57001000	L
C-C_3	0	-0.38210600	4.01191200	13.88047800	L
C-C_3	0	-0.18382200	2.76736000	13.04420500	L
C-C_3	0	-1.58405600	2.34909900	12.72359400	L
H-H_	0	-3.34123200	3.44934500	13.36356800	L
H-H_	0	-2.59283300	4.04320700	11.83727600	L
H-H_	0	0.42903000	4.74439600	13.68169200	L
H-H_	0	-0.36653800	3.74909200	14.96071100	L
H-H_	0	0.36521700	3.01777800	12.11029200	L
H-H_	0	0.36498900	1.97839400	13.60220700	L
H-H_	0	-1.65655700	1.91756100	11.70220900	L
H-H_	0	-1.94193200	1.60849000	13.47263900	L
C-C_3	0	2.29206900	13.06392700	-1.80614500	L
O-O_3	0	1.59949700	14.23905300	-1.47161800	L
C-C_3	0	0.35716100	13.82623900	-0.96024300	L
C-C_3	0	0.50931000	12.43814500	-0.36477300	L
C-C_3	0	1.84203500	11.96005300	-0.87209000	L
H-H_	0	2.06604600	12.79512000	-2.86109500	L
H-H_	0	3.38571700	13.23225500	-1.70975400	L
H-H_	0	0.01736900	14.54475900	-0.18444300	L
H-H_	0	-0.39205800	13.81554800	-1.78313800	L
H-H_	0	0.50710400	12.48442000	0.74586700	L
H-H_	0	-0.30972300	11.77269600	-0.70919300	L
H-H_	0	2.56060900	11.85416100	-0.03137000	L
H-H_	0	1.75088800	10.99067100	-1.40517000	L
C-C_3	0	-2.12948000	6.01258200	4.21769200	L
O-O_3	0	-1.30584700	4.89623400	4.46906200	L
C-C_3	0	-0.96110800	4.34150700	3.22473600	L
C-C_3	0	-1.17066400	5.38776400	2.15643800	L
C-C_3	0	-2.29189300	6.20067800	2.72082500	L
H-H_	0	-3.11687700	5.86568600	4.70204700	L
H-H_	0	-1.65113700	6.90952300	4.65942000	L

H-H_	0	0.09665800	4.00458400	3.23762100	L
H-H_	0	-1.61586900	3.46462400	3.03211700	L
H-H_	0	-0.25548300	6.00990800	2.05321300	L
H-H_	0	-1.42995900	4.94100400	1.17647900	L
H-H_	0	-2.21767700	7.26742300	2.42349900	L
H-H_	0	-3.26950200	5.78591700	2.38937400	L
C-C_3	0	-3.34862100	12.58763400	-0.12867200	L
O-O_3	0	-4.44466400	13.41744500	0.17745200	L
C-C_3	0	-4.95654400	13.91628700	-1.03794600	L
C-C_3	0	-4.09883500	13.43184900	-2.19083900	L
C-C_3	0	-3.36573000	12.26764700	-1.60726500	L
H-H_	0	-2.41083300	13.12180900	0.13539000	L
H-H_	0	-3.40602600	11.65763300	0.47285800	L
H-H_	0	-5.99872300	13.55137800	-1.16335800	L
H-H_	0	-4.97416900	15.02666300	-1.00768200	L
H-H_	0	-4.71514200	13.13293600	-3.06680800	L
H-H_	0	-3.37689700	14.22450000	-2.48849200	L
H-H_	0	-3.93469300	11.33180600	-1.79168700	L
H-H_	0	-2.34181200	12.17128900	-2.02565000	L
C-C_3	0	-7.55248500	1.04844400	-8.12666800	L
O-O_3	0	-7.61660700	1.86566500	-6.98702200	L
C-C_3	0	-8.24644500	1.11201400	-5.97947100	L
C-C_3	0	-8.98637500	-0.05858500	-6.60427700	L
C-C_3	0	-8.68876800	0.05467900	-8.07219300	L
H-H_	0	-7.62844500	1.67361200	-9.03938800	L
H-H_	0	-6.57780700	0.51870300	-8.14127300	L
H-H_	0	-7.47917400	0.74766000	-5.26185400	L
H-H_	0	-8.95789700	1.76580500	-5.43397500	L
H-H_	0	-8.59762000	-1.01684800	-6.19901000	L
H-H_	0	-10.07963700	0.00856000	-6.41464100	L
H-H_	0	-8.39750400	-0.92256000	-8.51275200	L
H-H_	0	-9.57225900	0.45671400	-8.61418300	L
C-C_3	0	5.70996300	10.07487000	-0.58735900	L
O-O_3	0	4.44188300	9.75031700	-0.07886000	L
C-C_3	0	4.59434600	9.65239200	1.31592600	L
C-C_3	0	6.03906200	9.30631800	1.63153500	L
C-C_3	0	6.75268300	9.45585200	0.31610500	L
H-H_	0	5.81518700	9.68772100	-1.62164600	L

H-H_	0	5.82393300	11.18153100	-0.61076300	L
H-H_	0	4.32174700	10.62387300	1.78108400	L
H-H_	0	3.91395000	8.86944200	1.71212200	L
H-H_	0	6.45235900	10.00630400	2.39052900	L
H-H_	0	6.12199200	8.26362100	2.00499200	L
H-H_	0	7.64651900	10.11049900	0.40455800	L
H-H_	0	7.05327800	8.45784400	-0.07370300	L
C-C_3	0	9.61185000	-8.76703700	3.66878700	L
O-O_3	0	8.82592200	-7.97223700	2.81104100	L
C-C_3	0	7.60660000	-7.71105500	3.46427500	L
C-C_3	0	7.48574000	-8.59845000	4.68239200	L
C-C_3	0	8.91040300	-8.92302700	5.00282600	L
H-H_	0	9.75721800	-9.76361600	3.19880400	L
H-H_	0	10.60736600	-8.29335600	3.80679600	L
H-H_	0	7.58793900	-6.64628400	3.77219300	L
H-H_	0	6.76296100	-7.88729800	2.76719300	L
H-H_	0	6.98147100	-8.07693600	5.52496000	L
H-H_	0	6.92899000	-9.52628300	4.42484400	L
H-H_	0	9.31358100	-8.18684500	5.73342800	L
H-H_	0	9.01612500	-9.95281600	5.40797100	L
C-C_3	0	5.77790800	-2.23996900	-9.00387700	L
O-O_3	0	5.29118400	-0.92331600	-9.09622500	L
C-C_3	0	4.48262300	-0.85095000	-10.24905800	L
C-C_3	0	4.48289200	-2.19339700	-10.95665200	L
C-C_3	0	5.66424000	-2.89181000	-10.36129400	L
H-H_	0	6.83190400	-2.22667500	-8.66078500	L
H-H_	0	5.17119300	-2.80131600	-8.26036800	L
H-H_	0	3.44873100	-0.56461100	-9.96328900	L
H-H_	0	4.89219200	-0.06715300	-10.92227200	L
H-H_	0	3.55114300	-2.75202100	-10.71689600	L
H-H_	0	4.58397300	-2.08260300	-12.05810200	L
H-H_	0	5.50939500	-3.98920500	-10.28636400	L
H-H_	0	6.57430000	-2.67770300	-10.96316800	L
C-C_3	0	-8.89678300	2.67539700	2.94165400	L
O-O_3	0	-8.94745200	1.34340200	2.50455100	L
C-C_3	0	-10.07235600	1.23691500	1.66544600	L
C-C_3	0	-10.47438200	2.61931700	1.18052500	L
C-C_3	0	-9.52049300	3.54563100	1.87780000	L

H-H_	0	-9.46415000	2.76925600	3.89087700	L
H-H_	0	-7.84281500	2.97120800	3.12320200	L
H-H_	0	-9.82311200	0.58528200	0.80315600	L
H-H_	0	-10.90788000	0.76746000	2.22671000	L
H-H_	0	-10.37190500	2.69741300	0.07921100	L
H-H_	0	-11.52460300	2.84403600	1.46652300	L
H-H_	0	-8.74011500	3.89357700	1.16873600	L
H-H_	0	-10.04095000	4.41815400	2.32479300	L
C-C_3	0	-3.97585300	-5.28488500	9.47000800	L
O-O_3	0	-3.78998700	-3.91234500	9.72891500	L
C-C_3	0	-2.76177300	-3.45017600	8.88357100	L
C-C_3	0	-2.08807100	-4.62964000	8.21801100	L
C-C_3	0	-3.13793700	-5.69365700	8.27606600	L
H-H_	0	-3.66810200	-5.86410500	10.36603100	L
H-H_	0	-5.05051100	-5.48722700	9.28034400	L
H-H_	0	-3.20247600	-2.77935000	8.11492100	L
H-H_	0	-2.02309100	-2.87145300	9.47548700	L
H-H_	0	-1.78637900	-4.39498200	7.17568500	L
H-H_	0	-1.19700600	-4.93843400	8.80631800	L
H-H_	0	-3.75826600	-5.66851200	7.35545400	L
H-H_	0	-2.69654600	-6.70566800	8.40193900	L
C-C_3	0	-3.89767300	-5.65001400	-3.43206000	L
O-O_3	0	-4.62768000	-6.74869400	-2.93959300	L
C-C_3	0	-5.94567600	-6.31457800	-2.70318600	L
C-C_3	0	-6.01809300	-4.81056500	-2.85778600	L
C-C_3	0	-4.84339900	-4.50802600	-3.72729200	L
H-H_	0	-3.16341400	-5.33649300	-2.66213600	L
H-H_	0	-3.34627900	-5.94481900	-4.34925200	L
H-H_	0	-6.61915900	-6.79917000	-3.44036000	L
H-H_	0	-6.25946500	-6.61899700	-1.68296600	L
H-H_	0	-6.97237600	-4.48540800	-3.32555600	L
H-H_	0	-5.89287400	-4.31907600	-1.87064800	L
H-H_	0	-5.14880900	-4.53645000	-4.79476300	L
H-H_	0	-4.39125400	-3.52222400	-3.48484300	L
C-C_3	0	9.70808000	4.23835100	2.98818100	L
O-O_3	0	9.60286100	4.04853200	4.38031700	L
C-C_3	0	10.90113200	3.84890000	4.88870000	L
C-C_3	0	11.91849800	4.18082100	3.81925900	L

C-C_3	0	11.14159400	4.03213300	2.55057600	L
H-H_	0	9.04374900	3.52737900	2.45871000	L
H-H_	0	9.37596300	5.26631000	2.74188400	L
H-H_	0	11.05494000	4.49129500	5.78159800	L
H-H_	0	11.00909600	2.78688200	5.19640700	L
H-H_	0	12.26343600	5.23080000	3.93620500	L
H-H_	0	12.79181100	3.49389300	3.85120800	L
H-H_	0	11.44907000	4.78078600	1.79030700	L
H-H_	0	11.26739200	3.00540800	2.14416700	L
C-C_3	0	10.45325200	7.97574700	3.63419200	L
O-O_3	0	11.65577500	7.65872000	2.97044400	L
C-C_3	0	11.47228600	7.94739700	1.60661300	L
C-C_3	0	9.99380600	7.92995000	1.30079700	L
C-C_3	0	9.36638900	8.27459400	2.61590700	L
H-H_	0	10.15396400	7.11566700	4.27081200	L
H-H_	0	10.61657100	8.85490100	4.29404300	L
H-H_	0	11.89592600	8.95214400	1.38741100	L
H-H_	0	12.00466800	7.19456600	0.98712700	L
H-H_	0	9.73250700	8.66989200	0.51573300	L
H-H_	0	9.68271300	6.91280600	0.97988400	L
H-H_	0	9.10327200	9.35486500	2.64096000	L
H-H_	0	8.45685400	7.66415800	2.79899100	L
C-C_3	0	-1.86282900	-5.49663900	-11.49327000	L
O-O_3	0	-1.00433700	-5.74585500	-10.40475300	L
C-C_3	0	-1.80319600	-6.11042100	-9.30224500	L
C-C_3	0	-3.23990400	-6.27339300	-9.75122500	L
C-C_3	0	-3.29489200	-5.46164700	-11.00488900	L
H-H_	0	-1.59291200	-4.53173700	-11.97206800	L
H-H_	0	-1.73826900	-6.30974700	-12.24088700	L
H-H_	0	-1.42538600	-7.05784600	-8.86262700	L
H-H_	0	-1.73610800	-5.31191000	-8.53290600	L
H-H_	0	-3.44873000	-7.34221400	-9.97834800	L
H-H_	0	-3.95846400	-5.90208500	-8.99022900	L
H-H_	0	-3.99850800	-5.89531400	-11.74859700	L
H-H_	0	-3.58873300	-4.41690600	-10.76834800	L
C-C_3	0	-5.14623600	-6.84778800	1.65158300	L
O-O_3	0	-4.28526400	-7.62881900	0.86443300	L
C-C_3	0	-3.27916000	-6.76853900	0.38651800	L

C-C_3	0	-3.71286000	-5.32449600	0.57453800	L
C-C_3	0	-5.09851300	-5.43320500	1.13659300	L
H-H_	0	-6.17991900	-7.24888900	1.59655000	L
H-H_	0	-4.80100900	-6.87750600	2.70766100	L
H-H_	0	-2.34014200	-6.96668500	0.94220000	L
H-H_	0	-3.10081500	-6.97857100	-0.68753200	L
H-H_	0	-3.03716300	-4.81880300	1.29641800	L
H-H_	0	-3.71687800	-4.76721700	-0.38498300	L
H-H_	0	-5.28483700	-4.70072500	1.94684700	L
H-H_	0	-5.85006000	-5.29853900	0.33123200	L
C-C_3	0	2.34746700	6.89280800	-0.44443700	L
O-O_3	0	2.87195300	6.88971600	-1.74690000	L
C-C_3	0	4.22621500	6.52002800	-1.64951100	L
C-C_3	0	4.51125200	5.97054700	-0.26478100	L
C-C_3	0	3.17663300	5.97450400	0.41846300	L
H-H_	0	2.38135700	7.92835200	-0.04770400	L
H-H_	0	1.29400600	6.54814900	-0.46187800	L
H-H_	0	4.44120400	5.74957900	-2.41663000	L
H-H_	0	4.86798500	7.40200200	-1.85431000	L
H-H_	0	4.92493300	4.94362600	-0.32319800	L
H-H_	0	5.22644100	6.62951500	0.27296200	L
H-H_	0	2.73954700	4.95273600	0.41570100	L
H-H_	0	3.24172300	6.34883400	1.46224700	L
C-C_3	0	-13.40587900	-5.29544200	-1.61787300	L
O-O_3	0	-13.27807400	-4.12493300	-2.39182300	L
C-C_3	0	-12.75181100	-4.49194900	-3.64698100	L
C-C_3	0	-12.34782900	-5.94990500	-3.62004500	L
C-C_3	0	-13.15752300	-6.50601700	-2.49256900	L
H-H_	0	-12.66420300	-5.26479900	-0.79147600	L
H-H_	0	-14.42296500	-5.34051200	-1.17347200	L
H-H_	0	-13.52904100	-4.32714700	-4.42418400	L
H-H_	0	-11.87399600	-3.85518700	-3.88594500	L
H-H_	0	-12.57302200	-6.45965900	-4.58225900	L
H-H_	0	-11.26401400	-6.03811100	-3.39271300	L
H-H_	0	-14.12339900	-6.90215000	-2.87759500	L
H-H_	0	-12.61222500	-7.30481000	-1.94644700	L
C-C_3	0	-6.40790400	-2.02440200	0.65424500	L
O-O_3	0	-6.87081400	-2.15483500	-0.66757200	L

C-C_3	0	-7.44403900	-0.92516800	-1.02212300	L
C-C_3	0	-6.78707100	0.16269300	-0.20685000	L
C-C_3	0	-6.31793000	-0.55328400	1.02516000	L
H-H_	0	-7.09986900	-2.55195500	1.34215900	L
H-H_	0	-5.41091100	-2.49960000	0.72702100	L
H-H_	0	-7.28992500	-0.74166400	-2.10640600	L
H-H_	0	-8.53532800	-0.95739500	-0.81053700	L
H-H_	0	-5.91896200	0.58008400	-0.76102600	L
H-H_	0	-7.50520300	0.97363500	0.03229500	L
H-H_	0	-5.27761200	-0.26206300	1.28892200	L
H-H_	0	-6.98562100	-0.33190300	1.88402100	L
C-C_3	0	0.36873800	-9.95792700	-7.64641900	L
O-O_3	0	1.70091300	-10.38938500	-7.81476800	L
C-C_3	0	2.23535000	-10.63667300	-6.53491000	L
C-C_3	0	1.33551800	-10.01266500	-5.49292900	L
C-C_3	0	0.00657100	-9.98506800	-6.17595200	L
H-H_	0	0.27308600	-8.92369900	-8.03825400	L
H-H_	0	-0.31332400	-10.61574600	-8.22615800	L
H-H_	0	2.29527600	-11.73532700	-6.37601100	L
H-H_	0	3.25914900	-10.21257600	-6.46641200	L
H-H_	0	1.30168400	-10.60977300	-4.55778000	L
H-H_	0	1.67546600	-8.97854400	-5.27105000	L
H-H_	0	-0.55968600	-10.91354700	-5.94238100	L
H-H_	0	-0.58792200	-9.09591900	-5.88010500	L
C-C_3	0	-9.34490900	-9.09771200	1.68683500	L
O-O_3	0	-10.32496700	-8.24061500	2.22557400	L
C-C_3	0	-11.45441400	-8.29464400	1.38531400	L
C-C_3	0	-11.28797500	-9.42846500	0.39700600	L
C-C_3	0	-9.80485500	-9.60346300	0.33563600	L
H-H_	0	-8.38198700	-8.55341400	1.59187700	L
H-H_	0	-9.19718900	-9.95312600	2.38084400	L
H-H_	0	-12.36894500	-8.44380100	1.99700800	L
H-H_	0	-11.54765200	-7.32813900	0.84456900	L
H-H_	0	-11.76273000	-10.35259400	0.79521500	L
H-H_	0	-11.71815900	-9.18096600	-0.59667600	L
H-H_	0	-9.52083200	-10.66520900	0.17061300	L
H-H_	0	-9.38288900	-8.96829300	-0.47207900	L
C-C_3	0	2.81119200	-5.76195800	-9.88944600	L

O-O_3	0	3.47965600	-5.06581700	-8.86973100	L
C-C_3	0	3.87055700	-6.03036700	-7.92111600	L
C-C_3	0	3.04664800	-7.29284500	-8.11332600	L
C-C_3	0	2.14447900	-6.96490800	-9.26766900	L
H-H_	0	2.05976800	-5.10275200	-10.37097700	L
H-H_	0	3.54757600	-6.08229800	-10.66005200	L
H-H_	0	4.95268500	-6.25159100	-8.04848000	L
H-H_	0	3.71018300	-5.62677900	-6.90138700	L
H-H_	0	3.70644000	-8.15059400	-8.36987900	L
H-H_	0	2.46010200	-7.53524600	-7.20111300	L
H-H_	0	2.06048000	-7.80844300	-9.98586600	L
H-H_	0	1.13816300	-6.68196100	-8.89185100	L
C-C_3	0	1.12393900	12.76923600	5.21259400	L
O-O_3	0	1.20083000	12.99414400	3.82528900	L
C-C_3	0	2.49591600	12.61868800	3.43262200	L
C-C_3	0	2.98001800	11.52900500	4.36305700	L
C-C_3	0	2.18258900	11.75507000	5.61744500	L
H-H_	0	1.28530600	13.73030100	5.74835400	L
H-H_	0	0.11060900	12.39176100	5.46653000	L
H-H_	0	2.47919000	12.25634600	2.38503900	L
H-H_	0	3.16841100	13.50302900	3.49152300	L
H-H_	0	2.74839100	10.52839300	3.93634700	L
H-H_	0	4.07325800	11.61181500	4.54551800	L
H-H_	0	1.71584500	10.80774100	5.96506800	L
H-H_	0	2.82827200	12.16647700	6.42440600	L
C-C_3	0	-3.57971700	-0.76153700	11.50848200	L
O-O_3	0	-3.33495700	-0.59020100	10.13597400	L
C-C_3	0	-3.83520000	0.67547200	9.77906800	L
C-C_3	0	-4.74527800	1.19586900	10.87843300	L
C-C_3	0	-4.76194900	0.09495500	11.89721000	L
H-H_	0	-3.78968200	-1.83123600	11.72056900	L
H-H_	0	-2.67707800	-0.45852100	12.08221900	L
H-H_	0	-2.98723700	1.37331600	9.61128000	L
H-H_	0	-4.40112600	0.58009100	8.83167500	L
H-H_	0	-4.32140800	2.12251600	11.31801200	L
H-H_	0	-5.76907500	1.40275100	10.49862900	L
H-H_	0	-4.65832300	0.48865400	12.93194500	L
H-H_	0	-5.70190000	-0.49246900	11.80918800	L

C-C_3	0	10.38789800	8.21601100	-2.36041900	L
O-O_3	0	9.13759300	8.83935800	-2.17791800	L
C-C_3	0	8.28524000	8.40650100	-3.21344200	L
C-C_3	0	9.02670100	7.44377200	-4.12110700	L
C-C_3	0	10.46333500	7.65112500	-3.75997900	L
H-H_	0	11.20107000	8.95394900	-2.19466300	L
H-H_	0	10.49910400	7.39881600	-1.61674500	L
H-H_	0	7.39020900	7.91492700	-2.77598300	L
H-H_	0	7.94870600	9.29193800	-3.79448200	L
H-H_	0	8.72352100	6.39947500	-3.89651200	L
H-H_	0	8.84482200	7.66456000	-5.19461300	L
H-H_	0	11.03853400	6.69992100	-3.79049000	L
H-H_	0	10.92454200	8.39640500	-4.44531300	L
C-C_3	0	-9.19606800	5.15358000	-7.05398500	L
O-O_3	0	-10.30798600	5.26099000	-6.20282700	L
C-C_3	0	-11.13765700	4.16129100	-6.49314400	L
C-C_3	0	-10.82114200	3.64835600	-7.88885300	L
C-C_3	0	-9.64440400	4.47282300	-8.32738100	L
H-H_	0	-8.78801800	6.16263400	-7.27300400	L
H-H_	0	-8.40726700	4.55265200	-6.55115100	L
H-H_	0	-10.96351500	3.36061300	-5.74220300	L
H-H_	0	-12.20069500	4.47667500	-6.42874100	L
H-H_	0	-10.55375000	2.57063300	-7.85131300	L
H-H_	0	-11.68374800	3.79289500	-8.57603300	L
H-H_	0	-8.83864100	3.84363500	-8.75791700	L
H-H_	0	-9.96465900	5.23442800	-9.07221200	L
C-C_3	0	8.78488300	-0.89977800	1.01979800	L
O-O_3	0	7.44225600	-0.94047600	1.42547600	L
C-C_3	0	6.80818800	0.16828300	0.83378200	L
C-C_3	0	7.85625700	1.15292100	0.34438800	L
C-C_3	0	9.15661200	0.54244500	0.77256600	L
H-H_	0	9.42564200	-1.33926800	1.81021800	L
H-H_	0	8.90219300	-1.49391600	0.08615900	L
H-H_	0	6.18765800	-0.18338600	-0.01434500	L
H-H_	0	6.14307500	0.65271500	1.57904300	L
H-H_	0	7.81300800	1.23218300	-0.76214100	L
H-H_	0	7.71716500	2.15740400	0.79475500	L
H-H_	0	9.94280800	0.64247900	-0.00444600	L

H-H_	0	9.50589400	1.01280900	1.71563600	L
C-C_3	0	12.60640700	-4.18628700	0.34788300	L
O-O_3	0	12.28003600	-5.53987100	0.56915700	L
C-C_3	0	11.15769700	-5.56018100	1.41480100	L
C-C_3	0	10.40387300	-4.26346000	1.23804800	L
C-C_3	0	11.48339900	-3.29729800	0.85596700	L
H-H_	0	13.55833300	-3.94499700	0.86771500	L
H-H_	0	12.75265300	-4.02507200	-0.74036900	L
H-H_	0	10.51087000	-6.42623600	1.16439900	L
H-H_	0	11.49917000	-5.66703700	2.46739200	L
H-H_	0	9.66899800	-4.35693900	0.41216200	L
H-H_	0	9.87863200	-3.96028200	2.16696100	L
H-H_	0	11.13962400	-2.58940000	0.07055500	L
H-H_	0	11.81646200	-2.72948800	1.75127200	L
C-C_3	0	7.18110000	-11.01239500	1.31191700	L
O-O_3	0	6.49979800	-9.86714200	0.85935100	L
C-C_3	0	7.35356400	-9.18172000	-0.02764500	L
C-C_3	0	8.67795400	-9.91080500	-0.13756400	L
C-C_3	0	8.38057700	-11.26365300	0.42667300	L
H-H_	0	7.51097700	-10.84522000	2.35976300	L
H-H_	0	6.49642000	-11.88667700	1.29241600	L
H-H_	0	6.86673100	-9.12286100	-1.02327900	L
H-H_	0	7.51587800	-8.14999600	0.33945100	L
H-H_	0	9.02626100	-9.97673400	-1.19080800	L
H-H_	0	9.44936500	-9.39925700	0.47931100	L
H-H_	0	8.10952700	-11.96622100	-0.39211000	L
H-H_	0	9.24063400	-11.66897100	1.00284500	L
C-C_3	0	-8.93953400	-5.22498900	1.01717100	L
O-O_3	0	-8.59839400	-6.20923100	0.06622600	L
C-C_3	0	-9.31560900	-5.91097000	-1.10587900	L
C-C_3	0	-9.61160800	-4.43222500	-1.11231500	L
C-C_3	0	-9.74698100	-4.12631800	0.34626100	L
H-H_	0	-9.52464000	-5.68857300	1.83835100	L
H-H_	0	-8.00999200	-4.80402700	1.45207400	L
H-H_	0	-8.72770700	-6.19740100	-2.00101000	L
H-H_	0	-10.26420100	-6.48893600	-1.10677200	L
H-H_	0	-8.75301100	-3.87522900	-1.54488000	L
H-H_	0	-10.53735800	-4.19513000	-1.67486400	L

H-H_	0	-9.36482800	-3.11268600	0.59028900	L
H-H_	0	-10.81235500	-4.20627300	0.64877900	L
C-C_3	0	-0.95923800	-1.77516800	-10.01221900	L
O-O_3	0	-1.10163100	-1.33464200	-8.68670000	L
C-C_3	0	-0.34074300	-2.21113100	-7.88861900	L
C-C_3	0	0.66195700	-2.94890200	-8.76076500	L
C-C_3	0	0.39060500	-2.43784800	-10.14558200	L
H-H_	0	-1.76897200	-2.50058500	-10.24571400	L
H-H_	0	-1.03715400	-0.91226800	-10.70678600	L
H-H_	0	0.18331200	-1.63306400	-7.09931400	L
H-H_	0	-1.02290600	-2.93018800	-7.39376000	L
H-H_	0	1.70759300	-2.72957800	-8.46104300	L
H-H_	0	0.48560300	-4.04429600	-8.70473900	L
H-H_	0	1.15438200	-1.68080100	-10.42986400	L
H-H_	0	0.37404900	-3.25578100	-10.89691900	L
C-C_3	0	12.97784500	-4.63879900	-4.53343400	L
O-O_3	0	12.28892300	-5.72923100	-5.10091800	L
C-C_3	0	12.66390300	-6.88851200	-4.39110900	L
C-C_3	0	13.51229100	-6.49877300	-3.19834500	L
C-C_3	0	14.04090300	-5.15695700	-3.58895300	L
H-H_	0	13.43581000	-4.02379700	-5.33686100	L
H-H_	0	12.25466300	-4.00813800	-3.97500700	L
H-H_	0	11.75786200	-7.43747700	-4.05909600	L
H-H_	0	13.24692500	-7.54916200	-5.06873000	L
H-H_	0	12.87693600	-6.40636100	-2.29011900	L
H-H_	0	14.33083900	-7.22839900	-3.01465800	L
H-H_	0	14.17601100	-4.49487700	-2.70758800	L
H-H_	0	15.00674800	-5.27002600	-4.12944600	L
C-C_3	0	-1.77534000	7.65878100	-6.64956700	L
O-O_3	0	-0.38199100	7.74468200	-6.82729800	L
C-C_3	0	0.11126000	6.43298400	-6.97711900	L
C-C_3	0	-1.03906100	5.44668200	-6.94639700	L
C-C_3	0	-2.14737800	6.23277200	-6.32640600	L
H-H_	0	-2.09037300	8.33110400	-5.82738500	L
H-H_	0	-2.28235600	7.98248100	-7.58455000	L
H-H_	0	0.66323700	6.34699200	-7.93479400	L
H-H_	0	0.81880000	6.22245500	-6.15103900	L
H-H_	0	-1.31218200	5.15521500	-7.98205000	L

H-H_	0	-0.79931000	4.54049700	-6.35098100	L
H-H_	0	-3.13694500	5.96691400	-6.75097400	L
H-H_	0	-2.15324300	6.07637800	-5.22687000	L
C-C_3	0	1.90809100	8.74329900	-9.45357900	L
O-O_3	0	1.04387400	9.69513400	-8.87443000	L
C-C_3	0	-0.15796900	9.66994100	-9.60375500	L
C-C_3	0	-0.27263800	8.33752600	-10.30676000	L
C-C_3	0	1.15707400	7.93093100	-10.49654200	L
H-H_	0	2.77278500	9.26425300	-9.91721100	L
H-H_	0	2.29449200	8.07481200	-8.65459500	L
H-H_	0	-1.01734700	9.82375300	-8.91765800	L
H-H_	0	-0.14755300	10.49593600	-10.34925400	L
H-H_	0	-0.79530300	7.60523800	-9.65358100	L
H-H_	0	-0.81115000	8.42678000	-11.27494100	L
H-H_	0	1.29197600	6.83750300	-10.34533400	L
H-H_	0	1.50027200	8.21014600	-11.51742500	L
C-C_3	0	-0.48116500	12.44379300	-4.82466900	L
O-O_3	0	-1.70655200	13.12258100	-4.67401800	L
C-C_3	0	-2.67858100	12.36355000	-5.34707500	L
C-C_3	0	-1.99623800	11.58858700	-6.44848900	L
C-C_3	0	-0.61191300	11.38432800	-5.91018700	L
H-H_	0	0.30635100	13.17642700	-5.10055400	L
H-H_	0	-0.19968900	11.97832600	-3.85672200	L
H-H_	0	-3.15841700	11.66551500	-4.62649100	L
H-H_	0	-3.45767500	13.03467400	-5.76633000	L
H-H_	0	-2.51280200	10.62731500	-6.65569400	L
H-H_	0	-1.95784900	12.20347000	-7.37538800	L
H-H_	0	-0.51493400	10.36801300	-5.47088600	L
H-H_	0	0.15360000	11.51662200	-6.70562700	L
C-C_3	0	14.69884900	-0.70121600	-4.26067900	L
O-O_3	0	14.46782300	-1.85947000	-3.49368900	L
C-C_3	0	14.08289100	-1.44988000	-2.20088300	L
C-C_3	0	14.18632400	0.05748700	-2.09471500	L
C-C_3	0	14.15652600	0.50155300	-3.52188000	L
H-H_	0	15.79362300	-0.58719000	-4.41718500	L
H-H_	0	14.20867700	-0.80212100	-5.25233600	L
H-H_	0	13.03548600	-1.76977700	-2.01984900	L
H-H_	0	14.73357300	-1.93866800	-1.44472400	L

H-H_	0	13.34727600	0.49165200	-1.50900800	L
H-H_	0	15.15757400	0.33853200	-1.63094700	L
H-H_	0	13.10950200	0.70243300	-3.83647500	L
H-H_	0	14.78267100	1.40402700	-3.68891100	L
C-C_3	0	-12.08200800	-5.62659900	5.84333200	L
O-O_3	0	-12.90670200	-4.49892100	5.65148800	L
C-C_3	0	-13.00247200	-4.26966000	4.26593400	L
C-C_3	0	-12.54360700	-5.50511800	3.52588700	L
C-C_3	0	-11.61281700	-6.15432000	4.50079100	L
H-H_	0	-11.21247500	-5.34998200	6.47732400	L
H-H_	0	-12.66692500	-6.41010800	6.37204500	L
H-H_	0	-14.05054000	-4.02158000	3.99640600	L
H-H_	0	-12.35730400	-3.40589600	4.00016600	L
H-H_	0	-13.41084500	-6.17226100	3.32522300	L
H-H_	0	-12.03408500	-5.25047400	2.57217000	L
H-H_	0	-11.67312200	-7.26301700	4.45188600	L
H-H_	0	-10.56945200	-5.82759200	4.30500800	L
C-C_3	0	-13.66660500	-1.18127700	1.99065500	L
O-O_3	0	-13.56609500	0.10283900	1.43025500	L
C-C_3	0	-13.13060000	-0.07966000	0.10569500	L
C-C_3	0	-12.35448400	-1.38172200	0.01672900	L
C-C_3	0	-12.65588300	-2.08270400	1.31155600	L
H-H_	0	-13.47287200	-1.12965300	3.08282900	L
H-H_	0	-14.69689100	-1.57087200	1.83245000	L
H-H_	0	-14.01354100	-0.10904500	-0.57026400	L
H-H_	0	-12.49006200	0.77508700	-0.19110100	L
H-H_	0	-12.69642100	-1.98504200	-0.85033400	L
H-H_	0	-11.26405500	-1.18668500	-0.07664300	L
H-H_	0	-13.07679900	-3.09732200	1.14016200	L
H-H_	0	-11.73314200	-2.16046400	1.92586100	L
C-C_3	0	5.37123000	-11.89863200	-1.91235900	L
O-O_3	0	4.84995300	-12.45930300	-3.09321200	L
C-C_3	0	3.45382800	-12.48290400	-2.94279900	L
C-C_3	0	3.05487400	-11.34318800	-2.03391400	L
C-C_3	0	4.27867800	-11.12218800	-1.19362400	L
H-H_	0	5.75832500	-12.71206500	-1.26091100	L
H-H_	0	6.21641400	-11.22704600	-2.17165500	L
H-H_	0	2.96753500	-12.37897000	-3.93522000	L

H-H_	0	3.14870200	-13.45542900	-2.49661700	L
H-H_	0	2.83762100	-10.43479000	-2.63606700	L
H-H_	0	2.17220400	-11.60843900	-1.41448300	L
H-H_	0	4.52271200	-10.04025700	-1.13211200	L
H-H_	0	4.12981100	-11.52547300	-0.16835800	L
C-C_3	0	-8.72519100	-1.76752300	9.70307500	L
O-O_3	0	-7.61463600	-0.90787300	9.58216800	L
C-C_3	0	-6.61617200	-1.59596800	8.86706000	L
C-C_3	0	-7.21995000	-2.82145400	8.22371600	L
C-C_3	0	-8.39471700	-3.11813400	9.09813800	L
H-H_	0	-8.99574200	-1.87871400	10.77475200	L
H-H_	0	-9.58816100	-1.31658800	9.16806900	L
H-H_	0	-6.18069000	-0.93079800	8.09348400	L
H-H_	0	-5.81136200	-1.89661900	9.57117000	L
H-H_	0	-7.56254300	-2.58024800	7.19402600	L
H-H_	0	-6.49951300	-3.66472700	8.19783000	L
H-H_	0	-9.24624200	-3.53304800	8.51546800	L
H-H_	0	-8.10022600	-3.83337800	9.89850500	L
C-C_R	0	2.33212600	0.12062500	1.02156600	H
C-C_R	0	2.48084700	1.29734900	2.89284700	H
C-C_3	0	2.99305600	-1.14787600	3.19503800	H
H-H_	0	2.13186500	-1.81651700	3.24668300	H
N-N_R	0	2.28786900	2.19045300	1.97115400	H
N-N_R	0	2.21165200	1.46404400	0.79580600	H
N-N_R	0	2.59104700	0.02776700	2.37161700	H
C-C_3	0	2.45829000	1.53909400	4.37531700	H
H-H_	0	1.55139000	2.09745400	4.61498400	H
H-H_	0	3.32645100	2.13307600	4.69476200	H
C-C_3	0	3.34247000	-0.61207900	4.59297900	H
O-O_3	0	2.38797200	0.31439800	5.07385000	H
C-C_R	0	1.93093200	2.21508400	-0.39983500	H
C-C_R	0	2.99027300	2.54202500	-1.26297500	H
C-C_R	0	0.62390300	2.67738000	-0.61597800	H
C-C_R	0	2.71151200	3.34257000	-2.37188500	H
C-C_R	0	0.40941800	3.51314000	-1.71805400	H
C-C_R	0	1.42986700	3.85215400	-2.60837800	H
H-H_	0	3.51896100	3.57434900	-3.06044100	H
H-H_	0	-0.59645800	3.88542300	-1.89297000	H

H-H_	0	3.36857400	-1.44617300	5.29573400	H
C-C_R	0	2.11946900	-0.99085500	0.13026100	H
O-O_R	0	1.84745900	-2.20684400	0.69014300	H
C-C_R	0	2.25514700	-0.85904900	-1.23171600	H
C-C_R	0	2.03266900	-1.87231700	-2.22178700	H
H-H_	0	2.50548800	0.12949100	-1.58992600	H
H-H_	0	2.20308000	-2.89060000	-1.86773100	H
H-H_	0	0.63248600	-1.95396800	-2.30985700	H
C-C_R	0	2.46672600	-1.63879000	-3.62139600	H
C-C_R	0	2.67829300	-2.73088900	-4.48539800	H
C-C_R	0	2.60333800	-0.34906400	-4.17702200	H
C-C_R	0	2.98567400	-2.54655900	-5.83014800	H
H-H_	0	2.60698900	-3.73877800	-4.08737800	H
C-C_R	0	2.92527400	-0.16670100	-5.51965400	H
H-H_	0	2.45256100	0.53154400	-3.56054500	H
C-C_R	0	3.10943800	-1.26245700	-6.36078000	H
H-H_	0	3.13719400	-3.41001500	-6.46798300	H
H-H_	0	3.04270300	0.84073400	-5.90665800	H
H-H_	0	3.34491700	-1.11724700	-7.40730100	H
C-C_3	0	4.38135000	2.02030700	-1.02314300	H
H-H_	0	4.82740500	2.46429600	-0.12860800	H
H-H_	0	4.37021000	0.93908400	-0.87476400	H
H-H_	0	5.02291900	2.23029700	-1.88055700	H
C-C_3	0	1.15447700	4.72758700	-3.80418700	H
H-H_	0	0.08292400	4.79808400	-4.00508700	H
H-H_	0	1.52661200	5.74646800	-3.65634200	H
H-H_	0	1.64292000	4.33568300	-4.70076200	H
C-C_3	0	-0.52099500	2.24857600	0.25659700	H
H-H_	0	-0.72135600	1.17932000	0.13969900	H
H-H_	0	-0.31764200	2.38141800	1.31855500	H
H-H_	0	-1.42684100	2.79839700	-0.00517200	H
H-H_	0	4.34089000	-0.14522000	4.56935700	H
C-C_3	0	4.20352000	-1.90431100	2.60683000	H
H-H_	0	4.04946000	-2.03217200	1.53561500	H
H-H_	0	5.10747700	-1.30375900	2.75212400	H
C-C_R	0	4.37797500	-3.26619500	3.25045800	H
C-C_R	0	5.43955700	-3.53257100	4.12286800	H
C-C_R	0	3.46417000	-4.29296100	2.96869800	H

C-C_R	0	5.59578700	-4.79618600	4.69532400	H
H-H_	0	6.15871600	-2.75139500	4.34798200	H
C-C_R	0	3.62162400	-5.55461300	3.53661400	H
H-H_	0	2.63509400	-4.09619100	2.29639700	H
C-C_R	0	4.68953200	-5.81291000	4.39803500	H
H-H_	0	6.42980600	-4.98784200	5.36389100	H
H-H_	0	2.90273300	-6.33383700	3.30900000	H
H-H_	0	4.81323800	-6.79762300	4.83689800	H
C-C_3	0	-2.24049400	1.71798400	-4.00508200	H
C-C_3	0	-0.91262200	0.97284200	-3.83365500	H
C-C_3	0	-1.00072500	-0.55082100	-4.04765800	H
C-C_3	0	-3.35513300	1.30002500	-3.03742600	H
C-C_R	0	-1.50090200	-1.29555600	-2.83243000	H
C-C_3	0	-3.70008900	-0.19352700	-3.12641800	H
H-H_	0	-2.59377400	1.55061200	-5.03111100	H
H-H_	0	-0.19000700	1.37067000	-4.55164700	H
H-H_	0	-1.63933800	-0.77029400	-4.91163200	H
H-H_	0	-3.07887600	1.53957500	-2.00349400	H
H-H_	0	-4.68992700	-0.38484000	-2.70572100	H
C-C_3	0	-1.10263900	-2.83776800	-1.04169500	H
H-H_	0	-0.21470000	-3.08840000	-0.45769500	H
C-C_3	0	-3.23668500	-1.57188100	-1.09814000	H
C-C_3	0	-2.07382500	-2.00402000	-0.21690000	H
H-H_	0	-1.55435700	-1.13556400	0.19699200	H
H-H_	0	-2.43411900	-2.57517100	0.63802700	H
H-H_	0	-3.79300300	-0.76186500	-0.61718800	H
N-N_R	0	-2.77092200	-1.07287500	-2.40697000	H
N-N_R	0	-0.67118500	-2.10396400	-2.22905400	H
H-H_	0	-3.75424900	-0.48795100	-4.17731300	H
H-H_	0	-4.25946300	1.87534900	-3.26672600	H
H-H_	0	-2.06429300	2.79616400	-3.90912800	H
H-H_	0	-0.49065400	1.17284100	-2.84356400	H
H-H_	0	-0.01393900	-0.94641400	-4.28502600	H
H-H_	0	-1.57838900	-3.77364600	-1.36234000	H
H-H_	0	-3.93810700	-2.39938900	-1.26839700	H
C-C_R	0	-0.46808700	-1.13407500	4.41695100	H
C-C_R	0	-0.78992200	0.23628900	4.52497000	H
C-C_R	0	-0.95196900	0.82087100	5.79731200	H

C-C_R	0	-0.81115200	0.00467200	6.90383500	H
C-C_R	0	-0.49678600	-1.37587400	6.77298900	H
C-C_R	0	-0.31115400	-1.96637600	5.53712700	H
H-H_	0	-1.17648700	1.87704200	5.88836800	H
H-H_	0	-0.93541200	0.42292100	7.89838900	H
H-H_	0	-0.39523900	-1.97678700	7.66990500	H
H-H_	0	-0.06322800	-3.01508300	5.42601100	H
N-N_R	0	-0.83910400	0.78186300	3.27363400	H
N-N_R	0	-0.56923500	-0.18665600	2.40873600	H
N-N_R	0	-0.33918100	-1.33293100	3.07139200	H
O-O_R	0	0.03139500	-2.44754500	2.45236200	H
H-H_	0	1.06832400	-2.19206000	1.37518600	H

S1-TS2b
-1 1

C	-0.27028300	-0.35587700	-0.64742800
O	-0.79975900	0.53777100	-1.29160800
C	1.17411800	-0.75620900	-0.87899300
C	1.31238400	-1.25379200	-2.32923300
H	1.48908700	-1.53397600	-0.17658500
H	1.77311800	0.14207900	-0.69589300
H	0.56595800	-2.03762100	-2.50145000
H	1.07734800	-0.42456100	-3.00339500
C	2.68267900	-1.80776200	-2.62832100
C	3.62593100	-1.07123500	-3.34852100
C	3.03813100	-3.07793900	-2.16187700
C	4.90504700	-1.57692100	-3.57694700
H	3.35443700	-0.09293600	-3.73869400
C	4.31152900	-3.58900200	-2.39367500
H	2.30190200	-3.65441400	-1.60508200
C	5.25233800	-2.83678800	-3.09641900
H	5.62633500	-0.98849800	-4.13619700
H	4.57360100	-4.57561600	-2.02279700
H	6.24718900	-3.23363700	-3.27285100
C	1.94198000	-0.98737000	2.58438300
C	1.77761300	-1.31417500	4.08871300
C	1.14024100	-0.05125300	4.71836600
C	0.38284600	0.48653900	3.54987700
C	0.02813400	0.35952300	1.35176900

H	1.85089400	-1.88048900	1.96963100
H	1.08710900	-2.15639100	4.18934100
H	0.49368800	-0.27652100	5.56725700
N	-0.52284000	1.38755100	3.34059400
N	-0.71783300	1.30250900	1.97279400
N	0.75246600	-0.12929100	2.38795100
C	-1.43393000	2.38263600	1.37287700
C	-0.93254500	2.95130600	0.20749400
C	-2.51871600	2.93958100	2.04646400
C	-1.55272600	4.09200200	-0.30059500
H	-0.04946100	2.52153600	-0.25220100
C	-3.10145300	4.09800900	1.54627200
H	-2.87548800	2.47453900	2.95852800
C	-2.61988100	4.67778300	0.37248900
H	-1.16696200	4.54462500	-1.20883300
H	-3.94504600	4.54364800	2.06487700
H	-3.08678800	5.57673900	-0.01844700
H	1.89434800	0.67008600	5.04741000
H	2.72283800	-1.57754800	4.56099700
C	3.20497800	-0.18121600	2.24690200
H	3.41433200	0.50486000	3.08186400
H	2.97916600	0.45272200	1.38173000
C	4.47712900	-0.99478000	1.92962100
C	4.31042300	-1.73286700	0.59841200
C	5.63564000	0.00029400	1.79400100
C	4.81660000	-2.00746800	3.02832900
H	3.50165900	-2.47211000	0.63296200
H	4.09283000	-1.03129200	-0.21587700
H	5.22858000	-2.27226400	0.33947400
H	5.81532600	0.52337700	2.74023800
H	6.55777400	-0.52021600	1.51247300
H	5.41369600	0.74935300	1.02762200
H	5.75828400	-2.51380800	2.78979600
H	4.93926000	-1.51612700	4.00054900
H	4.04473200	-2.77902700	3.12662800
C	-2.20748100	-1.70233100	-1.46310600
C	-2.12370900	-2.71052300	-2.42548000
C	-3.09437100	-2.82567400	-3.41628700

C	-4.13709300	-1.90877900	-3.48232600
C	-4.20388200	-0.88417900	-2.54334800
C	-3.26149700	-0.78087000	-1.51743400
H	-1.29697800	-3.41198100	-2.39173400
H	-3.01879900	-3.62513300	-4.14601100
H	-4.89044000	-1.98615900	-4.25889300
H	-5.01450400	-0.16253800	-2.58682000
C	-3.46017400	0.28186500	-0.47405800
O	-3.33412200	0.04879400	0.71671400
C	-3.96999900	1.58435000	-0.97301500
C	-4.84968200	2.26966000	-0.23115500
H	-3.67780100	1.88815500	-1.97418800
H	-5.10328700	1.85252300	0.74301900
C	-5.54003800	3.51359300	-0.59414300
C	-6.54241500	3.99746100	0.25478800
C	-5.22353100	4.24743300	-1.74646400
C	-7.21844100	5.17852700	-0.03683700
H	-6.78803700	3.43916900	1.15436300
C	-5.89703500	5.42635000	-2.03874400
H	-4.43204100	3.90364100	-2.40553600
C	-6.89766800	5.89602800	-1.18599900
H	-7.99158000	5.53910300	0.63396600
H	-5.63836700	5.98688700	-2.93131000
H	-7.41984900	6.81904000	-1.41624400
N	-1.20833500	-1.56532000	-0.44276200
S	-0.74477100	-2.97602500	0.31343400
O	0.10083300	-3.81940000	-0.54112900
O	-0.19959600	-2.62183000	1.62098500
C	-2.29043200	-3.81760700	0.55323600
C	-3.33720600	-3.14856000	1.18602000
C	-2.39120300	-5.14902200	0.17372300
C	-4.51015900	-3.84680100	1.43470600
H	-3.24173600	-2.09325100	1.42997500
C	-3.57610200	-5.83100100	0.43939500
H	-1.55938100	-5.63331300	-0.32633400
C	-4.64510800	-5.19399900	1.07229300
H	-5.34193500	-3.33944500	1.91548100
H	-3.67122300	-6.87208100	0.14540700

C	-5.92161200	-5.93387600	1.37375900
H	-6.79378900	-5.34545100	1.07727300
H	-5.95748000	-6.89293400	0.85367600
H	-6.00846200	-6.12780000	2.44748000
O	2.16620000	2.34199100	0.15497600
C	3.38573500	2.49849100	-0.10802300
C	4.25022300	3.34761700	0.68771300
C	4.03333300	1.82093300	-1.21329400
C	5.58312600	3.49649300	0.40832100
H	3.79819800	3.86744100	1.52708300
C	5.36794900	1.96707100	-1.48440700
H	3.41706000	1.17336800	-1.82890700
C	6.16053900	2.80827900	-0.67805300
H	6.21720200	4.13303700	1.01539600
H	5.83422900	1.43788400	-2.30879300
N	7.53904200	2.95838400	-0.95610700
O	8.03128100	2.34704600	-1.91129200
O	8.22221900	3.69802900	-0.23840800

S2-TS3b
0 1

C	-1.44581800	-0.58456100	-0.67544500
O	-0.72194700	-0.88879600	-1.64014400
C	-1.96167900	0.72100400	-0.47230100
C	-2.27533000	1.51038400	-1.72658200
H	-2.65509500	0.87786300	0.34590200
H	-2.30856700	2.57959900	-1.50313700
H	-1.45868000	1.34644900	-2.43914600
C	-3.60347900	1.11620900	-2.34743900
C	-4.63626200	2.05060600	-2.45844400
C	-3.83225300	-0.18486900	-2.81471600
C	-5.86780100	1.70159900	-3.01263000
H	-4.47217500	3.06547500	-2.10309500
C	-5.06004200	-0.53787700	-3.36589700
H	-3.03104000	-0.91665200	-2.75401600
C	-6.08481300	0.40401600	-3.46590500
H	-6.65601800	2.44459000	-3.08739700
H	-5.21734900	-1.54996100	-3.72659500
H	-7.04170800	0.12690100	-3.89690100

C	-3.43275700	-0.72991300	2.11994800
C	-3.58598700	-1.44827200	3.48971600
C	-3.21145200	-2.93258300	3.26637500
C	-2.32855200	-2.83617600	2.07049100
C	-1.65291200	-1.63195200	0.37104600
H	-2.98909000	0.25767500	2.25681200
H	-2.89142800	-1.00220800	4.20436000
H	-2.70436400	-3.37595600	4.12319300
N	-1.53638200	-3.62666900	1.41391500
N	-1.13774500	-2.87206500	0.34341000
N	-2.43307200	-1.61350500	1.47009800
C	-0.15306800	-3.44130700	-0.53663600
C	1.11517100	-3.68109300	-0.02527500
C	-0.51121300	-3.81028800	-1.82720800
C	2.05532000	-4.30577400	-0.83994400
H	1.35647800	-3.33575500	0.97384600
C	0.43956900	-4.42437100	-2.63372100
H	-1.51236600	-3.60260800	-2.18856800
C	1.71983200	-4.67403200	-2.13974100
H	3.05732800	-4.48135800	-0.46328500
H	0.17966500	-4.71050100	-3.64728000
H	2.46029700	-5.15008600	-2.77477000
H	-4.08407700	-3.54767200	3.02872800
H	-4.59549700	-1.34379500	3.88214600
C	-4.69556400	-0.67520700	1.24891500
H	-5.22216400	-1.63391400	1.35578200
H	-4.38206700	-0.60389900	0.19858700
C	-5.68596200	0.47985400	1.51334800
C	-6.25984200	0.44040000	2.93375600
C	-5.02596800	1.84440400	1.27876400
C	-6.82895700	0.31128700	0.50374400
H	-6.70134400	-0.53693500	3.15948400
H	-5.49747200	0.66083500	3.68842400
H	-7.04640200	1.19485500	3.03662200
H	-4.63049600	1.92284000	0.26074600
H	-5.76540100	2.64088000	1.41245600
H	-4.21315600	2.03924300	1.98701000
H	-7.56513900	1.11306700	0.62151700

H	-6.44820600	0.34589600	-0.52360100
H	-7.34255100	-0.64514800	0.65001300
C	3.63465400	-1.31441500	0.17321100
C	4.67539600	-1.93839900	-0.52001700
C	4.59719100	-2.12992200	-1.89418600
C	3.48651400	-1.66116600	-2.59091300
C	2.46404900	-1.01612000	-1.90467700
C	2.51194000	-0.83903400	-0.51787900
H	5.55330700	-2.26812500	0.03009200
H	5.40603400	-2.63215000	-2.41491800
H	3.41086700	-1.80882100	-3.66349000
H	1.57109300	-0.68057000	-2.42250500
C	1.32571300	-0.20371100	0.16502000
O	0.75054300	-0.81289900	1.09123000
C	0.88762600	1.06090500	-0.31314400
C	-0.23728300	1.61089400	0.31443700
H	1.36868000	1.51189700	-1.17360700
H	-0.42529200	1.18091800	1.29693300
C	-0.61644100	3.04126500	0.20746400
C	-1.52726600	3.57105500	1.12980700
C	-0.12539000	3.88249000	-0.79750200
C	-1.94982500	4.89335000	1.04651100
H	-1.90647500	2.92939100	1.92205500
C	-0.54264500	5.20770700	-0.88222800
H	0.58142100	3.49639600	-1.52540500
C	-1.45968600	5.71770600	0.03523600
H	-2.65846500	5.28139800	1.77161500
H	-0.15207500	5.84487600	-1.66955900
H	-1.78642600	6.75018100	-0.03494200
N	3.77357900	-1.15694600	1.59136100
H	4.45883000	-1.79679200	1.98690700
S	4.10978200	0.36709500	2.22680600
O	2.88133700	1.13892700	2.29264000
O	4.86272100	0.08646900	3.44646900
C	5.17189400	1.14867600	1.04314600
C	6.54413700	0.92221300	1.10512900
C	4.59854600	1.89775700	0.01956900
C	7.35631900	1.47239100	0.12165400

H	6.96306400	0.33418700	1.91558300
C	5.42917500	2.43511100	-0.95756500
H	3.52199300	2.04731100	-0.00249100
C	6.81219500	2.23289200	-0.91990100
H	8.42966200	1.31052400	0.15994400
H	4.99679300	3.02006400	-1.76412500
C	7.70834300	2.84320500	-1.96463000
H	8.12991200	3.78606500	-1.60159800
H	8.54304200	2.18032300	-2.20388800
H	7.15766800	3.05633400	-2.88293700

M03b
0 1

C	1.94537200	1.33682600	0.06736900
O	2.69430900	1.72199700	-1.03437400
C	2.35816400	0.17286200	0.77709400
C	3.50975500	-0.49818500	0.51152200
H	1.65909000	-0.24734200	1.49246300
H	4.24531100	-0.03875700	-0.14626800
H	2.78606500	0.92517800	-1.58162300
C	3.84770300	-1.82082200	1.02853300
C	5.17542000	-2.27669100	0.96699200
C	2.87137900	-2.70717900	1.52179100
C	5.51961200	-3.55305000	1.39729700
H	5.93949200	-1.61450100	0.56791300
C	3.21834400	-3.98060600	1.95968000
H	1.82639000	-2.40479200	1.51847800
C	4.54345600	-4.41275200	1.90009100
H	6.55324200	-3.88046500	1.33763300
H	2.44657500	-4.64777900	2.33171800
H	4.80980900	-5.41106200	2.23150100
C	-0.18973900	1.11083800	2.63580200
C	-1.59853400	1.52230200	3.17263600
C	-2.23185100	2.53027400	2.17837400
C	-1.16535300	2.70418700	1.15423300
C	0.81433300	2.07194000	0.36642900
H	-0.16441200	0.03425000	2.43725700
H	-2.23314900	0.64146900	3.28636600
H	-3.14076100	2.12512000	1.72581300

N	-0.98227800	3.50352700	0.16164200
N	0.27319600	3.14108400	-0.33278500
N	-0.12940200	1.83265900	1.35323400
C	0.89653300	4.05553700	-1.21589400
C	2.22734500	4.44084000	-1.04385000
C	0.11844300	4.63667400	-2.21865600
C	2.78490400	5.37505700	-1.90960600
H	2.81416100	4.00762600	-0.24234500
C	0.68501800	5.57899600	-3.07027700
H	-0.92193400	4.34548200	-2.30671500
C	2.02147300	5.94519400	-2.92694100
H	3.82122000	5.66873500	-1.77697500
H	0.07764400	6.02609600	-3.85080200
H	2.46229300	6.67619400	-3.59654100
H	-2.47972700	3.48488400	2.64579300
H	-1.48978300	1.97956200	4.15662800
C	0.97094300	1.54078300	3.54504400
H	0.73092100	2.53834800	3.93780400
H	1.87472800	1.65562600	2.93349400
C	1.31365200	0.59469900	4.71478100
C	0.09305700	0.29952600	5.59393000
C	1.88467200	-0.72697100	4.18744400
C	2.38455500	1.28731300	5.56616800
H	-0.33775700	1.22065500	6.00139300
H	-0.68919500	-0.23083400	5.04031000
H	0.38635600	-0.33364900	6.43765200
H	2.76789400	-0.55684700	3.56143800
H	2.17991600	-1.37154200	5.02205700
H	1.15022600	-1.28356900	3.59419700
H	2.68846600	0.64541700	6.39946400
H	3.27448100	1.51237200	4.96891300
H	2.00621000	2.22733800	5.98166300
C	-2.42727100	0.43414400	-0.57196900
C	-3.48927200	1.26282900	-0.95132200
C	-3.39569600	2.03532100	-2.09891000
C	-2.23774300	2.01343700	-2.87337200
C	-1.19212400	1.18001700	-2.50889700
C	-1.26643200	0.34657100	-1.38202400

H	-4.37471100	1.31819400	-0.32875600
H	-4.22731400	2.67456000	-2.37657100
H	-2.15043000	2.64053900	-3.75373800
H	-0.29018900	1.19469000	-3.10836700
C	-0.17252700	-0.62045400	-1.06723300
O	-0.17086300	-1.25794800	-0.00167200
C	0.88612600	-0.86492800	-2.06162500
C	1.86342800	-1.74601600	-1.76639400
H	0.86347700	-0.35022500	-3.01322900
H	1.78910500	-2.26224000	-0.81215500
C	3.02241100	-2.10394300	-2.58719500
C	3.87271900	-3.12057900	-2.12822300
C	3.32780500	-1.45999800	-3.79637000
C	4.99884000	-3.48581200	-2.85930900
H	3.64830700	-3.61588900	-1.18636900
C	4.45042000	-1.82733400	-4.52552200
H	2.69018800	-0.66212100	-4.16565100
C	5.28934100	-2.84117500	-4.05897300
H	5.65075600	-4.27008200	-2.48837500
H	4.67701500	-1.32061800	-5.45783800
H	6.16845000	-3.12263500	-4.62956300
N	-2.47520000	-0.28371800	0.63582100
H	-1.65628000	-0.89458500	0.77124500
S	-3.86264500	-0.86861900	1.32986500
O	-3.37821500	-1.77552100	2.36345400
O	-4.72466500	0.24625800	1.70859800
C	-4.68376600	-1.77535200	0.04711900
C	-6.00265900	-1.46962900	-0.26789100
C	-3.98646600	-2.78304200	-0.61743500
C	-6.63767200	-2.20081100	-1.26652000
H	-6.51282200	-0.67071800	0.25893400
C	-4.63746900	-3.49930500	-1.61179000
H	-2.94971300	-2.99466900	-0.36954400
C	-5.96961700	-3.22293800	-1.94619200
H	-7.66699600	-1.97032500	-1.52461900
H	-4.10577800	-4.28389600	-2.14155100
C	-6.67471500	-4.02929800	-3.00366200
H	-7.17018400	-4.89487900	-2.55206200

H	-7.43977500	-3.43628100	-3.50898500
H	-5.97120500	-4.40304700	-3.75043500

TS4b

0 1

C	1.94573900	1.06071600	0.70300000
O	2.88098700	1.72225600	-0.05815200
C	2.07615500	-0.29222800	0.94069400
C	3.06786300	-1.04868500	0.30328700
H	1.29715900	-0.80573800	1.48953500
H	3.96684800	-0.52189900	-0.00999400
H	3.02301600	1.19094600	-0.85580300
C	3.22685500	-2.48931600	0.51985700
C	4.50244600	-3.07151100	0.48888000
C	2.11357300	-3.33041000	0.68823000
C	4.66482700	-4.44316100	0.65897500
H	5.37018400	-2.43563200	0.33310300
C	2.27798500	-4.69914000	0.86455100
H	1.11491100	-2.90079400	0.64791000
C	3.55464900	-5.26215600	0.85395200
H	5.66139300	-4.87327900	0.63708200
H	1.40560200	-5.33217100	0.99514500
H	3.68106000	-6.33187800	0.98580100
C	-0.57367000	0.33982500	2.81234200
C	-1.96681700	0.79992100	3.34580300
C	-2.36381200	2.11825200	2.63625800
C	-1.12983300	2.47144000	1.89010700
C	0.84402000	1.84923500	1.14600100
H	-0.68541200	-0.56313600	2.20544800
H	-2.71557000	0.02766400	3.16800500
H	-3.18593800	1.95425500	1.93388400
N	-0.71423700	3.50039000	1.22459200
N	0.53326100	3.12165200	0.77509500
N	-0.21377000	1.45843800	1.90833900
C	1.31512300	4.12925300	0.12725000
C	2.60823800	4.41270300	0.55925600
C	0.70250600	4.88583500	-0.86675900
C	3.31033700	5.44553800	-0.05189700
H	3.05500600	3.82723500	1.35478200

C	1.41245500	5.92290300	-1.46365600
H	-0.31669100	4.65383900	-1.15354500
C	2.71725200	6.19983900	-1.06324900
H	4.32069900	5.66887900	0.27350100
H	0.94223000	6.51326800	-2.24289500
H	3.26887500	7.00806000	-1.53177200
H	-2.65053900	2.90819900	3.33173000
H	-1.89864000	0.96179500	4.42286600
C	0.48473700	0.17465300	3.91087400
H	0.34675900	0.99006600	4.63286300
H	1.48417500	0.30747400	3.47883700
C	0.46917800	-1.18009700	4.65184800
C	-0.92031700	-1.51979600	5.20351500
C	0.92312900	-2.30593400	3.71619300
C	1.45986700	-1.07707800	5.81728400
H	-1.28616000	-0.73814700	5.87784400
H	-1.65428100	-1.65782500	4.40223600
H	-0.87328200	-2.45505700	5.77065300
H	1.92440600	-2.11247800	3.31490300
H	0.95710500	-3.25800800	4.25607000
H	0.23548300	-2.43998200	2.87322500
H	1.51755100	-2.02867800	6.35532100
H	2.46443200	-0.82939200	5.45799900
H	1.15018800	-0.30283300	6.52709000
C	-2.23033200	0.90485200	-0.72052100
C	-3.23617200	1.86118300	-0.94967200
C	-3.03776500	2.89095400	-1.85594900
C	-1.83445700	3.00363600	-2.55526800
C	-0.83648400	2.06620000	-2.34311200
C	-1.00748900	0.99975700	-1.44824800
H	-4.16700300	1.78767700	-0.39879500
H	-3.82829800	3.61751500	-2.01590800
H	-1.67864600	3.81291500	-3.26047700
H	0.09000300	2.16029300	-2.89816300
C	0.03457600	-0.04423700	-1.30880900
O	-0.22181400	-1.07656600	-0.53500100
C	1.25433700	-0.02895600	-1.98770200
C	2.15304900	-1.09205900	-1.82256300

H	1.50968200	0.81769500	-2.61214800
H	1.72553700	-2.02442200	-1.46734500
C	3.40404100	-1.22519800	-2.58471900
C	3.97495800	-2.49814300	-2.73326800
C	4.06828500	-0.12465600	-3.14932400
C	5.16668500	-2.66704700	-3.43137000
H	3.47274200	-3.35643600	-2.29507000
C	5.25770000	-0.29435700	-3.84608900
H	3.65551200	0.87659800	-3.04878100
C	5.81228300	-1.56687300	-3.98990200
H	5.59112000	-3.66011900	-3.53864400
H	5.75600900	0.56792200	-4.27720100
H	6.74313300	-1.69678400	-4.53211600
N	-2.32421300	-0.13011200	0.20794900
H	-1.18080500	-0.88164400	-0.07255900
S	-3.72179700	-0.76917400	0.68343700
O	-3.36416900	-1.91202000	1.53223400
O	-4.64459300	0.21560600	1.27213700
C	-4.50763000	-1.36955200	-0.80232600
C	-5.86521900	-1.15544100	-1.00216400
C	-3.74097800	-2.06376800	-1.73786100
C	-6.46581600	-1.65591400	-2.15533700
H	-6.43314000	-0.59517000	-0.26725300
C	-4.35467500	-2.55474500	-2.88130300
H	-2.67459900	-2.19956400	-1.57696200
C	-5.72454400	-2.36173900	-3.10437500
H	-7.52598600	-1.48901800	-2.32240700
H	-3.76505400	-3.09133500	-3.61934600
C	-6.37538300	-2.91633100	-4.34427500
H	-6.46198100	-4.00549000	-4.27923900
H	-7.37746100	-2.50614900	-4.48313400
H	-5.78188000	-2.68825700	-5.23347700

S1-TS3c

0 1

C	-0.65535100	-0.58401900	0.06360100
C	-2.02762300	-2.26996800	0.27168500
C	-3.19224400	-0.05130800	0.36334100
H	-3.02695900	0.47806600	1.30101600

N	-0.85600200	-2.81525500	0.14892900
N	-0.01467200	-1.75807300	0.00171400
N	-1.97009600	-0.90241700	0.16119900
C	-3.27589200	-3.03315800	0.58893600
H	-3.02542100	-3.80260700	1.32076900
H	-3.66223500	-3.52824200	-0.31027500
C	-4.42108500	-1.00381800	0.42502800
O	-4.22417800	-2.17867200	1.17283700
C	-4.55729900	0.17066600	-1.59503800
C	-3.60910600	0.82952300	-0.80428800
C	-3.32104900	2.17171200	-1.03848300
C	-3.92564400	2.81276200	-2.11713400
C	-4.82426500	2.13313200	-2.94119900
C	-5.15923200	0.80830000	-2.67460800
C	-4.86558700	-1.19156400	-1.03981800
H	-2.63488500	2.70421100	-0.39550000
H	-5.28805100	2.64960500	-3.77592400
H	-5.89688500	0.28911100	-3.27914700
H	-4.28335300	-1.96533400	-1.55342900
H	-5.92016400	-1.46526800	-1.11566500
C	1.34734900	-2.04890400	-0.36103400
C	1.71048100	-1.86102000	-1.70007300
C	2.22036000	-2.52616100	0.61213900
C	3.04006000	-2.09019700	-2.03503200
C	3.54584700	-2.73928700	0.22414900
C	3.97512100	-2.50558300	-1.08012800
H	3.35917200	-1.93314000	-3.06237500
H	4.25735200	-3.09176100	0.96669900
H	-5.19916700	-0.44706200	0.95205100
H	-3.70326400	3.85715700	-2.31086800
C	-0.02683800	0.82501300	0.01062400
O	-0.62507300	1.60255300	-0.77111500
C	1.51041000	0.86050700	0.01130000
C	2.06708300	2.28423600	-0.07242300
H	1.85563300	0.35313300	-0.89270800
H	1.93877400	0.31927000	0.86199000
H	1.58686100	2.78192400	-0.92072900
H	1.82174600	2.86461900	0.82217400

C	3.56569500	2.23810700	-0.25434900
C	4.11661600	1.82645400	-1.47295200
C	4.43054400	2.54506900	0.79740500
C	5.49490900	1.72565000	-1.63583800
H	3.45451000	1.58312300	-2.30169600
C	5.81208200	2.44752400	0.63938700
H	4.01622800	2.86495900	1.75047100
C	6.34857000	2.03613800	-0.57746500
H	5.90428200	1.40785800	-2.59005400
H	6.46822400	2.69378800	1.46849500
H	7.42392300	1.96016600	-0.70324700
C	5.42279400	-2.67230200	-1.45932500
H	5.94877900	-3.31354800	-0.74930000
H	5.52305300	-3.10244000	-2.45866300
H	5.92335500	-1.69829100	-1.46659400
C	0.70813800	-1.37654100	-2.71626000
H	-0.19176700	-1.99990200	-2.71007800
H	0.39124300	-0.34550700	-2.51409200
H	1.13939900	-1.40609200	-3.71775700
C	1.76472900	-2.82664300	2.01467600
H	1.58389900	-3.90049500	2.12526900
H	2.53095000	-2.54081700	2.73903300
H	0.83404300	-2.31367200	2.26543600
C	-0.46177500	0.16861600	2.87770200
O	-1.45973400	-0.54702500	2.87325000
C	-0.27600200	1.33364900	1.99618700
C	-1.32247900	2.37056300	1.96114600
H	0.69687600	1.79433100	2.15728300
O	-2.49958200	2.19493700	2.25808700
C	-0.86446800	3.72304800	1.46243700
H	-0.00036000	4.08451500	2.02762900
H	-0.55754500	3.60046800	0.41605800
H	-1.68429700	4.43887900	1.53232700
C	0.68307400	-0.12578300	3.83180600
H	1.64398600	-0.12335400	3.30656800
H	0.72872600	0.66777700	4.58514500
H	0.52972200	-1.08605500	4.32571200

S3-TS4c

0 1

C	0.42034800	0.00983600	-0.27427900
C	1.84877200	-0.21111800	-1.89044000
C	2.88623200	-0.05861800	0.35716600
H	2.57297300	0.48974800	1.25114800
N	0.68315800	-0.28613400	-2.46840300
N	-0.20090000	-0.15618800	-1.43823400
N	1.72603200	-0.06250200	-0.53969300
C	3.23321400	-0.12824600	-2.44678900
H	3.20316700	0.15426700	-3.49847800
H	3.75304500	-1.09093900	-2.34502600
C	4.10338800	0.63799900	-0.35162300
O	3.88683700	0.90370600	-1.73191500
C	4.71653500	-1.60624300	0.33496900
C	3.35822200	-1.47380500	0.61197500
C	2.60035500	-2.55226700	1.05959400
C	3.23166400	-3.78348400	1.22069600
C	4.59426700	-3.92347400	0.94444900
C	5.34625700	-2.83701300	0.50323600
C	5.31117100	-0.29892400	-0.11992700
H	1.54599100	-2.42008800	1.28549700
H	5.07087300	-4.88995400	1.07424700
H	6.40467400	-2.94982300	0.28807000
H	5.89965900	-0.38540900	-1.03876700
H	5.97109400	0.11749200	0.64775500
C	-1.60758200	-0.39638900	-1.62995700
C	-2.12689500	-1.60272900	-1.14470900
C	-2.37496500	0.55161900	-2.31135600
C	-3.48563100	-1.83396600	-1.34267800
C	-3.73102100	0.27167800	-2.47068600
C	-4.30116400	-0.90878500	-1.99312900
H	-3.91902300	-2.75480600	-0.96243100
H	-4.35686300	0.99961500	-2.98132600
H	4.26242000	1.62148800	0.08834100
H	2.66209300	-4.64036500	1.56560100
C	-0.19940600	0.19416100	1.09469900
O	0.19993300	-0.57441300	1.96090500
C	-1.03689800	1.35041600	1.27975700

C	-2.07690100	1.15510200	2.38184800
H	-1.47253700	1.69940900	0.34046100
H	-1.56292300	0.82236200	3.28857500
H	-2.55147300	2.11314700	2.60792100
C	-3.15777500	0.15410400	2.03437600
C	-3.01614400	-1.19854300	2.36314000
C	-4.33811500	0.56742600	1.40933000
C	-4.03412500	-2.10938300	2.08951700
H	-2.10233300	-1.52729300	2.85182300
C	-5.36169300	-0.33795400	1.14258300
H	-4.46167400	1.61624100	1.14866200
C	-5.21568100	-1.67959800	1.48846000
H	-3.90980900	-3.15383400	2.36064800
H	-6.27684800	0.00479900	0.66773500
H	-6.01619100	-2.38631600	1.28955100
C	-5.76643200	-1.19046100	-2.19459700
H	-6.35209700	-0.26843300	-2.16371500
H	-5.93925900	-1.66012800	-3.16846700
H	-6.14302300	-1.86730200	-1.42405000
C	-1.76697100	1.81228300	-2.86111500
H	-1.04049800	2.24411500	-2.16854000
H	-1.22939600	1.60175200	-3.79171500
H	-2.54706800	2.54410600	-3.07873000
C	-1.26584800	-2.62153500	-0.44345900
H	-0.31061200	-2.76615600	-0.95921600
H	-1.05105300	-2.31894700	0.58686100
H	-1.78180800	-3.58239600	-0.40546900
C	1.27868300	3.28537200	-0.45973100
O	1.00655400	2.55051800	-1.39468700
C	0.25694900	3.65832800	0.58177400
C	0.31430700	2.62977100	1.77164000
H	-0.73630500	3.73728400	0.13411400
O	1.45759700	2.06435900	1.95001500
C	-0.27137800	3.29810400	3.02265700
H	0.45656200	4.05598400	3.33155700
H	-1.23071800	3.79406400	2.84987800
H	-0.37079500	2.57894500	3.83803800
C	2.66742500	3.82947300	-0.26714300

H	2.65226500	4.92192200	-0.33834300
H	2.97759100	3.56044100	0.74773300
H	3.34110000	3.41220900	-1.01679600
H	0.52441000	4.63438700	1.00512200

S4-TS5c
0 1

C	0.78327400	0.52095300	0.07172600
C	2.43356400	1.58934000	1.05559500
C	3.05862800	-0.67414800	0.30767600
H	2.49965900	-1.60178800	0.21267700
N	1.45222100	2.43595000	1.07574500
N	0.43041100	1.77680100	0.43724000
N	2.08326900	0.41127900	0.45302100
C	3.76930800	1.63764900	1.71923700
H	3.79894900	2.44479700	2.45070700
H	4.57296300	1.78385000	0.98513300
C	4.00795500	-0.72774300	1.55856900
O	3.89869600	0.40729600	2.40916800
C	5.31415800	-0.53848600	-0.48173000
C	3.97999400	-0.42646400	-0.86319700
C	3.61613500	-0.13324300	-2.17317400
C	4.62756300	0.06282200	-3.11072300
C	5.96977200	-0.04224800	-2.73564800
C	6.32327400	-0.34723100	-1.42249900
C	5.43439600	-0.88726100	0.98128500
H	2.56664200	-0.07524500	-2.44950300
H	6.74624000	0.11462300	-3.47759200
H	7.36809300	-0.42891600	-1.13784300
H	6.13178000	-0.24278700	1.52574300
H	5.78106100	-1.91779400	1.10791900
C	-0.77638200	2.48344900	0.12211100
C	-1.07580600	2.73516800	-1.22263200
C	-1.59431000	2.91107000	1.17332200
C	-2.27364200	3.39141600	-1.49913100
C	-2.77948500	3.56483900	0.84001000
C	-3.14284000	3.79905900	-0.48636700
H	-2.52921700	3.59509100	-2.53603000
H	-3.43533300	3.89850400	1.64041900

H	3.71879900	-1.56800600	2.19009000
H	4.37360200	0.29497600	-4.13986900
C	0.00080100	-0.50460400	-0.54760200
O	0.73656400	-1.43054800	-1.22597700
C	-1.36158300	-0.54950500	-0.37487700
C	-2.24475700	-1.54153900	-0.88038100
H	-1.78091300	0.21407200	0.27767100
H	-1.96981600	-1.97142900	-1.84105100
C	-3.68640100	-1.39532100	-0.64868100
C	-4.60810500	-1.88868500	-1.59056600
C	-4.20960500	-0.83344900	0.53058500
C	-5.97801700	-1.82158700	-1.36933400
H	-4.23038300	-2.32521500	-2.51204200
C	-5.58202700	-0.76064500	0.74926600
H	-3.53491000	-0.45136900	1.29367500
C	-6.47778300	-1.25531400	-0.19653200
H	-6.66174500	-2.20917700	-2.11895200
H	-5.95366600	-0.32057100	1.67014800
H	-7.54739000	-1.20179400	-0.02248300
C	-0.15579500	2.31451200	-2.33743800
H	0.89474300	2.46022600	-2.06782600
H	-0.29280900	1.25469400	-2.57794800
H	-0.36413200	2.89672100	-3.23652300
C	-4.45230800	4.46497900	-0.81868000
H	-4.38424200	5.02672100	-1.75290500
H	-5.24129100	3.71573700	-0.93981900
H	-4.76224700	5.14705700	-0.02393600
C	-1.20620100	2.70534300	2.61331400
H	-0.72869600	1.73565900	2.77215800
H	-0.48644100	3.46669400	2.92886200
H	-2.08364900	2.77341200	3.25856900
H	0.34062600	-2.35120800	-1.01692900
C	-0.06778600	-2.25133100	1.84384700
O	1.03017400	-2.66036500	1.51473200
C	-1.34066800	-2.97330200	1.44576700
C	-1.50452800	-3.35061900	-0.04135100
H	-2.23147400	-2.44732600	1.80137100
O	-0.48510500	-3.57096200	-0.76180700

C	-2.70985300	-4.24607300	-0.26325600
H	-2.43394300	-5.25403300	0.06927400
H	-3.58887100	-3.91774000	0.29497500
H	-2.95132400	-4.29068300	-1.32745800
C	-0.21277700	-1.07405300	2.78212400
H	-1.01714500	-0.41046800	2.45438100
H	-0.48600900	-1.44775100	3.77565200
H	0.73405000	-0.53176500	2.85577600
H	-1.30447800	-3.93582900	1.97872900

M05c
0 1

C	0.20314500	-1.47273500	-0.04731500
C	1.82365600	-2.29855100	-1.22431100
C	2.52171000	-0.50381600	0.31990100
H	1.99914900	0.37515500	0.71979400
N	0.76940700	-2.97216500	-1.59366200
N	-0.23593100	-2.44708400	-0.83700100
N	1.51581500	-1.39195000	-0.25310600
C	3.22563600	-2.26835900	-1.73892100
H	3.28626000	-2.76837000	-2.70492600
H	3.90453400	-2.76242700	-1.03095000
C	3.54601800	-0.07167400	-0.78175600
O	3.55464800	-0.90672300	-1.93451600
C	4.71485800	-0.97442400	1.13613300
C	3.36043700	-1.21576600	1.35625300
C	2.91929500	-2.00194400	2.41451900
C	3.87394600	-2.56287100	3.26106500
C	5.23435100	-2.32893100	3.04667200
C	5.66501400	-1.53137400	1.98700100
C	4.91045400	-0.07243000	-0.05877200
H	1.85645800	-2.16482800	2.57283200
H	5.96533100	-2.77406300	3.71409700
H	6.72399500	-1.35445200	1.82506200
H	5.69957200	-0.40364900	-0.73944900
H	5.16213100	0.94359900	0.26855700
C	-1.54700800	-3.04166200	-0.80744600
C	-1.89612500	-3.78381700	0.32524500
C	-2.39107800	-2.86625200	-1.90836400

C	-3.17819700	-4.33232800	0.35307200
C	-3.65823000	-3.43706800	-1.82596700
C	-4.06982400	-4.16436500	-0.70566600
H	-3.48037300	-4.91160100	1.22149800
H	-4.34410800	-3.30906000	-2.65955700
H	3.26823600	0.91070400	-1.16544000
H	3.55974200	-3.18406600	4.09328500
C	-0.59175200	-0.67492000	0.96357000
O	-0.18531800	-0.69912500	2.11411500
C	-1.76381100	0.01226500	0.45650400
C	-2.56306200	0.66943000	1.32228000
H	-1.91774100	-0.00104300	-0.61675800
H	-2.30536500	0.62098300	2.37869300
C	-3.76198300	1.42624400	0.97577000
C	-4.57534300	1.91017400	2.00923100
C	-4.11930300	1.69337800	-0.35530200
C	-5.73198500	2.62739200	1.72440400
H	-4.29163500	1.71909200	3.04078700
C	-5.27404600	2.41060100	-0.63828400
H	-3.48293700	1.35704300	-1.16778600
C	-6.08424100	2.87590200	0.39950300
H	-6.35530400	2.99460800	2.53289200
H	-5.54038500	2.61664200	-1.66964000
H	-6.98433900	3.43811600	0.17262600
C	-0.93206700	-4.00710800	1.46328700
H	0.07776800	-4.21376400	1.09488400
H	-0.87356600	-3.13671800	2.12469200
H	-1.25415200	-4.85913200	2.06355000
C	-5.44250600	-4.78284100	-0.66543400
H	-5.72650600	-5.05138400	0.35382000
H	-6.19224000	-4.09653400	-1.06627600
H	-5.47070500	-5.69231700	-1.27377000
C	-1.94049300	-2.10782100	-3.12585300
H	-1.49107000	-1.14502100	-2.86081300
H	-1.17533100	-2.67424600	-3.66623900
H	-2.78154000	-1.93669800	-3.79930900
C	-1.51519600	3.70466800	0.89019500
O	-1.20415900	3.46103900	-0.30209100

C	-0.85207300	3.19075900	2.03467100
C	0.30457400	2.39553600	1.98999200
H	-1.22852200	3.48191400	3.00821600
O	0.87091200	1.99378800	0.92974600
C	0.97264000	2.00629100	3.29349200
H	0.35368000	2.22218200	4.16597800
H	1.21499100	0.94104800	3.26864900
H	1.91272800	2.56078700	3.38469100
C	-2.68247100	4.64668900	1.11248000
H	-2.39856000	5.64319000	0.75900800
H	-3.53409200	4.31192000	0.51325400
H	-2.97995000	4.71274200	2.16031000
Li	0.30730600	2.46041900	-0.78952200
Cl	0.23197600	0.75326000	-2.33344300
C	1.95293000	3.76854400	-2.84382000
O	1.74370500	3.66389200	-1.43271500
C	3.01835300	3.64979000	-0.77163200
C	4.07529100	3.39345500	-1.86446200
C	3.23689300	2.99112100	-3.08650000
H	1.07701600	3.34204700	-3.33564900
H	2.06450800	4.82528600	-3.12333700
H	3.17633000	4.61174300	-0.27277000
H	2.97044100	2.86807800	-0.00673600
H	4.63171500	4.31056400	-2.07510600
H	4.79898200	2.62760300	-1.57352700
H	3.71728600	3.24559600	-4.03319100
H	3.00828600	1.92037400	-3.08139200

TS6c
0 1

C	0.25104900	-1.39856100	-0.07337300
C	1.87265200	-2.30270100	-1.20201200
C	2.59281100	-0.51892200	0.34086500
H	2.08527600	0.37499500	0.72156000
N	0.80271700	-2.92773600	-1.60587700
N	-0.20300100	-2.35427900	-0.87924900
N	1.57367400	-1.37894000	-0.24603700
C	3.29401600	-2.35176600	-1.65608200
H	3.37040300	-2.86813300	-2.61239600

H	3.91245100	-2.87246100	-0.91235700
C	3.66752900	-0.13869700	-0.73475600
O	3.71191700	-1.01524200	-1.85529700
C	4.74694000	-1.03469100	1.23333900
C	3.38064200	-1.24130200	1.41164100
C	2.88592600	-2.01611300	2.45450900
C	3.80064600	-2.60115100	3.32861900
C	5.17271600	-2.40164200	3.15725600
C	5.65639200	-1.61514000	2.11248800
C	5.00056600	-0.14215700	0.04250800
H	1.81358200	-2.14401100	2.57662100
H	5.87148300	-2.86610500	3.84584100
H	6.72411900	-1.46577900	1.98274000
H	5.81496300	-0.48231000	-0.60256600
H	5.24395300	0.87485200	0.37398100
C	-1.54048800	-2.88517900	-0.91588300
C	-1.97505300	-3.62874400	0.18559500
C	-2.32740300	-2.64477200	-2.04377800
C	-3.27614100	-4.12579200	0.14204800
C	-3.62005100	-3.16628500	-2.03482700
C	-4.10964600	-3.90346300	-0.95544100
H	-3.64426100	-4.70435000	0.98552300
H	-4.26129100	-2.98658600	-2.89407500
H	3.42756000	0.83489300	-1.16564300
H	3.44601100	-3.21589700	4.14949100
C	-0.50004100	-0.57029600	0.93952000
O	-0.04943500	-0.62015400	2.09995300
C	-1.61162100	0.14580300	0.46879500
C	-2.35185000	0.88612800	1.39932900
H	-1.83524600	0.13160300	-0.59129900
H	-2.17755500	0.61635900	2.43945200
C	-3.71909800	1.37585100	1.10909900
C	-4.58523900	1.64863800	2.17452500
C	-4.16603800	1.59405800	-0.19987400
C	-5.86905000	2.13098100	1.94145200
H	-4.24117300	1.48494400	3.19282100
C	-5.44904500	2.07873700	-0.43236600
H	-3.50456900	1.40240700	-1.03930000

C	-6.30311500	2.35017600	0.63518400
H	-6.52957800	2.33695400	2.77753400
H	-5.78211300	2.24840500	-1.45122100
H	-7.30249400	2.73058000	0.45034700
C	-1.07531900	-3.89061600	1.36660300
H	-0.09194100	-4.24548100	1.04088400
H	-0.91742500	-2.98692500	1.96489500
H	-1.51479500	-4.65157400	2.01304300
C	-5.50139100	-4.47899400	-0.98935700
H	-5.92116800	-4.55835700	0.01572500
H	-6.16745600	-3.86332900	-1.59756000
H	-5.48947700	-5.48385300	-1.42392500
C	-1.79653500	-1.86014700	-3.21075200
H	-1.33719200	-0.91992100	-2.88814400
H	-1.01680800	-2.42516900	-3.73151000
H	-2.59714700	-1.64387700	-3.91988800
C	-1.74514400	3.47466000	0.56311300
O	-1.21152800	3.30483200	-0.54273600
C	-1.35234500	2.75853000	1.76244900
C	0.00054600	2.29568800	1.94471500
H	-1.87353000	3.05268600	2.67035100
O	0.77684700	2.08121000	0.99449400
C	0.47609500	2.06460500	3.35890100
H	-0.30356700	1.58800300	3.95780400
H	1.36329600	1.43195500	3.34669500
H	0.71734000	3.02720300	3.82253600
C	-2.87441700	4.46984400	0.68912900
H	-2.46730800	5.40819600	1.08222600
H	-3.31180200	4.65594300	-0.29229600
H	-3.64240300	4.11943800	1.38315200
Li	0.41820100	2.37136000	-0.84309000
Cl	0.66107200	0.78142200	-2.43510900
C	2.06359800	3.95747400	-2.64082800
O	1.80506400	3.74284400	-1.24742600
C	3.05499400	3.60112100	-0.54999300
C	4.14926500	3.49980400	-1.62613400
C	3.35692300	3.20406200	-2.90569400
H	1.20637700	3.56929500	-3.19438200

H	2.17708700	5.03326000	-2.83021100
H	3.19916500	4.46425100	0.10761100
H	2.97951300	2.70497300	0.07453300
H	4.67778400	4.45241700	-1.72067200
H	4.88895900	2.72935600	-1.39362500
H	3.86713100	3.54010500	-3.81039200
H	3.12871700	2.13736900	-2.99473300

3.6 The frequencies of all the transition states

TS1

-159.2793	10.7779	17.3980
20.2839	23.8205	28.6809
33.1665	35.4749	45.9488
54.9910	64.8126	66.6935
73.6359	83.1880	90.0896
104.0405	106.8677	113.8055
118.0626	122.8172	128.1850
135.3798	147.9583	157.9336
170.8339	178.8497	201.6436
205.0826	214.5149	222.1893
231.8805	249.3115	254.3762
261.0261	276.6485	286.5861
297.4513	314.3274	326.1677
334.2048	351.3577	351.6052
376.0837	378.2571	393.8922
414.5868	416.4896	421.9271
430.8655	437.5703	453.9433
486.6324	494.6461	509.5372
510.8379	516.2801	524.2090
526.9026	547.8063	557.0632
559.7642	573.3199	583.2165
589.2282	599.8954	625.5480
630.6730	631.0838	632.5471
634.3473	667.4008	709.6461
711.9686	714.5143	716.5833
722.6965	728.0806	738.3567
760.8822	765.1307	779.9836
781.5441	786.4524	805.5235

806.1489	848.5487	856.1202
862.7426	875.3434	882.2236
882.6505	889.6730	891.8572
894.3242	921.0289	924.0798
945.6765	950.8648	963.4350
979.7153	1000.5878	1002.5526
1004.8009	1006.7508	1008.4041
1011.7614	1014.3879	1016.9740
1018.3761	1022.8720	1023.5337
1027.1264	1028.0227	1035.8391
1042.3472	1050.8034	1054.3157
1058.5173	1063.0214	1066.5448
1069.7330	1070.8622	1071.7385
1073.3420	1086.5610	1104.6510
1120.8995	1123.8869	1130.6885
1132.4373	1153.1891	1176.8706
1182.0448	1186.7524	1192.4105
1200.9283	1205.2568	1205.9838
1212.3479	1221.6984	1238.5973
1239.6265	1248.7790	1264.3486
1279.4645	1285.1536	1300.7770
1305.0620	1309.7394	1312.4113
1330.7589	1339.8170	1346.9758
1347.7022	1356.0613	1356.7652
1359.4390	1361.5804	1362.9374
1377.5285	1377.6974	1383.9965
1389.9349	1403.4451	1413.6322
1421.8396	1429.8113	1431.9433
1463.5996	1466.5946	1467.6049
1469.7846	1476.9564	1484.1094
1484.4086	1490.9127	1494.6645
1496.6839	1499.7453	1501.8542
1505.1011	1506.8676	1507.6280
1508.9934	1515.6959	1525.1116
1547.6982	1554.0654	1557.2159
1565.9074	1654.4677	1673.9039
1678.0459	1678.9268	1687.8545
1691.0549	1692.3229	1699.3331

1700.8471	1701.9568	1718.7183
3039.5549	3059.6553	3063.5782
3065.9132	3067.9389	3076.8044
3077.9328	3105.4617	3112.5127
3128.5777	3133.0023	3135.2525
3136.2374	3137.8924	3163.1096
3167.1554	3168.4472	3171.8781
3176.3790	3177.1280	3185.0454
3185.6957	3186.2628	3188.1929
3198.6446	3201.6900	3202.3480
3211.0241	3213.4409	3222.2260
3223.4469	3233.8854	3241.1911
3253.2202	3259.3075	3300.3135

TS2

-74.1421	14.1998	20.5397
22.5114	30.4599	34.6163
35.9100	48.9610	59.4946
63.3774	66.1737	73.9452
88.1744	90.1053	99.1490
106.5689	111.2904	118.7144
125.3499	132.5864	136.6344
145.8178	161.6184	168.0471
178.0799	203.2316	209.1714
221.6677	224.3346	230.9559
237.0988	253.9437	260.7271
266.3769	275.7093	283.0789
288.9488	312.4226	317.6109
334.5162	349.0124	353.7281
381.2052	390.2217	396.6480
412.4134	416.1850	420.3130
428.6374	433.5282	456.5967
477.0382	499.0468	503.7328
504.4477	510.4026	516.8726
523.8135	528.5505	542.4241
556.2616	560.9932	579.1376
585.4155	586.1499	590.6453
629.7034	632.3142	633.0435

636.2689	646.6285	663.6891
714.1238	718.5624	721.7211
722.8758	732.2259	750.9619
758.2389	772.1088	783.3963
787.9532	788.9096	798.7787
821.9409	830.9879	846.7330
851.3990	868.7063	868.9878
875.6476	876.0794	881.5296
892.1571	893.2050	904.5472
943.6685	951.2471	967.5813
983.8994	988.4257	998.0364
999.2325	1001.0621	1004.7727
1007.0783	1008.1462	1015.7075
1016.5104	1019.5052	1021.5021
1023.3831	1031.0917	1042.8248
1044.7134	1045.7648	1057.6964
1064.6622	1066.6720	1068.6013
1071.5949	1072.8985	1073.4963
1075.9470	1092.6662	1116.5826
1120.9598	1127.3808	1128.0022
1140.9096	1165.7638	1177.0244
1181.6397	1187.3253	1198.3074
1205.2732	1210.2578	1211.8991
1216.7621	1221.8215	1239.1981
1241.1165	1247.3076	1270.3118
1282.8080	1298.8017	1300.0390
1310.7718	1327.3709	1334.4241
1339.9777	1347.8261	1349.6714
1355.0181	1358.0627	1359.6418
1360.1455	1365.6126	1367.1662
1383.6296	1386.6147	1391.7358
1398.6492	1412.7178	1420.1038
1431.6700	1433.9766	1440.4415
1453.3846	1467.7370	1470.0688
1482.4090	1486.8346	1491.3570
1497.1616	1498.5245	1500.1183
1502.3754	1503.8155	1506.9198
1510.3045	1512.9272	1515.8269

1517.0739	1527.7522	1544.9635
1551.7926	1556.5949	1572.1275
1590.2874	1604.5324	1670.1400
1674.8493	1678.5480	1679.6203
1681.1037	1689.9209	1698.8140
1699.7479	1702.3551	1764.4975
3060.6722	3062.6919	3069.2114
3075.8352	3076.5023	3086.0252
3090.4877	3097.1323	3135.7520
3135.8420	3145.9966	3146.6941
3151.5416	3153.8770	3162.3083
3165.5209	3170.4697	3175.1509
3175.4951	3180.6378	3184.2714
3188.2055	3192.7559	3193.7016
3197.6219	3201.7528	3210.3817
3211.8073	3212.8734	3217.3473
3221.3480	3227.8655	3231.8541
3232.5151	3237.4120	3261.5377

TS3

-1075.8375	11.6068	18.2820
21.5236	26.1645	29.2196
36.0052	39.3008	40.9870
45.9482	52.4827	58.4964
68.0611	68.4080	73.3191
78.8225	83.3124	96.2789
104.5004	111.8160	119.3138
128.3680	139.3686	142.4771
152.6231	163.2552	178.3432
185.4309	202.4685	209.4189
220.7516	233.6536	243.1489
247.2684	253.8776	270.7807
283.9311	294.1314	307.6053
327.2410	332.6339	345.8418
357.3323	366.3337	375.6352
407.8812	415.6985	418.4307
423.0040	430.5037	433.4799
466.4448	482.1148	490.3994

499.1718	509.1406	517.2736
521.1999	524.6897	549.7793
556.2843	567.2898	578.4509
579.2021	585.3130	589.1897
614.2307	629.2485	630.1552
631.4278	647.4245	671.6117
708.0038	711.3153	714.1156
717.7856	726.4541	729.3028
743.7163	765.2918	777.2970
782.7805	787.7441	800.3132
819.5033	826.8507	845.1720
848.9931	856.1104	861.8534
871.4895	874.7158	880.3672
882.9332	891.6231	893.7980
913.2802	941.1109	953.3132
963.8757	978.6303	985.3501
987.1865	990.0829	994.1748
1005.5718	1006.0050	1007.5122
1014.5915	1016.5692	1016.7611
1019.9054	1030.1002	1036.4779
1039.8903	1041.4266	1048.9577
1057.6298	1058.6623	1063.2018
1067.2850	1067.3443	1068.1807
1073.4939	1074.6605	1112.5611
1123.3676	1126.1912	1128.8607
1133.3350	1147.0774	1169.0288
1175.1415	1178.2173	1193.9565
1202.4017	1203.8329	1205.8720
1208.6678	1212.1425	1219.9208
1239.4703	1245.3723	1250.7276
1270.1594	1284.0595	1295.0911
1321.1800	1328.9110	1335.4884
1338.8330	1340.0665	1344.7017
1349.4023	1350.6719	1354.6560
1357.8715	1360.3360	1362.9506
1368.4505	1386.2189	1393.4413
1415.0114	1417.8260	1419.1022
1428.0611	1429.3262	1436.6266

1450.9578	1455.0291	1463.2150
1479.6585	1486.1315	1490.1610
1492.3665	1493.5317	1496.3233
1496.7084	1500.3650	1503.1663
1504.8045	1508.0924	1511.3546
1519.2950	1521.5074	1537.1314
1542.4573	1550.1198	1550.5496
1601.1009	1604.8368	1606.3592
1671.0063	1671.8089	1672.1297
1673.9683	1685.8840	1687.0590
1690.4797	1693.7382	1694.2029
1801.4373	3042.7076	3056.1252
3062.5447	3072.5735	3075.4688
3076.0113	3096.4728	3118.3948
3124.6563	3137.7542	3138.4176
3146.3567	3147.3649	3169.5753
3171.0711	3176.7748	3180.3268
3182.6248	3183.4642	3190.8155
3198.8094	3202.9019	3208.2387
3209.8764	3211.2881	3219.3877
3222.9628	3223.3822	3223.6976
3226.4645	3232.8543	3237.0021
3242.9025	3252.7544	3253.9583

TS3E

-1248.4013	17.1685	21.8136
30.2321	33.8438	37.5111
39.6970	49.0329	50.7907
56.1317	60.9493	63.7670
66.9987	76.0422	80.0320
81.8732	93.2416	103.5031
110.4796	111.7572	117.7351
125.1825	133.7285	146.1684
167.1909	175.9139	181.9767
203.9049	209.5338	211.8914
234.0554	237.9292	248.8688
254.7833	264.7077	282.9072
290.8932	297.9426	310.1626

327.1485	332.3540	345.3063
345.5206	357.6230	368.4863
413.6679	415.7605	417.4672
429.7453	436.0840	438.8101
470.0042	483.3384	489.7095
491.7434	504.4328	511.0288
518.0529	523.7557	526.6080
554.3038	556.6391	580.4617
583.4024	591.7859	595.8316
608.0508	624.9080	630.3395
631.5902	645.3053	665.0081
671.2940	693.5545	712.2847
715.5492	719.9793	721.4000
732.3346	748.4271	766.3855
783.8114	788.8471	792.3085
820.3189	829.8838	840.0852
846.6747	855.2186	864.9559
869.7006	878.0234	885.0943
886.6321	893.9697	896.4879
924.8221	940.9221	956.9203
967.4473	980.4523	986.1318
989.1441	995.6629	1002.9447
1003.0733	1008.4961	1009.8651
1013.7454	1017.5548	1018.9382
1026.0811	1034.9931	1037.9419
1039.2604	1046.6260	1047.5939
1063.2026	1064.3190	1067.5960
1068.6557	1069.9578	1072.6146
1074.5746	1082.4924	1124.9612
1126.5485	1127.5494	1132.0394
1141.5232	1145.2040	1175.8909
1179.6071	1194.5835	1203.7041
1208.5879	1210.5474	1213.8494
1218.6301	1227.3724	1232.9277
1245.4120	1248.6043	1266.2680
1270.3052	1284.8629	1295.4098
1320.5425	1322.3039	1329.5067
1335.8035	1339.2140	1341.0453

1349.9424	1354.6881	1356.7186
1359.8616	1362.4726	1364.4962
1370.2108	1384.9276	1387.2239
1404.8684	1415.3579	1418.6106
1422.3111	1426.6808	1431.0505
1437.4329	1445.5849	1468.6322
1475.4885	1483.8923	1487.5118
1488.5757	1490.9848	1491.4586
1493.6979	1501.8455	1503.7878
1505.2081	1508.5362	1514.8377
1517.7251	1527.1999	1536.0437
1545.6758	1550.7279	1556.3283
1598.3747	1603.6397	1610.7797
1675.1853	1676.8353	1679.9805
1680.6514	1688.4925	1693.8895
1698.0323	1698.3501	1700.5246
1797.4242	3028.7647	3054.1254
3063.3324	3068.9198	3069.2210
3071.1699	3075.8585	3088.7589
3113.2715	3134.3940	3135.3316
3140.8679	3143.0968	3145.6807
3164.3555	3168.9212	3170.1580
3171.5341	3182.3239	3183.8980
3189.9743	3192.1072	3197.6830
3197.9936	3207.3231	3208.0329
3215.3669	3217.0424	3218.5781
3226.2710	3227.3289	3236.7120
3240.9416	3244.4094	3252.1114

TS3^{DBU}

-1291.2284	18.6693	21.1295
25.9701	29.7950	39.2200
41.9531	43.4222	51.4906
55.2027	60.9524	67.0394
72.6565	75.4070	81.0410
87.9683	98.4247	100.4544
109.2407	114.8699	120.6961
123.7025	132.0297	133.1664

146.9092	154.6607	175.0236
178.9208	182.9643	198.2548
213.1824	227.6494	233.7364
239.4762	242.8647	257.8601
267.9062	275.4782	280.6402
311.9268	318.6454	325.8126
340.6177	346.6423	362.0141
368.8911	374.8485	389.0679
404.9034	416.0391	420.2309
421.3897	427.2811	434.6608
439.9269	451.6500	485.4948
488.4478	499.7594	507.1272
519.4772	523.4363	525.7697
531.2967	535.7245	555.1487
573.8807	579.1843	582.1886
588.6899	625.8102	630.7716
631.8783	642.6235	649.9136
702.7349	713.4745	721.1218
723.8478	725.0772	731.8048
742.3480	752.5945	768.6060
772.3639	790.3793	793.0987
820.2524	828.7329	842.2319
852.4321	859.6960	862.2896
879.9163	881.8628	887.2675
888.8863	890.2627	898.0254
906.1630	922.1964	925.6376
938.5406	947.5042	959.7751
964.6361	975.1836	979.0744
993.8115	1002.5592	1004.1676
1007.8980	1011.0219	1013.3458
1017.9994	1018.3699	1022.6495
1024.5062	1032.5213	1035.6738
1038.8277	1043.1631	1045.7200
1049.1896	1056.2733	1062.9641
1065.6219	1066.3206	1068.4258
1069.7963	1070.7295	1079.8098
1101.4140	1111.8629	1117.0289
1119.3478	1126.5982	1127.2932

1135.0017	1144.2447	1149.9541
1170.2952	1178.5709	1180.6499
1187.2653	1196.8592	1202.6233
1203.8561	1206.5454	1211.0117
1217.0709	1227.3717	1239.9752
1243.9326	1247.8367	1250.3930
1255.2882	1266.5748	1270.9916
1279.1837	1286.3475	1297.7739
1313.0315	1323.4330	1324.3703
1326.8226	1328.4832	1336.6818
1341.9481	1343.4068	1345.7715
1355.7807	1357.1438	1360.2791
1362.6543	1363.0866	1367.1761
1371.0942	1377.8525	1386.4225
1389.9461	1394.5802	1398.3155
1403.8235	1408.4463	1418.1297
1419.6607	1421.8952	1431.0288
1435.9314	1452.8361	1462.3889
1464.7996	1470.7918	1475.6441
1480.1647	1481.5776	1486.1055
1486.5007	1487.6541	1488.8807
1494.0706	1495.3832	1495.9616
1497.2252	1498.3441	1504.1183
1507.0179	1509.1277	1513.4250
1516.3021	1518.4664	1520.1283
1530.2601	1538.7260	1541.4926
1550.5956	1553.9636	1586.3611
1599.2573	1663.2825	1674.6860
1680.9148	1687.3026	1692.5759
1696.8765	1700.8466	1715.9917
1779.3955	3046.7442	3047.3181
3047.8474	3050.5965	3065.5750
3066.0501	3069.7809	3070.6233
3072.7653	3080.5848	3081.2827
3083.6455	3091.5431	3092.6560
3096.8969	3105.9251	3115.2513
3120.8540	3124.4403	3125.9693
3140.6825	3140.9475	3149.9498

3150.5122	3153.4198	3154.5892
3157.2134	3158.4654	3163.0796
3168.5296	3171.9358	3174.6523
3176.0557	3177.8933	3179.7745
3181.3487	3184.1480	3191.9661
3193.2099	3201.5982	3209.9629
3211.1642	3215.4100	3217.5496
3218.2620	3229.5540	3229.9572

TS3E^{DBU}

-1360.6649	18.9730	24.9106
30.0234	36.4578	39.0444
50.3780	50.9534	58.8691
65.9264	67.4382	69.6865
80.1925	87.4513	89.9470
97.6666	102.1224	104.3339
109.7255	116.2277	120.4970
128.0373	133.6593	135.5294
139.0220	162.4829	182.6839
184.7666	210.2542	212.9397
236.1561	239.7369	244.5879
246.3953	258.0781	264.3159
268.1654	280.2468	288.4316
307.7710	313.6214	328.2101
336.9532	342.1610	348.4828
360.8308	364.8994	373.5860
386.4683	413.7039	416.3018
418.0286	419.2982	446.1542
446.8818	473.9717	483.4002
490.0363	491.9807	502.5518
513.9121	523.5971	524.2193
525.7442	530.5673	556.3482
575.9190	582.4020	589.1897
599.0415	608.9394	625.0583
630.2452	630.9632	654.4148
669.0404	698.5010	705.2222
711.5700	720.3919	721.5470
731.6297	735.5724	753.4986

766.8034	785.5170	792.0799
820.7347	824.1703	843.1000
848.4424	853.3556	859.0600
867.6360	876.9776	877.8930
882.8290	889.1081	892.0913
904.7164	916.8392	923.7553
939.9797	941.5597	952.7955
964.6676	975.9023	982.6368
994.4126	1002.7077	1002.9865
1004.7427	1004.8675	1015.2561
1018.0482	1018.7940	1022.5099
1026.7860	1029.8539	1038.3122
1038.5826	1039.7744	1044.0819
1045.4004	1062.1592	1063.5532
1067.3031	1068.6518	1069.8867
1072.9036	1073.3170	1082.6578
1103.8810	1120.1845	1125.2281
1125.9414	1128.8250	1130.4098
1132.3178	1141.5995	1145.4426
1178.4149	1180.4419	1185.9951
1194.6123	1205.0096	1206.9184
1207.9850	1218.0191	1223.5592
1227.9377	1233.2170	1243.3659
1246.0309	1250.2628	1252.2497
1268.8842	1271.5302	1272.5203
1279.7279	1281.2311	1296.5985
1317.9203	1324.8142	1327.4461
1330.3209	1332.4944	1341.1447
1343.3211	1344.9811	1347.0549
1360.0744	1360.9493	1362.1728
1362.5825	1362.7711	1365.9997
1371.2989	1374.0471	1383.3117
1390.1665	1393.2347	1396.9764
1407.4857	1411.1692	1412.8236
1417.0056	1420.0605	1427.1728
1429.6656	1430.8732	1446.5351
1466.9258	1472.2852	1477.8875
1481.9327	1485.0402	1488.3517

1489.0205	1490.3207	1490.6280
1491.6471	1492.2367	1492.8604
1494.9283	1500.1210	1504.2927
1505.8019	1508.0967	1509.2737
1509.4970	1518.2862	1518.9756
1525.1713	1543.0187	1547.9514
1553.6280	1554.5964	1598.9394
1606.1954	1674.5111	1675.9559
1681.1838	1688.5788	1693.3317
1699.8363	1701.5426	1710.0585
1786.1941	3027.3547	3050.0068
3052.2266	3062.2748	3069.6696
3070.1164	3070.5593	3071.6588
3071.8427	3073.3843	3075.3815
3079.3779	3079.4202	3084.9632
3086.3031	3094.8967	3110.6847
3113.8998	3117.6007	3118.3594
3122.8124	3137.9167	3138.5369
3142.4507	3143.2182	3143.2519
3143.6093	3149.6031	3155.1775
3164.5187	3171.9517	3174.3720
3176.8966	3177.6640	3179.9834
3193.4779	3194.6989	3195.2192
3197.4139	3205.7122	3206.0170
3209.6942	3210.6539	3217.3800
3219.4366	3228.1156	3229.7298

TS4

-1308.1782	15.2046	22.4715
25.2644	30.5213	36.1078
38.4901	40.8116	47.2471
53.1887	55.9439	58.9910
68.1881	71.2422	73.0838
77.1673	80.2923	85.5840
91.1056	95.9428	108.3228
114.7844	120.0830	124.0268
126.9845	130.3315	134.5316
150.6028	153.0063	158.0992

163.8257	180.3861	199.4915
206.9331	211.9044	215.3626
218.7292	227.7416	242.1268
248.6167	252.7702	255.7959
261.4474	272.8396	281.2661
284.0217	284.3576	319.4128
324.5664	334.5050	344.5325
360.2292	364.7890	370.1563
388.4960	390.9449	399.8232
414.1079	419.4422	419.6302
426.6869	430.0709	434.6991
442.1591	449.4859	457.4640
475.8743	496.5955	498.7098
507.7063	521.8399	526.7735
526.9893	527.7745	533.1239
537.1672	548.4343	561.8719
568.6561	581.7610	582.7537
588.8118	591.2966	594.8023
600.0908	616.4378	629.1203
633.1473	634.6708	647.8165
653.1794	684.2037	692.0332
717.6405	720.5026	721.4219
729.9783	736.3922	745.4796
758.1155	769.8644	771.0560
777.7088	779.2058	783.5426
793.3435	812.5681	819.5863
836.8901	845.9181	855.2253
856.8443	870.2828	871.3342
872.3092	881.7087	883.7732
887.1628	895.2500	904.4422
905.3842	914.2399	920.1977
923.1292	932.6073	937.5020
938.5005	952.6376	965.4821
974.4661	980.7897	990.6629
996.2376	1004.1135	1008.5057
1009.3496	1010.1435	1010.7670
1013.2521	1017.3519	1019.0793
1021.4445	1024.5883	1028.3421

1036.1547	1037.1628	1038.9288
1043.4371	1044.6131	1048.2269
1060.1675	1067.4309	1068.1599
1072.1820	1075.0534	1077.7846
1093.0689	1101.3555	1116.6338
1119.1073	1120.1072	1122.6297
1124.4628	1127.5340	1135.1152
1138.9314	1144.8391	1155.6186
1174.0879	1180.5434	1182.1203
1186.8120	1187.6972	1200.2530
1201.5144	1209.1920	1211.7770
1217.4912	1224.2576	1229.4680
1230.6155	1242.8227	1243.7070
1245.8275	1247.0808	1250.0056
1266.6369	1267.3216	1275.0601
1278.5897	1295.6708	1304.0817
1306.5422	1316.3830	1317.2023
1324.2070	1326.9250	1333.7440
1335.5583	1338.4015	1340.4903
1346.1749	1347.9176	1352.8455
1359.0677	1360.0629	1363.1794
1364.2473	1372.4136	1374.9723
1383.0372	1385.1779	1393.0426
1399.3289	1402.4371	1407.1507
1409.1413	1413.5824	1415.5426
1420.4150	1428.7483	1432.8397
1436.5757	1441.3399	1456.6372
1462.8515	1467.3955	1475.5281
1477.0803	1478.8505	1481.3153
1481.8224	1490.6315	1491.2243
1491.5122	1493.3885	1494.1516
1497.7855	1498.5810	1500.3733
1502.4930	1505.6171	1507.9418
1510.2633	1513.2976	1514.6921
1518.6634	1519.4426	1529.2837
1546.5098	1550.3892	1553.2685
1561.7728	1573.3766	1583.7191
1593.4265	1662.7367	1669.6356

1677.6320	1678.5834	1681.1669
1689.0187	1695.1428	1699.5668
1704.2247	1706.8289	1720.6481
2238.8255	3032.6667	3051.4666
3053.2450	3059.1108	3060.7833
3061.8616	3062.4035	3063.9991
3065.1507	3069.6977	3076.8224
3087.2832	3091.5781	3103.8769
3114.3843	3114.9253	3117.1148
3124.7098	3129.6967	3130.3480
3134.2523	3135.4211	3141.8510
3146.5617	3158.3212	3160.3014
3161.4723	3162.5346	3166.6118
3166.9347	3167.3473	3171.5039
3175.2790	3186.8050	3190.3868
3191.1281	3192.7864	3196.4433
3197.8178	3205.4601	3206.6313
3207.8632	3214.4958	3216.8790
3217.4875	3225.7453	3225.8282
3230.4269	3236.5066	3246.5973

TS4Z

-1315.7515	9.2801	16.9462
22.4779	29.3184	31.9924
36.9245	38.9572	42.5380
48.2063	49.9457	58.3028
62.3712	65.5534	66.7009
73.7059	76.8793	81.9034
83.6571	94.0828	100.7971
104.8212	111.0700	115.3518
120.1928	120.8914	130.4455
159.5764	164.4298	172.7904
174.3318	192.4361	193.3291
207.1860	211.8494	223.6668
233.6493	239.1069	245.6987
247.0651	248.2679	253.6514
257.7764	265.3319	272.4216
280.3973	286.8060	311.3808

317.9169	328.1115	343.6269
347.2479	354.9545	368.7815
384.0471	392.6937	399.3778
401.1918	415.4782	424.2412
429.0580	429.3643	433.5216
445.1254	450.0241	452.5749
486.0980	493.7851	498.7350
505.4179	518.4419	521.2204
525.1239	528.6036	529.3900
530.4419	545.0779	563.6175
569.8252	582.4611	586.4916
590.5067	596.8813	599.5012
613.6638	615.3945	628.7405
631.5949	637.0756	646.8216
653.8078	681.9276	702.0481
706.9351	721.9458	727.2266
727.3207	739.6525	749.1753
756.1056	764.3956	765.2815
778.7471	785.8389	788.1887
799.9825	807.9311	811.7206
817.0802	830.4626	850.5658
855.1139	862.5841	871.3817
885.4078	886.2885	888.1067
892.5820	898.6768	900.4313
912.0884	918.5982	919.4213
933.7638	934.0114	947.9071
964.8928	965.4480	968.7817
975.0429	979.5310	990.2312
998.1641	1004.1172	1005.2890
1005.8657	1013.2560	1014.0988
1015.1281	1018.3068	1020.3705
1020.8096	1022.7347	1025.7961
1036.1640	1038.6987	1041.9248
1045.8534	1052.9514	1057.2788
1060.7694	1065.5954	1069.0442
1071.4633	1072.0672	1078.8004
1099.4990	1101.5022	1114.3587
1119.2444	1123.2018	1123.9074

1130.2766	1132.3186	1137.4945
1144.9872	1152.6480	1160.6997
1173.0634	1174.3490	1177.3578
1185.4942	1192.2388	1194.4747
1202.5743	1207.6958	1210.7901
1217.0456	1223.7172	1225.5876
1237.8069	1238.8843	1241.0544
1246.4983	1250.8925	1253.0140
1263.3290	1270.6499	1274.6675
1285.5891	1294.9864	1302.0806
1306.7993	1315.5059	1319.0766
1320.8395	1323.3804	1326.6388
1336.5937	1339.7995	1342.9847
1345.3690	1347.9621	1354.0695
1359.5049	1361.0028	1362.4835
1365.3580	1372.3426	1373.6765
1381.4965	1389.2373	1392.4159
1398.0147	1402.6963	1404.9423
1412.1044	1414.5647	1416.7684
1420.2905	1429.5194	1431.1692
1435.4359	1440.6879	1454.8047
1468.3813	1472.4884	1475.8575
1476.9394	1480.0955	1483.3105
1483.6298	1485.4682	1488.3804
1491.0338	1492.8907	1493.0649
1494.8545	1495.7098	1495.8180
1497.3998	1500.2463	1501.8075
1504.1700	1506.7598	1510.1135
1513.4478	1517.0529	1525.5530
1530.4990	1546.5268	1553.5405
1554.9958	1560.5493	1563.0653
1598.4970	1654.9547	1664.9014
1666.2175	1672.3376	1687.9516
1695.9286	1699.1768	1699.7573
1705.2126	1709.2916	1719.4787
2374.2898	3034.6954	3051.2941
3051.6666	3062.1009	3063.8350
3065.3692	3067.2746	3070.8830

3071.4379	3073.1202	3077.2112
3078.3582	3087.5467	3090.2870
3107.5489	3111.3889	3111.5782
3116.9347	3124.0495	3132.0403
3139.6490	3142.5931	3143.7909
3145.1846	3147.1205	3148.3430
3150.1408	3155.5141	3166.2849
3171.1627	3173.3686	3178.4113
3183.2568	3185.7151	3191.7760
3197.8619	3198.5478	3202.8426
3203.7339	3204.6354	3209.1261
3214.2063	3215.7387	3215.9359
3221.9256	3228.0639	3229.4665
3232.9275	3235.6137	3238.8193

TS4^{HOBt}

-1230.7089	16.6624	21.8748
23.9582	26.4992	33.3899
40.2060	43.4181	50.5091
59.3374	64.5526	69.5681
75.8427	80.9523	87.8669
96.4280	102.6046	107.4888
129.4471	137.4266	148.7134
160.6725	163.7892	179.2142
193.9597	198.1524	208.1018
230.5860	235.5365	239.4354
246.9148	251.5271	260.8144
262.7312	282.0352	284.2153
289.0306	312.4183	317.7911
336.2194	353.7498	377.9261
384.3142	389.6925	401.3340
417.8023	419.7810	436.8733
438.0172	446.9167	462.6478
489.2709	504.4832	514.7139
521.0026	522.9945	524.6746
532.0913	544.9724	555.0413
564.4344	580.6619	584.1010
584.1868	590.5019	598.5679

605.4254	618.9379	630.2395
632.5528	633.6167	652.0286
680.1161	695.4576	715.9706
720.5382	731.6182	747.5735
757.2463	770.9671	774.7003
777.6827	781.4276	787.1584
793.3245	813.1985	836.7108
855.4797	860.3364	873.5572
876.9076	881.4692	892.1070
895.8095	901.6923	922.0693
935.4867	947.1182	952.7321
961.0071	966.7707	978.7897
979.5392	988.7589	1002.1924
1003.0143	1007.7089	1012.4011
1012.6284	1013.7611	1018.5327
1027.3076	1028.2212	1029.2125
1035.7604	1045.9292	1048.3166
1060.1603	1062.8940	1068.3549
1069.7697	1070.4380	1072.2842
1074.8376	1099.0443	1117.9664
1121.0267	1127.9341	1139.7261
1156.4969	1175.9900	1179.0421
1184.8338	1189.3440	1200.5075
1204.7516	1208.2867	1210.8793
1214.6511	1219.4497	1223.1672
1243.8047	1245.0328	1249.7736
1258.9197	1271.2206	1285.7432
1288.5964	1318.1535	1319.9930
1330.4913	1330.8295	1338.8547
1343.7627	1346.1886	1347.4933
1352.2458	1357.3404	1363.4113
1364.5488	1365.2473	1384.3770
1388.3776	1397.5425	1414.9312
1416.7658	1418.8773	1428.3334
1433.7345	1439.1005	1458.2903
1467.6362	1479.3192	1485.3231
1489.9750	1491.3939	1492.4044
1494.8456	1496.9395	1499.2464

1505.2206	1508.7028	1509.6012
1511.0029	1516.2968	1523.4067
1548.8785	1551.2056	1556.5607
1561.5072	1597.9718	1613.3449
1665.9570	1672.6796	1672.7599
1681.6887	1685.9577	1689.0546
1694.8547	1701.4784	1702.5161
1710.0610	3047.9915	3064.3293
3065.6604	3067.6850	3068.6772
3095.0260	3121.1546	3134.9100
3137.0013	3142.0414	3158.2223
3161.8469	3169.6499	3170.0040
3172.1319	3172.2561	3173.1032
3178.7014	3187.9799	3188.7653
3190.3384	3196.1280	3201.0214
3206.5440	3207.7064	3209.0404
3209.2042	3214.8110	3221.7609
3223.5117	3230.7169	3232.0544
3237.6889	3249.0816	3779.3053

TS4Z^{HOBt}

-1240.5311	17.6368	23.6454
30.3127	33.4640	37.8621
38.5791	40.7777	42.4644
47.5120	53.8761	59.5324
62.8518	78.5775	88.2927
100.7236	103.9023	110.5446
112.1662	117.7350	138.9589
154.5337	157.0913	169.1536
183.7192	191.0103	196.0726
209.2312	227.5779	235.6147
238.9949	247.9625	250.9947
265.5600	277.3202	280.4256
291.4483	318.6157	333.4905
343.4876	358.2083	372.5943
383.6313	389.0114	401.7053
420.2044	432.6265	438.6670
440.3789	451.4550	481.7922

490.3452	500.5956	510.9765
513.7201	522.2997	525.6456
527.9482	544.6379	562.0975
572.8919	579.7743	584.7472
587.7578	589.8505	599.9086
608.4647	618.7675	630.4962
633.1205	639.1032	654.2932
682.0275	699.5027	719.5132
734.2019	740.2993	754.6634
756.2706	769.0299	772.9044
780.2705	784.0856	784.8463
789.6735	811.8451	825.9453
854.5468	861.3953	874.0163
876.4114	880.3727	884.8942
888.1362	896.3354	921.9998
935.5134	950.2050	951.8027
956.8308	962.5344	975.5727
978.3863	1001.6460	1006.1673
1007.1684	1008.1883	1008.9337
1010.7511	1011.2674	1017.1532
1023.6427	1027.0330	1028.6608
1036.5589	1040.2976	1050.5218
1060.7233	1063.8227	1064.4988
1066.2845	1067.4239	1070.4084
1074.5748	1090.7447	1111.9197
1118.7747	1122.6636	1138.5080
1141.1369	1155.7430	1172.4773
1175.6315	1180.2330	1189.0347
1195.1644	1200.0047	1201.6043
1205.7933	1217.2338	1223.6907
1233.2156	1241.1260	1242.4016
1250.3516	1267.8257	1281.3743
1315.0742	1319.9415	1322.7441
1328.9638	1331.0499	1332.7679
1334.0837	1339.8511	1348.6506
1350.0864	1354.2846	1355.0613
1359.0683	1373.0808	1384.2125
1393.4861	1411.1720	1416.9380

1420.1565	1426.1478	1429.6573
1430.9600	1439.0670	1457.8287
1464.2067	1477.0031	1479.7559
1488.9255	1489.8425	1490.9014
1493.1469	1494.3567	1498.5220
1498.7753	1503.2047	1508.6747
1511.8286	1514.3251	1521.4827
1538.5386	1549.4074	1549.9973
1558.7994	1601.1936	1631.6495
1651.2775	1669.4735	1672.2966
1674.5719	1680.4597	1683.0084
1691.0486	1692.4006	1697.2197
1704.9269	3043.1881	3060.3868
3067.0973	3072.2167	3078.7763
3105.6285	3115.7630	3136.0441
3138.8996	3149.1721	3156.4253
3170.0247	3172.1037	3173.6613
3179.4918	3179.6010	3180.9566
3186.7561	3200.9726	3202.2066
3203.7511	3206.4194	3208.1659
3210.1344	3211.0268	3217.0087
3217.0979	3219.9236	3225.0562
3227.4893	3235.1231	3237.1759
3237.9916	3253.5046	3759.0111

TS4$^{DBU\text{-}DH+}$

-1413.5829	4.7613	15.5383
16.4832	21.0885	21.4385
22.3855	29.0926	30.8322
32.2727	38.8605	43.8839
47.4759	54.5721	57.4028
58.0314	63.5815	65.8045
71.8810	77.7910	80.9881
84.8266	91.0799	94.1468
95.9492	100.4102	106.5674
111.8506	122.0739	128.9236
138.7354	146.8418	161.7737
168.9575	176.5641	195.7195

197.5383	205.6248	217.0569
225.3036	236.0248	239.9195
243.3584	248.1075	249.0424
256.7417	263.4023	263.9196
278.0120	282.7135	309.5509
311.5470	314.4257	337.2654
348.9389	357.2144	363.1609
374.7833	384.0079	384.8016
394.1752	401.7383	410.3100
414.3439	422.8315	423.2001
424.5493	428.4505	437.6975
441.6383	451.0938	466.1840
486.5415	489.0900	493.3735
506.9423	515.9559	519.0928
521.7735	525.7073	528.2627
532.4878	534.2695	551.9726
556.8957	567.4996	580.7392
586.7964	589.8559	597.1297
628.1620	630.3948	631.2866
649.3098	654.5142	656.8732
695.0783	713.2779	719.6130
721.0040	724.1804	728.6115
732.1629	737.5737	743.5430
759.0243	779.1407	781.1496
793.0512	817.0607	818.3547
835.7698	845.7203	852.4778
853.8330	855.8468	860.9195
867.7499	877.8488	880.4649
885.7705	888.5826	891.6960
896.5219	898.8351	901.8526
905.7108	918.7441	921.1544
927.1014	929.2692	934.7016
937.7666	948.1370	949.6446
964.0184	979.8906	989.3090
990.8835	999.7913	999.8276
1004.7472	1005.7240	1006.3590
1010.2355	1014.2316	1016.8519
1017.0760	1021.3024	1023.5740

1028.7212	1030.5917	1036.6908
1037.6866	1038.3180	1041.6122
1049.3224	1062.4810	1063.8752
1066.5375	1067.0625	1068.9653
1072.8664	1073.5701	1081.1696
1100.8325	1101.6574	1105.3618
1112.4199	1117.8775	1120.0276
1121.2682	1123.8213	1125.5360
1128.5753	1131.1743	1136.0811
1147.3127	1147.9349	1171.9647
1176.7393	1182.3410	1187.4805
1188.7630	1199.2205	1202.4264
1204.5933	1206.8471	1217.3647
1218.6931	1221.5215	1229.3035
1234.1778	1239.2140	1240.3829
1242.6754	1244.0771	1246.3705
1248.0657	1265.2724	1268.0287
1270.9074	1275.3512	1278.1290
1281.7487	1296.2481	1296.6045
1300.9357	1316.4233	1320.6529
1323.2697	1326.1690	1326.7367
1327.9670	1330.8479	1332.2350
1341.1229	1344.7821	1351.2055
1353.3408	1354.3347	1357.0504
1358.8874	1360.6280	1363.3747
1364.8245	1375.4933	1376.6839
1377.3724	1384.1226	1384.1823
1384.9980	1390.3267	1393.7746
1394.5835	1405.7874	1407.8713
1409.2608	1409.8745	1415.8299
1416.1571	1416.8929	1424.1954
1425.6610	1428.1846	1433.9136
1454.6966	1461.9912	1473.5896
1476.1540	1480.1266	1480.2511
1482.1066	1487.4023	1487.5912
1488.9754	1489.5402	1490.9466
1493.6626	1494.5207	1494.9598
1495.8216	1496.0556	1498.7435

1499.8959	1500.2983	1504.2583
1504.9215	1505.6586	1508.7253
1511.8768	1512.8149	1514.6066
1518.3168	1518.9972	1520.7515
1528.5331	1544.4063	1547.7759
1548.6618	1550.7264	1568.4353
1591.1600	1618.2865	1658.2140
1666.0560	1672.5299	1679.0472
1687.1524	1691.7541	1692.4173
1692.9359	1707.5356	1718.3958
2411.3709	3032.6413	3038.4844
3050.1616	3052.3225	3056.8722
3057.2833	3058.8246	3058.9908
3064.2897	3066.6649	3066.7476
3068.0714	3069.5762	3073.3667
3076.4145	3078.0246	3086.3454
3088.3742	3089.1744	3096.0876
3097.0011	3097.8229	3109.3968
3109.5005	3113.6427	3116.2763
3116.9451	3118.6139	3119.9806
3122.8291	3123.3535	3125.2763
3132.3138	3137.1800	3142.1480
3145.7318	3146.6253	3151.5873
3152.2904	3154.0304	3156.8243
3165.2180	3170.0843	3170.8018
3171.3425	3173.5834	3175.0738
3176.5398	3183.4551	3185.6095
3188.6647	3200.3652	3205.4664
3207.2802	3210.3787	3212.6856
3218.2722	3220.9416	3229.5755
3236.7356	3241.0740	3243.2547

TS4Z$^{DBU-DH+}$

-1361.7026	14.3797	17.0744
22.1967	22.8242	27.8355
29.3700	31.1212	32.5999
35.0782	40.5388	46.8696
52.3763	58.0104	63.0889

64.6528	70.2055	71.7796
75.9315	85.3409	88.5895
89.6816	97.5634	98.6787
103.5976	108.7960	109.6296
115.5548	118.5100	121.5590
145.8425	161.4357	169.6243
178.0169	189.2858	203.2587
206.1139	211.9460	218.0689
226.4044	231.2076	236.9751
243.0107	248.6611	251.3614
257.0296	261.6254	268.6615
279.5978	283.5263	310.3652
313.9426	316.6122	335.2692
353.2603	353.6866	357.2942
380.2741	381.8728	384.5103
390.0392	403.5033	408.8999
410.9201	420.4236	423.7872
430.2302	432.0346	437.5645
445.0366	451.2974	486.2068
488.0955	491.7417	496.9793
506.2301	511.8855	513.5737
513.9898	524.9599	526.8998
528.6968	535.3790	548.7588
564.1513	571.9099	580.3867
590.9720	597.1132	611.4352
630.2785	632.0934	637.4766
648.0114	651.0699	652.2549
698.2179	710.2359	710.6638
720.4023	722.1237	726.6810
729.0842	741.2694	744.7308
755.0162	759.2450	789.0288
798.2096	810.3327	817.0776
821.0760	831.0468	849.0055
849.3552	855.5994	859.2309
860.1152	871.9587	880.9407
882.6376	885.9254	887.9491
893.7121	899.2033	901.7091
911.2462	920.3537	921.0323

922.3618	934.8022	936.5698
939.0662	957.8043	965.2206
973.0950	979.2670	986.4513
989.3335	990.5244	1002.8434
1003.0751	1007.4292	1007.7141
1013.2473	1014.1544	1016.4375
1018.6608	1019.4946	1020.1558
1025.6507	1026.8340	1031.5949
1035.7328	1037.7623	1039.4687
1044.4176	1050.9325	1060.8590
1067.2746	1068.3894	1069.2195
1069.7463	1070.1027	1073.6080
1100.0622	1101.2630	1102.0711
1117.1646	1118.5228	1119.7119
1121.9084	1123.4568	1124.0621
1126.8905	1132.0344	1135.4463
1146.9735	1148.1167	1161.5655
1173.0528	1178.2501	1185.3295
1187.3298	1191.7504	1202.7299
1204.7730	1207.7782	1213.3647
1220.2700	1220.5219	1224.2514
1233.8626	1238.0335	1241.7214
1246.4928	1249.7107	1250.6841
1251.8043	1261.1075	1267.7967
1270.4021	1273.9776	1274.4682
1287.8054	1294.1561	1300.4772
1308.5654	1311.4316	1319.7379
1324.8394	1325.9446	1327.0969
1333.5711	1338.9653	1343.1306
1343.8556	1350.3076	1350.7578
1356.4966	1360.6325	1362.4137
1362.4543	1363.9341	1364.8579
1373.3449	1374.8238	1376.0781
1378.9568	1383.4006	1386.5922
1392.3879	1393.1978	1398.7905
1400.0889	1405.2853	1407.0671
1409.6794	1411.2876	1412.2521
1415.4257	1418.1527	1418.4543

1429.2246	1436.7279	1440.7432
1455.1249	1466.5144	1471.2250
1473.0384	1478.3558	1479.8861
1483.5975	1484.1470	1484.4515
1486.6453	1487.8312	1488.3119
1489.0771	1490.7319	1492.0846
1495.5872	1496.1073	1496.2212
1496.8120	1498.6326	1501.0218
1503.3027	1505.7516	1507.0800
1510.5644	1510.9053	1513.7915
1514.5876	1518.9475	1524.7575
1525.5422	1533.0333	1545.9432
1551.5990	1554.1566	1565.8566
1568.8892	1592.8066	1658.1788
1666.0577	1678.0302	1689.3974
1692.7211	1697.0397	1698.0931
1699.2158	1709.8379	1721.7140
2464.2218	3049.2370	3049.3653
3050.3021	3052.3031	3054.2927
3058.1464	3062.0957	3062.5721
3062.9443	3064.5041	3064.7220
3065.2584	3065.3587	3069.2212
3070.1424	3076.6251	3079.0346
3080.1737	3082.0130	3086.2183
3088.5035	3089.9055	3108.0100
3111.4202	3111.9492	3116.0422
3116.3101	3117.0081	3117.0524
3118.3880	3120.0950	3120.2860
3128.1042	3131.2855	3134.7560
3135.5553	3136.6889	3140.8634
3144.8642	3145.2106	3145.4661
3146.9039	3149.5323	3166.2730
3167.1456	3168.0816	3168.1552
3169.7339	3171.1652	3185.0415
3189.4317	3191.2990	3196.9355
3197.6515	3202.1646	3209.6698
3210.1953	3219.3755	3221.8440
3222.2766	3229.1122	3234.2701

TS4^{HOAr}

-1429.0478	13.4728	21.1350
21.5250	26.9069	30.6627
34.9350	42.5223	44.9364
53.3906	56.2470	65.4014
69.1851	71.0947	82.7314
97.0097	103.5502	105.5378
112.1593	123.0917	140.9811
152.0490	160.9834	165.5583
174.4487	186.4231	202.2058
209.4290	210.7369	235.9888
239.1090	248.5032	253.8225
265.6659	280.7961	287.0085
304.9277	314.5450	317.6889
339.1750	348.1103	372.4442
376.5001	385.6001	394.7652
419.6848	420.8727	422.8640
433.7940	435.9607	445.1739
469.0406	485.1298	495.4519
504.3949	512.4969	522.7858
524.2889	528.3867	540.1921
553.6442	554.6136	568.5985
581.7160	583.6366	589.0007
594.1936	604.4369	629.5795
630.1364	640.6707	647.3209
652.5590	665.2241	694.8165
710.7464	720.9047	722.0086
734.5060	751.4603	763.3632
775.1025	784.4926	785.2071
795.5138	833.8346	837.7426
844.2106	856.6371	863.0681
873.9401	878.5993	885.6227
889.6509	893.0666	898.0350
902.0282	926.9559	928.4280
959.4678	963.7334	966.0678
980.8095	990.6625	993.5127
998.1609	1003.0406	1010.5706

1010.8326	1011.8318	1013.6620
1014.9647	1018.6075	1026.9351
1031.8313	1038.4532	1042.1550
1046.2475	1060.0420	1065.1903
1065.9032	1068.9848	1069.3628
1070.2403	1073.4576	1100.7353
1115.4282	1119.2458	1123.5867
1132.1451	1150.0972	1172.6172
1174.6895	1182.2368	1198.6224
1201.9439	1204.7651	1206.0623
1208.9029	1214.5737	1218.1372
1222.8786	1245.5419	1252.8750
1269.1278	1281.6178	1290.5992
1298.1259	1318.0475	1325.7613
1332.4462	1337.0068	1342.9162
1344.1773	1346.8519	1359.1482
1360.2162	1361.4580	1363.9199
1366.7831	1385.4142	1386.9156
1396.5227	1403.5100	1413.9456
1421.7632	1425.7053	1431.2888
1432.0911	1449.0074	1452.4070
1465.7191	1486.6093	1489.8560
1493.5844	1496.4093	1496.4834
1498.6683	1499.9420	1503.0220
1504.2682	1506.1691	1509.0551
1519.1470	1523.4374	1545.8373
1551.7118	1552.1606	1567.4824
1591.8592	1600.8262	1637.7378
1663.1591	1673.8761	1679.0578
1679.9692	1685.9068	1686.5438
1695.7307	1698.4203	1700.5872
1702.0134	3046.6611	3056.7973
3067.7080	3068.5605	3069.5720
3096.3901	3107.5100	3128.1483
3138.0994	3138.4762	3148.1100
3162.2836	3170.5940	3171.8874
3173.5120	3174.4284	3183.0337
3183.3638	3186.1175	3191.3886

3192.2367	3195.1636	3199.2851
3202.0015	3205.6064	3209.5575
3215.6759	3216.8316	3218.5212
3219.8421	3223.1102	3229.6599
3242.2425	3247.3650	3619.9335

TS4Z^{HOAr}

-1357.1597	16.1808	18.7845
28.5090	31.2728	36.1010
37.7425	44.0694	51.7187
57.2764	59.3462	62.8173
69.0068	80.2266	85.7238
97.0559	101.7980	108.9881
112.9063	113.8725	125.3383
141.8249	151.6053	157.4107
169.4609	187.2475	193.9185
198.3554	209.7044	229.4103
234.4259	237.0090	240.7364
251.2530	262.3459	270.5416
282.9738	314.7153	323.7523
341.3393	350.9733	362.6381
371.1074	385.0850	388.7024
391.0615	423.7115	427.4814
430.9310	432.9881	448.0320
452.8891	471.4685	496.2785
497.3771	505.9071	508.9445
524.3727	524.8990	528.8383
547.3740	559.9746	560.9689
576.2328	585.2841	589.4790
592.6542	605.3299	630.6593
633.0383	636.9077	642.1908
652.9717	663.6201	696.5198
722.8924	723.6706	731.0543
742.6428	752.8945	756.1567
769.3942	780.6478	790.9765
792.3773	828.4685	834.6270
835.6969	849.8186	860.7577
880.6352	882.1176	882.8015

889.7107	894.1864	895.5020
900.4294	916.9429	948.4099
957.0371	965.8342	966.3248
978.8477	983.3071	997.7599
1002.1119	1004.8026	1006.4823
1012.6319	1012.9897	1013.5030
1016.9208	1019.7909	1022.5982
1032.0362	1038.3852	1043.4013
1049.2074	1058.7477	1064.6497
1066.9291	1069.0317	1069.3970
1070.3549	1075.5892	1092.0667
1116.8544	1120.2802	1122.5008
1125.2149	1141.8301	1150.9408
1176.6779	1178.6877	1181.4746
1198.0005	1203.2440	1204.2682
1205.6856	1209.4864	1229.5580
1231.6496	1243.0423	1246.9934
1253.3220	1267.1137	1283.7853
1293.1022	1315.1758	1328.4794
1335.6360	1338.1449	1342.3488
1344.8129	1352.6977	1358.6883
1359.6857	1360.4500	1362.2193
1376.3970	1386.8539	1399.1617
1404.5532	1405.7580	1417.8921
1421.7519	1422.3309	1431.5610
1432.6794	1443.3929	1459.1027
1466.8977	1473.5641	1486.3844
1489.0309	1489.7387	1493.7992
1495.6038	1498.6023	1499.3306
1499.9164	1502.2487	1507.1990
1511.6391	1516.5296	1524.0568
1545.6083	1552.1267	1554.1906
1578.2307	1604.5242	1624.1369
1659.3025	1676.5028	1677.9612
1684.0527	1686.5623	1688.5177
1688.9751	1696.1288	1699.1583
1701.5858	3045.4034	3063.5318
3066.5615	3067.9567	3077.5193

3085.8520	3122.3369	3133.3424
3135.6574	3141.0130	3148.3444
3162.8535	3170.9208	3170.9779
3172.9684	3173.3151	3176.2900
3179.6135	3186.7096	3192.1884
3193.3142	3193.4167	3193.8324
3198.8769	3205.1440	3209.1668
3211.3830	3211.5244	3220.5308
3228.1294	3230.0903	3234.5996
3246.7377	3252.1100	3759.7876

TS4^{DH+}

-1356.9730	13.9641	20.9369
26.1835	27.6212	30.6965
36.0475	45.1400	46.8609
53.2756	60.5638	68.8097
70.1896	75.7117	81.0761
88.2586	89.6399	98.1610
112.2121	115.0087	121.2210
129.7696	143.3682	149.3013
153.6870	166.4397	172.3703
194.8888	198.5896	206.9538
213.6365	218.3401	237.7585
243.3720	245.0857	251.1893
260.0174	265.3738	274.6563
281.0247	312.8010	318.5875
335.8640	350.6617	365.8166
377.2337	384.1020	393.4710
403.9498	410.8916	418.4625
420.0222	420.8242	429.3635
437.9171	453.6917	481.4953
495.6415	499.5098	505.5536
518.0710	522.0553	525.5716
529.5249	535.9583	541.9952
559.2324	562.5962	579.1137
585.5334	586.2678	589.0171
629.5752	632.5169	633.8501
649.0522	651.5450	699.2674

713.3543	720.4026	721.1030
733.9566	735.6180	746.9033
756.5472	766.9257	776.3282
793.8192	818.4261	836.3486
845.6192	853.1620	857.6947
864.5524	875.9955	882.9933
884.3361	889.4159	894.7905
900.4583	905.9093	920.7901
922.8539	938.0907	950.5822
955.0202	959.7880	963.5730
980.0231	991.7726	1001.2430
1004.5855	1005.9851	1007.6581
1013.5290	1014.3531	1019.2798
1020.2453	1022.0645	1026.5159
1028.4207	1032.7659	1038.3093
1039.7470	1050.0096	1059.6247
1061.7757	1063.7306	1069.7931
1070.1102	1074.9429	1077.2632
1096.6870	1102.4356	1115.4647
1118.1981	1118.7231	1125.2483
1133.4173	1137.1091	1151.2779
1172.8643	1174.6704	1185.1862
1189.0753	1200.5973	1207.6987
1209.4431	1214.4540	1216.7053
1218.6931	1221.5049	1233.4267
1240.2343	1243.8161	1250.5884
1251.9517	1264.4627	1268.5790
1272.3547	1272.9645	1285.2702
1297.8837	1313.1392	1317.4209
1324.1799	1329.1780	1333.6730
1340.4440	1341.9980	1346.6091
1351.3256	1354.8199	1356.7723
1361.5457	1363.9514	1368.7452
1372.0125	1375.9092	1383.1724
1383.7308	1390.8931	1396.2143
1396.4480	1405.0660	1409.2634
1414.9233	1416.6251	1416.9442
1425.9189	1428.7657	1434.3122

1455.3140	1464.9329	1473.7908
1481.3769	1482.2971	1486.4245
1487.8960	1488.1630	1490.6852
1492.4928	1495.5280	1497.3819
1498.6758	1500.1049	1501.6587
1502.5258	1513.4196	1513.6646
1514.5922	1517.6808	1518.1160
1523.2516	1534.5095	1549.5766
1553.6292	1559.9415	1585.5686
1588.7858	1664.3419	1675.8532
1681.2605	1687.5212	1689.5415
1693.2338	1699.8514	1705.1413
1719.7770	3042.4097	3051.3967
3051.8112	3060.7139	3063.6215
3066.8746	3067.5742	3068.5648
3069.2198	3074.8196	3075.2218
3079.5440	3086.1333	3087.9359
3107.2151	3109.0204	3117.2603
3121.7999	3125.1896	3129.1422
3130.9260	3137.7956	3140.9857
3144.0066	3153.0216	3163.3625
3164.3051	3175.0669	3176.5206
3176.6280	3176.6553	3179.5120
3181.6564	3185.8782	3187.5346
3193.2932	3193.8519	3202.0606
3205.4221	3207.4360	3211.1845
3213.2398	3223.2418	3226.3619
3231.4664	3246.4921	3839.0216

TS4Z^{DH+}

-1341.4004	12.2868	14.5736
17.4258	19.3927	26.0197
27.6376	34.1314	39.7012
41.0972	44.2765	56.3669
62.8450	66.5547	71.1712
77.0844	92.7806	95.0362
96.8032	104.5879	113.2689
117.1378	135.2259	144.7653
158.0276	166.7584	171.5960

189.8580	194.2128	205.4577
212.6906	227.0051	229.5871
239.1259	247.8234	250.8936
254.7072	260.9103	278.2558
281.9613	309.4712	319.5615
336.1288	351.1579	355.8986
377.8737	383.2194	386.1363
394.4665	408.9129	420.6811
422.0324	432.3722	435.9164
437.7732	442.1032	480.5413
487.5535	490.8842	501.9208
509.9138	517.7790	521.7116
525.9085	527.7615	545.6590
562.3112	570.3023	582.7106
587.4862	588.9908	611.4200
630.2559	631.2954	638.4253
649.9094	655.1981	699.4356
708.0023	721.1791	727.2080
729.4933	741.9182	755.1401
757.0277	762.3407	784.2265
793.9800	809.2529	818.9284
828.5898	849.5298	857.2524
862.4151	882.9179	884.2794
886.1543	887.0022	890.6821
905.7825	915.0866	922.2469
923.5625	937.8610	953.7042
958.5438	962.5055	975.3074
979.4510	992.6791	1004.0774
1005.3826	1006.0680	1010.3048
1011.0482	1014.8987	1016.0928
1016.3368	1021.4447	1025.4004
1031.4875	1036.4710	1039.1262
1039.5602	1050.4108	1059.4642
1063.5363	1065.9261	1066.2690
1067.1406	1069.2754	1072.9120
1095.9046	1103.8746	1112.1993
1119.4768	1119.6595	1122.4392
1125.3397	1138.1029	1146.8096

1148.3212	1173.9437	1177.5002
1180.9582	1187.1057	1197.9696
1200.9537	1201.9916	1206.5310
1220.2683	1221.9545	1226.5658
1241.3429	1243.8956	1246.3383
1250.8351	1268.6608	1268.9922
1275.7706	1281.3600	1295.2484
1299.8631	1304.3988	1321.5228
1324.6511	1328.5687	1330.1832
1333.2538	1337.3134	1341.3712
1346.6721	1352.4306	1353.3577
1357.2405	1358.2868	1366.1916
1370.8587	1377.1491	1383.0907
1386.4757	1395.0077	1395.8817
1397.5433	1407.2528	1410.8412
1416.9648	1419.9297	1420.9198
1429.5411	1430.7572	1433.7428
1454.7411	1463.2082	1473.3072
1480.6236	1486.3508	1487.6071
1489.0792	1489.5761	1490.5116
1491.2157	1493.4548	1493.6783
1494.7495	1497.7266	1498.1712
1503.3149	1504.2384	1511.1096
1512.0444	1515.9418	1517.9658
1521.2431	1538.3568	1543.1708
1548.2749	1548.7183	1598.9016
1610.7804	1654.6477	1664.3789
1671.5255	1680.0613	1682.4441
1688.3960	1690.5078	1692.8269
1715.8654	3038.3189	3053.2129
3056.9487	3058.2689	3068.7092
3069.0078	3070.3452	3071.4909
3073.4530	3079.2474	3084.2834
3089.6198	3090.4948	3092.6679
3118.2520	3120.3666	3121.9264
3124.9828	3126.2416	3128.1140
3135.4728	3139.1521	3148.6421
3149.7318	3152.7542	3155.6445

3157.6180	3165.9576	3172.3762
3174.2063	3174.7497	3180.0638
3183.5975	3186.4829	3202.1921
3207.9231	3208.6758	3212.0958
3213.1961	3213.2511	3221.4110
3222.3278	3226.8926	3229.2285
3240.9155	3241.4892	3738.4152

TS4^{DBU}

-1190.0292	8.0582	11.7458
17.5233	24.0498	27.0127
34.8556	38.9382	42.8081
51.0426	57.5095	60.6920
71.5379	74.0283	77.7514
82.8955	90.0171	95.4405
98.4500	101.9654	111.0108
129.8547	150.7594	168.2108
174.7946	191.0830	194.9857
198.9269	204.0005	207.8734
216.7189	229.4256	240.4981
241.4464	248.3266	252.0124
265.0991	271.3954	278.5019
306.8315	312.6062	330.5946
347.8318	354.9914	367.8777
375.8486	388.5331	393.2877
405.5967	418.3980	419.1853
420.7094	429.2322	432.1150
442.3506	470.9942	486.1866
492.6878	504.2185	515.8245
522.1877	525.7191	526.3607
535.9794	551.9156	559.2310
573.7202	580.1302	585.0373
588.7728	617.6862	628.6671
629.4194	633.7335	648.8801
654.6789	684.3631	707.9004
714.2353	717.5737	725.5061
727.5250	741.5374	759.6754
766.1026	781.0840	797.1028

807.5865	819.9241	838.2177
841.6649	852.6472	855.3904
857.1338	879.4167	881.1189
882.3389	890.2651	891.8572
897.8404	904.1432	911.9468
922.8998	938.4845	950.4569
959.9487	963.1404	977.4494
981.2029	996.1444	997.9509
998.3476	1003.4390	1006.2780
1007.6309	1013.9925	1017.2257
1028.6913	1033.5339	1038.5552
1040.8034	1044.2162	1045.3227
1050.1657	1061.1737	1064.1649
1064.9150	1066.4059	1067.2955
1071.9012	1074.6953	1102.9753
1104.1819	1109.8317	1119.6412
1121.7430	1125.6082	1128.7143
1140.7934	1147.0723	1158.6312
1169.7572	1173.3612	1187.7979
1191.2121	1196.0484	1198.2475
1205.1833	1212.3920	1217.7512
1223.4889	1229.7427	1239.8391
1243.8495	1248.4527	1254.1899
1261.6790	1264.7006	1269.7345
1278.2813	1282.0099	1294.1723
1310.2260	1323.7951	1326.0433
1332.4589	1334.1849	1337.0793
1341.3189	1345.0598	1356.0292
1358.4031	1359.6488	1362.5815
1363.6754	1365.3828	1377.7254
1381.8065	1387.2604	1389.2445
1394.4875	1395.6071	1407.7731
1412.5481	1413.2151	1417.3025
1422.4424	1427.4970	1429.3235
1444.2075	1466.1924	1467.9333
1469.1050	1469.4007	1478.6519
1482.0044	1484.1184	1488.6724
1489.7187	1489.9878	1491.4847

1492.0760	1493.8009	1496.7320
1498.2659	1501.0657	1504.5926
1505.8964	1508.9240	1509.4667
1516.2788	1520.5808	1525.9854
1532.9299	1547.7362	1552.3814
1556.9970	1615.2727	1644.6069
1649.1885	1676.4202	1687.8535
1688.4168	1690.4265	1697.1858
1698.2396	1726.0896	3032.4241
3051.2145	3053.8995	3059.2867
3060.1587	3062.7425	3066.7103
3066.9200	3067.6341	3069.5533
3072.1127	3076.1280	3088.1967
3094.1912	3111.5244	3115.8864
3122.5877	3126.6905	3129.6720
3131.1462	3136.1266	3138.6148
3141.2678	3144.7822	3148.8023
3149.0338	3161.4916	3162.2763
3164.8673	3166.8370	3167.0588
3169.1778	3170.0868	3173.6145
3183.1068	3184.5783	3188.6571
3189.1415	3190.5983	3191.2629
3191.6872	3199.8324	3207.7187
3219.2560	3220.7161	3238.0087

TS4Z^{DBU}

-1118.9201	6.7485	13.7188
24.0337	26.1511	28.3931
31.1488	39.0261	43.9058
50.4831	56.4264	59.1889
67.3866	73.9397	80.8869
87.3109	90.8807	101.7193
106.9047	117.6550	119.3031
124.0139	125.8318	164.0439
167.7755	182.1982	185.9632
196.8813	203.8249	210.5389
216.8336	228.9551	239.4065
248.5882	249.5136	259.9975

261.0169	275.9575	285.6574
314.6821	321.6315	340.8165
352.0138	356.2788	376.8322
380.4921	388.4970	396.4643
402.3755	423.1688	426.4030
430.1676	434.0552	436.6881
446.3463	487.1271	489.3111
496.6583	506.1874	519.8103
520.4179	526.6645	528.0908
530.8402	554.9563	567.8972
575.6642	584.4383	588.4646
600.0710	628.4899	631.2811
632.8422	640.0853	654.0431
659.9514	693.3679	704.5948
723.8857	724.9254	734.9811
740.0316	748.6774	757.5157
760.2200	789.0499	798.2358
814.9149	821.4926	827.5006
845.1228	854.0724	860.2994
873.1956	878.2086	885.0906
888.2747	889.6411	889.8931
917.3010	920.6614	921.6583
923.8297	943.9427	952.3450
960.4145	966.6737	980.6814
993.0134	993.2748	1003.4492
1005.6707	1006.5950	1011.0562
1014.1635	1017.7431	1018.7392
1023.9581	1029.9180	1036.6358
1037.7914	1041.8655	1045.8731
1049.8748	1057.9931	1064.1272
1064.5288	1068.9780	1070.7147
1074.1465	1075.3810	1091.7719
1107.8994	1115.1673	1119.2627
1120.2474	1127.3658	1128.9435
1144.1258	1147.9072	1152.8028
1172.9322	1185.5596	1187.4158
1194.1163	1201.8793	1207.0383
1210.6303	1214.6315	1221.0792

1225.9634	1235.5339	1243.5885
1246.3261	1249.0740	1258.8520
1266.5018	1269.7168	1279.2096
1284.3306	1286.7763	1300.5116
1313.1307	1324.9058	1331.5214
1334.0978	1335.5303	1339.6404
1340.6947	1347.7076	1359.5970
1365.2553	1366.6979	1367.7440
1370.4158	1372.6179	1380.1696
1383.7259	1388.6444	1394.8395
1398.7016	1409.0391	1413.7624
1415.4848	1417.9120	1420.9445
1429.0049	1433.9633	1447.3714
1450.6201	1459.8711	1468.3782
1470.6401	1479.5735	1480.4503
1486.5594	1488.7723	1490.1920
1492.4331	1492.7776	1493.9742
1494.0858	1497.3784	1501.8750
1502.2364	1508.3453	1509.5073
1510.1845	1513.3506	1515.3283
1517.5670	1525.5615	1535.0060
1548.9137	1550.1591	1556.4717
1557.2941	1631.2170	1642.8984
1650.1818	1679.7428	1685.2594
1687.2935	1693.2612	1699.0755
1700.0394	1734.0422	3026.0589
3039.6546	3052.5860	3056.5159
3063.8555	3064.1266	3065.6471
3066.6711	3070.3674	3073.3131
3075.8296	3077.4581	3078.1520
3086.6072	3088.7106	3112.5188
3113.2830	3114.9783	3119.9050
3127.8283	3134.7394	3137.4419
3138.0294	3141.1311	3146.0042
3149.0257	3155.0393	3162.5167
3163.2883	3165.6545	3168.5688
3169.2033	3173.5486	3178.0408
3179.2639	3183.7113	3187.7314

3190.2893	3191.6121	3192.3857
3199.1075	3199.5704	3206.9893
3217.9413	3218.4881	3236.1163

TS4-5°

-1864.5680	11.8334	13.2319
18.4105	23.1684	26.7442
28.2649	32.8343	37.5908
42.8529	51.3234	53.2489
57.9586	62.9039	67.2677
68.1799	69.2662	71.9482
75.7136	79.1729	85.1965
95.9356	101.1416	104.0469
113.7892	117.2043	125.7229
130.0843	134.6320	139.6218
141.7297	160.2081	164.6392
168.3453	173.8428	175.3880
178.1710	183.7599	184.0565
198.7424	204.6620	205.6214
215.3203	223.0022	227.7765
229.8042	234.3376	245.5330
247.8132	254.2129	257.2978
262.1172	264.0572	275.9200
280.3523	282.5328	286.3956
287.4844	289.1371	296.3536
301.2099	305.3237	316.6413
320.4589	322.7144	329.5890
332.8457	338.4444	341.9797
347.8212	348.5622	352.7505
355.4998	357.2161	367.3125
373.2498	378.0650	381.5008
382.5504	388.1950	393.6397
399.3869	402.6581	406.0506
407.7637	414.2623	418.1879
420.4020	422.9353	427.9893
438.2408	443.2637	445.6241
460.6519	462.4539	470.8786
478.0001	489.7399	504.7334

510.7095	521.6916	528.4977
529.5269	531.9298	537.3317
539.0745	551.6379	555.1167
565.2660	576.2424	579.0345
585.8995	589.0744	590.6725
592.5801	616.8119	623.6223
627.9928	629.5255	632.0221
645.6135	672.5893	677.2158
688.1091	696.9235	711.7761
715.3047	721.3360	740.0700
753.4179	768.4166	768.8952
769.5313	770.6393	781.8973
786.3128	795.9364	802.2968
806.5808	817.6299	831.8372
838.4904	841.8102	865.4019
866.9935	873.2740	880.9668
884.7575	891.2128	893.0641
900.4526	906.0871	913.0936
915.5101	918.9218	919.8659
930.3430	932.7879	948.0377
948.6612	949.1145	949.4083
951.0365	953.3854	954.8405
956.1734	956.6081	958.9374
960.6115	961.9142	962.9139
964.3857	969.7860	972.8414
984.7968	1000.0710	1003.0480
1003.7957	1004.8750	1012.6178
1017.5011	1020.6018	1028.9898
1038.6016	1043.0030	1045.2246
1047.3552	1049.4696	1051.0111
1051.2506	1053.6202	1054.1514
1056.3078	1057.3576	1062.6302
1063.6933	1067.0837	1068.7099
1069.8110	1071.3492	1077.4572
1080.7241	1092.6775	1102.6449
1115.1271	1121.5530	1123.4503
1136.3752	1137.7895	1168.4197
1171.1689	1174.1798	1176.6296

1190.4456	1196.9936	1199.9850
1208.0880	1213.2830	1214.4970
1221.3706	1230.6669	1233.0595
1240.4893	1243.1415	1244.0808
1245.8436	1246.3993	1252.2901
1252.9848	1255.0612	1258.1202
1261.3212	1263.9045	1285.0592
1297.6878	1300.0062	1315.5817
1317.5500	1319.3877	1328.1329
1339.8921	1341.7534	1348.9891
1352.4449	1357.3721	1360.4424
1361.7687	1364.3421	1367.4500
1371.1466	1374.0710	1380.5842
1382.8016	1385.6168	1388.6810
1389.1553	1391.0693	1392.4246
1395.8206	1397.0798	1398.5609
1400.6052	1404.2716	1410.2904
1419.8573	1423.7633	1424.9471
1425.1159	1425.7244	1428.1459
1430.4653	1436.8927	1462.6536
1467.9173	1470.9139	1482.6902
1483.8109	1484.7526	1485.2471
1486.6905	1488.9648	1491.4112
1492.8557	1494.3091	1494.7925
1497.7946	1498.4216	1498.7816
1499.4239	1500.7078	1501.6777
1502.2442	1502.5284	1503.1275
1504.0235	1504.2977	1505.4311
1506.0949	1507.2773	1509.4585
1510.5806	1511.7776	1514.5542
1516.5217	1516.7006	1519.1530
1524.7612	1525.8518	1528.5117
1531.5806	1534.0808	1535.8498
1537.9429	1543.2547	1546.1187
1549.2798	1549.6489	1583.4052
1586.8509	1595.2800	1621.7252
1625.7263	1654.2465	1672.8392
1672.9459	1676.8594	1686.0936

1688.2594	1691.5396	1697.9500
1698.6454	1708.2151	3048.6009
3049.6827	3050.2270	3050.9957
3051.2895	3052.6859	3053.4983
3053.5545	3054.2662	3056.7721
3057.1713	3059.5266	3068.3622
3068.8071	3073.0439	3078.1009
3078.9016	3090.0762	3108.2628
3119.3777	3121.7843	3124.3518
3125.1113	3126.9421	3127.6092
3130.1122	3132.6206	3134.4250
3134.5806	3135.0210	3136.5687
3137.8853	3137.9632	3138.2438
3140.1227	3140.3546	3141.1238
3145.2011	3149.2625	3157.9183
3159.4871	3168.8855	3170.4800
3170.5127	3170.6050	3177.8094
3178.0081	3178.1170	3179.3402
3184.0781	3186.2458	3192.6571
3193.9623	3195.3403	3196.5800
3198.3514	3199.4990	3201.7813
3201.9979	3203.7083	3209.5974
3211.1755	3217.3702	3219.7680
3222.1559	3225.4813	3231.0949
3232.8778	3259.0493	3268.4260

TS4-5Z^{O}

-1858.4338	8.1778	13.4124
15.8649	17.9862	21.4222
26.8824	31.3430	34.0169
38.2461	40.0849	43.4108
44.5369	48.6992	50.9108
55.8147	57.0730	59.4972
65.4014	71.4972	73.9223
77.0734	78.1187	85.6731
91.0728	104.7664	107.6600
112.1234	122.1275	126.4219
137.0210	139.3467	148.6463

153.2442	160.3506	162.3390
170.0488	171.5983	175.4891
183.8398	196.1787	199.1589
212.3249	215.0969	220.1464
226.1929	228.3124	231.0069
232.6983	235.6622	240.0681
249.7678	256.6239	261.5187
268.2012	269.1423	276.9301
278.8153	279.6000	281.6901
292.3567	294.9786	300.9482
308.1185	311.5729	317.9903
321.7973	323.6789	329.4722
337.3382	346.5843	349.2435
353.2591	355.9391	364.1099
368.9310	371.1457	375.2052
382.0140	384.9824	390.2879
392.1428	398.7374	403.5052
404.9533	406.6772	416.3668
421.5902	424.9313	436.2458
439.2680	442.7722	449.0817
461.8288	462.4712	472.6797
480.4630	495.0157	497.1413
506.6274	509.6815	517.4735
520.1533	526.3500	528.7723
535.4207	539.9635	556.3324
561.6425	573.0446	580.4351
585.6650	589.4660	594.9483
611.5304	614.9959	626.5242
628.9283	632.6009	642.8760
649.0634	668.1272	676.7003
685.7737	707.7623	711.4116
712.2707	721.9607	733.9260
739.6252	752.0346	768.7827
769.4485	773.8594	780.0875
786.9346	793.7090	805.5934
811.9617	816.4553	828.4060
832.9996	842.0199	855.7084
861.6466	865.7549	875.9521

877.6799	884.8467	889.7730
895.3864	902.9290	915.0859
917.9338	918.5996	919.3838
927.0471	929.9538	942.8821
946.4350	947.8863	950.7604
951.4785	952.0158	954.4347
955.3322	957.3598	959.2423
961.5520	962.0537	964.5596
965.7683	965.9160	971.2202
982.6468	993.7910	1001.4155
1005.0797	1013.0760	1013.4671
1016.0415	1019.0305	1028.9028
1035.0575	1039.2022	1040.5294
1041.4447	1049.2757	1050.1930
1051.8679	1052.3524	1052.8833
1056.0562	1057.0096	1059.6895
1061.6710	1063.8961	1065.4989
1068.7635	1072.3445	1073.5801
1074.7099	1090.8312	1094.4237
1123.7813	1124.8814	1128.5884
1131.5900	1138.1286	1166.0996
1173.5699	1185.0161	1188.2334
1197.6350	1208.4277	1210.6244
1211.7112	1212.3302	1219.6876
1222.1791	1232.2921	1242.2412
1245.9563	1246.2325	1246.5087
1247.7698	1250.2298	1250.5475
1252.1729	1254.3976	1256.0772
1257.8179	1271.0920	1284.9914
1293.0906	1300.4000	1316.5458
1318.4817	1325.6270	1331.0397
1339.3220	1341.8344	1344.4415
1345.3166	1346.9354	1360.8170
1361.6620	1365.8753	1366.6562
1369.8679	1377.2817	1382.1783
1386.5715	1387.0332	1390.7838
1391.4287	1391.9998	1395.4080
1397.4786	1399.2187	1404.7671

1406.1604	1408.2092	1413.8689
1416.2389	1425.1506	1426.4405
1426.9793	1428.8175	1430.6834
1437.4717	1446.3401	1452.0453
1467.3058	1472.3778	1477.3911
1481.8262	1483.3457	1484.4031
1485.2409	1486.9577	1490.0663
1492.4435	1493.6675	1494.4806
1494.6676	1495.3904	1497.0335
1498.5694	1499.6907	1501.2178
1501.4886	1501.6012	1502.0389
1502.5624	1503.0585	1503.7600
1505.3015	1507.3408	1508.0592
1511.2845	1514.6273	1514.8170
1517.1338	1517.9710	1518.5641
1518.9307	1522.9702	1526.3311
1530.0109	1533.5283	1534.6474
1535.9815	1537.0856	1544.5835
1546.9928	1556.6715	1583.6344
1587.6691	1596.9185	1625.1010
1651.2615	1655.9201	1670.7324
1679.8783	1680.9745	1682.5070
1690.9429	1692.7156	1698.3316
1699.6887	1710.4081	3044.8045
3051.3100	3052.3503	3054.3711
3055.4817	3058.2737	3058.6403
3059.1381	3059.8709	3062.4595
3062.9090	3063.2164	3063.7078
3063.9398	3068.0655	3070.7557
3071.7817	3083.6062	3117.7219
3120.9972	3124.2147	3127.5272
3128.9144	3129.9408	3130.3414
3130.8917	3131.8646	3132.9008
3133.0477	3136.8219	3137.6288
3138.0556	3139.1153	3139.2685
3141.5740	3142.3817	3145.3675
3146.0598	3147.0689	3150.5064
3155.0060	3167.5853	3172.4815

3173.3570	3176.7611	3177.4833
3179.7010	3182.5764	3184.7938
3190.0207	3191.2419	3191.8447
3194.5504	3195.5828	3197.5579
3197.8065	3201.6237	3203.6667
3204.9935	3211.4221	3214.7919
3219.4465	3222.6640	3223.7891
3225.5475	3237.8159	3237.8750
3255.6721	3260.6891	3272.4802

TS5

-1027.8625	14.1787	16.0399
24.9896	25.9109	34.3399
35.4310	39.7530	41.8760
42.1870	45.4623	50.9966
56.7168	60.5988	63.5328
66.2027	72.3863	77.7278
82.6203	83.2588	91.4087
99.1987	103.7332	110.9894
116.8110	122.9064	124.3554
133.9024	139.1374	143.3756
150.1799	153.6623	157.6856
167.8937	171.1599	179.3555
182.8732	189.7575	193.9690
203.3129	211.0646	213.9327
221.6997	227.3188	231.6066
236.7627	242.1634	243.7980
253.8236	260.6741	263.6915
269.0753	275.1100	277.1765
278.8974	283.6996	289.2162
293.9586	298.5359	302.7240
305.5826	307.9947	310.1506
315.4330	318.6331	329.7319
332.5142	332.7047	342.4244
343.9699	353.6554	355.9145
362.8470	364.4572	369.5857
373.5520	382.6595	384.3084
388.2253	389.4155	392.6584

397.9609	400.4221	403.0539
406.5638	413.3431	416.0298
417.5222	420.3203	421.0443
441.5112	443.2489	445.8194
464.0272	465.4501	467.2804
468.2297	498.5130	506.6298
508.5432	520.8484	524.9210
526.0845	528.0865	534.6620
537.8534	546.7691	554.2726
566.7301	574.1854	575.4026
584.7686	587.7473	598.1182
598.3987	607.4862	625.7311
627.6366	630.7815	640.9680
657.8332	667.9942	669.8200
685.7826	695.8364	702.2760
711.4585	724.2425	744.4748
747.5741	756.3405	768.2710
774.0436	775.0594	781.4653
793.2351	800.9591	817.2039
825.5807	833.4340	842.3212
849.4986	852.3971	857.9968
872.5419	880.8338	883.2630
883.5010	890.0268	900.0031
907.3616	907.5314	913.8971
919.1116	919.2237	924.2900
930.4672	936.5030	949.0540
949.7073	950.2900	950.7312
950.9194	954.6611	955.0930
955.6869	958.9294	961.6694
962.7577	963.1437	963.9551
965.8358	967.4544	972.2443
983.1173	988.9254	996.6567
1003.6287	1008.7295	1011.3847
1013.7007	1016.4527	1030.4114
1039.0479	1039.5408	1040.6545
1044.8114	1049.5819	1049.7636
1049.9923	1053.1756	1054.7935
1056.2856	1057.5490	1057.8111

1060.8183	1062.6859	1064.0208
1064.6833	1070.5438	1071.1093
1082.3957	1104.0381	1105.0297
1119.6533	1123.3583	1130.6223
1134.2878	1148.1183	1173.1022
1177.3369	1179.1691	1194.0015
1201.1005	1204.6169	1206.8218
1210.3316	1220.5126	1231.7517
1236.8755	1240.8504	1241.1170
1243.0045	1243.6507	1246.8262
1249.9074	1252.0892	1253.4692
1254.6302	1257.0297	1262.9854
1265.5854	1283.5048	1287.6370
1297.1465	1301.5520	1316.1434
1319.2623	1324.2837	1331.2283
1341.0925	1343.9403	1346.9594
1349.8642	1352.1124	1356.3752
1360.7366	1363.4105	1364.6162
1373.9584	1375.0022	1382.5375
1384.2001	1388.9205	1391.9941
1392.2775	1394.1975	1394.9079
1397.6684	1401.6856	1404.2443
1408.3452	1409.4994	1415.9048
1420.8487	1422.7242	1424.5137
1427.0199	1429.2091	1430.4330
1436.5022	1448.9643	1459.8655
1461.5853	1465.9349	1481.1893
1483.8941	1484.6685	1485.5875
1485.8281	1486.0429	1486.4977
1492.5600	1492.6516	1493.6318
1493.8274	1495.8474	1498.2401
1498.4722	1499.3826	1500.0336
1501.0050	1502.4683	1502.8504
1503.5442	1503.9220	1505.1633
1505.6150	1505.7595	1506.8492
1507.5984	1508.0097	1512.8689
1514.7083	1517.7329	1518.2862
1519.4254	1520.2831	1523.8795

1525.2845	1534.7974	1535.4413
1536.3387	1537.5467	1541.8883
1547.4965	1555.4663	1567.2591
1584.1209	1588.8254	1611.1039
1621.3629	1640.0180	1661.5006
1677.3907	1683.1042	1685.9815
1688.4675	1692.0471	1697.3136
1699.6979	1705.2884	3049.4140
3051.0438	3053.3885	3055.5853
3055.8684	3056.6180	3057.3980
3058.0077	3060.5120	3062.3585
3064.4937	3065.3586	3068.2476
3069.4495	3069.5724	3071.8129
3081.3643	3089.2741	3126.1481
3126.3855	3126.9615	3128.5004
3128.8301	3131.2499	3132.6458
3133.3286	3134.0999	3134.8022
3136.6598	3137.0325	3137.4536
3139.4134	3144.0157	3147.6320
3149.6925	3149.7909	3153.4102
3163.4382	3169.5312	3171.3131
3174.7284	3175.8403	3177.9731
3179.1299	3180.0462	3183.4232
3184.3474	3186.2541	3190.7463
3193.9708	3194.2491	3194.8852
3195.0250	3195.9072	3197.3296
3200.7639	3201.5209	3203.9409
3209.3963	3210.0897	3212.5008
3218.7337	3223.6882	3227.6807
3231.3297	3233.3949	3246.8525
3247.0681	3250.4499	3259.0397

TS5$_{HTC}$

-1370.1044	20.6483	26.1719
29.8297	34.8294	40.0536
43.0170	48.2641	51.7680
54.0829	56.8806	61.3079
63.4473	67.5544	71.6454

75.4507	76.4791	79.6400
86.4951	91.0955	94.0009
99.2522	103.2251	105.3102
111.3968	112.6942	124.8336
131.1743	136.2947	140.3679
142.8027	159.4194	161.6695
164.2403	171.5023	182.0639
187.5113	192.8297	198.9921
210.8642	216.5567	221.0927
224.6981	229.2844	231.3536
240.2822	244.0465	246.8829
248.6239	253.2651	263.1117
271.5509	272.6722	277.0613
279.1322	280.6516	281.7631
288.0317	294.7650	301.8860
302.2646	307.3892	317.9472
318.3192	325.9937	331.5669
333.3108	341.6653	345.3811
352.1905	355.2871	362.4658
364.2470	367.0737	370.3800
375.7297	378.3567	380.2723
387.3875	393.2912	399.5150
400.1927	400.5187	407.0974
409.8335	412.6670	416.5199
420.8845	426.3578	426.4155
429.7459	442.6183	449.4046
450.4846	467.9713	472.1129
474.5570	490.2797	502.6430
507.1820	514.9531	524.0304
526.8005	528.3390	533.1439
533.9789	543.5979	552.1843
559.4004	565.8150	570.5884
579.8423	587.8538	592.8696
600.7274	615.9035	624.9892
630.6018	631.4675	638.4818
650.2660	653.6212	662.5381
683.3401	697.0713	700.0336
710.0273	723.0861	745.6437

758.1501	759.8390	768.1424
771.0241	779.1954	784.2208
790.1207	795.8239	819.3587
826.8689	832.1073	837.3225
840.7520	843.9362	859.0791
866.1497	866.8510	881.3633
889.2059	892.5233	901.6657
909.3816	913.5754	921.2975
925.2645	929.2271	930.4078
939.0905	946.4765	946.7465
947.6646	949.9359	953.1210
955.1930	956.2359	957.3468
957.6284	958.4360	961.1722
963.0738	967.0028	970.8232
971.3655	975.1861	977.9621
982.8147	996.3662	997.2352
1008.6553	1010.3495	1014.0661
1018.2792	1020.0833	1029.2645
1037.2193	1039.0253	1040.9197
1046.7607	1046.8374	1049.4831
1052.2094	1054.3418	1055.5059
1058.1018	1058.5857	1061.7239
1063.4045	1064.4925	1069.7909
1071.5700	1072.4469	1073.4008
1075.0769	1081.9905	1101.6251
1109.9308	1121.4644	1126.2624
1128.5998	1135.0593	1146.3340
1171.2454	1176.7139	1180.1910
1200.5638	1204.1579	1204.5802
1210.8536	1215.6719	1218.9838
1227.7211	1239.4862	1240.6710
1244.4009	1244.9730	1246.1485
1247.3600	1250.0809	1252.6426
1256.0280	1256.9952	1260.7008
1268.6150	1273.7835	1285.2198
1288.8725	1302.5461	1308.0108
1313.6342	1323.2064	1330.3400
1340.2920	1341.2348	1344.0379

1348.1245	1348.5329	1357.8160
1359.2444	1362.4158	1364.4829
1364.9998	1376.0967	1377.7578
1382.7666	1388.1071	1390.0054
1390.4762	1393.5881	1394.2679
1394.9740	1395.6195	1397.2171
1400.8524	1402.8140	1405.5049
1416.4872	1417.2406	1420.9268
1423.1586	1424.5561	1425.8581
1429.0520	1431.2275	1433.4315
1455.4323	1466.5406	1467.2630
1480.3128	1483.8515	1485.0364
1485.1203	1486.6569	1489.4371
1490.1820	1490.8949	1491.1405
1492.7165	1493.6345	1495.7662
1497.1722	1498.6618	1499.9089
1502.3247	1502.5874	1504.0130
1505.6724	1505.9182	1506.2913
1506.9894	1507.4974	1507.8564
1509.2433	1511.6153	1513.3529
1513.7416	1514.5113	1518.4043
1520.5470	1521.9441	1522.5799
1524.1174	1538.2649	1538.5569
1539.0438	1541.5893	1543.7054
1549.2299	1552.8204	1585.6939
1590.3349	1596.8288	1629.1759
1655.7128	1661.2727	1670.5402
1676.1218	1678.4377	1686.0849
1686.2787	1695.3174	1699.2526
1703.3287	1708.0839	3047.2844
3047.7162	3047.8670	3050.4355
3050.6506	3051.4054	3054.1078
3054.6705	3059.1785	3059.3005
3059.3899	3060.3371	3060.8425
3063.1874	3063.4934	3064.6503
3070.7738	3076.9774	3122.5423
3125.2630	3125.2783	3125.5431
3126.1811	3127.5618	3128.3366

3128.7310	3130.2851	3130.7782
3131.0556	3131.2004	3134.4547
3139.5816	3140.5742	3141.7156
3144.8662	3146.0741	3146.6882
3150.8479	3163.0493	3163.3419
3164.5080	3164.7449	3170.7034
3173.2251	3175.4161	3177.0760
3186.8340	3188.1736	3188.4303
3189.2155	3192.4083	3192.4323
3194.5660	3196.1504	3198.7746
3201.3464	3201.5954	3202.8853
3205.5296	3206.0577	3209.8262
3216.5419	3218.1595	3218.4479
3221.6083	3222.3881	3229.3278
3230.7706	3236.4111	3278.0375

$TS5_{HTC}^{C2'}$

-104.1774	15.2829	21.4238
22.8112	25.1794	28.9099
32.6380	36.6501	39.2400
52.2349	52.7729	57.1457
60.2293	64.7517	67.4694
70.2000	72.9811	80.1213
89.0615	92.5404	94.9038
96.6344	98.5074	106.7492
109.4815	110.0471	118.9962
124.7968	129.8516	141.8231
148.1322	153.1121	164.2014
166.8565	173.4045	182.6160
185.8579	188.0296	199.6727
202.0485	207.9943	217.2222
220.4062	226.0900	236.1582
240.5943	244.2278	248.4162
259.9777	261.4859	262.3473
263.0672	266.2458	272.4268
282.5780	286.1349	287.4324
290.2050	301.9197	307.1721
308.2085	310.2827	312.8979

314.0873	318.9237	323.6231
329.4019	333.0993	339.7602
348.9182	353.4842	356.6635
360.6287	368.1986	368.4145
376.9480	381.9250	383.8252
390.0173	392.0655	392.9789
400.9246	402.3129	403.7785
407.7184	409.8832	412.1120
418.4881	424.0867	428.0123
441.3471	442.1799	443.9488
462.6436	465.3437	468.4866
481.5219	497.3968	504.5473
509.1763	525.1956	527.1420
528.1866	530.7329	535.7052
539.6427	546.7041	563.3649
564.6372	572.2192	580.7070
586.7402	588.2557	591.0309
592.0443	626.9255	629.8959
631.0796	635.1408	638.0497
652.3641	668.0957	681.6228
687.8075	707.1089	714.1035
721.2913	740.0336	746.5845
756.8545	761.1436	763.8951
771.3913	775.5218	782.3256
788.4767	791.2508	812.7059
818.1371	837.0015	840.3590
855.6799	863.5206	867.6722
870.8379	873.1449	874.5597
887.8427	888.1309	900.6297
907.1212	908.1435	912.2255
919.4364	924.8725	937.1568
942.7956	946.0515	950.0486
950.1776	950.9560	953.9348
954.9790	956.9217	958.8609
959.7767	962.3357	963.3034
964.3092	966.7302	969.3405
979.9171	986.0670	987.3784
994.6652	998.6271	1003.0107

1005.0490	1015.8816	1016.1036
1020.3274	1023.8502	1024.5635
1034.6009	1037.0452	1039.0054
1044.7208	1047.5288	1049.8258
1055.3246	1055.7533	1057.0704
1059.1841	1062.9540	1063.9888
1065.6242	1066.4117	1069.7293
1070.8382	1073.4200	1075.6850
1076.0878	1088.4010	1107.3007
1112.9781	1122.7168	1124.5853
1128.4706	1138.1453	1142.1819
1161.8114	1174.1050	1186.0380
1199.4806	1205.5714	1209.1296
1211.3850	1222.7377	1231.2509
1236.2713	1242.2784	1245.0212
1245.3421	1246.9979	1247.5273
1250.7719	1253.6253	1255.9195
1260.4142	1260.5731	1262.7765
1270.8972	1283.3421	1298.9207
1303.9669	1306.5751	1318.9900
1325.8355	1331.0060	1336.6263
1338.7849	1340.8521	1347.3396
1349.8725	1352.4782	1353.2653
1354.5516	1359.9487	1363.7414
1366.8656	1367.3728	1372.3381
1380.6328	1383.9333	1388.1593
1388.1793	1388.5751	1389.8777
1390.7556	1392.2384	1394.5581
1399.2117	1402.9987	1406.4097
1418.5204	1420.9258	1422.3107
1422.6786	1424.1546	1429.1367
1433.1989	1434.0636	1448.4272
1451.5053	1464.1589	1467.0113
1480.9166	1482.7724	1482.9120
1484.8614	1485.0020	1487.5170
1489.4093	1492.7133	1493.6731
1494.6880	1496.6097	1500.0208
1500.9173	1501.4921	1501.9895

1503.0296	1503.2234	1503.7721
1505.1233	1505.6871	1505.9647
1506.2660	1507.0630	1508.0922
1508.5939	1510.9835	1512.4286
1517.9342	1520.4094	1521.9646
1524.4499	1526.9906	1528.4764
1534.2619	1535.4866	1537.6721
1541.6701	1545.8997	1549.7070
1553.3858	1558.9765	1572.3206
1583.9001	1632.4690	1645.9492
1655.9341	1666.1618	1678.3860
1683.2487	1687.3923	1688.4847
1690.4916	1693.2668	1702.8888
1708.6797	2115.0966	3046.1698
3047.3190	3047.9899	3049.9338
3050.1711	3050.5680	3051.0646
3052.5900	3054.4075	3054.6821
3057.2957	3058.9437	3062.4525
3063.0690	3066.1911	3068.8032
3074.5568	3079.6088	3113.0040
3115.0024	3115.5469	3115.8251
3126.3937	3126.4516	3128.6987
3129.2577	3130.4448	3131.9934
3132.4645	3133.4994	3133.9771
3134.8894	3135.8855	3142.2594
3142.3269	3145.0485	3148.7268
3153.1858	3160.5907	3165.9800
3174.1147	3174.3714	3176.5727
3176.9212	3180.9247	3181.0322
3181.3475	3184.2625	3184.5852
3185.3574	3185.4766	3190.2821
3198.4593	3199.5365	3201.5784
3203.8114	3206.4442	3206.7435
3208.7978	3209.3861	3215.4763
3220.2514	3223.1850	3224.4723
3234.9594	3235.2799	3239.3388
3265.8613	3271.1154	3287.4053

TS5$_{HTC}^{C4'}$

-1131.0794	20.8264	26.5759
34.6275	39.5005	41.7960
46.7429	48.8991	53.0053
56.0648	62.0112	65.3785
67.0139	71.5934	76.6165
77.6924	84.1867	86.7520
88.3939	90.5506	93.5954
97.9770	104.5263	105.2154
111.0458	116.2854	126.7685
133.7662	136.8369	142.6626
145.1286	152.1261	156.6509
164.4982	171.9543	177.3629
189.1278	190.3264	200.0956
204.2181	208.6171	213.9563
218.2439	229.2332	234.0274
238.1121	240.0423	247.8582
250.2645	255.9086	262.9692
266.9300	267.4138	276.1834
282.7662	286.3785	287.1824
294.6402	294.9974	300.9246
308.1306	309.8763	313.9385
316.7401	322.3321	324.6177
331.4460	335.4653	338.3109
340.0112	343.3166	363.0071
364.2880	368.9732	370.7740
372.7957	377.9373	382.5927
387.1732	388.0647	389.8328
397.9298	403.5935	403.9138
412.3159	414.5400	417.2228
419.2624	424.3850	432.2669
442.1449	446.2957	456.2734
460.5877	467.1444	468.7988
472.0540	499.6364	506.5281
507.9410	515.4206	526.9899
528.0413	529.2527	532.7275
542.7147	545.9013	551.7867
558.9265	572.4153	576.7854

584.1480	590.3390	593.1292
596.1604	610.3507	624.0291
628.4095	631.9077	639.8944
644.2662	661.1795	668.5140
679.3779	693.5413	709.4754
711.9780	723.3242	744.1309
749.1592	758.7263	769.1752
772.1249	773.8023	778.1842
788.5597	797.9538	800.7633
821.6664	831.5729	835.8012
838.6532	852.1878	858.9866
872.2999	877.6339	881.4172
883.0172	889.0169	891.6866
903.5906	905.5223	912.4867
917.6315	920.9295	927.3219
928.6294	941.0569	943.7403
950.0224	951.3480	951.7390
954.7757	955.0126	957.0626
957.3689	958.4779	960.4292
963.5812	966.6319	969.7043
972.7205	972.8421	980.5510
987.9601	1005.1119	1006.2510
1007.9163	1016.8323	1018.1868
1019.0308	1022.5745	1029.2450
1036.5107	1037.9577	1044.5160
1045.5880	1047.4318	1049.2969
1052.2165	1053.8146	1056.8726
1058.0391	1058.9681	1061.1487
1062.1678	1064.6143	1066.2943
1068.7301	1071.3736	1073.7426
1079.2742	1087.8670	1095.3009
1120.2962	1122.7648	1126.1182
1129.3093	1131.8857	1155.2130
1175.7344	1179.2515	1197.4557
1203.5611	1207.7419	1208.0833
1213.1226	1218.5434	1221.3516
1233.0957	1233.9894	1239.5123
1243.1625	1244.2688	1244.4543

1247.8701	1247.9294	1248.5876
1256.1237	1260.4056	1266.5616
1272.7508	1275.6098	1289.4323
1289.7389	1298.1557	1311.1401
1315.4790	1316.1698	1324.7318
1333.0679	1341.6360	1344.0420
1347.0631	1347.2582	1348.9457
1352.1233	1360.2521	1363.3744
1363.9243	1366.8424	1377.4693
1379.0490	1383.8559	1384.7453
1387.6078	1389.0329	1392.2909
1394.1630	1394.8136	1395.1545
1396.7307	1398.2871	1403.2939
1416.7096	1419.5800	1421.5270
1424.2051	1425.4624	1426.5206
1431.0938	1433.5962	1445.8384
1456.2403	1464.3283	1475.0226
1481.2412	1483.2211	1484.3497
1484.6752	1486.4580	1486.9985
1490.3324	1492.7097	1494.2961
1496.7955	1496.8473	1497.7640
1498.9323	1500.2372	1501.3261
1502.3797	1502.9557	1503.6766
1504.1321	1504.4007	1504.8038
1505.8672	1506.9738	1507.9544
1508.5821	1509.1999	1513.9273
1515.8372	1519.1646	1519.3239
1520.8391	1521.9010	1522.2060
1524.1743	1531.1854	1535.5603
1538.1342	1538.3941	1541.2222
1549.8176	1550.4785	1554.2251
1589.4953	1613.7091	1629.7295
1644.2887	1659.9114	1671.7610
1676.9667	1680.3564	1685.2237
1687.0071	1693.2066	1694.3516
1695.9804	1698.5078	3031.6391
3037.0494	3049.0567	3052.6741
3053.5045	3053.5453	3058.3941

3058.9623	3060.2313	3061.7526
3061.8253	3062.8758	3065.7701
3066.2905	3066.8255	3067.5471
3067.9628	3091.4737	3104.5463
3124.7177	3125.2611	3128.4906
3128.5684	3128.8101	3132.7505
3132.8585	3133.7663	3134.0360
3134.9959	3135.4346	3137.5016
3137.5437	3138.3969	3140.7570
3141.5949	3143.3987	3151.2079
3162.5347	3169.3170	3169.3502
3169.4459	3171.6807	3173.7781
3177.8183	3181.1230	3182.2057
3183.7916	3186.0211	3186.4338
3187.2304	3189.3756	3192.2845
3194.2800	3195.8212	3198.4084
3202.0388	3209.2590	3209.7945
3211.1490	3213.7450	3215.1338
3215.9737	3219.1894	3220.9226
3226.5132	3229.0248	3236.1248
3245.1921	3254.9204	3268.5637

$TS5_{HTC}^{C5'}$

-1087.9005	14.2312	19.2098
24.2471	27.1464	31.2079
34.5782	36.3170	38.8979
46.3549	53.6149	55.5881
57.5794	58.7496	64.3574
67.8353	70.5640	74.8102
75.5867	84.3486	88.1198
91.9093	101.1192	104.8993
110.0280	112.6600	117.1353
125.7681	130.2624	141.3426
149.4809	151.5676	154.7545
162.9537	164.6780	172.2727
179.2598	181.5242	195.9729
197.5872	202.8798	211.8207
217.5724	222.4611	228.1194

234.8561	236.8964	237.4808
241.0968	248.7168	261.2388
268.6014	272.8218	273.3284
279.8967	281.3764	282.9454
285.4196	290.2194	296.3879
300.3620	308.6014	313.2298
321.3648	322.8624	324.3072
326.7162	331.2289	339.0581
344.6726	351.1141	358.5760
363.4049	367.4466	368.2478
369.8283	378.3157	381.4958
383.6122	388.3187	391.6001
394.8022	400.9897	404.9657
406.8195	410.9322	416.0704
418.6555	419.6813	423.3531
437.7866	441.9797	445.3692
452.3680	459.9799	467.1969
470.1811	475.9873	503.2011
504.4887	514.2943	523.6635
524.4618	526.0731	530.5550
536.1806	542.5009	545.5232
552.2249	557.6958	576.7958
580.0854	583.9018	585.1173
592.6657	598.1748	627.1045
628.1208	630.5060	636.0383
652.2773	663.1124	668.6654
682.6163	704.6017	713.4099
715.1955	720.5464	739.0586
742.9298	754.4568	766.9176
770.6338	773.1230	782.3378
794.2464	801.6843	811.1044
824.2335	835.2094	837.5402
839.6328	858.3388	866.1533
874.8953	878.5271	882.3175
891.2392	897.1708	901.2375
904.8438	915.7516	919.1775
923.8735	925.8243	931.3149
943.3429	945.6345	948.5347

950.1934	950.5806	952.4851
953.3340	955.2690	956.4730
957.1490	958.7036	960.8918
961.5004	965.2655	965.6016
967.3256	969.9157	979.5813
997.3506	1003.9988	1005.4280
1008.8660	1014.3605	1016.6931
1017.3077	1024.3890	1027.0187
1033.0845	1036.2758	1037.6284
1043.9782	1046.4890	1048.8536
1052.3405	1052.9593	1053.8242
1055.0184	1055.4140	1056.6190
1056.7161	1063.8518	1064.8261
1065.6668	1069.2194	1073.6647
1077.7586	1082.3334	1094.7644
1119.0291	1122.5790	1125.4735
1128.5657	1132.2401	1144.4508
1176.7898	1179.2297	1180.3497
1200.9208	1207.1114	1208.1465
1212.0066	1214.2057	1220.6608
1224.3809	1238.5390	1242.4819
1244.7729	1245.6691	1248.0437
1249.2898	1249.7281	1253.7167
1255.6738	1257.7915	1259.3562
1266.9912	1284.1907	1291.6517
1296.8668	1309.8526	1312.6071
1316.5805	1322.7295	1328.0563
1339.1600	1341.6837	1344.0017
1345.9243	1351.7759	1357.4382
1360.0534	1361.3299	1364.0169
1366.6672	1377.1625	1378.4644
1379.6790	1385.3177	1387.7842
1388.4731	1390.3308	1392.2581
1396.5057	1397.0602	1397.5281
1400.5313	1406.7057	1412.8022
1413.6903	1417.8265	1422.1786
1424.9903	1427.5086	1428.3111
1428.5011	1436.1496	1453.3863

1458.0371	1463.6075	1472.1300
1476.3138	1480.0691	1480.5385
1483.4907	1483.7572	1485.6081
1487.3260	1488.2967	1490.4479
1492.2025	1495.7885	1498.3795
1498.5212	1499.3434	1500.0459
1501.3143	1501.4101	1502.9983
1503.9044	1505.0182	1505.2496
1505.6791	1506.7917	1506.8713
1507.4728	1510.1082	1513.4798
1515.3502	1517.1173	1517.8188
1518.0290	1518.3773	1525.1716
1529.2460	1533.1888	1533.7332
1534.8720	1541.7189	1546.0841
1551.1880	1555.6241	1602.2738
1645.6045	1659.3463	1660.1842
1669.0622	1674.6587	1677.6697
1682.5714	1689.3726	1692.1284
1693.2756	1699.7913	1706.3470
1723.4959	1744.5118	3044.7263
3047.8266	3049.8828	3050.5434
3054.3710	3054.8767	3055.0798
3055.1760	3056.7798	3058.6655
3060.4662	3063.8921	3064.6479
3066.5033	3070.1089	3073.1201
3081.2103	3088.1147	3114.6076
3119.6449	3124.4988	3125.3843
3125.4253	3126.0623	3128.4744
3128.6218	3130.8664	3133.8205
3135.1648	3135.2945	3137.3027
3140.7343	3142.8383	3142.9749
3152.9347	3155.5144	3157.1448
3158.0190	3167.2894	3171.6209
3172.6805	3172.7311	3173.5750
3180.0531	3181.4659	3181.9760
3183.4949	3184.8932	3188.1918
3188.4036	3188.5365	3190.2877
3191.2595	3196.5808	3198.8595

3200.5791	3202.0465	3204.8658
3206.2964	3208.7242	3209.2042
3216.7312	3220.4622	3221.2461
3221.7792	3222.9232	3227.0248
3229.8659	3254.6397	3260.2321

TS0^{OAr-}

-1352.2629	11.5430	11.9771
15.3610	32.2259	41.9641
55.1353	63.0398	73.2768
78.2746	90.0191	92.9670
113.1491	123.1707	143.9396
163.4910	188.9443	209.0492
221.2258	245.2133	276.4292
293.6024	309.0534	323.6563
346.6154	367.5038	382.7115
410.6734	416.5484	421.3005
426.4610	431.0595	476.4195
489.4742	500.6478	520.4262
531.9381	544.9636	558.8862
563.6723	593.3676	628.6663
632.3189	645.0789	646.6116
665.8021	699.2916	707.1342
721.6839	723.8109	726.8349
781.8056	787.8823	805.0731
820.5091	840.4191	846.0272
847.8858	851.2985	872.0678
888.5046	892.8723	897.1993
939.9085	973.8748	988.2888
991.5332	1003.2326	1010.1569
1013.4337	1016.4165	1017.8348
1019.8205	1038.1027	1038.5694
1066.3921	1069.5365	1077.5409
1112.8353	1123.1743	1128.6762
1139.9246	1150.7064	1155.7745
1170.1479	1176.0268	1207.3804
1208.7231	1213.0268	1252.8353
1290.6987	1298.2627	1329.8595

1338.8921	1350.0939	1357.1140
1359.9513	1363.3948	1382.9348
1421.4498	1421.7066	1438.3691
1455.3265	1482.4231	1491.3165
1493.4089	1501.3737	1504.5721
1533.8303	1553.3093	1557.4008
1582.4963	1606.3265	1622.5438
1674.0435	1677.4356	1686.8814
1691.7471	1698.4890	1698.5597
3071.7873	3132.6338	3142.8076
3163.5211	3196.9375	3203.5885
3212.1669	3213.2263	3217.3201
3219.4395	3221.3801	3224.5812
3227.2296	3232.9988	3237.3260
3244.5419	3247.5968	3248.1829

TS0^{DBU}

-1276.5719	12.3307	14.1666
21.9468	29.5220	33.8719
41.0637	51.9861	60.4777
65.9277	69.0848	86.3131
95.5061	103.6896	119.3231
155.2478	167.1854	191.3137
206.8048	221.1483	237.3202
256.0687	277.8146	299.5520
309.6739	328.5202	344.9406
349.3148	354.1154	384.0681
395.7177	407.6402	416.8608
417.7436	422.4007	435.5004
477.7513	485.5282	493.4569
514.5552	524.1832	532.9679
538.7037	564.2785	610.9233
626.8778	629.7049	645.7732
651.7004	703.5561	705.0339
710.7658	720.9051	725.6299
736.1957	784.4483	802.1274
816.8318	820.1223	838.0205
845.3774	850.0960	860.2736

867.4720	879.6155	885.0980
894.6222	920.0822	928.2612
940.1823	964.5326	984.6061
992.9018	1001.5516	1004.3669
1008.3288	1013.6216	1016.9607
1022.2257	1027.3874	1036.9742
1037.9165	1068.4422	1069.5525
1078.2067	1102.4131	1114.2941
1118.1284	1125.9994	1126.8680
1135.2216	1137.8351	1146.7544
1155.8012	1171.8185	1178.6697
1186.9867	1206.6634	1211.1231
1223.3510	1242.2444	1248.4370
1253.6389	1267.8825	1276.9158
1294.0700	1297.0450	1317.3379
1326.2320	1326.8075	1340.2775
1354.4012	1358.1124	1360.1020
1365.1949	1374.0850	1380.8066
1381.5676	1385.1770	1394.2299
1407.5203	1413.1977	1420.0441
1422.2119	1453.5893	1473.2108
1479.9989	1484.6818	1489.4349
1490.5012	1492.3610	1493.5161
1494.0396	1499.1112	1502.4622
1508.1778	1514.3834	1539.0601
1553.6533	1556.4005	1594.7021
1614.0589	1673.8785	1677.7428
1697.1089	1698.5092	1731.3158
3056.0354	3062.0119	3062.1236
3064.2694	3074.1487	3074.6909
3081.5933	3083.6812	3087.6205
3113.3261	3114.6886	3117.2321
3120.4039	3133.1359	3141.1570
3143.5673	3146.2817	3148.6069
3152.5986	3164.5974	3198.8812
3199.8237	3203.0039	3206.4790
3214.4883	3220.1956	3224.0021
3234.9673	3235.4281	3235.6483

TS0^{DQH-}

-1375.7516	8.4570	12.5873
15.7683	20.8703	27.1521
32.6215	35.7730	39.4756
41.2994	46.0364	53.5250
65.5591	70.8343	72.3593
73.5074	74.6104	88.5920
98.4906	108.8247	118.9075
125.1520	133.7934	149.1499
153.5642	162.1733	170.6952
176.7090	180.3728	184.3521
192.5651	197.7519	208.1385
213.2154	217.8841	222.2949
224.6679	229.8034	231.9089
247.1213	266.3672	269.3063
274.7421	277.4222	289.0870
291.3795	295.7688	312.1075
313.0460	322.9519	323.3245
327.9210	330.2573	335.5108
348.2148	354.0028	359.4465
366.9962	367.5556	372.4374
378.5815	381.2045	384.2222
389.9124	399.8332	405.2805
408.8639	413.9330	415.2152
415.9487	416.9448	425.7627
431.1682	444.8214	453.3936
456.3778	469.4390	474.8971
478.2384	487.8775	492.1638
525.1402	533.5678	540.5485
543.3422	546.3081	556.3447
562.6423	594.3209	621.3582
629.7084	630.2346	639.0944
643.1313	646.9232	647.5208
661.2494	674.3885	684.5243
692.1964	704.2581	719.2316
724.9361	772.5251	776.9491
780.1245	787.4634	789.7096

804.8795	814.0289	822.8453
836.4384	837.7406	845.1229
852.2202	870.6902	873.5948
886.8333	910.0968	918.5000
923.9625	930.9739	936.5453
942.2707	943.8211	946.7746
947.1006	950.6795	951.9561
953.3426	955.2407	955.8486
961.9262	963.3362	963.5649
965.1742	974.9275	977.5325
979.8397	983.8655	1001.3517
1015.9177	1018.5417	1020.0313
1033.9650	1039.1753	1045.2896
1048.6406	1048.6858	1050.5806
1053.4936	1058.5016	1062.5503
1063.9017	1066.4749	1074.4288
1089.4232	1112.8506	1133.8112
1142.4411	1149.6947	1156.0366
1156.6170	1159.5563	1171.2440
1184.8294	1185.3354	1212.0994
1222.6454	1230.7787	1232.8900
1242.1128	1244.3152	1246.2236
1248.1998	1248.6429	1250.4441
1253.8807	1254.9030	1283.1794
1287.7527	1289.7669	1298.4822
1301.0729	1306.7950	1313.0128
1321.3232	1324.7238	1333.3839
1343.5702	1347.5573	1356.0286
1356.8149	1373.5653	1384.5127
1387.4536	1391.2552	1392.7109
1397.1873	1401.6963	1401.9278
1403.5452	1411.9130	1420.4002
1421.2169	1424.3559	1425.5669
1428.7888	1446.4130	1454.9790
1459.5944	1468.0664	1479.6689
1481.7894	1485.1447	1485.3414
1487.8158	1493.2179	1493.9022
1494.5660	1495.2072	1497.7121

1498.3010	1500.2219	1500.6522
1501.8829	1503.9335	1504.5805
1504.8845	1506.3193	1507.7553
1509.1245	1510.7172	1513.3334
1515.9425	1516.9987	1517.8706
1528.4097	1531.6278	1532.0727
1534.3244	1537.6298	1550.1277
1560.8517	1561.4298	1611.8965
1624.8103	1671.8654	1674.3928
1678.5142	1686.5568	1692.3869
1698.3121	1699.4604	3043.5274
3047.2217	3047.4599	3049.7359
3052.4969	3054.9505	3055.8212
3057.1647	3062.1693	3064.9206
3069.8137	3072.6783	3073.7415
3111.6241	3113.3162	3116.8597
3121.3980	3127.7178	3128.1081
3129.8684	3131.0854	3132.0821
3133.1584	3135.2424	3139.1889
3141.4422	3143.8349	3144.9873
3148.2483	3150.3928	3151.7657
3153.2403	3154.4809	3166.2691
3176.8709	3184.2727	3187.4958
3198.3833	3204.4178	3207.2880
3214.8391	3215.4481	3215.8432
3216.2396	3219.0820	3221.0684
3224.3567	3227.3234	3234.9428
3235.0466	3237.4745	3243.7519
3248.7992	3254.1126	3945.7316

TS6(R)^{HOBt}

-292.7535	10.6145	18.3518
22.2377	24.9477	25.6647
29.0462	30.5518	33.3230
35.4117	39.8098	41.3577
44.8407	49.0191	50.2372
54.6478	58.4323	61.9736
65.9009	67.9680	79.5175

84.5712	90.0355	94.5331
101.8762	110.9311	114.3347
124.0053	127.6992	131.2147
135.4913	139.3973	150.4391
161.4004	170.5670	171.5862
180.6576	188.1014	193.7171
204.4490	206.3045	211.2856
216.5746	225.7170	234.6918
241.8832	243.4073	244.4421
251.9244	257.7808	268.0107
282.7456	283.5316	288.3832
298.7797	314.2137	321.4609
331.6488	335.6703	336.0886
338.8051	352.3581	373.2845
384.2211	389.4606	407.3647
413.5504	419.1246	420.8082
422.9823	427.7775	439.9195
442.1324	451.8730	471.7995
482.0588	491.5166	503.5905
506.7002	512.4854	517.6021
523.5037	528.0060	531.3009
534.5181	543.6261	557.5865
562.8680	564.7782	579.7833
582.4177	584.7666	586.9479
588.6030	608.5649	617.9896
620.9185	628.8153	629.0440
630.4965	634.4985	645.2238
668.9000	685.1929	689.9697
704.0064	709.4344	712.2636
719.4645	721.9947	723.4941
731.5132	755.3189	768.1478
768.8546	772.0949	775.1770
776.4815	784.4612	785.4179
791.0851	796.4662	811.0102
815.2415	819.8277	826.7969
831.9663	842.3277	843.8371
857.2956	872.1215	876.5885
877.1963	878.2993	879.1131

882.0831	885.4158	890.1189
905.2579	909.8392	920.3668
936.0628	945.6865	948.0434
957.2990	965.2945	979.6623
981.1419	986.5738	1002.4227
1002.5847	1005.4546	1006.2046
1007.6936	1014.0288	1014.4936
1014.6615	1016.5010	1017.9433
1020.7416	1021.7586	1026.4460
1026.8133	1028.7525	1029.5446
1039.3763	1040.2108	1046.3882
1048.2057	1054.2905	1063.5944
1064.5939	1065.2229	1065.6579
1066.8478	1067.1527	1068.0160
1069.9136	1071.0437	1074.5108
1078.7386	1113.4985	1118.7014
1121.9158	1133.7790	1137.9154
1138.5859	1145.6068	1151.6513
1155.3226	1169.8613	1174.3170
1175.4700	1177.1275	1178.8050
1183.0921	1201.0676	1202.7038
1205.2294	1207.1840	1208.0526
1212.2242	1222.9261	1231.5118
1238.2224	1239.6418	1243.7341
1250.1444	1251.0767	1272.1797
1283.8358	1289.1587	1315.7504
1317.7694	1322.6343	1326.6213
1328.0825	1330.5415	1332.8680
1334.2585	1340.1736	1345.4251
1346.2861	1352.4735	1356.1512
1357.4952	1361.1935	1362.5263
1373.5902	1378.1139	1388.0437
1396.9801	1403.2811	1413.5219
1418.8610	1421.9617	1424.1676
1427.6562	1432.6491	1441.1824
1442.4111	1447.5949	1459.7429
1464.5292	1465.4447	1481.1836
1488.5713	1493.4912	1493.6539

1494.1524	1495.9449	1496.0012
1497.4726	1501.2271	1502.9514
1503.5541	1505.4049	1508.7384
1509.4476	1521.4712	1532.3664
1538.9865	1545.2894	1548.6869
1550.6599	1551.3637	1558.3377
1568.4351	1618.8238	1620.7051
1661.4217	1666.2021	1668.9376
1671.5280	1672.2418	1675.5901
1684.0659	1685.5069	1687.7425
1688.7088	1690.0884	1694.2637
1716.7658	1718.6081	3004.1716
3033.7439	3050.7519	3057.6967
3073.1229	3078.4175	3081.1729
3106.9224	3136.3673	3140.3419
3148.9641	3149.0870	3156.8888
3161.2501	3171.9369	3174.2415
3177.7630	3177.9364	3179.9798
3185.3751	3195.8443	3200.7040
3202.3305	3203.3778	3204.6807
3206.2697	3206.5249	3208.6797
3209.4333	3212.5970	3214.2546
3217.1189	3217.3427	3220.1426
3223.0301	3225.4067	3225.7001
3227.5393	3233.2127	3233.9521
3235.4543	3237.4219	3239.6191
3245.8727	3251.6181	3252.1457
3255.8141	3295.4277	3302.0925

TS6(S)^{HOBt}

-290.7613	19.6054	22.2591
29.3909	31.3498	31.7376
33.8193	37.7194	42.3781
45.8726	49.0817	51.9852
56.2879	59.9982	62.0821
65.0245	66.5868	69.0714
75.1247	82.7701	83.5902
87.4479	89.1931	99.3033

110.7134	111.7241	120.4424
122.0917	127.2250	134.3463
139.3501	145.8738	150.3452
157.3956	162.0953	174.0703
179.7038	184.1094	194.0891
199.5045	208.4976	210.0619
215.9018	229.6114	232.3023
244.1604	245.3974	248.6148
253.7484	269.7362	275.8047
278.5285	289.7261	296.4545
305.2509	330.7913	331.2507
336.5148	344.3909	350.3361
354.0660	359.3999	384.7640
395.9635	402.8954	408.0844
413.1478	423.3221	424.1560
429.3910	430.4776	438.3470
444.7718	449.0384	468.0210
475.5733	480.0927	505.9751
506.2351	517.1144	517.8452
524.5183	525.2512	528.3114
530.7774	545.7578	555.9791
558.9756	567.2558	579.8960
584.9565	585.9297	590.6609
604.2017	608.9102	627.7058
628.4866	629.3837	631.6524
640.3357	644.1695	660.5561
662.7895	676.5857	679.4270
688.6948	696.3430	709.9335
719.8399	722.7273	724.2630
728.0424	751.6472	758.8502
764.4561	765.7528	771.8281
772.4356	773.5581	781.5210
784.1321	790.7890	794.5601
813.6650	814.9557	819.4628
827.3624	842.3460	844.6015
852.6264	871.9700	873.3245
874.3431	879.2206	880.4012
887.2457	894.1063	905.3522

907.4031	928.2600	934.5169
938.3446	947.4263	951.7683
965.0784	970.6903	977.0565
982.0496	986.2427	1000.2472
1000.4690	1005.4726	1009.1139
1012.6872	1012.7411	1013.7047
1015.5005	1016.6082	1017.0610
1017.5568	1018.3826	1026.8224
1026.9308	1030.3482	1032.0550
1036.9938	1041.7737	1045.9263
1050.2901	1052.9747	1054.1352
1055.5970	1061.4939	1065.8006
1068.3681	1068.6463	1071.0524
1071.3793	1073.4974	1073.6729
1093.8310	1118.6274	1119.1858
1123.5897	1125.9640	1133.7323
1140.7745	1145.1307	1148.9870
1152.2245	1173.3550	1175.5775
1175.8426	1177.1446	1180.1520
1188.3255	1200.7616	1205.0016
1207.4276	1207.7887	1209.5384
1211.1026	1221.4736	1226.3035
1232.4610	1242.0663	1246.4502
1253.5377	1258.9926	1266.5566
1282.2378	1286.4987	1299.6783
1312.4140	1325.3563	1328.3759
1329.9103	1332.4227	1337.1387
1342.5710	1344.3669	1349.5321
1357.6030	1359.0984	1359.7420
1360.1129	1363.0400	1363.8579
1374.6388	1375.7548	1389.0769
1391.1149	1399.5895	1416.4951
1419.3270	1420.9955	1428.8540
1430.3970	1436.5623	1439.2789
1451.8496	1453.4214	1467.0763
1472.6019	1478.6804	1484.8503
1488.4152	1491.9409	1493.4595
1494.7222	1495.5373	1498.1623

1498.5628	1500.9552	1504.2545
1505.3091	1510.0384	1515.9791
1518.6527	1522.6701	1536.1742
1541.1572	1547.2831	1549.6536
1552.6832	1553.2629	1560.7990
1580.9904	1622.3095	1634.9651
1668.0508	1668.7665	1672.9460
1675.6715	1675.9041	1682.5189
1686.8980	1693.3408	1693.6852
1695.1062	1697.3930	1698.7996
1712.6433	1730.3074	2855.9914
3066.3765	3066.7153	3070.1140
3070.9529	3073.8490	3085.2814
3087.3735	3136.3918	3141.6996
3143.5710	3152.0141	3154.4860
3162.7202	3164.2254	3164.9740
3165.2544	3167.6447	3179.6411
3180.0200	3182.8011	3186.3459
3187.8839	3193.9235	3196.1189
3196.3852	3197.8928	3201.2076
3201.5023	3205.1188	3205.3745
3206.0339	3208.4324	3209.4914
3210.3251	3213.7617	3215.3222
3217.4652	3221.5607	3221.6975
3221.9595	3222.5708	3227.6369
3234.8445	3235.2544	3237.7710
3249.2226	3257.2059	3315.7398

TS7(S)^{HOBt}

-1120.1903	10.6930	13.7036
16.3578	20.1040	26.8576
32.4440	35.5038	38.9805
42.4011	46.3944	48.3166
50.1623	57.0630	61.9273
68.6167	68.8452	74.8927
77.2510	82.8847	85.1337
92.6471	93.2353	93.7155
97.5718	104.5892	106.2810

108.9444	115.8842	116.8649
135.0581	138.8161	153.2824
159.6264	173.2471	178.0231
180.8548	188.5342	198.7627
203.5293	209.4096	212.3463
237.0101	242.2649	243.9345
247.8756	250.8191	256.2380
264.1844	270.3593	279.2032
284.7146	299.1123	303.9830
313.6219	316.8305	327.1877
348.5515	358.3532	364.6757
372.9733	382.7260	391.5506
396.5239	407.0614	410.0776
416.8948	417.2433	419.7549
424.0969	428.4861	443.2985
451.4801	465.1691	487.0445
491.0396	494.1031	504.1380
511.7778	515.9828	522.3276
532.8906	534.2410	542.8522
549.6221	556.4506	560.8274
569.7396	580.8170	582.5183
585.8355	588.4869	593.0672
605.7859	611.4656	625.0824
629.4522	631.0602	634.6453
639.3027	644.1464	645.6580
666.1994	672.6930	682.1710
710.4741	716.5666	718.7863
722.8822	727.7689	728.6718
740.7068	747.3352	754.1708
755.1508	768.7881	769.9440
779.6071	782.9962	793.6345
796.1477	802.3412	814.4470
824.2743	828.2998	835.0509
856.2461	862.2675	868.1313
877.0884	877.6870	882.7614
884.1094	887.2491	894.1042
900.6325	918.3811	920.9753
937.0482	945.7815	954.4483

961.3936	963.2493	969.4450
974.0549	978.9509	980.6612
994.3809	996.3805	1004.4033
1005.0755	1011.3512	1013.3897
1013.6857	1015.1253	1015.8051
1016.6478	1018.0215	1018.2169
1022.0108	1029.3746	1034.0483
1034.2066	1038.2862	1040.4080
1041.4446	1050.6561	1059.2160
1061.9738	1064.1214	1065.4928
1067.4196	1067.9887	1069.3162
1072.2007	1073.1552	1078.3283
1092.7672	1111.4819	1115.1822
1116.7874	1120.1439	1126.4413
1131.0087	1137.0730	1146.2263
1160.8934	1163.2223	1168.3404
1172.8133	1173.4897	1179.2373
1180.3429	1191.9981	1201.1220
1203.2472	1204.3258	1205.3821
1207.0271	1218.8611	1220.1193
1221.2294	1232.5446	1245.3881
1247.3286	1250.8173	1251.6871
1258.5128	1269.0145	1284.8643
1293.6939	1294.4199	1301.8964
1316.1922	1324.1871	1327.8576
1332.1533	1336.0913	1336.9314
1340.6764	1344.2239	1346.5700
1350.4843	1353.7723	1359.2795
1359.9598	1360.3079	1361.2916
1362.5904	1363.3068	1370.8488
1383.6427	1388.7963	1414.8476
1418.5749	1421.8287	1423.4132
1432.5207	1437.4685	1444.2778
1445.2978	1451.4271	1455.1456
1467.6840	1478.0563	1481.6569
1489.2154	1490.9229	1492.3806
1493.7740	1493.9494	1494.2672
1497.5338	1500.8310	1501.4262

1504.4832	1505.6689	1506.9239
1515.5449	1516.2125	1518.3017
1526.2241	1543.3065	1547.9379
1548.5989	1550.3680	1552.4680
1556.2180	1571.3477	1581.2219
1600.4343	1661.6473	1668.0715
1668.7775	1673.2917	1674.1776
1677.9716	1692.7599	1694.4947
1695.5739	1696.3606	1696.8477
1697.2582	1712.7441	1852.8514
3054.0691	3058.2799	3069.5290
3072.9035	3073.1543	3074.8620
3076.5005	3094.1003	3120.8586
3130.1095	3132.0314	3138.3874
3142.2799	3143.0857	3148.6095
3160.5838	3165.0860	3169.1192
3169.8455	3172.1626	3178.1541
3181.1717	3181.5552	3183.8826
3188.4131	3190.5451	3195.7330
3196.8253	3203.2920	3206.6979
3207.9662	3214.6616	3215.6327
3216.8195	3217.0424	3217.1695
3219.2856	3221.2328	3222.1588
3224.0773	3225.8699	3229.4142
3230.3001	3235.8317	3238.6165
3240.0563	3245.1729	3259.5251

TS7(R)^{HOBt}

-1127.1157	15.0549	19.5562
24.4276	28.5765	30.1400
31.6883	35.8129	39.9548
40.0630	45.3650	48.8406
54.8839	57.7913	66.8211
68.2094	72.0473	75.1296
77.1894	80.7720	83.6237
84.3275	89.8768	96.6823
102.6787	109.5949	116.3945
118.4152	125.6546	127.8674

132.5214	140.5156	144.3036
150.8819	154.5302	158.0434
169.4931	172.0634	183.3670
194.7264	205.5117	211.3011
217.7525	231.7936	237.4859
241.7605	247.4356	249.3100
255.3388	263.8833	268.6766
272.0784	286.3619	289.9338
302.2656	318.7659	328.7204
347.5873	350.1794	369.6954
372.8675	381.2081	387.6472
396.8822	411.0563	413.3537
415.3224	419.2898	420.8674
430.1461	438.4757	445.2711
448.1267	453.3104	473.0488
486.4174	488.4805	494.7961
505.8423	517.5029	522.2013
524.7606	528.2487	540.6697
543.9469	556.2478	563.7521
567.6315	581.2799	584.5790
589.5271	590.2746	593.5932
598.4231	614.3917	620.2080
627.1574	629.5908	631.0365
631.8980	643.7948	651.1016
664.2482	665.7418	680.6972
712.5571	715.8082	721.9499
722.9284	724.8158	726.7143
739.8302	745.5284	752.4666
762.5585	770.1352	771.8504
786.0554	788.4126	791.9883
794.8992	805.7558	811.4142
814.9913	830.4266	832.8568
840.1104	864.3355	866.3671
874.1209	882.2840	884.3986
885.3716	889.7945	898.7523
903.5499	926.0897	938.0574
946.3565	946.7730	961.7071
964.0813	965.0750	968.5834

976.8389	978.5346	981.2143
994.7035	995.4753	1004.6150
1005.8780	1009.4562	1011.3328
1013.7853	1014.9482	1015.5124
1017.0148	1018.0154	1020.3750
1020.6143	1024.2343	1028.6390
1036.4447	1042.1698	1042.9541
1045.6439	1054.2991	1055.5114
1061.2270	1063.3725	1065.3864
1067.3278	1067.7595	1070.3471
1072.5206	1074.8139	1078.9357
1099.0565	1109.5346	1112.4708
1117.0144	1118.5869	1124.7772
1130.4525	1141.1089	1144.5613
1153.2393	1164.1182	1170.3141
1178.4095	1179.8471	1180.1085
1181.3518	1190.4603	1199.0856
1199.8863	1206.4383	1209.7947
1210.7036	1212.6934	1218.0315
1219.1388	1240.5835	1244.4596
1245.9240	1249.5839	1252.1972
1258.6704	1270.3184	1283.4945
1291.0157	1299.9785	1304.9496
1318.6763	1325.2481	1330.8674
1331.4811	1333.0215	1337.1017
1337.8120	1338.4095	1342.3963
1345.0130	1357.5943	1357.7802
1361.2922	1361.5324	1363.0280
1369.4643	1373.7733	1383.5238
1383.8504	1387.6730	1409.8288
1418.7841	1420.5742	1424.5089
1428.9714	1434.0034	1437.6339
1441.3677	1453.6876	1455.8953
1463.1480	1474.9959	1482.3463
1485.2555	1493.1455	1493.2729
1494.6515	1495.0510	1497.2933
1498.6217	1502.5740	1503.5526
1503.5727	1505.5749	1508.3081

1511.7816	1518.0481	1518.3908
1540.2662	1544.3446	1545.2115
1548.4411	1554.1084	1554.5320
1565.5714	1568.1066	1603.0460
1629.5222	1658.6394	1670.3775
1671.3452	1671.4978	1675.2219
1679.0765	1687.2027	1690.6263
1693.8197	1695.2641	1696.8323
1697.8995	1706.4563	1863.5497
3049.0475	3056.0993	3065.9314
3069.5633	3069.9697	3070.2080
3072.2601	3092.1198	3118.3481
3125.6495	3138.0688	3139.1040
3141.8210	3142.9973	3143.7450
3143.7647	3154.5788	3162.4605
3169.3416	3170.7270	3176.1927
3178.3118	3179.5959	3187.1954
3189.0645	3189.1808	3195.3152
3196.4333	3199.7482	3201.8582
3204.3070	3207.5398	3209.7956
3210.1418	3211.3504	3212.4319
3213.4534	3218.0662	3219.6947
3223.0115	3224.5161	3226.4683
3228.8322	3231.1158	3233.3890
3234.4265	3238.7547	3240.5863

TS6(R)

-310.0700	10.5668	13.7276
19.4438	23.6233	27.8595
29.1743	31.8841	34.0712
37.9506	41.5008	45.8172
47.3677	51.3471	52.2359
59.9555	70.2897	71.7404
73.7761	95.9191	101.3879
104.9043	109.8121	112.3984
124.6435	125.9544	141.4411
145.4902	149.8260	155.5142
164.5286	182.5864	191.5260

200.0953	210.7730	213.6857
231.7035	235.1321	241.4455
261.4403	267.8944	270.4246
276.6718	285.2291	305.8775
308.7808	316.4142	325.4822
330.7963	345.2022	365.5987
369.9786	381.7264	385.7750
400.1667	414.5894	415.8074
417.5638	418.7456	419.9563
421.9269	426.3683	438.0951
464.5316	487.4132	493.2236
497.7574	509.2013	524.5334
525.6133	527.5868	538.3816
542.1009	552.0223	557.4634
566.2688	581.3053	587.7964
590.3531	593.0539	625.9154
628.4630	629.0130	632.1130
639.4317	641.1749	646.0968
677.9396	684.4184	702.3091
714.1244	716.5427	722.5455
723.9540	729.4192	749.3067
756.2075	764.6824	771.6484
784.1159	786.9610	795.3976
797.5052	817.9150	827.8337
840.4268	842.6680	845.4094
858.0854	869.1042	876.5291
876.7858	878.5657	889.3511
894.3715	903.9215	913.2822
927.6589	940.7832	953.3229
959.2893	965.1156	965.5275
982.6118	983.6553	990.1231
1002.7398	1004.1445	1004.7710
1008.7211	1012.9182	1015.2502
1016.6108	1017.1688	1018.6661
1023.5614	1026.3918	1036.8947
1037.1342	1037.2545	1042.7783
1045.7684	1049.0852	1054.7519
1056.0751	1062.5896	1066.3157

1067.2022	1068.8297	1068.9579
1070.2678	1072.5498	1080.2199
1092.4299	1104.6037	1112.5938
1117.1018	1120.7132	1128.6543
1141.0887	1145.0678	1161.7315
1176.8605	1177.0423	1180.7002
1182.0592	1185.3870	1199.8251
1203.1654	1205.3490	1209.4233
1209.7666	1212.3963	1223.3349
1230.9866	1248.2736	1253.5488
1256.5434	1266.1815	1280.7386
1283.1157	1287.5747	1321.7901
1328.1382	1331.4546	1332.1614
1336.7749	1339.9413	1342.5254
1344.8421	1352.8832	1359.5930
1362.3537	1363.4194	1364.7998
1371.8681	1378.6756	1383.7440
1396.4289	1400.4423	1416.7661
1420.7777	1422.5867	1432.4116
1436.2940	1440.0996	1454.9877
1463.6667	1466.6023	1480.4192
1484.7395	1489.9709	1492.0843
1493.3025	1493.6794	1494.0750
1494.3395	1496.2267	1499.6625
1502.8181	1503.0298	1509.5193
1520.2452	1522.7035	1539.8895
1544.4376	1548.9293	1554.4569
1555.7846	1560.5196	1588.4601
1618.4969	1667.8241	1668.5250
1673.1074	1676.5972	1677.2063
1685.5883	1691.8460	1694.8523
1696.2718	1697.6664	1699.4219
1706.2051	3048.8724	3063.1566
3066.3238	3070.4588	3071.8237
3078.0501	3087.8265	3131.7815
3142.1096	3142.1226	3151.0856
3160.2801	3163.3190	3165.0777
3166.7429	3169.7638	3171.7799

3177.8177	3179.9798	3190.8194
3192.1357	3192.3215	3197.8768
3201.2242	3202.7942	3203.0584
3203.4498	3206.1684	3207.5092
3209.3485	3213.0986	3213.7980
3215.7474	3216.0266	3218.2041
3220.2466	3225.5783	3229.4500
3229.5701	3233.3827	3240.5317
3243.3722	3250.5966	3309.6770

TS6(S)

-278.9359	14.4038	19.6909
22.9588	26.1497	29.3651
31.4205	35.8678	37.6111
43.6337	43.8921	45.0612
48.7732	49.8188	56.2988
68.2545	72.5808	75.4769
85.8269	93.4666	100.5676
107.3712	112.3298	122.5007
127.6531	130.3364	138.8519
142.5464	154.6308	166.0878
172.6176	178.4929	190.1811
200.8946	207.2146	208.4394
229.5662	238.5312	241.4997
247.4036	256.9896	259.7931
279.4934	279.8268	288.4538
300.1623	310.7593	323.0605
327.1735	342.0564	355.9465
370.3040	377.9004	383.5100
398.8866	402.1148	415.3235
415.6677	419.8435	420.7282
428.4763	435.8871	442.2142
464.0495	482.7706	483.8276
494.1364	502.7798	520.1559
522.9120	525.7710	530.8513
532.3954	552.8393	556.1980
563.0798	575.7360	580.2192
585.1634	588.0556	620.6823

628.9976	629.1138	630.8736
639.6818	645.5523	647.3560
674.3939	681.8526	690.5085
711.2658	712.7762	720.0272
721.7134	723.4015	737.5095
752.3825	761.5604	770.8942
780.9217	786.8300	790.4547
793.5699	811.4179	817.6945
827.1044	835.7418	839.5535
860.5443	861.9670	871.9431
876.4324	879.3576	884.3977
885.8867	887.4905	904.9317
916.6802	919.9102	947.8336
949.2697	954.9099	964.5231
980.9560	986.9887	1003.5241
1004.0186	1006.7399	1007.5385
1007.6123	1012.5912	1014.2750
1015.3260	1015.5969	1017.6593
1021.6554	1024.6866	1026.6269
1033.6522	1037.3142	1038.3564
1041.3511	1048.2293	1053.2760
1055.1517	1062.3359	1065.7864
1066.2467	1067.7326	1068.8553
1069.8832	1070.6697	1074.8132
1081.6562	1108.8936	1113.9331
1119.1474	1121.9228	1125.1634
1136.4894	1146.0608	1158.0391
1175.5363	1176.1834	1176.5988
1181.2806	1188.6756	1200.0565
1204.6108	1205.6376	1207.1108
1207.9110	1209.5763	1216.6004
1237.5031	1246.3042	1252.0204
1252.3075	1267.7720	1274.8534
1282.6706	1298.5806	1318.1407
1327.5130	1331.1068	1334.0434
1337.3763	1338.6279	1342.5601
1353.2271	1354.9092	1360.3500
1361.4654	1363.0609	1364.9587

1370.9374	1384.6584	1388.9516
1400.7979	1404.4388	1412.8581
1419.8285	1421.6274	1427.7627
1433.9477	1441.1858	1452.7045
1453.9516	1465.3741	1478.3142
1481.5830	1492.0329	1492.4899
1493.9759	1494.2534	1495.0872
1497.5026	1500.6145	1501.1414
1504.1013	1506.7801	1510.6555
1517.2795	1521.2466	1537.7429
1542.6911	1550.9002	1552.6868
1554.2644	1566.9728	1575.9908
1637.3544	1670.1655	1670.3553
1674.4228	1675.4881	1677.4301
1686.5935	1694.2568	1694.3912
1694.7399	1698.1748	1701.0053
1718.4293	3056.3558	3066.4436
3069.4032	3070.0640	3071.4678
3072.4182	3088.1675	3139.2990
3139.3885	3142.7989	3150.5510
3156.2032	3164.1568	3170.7640
3172.0214	3173.6620	3179.8727
3184.3746	3184.4687	3188.7079
3189.2089	3191.1425	3195.2459
3195.7479	3198.5677	3199.0512
3199.8843	3203.7906	3206.1526
3206.2552	3208.2150	3209.5025
3213.9605	3216.6141	3218.6542
3222.0378	3222.8382	3223.4001
3230.7378	3232.0699	3235.1020
3250.9903	3275.6248	3296.6682

TS7(S)^{HOAr}

-1395.3160	13.8680	20.9353
23.6723	26.2137	28.0060
30.0326	38.1699	42.0192
45.3826	49.7334	51.5270
54.8719	58.6321	63.4712

64.6262	73.1754	75.3676
78.2572	81.0257	83.7140
92.2483	95.3032	100.4260
106.9545	111.4522	116.5139
119.6109	125.6722	128.4221
131.8168	133.5493	141.6337
147.9251	155.2885	159.6288
160.4306	187.8869	195.9158
201.5682	205.5370	212.1883
223.7900	229.1576	235.0059
237.4972	244.7731	249.2116
257.3763	262.3018	269.4176
270.9057	279.5745	298.2049
305.3775	308.7227	314.5369
332.4389	346.0656	361.2710
367.0859	372.6340	379.5126
386.1286	395.8566	404.6347
407.9595	416.9640	417.7330
421.8848	424.9947	429.7558
432.8585	440.2444	445.3637
468.2589	483.3660	488.7225
495.3741	507.3280	511.6838
515.6396	519.4934	529.3328
531.9845	543.7315	552.3277
555.7873	557.7585	566.2031
574.5471	578.2591	583.3271
588.9248	594.6297	603.9827
627.5973	630.6913	631.0208
636.5265	642.2267	643.5773
651.1539	652.0244	667.7161
702.8580	710.9028	713.7548
715.2157	722.2530	723.0224
726.5481	729.1315	732.0690
738.3620	742.5147	759.7136
783.2627	785.8999	787.9449
790.8066	795.3669	816.8998
830.9516	833.3369	834.6135
845.0885	848.6447	857.9740

869.0261	874.2897	877.7663
880.0147	883.9457	886.3394
891.0148	899.2637	912.8097
928.2534	953.9918	954.9173
966.2045	969.4227	971.0034
974.5386	977.9691	990.4279
991.6834	994.1202	1004.3056
1004.7306	1005.5261	1007.4072
1008.4840	1011.9209	1012.9773
1014.6524	1016.2785	1017.8141
1017.9126	1018.8259	1026.8210
1030.2924	1030.4432	1036.9216
1039.7831	1041.9576	1045.7628
1050.0732	1063.7644	1065.1715
1066.0310	1069.5409	1070.2077
1071.2634	1073.0721	1073.2964
1075.2152	1099.6504	1104.5127
1117.1019	1119.2909	1124.2995
1127.3882	1129.5549	1131.9844
1137.1189	1148.8258	1166.7018
1169.6380	1176.0201	1177.9071
1180.2762	1184.5461	1186.6013
1202.1158	1205.6216	1206.0958
1210.4570	1214.1582	1216.1617
1217.4924	1221.1163	1222.4079
1245.1842	1251.3637	1251.6017
1266.5990	1269.4571	1273.4074
1290.1938	1293.0983	1300.2266
1324.1200	1326.8784	1329.5348
1333.4718	1340.6167	1342.7180
1346.1104	1351.6634	1352.0123
1360.2600	1360.5004	1362.0504
1362.7215	1364.9456	1369.7419
1371.8174	1380.1298	1384.0262
1386.7340	1396.9408	1400.4852
1420.7293	1421.4734	1423.0858
1431.6066	1432.4378	1439.9647
1448.1400	1449.5621	1453.0560

1462.9078	1467.8063	1474.1334
1484.6220	1488.9322	1491.4776
1491.9111	1492.5822	1494.9873
1496.6722	1498.2684	1500.7423
1503.1401	1504.7485	1507.8946
1511.0295	1521.4016	1532.3138
1532.9032	1540.1821	1551.7999
1553.0292	1554.1268	1557.9687
1570.3926	1580.9504	1598.1458
1628.8844	1661.7493	1665.7800
1671.7386	1673.2683	1680.1835
1680.5991	1689.9317	1691.9976
1693.7624	1694.4338	1697.0473
1697.9516	1700.7398	1843.6626
3052.6640	3055.5319	3056.6246
3058.5585	3060.2590	3068.5265
3071.1614	3073.2872	3094.2708
3129.4330	3132.0231	3132.9642
3134.8592	3138.6458	3142.0846
3155.0012	3161.6375	3169.3034
3174.5465	3174.9891	3175.7624
3178.3305	3180.9072	3186.5116
3192.6497	3197.0407	3197.7560
3198.3699	3200.2547	3202.2959
3206.7752	3207.2026	3211.5443
3212.4238	3212.8420	3215.2055
3219.6567	3220.9327	3224.1072
3224.1366	3224.5889	3226.2300
3231.8501	3235.5281	3241.6479
3243.6987	3253.9528	3276.1245

TS7(R)^{HOAr}

-1323.0675	11.6759	16.7504
20.4267	24.4522	30.1830
30.4868	32.5854	35.2266
36.1623	42.7904	44.3663
48.2146	53.4670	55.4317
57.7736	64.3770	66.7566

69.7598	71.8596	76.0535
82.2298	84.1077	88.5512
93.7004	100.3027	108.1531
114.0086	119.5442	121.8960
128.0769	133.6801	137.9702
149.4895	158.3201	165.0236
172.0985	175.0774	186.8678
193.0792	198.4788	211.6485
214.9020	221.5844	225.5553
236.7556	241.9971	247.6280
254.9970	259.2144	273.5132
277.5479	285.9270	292.9429
315.5930	318.7096	324.0764
341.6128	348.8807	354.2630
367.0937	376.6955	383.8890
399.4770	400.3177	406.7286
415.3125	416.0531	418.6866
419.8477	424.6420	436.0403
443.3771	445.0121	447.8141
455.1236	476.8606	485.8331
496.5490	503.9348	506.8517
518.1536	523.6273	530.2812
537.5188	545.9704	550.1250
556.6560	557.5572	560.2880
570.6909	580.8869	582.6819
589.2332	603.1585	619.2935
623.8543	628.6439	630.1627
630.3179	643.2973	644.5822
648.3366	656.8127	671.0166
687.3847	696.1042	708.9788
716.9360	720.3541	722.4792
723.8511	728.0179	737.0621
744.7998	758.2334	772.1394
776.1803	782.6849	786.1860
787.4019	800.6824	811.3976
830.1280	835.7492	841.9618
844.6039	850.1213	854.9367
871.7888	872.6015	877.0059

880.4473	884.9087	886.0028
894.5093	895.4165	901.4780
907.5320	931.0732	940.3723
954.9781	965.0525	973.8550
978.9960	989.7219	994.6455
995.7303	995.9113	1003.2929
1009.1135	1009.9427	1013.2294
1013.4097	1014.6055	1014.9102
1016.1371	1016.7257	1016.8955
1018.9237	1022.4301	1030.5020
1035.1860	1036.7809	1037.7780
1043.1490	1044.3324	1049.6687
1051.9787	1054.6558	1060.9842
1061.5621	1063.5182	1064.7718
1065.4083	1072.8757	1073.0403
1076.8040	1078.9776	1104.7539
1113.4464	1120.3821	1120.6997
1124.8108	1126.2901	1129.8097
1141.9497	1142.8765	1143.9102
1173.3034	1177.3347	1178.8568
1180.0791	1181.4064	1198.5227
1207.6310	1209.5155	1210.0652
1210.7588	1212.0666	1215.2300
1220.5831	1221.3655	1242.5049
1243.9685	1253.3214	1254.0720
1274.7722	1282.9653	1290.5485
1295.8164	1314.6834	1316.8051
1326.0021	1330.9340	1331.5897
1332.0738	1333.4387	1339.3184
1343.0546	1344.9012	1349.9559
1354.7786	1358.7008	1362.5306
1364.6357	1365.6444	1366.3280
1368.6575	1374.0184	1390.6977
1397.2230	1399.3903	1417.0228
1419.0755	1419.8358	1425.7597
1430.5170	1431.8200	1443.7201
1449.9354	1452.5006	1456.4424
1464.5110	1473.8708	1484.9168

1489.0438	1491.1212	1493.0190
1493.8590	1495.8400	1500.9719
1501.6670	1502.0541	1506.5056
1507.2273	1508.5504	1508.9342
1511.0322	1516.8806	1526.4469
1536.6869	1550.4072	1552.0518
1552.3115	1556.0605	1560.6611
1585.1812	1589.0245	1623.0220
1658.6781	1666.4025	1670.2134
1676.0416	1678.4177	1684.6252
1685.7363	1689.2857	1693.2422
1693.8442	1694.8312	1696.6456
1699.1284	1701.9546	1767.0933
3046.9007	3061.4856	3070.5523
3071.5284	3073.0654	3089.4136
3115.0335	3117.6527	3124.9515
3143.1986	3143.2268	3146.2189
3148.4744	3150.1997	3163.6375
3167.5820	3170.5878	3171.6711
3173.6407	3181.5040	3182.9624
3183.1457	3184.9096	3185.1685
3186.6860	3189.1113	3193.4334
3198.5535	3201.5955	3203.3014
3209.3169	3209.9583	3211.4811
3211.9590	3214.2390	3217.0149
3217.7525	3222.0147	3223.4935
3224.0320	3226.7710	3230.1424
3230.1907	3233.8306	3235.9465
3237.9169	3239.9883	3243.1262

TS7(S)$^{DH+}$

-1275.0766	10.3886	15.6197
19.4967	22.9283	24.6253
26.7397	30.4847	37.4475
43.4542	44.8146	47.8303
51.1986	55.2455	60.2902
61.4997	63.0882	66.2337
76.6755	77.4273	79.1472

88.7722	89.2144	93.5194
98.5270	99.0273	101.0026
106.2212	107.1956	112.3207
121.8504	125.2415	132.6009
136.9272	146.5677	161.2903
165.2976	167.0456	171.2861
177.1791	191.7222	197.1332
203.7376	212.7208	217.2519
227.7655	234.5713	237.1683
247.5403	251.8103	255.2035
256.7806	259.2199	267.8645
272.9401	288.1053	299.0541
306.2461	310.9537	329.0217
341.7865	347.1154	348.7784
360.3870	361.8944	369.8602
373.2750	395.9968	403.3307
408.9694	415.0668	415.7842
418.2364	421.5870	422.0684
426.1266	428.6684	436.2757
441.3112	463.3745	475.8778
485.7978	493.7652	501.3356
505.2943	511.6253	517.4476
522.6519	525.3236	533.3867
540.1333	548.8191	559.4724
568.2046	570.7826	586.1944
589.8904	596.4955	606.2846
614.0030	627.1743	628.0209
629.8364	641.0296	643.2850
646.6922	654.2375	656.7269
665.0078	708.9417	714.9089
716.6218	719.7299	721.8030
723.6222	730.0084	733.8750
741.8451	751.9695	759.6067
762.3146	771.7473	786.6758
790.0960	802.3961	809.8155
818.9823	828.3130	833.6529
843.9571	847.9215	858.0255
859.0798	865.8492	873.7683

878.8969	880.0634	881.2310
883.5931	893.4994	896.8231
909.6230	914.7865	919.8570
925.1968	939.7232	944.8557
946.5004	954.4775	963.4693
967.3380	968.4804	976.0964
979.9830	993.7824	994.9979
998.9244	1003.8694	1005.5351
1006.4082	1010.3714	1016.5291
1016.8220	1017.5568	1018.8225
1020.2207	1022.8450	1025.1988
1027.5694	1031.4033	1033.9529
1038.3476	1041.0276	1041.2520
1042.3261	1057.8986	1058.5404
1059.2508	1062.0877	1062.4333
1063.7137	1069.0347	1069.5019
1070.7055	1077.7574	1098.3961
1100.5586	1107.4641	1111.2712
1114.0043	1119.3058	1121.0837
1124.7030	1126.5264	1129.4254
1137.1533	1138.0754	1146.2456
1163.3515	1168.3082	1171.4868
1174.9112	1179.4491	1185.0489
1187.9900	1197.5485	1198.4779
1202.0197	1202.4480	1205.2424
1210.3235	1211.5250	1212.9844
1228.6314	1232.1035	1237.2137
1244.9179	1246.2184	1251.3551
1251.6425	1254.1328	1266.7951
1269.0784	1276.6793	1291.0156
1292.7471	1297.6949	1309.4233
1312.9419	1318.0377	1324.2583
1324.7947	1327.7798	1330.5186
1332.7531	1338.2103	1339.7367
1341.5567	1348.2476	1352.9180
1357.1869	1358.6663	1359.6880
1364.2360	1365.0639	1365.3586
1369.9049	1375.3202	1377.7630

1380.3264	1388.2509	1390.8156
1398.0681	1400.5424	1408.0772
1412.1962	1419.2683	1420.1804
1422.2913	1422.9325	1423.5800
1433.3562	1445.6070	1451.5646
1464.6371	1466.0083	1471.6002
1480.6145	1480.6362	1484.7822
1487.0816	1487.7216	1491.0436
1493.2144	1494.3976	1494.6689
1495.0181	1495.3179	1498.6460
1499.7948	1500.1393	1501.8268
1503.1807	1506.5440	1506.8597
1509.3854	1510.7309	1518.9370
1522.4093	1536.9464	1540.6442
1543.1387	1546.3203	1550.0826
1552.6386	1552.9263	1587.3365
1600.8094	1632.1544	1657.5278
1668.5267	1670.2873	1672.2678
1679.0893	1688.8133	1691.7363
1692.8082	1693.2440	1696.7217
1700.1789	1721.2708	1850.7586
3033.5042	3041.0007	3051.1608
3067.7192	3069.4919	3070.8235
3071.7202	3071.8754	3072.3586
3074.7149	3076.0527	3083.2889
3085.0788	3090.9447	3098.1319
3098.6123	3114.8225	3117.4016
3120.3561	3121.8647	3127.8664
3135.3657	3138.3529	3139.7840
3141.3934	3141.8209	3142.2026
3144.5963	3145.5035	3151.6961
3154.9238	3156.2315	3157.9469
3164.3919	3167.1303	3174.7844
3176.5947	3178.3129	3183.3214
3183.6949	3184.5887	3187.7091
3191.5203	3196.6455	3198.1932
3198.2290	3206.4040	3208.3080
3208.3601	3209.2088	3209.2788

3214.9702	3219.3079	3219.5053
3220.2703	3226.8995	3227.7618
3235.0610	3243.9831	3252.9419

TS7(R)$^{DH+}$

-1276.3462	16.2688	19.0816
24.2285	26.7768	29.4773
33.9805	35.9240	40.5580
47.0981	51.7989	54.3498
56.8469	58.9927	61.6940
65.2917	65.8444	69.3831
70.9228	79.5460	83.6534
86.4787	88.1625	92.1409
93.6452	96.3149	104.3339
120.3633	121.5203	126.3558
132.4196	134.8384	137.4399
144.9669	151.6184	161.2002
171.1157	174.7265	180.9265
189.1159	190.8340	198.1417
209.6870	213.3504	217.7050
223.2058	236.5240	236.8474
245.5973	247.9038	252.6361
260.3182	263.6019	266.9420
268.8399	289.4466	305.6025
309.9996	319.1422	328.2310
347.1082	348.1408	354.5694
357.1657	364.4369	370.6738
389.9473	396.2457	410.3876
410.8862	417.2306	417.5957
419.3632	422.3913	423.3268
435.1832	444.2094	445.2783
459.5031	461.9464	473.3673
483.5441	488.8040	492.8715
499.0480	509.0997	520.8275
524.2940	527.2722	532.3343
542.7852	546.5684	553.4460
565.0522	574.1200	579.8361
585.4386	590.0116	604.9294

613.7645	625.8467	628.8342
629.5917	631.7254	642.0138
646.2156	651.8828	652.6134
666.9596	707.9464	710.8457
714.9545	721.4139	722.2870
724.4656	730.4702	732.8698
735.5767	739.7303	759.0945
764.3384	773.9315	786.4279
787.7867	803.9164	810.6480
818.8158	828.7338	834.7738
837.7929	847.7590	858.8042
862.0620	866.2357	875.5199
880.4037	882.9608	885.1760
892.6020	894.8444	903.3082
910.1706	919.9456	923.4575
938.8283	939.2250	945.1032
957.5339	960.1179	964.9201
971.9567	974.9831	980.4479
995.1505	996.5801	999.0282
1000.7445	1002.7387	1006.2036
1011.5897	1012.8648	1013.6109
1015.3349	1016.9633	1018.9540
1019.9940	1023.2113	1024.5389
1025.6032	1032.1732	1035.2728
1039.3878	1045.1002	1047.9267
1050.7003	1059.2020	1063.0175
1063.3986	1063.9113	1066.2766
1067.6075	1069.7135	1071.4110
1073.4934	1083.5821	1104.2210
1105.0547	1113.0915	1114.5628
1119.4538	1120.0636	1124.8578
1126.5548	1127.4129	1132.2607
1134.9158	1141.0280	1146.3200
1165.4138	1172.6220	1175.0947
1175.9295	1180.7668	1182.1926
1189.3308	1199.1383	1202.7911
1205.1552	1207.3708	1207.6803
1211.4583	1221.0279	1223.3012

1224.8772	1240.4702	1245.1086
1248.6729	1251.0493	1252.8475
1253.7982	1258.8395	1267.9759
1270.1786	1277.8970	1286.6908
1289.8695	1298.7526	1312.0903
1315.6689	1321.7350	1325.4402
1328.5513	1329.9419	1332.7282
1337.3351	1338.3277	1339.5397
1344.2671	1350.4588	1356.1643
1357.7864	1359.1381	1362.0227
1364.0023	1364.9780	1369.3737
1375.8517	1377.2698	1383.6147
1387.8435	1389.6510	1394.1999
1397.4267	1407.2645	1411.4545
1411.7219	1421.2017	1421.7989
1423.0994	1424.6257	1426.3990
1432.7856	1442.6230	1449.2267
1452.7976	1466.9473	1471.1752
1480.3024	1483.1968	1484.5254
1484.7166	1485.6945	1490.8102
1495.2085	1495.4017	1496.6031
1496.7224	1499.0150	1501.3994
1501.8640	1502.7423	1503.4261
1505.2041	1509.3124	1509.5229
1511.1880	1514.0131	1518.0747
1519.9836	1538.9469	1543.2420
1545.4764	1548.3564	1552.3988
1554.3401	1554.7117	1581.7047
1613.0608	1623.9650	1664.9179
1670.6719	1671.3093	1674.0819
1678.0105	1687.1963	1693.1143
1694.9497	1695.7967	1696.2165
1699.0299	1712.3881	1863.3507
3038.4100	3052.1084	3055.6104
3056.2103	3066.5722	3066.7403
3067.9408	3071.0526	3071.3828
3077.1167	3079.2936	3083.7509
3085.7822	3086.4829	3088.4996

3088.6454	3107.3606	3116.5271
3119.3423	3123.2487	3125.6628
3134.0441	3135.7459	3138.1878
3139.6659	3142.2459	3144.4468
3147.8569	3150.0031	3152.3068
3158.0832	3158.5762	3164.2041
3165.0352	3176.2498	3176.4857
3178.9908	3182.3780	3183.9334
3185.6305	3190.0410	3194.0480
3195.2572	3196.6173	3196.8287
3197.1076	3197.1573	3202.6156
3206.0351	3206.5933	3209.3063
3211.1335	3212.2970	3214.8642
3219.5907	3223.6037	3224.3186
3230.0095	3230.4164	3241.2963

TS7(S)^{DT}

-1601.2538	11.8164	17.6763
18.9446	20.3456	24.7253
30.3034	35.8693	39.6753
43.1860	47.7165	50.2022
52.3487	57.5363	62.6791
66.7443	77.9248	81.6296
87.9591	94.2827	104.3891
108.6286	118.7298	128.9145
147.6579	151.4428	153.0197
159.1687	160.2690	171.5608
174.9266	196.1644	201.2354
202.5864	212.5518	222.1833
225.6700	230.2579	238.4922
242.8028	247.6643	258.0669
271.5141	280.3874	284.5996
297.8807	304.3978	332.8437
343.6202	348.7582	369.1623
379.1719	383.8593	392.5069
399.9313	409.8321	415.6815
418.6390	419.0199	424.7090
429.0771	432.0224	445.9635

456.6557	490.8040	496.5419
506.6887	510.3544	519.8070
530.6325	532.2877	544.1999
551.7842	559.4240	566.7402
571.1422	579.4924	582.3835
592.9084	606.4400	621.7543
627.5691	629.0675	629.9630
636.9910	643.3460	657.4038
661.0130	672.1024	697.3088
714.7442	719.2402	722.6622
723.1351	724.5001	734.1143
740.1323	750.1132	757.1961
767.8423	784.7996	788.5585
793.8307	803.4633	826.4715
839.1477	845.3639	857.1432
875.8145	879.7443	880.9793
881.7395	885.1885	887.1230
890.6473	894.8531	924.2813
931.8226	947.0861	958.3816
966.0075	967.4357	979.1280
989.9830	1003.0523	1003.5563
1007.7146	1009.5356	1010.8586
1011.4314	1015.1150	1016.2740
1016.3368	1019.5247	1023.4758
1026.4331	1034.1269	1037.1576
1038.0447	1038.5422	1043.7499
1054.6883	1060.4967	1063.2914
1064.5599	1065.9488	1066.5903
1067.0195	1069.5698	1070.0187
1072.3672	1073.9791	1080.3902
1112.1614	1115.8490	1117.6468
1119.6063	1126.0324	1139.5227
1145.8645	1159.3399	1168.7460
1175.8013	1176.8335	1177.2865
1177.8627	1193.1569	1201.4093
1203.7581	1205.8370	1206.9819
1209.6334	1211.5509	1215.8663
1217.8454	1241.0526	1246.2343

1248.7008	1258.9078	1261.8887
1265.4647	1281.9349	1299.6635
1303.3667	1320.4335	1328.5461
1332.7318	1333.5544	1335.0388
1336.6858	1342.2394	1343.4505
1352.0174	1352.6238	1355.8623
1359.6224	1361.9645	1364.9440
1381.9079	1384.9784	1414.7308
1420.2692	1421.5802	1428.4852
1428.9189	1441.5286	1447.9404
1454.3893	1460.1868	1472.6867
1484.5385	1486.6854	1488.1692
1488.6845	1492.4291	1494.4287
1494.9570	1495.3888	1496.6359
1499.4719	1503.1976	1504.7836
1507.5026	1514.6803	1519.4067
1534.5872	1536.7372	1547.7415
1549.3290	1549.5656	1550.8700
1566.6957	1643.7540	1657.4218
1664.4425	1668.4473	1670.9434
1673.7546	1683.3685	1683.8028
1687.2866	1687.6581	1693.3992
1693.7911	1716.3771	3042.2013
3058.1752	3063.7212	3064.1126
3069.4617	3079.6617	3092.6114
3133.3407	3135.0932	3135.5197
3137.5006	3150.9259	3166.1023
3170.1794	3174.7121	3175.1110
3175.7165	3177.7958	3180.1405
3181.2348	3184.1719	3190.5654
3201.8676	3202.1678	3203.5979
3205.4519	3209.5990	3212.6114
3212.6408	3213.5808	3218.5190
3220.4402	3224.1002	3225.5381
3230.8047	3233.6803	3235.1211
3235.9382	3237.8482	3240.6624
3242.3881	3242.8900	3263.2702

TS7(R)^{DT}

-1673.3468	5.4118	11.7102
20.4845	25.8581	30.5691
32.2717	39.6551	41.4582
45.0064	49.6578	54.9354
55.1941	60.3025	65.6226
66.2476	70.4053	73.8229
75.7705	82.8677	94.0114
100.2406	108.3580	119.8569
124.5418	136.6772	147.4018
153.2798	157.6231	163.1864
167.0508	174.1407	181.4803
192.5495	207.0466	209.1948
215.1208	228.1641	235.7504
242.8730	248.1973	258.5043
268.1773	272.5043	283.3545
301.7233	309.5743	320.6709
341.3758	344.6078	352.6376
367.0829	373.1456	386.7232
399.1736	404.6792	408.5808
414.5823	415.8703	418.1131
423.7723	435.4230	446.7927
464.3179	483.2763	485.9025
502.7155	507.4540	512.2893
520.8600	527.1243	546.3945
553.1277	558.9710	564.1081
575.5621	579.3586	582.1945
589.1136	597.1810	620.1857
626.2734	628.4432	629.5378
640.3088	647.2212	651.4104
664.4386	678.7095	704.5195
710.0207	711.8652	718.7007
721.7495	724.3077	736.2983
743.7575	750.3644	755.4627
778.8499	784.6713	793.0207
800.3534	811.0995	823.5691
833.7981	841.2058	859.2982
861.0295	863.9176	866.7886

869.9449	879.0871	881.3979
886.8076	908.2617	919.9234
936.6155	945.5519	948.1682
959.3455	966.2153	971.7805
980.4381	987.1746	993.0709
1002.1981	1003.4021	1009.0031
1013.3024	1013.5656	1015.6649
1017.0053	1017.9794	1022.4506
1023.0481	1031.6652	1037.7391
1038.0174	1040.9318	1046.1315
1050.2875	1050.7724	1059.1548
1061.1410	1063.7560	1065.0807
1068.7722	1070.7237	1071.1511
1073.2120	1078.1466	1088.5519
1109.5915	1113.1652	1119.5736
1123.6561	1130.6576	1144.7443
1147.0767	1161.9657	1173.4677
1174.7666	1177.8866	1180.7430
1187.6015	1193.2771	1207.3006
1209.7940	1210.7073	1213.7988
1214.8357	1217.4302	1226.9920
1235.3574	1247.0534	1254.2109
1256.3950	1267.3927	1270.7345
1278.7657	1286.8138	1295.2673
1315.9014	1323.5223	1333.2395
1335.7553	1337.0612	1339.1088
1341.4130	1347.7187	1356.4225
1359.7191	1361.1763	1363.8283
1367.1123	1368.6660	1373.1457
1383.4808	1387.0974	1414.6133
1417.4710	1418.0137	1427.5684
1428.8509	1451.7020	1457.8319
1461.4557	1464.3539	1480.3673
1490.4074	1490.9455	1492.0284
1493.6926	1494.3488	1496.5488
1496.6980	1497.2926	1500.3649
1500.5438	1503.8060	1506.4020
1513.4682	1519.3570	1528.9573

1539.1538	1544.6834	1552.7376
1553.5950	1557.6004	1561.1317
1583.6886	1632.1664	1663.5318
1671.3077	1672.2951	1673.1680
1675.3423	1688.6551	1693.8496
1696.3281	1697.4191	1700.0633
1700.7692	1728.7035	3038.8323
3066.1917	3066.7360	3069.0173
3071.6521	3072.6713	3082.1394
3137.1169	3137.5136	3137.6248
3142.9572	3143.7030	3144.9314
3157.8940	3162.4402	3164.3500
3165.2962	3178.6536	3179.5460
3180.8050	3187.8043	3188.9241
3193.7594	3195.6883	3197.3624
3199.7270	3200.4937	3201.6423
3202.3092	3204.6527	3205.0348
3206.5659	3210.8998	3211.4591
3216.7676	3218.2110	3220.4844
3227.2033	3227.4683	3231.4860
3234.7238	3236.0171	3237.8347

TS8(S)

-108.6643	14.9308	19.3621
23.1266	26.4978	29.4969
31.5385	31.9987	41.1817
42.3096	47.9895	51.5758
54.7065	56.1054	58.7872
66.5872	72.5770	81.7424
89.7860	92.9803	102.5835
103.6808	115.6133	123.5052
131.8279	142.2252	148.8824
158.6532	175.3904	183.9623
188.4504	195.2347	201.9385
213.0775	218.9034	226.1946
237.0888	243.3513	244.8383
260.7554	268.0497	277.9034
284.7398	286.1553	302.6672

316.8047	325.9917	342.8615
344.0216	345.6955	358.6975
368.0968	384.6010	391.7840
411.6439	413.9355	418.8788
421.8043	423.7929	424.6608
435.1544	449.0377	465.7330
484.5617	487.7861	506.2087
517.6691	521.5951	528.7389
530.3079	532.2738	543.1478
550.6497	561.1132	566.2032
585.8868	586.6894	591.3381
606.4167	611.4595	626.4426
627.7472	630.3878	631.2307
642.6005	645.9712	663.3388
668.0252	687.2308	712.0115
720.1425	721.4486	725.4484
729.4841	739.0937	754.5516
759.9575	767.2681	776.0606
781.7231	803.2129	806.9307
826.5686	832.5971	836.8781
846.1455	853.8506	863.3121
868.6551	877.7594	885.4281
885.8074	889.0617	912.3357
918.9151	934.9076	943.9182
950.3432	958.4833	963.4455
964.7577	973.8233	975.3033
980.8610	992.2053	993.2074
997.0519	1007.9895	1010.1848
1013.2295	1013.7464	1015.4818
1015.7144	1015.8741	1018.8412
1025.9379	1029.8067	1031.5863
1037.0023	1040.7781	1043.0634
1052.3424	1063.4307	1063.9936
1065.3451	1065.8977	1066.5323
1067.6164	1068.6590	1072.3941
1074.9740	1099.3914	1100.3285
1104.1408	1113.0855	1115.7541
1121.5931	1130.2371	1137.1497

1144.7834	1158.6652	1174.0480
1174.7338	1177.1619	1181.1101
1191.5250	1198.8562	1200.6085
1201.1553	1203.3905	1204.5533
1206.2928	1219.3810	1220.6727
1242.7030	1248.4675	1249.9091
1269.1445	1276.7190	1280.9268
1282.8334	1299.5563	1321.2675
1325.0293	1325.6816	1330.1818
1332.8135	1335.8826	1338.4561
1345.2910	1347.6164	1352.0454
1353.0273	1355.4307	1357.0295
1359.2532	1368.4443	1378.8461
1379.2707	1390.6673	1395.6748
1419.7137	1420.0287	1425.0570
1432.2574	1433.3733	1447.7853
1463.5500	1471.0597	1479.7828
1486.8967	1487.8952	1493.2612
1493.5055	1494.3402	1494.9434
1495.9887	1498.8126	1499.6094
1499.9345	1500.5666	1504.3868
1509.7678	1521.2705	1532.7953
1541.2523	1543.8351	1546.5384
1548.2528	1548.7796	1586.8571
1598.9299	1661.8947	1663.2268
1669.7643	1672.4987	1680.2173
1685.2348	1685.5646	1688.0069
1689.7780	1691.5725	1692.9711
1738.3385	3046.1939	3047.2058
3050.7426	3068.4382	3076.8645
3077.0169	3081.8908	3085.2472
3088.2774	3135.3454	3141.8841
3147.2293	3147.9625	3155.8635
3160.5371	3172.8213	3174.0213
3176.2618	3179.8747	3182.0675
3185.3376	3187.9144	3190.3421
3195.7640	3203.9319	3204.6339
3206.0423	3208.5232	3208.5452

3208.6219	3210.1016	3215.3299
3216.2660	3216.4527	3217.8374
3222.8925	3223.0371	3223.1432
3227.0021	3233.1190	3235.5754
3238.4322	3239.6289	3247.2088

TS8(R)

-50.0516	13.9585	22.8885
27.5327	28.7667	30.8592
33.4082	35.8858	39.5882
42.9446	44.5753	50.4603
53.1837	56.1052	67.0368
74.3517	74.9844	80.0952
85.1761	104.2972	106.8662
115.7015	119.7312	134.2310
139.2171	145.9433	155.1253
165.2010	166.1655	187.2612
189.6271	202.4361	206.7915
211.9961	221.2763	231.2696
233.4441	245.3680	256.2839
259.3423	267.5212	272.5767
285.2008	300.7187	304.0319
315.0705	329.2386	345.9386
359.5592	362.9557	371.8161
378.8158	391.8113	395.9059
408.1932	416.3350	416.7207
418.1069	420.2144	422.4053
432.5023	448.0772	475.0888
483.8788	495.7387	502.7290
513.2920	520.6081	529.7836
532.6463	535.4777	547.5279
556.3439	561.6140	564.2489
582.9839	586.9377	594.3339
607.8876	624.3723	626.7630
630.4517	632.1003	639.2294
645.9468	650.8027	660.9819
679.4541	692.7794	716.0744
719.5942	724.1559	724.5798

726.8151	743.5087	748.6400
750.9261	763.4783	778.6420
789.7654	793.5213	800.3431
823.7884	832.7199	840.9249
844.4291	856.2808	873.0926
875.8960	877.5237	879.1165
880.7673	886.5313	897.1871
914.7406	923.4948	942.7274
952.0597	959.9238	968.4175
972.1952	991.2765	995.5497
995.9691	999.2653	1001.7208
1010.4816	1011.8293	1013.2219
1013.3204	1013.7685	1017.8222
1017.9298	1019.3853	1021.0679
1022.2786	1027.9338	1038.4289
1041.5128	1042.6269	1048.2663
1049.8178	1050.7382	1056.6976
1064.9600	1064.9772	1066.7500
1067.1049	1069.7595	1070.9635
1073.4855	1089.3124	1107.0297
1110.9623	1112.4989	1117.9935
1125.5612	1126.4148	1152.7979
1155.8894	1160.2682	1173.9404
1174.2941	1175.9458	1176.1960
1196.8570	1198.1476	1198.9190
1205.2402	1208.0285	1211.5008
1214.5491	1218.2729	1236.7470
1241.5400	1247.6957	1257.1566
1268.2348	1272.3427	1276.6282
1282.1736	1303.0325	1316.3973
1324.9268	1329.7744	1330.7743
1335.0582	1343.2785	1346.0683
1349.9292	1354.4775	1355.3144
1356.6655	1359.3475	1361.4665
1363.2799	1365.2208	1385.0434
1387.1436	1392.9666	1398.4925
1418.0075	1421.1428	1424.2255
1434.3207	1436.6215	1454.9295

1457.6215	1468.4642	1473.9311
1482.0429	1482.9832	1491.1343
1492.5250	1492.7359	1494.9567
1497.1651	1500.0822	1500.5805
1504.0147	1506.1451	1507.2242
1510.9140	1514.2149	1517.9728
1541.4504	1546.8018	1549.2886
1554.3162	1561.1528	1582.1141
1670.7185	1671.9885	1677.6803
1680.2769	1681.0434	1687.4356
1694.2572	1694.5010	1695.7149
1699.2601	1701.4589	1712.2341
1725.6611	3055.2352	3056.2547
3066.4951	3068.6195	3071.8286
3073.8490	3075.9483	3077.1614
3108.0317	3138.9376	3146.6129
3147.4554	3153.2841	3157.6307
3164.2849	3164.4509	3168.3984
3173.7860	3182.7992	3184.5293
3186.6236	3187.2848	3189.3096
3191.1392	3192.1024	3192.4464
3193.7782	3195.4615	3197.3783
3197.4626	3198.7609	3204.6982
3205.7623	3207.0136	3207.5488
3208.3243	3212.1389	3213.6443
3217.4115	3217.5446	3227.2951
3228.2578	3232.1046	3241.9250

TS9(S)

-144.2547	7.9358	14.1456
20.2314	23.0086	24.1423
28.0811	30.8696	32.8826
34.4658	38.6407	42.5804
46.5187	53.2776	58.9106
61.8356	65.0688	77.0418
82.4834	91.1070	97.5530
103.8133	106.0846	117.0503
122.9333	130.4332	145.0085

156.2872	162.7434	171.3722
185.9316	197.4787	201.2865
210.3706	227.8122	233.1929
236.1100	239.6324	245.3013
254.8583	261.9228	275.8622
280.2639	282.2079	289.5893
315.3524	325.3571	331.3901
335.3629	344.8577	352.6756
366.7059	378.9911	387.5068
404.5185	415.3170	416.0201
416.1633	420.4410	423.4503
438.7179	446.1741	474.6679
479.9682	498.9358	506.6332
511.6245	525.7049	527.4136
532.6512	537.3910	546.8262
556.5016	560.6657	573.8428
581.5486	587.8514	601.8681
615.5839	620.3771	628.9368
630.8886	631.3407	632.7722
639.1840	645.1775	658.1444
671.5783	677.3955	710.7814
712.6929	715.2460	719.4338
722.8772	724.0319	724.9155
741.1732	756.6545	783.3094
790.0488	794.6969	808.6923
827.1615	830.6059	833.7253
854.4633	862.2706	864.2613
873.6054	878.6423	881.3671
884.1794	886.1085	892.4582
917.5960	943.9010	948.7568
954.0449	957.5392	961.8434
978.1954	982.4884	990.2556
996.7956	1001.4552	1003.4261
1003.9234	1007.7412	1011.0392
1013.8888	1014.9186	1016.0090
1016.3854	1019.7457	1022.1445
1025.2976	1030.2724	1033.4591
1035.9611	1039.7405	1040.7708

1051.5892	1056.3559	1063.2008
1063.3491	1065.5544	1065.9660
1066.9772	1067.7698	1069.7015
1072.3842	1086.9539	1099.3325
1105.1456	1111.7879	1115.0640
1116.9221	1122.6235	1124.4230
1141.8589	1149.8941	1174.6368
1175.9718	1176.1630	1177.5489
1191.5106	1199.4546	1202.2330
1203.7241	1204.5910	1208.1975
1214.0107	1221.4535	1230.6220
1237.9397	1248.5546	1250.0529
1264.4027	1265.7628	1280.0779
1294.3244	1307.5911	1318.5712
1324.2033	1325.9082	1326.3548
1328.6572	1333.6051	1342.2384
1344.1787	1347.9083	1348.4568
1350.7981	1352.2660	1353.6237
1356.3524	1357.6381	1362.8948
1379.7341	1384.6234	1385.7719
1403.4748	1417.6588	1419.1384
1420.4862	1428.2318	1432.9824
1449.3838	1459.1855	1463.1892
1479.4497	1481.8911	1482.8759
1487.9995	1488.7701	1489.9504
1493.5616	1494.9183	1495.4468
1497.0833	1502.4724	1502.9206
1503.4077	1504.0802	1509.5234
1518.8419	1546.3701	1546.9700
1548.7117	1549.4112	1565.6634
1663.4182	1665.6915	1670.5524
1672.4949	1677.8566	1683.6765
1684.7102	1690.9376	1691.9998
1693.0180	1693.2067	1718.5336
1728.9290	3018.5426	3045.1223
3063.3036	3075.6476	3079.6638
3080.9219	3081.5481	3083.4209
3109.6079	3138.3558	3145.5582

3148.1403	3150.4482	3154.6190
3165.6842	3168.6031	3170.0468
3170.2477	3171.9916	3174.7564
3176.7228	3178.2829	3193.6898
3195.7925	3200.9561	3202.9105
3205.9446	3209.1681	3210.8861
3213.4408	3213.9656	3217.9660
3220.8133	3221.2267	3224.9841
3225.2879	3226.0533	3226.5388
3236.3313	3237.6423	3237.9842
3238.4965	3259.1188	3272.8549

TS9(R)

-137.2054	15.6240	16.4607
23.0174	28.5245	31.9540
32.5544	33.5632	35.7225
41.6643	43.7637	50.2477
59.0379	60.2593	61.4624
67.1677	78.6020	85.7854
86.2185	95.1565	99.5987
109.7777	114.0892	121.3602
127.5354	133.6043	152.5004
154.4472	157.8762	174.0484
186.3317	197.5150	201.1038
209.5758	226.5587	234.1554
236.5735	244.9133	246.8312
257.0312	261.3771	275.1159
280.1444	284.6674	289.6561
315.6778	330.8930	333.8089
338.9212	347.5511	350.0674
372.5054	380.3627	393.0488
410.8619	416.5250	418.3202
419.9463	423.4508	426.3002
437.0909	448.3998	474.1171
482.4596	499.6098	505.8005
509.4028	522.8237	527.9385
533.5331	538.8589	547.3902
557.0208	565.4949	569.3889

581.1798	588.9869	604.2267
616.5275	622.3915	629.1753
630.3571	631.8006	632.9585
640.6644	645.4813	658.0368
671.9726	676.3600	711.0012
715.5633	718.5580	720.9277
724.2339	725.4189	727.7550
742.1497	757.8523	784.3342
787.8696	795.2249	805.9484
826.7811	833.0267	839.7165
853.9992	865.9943	870.1866
876.9357	879.7320	885.2494
886.5687	887.5810	894.4369
922.6994	943.5247	954.4609
954.5392	961.4918	963.7125
975.6568	988.6383	992.5600
1001.8291	1002.8102	1004.8516
1006.9616	1008.8956	1014.3157
1015.1311	1015.4661	1015.7429
1015.8749	1017.5508	1022.8271
1027.9046	1031.9111	1034.2128
1038.0637	1039.7750	1041.9964
1052.7516	1057.0792	1061.9957
1062.9650	1064.4318	1065.3267
1065.7195	1067.8294	1069.1985
1075.1215	1086.5188	1099.5713
1105.9641	1113.1382	1118.1481
1119.2371	1124.3364	1125.8682
1142.7625	1147.6000	1174.4176
1176.3552	1176.5101	1179.8391
1190.3146	1200.5745	1202.7748
1203.2112	1205.1864	1209.1596
1215.0360	1221.4927	1231.0793
1237.3868	1245.7944	1250.6837
1265.0032	1265.5761	1278.0700
1291.7963	1301.5198	1319.6261
1322.6662	1325.8174	1329.3477
1333.1340	1341.7586	1344.0609

1347.6889	1349.3730	1350.0034
1351.8576	1352.8486	1354.2093
1357.9575	1360.3855	1364.0677
1380.4578	1384.0246	1389.6152
1406.5417	1416.4603	1419.2827
1420.4791	1428.4236	1432.0080
1449.7622	1456.4078	1462.9886
1475.5067	1480.2526	1485.7104
1487.4805	1491.7527	1492.1091
1492.7608	1494.1251	1495.2340
1497.5158	1500.7124	1501.7241
1503.0755	1504.2017	1510.3330
1518.5056	1545.0066	1547.8023
1548.8039	1549.3175	1559.7005
1663.9507	1665.3454	1669.5404
1672.4007	1677.4752	1684.4303
1685.2814	1690.3381	1691.5543
1691.7551	1692.6754	1695.3146
1727.9487	3026.5798	3050.9245
3069.4244	3072.8489	3074.8551
3078.4250	3082.3183	3082.7724
3109.4257	3127.4119	3142.9142
3144.3335	3147.4560	3153.6146
3165.4315	3168.1949	3171.0324
3173.4171	3175.3744	3175.5057
3184.7309	3184.8732	3187.7816
3195.6227	3202.1646	3202.4789
3206.6881	3207.7915	3208.4542
3211.5620	3211.7343	3217.1907
3217.7341	3218.0964	3224.0672
3224.4752	3226.1062	3228.8446
3230.8652	3235.8698	3237.6109
3237.8990	3255.2984	3260.5339

TS3$_A$

-86.7148	14.3423	21.6381
25.6831	26.6912	31.5053
36.5783	40.8431	46.1196

47.9155	51.0544	59.4747
60.7463	67.1193	70.6098
75.7666	77.8601	83.4241
91.7428	99.6662	107.5068
113.8273	120.1927	123.5794
128.4706	141.6160	143.9133
157.0515	168.5242	174.4399
179.1761	186.1815	207.1628
209.0480	217.0989	221.6824
225.7843	236.5208	238.1672
257.1618	260.0781	276.1444
284.5280	287.3479	289.1398
301.3996	314.8875	322.1681
329.6283	339.8990	351.8006
358.1749	362.9488	384.0001
390.1514	394.3370	404.1155
417.2739	418.1758	420.6063
422.7627	429.7417	439.0917
454.2195	471.5944	483.6744
499.3999	502.4217	509.8114
519.1890	524.8527	531.8353
535.2799	547.7043	550.1018
557.9638	576.1069	580.6262
586.2368	587.8334	590.5013
613.5003	629.6652	630.6146
631.6173	634.4208	635.9812
644.4231	664.9608	694.1347
710.9870	718.7426	721.7977
723.4980	727.0111	730.6277
736.5319	754.8957	764.0434
767.0683	779.7688	786.9266
790.3407	810.4246	817.2177
819.5511	828.5566	843.2118
852.2723	861.5870	877.1526
880.7050	882.5234	883.5860
885.8003	898.9920	900.7731
911.2340	916.4315	946.7747
950.0736	956.7874	970.0576

983.8452	991.6294	993.2674
1002.7889	1003.3774	1006.2696
1006.7922	1011.0473	1015.7229
1016.8985	1017.2544	1017.5499
1019.5381	1024.0136	1027.9839
1030.0850	1035.5024	1040.0386
1043.0452	1046.7305	1048.3810
1052.9650	1062.6307	1063.7389
1065.2794	1068.0038	1069.9976
1070.5690	1071.5368	1072.4904
1073.0513	1074.6275	1087.4602
1110.3825	1115.4007	1120.9441
1122.7392	1129.5115	1137.9445
1153.6637	1157.8567	1166.2337
1172.7614	1177.7839	1179.3951
1195.4900	1196.7762	1199.0550
1206.6949	1207.5359	1210.9036
1212.2643	1221.6655	1240.3170
1241.6263	1244.1068	1251.8035
1270.7333	1285.7615	1294.1116
1297.0842	1314.8642	1325.4836
1327.4751	1329.0097	1337.4806
1342.4109	1344.3575	1347.8160
1348.4747	1352.4992	1354.1882
1357.3108	1359.2186	1360.6294
1362.7058	1367.1573	1382.6839
1394.3265	1400.0294	1419.9104
1420.6318	1424.0286	1424.4893
1430.8764	1434.7995	1441.5571
1450.4398	1466.1256	1473.2334
1482.4112	1490.0567	1491.8683
1493.2691	1493.7030	1494.5886
1494.9412	1498.4575	1500.0359
1503.7346	1505.1071	1505.8599
1510.1020	1518.6474	1523.1769
1527.5422	1538.7212	1549.1484
1552.6329	1553.1994	1554.2275
1565.0135	1665.9695	1666.5159

1669.8656	1674.3808	1676.8084
1684.8204	1688.1377	1694.3583
1695.0466	1697.6415	1700.1487
1700.2858	1700.6679	3049.2181
3058.2485	3066.9019	3068.1473
3073.9654	3076.2324	3095.3283
3097.5806	3135.7313	3139.1103
3143.4442	3144.9500	3152.6456
3154.6993	3155.9240	3160.9900
3164.2873	3167.9806	3169.8762
3174.0627	3186.2457	3186.4591
3191.2189	3191.6626	3194.1175
3195.8945	3197.3599	3197.9929
3198.9119	3201.0930	3203.1454
3203.1747	3203.8605	3208.5588
3209.5708	3211.5064	3214.6335
3218.4740	3220.0719	3224.6342
3225.4226	3228.4378	3231.1151
3234.7520	3238.7258	3312.6878

$TS4_A$

-171.1059	10.5138	15.5432
19.2267	25.4476	27.2807
34.6473	38.1831	41.4350
42.5731	42.9625	47.0967
57.3193	61.0058	63.7440
73.1210	81.6042	86.7701
89.6563	104.9532	108.7810
111.7854	119.3649	126.8575
132.2908	133.4531	147.0410
165.6529	172.5961	184.8151
193.5875	212.8804	216.3488
221.9193	223.9827	227.8783
234.6318	235.7933	247.9365
268.4945	272.6117	280.0849
285.6895	292.9131	298.9608
305.6732	317.8802	331.5690
339.0008	352.1268	359.5081

361.1410	377.4095	382.1427
388.5470	399.6167	405.7468
414.4604	421.6931	422.6139
427.1075	428.4598	442.2147
444.2365	467.4109	491.9001
499.7382	503.3632	511.2366
516.3463	524.6660	533.1991
544.3854	551.7670	556.4497
558.2603	568.8955	582.6503
585.1972	588.3682	592.2317
615.4837	623.8682	629.2200
630.3986	634.9198	635.5596
643.2879	665.8303	682.7907
702.1537	713.8229	719.5884
721.0318	723.7268	724.4271
727.9100	744.9199	754.9047
763.7111	774.3957	786.5265
795.2013	803.3250	815.5925
826.6632	833.4868	844.5801
849.7222	854.9083	874.0222
880.3664	882.1654	883.4151
884.0763	905.3484	915.7794
937.7529	941.6768	952.0102
957.9515	971.3343	976.5077
981.1224	993.1297	993.9794
998.1935	1004.3625	1005.6805
1009.0362	1010.4241	1011.5551
1014.8814	1016.5202	1017.2355
1018.3110	1022.1934	1023.8146
1025.0442	1029.5790	1035.9201
1042.2300	1049.6528	1053.1182
1053.5222	1062.6627	1062.9567
1065.0198	1066.8866	1068.1005
1070.6501	1071.1190	1072.3129
1074.7482	1075.5093	1088.4484
1108.3404	1116.9478	1117.1421
1121.3364	1127.9734	1133.5595
1145.3046	1157.3095	1174.0626

1174.7376	1175.6943	1178.6571
1191.8249	1197.5416	1203.5877
1204.9360	1208.6714	1210.1785
1212.4390	1221.1919	1238.9808
1242.1856	1246.6622	1254.6830
1272.0325	1284.4615	1292.9512
1300.9737	1321.1517	1329.8756
1333.1065	1334.3874	1338.3603
1340.6114	1341.0975	1345.5715
1345.6889	1354.1664	1356.0315
1357.3199	1357.7380	1358.9110
1359.5791	1361.7679	1379.7717
1386.3328	1390.9353	1400.3266
1418.8542	1420.5835	1426.9599
1435.3946	1436.7269	1444.1127
1453.7874	1460.7058	1466.3454
1477.9955	1491.2817	1492.1346
1493.0041	1495.1564	1495.8094
1497.8353	1499.9821	1500.7614
1501.7985	1504.2106	1509.2212
1511.5641	1516.2705	1517.2372
1525.3714	1527.7804	1548.7445
1552.2387	1552.4507	1553.2578
1555.9754	1615.2690	1666.6464
1669.9601	1675.4554	1676.0531
1681.8468	1692.7147	1694.4094
1696.1533	1696.3238	1698.2098
1699.5358	1731.1682	3041.3745
3050.3506	3064.5569	3066.7405
3073.7388	3076.7157	3078.7410
3091.0308	3122.3696	3125.7649
3137.4997	3138.2608	3141.6203
3144.1527	3149.3466	3153.6316
3161.2380	3165.0908	3171.5731
3173.4933	3176.2776	3192.0572
3192.9448	3193.0904	3194.2899
3194.7890	3196.3018	3196.5302
3198.8435	3199.7098	3204.4921

3207.1926	3208.6471	3210.2633
3212.5014	3214.7558	3218.0059
3218.8229	3226.7297	3226.9646
3230.0707	3232.7030	3234.8356
3240.0642	3244.4443	3318.2155

TS3$_B$

-239.4965	7.7104	19.6240
20.3796	22.4321	28.1952
28.7053	29.4911	34.3649
37.9078	42.9516	44.8535
49.8662	51.8093	66.7070
74.0444	74.8614	76.0830
86.1611	95.0145	101.3653
103.6986	110.2013	120.6162
125.9216	130.9212	150.5643
151.5641	169.7879	173.1867
177.4113	195.9740	201.5856
203.9231	209.3294	216.6666
230.3809	239.9643	242.1789
244.7159	248.0720	253.2865
278.8770	284.4064	288.4096
298.8871	307.0620	313.2804
320.8674	338.4839	344.4859
365.9391	367.2662	372.6549
385.8129	401.6097	407.6226
418.5664	419.0454	421.1481
430.8521	437.7776	446.1565
463.0353	468.6169	476.7296
484.2171	493.6725	505.6723
515.8177	518.1095	524.5597
532.5933	534.7510	549.0916
555.4266	567.3767	572.5789
584.2279	586.5892	589.1493
627.2122	627.9155	631.0218
631.9569	635.1229	643.9016
648.2405	673.5080	696.8420
712.0176	717.9997	718.4269

721.5999	724.1853	731.7780
738.8456	750.6193	763.6535
767.2276	781.7629	785.3503
789.2254	793.0336	815.7995
821.5155	829.4883	839.9071
853.6612	857.7697	865.9579
871.0960	876.8132	879.9709
881.5560	885.8970	889.5975
896.6225	916.0651	955.0399
962.5473	963.9638	966.4993
980.2298	981.5326	997.0207
1000.4252	1006.1852	1008.1040
1010.1808	1012.2161	1014.1534
1016.1097	1017.7132	1018.9190
1019.5565	1026.8413	1027.2878
1029.6378	1034.9254	1038.4301
1039.0554	1043.5265	1051.3912
1053.0039	1065.1754	1065.7815
1066.3775	1067.3871	1069.9202
1070.2647	1072.5226	1072.6822
1074.7913	1084.3548	1103.5901
1113.3101	1117.3598	1123.7102
1128.5121	1130.3336	1137.2946
1152.2360	1159.4199	1167.0776
1175.2036	1176.3764	1178.5743
1179.7466	1196.8935	1202.7844
1206.8587	1208.3328	1209.7347
1211.8109	1218.0683	1235.6540
1241.4509	1252.1745	1253.8200
1271.0341	1281.9240	1287.8560
1297.6767	1305.5078	1319.1171
1327.6871	1333.7869	1337.9792
1338.1933	1341.4686	1346.4963
1351.2051	1353.3482	1355.3977
1360.8511	1362.7332	1365.0901
1365.7019	1385.0960	1392.2207
1393.3978	1400.8205	1413.6720
1420.9798	1422.9273	1427.6688

1435.2900	1435.7482	1448.9890
1453.2328	1466.8705	1474.5477
1476.4093	1482.2203	1485.6682
1492.3409	1493.7572	1494.6912
1495.5528	1496.5908	1499.2964
1500.4915	1501.2305	1502.5680
1504.8165	1506.6129	1509.7857
1525.6618	1534.4202	1545.7854
1547.8129	1551.5881	1554.0666
1557.6148	1575.5622	1662.2541
1667.6777	1673.8008	1674.2184
1679.0642	1688.5913	1691.6838
1695.0944	1695.5882	1695.9279
1697.6389	1700.1310	3042.5075
3063.8989	3064.6686	3066.3502
3070.5501	3071.6171	3084.0407
3097.6049	3100.8248	3134.2924
3140.3028	3140.7193	3140.9496
3141.4517	3142.6424	3157.8204
3162.2974	3169.4775	3169.8900
3174.7082	3176.0111	3178.1927
3186.2281	3187.8029	3195.1423
3197.9700	3200.0538	3200.3572
3202.2841	3206.2821	3206.9428
3207.0333	3207.7324	3212.6166
3212.9975	3216.0941	3216.9349
3221.1199	3221.8425	3221.9326
3230.6373	3232.6962	3247.0252
3248.5959	3261.6327	3323.6404

TS4$_B$

-156.0008	13.8129	18.0569
23.5685	24.8261	29.8180
31.5925	33.6394	38.5578
41.7393	42.9673	48.5794
52.6456	59.9874	63.5550
65.3905	66.9544	75.8543
86.8458	90.7278	98.1151

107.8177	111.3480	116.9550
126.2944	131.0635	146.6154
156.4973	162.0880	172.3616
194.3946	204.2848	204.7940
209.5911	220.0363	229.1556
233.9788	236.0148	240.5692
248.5767	255.5732	265.5665
281.7685	284.3122	286.3347
292.1480	299.9674	311.2223
334.9462	341.0661	346.7317
373.1924	376.3521	379.4331
392.4576	410.4190	414.3227
415.6556	416.7314	423.1361
427.2439	438.9465	444.5246
468.4719	488.7414	489.9449
493.7241	502.5220	514.6490
517.7817	522.2598	530.6244
538.3312	552.6463	556.4529
558.0427	570.2422	579.7621
583.3971	588.1720	613.0038
628.7436	629.6400	630.7255
632.5645	635.3485	641.8014
645.5543	667.7975	689.2863
707.4061	713.7105	719.2276
720.9502	722.9855	731.3170
742.1272	759.4983	764.8510
766.1258	780.1259	788.2254
801.8320	807.4517	821.0230
826.0740	835.8329	837.3785
850.6857	866.8977	877.1902
879.9398	880.9171	885.8977
893.4277	902.7339	929.6586
939.9335	945.5128	954.3135
968.4068	979.4942	981.7944
984.8469	999.9665	1000.4300
1003.2848	1005.7933	1008.9889
1012.4692	1015.2995	1016.6333
1017.5789	1022.9505	1023.1807

1024.4099	1028.0091	1030.5112
1035.2959	1036.7554	1037.1460
1039.2663	1048.3500	1052.1617
1063.8357	1065.5121	1068.0620
1069.0481	1069.7878	1071.9570
1073.4186	1074.6344	1080.1731
1084.0473	1101.9809	1112.4731
1117.8112	1119.9475	1125.1256
1129.5959	1132.7204	1144.5489
1172.8965	1176.4949	1176.6691
1184.2590	1191.5791	1201.1896
1202.4640	1207.5684	1208.9051
1212.7161	1214.3830	1221.9709
1224.0571	1238.8358	1247.4524
1248.8813	1253.8972	1270.8956
1281.0724	1282.0495	1294.1066
1305.3792	1317.9026	1327.3234
1332.4105	1334.5687	1338.6109
1339.9097	1347.8214	1350.3225
1351.5282	1355.0709	1358.0589
1359.3883	1360.5783	1361.0868
1362.0359	1374.3286	1379.0999
1385.8591	1390.6304	1404.2201
1420.9157	1424.0828	1428.5640
1431.9082	1439.5816	1456.0384
1459.1476	1467.5174	1475.1685
1484.5574	1490.3546	1492.9335
1494.2151	1494.3979	1495.3081
1497.4604	1498.4897	1502.0827
1503.1801	1504.1989	1504.9666
1507.1998	1508.6535	1509.8027
1522.2798	1525.6010	1546.9374
1550.2059	1552.4526	1554.3254
1557.0491	1564.7533	1654.8429
1672.9250	1676.8000	1677.8938
1682.0023	1686.2804	1692.4990
1694.0180	1697.3697	1697.8028
1698.5610	1699.8051	3037.9358

3050.1086	3062.0176	3074.0886
3075.5101	3076.7297	3078.3224
3090.1800	3118.9050	3120.4827
3123.5045	3131.5908	3136.5096
3143.7173	3143.7541	3145.4021
3155.6417	3164.5118	3168.6268
3170.1703	3172.7599	3174.2988
3177.7049	3185.6066	3186.9653
3188.4006	3194.6352	3195.4315
3198.1891	3202.1247	3203.4483
3203.5296	3205.3929	3207.5623
3210.8924	3211.7663	3212.8566
3214.1436	3215.2073	3220.3171
3223.1847	3223.8791	3226.6928
3231.1692	3233.0729	3236.7648

TS4$_C$(RR)

-265.5490	7.8486	17.0944
22.4547	26.8409	31.3962
36.9681	37.8993	44.1374
44.5112	46.4858	49.0468
54.5755	61.9464	66.1464
75.1062	82.7288	85.1989
96.5666	97.4247	99.8757
107.1044	115.3466	118.0258
129.7189	137.1402	142.5657
148.2244	153.2220	174.2394
183.2419	186.5441	209.8293
211.4871	217.0539	224.3793
226.1684	234.0575	242.6864
249.0954	255.3177	265.1202
275.6235	279.8281	288.5516
294.5342	310.8445	313.8822
323.2558	335.9856	344.9515
352.3321	366.4865	376.1357
378.7408	386.8952	395.5553
405.3664	414.0288	418.1106
419.4433	420.7254	425.2333

427.3096	446.4890	456.5714
482.0033	492.0351	506.4112
510.7411	518.8933	526.2006
526.7488	540.1564	544.9500
556.5384	557.9170	565.4997
574.1308	581.2110	589.4692
594.5377	626.6043	630.1010
630.5734	631.4629	637.0496
638.9200	644.9482	668.1397
686.2825	713.3851	717.0411
721.3618	723.8432	724.8398
734.4303	753.5404	754.8203
776.4674	781.8985	786.7447
793.2562	794.0656	819.0438
826.0358	829.4674	836.0146
849.7720	853.9982	870.3019
872.9588	877.1762	882.3276
883.1522	883.8880	899.7819
914.3236	947.4239	951.0874
956.0801	960.8313	967.3644
979.1821	983.7325	1000.4956
1000.7776	1002.4979	1003.1885
1005.5235	1008.8936	1013.1599
1014.8359	1015.8992	1016.3745
1018.4830	1020.7482	1026.2616
1027.9758	1034.9657	1039.4943
1041.1065	1044.9543	1045.7622
1050.2828	1057.6222	1062.6030
1064.2004	1064.8644	1065.0572
1066.2581	1066.5411	1068.0591
1072.3989	1080.5686	1106.7608
1112.5277	1115.5168	1117.6985
1122.3963	1127.0288	1136.6183
1144.0610	1161.2942	1172.8190
1175.7884	1176.4402	1182.8437
1197.7890	1198.7698	1203.7018
1205.3955	1206.6899	1208.2800
1209.8492	1223.1810	1238.7670

1240.8772	1243.2295	1248.9425
1266.7372	1281.9822	1283.7882
1309.5919	1310.8455	1320.1465
1325.6816	1326.2348	1331.7027
1333.0403	1336.9273	1343.0204
1344.2148	1349.1387	1351.5231
1352.6941	1353.7363	1356.8479
1359.7149	1374.2579	1387.3684
1402.3006	1407.3736	1415.3640
1420.8142	1424.4614	1425.6098
1442.7083	1446.5253	1450.1301
1464.1941	1470.3880	1476.8160
1484.1440	1486.4120	1490.5814
1492.6511	1494.0965	1494.8045
1496.3195	1499.7782	1500.6161
1501.6276	1502.4606	1504.8868
1505.6714	1507.2771	1511.3155
1519.5067	1520.0084	1529.4805
1541.8626	1547.0472	1548.2456
1549.6501	1551.4127	1662.1564
1663.9083	1668.8102	1671.4443
1678.4416	1683.6697	1687.0825
1688.8583	1689.5895	1691.1151
1692.9674	1703.1882	3039.1899
3064.5099	3073.0800	3074.1841
3075.9208	3077.2662	3077.5007
3083.2170	3092.6022	3121.5867
3141.9482	3145.2061	3147.2553
3150.9715	3157.4862	3158.9478
3163.4719	3169.4197	3171.3927
3172.5884	3176.9485	3178.6300
3188.5813	3188.8962	3194.9304
3203.7463	3204.7976	3206.6252
3207.2229	3211.9482	3213.2352
3214.1753	3218.1695	3218.3697
3219.3873	3223.2367	3227.4546
3228.9826	3230.5488	3234.4488
3234.4935	3236.1056	3239.6382

3239.8605	3254.9015	3307.1300

TS4$_C$(RS)

-309.4778	9.8175	13.8618
18.6243	26.2535	29.0624
32.0942	33.1595	39.4479
44.4336	49.8694	54.5571
62.7319	65.8430	68.0910
71.1504	74.9081	76.8338
84.6853	86.5767	104.2790
109.2771	112.3455	127.1057
131.4184	137.1791	143.8416
151.2546	163.3819	171.6737
195.8900	204.1735	208.4980
214.4330	220.3245	228.4540
234.1806	239.0959	243.9032
258.5599	267.3589	280.4795
286.5500	292.4517	297.1441
302.8949	306.2180	316.5226
324.7551	326.7078	349.0411
356.2612	363.2814	370.3302
381.1304	383.1476	405.1934
408.3723	414.9151	419.0322
419.8652	424.8326	429.8076
437.4199	450.3314	464.4878
485.2453	490.7591	493.3477
506.3981	514.6446	520.6772
527.3526	531.8487	549.5400
555.3490	562.4124	572.0664
578.8097	581.8610	586.5075
590.1863	614.5953	626.2236
628.0627	631.3416	632.1971
639.7549	645.0676	671.7976
714.0532	716.6586	719.8485
721.6989	723.4850	727.7499
732.7102	747.3609	752.6640
763.7533	777.6903	789.8137
792.1468	797.9358	817.8176

828.0794	834.2826	838.6296
854.1402	867.7201	870.2442
872.5984	873.0492	878.1459
886.0074	887.9909	900.2272
902.0510	921.0003	951.0577
963.6634	967.6030	969.4083
980.2426	985.9656	998.9432
1002.2399	1003.2650	1004.3316
1009.0414	1011.9063	1012.5220
1014.8544	1015.8937	1018.0729
1021.1480	1024.4487	1027.3519
1027.9104	1037.1197	1038.9868
1044.5434	1047.7742	1052.0712
1052.8796	1056.4430	1062.6341
1066.4141	1066.9635	1067.5944
1069.9580	1070.3607	1073.2955
1073.9567	1079.8610	1107.3393
1112.0620	1112.9342	1115.9542
1125.7120	1126.9990	1136.0456
1141.8383	1161.5905	1174.5419
1175.8365	1180.2559	1186.4203
1194.7240	1204.5973	1205.7885
1206.8465	1207.5020	1210.6419
1215.1395	1224.2766	1238.4149
1247.0281	1248.6748	1251.7237
1266.2687	1274.0766	1278.5993
1290.3367	1312.8638	1324.2011
1327.8824	1328.7156	1332.1725
1334.2458	1342.4046	1344.8236
1352.4553	1355.9957	1356.9181
1357.8137	1361.1434	1363.7511
1381.3766	1384.9907	1390.4816
1402.6470	1410.4922	1418.3403
1422.2547	1427.7695	1428.9521
1435.8880	1451.0677	1453.6825
1467.6669	1469.2249	1480.9658
1482.3031	1489.5228	1491.0229
1491.6073	1492.4792	1492.9062

1496.5527	1497.9293	1500.1466
1502.6407	1504.3746	1505.1276
1507.3587	1507.7869	1510.7029
1518.5038	1523.5272	1540.8114
1545.9195	1551.4900	1553.5020
1555.9946	1583.3664	1668.4530
1669.0143	1673.7363	1676.9212
1679.4336	1692.3194	1692.5358
1696.2152	1696.7494	1697.6159
1701.2609	1740.2993	2996.6931
3055.4095	3061.5208	3064.3247
3067.4332	3073.4160	3073.8183
3082.7396	3097.9916	3104.8267
3129.5331	3132.8812	3144.0973
3144.2246	3158.0973	3161.0071
3165.7625	3166.3846	3167.8449
3175.4859	3179.8829	3180.8722
3181.8062	3182.5581	3185.8062
3188.2442	3193.3927	3195.3881
3197.7384	3198.9127	3199.1849
3200.5847	3201.3553	3206.7093
3209.7391	3210.5139	3212.4690
3213.2762	3214.2373	3217.7454
3225.6930	3227.3048	3230.2610
3230.6806	3230.8015	3235.6155

TS4$_C$(SR)

-332.5211	20.4133	22.3851
27.0436	30.0798	36.6706
42.7435	48.6687	50.8178
60.8912	62.1047	69.0082
73.6979	79.3867	84.5352
93.2603	100.6553	106.8819
111.2333	114.1422	119.9305
124.0156	130.1248	133.8473
144.1450	149.4171	156.2279
166.2626	175.8378	192.7458
193.7837	207.3224	216.6304

219.5511	225.2305	227.3316
230.1662	235.4425	240.8098
248.7585	253.6755	259.3912
275.3827	282.6457	287.5736
292.8956	308.2792	329.0052
335.6576	336.6154	347.0218
358.5403	366.9781	372.5430
383.8991	391.4092	392.9303
403.6710	413.5326	418.8221
421.3537	423.7200	426.6303
431.0903	446.2171	470.5842
490.2594	495.6634	502.0413
511.9314	518.0667	523.6180
528.3156	538.8406	545.1703
560.7308	566.2131	567.8258
578.1610	585.0126	591.2699
599.4819	622.8656	627.8553
629.8124	630.3415	630.9105
639.0463	645.5143	698.3085
707.6591	713.6391	717.5508
720.7826	721.6301	726.5725
732.3476	747.7365	748.6062
758.9845	773.8076	785.4162
790.5619	799.1152	822.9636
827.7105	832.5075	833.5996
853.1606	859.8024	862.3038
865.8590	870.5368	874.6868
881.9838	885.2288	890.4018
910.1878	937.6132	957.7436
960.4311	964.7392	966.2514
973.7028	986.6733	993.7773
995.4338	1006.0639	1010.7531
1011.8934	1011.9583	1015.8538
1016.6860	1017.0945	1017.6678
1018.4020	1024.8585	1029.8220
1035.5422	1036.8843	1039.9225
1041.6767	1042.5952	1049.4434
1052.8765	1060.6146	1064.3655

1065.7621	1066.2855	1067.9071
1069.0297	1069.3220	1071.7158
1074.6223	1082.3804	1106.0961
1109.7113	1112.3617	1114.9186
1119.4742	1126.2435	1135.1375
1143.3476	1166.0255	1171.6566
1173.3574	1180.3855	1188.1632
1200.3754	1202.4648	1203.1091
1203.4146	1206.7048	1210.3033
1216.7175	1230.2538	1244.3000
1248.1669	1250.1591	1250.7048
1263.5258	1270.5031	1287.2504
1288.9764	1315.2035	1322.8796
1324.4611	1326.3518	1331.8383
1333.4522	1337.2800	1341.8364
1354.5018	1354.9612	1356.0244
1359.0266	1359.4468	1364.4653
1370.2201	1382.6596	1391.1874
1399.9477	1414.7820	1418.7137
1421.0581	1422.4803	1431.6781
1434.5899	1448.9443	1450.1436
1467.7449	1473.1521	1476.2889
1486.2880	1488.7726	1490.7538
1492.6394	1492.8767	1494.6232
1495.4724	1496.5661	1498.0659
1504.4679	1505.7432	1506.9527
1507.1313	1508.4833	1509.1168
1512.0830	1525.6621	1541.0412
1543.7152	1549.1378	1553.4268
1555.6887	1572.8471	1664.9764
1667.2147	1670.0452	1675.1377
1679.8314	1688.4485	1690.2394
1695.1218	1695.3537	1696.3348
1700.7762	1709.7599	3051.0019
3062.1680	3064.4327	3067.4039
3068.4801	3070.1179	3072.8976
3082.0754	3084.3194	3129.5245
3139.7899	3140.7711	3148.8583

3149.0385	3152.3908	3158.9867
3161.5903	3162.9153	3162.9754
3168.4913	3168.7097	3172.4984
3174.3582	3187.7358	3189.5917
3191.6888	3191.8206	3192.8628
3193.3047	3195.6219	3198.1462
3202.2117	3203.0175	3205.6102
3206.4591	3209.1686	3211.9592
3213.9489	3219.4458	3224.9457
3225.0243	3228.6377	3229.0434
3234.7626	3239.3078	3239.5755

TS4$_C$(SS)

-203.2642	13.0147	19.6847
21.0001	23.1161	29.9089
33.9773	35.7803	40.5844
44.4671	45.1426	48.8657
51.2455	57.1853	65.6969
72.3790	77.6883	83.2352
88.6065	100.9046	101.3141
103.1711	111.9130	120.9557
128.2273	137.3747	151.4915
161.0280	162.3348	172.3649
174.7972	184.7133	190.4301
199.2023	214.5868	219.1362
223.2282	234.3450	237.7743
242.8070	257.8925	272.1317
279.7496	283.9549	284.2500
303.3886	312.7226	325.4963
336.1363	342.7746	344.0998
350.9969	363.3836	373.9334
377.1197	382.5806	393.0981
411.5041	417.7391	418.5909
419.5642	423.3161	427.7730
434.5585	443.5637	458.6305
488.1974	488.5948	502.4458
508.2089	514.2587	526.1837
528.2447	534.5906	540.4809

544.1759	554.6393	572.0491
572.7944	577.7147	587.6054
591.5562	616.1904	627.3212
628.8593	629.6350	630.6700
644.6754	650.9509	655.1483
679.7425	711.2865	713.2364
718.8826	719.0647	724.0800
730.8409	737.0360	750.6578
763.6768	777.5812	784.7415
790.2778	792.0654	817.4704
824.7558	827.0950	835.2388
843.0449	854.9553	864.0984
873.1271	873.7010	876.1672
885.3718	886.7658	895.0863
920.4422	934.1256	953.3644
958.7152	962.9125	968.1149
982.3760	989.2740	994.7473
997.0502	1003.0914	1011.8593
1012.3597	1013.1620	1014.7462
1015.4378	1015.6436	1016.2307
1018.5498	1019.2821	1023.9621
1034.8365	1035.9730	1041.3475
1043.5659	1047.6402	1048.3125
1057.7264	1061.2099	1062.8670
1062.9245	1064.2989	1065.3683
1066.4389	1067.0288	1069.1964
1072.6780	1075.5947	1102.5281
1103.8538	1114.0965	1114.6904
1123.0036	1129.3304	1137.6204
1152.0891	1160.4786	1172.7808
1176.0714	1176.4580	1184.6558
1198.6547	1200.1114	1204.0723
1204.9403	1207.3735	1208.8800
1214.2885	1231.3648	1234.2728
1239.7656	1250.2358	1254.5601
1272.1836	1284.2050	1296.8184
1303.2342	1314.0582	1321.4847
1328.5294	1329.2013	1331.6499

1337.3699	1338.9321	1345.6315
1346.5577	1347.7754	1351.4407
1355.0349	1357.7272	1358.4006
1362.8281	1382.8633	1392.9198
1402.3319	1416.4530	1418.3066
1418.4939	1426.1294	1430.8601
1448.9066	1450.3007	1458.1746
1461.5987	1469.4542	1473.4670
1478.8946	1484.6426	1487.5276
1488.8003	1491.1200	1492.1365
1493.4503	1494.4245	1496.9780
1500.4381	1501.6426	1502.3868
1503.6372	1507.5509	1511.4119
1516.8987	1523.1702	1532.8800
1541.9177	1544.6038	1548.0873
1550.8432	1568.3279	1657.1085
1662.2383	1664.7383	1668.4540
1671.0025	1674.5120	1682.1316
1683.4183	1688.0323	1688.0852
1688.2030	1692.6197	3037.1413
3057.4785	3059.1779	3070.8690
3072.8885	3075.5866	3077.2012
3081.7833	3095.0450	3136.1961
3142.2639	3143.1216	3146.5585
3148.5519	3153.3889	3157.4606
3159.9803	3167.4736	3170.9079
3172.2850	3178.2278	3185.7678
3188.8168	3191.3260	3195.5392
3201.3950	3202.0873	3204.3171
3205.0526	3208.8496	3209.4785
3210.4384	3212.8004	3216.2618
3216.6885	3223.1693	3225.1742
3226.4518	3232.2297	3232.5626
3235.9447	3236.7023	3238.0784
3242.5247	3274.0584	3289.7045

TS5$_C$(RR)

-199.9944	12.6410	21.1139
29.1071	33.4858	35.1332

36.8071	42.3720	44.7461
48.1546	51.5204	62.6120
65.0039	67.3551	72.9013
79.3905	83.2699	86.7431
97.6494	107.1915	110.9714
111.5114	117.5609	121.0008
132.7587	138.5628	146.0501
162.1962	167.6041	183.9479
188.5978	196.8878	201.8409
217.0582	219.5459	227.8401
238.0495	239.6036	249.9987
258.0020	269.2362	276.8713
284.5310	288.2926	298.9498
303.0384	314.6263	321.8731
342.2147	346.9602	350.6482
352.8745	370.8538	380.2260
388.8751	400.9890	414.5338
418.8205	420.5925	423.8328
424.7153	427.2305	451.0156
458.4741	466.3420	473.2384
490.1761	496.6742	509.0604
513.3499	519.6398	527.6474
530.3547	538.6419	553.8727
558.2996	571.0825	582.5726
584.2653	588.9198	612.0077
617.6342	627.7526	630.4192
631.9099	634.3871	635.0294
641.8243	660.5864	676.0598
708.9428	713.1511	721.0722
722.0813	722.8699	726.9280
740.8795	751.2599	760.2846
764.8335	775.3997	787.3417
798.2550	819.8583	827.2356
839.4811	847.5390	851.6166
860.4209	871.0715	880.2395
882.6142	883.3404	886.2087
899.1561	912.1992	936.7354
943.0550	953.5084	961.5708

966.5255	972.4379	976.4989
978.1827	987.1328	1000.8685
1002.2507	1005.8901	1006.2025
1008.4356	1012.1740	1013.6458
1014.9130	1015.8271	1016.7124
1023.3787	1025.1910	1028.6688
1030.4728	1033.3966	1039.4969
1041.1626	1045.2335	1046.6809
1059.7997	1062.4345	1062.8289
1064.8440	1065.3681	1066.6628
1067.2740	1067.3618	1069.2474
1098.6725	1106.3681	1112.2411
1115.5404	1117.3114	1118.7880
1124.5012	1136.9460	1137.3161
1155.3444	1166.5169	1175.3983
1176.7618	1177.3495	1187.4354
1191.6127	1203.6665	1203.8830
1206.0665	1207.0080	1211.3039
1216.9188	1223.2119	1236.4386
1238.6131	1239.6079	1247.1673
1249.7022	1266.5291	1280.1500
1289.5777	1299.3932	1312.4547
1319.5850	1323.2627	1327.9364
1331.0912	1334.2373	1339.0983
1340.9416	1348.1812	1351.4724
1352.4282	1352.7201	1355.8127
1359.9829	1362.9029	1377.1874
1383.1964	1391.1055	1399.0243
1404.3649	1409.2857	1417.4358
1418.5291	1427.2133	1434.0697
1449.0799	1456.9999	1464.9236
1469.5537	1478.3422	1489.0127
1489.9664	1490.1306	1491.3799
1493.5988	1494.6494	1494.9024
1497.4075	1499.4662	1501.8829
1502.0150	1504.5537	1506.9596
1511.2710	1518.7976	1519.8950
1547.4756	1548.2741	1548.4690

1549.0868	1551.6001	1663.6380
1668.9479	1669.2138	1671.2983
1679.6696	1684.8763	1688.0177
1690.5752	1691.2981	1692.9027
1694.7192	1696.5372	3034.7059
3056.2001	3072.3038	3075.0695
3075.0900	3075.8480	3079.5546
3080.5388	3087.8847	3125.5929
3136.3970	3141.5792	3144.1289
3144.5112	3149.3854	3153.6440
3162.8539	3165.6380	3167.0134
3173.3802	3173.9606	3174.4409
3182.4774	3191.4577	3199.2503
3203.0917	3203.2376	3203.3808
3204.5065	3204.8839	3205.5556
3214.3196	3216.2326	3216.2680
3218.5461	3219.9091	3220.4860
3223.2982	3226.3747	3229.5171
3232.3682	3236.1919	3239.2010
3239.9231	3240.9266	3243.8947

TS5$_C$(RS)

-154.9057	15.2954	21.0773
25.7444	29.2318	33.2322
39.5842	43.9849	48.3302
52.4425	57.5767	62.5153
65.0413	65.5349	69.1878
79.3261	83.8469	92.6309
94.0480	103.5270	107.3315
118.3527	123.9074	133.5505
143.9593	154.5849	161.5897
171.1607	185.3679	196.1910
200.3660	204.4449	214.5292
218.8103	227.6473	233.2594
237.2806	247.3252	253.8852
259.2102	275.4480	278.6842
283.2397	290.2992	297.7611
301.9665	307.9204	322.0376

325.3918	336.5830	352.9839
356.0547	369.7058	377.7377
378.1986	395.8373	413.9051
416.9499	419.6403	420.8128
421.4597	430.3231	440.6287
470.5342	484.5227	486.4178
488.9854	495.4505	511.4904
514.5842	521.7831	523.1338
531.6914	534.8588	557.2355
558.7786	573.0214	582.9329
588.0595	592.4665	602.7323
617.0455	628.9242	630.5728
631.4212	635.6848	644.4324
652.6169	663.4327	690.8238
712.3727	716.3368	718.2670
720.8510	722.0324	723.1208
737.8177	741.6404	750.1512
763.4479	783.1690	789.6543
802.0488	808.4821	827.5355
834.6303	838.6413	853.7535
870.6207	870.9736	878.6415
883.5293	883.8401	885.8433
892.3266	909.0826	916.2017
941.2090	944.7090	953.3779
970.5802	979.3962	981.0409
983.1388	1000.9538	1005.1093
1006.2307	1006.6381	1006.9813
1011.4427	1013.3106	1014.4722
1014.7379	1015.6760	1016.5098
1022.3096	1026.0625	1027.4625
1028.1589	1033.2202	1040.3635
1041.4063	1048.7827	1054.6151
1060.8110	1062.6949	1064.5261
1064.7897	1065.9194	1069.3699
1070.3183	1071.4315	1076.5794
1092.2467	1101.8417	1109.2710
1114.4111	1119.5966	1123.6156
1125.3341	1128.3323	1138.7685

1159.8205	1172.5035	1175.0063
1176.1412	1176.8708	1188.5055
1198.7016	1202.9327	1204.6325
1205.9406	1207.2008	1213.0276
1215.0047	1221.1796	1233.7604
1236.1149	1244.0195	1246.4539
1249.5870	1266.6586	1279.8546
1285.4090	1298.7962	1319.7423
1323.3689	1327.4860	1330.1056
1331.5278	1334.7016	1336.1038
1340.8078	1348.4116	1351.6671
1352.0795	1354.0181	1357.2548
1358.0556	1368.8291	1378.5429
1391.2792	1398.8968	1401.9764
1416.4885	1419.5521	1421.2647
1422.0230	1431.4730	1433.0016
1449.1878	1458.8404	1463.2685
1473.2640	1479.0271	1487.2689
1488.8055	1493.2235	1493.3538
1495.2577	1495.4218	1495.9983
1499.4813	1499.7108	1499.8702
1501.2226	1507.1686	1510.2882
1511.8962	1520.1005	1522.9490
1547.4365	1549.1188	1550.1834
1551.6015	1555.8342	1664.2685
1665.5166	1669.4030	1671.6049
1677.0008	1685.3766	1687.7950
1689.1856	1692.9701	1693.7184
1695.9367	1712.9526	3033.5276
3053.8880	3069.7643	3069.9210
3070.5935	3078.8865	3083.4980
3089.0647	3100.9802	3105.9860
3110.8424	3140.8045	3149.1450
3150.3164	3155.5636	3157.5925
3167.6809	3169.0581	3169.2357
3174.6685	3177.6416	3182.3318
3182.8896	3189.0389	3196.9350
3204.3222	3206.1308	3206.4161

3207.3039	3207.7422	3211.4324
3213.6318	3218.4567	3220.0599
3220.8343	3221.1812	3221.9633
3227.4733	3227.5989	3231.6899
3236.3258	3238.9471	3239.6297
3241.4216	3242.6086	3280.0310

TS5$_C$(SR)

-166.2151	19.9938	26.6318
28.2545	30.0735	34.5996
45.7454	53.1369	57.2927
65.0998	69.4686	76.2817
80.5860	83.1220	96.1529
99.2780	105.7250	107.8924
113.1603	119.2090	122.2556
130.6354	133.8499	136.7356
149.7995	156.3366	165.3977
173.3843	177.8788	188.8845
199.5965	202.0426	214.2472
217.0258	225.4711	230.7604
235.0457	238.9964	244.2691
256.6548	263.0984	268.8667
275.5030	284.2145	288.4930
289.8088	311.9536	322.8365
332.2322	336.0009	343.0656
348.2184	352.8448	361.6157
372.6736	381.7794	404.3871
414.8027	417.2957	420.4588
422.8252	425.4159	436.3956
439.5423	473.2051	480.5366
489.7457	501.9893	511.8135
512.7731	524.3042	525.8654
527.1372	542.8849	558.8940
562.2836	572.9837	581.8536
582.3648	591.8866	615.3412
622.6355	628.8222	629.6431
630.4731	634.2812	642.5867
657.7850	681.8975	692.1213

699.2942	707.9020	717.0724
718.8897	721.6961	724.3133
733.4984	738.6687	754.6670
771.3266	784.1332	790.5076
808.5171	823.8384	834.9726
840.0631	855.9460	859.3862
872.0494	877.2138	879.2370
882.8674	883.4187	889.3778
892.7895	918.5037	925.9417
947.0576	950.4710	953.1393
956.1187	966.1041	982.1698
987.1847	990.6497	999.2919
1003.7865	1004.6525	1005.9893
1006.9447	1009.2518	1016.2209
1016.5223	1017.3395	1017.5651
1018.4537	1023.3598	1024.4980
1026.8982	1029.1488	1036.1104
1036.8816	1040.8277	1048.2224
1059.1408	1061.6391	1066.8389
1067.4544	1068.4559	1068.8477
1069.6573	1075.4416	1076.6009
1087.4191	1094.9251	1104.4771
1110.9189	1117.8232	1120.2814
1124.4108	1136.4444	1143.6123
1155.9664	1172.6783	1174.8406
1175.3387	1178.9423	1188.4030
1190.5533	1200.7013	1202.6632
1203.4219	1210.0090	1211.6449
1218.1886	1224.0223	1237.3933
1242.4561	1246.6263	1251.3797
1251.9440	1264.1852	1279.4183
1302.6187	1318.7508	1321.6734
1322.7671	1329.7915	1332.8819
1334.4132	1338.7287	1342.8001
1346.4622	1351.1384	1356.1777
1357.8017	1358.2593	1359.4214
1361.7798	1368.7658	1377.5264
1387.3164	1405.1242	1409.0531

1410.7482	1414.9349	1415.7780
1417.6346	1429.3001	1430.3109
1451.5835	1463.1318	1469.3456
1476.9223	1479.0444	1482.5619
1488.1830	1489.4504	1490.6798
1491.6429	1492.7301	1493.9392
1496.0215	1499.0973	1501.0785
1504.5050	1505.2465	1505.7274
1508.2299	1512.7424	1526.0361
1550.3839	1551.0844	1551.9098
1555.7095	1564.1060	1667.8232
1673.0183	1674.2171	1674.5932
1675.3586	1686.0189	1689.1607
1697.3915	1699.8210	1700.5316
1701.1206	1741.3323	3030.5045
3059.6900	3061.1308	3067.6436
3068.4196	3070.3520	3070.4479
3071.7087	3075.5725	3126.7919
3137.9524	3139.4127	3139.8159
3145.5095	3150.3666	3152.6433
3153.0655	3156.3992	3161.6001
3163.0494	3163.3966	3163.9527
3165.5321	3171.7914	3174.6171
3182.6512	3185.7842	3191.5268
3192.7769	3196.4908	3197.6947
3197.7794	3199.9294	3203.7765
3205.0569	3206.2322	3208.7978
3213.7651	3213.8017	3215.1611
3224.0804	3226.7651	3228.3605
3241.6092	3257.1385	3258.4926

TS5$_C$(SS)

-177.3416	20.6057	25.6520
27.2466	31.1914	33.0890
36.1826	41.6626	46.6213
50.9684	51.8406	60.2290
66.8343	68.1609	75.4869
81.8179	82.2353	91.5903

95.9451	103.4816	104.7524
111.9978	122.5822	130.5920
134.6867	143.5126	147.8435
161.5961	168.2043	180.1533
191.4394	199.7797	202.5011
212.5013	215.5919	223.8088
231.3968	237.2647	239.8517
245.8035	253.9610	266.0002
275.8280	285.5519	294.6975
304.0341	308.8004	320.7307
339.6291	342.0414	349.5624
352.8062	357.5794	378.3803
384.4142	395.8627	409.0892
417.6029	419.0062	420.3103
420.9397	424.3810	436.8639
445.0038	469.1661	478.7436
496.4753	503.6359	510.4552
513.2648	519.0463	528.2753
531.8208	533.2711	553.8274
562.9725	571.0939	575.2176
582.6843	587.5579	612.1094
620.7362	629.7974	629.9156
630.3776	633.3873	642.6806
646.9478	659.2509	676.4324
700.5396	710.1555	714.7175
720.2015	722.8753	723.9432
729.3869	742.0249	756.2476
774.0386	791.2964	796.3916
803.6334	813.8321	831.2476
836.7226	840.6476	849.5070
862.4569	881.2698	884.7164
884.8281	885.6782	900.1393
905.0435	916.7393	928.8517
955.6713	959.9592	962.3492
965.9732	976.6129	978.7893
982.0868	996.1497	997.8043
1001.3537	1003.2792	1005.3880
1009.6369	1011.6282	1015.5459

1016.4979	1016.6173	1017.1016
1018.5761	1022.5249	1027.3162
1031.1249	1038.8150	1039.3752
1043.2072	1044.2732	1049.6936
1061.3031	1062.6513	1063.1554
1065.3339	1066.5159	1066.9736
1067.7255	1069.0161	1076.0116
1100.0021	1104.2042	1110.4466
1114.0309	1117.1906	1120.3577
1124.2087	1140.6627	1141.8225
1149.5254	1166.3655	1174.3507
1175.5003	1178.2312	1190.4423
1197.7516	1201.5709	1202.2165
1204.7967	1207.4016	1210.1587
1212.5453	1215.3041	1237.6076
1243.5463	1244.9901	1250.1169
1252.6264	1265.8201	1279.6868
1303.6723	1308.8915	1312.2260
1319.5259	1327.4155	1330.6098
1331.8821	1336.4348	1338.6243
1341.9730	1348.2251	1350.0294
1350.9869	1351.3088	1357.4492
1358.6795	1362.1171	1381.6083
1388.5201	1396.0210	1405.5327
1410.9932	1418.0815	1418.5420
1421.4275	1429.7550	1430.6665
1447.8205	1453.7042	1463.0432
1480.0029	1480.4687	1481.3904
1488.7133	1489.5617	1490.7086
1491.7773	1493.0913	1495.0973
1495.5394	1496.2790	1498.6529
1502.2174	1502.6630	1503.0226
1505.7938	1506.5902	1519.9097
1546.4937	1547.9252	1548.7788
1550.6796	1555.5110	1660.5669
1669.5555	1669.8764	1670.0802
1674.3281	1683.1701	1683.8889
1691.0381	1691.5673	1692.3289

1692.8468	1718.2078	3025.8942
3049.6042	3066.6005	3076.0290
3077.0430	3079.3591	3081.1424
3081.6042	3085.0973	3135.4707
3139.7224	3145.6322	3147.3640
3149.3564	3151.8507	3154.1991
3160.1531	3164.0466	3166.0298
3170.4953	3173.2938	3177.9031
3178.9811	3182.3894	3196.6921
3200.1970	3202.0748	3203.3113
3204.2900	3208.1450	3211.3855
3212.9242	3217.8163	3217.9614
3218.6575	3219.8295	3221.1837
3223.4118	3225.6042	3227.1620
3236.0155	3236.3910	3236.9075
3244.0439	3252.4562	3254.8437

TS5$_D$(RR)

-381.0997	13.5601	20.1262
22.7949	27.5006	32.8697
34.1395	40.6262	44.5656
47.2961	51.7036	60.2485
64.4355	72.9223	74.7377
78.2456	85.4260	87.4238
93.3451	93.6486	105.6914
117.4759	121.2844	124.1523
135.2707	140.8691	143.9946
154.3081	163.6979	170.1196
176.6100	182.8032	190.0412
192.6332	200.2637	213.9089
219.9489	232.8186	239.5857
242.9177	248.4209	254.5285
265.0998	271.5310	278.3039
281.9254	292.9960	305.3525
320.1379	337.3885	343.4228
365.4560	379.2618	383.1124
383.2702	397.2828	409.3610
416.1016	418.8828	422.7064

426.2803	428.1825	429.0134
445.8946	461.5512	485.3991
488.3369	503.1503	505.2410
520.0491	523.1167	525.4561
530.0246	535.5241	536.1039
557.9686	561.1595	564.9575
566.1608	581.9736	586.5310
589.0227	595.4915	618.8979
627.2792	628.8909	629.2718
629.7871	644.2586	657.9556
699.9520	701.7810	709.2568
721.3660	722.9667	725.3068
727.5028	745.5032	754.9655
756.9147	778.8402	783.2042
784.6111	793.9946	819.0086
830.8473	833.2611	843.0796
853.0548	858.1977	865.4243
869.6440	878.4954	879.4561
888.2135	893.2121	904.7799
907.2442	913.1031	938.5597
948.7578	957.3388	960.0591
966.0551	980.0327	981.5051
988.4762	997.9585	999.3877
1003.0431	1005.9670	1007.7797
1009.5160	1011.3838	1014.7940
1017.3641	1018.2554	1019.7764
1022.2712	1030.2169	1034.5893
1036.1789	1038.1931	1043.4268
1047.9654	1049.4499	1056.0252
1063.7889	1065.0131	1066.5920
1068.2440	1069.0495	1070.4896
1070.9393	1074.6536	1091.3766
1101.7350	1114.8834	1119.7741
1124.4199	1127.2304	1132.6831
1137.5410	1166.8549	1172.4378
1174.3286	1180.6397	1184.0540
1197.8730	1201.4709	1205.3524
1208.8362	1210.3118	1214.6699

1219.6780	1238.0716	1240.5144
1244.1776	1255.6170	1258.8849
1261.4271	1285.7887	1290.0430
1314.0461	1317.1569	1323.1605
1327.8183	1329.3258	1335.3891
1339.2364	1344.1839	1346.9271
1354.2946	1356.3473	1357.8598
1361.3819	1365.6007	1368.4932
1374.7459	1376.4143	1386.0474
1390.3726	1409.2427	1412.3872
1419.0380	1420.9188	1428.2227
1434.9120	1437.5677	1452.1877
1460.1149	1463.1066	1466.4662
1471.7035	1484.6324	1488.9072
1491.4061	1492.7952	1495.1342
1496.0051	1496.7864	1498.0673
1498.4507	1501.5307	1502.8500
1503.5189	1512.6303	1518.3688
1520.4087	1523.2549	1547.0414
1549.6730	1551.3895	1554.1885
1555.5704	1572.3879	1650.6669
1658.6279	1666.0086	1671.3018
1674.8308	1686.2043	1688.5711
1691.8664	1694.0708	1697.6060
1697.9841	1704.0277	3052.0563
3061.5960	3062.7852	3063.6467
3067.6198	3073.0887	3073.4951
3084.4807	3131.4018	3133.6407
3137.1010	3145.2963	3148.0381
3155.1830	3163.1675	3166.3615
3167.4652	3170.1085	3170.1530
3171.4211	3171.6110	3172.8490
3189.5150	3189.5574	3189.6514
3192.0927	3193.0947	3195.2992
3196.2333	3198.7088	3199.1298
3202.1846	3202.7245	3203.5881
3209.3014	3211.5225	3212.4521
3213.8818	3223.1474	3223.6125

3232.7725	3234.2984	3236.7936
3238.7169	3248.9921	3255.2877

TS5$_D$(RS)

-347.0644	11.8864	16.0402
19.1538	24.7642	29.9216
32.9830	35.1490	45.7725
52.6687	56.1709	58.0410
64.5734	69.8829	73.3848
74.9687	80.5033	87.1777
94.2897	97.8925	105.7593
110.7527	123.7365	136.0849
139.6735	142.0813	153.1182
159.7471	169.5531	170.3515
190.9606	195.4541	197.7907
206.3363	209.6936	219.7821
220.7887	224.9041	236.3461
241.8840	249.8103	251.5841
255.1509	260.9101	268.2747
281.3277	287.4334	305.6413
310.0481	337.1163	351.8153
362.2895	374.0072	383.5309
391.1268	394.3423	408.6091
412.3466	417.2589	418.1093
418.9752	420.2312	421.6006
447.2324	450.9875	479.2976
484.2259	498.1643	507.2328
516.9507	520.7614	526.6036
529.8354	532.5482	541.3459
549.4364	554.9418	559.0512
578.1502	581.3645	582.9297
588.0971	593.1816	619.4399
628.8461	629.5777	630.0716
631.4933	644.9416	651.4232
680.3479	693.1009	702.5733
710.0354	713.9016	718.4276
723.3765	733.3040	737.6603
754.7678	759.9769	773.8044

780.7139	787.3110	793.7824
827.6242	838.1482	839.6971
853.4403	863.0239	864.6222
867.3625	868.9928	873.3173
880.9898	887.9889	892.4527
900.8480	919.6274	929.3776
941.8563	944.2934	955.4384
968.8088	981.4999	983.2602
991.5869	994.1468	997.3725
998.3218	1003.7749	1009.8758
1013.2042	1013.8469	1014.1659
1014.7540	1017.8653	1022.3688
1023.2817	1025.4215	1032.1098
1040.1850	1042.2329	1045.9597
1046.7023	1047.6857	1062.2552
1063.7015	1064.1172	1067.1025
1067.5106	1068.9670	1070.8509
1073.1851	1075.4328	1097.1953
1098.5422	1114.2322	1117.9710
1122.1145	1123.3218	1131.3033
1137.6268	1159.2368	1171.9865
1175.5556	1179.6289	1181.7427
1198.5970	1201.3863	1204.6574
1205.6604	1206.5686	1215.6516
1217.2089	1236.3460	1238.4697
1243.2159	1251.6358	1257.6332
1263.9101	1267.9221	1282.0256
1311.5039	1316.2697	1320.8222
1324.4908	1331.1676	1333.9790
1336.5004	1341.2400	1342.7532
1343.0404	1355.0508	1355.5746
1358.0562	1358.6676	1364.4275
1368.5961	1378.1630	1387.1980
1390.4614	1407.5051	1413.2533
1417.3218	1419.1715	1421.5972
1431.8531	1432.9918	1434.0801
1451.3887	1460.0609	1467.6481
1476.7917	1484.7970	1487.8502

1489.7409	1491.9829	1494.0812
1496.3727	1498.0120	1501.0572
1502.3532	1503.0394	1503.2449
1503.9092	1505.6063	1513.4486
1518.3790	1525.2652	1542.9542
1548.8171	1549.5354	1549.9741
1554.5122	1564.0606	1645.5187
1660.5469	1665.2533	1669.8363
1675.9448	1683.8744	1686.8242
1693.9924	1694.6857	1696.5531
1698.8343	1703.5938	3039.5055
3058.1265	3058.2067	3066.0987
3070.4987	3072.8087	3075.7074
3086.0711	3129.8670	3136.3233
3142.6796	3152.9086	3157.0843
3166.2215	3167.7251	3168.7550
3170.8710	3173.5776	3176.3167
3176.5540	3180.2729	3184.6408
3184.9899	3189.0396	3189.4581
3190.0252	3191.4667	3194.5779
3197.9853	3204.1945	3205.4372
3206.7313	3207.1398	3208.3093
3212.6302	3214.7832	3219.6983
3224.8971	3227.3378	3228.4738
3230.9199	3232.8147	3236.7984
3244.5109	3262.6166	3523.3951

TS5$_D$(SR)

-332.0979	11.3562	15.4026
21.5254	24.4442	27.8892
30.6358	32.7527	36.4190
38.6313	41.9607	56.3349
61.3565	67.1016	73.7550
75.4700	88.5647	90.5703
92.5322	95.9985	102.1046
104.3802	111.1085	120.0621
132.0734	134.3931	142.7753
152.1497	163.8544	184.0960

190.6190	206.2860	214.3818
217.5988	223.5630	233.8618
238.6313	243.6810	246.4267
248.7471	262.3037	268.4958
275.5394	287.5535	293.3571
297.7079	311.2005	319.0663
332.9457	337.2688	346.4109
358.2305	359.6084	378.5206
384.0858	391.1583	402.8355
413.0195	413.4647	415.9014
418.1074	423.2645	427.9585
448.8007	454.2195	465.0812
489.5460	496.6645	509.7032
514.4090	522.6743	525.9372
530.8983	536.5263	547.6690
551.5515	553.2951	559.8746
573.3644	583.1351	584.1987
591.1266	591.7278	625.7981
628.5289	630.0400	631.1731
636.4555	643.0586	644.9708
649.5498	671.3806	694.0273
709.9924	718.3364	722.0808
723.7669	733.6169	740.4752
759.3933	765.0412	775.6511
782.5937	788.5007	801.5549
824.5358	833.6400	838.7337
854.9456	860.6708	867.0569
867.6981	870.0513	879.6323
880.3804	883.1311	891.8270
897.2522	915.7404	934.9360
946.6132	951.0227	957.8003
969.7956	982.0283	984.7316
991.1418	998.7188	1002.1500
1004.5666	1005.6731	1009.1019
1012.3767	1012.7026	1013.2039
1017.0030	1019.0593	1023.7049
1026.8546	1029.4615	1030.3445
1038.5064	1042.0600	1042.7350

1046.9476	1047.1356	1061.2937
1063.8014	1066.4858	1066.9298
1068.3088	1069.2630	1071.5329
1074.3730	1075.4585	1097.6045
1108.7311	1115.0119	1115.2059
1121.4897	1125.3346	1128.3253
1138.1926	1159.0657	1174.3019
1176.2673	1177.4283	1178.3081
1199.7427	1202.1210	1205.8326
1206.3915	1208.3286	1210.1174
1222.4184	1231.3630	1238.1965
1248.1212	1251.0951	1263.6754
1267.3616	1285.9924	1292.7585
1308.6275	1313.8602	1320.3064
1325.9322	1328.3478	1330.0075
1339.4508	1343.3360	1344.1384
1350.1416	1353.9319	1357.1570
1358.0659	1360.3224	1362.1081
1363.8589	1371.9652	1382.8215
1391.7166	1408.7760	1414.2673
1417.5114	1421.3398	1421.6830
1431.3671	1435.5987	1443.0518
1451.4634	1458.5113	1468.4291
1482.0943	1487.7809	1490.2303
1491.3892	1492.6262	1495.6163
1496.6712	1499.1481	1501.8076
1502.9365	1503.6775	1504.8322
1506.5961	1514.1495	1517.3971
1523.8733	1525.9127	1540.3509
1547.5465	1551.1298	1551.7800
1553.0685	1563.2916	1629.2960
1658.8619	1667.8507	1668.9270
1678.7174	1687.1323	1688.5440
1690.8945	1693.5961	1698.2742
1699.4867	1707.8593	3048.3858
3062.9371	3069.7326	3071.6303
3072.8852	3076.0139	3079.4189
3084.1551	3133.3142	3140.3899

3142.7850	3147.5018	3155.3401
3158.8008	3162.4200	3165.1127
3166.7418	3168.8062	3172.6354
3177.0771	3186.2536	3188.0313
3188.3061	3188.8830	3189.7046
3193.7951	3197.2054	3200.5493
3204.8735	3204.8944	3205.5774
3205.8316	3208.0393	3210.2032
3211.1279	3214.5691	3216.6507
3221.6572	3224.6935	3229.2659
3234.2630	3235.2660	3235.4237
3238.4949	3238.7079	3596.8129

TS5$_D$(SS)

-352.7190	22.3135	26.4276
30.2207	32.3471	39.3884
43.0567	44.7515	48.6093
51.6623	54.5907	58.0003
61.4998	66.5753	69.4192
77.0135	86.3264	92.2115
95.7553	99.3814	106.9989
108.4580	112.8231	128.3104
134.5838	137.2620	149.2898
156.8445	162.7281	166.1743
182.4266	185.1235	197.2124
205.6378	208.3432	215.8839
223.1910	236.6416	238.3790
240.9732	246.5959	259.1310
264.2436	274.2438	278.3338
281.6155	297.3419	309.9573
318.3491	327.6647	343.9965
347.6417	364.0889	367.9895
383.7108	390.3061	402.4744
415.1432	416.1983	419.4527
424.3365	427.4948	430.9866
440.9866	447.0909	483.6727
488.7946	494.2370	498.5307
510.1646	520.1375	522.2321

525.7945	527.7543	542.5979
546.3282	552.2303	563.5490
569.3590	581.2308	583.0371
587.6585	592.6841	627.5564
628.2461	630.2734	637.0994
641.6001	643.4647	644.8287
666.3387	694.7301	701.8426
718.0197	719.4637	720.2538
732.8277	745.1980	753.3904
759.7471	770.8455	777.4594
785.9761	792.0667	802.9197
820.9637	834.0550	838.9071
843.8198	851.7341	858.7599
867.4907	874.1641	876.5697
882.7017	887.4016	888.5097
902.9836	917.5393	921.4040
937.1793	941.5522	954.6413
961.8235	980.3072	986.5703
992.5348	996.9825	1004.5006
1006.1306	1006.1640	1007.5982
1009.7719	1011.9845	1012.7849
1013.1888	1016.3065	1018.0484
1023.5065	1025.9493	1030.0403
1033.9023	1036.1007	1039.5175
1039.9068	1048.1722	1061.9580
1063.1521	1064.6395	1065.3485
1065.6755	1066.3016	1069.4071
1070.6621	1071.2929	1099.2684
1108.0176	1112.8840	1114.4173
1116.9184	1125.0397	1134.3445
1140.2663	1161.6474	1172.1977
1175.3973	1177.5145	1178.9554
1198.6912	1200.6428	1203.7369
1205.8222	1207.7020	1216.6634
1219.2853	1227.8905	1241.9499
1244.8921	1247.1057	1251.3873
1265.1642	1280.4362	1312.0549
1320.3522	1324.8974	1325.9808

1329.6138	1331.6031	1335.2201
1335.6744	1339.6210	1341.3038
1346.5702	1353.7714	1356.5850
1356.8719	1358.5618	1362.3598
1367.6948	1377.1088	1386.2905
1405.0463	1409.7309	1415.0878
1416.0948	1420.1024	1425.0085
1428.6795	1442.6314	1445.4123
1454.6780	1459.3183	1463.9391
1476.0382	1484.9265	1487.0112
1488.9871	1489.1389	1493.5616
1495.1235	1495.2214	1497.6893
1498.6635	1501.4214	1502.1858
1502.9887	1510.6806	1513.4421
1520.7872	1522.6254	1539.8769
1548.0688	1548.2839	1550.2126
1552.5534	1575.5634	1636.5250
1648.6500	1657.7892	1659.6324
1670.0411	1675.8570	1681.1991
1682.5048	1682.7503	1684.2911
1690.5375	1691.5228	3034.5026
3056.9029	3068.7009	3073.2794
3077.8783	3078.2487	3086.0658
3097.7239	3112.2917	3138.2719
3141.1092	3146.6424	3148.7605
3150.2445	3156.2595	3159.8659
3166.7618	3171.1251	3172.2275
3172.4846	3173.7459	3179.8223
3186.4697	3188.4187	3199.0897
3201.6993	3204.2492	3208.1445
3208.3971	3209.7823	3212.7841
3214.3547	3216.1071	3219.3072
3219.5714	3223.0251	3225.6883
3228.5032	3235.2995	3235.7042
3238.4982	3238.9901	3240.3807
3244.1813	3249.9625	3267.4135

TS6$_D$(RR)

-1255.2844	17.2388	22.1109
24.5452	27.0514	32.8585
34.9281	41.5699	43.4782
49.1225	51.0293	57.7522
60.6179	64.6262	67.2206
75.4522	79.3721	82.2340
95.3741	97.0504	101.6511
105.1040	110.6209	114.4659
123.5071	125.9752	142.4227
160.2774	169.6381	175.2028
197.1092	200.4085	201.8231
204.5662	208.1938	218.4480
229.9437	237.9402	248.8258
251.9757	257.8844	262.9914
275.9010	285.1957	290.1763
301.5169	304.1332	313.1708
329.9676	332.0590	353.8098
363.7664	381.4572	386.0392
389.6972	396.3302	400.2736
417.6580	419.1720	420.0627
424.2721	426.1858	432.5558
442.2707	456.8417	491.2467
491.7412	502.9962	511.5812
518.5671	529.7296	532.6101
543.1444	556.6115	565.3017
568.9738	576.3835	582.2152
588.2871	592.8405	615.3739
620.3739	631.0331	631.1479
631.7408	635.0337	645.4603
647.1844	672.1837	680.5730
712.1123	715.9388	720.4515
722.3189	724.7588	725.2994
734.4431	741.8602	757.4490
759.3862	780.3095	786.8812
793.9667	796.6770	831.2447
836.2822	853.5380	858.5091
859.7843	874.8599	879.9256
881.1585	888.1001	890.1401

891.5936	892.3417	902.4902
932.9084	941.0798	943.0990
953.2302	963.1970	967.2601
980.9917	999.3790	1001.3021
1003.1130	1004.3192	1008.3880
1012.9013	1013.2044	1015.5233
1016.6140	1018.1436	1018.5678
1019.7715	1024.1342	1028.0662
1031.0590	1038.6015	1041.5393
1044.8486	1050.3143	1050.8660
1061.8343	1064.5476	1065.5705
1066.6318	1068.0324	1069.8732
1070.8493	1071.1500	1077.0375
1081.4039	1106.8010	1110.2963
1114.5934	1125.9914	1127.3489
1131.3744	1141.4545	1148.1049
1155.0361	1159.9861	1172.0646
1174.6270	1177.9904	1183.6801
1195.4471	1203.2744	1205.6558
1207.0148	1208.7423	1211.2746
1214.8438	1217.3408	1229.2647
1244.4360	1246.3226	1250.4727
1251.9694	1262.5349	1267.4972
1283.6166	1287.1548	1299.9487
1322.2195	1324.8708	1330.1424
1338.1780	1339.0389	1344.0644
1344.5706	1347.9808	1352.3949
1359.2705	1360.1700	1363.0003
1366.9259	1368.4979	1370.6354
1383.7334	1388.7247	1419.0769
1419.5870	1422.1012	1423.4245
1434.4285	1437.2234	1448.0385
1450.0999	1463.6540	1467.3825
1482.0597	1487.0143	1488.3621
1489.1853	1491.5702	1493.6316
1495.3329	1497.1553	1497.5802
1499.0841	1501.7735	1507.4923
1508.8068	1509.6444	1512.8133

1515.7518	1520.6607	1540.0449
1551.4674	1552.9808	1554.2446
1557.0292	1577.6074	1663.2491
1665.5464	1674.4502	1677.0153
1680.3276	1690.3263	1691.8567
1693.2555	1698.4721	1701.4972
1701.9910	1708.0230	1787.2204
3046.0140	3055.1429	3058.7032
3064.3251	3071.8299	3075.4001
3081.6645	3094.2630	3100.7176
3127.6629	3133.4999	3137.1327
3142.6842	3161.4160	3162.5180
3162.7253	3166.3548	3167.5490
3172.9177	3173.6582	3176.5469
3179.7805	3189.3156	3189.6789
3192.2970	3192.8896	3197.2813
3198.2639	3198.6028	3202.7428
3204.6729	3205.1144	3207.3092
3208.0656	3209.7402	3210.0798
3211.6804	3217.2690	3218.0187
3224.6404	3227.0513	3230.5450
3230.6933	3241.6766	3264.4885

TS6$_D$(RS)

-1272.4690	15.1906	18.3567
19.2301	23.9845	29.7256
30.0916	38.6305	41.5443
43.2599	48.3832	51.5275
53.9809	58.3277	63.4625
65.5321	72.8327	75.5430
83.5759	89.7515	93.2023
102.8828	109.8097	116.6884
136.3935	142.7605	151.4734
159.1455	170.2476	177.9063
190.2168	202.3525	205.9957
210.2642	220.6381	236.8089
244.2926	248.7476	249.7994
254.1531	263.6084	274.8040

280.5028	287.2128	296.6960
302.0351	313.7726	321.5820
323.8374	340.7960	352.8733
360.4400	366.3692	379.5415
388.8742	395.7322	403.5212
419.0574	420.2795	420.7312
423.8688	430.2714	437.2498
445.3979	461.9329	485.8024
494.7110	502.6160	508.0587
517.3751	526.2503	531.0932
545.1513	552.7935	557.5898
564.8727	573.6389	579.9565
587.7489	590.0610	614.6468
625.7306	630.2085	631.1665
632.5893	636.4816	645.4004
647.0005	671.1740	674.2899
682.6576	714.7052	723.7273
724.4274	725.5784	726.3803
735.0538	740.9630	753.5407
769.0892	780.9365	786.6293
794.4388	805.3865	829.0748
830.9366	844.8858	847.2727
856.0303	864.5223	878.5390
880.1822	880.4058	882.2401
885.4624	886.4318	904.8749
934.4209	942.0428	949.1528
955.5863	960.2357	966.0714
981.5137	990.5900	1000.1902
1003.6831	1006.7828	1009.2124
1009.5476	1013.4535	1013.8606
1014.2358	1015.9250	1017.0201
1017.5732	1025.2431	1027.5698
1034.8736	1035.1291	1041.6431
1046.9611	1052.5362	1060.1576
1061.5214	1065.6914	1066.4025
1068.8873	1069.5935	1070.1992
1070.7399	1073.8525	1076.2996
1082.9007	1100.9711	1109.1934

1112.5564	1121.2356	1128.2784
1140.6639	1143.2492	1147.7088
1156.0418	1169.1233	1171.2113
1175.7316	1178.9262	1188.0778
1200.1883	1200.7716	1206.0426
1208.3676	1209.5244	1210.6920
1218.7145	1222.9616	1230.4613
1234.1827	1243.4882	1249.1636
1255.5894	1269.9504	1272.0542
1284.3630	1291.9751	1298.4123
1314.9236	1322.5316	1329.4472
1332.5107	1333.3897	1341.2973
1347.0377	1347.5826	1349.6513
1356.5632	1359.4450	1360.8609
1362.1880	1364.9056	1367.6050
1386.6909	1392.8545	1414.8926
1420.0623	1421.9736	1423.9129
1433.2356	1433.8033	1445.3755
1453.9465	1461.6372	1467.8959
1480.9051	1489.5802	1490.8215
1491.0528	1491.2646	1493.3105
1493.8171	1494.0260	1497.4789
1499.0637	1500.6372	1502.6739
1506.0869	1507.0828	1511.1769
1516.4653	1523.1342	1541.1099
1544.2855	1550.1874	1550.5686
1557.1757	1584.9586	1664.5148
1665.6268	1671.5971	1672.2741
1676.8149	1689.2555	1691.5140
1693.0254	1695.9176	1699.0932
1699.4171	1713.5358	1788.2257
3052.9872	3066.4779	3067.0769
3069.0632	3071.5575	3072.4688
3076.2051	3091.6234	3104.8700
3133.0500	3136.4658	3142.7135
3145.8828	3150.3999	3161.1817
3163.7184	3165.5853	3172.2580
3176.6258	3179.7510	3182.8281

3184.3771	3184.3834	3185.2618
3193.8048	3195.3709	3196.2459
3196.9916	3197.0879	3199.7617
3202.1047	3205.5616	3206.4102
3208.9140	3211.9225	3213.5698
3213.6341	3214.9764	3220.0214
3221.5222	3224.4021	3230.4420
3232.5370	3234.3783	3254.2726

TS6$_D$(SR)

-1218.1535	13.1786	15.6056
22.5211	25.1534	28.8095
33.8356	36.4655	39.8217
42.4775	49.1444	52.6889
54.4453	64.3265	65.4847
69.5266	74.6524	78.9610
84.2144	85.9886	90.3149
94.3194	104.4112	111.1768
113.0040	118.6168	126.6640
144.6608	154.7465	171.1396
174.0251	199.2094	202.0634
206.2600	219.0730	222.5222
232.4249	237.5086	241.3704
246.1064	248.7702	256.9174
267.2264	272.7730	281.2621
289.7459	310.4252	313.1979
319.0288	328.9594	338.4089
355.2476	359.3615	366.8977
372.4893	390.8433	408.8802
415.3484	419.8527	420.8282
422.3861	424.3508	433.7403
437.6244	454.6313	478.9506
486.5692	499.6681	510.5019
520.6410	521.6912	523.5890
529.3956	552.8784	558.3644
560.3080	569.4140	579.0996
584.1016	587.7716	600.7730
614.5412	624.5483	628.1979

629.6669	630.3967	639.4042
646.6591	651.4936	671.9642
708.0466	710.8293	715.2395
721.3927	722.8399	726.7263
730.5417	739.4214	746.7867
768.9747	783.0381	787.9218
796.8439	818.4255	824.5676
830.2217	841.0789	844.3962
859.9615	872.9125	873.3537
877.3598	878.1881	883.1774
885.0850	886.6163	900.9662
913.3381	940.5790	954.9756
955.8399	958.7401	962.5325
981.2862	985.1948	1002.7785
1004.0407	1004.8692	1006.9541
1007.1850	1011.9418	1013.2659
1014.7866	1015.6786	1016.3689
1019.8733	1025.5733	1028.5170
1032.2483	1035.5290	1038.4786
1046.5052	1051.2759	1056.3904
1058.7619	1063.2870	1064.6312
1066.0640	1066.8202	1067.8557
1068.2334	1072.2422	1072.7772
1074.1911	1104.9609	1115.2830
1117.9492	1123.2401	1129.3541
1134.0345	1139.6232	1144.6419
1153.0455	1161.1878	1175.4906
1175.9126	1176.8222	1189.3877
1197.4750	1203.3481	1204.9774
1205.3716	1206.8838	1213.3657
1216.1234	1221.6832	1229.5459
1241.2945	1242.3946	1249.2274
1253.0670	1257.1517	1268.1861
1280.6441	1282.4670	1312.7201
1323.5071	1325.5395	1327.4977
1328.7164	1334.9237	1338.0072
1341.6338	1342.3970	1345.8462
1352.7262	1355.4132	1360.3936

1361.8222	1364.9404	1370.1966
1382.5932	1387.8442	1412.4617
1414.0480	1417.6759	1420.8991
1425.8088	1427.2925	1445.6274
1446.6453	1459.7714	1464.0399
1477.0494	1480.0823	1488.4860
1488.7934	1491.0530	1491.4154
1492.2470	1492.9013	1496.7503
1500.9710	1502.4056	1503.4720
1503.8665	1505.7787	1509.3602
1522.6080	1523.5405	1540.5805
1546.1178	1547.6682	1548.2610
1551.3189	1601.7780	1663.4546
1666.5786	1666.8375	1667.9338
1672.6642	1683.6056	1688.5057
1689.3579	1690.0286	1690.2610
1692.3785	1737.6706	1820.1933
3041.3282	3059.0971	3073.8784
3075.5980	3076.7322	3077.2729
3087.0166	3089.7646	3102.6067
3108.1411	3134.7444	3137.5982
3143.9779	3146.2650	3146.6379
3148.4834	3161.2090	3169.9839
3170.2323	3176.7136	3181.1560
3181.3754	3192.7030	3196.4742
3198.1014	3202.2405	3205.6366
3205.7679	3206.3635	3207.8872
3210.2988	3213.6054	3216.2064
3218.1232	3221.5663	3224.1148
3225.5378	3226.5150	3229.2291
3231.4186	3232.9645	3235.8215
3238.4153	3241.3633	3246.0220

TS6$_D$(SS)

-1189.8408	12.4498	15.3241
23.1300	23.3728	27.3737
29.3282	33.9382	38.6555
48.9736	49.4638	55.4236

57.3264	63.8594	64.5151
71.6170	75.4985	85.8948
92.1213	97.5862	103.5564
104.9151	109.0443	114.2773
114.3824	117.2637	135.8042
145.8725	154.8369	183.3530
194.6435	200.8584	203.9645
214.4250	216.3458	220.0567
222.9231	238.8640	241.9406
243.8325	252.8556	262.9242
268.3736	275.2209	281.5956
283.3517	297.5740	309.1514
316.2214	327.4705	331.7795
357.4193	361.6544	370.7704
382.2406	387.5388	404.1613
415.4057	418.5113	421.3493
422.5585	423.3138	430.3477
447.2139	464.8607	481.9059
493.8307	502.2438	513.4429
517.1215	521.2558	526.9810
541.3914	554.7804	558.2828
568.4529	576.6808	580.2377
586.1305	589.7060	608.2293
621.2594	624.6229	626.5962
630.0972	630.2532	641.0467
646.4499	662.8635	682.4610
710.2952	715.3699	718.9551
723.2803	724.5328	725.8367
733.6869	739.0338	748.6223
780.6258	780.9117	792.0851
797.1991	815.0386	823.4821
830.7238	847.2294	847.4245
861.2236	875.1502	878.8959
880.1808	881.1437	884.6745
888.8422	894.2377	906.9614
915.4467	942.3487	948.3537
961.6194	963.4334	966.7442
982.7017	995.3852	1001.0668

1002.0325	1003.6870	1013.5880
1013.8685	1014.5359	1015.2110
1016.5396	1016.9741	1017.8109
1020.5002	1022.0347	1030.2659
1035.1984	1036.5739	1041.8366
1047.4650	1048.8316	1050.3750
1056.3926	1064.3700	1065.3092
1065.9851	1066.5758	1068.2560
1069.5802	1072.5988	1073.1293
1074.4209	1106.1304	1112.3260
1115.7444	1125.5250	1129.2462
1131.3476	1137.0485	1141.6176
1147.0285	1159.1128	1174.0116
1176.3821	1177.2919	1181.0247
1190.6452	1199.9186	1201.8651
1204.2302	1207.8415	1208.6005
1209.7941	1221.8118	1225.7460
1246.7257	1249.2797	1253.0861
1254.2727	1265.2007	1266.4682
1281.7823	1288.1321	1305.2644
1319.6635	1326.6532	1329.8355
1336.3098	1338.6369	1340.9882
1349.7997	1350.6648	1356.3486
1357.6430	1359.5845	1362.9594
1363.7981	1368.8882	1373.3069
1382.3527	1387.5659	1410.9398
1415.6528	1417.1849	1419.9869
1423.2089	1428.5957	1445.1582
1448.9971	1462.9039	1469.8745
1479.0733	1483.0085	1485.6541
1489.7300	1492.4577	1493.2082
1495.4730	1496.9079	1497.0972
1499.6485	1500.8817	1503.6346
1505.0930	1507.4935	1508.2119
1521.5277	1526.9440	1543.8548
1547.9068	1551.9809	1553.0210
1553.4376	1600.6476	1664.8783
1666.6749	1673.3642	1674.1528

1676.4080	1693.4343	1695.1295
1697.1073	1697.1486	1698.0811
1698.4514	1723.2670	1808.7006
3053.8574	3065.2387	3067.3215
3070.2496	3070.9206	3072.0983
3077.6260	3086.3675	3088.9009
3114.8519	3130.7404	3139.0759
3141.1551	3141.5765	3144.3900
3148.9386	3157.4674	3165.1953
3167.2385	3173.4102	3176.4219
3178.9326	3180.7402	3187.2950
3187.7098	3195.0707	3195.0894
3196.6373	3198.4695	3203.7415
3205.1297	3205.3656	3207.1169
3207.4537	3208.0951	3213.1761
3217.3383	3221.0642	3224.2770
3226.7538	3226.9669	3230.9254
3231.4065	3242.9429	3245.9733

$TS7_D$(RR)

-63.3639	23.0053	24.8171
31.7094	32.9233	35.8832
43.0269	47.4909	49.2115
56.8812	59.0511	59.8175
64.5996	71.9649	73.8372
78.4970	86.1569	92.2973
93.1248	103.7048	108.6688
118.0361	129.7109	139.6194
143.5012	150.1882	156.3936
172.3736	183.8389	196.7180
199.6364	203.2101	204.9847
215.6837	223.6264	232.4648
235.4698	242.3964	248.1845
256.3683	272.7577	279.4104
282.0777	297.5815	300.5982
303.6534	325.0086	333.7470
344.4235	348.5561	370.2263
385.1322	398.2832	401.8620

405.7867	417.5003	419.0490
421.6004	423.8169	426.9476
428.4100	438.1902	450.4891
456.6932	476.5791	499.3228
505.6646	512.3338	514.4704
520.9208	527.1105	530.6800
535.3979	544.5524	557.4921
559.5917	583.8464	588.2877
589.8508	592.5955	600.0353
620.0721	628.3491	630.2581
632.7092	632.8409	645.5192
648.7753	655.6327	699.0269
716.9731	721.0425	721.1736
724.6011	726.3721	733.7800
745.3608	755.2725	761.1618
763.6459	777.4222	798.5467
801.5026	828.2842	835.3517
841.5884	852.0187	865.9705
873.6708	875.8584	876.4058
882.8703	889.5697	891.2684
912.4988	915.6038	923.9082
949.1043	953.1029	958.9451
967.6375	974.0381	975.8979
985.0643	993.1620	1001.0435
1002.6105	1004.4493	1005.1434
1013.6851	1014.0325	1015.6567
1016.7846	1017.7203	1018.0718
1018.8484	1022.8500	1024.9984
1035.4335	1040.7112	1043.2681
1048.0781	1050.4662	1057.1621
1061.2297	1062.0856	1066.4856
1069.0814	1071.7178	1072.6608
1073.3113	1079.0603	1096.6111
1104.7720	1106.1150	1113.7857
1115.6097	1118.6192	1134.5630
1136.1728	1138.4159	1152.2682
1158.3828	1164.2359	1174.5546
1176.0526	1179.3053	1184.4791

1201.7298	1203.2554	1207.5669
1209.5942	1212.8243	1215.5210
1225.3032	1228.0582	1236.8921
1249.1134	1251.1849	1252.6187
1254.5191	1272.8014	1281.0338
1283.0765	1304.6799	1322.8900
1324.7058	1335.4682	1338.5974
1339.6914	1344.0500	1346.8406
1354.7734	1359.3491	1360.5102
1362.4902	1365.1650	1369.6002
1372.5164	1376.3566	1386.0144
1408.4561	1409.7247	1417.8413
1420.2433	1423.9926	1426.2784
1433.2836	1443.6735	1450.4498
1465.8134	1466.2633	1482.0146
1485.6148	1487.9428	1490.4735
1492.7107	1494.6885	1495.1915
1499.4148	1499.7311	1500.7218
1504.8779	1505.5042	1509.8600
1514.0122	1517.5509	1520.5424
1524.6308	1526.8324	1537.5126
1549.7153	1552.8736	1555.9246
1558.3882	1566.2629	1670.0101
1672.2678	1673.4910	1676.7790
1681.1187	1685.7910	1690.3490
1695.5777	1696.0318	1698.2061
1701.1929	1702.1093	3050.8886
3058.1914	3065.3942	3066.7772
3070.4815	3078.4171	3080.6816
3084.4207	3096.2729	3111.5668
3130.8284	3137.8259	3141.8590
3151.6160	3157.2842	3164.7781
3165.1241	3172.8161	3176.0366
3176.2147	3177.9849	3185.8808
3187.1827	3191.0934	3193.1428
3194.5388	3196.1613	3197.4675
3198.5507	3199.8619	3200.5800
3200.9266	3202.5525	3205.4714

3206.7243	3207.1702	3216.2827
3218.8967	3219.8246	3221.1110
3226.5802	3227.4483	3230.9961
3236.6577	3240.3497	3254.0875

TS7$_D$(RS)

-26.2269	17.8559	20.5766
24.4084	26.5049	30.8880
33.4671	36.8456	42.2598
44.7002	48.5821	53.9086
57.2015	59.5945	63.9187
66.7969	76.1141	78.7364
84.4140	94.5203	102.8071
106.2891	116.7837	128.0007
131.8101	140.8004	141.9832
150.5450	165.8567	170.8559
179.8730	187.0325	198.5741
219.2532	226.4727	232.0011
237.3737	240.2731	245.0931
252.7671	270.2790	281.5884
284.1408	287.6558	299.4861
306.0022	315.9202	326.2250
338.2283	342.2956	346.5259
378.2091	382.1033	385.3264
393.9645	403.0857	418.2012
419.5605	420.2764	420.6945
426.8878	429.4120	433.2572
441.1278	472.5201	493.5532
497.5540	502.6461	511.8612
518.4853	521.5231	528.7706
529.5626	547.5285	547.5807
563.7724	567.1873	585.0327
588.6437	597.1456	603.2285
617.6234	627.5595	628.8451
630.3765	631.7470	644.8529
648.8537	659.7748	665.2055
715.1428	720.2894	722.5337
723.0082	724.3879	729.2818

745.8548	755.5387	757.8551
760.0550	785.7460	797.6027
802.1543	821.6045	836.8908
838.8047	852.5395	861.4714
873.9780	878.9819	881.0220
882.6989	884.6430	887.4043
905.8110	915.4015	921.6958
947.4729	949.8152	954.9093
956.0530	966.4729	976.7771
981.3516	982.8926	991.0820
1002.4284	1005.7680	1007.2873
1009.1851	1014.6868	1014.7992
1015.1685	1015.9675	1018.8896
1023.5310	1024.4314	1029.7321
1032.5897	1037.8177	1045.4775
1050.8012	1056.7940	1063.1871
1064.1082	1064.8890	1064.9567
1067.1052	1067.9321	1068.1303
1072.3859	1074.9780	1092.6097
1099.9224	1106.2538	1110.3756
1115.9629	1122.8128	1129.4173
1134.2080	1137.7677	1151.0217
1156.7914	1173.1167	1176.3884
1176.5976	1177.1560	1185.0724
1194.9796	1204.0734	1205.7222
1206.3599	1206.9473	1214.4843
1216.9308	1225.2481	1242.0005
1243.1552	1244.9118	1249.6249
1253.2565	1274.8722	1282.0818
1283.5433	1301.0313	1325.1402
1326.0528	1333.0436	1334.6904
1336.6921	1337.3011	1340.2203
1342.4718	1344.3207	1355.7457
1357.4036	1358.8519	1361.3048
1365.6422	1373.7762	1381.4798
1403.4823	1410.4724	1416.3364
1419.4829	1419.6503	1421.0659
1429.2749	1437.5158	1447.2791

1461.5648	1470.1631	1474.0325
1484.8461	1487.8086	1490.9735
1492.5919	1494.4254	1495.0112
1495.4268	1495.9008	1496.6625
1499.2520	1501.8152	1503.6165
1504.7084	1509.8645	1511.2329
1519.6540	1539.0285	1543.9566
1547.3803	1548.5346	1550.8580
1551.5565	1589.7221	1661.0803
1667.6568	1668.9338	1672.7014
1680.2112	1682.9111	1687.1390
1688.5735	1690.4041	1692.5497
1694.3704	1696.0259	3049.4636
3051.8726	3070.9084	3072.1108
3074.3847	3075.4202	3075.9710
3079.3427	3095.7670	3118.9999
3137.5725	3144.4232	3145.3283
3146.7578	3146.9313	3152.7512
3167.6647	3169.7046	3171.9818
3172.4092	3175.1566	3181.2912
3181.4780	3183.9587	3196.5126
3200.7369	3202.3371	3205.2414
3206.2368	3209.9185	3210.4106
3212.0892	3212.9293	3213.6055
3216.2782	3216.8410	3219.0234
3224.0797	3224.1680	3227.8644
3232.6458	3234.6528	3237.1627
3237.9835	3239.0551	3244.8894

TS7$_D$(SR)

-56.2362	17.2699	22.9598
27.5962	28.5033	32.3761
34.1793	38.1627	43.7387
44.9253	46.6662	49.0887
55.9425	66.1685	71.2723
78.9061	86.6163	98.0474
100.3042	101.9285	103.1737
111.0750	130.7862	131.9174

137.7554	140.6069	159.8470
161.5157	168.7019	193.5748
198.4198	201.9319	207.4637
224.8464	228.9189	233.7988
236.3993	240.7337	246.8168
259.0844	262.2157	267.1366
284.9756	299.9202	305.1412
321.6352	337.5536	340.6331
344.0288	360.0065	376.7528
383.5553	391.7309	397.7540
398.8660	404.8970	413.1546
415.2007	416.1646	421.6618
432.9466	438.3784	446.7576
450.0912	471.9011	487.0152
493.2224	501.7396	510.8900
525.3503	527.0317	529.0697
538.1480	548.5989	554.4568
559.7196	569.4111	582.1231
586.9440	591.9124	605.7499
624.7907	629.2436	630.3867
634.3567	639.2333	645.9790
652.3980	658.1148	672.6826
712.1855	721.2338	722.3006
725.0000	727.1973	734.9008
737.4283	749.6353	760.3392
761.6183	789.1768	792.3582
799.7003	823.5228	829.1940
854.4867	861.8033	870.6566
871.9202	880.0527	885.7900
888.2119	894.3664	895.9825
899.7668	906.0488	919.4177
952.8534	956.4635	960.7483
969.9110	974.2957	979.8735
996.9377	999.2704	1003.0316
1003.8472	1008.8411	1012.8625
1013.0588	1014.9648	1016.3836
1016.9005	1019.1112	1019.6830
1028.2406	1030.7001	1033.0023

1040.7789	1048.3176	1051.3667
1055.2512	1057.1235	1059.5394
1061.8529	1064.6423	1065.4422
1066.8872	1068.9764	1069.9808
1074.1521	1078.7298	1093.9777
1104.0080	1108.0256	1118.7628
1124.0192	1126.3900	1131.1974
1134.2887	1144.4598	1150.5330
1165.9287	1178.1865	1179.5398
1180.7998	1187.7010	1189.0707
1198.2200	1205.6008	1208.0225
1210.8414	1212.1053	1222.7309
1224.7123	1226.4828	1234.8337
1244.8262	1251.5190	1255.0454
1263.6961	1269.0458	1279.5404
1283.2752	1306.1045	1324.2862
1328.9602	1330.0391	1337.4110
1340.7253	1343.5562	1347.1842
1348.4914	1353.2929	1355.4413
1361.6184	1361.7097	1371.0353
1376.8514	1377.5517	1389.6131
1402.9933	1412.4153	1418.0897
1421.3700	1421.5889	1425.1739
1434.3171	1435.7472	1451.4764
1453.3503	1465.7252	1476.7948
1483.5365	1484.4685	1487.1764
1490.8749	1493.6292	1493.7898
1495.8169	1497.4056	1499.6220
1501.7617	1502.2547	1503.6625
1506.3878	1513.0181	1514.1953
1520.5128	1539.1142	1542.3335
1552.7512	1554.0365	1554.6680
1560.0816	1578.0168	1664.8330
1675.1170	1676.2975	1677.0995
1679.9254	1689.6808	1695.9482
1696.4833	1697.0036	1697.2859
1698.5374	1706.6126	3049.5901
3066.3965	3067.1853	3070.2743

3071.9669	3079.3371	3084.1867
3091.4294	3121.2742	3131.9157
3134.8666	3137.6753	3145.6834
3152.1847	3152.7133	3156.2716
3158.9185	3162.2543	3177.2569
3180.9887	3181.7566	3185.3136
3194.3924	3195.2390	3195.9100
3197.2296	3197.5033	3198.1944
3199.7804	3200.0678	3202.6283
3204.7011	3208.7574	3212.9772
3213.5925	3214.4727	3216.5997
3219.8788	3223.7706	3229.3513
3231.6106	3231.8917	3237.9028
3239.5696	3242.0591	3249.8104

TS7$_D$(SS)

-51.4485	16.4677	21.1786
22.5435	28.7809	31.4609
39.7553	44.6915	47.1321
48.5648	59.5801	61.5444
65.9894	70.8522	73.5014
81.5830	95.3061	100.5005
109.4319	110.3991	119.7552
122.1753	123.5576	130.6740
143.9055	147.4481	161.1002
166.4559	173.4122	186.4917
193.4532	204.8693	207.8789
213.5411	225.2383	230.5198
238.2357	242.6594	245.5116
258.5807	261.0508	266.0293
276.2570	288.5004	297.8385
304.7170	319.8554	332.1376
341.4573	351.7693	370.5750
374.4938	384.5214	385.2593
399.9488	403.7186	412.9570
415.2477	421.0639	421.7572
432.0502	435.4940	441.9037
446.6483	484.0213	491.1148

498.9210	510.9774	512.6945
523.7238	529.9644	533.7746
540.1089	543.8452	556.7287
560.8890	575.0182	578.9309
585.1711	590.6778	601.0169
612.8159	629.1996	629.7541
632.8386	636.3871	646.5583
649.6151	664.9010	675.2658
713.1789	719.6070	720.6267
723.0492	724.7330	734.5430
735.7614	745.3053	756.5232
763.4564	779.4480	787.7223
800.9711	821.7777	828.7830
850.8251	861.2208	869.0877
875.0950	879.5580	881.3260
883.3110	885.2107	887.3700
900.5569	916.4762	929.0320
950.7634	951.4018	956.0087
969.0072	976.7157	993.2500
994.8377	998.4965	1002.6268
1004.6937	1007.3767	1015.2465
1015.9415	1016.4154	1017.8575
1018.1800	1018.7477	1023.0858
1025.6439	1028.0701	1028.9590
1039.4355	1044.3796	1048.5908
1050.9029	1052.8829	1055.1413
1063.0723	1065.7870	1066.8554
1068.1396	1069.9724	1070.5226
1071.3357	1077.6001	1095.8074
1099.4090	1109.4124	1113.0078
1123.3835	1126.9077	1132.4279
1133.4566	1143.6506	1153.3627
1162.5507	1173.2711	1175.1466
1176.5927	1178.7449	1187.9233
1199.8348	1204.3524	1207.4426
1208.8648	1211.4090	1212.7501
1221.6443	1227.2654	1234.6740
1241.6829	1251.3009	1254.4249

1269.3652	1271.1696	1274.4294
1281.5973	1296.7353	1322.2008
1326.8584	1330.1812	1334.5376
1335.4720	1336.6816	1343.8302
1346.5817	1350.9290	1360.3446
1362.0683	1363.7844	1365.4760
1365.9762	1372.3809	1391.1817
1399.0615	1407.8803	1412.4425
1420.0812	1420.1010	1425.7813
1431.9781	1435.5917	1447.7082
1450.9199	1468.9469	1470.0698
1474.0114	1477.6840	1490.4005
1491.9399	1492.8254	1493.2129
1496.7644	1497.4951	1497.9532
1504.1878	1504.9669	1505.5939
1506.1894	1509.8609	1513.2253
1519.1270	1519.5790	1535.5128
1548.7158	1553.3300	1555.1843
1555.9194	1570.6958	1667.0234
1671.5144	1676.3351	1676.9637
1683.5550	1688.4895	1696.2426
1696.3129	1697.9937	1699.4588
1700.1145	1719.9729	3055.0886
3064.2918	3070.3247	3071.2512
3076.4667	3077.9535	3082.3577
3084.1210	3102.8769	3110.3844
3138.6405	3138.8131	3146.8946
3148.4176	3162.4635	3165.0613
3166.9430	3168.1400	3171.9783
3175.5882	3175.6280	3177.0932
3177.3099	3181.4136	3182.3863
3189.0644	3190.5156	3190.7846
3193.3989	3194.1769	3196.7199
3196.8574	3197.1631	3200.5173
3205.2006	3209.0190	3212.6051
3213.0536	3215.8501	3221.5419
3225.3285	3226.5194	3228.2188
3234.0436	3254.3304	3266.9870

$TS8_D$(RR)

-163.4647	16.3653	24.2718
30.1323	38.5469	42.3430
44.2028	49.4892	54.4070
58.2019	63.9545	76.8571
82.1293	90.1379	97.6206
104.5812	106.0781	109.5415
113.1852	117.9021	133.5321
134.7586	142.8280	145.7587
155.4742	162.9256	170.8472
176.4558	181.7505	184.9965
200.6171	205.8478	216.4718
220.5054	226.1164	237.0177
238.5065	252.2271	256.9123
264.1652	268.7313	282.2356
288.1369	295.1157	300.7968
307.3592	319.0841	338.2094
342.9945	356.3880	358.2523
366.2535	377.8235	388.6844
394.9677	405.1060	418.1022
419.6022	420.7893	423.4315
438.5292	440.1937	447.0565
466.6246	485.0304	492.2391
498.3164	506.0677	518.4548
528.5314	531.1001	532.6397
538.9730	550.2099	555.1316
571.1485	576.7192	584.3723
590.5382	600.3249	614.1118
629.6778	629.9011	634.5148
636.2525	642.7683	644.2912
658.6335	665.6026	684.2602
707.9958	715.9082	717.3332
722.9976	726.3224	729.4579
734.2512	746.5882	759.3070
780.4108	792.6627	793.0977
808.8196	811.6756	832.0452
834.4663	851.9449	863.9406

865.7163	878.5175	879.6311
888.4514	890.6119	894.6249
919.5085	923.4490	935.7876
950.4440	958.8423	960.8187
968.3330	970.8164	976.6899
978.4612	995.1010	997.8538
1000.5479	1001.2631	1008.9995
1012.0044	1012.9744	1016.7897
1017.0462	1018.0344	1020.9674
1021.4606	1025.8765	1027.1710
1034.9769	1038.6347	1041.7774
1048.7774	1051.0872	1055.8533
1063.9923	1065.7016	1067.6243
1067.7276	1069.3866	1070.8971
1074.4608	1081.7318	1084.5058
1102.5461	1104.5415	1110.2413
1111.2147	1118.8618	1123.6082
1124.6227	1128.3289	1134.5411
1150.7991	1171.6262	1171.8865
1179.4552	1187.7023	1190.4108
1195.8917	1201.9697	1203.5347
1211.3567	1214.3740	1216.2768
1224.2332	1224.9645	1239.5199
1244.7577	1247.8444	1251.7277
1267.5283	1270.0210	1283.8816
1288.2310	1300.4779	1318.1869
1322.4826	1328.3807	1332.7184
1336.2046	1338.4797	1343.2461
1345.9203	1353.3635	1360.1669
1361.0279	1364.4708	1365.4869
1368.3525	1371.7696	1385.2145
1388.3606	1396.5702	1408.1783
1415.7410	1416.5784	1420.3798
1420.5914	1430.9567	1440.1891
1451.8714	1453.5591	1467.4199
1476.4362	1479.8729	1485.2078
1491.9096	1492.8271	1496.9025
1497.2177	1498.1613	1499.3269

1500.1117	1501.3384	1502.7395
1503.2245	1510.0589	1510.5166
1512.4889	1514.2760	1521.6156
1549.4524	1551.7333	1555.2934
1557.4377	1560.0079	1613.2377
1673.2249	1675.5731	1675.6045
1677.4141	1685.1981	1690.1893
1692.9093	1698.4887	1698.9417
1700.0477	1702.1738	3031.7648
3062.2652	3068.9994	3069.3880
3069.5951	3072.3512	3076.3621
3082.4941	3089.3341	3115.0759
3139.2709	3142.2686	3143.3513
3155.9515	3164.3784	3166.1747
3167.2642	3168.1564	3170.9454
3173.7878	3184.5300	3185.4488
3187.7435	3189.3217	3189.9645
3191.7640	3193.1123	3193.4309
3196.4033	3199.1983	3200.3404
3201.4181	3203.0678	3204.0075
3212.0095	3213.0726	3216.6023
3219.5572	3221.2687	3226.1294
3226.3751	3232.9779	3237.9435
3240.4427	3250.2380	3255.0917

TS8$_D$(RS)

-178.2907	13.0139	22.3509
25.1387	26.0513	32.4694
36.8535	40.2831	43.7731
47.9558	50.6510	59.9024
64.7463	69.1296	76.9787
88.5411	90.6129	92.6007
96.4699	100.9479	108.9258
119.5831	124.6132	130.7414
137.9353	148.5495	156.9883
170.7195	179.7161	181.7451
196.8454	205.9627	216.3335
220.8953	222.9788	235.5223

241.3325	245.1240	250.2384
256.0120	265.7072	281.1029
283.2754	295.1039	309.4681
324.0216	328.1431	334.7139
339.1839	340.6025	349.6801
367.5067	380.3031	390.4338
401.2467	412.2651	416.1399
417.1324	420.1453	422.3518
427.9741	432.5915	439.8846
453.8860	478.7032	483.6389
487.9644	503.9089	509.9401
516.1625	526.8838	528.3048
532.8829	543.6914	554.5873
567.8838	576.4682	581.6593
589.2803	594.0137	617.0577
626.8148	628.3836	630.7486
632.3685	640.1480	644.6147
647.3558	658.0553	668.1910
700.9271	713.3841	716.6029
717.1795	719.5990	723.0934
724.9227	743.2003	759.5856
764.5157	791.3932	793.6724
810.5870	813.2540	830.5097
841.7188	851.8723	855.1714
868.2974	872.1447	878.0784
883.3203	886.0203	893.3468
911.1145	915.0034	941.0294
956.3270	957.9302	964.8174
968.2927	978.4684	986.8646
994.5446	998.1283	1002.8213
1005.4851	1008.3200	1009.0067
1013.6642	1014.8286	1017.2066
1017.6594	1018.0471	1020.8386
1021.9484	1028.1227	1031.5344
1034.4276	1042.1962	1044.6362
1045.7277	1051.4440	1059.2332
1064.3437	1064.4480	1066.4588
1067.1075	1068.9829	1070.5930

1070.7059	1074.7148	1087.9979
1094.0406	1106.0239	1114.3707
1115.3135	1120.2097	1124.5876
1125.1262	1126.9460	1154.0276
1156.9989	1173.3657	1176.5323
1177.7012	1180.0843	1192.6874
1195.9036	1203.8864	1206.1608
1209.4328	1216.8467	1217.8716
1221.0856	1224.3268	1240.6746
1246.4791	1249.6841	1258.6810
1264.4718	1277.1296	1280.2290
1285.2978	1308.8393	1327.8649
1330.3505	1336.2568	1337.9930
1339.6429	1343.6072	1345.3702
1347.3134	1352.9715	1355.1734
1358.8516	1361.3534	1362.3833
1364.1814	1372.4956	1383.2728
1387.3765	1401.9131	1407.4590
1417.2256	1421.2786	1421.6851
1423.6958	1433.2957	1438.0275
1458.6165	1460.7901	1466.5133
1478.5934	1481.9336	1486.7896
1487.3761	1490.4161	1494.8737
1496.0661	1496.8671	1497.2672
1497.4379	1501.9030	1504.3020
1505.5755	1505.6692	1506.1804
1509.8145	1517.1442	1521.7836
1553.0548	1553.5478	1556.6946
1557.6327	1566.1985	1666.9869
1674.6223	1674.9021	1676.0945
1681.7802	1682.5074	1687.2715
1695.6292	1696.3970	1698.6272
1700.5966	1701.4827	3025.1195
3056.8619	3070.5610	3071.5555
3073.3470	3074.1441	3074.6511
3084.7166	3123.9327	3126.6242
3140.4032	3142.1431	3144.7193
3148.8681	3149.6782	3159.4078

3166.2161	3167.8846	3169.6314
3172.0268	3172.2889	3173.4463
3181.6317	3185.3408	3186.1020
3188.0733	3190.0582	3190.9130
3191.6035	3192.2361	3201.3934
3201.5410	3206.0821	3206.2302
3208.1129	3212.3780	3213.5659
3213.9664	3220.6156	3221.5366
3222.2534	3232.0472	3235.7830
3249.3105	3253.9123	3254.2972

$TS8_D$(SR)

-182.3765	16.8797	19.5766
23.6284	26.8590	30.0751
34.3782	42.7429	47.0715
47.9047	56.9878	62.6918
66.6612	75.4184	76.6909
79.6476	85.8292	88.0541
91.3787	96.0996	103.6693
120.2373	123.6562	128.2732
143.0432	147.5447	156.3507
157.8253	167.4636	180.6850
186.8962	191.5452	212.1075
213.7099	219.8524	230.0095
235.5979	240.2493	247.7115
251.5268	267.5987	279.9751
280.7131	294.4551	305.8100
312.7375	320.6567	325.9074
335.4889	338.3109	346.5153
362.5409	364.4000	368.4157
404.5966	407.8653	415.2065
421.1810	422.2358	424.3524
424.8202	432.0316	450.1451
456.9328	479.9488	484.1011
491.3942	505.2012	510.4520
513.5233	523.8086	524.6488
527.4228	541.8815	561.5766
568.6723	572.6974	589.1541

594.3954	610.0807	627.5924
629.1305	631.7547	637.4709
638.0953	643.2944	643.7964
647.5037	654.7851	665.0320
699.1653	708.4157	720.8591
722.1129	723.8878	727.0243
728.2887	756.5701	758.7129
768.5157	779.7371	796.3080
807.5566	817.1713	827.0837
840.9332	850.1043	853.0476
869.0967	870.6308	881.6859
883.9018	887.8267	893.3341
913.5758	913.8293	946.0170
949.6131	955.9220	959.8092
962.7839	966.3104	980.1374
983.4949	990.5496	1000.7672
1001.2531	1005.3875	1007.4429
1012.9467	1015.1535	1017.2015
1018.5025	1020.3081	1020.8650
1022.3288	1029.8075	1031.2841
1033.9941	1035.0852	1035.6723
1043.4075	1044.7656	1059.8224
1061.4344	1062.0272	1064.1184
1067.1734	1068.2511	1070.1436
1071.6037	1072.6243	1086.4041
1088.6165	1101.6233	1108.8802
1117.6520	1121.3491	1125.4927
1126.3874	1129.7798	1149.2159
1157.1464	1172.9303	1175.0147
1180.4659	1186.5791	1187.5736
1191.7337	1204.2083	1205.2011
1207.1143	1211.1091	1215.9583
1218.1950	1220.9080	1242.4434
1247.8083	1248.9048	1254.4315
1264.2151	1269.5389	1280.2922
1282.7891	1290.6565	1310.1589
1329.4967	1336.4755	1337.6032
1339.0107	1342.0738	1346.1458

1346.6403	1356.4991	1357.8863
1360.5854	1362.4017	1364.0135
1366.5952	1376.0215	1379.3963
1385.2042	1400.2992	1414.8791
1415.1286	1418.2400	1418.8699
1422.2545	1425.1977	1430.5260
1456.6447	1464.8534	1470.0209
1474.0062	1476.5284	1487.8881
1488.9288	1491.9890	1492.4205
1497.8802	1498.8878	1500.4777
1501.1608	1502.1562	1504.4140
1504.5826	1509.1270	1510.3128
1520.3472	1522.6260	1525.1061
1552.0859	1553.2368	1555.3785
1558.8915	1570.3638	1671.4057
1675.2169	1676.2319	1678.2215
1681.0325	1690.9893	1692.3433
1697.4391	1699.4610	1700.2557
1701.3265	1708.0561	3057.5171
3064.9069	3067.8783	3068.7104
3071.0180	3075.2796	3076.6398
3084.7532	3106.6093	3120.6548
3120.7539	3137.1792	3138.4838
3139.9095	3146.7546	3158.6572
3162.6225	3163.1808	3163.7335
3166.8607	3169.0823	3173.2224
3175.0040	3186.1893	3186.8044
3188.2667	3191.4116	3193.7613
3194.5264	3196.8434	3199.1725
3199.8445	3201.9329	3204.0719
3206.3174	3208.2838	3211.9242
3214.6568	3218.3396	3220.0039
3223.0513	3224.1647	3225.4159
3232.4620	3237.0049	3238.5001

TS8$_D$(SS)

-171.5459	9.4231	18.7781
24.0903	28.6721	33.0044

38.1632	40.2419	46.3046
48.6698	51.3292	55.5713
56.3535	67.0080	73.4551
80.1250	82.3577	87.5912
99.3547	105.3002	110.3787
120.0311	121.7702	127.4755
133.7997	152.4631	158.9540
167.0577	169.1333	188.1380
197.8109	208.9142	209.8299
214.5783	222.5795	229.3115
235.8327	241.8693	252.5434
267.5530	274.2330	277.7661
282.7708	290.1385	308.8827
314.3503	318.3903	322.5999
337.0096	344.0850	346.5183
350.7818	361.7947	367.9540
377.8429	392.9894	413.9609
414.7807	420.6026	428.6019
435.1500	442.5845	448.1284
463.6030	478.2426	484.7732
496.7826	506.4122	511.1109
517.8633	522.5787	525.5604
537.9910	552.5237	557.6739
562.4607	573.9003	587.4084
591.5017	595.0327	618.7780
622.0748	626.3370	629.5865
631.0551	634.1282	644.1263
657.6854	663.5216	675.7691
696.6672	715.2543	716.6063
718.7192	725.9003	730.8243
734.0959	752.7884	759.1454
774.8369	785.2713	794.5203
801.1846	817.7506	830.5762
836.4525	844.5474	858.3048
864.4226	874.1220	885.3253
885.7865	891.9017	908.9128
915.1687	922.8859	939.0582
945.1606	955.2073	967.9921

968.2749	973.1022	979.3149
981.8472	984.3600	997.4512
999.0063	999.1701	1009.7382
1012.3034	1014.8581	1014.9626
1015.6302	1017.6531	1020.0037
1021.5433	1024.8072	1028.6404
1032.7560	1036.0773	1040.7126
1047.4834	1051.0218	1055.3069
1060.4797	1061.3127	1065.0880
1067.1331	1067.6277	1068.0897
1071.1933	1080.3463	1085.9796
1094.8163	1095.2180	1104.6031
1116.0733	1120.1713	1126.0564
1128.9246	1131.6222	1142.5478
1156.0583	1173.4344	1174.5036
1175.5944	1177.7757	1190.5344
1194.2440	1201.4506	1205.1440
1205.8868	1209.4072	1217.9567
1219.4618	1221.5688	1232.3422
1242.0679	1244.5733	1254.1724
1259.5841	1269.0998	1274.7386
1280.5073	1299.5684	1315.0862
1330.9962	1331.4951	1335.4341
1338.6723	1339.1290	1340.9305
1347.1155	1348.6866	1353.8591
1356.8646	1358.3295	1361.2329
1364.5226	1364.8833	1385.7645
1391.0115	1399.7116	1408.7289
1414.9741	1417.0087	1418.9592
1424.4105	1426.1029	1433.0265
1451.3510	1458.4794	1467.2236
1470.7002	1487.6163	1488.9747
1489.3203	1492.0318	1493.8772
1494.0519	1496.5311	1498.4652
1499.3137	1500.4133	1501.8384
1504.7355	1506.4682	1509.4960
1514.7543	1516.1492	1521.6715
1548.0565	1549.4525	1552.2114

1555.8029	1557.9106	1667.9702
1668.5634	1674.9736	1676.5330
1685.2429	1689.0751	1690.8505
1692.9912	1696.2246	1698.2903
1699.6359	1705.4176	3046.6724
3056.6951	3067.8683	3070.6399
3072.9972	3074.1050	3074.9995
3076.6859	3079.0989	3118.5144
3121.4911	3135.0514	3146.5345
3149.6471	3149.8110	3156.1157
3158.4552	3164.1595	3164.7274
3170.4220	3184.4749	3187.2802
3188.4535	3189.3124	3189.4091
3191.0632	3194.1496	3196.7445
3198.0397	3199.3812	3199.6855
3201.1657	3205.4714	3206.2520
3209.2940	3210.6818	3212.9385
3214.4322	3222.2810	3222.9186
3227.9638	3228.2453	3229.9354
3233.6852	3239.9576	3247.6044

TS6(R)$^{HOBt}{}_{L3}$

-307.5145	6.0350	10.9840
14.8474	18.2844	20.6309
23.2937	25.9028	28.1972
30.8662	33.0902	36.1621
38.4612	41.9702	42.6918
43.2816	45.3152	50.2288
50.9794	52.0480	54.8055
58.3534	64.1360	67.9373
70.3511	71.0801	85.1648
89.9160	96.6291	102.3182
113.8200	124.4130	130.9968
133.7348	137.9585	143.2823
148.5850	166.0955	185.2702
188.3730	200.6500	206.1833
215.6504	220.6460	227.8147
232.1262	235.3737	242.7245

254.8265	258.6344	264.5586
269.0631	281.7005	283.4675
301.2187	313.0736	313.7162
318.2074	327.7831	332.7597
350.8408	360.7738	371.1302
379.4648	390.0286	408.4614
410.7464	411.6948	416.6582
416.9790	419.8860	422.1376
434.1531	439.3217	460.2469
476.0346	484.7033	490.7951
508.9417	510.7792	516.6852
521.8793	529.4429	531.1860
536.0315	537.5757	546.8700
553.8794	557.5408	571.6584
576.6707	579.5009	585.4770
591.0049	602.9596	622.8441
627.8750	629.1721	631.5892
632.3726	634.3754	646.5863
651.1781	659.6051	672.4406
686.2327	705.1453	708.4343
712.3114	714.0416	716.5848
725.0106	734.2710	749.0383
753.1329	755.4618	762.0849
771.0887	775.5077	777.6858
779.8876	784.0909	798.8265
802.3638	802.7667	814.8278
830.0616	831.9932	833.6423
843.8540	852.3075	860.3847
862.6067	862.9621	869.1192
869.5760	878.1303	895.2912
903.7263	908.2974	925.8542
928.8973	933.9783	935.5476
942.0503	954.7414	957.6855
968.4612	974.5544	976.2544
977.6630	980.2022	983.3308
984.5599	992.0950	995.4669
1003.7616	1004.1556	1006.8000
1012.7798	1013.0276	1013.7624

1014.8525	1017.5205	1021.0518
1032.9814	1037.6756	1038.4574
1041.6794	1041.8851	1048.6688
1051.9827	1054.1953	1054.9715
1056.3963	1061.8290	1063.4872
1063.6827	1065.5757	1071.5427
1083.3358	1083.4364	1101.2601
1107.9861	1112.0954	1113.6143
1117.9787	1120.2626	1138.3653
1138.7870	1141.5110	1152.1396
1158.6929	1170.3082	1182.4037
1182.5732	1183.9697	1189.2722
1191.3085	1194.9558	1204.0739
1206.1037	1206.6146	1209.6962
1211.1612	1219.4697	1223.2332
1230.7909	1234.8149	1263.5605
1270.8519	1278.2665	1289.0885
1295.6451	1316.9375	1319.3142
1326.8440	1327.9422	1330.5323
1332.1487	1333.1367	1335.8405
1338.4642	1338.8248	1348.1211
1358.5490	1359.7496	1362.6573
1364.8036	1366.9674	1370.7646
1375.4485	1383.5115	1410.4492
1411.4564	1421.5224	1422.4100
1426.1058	1427.3470	1428.8937
1432.9031	1437.7195	1438.3319
1450.4479	1461.5582	1470.9286
1483.2992	1483.8454	1483.9675
1488.8441	1491.7182	1492.5636
1493.5026	1493.7819	1493.8737
1497.7733	1499.6202	1503.6575
1506.4213	1510.8415	1513.3285
1520.3444	1527.8321	1531.3009
1532.8543	1534.6798	1537.8105
1539.8685	1566.6906	1573.4790
1618.7549	1623.3765	1627.6715
1630.7176	1636.5225	1636.5887

1637.1888	1639.5040	1650.4256
1651.6337	1653.7627	1654.0260
1658.3293	1669.9991	3009.0621
3015.0755	3042.6181	3043.2023
3050.1604	3060.2124	3061.4790
3103.1867	3104.8434	3108.8837
3110.3532	3113.9550	3125.4570
3132.7113	3133.0811	3140.1348
3141.0663	3141.4054	3145.3773
3146.6818	3174.6773	3178.8894
3182.2228	3184.2514	3185.2868
3185.9514	3186.0962	3188.0030
3188.3542	3188.9990	3191.7129
3193.4594	3193.9126	3193.9250
3195.8501	3200.5891	3202.6178
3203.6041	3205.7167	3210.0053
3210.9601	3212.9357	3217.1147
3218.1959	3219.1585	3223.3707
3229.7169	3254.0975	3299.4111

TS6(S)$^{HOBt}{}_{L3}$

-266.0891	1.4224	12.0235
13.9351	16.2398	19.1965
21.9826	24.0683	26.1991
27.1413	30.2694	33.1836
37.6420	41.6683	41.9591
43.5208	44.3176	47.2719
50.6601	55.1812	57.9289
58.7034	64.1855	69.4830
74.4756	76.8872	92.5743
100.5775	104.7928	110.1795
119.0060	122.8654	125.4355
142.0378	148.0890	150.7971
153.2450	164.8991	187.5252
188.2561	191.7145	197.5039
199.7792	225.7610	229.0763
232.2556	234.2288	245.6612
256.2850	264.0446	275.3088

278.8496	283.9194	288.6604
291.1161	301.6559	319.9368
322.7721	332.3120	339.4034
346.8407	360.5141	363.1713
367.2625	384.8357	393.0169
414.7063	416.2286	416.8588
421.1170	422.8643	424.7047
440.3391	456.9360	462.8920
473.4534	481.2625	488.3747
504.6986	510.2827	511.0452
514.7171	523.6008	528.5496
530.7256	537.3226	549.7123
550.4418	553.3112	571.8254
574.5847	577.7675	586.4101
601.7094	601.8828	625.3912
626.8798	631.9415	632.2284
634.2861	645.0350	645.6967
650.0418	660.7099	676.1526
680.3359	682.3818	705.1015
711.7048	712.1892	718.2869
720.2224	726.1486	746.7119
750.3447	752.7479	755.3771
765.0823	766.8095	771.4010
773.1385	777.8770	782.2226
799.8794	800.7707	802.1150
813.7671	819.9650	827.8881
836.7583	838.3984	856.8014
860.6807	864.8968	869.3516
869.6359	876.2965	890.3705
896.1883	905.4186	906.2668
928.2784	931.4613	939.5298
943.0143	955.1791	958.3159
958.5001	972.8133	973.6249
977.5991	984.4686	984.7258
986.2702	986.7978	995.5399
1001.5269	1003.3560	1005.7508
1008.4277	1012.4734	1013.2579
1013.8950	1016.5544	1018.9919

1020.9473	1025.6445	1031.2163
1037.4078	1040.2090	1041.4488
1042.9400	1052.8141	1054.4731
1056.6291	1061.2004	1063.7426
1064.0712	1066.6841	1073.7373
1079.2368	1089.4509	1102.0087
1108.1031	1109.5619	1112.8678
1114.2486	1114.8122	1129.4072
1137.9774	1142.9245	1155.0651
1158.2199	1170.0295	1181.7992
1182.7457	1183.2092	1184.4097
1190.8982	1193.5566	1204.3609
1205.1871	1206.6976	1209.3069
1210.8665	1220.5738	1224.0148
1235.0575	1241.9674	1248.1770
1275.0026	1278.9122	1282.9748
1287.3755	1300.2969	1315.2650
1321.3804	1328.7485	1331.3724
1332.0473	1334.9328	1338.0422
1339.5326	1346.5301	1346.8732
1358.4153	1359.1891	1364.4811
1365.4718	1368.2178	1370.5894
1376.4999	1392.9375	1408.3262
1409.1657	1420.6420	1421.8952
1424.1424	1424.5889	1425.0682
1429.9906	1434.3519	1436.8446
1449.5002	1452.9737	1472.7616
1483.4921	1484.6204	1487.5704
1489.8953	1490.3091	1492.7590
1493.2548	1494.3038	1494.5982
1496.4407	1499.4723	1499.7077
1502.6949	1514.2392	1514.9874
1522.2584	1525.6474	1529.2594
1533.4110	1534.8056	1539.6783
1541.4708	1573.6745	1577.4953
1619.3741	1622.4688	1628.6442
1631.3099	1635.7002	1635.7692
1636.9015	1643.4827	1649.4232

1651.9211	1653.3579	1653.7813
1657.3441	1671.8373	3026.7947
3040.8723	3043.1102	3049.2069
3050.7691	3051.2276	3064.3041
3098.1835	3105.4303	3111.3416
3115.2802	3121.8315	3129.8418
3132.5606	3134.0071	3134.8255
3139.5047	3141.7103	3148.6431
3159.2468	3171.6991	3178.6430
3179.4422	3182.5257	3183.9743
3185.6546	3185.9615	3187.2320
3187.7744	3187.8735	3192.2741
3192.9560	3194.1513	3196.1976
3199.4514	3201.6775	3204.6328
3204.7579	3205.9174	3212.0466
3213.2785	3213.7454	3217.6810
3223.0156	3224.0601	3224.1862
3226.9777	3253.6002	3311.5948

TS6(R)$^{HOBt}{}_{L4}$

-344.8849	13.8127	18.4381
24.0964	27.0182	27.8177
29.4476	31.6934	36.1892
37.4827	42.8410	44.9861
48.3458	52.1133	54.1178
58.4066	64.2665	64.6994
68.8111	72.0208	79.9065
82.7676	89.3583	93.2773
95.2621	98.2677	103.8761
111.3273	119.2792	121.1698
127.0649	130.8256	134.4094
142.2490	145.5245	160.8637
178.7610	188.4948	196.7358
209.5605	213.1943	216.5756
230.9206	234.5951	240.7482
245.5835	247.3109	253.6948
259.0883	267.9001	268.7078
283.9104	290.7580	293.4647

307.3911	310.5201	324.6813
331.6776	339.3735	341.7453
352.3060	367.9711	372.8667
376.0598	395.6200	411.7623
415.9698	418.4837	419.7451
420.6745	423.3527	424.3027
436.8463	443.0902	461.9720
487.9311	492.7952	504.5340
509.5354	514.9263	518.9073
527.9372	530.5411	532.5598
541.1888	553.8396	562.0269
564.1535	569.0417	582.1260
582.7596	587.5955	589.0273
608.2258	612.6904	627.1347
628.6744	630.0231	635.1366
640.2863	643.8217	645.2665
671.3930	675.6652	685.4407
698.3617	708.8142	714.7908
719.8288	722.0107	722.5026
748.8250	756.6928	764.8889
767.8487	770.0162	771.9431
778.2969	783.0813	785.6725
792.7259	793.5071	796.3490
810.8680	811.9059	819.2872
833.1784	845.0267	845.8411
856.2265	876.0985	877.3933
878.5419	879.9942	882.6756
885.6304	887.1131	898.2660
911.7220	921.0187	930.2403
936.0464	939.5581	948.9558
957.3632	959.0149	965.5933
981.8175	984.3884	992.5547
997.6233	1005.0760	1005.5950
1008.1421	1008.2508	1013.6453
1014.0947	1014.7082	1015.7709
1018.9480	1023.4651	1026.2034
1027.1770	1028.5510	1029.2048
1037.3530	1039.8696	1043.7546

1050.3313	1052.1483	1060.2669
1063.8351	1064.1399	1064.9938
1067.0929	1067.7693	1069.1649
1071.0056	1071.2110	1072.6484
1092.5442	1097.9985	1112.8083
1117.4512	1124.1375	1135.9657
1138.1120	1145.1621	1147.4686
1149.3050	1169.9403	1171.3251
1171.5724	1172.8208	1178.4840
1182.6697	1189.3579	1194.4885
1198.9634	1201.9376	1204.5387
1207.8922	1208.6512	1230.7837
1239.8851	1240.2857	1246.8147
1250.7692	1252.3922	1270.6149
1276.8880	1280.0392	1306.1418
1314.3196	1322.4823	1328.0439
1329.7308	1330.6411	1332.9076
1333.3618	1337.3013	1341.9193
1344.7505	1351.0379	1352.7335
1357.4240	1359.0971	1364.5339
1377.8706	1380.0288	1381.8539
1390.2591	1392.7394	1414.7076
1418.9902	1419.9515	1421.3890
1428.5691	1430.5060	1440.2487
1447.0247	1458.3116	1461.3592
1466.5943	1476.5719	1480.4619
1480.8369	1481.7359	1483.6837
1486.3854	1487.1929	1488.3777
1491.5005	1494.2565	1494.6959
1498.6302	1501.9874	1504.5692
1516.3270	1517.4718	1535.9168
1541.3745	1544.7079	1546.9470
1547.3551	1550.0969	1554.3275
1590.0415	1608.9918	1614.4544
1661.9804	1663.5182	1665.7678
1668.5216	1671.5088	1674.7148
1681.9847	1685.7783	1686.0574
1688.1359	1688.7456	1692.1439

1704.5071	1714.7633	3052.7262
3056.6697	3073.3561	3075.0403
3077.4556	3078.8157	3084.2972
3091.3310	3140.6194	3147.4431
3147.5128	3148.7066	3157.8830
3158.2837	3164.9259	3166.6370
3171.2752	3171.2812	3174.1401
3177.4171	3188.4247	3200.5893
3202.9540	3203.8688	3208.4195
3208.5076	3208.9157	3211.5622
3212.8865	3213.0645	3216.7332
3217.6957	3219.3759	3220.3108
3224.3510	3224.7956	3225.7074
3226.0314	3226.5326	3231.3237
3235.8307	3237.2126	3238.0367
3238.2324	3241.7811	3246.8320
3257.0669	3259.9404	3320.5737

TS6(S)^{HOBt}$_{L4}$

-320.0532	14.5544	18.3980
20.9542	22.5787	24.6358
28.9299	30.5451	34.0968
37.6055	38.9647	40.7320
41.8477	49.7879	51.0612
56.5500	58.2724	66.4240
74.0752	75.9136	79.4129
85.6903	88.9890	100.5667
104.5563	110.6593	115.6121
117.4515	122.0684	126.8607
127.3396	141.5840	149.0370
159.5833	163.8971	167.9257
178.0560	187.9540	199.4078
202.4788	205.5022	208.0186
213.9906	233.9886	239.5028
246.5856	249.6523	256.2369
258.7198	261.1986	270.1553
277.3886	283.8337	291.2034
301.3991	310.5180	328.2043

336.0348	339.6716	346.6378
356.0716	366.0217	373.5568
383.1830	401.9159	405.1003
413.4964	415.7845	419.7894
425.5981	426.9332	437.8174
443.6282	451.0799	467.8549
475.3880	498.3094	505.0405
512.3987	513.4313	517.6636
519.6462	524.8865	532.8467
537.1848	555.4479	556.0586
558.6594	565.3652	577.5270
584.0803	584.8763	588.2174
601.5425	608.2274	627.3329
627.7914	630.0287	633.9059
637.5333	644.3038	657.0267
672.5136	677.6027	678.5989
687.0228	695.9884	710.7169
719.7000	722.0564	726.8884
732.0436	750.9192	753.0982
759.7129	771.1686	771.7200
774.7952	776.9886	784.7761
788.8751	793.4222	795.5221
811.0294	822.2790	822.5927
828.4738	837.7966	842.0478
850.4637	867.3184	869.4625
871.4794	881.0928	884.4569
886.8227	891.1765	903.7006
908.0294	919.0178	919.5484
936.0281	944.1994	960.7495
962.7755	967.2336	979.1507
980.7651	982.2996	984.6561
1000.3545	1002.0886	1011.0563
1013.3709	1013.7623	1014.0893
1015.0411	1016.9093	1017.0856
1018.0447	1024.3929	1026.3298
1026.6335	1029.1799	1030.3875
1031.0449	1040.7583	1042.9846
1044.5183	1049.2682	1057.6052

1057.9580	1063.4589	1065.3325
1066.1310	1067.4361	1068.5419
1068.7590	1070.0740	1070.3228
1095.4190	1111.4479	1115.3948
1120.2856	1124.0222	1137.2181
1140.1539	1143.1838	1144.8354
1147.6101	1149.5739	1170.7591
1171.1576	1172.7593	1182.4204
1184.2686	1186.3987	1200.8636
1201.5305	1202.8807	1205.0887
1206.9230	1223.1069	1227.3398
1234.3341	1237.3574	1246.4292
1251.5977	1255.9668	1269.0675
1275.5375	1280.4221	1296.6003
1312.0168	1319.4113	1326.7343
1327.4316	1329.3791	1330.0973
1335.8368	1342.7387	1347.3912
1352.5702	1353.0831	1355.8602
1357.2651	1360.1389	1371.4362
1372.4340	1385.8168	1391.1335
1392.2357	1398.8156	1411.3817
1415.4955	1417.3864	1426.3810
1433.1751	1438.1429	1443.3948
1446.2682	1449.0790	1459.8711
1467.2547	1467.9751	1473.2237
1482.4372	1484.4817	1484.6847
1485.5383	1489.3404	1492.2353
1492.5551	1496.5791	1499.9095
1501.2025	1504.0928	1506.0080
1511.7494	1515.2391	1530.0245
1533.2301	1544.0286	1548.4904
1549.7558	1556.1496	1560.6328
1573.2076	1609.9175	1614.6316
1662.4057	1664.7410	1667.3933
1668.9508	1669.9163	1674.7047
1679.9632	1686.2227	1687.0087
1687.2674	1687.7594	1690.9066
1709.9066	1713.4418	3061.7281

3076.9511	3077.5794	3077.6474
3078.0495	3083.6274	3092.0230
3102.4520	3146.9794	3147.6031
3149.4486	3151.2870	3158.0949
3165.3121	3170.3629	3171.6437
3171.8400	3176.7907	3195.2845
3201.4939	3201.6291	3205.0049
3205.5017	3208.8836	3210.2383
3210.8286	3211.1510	3212.2888
3214.8970	3216.9645	3217.0238
3217.9173	3220.2462	3221.0280
3224.4702	3224.5496	3224.9836
3226.2866	3228.6582	3229.6694
3235.2908	3235.5640	3236.1390
3237.9482	3240.0948	3246.8117
3254.0746	3273.7450	3351.9914

TS6(R)^{HOBt}$_{L5}$

-352.9933	12.3281	17.2847
25.1161	27.2100	28.3669
30.4543	33.4657	37.6419
41.0063	44.2213	45.2985
46.5002	53.9099	56.8366
61.5101	63.5727	69.9032
74.2986	77.9625	85.9223
91.3705	95.5744	98.1764
101.7264	106.6584	108.7180
113.6384	125.5351	130.2573
133.7597	136.2298	153.0243
162.5094	165.8400	167.7173
178.5154	190.3287	194.3016
204.0821	208.3378	210.7963
217.9342	218.9360	229.3896
237.7845	240.7016	242.6759
255.6140	263.7018	272.0986
280.3321	283.4991	291.1131
298.0240	314.3656	322.3307
331.3315	336.9994	338.9553

341.3565	352.8333	374.1491
387.9739	395.0178	416.1231
419.0898	422.3366	424.5356
426.6309	431.9690	441.5306
443.8487	457.6614	475.9399
487.4443	492.8512	508.4833
510.7784	516.0368	520.7031
525.9201	531.1301	538.1242
538.5987	544.1722	562.8201
564.3859	569.3494	582.5427
585.2923	587.3525	591.5596
594.7496	611.6374	622.0994
627.3418	637.0958	637.8268
639.0309	640.6342	654.4499
667.0194	689.0004	694.9562
705.7498	711.4218	713.3179
721.8090	725.5236	728.7616
732.9296	760.6779	769.8436
771.2088	771.9071	772.9266
779.5776	788.3343	789.5920
794.0807	799.8927	810.0221
813.0541	818.9604	825.2279
830.9286	837.4736	847.6434
863.1443	866.4752	873.4108
877.3831	880.0729	883.3974
885.9067	886.5961	900.1238
907.4142	914.3019	919.3528
940.7875	942.3126	947.5994
959.9402	971.2005	979.5661
985.0919	991.6612	1002.1139
1002.3537	1006.6902	1008.5382
1014.5802	1015.8194	1017.4790
1023.0470	1023.2805	1025.4145
1025.8860	1026.0655	1026.4630
1029.3282	1032.3249	1039.7552
1047.0533	1047.3899	1052.3008
1053.6711	1057.0404	1067.4592
1070.0763	1070.8957	1071.2993

1071.6765	1072.7500	1075.2941
1075.7242	1077.4115	1082.1028
1091.4409	1123.2614	1126.5065
1127.8050	1135.9362	1140.1827
1145.0299	1154.1246	1157.7006
1161.2623	1175.7799	1180.2557
1187.4725	1191.1719	1192.0985
1192.6604	1204.1529	1217.0699
1218.2480	1220.3321	1220.5229
1223.4661	1233.8368	1234.2413
1241.4717	1248.7201	1250.2678
1251.7278	1254.2575	1281.2122
1298.5671	1302.1541	1312.7158
1328.4270	1331.8527	1338.9362
1339.2646	1343.1958	1344.0495
1348.8284	1353.0585	1357.2931
1359.6944	1362.3759	1365.6120
1366.9140	1371.2586	1374.9993
1375.8104	1388.5666	1398.2304
1404.0550	1416.6046	1421.7047
1429.2726	1434.4976	1435.9637
1439.6210	1442.1777	1443.3503
1452.0246	1454.8944	1457.1407
1470.1807	1475.1105	1487.8963
1494.7184	1495.5130	1498.2697
1501.5206	1502.1060	1504.1026
1504.9243	1509.1232	1509.3961
1509.8581	1510.8455	1512.0253
1513.3242	1525.1276	1536.8271
1546.4578	1547.4446	1552.9876
1557.1481	1561.1250	1563.6453
1573.3733	1611.9833	1630.2920
1661.8412	1665.3951	1668.9119
1671.4928	1672.4853	1675.1772
1684.0997	1691.3637	1691.8720
1692.4130	1693.4987	1696.9063
1714.3545	1715.9977	3029.5650
3037.2712	3054.5800	3061.6582

3068.6255	3069.1570	3110.4912
3137.0728	3138.5716	3143.3327
3149.1580	3164.6564	3165.1417
3168.3121	3171.7485	3171.9248
3172.4027	3184.0357	3187.8203
3197.0155	3203.0753	3203.6450
3204.1760	3205.7367	3208.6598
3210.2241	3210.5215	3212.3055
3212.6611	3213.9589	3214.9119
3217.0946	3218.2835	3219.6576
3222.8089	3225.2284	3225.3452
3231.0524	3231.6228	3234.7873
3235.8651	3237.1538	3239.5248
3245.4490	3253.6575	3255.3387
3276.9077	3293.7564	3318.8011

TS6(S)^{HOBt}$_{L5}$

-353.7210	13.5213	17.8504
20.7347	22.4859	24.1509
26.9829	28.9131	37.3309
37.9737	43.8647	47.2937
51.2346	52.7784	59.8311
64.3798	72.9476	76.8041
79.4997	84.1135	89.0407
97.8112	106.5271	110.4356
111.7570	116.7687	121.0183
123.1748	128.6511	129.3889
137.9688	145.8810	152.5449
157.5285	166.1167	176.7472
179.9092	184.1420	198.1586
204.8228	211.2595	218.6480
230.1971	236.2424	244.8671
246.6973	249.0741	255.5872
258.5486	263.0635	275.5624
278.3808	280.9735	291.0897
302.0508	308.0366	331.6740
332.7072	334.9002	352.3611
354.4365	363.9357	376.1455

387.8119	400.7455	412.6577
418.3592	423.7443	425.3303
430.2284	431.6878	443.5093
444.4526	449.6462	473.2775
478.3004	498.9265	512.0004
512.8684	518.1572	523.8523
527.4241	531.6662	537.3003
541.1010	559.1142	560.4092
561.1462	568.5661	580.9860
584.3894	587.9432	591.6340
607.6017	610.6199	633.4730
635.7416	636.5019	637.1516
641.5267	651.8897	665.6615
666.0607	681.7024	684.5464
689.9745	698.9281	717.4675
719.9913	724.6875	728.8561
729.8776	749.7190	757.0602
758.6731	765.0019	766.8318
774.0996	775.0265	785.2446
789.9438	793.1797	798.4331
812.7726	814.8377	820.1175
825.9029	843.2145	846.9956
856.7483	861.2606	869.7316
876.6022	880.0999	882.4400
886.4571	886.5497	908.9704
911.8215	918.6468	931.4063
942.1056	948.1200	948.6055
961.7584	970.5942	977.8420
985.1287	987.6805	999.3014
999.7467	1003.4358	1004.5201
1009.2006	1012.8688	1015.0349
1021.0069	1021.5062	1022.0009
1025.7620	1027.0009	1028.0746
1031.2754	1032.1406	1036.5701
1037.6152	1046.3521	1047.2260
1049.8712	1053.1043	1060.6181
1066.5841	1067.8831	1069.6078
1072.1046	1072.9687	1074.7716

1075.3121	1075.8086	1080.8540
1096.8056	1123.6251	1126.9658
1130.0416	1131.2994	1140.8249
1141.4179	1144.4170	1152.4388
1159.9337	1174.9980	1185.2840
1188.7359	1189.4523	1191.2430
1191.5471	1191.8809	1216.5976
1217.1365	1217.8681	1224.3084
1226.4521	1228.8669	1234.1433
1234.3101	1236.4144	1245.8845
1251.2343	1263.7854	1270.5137
1289.4997	1300.2848	1309.2236
1311.1578	1326.3081	1335.8542
1337.4148	1339.6049	1341.7377
1345.4247	1349.3334	1351.9023
1358.5900	1364.1178	1366.8381
1369.6455	1375.9592	1376.6594
1377.4989	1391.8308	1394.7565
1395.0504	1415.2632	1430.6942
1433.8895	1434.3385	1438.6976
1440.2074	1444.6861	1445.6591
1454.5355	1461.0995	1465.4464
1468.7090	1488.5304	1493.6319
1498.0295	1498.8558	1499.3758
1501.4886	1503.2058	1503.6010
1507.6113	1509.3677	1510.9568
1511.1725	1511.8363	1514.6833
1524.0718	1525.4922	1539.9097
1543.4530	1552.3437	1555.9226
1559.9666	1560.0861	1564.8696
1573.7773	1610.1078	1623.3027
1661.2446	1663.5648	1666.8293
1669.8497	1672.0651	1673.8565
1679.7822	1690.4016	1690.5958
1690.7928	1691.1790	1696.5549
1708.3297	1710.5919	3043.0076
3066.6764	3067.7570	3075.0301
3080.4045	3081.6111	3097.7521

3141.3473	3152.5871	3155.0479
3158.1313	3162.2180	3164.4281
3167.7576	3168.0862	3174.0846
3176.1100	3181.7819	3184.7192
3199.9030	3208.6235	3209.1213
3211.4085	3216.5613	3218.7712
3219.5819	3220.0371	3221.5420
3221.9204	3222.8557	3225.4323
3225.7616	3229.1188	3230.1442
3233.4330	3234.5219	3237.3933
3238.5838	3242.2965	3244.2423
3246.2950	3247.1946	3250.3705
3250.5592	3257.5411	3257.9054
3280.7060	3286.3232	3355.4602

$TS0^{DBU+R1}$

-1244.8245	13.1889	21.9195
23.6640	25.8809	28.5663
36.5689	41.2111	43.0998
46.0451	49.2993	53.4970
56.6229	58.8252	63.1619
67.3810	70.0406	76.3227
79.6104	82.7463	86.2081
91.2439	92.9952	96.3679
98.7437	104.9837	106.0388
110.5718	117.8249	122.2975
143.4845	151.4953	168.4074
175.1685	179.7069	187.1350
196.6491	207.6205	217.4867
221.8107	237.6218	238.6876
240.3154	251.2840	255.8623
259.0660	269.7971	280.0386
288.3361	311.4477	331.8381
335.4474	349.7533	350.3089
352.1876	359.8077	365.7473
377.9573	388.3482	395.2372
405.8512	408.7896	414.5034
416.9155	429.8462	440.0961
445.7770	458.4945	483.4801

488.2106	496.6337	511.8017
512.6148	514.1948	517.3769
524.2627	529.0384	529.9438
546.4800	558.2228	567.8827
572.1681	582.6330	591.6106
612.8649	615.5275	628.3050
631.1447	634.2671	635.2832
645.9645	657.0723	699.3257
707.1883	713.1667	714.0680
716.7729	721.5009	733.1841
738.8710	740.3545	756.5581
775.4675	785.2847	787.0943
812.5222	814.5166	822.0696
835.3819	841.6585	848.0853
852.9175	856.2654	861.8164
873.5544	878.8416	887.1128
887.6116	888.1837	891.9833
899.8344	905.3505	922.3506
925.6812	939.5315	940.0896
953.8699	967.2158	968.0816
984.2996	988.4266	990.1425
994.7040	999.6112	1002.7494
1003.7939	1005.7781	1014.0607
1017.4475	1018.3338	1022.4349
1023.2424	1025.1614	1029.5076
1033.0654	1036.6645	1043.1969
1043.2997	1046.4062	1054.5986
1060.9695	1067.6266	1068.3264
1071.0182	1076.6151	1077.9430
1086.6208	1095.2554	1105.5552
1112.1117	1117.9399	1119.8893
1127.3985	1128.0080	1128.5885
1133.5961	1146.2223	1150.6850
1154.3484	1173.7068	1179.9807
1180.6797	1187.6505	1188.6612
1191.0923	1204.7861	1207.6139
1213.9107	1222.4720	1225.6177
1236.5694	1243.7108	1245.9047

1250.5188	1257.5110	1264.7047
1269.9425	1273.4647	1276.9901
1278.8253	1290.3056	1298.6029
1301.6889	1311.9050	1322.2447
1325.9363	1328.9013	1329.3335
1333.4170	1341.3103	1346.2642
1351.8876	1354.8967	1357.7385
1359.9119	1362.2018	1362.6695
1363.1137	1368.1839	1376.7898
1381.5732	1384.1450	1386.4045
1388.1483	1398.7592	1403.3323
1409.3180	1412.7501	1413.0066
1421.1528	1421.5774	1423.6723
1426.9905	1432.3166	1444.1127
1465.8244	1470.0370	1475.2048
1478.7382	1480.3947	1482.5026
1483.7493	1484.9334	1486.8609
1488.0020	1488.5061	1490.4297
1493.3524	1494.8460	1495.4244
1499.5311	1499.6901	1502.4174
1504.1528	1504.4529	1506.9082
1509.4197	1513.0895	1515.8952
1521.5961	1526.2626	1548.9539
1551.9419	1554.7513	1558.2766
1578.2678	1644.8016	1669.8532
1676.7078	1679.6707	1682.3957
1688.3529	1690.8418	1696.2689
1696.9858	1699.1886	1721.3945
1736.7667	1853.1263	3047.3192
3054.1671	3054.4640	3059.8536
3062.0405	3067.1435	3068.5771
3069.8057	3070.0440	3070.2727
3070.6637	3074.0355	3079.8162
3085.7042	3086.3746	3097.4457
3097.9016	3112.1630	3115.4459
3120.0950	3122.8450	3124.0150
3130.7290	3140.7670	3141.3093
3146.7476	3149.3674	3150.6534

3151.6311	3156.4239	3160.8986
3165.2482	3169.5751	3174.1909
3180.0228	3180.8906	3184.3305
3191.9473	3193.7789	3195.4211
3201.9639	3203.7806	3210.4665
3212.8105	3217.0642	3219.1302
3230.8365	3236.2379	3238.4404
3249.9476	3263.9537	3271.9707

TS0^{DBU}

-1216.3111	17.7450	24.2542
28.9119	34.9860	39.4043
40.6110	47.2177	53.1248
62.2287	65.6898	76.7165
86.9158	87.8996	100.3884
101.8366	120.1453	141.2278
143.8095	166.0213	168.2920
188.2406	200.0790	212.5577
231.5427	233.9626	238.7022
239.2714	254.3577	262.9856
276.9944	309.4103	318.6306
338.1606	349.9802	357.2922
369.4970	381.2448	392.3149
406.5349	418.4019	436.6007
443.2601	478.9143	482.9293
491.2713	506.8775	511.8788
520.6812	524.0709	529.5444
559.0593	569.7049	581.5559
588.3040	613.9355	631.0813
632.9858	653.2217	705.8287
710.3020	713.5520	720.0643
731.9399	740.0846	754.2658
785.8111	813.3191	822.6537
847.8633	853.2488	861.0316
874.4381	887.3006	887.8850
892.9094	901.4135	923.3989
924.6554	938.9362	949.6814
962.9899	979.8837	991.3892
997.4774	999.8747	1005.8978

1014.5184	1019.3719	1023.6983
1028.1709	1035.1178	1036.0946
1039.2473	1046.3879	1049.9329
1060.4181	1065.2118	1065.9880
1069.4509	1071.2422	1073.5432
1101.9415	1114.3346	1120.3267
1126.6718	1128.3297	1132.1379
1139.3959	1147.1845	1181.1089
1187.0411	1192.3704	1207.8924
1213.2689	1219.1673	1223.5004
1242.6843	1243.7529	1251.2474
1258.6845	1264.4923	1265.1922
1278.1948	1289.3047	1297.2074
1322.1356	1324.0908	1326.1044
1329.8850	1338.5361	1338.6509
1342.4753	1355.8244	1357.6714
1362.5755	1363.6182	1367.4164
1373.9987	1380.4714	1382.8425
1388.2818	1397.4239	1399.2743
1410.9063	1412.5358	1416.0686
1420.8871	1423.3984	1424.8448
1431.9173	1445.0981	1466.8992
1471.2315	1479.4390	1480.8975
1484.5767	1486.8120	1488.5638
1490.0139	1490.8960	1491.8680
1492.4624	1493.7821	1496.0831
1497.8326	1500.0097	1504.1481
1509.9275	1512.2554	1515.1749
1520.4661	1525.8435	1554.7442
1558.2273	1579.0637	1654.6062
1675.9823	1682.9724	1689.7353
1698.3281	1700.9729	1725.3575
3046.2340	3048.8398	3058.3576
3058.4375	3063.1579	3065.5966
3069.2641	3070.5657	3070.9560
3074.5425	3075.0134	3075.9340
3087.1610	3088.9978	3103.0923
3112.4010	3116.1378	3120.9497

3123.9380	3126.4764	3127.9087
3131.6963	3140.4855	3141.7473
3145.8638	3147.7420	3155.1920
3167.6731	3167.9412	3169.9183
3173.2482	3179.5710	3187.3079
3194.5514	3206.7001	3210.8903
3211.5875	3215.6828	3231.9197

TS4$_{\mathbf{B3LYP}}$ (B3LYP/6-31G(d,p)/IEF-PCM$_{THF}$)

-1438.0689	8.3545	11.4880
13.9669	14.4855	19.4268
22.0634	25.6613	31.5537
32.7971	33.5309	38.1904
43.5169	44.6291	46.9622
52.4494	54.2856	56.7590
61.1594	65.6242	75.6235
81.1787	92.6886	96.1458
100.7710	106.6407	109.8167
122.2740	134.9608	143.5235
147.8628	170.0568	187.8945
189.4262	193.9023	203.2111
205.5642	211.8868	223.5520
229.6474	231.2065	234.1155
242.4777	243.5723	254.1169
267.6402	277.5595	301.5919
306.4435	326.5898	334.3310
343.4382	351.1355	362.1470
367.5501	377.5009	385.1332
386.1369	395.2521	407.5000
414.9260	417.9151	418.9131
428.8312	438.3900	442.2938
463.5782	484.6020	486.1134
508.9994	510.6808	516.5403
522.6487	525.6898	529.5653
531.0713	544.5812	553.7081
565.2335	577.3164	579.9396
581.4017	587.1093	590.9244
594.4103	609.5864	624.9152
633.6918	634.0685	643.4309

647.6101	651.4554	676.6288
699.1732	712.4001	713.7930
716.1641	722.3971	727.7140
750.2684	759.6763	766.6669
767.9978	769.2282	770.5970
779.7161	802.0062	802.7403
825.7418	842.3147	843.8994
852.8257	854.2826	862.4256
864.3820	866.2132	874.0873
874.6730	885.4599	894.4846
902.1250	902.9209	907.2811
908.8817	926.5324	930.8853
934.0350	946.5887	949.4672
953.6740	968.7611	972.1496
975.1595	981.8701	982.7556
984.1237	985.8168	990.9762
996.8571	1003.8634	1005.9503
1008.1631	1015.0082	1015.0938
1016.6151	1018.7329	1022.8793
1035.8980	1039.6216	1040.8477
1054.8859	1055.2261	1058.6696
1061.3409	1064.4626	1065.1579
1066.3425	1080.0472	1088.2133
1093.0252	1102.3478	1102.8191
1115.2082	1117.5777	1118.4694
1125.8491	1135.0211	1136.1283
1141.1925	1164.3150	1165.2055
1178.9033	1180.0294	1180.9530
1182.8218	1197.1489	1203.7356
1205.3505	1206.5685	1208.1892
1217.7371	1219.2053	1225.6842
1232.1614	1233.6237	1234.2293
1241.8204	1260.4530	1262.1739
1267.6785	1275.7072	1284.1775
1291.5475	1295.5278	1297.9144
1317.0931	1323.9328	1326.0354
1329.5663	1333.0812	1334.0832
1335.8819	1339.3813	1352.9114

1355.3219	1355.6161	1363.5604
1364.2437	1366.4224	1373.6228
1377.0561	1385.4974	1386.2605
1389.2295	1394.6283	1395.7011
1400.0283	1405.7342	1415.9255
1419.3999	1421.8906	1423.0227
1423.3889	1427.9316	1432.1294
1445.7789	1449.2564	1469.5161
1470.1337	1476.4478	1484.2828
1485.0915	1489.0400	1490.4430
1491.0940	1491.9971	1493.1547
1493.4675	1494.0585	1494.1656
1495.2586	1498.3742	1500.3547
1505.0938	1506.6247	1509.7501
1511.0429	1513.4207	1515.5927
1519.5879	1527.8658	1530.1051
1532.5540	1538.1350	1540.8314
1574.1549	1623.5336	1625.6932
1629.5870	1633.8510	1636.8200
1641.9633	1653.4929	1657.0223
1658.2493	1658.5476	1678.9231
2650.4655	3001.7906	3006.4532
3014.6588	3022.7351	3024.5865
3038.5292	3042.2847	3042.8167
3045.9228	3048.6136	3053.3414
3053.6440	3060.0235	3070.7519
3072.3049	3083.2500	3087.4376
3088.2907	3098.9726	3099.8623
3103.9569	3104.1485	3111.7536
3116.1939	3118.1334	3121.6727
3132.3455	3136.3742	3139.4270
3140.7547	3143.1606	3144.5310
3148.7759	3167.4113	3171.4347
3176.1749	3181.2210	3183.0534
3183.1631	3183.4405	3183.5277
3187.7058	3191.7759	3195.4230
3199.1956	3202.4211	3206.6923
3208.4202	3220.8059	3229.5296

TS5$_{\mathbf{B3LYP}}$ (B3LYP/6-31G(d,p)/IEF-PCM$_{THF}$)

-1126.9985	7.8069	10.8097
14.8794	16.2967	19.6698
25.6776	27.6906	33.7451
35.4607	38.4125	39.0593
43.4221	46.9178	47.9190
51.3910	55.0817	59.6577
61.0277	66.9752	69.2909
70.8299	76.2048	79.4113
90.5517	98.9472	99.6464
112.5243	119.1035	124.9381
128.9443	137.2575	141.0730
146.8386	153.3893	158.5404
164.1302	171.1555	177.5535
186.4119	196.1539	201.4993
208.6331	218.1058	221.8001
230.0953	231.1800	232.8610
238.5261	241.8117	251.3436
258.6018	262.6377	265.6694
266.8536	269.3036	273.2798
274.6554	278.0869	281.9065
284.5785	293.0455	297.4726
304.1059	304.3062	313.1497
316.0380	318.3012	326.7939
336.1737	341.4941	344.6624
350.5650	350.7233	356.5923
359.1974	360.0983	367.6810
376.4205	378.5766	379.8140
388.2687	393.0531	396.2733
399.7081	401.1535	404.9293
409.0546	412.6358	419.1992
422.8649	432.1738	435.0039
459.6407	462.8941	463.8200
467.4279	488.6105	493.6394
508.3772	512.2195	517.1953
524.7995	528.8624	530.8587
533.7360	539.4016	549.1379

563.3223	567.5259	576.9665
578.6190	583.2120	590.5467
597.5399	604.6182	611.1701
620.6460	630.3362	633.7620
641.4615	660.4292	665.1301
675.3823	678.2822	681.1722
697.1837	708.7359	715.8222
732.5363	751.1137	752.3668
756.0733	767.5769	772.7454
780.4489	789.8680	803.8014
809.2114	815.8227	829.4386
831.4938	838.7015	844.2563
863.4322	869.6625	874.7981
876.9803	890.5649	892.3197
893.0688	897.8462	898.6156
905.2938	905.6815	907.6917
920.0708	923.3626	933.3110
935.4040	935.6616	936.6016
937.5893	938.4180	938.5493
939.4640	941.5684	947.7064
952.7655	965.1363	965.5627
966.0147	966.8862	970.4650
972.9569	973.3077	975.2800
982.1726	986.2021	999.2594
1006.5013	1009.6237	1014.2143
1019.5616	1035.2473	1038.2540
1040.6191	1040.7839	1046.6375
1051.3681	1052.0673	1052.6089
1054.7525	1055.1521	1055.5602
1055.9251	1056.2584	1058.9789
1061.3719	1062.6417	1063.3942
1066.9193	1080.9416	1094.6443
1096.1245	1111.0379	1113.0398
1118.3543	1132.2156	1135.8321
1153.4093	1174.1771	1182.0913
1183.5109	1183.7627	1205.5415
1206.1885	1206.6049	1207.6674
1216.3588	1220.4537	1224.7250

1225.6842	1228.2306	1228.7235
1229.8646	1233.6385	1236.0073
1237.3229	1240.3283	1242.7845
1259.2667	1267.5048	1278.7866
1281.6089	1285.0755	1287.8176
1291.9529	1317.0036	1322.3922
1325.9377	1330.3059	1335.1583
1337.0164	1340.1971	1341.5997
1345.0551	1352.4120	1355.2279
1361.5147	1366.5579	1369.5265
1371.7288	1379.8792	1381.9253
1387.6757	1390.6863	1391.1369
1394.3037	1397.0197	1397.8387
1398.9863	1400.8780	1404.7429
1409.4848	1421.6937	1422.9002
1425.7152	1426.1489	1426.8593
1427.5111	1428.5056	1430.1438
1434.6158	1443.0254	1449.3450
1459.3185	1464.5285	1474.7597
1484.9744	1486.4279	1486.6184
1487.8756	1488.6129	1489.6227
1490.5578	1490.7334	1491.7929
1493.4420	1494.0482	1494.4306
1497.3116	1498.1465	1500.0438
1501.3869	1502.9623	1504.7801
1506.0028	1506.1477	1507.0790
1507.9904	1508.2714	1509.1034
1510.0292	1510.7047	1511.3247
1513.1168	1518.4341	1519.5203
1520.8092	1521.2582	1525.3488
1533.3518	1534.8350	1536.8177
1537.5689	1538.0715	1539.4832
1544.3334	1556.4281	1563.6637
1593.1021	1594.0847	1617.2773
1630.2104	1631.8184	1637.0615
1638.9759	1650.5781	1653.4089
1656.6753	1658.5974	3011.8834
3016.3634	3033.6503	3033.7487

3034.2413	3034.6703	3035.9166
3036.4450	3037.1803	3037.5367
3040.9170	3041.3072	3043.1196
3044.1974	3044.7793	3047.2802
3053.3197	3061.8133	3094.9094
3094.9704	3096.6933	3097.6123
3099.1581	3100.4806	3100.7025
3102.2743	3104.7218	3106.2081
3106.8278	3106.9236	3109.4193
3109.6056	3109.7961	3109.9651
3112.3617	3113.1204	3113.9501
3133.7071	3135.5306	3140.7809
3141.2890	3142.6794	3142.7446
3151.6786	3156.6697	3160.2938
3161.0299	3162.6679	3165.4615
3165.8444	3173.4657	3175.5800
3182.5490	3182.8841	3182.8843
3184.5251	3184.7208	3187.8007
3189.5227	3191.5619	3198.1881
3198.6912	3203.1303	3208.6795
3211.6182	3231.1567	3237.0289
3242.6916	3251.3339	3253.1627

TS4$_{\textbf{explicit}}$ (ONIOM(M06-2X/6-31G(d, p):UFF))

-1237.9778	6.2015	8.3909
8.6809	9.9686	10.9972
11.8519	12.0279	12.6216
13.0834	13.0904	13.4764
13.8147	13.9932	14.4003
14.8253	15.2560	15.4767
16.1477	16.6223	16.8579
16.9714	17.1688	17.7673
18.0393	18.2479	18.5422
18.5740	18.7401	19.9236
19.9995	20.0301	20.3462
20.7099	21.1473	21.4056
21.6742	21.7935	21.9658
22.1347	22.4367	23.1282

23.2601	23.5215	23.7154
23.8170	24.1583	24.4583
24.7790	25.1341	25.3407
25.4056	25.8403	25.9687
26.2419	26.3491	26.4401
26.7289	26.9807	27.2581
27.2971	27.7077	27.8220
28.0042	28.3486	28.5428
28.8574	29.2162	29.5574
29.7658	30.0352	30.0776
30.3673	30.7174	30.9050
31.2553	31.3294	31.5392
31.6786	32.0064	32.2400
32.3182	32.6061	32.7072
32.9764	33.4282	33.6833
33.9092	34.1606	34.2850
34.4533	34.8248	34.9600
35.1074	35.4313	35.4491
35.7329	35.9197	36.0595
36.4444	36.5931	36.7090
36.8644	37.1934	37.2933
37.5342	37.6104	38.1214
38.4223	38.5575	38.6996
38.7995	38.9304	39.0774
39.2835	39.5202	39.5742
39.9864	40.1388	40.2064
40.4570	40.6215	40.7897
40.9511	41.2719	41.4041
41.4937	41.5969	41.9774
42.1274	42.3102	42.4179
42.5753	42.8765	42.9617
43.1950	43.2701	43.3941
43.6890	43.8360	44.0056
44.2716	44.4781	44.5890
44.6909	44.7587	45.0280
45.0614	45.4210	45.4972
45.7598	46.0500	46.4078
46.6290	46.7547	46.8895

47.0270	47.1157	47.3504
47.6288	47.8707	47.9299
48.3361	48.3738	48.4616
48.6766	48.8262	49.0131
49.0668	49.3026	49.5681
49.8240	50.0604	50.1730
50.2601	50.3371	50.7357
50.8310	51.0850	51.2246
51.3874	51.4201	51.9399
52.0721	52.1538	52.4018
52.5518	52.7468	53.0044
53.1268	53.2919	53.3718
53.6251	53.7606	54.0125
54.1185	54.2233	54.5067
54.6821	54.7789	54.9017
55.0791	55.2425	55.5053
55.8144	56.1497	56.2274
56.2791	56.5652	56.5873
56.7151	57.0022	57.1643
57.2370	57.3517	57.7585
57.7618	57.8322	57.9407
58.2138	58.3728	58.7023
58.7969	58.9603	58.9705
59.2426	59.4195	59.5931
59.6427	59.8406	60.0758
60.3031	60.4736	60.6815
60.8210	60.9043	61.0727
61.1756	61.3765	61.4983
61.8924	62.0006	62.0983
62.2585	62.4482	62.5091
62.6202	62.7862	63.0422
63.2356	63.3073	63.5785
63.6809	63.7958	63.9728
64.0639	64.3941	64.6111
64.6393	64.9666	65.0945
65.3452	65.3749	65.5240
65.7798	65.8940	66.0920
66.1964	66.4549	66.6271

66.8139	66.8638	67.1164
67.4464	67.5490	67.7150
67.7711	68.0418	68.1364
68.2409	68.3957	68.6505
68.8371	69.1010	69.2622
69.3621	69.7289	69.8511
70.0260	70.2685	70.2772
70.6150	70.7308	71.0670
71.1199	71.1905	71.3708
71.5668	71.7118	71.8958
72.1456	72.3084	72.4456
72.8760	72.9125	73.0548
73.2236	73.4109	73.5601
73.7365	73.9207	74.1256
74.3791	74.4566	74.7216
74.8269	75.1196	75.1321
75.2205	75.4702	75.5644
75.8213	76.1154	76.1415
76.2537	76.6861	76.8398
76.9387	77.0786	77.3955
77.4865	77.6079	77.7476
77.9728	78.0746	78.3712
78.4851	78.7007	78.8130
79.1285	79.3363	79.4889
79.5297	79.9167	80.0055
80.1364	80.2305	80.3748
80.4700	80.6783	80.8489
81.1267	81.2843	81.5100
81.6581	81.9770	82.1745
82.3095	82.5179	82.6335
82.7210	82.8834	83.1348
83.3972	83.6933	83.7973
84.2317	84.4524	84.5078
84.6099	84.8511	84.9929
85.1527	85.3561	85.4871
85.5805	85.8637	86.0336
86.2015	86.2941	86.4332
86.4852	86.9673	87.0417

87.2149	87.5141	87.8053
87.9168	88.0372	88.1572
88.4306	88.6959	88.7011
88.9122	89.1328	89.2944
89.3962	89.5665	89.7886
89.8783	90.1090	90.3131
90.3944	90.6053	90.6253
90.7311	91.0176	91.1257
91.3108	91.8150	91.8874
92.1006	92.3922	92.5707
92.9431	93.0897	93.1348
93.2883	93.3788	93.7113
93.9438	94.0593	94.4375
94.5190	94.6941	94.7764
95.0019	95.0958	95.4438
95.5958	95.7852	96.1273
96.2620	96.3071	96.4480
96.5928	97.0853	97.2497
97.5353	97.6009	97.9021
98.0121	98.2833	98.3128
98.5460	98.8492	99.1558
99.2017	99.4482	99.6498
99.7844	100.0829	100.3785
100.5642	100.7970	101.0446
101.2162	101.2790	101.5675
101.8953	101.9922	102.0727
102.2745	102.3972	102.9704
103.2414	103.2557	103.4985
103.6345	103.9203	104.1226
104.1619	104.4472	104.8320
104.8783	105.3629	105.4759
105.5403	105.7790	106.0235
106.2833	106.5132	106.7717
106.9257	107.0620	107.2663
107.4142	107.9782	108.1325
108.3008	108.6272	108.6865
109.2376	109.4308	109.5762
109.8217	110.1204	110.3421

110.4922	110.7297	110.8942
111.2727	111.3220	111.8536
112.0765	112.2750	112.3398
112.5027	112.6390	112.9396
113.1198	113.2627	113.6693
113.7350	114.1316	114.3498
114.3676	114.6605	114.7824
115.0203	115.2958	115.4232
115.7312	115.9733	116.0976
116.4709	116.5894	117.0423
117.0658	117.5161	117.8428
118.0961	118.4254	118.6408
118.8816	118.9547	119.0654
119.5481	119.5695	119.9135
120.4535	120.5342	120.7717
120.9618	121.1129	121.2779
121.7148	121.8034	122.4400
122.5559	123.0472	123.1459
123.4341	123.8254	123.8838
124.2459	124.3571	124.8742
124.9905	125.2354	125.4901
125.7932	125.9490	126.2647
126.4325	126.8126	127.0573
127.4943	127.6124	128.0614
128.0903	128.4326	128.8842
129.0874	129.2839	129.8017
130.3124	130.4093	130.8029
130.8562	131.1096	131.3879
131.5343	131.8815	131.9665
132.5825	132.8822	132.9707
133.4064	133.8436	134.1446
134.3452	134.6411	134.8942
135.2431	135.8810	136.0441
136.7308	136.9412	137.6327
138.0483	138.1817	138.4984
139.0114	139.4388	139.6315
139.7983	140.3160	140.5133
140.7797	141.0029	141.7955

142.0984	142.2098	142.9492
143.0075	143.4884	143.8480
144.0312	144.7294	144.8480
145.0139	145.2794	146.0122
146.3194	146.6916	146.7929
147.1139	147.6362	147.9725
148.2199	148.4138	148.5748
148.8259	149.1594	149.4182
149.8251	150.8534	151.9292
152.1321	152.6678	152.9617
153.1067	153.4545	153.9178
154.9410	155.5240	156.0948
156.2155	156.8599	157.4715
158.2265	158.4697	158.9131
159.3267	159.8644	159.9510
160.3857	160.5498	160.9365
161.1013	161.6187	161.9730
162.4148	162.9735	163.4608
163.9989	165.3338	166.6069
167.4891	167.7006	168.4573
170.1991	170.4680	171.1084
171.7524	171.9750	172.4596
173.0003	174.4443	174.7187
174.9555	175.3619	175.6349
176.3268	177.4216	177.9466
178.9191	179.0095	179.3926
179.8988	182.1424	183.4258
184.6883	185.6774	185.8577
186.2058	188.2990	188.7189
190.9956	191.8899	192.1519
192.5558	193.1104	193.6635
193.8657	194.7331	195.6369
196.8744	197.5248	199.1476
199.6664	200.2200	200.7841
201.8028	202.8478	204.7783
204.9497	208.8086	211.3514
211.6504	212.7447	214.7222
215.9399	217.5375	218.4622

222.1856	223.9626	225.7246
228.7400	229.6389	230.6882
235.2844	235.8570	237.1497
237.5141	237.7352	238.4164
239.2727	240.4426	245.1701
246.3069	247.2225	248.1446
248.4491	250.9647	251.2916
251.6464	251.7747	251.9092
252.6550	253.2939	253.6742
254.7770	255.6671	256.5698
257.2861	257.9743	258.6087
258.7920	259.0126	259.1825
259.5799	259.6823	260.5241
260.8394	260.8587	261.1223
261.7451	261.8472	262.6808
262.9151	263.1374	264.2249
264.6803	265.0855	265.7665
265.9754	266.7950	267.4778
267.9457	269.2526	269.4188
269.7345	270.4005	270.5408
271.3159	271.5351	272.0268
272.2004	272.3747	272.4667
274.0461	274.2331	274.4371
274.9924	275.0551	276.2540
277.2924	278.0471	278.1977
278.3380	278.9103	280.2885
280.6080	281.8223	282.6878
283.7487	285.3530	286.1201
286.9450	287.4276	288.6994
290.4103	291.4974	291.9319
292.3975	292.5526	293.3663
293.8790	294.5846	294.8252
295.3960	295.9942	296.5063
297.5299	298.5863	299.1382
299.9071	300.6559	302.3682
303.6547	304.6784	305.7532
307.9671	309.0139	309.9812
320.5830	322.4043	324.2714

325.8296	327.7124	329.5033
329.5578	334.7638	340.4166
343.1374	343.7430	348.9853
353.7761	361.9303	375.0539
378.7423	386.0965	399.4673
402.8709	415.3464	427.3395
431.2663	431.5938	435.6945
439.5353	446.0193	450.8807
463.2162	468.5628	477.3181
503.0245	505.3190	519.0686
527.1872	531.6272	532.6856
538.4461	544.2175	546.3899
556.7388	565.5726	577.0398
586.2266	589.8728	590.7764
596.7695	607.6145	609.8002
622.3043	636.1042	637.1073
643.6468	652.7776	661.3609
685.4555	692.8725	724.0082
729.1417	734.0691	737.5101
742.3073	743.5532	767.3737
777.1501	778.5782	778.7298
778.9533	779.1234	779.1249
779.1514	779.3562	779.6962
779.7635	780.2854	780.5837
780.7448	780.9881	781.1214
781.1736	781.2898	781.5113
781.6134	781.7212	781.7508
782.0607	782.1165	782.2456
782.6480	782.7470	782.8436
782.8458	783.0375	783.1102
783.1460	783.3753	783.4273
783.6018	783.7540	783.8655
783.9416	784.0696	784.1210
784.1817	784.2296	784.6510
784.6591	784.7343	784.7679
784.8168	785.2175	785.2315
785.2677	785.4904	785.7148
785.8723	786.0478	786.2713

786.2833	786.4317	786.6319
786.9971	787.1410	787.3122
787.4607	787.8730	788.2058
788.4269	788.6413	788.7076
788.9020	789.0551	789.1277
789.3489	789.4714	789.8161
790.2214	790.2370	790.4632
790.9630	791.1199	791.4762
791.6167	791.9260	791.9501
792.4236	792.4729	792.6732
792.9453	793.1468	793.3668
793.4436	794.0442	794.4861
794.5755	794.6415	795.0170
795.1833	795.2187	795.8269
795.9091	796.0058	796.0697
796.3046	796.5708	796.8416
798.4076	798.4982	800.6285
803.7944	819.3256	820.8871
820.9425	821.2337	821.3022
821.4675	821.6867	821.8132
822.0654	822.2668	822.2780
822.3541	822.3678	822.4930
822.5583	822.5798	822.6720
822.7023	822.9565	823.0295
823.0556	823.2810	823.2995
823.3195	823.5286	823.5686
823.6935	823.7112	823.7274
823.7446	823.8251	823.8629
823.9539	824.0197	824.0977
824.1816	824.1925	824.2624
824.3340	824.3551	824.3727
824.4076	824.4711	824.5793
824.6211	824.6966	824.8216
824.8702	824.9525	824.9609
825.0128	825.0832	825.1704
825.2120	825.2796	825.3302
825.3750	825.4755	825.5286
825.5938	825.6288	825.6718

825.7991	825.9414	825.9686
826.0283	826.0441	826.0514
826.0639	826.1570	826.2205
826.4340	826.4668	826.5153
826.5996	826.6211	826.6635
826.8512	827.0102	827.0741
827.1448	827.2733	827.3107
827.3312	827.4590	827.5188
827.6583	827.7078	827.9360
827.9982	828.0649	828.3403
828.4019	828.4851	828.5987
829.1808	829.7109	829.9277
830.3264	830.8115	831.3949
832.4739	841.3157	845.0865
859.2141	864.9803	866.7249
876.6174	883.3913	886.7031
895.5468	901.5915	903.0207
907.3899	912.8442	916.1518
928.9058	936.0151	938.8544
948.3511	948.5767	950.4357
953.7341	953.9220	954.3706
955.3991	955.8611	955.9323
955.9900	957.1371	957.3225
958.2082	958.5962	958.8685
959.2647	959.4554	959.8697
959.9707	960.0068	960.8378
960.9480	961.1632	961.3771
961.7438	961.9638	962.0894
962.1826	962.3133	962.5629
962.9053	963.1391	963.1715
963.3738	963.5391	963.6344
963.8771	964.0144	964.0647
964.0853	964.2934	964.6773
964.7709	964.7919	964.8319
964.8440	964.8650	965.0698
965.6930	965.8936	966.0177
966.0517	966.2344	966.3484
966.4059	966.7340	966.7435

966.8069	967.0387	967.1621
967.3274	967.5231	967.6230
967.8197	967.8566	968.1516
968.2483	968.7401	968.7975
968.8901	969.1106	969.3784
969.4262	969.5031	970.0533
970.2254	970.3211	970.5302
970.8135	970.8929	970.9965
971.2355	971.5168	971.5806
971.9415	972.0661	972.0893
972.4322	972.5402	972.6352
972.8147	972.8275	972.9873
973.4364	973.5526	973.6408
973.6895	973.7526	973.8198
973.9504	974.0554	974.1896
974.2283	974.5105	974.5519
974.6168	974.6307	974.7509
974.7839	975.0093	975.0715
975.0934	975.1051	975.2680
975.4784	975.5059	975.5290
975.5496	975.9191	975.9801
976.0266	976.2020	976.2688
976.2953	976.5440	976.6628
976.6872	976.7070	976.8447
976.9950	977.0400	977.0585
977.1151	977.1263	977.2412
977.3956	977.5909	977.6615
977.7648	977.9042	978.1076
978.2284	978.2544	978.3889
978.4074	978.4260	978.6402
978.6675	979.0115	979.0337
979.2326	979.4647	979.4872
979.6979	979.8055	980.0834
980.1075	980.2308	980.2798
980.4389	980.5747	980.6615
980.8076	980.9368	980.9639
981.0560	981.1652	981.1920
981.2209	981.2384	981.2906

981.4477	981.5317	981.5424
981.7274	981.8648	981.9407
982.1079	982.5148	982.5858
982.7036	982.8947	983.2195
983.2662	983.3792	983.4561
983.5275	983.5963	983.7821
983.8425	984.0620	984.1140
984.1549	984.2045	984.2306
984.2592	984.4657	984.6317
984.7792	984.9393	985.2499
985.4880	985.5362	985.5654
985.6962	985.7661	986.0864
986.1646	986.3991	986.5705
986.6069	986.6276	986.6929
986.8361	986.9387	987.1051
987.1945	987.2311	987.4333
987.5682	987.6862	987.7165
987.7540	987.8234	987.8595
987.9878	988.1688	988.1940
988.2904	988.2955	988.7273
988.7744	989.3077	989.4029
989.7584	989.7974	989.8492
990.3042	990.3325	990.4852
990.8141	990.9594	991.1425
991.4803	991.4977	991.6347
991.6855	992.0978	992.1147
992.2767	992.3321	992.4230
992.6533	993.1331	993.2261
993.7264	993.9164	993.9909
994.0440	994.1272	994.2272
994.5074	994.6458	994.6781
994.8887	995.6088	995.6812
995.7955	995.9411	996.1979
996.3117	996.5195	996.5966
996.6239	996.8525	996.9849
997.8760	998.0053	998.1540
998.2223	998.2950	998.7134
998.7529	999.3332	999.5722

1000.3146	1000.9951	1001.1231
1001.5087	1001.8364	1001.8417
1002.0827	1002.3177	1002.4297
1002.6288	1002.7457	1002.9271
1003.5920	1004.7253	1005.8170
1006.4753	1007.0663	1007.9424
1009.6860	1009.7608	1011.6868
1013.2519	1013.9806	1014.6678
1015.6181	1018.1014	1019.3078
1019.9460	1022.8118	1026.8200
1028.9313	1030.2768	1033.9396
1040.4787	1041.6386	1044.6434
1051.9710	1058.0023	1060.2638
1062.0814	1078.1762	1078.6791
1078.9181	1079.6208	1080.4526
1080.8858	1080.9634	1080.9796
1080.9985	1081.0797	1081.1660
1081.6544	1081.7257	1081.9487
1081.9633	1082.2494	1082.4508
1082.5168	1082.5346	1082.8478
1082.9871	1083.0653	1083.1071
1083.1882	1083.4699	1083.5105
1083.7240	1083.7499	1083.9242
1084.2492	1084.3745	1084.5004
1084.5920	1084.6499	1084.7170
1084.7367	1084.8892	1084.9846
1085.2340	1085.3340	1085.6229
1085.6805	1085.7926	1085.9272
1086.3075	1086.3686	1086.4122
1086.5797	1086.6042	1086.8402
1086.9550	1087.0974	1087.1143
1087.2472	1087.3679	1087.5993
1087.6297	1087.6502	1087.6779
1087.7374	1087.7468	1088.0743
1088.1474	1088.2666	1088.3344
1088.3821	1088.6487	1088.6604
1088.6829	1088.9034	1088.9995
1089.1721	1089.3279	1089.4830

1089.5991	1089.7872	1089.9297
1090.1005	1090.2598	1090.2912
1090.4046	1090.4801	1090.7491
1090.8623	1091.0185	1091.2789
1091.3184	1091.8472	1092.2157
1092.4888	1093.0781	1093.3757
1093.4289	1094.1406	1094.6637
1094.6804	1095.0269	1095.3245
1095.5219	1096.1716	1096.2106
1096.5442	1096.6324	1096.6977
1096.7433	1096.7849	1096.9536
1097.1730	1097.2637	1097.3087
1097.6290	1097.6996	1097.7830
1097.8307	1098.0468	1098.1881
1098.7541	1098.8579	1098.9272
1098.9744	1099.1005	1099.2413
1099.3510	1099.3817	1099.4783
1099.6271	1099.6499	1099.9268
1099.9789	1100.0530	1100.0847
1100.2153	1100.2566	1100.4403
1100.5545	1100.5869	1100.7043
1100.7413	1100.8359	1101.0122
1101.0919	1101.1488	1101.3384
1101.4688	1101.6774	1101.7046
1101.8036	1101.9206	1102.0878
1102.1799	1102.2764	1102.3684
1102.4343	1102.4865	1102.5941
1102.7500	1102.7545	1102.7982
1102.8226	1102.8443	1102.9561
1103.1807	1103.3089	1103.3189
1103.4073	1103.6054	1103.8124
1103.9072	1104.0543	1104.0578
1104.1392	1104.3946	1104.5869
1104.8279	1104.8669	1105.1599
1105.2210	1105.2612	1105.3470
1105.4937	1105.6036	1105.6680
1105.9505	1106.0551	1106.3541
1106.4143	1106.6354	1106.8296

1106.8775	1107.1600	1107.7181
1107.8499	1109.0864	1109.2192
1109.2487	1109.7947	1109.8193
1110.1306	1110.6770	1110.7256
1111.1525	1111.8039	1112.3090
1112.3914	1113.7837	1115.0764
1115.4279	1115.5303	1115.9645
1116.2326	1116.3700	1116.4158
1116.4958	1117.0360	1117.2624
1117.3673	1117.4568	1117.9325
1117.9685	1118.1563	1118.3850
1118.4232	1118.5375	1118.5972
1118.7959	1119.1135	1119.2547
1119.2584	1119.4187	1119.6125
1119.8453	1119.9368	1120.2449
1120.5441	1120.5575	1120.6135
1121.0035	1121.2153	1121.5081
1121.6606	1121.7772	1121.9736
1122.0561	1122.4884	1122.6612
1123.0959	1123.3282	1123.5416
1123.8330	1124.0739	1124.5657
1124.6367	1124.8332	1124.9458
1125.2811	1125.6453	1126.0099
1126.2504	1126.7205	1126.9362
1127.6291	1127.7876	1127.9283
1127.9936	1128.4955	1129.0875
1129.5183	1130.1229	1130.5042
1130.5923	1130.8108	1131.1000
1131.2088	1131.2525	1132.3073
1133.1128	1133.5651	1133.7328
1134.4308	1135.0448	1135.5448
1135.8477	1137.0261	1137.7126
1139.0326	1139.3727	1139.4076
1139.8358	1139.8918	1139.9785
1140.3065	1140.5701	1141.0510
1141.3123	1141.4679	1141.6301
1142.5955	1143.4355	1143.8616
1144.7279	1145.4311	1145.9393

1146.6108	1147.0037	1147.1618
1148.4965	1148.7286	1148.7582
1149.8330	1149.9254	1151.7022
1152.2658	1154.6895	1154.7156
1155.8744	1156.9114	1160.0315
1162.0883	1167.1494	1169.9398
1170.3263	1170.7659	1170.9898
1171.9967	1172.1484	1173.0774
1173.2058	1174.6293	1174.8246
1176.2609	1176.7433	1177.3991
1177.5402	1177.6540	1177.8448
1177.8561	1179.3542	1179.5598
1179.7881	1180.5764	1180.7109
1180.8269	1180.9289	1181.1154
1181.8203	1182.1046	1182.9791
1183.0110	1183.1133	1183.2759
1183.3089	1183.6284	1184.2445
1184.2775	1184.4118	1184.7154
1185.5347	1185.7560	1185.8297
1185.9836	1186.0620	1186.0989
1186.4765	1186.8042	1186.8425
1186.9486	1187.0243	1187.1041
1187.3088	1187.7340	1187.9682
1187.9944	1188.0391	1188.4583
1188.5585	1188.5635	1188.6059
1188.9837	1189.0181	1189.0341
1189.1903	1189.3577	1189.7038
1189.7373	1190.0906	1190.5400
1190.8552	1190.9376	1191.0940
1191.2474	1191.3358	1191.5185
1191.5233	1191.9030	1191.9449
1192.0010	1192.0315	1192.4643
1192.5350	1192.6981	1192.8702
1192.9654	1193.1516	1193.3765
1194.5081	1194.9579	1195.1074
1195.2335	1195.3426	1196.3871
1196.9323	1197.5551	1197.6159
1197.7728	1198.2016	1198.9529

1199.3798	1199.6774	1201.5382
1203.7414	1204.1161	1204.2446
1204.5137	1204.9657	1206.6609
1214.2461	1220.0883	1223.0381
1229.1712	1230.5739	1232.4487
1234.8161	1234.8595	1234.8860
1235.4309	1236.6912	1237.3461
1237.6528	1237.8692	1238.0959
1238.2681	1238.6526	1238.8816
1238.9774	1239.1851	1239.2833
1239.3604	1239.5800	1239.7379
1239.8814	1239.9582	1240.5296
1241.0815	1241.1114	1241.2208
1241.5582	1241.8575	1241.8875
1242.1176	1242.4987	1242.8762
1243.3272	1243.3583	1243.7335
1243.9077	1244.3419	1244.4455
1244.4761	1244.7882	1245.0660
1245.1799	1245.2879	1245.2974
1245.4478	1245.7561	1245.9062
1246.5428	1246.7511	1246.8481
1246.8643	1246.9067	1246.9572
1247.0800	1247.6547	1247.9402
1248.4113	1248.4265	1249.0269
1249.2641	1249.6209	1249.9125
1250.9687	1251.2214	1251.5110
1251.6418	1251.7501	1251.8987
1252.2600	1252.6361	1253.6280
1254.1392	1254.1536	1254.4375
1255.0048	1255.0916	1255.4402
1256.4201	1256.9953	1257.6686
1257.9120	1258.1149	1258.4374
1258.5055	1258.9905	1259.3764
1260.0929	1260.2899	1260.3549
1260.6443	1261.0538	1261.2124
1262.0174	1262.2255	1262.8977
1263.0259	1263.4928	1263.5623
1264.0172	1265.0163	1265.3258

1265.5241	1265.9101	1266.0123
1266.1469	1267.3609	1271.4017
1271.4184	1276.9942	1283.5944
1285.5261	1286.2403	1286.3028
1287.9702	1288.1210	1288.2750
1288.5758	1288.5815	1288.6100
1288.9042	1288.9529	1289.2093
1289.3732	1289.6470	1290.1525
1290.8711	1290.9073	1291.3300
1291.8237	1291.9272	1292.1918
1292.2425	1292.3838	1292.4233
1292.8394	1293.2661	1293.6044
1293.7284	1293.7672	1294.1120
1294.1321	1294.1359	1294.4345
1294.6398	1294.6735	1294.7997
1294.8903	1294.9706	1295.1441
1295.2814	1295.2896	1295.4252
1295.5903	1295.6294	1295.7829
1295.9557	1295.9741	1296.1930
1296.2707	1296.4422	1296.5990
1296.9140	1297.0163	1297.0514
1297.1500	1297.4613	1297.5644
1297.6616	1297.7106	1298.0565
1298.2074	1298.5096	1298.5957
1298.6741	1298.8087	1299.0513
1299.0990	1299.1824	1299.2534
1299.4037	1299.6887	1299.8974
1300.0297	1300.2494	1300.2657
1300.5644	1300.7265	1301.0202
1301.0631	1301.1244	1301.3670
1301.7419	1301.7979	1302.3161
1302.3328	1302.5337	1302.9792
1304.2452	1304.4599	1304.7255
1305.0020	1305.1302	1305.7187
1305.8755	1306.4963	1306.6554
1306.8163	1307.2316	1307.5083
1307.7019	1308.0333	1308.2596
1309.4199	1310.0783	1319.4034

1328.0645	1329.3669	1335.2691
1337.3037	1340.5591	1344.2730
1345.9481	1350.2013	1351.6616
1352.6185	1352.8394	1355.4618
1356.4253	1356.6276	1356.9299
1356.9969	1357.3300	1357.4809
1357.6554	1358.4106	1359.4846
1360.1118	1360.1950	1360.3493
1360.6945	1361.2286	1361.3874
1361.4736	1361.6592	1362.0345
1362.8176	1363.0978	1363.2313
1363.4983	1363.7273	1364.0315
1364.1995	1364.2439	1364.4671
1365.1927	1365.2733	1365.3884
1365.4454	1365.5018	1365.6832
1365.8242	1366.0780	1366.6973
1367.1316	1367.1783	1367.3415
1368.1959	1368.2761	1368.5928
1368.7388	1369.0673	1369.1010
1369.4009	1369.5417	1369.5943
1369.6359	1369.7909	1369.9969
1370.1894	1371.9636	1372.2006
1372.8090	1373.4708	1373.5117
1373.6437	1373.8487	1373.8746
1374.1030	1375.8514	1376.3681
1377.2362	1377.3130	1377.8726
1378.0678	1378.2355	1378.3437
1378.6352	1378.7582	1379.9440
1380.4122	1381.0539	1382.1626
1382.5303	1384.7311	1386.5170
1387.1117	1387.3513	1387.7078
1388.0551	1388.3831	1388.5075
1388.6276	1389.6879	1389.7624
1389.8497	1390.4412	1390.7545
1391.0203	1391.0611	1391.2722
1391.7929	1391.9920	1392.1107
1393.2974	1393.3706	1394.9712
1395.2293	1395.4383	1396.3862

1397.0077	1398.0151	1398.4913
1398.8128	1398.8687	1398.9591
1399.3166	1399.3252	1399.3896
1399.6650	1399.6698	1399.8556
1400.0642	1400.2357	1400.2921
1400.4115	1400.4738	1400.4956
1400.8217	1401.6882	1402.2153
1402.2201	1402.2407	1402.3660
1402.5112	1402.6609	1402.8775
1403.0600	1403.4434	1403.8284
1404.0263	1404.0569	1404.9723
1405.0959	1406.2037	1406.5876
1406.6453	1406.6647	1407.0556
1407.4233	1407.8629	1407.8779
1408.0328	1408.1632	1408.3069
1409.1951	1409.4386	1409.5103
1409.7836	1410.2231	1410.2749
1410.5395	1410.6788	1410.7561
1410.9902	1411.0101	1411.0886
1411.0968	1411.4198	1411.4655
1411.5564	1411.5976	1411.6435
1411.8259	1411.9044	1411.9260
1412.1108	1412.1794	1412.2196
1412.3739	1412.4724	1412.6112
1412.6582	1412.6657	1412.9438
1413.0054	1413.0370	1413.2036
1413.4064	1413.5007	1413.5712
1413.6854	1413.7498	1413.8142
1413.8734	1414.0242	1414.0761
1414.0828	1414.3505	1414.4114
1414.4489	1414.8052	1415.1114
1415.6691	1415.7474	1416.3132
1416.4044	1416.7102	1416.9534
1417.2692	1417.3635	1417.6046
1418.4853	1418.5931	1418.9898
1419.9784	1420.0469	1421.6849
1423.2038	1424.4489	1431.6295
1433.6245	1434.6260	1441.8679

1445.0643	1449.3039	1460.7830
1462.4911	1463.7853	1464.5787
1464.7968	1465.2593	1466.6793
1466.8419	1467.7149	1467.8979
1467.9244	1468.6870	1469.0403
1469.1792	1469.1875	1469.3299
1469.4088	1469.7861	1469.8929
1469.8961	1470.0798	1470.3601
1470.3787	1470.5153	1470.7681
1470.8013	1470.8176	1470.9639
1470.9783	1471.0618	1471.0683
1471.0972	1471.2636	1471.3616
1471.4025	1471.4148	1471.4595
1471.5274	1471.5670	1471.6484
1471.7417	1471.7792	1471.8031
1471.9135	1472.1033	1472.1413
1472.2887	1472.3324	1472.3735
1472.5121	1472.5477	1472.6625
1472.6971	1472.7081	1472.7892
1472.8637	1472.8702	1472.9007
1473.0829	1473.1857	1473.2133
1473.3381	1473.3465	1473.3909
1473.4439	1473.4710	1473.5555
1473.6007	1473.6615	1473.7139
1473.7259	1473.8339	1473.8955
1473.9139	1474.0476	1474.1236
1474.1349	1474.1730	1474.2882
1474.3698	1474.5344	1474.5925
1474.6458	1474.6638	1474.7056
1474.7775	1474.8535	1475.0249
1475.0302	1475.0894	1475.1901
1475.2369	1475.2856	1475.3430
1475.4299	1475.4405	1475.4822
1475.4962	1475.5060	1475.6120
1475.6336	1475.8242	1475.8723
1475.9358	1476.0220	1476.0483
1476.0918	1476.1716	1476.2038
1476.2262	1476.2532	1476.3532

1476.3560	1476.4782	1476.5697
1476.6527	1476.7317	1476.7533
1476.7690	1476.8273	1476.8944
1476.9069	1476.9857	1477.0156
1477.0828	1477.2416	1477.2880
1477.3137	1477.3460	1477.4370
1477.4875	1477.5999	1477.7383
1477.8412	1477.8793	1477.9694
1477.9859	1478.0963	1478.2666
1478.3144	1478.3969	1478.4428
1478.5656	1478.6108	1478.6419
1478.8199	1478.8804	1478.9479
1479.0490	1479.0609	1479.0983
1479.1307	1479.2293	1479.3449
1479.4225	1479.4280	1479.5018
1479.6077	1479.6209	1479.6905
1479.7391	1479.8716	1479.9533
1479.9711	1480.0286	1480.1254
1480.2504	1480.2839	1480.3578
1480.5062	1480.5432	1480.6781
1480.8372	1480.8994	1481.1029
1481.1406	1481.3052	1481.3604
1481.3846	1481.4352	1481.4960
1481.6829	1481.7673	1481.9361
1481.9565	1482.0538	1482.1266
1482.2037	1482.2716	1482.3919
1482.6486	1482.7418	1482.7462
1482.8449	1482.9637	1483.0146
1483.1018	1483.1614	1483.2720
1483.3050	1483.5132	1483.6805
1483.7624	1483.8538	1483.8865
1484.0150	1484.1230	1484.1473
1484.2599	1484.4174	1484.5065
1484.5542	1484.6380	1484.7310
1484.9437	1485.0718	1485.1100
1485.1894	1485.2002	1485.3519
1485.4012	1485.4562	1485.4774
1485.5662	1485.6658	1485.7610

1485.8532	1486.1027	1486.1468
1486.2186	1486.2935	1486.4651
1486.5581	1486.6002	1486.6830
1486.7477	1486.9361	1486.9895
1487.0118	1487.1516	1487.2860
1487.3410	1487.4188	1487.4560
1487.4571	1487.7579	1487.9787
1488.0589	1488.1141	1488.2362
1488.3589	1488.3928	1488.4661
1488.6227	1488.7175	1488.9574
1489.0253	1489.2539	1489.3598
1489.5392	1489.5963	1489.6553
1489.9205	1490.1118	1490.3041
1490.4001	1490.5152	1490.6213
1490.8801	1490.9015	1491.0694
1491.2282	1491.3158	1491.3954
1491.6184	1491.8536	1491.8935
1491.9133	1492.0965	1492.1614
1492.2535	1492.5137	1492.5279
1492.6817	1492.7784	1492.8519
1492.8848	1492.9101	1493.0287
1493.2022	1493.2688	1493.3404
1493.3858	1493.5557	1493.6654
1493.7579	1493.7821	1493.9310
1494.0305	1494.0480	1494.2337
1494.2968	1494.3096	1494.4052
1494.4328	1494.6035	1494.8445
1494.9313	1494.9587	1495.0088
1495.0571	1495.0777	1495.4169
1495.4884	1495.5822	1495.9009
1495.9742	1496.0515	1496.1621
1496.2779	1496.3628	1496.4225
1496.5751	1496.6173	1496.6854
1496.7733	1496.9081	1496.9250
1496.9583	1496.9841	1497.0191
1497.0718	1497.1754	1497.2360
1497.3434	1497.4730	1497.8311
1497.8817	1498.0498	1498.1270

1498.2332	1498.4817	1498.5950
1498.7916	1499.0046	1499.1526
1499.2178	1499.5370	1499.7105
1499.7732	1499.9480	1499.9847
1500.2028	1500.3268	1500.5693
1500.6239	1500.6867	1500.8450
1500.9556	1501.1217	1501.1450
1501.3314	1501.4646	1501.5810
1501.6685	1501.8292	1501.9689
1502.2575	1502.3508	1502.4407
1502.6521	1502.6974	1502.8330
1502.8856	1502.9057	1503.2885
1503.4837	1503.6105	1503.7559
1503.9021	1504.0127	1504.1180
1504.6986	1504.8206	1504.9192
1505.2220	1505.4163	1505.7288
1505.9534	1506.1143	1506.1610
1506.3611	1506.7222	1507.7517
1507.8138	1508.1872	1508.3063
1508.3437	1509.1763	1509.2183
1509.4764	1510.3894	1510.4802
1511.2927	1511.4232	1511.5273
1512.1346	1513.1653	1514.2087
1514.3988	1514.4754	1514.6290
1515.7446	1517.4522	1518.0914
1518.2784	1518.4299	1518.8947
1519.5546	1520.5839	1520.8059
1521.0396	1521.5713	1521.6020
1521.6148	1522.2250	1522.7208
1523.1092	1523.2202	1523.5029
1523.9640	1524.0049	1524.2666
1524.3648	1524.5078	1524.9507
1524.9815	1524.9933	1525.4185
1525.6028	1525.6999	1525.7749
1526.4707	1526.6192	1527.0098
1528.1661	1528.5990	1528.9652
1529.2779	1529.3896	1529.5672
1529.7011	1529.7194	1529.8220

1529.8354	1529.9579	1530.0681
1530.3272	1530.4659	1530.8221
1531.2943	1531.4113	1531.5400
1531.5995	1531.6890	1531.7066
1531.7244	1532.1380	1532.2181
1532.2933	1532.7713	1533.0353
1533.0628	1533.1699	1533.2161
1533.2564	1533.2621	1533.8791
1534.0077	1534.0440	1534.0558
1534.2931	1535.0784	1535.1478
1535.3045	1535.8906	1536.2811
1536.4232	1536.9157	1537.3755
1537.8511	1538.0903	1538.1911
1538.5351	1538.5745	1539.0771
1539.4136	1539.5807	1539.7081
1539.8917	1540.4693	1540.6670
1540.8561	1541.0778	1541.3038
1541.6393	1542.4626	1542.5962
1542.9765	1543.0462	1544.3441
1544.4067	1544.4364	1545.4244
1546.0100	1546.0812	1546.7513
1548.0804	1548.1341	1548.9488
1550.0380	1551.1290	1552.2841
1552.8257	1554.5113	1559.7087
1560.9058	1563.2388	1564.2665
1570.9403	1576.4792	1576.5297
1576.6739	1577.4489	1577.6242
1577.7804	1577.9279	1578.4759
1578.6105	1578.6636	1578.7267
1578.8227	1578.8785	1579.0595
1579.0811	1579.2120	1579.2714
1579.3480	1579.4346	1579.4374
1579.5167	1579.5390	1579.6272
1579.7635	1580.0739	1580.2044
1580.3692	1580.4087	1580.4890
1580.5733	1580.6478	1580.7830
1580.7937	1581.0312	1581.1444
1581.2187	1581.2698	1581.4264

1581.6030	1582.0360	1582.0608
1582.2657	1582.3130	1582.5275
1582.5558	1582.6199	1582.6987
1582.7551	1583.1327	1583.2516
1583.3906	1583.4247	1583.6701
1583.7878	1584.0331	1584.0895
1584.1719	1584.4197	1584.4820
1584.8054	1584.9034	1585.1809
1585.3736	1585.6539	1585.9689
1586.0600	1586.2260	1586.2840
1586.3099	1586.4044	1587.0871
1587.1637	1587.4230	1587.4577
1587.7154	1587.7949	1587.8264
1587.9125	1588.0242	1588.3673
1588.4060	1588.6324	1589.0858
1590.6692	1590.7319	1591.0113
1591.1597	1591.6771	1591.7738
1591.8158	1593.2866	1593.4311
1593.7179	1594.1198	1594.7271
1594.9943	1597.0418	1597.4640
1598.0204	1598.6087	1599.2235
1601.0382	1604.0663	1604.6564
1605.4690	1605.7826	1606.3434
1606.3870	1606.4182	1606.4246
1606.4972	1606.6541	1606.9193
1607.5015	1607.7745	1607.8958
1607.9495	1608.1428	1608.3858
1608.4218	1608.4740	1608.4933
1608.5751	1608.7617	1608.7689
1608.7842	1608.8123	1608.9938
1609.2744	1609.3285	1609.5028
1609.5630	1609.6790	1609.7610
1609.9929	1610.0658	1610.2006
1610.2964	1610.4884	1610.5167
1610.8501	1611.1227	1611.2417
1611.3323	1611.3600	1611.3671
1611.4186	1611.6157	1611.7531
1611.7863	1612.0477	1612.0603

1612.1361	1612.3084	1612.5489
1612.7698	1612.8266	1612.9980
1613.3064	1613.5510	1613.7109
1613.8349	1613.8793	1614.0668
1614.0886	1614.4250	1614.4400
1614.6736	1614.8102	1614.9692
1615.0484	1615.1906	1615.3673
1615.5486	1615.6500	1615.8527
1615.8878	1615.9761	1616.1582
1616.2691	1616.4369	1616.4578
1616.8254	1617.4445	1617.6888
1617.7386	1617.9808	1618.0993
1618.2734	1618.6427	1618.8373
1619.1222	1619.2464	1619.3637
1619.5868	1619.8454	1619.8587
1620.0245	1621.5774	1622.4209
1622.9587	1626.6683	1653.6510
1655.1389	1655.1997	1655.2345
1655.3123	1655.5352	1655.7734
1656.1951	1656.2306	1656.4741
1657.0184	1657.0445	1657.0594
1657.2890	1657.3753	1657.4565
1657.5133	1657.6660	1657.7743
1657.9239	1657.9851	1658.2650
1658.2664	1658.6283	1658.6412
1658.9195	1659.1869	1659.4585
1659.5422	1659.6751	1659.7388
1659.7676	1659.9211	1660.0266
1660.0716	1660.2067	1660.3147
1660.4411	1660.6100	1660.6189
1660.6677	1660.6702	1660.7208
1660.8079	1660.9877	1661.0776
1661.2728	1661.3761	1661.4047
1661.4275	1661.5919	1661.7730
1661.8721	1662.1577	1662.3036
1662.7322	1663.3428	1663.7821
1664.7588	1664.8781	1664.9674
1664.9835	1665.1499	1665.3326

1665.5376	1665.6580	1665.8761
1666.5180	1666.9532	1667.2334
1667.5402	1668.9119	1668.9372
1669.1787	1670.0969	1670.2601
1670.6351	1670.8682	1671.1708
1671.6979	1671.9120	1672.2191
1672.2735	1672.3924	1672.4872
1672.5134	1672.7022	1672.8306
1672.8860	1672.9966	1673.4508
1673.7906	1673.8730	1674.1183
1674.3511	1674.8533	1676.6352
1676.6823	1677.1911	1677.2892
1677.4440	1678.3435	1687.6391
1688.0273	1690.1024	1698.7425
1704.9172	1705.8735	1710.4731
1713.2392	1740.5845	1808.8054
1809.5204	1809.6177	1809.6444
1809.6997	1809.9769	1810.0042
1810.0518	1810.1461	1810.1649
1810.3362	1810.3546	1810.3808
1810.4180	1810.6420	1810.7183
1810.7674	1810.8515	1811.0382
1811.0676	1811.0777	1811.3216
1811.5724	1811.6302	1811.6469
1811.6607	1811.8173	1811.9525
1812.1332	1812.1625	1812.1659
1812.2678	1812.2762	1812.3511
1812.3688	1812.4498	1812.5905
1812.6226	1812.6532	1812.6590
1812.7108	1812.7680	1812.7889
1812.8479	1812.8542	1813.0546
1813.1000	1813.2176	1813.2792
1813.2794	1813.4261	1813.4410
1813.5229	1813.6415	1813.7470
1813.9172	1814.1082	1814.2455
1814.2867	1814.3528	1814.3583
1814.3924	1814.3989	1814.4139
1814.4664	1814.5114	1814.5876

1814.7966	1815.0098	1815.2842
1815.3458	1815.3879	1815.4142
1815.4490	1815.4839	1815.6115
1815.8244	1815.8860	1816.4963
1816.6649	1816.6948	1816.7546
1816.7880	1816.8737	1816.9996
1817.0506	1817.0652	1817.1815
1817.3975	1817.5887	1817.7541
1818.3307	1818.5453	1818.6387
1818.7312	1819.6605	1820.2680
1820.7092	1820.8161	1821.5835
1835.4775	1837.7813	1838.2694
1839.3618	1839.5673	1839.6984
1840.3810	1840.4482	1840.4729
1840.5844	1840.5935	1841.2457
1841.4616	1841.7792	1841.8983
1842.3076	1842.5851	1843.6497
1843.6825	1843.9227	1844.2300
1844.4592	1845.0021	1845.0398
1845.5062	1845.8244	1845.8778
1846.2389	1846.3605	1846.6271
1846.6641	1846.6683	1846.9693
1846.9904	1847.0256	1847.5587
1847.6788	1847.7775	1848.1065
1848.1156	1848.1218	1848.1644
1848.1962	1848.2364	1848.4272
1848.4910	1848.5081	1848.5336
1848.5447	1848.6086	1848.6439
1848.8487	1849.0660	1849.2073
1849.4335	1849.5377	1849.6327
1849.7257	1849.7871	1849.8001
1849.8236	1849.9245	1849.9343
1849.9634	1850.0328	1850.1130
1850.1641	1850.1692	1850.2531
1850.4681	1850.4942	1850.5724
1850.6122	1850.7371	1850.9725
1851.0183	1851.0648	1851.6185
1851.8601	1851.9380	1851.9977

1852.1426	1852.1620	1852.2180
1852.2651	1852.5325	1853.0005
1853.0170	1853.0463	1853.2051
1853.6389	1853.6953	1855.0698
1855.3996	1855.7555	1856.0198
1856.2792	1856.5493	1856.9763
1857.3006	2147.7741	2873.3799
2873.4788	2873.5012	2873.7799
2873.9138	2874.2520	2874.3355
2874.5200	2874.5559	2874.7005
2874.7891	2874.9772	2874.9870
2875.0134	2875.1068	2875.1541
2875.2097	2875.2128	2875.3431
2875.3567	2875.4009	2875.4291
2875.5017	2875.5099	2875.5757
2875.6884	2875.8786	2875.9190
2875.9974	2876.0228	2876.0792
2876.1295	2876.1543	2876.2105
2876.4371	2876.6695	2876.6728
2876.7255	2876.7326	2876.9164
2877.0259	2877.1082	2877.1191
2877.1585	2877.1870	2877.3640
2877.4529	2877.5108	2877.6627
2877.7174	2877.8250	2877.8330
2877.8569	2877.9431	2877.9795
2878.0974	2878.1832	2878.2556
2878.3626	2878.5249	2878.7695
2878.9139	2878.9565	2879.1983
2879.6056	2879.6963	2879.7418
2880.2318	2880.4004	2880.4876
2880.9551	2880.9857	2881.0494
2881.0821	2881.1879	2881.2695
2881.3759	2881.4395	2881.5168
2881.5976	2881.9654	2882.2736
2882.7304	2882.7903	2882.8290
2883.0055	2883.2163	2883.4225
2883.5141	2883.5559	2883.6155
2883.7025	2883.7721	2883.8089

2883.8478	2883.8945	2883.9227
2884.0404	2884.0581	2884.0777
2884.1139	2884.2321	2884.3677
2884.4440	2884.5698	2884.6795
2884.7122	2884.7756	2884.8678
2884.9246	2884.9603	2884.9985
2885.0268	2885.0930	2885.1734
2885.2008	2885.2834	2885.3060
2885.3803	2885.4525	2885.4917
2885.6046	2885.6322	2885.6508
2885.6905	2885.7322	2885.7754
2885.8655	2885.9629	2885.9722
2885.9965	2886.0082	2886.2509
2886.2941	2886.3045	2886.3327
2886.3510	2886.4193	2886.4999
2886.6181	2886.6864	2886.7267
2886.7718	2886.8333	2886.9395
2886.9704	2887.0680	2887.1858
2887.3382	2887.4413	2887.6359
2887.7513	2887.8605	2887.8891
2887.9969	2888.2420	2888.2797
2888.4730	2888.4732	2888.5281
2888.7990	2888.8530	2888.9021
2888.9549	2889.0153	2889.0775
2889.1539	2889.1854	2889.1884
2889.4142	2889.4511	2889.6310
2889.6510	2889.6608	2889.6695
2889.9341	2890.0701	2890.1192
2890.2589	2890.2704	2890.3650
2890.4390	2890.5358	2890.5798
2890.6384	2890.8596	2890.8811
2890.9379	2891.0150	2891.0337
2891.4219	2891.5191	2891.5372
2891.6949	2891.7143	2891.7984
2891.8622	2891.9215	2891.9622
2891.9798	2892.0232	2892.0269
2892.1139	2892.1796	2892.2163
2892.2334	2892.3474	2892.3528

2892.3813	2892.3998	2892.4920
2892.5260	2892.5738	2892.6036
2892.6552	2892.7427	2892.8309
2892.9035	2893.0676	2893.0777
2893.0940	2893.1661	2893.1989
2893.2093	2893.2283	2893.3010
2893.3641	2893.3880	2893.3963
2893.4212	2893.4885	2893.5322
2893.5788	2893.6128	2893.6309
2893.6974	2893.7938	2893.8166
2893.8631	2893.8995	2893.9778
2893.9983	2894.0767	2894.1143
2894.1979	2894.2593	2894.3755
2894.4057	2894.4169	2894.6331
2894.6843	2894.7332	2894.7999
2894.8293	2894.9462	2895.0460
2895.1383	2895.2297	2895.2956
2895.3557	2895.3911	2895.4486
2895.4721	2895.6615	2895.7029
2895.7135	2895.7486	2895.7818
2895.8620	2895.9630	2896.0323
2896.1159	2896.1353	2896.2304
2896.3183	2896.4108	2896.4396
2896.5057	2896.5420	2896.6597
2896.6969	2896.7338	2896.7667
2896.8797	2896.9493	2897.1164
2897.1571	2897.2210	2897.2733
2897.3139	2897.4529	2897.4680
2897.4946	2897.5465	2897.5839
2897.6935	2897.7281	2897.7928
2897.8884	2897.9712	2898.0098
2898.1262	2898.1651	2898.2198
2898.3019	2898.3154	2898.4218
2898.6043	2898.7034	2898.7479
2898.7938	2898.8933	2898.9424
2898.9914	2899.0529	2899.0829
2899.1844	2899.2595	2899.4338
2899.4472	2899.5826	2899.6431

2899.8259	2899.9727	2900.0048
2900.0530	2900.1397	2900.1914
2900.2343	2900.2703	2900.3242
2900.4115	2900.4981	2900.7353
2900.7621	2900.8592	2900.9368
2901.0148	2901.0619	2901.0755
2901.0957	2901.2425	2901.6071
2901.6517	2901.7417	2901.9859
2902.0174	2902.1363	2902.1811
2902.2561	2902.5612	2902.6031
2902.8632	2902.9543	2902.9893
2903.2128	2903.3360	2903.6821
2903.7313	2903.8195	2903.8830
2904.0492	2904.0931	2904.5214
2904.6426	2904.6947	2905.2321
2905.4856	2905.5196	2905.5872
2905.6758	2905.7079	2905.8924
2906.0287	2906.5204	2906.7131
2906.8239	2907.0519	2907.3876
2907.5455	2907.7774	2907.8060
2907.8499	2907.9719	2908.0739
2908.2538	2909.6118	2910.0523
2910.8030	2910.8494	2910.8573
2910.9804	2911.1935	2911.4020
2911.6976	2912.3954	2914.2380
2915.5222	2916.1526	2917.0927
2950.5278	2950.8147	2950.9325
2951.0685	2951.2410	2951.3207
2951.3356	2951.3491	2951.5114
2951.5212	2951.5517	2951.5713
2951.6113	2951.6204	2951.7805
2951.8151	2951.8718	2951.9210
2951.9374	2952.0321	2952.2547
2952.4868	2952.5977	2952.9724
2952.9967	2953.0250	2953.2029
2953.2299	2953.2681	2953.2988
2953.3596	2953.3961	2953.4526
2953.4646	2953.4730	2953.4978

2953.5140	2953.5359	2953.5567
2953.5579	2953.6574	2953.6968
2953.7258	2953.7706	2953.8147
2953.8894	2953.9087	2954.0004
2954.0060	2954.1728	2954.1991
2954.2928	2954.3139	2954.3845
2954.5333	2954.5825	2954.6578
2954.6639	2954.7330	2954.8384
2954.8461	2954.9333	2954.9500
2954.9554	2954.9836	2955.0293
2955.1141	2955.1454	2955.2080
2955.2427	2955.3446	2955.4123
2955.4284	2955.4521	2955.4636
2955.4959	2955.5480	2955.5643
2955.6355	2955.6669	2955.6934
2955.7139	2955.7298	2955.7781
2955.7911	2955.8402	2955.8817
2955.9575	2956.0195	2956.0464
2956.0544	2956.1083	2956.1114
2956.1181	2956.1555	2956.2146
2956.2802	2956.3389	2956.4053
2956.4346	2956.5173	2956.5188
2956.6016	2956.6345	2956.6587
2956.6733	2956.8047	2956.8086
2956.8694	2956.8958	2956.9429
2957.0103	2957.0417	2957.0534
2957.0662	2957.1056	2957.1129
2957.1823	2957.2758	2957.2836
2957.3010	2957.3341	2957.3462
2957.3683	2957.4731	2957.4759
2957.4905	2957.5520	2957.5774
2957.6526	2957.7149	2957.7340
2957.7463	2957.7774	2957.7946
2957.8503	2957.8635	2957.9096
2957.9300	2957.9510	2957.9553
2958.0349	2958.0835	2958.0925
2958.1058	2958.1798	2958.1901
2958.2017	2958.2255	2958.2568

2958.2833	2958.3048	2958.3068
2958.3925	2958.4451	2958.5611
2958.6208	2958.6652	2958.6677
2958.6777	2958.6963	2958.7291
2958.7800	2958.8171	2958.9036
2958.9156	2958.9357	2959.0032
2959.0387	2959.0622	2959.1087
2959.1142	2959.1889	2959.2022
2959.2407	2959.2473	2959.2569
2959.2800	2959.3131	2959.3164
2959.3775	2959.3802	2959.4122
2959.4302	2959.4610	2959.4822
2959.5319	2959.5877	2959.5961
2959.6341	2959.6868	2959.7055
2959.7125	2959.7343	2959.7993
2959.8265	2959.8414	2959.8906
2959.9672	2959.9962	2960.0285
2960.0573	2960.0818	2960.1252
2960.1792	2960.2116	2960.2748
2960.3424	2960.4153	2960.4623
2960.4719	2960.4895	2960.5189
2960.5454	2960.5891	2960.6201
2960.6239	2960.6670	2960.7033
2960.7280	2960.8188	2960.8227
2960.8282	2960.8611	2960.8906
2960.9267	2961.0034	2961.0256
2961.0417	2961.2057	2961.3295
2961.3974	2961.4077	2961.4455
2961.5257	2961.5619	2961.6710
2961.6966	2961.7840	2961.8095
2961.8631	2961.9364	2961.9419
2961.9641	2962.0153	2962.0427
2962.0975	2962.1352	2962.1524
2962.2503	2962.3307	2962.3491
2962.3656	2962.4189	2962.4506
2962.5300	2962.5649	2962.7398
2962.7814	2962.7878	2962.8184
2962.8982	2962.9128	2962.9705

2963.0358	2963.0516	2963.1545
2963.2032	2963.2244	2963.2395
2963.3064	2963.3488	2963.3561
2963.4278	2963.4416	2963.4783
2963.4946	2963.5793	2963.6244
2963.7220	2963.8228	2963.8344
2963.8736	2963.8882	2963.8990
2963.9436	2964.0605	2964.1980
2964.2340	2964.2538	2964.2575
2964.3086	2964.3568	2964.4559
2964.4672	2964.4757	2964.5573
2964.5794	2964.6276	2964.6780
2964.7449	2964.7632	2964.8052
2964.8743	2964.9710	2965.0592
2965.1731	2965.3136	2965.3621
2965.4594	2965.5424	2965.5712
2965.6519	2965.8074	2966.1823
2966.2635	2966.3305	2966.3353
2966.4182	2966.4521	2966.5076
2966.5705	2966.7032	2966.8370
2966.9142	2967.3003	2967.4529
2967.5233	2967.5550	2967.6243
2967.7098	2967.7515	2967.8547
2967.9491	2968.0367	2968.0592
2968.2098	2968.2365	2968.2770
2968.3535	2968.4519	2968.6553
2968.6762	2968.7718	2968.8295
2968.8572	2968.9247	2969.0020
2969.0621	2969.1072	2969.1790
2969.2026	2969.2646	2969.4064
2969.4331	2969.5335	2969.5840
2969.6584	2969.6800	2969.9544
2970.2169	2970.3249	2970.5936
2970.7538	2970.9315	2970.9858
2971.1685	2971.8351	2971.8958
2972.0306	2972.0868	2972.2765
2972.2831	2972.6318	2972.6971
2972.7210	2973.4632	2973.4737

2974.4038	2974.4604	2974.9784
2975.0465	2975.1791	2975.7175
2976.1718	2976.3558	2976.9573
2978.0074	2978.0448	2978.1490
2978.5582	2978.6951	2978.9365
2979.2089	2980.1231	2980.5225
2982.1277	2982.5229	2986.3281
2986.6240	3028.5819	3064.5053
3066.2486	3067.9603	3079.1512
3081.3579	3085.5425	3088.9165
3089.3697	3089.6043	3090.4794
3097.1697	3107.4787	3112.8522
3125.1204	3126.9943	3132.8955
3136.9464	3156.8491	3158.4226
3159.8900	3160.9250	3162.5868
3165.0292	3168.5803	3169.5833
3173.2878	3174.4952	3178.4119
3180.7723	3192.0500	3192.1606
3195.0437	3197.6654	3198.1785
3206.0437	3208.4187	3210.9643
3217.0241	3221.3209	3221.5394
3232.0429	3234.6947	3238.2396
3241.5746	3244.4122	3251.0221
3256.0077	3270.7307	3279.2278

TS5$_{explicit}$ (ONIOM(M06-2X/6-31G(d, p):UFF))

-1252.4795	6.7762	8.0571
9.2065	9.7148	10.6104
11.2637	11.6173	12.1334
12.5536	13.3957	13.7895
14.8385	15.0982	15.1824
16.0394	16.3414	16.5904
16.8881	17.3609	17.5936
17.8764	17.9204	18.2641
18.4993	18.8089	19.3818
19.5563	19.6995	20.2660
20.4432	20.6199	20.7166
21.1949	21.4625	21.6068

21.9901	22.3026	22.3559
22.9741	23.2436	23.2798
23.4480	23.6295	23.9381
24.2386	24.2871	24.5969
24.8302	25.1788	25.6126
25.7513	26.0075	26.1901
26.7899	27.0025	27.1360
27.3299	27.5157	27.6863
27.9656	28.3049	28.3124
28.5442	28.7016	29.2450
29.3213	29.5796	29.8460
30.0675	30.2436	30.5410
30.8639	31.0182	31.0793
31.4581	31.6948	31.7845
32.2034	32.3524	32.4655
32.8822	33.0335	33.2652
33.7405	33.8139	33.8861
34.1075	34.3290	34.4638
34.6786	34.9750	35.3266
35.5184	35.7418	35.7592
35.9393	36.2217	36.3394
36.4022	36.6931	36.8358
36.9675	37.1927	37.4682
37.5996	37.7818	38.0125
38.2162	38.3442	38.4767
38.6234	38.9183	38.9756
39.1567	39.2808	39.6552
39.7733	39.8931	40.1733
40.4507	40.5391	40.7324
40.8075	41.1049	41.2979
41.3518	41.6522	41.8144
42.1009	42.3197	42.5034
42.6914	42.8822	43.0416
43.2177	43.3429	43.5523
43.7118	43.9032	44.1627
44.3563	44.5757	44.7627
44.9456	45.1505	45.2812
45.5739	45.8553	46.0402

46.0743	46.2545	46.5074
46.6102	46.6710	47.0642
47.1865	47.2594	47.2949
47.5420	47.6662	47.8727
48.0316	48.0699	48.2118
48.4203	48.6334	48.7916
48.9838	49.1694	49.2774
49.5239	49.7778	49.9871
50.1644	50.3194	50.4042
50.5817	50.6422	50.7807
51.0057	51.3660	51.5048
51.7646	51.8597	52.0120
52.1213	52.3463	52.4466
52.5335	52.8589	53.0022
53.1623	53.3205	53.4714
53.5872	53.9515	54.1733
54.3246	54.3490	54.6869
54.7578	54.9529	55.0428
55.3056	55.4112	55.5222
55.8695	56.2125	56.4340
56.5513	56.6311	56.8709
57.0953	57.2096	57.3910
57.4499	57.5583	57.8269
58.0890	58.2414	58.3995
58.5414	58.7341	59.0693
59.1989	59.3518	59.4379
59.4608	59.4990	60.0034
60.1205	60.3418	60.3738
60.5501	60.6307	60.8016
61.0025	61.1981	61.2534
61.3498	61.7428	61.7751
61.9904	62.2282	62.4824
62.5651	62.7683	62.8408
63.0888	63.1357	63.3169
63.5112	63.7188	63.8988
63.9484	64.0971	64.3488
64.4008	64.5905	64.6345
64.8781	65.0820	65.1548

65.2691	65.3051	65.6043
65.7541	65.9192	66.0458
66.2222	66.3174	66.4888
66.8418	67.0203	67.0805
67.4111	67.4785	67.5431
67.8531	68.0111	68.0264
68.2888	68.4226	68.5094
68.7375	69.0642	69.2600
69.4185	69.6184	69.6908
69.8367	70.2201	70.3213
70.5933	70.6906	70.7925
70.8210	71.1932	71.3990
71.6163	71.6862	71.8137
72.0237	72.1133	72.4600
72.6399	72.7562	72.8623
73.1530	73.2305	73.4012
73.6924	73.8428	73.8805
74.1700	74.5333	74.6150
74.7475	74.8344	75.0059
75.1681	75.2440	75.3424
75.7531	75.8440	76.0266
76.0882	76.2840	76.5163
76.8320	77.0605	77.0969
77.4555	77.6640	77.9439
78.0435	78.0895	78.3231
78.4667	78.6217	78.7027
78.9485	79.0497	79.2723
79.5094	79.6885	79.8499
80.1290	80.3315	80.4577
80.8312	80.9073	81.1973
81.3277	81.4118	81.6144
81.7368	81.8238	82.1279
82.2583	82.5311	82.6014
82.8669	82.9015	83.1387
83.2026	83.4843	83.6952
83.7353	84.0794	84.2014
84.5311	84.5678	84.8792
85.0914	85.2321	85.4855

85.5799	85.9107	86.1024
86.2511	86.3776	86.4656
86.6852	86.8401	87.0793
87.3801	87.4948	87.5773
87.8556	87.9504	88.1539
88.4434	88.4903	88.7387
88.7905	89.0410	89.2012
89.2901	89.3607	89.7025
89.7809	90.0022	90.2377
90.5347	90.5985	90.8538
90.9818	91.2906	91.3341
91.3987	91.4557	91.7750
92.1088	92.3593	92.3770
92.4810	92.7409	92.7863
93.0069	93.1137	93.3977
93.5158	93.6374	93.7650
94.0996	94.2162	94.3470
94.5855	94.7239	94.9049
95.1065	95.2900	95.3583
95.4987	95.9022	96.0646
96.2533	96.6464	96.7030
96.8034	97.1350	97.2366
97.3687	97.7141	97.9769
98.2581	98.3054	98.4909
98.5899	98.7497	98.7786
99.0854	99.1673	99.2906
99.4213	99.6758	100.2930
100.3162	100.4254	100.7026
100.8212	101.0950	101.2762
101.4280	101.5839	101.8103
101.9692	102.1988	102.3723
102.6885	102.8283	103.0124
103.2793	103.4162	103.7504
103.8990	104.0758	104.3135
104.5257	104.6950	104.8470
105.0922	105.3102	105.4029
105.5452	105.8151	106.1224
106.4602	106.4965	106.7926

106.9416	107.2965	107.3917
107.6260	107.7569	108.0541
108.0737	108.3884	108.7276
108.9496	109.0734	109.3108
109.4792	109.6668	109.8185
110.0718	110.2453	110.5950
110.6911	110.9798	111.1916
111.8384	111.9114	111.9758
112.1413	112.3363	112.7146
112.8493	113.1349	113.3090
113.4169	113.8628	114.0878
114.3724	114.6069	115.0030
115.0738	115.5562	115.7764
116.0537	116.1557	116.4715
116.5693	116.9331	117.0340
117.1563	117.4957	117.6156
118.0067	118.0591	118.2988
118.5952	118.9024	119.0666
119.1858	119.2207	119.4878
119.7361	120.1744	120.4705
120.8171	120.8774	121.1633
121.2727	121.6265	121.9382
122.1456	122.3764	122.4091
122.9912	123.2825	123.4271
123.8286	124.0083	124.1711
124.4823	124.9920	125.1196
125.4529	125.7105	126.1365
126.4355	126.6908	126.9982
127.3835	127.8927	128.2027
128.4887	128.6883	129.1318
129.5130	129.9547	130.2305
130.5357	130.9400	131.0747
131.4103	131.4703	131.7976
131.8897	132.5253	132.7980
132.9633	133.1380	133.6033
133.9800	134.3759	134.5355
134.8627	135.2577	135.7385
135.8841	136.5070	136.7067

136.9175	137.3278	137.7952
137.9798	138.7366	138.9336
139.0788	139.2921	139.6339
139.8482	140.0224	140.1790
140.7955	141.3433	141.6337
141.7037	141.8797	142.0459
142.2160	142.4741	142.9032
143.0474	143.5328	143.8270
144.0620	144.6162	144.9643
145.2086	145.6428	145.9503
146.0653	146.6695	146.6928
147.1468	147.4101	147.5626
148.0874	148.2761	148.7463
149.0439	149.3708	149.6125
149.6384	150.0899	150.5817
151.3153	151.6756	151.9502
152.2525	152.9526	153.3883
154.0908	154.2563	154.5202
154.6951	156.2221	156.6685
156.7307	157.3212	157.7352
157.8898	157.9336	158.6473
158.8766	159.7278	160.1703
160.6908	161.8486	162.7803
163.0259	163.5204	163.9950
164.1633	164.9298	165.5462
166.1830	166.2621	167.6986
167.9308	169.0913	169.4693
171.0237	171.1460	172.7821
173.0230	173.0457	174.4324
175.6092	176.1672	176.7660
177.6435	178.0119	179.3063
180.4233	182.3297	182.4230
183.0955	183.5359	184.8864
185.5081	186.0134	186.1508
187.0987	189.2590	190.1136
190.9861	191.0311	192.3273
193.6525	195.0297	195.3941
198.2391	199.5078	200.8889

201.7582	203.2233	203.7846
206.5552	207.2577	207.9742
209.6360	210.1666	212.5709
213.7159	214.3008	216.6469
219.0934	222.2672	225.6982
227.0355	228.0595	230.0342
230.9015	231.6194	232.4256
235.0015	236.3840	239.5830
241.4347	242.8355	245.6935
245.7191	246.5365	246.6000
248.7012	249.4326	249.4518
250.3665	250.5963	251.0085
251.3765	252.7647	253.1221
253.3207	253.9772	254.0998
254.1790	254.5708	254.9860
255.1983	255.3939	255.4426
255.7963	256.3779	256.9957
257.2202	257.9195	258.5975
259.1695	260.2219	260.4011
260.5643	261.2591	261.5375
262.0874	262.5486	262.8782
263.1236	263.7065	266.2222
267.1100	267.3900	267.8218
268.6928	269.4339	270.0681
270.6733	271.2175	271.2575
272.0173	272.7843	273.0150
274.2985	274.4992	274.7008
275.2725	275.8808	277.2557
277.6903	277.9239	278.0213
278.2943	278.7896	279.1788
279.8538	280.0442	280.5448
281.4756	281.9951	282.2825
282.4773	283.5364	283.8893
285.3668	287.2981	288.3747
289.2271	289.5191	290.2987
290.4667	290.5475	290.9085
291.1297	291.8050	292.3356
293.2797	293.8782	295.0599

295.8129	296.6183	296.8047
297.4131	298.6707	298.9625
299.2338	299.4479	302.8126
303.1988	305.4963	305.8513
306.2913	306.5224	307.9071
309.1966	311.7443	312.5144
313.8885	314.4705	314.8505
315.2466	317.2972	320.0293
322.3692	325.1127	329.1557
333.2858	333.8721	336.0968
340.0214	343.1185	347.1638
350.3079	353.2492	356.5959
365.6899	369.7652	370.9789
378.5616	381.0322	387.7563
390.2942	392.6934	402.8257
405.2422	410.7268	414.6459
416.2823	420.8274	426.4340
429.6590	431.3496	432.9964
434.8066	444.2432	455.2269
456.8869	462.3116	470.4198
472.9070	475.8577	480.2877
504.9196	507.7086	512.5825
526.8711	528.4390	532.6931
537.0430	544.4621	547.4584
559.9387	565.8942	572.4455
579.4391	581.9128	594.3116
597.9841	602.3370	603.9927
614.5458	627.9055	630.8693
634.7586	646.6892	661.8747
673.3986	675.0684	680.4119
691.0504	703.5295	711.6173
734.7316	742.6503	757.9493
764.3273	775.2056	777.4847
777.9675	778.1347	778.2220
778.4323	779.0674	779.1913
779.2510	779.4381	779.8052
779.9620	779.9884	780.0766
780.2020	780.2422	780.3864

780.5081	780.6292	780.6765
780.6847	780.7698	780.8510
780.8586	781.2747	781.5812
781.5959	782.0420	782.4487
782.4542	782.5677	782.6978
783.0166	783.0416	783.1503
783.3216	783.6496	783.6620
783.7229	783.8292	784.2232
784.3383	784.3751	785.1754
785.2012	785.4110	785.4813
785.9067	786.0122	786.0860
786.1967	786.3032	786.4631
786.4984	786.6847	786.8694
787.1637	787.3794	787.4592
787.5289	787.6513	787.7155
787.8743	787.9165	788.0573
788.2060	788.5284	788.8074
788.9405	789.1712	789.3749
789.5220	790.2211	790.3073
790.5880	790.9740	791.0104
791.0897	791.1977	791.2617
791.7223	792.6211	792.6402
792.9723	793.1200	793.1563
793.6624	793.8556	794.8577
794.9118	795.1741	795.5978
796.1368	796.3050	796.5826
797.6630	797.6779	797.8531
797.8883	798.1790	798.7727
799.2539	799.3131	799.4097
801.9683	806.7912	818.2013
820.8424	821.0503	821.5106
821.5446	821.8026	821.8799
821.9577	821.9647	822.0209
822.0365	822.1739	822.1975
822.2390	822.2883	822.3596
822.3812	822.4370	822.5720
822.8483	822.9026	822.9744
822.9812	823.0876	823.0908

823.2010	823.2187	823.3526
823.3552	823.3882	823.4396
823.5476	823.5820	823.5989
823.7086	823.7552	823.8005
823.8744	823.9037	824.0796
824.1945	824.2488	824.2938
824.3224	824.3791	824.4039
824.4088	824.4823	824.6256
824.7647	824.8674	824.9595
825.1192	825.1298	825.2634
825.2870	825.3342	825.3667
825.4444	825.4510	825.5128
825.6891	825.7379	825.8301
825.9060	825.9419	825.9466
825.9572	825.9940	826.0665
826.1190	826.1645	826.2238
826.2562	826.3652	826.4311
826.4730	826.6405	826.7444
826.7637	826.9216	826.9539
826.9747	827.0560	827.1601
827.3960	827.4122	827.4478
827.4788	827.9747	828.0583
828.1267	828.3636	828.8312
829.0118	829.0407	829.3715
829.7002	829.9164	830.6046
831.3183	831.7940	839.5713
845.4419	855.2343	857.5163
859.0813	878.1292	880.0094
889.9043	903.8339	913.0690
918.1277	920.5239	923.2586
924.0160	928.2205	935.2853
937.5639	947.3739	950.4175
951.8616	952.7813	952.8159
953.4871	953.9700	954.4914
954.6756	954.7775	955.5387
955.6247	955.6866	956.4289
957.6802	958.3485	959.4758
959.6274	959.7775	960.1178

960.3398	961.4309	961.4423
961.5261	961.6054	961.8007
961.9543	962.3579	962.5319
962.6679	962.7182	962.9648
962.9979	963.6205	963.6291
963.6955	963.7379	963.9639
964.0404	964.1235	964.1501
964.3210	964.3355	964.8613
964.9636	965.0841	965.2228
965.4677	965.5013	965.5888
965.6058	965.6431	965.7490
965.8953	965.9619	966.0372
966.0541	966.1420	966.1899
966.5337	966.5740	966.5977
966.9041	967.1752	967.2352
967.6613	967.7831	968.0513
968.2194	968.2587	968.4396
968.6092	968.8497	968.8967
968.9433	969.5546	969.6177
969.6362	969.8451	970.0353
970.0889	970.4685	970.5322
970.6395	971.0491	971.1520
971.5301	971.6247	971.7801
972.0189	972.0649	972.1646
972.2626	972.3267	972.3420
972.6355	972.7407	972.8465
973.3245	973.4708	973.5048
973.5266	973.5726	973.7905
973.8547	973.9139	974.0404
974.0671	974.2252	974.3398
974.5205	974.5934	974.7162
974.7654	974.7977	974.8281
974.9184	974.9696	975.0617
975.1126	975.1807	975.2220
975.2428	975.3060	975.5622
975.6818	975.7838	975.8199
975.9365	976.0413	976.0968
976.0974	976.2475	976.2868

976.3775	976.5236	976.6480
976.7282	976.8192	976.9119
977.0025	977.1067	977.1352
977.3144	977.5001	977.7072
977.8598	977.9562	978.1598
978.2356	978.3939	978.4948
978.5058	978.5358	978.6222
978.6312	978.6815	978.8172
978.8271	978.9986	979.0281
979.1219	979.1859	979.2169
979.3163	979.3778	979.4963
979.5326	979.6067	979.6596
979.8672	979.9649	979.9800
980.0284	980.0763	980.1184
980.2359	980.2771	980.3857
980.4636	980.6161	980.6322
980.7586	980.8879	981.1071
981.2249	981.4238	981.6075
981.8567	981.9363	982.0623
982.1566	982.1885	982.3801
982.6773	982.8335	982.9166
983.3037	983.3224	983.3908
983.5840	983.7595	983.8006
983.9844	984.0811	984.1318
984.2305	984.3377	984.3691
984.5279	984.6228	984.6940
984.9248	985.0899	985.1502
985.3721	985.4330	985.6490
985.7198	985.7953	985.8183
985.8717	985.9571	986.1522
986.2393	986.3894	986.4744
986.4973	986.8568	987.1676
987.4458	987.5538	987.7690
987.8738	988.0670	988.0953
988.1306	988.2759	988.3143
988.4414	988.5011	988.6872
988.7227	988.7402	989.0109
989.1459	989.2401	989.4454

989.5335	989.7996	990.2627
990.3086	990.5088	990.6954
990.7537	991.3062	991.3903
991.4126	991.4716	991.5521
991.6180	991.9092	991.9613
992.1158	992.2321	992.3127
992.3511	992.8357	992.9431
993.0609	993.1278	993.1973
993.5435	993.7463	993.8920
993.9753	994.3308	994.4389
994.6566	994.6847	994.7196
994.9864	995.1052	995.1698
995.3757	995.4868	995.8402
996.4229	997.1465	997.5773
997.7583	998.1725	998.4556
998.6028	998.8154	999.0020
999.4443	999.7494	999.8299
1000.0721	1000.1457	1000.5201
1000.9115	1001.3620	1001.5414
1002.0583	1003.4065	1003.7286
1004.0043	1004.7400	1004.8124
1004.8671	1005.4094	1006.2310
1006.4723	1007.3923	1007.5104
1008.6058	1009.6149	1010.2111
1012.2321	1012.5834	1018.2208
1019.9624	1021.8355	1026.5130
1037.5540	1044.1598	1054.3576
1055.2818	1059.4904	1060.6081
1061.4720	1062.5535	1064.3418
1065.5513	1067.6441	1068.7637
1070.1104	1071.9121	1074.5286
1075.1288	1075.3233	1076.8504
1078.0818	1080.2779	1080.6305
1080.7064	1080.7331	1081.1474
1081.6732	1081.7318	1081.9625
1082.0916	1082.2688	1082.3430
1082.4000	1082.4273	1082.4814
1082.6821	1083.0184	1083.0802

1083.5466	1083.6164	1083.6211
1083.9367	1083.9979	1084.0816
1084.0866	1084.4220	1084.6654
1084.9534	1085.0723	1085.1132
1085.2427	1085.3097	1085.3365
1085.5720	1085.6138	1085.7177
1085.8822	1085.9221	1085.9809
1085.9896	1086.2208	1086.7150
1086.8168	1086.8278	1086.8809
1087.2960	1087.3453	1087.4256
1087.5053	1087.5326	1087.7492
1088.0089	1088.0394	1088.1476
1088.2032	1088.2642	1088.4058
1088.4227	1088.4824	1088.5023
1088.7497	1088.8618	1089.0487
1089.1647	1089.2549	1089.2823
1089.4794	1089.5920	1089.9676
1090.0377	1090.2793	1090.3708
1090.4889	1090.5468	1090.5571
1090.6633	1091.5716	1091.6216
1091.9426	1092.0724	1092.2294
1092.9327	1093.0754	1093.3780
1093.5141	1093.6922	1093.7497
1094.0368	1094.0943	1094.9578
1095.1169	1095.1361	1095.2193
1095.4022	1095.6110	1095.9502
1096.0202	1096.1096	1096.3478
1096.7358	1096.9491	1097.0316
1097.3230	1097.3816	1097.3935
1097.6551	1097.6719	1097.7507
1097.8359	1097.9247	1097.9749
1098.3601	1098.4902	1098.5418
1098.5481	1098.6462	1098.7246
1098.7756	1098.9219	1098.9844
1099.2326	1099.2434	1099.3642
1099.4264	1099.4988	1099.5519
1099.6090	1099.6552	1099.7937
1099.8409	1100.0880	1100.1157

1100.1427	1100.6136	1100.6298
1100.6997	1100.7546	1100.8235
1100.9951	1101.1523	1101.1904
1101.2994	1101.4016	1101.4162
1101.5856	1101.6782	1101.7960
1101.8316	1101.8860	1102.0260
1102.0744	1102.1653	1102.2398
1102.3047	1102.4689	1102.6480
1102.6810	1102.7599	1102.7858
1103.1930	1103.4171	1103.5481
1103.6660	1103.7565	1103.7903
1104.0130	1104.1101	1104.1293
1104.2650	1104.4624	1104.5820
1104.8862	1105.3727	1105.4667
1105.6723	1105.8073	1105.9062
1106.1868	1106.6337	1106.8177
1107.1703	1107.3169	1107.3666
1107.3928	1107.5000	1108.1717
1108.1845	1108.3663	1108.7052
1108.7784	1108.7906	1108.9515
1109.0062	1109.9013	1110.1914
1110.7960	1111.0620	1111.2345
1111.6876	1112.6969	1113.8884
1114.0758	1114.4772	1114.9475
1115.2803	1116.0268	1116.2106
1116.3254	1116.4592	1116.8090
1117.1711	1117.1799	1117.3594
1117.4098	1117.5860	1117.9186
1118.3615	1118.5310	1118.7440
1118.9262	1119.0765	1119.4248
1119.6974	1119.9144	1119.9910
1120.1128	1120.2330	1120.3413
1120.3863	1120.6291	1120.6728
1121.0080	1121.6035	1121.6150
1121.7343	1121.7728	1122.0518
1122.3200	1122.8774	1123.0663
1123.1003	1123.1373	1123.3751
1123.4687	1123.7402	1123.7936

1123.9866	1124.1553	1124.4715
1124.5238	1124.7541	1125.2150
1125.4110	1125.8406	1125.8888
1126.0654	1126.8415	1127.0240
1127.3109	1127.4136	1127.5258
1127.8425	1128.0456	1128.2406
1128.4584	1129.2830	1129.3640
1129.7457	1129.9425	1131.2312
1131.2758	1131.4986	1132.8392
1133.0178	1133.4143	1133.7174
1134.2880	1134.6835	1136.1886
1137.2781	1137.6724	1138.8030
1139.3057	1140.4134	1140.8919
1140.9442	1141.6120	1142.1732
1142.7011	1142.8166	1143.8163
1145.1735	1146.2361	1146.3753
1146.7550	1147.5617	1148.0491
1148.8422	1149.2490	1150.0731
1150.2432	1151.5105	1152.0168
1152.9043	1153.3862	1154.7812
1155.1232	1157.4414	1158.4101
1163.9029	1166.9221	1169.3084
1169.3790	1169.5481	1170.0439
1170.1277	1172.0742	1172.2723
1172.7760	1173.9324	1174.4310
1174.6201	1174.6509	1174.8058
1175.1965	1175.5301	1175.8440
1176.3004	1176.5733	1177.4850
1178.1023	1178.7208	1178.9655
1179.6011	1180.1212	1181.1720
1181.8839	1183.4303	1183.5204
1184.2468	1184.3053	1184.4689
1184.7105	1185.2555	1185.5273
1185.6676	1186.2419	1186.4585
1186.5988	1187.1147	1187.3272
1187.4068	1187.4377	1187.8047
1187.8147	1187.9879	1188.0074
1188.0270	1188.0792	1188.2033

1188.4565	1188.5318	1188.8362
1188.8779	1189.1796	1189.2173
1189.2258	1189.4644	1189.7398
1189.8807	1189.9359	1190.0938
1190.0981	1190.5357	1190.6416
1190.7273	1191.2543	1191.3916
1191.6406	1192.1982	1192.4373
1192.9083	1192.9381	1193.1624
1193.2728	1193.3248	1193.3917
1194.1923	1195.1859	1195.3177
1195.3336	1195.7336	1195.8143
1196.1290	1196.1874	1196.4193
1196.6344	1196.7838	1197.1863
1197.2744	1197.7184	1197.9031
1198.0543	1200.5306	1201.4880
1201.6403	1202.0103	1203.5275
1203.7323	1204.4724	1204.7874
1205.6259	1206.4133	1208.8369
1214.1288	1217.8264	1223.5545
1224.7610	1232.8218	1233.8485
1235.4686	1235.5323	1235.9898
1235.9979	1236.0588	1236.3738
1236.6395	1236.8835	1237.4201
1237.5434	1237.5759	1237.5895
1238.0027	1238.1935	1238.7855
1238.8886	1238.9699	1239.0263
1239.0590	1239.5414	1239.6964
1239.7861	1239.8988	1240.2248
1240.2433	1240.3073	1240.5593
1240.8137	1240.9118	1241.1323
1241.2057	1241.2822	1241.6078
1242.4979	1243.2987	1243.7460
1243.8557	1243.9181	1244.3282
1244.6875	1244.8993	1244.9981
1245.1189	1245.1483	1245.5188
1245.6072	1245.7223	1246.3533
1246.7011	1246.7813	1247.1530
1247.2013	1247.3130	1247.3736

1247.4905	1247.7530	1247.9249
1248.0787	1248.3160	1248.5487
1248.7489	1248.8487	1249.1213
1250.6056	1250.7396	1251.0791
1251.7330	1252.7773	1252.8839
1253.1638	1253.3359	1253.9331
1254.2599	1254.3125	1255.0082
1255.4055	1255.9211	1256.8589
1257.0819	1257.3616	1258.0379
1258.0768	1258.6479	1258.7261
1258.9401	1259.0192	1259.3214
1259.8178	1260.1013	1260.6271
1261.3063	1262.1267	1262.4366
1262.7688	1263.1219	1263.2581
1263.4297	1263.8219	1264.0478
1264.0735	1264.5139	1264.5535
1265.4023	1266.4147	1267.3429
1267.9110	1268.7679	1269.4010
1270.2356	1274.0764	1274.9168
1283.5181	1284.1408	1285.5964
1285.8255	1286.4723	1286.7941
1287.1227	1287.1472	1287.7168
1288.1626	1289.2335	1289.5738
1290.1058	1290.3990	1290.6590
1291.0335	1291.0857	1291.1200
1291.3225	1291.3582	1291.3917
1291.4205	1291.8227	1291.8726
1292.0785	1292.2841	1292.3498
1292.4522	1292.6719	1292.7031
1293.0566	1293.0975	1293.3810
1293.4543	1293.5529	1293.5555
1293.7776	1294.0346	1294.6161
1294.8068	1295.0265	1295.1027
1295.1325	1295.1942	1295.4085
1295.5779	1295.6113	1295.9534
1295.9718	1296.1084	1296.7859
1296.7977	1296.8206	1296.8647
1296.8804	1296.9532	1297.2498

1297.4368	1297.5599	1297.7672
1297.9195	1298.0905	1298.2440
1298.2875	1298.7655	1298.8067
1298.8257	1299.0563	1299.2894
1299.4276	1299.7724	1299.8324
1299.9617	1300.0314	1300.0702
1300.1193	1300.1517	1300.5569
1300.8596	1301.3409	1301.3569
1301.8438	1302.0425	1302.2704
1302.2953	1302.9972	1303.3392
1303.4409	1304.0357	1304.3016
1304.5753	1304.6017	1306.2308
1306.2832	1306.3610	1306.4324
1307.8631	1308.2029	1308.3860
1308.6605	1309.3687	1310.2114
1312.1989	1312.4971	1315.7048
1326.4440	1327.6560	1329.9757
1330.7546	1337.1167	1346.3165
1353.1600	1353.5787	1353.5894
1354.7479	1356.0615	1356.4166
1356.6301	1356.8760	1357.2892
1357.3615	1357.4872	1357.5652
1358.2935	1358.4863	1359.0067
1359.2571	1359.6399	1360.3473
1360.3855	1360.5971	1360.7535
1360.8267	1361.5582	1361.7379
1361.8216	1361.8766	1362.0379
1362.5460	1362.6405	1363.3407
1363.3790	1363.4117	1363.4683
1363.5475	1363.8531	1363.9051
1364.2937	1364.5817	1364.6382
1364.7075	1364.9508	1365.4727
1365.5292	1365.7817	1365.9798
1366.0170	1366.8317	1367.1747
1367.2429	1367.7615	1367.8854
1367.9332	1368.6115	1368.7231
1368.9393	1369.1736	1369.6661
1369.9117	1370.4337	1370.6979

1370.7731	1371.0690	1371.1524
1372.2328	1372.5665	1373.2788
1373.3403	1373.6962	1373.8944
1374.0281	1374.1337	1374.6532
1375.0483	1375.3480	1377.3047
1377.7197	1378.0034	1380.1972
1380.4216	1380.6865	1381.6095
1381.8183	1381.8260	1383.6902
1384.1620	1384.8543	1385.2557
1386.9185	1388.8635	1389.6192
1390.3370	1390.8188	1390.9097
1391.0638	1391.2611	1392.1734
1392.5415	1394.0125	1394.7606
1394.8224	1395.5751	1395.7911
1396.2212	1396.3204	1396.4976
1397.0209	1397.0868	1397.1448
1397.4016	1397.6491	1397.8632
1397.8960	1397.9326	1398.0754
1398.1158	1398.1263	1398.4478
1398.8315	1399.7014	1399.7352
1399.7659	1400.3984	1400.4635
1400.5224	1400.6477	1401.3049
1401.5670	1401.6326	1403.2547
1403.6994	1403.8870	1404.0019
1404.1499	1404.1753	1404.1941
1404.4984	1404.6118	1405.8583
1406.0829	1406.3699	1406.5331
1406.6368	1407.0413	1407.4830
1407.6810	1408.2386	1408.3747
1408.5100	1408.7068	1408.7653
1409.1301	1409.4679	1409.5691
1410.2763	1410.3101	1410.3585
1410.6910	1410.7626	1410.7980
1410.9674	1411.0648	1411.1007
1411.2921	1411.2945	1411.3269
1411.3532	1411.4058	1411.5002
1411.9010	1411.9731	1412.1326
1412.1672	1412.2651	1412.4098

1412.5356	1412.6095	1412.6560
1412.6698	1413.0100	1413.0134
1413.0220	1413.0492	1413.0959
1413.1631	1413.2670	1413.3719
1413.6475	1413.7237	1413.8174
1413.8431	1414.0261	1414.0766
1414.1078	1414.4550	1414.5396
1414.7053	1414.8903	1415.1727
1415.2546	1415.3262	1415.5910
1415.7661	1415.9333	1416.1058
1416.2923	1416.5321	1416.6782
1416.9353	1417.3042	1417.4499
1417.7791	1417.8460	1418.4377
1418.5183	1418.9549	1419.2745
1419.5785	1420.7681	1421.0509
1421.9823	1422.2048	1422.4077
1429.1515	1434.6659	1438.9501
1440.8836	1442.5264	1443.9993
1444.3370	1453.3481	1458.8549
1460.8209	1460.9312	1463.1426
1463.9109	1464.3350	1465.5963
1465.9939	1466.0120	1466.3413
1466.6159	1467.0147	1468.3797
1468.5813	1468.6812	1469.1741
1469.2452	1469.3336	1469.4728
1469.7647	1469.9458	1470.0126
1470.1439	1470.3706	1470.3975
1470.4758	1470.4811	1470.4868
1470.5628	1470.6006	1470.7221
1470.8289	1470.8956	1470.9327
1470.9472	1471.1439	1471.1927
1471.2702	1471.3560	1471.3664
1471.5840	1471.6179	1471.6655
1471.7147	1471.7877	1471.7971
1471.8830	1471.9760	1472.0978
1472.2149	1472.2826	1472.3871
1472.4357	1472.4509	1472.4637
1472.5662	1472.6235	1472.6477

1472.6957	1472.9081	1472.9420
1472.9609	1472.9788	1473.0639
1473.0988	1473.1542	1473.2392
1473.3133	1473.4109	1473.4638
1473.5773	1473.6821	1473.6885
1473.8529	1474.0748	1474.0915
1474.1026	1474.1756	1474.3776
1474.4755	1474.5007	1474.5910
1474.7312	1474.8214	1474.8742
1474.9677	1475.0016	1475.0267
1475.0797	1475.2123	1475.2247
1475.3213	1475.4255	1475.4630
1475.5120	1475.5508	1475.5838
1475.6163	1475.6606	1475.7803
1475.8114	1475.8398	1475.8577
1475.9054	1475.9425	1475.9879
1476.0107	1476.0545	1476.0849
1476.0972	1476.1374	1476.1753
1476.2882	1476.4086	1476.4928
1476.6591	1476.6671	1476.6971
1476.8209	1476.8463	1476.8736
1477.0921	1477.1602	1477.1727
1477.2772	1477.3096	1477.3189
1477.4237	1477.4410	1477.5096
1477.5300	1477.6931	1477.7334
1477.8183	1477.9098	1477.9473
1478.0196	1478.0498	1478.0545
1478.1375	1478.2044	1478.2747
1478.3236	1478.3943	1478.4105
1478.5087	1478.5359	1478.6310
1478.6975	1478.7544	1478.8695
1478.9235	1478.9422	1479.0512
1479.1478	1479.2345	1479.5228
1479.5562	1479.7682	1479.8054
1479.9622	1480.1922	1480.2551
1480.2988	1480.4033	1480.4310
1480.5533	1480.5704	1480.6177
1480.8624	1481.0740	1481.0822

1481.1139	1481.1738	1481.2176
1481.2683	1481.3159	1481.6242
1481.7027	1481.7154	1481.8125
1481.8596	1481.9098	1481.9331
1481.9724	1482.0371	1482.2818
1482.4082	1482.4515	1482.7252
1482.7639	1482.9547	1482.9610
1483.0700	1483.1230	1483.2229
1483.3223	1483.3283	1483.4159
1483.5204	1483.5637	1483.6423
1483.7953	1483.8099	1483.8492
1483.9328	1483.9994	1484.1286
1484.1384	1484.1743	1484.2392
1484.4153	1484.4378	1484.4818
1484.5834	1484.8496	1484.9711
1485.0863	1485.2766	1485.2951
1485.3356	1485.4286	1485.6031
1485.7260	1485.8024	1485.9423
1486.0536	1486.0604	1486.1586
1486.2017	1486.2404	1486.3407
1486.3447	1486.5209	1486.6260
1486.6732	1486.7051	1486.7450
1486.7911	1487.0763	1487.1378
1487.2944	1487.4259	1487.4558
1487.4961	1487.7572	1487.7714
1487.8158	1487.9142	1488.1759
1488.3698	1488.4315	1488.5227
1488.5513	1488.6434	1488.7841
1488.9261	1489.0390	1489.0869
1489.3174	1489.3996	1489.6365
1489.6553	1489.8063	1489.9641
1490.3177	1490.4793	1490.5474
1490.6729	1490.7581	1490.7766
1490.8652	1490.9404	1491.0796
1491.3771	1491.4579	1491.6095
1491.6530	1491.7949	1491.9735
1492.0754	1492.3378	1492.3776
1492.6507	1492.6824	1492.8323

1492.9160	1493.1251	1493.3016
1493.4154	1493.4604	1493.5126
1493.9302	1494.0163	1494.0977
1494.2453	1494.3049	1494.3320
1494.3731	1494.4000	1494.4337
1494.5362	1494.6130	1494.6354
1494.7034	1494.7654	1494.8278
1494.8818	1495.1086	1495.1896
1495.2682	1495.3339	1495.4303
1495.5161	1495.6201	1495.6331
1495.7272	1495.8497	1495.9060
1496.0421	1496.2405	1496.2654
1496.3852	1496.4091	1496.4779
1496.6165	1496.7195	1496.8213
1496.9635	1497.0895	1497.1377
1497.2492	1497.3066	1497.3756
1497.4134	1497.5106	1497.5324
1497.5766	1497.7683	1497.7837
1497.9600	1498.0625	1498.1371
1498.3156	1498.4839	1498.5889
1498.8046	1499.0128	1499.1191
1499.3636	1499.4263	1499.6408
1499.6549	1499.7430	1499.8178
1500.0746	1500.1865	1500.2773
1500.3479	1500.4669	1500.6337
1500.7431	1500.7731	1501.0307
1501.0440	1501.2179	1501.3853
1501.5079	1501.6205	1501.8043
1502.2341	1502.3179	1502.3904
1502.6230	1502.7680	1502.9435
1503.2012	1503.4485	1503.4895
1503.8695	1503.9868	1504.0208
1504.2055	1504.8440	1505.3280
1505.4304	1505.5621	1505.7312
1505.8869	1506.2632	1507.0125
1507.2001	1507.3781	1507.4633
1507.6640	1507.9459	1508.1474
1508.4197	1508.5035	1509.0356

1509.1515	1509.4869	1509.9210
1510.1547	1510.6701	1510.8662
1511.2111	1511.6591	1511.8707
1512.0765	1512.5426	1512.9141
1513.5058	1513.6108	1513.8801
1514.5117	1514.7097	1515.1057
1515.4965	1515.9405	1516.3474
1517.1749	1518.1110	1518.7768
1519.5551	1519.7544	1520.0614
1520.7171	1520.7385	1520.7640
1520.9430	1521.1563	1522.4676
1522.4817	1523.0063	1523.2509
1523.4605	1524.1365	1524.2265
1524.3191	1524.5172	1524.6046
1524.9459	1524.9888	1525.2241
1525.5161	1525.5162	1525.6321
1525.7342	1525.9373	1526.6338
1526.9745	1527.1638	1527.5551
1527.8397	1527.8984	1528.0620
1528.1132	1528.2075	1528.3586
1528.4937	1528.5432	1528.7782
1528.8954	1528.9520	1529.0105
1529.1084	1529.1494	1529.2359
1529.2758	1529.3917	1529.8765
1530.1771	1530.5008	1530.7110
1530.8401	1530.9039	1531.2030
1531.3392	1531.5699	1531.6111
1531.7123	1531.7767	1532.1956
1532.2801	1532.2963	1532.3864
1532.6543	1532.7018	1532.9128
1533.5273	1533.7505	1534.3345
1534.3894	1534.6508	1534.6598
1534.7491	1535.0444	1535.4095
1535.4573	1536.4904	1536.5936
1537.1112	1537.3041	1537.8594
1537.9154	1538.6158	1538.7456
1539.0458	1539.3747	1539.5183
1539.6987	1540.2187	1540.2774

1540.6200	1541.1718	1541.4349
1541.9373	1541.9644	1542.3936
1542.7331	1542.8005	1543.7164
1543.8162	1544.2632	1544.4793
1544.9032	1545.3612	1545.9508
1546.0378	1546.6904	1548.9388
1549.2327	1549.3278	1550.3022
1550.7771	1551.0144	1551.1700
1551.4227	1553.7830	1555.1849
1556.1276	1556.3051	1557.0130
1560.6767	1566.2561	1576.9670
1577.5325	1577.7395	1577.9633
1577.9899	1578.0656	1578.0898
1578.1314	1578.2529	1578.3688
1578.4033	1578.7930	1578.8074
1578.8763	1578.9267	1578.9523
1579.0871	1579.1155	1579.5085
1579.6306	1579.7431	1579.9114
1579.9647	1579.9994	1580.0963
1580.1234	1580.1396	1580.1869
1580.2855	1580.3154	1580.4583
1580.6759	1580.8260	1580.8300
1581.0298	1581.0692	1581.1499
1581.3744	1581.4527	1581.4647
1581.5871	1581.6563	1581.7442
1581.7946	1582.2749	1582.3600
1582.5187	1582.6792	1582.8440
1582.9199	1583.0454	1583.0611
1583.1762	1583.6939	1583.7148
1583.9707	1584.1710	1584.7178
1584.8349	1585.1044	1585.2499
1585.3601	1585.5413	1585.6331
1585.7186	1585.9911	1586.1305
1586.3882	1586.4601	1586.6171
1586.6315	1586.6693	1587.3592
1587.8592	1587.9125	1588.0068
1588.3001	1588.4176	1588.5464
1588.5689	1589.1926	1589.3775

1589.4657	1589.7096	1589.9773
1590.3832	1590.4699	1590.8796
1591.4145	1591.6284	1591.9327
1592.8298	1593.5817	1593.7820
1594.0952	1594.6402	1595.6655
1596.0128	1596.9061	1597.3147
1599.8897	1600.0485	1605.4633
1605.6557	1605.6954	1605.7023
1605.8872	1606.1673	1606.2644
1606.4426	1606.5203	1606.7989
1606.8874	1606.9512	1607.0485
1607.1264	1607.3096	1607.4000
1607.4436	1607.6192	1607.8543
1607.9248	1608.0342	1608.0563
1608.0782	1608.1895	1608.3605
1608.5162	1608.6583	1608.7167
1608.7993	1608.9355	1608.9637
1609.1793	1609.2764	1609.5492
1609.5702	1609.7312	1609.8237
1609.8920	1610.3020	1610.7481
1610.7582	1610.8227	1610.9520
1611.0251	1611.0870	1611.1829
1611.2371	1611.3026	1611.3664
1611.5043	1611.5808	1611.7218
1611.7318	1611.9161	1612.1477
1612.2210	1612.2544	1612.6135
1612.9243	1612.9587	1612.9923
1613.0205	1613.1211	1613.2028
1613.4759	1613.5385	1613.6159
1614.1795	1614.1954	1614.2006
1614.3282	1614.3865	1614.6319
1614.7502	1614.8403	1614.9489
1614.9707	1615.5483	1615.6600
1615.7446	1616.0955	1616.1236
1616.5049	1617.2866	1617.3298
1617.4982	1618.2096	1618.3883
1618.4554	1618.9375	1619.4490
1619.5553	1620.6454	1620.7396

1621.6881	1621.8870	1622.0159
1622.0764	1623.2653	1624.6175
1625.2519	1627.0743	1639.8527
1654.0128	1654.5534	1654.7819
1655.0755	1655.1528	1655.3972
1655.7466	1655.7624	1655.9938
1656.0160	1656.2298	1656.6537
1656.7078	1656.7749	1656.8894
1657.1092	1657.1755	1657.2931
1657.3708	1657.6542	1657.7278
1657.7616	1657.8225	1657.9421
1657.9541	1658.0215	1658.2064
1658.2112	1658.2335	1658.2708
1658.3502	1658.5960	1658.7147
1658.9316	1659.0276	1659.0697
1659.3073	1659.3733	1659.5622
1659.6700	1659.6926	1659.9877
1660.2403	1660.2716	1660.3943
1660.4307	1660.9507	1660.9646
1661.1688	1661.2428	1661.3278
1661.5069	1661.5864	1661.9391
1662.0203	1662.1735	1662.3319
1662.5962	1662.6133	1663.1323
1663.1869	1663.3240	1663.6911
1663.7285	1664.0686	1664.2303
1664.9701	1665.3253	1665.5210
1666.0301	1666.2230	1666.3072
1667.3201	1667.6215	1668.6573
1669.4252	1669.7355	1670.6508
1671.9700	1672.0895	1672.1612
1672.1992	1672.8440	1673.0594
1673.2113	1673.2566	1673.3540
1673.4257	1674.0114	1674.4399
1674.5196	1675.3260	1675.7566
1676.0203	1676.1876	1676.2407
1676.4933	1676.6659	1676.9954
1677.4525	1679.0518	1683.4287
1684.2999	1686.2161	1694.9216

1699.9406	1701.0112	1702.8388
1709.5430	1808.8893	1809.0026
1809.7900	1810.1515	1810.2314
1810.2977	1810.3458	1810.4675
1810.6402	1810.7673	1810.8112
1810.8183	1811.0285	1811.1486
1811.1738	1811.2444	1811.3837
1811.4811	1811.6863	1811.7198
1811.7805	1811.8129	1811.8195
1811.9383	1811.9891	1812.0442
1812.1848	1812.2855	1812.2914
1812.3195	1812.5392	1812.6187
1812.8432	1812.9069	1812.9753
1813.0230	1813.0604	1813.1942
1813.2438	1813.4422	1813.4755
1813.4846	1813.5093	1813.5528
1813.6177	1813.6840	1813.6977
1813.7488	1813.7769	1813.8083
1814.0857	1814.1190	1814.1250
1814.2737	1814.3695	1814.3744
1814.3911	1814.3936	1814.4908
1814.5480	1814.5921	1814.7087
1814.7173	1814.8874	1814.9573
1814.9581	1815.2532	1815.2577
1815.3673	1815.4882	1815.4926
1815.5598	1815.7438	1815.8875
1816.0171	1816.0307	1816.2731
1816.4298	1816.4740	1816.5136
1816.7413	1816.7915	1816.8005
1816.9751	1817.0829	1817.1862
1817.3356	1817.4286	1817.5929
1817.6142	1817.9194	1817.9863
1818.5668	1818.6942	1819.3216
1819.3905	1819.6712	1821.2270
1821.4635	1822.2585	1836.6413
1836.9326	1837.8175	1837.8506
1838.6565	1839.3191	1840.4930
1840.6225	1840.9186	1841.0105

1841.7021	1842.0240	1842.2251
1842.7872	1843.4121	1843.9358
1844.0694	1844.1101	1844.1188
1844.1576	1844.3855	1844.6744
1845.0780	1845.0996	1845.7770
1846.1705	1846.4222	1846.4407
1846.6303	1847.0068	1847.2099
1847.2255	1847.2317	1847.3192
1847.3595	1847.4043	1847.4896
1847.7318	1848.0630	1848.1670
1848.2366	1848.3799	1848.4467
1848.4841	1848.5305	1848.7364
1848.7469	1848.8836	1848.9272
1849.0500	1849.0923	1849.3345
1849.3655	1849.4876	1849.5193
1849.5435	1849.6573	1849.7578
1849.7722	1850.0573	1850.1290
1850.1305	1850.1539	1850.1554
1850.3749	1850.4163	1850.4447
1850.4975	1850.6518	1850.7206
1850.9554	1851.0227	1851.2000
1851.2191	1851.2403	1851.2406
1851.3528	1852.0238	1852.0576
1852.0687	1852.2179	1852.2964
1852.5315	1852.6666	1852.9583
1853.0038	1853.1163	1853.3771
1853.8791	1854.0180	1854.0217
1854.3608	1854.5515	1855.2226
1855.5322	1856.1905	1856.3055
1856.8262	1857.5412	1857.7905
2872.5996	2873.1365	2873.2769
2873.4365	2873.8130	2873.8303
2873.8898	2874.1738	2874.1830
2874.2917	2874.4137	2874.7880
2874.9328	2875.2181	2875.2905
2875.3146	2875.3476	2875.5335
2875.5529	2875.6767	2875.6855
2875.7916	2875.8196	2875.8501

2875.8617	2875.9488	2876.0414
2876.0629	2876.2626	2876.3158
2876.3558	2876.4331	2876.6301
2876.6712	2876.6774	2876.6968
2876.7192	2876.7411	2876.7945
2876.8970	2876.9525	2876.9540
2877.0125	2877.0451	2877.0925
2877.1998	2877.2083	2877.2179
2877.2610	2877.3182	2877.4284
2877.4606	2877.5373	2877.5609
2877.7564	2877.8993	2877.9203
2878.2422	2878.3061	2878.3812
2878.4020	2878.8118	2878.8485
2879.1829	2879.4171	2879.4303
2879.5951	2879.7955	2879.8566
2879.8979	2880.2631	2880.3116
2880.5261	2880.5956	2880.5997
2880.6229	2880.8135	2880.9978
2881.0564	2881.1430	2881.3389
2881.8746	2881.9626	2882.0883
2882.2562	2882.7462	2882.8365
2883.0217	2883.2744	2883.3426
2883.4142	2883.4799	2883.5722
2883.6354	2883.6405	2883.8084
2883.9094	2883.9149	2884.1260
2884.1720	2884.1904	2884.2222
2884.3438	2884.4759	2884.5355
2884.5941	2884.6298	2884.6921
2884.6992	2884.7385	2884.7843
2884.8619	2884.9562	2885.0875
2885.1085	2885.1392	2885.3180
2885.3490	2885.3769	2885.4405
2885.5460	2885.5874	2885.5986
2885.7127	2885.7734	2885.8541
2885.9408	2885.9591	2885.9761
2885.9923	2886.0414	2886.0909
2886.1618	2886.2762	2886.4328
2886.5051	2886.5737	2886.5902

2886.6332	2886.6919	2886.7167
2886.7428	2886.8763	2886.9150
2887.0583	2887.0681	2887.0772
2887.3868	2887.4207	2887.4802
2887.6312	2887.7084	2887.7896
2887.9288	2888.0079	2888.0200
2888.0465	2888.1390	2888.1484
2888.1862	2888.3737	2888.4000
2888.4950	2888.5595	2888.7087
2888.7477	2888.9656	2888.9901
2889.0542	2889.2582	2889.3581
2889.3938	2889.4626	2889.8956
2889.9384	2890.0205	2890.1792
2890.2219	2890.4290	2890.5653
2890.5890	2890.6309	2890.6524
2890.6740	2890.8527	2890.9033
2890.9617	2890.9757	2891.0496
2891.0890	2891.1794	2891.2960
2891.3821	2891.4393	2891.4633
2891.5743	2891.5912	2891.6385
2891.8288	2891.8495	2891.8658
2891.8878	2891.9065	2891.9869
2892.0693	2892.1177	2892.2063
2892.2351	2892.2558	2892.2664
2892.2993	2892.3714	2892.3804
2892.4438	2892.4715	2892.4947
2892.5944	2892.6729	2892.7123
2892.7230	2892.8186	2892.8492
2892.9098	2892.9485	2893.1105
2893.1456	2893.2208	2893.2435
2893.2999	2893.3443	2893.4314
2893.5370	2893.6380	2893.7286
2893.7670	2893.8785	2893.9060
2894.0274	2894.0626	2894.0871
2894.1746	2894.2102	2894.2692
2894.3613	2894.4204	2894.4293
2894.4598	2894.5216	2894.5674
2894.6141	2894.6966	2894.7201

2894.9625	2895.0929	2895.1082
2895.1112	2895.3228	2895.3891
2895.4156	2895.4300	2895.4726
2895.5478	2895.5873	2895.7106
2895.7882	2895.8054	2895.8437
2895.9945	2896.0362	2896.0786
2896.2112	2896.2790	2896.2863
2896.3494	2896.4445	2896.5189
2896.5423	2896.5719	2896.5776
2896.7648	2896.7837	2896.8294
2897.0544	2897.0612	2897.0952
2897.1835	2897.2735	2897.3727
2897.4200	2897.4494	2897.5501
2897.6244	2897.6603	2897.7079
2897.7215	2897.7777	2897.8171
2897.9732	2898.0445	2898.0957
2898.1278	2898.1777	2898.2390
2898.2901	2898.3513	2898.3785
2898.4665	2898.5551	2898.5893
2898.6204	2898.7530	2898.8017
2898.8443	2898.8913	2898.9356
2899.0834	2899.2480	2899.3639
2899.5390	2899.6662	2899.8985
2899.9186	2900.1303	2900.2638
2900.3261	2900.3754	2900.3944
2900.5714	2900.6260	2900.8408
2900.9952	2901.1876	2901.2746
2901.3457	2901.4336	2901.5525
2901.6387	2901.7487	2901.8326
2901.9087	2902.1505	2902.1984
2902.2880	2902.4749	2902.4877
2902.5593	2902.6089	2902.7554
2902.8482	2902.8792	2903.1478
2903.1727	2903.3429	2903.4241
2903.5150	2903.5636	2903.6019
2903.8563	2904.0356	2904.2932
2904.3838	2904.4934	2904.5165
2904.6095	2904.9864	2905.1080

2905.2943	2905.4250	2905.4670
2905.5019	2905.6019	2905.9603
2906.0135	2906.3163	2906.4214
2906.7007	2906.9396	2907.0570
2907.1551	2907.5676	2907.7630
2907.7954	2908.1161	2908.1713
2908.4049	2908.9089	2909.0135
2909.4999	2909.7096	2909.7353
2911.2037	2911.3518	2911.3830
2911.3969	2911.9782	2912.2902
2912.5153	2912.9191	2913.7730
2914.4264	2950.6855	2950.7161
2950.8039	2951.0071	2951.0082
2951.0681	2951.1490	2951.1788
2951.3056	2951.3718	2951.5159
2951.5264	2951.5274	2951.7407
2951.7576	2951.8248	2951.8877
2951.9057	2951.9123	2951.9366
2951.9856	2952.0226	2952.0451
2952.1014	2952.2057	2952.3978
2952.5748	2952.6366	2952.6896
2952.9956	2953.0210	2953.1124
2953.1187	2953.1449	2953.2100
2953.2525	2953.2702	2953.2867
2953.3299	2953.3333	2953.3529
2953.3624	2953.3931	2953.4365
2953.4630	2953.5095	2953.7481
2954.0061	2954.1130	2954.1583
2954.2000	2954.2481	2954.2602
2954.4219	2954.4438	2954.4443
2954.5056	2954.6318	2954.6586
2954.7720	2954.7810	2954.8151
2954.8525	2954.8644	2954.8831
2954.9883	2955.0631	2955.1021
2955.1196	2955.2004	2955.2332
2955.2752	2955.3010	2955.3686
2955.3899	2955.4107	2955.4422
2955.5619	2955.5959	2955.6014

2955.6294	2955.6592	2955.6736
2955.6760	2955.7293	2955.8730
2955.8857	2955.9036	2955.9496
2956.0381	2956.0653	2956.1054
2956.1506	2956.1835	2956.2012
2956.2310	2956.2646	2956.3135
2956.3719	2956.3956	2956.4402
2956.4684	2956.4767	2956.5519
2956.5822	2956.5927	2956.6166
2956.6274	2956.6792	2956.7140
2956.7441	2956.7683	2956.7873
2956.8010	2956.8298	2956.8576
2956.8918	2956.9454	2956.9570
2957.0091	2957.0121	2957.0472
2957.1198	2957.1472	2957.1645
2957.2202	2957.3070	2957.3258
2957.3421	2957.3654	2957.4046
2957.4170	2957.4474	2957.4556
2957.4943	2957.5727	2957.7118
2957.7365	2957.7504	2957.7870
2957.7960	2957.8127	2957.8321
2957.8482	2957.8600	2957.8666
2957.8931	2957.9266	2957.9549
2957.9689	2958.0879	2958.1351
2958.1608	2958.1800	2958.2606
2958.2837	2958.3412	2958.3778
2958.4032	2958.4193	2958.4615
2958.5141	2958.6877	2958.8041
2958.8440	2958.8489	2958.8637
2958.9570	2958.9748	2958.9868
2959.0297	2959.0509	2959.0600
2959.0882	2959.1209	2959.1480
2959.1687	2959.1944	2959.2055
2959.2172	2959.2407	2959.2604
2959.3044	2959.3460	2959.4279
2959.4289	2959.4618	2959.4910
2959.5010	2959.5673	2959.6191
2959.7092	2959.7501	2959.7623

2959.8132	2959.8190	2959.8668
2959.9098	2959.9274	2959.9400
2960.0186	2960.1867	2960.1995
2960.2361	2960.3331	2960.3838
2960.4034	2960.4324	2960.4996
2960.5040	2960.5751	2960.5916
2960.6440	2960.6551	2960.6877
2960.7095	2960.7231	2960.7639
2960.8131	2960.9819	2961.0109
2961.0319	2961.0477	2961.0561
2961.1359	2961.1794	2961.2436
2961.2655	2961.2769	2961.2805
2961.3130	2961.3454	2961.4308
2961.4435	2961.4753	2961.5108
2961.5624	2961.6442	2961.6681
2961.7203	2961.8208	2961.8520
2961.9355	2962.0418	2962.0656
2962.1727	2962.2097	2962.2801
2962.3126	2962.3369	2962.3398
2962.3741	2962.3990	2962.4134
2962.5492	2962.5567	2962.6006
2962.6650	2962.6795	2962.7602
2962.7933	2962.8445	2962.8684
2962.8799	2962.9074	2962.9112
2962.9845	2963.0349	2963.0622
2963.0798	2963.1528	2963.2295
2963.3219	2963.3645	2963.5307
2963.5482	2963.8243	2963.8510
2964.0183	2964.0437	2964.1226
2964.1862	2964.2101	2964.2719
2964.2769	2964.3449	2964.3892
2964.4061	2964.4128	2964.5525
2964.7338	2964.7366	2964.7814
2964.7823	2964.9011	2964.9563
2964.9899	2965.0133	2965.0227
2965.0332	2965.2531	2965.2560
2965.2650	2965.4047	2965.7962
2965.8422	2965.9016	2965.9585

2966.0404	2966.1319	2966.1330
2966.1841	2966.2849	2966.2939
2966.4710	2966.5751	2966.5846
2966.7489	2966.9529	2967.0094
2967.0416	2967.0992	2967.1650
2967.3371	2967.4310	2967.4517
2967.5045	2967.5143	2967.5907
2967.6070	2967.6352	2967.7153
2967.8583	2967.9042	2967.9119
2967.9645	2968.0110	2968.5133
2968.7401	2968.7511	2968.8406
2968.9204	2969.0356	2969.0707
2969.1775	2969.4048	2969.4450
2969.6098	2969.6299	2969.7540
2969.8392	2970.0727	2970.1077
2970.2471	2970.3182	2970.6063
2970.6248	2970.7779	2971.0687
2971.1325	2971.3942	2971.4348
2971.4567	2971.5438	2971.6237
2971.6711	2971.7185	2971.8784
2972.0995	2972.4049	2972.4896
2972.7721	2973.1387	2973.3629
2973.4764	2973.5253	2973.7156
2974.1218	2974.2240	2974.7251
2974.8205	2975.3762	2975.5003
2975.9387	2976.0382	2976.1178
2976.1336	2976.1408	2976.2011
2976.6172	2977.0956	2977.1385
2977.2095	2978.2269	2978.3732
2979.2626	2979.7170	2981.3285
2981.3500	2982.4640	3047.6708
3065.1576	3065.2605	3074.5396
3078.4617	3078.6664	3078.7158
3078.9959	3082.1744	3083.0113
3085.0199	3088.6991	3089.8619
3093.4197	3100.6832	3102.4105
3104.8133	3108.7154	3142.3141
3147.3556	3151.5009	3152.2675

3153.1205	3157.8030	3158.7880
3158.9634	3161.6892	3161.7186
3163.1568	3164.7620	3166.3328
3171.2279	3173.6313	3174.9159
3175.2774	3179.8477	3181.6052
3182.6918	3182.7830	3187.7769
3191.0764	3192.8676	3198.4658
3199.7422	3204.1089	3204.3856
3205.0177	3205.9287	3211.0714
3217.4509	3220.4463	3223.8272
3226.9120	3230.9347	3233.2133
3234.1830	3235.7376	3237.8926
3239.0909	3242.5850	3248.1077
3252.5719	3254.8864	3258.8884
3261.8498	3262.1097	3262.7734
3276.1458	3282.0657	3283.8873

TS4$_{\mathbf{explicit}}$ (ONIOM(B3LYP/6-31G(d, p):UFF))

-1351.6173	1.2321	4.5698
5.7860	9.5877	9.8283
10.7326	11.0959	11.6172
11.8025	12.0337	12.4812
12.7422	12.8671	12.9778
13.5642	14.1307	14.2772
15.3349	15.5148	15.7583
15.8718	16.5953	16.7066
17.2960	17.5555	17.8846
17.9749	18.2372	18.7334
18.7970	19.2477	19.6263
20.0625	20.2081	20.3337
20.7132	21.1531	21.6022
21.7145	22.0715	22.3596
22.6092	22.9880	23.4558
23.5537	23.6185	23.8265
24.0053	24.2758	24.4579
24.5443	24.6809	25.0531
25.2294	25.5509	25.6905
26.0225	26.2845	26.3734

26.6947	26.8998	26.9908
27.1924	27.4543	27.7149
27.9053	28.2395	28.5364
28.8748	29.0191	29.3423
29.7719	30.0653	30.2223
30.3760	30.7290	30.8490
31.0802	31.5183	31.6120
31.7182	31.8391	32.0789
32.2957	32.8191	32.9981
33.2751	33.4233	33.5673
33.8550	34.0482	34.5633
34.7347	34.9425	35.0663
35.3826	35.4095	35.6753
36.0819	36.1769	36.4251
36.4931	36.7341	36.9759
37.0210	37.1787	37.5947
37.6868	37.8709	37.9654
38.2157	38.3036	38.4117
38.7040	38.8057	38.9931
39.2965	39.4981	39.6721
39.7323	39.9719	40.1997
40.4379	40.6518	40.7460
40.9091	41.1637	41.1837
41.4271	41.4458	41.7935
41.8622	41.9965	42.2650
42.4478	42.6635	42.8072
43.3375	43.4504	43.5157
43.6841	43.9310	44.1258
44.3057	44.5428	44.6437
44.7622	44.8909	45.0098
45.2864	45.4500	45.7698
46.0191	46.0436	46.2774
46.5351	46.6372	46.8752
47.0171	47.2432	47.3917
47.6518	47.8295	48.1024
48.2191	48.4198	48.6593
48.7981	49.1131	49.1638
49.3518	49.5875	49.8034

49.8511	50.0889	50.2425
50.4747	50.6880	50.8326
50.9887	51.1354	51.2863
51.3817	51.4992	51.6018
51.6632	51.9152	52.0338
52.1702	52.5586	52.6842
52.9053	52.9457	53.2866
53.5053	53.7066	53.8441
53.9241	54.1096	54.3491
54.6117	54.8417	54.8900
55.0390	55.3508	55.3797
55.4773	55.6370	55.6795
56.0733	56.1836	56.4072
56.7508	56.9948	57.2273
57.4021	57.4259	57.6526
57.7714	57.9332	58.3110
58.4044	58.5926	58.8172
59.1538	59.2781	59.4972
59.5336	59.6515	59.7291
59.8897	60.1057	60.3685
60.4940	60.6210	60.9317
60.9949	61.0457	61.2642
61.4837	61.5850	61.8530
61.9478	62.1497	62.2556
62.4188	62.5584	62.7518
62.9472	63.1657	63.3377
63.4656	63.5805	64.0215
64.0411	64.1206	64.3348
64.5555	64.7492	64.8446
65.1391	65.3976	65.4184
65.4838	65.7346	66.0591
66.1795	66.3908	66.5001
66.7494	66.8246	67.0907
67.2193	67.4622	67.5098
67.6151	67.6856	67.7903
67.8654	67.9757	68.3627
68.5554	68.6478	69.1961
69.2566	69.2968	69.5370

69.6498	69.8207	69.9845
70.0115	70.2742	70.4744
70.6424	70.9412	71.0440
71.1314	71.4620	71.5016
71.6197	71.6842	71.9357
72.1158	72.2369	72.4315
72.6653	72.8527	73.0917
73.1496	73.3322	73.5095
73.6814	73.7977	74.0486
74.3059	74.4427	74.7389
74.8047	75.1171	75.2370
75.3035	75.5141	75.7439
76.0606	76.0978	76.2720
76.4097	76.7465	76.8740
76.9900	77.1314	77.2551
77.5154	77.8636	77.9958
78.1889	78.3241	78.4324
78.6889	78.7801	78.9306
79.0355	79.1722	79.3676
79.6347	79.7217	79.9975
80.1394	80.3645	80.5625
80.6426	80.9025	81.1222
81.3404	81.5414	81.7038
81.8214	81.9317	82.2106
82.3742	82.4446	82.6608
82.7855	82.8464	83.0853
83.3830	83.4917	83.8004
83.9889	84.2668	84.2880
84.5485	84.8119	84.8771
85.1279	85.1438	85.3379
85.5618	85.6298	85.7229
86.0033	86.1326	86.4080
86.4655	86.7516	86.8790
87.0444	87.1570	87.3552
87.3951	87.5414	87.6759
87.9069	88.1921	88.2092
88.5557	88.6702	88.7908
88.8780	89.2604	89.5126

89.6219	89.7153	89.9916
90.0690	90.2446	90.6833
90.7842	90.8268	91.0328
91.3708	91.4896	91.9281
92.0108	92.1319	92.3395
92.4048	92.6499	92.7709
92.9562	93.0753	93.4648
93.7406	93.8443	93.9564
94.2777	94.3593	94.5874
94.7497	94.9437	95.1127
95.5387	95.7600	96.0483
$$
[truncated: 73,505 more chars]
